# Aryl hydrocarbon receptor promotes the proliferation and aggressive progression of colorectal cancer under high-fat diet via activating INPP4B/AKT/SGK3 signaling pathway

**DOI:** 10.3389/fonc.2026.1907461

**Published:** 2026-08-28

**Authors:** Qiguan Dong, Tong Wu, Yuchao Yang, Yan Yang, Bo Gao, Dianzhi Shi, Hengjiao Wang, Ying Yan

**Affiliations:** 1 Graduate School of Dalian Medical University, Dalian, Liaoning, China; 2 Department of Radiation Oncology, General Hospital of Fushun Mining Bureau of Liaoning Health Industry Group, Fushun, Liaoning, China; 3 Department of Radiation Oncology, General Hospital of Northern Theater Command, Shenyang, Liaoning, China; 4 Department of Neurology, General Hospital of Fushun Mining Bureau of Liaoning Health Industry Group, Fushun, Liaoning, China; 5 Department of Surgical Oncology, General Hospital of Fushun Mining Bureau of Liaoning Health Industry Group, Fushun, Liaoning, China; 6 Department of Clinical Laboratory, General Hospital of Fushun Mining Bureau of Liaoning Health Industry Group, Fushun, Liaoning, China

**Keywords:** AKT/SGK3 pathway, aryl hydrocarbon receptor, colorectal cancer, high-fat diet, INPP4B, tumor aggressive progression

## Abstract

**Introduction:**

Epidemiological evidence confirms high-fat diet (HFD) as a key risk factor for colorectal cancer (CRC) progression. Aryl hydrocarbon receptor (AhR), a ligand-dependent nuclear transcription factor enriched in intestinal tissues, regulates intestinal metabolism and tumorigenesis. However, its role and mechanism in HFD-induced CRC progression remain unclear.

**Methods:**

Here, we constructed a HFD-induced obese CRC tumor-bearing mouse model and a free fatty acid (FFA)-treated CRC cell model to investigate AhR function and mechanism underlying in HFD-related CRC.

**Results and discussion:**

We found that HFD significantly promoted CRC progression, accompanying with activation of AhR with upregulated level of kynurenine (KYN) synthesis-related enzymes and increased KYN levels in tumor tissue and peripheral blood. The specific inhibition of AhR alleviated tumor growth and metastasis in tumor-bearing mouse with HFD treatment. *In vitro*, FFA treatment induced AhR activation with high level of KYN secretion, which enhanced CRC cell growth, invasion and epithelial-mesenchymal transition (EMT). Genetic knockdown of AhR reversed FFA-induced malignant phenotypes. Quantitative proteomic analysis identified that inositol polyphosphate 4-phosphatase type II (INPP4B) might be a potential target for AhR function. Mechanistically, activated AhR directly transcriptionally upregulated INPP4B to trigger AKT/SGK3 pathway activation, and knockdown of INPP4B abolished AhR activation-induced oncogenic effects. In conclusion, AhR promotes HFD-related subcutaneous tumor growth and invasion/migration-related phenotypes of CRC via activating the INPP4B/AKT/SGK3 pathway, which may provide a potential target for the prevention or treatment of HFD-associated CRC.

## 1 Introduction

Colorectal cancer (CRC) imposes a substantial global health burden and remains a leading contributor to cancer-associated mortality worldwide. The global burden of CRC continues to increase, partly in parallel with increasing consumption of high-fat, calorie-rich diets (1, 2). Although advances in screening, diagnosis and treatment have improved disease management, many patients still present with advanced-stage disease because of the lack of reliable early symptoms (3). CRC is also characterized by aggressive progression, frequent metastasis and high recurrence rates (4), while conventional chemotherapy causes severe adverse effects and available targeted therapy targets remain limited. As a result, the long-term survival and overall prognosis of CRC patients remain unsatisfactory, making it urgent to explore novel molecular mechanisms of CRC progression and identify potential therapeutic targets.

Emerging evidence has demonstrated that a high fat diet (HFD) and obesity not only increase CRC incidence risk, but also accelerate tumor progression through metabolic reprogramming and dysregulated lipid metabolism. Excessive lipid accumulation and imbalanced fatty acid metabolism have been shown to promote CRC cell proliferation, invasion, and immune evasion (5). However, the molecular mechanisms underlying HFD-associated CRC progression remain incompletely understood.

Aryl Hydrocarbon Receptor (AhR), a ligand activated member of the basic helix loop helix Per ARNT Sim (bHLH PAS) transcription factor family (6). AhR is highly expressed in intestinal and functions as an important molecular sensor linking environmental, dietary and metabolic signals to gene transcription programs. The classical AhR signaling is activated when exogenous or endogenous ligands bind to cytoplasmic AhR, triggering conformational changes that expose its nuclear localization signal. Upon activation, AhR translocates to the nucleus, where it regulates the transcription of downstream target genes (7). Accumulating evidence has identified AhR as a central regulator of metabolic adaptation, inflammatory signaling, tumor progression and cellular plasticity, indicating its key role in maintaining physiological homeostasis, especially for intestine (8). AhR interacts with gut microbiota-derived metabolites and dietary components to protect intestinal stem cell and epithelial barrier integrity. In addition, AhR modulates intestinal permeability and alleviates inflammatory injury, thereby contributing to intestinal barrier repair and intestinal homeostasis (9). Consequently, increasing attention has been directed toward the involvement of AhR in intestinal inflammation, barrier dysfunction and colorectal tumorigenesis, highlighting its potential as a therapeutic target.

Recent studies have increasingly implicated AhR signaling in cancer development and progression across a broad range of malignancies. Aberrant AhR activation has been shown to facilitate malignant phenotypes by enhancing cellular proliferation, migratory and invasive capacities, as well as epithelial mesenchymal transition (EMT), through the modulation of multiple downstream signaling networks in lung, prostate, breast and renal cancers, as well as glioma and melanoma (10–16). In addition, AhR can induce immunosuppression and drive immune escape within the tumor microenvironment, ultimately exerting clear pro-tumor effects. Among the endogenous ligands of AhR, the tryptophan metabolite kynurenine (KYN) has been identified as an important activator that promotes the progression of multiple tumors via AhR activation (13). In addition to direct pro-tumor effect, activation of the IDO/KYN/AhR signaling axis has also been reported to regulate the tumor immune microenvironment. KYN-mediated AhR activation suppresses anti-tumor immunity and promotes immune tolerance by regulating CD4^+^ T cell function and FoxP3^+^ regulatory T cells through the AhR-FoxP3 pathway (17–19).

Increasing evidence has highlighted AhR as an important regulator of CRC development and progression. A significant positive correlation between the tryptophan metabolic enzyme TDO2 and AhR in CRC tissues was reported, and TDO2 knockdown suppresses CRC progression by blocking the TDO2/KYN/AhR signaling axis (20). In addition, CRC cells upregulate tryptophan transporters (SLC1A5, SLC7A5) and metabolic enzymes like AFMID to promote KYN production and induce AhR nuclear translocation, enhancing tumor cell proliferation and migration (21). Moreover, elevated expression of AhR in colorectal tumors has been increasingly linked to metabolic reprogramming, a hallmark of cancer progression. Functional studies have demonstrated that disruption of AhR signaling impairs tumor cell growth, accompanied by reduced ATP generation and alterations in fatty acid metabolism (5). AhR also interacts extensively with classical oncogenic pathways, including Wnt/β catenin and c-Myc, and regulates cell cycle, proliferation and tumor microenvironment immunity to drive CRC progression. Given AhR can respond to various endogenous metabolites and alters lipid metabolism, it is reasonable to speculate that AhR plays an important role in HFD-associated CRC (22). However, the specific role of AhR and downstream molecular mechanisms underlying HFD-related CRC remain to be clarified.

INPP4B (inositol polyphosphate 4 phosphatase type II) functions as a critical modulator of phosphoinositide signaling by regulating the intracellular balance between PI(3,4)P2 and PI(3)P. SGK3, a member of the AGC protein kinase family, contains a PX domain that binds to PI(3)P produced by INPP4B, thereby activating downstream signaling pathways and promoting tumor progression through inhibition of tumor suppressors such as NDRG1 (23). Recent evidence indicates that INPP4B expression is significantly increased in CRC tissues, and elevated INPP4B expression is associated with unfavorable clinical outcomes, supporting a potential oncogenic role for INPP4B in CRC progression (24). However, whether AhR participates in HFD induced CRC through regulation of the INPP4B/AKT/SGK3 signaling pathway has not yet been reported.

Taken together, AhR functions in both tumor progression and metabolic regulation, but its role and underlying mechanisms in HFD-associated CRC remain unclear. Present study aims to establish an obese tumor bearing mouse model using a HFD and an *in vitro* colon cancer cell model induced by fatty acids. Using these complementary *in vivo* and *in vitro* models, we examined the contribution of AhR to CRC progression and evaluated whether AhR promotes the tumor-promoting effects of a HFD through the INPP4B/AKT/SGK3 signaling pathway. This work provides mechanistic insight into the molecular link between HFD induced metabolic alterations and CRC progression, and highlights potential targets for CRC prevention and therapeutic intervention.

## 2 Materials and methods

### 2.1 Reagents and assay kits

Free fatty acids containing oleic acid (HY-N1446) and palmitic acid (HY-N0830) (at a 2:1 volume ratio), drug doxorubicin (HY-15142, 5 mg/kg) were obtained from MCE (Shanghai, China). Cell Counting Kit-8 (CK100001) and the specific AhR antagonist CH-223191 (C101299, 10 or 20 mg/kg/d) was purchased from Chemegen (Shanghai, China). Analytical commercial kits, including TC (A111-1-1), TG (A110-1-1), HDL-C (A112-1-1) and LDL-C (A113-1-1) were bought from Nanjing Jiancheng (Nanjing, China). The primary antibodies utilized for protein detection encompassed anti-AhR (Proteintech, 67785-1-1g), anti-INPP4B (Abclonal, A16461), anti-AKT (Proteintech, 10176-2-AP), anti-p-AKT (Ser473) (Proteintech, 664444-1-1G), anti-p-AKT (Thr308) (CST, 13038S), anti-SGK3 (Servicebio, GB11983), anti-p-SGK3 (Thr320, Absin, abs172853), anti-Ki67 (Beyotime, AB2008), anti-MMP2 (Proteintech, 10373-2-AP), anti-MMP9 (Abcam, Rm1020), anti-CD31 (Abclonal, A19014), anti-Zeb1 (Proteintech, 21544-1-AP), anti-E-cadherin (CST, 14472S), anti-N-cadherin (CST, 13006S), anti-Vimentin (CST, 5741S), anti-Histone H3 (Proteintech, 17168-1-AP) and anti-*β*-actin (Proteintech, 669009-1-1g). Targeted small interfering RNAs (siINPP4B, siNC) and short hairpin RNAs (shAhR, shNC) were synthesized and sequence-verified by GenePharma (Shanghai, China).

### 2.2 Animal model

Male C57BL/6J mice (7–8 weeks old) were maintained under specific pathogen-free conditions with controlled temperature and humidity. Mice were fed either a standard chow diet or a high-fat diet (HFD) for 3 months to induce obesity-associated metabolic phenotype. The HFD used in this study is the obesity model diet (D12492) from SPF Biotechnology Co., Ltd. (SFD014, Beijing), which is a widely recognized and peer-validated diet for establishing diet-induced obesity rodent models. This diet is formulated with standardized raw materials including 31.7% lard, 25.8% casein, 8.9% sucrose and etc., which delivers 60% of total calories from fat. For calorie intake regulation, we strictly controlled the feeding process: all animals were raised in separate cages, the daily food intake of each rat was accurately recorded, and the total calorie intake of the HFD group and the control group was calculated and matched to exclude the interference of inconsistent total energy on the experimental results. Subsequently, 8 × 10^5^ CT26 colorectal cancer cells suspended in 0.2 mL PBS were inoculated subcutaneously into the left axillary region to establish the a subcutaneous ectopic xenograft tumor model. During the *in vivo* experiment, one mouse in the HFD group suffered an unexpected accidental death 2 days before the scheduled terminal sampling. We collected its tumor tissue and all corresponding biological samples for subsequent downstream detection. All tumor volume data included in the quantitative growth curve were recorded completely and accurately throughout the administration period before this accidental death, and all collected samples from this mouse have been included in the subsequent biochemical detection analysis.

Animal experiments were conducted in accordance with institutional guidelines and were approved by Ethics Review of Animal Experiments from Animal Care and Use Committee of the General Hospital of Northern Theater Command (2026022).

### 2.3 *In vivo* pharmacological interventions

After subcutaneous tumor nodules reaching 50 mm^2^, mice in corresponding pharmacological treatment groups continuously received the AhR antagonist CH-223191 (10 or 20 mg/kg) or DOX (5 mg/kg) dissolved in saline via standard intraperitoneal administration for 2 weeks daily. The mice in vehicle groups were administrated with equivalent saline. CH223191 was dissolved in DMSO for stock solution (16.67 mg/mL). For working solution preparation for *in vivo* administration, stock solution (600 *µ*L) was dissolved in PEG300 (2400 *µ*L) containing Tween 80 (300 µL), which was then homogeneously diluted with saline (2700 *µ*L). The DOX drug powder was dissolved in saline for stock solution (0.833 mg/mL).

### 2.4 Animal physiological monitoring and biological specimen collection

Following the visual and palpable appearance of the subcutaneous tumor nodules, tumor dimensions were systematically recorded every other day, and the total tumor volume was computed using the geometric equation: Volume = 1/2 × length × width^2^. Systemic metabolic fluctuations were rigorously monitored; total body weight was measured utilizing a standardized scale. Fasting blood glucose levels were quantified using an automated glucometer following a 12-hour fasting period via tail vein puncture, wherein the initial blood droplet was wiped away to ensure accuracy. Serum lipid parameters, including total cholesterol (TC), triglycerides (TG), high-density lipoprotein cholesterol (HDL-C), and low-density lipoprotein cholesterol (LDL-C), were determined using corresponding commercial assay kits. Eighteen days after tumor cell inoculation, mice were anesthetized and peripheral blood samples were obtained from the orbital venous plexus. Following clot formation at room temperature for 4 h, samples were centrifuged at 5,000 *g* for 10 min at 4 °C to separate the serum fraction. Tumor tissues were collected immediately after sacrifice, frozen in liquid nitrogen, and preserved at -80 °C until further analysis.

### 2.5 Immunohistochemistry analysis

Paraffin-embedded tissue specimens were sectioned and incubated at 60 °C for 2 h prior to staining. Slides were deparaffinized with xylene (two 5-min cycles) and rehydrated through a descending ethanol gradient (100%, 100%, 95%, 95%, 85%, and 75% for 10 s each), followed by a 1-min immersion in distilled water. Antigen retrieval was performed utilizing a citrate buffer solution via a high-pressure cooker (timed for 2 min after reaching peak pressure) or microwave heating (high power for 10 min), after which the slides cooled naturally to room temperature. Endogenous peroxidase activity was quenched by incubation with a blocking reagent for 15 min. The sections were subsequently incubated with primary antibodies against Ki67 (1:200), MMP2 (1:200), MMP9 (1:200), and CD31 (1:200) overnight at 4 °C or for 30 min at room temperature, followed by incubation with the appropriate secondary antibodies at 37 °C for 30 min. Antibody binding was detected using a DAB substrate solution, and nuclei were counterstained with hematoxylin. Following differentiation in acid alcohol, the sections were dehydrated, rendered transparent in xylene, and mounted using neutral resin for microscopic examination. For each individual animal, we randomly selected three non-overlapping high-power fields (40× magnification) from its IHC tissue section, quantified the target protein positive signal (integrated optical density, IOD, or percentage of positive stained cells) in each field independently, then calculated the mean value of these multiple fields as the single aggregated representative data point for that animal. The entire IHC quantification process was performed in a fully blinded manner: all tissue slides were relabeled with anonymous random numbers before scanning and image analysis, the researchers who operated the ImageJ quantification and calculated the aggregated values for each animal had no access to the group allocation, treatment information or experimental hypothesis of the samples during the whole process. The group information was unblinded only after all quantitative results were fully recorded and archived.

### 2.6 Cell culture

Homo sapiens (Human) Colon Tumor 116 (HCT116) and BALB/c Mouse Colon Tumor 26 (CT26) were obtained from Cell Bank of the Chinese Academy of Sciences (Shanghai, China) and maintained in complete growth media within a humidified incubator containing 5% CO_2_ at 37 °C. Two cell lines are verified by STR and mycoplasma test performed by a certified third-party institution (Zhongqiao Xinzhou Biotechnology CO., Ltd., Shanghai, China). Two cell lines match with the reference profile from ATCC, DSMZ, JCRB and so on without intra or inter-species cross contamination or mycoplasma detected. To simulate a high-fat microenvironment *in vitro*, cells in the exponential growth phase were seeded into culture plates. Upon cellular attachment, the original culture medium was replaced with media containing 1 mM FFA mixture composed of oleic acid and palmitic acid at a 2:1 volume ratio for 24 h. In parallel, cells were selectively treated with exogenous KYN at precise concentrations of 0, 200, and 400 *μ*M to explore ligand-dependent AhR activation.

### 2.7 Cell transfection

Cells (1.5 × 10^5^) cells per well were seeded into 6-well plates. The cells were cultured in an incubator at 37 °C with 5% CO_2_ for 12 h. Prior to transfection, cells were gently washed twice with 1 mL of PBS per well. The prepared siRNA (siINPP4B, siNC, 100 pmol/well), shRNA (shAhR, shNC, 2 *μ*g/well) or overexpression plasmid (OE-AhR, OE-NC, 2 *μ*g/well) were transfected using Lipofectamine™ 8000 (Beyotime) into the culture medium, gently mixed, and further incubated for 48 h. The siINPP4B and shAhR sequences were listed in **Supplementary Table 1**.

### 2.8 Cell proliferation, migration, and invasion assays

Cellular viability HCT116 and CT26 cells treated with FFA for 24, 48, and 72 h. FFA was dissolved in 10% BSA (fat acid free) solution and diluted with DMEM. Add 1% BSA (fat acid free) as the solvent control group. Cellular proliferation was measured utilizing the CCK-8 assay. A freshly prepared CCK-8 reaction solution (CCK-8 reagent: culture medium = 1:10) was added to the 96-well plate, followed by incubation for 2 h at 37 °C. Cell viability was subsequently assessed by determining the absorbance at 450 nm using a microplate reader. Cell viability was measured using following formula: Cell viability (%) =100 × (A_450, sample_-A_450, blank_)/(A_450, control_-A_450, blank_).

Cellular migration was examined using a wound-healing scratch assay by recording the cellular migration area at 0 h and 24 h, while invasion capabilities were meticulously evaluated utilizing Matrigel-coated Transwell chambers.

### 2.9 Detection of KYN

The concentration of kynurenine (KYN) in serum, colon cancer tissues and CT26 cell supernatant was quantitatively measured utilizing a specific KYN ELISA kit (CUSABIO, CSB-E13659h), and the level of KYN in HCT116 cells was measured using a KYN ELISA kit (FineTest, EM1862), according to the manufacturer’s instructions.

### 2.10 Cell immunofluorescence

HCT116 and CT26 cells cultured on glass coverslips were subjected to FFA or control treatments, subsequently fixed with 4% PFA, and permeabilized with Triton X-100. After blocking, the coverslips were incubated with the anti-AhR (1:200) primary antibody overnight at 4 °C. Fluorophore-conjugated secondary antibodies were applied in the dark, and cellular nuclei were definitively counterstained using DAPI. The merged fluorescent signals demonstrating AhR nuclear translocation were captured using a Olympus IX81 microscope.

### 2.11 Dual-luciferase reporter assay

To validate the direct transcriptional regulation of INPP4B by AhR, a dual-luciferase reporter assay system was established. The ~ 2.1 kb promoter region (spanning -2000bp to +100 bp relative to the TSS) of the INPP4B gene was cloned into the pGL3-Basic reporter vector. HEK293T cells were co-transfected with corresponding reporter plasmids and AhR overexpression construct. Relative luciferase activity was measured 36 h post-transfection, with 3 independent biological replicates conducted. The promoter sequence and AhR expression plasmid sequence are listed in **Supplementary Table 1**.

### 2.12 Quantitative real-time PCR

Total RNA was isolated from tumor tissues or cultured cells using RNAiso Plus reagent (Takara, Tokyo, Japan) according to the manufacturer’s instructions. RNA samples were subsequently reverse-transcribed into cDNA using the PrimeScript™ RT-PCR Kit (Takara) in 10 *μ*L reaction mixtures at 37 °C for 15 min, followed by enzyme inactivation at 85 °C for 5 s. Quantitative real-time PCR analysis was conducted on an ABI 7500 Fast Real-Time PCR System (Thermo Fisher Scientific, Rockford, IL, USA) using PrimeScript™ RT Master Mix (Takara). Amplification was initiated with denaturation at 94 °C for 2 min, followed by 40 cycles consisting of 95 °C for 5 s and 60 °C for 10 s. Gene expression levels were normalized to GAPDH and quantified using the comparative 2^^-ΔΔCt^ method. ΔΔCt=(Ct, target - Ct, reference)_Experimental_ - (Ct, target - Ct, reference)_Control_. The relative fold change of genes was calculated using the formula 2^^-ΔΔCt^. Primer sequences are listed in **Supplementary Table 2**.

### 2.13 Nuclear and cytoplasmic protein extraction

The nuclear and cytoplasmic protein fractions were independently isolated utilizing a commercial Nuclear and Cytoplasmic Protein Extraction Kit (Beyotime, Shanghai, China). Tissues or cultured cells were harvested, resuspended in cytoplasmic extraction buffer containing protease inhibitors, and incubated on ice to induce cell membrane lysis. Following centrifugation, the supernatant comprising the cytoplasmic fraction was collected. The remaining intact nuclei were subsequently lysed using a high-salt nuclear extraction buffer, sonicated, and centrifuged to acquire the highly purified nuclear protein fraction. Histone H3 and *β*-actin were strictly utilized as the internal loading controls for the nuclear and cytoplasmic fractions, respectively.

### 2.14 Western blotting

Tumor tissues preserved at -80 °C or collected cell pellets were processed for total protein extraction using RIPA lysis buffer (Beyotime, Shanghai, China) supplemented with protease inhibitors (Solarbio, Beijing, China). Samples were mechanically disrupted under liquid nitrogen and subsequently incubated on ice for 30 min to ensure complete protein release. After clarification by centrifugation at 12,000 *g* for 10 min at 4 °C, the supernatants were collected and protein concentrations were determined using the Bradford method. Protein samples were diluted with PBS as required and combined with 5× loading buffer at a ratio of 4:1 prior to electrophoresis. Equivalent amounts of protein were resolved by SDS-PAGE and electrotransferred onto methanol-activated PVDF membranes under a constant current of 200 mA for 90 min. Membranes were blocked in 5% skim milk for 1 h at room temperature, followed by incubation with primary antibodies at 4 °C overnight and the corresponding HRP-labeled secondary antibodies for 1–2 h at room temperature. Protein signals were detected using an enhanced chemiluminescence (ECL) substrate and subsequently analyzed.

### 2.15 Proteomic mass spectrometry and pathway enrichment analysis

To systematically screen for differentially expressed proteins (DEPs) and identify potential signaling pathways driving tumor progression, comprehensive proteomic mass spectrometry analysis was conducted on tumor tissues excised from both the normal diet control mice (ND-TB) and the HFD-induced obesity CRC murine models (HFD-TB) (n=3 per group). The proteomic mass spectrometry analysis was conducted by Majorbio platform. Protein (200 *μ*g for each sample) digestion was performed with FASP method. The peptide was desalted with C18 StageTip for further LC-MS analysis. Total proteins were meticulously extracted, followed by standardized preparation, separation, identification, and precise quantitative analysis via mass spectrometry. Using MaxQuant software version 1.6.0.16, the MS data were examined and compared to the UniProtKB Rattus norvegicus database. Multiple quality control indicators including peptide spectrum match (PSM) false discovery rate (FDR < 1%), peptide coverage and protein unique peptide count are strictly verified. Proteins that had a *P*-value< 0.05 and a fold change of ≥1.5 were categorized as significantly differentially regulated. The differential expression profiles between the two groups were visually represented utilizing volcano plots. Ingenuity Pathway Analysis (IPA) was employed to analyze the DEPs, successfully identifying the top 10 significantly enriched signaling pathways. Additionally, the PANTHER classification system was utilized to rigorously categorize the significantly altered proteins across cellular components, molecular functions, and biological processes. Key differentially expressed genes were screened, and Gene Ontology (GO) and KEGG enrichment analyses were conducted to validate the specific AhR-mediated signaling pathways.

Omics and Text based Target Enrichment and Ranking (OTTER, http://otter-simm.com/otter.html) is a novel computational approach for enriching targets from omics data of investigated chemicals. OTTER first performs text mining on the differentially expressed genes (DEGs) in PubMed abstracts related to keywords.

### 2.16 Bioinformatics data retrieval and processing

Transcriptome RNA-sequencing data and corresponding clinical annotations for 258 patients diagnosed with colon adenocarcinoma (COAD) were systematically downloaded from the official data portal of The Cancer Genome Atlas (TCGA) database (https://portal.gdc.cancer.gov/), (https://portal.gdc.cancer.gov/)). To evaluate prognostic relevance, the clinical dataset was stratified into an INPP4B high-expression cohort (n = 129, 50.0%) and an INPP4B low-expression cohort (n = 129, 50.0%) using the median mRNA expression value of 12.77 fpkm (ranging from 1.50 to 265.65 fpkm) as the definitive grouping threshold.

The GSE datasets were obtained from NCBI Gene Expression Omnibus (GEO) database. GSE72970 dataset contains transcriptomic expression profiles of primary tumors from 124 metastatic colorectal cancer (mCRC) patients, all of whom have detectable INPP4B expression. The entire cohort is OS-ready, with complete, well-annotated overall survival follow-up data available for all 124 included cases. The public GEO dataset GSE161158 contains transcriptomic expression profiles of colorectal cancer, with a total of 191 DFS-ready cases that have complete, well-annotated disease-free survival follow-up data, among which 46 confirmed DFS events were recorded during the follow-up period. Use GPL570 platform RMA standardized chip expression values from the series matrix. INPP4B is mapped to four probes (1563833-at, 205376-at, 223878-at, 235046uat) by platform annotation, and the mean is taken and summarized. OS and RFS establish analysis sets based on corresponding follow-up fields, and records without corresponding outcomes, expressions, or model covariates are not included in this endpoint or model. Survival analysis was performed by pre grouping based on the median expression within the team, using Kaplan Meier method, log rank test, and Cox proportional hazards model. Multivariate model adopts a complete case analysis and tests the hypothesis of proportional hazards. The effective sample size for different endpoints and models is determined based on the completeness of the corresponding outcomes and covariates.

We strictly set the P value from the Cox proportional hazards model as the only threshold for significance judgment. Prior to formal statistical testing, we verified the proportional hazards (PH) assumption via log cumulative hazard function plots, confirming that INPP4B expression fully meets the constant hazard ratio premise required for Cox regression analysis. We also adopted a symmetric top/tail grouping rule, ensuring that the proportion of samples included in both the high-expression and low-expression subgroups is no less than 25% of the total cohort size. This rule eliminates the statistical bias caused by arbitrary cut-off setting, and makes the grouping strategy fully reproducible for follow-up related studies. For the significance threshold setting in this study, only the Wald P-value derived from the Cox proportional hazards model was adopted as the exclusive criterion, with the significance cutoff defined as Cox P < 0.05. The log-rank P-value was only treated as an auxiliary reference for the Kaplan-Meier (KM) curve visualization, and was not applied as a variable screening basis at any stage of the analysis.

### 2.17 Survival analysis and Cox proportional hazards regression modeling

Overall survival (OS) was analyzed using Kaplan-Meier survival curves, and differences between the high and low INPP4B expression groups were compared using the log-rank test. To determine the independent prognostic significance of INPP4B expression, univariate and multivariate Cox regression analyses were performed with adjustment for clinicopathological variables, including age, sex, TNM stage (III/IV), lymph node metastasis, and perineural invasion. Time-dependent Receiver Operating Characteristic (ROC) curves were generated to assess predictive accuracy across 1-year, 3-year, and 5-year survival intervals.

### 2.18 Statistical analysis

All statistical analyses and graphical representations were executed utilizing GraphPad Prism 8.0 software. Continuous experimental variables were evaluated using the two-tailed Student’s *t*-test for comparisons between groups, while non-parametric clinical variables were analyzed via the Mann-Whitney U test. The two-way ANOVA was used to evaluate the change of body weight or tumor volume. Categorical datasets, including lymph node metastasis and perineural invasion, were systematically compared using the Chi-square test. The Gaussian normal distribution of the datasets was pre-evaluated utilizing the Shapiro-Wilk test. A two-sided *P*-value < 0.05 was defined as indicating a statistically significant difference across all analyses.

## 3 Results

### 3.1 High-fat diet enhances obesity-related metabolic profiles and accelerates colorectal cancer progression *in vivo*

**Figure 1:**
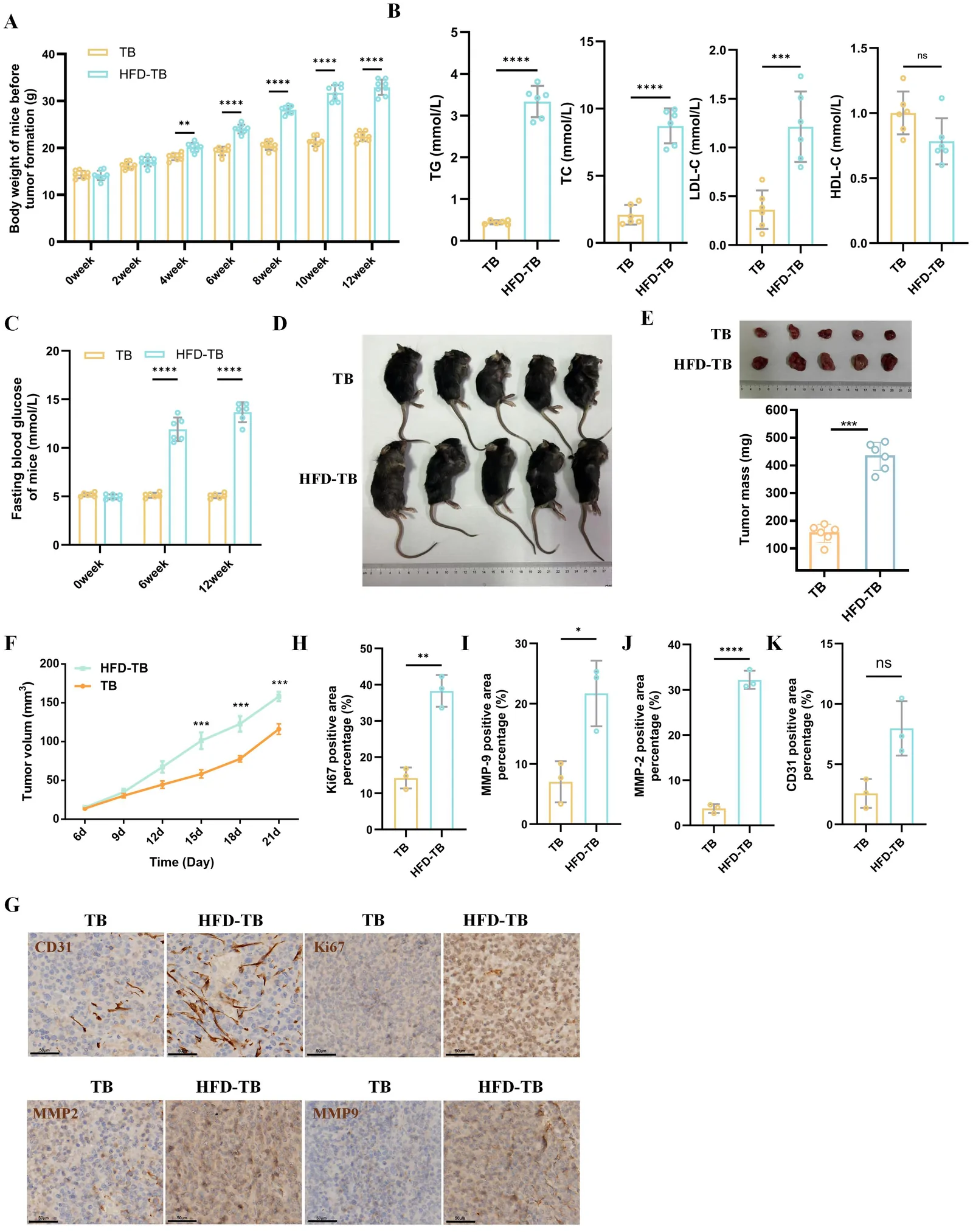
Effects of HFD on systemic metabolism and colorectal cancer development *in vivo*. **(A)** Longitudinal body weight growth curves of mice over the course of dietary intervention. The two-way ANOVA comparison was used to evaluate the change of body weight. **(B)** Serum lipid profiles including triglycerides (TG), total cholesterol (TC), low-density lipoprotein cholesterol (LDL-C), and high-density lipoprotein cholesterol (HDL-C) in mice from the control and HFD groups. **(C)** Fasting blood glucose levels measured at the experimental endpoint. The two-way ANOVA comparison was used to evaluate the glucose levels. **(D)** Representative macroscopic photographs showing the overall physiological appearance of the mice. **(E)** Representative photographs of excised tumor tissues and corresponding statistical quantification of tumor mass. **(F)** Longitudinal tumor volume growth curves monitored over time (n=6). The two-way ANOVA comparison was used to evaluate the change of tumor volume. **(G)** Representative photographs of immunohistochemical (IHC) staining for Ki67, MMP9, MMP2, and CD31 in harvested tumor sections (n=3 fields per animal). Scale bar = 50 *µ*m. Quantitative evaluation and statistical analysis of the positivity rates or densities for Ki67 **(H)**, MMP9 **(I)**, MMP2 **(J)**, and CD31 **(K)**. The two-tailed Student’s *t*-test was used for comparisons in other groups. Data are presented as the mean ± standard deviation (SD) (n=6 per group). ^*^P < 0.05, ^**^P < 0.01, ^***,****^P < 0.001 vs. the TB group; NS, no significance (Student’s *t*-test).

To systematically evaluate the impact of HFD on systemic metabolism and the progression of colorectal cancer *in vivo*, a mouse model of diet-induced obesity with colorectal tumor xenografts was established. Progressing in weight gain after dietary intervention, lipid profiling was shown to be imbalanced with high levels of triglycerides (TG), total cholesterol (TC), and low-density lipoprotein cholesterol (LDL-C) in serum (**Figure 1A, B**). Consistently, the fasting blood glucose levels were prominently elevated in the HFD group, indicating the development of metabolic impairment (**Figure 1C**). The representative photographs of the mice further supported the metabolic disorder in HFD mice with expanded tumor appearance (**Figure 1D**). Measurement of endpoint tumor weights and morphological analysis of the excised tumor tissues demonstrated that HFD significantly increased both tumor volumes and weights (**Figure 1E**). Tumor growth curves further confirmed this enhanced proliferation phenotype, revealing a markedly faster growth trajectory in the HFD group (**Figure 1F**).

The impact of HFD on tumor histopathology was further confirmed by IHC staining. The percentage of Ki67-positive cells, a definitive indicator of cell proliferation, was markedly increased in tumors from HFD-fed mice (**Figure 1G, H**). To determine the metastatic and invasive potential of the tumors, the expression levels of matrix metalloproteinase 2, 9 (MMP-2, 9) were quantified. The levels of MMP-9 and MMP-2 were significantly upregulated in the HFD group (**Figure 1G, J-I**). However, there is no statistically significant difference in tumor-associated angiogenesis was observed (**Figure 1K**). Collectively, these results robustly demonstrate that HFD alters systemic lipid and glucose metabolism, thereby profoundly promoting colorectal cancer progression phenotypes.

### 3.2 HFD activates the KYN/AhR signaling pathway in colorectal cancer tissues

**Figure 2:**
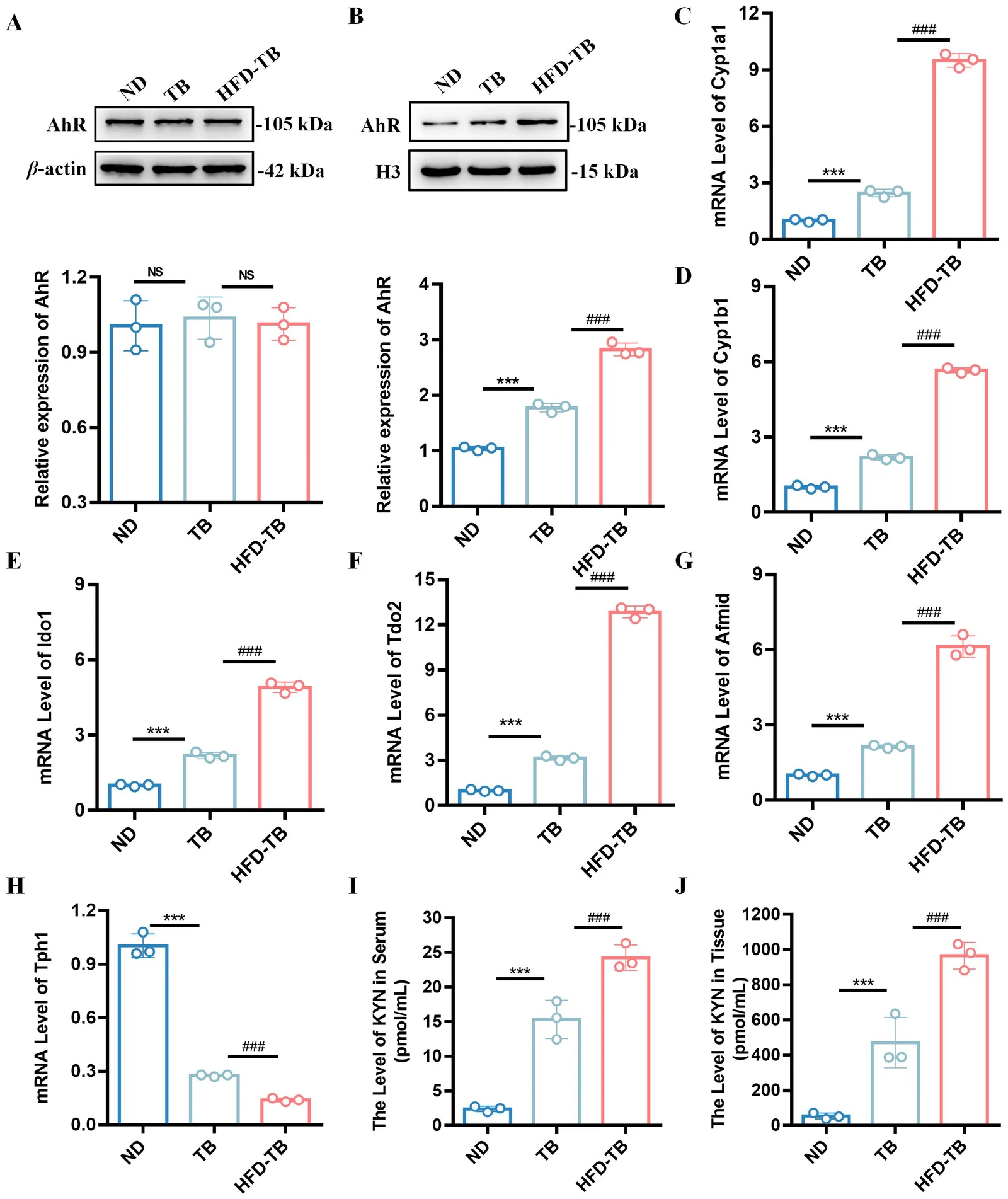
Activation of the KYN/AhR signaling axis in tumor tissue under HFD conditions *in vivo*. **(A, B)** Western blot analysis and quantification of total AhR protein expression in normal diet (ND) colon tissue, tumor-bearing (TB) colon tissues and tumor-bearing colon tissues from HFD-treated mice (HFD-TB). *β*-actin was used as a loading control. **(B)** Western blot analysis and quantification of nuclear AhR protein levels. Histone H3 (H3) was utilized as a nuclear loading control. **(C, D)** Quantitative PCR analysis of the mRNA expression levels of AhR downstream target genes, including *Cyp1a1* and *Cyp1b1*. **(E–H)** Quantitative PCR analysis of the mRNA levels of *Ido1*, *Tdo2*, *Afmid* and *Tph1*. **(I, J)** Detection of KYN concentrations in colon tumor tissues **(I)** and serum **(J)** using commercial detection kits. Data are presented as the mean ± SD (per group). ***P < 0.001 vs. the ND group; ^###^*P* < 0.001 vs. the TB group. NS, no significance. (n=3, One-way ANOVA).

The activation of aryl hydrocarbon receptor (AhR) signaling cascade was further examined during HFD-exacerbated colorectal cancer progression. Although the total level of AhR remained unchanged, the nuclear level of AhR was prominently increased in the tumor tissues, especially in the tumor tissues from HFD-treated mice (**Figure 2A, B**). Consistent with the enhanced nuclear accumulation of AhR, the mRNA expression levels of its classical downstream transcriptional target genes, *Cyp1a1* and *Cyp1b1*, were also significantly upregulated in the tumor tissues from HFD-treated mice (**Figure 2C, D**), further confirming the activation of the AhR signaling pathway. Since AhR is a ligand-activated transcription factor, we further quantified expression of key rate-limiting enzymes in the kynurenine (KYN) synthesis pathway (which generates well-characterized endogenous AhR ligands). The mRNA level of indoleamine 2,3-dioxygenase 1 (Ido1), tryptophan 2,3-dioxygenase (Tdo2), and arylformamidase (Afmid) were significantly increased in the tumor tissues, especially in the tumor tissues from HFD-treated mice (**Figures 2E–G**). Conversely, the mRNA level of tryptophan hydroxylase 1 (Tph1), an enzyme responsible for shifting tryptophan metabolism toward the serotonin pathway, exhibited a marked downregulation or distinct suppression in the HFD group (**Figure 2H**). In alignment with the transcriptional upregulation of KYN-synthesizing enzymes, the concentrations of KYN were profoundly elevated in the colorectal tumor tissues and the serum from HFD-treated mice (**Figure 2I, J**). Collectively, these data strictly demonstrated HFD further promoted the activation of KYN/AhR pathway in colon cancer tissues.

### 3.3 FFA induce KYN/AhR pathway activation in colorectal cancer cells

**Figure 3:**
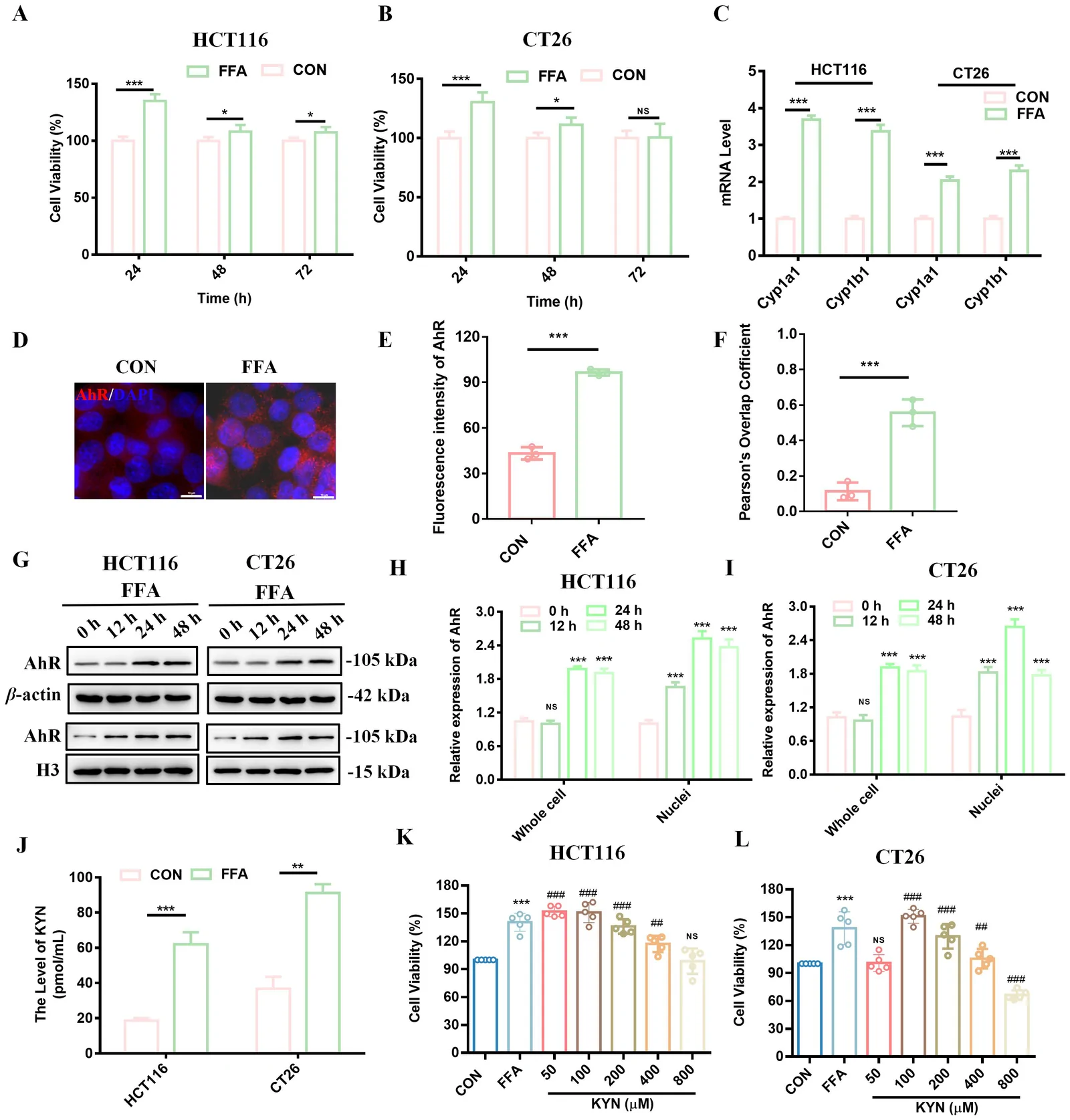
FFA stimulates AhR nuclear translocation and downstream transcriptional activation *in vitro*. **(A, B)** Cellular viability HCT116 and CT26 cells treated with FFA for 24, 48, and 72 h. FFA was diluted using an equal volume of 10% BSA solution (fatty acid free) and added into DMEM. Add 1% BSA (fatty acid free) as the solvent control group. The two-way ANOVA comparison was used to evaluate the cell viability (n=5). **(C)** Quantitative PCR analysis of *Cyp1a1* and *Cyp1b1* mRNA levels following FFA intervention (24 h). The one-way ANOVA comparison was used to evaluate the cell viability (n=3). **(D)** Representative immunofluorescence staining of AhR (Red) in cells after FFA treatment. Scale bar = 50 *µ*m. **(E, F)** The intensity of AhR and nuclear distribution were quantified (n=3 from three independent cell crawling views). The two-tailed Student’s *t*-test was used for comparisons. **(G–I)** Western blot analysis and quantification of total and nuclear AhR in cells treated with FFA for 0, 12, 24, and 48 h (n=3). **(J)** The concentration of KYN in cells treated with FFA (n=3). **(K–L)** Cellular viability of HCT116 and CT26 cells treated with different doses of KYN (n=3). The one-way ANOVA comparison was used to evaluate in **(H–L)**. Data are presented as the mean ± SD (per group). ^*^P < 0.05, ^**^P < 0.01, ***P < 0.01 vs. CON; ***P < 0.01 vs. 0 h; ^##^*P* < 0.01, ^###^*P* < 0.001 vs. the FFA group. NS, no significance.

To further evaluate the effect of high-fat exposure on colorectal cancer cells and the activation of KYN/AhR pathway, murine CT26 and human HCT116 cells were treated with free fatty acids (FFA). The results showed that FFA treatment significantly promoted the proliferation of cells at different time point, especially for 24 h after treatment (**Figure 3A, B**). FFA treatment significantly upregulated the mRNA levels of *Cyp1a1* and *Cyp1b1* in both cell lines (**Figure 3C**), and induced AhR nuclear translocation (**Figures 3D–F**). Furthermore, we found that FFA promoted the expression and nuclear level of AhR in a time-dependent manner, especially for 24 h after treatment (**Figures 3G–I**). Consistently, we found that FFA treatment also significantly promoted the production of KYN in both cells (**Figure 3J**). Consistent with the effect of FFA treatment, KYN treatment also significantly promoted the viability of cells, indicating FFA-induced activation of the KYN/AhR pathway is critical for the growth of colorectal cancer cells.

### 3.4 Inhibition of AhR attenuates FFA-induced proliferation, invasion, migration, and EMT in colorectal cancer cells

**Figure 4:**
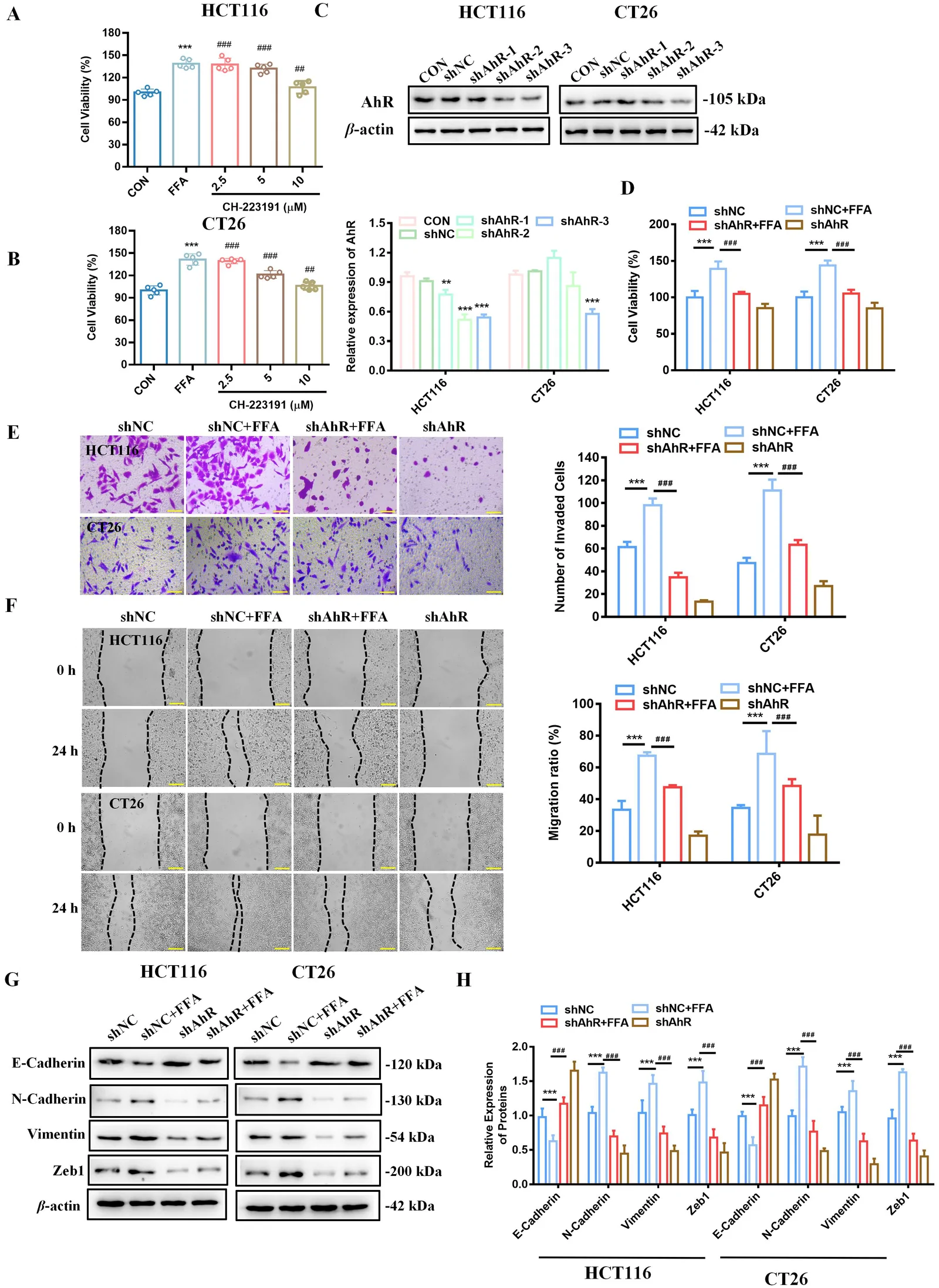
Pharmacological inhibition or genetic knockdown of AhR suppresses FFA-induced proliferation, invasion, migration and EMT. **(A, B)** CCK-8 assay showing the viability of **(A)** HCT116 and **(B)** CT26 cells treated with FFA and different concentration of CH-223191 (2.5, 5, 10 μM) (n=5). **(C)** The protein level of AhR in cells transfected with shNC or different sequences of shAhR (n=3). **(D)** CCK-8 assay validating cell proliferation following transfection with shNC or shAhR under baseline or FFA treatment conditions in both cell lines (n=5). shNC intervention as a control group. **(E)** Representative microscopic images and statistical quantification of transwell invasion assays showing the invasive capacity across different genetic groups (scale bars, 100 *μ*m) (n=3). **(F)** Representative images and quantitative analysis of wound healing assays tracking the migration ratio at 0 and 24 h post-scratching (scale bars, 200 *μ*m) (n=3). **(G, H)** Western blot analysis and quantification of epithelial-mesenchymal transition (EMT)-associated markers (E-cadherin, N-cadherin, Vimentin, and ZEB1) across different intervention cohorts, with *β*-actin utilized as an internal loading control (n=3). Data are presented as the mean ± SD (per group). ***P < 0.001 vs. the CON; **P < 0.01, ***P < 0.001 vs. shNC group; *^##^P* < 0.01, ^###^*P* < 0.001 vs. the FFA group; ^###^*P* < 0.001 vs. shNC + FFA group (One-way ANOVA).

We further clarify whether FFA-driven malignant phenotypes are directly dependent on the functional activation of the AhR signaling cascade. The administration of CH-223191, a selective pharmacological inhibitor of AhR, significantly abrogated the FFA-increased cell proliferation in both cell lines in a dose-dependent manner (**Figure 4A, B**). In alignment with the pharmacological inhibition data, transfection with shAhR with 50% knockdown efficiency prominently suppressed the accelerated cell proliferation triggered by FFA exposure in both HCT116 and CT26 cell lines (**Figure 4C, D**). FFA treatment markedly enhanced the number of invaded colorectal cancer cells, however, this enhancement was significantly attenuated upon shRNA intervention (**Figure 4E**). Furthermore, to determine the longitudinal cell motility, scratch assays were performed. The data revealed that the increased migration ratio induced by FFA exposure was robustly compromised following AhR silencing, leading to a broader wound area at 24 h post-scratching relative to the FFA group (**Figure 4F**).

Given that the epithelial-mesenchymal transition (EMT) serves as a critical prerequisite for tumor cell invasion and migration, the regulatory role of the AhR pathway in FFA-induced EMT was subsequently evaluated. The results revealed that FFA exposure led to a substantial reduction in the epithelial marker E-cadherin, accompanied by a simultaneous upregulation of mesenchymal markers, including N-cadherin, Vimentin, and ZEB1 (**Figure 4G, H**). Notably, shAhR effectively reversed these alterations, leading to the restoration of E-cadherin expression and the distinct suppression of N-cadherin, Vimentin, and ZEB1 protein levels under FFA-treated conditions (**Figure 4G, H**). Collectively, these results firmly demonstrate that AhR is an essential regulator for FFA-induced proliferation, invasion, migration, and EMT in colorectal cancer cells.

### 3.5 Pharmacological inhibition of AhR suppresses HFD-promoted colorectal cancer growth and aggressive progression *in vivo*

**Figure 5:**
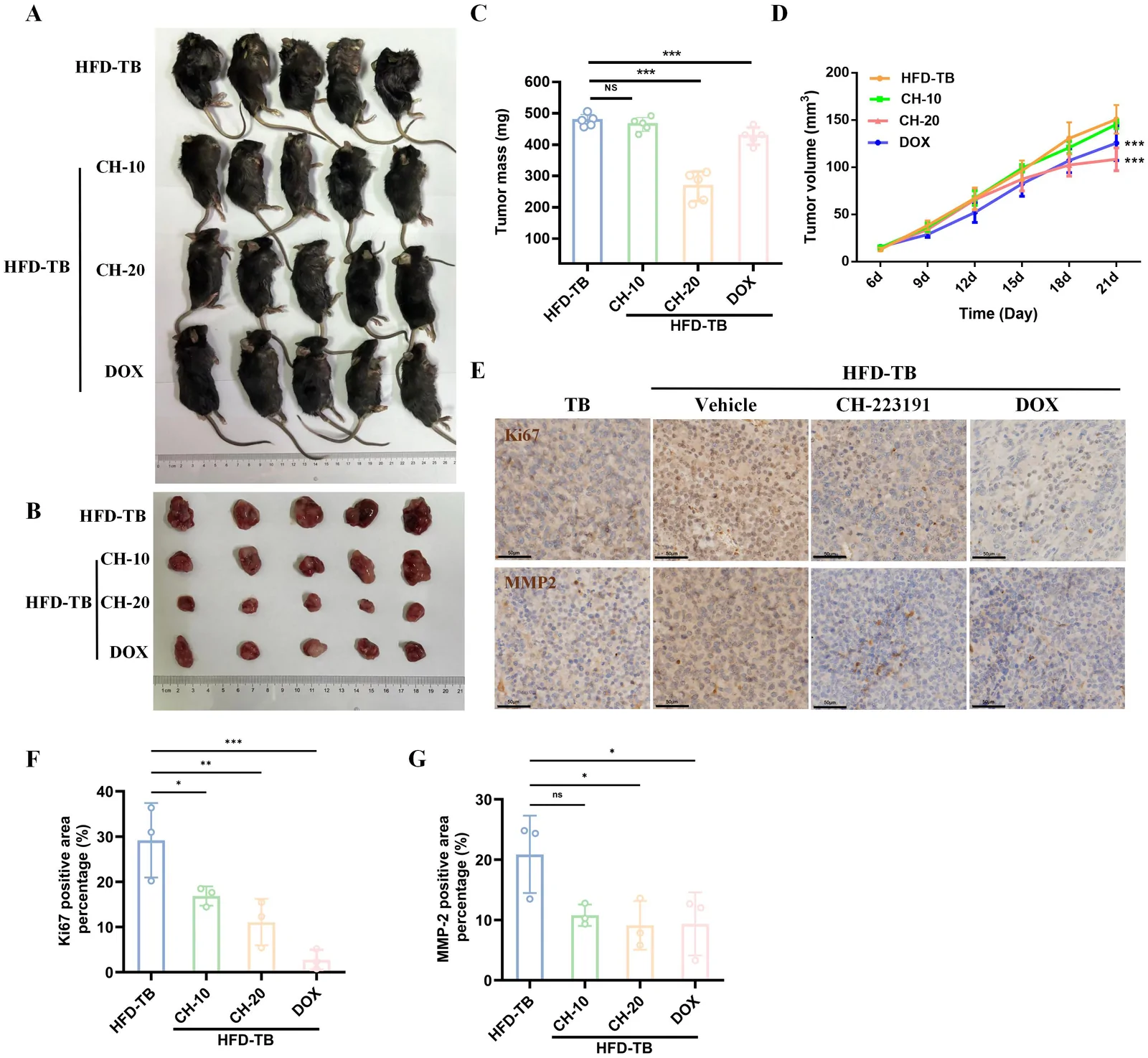
Pharmacological blockade of AhR restricts HFD-accelerated colorectal cancer growth and aggressive phenotypes *in vivo*. **(A, B)** Representative photographs capturing the overall physiological and tumor appearance of HFD-fed tumor-bearing mice following a 2-week administration of vehicle (Saline), CH-223191 (10 or 20 mg/kg/d), or Doxorubicin (DOX; 5 mg/kg) (n=5). HFD mice treated with saline as a vehicle control groups and DOX treatment as a positive control group to systemically evaluate the role of CH223191 in CRC progression. **(C)** Representative photographs of excised tumor tissues and corresponding statistical quantification of tumor mass. **(D)** Longitudinal tumor volume growth curves monitored over time. The two-way ANOVA comparison was used to evaluate the tumor volume (n=5). **(E, F)** Representative photographs of the excised tumor masses and the corresponding statistical analysis of the Ki67-positive cell percentage determined via IHC staining. **(E, G)** Statistical analysis of the final tumor weights and representative IHC staining with quantitative area analysis for MMP2. The one-way ANOVA comparison was used to evaluate in **(C, F, G)** Data are presented as the mean ± SD (per group). *P < 0.05, **P < 0.01 and ***P < 0.001 vs. the HFD-TB group. NS, no significance.

To firmly validate the functional role of the AhR signaling pathway in driving HFD-induced colorectal cancer progression *in vivo*, a rescue experiment was performed in HFD-fed mice bearing colorectal tumor xenografts by administering CH-223191, alongside Doxorubicin (DOX) as a positive control drug. The general physiological condition and macroscopic alterations of the experimental mice were first evaluated (**Figure 5A**). Visual inspection of the excised tumor masses revealed that both CH-223191 administration (20 mg/kg/d) and DOX treatment (5 mg/kg) markedly reduced the tumor size and volume compared with the HFD vehicle group (**Figures 5B–D**). In alignment with these macroscopic observations, quantitative immunohistochemical (IHC) analysis demonstrated that the percentage of Ki67-positive cells was significantly decreased in the tumor tissues of mice treated with CH-223191 or DOX relative to the HFD vehicle counterparts (**Figure 5E, F**). To evaluate tumor invasive capacity at the histological level, IHC staining for matrix metalloproteinase-2 (MMP2) was performed. Statistical evaluation revealed that the MMP2-positive staining area was notably suppressed following AhR inhibition or DOX administration, indicating an impairment existing in extracellular matrix degradation (**Figure 5E, G**). Collectively, these *in vivo* findings indicate that targeting the AhR pathway effectively counteracts HFD-promoted colorectal cancer growth, proliferation, and invasive potential.

### 3.6 Proteomic profiling and bioinformatic analysis identify INPP4B as a key downstream target of AhR in HFD-induced colorectal cancer

**Figure 6:**
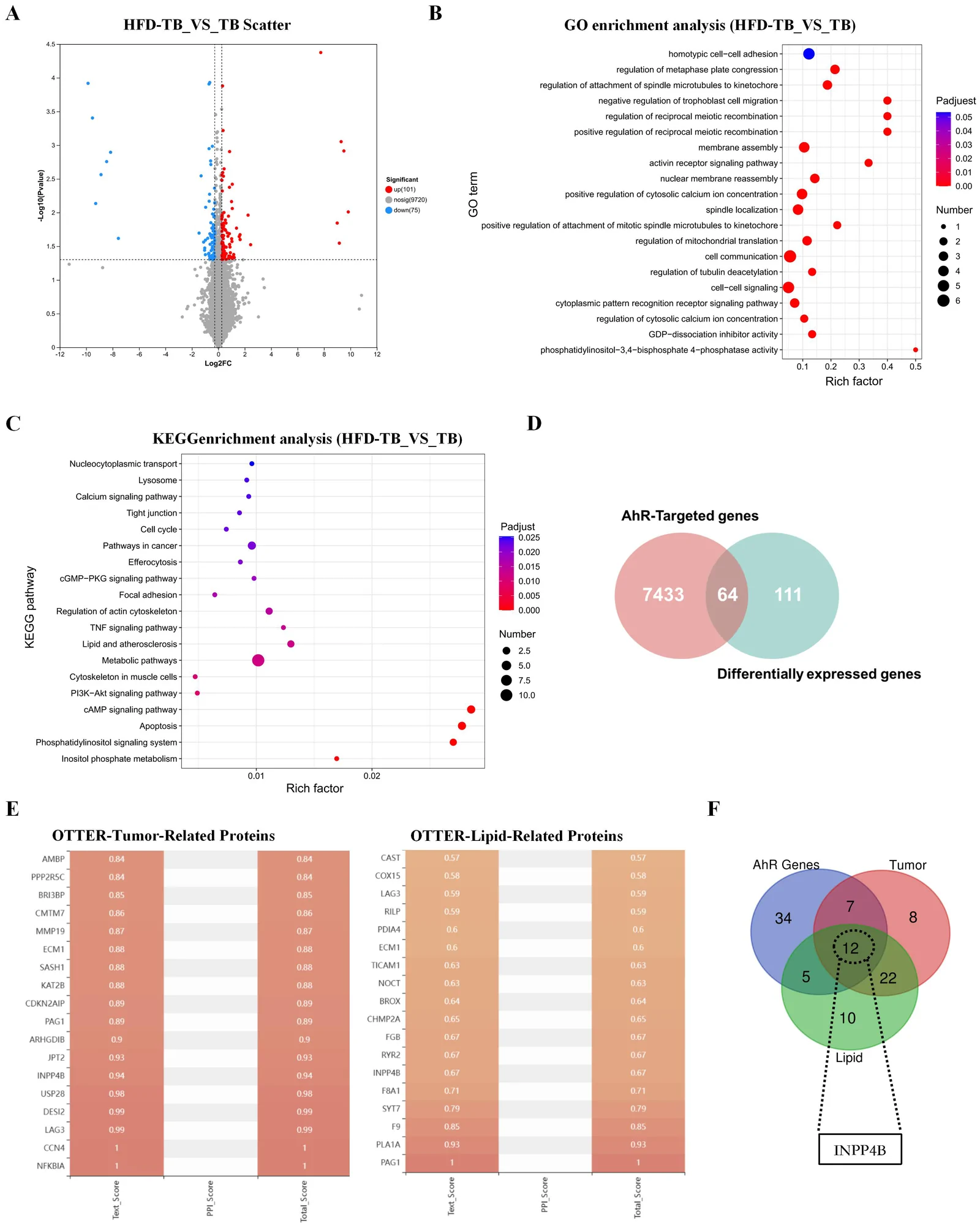
Quantitative proteomic analysis and bioinformatic workflows identifying INPP4B as an AhR-related target under HFD stress. **(A)** Volcano plot analysis mapping the 176 differentially expressed proteins (DEPs), distinguishing 101 upregulated (red) and 75 downregulated (blue) proteins in HFD tumor tissues relative to normal control tumors (n=3 per group). **(B)** Gene Ontology (GO) functional enrichment analysis classifying the core biological processes of the identified DEPs. **(C)** Kyoto Encyclopedia of Genes and Genomes (KEGG) pathway enrichment analysis illustrating the primarily modified signaling networks. **(D)** Two-way Venn diagram demonstrating the intersection between the identified DEPs and known AhR target genes, revealing 64 overlapping candidates. **(E)** OTTER sorting algorithm heat map screening and ranking core proteins associated with tumor development and lipid pathways. **(F)** Three-way Venn diagram indicating the definitive intersection among AhR target genes, tumor-associated genes, and lipid-associated genes, narrowing down the candidates to 12 core differential genes including INPP4B.

To systematically elucidate the global molecular changes and identify the primary downstream effectors driving HFD-induced colorectal cancer progression, high-throughput quantitative proteomic analysis was performed on the harvested colorectal tumor tissues. Volcano plot analysis was first utilized to screen for differentially expressed proteins (DEPs) (listed in **Supplementary Table 3**) between the normal diet tumor tissues (TB) and HFD-induced tumor tissues (HFD-TB). Based on the cut-off criteria, a total of 176 DEPs were identified, including 101 significantly upregulated proteins and 75 significantly downregulated proteins in the HFD group (**Figure 6A**). To explore the biological functional attributes of these DEPs, Gene Ontology (GO) functional enrichment analysis was conducted, which revealed that the DEPs were predominantly involved in metabolic reprogramming, cellular proliferation, and migration-related biological processes (**Figure 6B**). Concurrently, Kyoto Encyclopedia of Genes and Genomes (KEGG) pathway enrichment analysis was performed to distinguish the primary signaling pathways activated under HFD stress, highlighting significant enrichment in lipid metabolism pathways, PI3K/AKT pathway, Lysosome, Actin cytoskeleton, and oncogenic receptor signaling cascades (**Figure 6C**).

To further narrow down the critical downstream targets regulated by the AhR pathway within this HFD-responsive proteome, an intersection analysis was performed. As shown in the Venn diagram in **Figures 6D** cross-referencing the identified DEPs with established AhR downstream target genes yielded an overlapping consensus of 64 co-enriched differential proteins. To refine these candidates based on clinical and functional relevance, the OTTER sorting algorithm was applied to score and rank the upregulated DEPs, specifically extracting core proteins closely associated with tumor progression and lipid metabolism pathways (**Figure 6E**). Subsequently, a three-way Venn diagram analysis was performed to intersect the AhR target genes, tumor-associated genes, and lipid metabolism-associated genes identified via the OTTER analysis, which successfully restricted the candidate pool to 12 core differential genes (**Figure 6F**). Comprehensive functional annotation and literature retrieval of these 12 candidates were executed, ultimately identifying Inositol Polyphosphate-4-Phosphatase Type II (INPP4B) as the target of interest due to its specific and potentially unique oncogenic role in colorectal cancer. These sequential proteomic and bioinformatic screenings indicate that INPP4B is a critical AhR-associated target involved in HFD-driven colorectal malignancy.

### 3.7 Clinical pathological characterization and prognostic value of INPP4B expression in patients with colorectal cancer based on bioinformatic analysis

**Figure 7:**
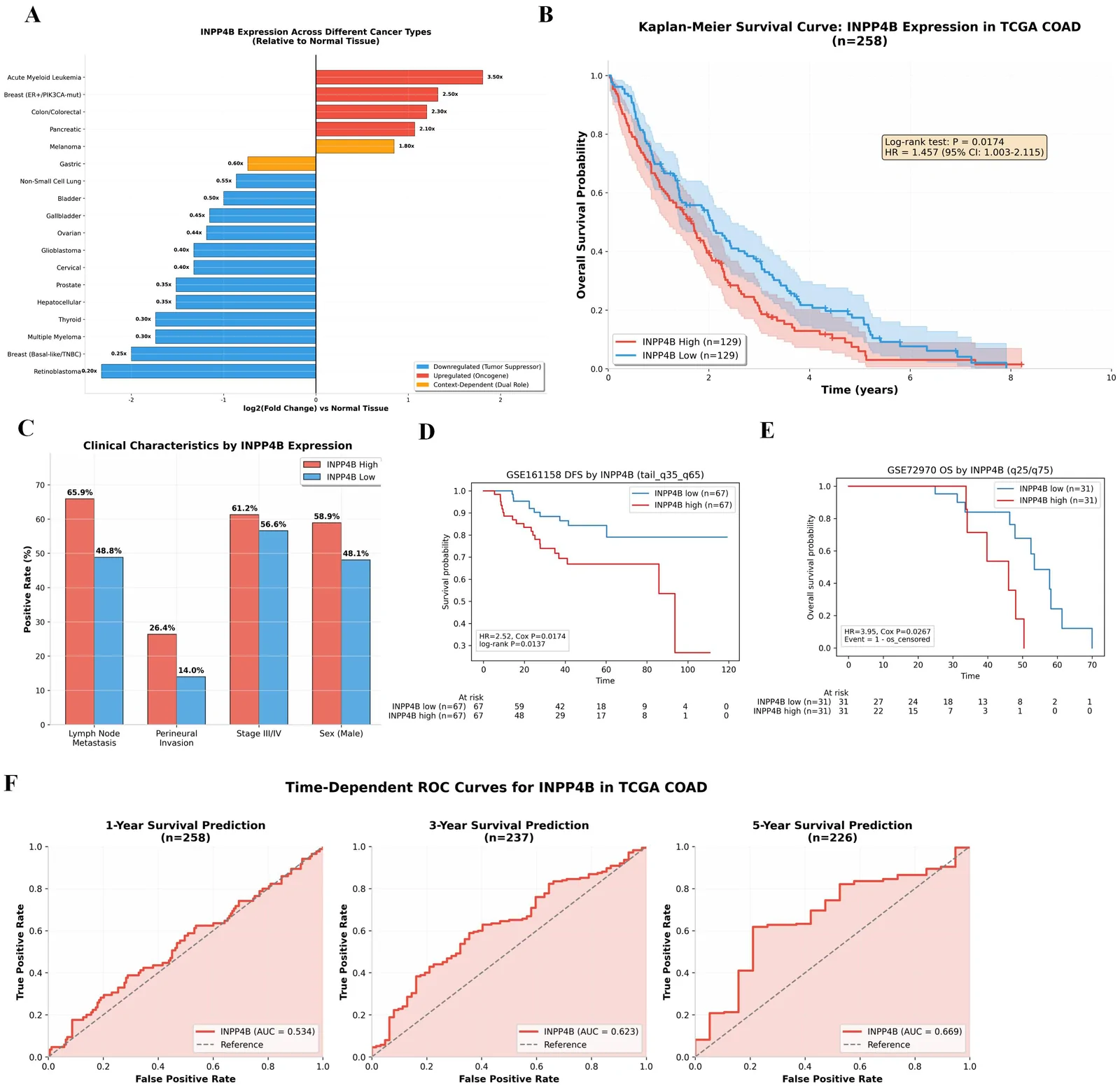
Bioinformatic analysis of INPP4B in patients with colorectal cancer. **(A)** Pan-cancer expression profile of INPP4B across multiple human malignant tissue types extracted from public databases. **(B)** Kaplan-Meier overall survival (OS) curves of patients with colorectal cancer stratified into high (n = 129) and low (n = 129) INPP4B expression cohorts based on the median value within the TCGA COAD dataset. **(C)** Table and statistical charts detailing the correlation between INPP4B expression and clinicopathological characteristics, highlighting significant associations with lymph node metastasis (*P* = 0.008) and perineural invasion (*P* = 0.020). **(D)** Kaplan-Meier OS curves stratified by INPP4B expression level in the GSE72970 cohort. Patients were divided into INPP4B low-expression group (n=31) and INPP4B high-expression group (n=31) using the 25th and 75th percentiles (q25/q75) of INPP4B expression as the cutoff criteria. The number of patients at risk at each follow-up time interval is listed below the survival curve. **(E)** Kaplan-Meier disease-free survival (DFS) curves stratified by INPP4B expression in the GSE161158 cohort. Patients were divided into the INPP4B low-expression group (n=67) and INPP4B high-expression group (n=67), with the top and bottom 35% quantiles (tail_q35_q65) of INPP4B expression level set as the grouping cutoff. The number of patients remaining at risk at each follow-up time interval is summarized in the table below the survival curves. **(F)** Time-dependent receiver operating characteristic (ROC) curves evaluating the prognostic predictive performance of INPP4B expression at 3-year (AUC = 0.623) and 5-year (AUC = 0.669) benchmarks.

To elucidate the clinical relevance and potential prognostic significance of INPP4B in colon cancer, bioinformatic profiling was executed utilizing the TCGA COAD dataset (n=258). The pan-cancer expression profile of INPP4B was first evaluated across various human malignancies. As shown in **Figure 7A**, INPP4B exhibited highly tissue-specific expression patterns, and highly expressed in colon cancer tissues, demonstrating distinct variations between different tumor types and emphasizing its unique functional divergence across diverse oncogenic contexts.

To determine the direct correlation between INPP4B expression and the clinical outcomes of patients with colon cancer, Kaplan-Meier survival analysis with the log-rank test was performed. The cohorts were bifurcated into high and low expression groups based on the median expression level (n=129 per group). The resulting survival curves demonstrated that patients with high INPP4B expression possessed a significantly shorter median overall survival time and a lower survival probability compared with those with low INPP4B expression (**Figure 7B**). To evaluate its independent prognostic capacity, a risk-stratified survival analysis based on univariate and multivariate Cox proportional hazards regression models were conducted (**Supplementary Table 4**). The univariate analysis indicated that high expression of INPP4B is a risk factor for poor prognosis (HR = 1.382, *P* = 0.018). But the independent prognostic value of INPP4B was diluted by strong prognostic factors such as lymph node metastasis and staging, suggesting that INPP4B may indirectly affect prognosis by promoting metastasis in the multivariate analysis ((HR = 1.201, *P* = 0.196). Notably, while the independent prognostic value of INPP4B was partially attenuated when adjusted for strong confounding factors such as tumor stage and lymph node status, these data indicated that INPP4B may impact patient survival indirectly by contributing to tumor aggressive behavior. Consequently, the association between INPP4B expression and classical clinicopathological characteristics was systematically evaluated utilizing the Chi-squared test and Mann-Whitney U test (**Figure 7C**). Statistical quantification revealed that elevated expression of INPP4B was significantly correlated with positive lymph node metastasis (65.9% vs 48.8%, X2 = 6.99, *P* = 0.008) and the presence of perineural invasion (26.4% vs 14.0%, X^2^ = 5.42, *P* = 0.020). Conversely, no significant correlation was observed between INPP4B expression and other clinical parameters, including TNM stage, sex, or age, indicating that INPP4B preferentially drives metastatic and infiltrative phenotypes. In order to Further evaluate the effect of INPP4B on prognosis in CRC, two independent dataset (GSE72970 and GES161158) containing colon and rectal adenocarcinoma data from GPL570 platform were applied. The analysis of GSE72970 showed that the high INPP4B expression group had an increased risk of death (HR = 3.949, 95% CI 1.172-13.304, Cox *P* = 0.0267) (**Figure 7D**). The analysis of GSE161158 showed that the high INPP4B expression group had an increased risk of recurrence/disease progression (HR = 2.524, 95% CI 1.177-5.411, Cox *P* = 0.0174) (**Figure 7E**). These results indicated that INPP4B might be associated with the prognosis of colorectal cancer patients.

Finally, time-dependent receiver operating characteristic (ROC) curve analysis was employed to assess the efficacy of INPP4B expression in predicting long-term survival. The analysis yielded area under the curve (AUC) values of 0.623 at the 3-year survival point and 0.669 at the 5-year survival point, demonstrating a moderated potential predictive performance for patient prognosis (**Figure 7F**). Collectively, these clinical bioinformatic data support the hypothesis that INPP4B acts as an oncogenic driver might associated with aggressive clinical progression and poor prognosis in colorectal cancer.

### 3.8 AhR transcriptionally upregulates INPP4B expression to activate the AKT/SGK3 pathway under lipid stress

**Figure 8:**
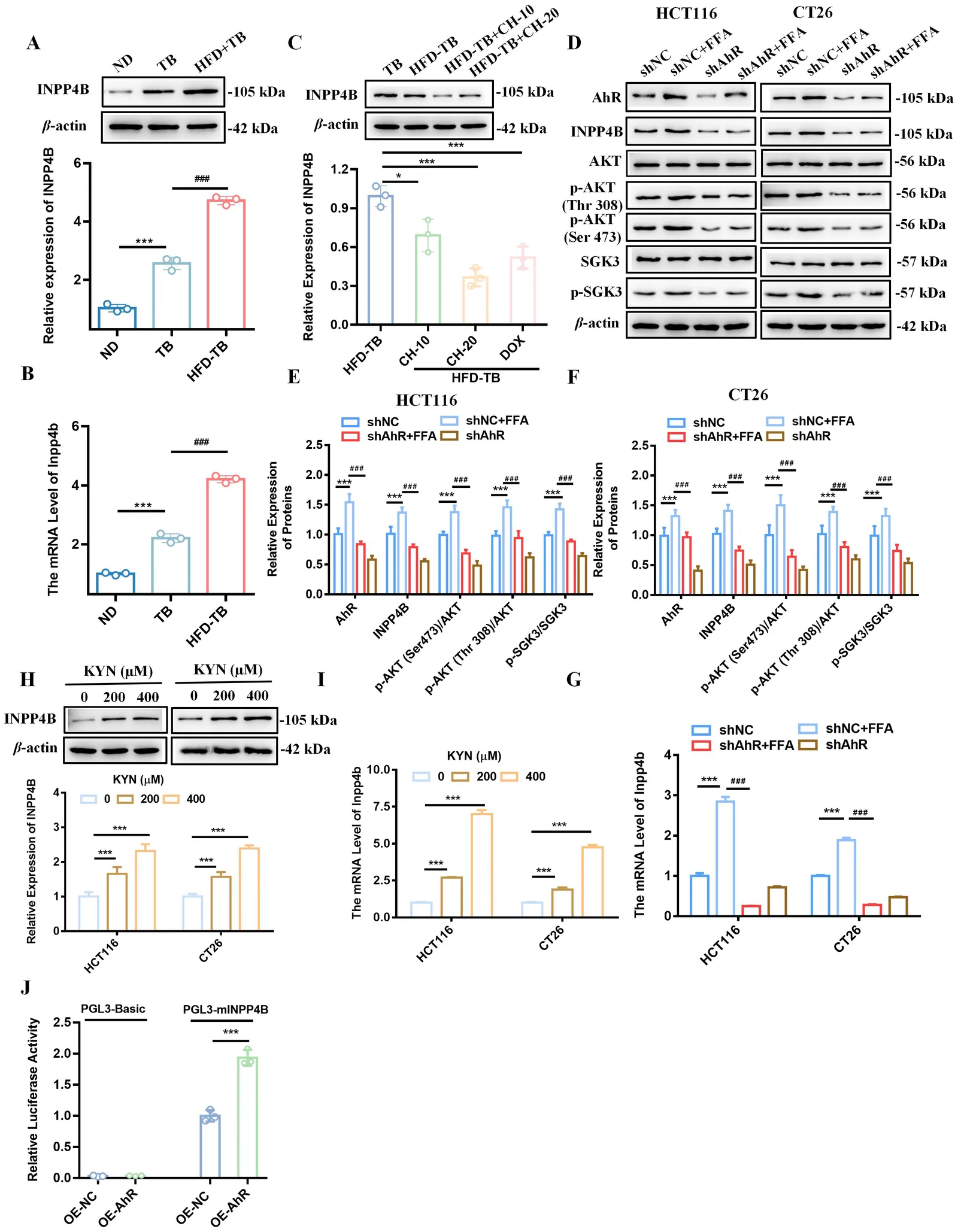
AhR directly regulates the transcription of INPP4B and activates downstream AKT/SGK3 signaling cascades stimulated by lipid. **(A)** Western blot analysis and relative quantitative statistics of INPP4B protein expression in tumor tissues from the normal diet (ND) colon tissue, normal diet (ND) colon tumor tissue (TB), and HFD colon tumor tissue (HFD+TB) groups (n=3). **(B)** Quantitative PCR analysis showing the relative mRNA expression levels of *Inpp4b* in corresponding animal tumor tissues (n=3). **(C)** Western blot analysis and relative quantitative profiles of INPP4B protein levels in HFD-derived tumors following the administration of vehicle, CH-223191 (10 or 20 mg/kg), or DOX (5 mg/kg). **(D)** Western blot analysis of AhR, INPP4B, total AKT, p-AKT (Thr308), p-AKT (Ser473), total SGK3, and p-SGK3 protein levels in HCT116 and CT26 cells transfected with shNC or shAhR under FFA stimulation. **(E, F)** Separate quantitative statistical analysis profiles validating the relative protein expression and phosphorylation ratios derived from the western blot bands in **(D)** for **(E)** HCT116 and **(F)** CT26 cells, respectively (n=3). **(G)** Quantitative PCR analysis tracking *Inpp4b* mRNA expression following AhR silencing under FFA stress (n=3). **(H)** Western blot profiles and corresponding quantification of INPP4B protein levels in cells treated with escalating concentrations of KYN (0, 200, and 400 *μ*M) (n=3). **(I)** Quantitative PCR determination of *Inpp4b* mRNA expression levels regulated by different concentrations of KYN (n=3). **(J)** Relative luciferase activity in colorectal cancer cells co-transfected with pGL3-Basic or pGL3-mINPP4B-promoter plasmids alongside the OE-NC or OE-AhR expression vectors (n=3). Data are presented as the mean ± SD (per group). ***P < 0.001 vs. the ND group; *P < 0.05, ***P < 0.001 vs. HFD-TB group; ***P < 0.001 vs. shNC group; ***P < 0.001 vs. 0 *µ*M group; ^###^*P* < 0.001 vs. the TB group; ^###^*P* < 0.001 vs. the shNC+FFA group (one-way ANOVA followed by Tukey’s *post-hoc* test).

To validate the specific regulatory mechanism through which AhR modulates INPP4B expression in colorectal cancer, a series of *in vivo* and *in vitro* genetic and transcriptional assays were implemented. The results demonstrated that the protein and mRNA level of INPP4B was markedly increased in the HFD-induced tumor tissues of mice compared with those of the normal control counterparts (**Figure 8A, B**). Notably, pharmacological blockade of AhR via CH-223191 resulted in a prominent, dose-dependent decrease in INPP4B protein expression, closely mirroring the patterns observed in the Doxorubicin (DOX) positive control group (**Figure 8C**), suggesting that HFD-driven INPP4B expression is dependent on active AhR signaling.

To further elucidate the downstream signaling consequences of this regulation *in vitro*, genetic knockdown of AhR was performed in colorectal cancer cells under FFA stimulation. Western blot analysis revealed that FFA treatment robustly stimulated the protein expression of both AhR and INPP4B, which subsequently triggered the activation of their downstream effectors, evidenced by the increased phosphorylation levels of AKT (at p-AKT Ser473 and p-AKT Thr308) and SGK3 (**Figure 8D**). Conversely, transfection with shAhR significantly abrogated the FFA-induced elevation of INPP4B protein and prominently suppressed the phosphorylation of both AKT and SGK3 in HCT116 and CT26 cells (**Figure 8D**). Quantitative statistical evaluations of these blotting bands across independent validation sets firmly confirmed the requirement of AhR for FFA-induced INPP4B and AKT/SGK3 pathway activation in HCT116 (**Figure 8E**) and CT26 (**Figure 8F**) cells, respectively. In alignment with the protein alterations, the FFA-stimulated increase in *Inpp4b* mRNA level was effectively reversed upon AhR silencing (**Figure 8G**).

Furthermore, to verify whether the endogenous AhR ligand kynurenine (KYN) can recapitulate the stimulatory effects of lipid stress on INPP4B, cells were incubated with escalating concentrations of KYN. The results demonstrated that KYN treatment induced a prominent and dose-dependent increase in INPP4B protein expression in both cell lines (**Figure 8H**). Concurrently, the transcriptional level of *Inpp4b* mRNA was significantly upregulated by KYN administration in a concentration-progressive manner (**Figure 8I**). Finally, to clarify whether AhR directly controls the transcription of INPP4B by binding to its promoter region, a dual-luciferase reporter gene assay was performed. The luciferase activity analysis revealed that co-transfection of the pGL3-INPP4B-promoter plasmid with the AhR expression vector (OE-AhR) mediated a profound increase in relative luciferase activity compared with the OE-NC control cohorts, whereas no response was observed in the pGL3-Basic empty vectors (**Figure 8J**). Collectively, these findings provide compelling evidence that AhR directly binds to the INPP4B promoter and transcriptionally drives its expression, activating the downstream AKT/SGK3 signaling cascade under a high-fat microenvironment.

### 3.9 Knockdown of INPP4B attenuates KYN-induced proliferation, invasion, migration, and EMT signaling activation in colorectal cancer cells

**Figure 9:**
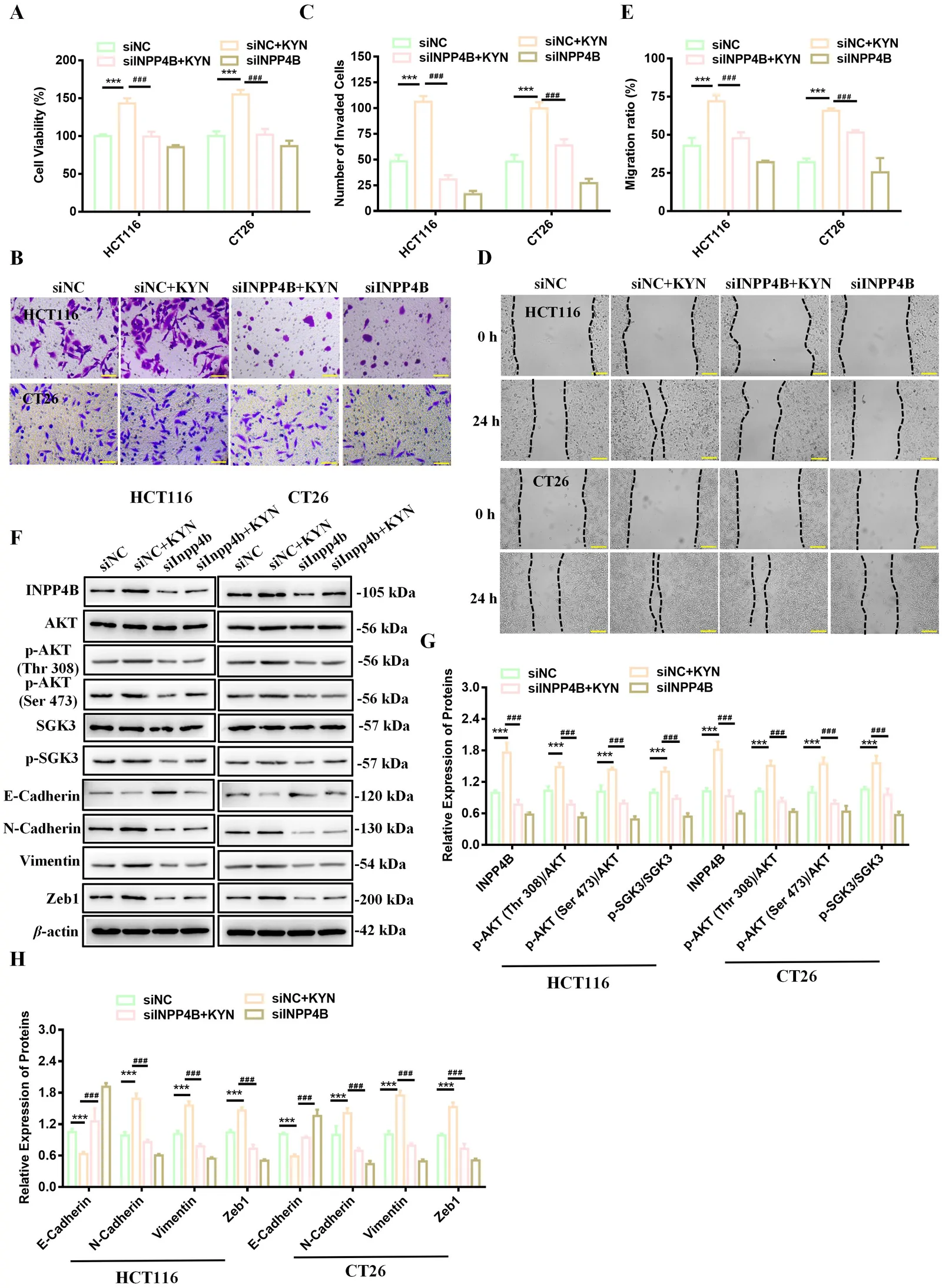
Silencing of INPP4B blocks KYN-induced cell proliferation, invasion, migration, and downstream AKT/SGK3-EMT activation *in vitro*. **(A)** CCK-8 assay showing the viability of HCT116 and CT26 cells across different intervention cohorts including siNC, siNC+KYN, siINPP4B+KYN, and siINPP4B groups (n=5). **(B, C)** Representative microscopic photographs and corresponding statistical quantification of transwell invasion assays (scale bars, 100 *μ*m) (n=3). **(D, E)** Representative microscopic images and statistical evaluation of the migration ratio captured at 0 and 24 h post-scratching during the wound healing assays (scale bars, 200 *μ*m) (n=3). **(F)** The protein expression and phosphorylation level of INPP4B, total AKT, p-AKT (Thr308), p-AKT (Ser473), total SGK3, p-SGK3, E-cadherin, N-cadherin, Vimentin, and ZEB1 in HCT116 and CT26 cells, with *β*-actin utilized as an internal loading control. **(G, H)** Relative quantitative statistical analysis of western blot bands validating the relative expression and phosphorylation ratios for the AKT/SGK3 pathway effectors and the EMT-associated markers across distinct genetic groups (n=3). Data are presented as the mean ± SD (per group). ***P < 0.001 vs. the siNC control group; ^###^*P* < 0.001 vs. the siNC+KYN group (one-way ANOVA followed by Tukey’s *post-hoc* test).

To further elucidate whether kynurenine (KYN)-mediated AhR activation accelerates colorectal cancer progression through the specific upregulation of INPP4B, a series of genetic functional rescue assays were executed *in vitro* using HCT116 and CT26 cell lines. As shown in **Figures 9A**, CCK-8 analysis demonstrated that exogenous KYN treatment significantly enhanced the cell proliferation, however, which was effectively abrogated or suppressed following the transfection of small interfering RNAs targeting INPP4B (siINPP4B). The transwell analysis indicated that KYN administration mediated a profound increase in the number of invaded cells (**Figure 9B, C**). Conversely, genetic ablation of INPP4B via siRNA prominently compromised the KYN-stimulated invasive capacity, reversing the cell count closer to control levels in both cell lines (**Figure 9B, C**). In alignment with these cell invasion data, longitudinal scratch assays revealed that the accelerated wound healing rate and increased migration ratio triggered by KYN exposure were robustly blunted by siINPP4B co-treatment (**Figures 9D, E**).

To clarify the precise signaling mechanisms through which the KYN/AhR/INPP4B axis regulates malignant progression and the metastatic phenotype, western blot analysis was performed to evaluate the activation status of the downstream AKT/SGK3 pathway and epithelial-mesenchymal transition (EMT)-associated markers (**Figure 9F**). The protein expression data revealed that KYN administration significantly upregulated the protein levels of INPP4B, which concurrently enhanced the phosphorylation of AKT (at both p-AKT Thr308 and p-AKT Ser473) and SGK3. This activation was accompanied by a robust induction of EMT signaling, evidenced by the marked downregulation of the epithelial marker E-cadherin and the simultaneous upregulation of mesenchymal hallmarks, including N-cadherin, Vimentin, and ZEB1. Crucially, genetic silencing of INPP4B via siRNA effectively reversed these KYN-mediated protein alterations, resulting in the distinct inactivation of AKT/SGK3 phosphorylation (**Figure 9G**) and the restoration of epithelial phenotype expression over the mesenchymal status (**Figure 9H**). Collectively, these comprehensive loss-of-function data strictly demonstrate that INPP4B is an indispensable downstream molecular node through which KYN-activated AhR signaling coordinates colorectal cancer proliferation, invasion, migration, and EMT.

## 4 Discussion

In the present study, we systematically investigated the role of AhR in HFD-induced CRC progression and explored its potential molecular mechanism. Our results showed that HFD markedly promoted the proliferation, migration, invasion, and EMT of CRC cells, accompanied by abnormal activation of the AhR signaling pathway. Further analysis demonstrated that HFD increased the level of the endogenous AhR ligand Kyn, which promoted AhR nuclear translocation and activation. Activated AhR subsequently increased the transcriptional expression of INPP4B and activated the AKT/SGK3 signaling pathway, ultimately promoting EMT and invasion-related phenotypes of CRC. These findings suggest that AhR may serve as an important molecular link between metabolic disorders and malignant tumor phenotypes, and plays a critical role in HFD induced CRC progression.

AhR has recently attracted increasing attention for its involvement in metabolic regulation. Previous studies have shown that the AhR signaling pathway participates in several key metabolic processes, including fatty acid metabolism and cholesterol synthesis and degradation (5). Obesity induced by a HFD is widely recognized as an important risk factor for CRC. Recent evidence suggests that CRC cells exhibit a strong dependence on fatty acids (25). Fatty acids derived from a HFD, especially animal derived saturated fatty acids, not only provide energy to support tumor cell proliferation but also promote tumor progression by remodeling the tumor microenvironment and suppressing the antitumor activity of immune cells (26). Here we found that inhibition of AhR significantly decreased the CRC progression, but it was uncleared that whether AhR regulated glucose and lipid dysfunction after HFD treatment. The corresponding metabolic profiles are needed to be further investigated.

Recent studies have shown that HFD not only induces obesity and chronic inflammation, but also participates in tumor progression by altering gut microbiota composition and tryptophan metabolism (27, 28). KYN, a major metabolite of tryptophan metabolism, is also a major endogenous ligand of AhR. Increasing evidence has demonstrated that KYN can promote tumor cell proliferation, immune escape, and metastasis in multiple cancers (29). In the present study, we found that HFD markedly increased KYN levels. In contrast, the total protein expression of AhR showed no obvious change, whereas nuclear AhR expression was significantly increased. These findings suggest that HFD mainly promotes AhR activation and nuclear translocation rather than simply increasing AhR expression. Our results are consistent with previous studies showing that the KYN/AhR axis contributes to malignant tumor progression (30). Notably, several microbiota derived indole metabolites, including indole 3 acetic acid (IAA), indole 3 aldehyde (IAld), and indole propionic acid (IPA), can also regulate AhR activity and participate in intestinal homeostasis, inflammation, and tumor regulation (30–32). Since HFD can markedly alter gut microbiota composition, other microbiota derived AhR ligands besides KYN may also contribute to HFD associated CRC progression. The underlying mechanisms still require further investigation.

INPP4B was initially considered a PTEN like tumor suppressor that inhibits tumor progression through negative regulation of the PI3K/AKT signaling pathway. Increasing evidence has demonstrated that INPP4B exhibits bidirectional regulatory effects in different tumor types, and its biological function is highly dependent on tissue context and signaling background (33). In breast cancer, melanoma and prostate cancer, INPP4B is generally considered a tumor suppressor (34–36), whereas in acute myeloid leukemia and CRC, it more frequently exhibits oncogenic effects (37). Recent studies have shown that INPP4B is not simply a negative regulator of AKT signaling. INPP4B can also increase PI(3)P levels and subsequently recruit and activate SGK3, thereby promoting tumor cell survival, migration, and EMT (38–40). Therefore, INPP4B may regulate different PI3K signaling outputs in different tumor contexts. The recently proposed concept of PI3K signaling rewiring suggests that INPP4B can shift PI3K signaling from a classical AKT dependent pattern toward an SGK3 dependent pattern under specific tumor conditions, thereby promoting tumor survival and metastasis (23, 33). As a metabolically active tumor type, CRC frequently exhibits abnormal activation of KRAS, PIK3CA, and related PI3K signaling pathways, which may facilitate this type of signaling remodeling (41). In the present study, AhR activation markedly increased INPP4B expression and was accompanied by activation of the AKT/SGK3 pathway and enhanced EMT. These findings suggest that INPP4B mainly exerts an oncogenic role in our model, which is generally consistent with previous studies reporting the tumor promoting effect of INPP4B in CRC.

EMT is an important biological process that enables tumor cells to acquire invasive and metastatic abilities. Its major characteristics include loss of cell polarity, reduced cell adhesion, and cytoskeleton remodeling (42). Previous studies have shown that AKT/SGK3 signaling not only participates in tumor cell proliferation and survival, but also promotes actin cytoskeleton reorganization and focal adhesion formation through regulation of GSK3β, FAK, and Rho GTPases, thereby facilitating EMT and cell migration (43). In recent years, increasing evidence has suggested that INPP4B is involved not only in PI3K signaling regulation, but also in membrane lipid metabolism and intracellular membrane dynamics. As a phosphoinositide phosphatase, INPP4B catalyzes the dephosphorylation of PtdIns(3,4)P2 to generate PtdIns(3)P, thereby affecting the phospholipid composition of the plasma membrane and endosomal membranes and further regulating vesicle trafficking and membrane dynamics (33). Previous studies have demonstrated that INPP4B can influence lysosomal membrane phospholipid composition as well as lysosome transport, reorganization, autophagy, and exocytosis. In pancreatic ductal adenocarcinoma, INPP4B has been reported to promote cytoskeleton remodeling and tumor metastasis through regulation of lysosomal exocytosis, suggesting that INPP4B may participate in the dynamic interaction between membrane trafficking and cytoskeleton remodeling during tumor cell migration (24). However, whether INPP4B regulates CRC metastasis through a similar mechanism remains unclear. In the present study, knockdown of INPP4B alone markedly suppressed the expression of EMT related genes and reduced the migration and invasion abilities of CRC cells, further supporting the oncogenic role of INPP4B in CRC aggressive progression.

Proteomic analysis in our study showed that HFD significantly affected pathways related to cytoskeleton remodeling, membrane dynamics, lysosomes, and intracellular trafficking, indicating that the AhR/INPP4B signaling axis may participate not only in classical PI3K signaling activation, but also in the regulation of cytoskeleton dynamics and membrane trafficking, thereby promoting CRC cell migration and invasion. Notably, in the cell invasion assay, we observed marked cell aggregation after INPP4B knockdown, suggesting enhanced cell adhesion. Together with the enrichment of cytoskeleton and membrane dynamics related pathways in proteomic analysis, these findings further support the involvement of INPP4B in cytoskeleton remodeling and regulation of cell motility. Based on previous studies, we speculate that under HFD conditions, AhR activation may induce INPP4B expression and subsequently regulate lysosome related membrane lipid metabolism and exocytosis, thereby contributing to cytoskeleton remodeling and EMT progression. However, since the main focus of the present study was the AhR/INPP4B/AKT/SGK3 signaling axis, lysosomal exocytosis, cytoskeleton rearrangement, and related phospholipid metabolic changes were not further investigated. Future studies are still needed to determine whether AhR/INPP4B promotes CRC metastasis through regulation of lysosomal membrane dynamics, membrane lipid metabolism, and cytoskeleton remodeling, which may further improve our understanding of HFD driven metabolic reprogramming and malignant progression in CRC.

In the current study, we did not use dedicated *in vitro* metastatic CRC cell lines (such as the highly metastatic Lovo or SW620 sublines with confirmed high lung/liver metastasis tropism) or *in vivo* orthotopic CRC spontaneous metastasis models. All our *in vitro* functional assays were performed on standard HCT116 and CT26 cell lines, and our *in vivo* validation was based on the subcutaneous ectopic xenograft tumor model. Although we did not use specialized metastatic CRC models, we have evaluated key malignant phenotypes closely related to CRC metastatic progression. We verified the migration/invasion-related phenotypes of HCT116 and CT26 cells using Transwell migration and invasion assays, wound healing assays, and detected the expression of EMT-related markers (E-cadherin, N-cadherin, Vimentin and ZEB1). We also performed IHC staining of MMP2/MMP9 for the above migration/invasion-related phenotypes on subcutaneous xenograft tumor tissues. In the subsequent study, we will introduce clinically validated highly metastatic CRC cell lines and the orthotopic cecum implantation CRC spontaneous metastasis model.

In conclusion, the present study revealed a potential mechanism by which HFD promotes CRC progression. HFD induced the accumulation of the endogenous AhR ligand KYN, which subsequently promoted AhR activation and nuclear translocation. Activated AhR further increased INPP4B expression and stimulated the downstream AKT/SGK3 signaling pathway, ultimately promoting EMT and invasion-related phenotypes of CRC. Our findings further expand the understanding of the role of AhR in tumor metabolic reprogramming and suggest that the AhR/INPP4B signaling axis may serve as a potential therapeutic target for HFD associated CRC. These findings may provide a new insight into the prevention and treatment of metabolism related CRC.

## Data Availability

The datasets presented in this study can be found in online repositories. The names of the repository/repositories and accession number(s) can be found in the article/supplementary material.
