# Supplementary material for: Aryl hydrocarbon receptor promotes the proliferation and aggressive progression of colorectal cancer under high-fat diet via activating INPP4B/AKT/SGK3 signaling pathway
Source: Front Oncol. 2026 Aug 28;16:1907461. doi: 10.3389/fonc.2026.1907461 (PMC13561949; doi:10.3389/fonc.2026.1907461)
Supplement: Supplementary file 1 [file DataSheet1.pdf]

Supplementary Figure 1

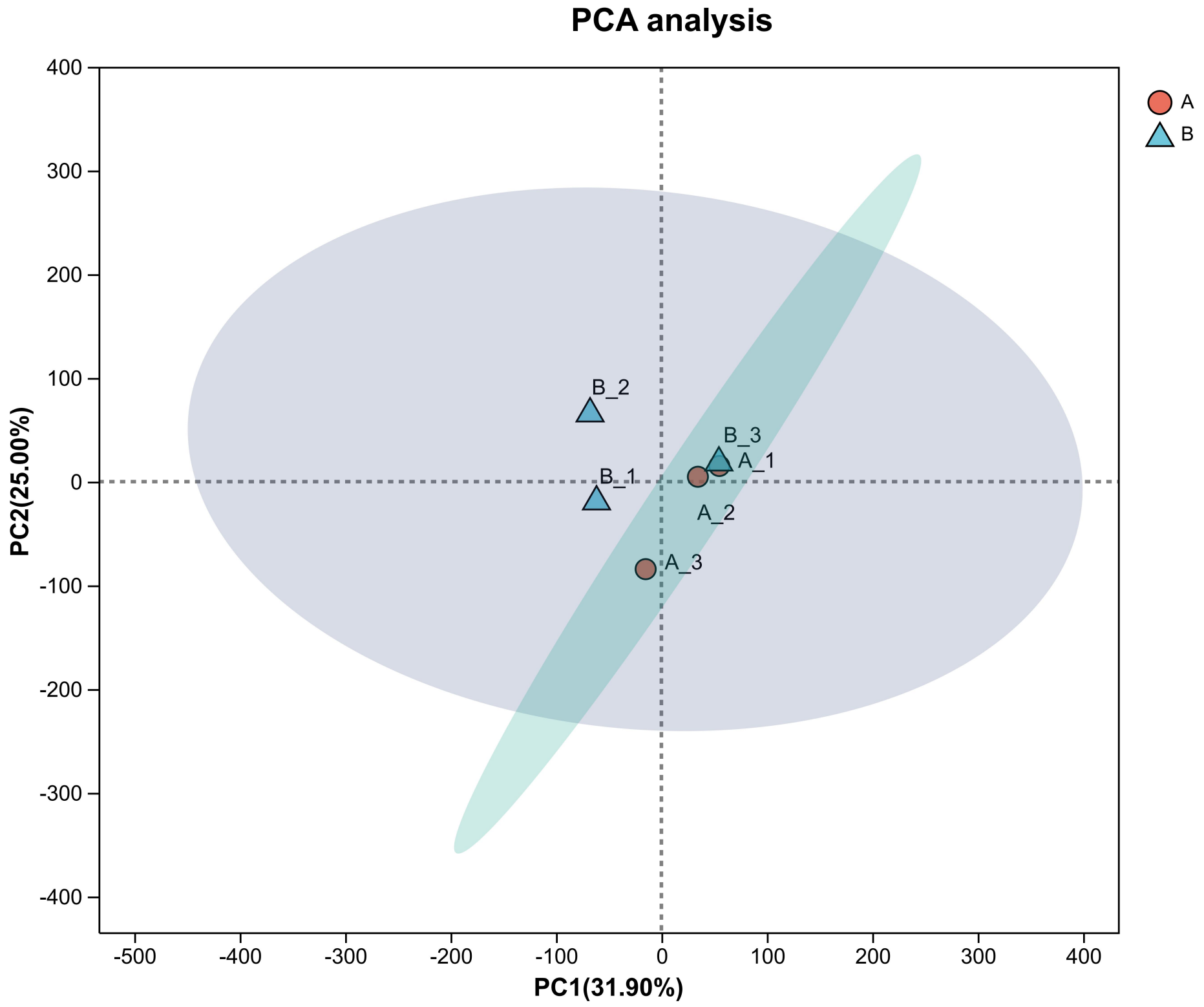

Supplementary Figure 1. The PCA analysis of different expression genes between tumor tissues from normal diet (TB) and high fat diet (HFD-TB). A, represent normal diet; B, represent high fat diet.
