# Supplementary material for: Aryl hydrocarbon receptor promotes the proliferation and aggressive progression of colorectal cancer under high-fat diet via activating INPP4B/AKT/SGK3 signaling pathway
Source: Front Oncol. 2026 Aug 28;16:1907461. doi: 10.3389/fonc.2026.1907461 (PMC13561949; doi:10.3389/fonc.2026.1907461)
Supplement: Supplementary file 2 [file Table4.pdf]

Accession_i	description	gene_name	symbol	entrez_id	protein_na	B_vs_A	sum	cog_descrip
Q8BH24	TM9S4_MC	Tm9sf4	Tm9sf4	99237	Transmemb	no|no chan	0	ENOG410X
A2RSX7	TYW5_MO	Tyw5	Tyw5	68736	tRNA wybu	no|no chan	0	ENOG410X
Q9CZY2	TCAL8_MC	Tceal8	Tceal8	66684	Transcripti	no|no chan	0	ENOG4111
P20934	EVI2A_MC	Evi2a	Evi2a	14017	Protein EV	yes|up	1	ENOG410X
Q8C0S1	DI3L1_MO	Dis3l	Dis3l	213550	DIS3-like e	no|no chan	0	COG0557(c
Q8K0Z7	TACO1_M	Taco1	Taco1	70207	Translation	no|no chan	0	COG0217(t
A0A2R8VK	A0A2R8VK	Tfcp2	Tfcp2	21422	Transcripti	no|no chan	0	ENOG410X
P17125	TGFB3_MC	Tgfb3	Tgfb3	21809	Transformi	no|no chan	0	ENOG410X
A0A140DK	A0A140DKP8_MOUSE Gag (Fragment) OS=M				Gag	no|no chan	0	ENOG410Z
O70250	PGAM2_M	Pgam2	Pgam2	56012	Phosphogly	no|no chan	0	COG0588(C
O70252	HMOX2_M	Hmox2	Hmox2	15369	Heme oxyg	no|no chan	0	COG5398(F
O35841	API5_MOU	Api5	Api5	11800	Apoptosis i	no|no chan	0	ENOG410X
E9PWT4	E9PWT4_M	H2-Q7	H2-Q7	15018	Histocomp	no|no chan	0	ENOG4111
E9Q3P4	E9Q3P4_M	Cenpf	Cenpf	108000	Centromere	no|no chan	0	ENOG410X
Q9WUD1	CHIP_MOU	Stub1	Stub1	56424	E3 ubiquiti	no|no chan	0	ENOG410Z
A0A087WP	A0A087WP	Abhd14b	Abhd14b		Putative pr	no|no chan	0	COG0596(F
P24527	LKHA4_M	Lta4h	Lta4h	16993	Leukotriene	no|no chan	0	COG0308(E
O88188	LY86_MOU	Ly86	Ly86	17084	Lymphocyt	no|up	0	ENOG410Y
Q07968	F13B_MOU	F13b	F13b	14060	Coagulatio	no|no chan	0	ENOG410Y
Q5NCB2	Q5NCB2_M	Gm12185	Gm12185	620913	Predicted g	no|up	0	ENOG4112
Q8BJT9	EDEM2_M	Edem2	Edem2	108687	ER degrada	no|no chan	0	ENOG410X
Q543W6	Q543W6_M	Chek2	Chek2	50883	Serine/thre	no|no chan	0	ENOG410Y
E9QB01	E9QB01_M	Ncam1	Ncam1	17967	Neural cell	no|down	0	ENOG410X
E9QB02	E9QB02_M	Mars1	Mars1	216443	Methionine	no|no chan	0	COG0143(A
A0A097BW	A0A097BW	Postn	Postn	50706	Periostin is	no|down	0	COG2335(E
A0A9L6H2	A0A9L6H2	Jmjd8	Jmjd8	72106	Jumonji dor	no|no chan	0	ENOG410Z
Q921C5	BICD2_MC	Bicd2	Bicd2	76895	Protein bic	no|no chan	0	ENOG410X
Q8R2L5	RT18C_MC	Mrps18c	Mrps18c	68735	Small ribos	no|no chan	0	COG0238(s
P48967	MPIP3_MC	Cdc25c	Cdc25c	12532	M-phase in	no|no chan	0	COG5105(t
A0A1L1SV	A0A1L1SV	Usp47	Usp47		Ubiquitin c	no|no chan	0	COG5077(F
P48962	ADT1_MO	Slc25a4	Slc25a4	11739	ADP/ATP	no|up	0	ENOG410X
P52479	UBP10_MC	Usp10	Usp10	22224	Ubiquitin c	no|no chan	0	ENOG410X
B1AY85	B1AY85_M	Plxdc2	Plxdc2	67448	Plexin dom	no|no chan	0	ENOG410Y
Q8BLF1	NCEH1_M	Nceh1	Nceh1	320024	Neutral ch	no|no chan	0	COG0657(e
P24860	CCNB1_M	Ccnb1	Ccnb1	268697	G2/mitotic	no|no chan	0	COG5024(g
Q8BLF3	Q8BLF3_M	Dnajc14	Dnajc14	74330	J domain-c	no|no chan	0	ENOG410Z
Q8BTW3	EXOS6_M	Exosc6	Exosc6	72544	Exosome cc	no|no chan	0	COG0689(F
Q69ZL1	FGD6_MO	Fgd6	Fgd6	13998	FYVE, Rho	no|no chan	0	ENOG410X
Q8BTW8	CK5P1_MC	Cdk5rap1	Cdk5rap1	66971	Mitochond	no|no chan	0	COG0621(C
Q8BTW9	PAK4_MO	Pak4	Pak4	70584	Serine/thre	no|no chan	0	ENOG410X
Q924W7	DEN2B_M	Dennd2b	Dennd2b	76954	DENN dom	no|no chan	0	ENOG410X
Q91YT2	RN185_MC	Rnf185	Rnf185	193670	E3 ubiquiti	no|no chan	0	ENOG4111

Q3UAY3	Q3UAY3_M	Gtf3c4	Gtf3c4	269252	General tra	no | no chan	0	ENOG410Y
Q3UU43	Q3UU43_M	Chpf2	Chpf2	100910	Hexosyltrar	no | no chan	0	ENOG410X
Q0PNF0	Q0PNF0_M	Gulp1	Gulp1	70676	Gulp-2	no | no chan	0	ENOG410Z
Q8CF89	TAB1_MOU	Tab1	Tab1	66513	TGF-beta-c	no | no chan	0	COG0631(M
Q9JKK7	TMOD2_M	Tmod2	Tmod2	50876	Tropomodu	no | no chan	0	ENOG410Y
Q61407	Q61407_M	Cfhr2	Cfhr2	545366	Complemer	no | no chan	0	ENOG410Y
Q3ZK22	VEZA_MO	Vezt	Vezt	215008	Vezatin	no | no chan	0	ENOG410Y
P32261	ANT3_MO	Serpinc1	Serpinc1	11905	Antithromb	no | up	0	COG4826(M
A0A1D5RL	A0A1D5RL	Sipa1l2	Sipa1l2		Signal-indu	no | no chan	0	ENOG410X
A0A2C9F2I	A0A2C9F2I	Pxk	Pxk	218699	PX domain	no | no chan	0	ENOG410Z
Q3U0T9	Q3U0T9_M	Rab35	Rab35	77407	Rab35	no | no chan	0	ENOG4111
Q9ERB0	SNP29_MO	Snap29	Snap29	67474	Synaptosom	no | no chan	0	ENOG410Y
P70419	GALT3_MO	Galnt3	Galnt3	14425	Polypeptide	no | up	0	ENOG410X
P56394	COX17_MO	Cox17	Cox17	12856	Cytochrome	no | no chan	0	ENOG410X
Q9EP78	CHST7_MO	Chst7	Chst7	60322	Carbohydrc	no | down	0	ENOG4111
P56391	CX6B1_MO	Cox6b1	Cox6b1	110323	Cytochrome	no | up	0	ENOG4112
P56390	CKS2_MOU	Cks2	Cks2	66197	Cyclin-depe	no | no chan	0	ENOG4111
P56399	UBP5_MOU	Usp5	Usp5	22225	Ubiquitin c	no | no chan	0	COG5207(u
Q80W68	KIRR1_MO	Kirrel1	Kirrel1	170643	Kin of IRR	no | no chan	0	ENOG410X
A8C756	THADA_M	Thada	Thada	240174	tRNA (32-2	no | no chan	0	COG5543(t
O88322	NID2_MOU	Nid2	Nid2	18074	Nidogen-2	no | no chan	0	ENOG4111
Q9R0Q6	ARC1A_MO	Arpc1a	Arpc1a	56443	Actin-relate	no | no chan	0	ENOG410X
Q80W65	PCSK9_MO	Pcsk9	Pcsk9	100102	Proprotein	no | no chan	0	COG1404(U
Q08297	RAD51_MO	Rad51	Rad51	19361	DNA repai	no | no chan	0	COG0468(L
A0A494BB1	A0A494BB1	Asdurf	Asdurf		Asnsd1 ups	no | no chan	0	
D3Z7R9	D3Z7R9_M	Ccpg1	Ccpg1	72278	Cell cycle p	no | no chan	0	ENOG4110
E9QMB7	E9QMB7_M	Naa30	Naa30		N(alpha)-ac	no | no chan	0	COG0456(K
Q8VCW8	ACSF2_MO	Acsf2	Acsf2	264895	Medium-ch	no | down	0	COG0318(I
P60521	GBRL2_MO	Gabarapl2	Gabarapl2	93739	Gamma-am	no | no chan	0	ENOG4111
P19096	FAS_MOUS	Fasn	Fasn	14104	Fatty acid s	no | no chan	0	COG3321(s
P53702	CCHL_MO	Hccs	Hccs	15159	Holocytoch	no | no chan	0	ENOG410Y
Q80U44	ZFY16_MO	Zfyve16	Zfyve16	218441	Zinc finger	no | no chan	0	ENOG410X
A2A4Z0	A2A4Z0_M	Ube2c	Ube2c	68612	Ubiquitin-c	no | no chan	0	ENOG4111
Q9QWT9	KIFC1_MO	Kifc1	Kifc1	16580; 1005	Kinesin-like	no | no chan	0	COG5059(I
Q8R1K4	AT2L2_MO	Phykpl	Phykpl	72947	5-phosphoh	no | no chan	0	COG0160(t
Q8R1K1	UBAC2_MO	Ubac2	Ubac2	68889	Ubiquitin-c	no | no chan	0	ENOG4111
Q8K124	PKHO2_M	Plekho2	Plekho2	102595	Pleckstrin h	no | no chan	0	ENOG4111
O08911	MK12_MO	Mapk12	Mapk12	29857	Mitogen-ac	no | no chan	0	ENOG410X
O08912	GALT1_MO	Galnt1	Galnt1	14423	Polypeptide	no | no chan	0	ENOG410X
O08915	AIP_MOUS	Aip	Aip	11632	AH recepto	no | no chan	0	ENOG410Y
O08917	FLOT1_MO	Flot1	Flot1	14251	Flotillin-1	no | no chan	0	COG2268(c
O08919	NUMBL_M	Numbl	Numbl	18223	Numb-like	no | up	0	ENOG410X
A0A286YC9	A0A286YC9	Thoc7	Thoc7		THO comp	no | no chan	0	ENOG4111

A1L0U6	A1L0U6_M	Nckap1	Nckap1	50884	Nck-associc	no | no chan	0	ENOG410
J3QN46	J3QN46_M	Rpain	Rpain	69723	RPA intera	no | no chan	0	ENOG4111
G5E8Q0	G5E8Q0_M	Lyst	Lyst	17101	Lysosomal	no | no chan	0	ENOG410
A0A2I3BR2	A0A2I3BR2	Fam107b	Fam107b		Family with	no | no chan	0	ENOG4111
E9Q9B0	E9Q9B0_M	Kif1c	Kif1c		Kinesin fan	no | no chan	0	COG5059(
Q9DBS2	TPRGL_M	Tprg1l	Tprg1l	67808	Tumor prot	no | no chan	0	ENOG411
Q9DBS1	TMM43_M	Tmem43	Tmem43	74122	Transmemb	no | no chan	0	ENOG410
E9Q9B3	E9Q9B3_M	Spryd3	Spryd3	223918	SPRY domc	no | no chan	0	ENOG410
A0A0R4J0V	A0A0R4J0V	Lrrc40	Lrrc40	67144	Leucine ricl	no | no chan	0	COG4886(
Q9DBS5	KLC4_MO	Klc4	Klc4	74764	Kinesin ligl	no | no chan	0	COG0457(I
Q9DBS8	POC5_MO	Poc5	Poc5	67463	Centrosome	no | no chan	0	ENOG410
A2A863	ITB4_MOU	Itgb4	Itgb4	192897	Integrin bet	no | no chan	0	ENOG410
Q6KAU3	Q6KAU3_M	Mrps25	Mrps25	64658	Small ribos	no | no chan	0	ENOG410
Q6KAU4	MB12B_M	Mvb12b	Mvb12b	72543	Multivesicu	no | no chan	0	ENOG411
E9Q238	KANK1_M	Kank1	Kank1	107351	KN motif a	no | no chan	0	COG0666(r
A0A0M5HI	A0A0M5HI	Prl2c3	Prl2c3	26421; 1881	Growth hor	no | up	0	
Q8CJF7	ELYS_MOU	Ahctf1	Ahctf1	226747	Protein EL	no | no chan	0	ENOG410
Q8CJF9	AGO3_MC	Ago3	Ago3	214150	Protein arg	no | no chan	0	ENOG410
P97930	KTHY_MC	Dtymk	Dtymk	21915	Thymidylat	no | no chan	0	COG0125(I
Q6NV52	Q6NV52_M	Tacc3	Tacc3	21335	Transformi	no | no chan	0	ENOG410
Q3TLA1	Q3TLA1_M	Rbm38	Rbm38	56190	RNA-bindi	no | no chan	0	ENOG4111
Q3U4H0	Q3U4H0_M	Mapre1	Mapre1	13589	Microtubul	no | no chan	0	COG5217(r
P06869	UROK_MC	Plau	Plau	18792	Urokinase-t	no | no chan	0	COG5640(I
Q3TQH4	Q3TQH4_M	Chst12	Chst12	59031	Carbohydrc	no | no chan	0	ENOG4111
E0CXS8	E0CXS8_M	Gm8113	Gm8113		Predicted g	no | no chan	0	ENOG410
A0A0B4J1F	A0A0B4J1F	Tmem131l	Tmem131l	229473	Transmemb	no | no chan	0	ENOG410
E0CXS2	E0CXS2_M	Tle3	Tle3		Transducin	no | no chan	0	ENOG410
A2ALB2	A2ALB2_M	Rexo4	Rexo4	227656	RNA exonu	no | no chan	0	COG0847(I
B2RWZ3	B2RWZ3_M	Aoah	Aoah	27052	Acyloxyacy	no | no chan	0	ENOG410
A0A0G2JFI	A0A0G2JFI	Elovl6	Elovl6		Elongation	no | no chan	0	ENOG410
D3Z1L1	D3Z1L1_M	Tmcc3	Tmcc3		Transmemb	no | no chan	0	ENOG410
Q9CQW1	YKT6_MO	Ykt6	Ykt6	56418	Synaptobre	no | no chan	0	COG5143(
Q9CQW0	EMC6_MO	Emc6	Emc6	66048	ER membrc	no | no chan	0	ENOG4111
Q546B3	Q546B3_M	Satb2	Satb2	212712	DNA-bindi	no | no chan	0	ENOG4111
Q9CX48	ZCH10_M	Zcchc10	Zcchc10	67966	Zinc finger	no | no chan	0	ENOG410
A0A1B0GS	A0A1B0GS	Bmal1	Bmal1		Basic helix-	no | no chan	0	ENOG410
Q91V76	CK054_MOUSE	Ester hydrolase C		70984	Ester hydro	no | no chan	0	ENOG410
A0A1B0GS	A0A1B0GS	Ifitm6	Ifitm6		Interferon i	no | no chan	0	ENOG411
A0A571BG	A0A571BG	Kcnab2	Kcnab2		Voltage-gat	yes | up	1	COG0667(
Q8BZM0	KLH12_M	Klhl12	Klhl12	240756	Kelch-like	no | no chan	0	ENOG410
Q1W617	SHRM4_M	Shroom4	Shroom4	208431	Protein Shr	yes | up	1	ENOG4111
Q91V79	FITM1_MC	Fitm1	Fitm1	68680	Fat storage	no | up	0	ENOG4111
D3YVL0	D3YVL0_M	Mov10	Mov10		RNA helicc	no | no chan	0	COG1112(

P59913	PCMD1_M	Pcmtd1	Pcmtd1	319263	Protein-L-is	no|no chan	0	COG2518(M
Q80XE1	RIC8B_MC	Ric8b	Ric8b	237422	Chaperone	no|no chan	0	ENOG410X
A0A075B5F	A0A075B5F	Ighv7-3	Ighv7-3		Immunoglo	no|down	0	ENOG410Y
Q4VC33	MAEA_MC	Maea	Maea	59003	E3 ubiquiti	no|no chan	0	ENOG410X
A0A075B5F	A0A075B5F	Ighv5-16	Ighv5-16		Immunoglo	no|up	0	ENOG410Y
A0A075B5F	A0A075B5F	Ighv14-2	Ighv14-2		Immunoglo	no|no chan	0	ENOG4111
A0A075B5F	A0A075B5F	Ighv4-1	Ighv4-1		Immunoglo	no|up	0	ENOG410Y
A0A075B5F	A0A075B5F	Ighv14-3	Ighv14-3		Immunoglo	no|down	0	ENOG4111
B0R058	B0R058_M(	Gpcpd1	Gpcpd1		Glyceropho	no|no chan	0	COG0584(g
Q66GT5	PTPM1_M(	Ptpmt1	Ptpmt1	66461	Phosphatid	no|no chan	0	ENOG4111
P50428	ARSA_MO	Arsa	Arsa	11883	Arylsulfata	no|no chan	0	COG3119(S
Q4G5Y1	KLDC2_M	Klhdc2	Klhdc2	69554	Kelch dom	no|no chan	0	ENOG410Y
P50396	GDIA_MO	Gdi1	Gdi1	14567	Rab GDP d	no|no chan	0	COG5044(C
P50427	STS_MOUS	Sts	Sts	20905	Steryl-sulfa	no|no chan	0	COG3119(S
O55042	SYUA_MO	Snca	Snca	20617	Alpha-synu	no|no chan	0	ENOG4111
B2M1R6	B2M1R6_M	Hnrnpk	Hnrnpk	15387	Heterogene	no|no chan	0	ENOG4111
B2M1R7	B2M1R7_M	Pcbp2	Pcbp2	18521	Poly(RC) bi	no|no chan	0	ENOG410X
Q75UR0	ANO5_MC	Ano5	Ano5	233246	Anoctamin	no|no chan	0	ENOG410X
Q3TE80	Q3TE80_M	2410002F23	2410002F23	668661	RIKEN cD	no|no chan	0	ENOG410Z
Q6P8X1	SNX6_MO	Snx6	Snx6	72183	Sorting nex	no|no chan	0	ENOG410X
P59708	SF3B6_MO	Sf3b6	Sf3b6	66055	Splicing fac	no|no chan	0	ENOG4111
D3YYR5	D3YYR5_M	P2rx4	P2rx4	18438	Purinergic r	no|no chan	0	ENOG410X
Q6ZQ29	TAOK2_M	Taok2	Taok2	381921	Serine/thre	no|no chan	0	ENOG410Y
Q3UM23	Q3UM23_M	Rnh1	Rnh1	107702	Ribonuclea	no|up	0	ENOG410Z
Q3UCL5	Q3UCL5_MOUSE	Ferritin OS=Mu		14325	Ferritin	no|no chan	0	COG1528(P
O35309	NMI_MOU	Nmi	Nmi	64685	N-myc-inte	no|up	0	ENOG4111
F8VQK5	F8VQK5_M	Sash1	Sash1	70097	SAM and Sl	yes|up	1	ENOG410Z
A2AT91	A2AT91_M	Plcb4	Plcb4		Phosphoinc	no|no chan	0	ENOG410X
Q9CWI3	BCCIP_MC	Bccip	Bccip	66165	BRCA2 and	no|no chan	0	ENOG4111
Q8BJ34	MARF1_M	Marf1	Marf1	223989	Meiosis reg	no|no chan	0	ENOG410X
Q8C0P0	GWL_MOU	Mastl	Mastl	67121	Serine/thre	no|no chan	0	ENOG410Z
Q8BND4	INT6L_MC	Ints6l	Ints6l	236790	Integrator c	no|no chan	0	ENOG410X
Q9JLJ0	LITAF_MC	Litaf	Litaf	56722	Lipopolysac	no|no chan	0	ENOG4112
P45878	FKBP2_M(	Fkbp2	Fkbp2	14227	Peptidyl-pr	yes|up	1	COG0545(r
P60898	RPB9_MOU	Polr2i	Polr2i	69920	DNA-direc	no|no chan	0	COG1594(p
A0A1W2P8	A0A1W2P8	Ank3	Ank3		Ankyrin 3, e	no|no chan	0	COG0666(t
B0LAD7	B0LAD7_M	Lrp10	Lrp10	65107	Low-density	no|no chan	0	ENOG410X
Q9CYU6	DPH7_MO	Dph7	Dph7	67228	Diphthine r	no|no chan	0	ENOG4111
Q8BND3	WDR35_M	Wdr35	Wdr35	74682	WD repeat-	no|no chan	0	ENOG410X
Q9R060	NUBP1_M(	Nubp1	Nubp1	26425	Cytosolic F	no|no chan	0	COG0489(D
E9PYX5	E9PYX5_M	Atp13a2	Atp13a2		ATPase typ	no|no chan	0	ENOG410X
Q9ER73	ELP4_MOU	Elp4	Elp4	77766	Elongator c	no|no chan	0	ENOG410X
Q9D517	PLCC_MO	Agpat3	Agpat3	28169	1-acyl-sn-gl	no|no chan	0	COG0204(I

F8WJK8	F8WJK8_M	St13	St13		Suppressior	no | no chan	0	ENOG410
Q5SXR6	Q5SXR6_M	Cltc	Cltc		Clathrin he	no | no chan	0	ENOG410
Q9ER41	TOR1B_M(	Tor1b	Tor1b	30934	Torsin-1B	no | no chan	0	ENOG410
Q8BUR4	DOCK1_M	Dock1	Dock1	330662	Dedicator c	no | no chan	0	ENOG410
Q2TBF9	Q2TBF9_M	Map1lc3b	Map1lc3b	67443	Microtubul	no | no chan	0	ENOG4111
Q6LCK2	Q6LCK2_M	Eif3e	Eif3e	16341	Int-6 protei	no | no chan	0	ENOG410
Q8K2M0	RM38_MO	Mrpl38	Mrpl38	60441	Large ribos	no | no chan	0	COG1881(I
E9Q7G0	NUMA1_M	Numa1	Numa1	101706	Nuclear mi	no | no chan	0	ENOG4112
A0A1L1SRI	A0A1L1SRI	Prdm10	Prdm10		PR domain	no | no chan	0	COG5048(S
O88668	CREG1_M(	Creg1	Creg1	433375	Protein CR	no | up	0	ENOG4111
A0A2I3BPF	A0A2I3BPF	Tsc2	Tsc2	22084	TSC compl	no | no chan	0	ENOG410
O88667	RAD_MOU	Rrad	Rrad	56437	GTP-bindi	no | no chan	0	COG1100(I
P30282	CCND3_M	Ccnd3	Ccnd3	12445	G1/S-speci	no | no chan	0	ENOG410
B1ASP2	B1ASP2_M	Jak1	Jak1	16451	Tyrosine-pr	no | no chan	0	COG0515(u
D3Z6Q7	D3Z6Q7_M	Tfeb	Tfeb		Transcripti	no | no chan	0	ENOG411(
Q3TPE9	ANKY2_M	Ankmy2	Ankmy2	217473	Ankyrin rep	no | no chan	0	ENOG410
Q3TM89	Q3TM89_M	Pcnp	Pcnp	76302	PEST prote	no | no chan	0	ENOG4111
A0A0A6YX	A0A0A6YX	Hyal2	Hyal2		Hyaluronid	no | no chan	0	ENOG410
Q61090	FZD7_MO	Fzd7	Fzd7	14369	Frizzled-7	no | no chan	0	ENOG410
Q8R3B7	BRD8_MO	Brd8	Brd8	78656	Bromodom	no | no chan	0	ENOG410Y
Q9DB05	SNAA_MO	Napa	Napa	108124	Alpha-solu	no | no chan	0	ENOG410
A0A1L1SSS	A0A1L1SSS	Ncoa7	Ncoa7		Nuclear rec	no | up	0	ENOG411(
Q9Z2U2	ZN292_MC	Zfp292	Zfp292	30046	Zinc finger	no | no chan	0	COG5048(V
B5B2P6	B5B2P6_M	Nfatc2	Nfatc2	18019	Nuclear fac	no | no chan	0	ENOG411(
Q6P3D0	NUD16_M	Nudt16	Nudt16	75686	U8 snoRNA	no | no chan	0	ENOG4111
D3YUQ5	D3YUQ5_M	Rundc3b	Rundc3b	242819	RUN doma	no | no chan	0	ENOG410
B9EHY2	B9EHY2_M	Zmpste24	Zmpste24	230709	CAAX pren	no | no chan	0	COG0501(c
Q5SV80	MYO19_M	Myo19	Myo19	66196	Unconventi	no | no chan	0	COG5022(I
Q91YP0	L2HDH_M	L2hgdh	L2hgdh	217666	L-2-hydrox	no | no chan	0	COG0579(r
B1AU74	B1AU74_M	Mospd2	Mospd2	76763	Motile sper	no | no chan	0	ENOG410Y
B1AU75	B1AU75_M	Nasp	Nasp	50927	Nuclear au	no | no chan	0	ENOG411(
Q5SUE8	ANR40_M(	Ankrd40	Ankrd40	71452	Ankyrin rep	no | no chan	0	ENOG410
Q8BW88	PKHS1_M(	Plekhs1	Plekhs1	226245	Pleckstrin h	no | up	0	ENOG410Y
D3Z0K6	D3Z0K6_M	Rsbn1l	Rsbn1l	242860	Round sper	no | no chan	0	ENOG410
A0A0A6YX	A0A0A6YX	Ighv7-4	Ighv7-4		Immunoglo	no | up	0	ENOG410Y
A0A0A6YX	A0A0A6YX	Lrba	Lrba	80877	LPS-respon	no | no chan	0	ENOG410
P43023	CX6A2_M(	Cox6a2	Cox6a2	12862	Cytochrom	no | up	0	ENOG4112
Q05117	PPA5_MOU	Acp5	Acp5	11433	Tartrate-re	no | down	0	COG1409(c
P43025	TETN_MO	Clec3b	Clec3b	21922	Tetranectin	no | down	0	ENOG410
P43024	CX6A1_M(	Cox6a1	Cox6a1	12861	Cytochrom	no | no chan	0	ENOG4112
Q9D0B6	PBDC1_M(	Pbdc1	Pbdc1	67683	Protein PBI	no | no chan	0	ENOG4111
Q8CHA4	Q8CHA4_M	Sorcs2	Sorcs2	81840	MKIAA132	no | down	0	ENOG410Y
Q9CZD3	GARS_MO	Gars1	Gars1	353172	Glycine--tR	no | no chan	0	COG0423(C

A0A1Y7VK	A0A1Y7VK	Zfp825	Zfp825		Zinc finger	no|no chan	0	COG5048(Z
Q61081	CDC37_MO	Cdc37	Cdc37	12539	Hsp90 co-cl	no|no chan	0	ENOG410X
Q8BGW1	FTO_MOU	Fto	Fto	26383	Alpha-keto	no|no chan	0	ENOG4111
A0AAA9W	A0AAA9W	Cacna1h	Cacna1h		Calcium ch	no|up	0	ENOG410X
Q62415	ASPP1_MC	Ppp1r13b	Ppp1r13b	21981	Apoptosis-s	no|no chan	0	ENOG410Y
Q6PDM1	MSL1_MO	Msl1	Msl1	74026	Male-specif	no|no chan	0	ENOG410X
C9DT14	C9DT14_M	Tardbp	Tardbp	230908	TDP-43 var	no|no chan	0	ENOG410Z
A7M7S2	A7M7S2_M	Nprl3	Nprl3		GATOR co	no|no chan	0	ENOG410X
A0A1Y7VM	A0A1Y7VM	Akr1c20	Akr1c20		Aldo-keto r	no|down	0	COG0656(Y
A0A494BA	A0A494BA	Duox2	Duox2	214593	NAD(P)H c	no|up	0	ENOG410X
Q8VDN2	AT1A1_MC	Atp1a1	Atp1a1	11928	Sodium/po	no|no chan	0	COG0474(V
Q8BPF4	Q8BPF4_MOUSE	Globin domain-		110257	Globin don	no|up	0	COG1018(t
Q3U3I9	ZN865_MC	Znf865	Znf865	319748	Zinc finger	no|no chan	0	COG5048(F
A0A217FL4	A0A217FL4	Nudt14	Nudt14	66174	Nudix hydr	no|no chan	0	COG0494(L
Q9QZB9	DCTN5_M	Dctn5	Dctn5	59288	Dynactin su	no|no chan	0	ENOG410Y
P08032	SPTA1_MC	Spta1	Spta1	20739	Spectrin alp	no|no chan	0	COG5069(C
P08030	APT_MOU	Aprt	Aprt	11821	Adenine ph	no|up	0	COG0503(C
D3YVQ6	D3YVQ6_M	Rab17	Rab17		RAB17, me	no|no chan	0	ENOG410Y
Q9DAK9	PHP14_MC	Phpt1	Phpt1	75454	14 kDa pho	no|no chan	0	ENOG4111
B2RUK5	B2RUK5_M	Mccc2	Mccc2	78038	methylcrot	no|no chan	0	COG4799(S
B2RUK7	B2RUK7_M	Tpcn2	Tpcn2	233979	Two pore se	no|no chan	0	ENOG4111
B2RUK2	B2RUK2_M	Erbin	Erbin	59079	Erbb2 inter	no|no chan	0	COG4886(S
Q9WV98	TIM9_MOU	Timm9	Timm9	30056	Mitochondi	no|no chan	0	ENOG4111
Q9DBB4	NAA16_MC	Naa16	Naa16	66897	N-alpha-ac	no|no chan	0	ENOG410X
B2RUK9	B2RUK9_M	Zfp456	Zfp456	408065	Zinc finger	yes|up	1	COG5048(C
Q8BG80	FBX46_MC	Fbxo46	Fbxo46	243867	F-box only	no|no chan	0	ENOG410Z
Q8BG81	PDIP3_MC	Poldip3	Poldip3	73826	Polymerase	no|no chan	0	ENOG4111
Q8BG87	TET3_MOU	Tet3	Tet3	194388	Methylcyto	no|no chan	0	ENOG410X
Q8BYY4	TT39B_MC	Ttc39b	Ttc39b	69863	Tetratricop	no|up	0	ENOG410X
Q3TV62	Q3TV62_M	Tor1a	Tor1a	30931	Torsin	no|no chan	0	ENOG410X
A0A0J9YTS	A0A0J9YTS	Tspan9	Tspan9		Tetraspanir	no|no chan	0	ENOG4111
Q58E59	Q58E59_M	Sf3a3	Sf3a3	75062	Splicing fac	no|no chan	0	COG5188(S
Q9WV96	T10B_MOU	Timm10b	Timm10b	14356	Mitochondi	no|no chan	0	ENOG4111
A0A6Q6RC	A0A6Q6RC	Snx14	Snx14	244962	Sorting nex	no|no chan	0	ENOG410X
A2A6U3	A2A6U3_M	Septin9	Septin9	53860	Septin 9	no|no chan	0	COG5019(C
Q9WV91	FPRP_MOU	Ptgfrn	Ptgfrn	19221	Prostaglanc	no|no chan	0	ENOG4111
A2AAE1	BLTP1_MC	Bltp1	Bltp1	229227	Bridge-like	no|no chan	0	ENOG410X
Q8C3S2	TNG6_MO	Tango6	Tango6	272538	Transport o	no|no chan	0	ENOG410X
F8WIJ0	F8WIJ0_MO	Slc12a4	Slc12a4	20498	Solute carri	no|no chan	0	COG0531(A
P62631	EF1A2_MC	Eef1a2	Eef1a2	13628	Elongation	no|up	0	COG5256(s
A0A2R8VH	A0A2R8VH	Septin3	Septin3		Neuronal-s	no|down	0	COG5019(t
A0A668KM	A0A668KM	Slc4a4	Slc4a4		Anion exch	no|no chan	0	ENOG410X
Q3TNL8	IPRI_MOU	Itprip	Itprip	414801	Inositol 1,4,	no|no chan	0	ENOG411C

A0A0R4J12	A0A0R4J12	Srpk2	Srpk2	20817	non-specifi	no|up	0 ENOG410
Q91VR8	BRK1_MO	Brk1	Brk1	101314	Protein BR	no|no chan	0 ENOG4111
Q91VR7	MLP3A_M	Map1lc3a	Map1lc3a	66734	Microtubul	no|no chan	0 ENOG4111
Q3TNL1	Q3TNL1_M	G6pdx	G6pdx	14381	Glucose-6-p	no|up	0 COG0364(
Q91VR5	DDX1_MC	Ddx1	Ddx1	104721	ATP-depen	no|no chan	0 COG0513(
P20060	HEXB_MC	Hexb	Hexb	15212	Beta-hexos	no|no chan	0 COG3525(
A2BEE9	A2BEE9_M	Dlg3	Dlg3	53310	Discs large	no|no chan	0 COG0194(
Q9QZD4	XPF_MOU	Ercc4	Ercc4	50505	DNA repai	no|no chan	0 COG1948(
A2AQJ5	A2AQJ5_M	Tmem87a	Tmem87a	211499	Transmemb	no|no chan	0 ENOG410
Q9QZD9	EIF3I_MO	Eif3i	Eif3i	54709	Eukaryotic	no|no chan	0 ENOG410
Q9QZD8	DIC_MOU	Slc25a10	Slc25a10	27376	Mitochond	no|no chan	0 ENOG410
A0A1D5RL	A0A1D5RL	Adgrg1	Adgrg1		Adhesion G	no|no chan	0 ENOG410
B7ZNK8	B7ZNK8_M	Pex7	Pex7	18634	Peroxin-7	no|no chan	0 ENOG410
A0A0R4J29	A0A0R4J29	Eif2d	Eif2d	16865	Eukaryotic	no|no chan	0 ENOG410
Q8C7C3	Q8C7C3_M	Tpm3	Tpm3	59069	Tropomyos	no|no chan	0 ENOG410
A0A2I3BQ	A0A2I3BQ	Asxl1	Asxl1		ASXL trans	no|no chan	0 ENOG410
Q3THZ3	Q3THZ3_M	Atp6ap1	Atp6ap1	54411	V-type prot	no|no chan	0 ENOG410
A0A494B9I	A0A494B9I	Naa40	Naa40		N-alpha-ac	no|no chan	0 ENOG411
Q3UXD9	Q3UXD9_	Pecr	Pecr	111175	Peroxisoma	no|no chan	0 COG1028(
B7ZNK7	B7ZNK7_M	Tle5	Tle5	14797	Aes protein	no|no chan	0 ENOG410
Q3UU41	SCIMP_MC	Scimp	Scimp	327957	SLP adapte	no|no chan	0 ENOG4111
A0A068BFS	A0A068BFS	Ring1	Ring1	19763	RING-type	no|no chan	0 ENOG410
Q8C8M9	Q8C8M9_M	Ttc5	Ttc5	219022	Tetratricop	no|no chan	0 ENOG410
P11438	LAMP1_M	Lamp1	Lamp1	16783	Lysosome-a	no|no chan	0 ENOG410
B2RQW1	B2RQW1_	Tnfaip2	Tnfaip2	21928	Tumor necr	no|no chan	0 COG5173(
B2RY04	DOCK5_M	Dock5	Dock5	68813	Dedicator c	no|no chan	0 ENOG410
A0A1W2P8	A0A1W2P8	Trdn	Trdn		Triadin	no|down	0 ENOG410
A0A140T8F	A0A140T8F	Jade3	Jade3	382207	Jade family	no|no chan	0 COG5141(
A0A140T8F	A0A140T8F	Rcor1	Rcor1	217864	REST corep	no|no chan	0 ENOG410
Q9Z277	BAZ1B_M	Baz1b	Baz1b	22385	Tyrosine-pr	no|no chan	0 COG5076(
Q66JQ7	KNL1_MO	Knl1	Knl1	76464	Outer kinet	no|no chan	0 ENOG410
Q3TPZ5	Q3TPZ5_M	Dctn2	Dctn2	69654	Dynactin s	no|no chan	0 ENOG410
Q3U5W1	Q3U5W1_	Stat2	Stat2	20847	Signal trans	no|up	0 ENOG410
Q6PEV3	WIPF2_MC	Wipf2	Wipf2	68524	WAS/WAS	no|no chan	0 ENOG4111
Q9JMD1	SMBT1_M	Sfmbt1	Sfmbt1	54650	Scm-like wi	no|down	0 ENOG410
Q9JMD0	ZN207_MC	Znf207	Znf207	22680	BUB3-inter	no|no chan	0 ENOG410
Q8K3J5	ZN131_MC	Znf131	Znf131	72465	Zinc finger	no|no chan	0 COG5048(
Q9WVL1	AP4S1_MO	Ap4s1	Ap4s1	11782	AP-4 compl	no|no chan	0 COG5030(
Q9WVL0	MAAI_MO	Gstz1	Gstz1	14874	Maleylacet	no|no chan	0 COG0625(
Q3TBU7	Q3TBU7_M	Agfg2	Agfg2	231801	ArfGAP wi	no|no chan	0 ENOG4112
A0A0N4SU	A0A0N4SU	Rasgrp4	Rasgrp4		RAS guanyl	no|down	0 ENOG410
Q99PT1	GDIR1_M	Arhgdia	Arhgdia	192662	Rho GDP-c	no|no chan	0 ENOG4111
Q9CQ80	VPS25_MO	Vps25	Vps25	28084	Vacuolar p	no|no chan	0 ENOG4111

E9PZM4	CHD2_MO	Chd2	Chd2	244059	Chromodor	no|no chan	0	COG0553(c
E9PZM7	E9PZM7_M	Scaf11	Scaf11	72193	SR-related	no|no chan	0	ENOG4111
Q9CQ85	TIM22_MC	Timm22	Timm22	56322	Mitochondı	no|no chan	0	COG5596(v
Q9CQ86	MIEN1_M(	Mien1	Mien1	103742	Migration c	no|no chan	0	ENOG4112
Q3TIT7	Q3TIT7_M	Bcar1	Bcar1	12927	SH3 domaiı	no|no chan	0	ENOG410Z
Q6PIX9	CQ080_MC	Mtnap1	Mtnap1	276852	Mitochondı	no|up	0	ENOG4111
G3X9Y6	G3X9Y6_M	Akr1c19	Akr1c19	432720	Aldo-keto r	no|no chan	0	COG0656(F
G3X9Y5	G3X9Y5_M	Ube4a	Ube4a	140630	Ubiquitin c	no|no chan	0	COG5113(c
Q6NVF4	HELB_MO	Helb	Helb	117599	DNA helicc	no|no chan	0	COG0507(e
Q6NVF0	OCRL_MO	Ocrl	Ocrl	320634	Inositol pol	no|no chan	0	COG5411(F
Q6PIX5	RHDF1_M(	Rhbdf1	Rhbdf1	13650	Inactive rh	no|no chan	0	COG0705(N
Q8C4C9	Q8C4C9_M	Dnajb12	Dnajb12	56709	DnaJ heat s	no|no chan	0	COG0484(t
Q9D1H8	RM53_MO	Mrpl53	Mrpl53	68499	Large ribos	no|no chan	0	ENOG4111
H3BK65	H3BK65_M	Eps15	Eps15	13858	Epidermal (	no|no chan	0	ENOG410X
Q3UQV4	Q3UQV4_N	Cdyl2	Cdyl2	75796	Chromo do	no|no chan	0	COG1024(N
Q4QQK5	Q4QQK5_N	Glipr1	Glipr1	73690	GLI pathog	no|no chan	0	COG2340(N
Q91Z67	SRGP2_MC	Srgap2	Srgap2	14270	SLIT-ROB(	no|no chan	0	ENOG411(
Q9D1H7	GET4_MO	Get4	Get4	67604	Golgi to EF	no|no chan	0	ENOG410X
Q9D1H6	NDUF4_M	Ndufaf4	Ndufaf4	68493	NADH deh	no|no chan	0	ENOG4111
Q6S7F2	E2F7_MOU	E2f7	E2f7	52679	Transcripti	no|down	0	ENOG4111
Q920E7	Q920E7_MOUSE Pterin-mimicking anti-idiotc				Pterin-mim	no|no chan	0	ENOG410Y
A0AUN0	A0AUN0_M	Sec23ip	Sec23ip	207352	Sec23ip pro	no|no chan	0	ENOG410Y
E9PYP2	E9PYP2_M	Nbeal1	Nbeal1	269198	Neurobeacl	no|up	0	ENOG410X
P37889	FBLN2_M(	Fbln2	Fbln2	14115	Fibulin-2	no|no chan	0	ENOG410Y
Q8BMJ3	IF1AX_MC	Eif1ax	Eif1ax	66235	Eukaryotic	no|no chan	0	COG0361(k
Q8BH02	TOR4A_M(	Tor4a	Tor4a	227612	Torsin-4A	no|no chan	0	ENOG410X
Q8BH01	TMCO3_M	Tmco3	Tmco3	234076	Transmemb	no|no chan	0	COG0475(S
A2AKN9	A2AKN9_N	Mup2	Mup2	17841	Major urinc	no|down	0	ENOG4111
A2AKN8	A2AKN8_N	Mup8	Mup8	100041687	Major urinc	no|down	0	ENOG4111
Q3TCI8	Q3TCI8_M	Gne	Gne	50798	UDP-N-acc	no|no chan	0	COG1940(F
E9Q3N0	E9Q3N0_M	Recql	Recql	19691	ATP-depen	no|no chan	0	COG0514(i
E9Q3N1	E9Q3N1_M	Slc7a1	Slc7a1		Solute carri	no|down	0	COG0531(C
Q3TCI2	Q3TCI2_M	Amfr	Amfr	23802	E3 ubiquiti	no|no chan	0	COG5243(F
Q3TCI7	Q3TCI7_M	Ldha	Ldha	16828	L-lactate de	no|up	0	COG0039(p
Q91YI6	GLD2_MO	Tent2	Tent2	100715	Poly(A) RN	no|no chan	0	COG5260(c
O70279	ESS2_MOU	Ess2	Ess2	27886	Splicing fac	no|no chan	0	ENOG410Y
B2RXT3	B2RXT3_M	Ogdhl	Ogdhl	239017	oxoglutarat	no|no chan	0	COG0567(S
Q8BX80	ENASE_M(	Engase	Engase	217364	Cytosolic er	no|no chan	0	COG4724(e
Q3T9U1	Q3T9U1_M	Mob1b	Mob1b	68473	MOB1, Mp:	no|no chan	0	ENOG410X
E9PWZ3	RL3L_MOU	Rpl3l	Rpl3l	66211	Large ribos	no|no chan	0	COG0087(c
O70274	TP4A2_MC	Ptp4a2	Ptp4a2	19244	Protein tyrc	no|no chan	0	ENOG4111
Q9R1X5	MRP5_MO	Abcc5	Abcc5	27416	ATP-bindir	no|no chan	0	COG1132(A
A0A1Y7VI	A0A1Y7VI	Zfp410	Zfp410		Zinc finger	no|no chan	0	COG5048(I

E9QPI5	E9QPI5_M	Pds5a	Pds5a	71521	PDS5 cohes	no	no chan	0	ENOG410)
B7ZP28	B7ZP28_M	Dennd2a	Dennd2a	209773	Dennd2a p	no	no chan	0	ENOG410)
A7YL62	A7YL62_M	Apoa2	Apoa2	11807	Apolipopro	no	no chan	0	ENOG4111
B7ZP24	B7ZP24_M	Pcgf5	Pcgf5	76073	Pcgf5 prote	no	no chan	0	ENOG410)
Q69Z99	ZN512_MC	Znf512	Znf512	269639	Zinc finger	no	no chan	0	COG5048(S
B7ZP20	B7ZP20_M	Ankfy1	Ankfy1	11736	Ankyrin rep	no	no chan	0	COG0666(C
H7BWY6	H7BWY6_M	Rbp4	Rbp4	19662	Retinol-bin	no	no chan	0	ENOG4111
Q3TSB2	Q3TSB2_M	Zbed3	Zbed3	72114	Zinc finger	yes	down	1	ENOG4111
H7BWY8	H7BWY8_M	Tsr2	Tsr2	69499	Pre-rRNA-p	no	no chan	0	ENOG4112
Q8BRG8	TM209_MC	Tmem209	Tmem209	72649	Transmemb	no	no chan	0	ENOG410)
A0A0U1RQ	A0A0U1RQ	Cbl	Cbl		E3 ubiquiti	no	no chan	0	ENOG410Y
Q5NCY0	KDM6B_M	Kdm6b	Kdm6b	216850	Lysine-spec	no	no chan	0	ENOG410Z
Q3UTE1	Q3UTE1_M	Gm4832	Gm4832	225058	Small ribos	no	down	0	COG2004(4
P59114	CAPAM_M	Pcif1	Pcif1	228866	mRNA (2'-(	no	no chan	0	ENOG410)
P47791	GSHR_MO	Gsr	Gsr	14782	Glutathion	no	no chan	0	COG1249(c
B2KGP3	B2KGP3_M	Ppm1e	Ppm1e	320472	Protein pho	no	down	0	COG0631(r
Q8CE10	Q8CE10_M	Pcdhga9	Pcdhga9	93717	Cadherin d	no	no chan	0	ENOG410)
A8CVP4	A8CVP4_M	Lims1	Lims1	110829	LIM and se	no	no chan	0	ENOG410)
Q14DH7	ACSS3_MC	Acss3	Acss3	380660	Acyl-CoA s	no	down	0	COG0365(r
Q78HU3	MB12A_M	Mvb12a	Mvb12a	73711	Multivesicu	no	no chan	0	ENOG411(
Q8BTU1	CFA20_MC	Cfap20	Cfap20	14894	Cilia- and f	no	no chan	0	ENOG410)
Q3UU20	Q3UU20_M	Hid1	Hid1	217310	Protein HII	no	no chan	0	ENOG410)
Q5U4C3	SFR19_MO	Scaf1	Scaf1	233208	Splicing fac	no	no chan	0	ENOG4111
Q99LI7	CSTF3_MC	Cstf3	Cstf3	228410	Cleavage st	no	no chan	0	COG5107(c
B7ZCG3	B7ZCG3_M	Bpifa2	Bpifa2		BPI fold co	no	up	0	ENOG4111
Q99LI9	CLP1_MOU	Clp1	Clp1	98985	Polyribonu	no	no chan	0	COG5623(F
Q3UA32	Q3UA32_M	Galnt2	Galnt2	108148	Polypeptide	no	no chan	0	ENOG410)
Q91XE4	ACY3_MO	Acy3	Acy3	71670	N-acyl-aror	no	no chan	0	COG2988(T
Q61466	SMRD1_M	Smarcd1	Smarcd1	83797	SWI/SNF-r	no	no chan	0	COG5531(S
Q0PD23	Q0PD23_M	Rab32	Rab32	67844	Ras-related	no	up	0	ENOG410Y
Q3UJC8	CE034_MOUSE	Uncharacterized p		67392	Uncharacte	no	no chan	0	ENOG410Y
Q0PD20	Q0PD20_M	Rab34	Rab34	19376	Rab34	no	no chan	0	ENOG410)
G3UXN0	G3UXN0_M	Ctdspl	Ctdspl	69274	protein-seri	no	no chan	0	COG5190(C
A0A087WS	A0A087WS	Cep57l1	Cep57l1		Centrosome	no	no chan	0	ENOG410)
Q811U4	MFN1_MC	Mfn1	Mfn1	67414	Mitofusin-1	no	no chan	0	COG0699(T
Q07235	GDN_MOU	Serpine2	Serpine2	20720	Glia-derive	no	no chan	0	COG4826(F
Q80YS6	AFAP1_MC	Afap1	Afap1	70292	Actin filam	no	no chan	0	ENOG4111
Q08761	PROS_MO	Pros1	Pros1	19128	Vitamin K-	no	up	0	ENOG410Z
Q00519	XDH_MOU	Xdh	Xdh	22436	Xanthine d	no	no chan	0	COG4630(p
D3Z645	D3Z645_M	Vps29	Vps29		Vacuolar p	no	no chan	0	COG0622(F
D3Z644	D3Z644_M	Cep20	Cep20		Centrosome	no	down	0	ENOG4111
Q8C078	KKCC2_M	Camkk2	Camkk2	207565	Calcium/cc	no	no chan	0	ENOG410Y
Q8BGV0	SYNM_MC	Nars2	Nars2	244141	Asparaginy	no	no chan	0	COG0017(c

Q14BV6	YDJC_MO	Ydjc	Ydjc	69101	Carbohydrc	no	no chan	0	COG3394(U
A0A7U3L6	A0A7U3L6	Fgf21	Fgf21	56636	Fibroblast g	no	no chan	0	ENOG4111
Q8BN57	CC033_MOUSE	Protein C3orf33 h		329659	Protein C3c	no	no chan	0	ENOG4112
Q8C052	MAP1S_MC	Map1s	Map1s	270058	Microtubul	no	no chan	0	ENOG410X
Q8C050	KS6A5_MC	Rps6ka5	Rps6ka5	73086	Ribosomal	no	no chan	0	ENOG410X
Q9EP52	TWSG1_M	Twsg1	Twsg1	65960	Twisted gas	no	no chan	0	ENOG411C
Q9EP53	TSC1_MOU	Tsc1	Tsc1	64930	Hamartin	no	no chan	0	ENOG411C
P62821	RAB1A_MC	Rab1a	Rab1a	19324	Ras-related	no	no chan	0	ENOG410X
P62827	RAN_MOU	Ran	Ran	19384	GTP-bindir	no	no chan	0	ENOG410X
Q3U0S4	Q3U0S4_M	Ctdp1	Ctdp1	67655	RNA polym	no	no chan	0	COG5190(p
A0JLX6	A0JLX6_M	Micu3	Micu3	78506	Efha2 prote	no	no chan	0	ENOG410Z
P51855	GSHB_MO	Gss	Gss	14854	Glutathion	no	no chan	0	ENOG410X
A7VJ98	A7VJ98_MC	Gmfb	Gmfb	63985	Glia matur	no	no chan	0	ENOG4111
Q8C297	Q8C297_M	Tmem14c	Tmem14c	66154	Transmeml	no	no chan	0	ENOG4112
O88307	SORL_MO	Sorl1	Sorl1	20660	Sortilin-relc	no	no chan	0	ENOG410Y
P08730	K1C13_MC	Krt13	Krt13	16663	Keratin, typ	no	up	0	ENOG410Y
Q8BGV9	ATG4D_M	Atg4d	Atg4d	235040	Cysteine pr	no	down	0	ENOG410X
Q9CWR1	WDR73_M	Wdr73	Wdr73	71968	WD repeat-	no	no chan	0	ENOG410X
Q6GTM0	Q6GTM0_I	Ifit2	Ifit2	15958	Ifit2 proteir	no	no chan	0	COG0457(t
Q8R322	GLE1_MO	Gle1	Gle1	74412	mRNA exp	no	no chan	0	ENOG410X
Q61285	ABCD2_M	Abcd2	Abcd2	26874	ATP-bindir	no	down	0	COG4178((
Q8R326	PSPC1_MC	Pspc1	Pspc1	66645	Paraspeckle	no	no chan	0	ENOG410X
A0A6I8MW	A0A6I8MW	Golga2	Golga2		Golgin A2	no	no chan	0	ENOG4111
P46467	VPS4B_MC	Vps4b	Vps4b	20479	Vacuolar p	no	no chan	0	ENOG410X
Q3UVA4	Q3UVA4_M	Pik3cg	Pik3cg	30955	phosphatid	no	no chan	0	COG5032(c
P46460	NSF_MOU	Nsf	Nsf	18195	Vesicle-fusi	no	no chan	0	COG0464(F
Q8R1M0	HMCES_M	Hmces	Hmces	232210	Abasic site	no	down	0	COG2135(c
Q8R1M3	Q8R1M3_M	Polr1d	Polr1d	20018	Polymerase	no	no chan	0	ENOG4111
O88456	CPNS1_MC	Capns1	Capns1	12336	Calpain sm	no	up	0	ENOG410Y
A0A991EK	A0A991EK	Fxr2	Fxr2		FMR1 auto	no	no chan	0	ENOG410Z
Q71B07	D19L3_MC	Dpy19l3	Dpy19l3	233115	Protein C-n	no	no chan	0	ENOG410X
A0A0R4J0C	A0A0R4J0C	Loxl1	Loxl1	16949	protein-lysi	no	no chan	0	ENOG411C
G1UCX4	G1UCX4_M	Saal1	Saal1	78935	Synoviocyte	no	no chan	0	ENOG411C
A0A0R4J0C	A0A0R4J0C	Trim56	Trim56	384309	RING-type	no	no chan	0	ENOG410Y
A0A0R4J0C	A0A0R4J0C	Slc36a4	Slc36a4	234967	Solute carri	no	no chan	0	COG0814(M
A0A0R4J0C	A0A0R4J0C	Cog7	Cog7	233824	Conserved c	no	no chan	0	ENOG410X
E9Q9D5	RBL2A_MC	Rabl2	Rabl2	68708	Rab-like pr	no	no chan	0	COG1100(F
A0A0A6YW	A0A0A6YW	Rab3gap2	Rab3gap2	98732	RAB3 GTP	no	no chan	0	ENOG410X
Q546K1	Q546K1_M	Cygb	Cygb	114886	superoxide	no	down	0	COG1018(I
A0A0A6YW	A0A0A6YW	Map4k4	Map4k4	26921	non-specifi	no	no chan	0	ENOG410X
A2A841	A2A841_M	Epb41	Epb41		Protein 4.1	no	no chan	0	ENOG410X
O08648	M3K4_MO	Map3k4	Map3k4	26407	Mitogen-ac	no	no chan	0	ENOG410X
D3Z7P0	D3Z7P0_M	Babam2	Babam2		BRISC and	no	no chan	0	ENOG410Y

D3Z7P3	GLSK_MO	Gls	Gls	14660	Glutamina:	no | no chan	0	COG2066(s
A0A1Y7VN	A0A1Y7VN	Ndufs6	Ndufs6		NADH deh	no | no chan	0	ENOG4111
P82349	SGCB_MO	Sgcb	Sgcb	24051	Beta-sarcog	no | no chan	0	ENOG4111
P82348	SGCG_MO	Sgcg	Sgcg	24053	Gamma-sa	no | up	0	ENOG4111
P82347	SGCD_MO	Sgcd	Sgcd	24052	Delta-sarco	no | no chan	0	ENOG4111
Q8K1L2	SPIN4_MO	Spin4	Spin4	270624	Spindlin-4	no | no chan	0	ENOG4111
B2RPZ7	B2RPZ7_M	Fam234b	Fam234b	74525	Protein FA	no | down	0	ENOG410
B7U582	B7U582_MOUSE	Heat	shock-relat	15512	Heat shock-	no | no chan	0	COG0443(F
D3YTP3	D3YTP3_M	Mtx3	Mtx3	382793	Metaxin	no | no chan	0	ENOG411(
Q8C1F4	CGAT2_M	Csgalnact2	Csgalnact2	78752	Chondroiti	no | no chan	0	ENOG410
Q9CQU3	RER1_MO	Rer1	Rer1	67830	Protein RE	no | no chan	0	COG5249(F
Q9CQU0	TXD12_M	Txndc12	Txndc12	66073	Thioredoxi	no | no chan	0	ENOG4111
Q62481	VPS72_MO	Vps72	Vps72	21427	Vacuolar p	no | no chan	0	ENOG4111
Q9CQU5	ZWINT_M	Zwint	Zwint	52696	Outer kinet	no | no chan	0	ENOG4111
P63017	HSP7C_M	Hspa8	Hspa8	15481	Heat shock	no | no chan	0	COG0443(F
Q9CQU8	IMP1L_M	Immp1l	Immp1l	66541	Mitochondi	no | no chan	0	COG0681(T
Q9CPU4	MGST3_M	Mgst3	Mgst3	66447	Glutathion	no | up	0	ENOG4111
P63011	RAB3A_M	Rab3a	Rab3a	19339	Ras-related	no | no chan	0	ENOG410
Q3TT94	Q3TT94_M	Ppp2r2a	Ppp2r2a	71978	Serine/thre	no | no chan	0	COG5170(t
Q8BGH2	SAM50_M	Samm50	Samm50	68653	Sorting and	no | no chan	0	COG4775(C
F8VPU2	FARP1_M	Farp1	Farp1	223254	FERM, AR	no | no chan	0	ENOG410
D3Z1N4	D3Z1N4_M	Pbrm1	Pbrm1		Polybromo	no | no chan	0	COG5076(-
F8VPU6	USP9Y_M	Usp9y	Usp9y	107868	Ubiquitin c	no | no chan	0	COG5077(T
Q5SQX6	CYFP2_M	Cyfip2	Cyfip2	76884	Cytoplasmi	no | no chan	0	ENOG410
G5E8S8	G5E8S8_M	Mpp7	Mpp7	75739	MAGUK p	no | down	0	COG0194(U
B2RWX4	B2RWX4_N	Rint1	Rint1	72772	Rint1 prote	no | no chan	0	ENOG410
Q3UMG1	Q3UMG1_	Ly75	Ly75	17076	Lymphocyt	no | no chan	0	ENOG410
P40142	TKT_MOU	Tkt	Tkt	21881	Transketol	no | no chan	0	COG0021(T
P63242	IF5A1_MO	Eif5a	Eif5a	276770	Eukaryotic	no | no chan	0	COG0231(I
Q8CFB4	GBP5_MO	Gbp5	Gbp5	229898	Guanylate-	no | up	0	ENOG410
A2A513	A2A513_M	Krt10	Krt10	16661	Keratin, typ	no | no chan	0	ENOG410
Q8QZY1	EIF3L_MO	Eif3l	Eif3l	223691	Eukaryotic	no | no chan	0	ENOG410
Q8QZY6	TSN14_M	Tspan14	Tspan14	52588	Tetraspani	no | no chan	0	ENOG4111
E9PXX7	E9PXX7_M	Txndc5	Txndc5	105245	protein dis	no | no chan	0	COG0526(p
A2AS03	A2AS03_M	Helz2	Helz2	229003	RNA helico	no | no chan	0	COG1112(F
F6SQM4	F6SQM4_M	Fam234a	Fam234a		Family with	no | no chan	0	ENOG4111
Q8QZY9	SF3B4_MO	Sf3b4	Sf3b4	107701	Splicing fac	no | no chan	0	ENOG410
A0A0A6YW	A0A0A6YW	Mpzl1	Mpzl1		Myelin prot	no | up	0	ENOG4111
B9EJ96	B9EJ96_M	Arhgap26	Arhgap26	71302	Rho GTPas	no | no chan	0	ENOG410
P11087	CO1A1_M	Col1a1	Col1a1	12842	Collagen al	no | no chan	0	ENOG410
A0A075B5	A0A075B5	Ighv10-3	Ighv10-3		Immunoglo	no | up	0	ENOG410
Q7TMX7	Q7TMX7_	Sla2	Sla2	77799	Src-like-ad	no | down	0	ENOG4111
Q7TMX6	Q7TMX6_	Exosc7	Exosc7	66446	Ribosomal	no | no chan	0	COG2123(T

A0A075B5T	A0A075B5T	Ighv6-6	Ighv6-6		Immunoglo	no | no chan	0	ENOG410Y
O09111	NDUBB_M	Ndufb11	Ndufb11	104130	NADH deh	no | no chan	0	ENOG4112
Q8C5N5	PDD2L_M	Pdcd2l	Pdcd2l	68079	Programme	no | no chan	0	ENOG410X
Q3UVC9	Q3UVC9_M	Naa80	Naa80	56441	N-acetyltra	no | no chan	0	ENOG4112
Q3UH59	Q3UH59_M	Myh10	Myh10	77579	Myosin, hec	no | no chan	0	COG5022(I
Q6ZQ08	CNOT1_M	Cnot1	Cnot1	234594	CCR4-NOT	no | no chan	0	COG5103(C
Q9CPY3	CDCA5_M	Cdca5	Cdca5	67849	Sororin	no | no chan	0	ENOG410Y
Q9CPY0	MRM2_MC	Mrm2	Mrm2	68017	rRNA meth	no | no chan	0	COG0293(3
O54724	CAVN1_M	Cavin1	Cavin1	19285	Caveolae-a	no | no chan	0	ENOG410X
Q9CPY6	GID4_MO	Gid4	Gid4	66771	Glucose-in	no | no chan	0	COG5073(C
Q9CPY7	AMPL_MO	Lap3	Lap3	66988	Cytosol am	no | up	0	COG0260(E
O88947	FA10_MOU	F10	F10	14058	Coagulatio	no | up	0	COG5640(S
Q8BJ56	PLPL2_MC	Pnpla2	Pnpla2	66853	Patatin-like	no | no chan	0	ENOG410X
M0QWG1	M0QWG1_	Gse1	Gse1		Genetic sup	no | down	0	ENOG410X
B2RTN5	B2RTN5_M	Arhgap35	Arhgap35	232906	Rho GTPas	no | no chan	0	ENOG410X
Q9Z1Z0	USO1_MO	Uso1	Uso1	56041	General ves	no | no chan	0	ENOG410X
A0A1B0GT	A0A1B0GT	Clcn4	Clcn4	12727	Chloride ch	no | no chan	0	COG0038(P
A0A5F8MP	A0A5F8MP	Kat6a	Kat6a		Histone ace	no | no chan	0	COG5027(A
Q9QXE2	DPOLL_M	Poll	Poll	56626	DNA polyn	no | no chan	0	COG1796(C
A0A1B0GT	A0A1B0GT	Bnip3	Bnip3		BCL2/aden	no | up	0	ENOG4111
Q3V3W7	Q3V3W7_M	F13a1	F13a1	74145	Transglutar	no | no chan	0	ENOG410X
A0A5F8MP	A0A5F8MP	Eed	Eed		Embryonic	no | no chan	0	ENOG410X
Q9QXE7	TBL1X_MC	Tbl1x	Tbl1x	21372	F-box-like/	no | no chan	0	ENOG410X
Q3TV93	Q3TV93_M	Mad2l1	Mad2l1	56150	HORMA do	no | no chan	0	ENOG410X
Q8R2Q4	RRF2M_M	Gfm2	Gfm2	320806	Ribosome-r	no | no chan	0	COG0480(C
Q8C0V9	FRMD6_M	Frmd6	Frmd6	319710	FERM dom	no | no chan	0	ENOG410X
Q8C0V0	TLK1_MO	Tlk1	Tlk1	228012	Serine/thre	no | no chan	0	ENOG410Y
E9Q804	E9Q804_M	Ankrd17	Ankrd17	81702	Ankyrin rep	no | no chan	0	COG0666(M
Q80U46	Q80U46_M	Tcf20	Tcf20	21411	MKIAA029	no | no chan	0	ENOG410Z
Q8R2Q8	BST2_MOU	Bst2	Bst2	69550	Bone marro	no | up	0	ENOG4111
Q9R000	ITBP2_MO	Itgb1bp2	Itgb1bp2	26549	Integrin bet	no | no chan	0	ENOG410X
E9PYZ7	E9PYZ7_M	Rbm26	Rbm26		RNA bindi	no | no chan	0	ENOG4111
Q9R008	KIME_MO	Mvk	Mvk	17855	Mevalonate	no | no chan	0	COG1577(r
E9PV91	E9PV91_M	Adamts4	Adamts4		A disintegri	no | no chan	0	ENOG410X
E9PV93	E9PV93_M	Ly9	Ly9		Lymphocyt	no | no chan	0	ENOG410Z
A0A1W2P8	NOVA2_M	Nova2	Nova2	384569	RNA-bindi	no | down	0	ENOG410X
Q9D3U0	PUS10_MC	Pus10	Pus10	74467	tRNA pseu	no | no chan	0	COG1258(F
A0A494B9Y	A0A494B9Y	Zfpl1	Zfpl1		Zinc finger	no | no chan	0	ENOG410Z
Q3TH73	TTYH2_MC	Ttyh2	Ttyh2	117160	Protein twe	no | up	0	ENOG410Z
Q3U7Z6	Q3U7Z6_M	Pgam1	Pgam1	18648	Phosphogly	no | no chan	0	COG0588(t
E9QAJ9	E9QAJ9_M	Arhgap17	Arhgap17	70497	Rho GTPas	no | no chan	0	ENOG410X
Q9DCZ1	GMPR1_M	Gmpr	Gmpr	66355	GMP reduc	no | no chan	0	COG0516(F
Q9D8C3	ALG13_MC	Alg13	Alg13	67574	UDP-N-ace	no | no chan	0	COG5017(g

E9Q8K8	E9Q8K8_M	Zc3h4	Zc3h4	330474	Zinc finger	no	no chan	0	ENOG410Y
Q6DID3	SCAF8_MC	Scaf8	Scaf8	106583	SR-related	no	no chan	0	ENOG410X
O70138	MMP8_MC	Mmp8	Mmp8	17394	Neutrophil	no	up	0	ENOG410X
Q4FZK2	Q4FZK2_M	Eef1g	Eef1g	67160	Elongation	no	no chan	0	COG0625(s
A0A0N4SW	A0A0N4SW	Itfg2	Itfg2		Integrin alp	no	no chan	0	ENOG410X
E9Q7E2	ARID2_MC	Arid2	Arid2	77044	AT-rich int	no	no chan	0	ENOG410X
F6UNW2	F6UNW2_M	Tmem161a	Tmem161a		Transmemb	no	down	0	ENOG410X
D3YWI4	D3YWI4_M	Mpv17	Mpv17		Protein Mp	no	no chan	0	ENOG4111
Q9CYJ3	Q9CYJ3_M	Exosc10	Exosc10	50912	HRDC dom	no	no chan	0	COG0349(I
Q8BIS8	CC126_MC	Ccdc126	Ccdc126	57895	Coiled-coil	no	no chan	0	ENOG4111
Q8BIS1	Q8BIS1_M	Zfp9	Zfp9	22750	Zinc finger	no	no chan	0	COG5048(I
D3Z6S9	ERIP6_MC	Erich6	Erich6	545527	Glutamate-	no	no chan	0	ENOG4111
Q8BVV5	Q8BVV5_M	Aen	Aen	68048	Exonucleas	no	down	0	COG0847(I
Q3TJ55	Q3TJ55_M	Stx2	Stx2	13852	Syntaxin 2	no	no chan	0	COG5074(C
D3Z6S1	D3Z6S1_M	Tmem214	Tmem214		Transmemb	no	no chan	0	ENOG410Z
Q3U054	Q3U054_M	Cdkn2c	Cdkn2c	12580	Uncharacte	no	no chan	0	COG0666(I
Q8C2U7	Q8C2U7_M	Aimp1	Aimp1	13722	tRNA-bind	no	no chan	0	COG0073(I
Q99M00	Q99M00_M	Fabp4	Fabp4	11770	Fabp4 prot	no	down	0	ENOG4111
Q99M01	SYFM_MO	Fars2	Fars2	69955	Phenylalan	no	no chan	0	COG0072(C
Q3UGB8	Q3UGB8_M	Derl1	Derl1	67819	Derlin	no	no chan	0	COG5291(r
Q6P3Z3	THAP4_MC	Thap4	Thap4	67026	Peroxynitri	no	no chan	0	ENOG4111
A1A596	A1A596_M	Pwp2	Pwp2	110816	Pwp2 prote	no	no chan	0	ENOG410X
Q3UGB7	Q3UGB7_M	Mcph1	Mcph1	244329	Microcepha	yes	down	1	ENOG4112
Q4FJY6	Q4FJY6_M	Ifit3	Ifit3	15959	Ifit3 protein	no	no chan	0	COG0457(C
D3Z0I5	D3Z0I5_M	Trim59	Trim59		Tripartite n	no	no chan	0	ENOG410Y
Q8C0J6	SWAHC_M	Sowahc	Sowahc	268301	Ankyrin rep	no	no chan	0	COG0666(t
Q3TJN6	Q3TJN6_M	Snx5	Snx5	69178	Sorting nex	no	no chan	0	ENOG410X
Q04841	3MG_MOU	Mpg	Mpg	268395	DNA-3-me	no	no chan	0	COG2094(3
A0A8Q0PQ	A0A8Q0PQ	Hypk	Hypk	67693	Huntingtin	no	no chan	0	ENOG4111
Q8BP16	Q8BP16_M	Macroh2a2	Macroh2a2	404634	Core histon	no	down	0	COG5262(h
Q3UDQ3	Q3UDQ3_M	Ipo13	Ipo13	230673	Importin-1	no	no chan	0	ENOG410X
A0A068BE	A0A068BE	Hsd17b8	Hsd17b8	14979	H2-K regio	no	no chan	0	COG1028(s
Q569Y3	Q569Y3_M	Znhit2	Znhit2	29805	Zinc finger,	no	no chan	0	ENOG4111
Q3ULL5	Q3ULL5_M	Eif2s2	Eif2s2	67204	Eukaryotic	no	no chan	0	COG1601(t
Q3ULL7	Q3ULL7_M	Zfp84	Zfp84	74352	Uncharacte	no	no chan	0	COG5048(c
O88796	RPP30_MC	Rpp30	Rpp30	54364	Ribonuclea	no	no chan	0	COG1603(I
P20152	VIME_MO	Vim	Vim	22352	Vimentin	no	no chan	0	ENOG410X
Q91WC0	SETD3_MC	Setd3	Setd3	52690	Actin-histic	no	no chan	0	ENOG410Y
Q9CZB6	MED7_MC	Med7	Med7	66213	Mediator of	no	no chan	0	ENOG410Z
P01027	CO3_MOU	C3	C3	12266	Complemen	no	no chan	0	ENOG410X
A0A494BA	A0A494BA	Rad9a	Rad9a		RAD9 chec	no	no chan	0	ENOG410X
G3X8X0	G3X8X0_M	Dhx16	Dhx16	69192	RNA helico	no	no chan	0	COG1643(C
B1AUX5	B1AUX5_M	Arhgap4	Arhgap4	171207	Rho GTPas	no	no chan	0	ENOG410Y

A0A571BD	A0A571BD	Ube4b	Ube4b		Ubiquitin c	no | no chan	0	COG5113(c
B1AUX2	B1AUX2_M	Hcfc1	Hcfc1	15161	Host cell fa	no | no chan	0	ENOG410Y
G3X8X6	G3X8X6_M	Siglec1	Siglec1	20612	Sialic acid l	no | down	0	ENOG410Z
G3X8X7	G3X8X7_M	Vps16	Vps16	80743	Vacuolar p	no | no chan	0	ENOG410X
P59325	IF5_MOUS	Eif5	Eif5	217869	Eukaryotic	no | no chan	0	COG1601(s
Q9DB27	MCTS1_M	Mcts1	Mcts1	68995	Malignant	no | no chan	0	COG2016(F
Q9DB20	ATPO_MO	Atp5po	Atp5po	28080	ATP syntha	no | no chan	0	COG0712(F
A0A0A6YX	A0A0A6YX	Atp6v1h	Atp6v1h	108664	V-type prot	no | no chan	0	COG5231(s
Q9DB28	POP5_MO	Pop5	Pop5	117109	Ribonuclea	no | no chan	0	COG1369(F
Q9DB29	IAH1_MO	Iah1	Iah1	67732	Isoamyl ace	no | no chan	0	COG2755(I
Q3U3K5	Q3U3K5_M	Leprotl1	Leprotl1	68192	Leptin rece	no | no chan	0	ENOG4111
Q80TJ7	PHF8_MO	Phf8	Phf8	320595; 740	Histone lysi	no | no chan	0	ENOG410X
A0A8D4US	A0A8D4US	Mrtfb	Mrtfb	239719	Megakaryol	no | no chan	0	ENOG411C
Q5SWK7	RN145_MC	Rnf145	Rnf145	74315	RING finge	no | no chan	0	COG5243(z
D3Z3Y1	D3Z3Y1_M	Pigg	Pigg	433931	GPI ethano	no | no chan	0	COG1524(F
O35639	ANXA3_M	Anxa3	Anxa3	11745	Annexin A3	no | no chan	0	ENOG410X
Q8BJM3	R3HCL_M	R3hcc1l	R3hcc1l	52013	Coiled-coil	no | no chan	0	ENOG411C
B2RUE3	B2RUE3_M	Utp14b	Utp14b	195434	UTP14, U3	no | no chan	0	COG5644(S
A5GZX3	A5GZX3_M	Glo1	Glo1	109801	Lactoylglut	no | no chan	0	ENOG4111
B7ZC90	B7ZC90_M	Crocc	Crocc		Ciliary root	no | no chan	0	ENOG410X
A0A0J9YTU	A0A0J9YTU	Specc1	Specc1	432572	Sperm antig	no | no chan	0	COG5069(-
P58544	BTBD1_M	Btbd1	Btbd1	83962	BTB/POZ	no | no chan	0	ENOG410X
S4R1P5	S4R1P5_M	Dst	Dst	13518	Dystonin	no | no chan	0	COG5069(S
A0A338P71	A0A338P71	Rnaset2b	Rnaset2b		Ribonuclea	no | no chan	0	ENOG4111
P42208	SEPT2_MC	Septin2	Septin2	18000	Septin-2	no | no chan	0	COG5019(C
D3Z325	D3Z325_M	Tnfaip8	Tnfaip8	106869	Tumor necr	no | no chan	0	ENOG4111
A0A0G2JE	CFA47_MC	Cfap47	Cfap47	636104	Cilia and fl	no | no chan	0	ENOG410Z
O35242	FAN_MOU	Nsmaf	Nsmaf	18201	Protein FA	no | no chan	0	ENOG410X
A2AHY3	A2AHY3_M	Id1	Id1	15901	Inhibitor of	no | no chan	0	ENOG4111
A0A0H2UK	A0A0H2UK	Usp33	Usp33	170822	Ubiquitin c	no | no chan	0	COG5560(A
A0A0J9YV	A0A0J9YV	Ppm1g	Ppm1g		Protein pho	no | no chan	0	COG0631(S
Q3U2S8	HVCN1_M	Hvcn1	Hvcn1	74096	Voltage-gat	no | no chan	0	ENOG4111
A0A0U1RN	A0A0U1RN	Bcl7c	Bcl7c	12055	B cell CLL/	no | up	0	ENOG4111
A0A2R8VH	A0A2R8VH	Zfp647	Zfp647		Zinc finger	yes | down	1	COG5048(N
Q7TSY8	SGO2A_M	Sgo2a	Sgo2a	68549	Shugoshin 2	no | no chan	0	ENOG410Z
A0A0R4J10	A0A0R4J10	Apeh	Apeh		Acylamino-	no | no chan	0	COG1506(s
A0A0R4J10	A0A0R4J10	Dab2	Dab2	13132	Disabled 2,	no | no chan	0	ENOG410X
Q9EPE9	AT131_MC	Atp13a1	Atp13a1	170759	Endoplasm	no | no chan	0	COG0474(F
A0A0R4J10	A0A0R4J10	Slc4a2	Slc4a2	20535	Anion exch	no | no chan	0	ENOG410X
Q8CIB6	TM230_MC	Tmem230	Tmem230	70612	Transmemb	no | up	0	ENOG4111
B2M0S2	B2M0S2_M	Gm45927	Gm45927	105943584	Secretory cc	no | no chan	0	ENOG410X
Q91VT4	CBR4_MO	Cbr4	Cbr4	234309	3-oxoacyl-[	no | no chan	0	COG1028(I
A0A1E1GJ	A0A1E1GJ	Wgn-scFv	Wgn-scFv		Single-chai	no | no chan	0	ENOG4111

P19426	NELFE_M(	Nelfe	Nelfe	27632	Negative el	no|no chan	0	ENOG411(
B2RSW8	B2RSW8_M	Pcm1	Pcm1	18536	Pericentriol	no|no chan	0	ENOG410Y
A0A1Y7VJ\	A0A1Y7VJ\	Fbln5	Fbln5		Fibulin-5	no|down	0	ENOG411(
A0A286YD	A0A286YD	Tbc1d5	Tbc1d5	72238	TBC1 domc	no|no chan	0	ENOG410Y
Q3U0M1	TPPC9_MC	Trappc9	Trappc9	76510	Trafficking	no|no chan	0	ENOG410Y
Q80X85	RT07_MOU	Mrps7	Mrps7	50529	Small ribos	no|no chan	0	COG0049(C
Q3U0M8	Q3U0M8_M	Elac2	Elac2	68626	Zinc phosp	no|no chan	0	COG1234(Z
Q0WXH3	Q0WXH3_	H2-M3	H2-M3	14991	MHC class	no|up	0	ENOG4111
A2VCT7	A2VCT7_M	Chsy1	Chsy1	269941	Hexosyltran	no|no chan	0	ENOG410X
A0A494B9F	A0A494B9F	Ndfip1	Ndfip1		Nedd4 fami	no|no chan	0	ENOG4111
Q91WP0	MASP2_MC	Masp2	Masp2	17175	Mannan-bi	no|no chan	0	COG5640(c
Q6KAU8	A16L2_MO	Atg16l2	Atg16l2	73683	Protein Atg	no|no chan	0	ENOG410Y
Q3TAV1	Q3TAV1_M	Gpnmb	Gpnmb	93695	PKD doma	no|up	0	ENOG411(
E0CY07	E0CY07_M	Tmem167	Tmem167		Protein kish	no|no chan	0	ENOG4112
Q80ZP8	Q80ZP8_M	Manf	Manf	74840	Armet prote	no|no chan	0	ENOG4111
Q5SSW2	PSME4_MC	Psme4	Psme4	103554	Proteasome	no|no chan	0	ENOG410X
A0A140T8F	A0A140T8F	Igkv6-14	Igkv6-14	667881	Immunoglo	no|up	0	ENOG410Y
A0A2R8W6	A0A2R8W6	Larp4	Larp4		La ribonucl	yes|down	1	ENOG410X
A0A140T8F	A0A140T8F	Igkv12-46	Igkv12-46	692245	Immunoglo	no|no chan	0	ENOG410Y
A0A140T8F	A0A140T8F	Igkv8-24	Igkv8-24	677858	Immunoglo	no|no chan	0	ENOG410Y
Q99KR6	RNF34_MC	Rnf34	Rnf34	80751	E3 ubiquiti	no|no chan	0	ENOG410Y
F6ZDS4	TPR_MOU	Tpr	Tpr	108989	Nucleoprot	no|up	0	ENOG410X
P15089	CBPA3_MC	Cpa3	Cpa3	12873	Mast cell cc	no|down	0	COG2866(Z
E9QMY1	E9QMY1_M	Cd200	Cd200		CD200 mol	no|no chan	0	ENOG410Y
P62281	RS11_MOU	Rps11	Rps11	27207	Small ribos	no|no chan	0	COG0186(C
Q66JW2	Q66JW2_M	Golga1	Golga1	76899	Golga1 pro	no|no chan	0	ENOG410Z
Q8BK72	RT27_MOU	Mrps27	Mrps27	218506	Small ribos	no|no chan	0	ENOG411(
P25444	RS2_MOUS	Rps2	Rps2	16898	Small ribos	no|no chan	0	COG0098(I
Q9WVJ3	CBPQ_MO	Cpq	Cpq	54381	Carboxypep	no|no chan	0	COG2234(M
Q3UK38	Q3UK38_M	Dimt1	Dimt1	66254	rRNA aden	no|no chan	0	COG0030(S
Q5DTT3	TASO2_MC	Tasor2	Tasor2	105203	Protein TAS	no|no chan	0	ENOG410Y
E9Q2E2	E9Q2E2_M	Sdccag8	Sdccag8		Serological	no|no chan	0	ENOG410X
Q0VAW6	Q0VAW6_I	Ark2n	Ark2n	212163	Arkadia (RI	no|no chan	0	ENOG410Z
Q60I30	Q60I30_MC	Itpa	Itpa	16434	Inosine trip	no|no chan	0	COG0127(F
E9Q2E4	E9Q2E4_M	Hectd4	Hectd4	269700	HECT dom	no|no chan	0	COG5184(r
E9Q2E5	E9Q2E5_M	Rapgef3	Rapgef3	223864	Rap guanin	no|down	0	ENOG410X
Q9CYC5	DSN1_MO	Dsn1	Dsn1	66934	Kinetochor	no|no chan	0	ENOG410Y
A0A0N4SW	A0A0N4SW	Tmcc1	Tmcc1	330401	Transmemb	no|no chan	0	ENOG410Y
Q8BKZ9	ODPX_MC	Pdhx	Pdhx	27402	Pyruvate de	no|no chan	0	COG0508(Z
Q9DCV7	K2C7_MOU	Krt7	Krt7	110310	Keratin, typ	no|down	0	ENOG410Y
Q3TIR3	RIC8A_MC	Ric8a	Ric8a	101489	Chaperone	no|no chan	0	ENOG410X
A2AJ30	A2AJ30_MC	Paxx	Paxx		Non-homol	no|no chan	0	ENOG410Y
Q6PGB6	NAA50_MC	Naa50	Naa50	72117	N-alpha-ac	no|no chan	0	

Q791G4	Q791G4_M	Podxl	Podxl	27205	Podocalyxi	no|no chan	0	ENOG410
A0A1B0GR	A0A1B0GR	Bpnt1	Bpnt1	23827	3'(2'),5'-bisp	no|no chan	0	ENOG410
A0A1B0GR	A0A1B0GR	Rfx1	Rfx1		Regulatory	no|no chan	0	ENOG410
Q3V244	Q3V244_M	Eif4h	Eif4h	22384	Eukaryotic	no|no chan	0	COG0724(
A0A1B0GR	A0A1B0GR	Gm17949	Gm17949		Predicted g	no|no chan	0	COG0563(
D3YZS0	D3YZS0_M	Ramac	Ramac		RNA guani	no|no chan	0	ENOG411
Q7TST5	LAMP3_M	Lamp3	Lamp3	239739	Lysosome-a	no|down	0	ENOG410
Q91Z05	Q91Z05_M	Ighg	Ighg	380794	Ighg protei	no|no chan	0	ENOG410
Q8C9C2	Q8C9C2_M	Minpp1	Minpp1	17330	Multiple in	no|no chan	0	ENOG4111
Q3UI98	Q3UI98_M	Gtf3c5	Gtf3c5	70239	General tra	no|no chan	0	ENOG4111
E9PZ21	E9PZ21_M	Rbms1	Rbms1	56878	RNA bindi	no|no chan	0	ENOG410
Q99JX1	TAF11_MC	Taf11	Taf11	68776	Transcripti	no|no chan	0	COG5251(
A0A3B2W4	A0A3B2W4	Snrpc	Snrpc		U1 small n	no|no chan	0	COG5136(
Q3UFE0	Q3UFE0_M	Gm2545	Gm2545	100040000	Uncharacte	no|no chan	0	
A0A4U9FF	A0A4U9FF	Iglc2	Iglc2		IgL2	no|no chan	0	ENOG411
Q8BH69	SPS1_MOU	Sephs1	Sephs1	109079	Selenide, w	no|no chan	0	COG0709(
Q8BMD8	SCMC1_M	Slc25a24	Slc25a24	229731	Mitochond	no|no chan	0	ENOG410
Q5SVD0	RFLB_MO	Rflnb	Rflnb	76566	Refilin-B	no|no chan	0	ENOG410
Q9CQN7	RM41_MO	Mrpl41	Mrpl41	107733	Large ribos	no|no chan	0	ENOG4111
Q9CRD0	OCAD1_M	Ociad1	Ociad1	68095	OCIA dom	no|no chan	0	ENOG4111
Q8BH65	DEN6A_M	Dennd6a	Dennd6a	211922	Protein DE	no|no chan	0	ENOG410
Q8BH64	EHD2_MO	Ehd2	Ehd2	259300	EH domain	no|no chan	0	ENOG410
A2ABV5	MED14_M	Med14	Med14	26896	Mediator o	no|no chan	0	ENOG410
Q91YK2	RRP1B_M	Rrp1b	Rrp1b	72462	Ribosomal	no|no chan	0	ENOG4111
Q91YK7	Q91YK7_M	Fcrl2	Fcrl2	80891	Fc receptor	no|down	0	ENOG410
Q9D4L6	Q9D4L6_M	Rnf220	Rnf220	66743	Ring finger	no|no chan	0	ENOG4111
Q76KJ5	RPA34_MC	Polr1g	Polr1g	70333	DNA-direc	no|no chan	0	ENOG411
P23953	EST1C_MC	Ces1c	Ces1c	13884	Carboxyles	no|up	0	COG2272(
P23950	TISB_MOU	Zfp36l1	Zfp36l1	12192	mRNA dec	no|no chan	0	COG5063(
A2A5A3	A2A5A3_M	Slc35c2	Slc35c2		Solute carri	no|no chan	0	COG0697(
A2APY7	NDUF5_M	Ndufaf5	Ndufaf5	69487	Arginine-h	no|no chan	0	COG0500(
A2APY6	A2APY6_M	Esf1	Esf1	66580	ESF1, nucle	no|no chan	0	COG5638(
B2RXR8	B2RXR8_M	Adamts14	Adamts14	237360	A disintegr	no|no chan	0	ENOG410
B9EKB3	B9EKB3_M	Wwp1	Wwp1	107568	E3 ubiquiti	no|no chan	0	COG5021(
A0A1L1SS2	A0A1L1SS2	Rpl29	Rpl29		60S ribosom	no|no chan	0	ENOG411
B9EKB0	B9EKB0_M	Lamb1	Lamb1	16777	Laminin B1	no|no chan	0	ENOG410
B2RXR2	B2RXR2_M	Pik3r4	Pik3r4	75669	non-specifi	no|no chan	0	ENOG410
B2RXR1	B2RXR1_M	Ccdc88b	Ccdc88b	78317	Coiled-coil	no|up	0	ENOG410
B2RXR6	ANR44_M	Ankrd44	Ankrd44	329154	Serine/thre	no|no chan	0	COG0666(
P47809	MP2K4_M	Map2k4	Map2k4	26398	Dual specif	no|no chan	0	ENOG410
B7ZP49	B7ZP49_M	Zpbp	Zpbp	53604	Zpbp prote	no|no chan	0	ENOG410
B7ZP48	B7ZP48_M	Glp1r	Glp1r	14652	Glp1r prote	no|down	0	ENOG410
B1AWM0	B1AWM0_	Cnbd1	Cnbd1		Cyclic nucl	no|down	0	ENOG4111

Q8VE88	F1142_MO	Fam114a2	Fam114a2	67726	Protein FAI	no | no chan	0	ENOG411(
Q8BH86	GLUCM_M	Dglucy	Dglucy	217830	D-glutamat	no | no chan	0	COG4336(U
B7ZP42	B7ZP42_M	Cask	Cask	12361	Cask protei	no | no chan	0	COG0194(F
A0A1B0GS	A0A1B0GS	Nup98	Nup98	269966	Nuclear po	no | no chan	0	ENOG410X
A0A1B0GS	A0A1B0GS	Pdlim3	Pdlim3	53318	PDZ and L	no | up	0	ENOG410X
B7ZP47	B7ZP47_M	Wapl	Wapl	218914	WAPL cohe	no | no chan	0	ENOG410X
Q6PD19	ARMD3_M	Armh3	Armh3	71617	Armadillo-l	no | no chan	0	ENOG410X
Q99KC8	VMA5A_M	Vwa5a	Vwa5a	67776	von Willeb	no | up	0	COG2304(t
A0A4P3AP	A0A4P3AP	Mapkapk2	Mapkapk2	17164	non-specifi	no | no chan	0	ENOG410X
P01901	HA1B_MO	H2-K1	H2-K1	14972	H-2 class I	no | up	0	ENOG4111
Q52L50	Q52L50_M	Rap1b	Rap1b	215449	small monc	no | up	0	COG1100(-
Q3UT40	Q3UT40_M	Mospd1	Mospd1	70380	Motile sper	no | no chan	0	ENOG4111
P08882	GRAC_MC	Gzmc	Gzmc	14940	Granzyme	no | up	0	COG5640(c
P08883	GRAF_MC	Gzmf	Gzmf	14943	Granzyme	yes | up	1	COG5640(p
Q3V0N3	Q3V0N3_M	Ammecr1l	Ammecr1l	225339	AMMECR1	no | no chan	0	COG2078(c
Q9CRJ8	Q9CRJ8_M	Tfpt	Tfpt	69714	TCF3 fusio	no | no chan	0	ENOG4111
O35114	SCRB2_MC	Scarb2	Scarb2	12492	Lysosome n	no | no chan	0	ENOG410X
Q91XC0	AJUBA_M	Ajuba	Ajuba	16475	LIM domai	no | no chan	0	ENOG410Y
Q3U260	Q3U260_M	B4galt3	B4galt3	57370	Beta-1,4-ga	no | no chan	0	ENOG410Z
Q0P4Z5	Q0P4Z5_M	Gpatch3	Gpatch3	242691	Gpatch3 pr	no | no chan	0	ENOG410X
Q91XC9	PEX16_MC	Pex16	Pex16	18633	Peroxisoma	no | no chan	0	ENOG4111
Q3U269	TSTD2_MC	Tstd2	Tstd2	272027	Thiosulfate	no | no chan	0	COG1054(U
D3Z4P2	D3Z4P2_M	Lman2l	Lman2l	214895	Lectin, mar	no | no chan	0	ENOG410Y
E0CYH7	E0CYH7_M	Filip1l	Filip1l	78749	Filamin A i	no | no chan	0	ENOG410Z
E0CYH0	E0CYH0_M	Wtap	Wtap	60532	Pre-mRNA-	no | no chan	0	ENOG410X
P70188	KIFA3_MC	Kifap3	Kifap3	16579	Kinesin-ass	no | no chan	0	ENOG410X
Q8BTS3	Q8BTS3_M	Gbp9	Gbp9	236573	Guanylate-	no | up	0	ENOG410X
Q8BTS4	NUP54_MC	Nup54	Nup54	269113	Nuclear po	no | no chan	0	ENOG410X
A2AQE2	A2AQE2_M	Secisbp2l	Secisbp2l	70354	SECIS bind	no | no chan	0	ENOG410Z
Q3UJA1	Q3UJA1_M	Rrp12	Rrp12	107094	Ribosomal	no | no chan	0	ENOG410X
Q4FJN2	Q4FJN2_M	Fkbp5	Fkbp5	14229	peptidylpro	no | no chan	0	COG0545(A
Q80YQ8	RMD5A_M	Rmnd5a	Rmnd5a	68477	E3 ubiquiti	no | no chan	0	COG5109(F
Q80YQ2	MED23_M	Med23	Med23	70208	Mediator o	no | no chan	0	ENOG410Z
G3UZT8	G3UZT8_M	Jarid2	Jarid2		Jumonji an	no | down	0	ENOG410Y
Q8BUI3	LRWD1_M	LRWD1	LRWD1	71735	Leucine-ric	no | no chan	0	COG4886(N
Q3U0P5	ENTP6_M	Entpd6	Entpd6	12497	Ectonucleo	no | no chan	0	COG5371(t
Q3U0P4	Q3U0P4_M	Il36g	Il36g	215257	Interleukin	no | up	0	ENOG4111
Q3TGW2	EEPD1_M	Eepd1	Eepd1	67484	Endonuclec	no | no chan	0	COG1555(C
M0QW48	M0QW48_M	Metap2	Metap2		Methionine	no | no chan	0	COG0024(T
A0A0U1RP	A0A0U1RP	Emsy	Emsy		EMSY, BRC	no | no chan	0	ENOG410Y
D3Z667	D3Z667_M	Dnah2	Dnah2	327954	Dynein, axc	no | no chan	0	COG5245(f
Q14AZ9	Q14AZ9_M	Ptgr3	Ptgr3	225791	Zinc bindin	no | no chan	0	COG2130(s
D3YXQ1	D3YXQ1_M	Tceanc2	Tceanc2		Transcripti	no | no chan	0	COG1594(I

Q91WU4	TMCO4_M	Tmco4	Tmco4	77056	Transmemb	no | no chan	0	ENOG410Y
D3YXQ5	D3YXQ5_M	Atp8b2	Atp8b2		Phospholip	no | no chan	0	COG0474(V
P70452	STX4_MOU	Stx4	Stx4	20909	Syntaxin-4	no | no chan	0	COG5074(p
A0A1W2P7	A0A1W2P7	Gm49339	Gm49339		Predicted g	no | no chan	0	ENOG4111
A0A1W2P7	A0A1W2P7	Abracl	Abracl		Costars fam	no | no chan	0	ENOG410X
Q9R0M5	TPK1_MOU	Tpk1	Tpk1	29807	Thiamine p	no | no chan	0	COG1564(t
E9QAT4	SC16A_MC	Sec16a	Sec16a	227648	Protein tran	no | no chan	0	ENOG4111
Q91WU0	EST1F_MC	Ces1f	Ces1f	234564	Carboxylest	no | down	0	COG2272(C
Q80W21	GSTM7_MO	Gstm7	Gstm7	68312	Glutathion	no | no chan	0	ENOG411C
A0JLV3	A0JLV3_MO	Hist1h2bj	Hist1h2bj	319183	Histone H2	no | no chan	0	ENOG4111
A0A9L6KD	A0A9L6KD	Creld1	Creld1		protein disu	yes | up	1	ENOG411C
Q78HW2	Q78HW2_M	Tmem186	Tmem186	66690	Transmemb	no | down	0	ENOG4111
Q8BLB7	LMBL3_MO	L3mbtl3	L3mbtl3	237339	Lethal(3)mb	no | no chan	0	ENOG410Y
Q7TNS2	MIC10_MC	Micos10	Micos10	433771	MICOS com	no | no chan	0	ENOG4112
K7T926	K7T926_MOUSE	IgM heavy chain variable reg			IgM heavy c	no | down	0	ENOG410Y
Q8C5B3	Q8C5B3_M	Lamb3	Lamb3	16780	Uncharacte	no | no chan	0	ENOG410X
Q8K426	RETNG_M	Retnlg	Retnlg	245195	Resistin-lik	no | no chan	0	ENOG4102
Q3U3G8	Q3U3G8_M	Serhl	Serhl	68607	Serine hydr	no | no chan	0	COG0596(T
Q6PHU5	SORT_MO	Sort1	Sort1	20661	Sortilin	no | no chan	0	ENOG4102
Q3TUQ7	Q3TUQ7_M	Prkaa1	Prkaa1	105787	Acetyl-CoA	no | no chan	0	ENOG410X
H3BLB7	H3BLB7_M	Igfbp4	Igfbp4		Insulin-like	no | no chan	0	ENOG4111
Q8VIK2	M17L2_MC	Mpv17l2	Mpv17l2	234384	Mpv17-like	no | no chan	0	ENOG4111
Q3UIA4	Q3UIA4_M	Aldh1a3	Aldh1a3	56847	Aldehyde de	no | no chan	0	COG1012(I
A0A1W2P7	A0A1W2P7	Pcnt	Pcnt	18541	Pericentrin	no | no chan	0	ENOG411C
Q9CQW9	IFM3_MOU	Ifitm3	Ifitm3	66141	Interferon-i	no | no chan	0	ENOG4112
Q8BPH4	Q8BPH4_M	Taf7	Taf7	24074	Transcripti	no | no chan	0	COG5414(I
Q9JHU9	INO1_MOU	Isyna1	Isyna1	71780	Inositol-3-p	no | no chan	0	COG1260(s
Q8C3I8	HGH1_MC	Hgh1	Hgh1	59053	Protein HG	no | no chan	0	ENOG410Y
Q3U3G2	Q3U3G2_M	Rnf126	Rnf126	70294	RING-type	no | up	0	ENOG4112
Q5SRX1	TM1L2_MC	Tom1l2	Tom1l2	216810	TOM1-like	no | no chan	0	ENOG410Y
Q3U8U1	Q3U8U1_M	Vav1	Vav1	22324	Proto-onco	no | no chan	0	ENOG410X
A4FU75	A4FU75_M	Dagla	Dagla	269060	sn-1-specifi	no | no chan	0	ENOG410X
P27661	H2AX_MO	H2ax	H2ax	15270	Histone H2	no | no chan	0	COG5262(X
Q8C3X2	CC90B_MC	Ccdc90b	Ccdc90b	66365	Coiled-coil	no | no chan	0	ENOG4111
Q91ZW2	OFUT1_MO	Pofut1	Pofut1	140484	GDP-fucos	no | no chan	0	ENOG410Y
Q91ZW3	SMCA5_MO	Smarca5	Smarca5	93762	SWI/SNF-r	no | no chan	0	COG0553(k
A0A0R4J0S	A0A0R4J0S	Uaca	Uaca	72565	Uveal autoa	no | no chan	0	COG0666(C
A0A0R4J0S	A0A0R4J0S	Llgl1	Llgl1	16897	LLGL1 scri	no | no chan	0	ENOG410X
A0A0R4J0S	A0A0R4J0S	Rtn4ip1	Rtn4ip1	170728	Reticulon 4	no | no chan	0	COG0604(t
A0A0R4J0S	A0A0R4J0S	Igfals	Igfals	16005	Insulin-like	no | up	0	COG4886(C
A0A0R4J0S	A0A0R4J0S	Cdc42ep1	Cdc42ep1	104445	CDC42 effe	no | no chan	0	ENOG4111
D3YUJ1	D3YUJ1_M	Rnf181	Rnf181	66510	E3 ubiquiti	no | no chan	0	ENOG4112
D3YUJ0	D3YUJ0_M	Evc	Evc		EvC ciliary	no | no chan	0	ENOG4102

A0A1R3UL	A0A1R3UL	Klk1b26	Klk1b26	16618	Kallikrein	no	no chan	0	COG5640(p
A0ABA7IX	A0ABA7IXA3_MOUSE CDC-like kinase 2 OS=		Clk2			no	no chan	0	ENOG410X
O08663	MAP2_MO	Metap2	Metap2	56307	Methionine	no	no chan	0	COG0024(k
Q91V61	SFXN3_M(	Sfxn3	Sfxn3	94280	Sideroflexir	no	no chan	0	ENOG410Y
A2AFW6	A2AFW6_M	Mtch2	Mtch2	56428	Mitochondı	no	down	0	ENOG410X
Q8VC51	TCAB1_M(	Wrap53	Wrap53	216853	Telomerase	no	no chan	0	ENOG410X
C6EQG6	C6EQG6_MOUSE ASL1/Ifi205 fusion protein		ASL1/Ifi20.			no	up	0	ENOG410Y
B1ARW4	B1ARW4_M	Ndufs5	Ndufs5		NADH deh	no	no chan	0	ENOG4112
A0A494BB!	A0A494BB!	Tmem179b	Tmem179b	67706	Transmemb	no	no chan	0	ENOG4112
B1ARW8	CA122_MOUSE Uncharacterized p			68920	Uncharacte	no	no chan	0	ENOG4111
Q3TLE2	Q3TLE2_M	Fermt2	Fermt2	218952	PH domain	no	no chan	0	ENOG410X
Q3UDF0	GTR6_MO	Slc2a6	Slc2a6	227659	Solute carri	no	no chan	0	ENOG410X
D3YTR3	D3YTR3_M	Mex3d	Mex3d	237400	Mex3 RNA	no	up	0	ENOG410Y
E9Q317	E9Q317_M	Sap18b	Sap18b	20220; 1000	Histone dec	no	no chan	0	ENOG4111
P63038	CH60_MOI	Hspd1	Hspd1	15510	60 kDa hea	no	no chan	0	COG0459(t
E9PX59	E9PX59_M	Samd9l	Samd9l	209086	Sterile alph	no	no chan	0	ENOG4112
A8IP69	A8IP69_M(	Ywhag	Ywhag	22628	14-3-3 prote	no	up	0	COG5040(t
E9PX53	E9PX53_M	Ppp4r1	Ppp4r1	70351	Protein phc	no	no chan	0	ENOG410X
A0A2K6EL	A0A2K6EL	Mbnl2	Mbnl2		Muscleblin	yes	up	1	ENOG410Z
E9PX50	E9PX50_M	Slc8a3	Slc8a3	110893	Solute carri	no	no chan	0	ENOG410X
Q5SQF8	SP30L_MO	Sap30l	Sap30l	50724	Histone dec	no	no chan	0	ENOG4111
P63037	DNJA1_M(	Dnaja1	Dnaja1	15502	DnaJ homo	no	no chan	0	COG0484(t
Q8K1M6	DNM1L_M	Dnm1l	Dnm1l	74006	Dynamin-1	no	no chan	0	
Q99P51	RASF3_MC	Rassf3	Rassf3	192678	Ras associa	no	no chan	0	ENOG410Y
D3Z1H6	D3Z1H6_M	Trpv4	Trpv4		Transient r	no	no chan	0	ENOG411(
Q3V2Q8	N42L1_MC	N4bp2l1	N4bp2l1	100637	NEDD4-bi	no	no chan	0	ENOG411(
Q3UJ95	Q3UJ95_M	Pip4k2b	Pip4k2b	108083	PIPK doma	no	no chan	0	COG5253(p
Q8BZM1	GLMN_M(	Glmn	Glmn	170823	Glomulin	no	no chan	0	ENOG410Y
P40124	CAP1_MOI	Cap1	Cap1	12331	Adenylyl cy	no	no chan	0	ENOG410X
Q0VBL3	RBM15_M(	Rbm15	Rbm15	229700	RNA-bindi	no	no chan	0	ENOG410X
Q3TFP0	Q3TFP0_M	Srsf10	Srsf10	14105	Serine and	no	no chan	0	ENOG410Y
A0A1Y7VM	A0A1Y7VM	Amotl1	Amotl1	75723	Angiomotir	no	no chan	0	ENOG410X
Q3UBT6	Q3UBT6_M	Csnk1d	Csnk1d	104318	Casein kina	no	no chan	0	ENOG410X
A0A075B5\	A0A075B5\	Ighv1-47	Ighv1-47		Immunoglo	no	down	0	ENOG4111
A0A075B5\	A0A075B5\	Ighv1-43	Ighv1-43		Immunoglo	no	up	0	ENOG4111
Q9CZ82	MED18_M	Med18	Med18	67219	Mediator o	no	no chan	0	ENOG411(
A0A075B5\	A0A075B5\	Ighv1-39	Ighv1-39		Immunoglo	no	no chan	0	ENOG4111
O09130	NF2IP_MC	Nfatc2ip	Nfatc2ip	18020	NFATC2-ir	no	no chan	0	COG5227((
O09131	GSTO1_M(	Gsto1	Gsto1	14873	Glutathion	no	no chan	0	ENOG410X
A0A2R8VJ\	A0A2R8VJ\	Aco2	Aco2		Aconitase 2	no	up	0	COG1048(c
Q7TQJ1	MALD1_M	Marveld1	Marveld1	277010	MARVEL d	no	no chan	0	ENOG4111
O09135	O09135_M(	USF1	USF1	22278	Upstream s	no	no chan	0	ENOG411(
A0A5F8MC	A0A5F8MC	Ppp6r2	Ppp6r2	71474	Protein phc	no	no chan	0	ENOG410X

Q7TQJ8	WTIP_MO	Wtip	Wtip	101543	Wilms tum	no|up	0	ENOG410
A0A5F8MC	A0A5F8MC	Rxra	Rxra		Retinoic ac	no|no chan	0	ENOG410
Q3UH70	Q3UH70_M	Brd4	Brd4	57261	Bromodom	no|no chan	0	COG5076(
D3YYN7	D3YYN7_M	Atp1a2	Atp1a2		Sodium/po	no|up	0	COG0474(
Q923L7	Q923L7_M	Ear6	Ear6	93719	Ear6 protei	no|down	0	ENOG4111
Q3UR97	SNX21_MC	Snx21	Snx21	101113	Sorting nex	no|no chan	0	ENOG4111
E0CYW1	E0CYW1_M	Ubl5	Ubl5		Ubiquitin-l	no|no chan	0	ENOG4111
Q9CPV5	PMF1_MO	Pmf1	Pmf1	67037	Polyamine-	no|no chan	0	ENOG4111
B2RTL5	B2RTL5_M	Aldh1a7	Aldh1a7	26358	Aldehyde d	no|no chan	0	COG1012(
B2RTL6	B2RTL6_M	Thbs4	Thbs4	21828	Thrombosp	no|no chan	0	ENOG410
Q9CR80	FA32A_MC	Fam32a	Fam32a	67922	Protein FA	no|no chan	0	ENOG4112
A7E2R9	A7E2R9_M	Nfam1	Nfam1	74039	Nfam1 prot	no|up	0	ENOG4111
B2RTL2	B2RTL2_M	Pde4a	Pde4a	18577	Phosphodie	no|no chan	0	ENOG410
O35344	IMA4_MO	Kpna3	Kpna3	16648	Importin su	no|no chan	0	COG5064(
O35345	IMA7_MO	Kpna6	Kpna6	16650	Importin su	no|no chan	0	COG5064(
Q9CR89	ERGI2_MC	Ergic2	Ergic2	67456	Endoplasm	no|no chan	0	ENOG410
A2A9W3	A2A9W3_M	Mif4gd	Mif4gd	69674	MIF4G don	no|no chan	0	ENOG410
A0A1Y7VL	A0A1Y7VL	Yae1d1	Yae1d1		Yae1 doma	yes|up	1	ENOG4111
A2A9W7	A2A9W7_M	Gga3	Gga3	260302	Golgi assoc	no|no chan	0	ENOG410
Q683Y7	Q683Y7_M	IGHV	IGHV		Immunoglo	no|no chan	0	ENOG4111
B7ZN90	B7ZN90_M	Itgal	Itgal	16408	Itgal protei	no|up	0	ENOG410
O88967	YMEL1_M	Yme1l1	Yme1l1	27377	ATP-depen	no|no chan	0	COG0465(
O88968	TCO2_MO	Tcn2	Tcn2	21452	Transcobal	no|no chan	0	ENOG410
A0A5F8MP	A0A5F8MP	Klc1	Klc1		Kinesin ligl	no|no chan	0	COG0457(
Q8BJ71	NUP93_MC	Nup93	Nup93	71805	Nuclear po	no|no chan	0	ENOG410
H7BX33	H7BX33_M	4933434E2(	4933434E20Rik		RIKEN cD	no|no chan	0	ENOG410
F8WIU9	F8WIU9_M	Smim7	Smim7		Small integ	no|no chan	0	ENOG4111
F8WIU7	F8WIU7_M	Prmt9	Prmt9	102182	Protein arg	no|no chan	0	ENOG410
A0A1B0GT	A0A1B0GT	Gm45785	Gm45785		BET1-like p	no|up	0	ENOG4111
Q9D735	TRIR_MO	Trir	Trir	68544	Telomerase	no|no chan	0	ENOG4111
F8WIU1	F8WIU1_M	Adissp	Adissp	67326	Adipose-sec	no|no chan	0	ENOG4111
O70480	VAMP4_M	Vamp4	Vamp4	53330	Vesicle-ass	no|down	0	COG5143(
O70481	UBR1_MO	Ubr1	Ubr1	22222	E3 ubiquiti	no|no chan	0	ENOG410
Q3UHB1	NT5D3_M	Nt5dc3	Nt5dc3	103466	5'-nucleotid	no|no chan	0	ENOG410
P10605	CATB_MO	Ctsb	Ctsb	13030	Cathepsin I	no|no chan	0	COG4870(
G3UZY2	G3UZY2_M	Txn2	Txn2		Thioredoxi	no|up	0	COG0526(
Q8R2S9	ARP8_MO	Actr8	Actr8	56249	Actin-relat	no|no chan	0	COG5277(
Q03147	CDK7_MO	Cdk7	Cdk7	12572	Cyclin-depe	no|no chan	0	ENOG410
Q3U9S2	Q3U9S2_M	Edem1	Edem1	192193	alpha-1,2-M	no|no chan	0	ENOG410
P22437	PGH1_MO	Ptgs1	Ptgs1	19224	Prostaglanc	no|no chan	0	ENOG410
P16460	ASSY_MO	Ass1	Ass1	11898	Argininosu	no|no chan	0	COG0137(
E9PYT3	E9PYT3_M	Atl3	Atl3	109168	Atlastin GT	no|no chan	0	ENOG410
P62073	TIM10_MC	Timm10	Timm10	30059	Mitochond	no|no chan	0	ENOG4112

P62071	RRAS2_MC	Rras2	Rras2	66922	Ras-related	no | no chan	0	COG1100(c
P62077	TIM8B_MC	Timm8b	Timm8b	30057	Mitochondı	no | no chan	0	ENOG4112
P62075	TIM13_MC	Timm13	Timm13	30055	Mitochondı	no | no chan	0	ENOG4112
Q7TNL5	Q7TNL5_M	Ppp2r5d	Ppp2r5d	21770	Serine/thre	no | no chan	0	ENOG410X
Q7TNL7	Q7TNL7_M	Dusp9	Dusp9	75590	Dual specif	no | no chan	0	COG2453(u
F8WGX0	F8WGX0_M	Fgd4	Fgd4		FYVE, Rho	no | up	0	ENOG410X
P50462	CSRP3_MC	Csrp3	Csrp3	13009	Cysteine an	no | up	0	ENOG410Z
F8WGX5	F8WGX5_M	Dmxl1	Dmxl1		Dmx-like 1	no | no chan	0	ENOG410X
Q9D880	TIM50_MC	Timm50	Timm50	66525	Mitochondı	no | no chan	0	COG5190(r
B0R091	B0R091_M	Chp1	Chp1		Calcineurin	no | no chan	0	COG5126(F
Q91WM2	HDHD5_M	Hdhd5	Hdhd5	214932	Haloacid de	no | no chan	0	COG0647(F
Q91WM1	STRBP_MC	Strbp	Strbp	20744	Spermatid p	no | no chan	0	ENOG410Z
A0A1L1SQ.	A0A1L1SQ.	Tln2	Tln2		Talin 2	no | no chan	0	ENOG410X
Q9DCX2	ATP5H_MC	Atp5pd	Atp5pd	71679	ATP syntha	no | no chan	0	ENOG4111
O70157	TOP3A_MC	Top3a	Top3a	21975	DNA topoi	no | no chan	0	COG0550(M
E9Q8I7	E9Q8I7_M	Nfxl1	Nfxl1	100978	Nuclear tra	no | no chan	0	ENOG410X
B2RRR9	B2RRR9_M	Fut11	Fut11	73068	Alpha-(1,3)-	no | no chan	0	ENOG410Z
Q8CAQ8	MIC60_MC	Immt	Immt	76614	MICOS con	no | no chan	0	ENOG410Y
A0A0N4SW	A0A0N4SW	Gng12	Gng12		Guanine nu	no | no chan	0	ENOG4112
Q75N62	GIMA8_MC	Gimap8	Gimap8	243374	GTPase IM	no | no chan	0	ENOG410X
A0A2I3BPT	A0A2I3BPT	App	App	11820	Amyloid-be	no | no chan	0	ENOG410Z
E9QNX9	E9QNX9_M	Igf1r	Igf1r	16001	Tyrosine-pr	no | no chan	0	COG0515(S
Q9DC48	PRP17_MC	Cdc40	Cdc40	71713	Pre-mRNA-	no | no chan	0	ENOG410X
Q99M28	RNPS1_MC	Rnps1	Rnps1	19826	RNA-bindi	no | no chan	0	ENOG4111
Q8C0T5	SI1L1_MO	Sipa1l1	Sipa1l1	217692	Signal-indu	no | no chan	0	ENOG410X
Q8BVP5	KC1G2_MC	Csnk1g2	Csnk1g2	103236	Casein kina	no | no chan	0	ENOG410X
Q8C0T1	FM1AB_MC	Fem1al	Fem1al	216622	Protein fem	no | no chan	0	COG0666(C
P34928	APOC1_MC	Apoc1	Apoc1	11812	Apolipopro	no | no chan	0	ENOG410Z
Q8VBX6	MPDZ_MC	Mpdz	Mpdz	17475	Multiple PI	no | no chan	0	ENOG411C
Q99K30	ES8L2_MO	Eps8l2	Eps8l2	98845	Epidermal g	no | up	0	ENOG410X
Q6NZQ2	DDX31_MC	Ddx31	Ddx31	227674	ATP-depen	no | no chan	0	ENOG410X
Q3TG23	Q3TG23_M	Zcchc17	Zcchc17	619605	Nucleolar p	no | up	0	COG1185(I
Q3TG21	Q3TG21_M	Atp6v1c1	Atp6v1c1	66335	V-type prot	no | no chan	0	COG5127(A
Q9D8M4	RL7L_MOU	Rpl7l1	Rpl7l1	66229	Large ribos	no | no chan	0	COG1841(T
Q9JIB0	MOG1_MC	Rangrf	Rangrf	57785	Ran guanin	no | no chan	0	ENOG4111
D3YUU4	D3YUU4_M	Cln3	Cln3		Battenin	no | no chan	0	ENOG410X
P51150	RAB7A_MC	Rab7a	Rab7a	19349	Ras-related	no | no chan	0	ENOG410X
Q80UN9	MOD5_MC	Trit1	Trit1	66966	tRNA dime	no | no chan	0	COG0324(C
P29788	VTNC_MC	Vtn	Vtn	22370	Vitronectin	no | up	0	ENOG410X
Q61584	FXR1_MOU	Fxr1	Fxr1	14359	RNA-bindi	no | no chan	0	ENOG410Z
Q61586	GPAT1_MC	Gpam	Gpam	14732	Glycerol-3-	no | no chan	0	COG2937(c
Q8BKG3	PTK7_MOU	Ptk7	Ptk7	71461	Inactive tyr	no | down	0	COG0515(I
Q00493	CBPE_MO	Cpe	Cpe	12876	Carboxypep	no | up	0	ENOG410X

B1AR09	B1AR09_M	Mllt6	Mllt6	246198	Myeloid/ly	no | no chan	0	COG5141(p
Q8BP71	RFOX2_M	Rbfox2	Rbfox2	93686	RNA bindi	no | no chan	0	ENOG4111
Q04863	RELB_MO	Relb	Relb	19698	Transcripti	no | no chan	0	ENOG410Z
Q8BP74	PSTK_MO	Pstk	Pstk	214580	L-seryl-tRN	no | no chan	0	ENOG4111
Q8BP78	F10C1_MO	Fra10ac1	Fra10ac1	70567	Protein FR	no | no chan	0	ENOG410Y
Q924H7	WAC_MO	Wac	Wac	225131	WW domai	no | no chan	0	ENOG4111
A2A7B5	A2A7B5_M	Prdm2	Prdm2	110593	PR domain	no | no chan	0	ENOG410Z
D6RG69	D6RG69_M	Ccdc167	Ccdc167		Coiled-coil	no | no chan	0	ENOG410Y
E9Q1S3	E9Q1S3_M	Sec23a	Sec23a		Protein tra	no | no chan	0	COG5047(t
Q7TPQ8	Q7TPQ8_M	Gcnt2	Gcnt2	14538	Glucosami	no | no chan	0	ENOG410X
Q3UQI9	MINY4_M	Mindy4	Mindy4	330323	Probable ul	no | up	0	ENOG410X
Q7TPQ3	SHPRH_M	Shprh	Shprh	268281	E3 ubiquiti	no | no chan	0	COG0553(F
A2ASF9	A2ASF9_M	Pde1a	Pde1a		Phosphodie	no | up	0	ENOG410X
P02463	CO4A1_M	Col4a1	Col4a1	12826	Collagen al	no | no chan	0	ENOG410X
E9Q481	E9Q481_M	Ppp4r3a	Ppp4r3a	68734	SMEK hom	no | no chan	0	ENOG410X
Q8CGK3	LONM_M	Lonp1	Lonp1	74142	Lon protea	no | no chan	0	COG0466(V
P05202	AATM_M	Got2	Got2	14719	Aspartate a	no | no chan	0	COG1448(c
P05201	AATC_MO	Got1	Got1	14718	Aspartate a	no | no chan	0	COG1448(c
Q3ULP3	Q3ULP3_M	Ralbp1	Ralbp1	19765	Rho-GAP d	no | no chan	0	ENOG410X
A2AKW0	A2AKW0_	Slc25a51	Slc25a51	230125	Solute carri	no | no chan	0	ENOG410X
Q497T6	Q497T6_M	Commd2	Commd2	52245	COMM dor	no | no chan	0	ENOG410X
Q8BTJ4	ENPP4_M	Enpp4	Enpp4	224794	Bis(5'-aden	no | no chan	0	COG1524(r
Q60989	XIAP_MO	Xiap	Xiap	11798	E3 ubiquiti	no | no chan	0	ENOG410Y
Q3TW27	Q3TW27_M	Nelfcd	Nelfcd	57314	Negative el	no | no chan	0	ENOG410X
Q3V2N5	Q3V2N5_M	Sntb2	Sntb2	20650	Beta-2-synt	no | no chan	0	ENOG4111
Q8BR94	Q8BR94_MOUSE	Ectonucleoside t		67464	Ectonucleo	no | no chan	0	COG5371(F
Q8BPZ8	ABRX1_M	Abraxas1	Abraxas1	70681	BRCA1-A c	no | no chan	0	ENOG411(
Q812F8	MGT4B_M	Mgat4b	Mgat4b	103534	Alpha-1,3-r	no | no chan	0	ENOG410X
Q9EQC8	Q9EQC8_M	Prcc	Prcc	94315	Papillary R	no | no chan	0	ENOG4111
B1AUQ7	B1AUQ7_M	Cetn2	Cetn2		Centrin 2	no | no chan	0	COG5126(C
A0A146E66	A0A146E66	Islr	Islr	26968	Mesenchym	no | no chan	0	COG4886(i
E9PWC8	E9PWC8_M	Papola	Papola		Poly(A) pol	no | no chan	0	COG5186(F
Q9CQJ8	NDUB9_M	Ndufb9	Ndufb9	66218	NADH deh	no | no chan	0	ENOG4111
P08071	TRFL_MO	Ltf	Ltf	17002	Lactotransf	no | up	0	ENOG410X
P08074	CBR2_MO	Cbr2	Cbr2	12409	Carbonyl re	no | down	0	ENOG410X
B2RUG8	B2RUG8_M	Asxl2	Asxl2	75302	Asxl2 prote	no | no chan	0	ENOG4111
B2RUG9	B2RUG9_M	Apc	Apc	11789	APC, WNT	no | down	0	ENOG410X
Q9Z0E6	GBP2_MO	Gbp2	Gbp2	14469	Guanylate-	no | up	0	ENOG410X
Q9D7K2	TEN1L_M	Ten1	Ten1	69535	CST compl	no | no chan	0	ENOG4111
B2RUG2	B2RUG2_M	Mib2	Mib2	76580	RING-type	no | no chan	0	ENOG410X
B2RUG3	B2RUG3_M	Slc27a3	Slc27a3	26568	long-chain-	no | no chan	0	ENOG410X
B2RUG4	B2RUG4_M	Gli3	Gli3	14634	GLI-Krupp	no | no chan	0	COG5048(S
B2RUG5	B2RUG5_M	Cfhr4	Cfhr4	214403	EG214403	no | no chan	0	ENOG410Y

Q8C156	CND2_MC	Ncaph	Ncaph	215387	Condensin	no | no chan	0	COG5229(I
Q9QUM9	PSA6_MOU	Psma6	Psma6	26443	Proteasome	no | no chan	0	ENOG410X
Q8C153	Q8C153_M	Eef2	Eef2	13629	Tr-type G d	no | no chan	0	COG0480((
P62983	RS27A_MC	Rps27a	Rps27a	78294	Ubiquitin-r	no | no chan	0	COG5272((
Q3TKV1	Q3TKV1_M	Psmd2	Psmd2	21762	26S proteas	no | no chan	0	COG5110(2
P35295	RAB20_MC	Rab20	Rab20	19332	Ras-related	no | no chan	0	ENOG410Y
P35293	RAB18_MC	Rab18	Rab18	19330	Ras-related	no | no chan	0	ENOG410X
Q3ULN6	Q3ULN6_M	Sccpdh	Sccpdh	109232	Saccharopi	no | no chan	0	COG3268(s
P35290	RAB24_MC	Rab24	Rab24	19336	Ras-related	no | no chan	0	ENOG410Y
P42228	STAT4_MC	Stat4	Stat4	20849	Signal trans	no | no chan	0	ENOG410X
A0A286YD	A0A286YD	Selenok	Selenok		Selenoprote	no | no chan	0	ENOG410X
Q3TYX3	SMYD5_M	Smyd5	Smyd5	232187	Protein-lysi	no | no chan	0	COG2940(p
Q3TYX2	LRN4L_M	Lrrn4cl	Lrrn4cl	68852	LRRN4 C-t	no | down	0	ENOG410Y
A0A0G2JEI	A0A0G2JEI	Sh2b2	Sh2b2	23921	SH2B adap	no | no chan	0	ENOG4110
Q8BSX3	Q8BSX3_M	Pck1	Pck1	18534	Phosphoen	no | down	0	COG1274((
Q91VV4	DEN2D_M	Dennd2d	Dennd2d	72121	DENN don	no | down	0	ENOG410X
Q3TNH5	ARB2A_M	Arb2a	Arb2a	68675	Cotranscrip	no | no chan	0	ENOG410Z
Q9EPK5	WWTR1_M	Wwtr1	Wwtr1	97064	WW domai	no | no chan	0	ENOG4111
P18155	MTDC_MC	Mthfd2	Mthfd2	17768	Bifunctionc	no | down	0	COG0190(I
A0A338P7L	A0A338P7L	Chmp2b	Chmp2b		Charged m	no | no chan	0	ENOG4111
Q99PU7	BAP1_MOU	Bap1	Bap1	104416	Ubiquitin c	no | no chan	0	ENOG410X
A0A0R4J0L	A0A0R4J0L	Cog6	Cog6	67542	Conserved	no | no chan	0	ENOG410Y
Q6VH22	IF172_MOU	Ift172	Ift172	67661	Intraflagell	no | no chan	0	ENOG410X
Q6DG52	CHUR_MC	Churc1	Churc1	211151	Protein Chu	no | no chan	0	ENOG4112
Q05CC8	Q05CC8_M	1600012H0	1600012H0	67912	1600012H0	no | up	0	ENOG4112
A0A571BE	A0A571BE	Plin4	Plin4		Perilipin 4	no | no chan	0	ENOG4112
Q4QQL3	Q4QQL3_M	Ctso	Ctso	229445	Cathepsin (	no | no chan	0	COG4870(c
B7ZNG4	TROAP_M	Troap	Troap	78733	Tastin	no | no chan	0	ENOG4111
A0A140LIZ	A0A140LIZ	Nhsl1	Nhsl1		NHS like 1	no | no chan	0	ENOG410X
B7ZNG0	KIF7_MOU	Kif7	Kif7	16576	Kinesin-lik	no | no chan	0	COG5059(c
Q9D0N8	TMM88_M	Tmem88	Tmem88	67020	Transmemb	no | no chan	0	ENOG4112
Q9D903	EBP2_MOU	Ebna1bp2	Ebna1bp2	69072	Probable rF	no | no chan	0	ENOG410Y
Q3THV0	Q3THV0_M	Bop1	Bop1	12181	Ribosome b	no | no chan	0	ENOG410X
B6DQM2	B6DQM2_M	Abhd15	Abhd15	67477	Alpha/beta	no | down	0	COG0429(A
Q9D0N7	CAF1B_MC	Chaf1b	Chaf1b	110749	Chromatin	no | no chan	0	ENOG410X
A2BE45	A2BE45_M	Srpx	Srpx		Sushi-repea	no | down	0	ENOG410X
Q810V0	MPP10_MC	Mphosph10	Mphosph10	67973	U3 small nu	no | no chan	0	COG5384(u
Q3TMP1	Q3TMP1_M	Gtf3c3	Gtf3c3	98488	General tra	no | no chan	0	ENOG410X
Q542V3	Q542V3_M	Srsf4	Srsf4	57317	Serine and	no | no chan	0	COG0724(T
Q9D1I6	RM14_MO	Mrpl14	Mrpl14	68463	Large ribos	no | no chan	0	ENOG4111
Q3U308	CTU2_MO	Ctu2	Ctu2	66965	Cytoplasmi	no | no chan	0	ENOG410Y
Q9D1I5	MCEE_MC	Mcee	Mcee	73724	Methylmalc	no | no chan	0	COG0346(M
F6XX07	F6XX07_M	Gm21970	Gm21970		Predicted g	no | no chan	0	ENOG410Y

Q9JMH6	TRXR1_M(	Txnrd1	Txnrd1	50493	Thioredoxi	no	no chan	0	COG1249(Z
P49443	PPM1A_M(	Ppm1a	Ppm1a	19042	Protein phc	no	no chan	0	COG0631(e
P49442	INPP_MOU	Inpp1	Inpp1	16329	Inositol pol	no	up	0	ENOG410X
Q148R8	Q148R8_M	Ints2	Ints2	70422	Integrator c	no	no chan	0	ENOG410X
Q540D7	Q540D7_M	Akr1a1	Akr1a1	58810	Aldo-keto r	no	no chan	0	COG0656(p
E9PVX6	KI67_MOU	Mki67	Mki67	17345	Proliferatio	no	no chan	0	ENOG410Z
P81117	NUCB2_M(	Nucb2	Nucb2	53322	Nucleobinc	no	no chan	0	ENOG410X
Q9WVH4	FOXO3_M	Foxo3	Foxo3	56484	Forkhead b	no	no chan	0	COG5025(M
G5E881	G5E881_M	Tamm41	Tamm41	68971	Phosphatid	no	no chan	0	ENOG410Z
Q4VA29	Q4VA29_M	Rtraf	Rtraf	68045	RNA transc	no	no chan	0	ENOG410X
Q3TH57	Q3TH57_M	Tcerg1	Tcerg1	56070	Transcripti	no	no chan	0	ENOG410X
G5E884	G5E884_M	Pak1	Pak1	18479	non-specifi	no	up	0	ENOG410X
A2AEM2	A2AEM2_M	Clcc1	Clcc1	229725	Chloride ch	no	no chan	0	ENOG411C
Q20BD0	Q20BD0_M	Hnrnpab	Hnrnpab	15384	Heterogene	no	no chan	0	COG0724(A
Q8BKX6	SMG1_MO	Smg1	Smg1	233789	Serine/thre	no	no chan	0	COG5032(B
Q3V268	Q3V268_M	Kat8	Kat8	67773	Histone ace	no	no chan	0	COG5027(i
Q8BKX1	BAIP2_MC	Baiap2	Baiap2	108100	BAR/IMD	no	no chan	0	ENOG4111
Q91Z22	PORIM_M(	Tmem123	Tmem123	71929	Porimin	no	no chan	0	ENOG410Z
Q3TIP8	Q3TIP8_M	Clic1	Clic1	114584	Chloride in	no	no chan	0	ENOG410Z
A0A1B0GR	A0A1B0GR	Cyth2	Cyth2		Cytohesin-2	no	up	0	COG5307(M
P0C8K7	SMIM1_M(	Smim1	Smim1	68859	Small integ	no	down	0	ENOG410Y
Q99J09	MEP50_M(	Wdr77	Wdr77	70465	Methyloson	no	no chan	0	ENOG4111
Q99J08	S14L2_MO	Sec14l2	Sec14l2	67815	SEC14-like	no	down	0	ENOG410X
Q9D7N9	APMAP_M	Apmap	Apmap	71881	Adipocyte p	no	no chan	0	COG3386(S
Q9CPW2	FDX2_MO	Fdx2	Fdx2	68165	Ferredoxin-	no	no chan	0	COG0633(F
Q920A5	RISC_MOU	Scpep1	Scpep1	74617	Retinoid-in	no	no chan	0	COG2939(c
Q920A7	AFG31_M(	Afg3l1	Afg3l1	114896	Mitochondi	no	no chan	0	COG0465(A
Q3UD69	Q3UD69_M	Kat2a	Kat2a	14534	Histone ace	no	no chan	0	COG5076(c
H3BK24	H3BK24_M	Cux1	Cux1	13047	Homeobox	no	no chan	0	ENOG410X
Q920D3	MED28_M(	Med28	Med28	66999	Mediator of	no	no chan	0	ENOG4111
Q9JJ89	CCD86_M(	Ccdc86	Ccdc86	108673	Coiled-coil	no	no chan	0	ENOG4112
P05784	K1C18_MC	Krt18	Krt18	16668	Keratin, typ	no	no chan	0	ENOG4111
Q9CSN5	Q9CSN5_M	Ppil4	Ppil4	67418	Peptidyl-pr	no	no chan	0	COG0652(I
A0A1C7CY	A0A1C7CY	Fhip1b	Fhip1b	74349	FHF compl	no	no chan	0	ENOG410X
Q8K2J0	PLCD3_M(	Plcd3	Plcd3	72469	1-phosphat	no	no chan	0	ENOG410X
Q9D945	LLPH_MO	Llph	Llph	66225	Protein LLI	no	no chan	0	ENOG4111
Q3TZ84	Q3TZ84_M	Epn3	Epn3	71889	ENTH dom	no	no chan	0	ENOG410X
Q8BMF4	ODP2_MO	Dlat	Dlat	235339	Dihydrolip	no	no chan	0	COG0508(A
D3Z2D8	D3Z2D8_M	Tmem63b	Tmem63b		Transmeml	no	no chan	0	COG5594(t
Q8R0Z5	MFRN2_M	Slc25a28	Slc25a28	246696	Mitoferrin-	no	no chan	0	ENOG410X
H3BJJ0	H3BJJ0_M(	Tspan6	Tspan6		Tetraspanii	no	down	0	ENOG4111
Q91YE7	RBM5_MO	Rbm5	Rbm5	83486	RNA-bindi	no	no chan	0	ENOG410Y
P61514	RL37A_MC	Rpl37a	Rpl37a	19981	Large ribos	no	no chan	0	COG1997(F

Q91YE3	EGLN1_M	Egln1	Egln1	112405	Egl nine ho	no|no chan	0	ENOG410Z
A6H6S8	A6H6S8_M	Bag4	Bag4	67384	BCL2-assoc	no|no chan	0	ENOG4111
A0A087WP	A0A087WP	Glb1l	Glb1l		Galactosid	no|no chan	0	COG1874(k
B2RXP1	B2RXP1_M	Lrch3	Lrch3	70144	Leucine-ric	no|no chan	0	COG4886(I
P56371	RAB4A_M	Rab4a	Rab4a	19341	Ras-related	no|no chan	0	ENOG410X
A0A9R1SP2	A0A9R1SP2	Nptxr	Nptxr	73340	Neuronal p	yes|down	1	ENOG410X
O35280	CHK1_MO	Chek1	Chek1	12649	Serine/thre	no|no chan	0	ENOG410X
Q80XB4	NRAP_MO	Nrap	Nrap	18175	Nebulin-rel	no|up	0	ENOG4111
O35286	DHX15_M	Dhx15	Dhx15	13204	ATP-depen	no|no chan	0	COG1643(S
O55023	IMPA1_M	Impa1	Impa1	55980	Inositol mo	no|no chan	0	COG0483(t
O55022	PGRC1_M	Pgrmc1	Pgrmc1	53328	Membrane-	no|no chan	0	ENOG4111
Q3TN06	Q3TN06_M	Fez2	Fez2	225020	Fasciculati	no|no chan	0	ENOG410Z
Q99KA4	Q99KA4_M	Igh-VJ558	Igh-VJ558	16061	Igh protein	no|no chan	0	ENOG410Z
O55026	ENTP2_M	Entpd2	Entpd2	12496	Ectonucleo	no|down	0	COG5371(t
Q9JKF1	IQGA1_M	Iqgap1	Iqgap1	29875	Ras GTPas	no|no chan	0	COG5261(C
Q9CZX7	PP4P2_MO	Pip4p2	Pip4p2	72519	Type 2 pho	no|no chan	0	ENOG410X
O55029	COPB2_M	Copb2	Copb2	50797	Coatomer s	no|no chan	0	ENOG410X
Q6PFB2	Q6PFB2_M	Rcc1	Rcc1	100088	Regulator c	no|no chan	0	COG5184(I
Q640N1	AEBP1_M	Aebp1	Aebp1	11568	Adipocyte e	no|no chan	0	ENOG410X
Q640N2	AR13B_M	Arl13b	Arl13b	68146	ADP-ribosy	no|no chan	0	ENOG410X
Q06890	CLUS_MO	Clu	Clu	12759	Clusterin	no|up	0	ENOG410Y
Q9ESJ4	SPN90_M	Nckipsd	Nckipsd	80987	NCK-interc	no|no chan	0	ENOG410Y
Q5U3K5	RABL6_M	Rabl6	Rabl6	227624	Rab-like pr	no|no chan	0	ENOG410X
A0A1W2P6	A0A1W2P6	Fmnl1	Fmnl1		Formin-like	no|no chan	0	ENOG410Y
Q8CE50	SNX30_M	Snx30	Snx30	209131	Sorting nex	no|no chan	0	COG5391(c
O35130	NEP1_MO	Emg1	Emg1	14791	Ribosomal	no|no chan	0	COG1756(M
O54998	FKBP7_M	Fkbp7	Fkbp7	14231	Peptidyl-pr	no|no chan	0	COG0545(A
P48678	LMNA_MO	Lmna	Lmna	16905	Prelamin-A	no|no chan	0	ENOG410Y
O35134	RPA1_MO	Polr1a	Polr1a	20019	DNA-direc	no|no chan	0	COG0086(I
A0A1W2P6	A0A1W2P6	Baz2a	Baz2a		Bromodom	no|no chan	0	COG5076(I
F6ZQA3	F6ZQA3_M	Numa1	Numa1		Nuclear mi	no|no chan	0	ENOG4112
Q8BH43	WASF2_M	Wasf2	Wasf2	242687	Actin-bindi	no|no chan	0	ENOG410Y
Q8BH44	COR2B_M	Coro2b	Coro2b	235431	Coronin-2B	no|no chan	0	ENOG410X
P22599	A1AT2_M	Serpina1b	Serpina1b	20701	Alpha-1-an	no|no chan	0	COG4826(r
Q8BH48	UBAP1_M	Ubap1	Ubap1	67123	Ubiquitin-c	no|no chan	0	ENOG410Z
Q69ZB8	ZCHC2_M	Zcchc2	Zcchc2	227449	Zinc finger	no|no chan	0	ENOG4111
P34022	RANG_MO	Ranbp1	Ranbp1	19385	Ran-specifi	no|no chan	0	COG5171(r
Q3TEW0	Q3TEW0_M	Bnip3l	Bnip3l	12177	BCL2/aden	no|up	0	ENOG4111
A2AM95	A2AM95_M	Emd	Emd	13726	Emerin	no|no chan	0	ENOG410Z
Q8BTQ1	Q8BTQ1_M	Elavl1	Elavl1	15568	ELAV-like	no|up	0	ENOG410X
D3Z4V1	D3Z4V1_M	Plrg1	Plrg1		Pleiotropic	no|no chan	0	ENOG410X
Q99LM9	TADA1_M	Tada1	Tada1	27878	Transcripti	no|no chan	0	ENOG4111
Q03350	TSP2_MOU	Thbs2	Thbs2	21826	Thrombosp	no|no chan	0	ENOG410X

P10417	BCL2_MOU	Bcl2	Bcl2	12043	Apoptosis r	no|no chan	0	ENOG4112
A0A087WS	A0A087WS	Trip12	Trip12		E3 ubiquiti	no|no chan	0	COG5021(I
Q8C894	Q8C894_M	Lipt1	Lipt1	623661	BPL/LPL c	no|no chan	0	COG0095(I
S4R1D4	S4R1D4_M	Ppfia1	Ppfia1		Protein tyro	no|no chan	0	ENOG410X
A0A0N5E9	A0A0N5E9	Rfc1	Rfc1	19687	Replication	no|no chan	0	COG5275(r
Q5NC05	TTF2_MOU	Ttf2	Ttf2	74044	Transcripti	no|no chan	0	COG0553(r
P35991	BTK_MOU	Btk	Btk	12229	Tyrosine-pr	no|no chan	0	COG0515(I
Q8BMI4	GEN_MOU	Gen1	Gen1	209334	Flap endon	no|no chan	0	COG0258(S
Q543Y8	Q543Y8_M	Tmem115	Tmem115	56395	Transmemb	no|no chan	0	ENOG410X
Q00558	HAP40_MO	F8a1	F8a1	14070	40-kDa hun	yes|up	1	ENOG410X
Q78T54	VMA21_MO	Vma21	Vma21	67048	Vacuolar A	no|no chan	0	ENOG4112
E9PX89	E9PX89_M	Bola3	Bola3		BolA family	no|no chan	0	COG0271(I
Q8CGN5	PLIN1_MO	Plin1	Plin1	103968	Perilipin-1	no|down	0	ENOG4111
A0A140LIC	A0A140LIC	Slc35a2	Slc35a2		Solute carri	no|no chan	0	COG0697(r
A0A494B9J	A0A494B9J	Ankrd2	Ankrd2	56642	Ankyrin rep	no|up	0	COG0666(I
Q8BUK6	HOOK3_M	Hook3	Hook3	320191	Protein Hoo	no|no chan	0	ENOG410X
Q3TGY1	Q3TGY1_M	Rsrc1	Rsrc1	66880	Arginine/se	no|no chan	0	COG1358(s
G3UZ44	G3UZ44_M	Prrx1	Prrx1	18933	Paired relat	no|no chan	0	ENOG410Y
D3YXS1	D3YXS1_M	Polr3gl	Polr3gl	69870	Polymerase	no|no chan	0	ENOG4112
Q9EP97	SENP3_MO	Senp3	Senp3	80886	Sentrin-spe	no|no chan	0	COG5160(N
E9Q5N9	E9Q5N9_M	Zfp395	Zfp395	380912	Zinc finger	no|no chan	0	ENOG4111
Q5SUA5	MYO1G_M	Myo1g	Myo1g	246177	Unconventi	no|no chan	0	COG5022(I
O88342	WDR1_MO	Wdr1	Wdr1	22388	WD repeat-	no|no chan	0	ENOG410X
F6R8A9	F6R8A9_M	Mfsd8	Mfsd8		Major facili	no|no chan	0	ENOG410X
Q9Z0Y1	DCTN3_M	Dctn3	Dctn3	53598	Dynactin su	no|no chan	0	ENOG410Y
A2AH85	A2AH85_M	Eftud2	Eftud2	20624	116 kDa U5	no|no chan	0	COG0480(p
Q9QYF1	RDH11_MO	Rdh11	Rdh11	17252	Retinol deh	no|no chan	0	COG1028(c
Q8BSU7	MOB3A_M	Mob3a	Mob3a	208228	MOB kinas	no|down	0	ENOG410X
P53762	ARNT_MO	Arnt	Arnt	11863	Aryl hydroc	no|no chan	0	ENOG410X
Q9JHW4	SELB_MOU	Eefsec	Eefsec	65967	Selenocyste	no|no chan	0	COG3276(S
Q9JHW2	NIT2_MOU	Nit2	Nit2	52633	Omega-ami	no|no chan	0	COG0388(r
Q3UIG2	Q3UIG2_M	Pex3	Pex3	56535	Peroxisoma	no|no chan	0	ENOG4110
A0A1L1SQ	A0A1L1SQ	Dock6	Dock6	319899	Dedicator c	no|no chan	0	ENOG410X
Q9DCC4	P5CR3_MO	Pycr3	Pycr3	66194	Pyrroline-5-	no|no chan	0	COG0345(S
D3YX85	D3YX85_M	Asap2	Asap2	211914	ArfGAP wi	no|no chan	0	COG5347(Y
B1AXZ0	B1AXZ0_M	Elavl2	Elavl2		ELAV-like	no|no chan	0	ENOG410X
A0A0G2JE	A0A0G2JE	Igkv12-38	Igkv12-38	620050	Immunoglo	no|down	0	ENOG410Y
P27641	XRCC5_MO	Xrcc5	Xrcc5	22596	X-ray repai	no|no chan	0	ENOG410Y
B2RWS7	B2RWS7_M	Zfp609	Zfp609	214812	Zinc finger	no|no chan	0	ENOG410X
D3Z6H6	D3Z6H6_M	Tmem219	Tmem219		Transmemb	no|down	0	ENOG4110
D3Z6H3	D3Z6H3_M	Dctn6	Dctn6	22428	Dynactin su	no|no chan	0	ENOG410Y
Q3U8W9	Q3U8W9_M	Hnrnpr	Hnrnpr	74326	RRM doma	no|no chan	0	ENOG410X
A0A2C9F2I	A0A2C9F2I	Anxa7	Anxa7		Annexin	no|up	0	ENOG410X

Q9QY30	ABCBB_M(	Abcb11	Abcb11	27413	Bile salt ex	no	no chan	0	COG1132(t
A0A0G2JG	A0A0G2JG	Elp6	Elp6		Elongator c	no	up	0	ENOG4111
E9Q4T4	E9Q4T4_M	Ccdc71l	Ccdc71l	72123	Coiled-coil	no	no chan	0	ENOG4111
P0DN89	TM254_MC	Tmem254	Tmem254	66039	Transmemb	no	no chan	0	ENOG4111
D3YZ20	D3YZ20_M	Polr2j	Polr2j		Polymerase	no	no chan	0	COG1761(I
Q9DBY8	NVL_MOU	Nvl	Nvl	67459	Nuclear val	no	no chan	0	COG0464(I
P49290	PERE_MO	Epx	Epx	13861	Eosinophil	no	down	0	ENOG410X
E9Q9H0	E9Q9H0_M	Dlg1	Dlg1	13383	Disks large	no	no chan	0	COG0194(I
A0A1L1SSH	A0A1L1SSH	Sparc	Sparc		SPARC	no	no chan	0	ENOG411(
A0A0A6YW	A0A0A6YW	Dclk2	Dclk2		non-specifi	no	no chan	0	ENOG410Y
A2AUK8	A2AUK8_M	Epb41l1	Epb41l1	13821	Erythrocyte	no	no chan	0	ENOG410Y
A0A0A6YW	A0A0A6YW	Ighv1-11	Ighv1-11		Immunoglo	no	up	0	ENOG4111
Q9WTU0	PHF2_MOI	Phf2	Phf2	18676	Lysine-spec	no	no chan	0	ENOG410Y
Q9EPN1	NBEA_MC	Nbea	Nbea	26422	Neurobeacl	no	no chan	0	ENOG410X
O08688	CAN5_MO	Capn5	Capn5	12337	Calpain-5	no	no chan	0	ENOG410X
A2AFY7	A2AFY7_M	Ttc17	Ttc17	74569	Tetratricop	no	no chan	0	ENOG410X
Q8BWQ6	VP35L_MC	Vps35l	Vps35l	71517	VPS35 endc	no	no chan	0	ENOG410X
D6RDQ7	D6RDQ7_I	Btbd10	Btbd10		BTB domai	no	up	0	ENOG410X
Q8BWQ4	CMTR2_M	Cmtr2	Cmtr2	234728	Cap-specifi	no	no chan	0	ENOG410X
A0A494BB7	A0A494BB7	Wdr26	Wdr26		WD repeat-	no	no chan	0	ENOG410X
B1ARU4	B1ARU4_M	Macf1	Macf1	11426	Microtubul	no	no chan	0	COG5069(I
A0A3Q4EC	A0A3Q4EC	Dsg3	Dsg3	13512	Desmoglein	no	up	0	ENOG410X
D3YTT6	D3YTT6_M	Osbpl3	Osbpl3	71720	Oxysterol-b	no	no chan	0	ENOG410X
Q8CIK8	RFWD3_M	Rfwd3	Rfwd3	234736	E3 ubiquiti	no	no chan	0	ENOG410X
D3YTT9	D3YTT9_M	Zzz3	Zzz3	108946	Zinc finger,	no	no chan	0	ENOG410Z
P06800	PTPRC_M(	Ptprc	Ptprc	19264	Receptor-ty	no	no chan	0	COG5599(g
P06801	MAOX_M(	Me1	Me1	17436	NADP-dep	no	no chan	0	COG0281(r
P05064	ALDOA_M	Aldoa	Aldoa	11674	Fructose-bi	no	no chan	0	COG3588(f
Q9CQY1	ATG12_M(	Atg12	Atg12	67526	Ubiquitin-l	no	no chan	0	ENOG4111
A0A0R4J19	A0A0R4J19	Cass4	Cass4	320664	Cas scaffolc	no	no chan	0	ENOG410X
P05063	ALDOC_M	Aldoc	Aldoc	11676	Fructose-bi	no	up	0	COG3588(f
B2RU79	B2RU79_M	Ltbp4	Ltbp4	108075	Latent tran	no	no chan	0	ENOG410Y
Q99P30	NUDT7_M	Nudt7	Nudt7	67528	Peroxisoma	no	no chan	0	COG0494(I
A0A0B4J1E	A0A0B4J1E	Kpna4	Kpna4	16649	Karyopheri	no	no chan	0	COG5064(u
B2KFW6	B2KFW6_M	Phc2	Phc2	54383	Polyhomeo	no	no chan	0	ENOG4111
Q9CQH8	RPP14_MC	Rpp14	Rpp14	67053	Ribonuclea	no	no chan	0	ENOG4112
Q8C1E7	TACAN_M	Tmem120a	Tmem120a	215210	Transmemb	no	no chan	0	ENOG410X
A3KGB4	TBC8B_M(	Tbc1d8b	Tbc1d8b	245638	TBC1 domc	no	no chan	0	COG5210((
A0A0B4J1E	A0A0B4J1E	Snw1	Snw1	66354	SNW doma	no	no chan	0	ENOG410X
A0A3B0J1H	A0A3B0J1H	Adid	Adid	11450	Adiponecti	no	no chan	0	ENOG4111
Q64511	TOP2B_M(	Top2b	Top2b	21974	DNA topoi	no	no chan	0	COG0188(I
Q64514	TPP2_MOI	Tpp2	Tpp2	22019	Tripeptidyl-	no	no chan	0	COG1404(p
A2A559	PGAP3_M(	Pgap3	Pgap3	320655	Post-GPI a	no	no chan	0	COG5237(I

Q3UJ70	Q3UJ70_M(	Hmgcs1	Hmgcs1	208715	Hydroxyme	no | down	0	COG3425(t
Q3UBR8	Q3UBR8_M	Timm17b	Timm17b	21855	Mitochondı	no | up	0	COG5596(t
Q6S9I0	Q6S9I0_M(	Kng2	Kng2	385643	Kininogen :	no | up	0	ENOG4111
Q6PAC3	DCA13_M(	Dcaf13	Dcaf13	223499	DDB1- and	no | no chan	0	ENOG410)
A0A5F8MQ	A0A5F8MQ	Bin2	Bin2		Bridging in	no | up	0	ENOG410Y
O09159	MA2B1_M(	Man2b1	Man2b1	17159	Lysosomal (	no | no chan	0	ENOG410)
Q3U4N2	Q3U4N2_M	Icmt	Icmt	57295	Protein-S-is	no | no chan	0	COG2020(i
A2A9Q2	A2A9Q2_M	Nrdc	Nrdc	230598	Nardilysin (	no | no chan	0	COG1025(r
A2RSL2	A2RSL2_M	Morc4	Morc4	75746	Microrchidi	no | down	0	ENOG411(
Q9JK42	PDK2_MO	Pdk2	Pdk2	18604	[Pyruvate d	no | up	0	COG0642(F
Q9CZ83	RM55_MO	Mrpl55	Mrpl55	67212	Large ribos	no | no chan	0	ENOG4112
A0A1Y7VL	A0A1Y7VL	Pacs2	Pacs2	217893	Phosphofur	no | no chan	0	ENOG410)
Q9CR68	UCRI_MO	Uqcrfs1	Uqcrfs1	66694	Cytochrom(	no | no chan	0	COG0723(C
B2RTB6	B2RTB6_M	Krt86	Krt86	16679	Keratin 86	no | up	0	ENOG4111
Q9CR61	NDUB7_M	Ndufb7	Ndufb7	66916	NADH deh	no | no chan	0	ENOG4112
Q9CR60	GOT1B_M	Golt1b	Golt1b	66964	Vesicle trar	no | up	0	COG5120(g
B2RTB0	B2RTB0_M	Pdap1	Pdap1	231887	PDGFA ass	no | no chan	0	ENOG4111
Q9CZ57	NSUN4_M(	Nsun4	Nsun4	72181	5-cytosine r	no | no chan	0	COG0144(r
Q9Z1F9	SAE2_MOU	Uba2	Uba2	50995	SUMO-acti	no | no chan	0	COG0476(C
O88907	PIAS1_MO	Pias1	Pias1	56469	E3 SUMO-p	no | no chan	0	ENOG410)
F8WIS9	F8WIS9_M(	Camk2a	Camk2a		calcium/ca	no | up	0	ENOG410)
A0A0R4J1Y	A0A0R4J1Y	Osgep	Osgep	66246	N(6)-L-thre	no | no chan	0	COG0533(F
A0A1B0GT	A0A1B0GT	Tmem143	Tmem143	70209	Transmemb	no | no chan	0	ENOG411(
A0A0R4J1Y	A0A0R4J1Y	Sphkap	Sphkap	77629	SPHK1 inte	no | down	0	ENOG4111
A0A0R4J1Y	A0A0R4J1Y	Nfya	Nfya	18044	Nuclear tra	no | no chan	0	COG5224(N
Q3UZA1	CPZIP_MC	Rcsd1	Rcsd1	226594	CapZ-inter(	no | no chan	0	ENOG410Y
B9EI26	B9EI26_M(	Arhgap25	Arhgap25	232201	Rho GTPas	no | no chan	0	ENOG410)
B9EI21	B9EI21_M(	Zmat3	Zmat3	22401	Zinc finger	no | no chan	0	ENOG411(
O70310	NMT1_MC	Nmt1	Nmt1	18107	Glycylpepti	no | no chan	0	COG5092(A
Q3UAP1	Q3UAP1_M	Preb	Preb	50907	Prolactin re	no | no chan	0	ENOG410)
Q8R2U0	SEH1_MOU	Seh1l	Seh1l	72124	Nucleopori	no | no chan	0	ENOG410)
Q8K0D7	GET1_MO	Get1	Get1	71446	Guided ent	no | no chan	0	ENOG4112
Q8R2U4	NTM1A_M	Ntmt1	Ntmt1	66617	N-terminal	no | no chan	0	ENOG410)
Q8R1J9	TOR2A_M(	Tor2a	Tor2a	30933	Torsin-2A	no | no chan	0	ENOG410)
B2RSH2	GNAI1_M(	Gnai1	Gnai1	14677	Guanine nu	no | down	0	ENOG410)
B1AYU7	B1AYU7_M	Cwc22	Cwc22	80744	Pre-mRNA-	no | no chan	0	ENOG410)
Q3URG1	Q3URG1_M	Tppp	Tppp	72948	Tubulin po	no | up	0	ENOG4111
Q91WK1	SPRY4_MC	Spryd4	Spryd4	66701	SPRY domc	no | no chan	0	ENOG4111
H3BIX4	H3BIX4_M	Nptn	Nptn		Neuroplast	no | no chan	0	ENOG4111
Q91WK5	GCSH_MO	Gcsh	Gcsh	68133	Glycine cle(	no | no chan	0	COG0509(T
Q922H9	ZN330_MC	Znf330	Znf330	30932	Zinc finger	no | no chan	0	ENOG410)
A2AW05	A2AW05_M	Ssrp1	Ssrp1		FACT comp	no | no chan	0	COG5165(C
A0A1L1SRI	A0A1L1SRI	Rnf111	Rnf111		RING-type	no | no chan	0	ENOG4112

B9EIE8	B9EIE8_M(	Lyrm7	Lyrm7	75530	Complex II	no | no chan	0	ENOG4112
B9EIE9	B9EIE9_M(	Adss2	Adss2	11566	Adenylosuc	no | no chan	0	COG0104(I
E9Q7Y0	E9Q7Y0_M	Foxm1	Foxm1		Forkhead b	no | no chan	0	COG5025(\
Q8BIW1	PRUN1_M(	Prune1	Prune1	229589	Exopolyph(	no | no chan	0	COG1227(N
P84089	ERH_MOU	Erh	Erh	13877	Enhancer o	yes | up	1	ENOG4111
Q14B71	CDCA2_M(	Cdca2	Cdca2	108912	Cell divisio	no | no chan	0	ENOG410Y
P84086	CPLX2_M(	Cplx2	Cplx2	12890	Complexin-	no | no chan	0	ENOG4111
P84084	ARF5_MO	Arf5	Arf5	11844	ADP-ribosy	no | no chan	0	COG1100(C
A1E2H8	A1E2H8_M	Aif1	Aif1	11629	Allograft in	no | no chan	0	ENOG4112
F8WHS8	F8WHS8_M	Pias2	Pias2	17344	Protein inh	no | no chan	0	ENOG410X
Q3TJ95	Q3TJ95_M(	Cltb	Cltb	74325	Clathrin lig	no | no chan	0	ENOG4111
E9QNZ2	E9QNZ2_M	Dzip3	Dzip3	224170	RING-type	no | no chan	0	ENOG410Y
Q3TPC9	Q3TPC9_M	Zc3h8	Zc3h8	57432	C3H1-type	no | no chan	0	COG5084(C
D3Z6W7	D3Z6W7_M	Gxylt1	Gxylt1	223827	Glucoside x	no | no chan	0	ENOG410X
Q9D8S9	BOLA1_M(	Bola1	Bola1	69168	BolA-like p	no | no chan	0	COG0271(t
Q6ZWQ9	Q6ZWQ9_	Myl12a	Myl12a	67268	Myosin, ligl	no | no chan	0	COG5126(S
Q793L1	Q793L1_M(	Emc9	Emc9	85308	Cgi112	no | up	0	ENOG410Y
G3UWI9	G3UWI9_M	Sumo3	Sumo3	20610	Small ubiq	no | down	0	COG5227(c
Q8VBZ3	CLPT1_MC	Clptm1	Clptm1	56457	Putative lip	no | no chan	0	ENOG410X
Q8VBZ0	DHRSX_M	Dhrsx	Dhrsx	236082	Polyprenol	no | no chan	0	COG1028(I
Q8BVR6	RSPRY_M(	Rspry1	Rspry1	67610	RING finge	no | down	0	ENOG410X
Q3V1M1	IGS10_MO	Igsf10	Igsf10	242050	Immunoglo	no | no chan	0	ENOG410Y
B8JJZ4	B8JJZ4_MC	Zfp808	Zfp808	630579	Zinc finger	no | down	0	COG5048(S
Q80UL3	Q80UL3_M	Galk1	Galk1	14635	galactokina	no | no chan	0	COG0153(C
Q3U6P5	Q3U6P5_M	Hnrnpc	Hnrnpc	15381	RRM doma	no | no chan	0	COG0724(C
Q8BKE9	IFT74_MO	Ift74	Ift74	67694	Intraflagell	no | no chan	0	ENOG410X
Q8BKE6	CP20A_MC	Cyp20a1	Cyp20a1	77951	Cytochrom(	no | no chan	0	COG2124(I
B1AR69	B1AR69_M	Myh13	Myh13	544791	Myosin, hec	no | up	0	COG5022(N
A0A2I3BRI	A0A2I3BRI	Tbc1d23	Tbc1d23		TBC1 domc	no | no chan	0	ENOG410Z
A0A1X9HI	A0A1X9HIM5_MOUSE	IgG light chain variab			IgG light cl	no | no chan	0	ENOG410Y
Q6NXY9	RPC7_MO	Polr3g	Polr3g	67486	DNA-direc	no | no chan	0	ENOG4112
E9QN12	E9QN12_M	Pdgfrb	Pdgfrb		Platelet-der	no | no chan	0	COG0515(S
Q8BP56	PGGHG_M	Pgghg	Pgghg	212974	Protein-glu	no | no chan	0	COG1554(I
F6Q7H5	F6Q7H5_M	Srrd	Srrd		SRR1 doma	yes | down	1	ENOG4111
Q3TAF9	Q3TAF9_M	Bgn	Bgn	12111	Biglycan	no | no chan	0	COG4886(I
Q3TAF8	Q3TAF8_M	Hps4	Hps4	192232	Hermansky	no | no chan	0	ENOG411C
E9QN14	E9QN14_M	Srgap3	Srgap3	259302	SLIT-ROBC	no | no chan	0	ENOG410X
Q3TR90	Q3TR90_M	Hdgfl3	Hdgfl3	29877	PWWP don	no | no chan	0	ENOG411C
B7FAU9	B7FAU9_M	Flna	Flna	192176	Filamin, alp	no | no chan	0	COG5069(c
O70172	PI42A_MO	Pip4k2a	Pip4k2a	18718	Phosphatid	no | no chan	0	COG5253(N
E9Q1U6	E9Q1U6_M	Ctif	Ctif	269037	CBP80/20-c	no | no chan	0	ENOG410X
P70280	VAMP7_M(	Vamp7	Vamp7	20955	Vesicle-ass(	no | no chan	0	COG5143(s
Q9DD02	HIKES_MC	Hikeshi	Hikeshi	67669	Protein Hik	no | no chan	0	ENOG4111

Q8C9X6	EPC1_MOI	Epc1	Epc1	13831	Enhancer o	no|no chan	0	ENOG410)
Q07133	H1T_MOU	H1-6	H1-6	107970	Histone H1	yes|up	1	ENOG4112
Q9CZN8	GATA_MC	Qrsl1	Qrsl1	76563	Glutamyl-t	no|no chan	0	COG0154(/
Q9CZN7	GLYM_MC	Shmt2	Shmt2	108037	Serine hydr	no|no chan	0	COG0112((
Q544F6	Q544F6_M	Cotl1	Cotl1	72042	Cotl1 prote	no|no chan	0	ENOG4112
P56726	SMO_MOU	Smo	Smo	319757	Protein smo	no|down	0	ENOG410)
Q9D0C4	TRM5_MO	Trmt5	Trmt5	76357	tRNA (guai	no|no chan	0	COG2520(S
P57784	RU2A_MO	Snrpa1	Snrpa1	68981	U2 small nu	no|no chan	0	COG4886(e
P57787	MOT4_MC	Slc16a3	Slc16a3	80879	Monocarbo	no|up	0	ENOG4111
Q8C7J6	Q8C7J6_M	Kctd12b	Kctd12b	207474	Potassium c	no|no chan	0	ENOG410Z
Q8BPX9	S15A3_MO	Slc15a3	Slc15a3	65221	Solute carri	no|up	0	COG3104(z
Q8VDP4	CCAR2_M(	Ccar2	Ccar2	219158	Cell cycle a	no|no chan	0	ENOG4111
A0AA74KU	A0AA74KU	Pogz	Pogz		Pogo transp	no|no chan	0	COG5048(Z
Q8VDP2	STEEP_M(	Steep1	Steep1	77644	STING ER	no|no chan	0	ENOG410)
P51175	PPOX_MO	Ppox	Ppox	19044	Protoporph	no|no chan	0	COG1232(p
Q61033	LAP2A_M(	Tmpo	Tmpo	21917	Lamina-ass	no|no chan	0	ENOG4111
Q3UGF1	WDR19_M	Wdr19	Wdr19	213081	WD repeat-	no|no chan	0	ENOG410)
Q3T9L0	Q3T9L0_M	Ddx39a	Ddx39a	68278	RNA helico	no|no chan	0	COG0513(s
B2RUA1	B2RUA1_M	Pnisr	Pnisr	66625	Splicing fac	no|no chan	0	ENOG4111
B1AT82	B1AT82_M	Prpsap1	Prpsap1	67763	Phosphorib	no|no chan	0	COG0462(U
P32507	NECT2_M(	Nectin2	Nectin2	19294	Nectin-2	no|no chan	0	ENOG4111
B2RUA5	B2RUA5_M	Ppp6r1	Ppp6r1	243819	SAPS doma	no|no chan	0	ENOG410)
Q4GX45	Q4GX45_M	Paox	Paox	212503	Peroxisoma	no|no chan	0	ENOG410)
Q64695	EPCR_MO	Procr	Procr	19124	Endothelial	no|up	0	ENOG4111
A0A0N4SV	A0A0N4SV	Tmem176a	Tmem176a		Transmemb	no|down	0	ENOG410Z
O08856	ELL_MOU	Ell	Ell	13716	RNA polym	no|no chan	0	ENOG410Z
B1AT89	B1AT89_M	Sphk1	Sphk1		Sphingosin	no|no chan	0	COG1597(c
Q99KZ6	ZN639_MC	Znf639	Znf639	67778	Zinc finger	no|no chan	0	COG5048(F
Q9D7I5	LHPP_MO	Lhpp	Lhpp	76429	Phospholys	no|no chan	0	COG0647(c
P31310	HXA10_M(	Hoxa10	Hoxa10	15395	Homeobox	no|no chan	0	ENOG4111
Q05D01	Q05D01_M	Topors	Topors	106021	RING-type	no|down	0	ENOG410)
Q9D7I8	FA83D_M(	Fam83d	Fam83d	71878	Protein FAI	no|no chan	0	ENOG410Z
Q3V0Z8	Q3V0Z8_M	Ddx5	Ddx5	13207	Probable A	no|no chan	0	COG0513(c
A0A1B0GS	A0A1B0GS	Grtp1	Grtp1		GH regulat	no|no chan	0	COG5210(r
Q9ES74	NEK7_MO	Nek7	Nek7	59125	Serine/thre	no|no chan	0	ENOG410)
Q9ES70	NEK6_MO	Nek6	Nek6	59126	Serine/thre	no|no chan	0	ENOG410)
Q3UU56	Q3UU56_M	Gm29609	Gm29609	71752	Predicted g	no|no chan	0	ENOG410Y
Q921X9	PDIA5_MC	Pdia5	Pdia5	72599	Protein dis	no|no chan	0	COG0526(N
A0A1Z3MI	A0A1Z3MI	Ifi205	Ifi205	226695	Ifi205	no|up	0	ENOG410Y
E9Q6U4	E9Q6U4_M	Pfdn4	Pfdn4	109054	Prefoldin su	no|no chan	0	COG1382(N
A0A0D3M(	A0A0D3M(	Obp1a	Obp1a	18249	Odorant bi	no|no chan	0	ENOG4111
Q99LJ0	CT2NL_M(	Cttnbp2nl	Cttnbp2nl	80281	CTTNBP2 I	no|no chan	0	ENOG410Y
Q6NXL6	SENP5_M(	Senp5	Senp5	320213	Sentrin-spe	no|no chan	0	COG5160(U

Q8BY46	ZN574_MC	Znf574	Znf574	232976	Zinc finger	no|no chan	0	COG5048(C
D3Z497	D3Z497_M	Rnf7l	Rnf7l		cullin-RIN(	no|no chan	0	COG5194(p
S4R197	S4R197_MC	Pde10a	Pde10a		Phosphodie	no|no chan	0	ENOG410X
Q9QUN9	DKK3_MC	Dkk3	Dkk3	50781	Dickkopf-re	no|down	0	ENOG410X
Q99LJ8	NGBR_MC	Nus1	Nus1	52014	Dehydrodol	no|up	0	COG0020(C
Q7M759	AB17B_MC	Abhd17b	Abhd17b	226016	Alpha/beta	no|no chan	0	COG1073(I
A0A338P7E	A0A338P7E	Dynlt1a	Dynlt1a		Dynein ligh	no|no chan	0	ENOG4111
Q3UJZ3	ARMC7_M	Armc7	Armc7	276905	Armadillo r	no|no chan	0	ENOG4111
E9QKK1	E9QKK1_M	Cenpe	Cenpe	229841	Centromere	no|no chan	0	COG5059(r
Q3V1H1	CKAP2_MC	Ckap2	Ckap2	80986	Cytoskeleto	no|no chan	0	ENOG4111
A0A1D5RL	A0A1D5RL	Efr3a	Efr3a		EFR3 homo	no|no chan	0	ENOG410Y
E9QKK8	E9QKK8_M	Atp11c	Atp11c	320940	Phospholip	no|no chan	0	COG0474(f
A0A286YD	A0A286YD	Myof	Myof	226101	Myoferlin	no|no chan	0	ENOG410X
E9QQ10	E9QQ10_M	Akap9	Akap9	100986	A kinase an	no|no chan	0	ENOG411C
C0LQ87	C0LQ87_M	Sync	Sync	68828	Syncoilin is	no|up	0	ENOG410Y
Q9D0L4	ADCK1_M	Adck1	Adck1	72113	AarF doma	no|no chan	0	COG0661(C
B1Q450	B1Q450_M	HBB1	HBB1	15129	Hemoglobi	no|up	0	COG1018(I
Q3U0I9	Q3U0I9_M	Snx8	Snx8	231834	Sorting nex	no|no chan	0	COG5391(i
Q8C547	HTR5B_MC	Heatr5b	Heatr5b	320473	HEAT repe	no|no chan	0	ENOG410X
Q9D0L8	MCES_MO	Rnmt	Rnmt	67897	mRNA cap	no|no chan	0	ENOG410Y
Q8C7E9	CSTFT_MC	Cstf2t	Cstf2t	83410	Cleavage st	no|no chan	0	ENOG410X
Q8C8G3	Q8C8G3_M	Tmem135	Tmem135	72759	Transmemb	no|no chan	0	ENOG410Y
E0CY45	E0CY45_M	Runx2	Runx2		Runt-relate	no|no chan	0	ENOG4111
Q5SV85	SYNRG_M	Synrg	Synrg	217030	Synergin ga	no|no chan	0	ENOG410X
Q0VGU0	Q0VGU0_M	Sar1b	Sar1b	66397	Small COP	no|no chan	0	ENOG410Y
Q8K394	PLCL2_MC	Plcl2	Plcl2	224860	Inactive ph	no|no chan	0	ENOG410X
A2VCP3	A2VCP3_M	P2rx7	P2rx7	18439	P2X purino	no|down	0	ENOG411C
Q8K396	MND1_MC	Mnd1	Mnd1	76915	Meiotic nuc	no|no chan	0	COG5124(r
Q8K397	Q8K397_M	Cst6	Cst6	73720	Cst6 protein	no|no chan	0	ENOG410Y
Q6ZWM4	LSM8_MO	Lsm8	Lsm8	76522	U6 snRNA-	no|no chan	0	ENOG4112
A3KGF7	PLCB2_MC	Plcb2	Plcb2	18796	1-phosphat	no|up	0	ENOG410X
A0A1Y7VL	A0A1Y7VL	Prxl2c	Prxl2c		Peroxiredox	no|no chan	0	ENOG4111
A1L2Z3	A1L2Z3_M	Emc1	Emc1	230866	ER membra	no|no chan	0	ENOG410X
Q3TZ45	Q3TZ45_M	Acss1	Acss1	68738	Acetyl-coen	no|no chan	0	COG0365(C
A0A8Z1S2C	A0A8Z1S2C	Clk3	Clk3	102414	dual-specifi	no|no chan	0	ENOG410X
Q8CFJ9	WDR24_M	Wdr24	Wdr24	268933	GATOR2 c	no|no chan	0	ENOG410X
E9Q7M2	T22D2_MC	Tsc22d2	Tsc22d2	72033	TSC22 dom	no|no chan	0	ENOG410Y
Q4PJX1	ODR4_MC	Odr4	Odr4	226499	Protein odr	no|no chan	0	ENOG410X
Q91W98	S15A4_MO	Slc15a4	Slc15a4	100561	Solute carri	no|no chan	0	COG3104(t
Q3U5Q7	CMPK2_M	Cmpk2	Cmpk2	22169	UMP-CMP	no|up	0	COG0125(s
A0A172Q3	A0A172Q3	Cd248	Cd248	70445	Endosialin	no|no chan	0	ENOG410Z
Q9D384	U1SBP_MC	Snrnp35	Snrnp35	76167	U11/U12 sr	no|no chan	0	COG0724(I
Q9CXF7	CHD1L_M	Chd1l	Chd1l	68058	Chromodor	no|no chan	0	COG0553(C

A1IGU4	ARH37_MC	Arhgef37	Arhgef37	328967	Rho guanin	no	no chan	0	ENOG410Y
A2A4A6	A2A4A6_M	Plcg1	Plcg1	18803	1-phosphat	no	no chan	0	ENOG410X
Q4KL39	Q4KL39_M	Dusp12	Dusp12	80915	protein-tyrc	no	no chan	0	COG2453(I
Q3UPD0	Q3UPD0_M	Scfd2	Scfd2	212986	Sec1 family	no	no chan	0	ENOG411C
E9Q2I1	E9Q2I1_MC	Clcn3	Clcn3		Chloride ch	no	no chan	0	COG0038(i
E9PY58	E9PY58_M	Clpb	Clpb		ClpB caseir	no	no chan	0	COG0542(A
Q148P2	Q148P2_MC	Oxsm	Oxsm	71147	3-oxoacyl-[(	no	no chan	0	COG0304(C
Q9D1F4	AKTS1_MC	Akt1s1	Akt1s1	67605	Proline-rich	no	no chan	0	ENOG4112
P26011	ITB7_MOU	Itgb7	Itgb7	16421	Integrin bet	no	no chan	0	ENOG410X
Q0QEZ4	Q0QEZ4_M	Sdhb	Sdhb	67680	Succinate d	no	no chan	0	COG0479(s
A0A571BD	A0A571BD	Aak1	Aak1		AP2 associc	no	up	0	ENOG410Y
Q9D1F0	Q9D1F0_M	Rtl8b	Rtl8b	66158; 5531	Protein LD	no	no chan	0	ENOG4111
Q80YR5	SAFB2_MC	Safb2	Safb2	224902	Scaffold att	no	no chan	0	ENOG4111
P61358	RL27_MOU	Rpl27	Rpl27	19942	Large ribos	no	no chan	0	COG2163(A
Q9DBF1	AL7A1_MC	Aldh7a1	Aldh7a1	110695	Alpha-amir	no	no chan	0	COG1012(c
H3BK01	H3BK01_M	Taf6l	Taf6l	225895	TATA-box	no	no chan	0	COG5095(C
Q9DBF7	CWC25_MC	Cwc25	Cwc25	67480	Pre-mRNA	no	no chan	0	ENOG410X
Q99J27	ACATN_M	Slc33a1	Slc33a1	11416	Acetyl-coen	no	no chan	0	COG0477(I
Q99J25	MRM1_MC	Mrm1	Mrm1	217038	rRNA meth	no	no chan	0	COG0566(I
P42867	GPT_MOU	Dpagt1	Dpagt1	13478	UDP-N-acе	no	no chan	0	COG0472(p
Q8R4Z4	ETV3_MOU	Etv3	Etv3	27049	ETS translc	no	no chan	0	ENOG410Z
Q00915	RET1_MOU	Rbp1	Rbp1	19659	Retinol-bin	no	down	0	ENOG4111
Q3UFY7	5NT3B_MC	Nt5c3b	Nt5c3b	68106	7-methylgu	no	no chan	0	ENOG410Z
Q3UFY8	TM10C_MC	Trmt10c	Trmt10c	52575	tRNA meth	no	no chan	0	ENOG4111
A2ANC6	A2ANC6_M	Ap5s1	Ap5s1	69596	Adaptor-rel	no	no chan	0	ENOG4111
A0A0G2JD	A0A0G2JD	Slc35a3	Slc35a3		UDP-N-acе	no	no chan	0	COG0697(I
Q3LAC4	PREX2_MC	Prex2	Prex2	109294	Phosphatid	no	no chan	0	ENOG410X
Q9CSH3	RRP44_MC	Dis3	Dis3	72662	Exosome cc	no	no chan	0	COG0557(3
Q9CY25	MIS12_MO	Mis12	Mis12	67139	Protein MIS	no	no chan	0	ENOG4111
A0A140T8T	A0A140T8T	Slit2	Slit2	20563	Slit guidanc	no	up	0	COG4886(M
O55222	ILK_MOUS	Ilk	Ilk	16202	Scaffold prc	no	no chan	0	COG0666(T
Q9D4H1	EXOC2_M	Exoc2	Exoc2	66482	Exocyst cor	no	no chan	0	ENOG410X
Q9D4H2	GCC1_MO	Gcc1	Gcc1	74375	GRIP and c	no	no chan	0	ENOG410Y
Q9D968	HCFC2_MC	Hcfc2	Hcfc2	67933	Host cell fa	no	no chan	0	ENOG410Y
O55229	CHKB_MC	Chkb	Chkb	12651	Choline/etl	no	no chan	0	COG0510(T
Q9D964	GATM_MC	Gatm	Gatm	67092	Glycine am	no	no chan	0	ENOG410Y
A2AKH7	A2AKH7_M	Lrrc57	Lrrc57		Leucine ricl	no	no chan	0	COG4886(I
Q9D4H9	PHF14_MC	Phf14	Phf14	75725	PHD finger	no	no chan	0	COG5141(e
A2AKH4	A2AKH4_M	Snap23	Snap23	20619	Synaptosom	no	no chan	0	ENOG410Y
Q99JY8	PLPP3_MC	Plpp3	Plpp3	67916	Phospholip	no	no chan	0	ENOG4111
A0A068BD	A0A068BD	Kank3	Kank3	80880	Putative KN	no	no chan	0	COG0666(I
Q99JY3	GIMA4_MC	Gimap4	Gimap4	107526	GTPase IM	no	no chan	0	ENOG410Y
P01887	B2MG_MC	B2m	B2m	12010	Beta-2-micr	no	up	0	ENOG410Y

Q543C5	Q543C5_M	Ogn	Ogn	18295	Mimecan	no	down	0	COG4886(p
Q99JY4	TRABD_M	Trabd	Trabd	67976	TraB doma	no	no chan	0	COG1916(t
A0A5H1ZR	A0A5H1ZR	Trp53bp1	Trp53bp1	27223	Transforma	no	no chan	0	ENOG411C
A0A5H1ZR	A0A5H1ZR	Nelfb	Nelfb		Negative el	no	no chan	0	ENOG410Y
O35737	HNRH1_M	Hnrnph1	Hnrnph1	59013	Heterogene	no	no chan	0	ENOG410Z
A0A5H1ZR	A0A5H1ZR	Spire1	Spire1	68166	Spire type a	no	no chan	0	ENOG4111
A0A087WR	A0A087WR	2310022A1(	2310022A10Rik		RIKEN cD	no	no chan	0	ENOG4112
Q9CYG7	TOM34_M(	Tomm34	Tomm34	67145	Mitochond	no	no chan	0	COG0457(C
Q8BRM2	GORAB_M	Gorab	Gorab	98376	RAB6-inter	no	no chan	0	ENOG4111
Q3TSX8	Q3TSX8_M	Tomm70a	Tomm70a	28185	Uncharacte	no	no chan	0	ENOG410Y
Q99KG3	RBM10_M(	Rbm10	Rbm10	236732	RNA-bindi	no	no chan	0	ENOG410Y
Q3U1V6	UEVLD_M	Uevld	Uevld	54122	Ubiquitin-c	no	no chan	0	COG0039(C
Q99L88	SNTB1_M(	Sntb1	Sntb1	20649	Beta-1-synt	no	down	0	ENOG410Z
Q99L85	ELP5_MOU	Elp5	Elp5	54351	Elongator c	no	no chan	0	ENOG4112
Q9WVS8	MK07_MO	Mapk7	Mapk7	23939	Mitogen-ac	no	no chan	0	ENOG410Z
Q6PFD6	KI18B_MO	Kif18b	Kif18b	70218	Kinesin-like	no	no chan	0	COG5059(I
E9PUB7	E9PUB7_M	Msto1	Msto1	229524	Protein mis	no	no chan	0	ENOG410Z
A6H687	SAC31_MC	Sac3d1	Sac3d1	66406	SAC3 doma	no	no chan	0	COG5079(r
E9PUB2	E9PUB2_M	Bri3	Bri3	55950	Membrane	no	no chan	0	ENOG4112
A0A1L1SU!	A0A1L1SU!	Cdk17	Cdk17		Cyclin depe	no	no chan	0	ENOG410Z
Q5XJY4	PARL_MO	Parl	Parl	381038	Presenilin-c	no	no chan	0	COG0705(r
Q5XJY7	Q5XJY7_M	Gtf2ird1	Gtf2ird1	57080	General tra	no	no chan	0	ENOG411C
Q99MR9	PPR3A_M(	Ppp1r3a	Ppp1r3a	140491	Protein pho	no	no chan	0	ENOG4111
Q9CRF7	Q9CRF7_M	Srp19	Srp19	66384	Signal reco	no	no chan	0	COG1400(I
P62137	PP1A_MOU	Ppp1ca	Ppp1ca	19045	Serine/thre	no	no chan	0	ENOG410Z
Q8C3J5	DOCK2_M	Dock2	Dock2	94176	Dedicator c	no	no chan	0	ENOG410Z
P70670	NACAM_M	Naca	Naca	17938	Nascent po	no	up	0	COG1308(s
P70671	IRF3_MOU	Irf3	Irf3	54131	Interferon r	no	no chan	0	ENOG410Y
Q3TEU8	Q3TEU8_M	Coro1c	Coro1c	23790	Coronin	no	no chan	0	ENOG410Z
P70677	CASP3_MC	Casp3	Casp3	12367	Caspase-3	no	no chan	0	ENOG410Z
Q99LC9	PEX6_MOU	Pex6	Pex6	224824	Peroxisoma	no	no chan	0	ENOG410Z
Q99LC5	ETFA_MO	Etfa	Etfa	110842	Electron tra	no	no chan	0	COG2025(E
Q99LC2	CSTF1_MC	Cstf1	Cstf1	67337	Cleavage st	no	no chan	0	ENOG410Z
Q99LC3	NDUAA_M	Ndufa10	Ndufa10	67273	NADH deh	no	no chan	0	ENOG411C
A2A9L4	A2A9L4_M	Trim63	Trim63		Tripartite n	no	up	0	ENOG411C
A0A3Q4EI	A0A3Q4EI	BC004004	BC004004		cDNA sequ	no	no chan	0	ENOG410Y
Q3UCW0	Q3UCW0_I	Tsg101	Tsg101	22088	Tumor susc	no	no chan	0	ENOG410Z
A2AQA7	A2AQA7_M	Aqr	Aqr	11834	RNA helica	no	no chan	0	ENOG410Z
A0A0N4SV	A0A0N4SV	Zfp638	Zfp638	18139	Zinc finger	no	no chan	0	ENOG4111
P58774	TPM2_MO	Tpm2	Tpm2	22004	Tropomyos	no	up	0	ENOG410Z
A0A1D5RM	A0A1D5RM	Gas8	Gas8		Dynein reg	no	no chan	0	ENOG410Z
A2AIV2	VIR_MOUS	Virma	Virma	66185	Protein viri	no	no chan	0	ENOG410Z
Q9CZD0	MMAC_M(	Mmachc	Mmachc	67096	Cyanocoba	no	no chan	0	ENOG410Z

A2AIV8	CARD9_M(	Card9	Card9	332579	Caspase rec	no|no chan	0	ENOG410Y
D3YXM0	D3YXM0_M	Sirpb1c	Sirpb1c		Signal-regu	no|up	0	ENOG410Y
Q9DC63	FBX3_MOU	Fbxo3	Fbxo3	57443	F-box only	no|no chan	0	COG2967(s
A0A2U3TZ	A0A2U3TZ	Triobp	Triobp	110253	TRIO and I	no|no chan	0	ENOG410Z
Q8VEK0	CC50A_MC	Tmem30a	Tmem30a	69981	Cell cycle c	no|no chan	0	COG5035(C
P35174	CYT2_MOU	Stfa2	Stfa2	20862	Stefin-2	no|up	0	ENOG4112
F6YRW4	F6YRW4_M	Kdm2a	Kdm2a	225876	Lysine (K)-s	no|no chan	0	ENOG410X
A0A3B2WC	A0A3B2WC	Khsrp	Khsrp		KH-type sp	no|no chan	0	ENOG410X
Q9JJC6	RIPL1_MO	Rilpl1	Rilpl1	75695	RILP-like p	no|no chan	0	ENOG410Z
Q9DCA5	BRX1_MO	Brix1	Brix1	67832	Ribosome b	no|no chan	0	COG5154(r
A0A068BIT	A0A068BIT	Psmb8	Psmb8	16913	Proteasome	no|up	0	ENOG410X
A0A068BIT	A0A068BIT	Slc39a7	Slc39a7	14977	Zinc transp	no|down	0	COG0428(C
B2RRI5	B2RRI5_M	Lrig2	Lrig2	269473	Leucine-ric	no|down	0	COG4886(S
P29594	CASP2_MC	Casp2	Casp2	12366	Caspase-2	no|no chan	0	ENOG410Z
B2RRI2	B2RRI2_M	Sbno1	Sbno1	243272	Sbno1 prote	no|no chan	0	ENOG410X
Q9D1C8	VPS28_MO	Vps28	Vps28	66914	Vacuolar p	no|no chan	0	ENOG4111
A0AAQ4VI	A0AAQ4VMW3_MOUSE LIM domain only 7 (		Lmo7			no|no chan	0	ENOG410Z
A0AAQ4VI	A0AAQ4VMW9_MOUSE Solute carrier family		Gm58354			no|no chan	0	ENOG4111
Q78XF5	OSTC_MO	Ostc	Ostc	66357	Oligosacch	no|no chan	0	ENOG4111
A0A0G2JG	A0A0G2JG	Nexn	Nexn		Nexilin	no|no chan	0	ENOG411C
Q9D6U8	F162A_MO	Fam162a	Fam162a	70186	Protein FA	no|up	0	ENOG4111
Q6DYE8	ENPP3_M(	Enpp3	Enpp3	209558	Ectonucleo	no|down	0	COG1524(t
P28352	APEX1_M(	Apex1	Apex1	11792	DNA repai	no|no chan	0	COG0708(I
D3YZ00	D3YZ00_M	Nfix	Nfix		Nuclear fac	no|no chan	0	ENOG410Y
Q3UNA4	NXT2_MO	Nxt2	Nxt2	237082	NTF2-relat	no|no chan	0	ENOG4111
D3YZ08	D3YZ08_M	Terf2	Terf2	21750	Telomeric r	no|no chan	0	ENOG4111
D3YZ09	D3YZ09_M	Gar1	Gar1		H/ACA rib	no|no chan	0	COG3277(h
Q6NZP2	GPBL1_M(	Gpbp1l1	Gpbp1l1	77110	Vasculin-lil	no|up	0	ENOG411C
E9Q9J4	E9Q9J4_M	Ppip5k2	Ppip5k2		Inositol hex	no|no chan	0	ENOG410X
A2AUM9	CE152_MC	Cep152	Cep152	99100	Centrosome	no|no chan	0	ENOG410X
D3YUN0	D3YUN0_M	Dgcr6	Dgcr6		DiGeorge s	no|up	0	ENOG4111
D3YUN3	D3YUN3_M	Tnni1	Tnni1		Troponin I,	no|up	0	ENOG410Y
A0A0B4J1C	A0A0B4J1C	Fcgr2b	Fcgr2b	14130	Fc receptor	no|no chan	0	ENOG410Y
A0A0B4J1C	A0A0B4J1C	Elf2	Elf2		E74-like fac	no|no chan	0	ENOG4111
P54726	RD23A_M(	Rad23a	Rad23a	19358	UV excisior	no|no chan	0	COG5272(s
Q5SS80	DHR13_M(	Dhrs13	Dhrs13	70451	Dehydroger	no|no chan	0	COG1028(T
Q6PHQ8	NAA35_M(	Naa35	Naa35	78689	N-alpha-ac	no|no chan	0	ENOG410X
Q8BWW9	PKN2_MO	Pkn2	Pkn2	109333	Serine/thre	no|no chan	0	ENOG410X
Q3TUU5	TEX30_MC	Tex30	Tex30	75623	Testis-expre	no|no chan	0	COG3571(F
Q3TUU0	Q3TUU0_M	Caap1	Caap1	67770	Caspase act	no|no chan	0	ENOG4111
Q6P6M7	SPCS_MOU	Sepsecs	Sepsecs	211006	O-phospho	no|no chan	0	COG0076(E
Q3UDJ2	Q3UDJ2_M	Sgpl1	Sgpl1	20397	sphinganin	no|no chan	0	COG0076(c
Q922J9	FACR1_M(	Far1	Far1	67420	Fatty acyl-C	no|no chan	0	ENOG410X

Q9Z315	SNUT1_MO	Sart1	Sart1	20227	U4/U6.U5 t	no | no chan	0	ENOG410
Q922J3	CLIP1_MO	Clip1	Clip1	56430	CAP-Gly do	no | no chan	0	COG5244(
D3YTV2	D3YTV2_M	Fzr1	Fzr1		Fizzy and c	no | no chan	0	ENOG410
Q8BHB4	WDR3_MO	Wdr3	Wdr3	269470	WD repeat-	no | no chan	0	ENOG410
Q08639	TFDP1_MO	Tfdp1	Tfdp1	21781	Transcripti	no | no chan	0	ENOG410
B2MWM9	B2MWM9_	Calr	Calr	12317	Calreticulir	no | no chan	0	ENOG410
P63073	IF4E_MOU	Eif4e	Eif4e	13684	Eukaryotic	no | no chan	0	COG5053(t
Q9CPT5	NOP16_MO	Nop16	Nop16	28126	Nucleolar p	no | no chan	0	ENOG4111
D3Z776	D3Z776_M	Socs6	Socs6		Suppressor	no | up	0	ENOG4111
A0AAA9W	A0AAA9W	Akap17a	Akap17a		A-kinase ar	no | down	0	ENOG410
Q8BGB7	ENOPH_M	Enoph1	Enoph1	67870	Enolase-ph	no | no chan	0	COG4229(I
B0V2M9	B0V2M9_M	Kdm4b	Kdm4b		[histone H3	no | no chan	0	COG5141(I
Q8BGB5	LIMD2_MO	Limd2	Limd2	67803	LIM domai	no | no chan	0	ENOG4111
Q8BGB2	TTC7A_MO	Ttc7a	Ttc7a	225049	Tetratricop	no | no chan	0	ENOG410
Q5SQB7	Q5SQB7_M	Npm1	Npm1	18148	Nucleopho	no | no chan	0	ENOG4111
B2RWB7	B2RWB7_M	Tigar	Tigar	319801	Fructose-2,(	no | no chan	0	COG0406((
F8VPK0	SKI3_MOU	Skic3	Skic3	218343	Superkiller	no | no chan	0	ENOG410
A0A0R4J28	A0A0R4J28	Dxo	Dxo	112403	Decapping	no | no chan	0	ENOG410
Q3UY05	Q3UY05_M	Ndufs8	Ndufs8	225887	NADH deh	no | no chan	0	COG1143(
Q91ZS8	RED1_MO	Adarb1	Adarb1	110532	Double-stra	no | no chan	0	ENOG410
Q5RL55	Q5RL55_M	Abcf1	Abcf1	224742	ATP-bindir	no | no chan	0	COG0488((
P97355	SPSY_MOU	Sms	Sms	20603	Spermine sy	no | no chan	0	COG0421(s
Q5RL51	GSTCD_M	Gstcd	Gstcd	67553	Glutathion	no | no chan	0	ENOG410
P59999	ARPC4_MO	Arpc4	Arpc4	68089	Actin-relate	no | no chan	0	ENOG4111
Q61810	LTBP3_MO	Ltbp3	Ltbp3	16998	Latent-tran	no | no chan	0	ENOG411(
D3YVD2	D3YVD2_M	Cep44	Cep44		Centrosome	no | down	0	ENOG4111
A0A2X0U2	A0A2X0U2	ARHGAP3	ARHGAP3	226652	ARHGAP3	no | no chan	0	ENOG410
P97358	TAF1B_MO	Taf1b	Taf1b	21340	TATA box-	no | no chan	0	ENOG410
Q8QZS1	HIBCH_MO	Hibch	Hibch	227095	3-hydroxyis	no | no chan	0	COG1024(
Q8QZS3	FLCN_MO	Flcn	Flcn	216805	Folliculin	no | no chan	0	ENOG410
E9PXR4	E9PXR4_M	Rab40c	Rab40c		Rab40C, mo	no | up	0	ENOG410
Q04750	TOP1_MO	Top1	Top1	21969	DNA topoi	no | no chan	0	COG3569(I
O09174	AMACR_M	Amacr	Amacr	17117	Alpha-meth	no | no chan	0	COG1804(I
Q7TMR0	PCP_MOU	Prcp	Prcp	72461	Lysosomal	no | no chan	0	ENOG410
Q8CJ40	CROCC_M	Crocc	Crocc	230872	Rootletin	no | no chan	0	ENOG410
O09172	GSH0_MO	Gclm	Gclm	14630	Glutamate-	no | no chan	0	COG0656((
A0ZVB6	A0ZVB6_M	Btaf1	Btaf1	107182	TBP-associ	no | no chan	0	ENOG410
Q1HDU4	Q1HDU4_I	Arhgap9	Arhgap9	216445	ArhGAP9	no | no chan	0	ENOG410
Q571L5	Q571L5_M	Ezh2	Ezh2	14056	Histone-lys	no | no chan	0	COG2940(c
Q9D787	PPIL2_MO	Ppil2	Ppil2	66053	RING-type	no | no chan	0	COG0652(S
Q3TLI0	TPC10_MO	Trappc10	Trappc10	216131	Trafficking	no | no chan	0	ENOG410
Q9CR47	NSA2_MO	Nsa2	Nsa2	59050	Ribosome b	no | no chan	0	COG2007(4
Q9CR46	SKA2_MO	Ska2	Ska2	66140	SKA compl	no | no chan	0	ENOG4111

Q9CWQ0	DPH5_MO	Dph5	Dph5	69740	Diphthine ı	no | no chan	0	COG1798(S
B2X2E0	B2X2E0_M	Parp8	Parp8	52552	Poly [ADP-	no | up	0	ENOG410Z
Q9CWQ8	CAST1_MC	Castor1	Castor1	71962	Cytosolic aı	no | no chan	0	ENOG4111
F8VQC1	F8VQC1_M	Srp72	Srp72	66661	Signal reco	no | no chan	0	ENOG410X
A0A0R4J1C	A0A0R4J1C	Steap3	Steap3	68428	STEAP fam	no | down	0	COG2085(F
P62309	RUXG_MC	Snrpg	Snrpg	68011	Small nucle	no | no chan	0	ENOG4112
P15105	GLNA_MC	Glul	Glul	14645	Glutamine	no | down	0	COG0174(c
P62307	RUXF_MO	Snrpf	Snrpf	69878	Small nucle	no | no chan	0	ENOG4111
P04939	MUP3_MO	Mup3	Mup3	17842	Major urinc	no | down	0	ENOG4111
P62301	RS13_MOU	Rps13	Rps13	68052	Small ribos	no | no chan	0	COG0184(c
B7ZN55	B7ZN55_M	Asah2	Asah2	54447	Neutral cer	no | no chan	0	ENOG410X
A0A0R4J1C	A0A0R4J1C	Rad51ap1	Rad51ap1	19362	RAD51 ass	no | no chan	0	ENOG4111
Q62273	S26A2_MO	Slc26a2	Slc26a2	13521	Sulfate tran	no | no chan	0	COG0659(s
B1AXH1	NHSL2_MC	Nhsl2	Nhsl2	100042480	NHS-like p	no | no chan	0	ENOG410Z
Q3TZ32	Q3TZ32_M	Aars1	Aars1	234734	Alanine--tR	no | no chan	0	COG0013(C
B2RSN3	B2RSN3_M	Tubb2b	Tubb2b	73710	Tubulin be	no | no chan	0	COG5023(e
F8WGD7	F8WGD7_I	Slc35b3	Slc35b3	108652	Adenosine	no | no chan	0	COG0697(F
F8WGD4	F8WGD4_I	Nek3	Nek3	23954	NIMA (nev	yes | up	1	ENOG410Y
F8WGD2	F8WGD2_I	Rin2	Rin2	74030	Ras and Ra	no | no chan	0	ENOG410Z
Q3URE1	ACSF3_MC	Acsf3	Acsf3	257633	Malonate--	no | no chan	0	COG0318(f
Q0VGN4	Q0VGN4_I	Stx16	Stx16	228960	Stx16 prote	no | no chan	0	COG5325(c
Q0VGN5	Q0VGN5_I	BC094435	BC094435	639390	BC094435 p	no | no chan	0	COG2801(F
H6WCS3	H6WCS3_M	il6st	il6st	16195	Interleukin	no | down	0	ENOG410Y
Q3UH38	Q3UH38_M	Unc5b	Unc5b	107449	Netrin rece	no | no chan	0	ENOG410X
H3BIZ7	H3BIZ7_M	Frmd4a	Frmd4a	209630	FERM dom	no | no chan	0	ENOG410X
Q8BK67	RCC2_MO	Rcc2	Rcc2	108911	Protein RC	no | no chan	0	COG5184(C
P30204	MSRE_MO	Msr1	Msr1	20288	Macrophag	no | up	0	ENOG410Y
A7VJA4	A7VJA4_M	Gmfg	Gmfg	63986	Glia matur	no | up	0	ENOG4111
A0A3B2WA	A0A3B2WA	Nfkbie	Nfkbie	18037	Nuclear fac	no | no chan	0	COG0666(F
Q8CB96	RASF4_MC	Rassf4	Rassf4	213391	Ras associa	no | up	0	ENOG410Y
A0A2I3BPX	A0A2I3BPX	Ano6	Ano6	105722	Anoctamin	no | no chan	0	ENOG410X
Q5SU47	Q5SU47_M	Cpeb4	Cpeb4		Cytoplasmi	no | no chan	0	ENOG410Y
F8WHU7	F8WHU7_I	Stag1	Stag1	20842	Cohesin sul	no | no chan	0	COG5537(S
Q9Z2B9	KS6A4_MC	Rps6ka4	Rps6ka4	56613	Ribosomal	no | no chan	0	ENOG410X
Q9D4G9	RMI1_MOU	Rmi1	Rmi1	74386	RecQ-medi	no | no chan	0	ENOG410Z
A0A2I3BPX	A0A2I3BPX	Elf1	Elf1	13709	E74 like ET	no | no chan	0	ENOG4111
Q5SX19	PIGL_MOU	Pigl	Pigl	327942	N-acetylglu	no | no chan	0	COG2120(I
P34960	MMP12_M	Mmp12	Mmp12	17381	Macrophag	no | up	0	ENOG410X
Q60866	PTER_MO	Pter	Pter	19212	N-acetyltau	no | no chan	0	COG1735(p
P49718	MCM5_MC	Mcm5	Mcm5	17218	DNA replic	no | no chan	0	COG1241(c
Q8BVL3	SNX17_MC	Snx17	Snx17	266781	Sorting nex	no | no chan	0	ENOG410X
Q3TG64	Q3TG64_M	Stk19	Stk19	54402	Serine/thre	no | no chan	0	ENOG410Y
P49717	MCM4_MC	Mcm4	Mcm4	17217	DNA replic	no | no chan	0	COG1241(c

Q99JZ4	Q99JZ4_M(	Sar1a	Sar1a	20224	Small COP	no | no chan	0	ENOG410Y
P49710	HCLS1_MC	Hcls1	Hcls1	15163	Hematopoi	no | no chan	0	ENOG410X
Q8VBU8	BANP_MO	Banp	Banp	53325	Protein BA	no | no chan	0	ENOG410Z
O70378	EMC8_MO	Emc8	Emc8	18117	ER membr	no | no chan	0	ENOG410Y
A6PWR8	A6PWR8_M	Usp43	Usp43	216835	ubiquitinyl	no | up	0	COG5560(p
Q3UHN9	NDST1_M(	Ndst1	Ndst1	15531	Bifunction	no | no chan	0	ENOG410X
Q9D5T0	ATAD1_M	Atad1	Atad1	67979	Outer mito	no | no chan	0	COG0464(A
Q91Y74	SIA4C_MO	St3gal4	St3gal4	20443	CMP-N-ac	no | no chan	0	ENOG410X
E9QN31	E9QN31_M	Nop2	Nop2	110109	NOP2 nucl	no | no chan	0	COG0144(C
Q5SV02	Q5SV02_M	Rad50	Rad50	19360	RAD50 dou	no | no chan	0	COG0419(p
Q8BKC5	IPO5_MOU	Ipo5	Ipo5	70572	Importin-5	no | no chan	0	ENOG410X
Q8VC88	GRAN_MC	Gca	Gca	227960	Grancalcin	no | up	0	ENOG410Y
Q4QQP1	Q4QQP1_M	Commd8	Commd8	27784	COMM dor	no | no chan	0	ENOG4111
Q3UQM7	Q3UQM7_	Slc7a5	Slc7a5	20539	Large neutr	no | no chan	0	COG0531(c
Q3TJP6	Q3TJP6_M	Cd34	Cd34	12490	Hematopoi	no | no chan	0	ENOG410Y
Q8K2U2	ALKB6_M(	Alkbh6	Alkbh6	233065	Alpha-keto	no | no chan	0	ENOG410X
O35074	PTGIS_MC	Ptgis	Ptgis	19223	Prostacycli	no | down	0	COG2124(I
O35075	VP26C_MC	Vps26c	Vps26c	13185	Vacuolar p	no | no chan	0	ENOG410X
E9Q8M9	E9Q8M9_M	Rgl1	Rgl1	19731	Ral guanin	no | no chan	0	ENOG410Z
Q8R4E9	CDT1_MO	Cdt1	Cdt1	67177	DNA replic	no | no chan	0	ENOG410X
O70194	EIF3D_MC	Eif3d	Eif3d	55944	Eukaryotic	no | no chan	0	ENOG410X
A2A7F4	A2A7F4_M	Rlf	Rlf	109263	Rearranged	no | no chan	0	COG5048(C
A2A7F7	A2A7F7_M	Mthfr	Mthfr	17769	Methylenet	no | no chan	0	COG0685(M
A0A088ML	IQIP1_MO	Iqcj-Schip1	Iqcj-Schip1	100505386	IQCJ-SCHI	no | no chan	0	ENOG4111
Q497H0	ZFAN3_M(	Zfand3	Zfand3	21769	AN1-type z	no | up	0	ENOG4111
A2ADB0	A2ADB0_M	Spen	Spen	56381	Spen family	no | no chan	0	ENOG410Z
Q8CHY3	DYM_MOU	Dym	Dym	69190	Dymeclin	no | no chan	0	ENOG410X
O88736	DHB7_MO	Hsd17b7	Hsd17b7	15490	3-keto-ster	no | no chan	0	ENOG410X
Q3TXU5	DHYS_MO	Dhps	Dhps	330817	Deoxyhypu	no | no chan	0	COG1899(c
P55850	DSC3_MOU	Dsc3	Dsc3	13507	Desmocolli	yes | up	1	ENOG410X
A0A5S8DH	A0A5S8DH	Phf20l1	Phf20l1	239510	PHD finger	no | no chan	0	ENOG410Y
Q3U699	Q3U699_M	Cfap410	Cfap410	67884	Cilia and fl	no | no chan	0	ENOG410Y
B1AUR6	MMS22_M(	Mms22l	Mms22l	212377	Protein MM	no | no chan	0	ENOG410Z
Q60943	I17RA_MO	Il17ra	Il17ra	16172	Interleukin	no | no chan	0	ENOG4111
Q8VDV8	MITD1_M(	Mitd1	Mitd1	69028	MIT domai	no | no chan	0	ENOG410X
Q6P5H2	NEST_MO	Nes	Nes	18008	Nestin	no | no chan	0	ENOG410X
Q3U3Q1	ULK3_MO	Ulk3	Ulk3	71742	Serine/thre	no | up	0	ENOG410X
Q3U3Q3	Q3U3Q3_M	Utp18	Utp18	217109	UTP18, sm	no | no chan	0	ENOG410Y
A0A2U3TZ	A0A2U3TZ	Polr2f	Polr2f		Polymerase	no | no chan	0	COG1758(F
E9PWG9	E9PWG9_M	Map3k5	Map3k5		mitogen-ac	no | no chan	0	ENOG410X
Q8CG46	SMC5_MO	Smc5	Smc5	226026	Structural r	no | no chan	0	COG1196(F
Q9DAB3	Q9DAB3_M	Arl8b	Arl8b	67166	ADP-ribosy	no | no chan	0	ENOG410X
Q8CG48	SMC2_MO	Smc2	Smc2	14211	Structural r	no | no chan	0	COG1196(F

A0A338P6N	A0A338P6N	Crybg3	Crybg3		Beta-gamm	no|no chan	0	
E9PWG4	E9PWG4_M	Myl1	Myl1	17901	Myosin ligh	no|up	0	COG5126(I
A0A0G2JE:	A0A0G2JE:	Ube2d3	Ube2d3		Ubiquitin-c	no|no chan	0	COG5078(N
A0A0G2JE:	A0A0G2JE:	Nme6	Nme6		Nucleoside	no|no chan	0	COG0105(N
Q8BYA0	TBCD_MO	Tbcd	Tbcd	108903	Tubulin-sp	no|no chan	0	COG5234(C
D3Z3C3	D3Z3C3_M	Hpse	Hpse		Heparanas	no|no chan	0	ENOG410Y
B2RUC7	B2RUC7_M	Strap	Strap	20901	Serine-thre	no|no chan	0	ENOG410X
Q5SF07	IF2B2_MO	Igf2bp2	Igf2bp2	319765	Insulin-like	no|no chan	0	ENOG410Z
Q01279	EGFR_MO	Egfr	Egfr	13649	Epidermal	no|no chan	0	ENOG410X
Q3UW32	Q3UW32_M	Anp32b	Anp32b	67628	Acidic leuci	no|no chan	0	ENOG4111
Q3TKR5	Q3TKR5_M	Rpl5	Rpl5	100503670	Large ribos	no|up	0	COG0256(T
D3Z2V6	D3Z2V6_M	Mras	Mras		Muscle and	no|no chan	0	COG1100(r
Q3U962	CO5A2_M	Col5a2	Col5a2	12832	Collagen al	no|no chan	0	ENOG411C
Q8BG07	PLD4_MO	Pld4	Pld4	104759	5'-3' exonuc	no|up	0	ENOG410X
O54785	LIMK2_M	Limk2	Limk2	16886	LIM domai	no|no chan	0	COG0515(I
O54784	DAPK3_M	Dapk3	Dapk3	13144	Death-asso	no|no chan	0	ENOG410X
O54786	DFFA_MO	Dffa	Dffa	13347	DNA fragm	no|no chan	0	ENOG4111
Q3ULB1	Q3ULB1_M	Tes	Tes	21753	Testin	no|no chan	0	ENOG410X
O54782	MA2B2_M	Man2b2	Man2b2	17160	Epididymis	no|no chan	0	ENOG410X
Q8C112	Q8C112_M	P3h4	P3h4	66180	Synaptonen	no|no chan	0	ENOG4111
Q9ES52	SHIP1_MO	Inpp5d	Inpp5d	16331	Phosphatid	no|no chan	0	COG5411(i
Q921V3	Q921V3_M	Nfs1	Nfs1	18041	cysteine des	no|no chan	0	COG1104(c
Q8C115	PKHH2_M	Plekhh2	Plekhh2	213556	Pleckstrin h	no|no chan	0	ENOG410X
Q91WG4	ELP2_MO	Elp2	Elp2	58523	Elongator c	no|no chan	0	ENOG410X
P28654	PGS2_MO	Dcn	Dcn	13179	Decorin	no|down	0	COG4886(I
P28656	NP1L1_M	Nap1l1	Nap1l1	53605	Nucleosom	no|no chan	0	ENOG410X
P28650	PURA1_M	Adss1	Adss1	11565	Adenylosuc	no|up	0	COG0104(I
P28653	PGS1_MO	Bgn	Bgn	12111	Biglycan	no|down	0	COG4886(I
Q9QZ11	EXO1_MO	Exo1	Exo1	26909	Exonucleas	no|no chan	0	COG0258(T
P28658	ATX10_M	Atxn10	Atxn10	54138	Ataxin-10	no|no chan	0	ENOG410Y
B7ZC28	B7ZC28_M	Scarf1	Scarf1		Scavenger r	no|no chan	0	ENOG410X
Q91VZ6	SMAP1_M	Smap1	Smap1	98366	Stromal me	no|no chan	0	COG5347(p
P04117	FABP4_M	Fabp4	Fabp4	11770	Fatty acid-l	no|down	0	ENOG4111
A0A0U1RN	A0A0U1RN	Zfp646	Zfp646		Zinc finger	no|no chan	0	COG5048(c
Q91WG3	TRUB2_M	Trub2	Trub2	227682	Pseudourid	no|no chan	0	ENOG411C
Q91VZ1	Q91VZ1_M	Snx2	Snx2	67804	Sorting nex	no|no chan	0	COG5391(T
B7ZC21	B7ZC21_M	Pkig	Pkig		Protein kin	no|no chan	0	ENOG410Z
Q91WG2	RABE2_M	Rabep2	Rabep2	70314	Rab GTPas	no|no chan	0	ENOG410X
B7ZC24	B7ZC24_M	Ncoa5	Ncoa5	228869	Nuclear rec	no|no chan	0	ENOG410X
P15920	VPP2_MO	Atp6v0a2	Atp6v0a2	21871	V-type prot	no|no chan	0	COG1269(N
Q3UJX2	Q3UJX2_M	Ctnnbl1	Ctnnbl1	66642	Beta-cateni	no|no chan	0	ENOG410X
A0A1N9PT	A0A1N9PT	Fbrs	Fbrs		Fibrosin	no|no chan	0	ENOG4111
O08832	GALT4_M	Galnt4	Galnt4	14426	Polypeptide	no|no chan	0	ENOG410X

A0A0N4SV	A0A0N4SV	Cnbp	Cnbp		CCHC-type	no|up	0	COG5082(t
Q543B9	Q543B9_M	Prep	Prep	19072	Prolyl endo	no|no chan	0	COG1505(p
O08789	MNT_MOU	Mnt	Mnt	17428	Max-bindin	no|no chan	0	ENOG4111
Q80T06	Q80T06_M	Eef1d	Eef1d	66656	Elongation	no|no chan	0	COG2092(I
A0A075B67	A0A075B67	Igkv4-53	Igkv4-53		Immunoglo	no|no chan	0	ENOG4111
A0A286YD	A0A286YD	Cmah	Cmah		Cytidine m	no|no chan	0	ENOG410Y
Q3U0K0	Q3U0K0_M	Ifi47	Ifi47	15953	IRG-type G	no|up	0	ENOG4112
Q8BUR3	FOXJ3_MO	Foxj3	Foxj3	230700	Forkhead b	no|no chan	0	COG5025(I
Q6Q783	KMT5C_M	Kmt5c	Kmt5c	232811	Histone-lys	no|no chan	0	ENOG410X
Q8BR90	RIMC1_MO	Rimoc1	Rimoc1	106064	RAB7A-inte	no|no chan	0	ENOG4111
Q8BUR9	MZT1_MO	Mzt1	Mzt1	76789	Mitotic-spi	no|no chan	0	ENOG410X
Q6P9L6	KIF15_MO	Kif15	Kif15	209737	Kinesin-like	no|no chan	0	COG5059(s
Q6ZQH8	NU188_MO	Nup188	Nup188	227699	Nucleopori	no|no chan	0	ENOG410Z
Q8C569	F118B_MO	Fam118b	Fam118b	109229	Protein FA	no|no chan	0	ENOG410Y
Q8C7K6	PCYXL_MO	Pcyox1l	Pcyox1l	240334	Prenylcyste	no|no chan	0	ENOG410Y
Q3UXL4	KIZ_MOUS	Kiz	Kiz	228730	Centrosome	no|no chan	0	ENOG4111
Q8C561	LMBD2_M	Lmbrd2	Lmbrd2	320506	G-protein c	no|up	0	ENOG410X
Q9D0B5	TSTD3_MO	Tstd3	Tstd3	77032	Thiosulfate	no|no chan	0	COG0607(F
Q9D0B0	SRSF9_MO	Srsf9	Srsf9	108014	Serine/argi	no|no chan	0	COG0724(r
Q8R138	TM119_MO	Tmem119	Tmem119	231633	Transmemb	no|no chan	0	ENOG410Y
E0CY69	E0CY69_M	Tcf19	Tcf19		Transcripti	no|up	0	ENOG4111
Q3URX8	Q3URX8_M	Gemin4	Gemin4	276919	Gem-associ	no|no chan	0	ENOG410X
Q80ZJ1	RAP2A_MO	Rap2a	Rap2a	76108	Ras-related	no|no chan	0	COG1100(r
Q9WV95	PHLA3_MO	Phlda3	Phlda3	27280	Pleckstrin h	no|up	0	ENOG410Y
A0A509	A0A509_M	Myot	Myot	58916	Myotilin	no|up	0	ENOG410Z
P70274	SEPP1_MO	Selenop	Selenop	20363	Selenoprote	no|no chan	0	ENOG4111
Q62093	SRSF2_MO	Srsf2	Srsf2	20382	Serine/argi	no|no chan	0	ENOG4111
Q7TPD1	FBX11_MO	Fbxo11	Fbxo11	225055	F-box only	no|no chan	0	ENOG410Y
A2TJ61	A2TJ61_MO	Pglyrp2	Pglyrp2	57757	Peptidoglyc	no|no chan	0	ENOG4111
Q3V117	Q3V117_M	Acly	Acly	104112	ATP-citrate	no|no chan	0	COG0074(S
Q3TJC2	Q3TJC2_M	Pafah1b3	Pafah1b3	18476	Platelet-act	no|no chan	0	ENOG410X
Q3TBM0	Q3TBM0_M	Blzf1	Blzf1	66352	Golgin-45	no|no chan	0	ENOG410Y
A0A1L1SS3	A0A1L1SS3	Anln	Anln		Anillin, act	no|no chan	0	ENOG410(
A0A679AX	A0A679AX	Gsdmd	Gsdmd	69146	Gasdermin	no|no chan	0	ENOG4111
Q66JY4	Q66JY4_M	Cdc7	Cdc7	12545	non-specifi	no|down	0	ENOG410Y
E9Q2K8	E9Q2K8_M	S100a14	S100a14	66166	S100 calciu	no|down	0	ENOG4111
A0A1Z3MF	A0A1Z3MF	pyhinA	pyhinA	226691	PyhinA	no|no chan	0	ENOG410Y
P97290	IC1_MOUS	Serping1	Serping1	12258	Plasma pro	no|no chan	0	COG4826(s
E9Q0H6	E9Q0H6_M	Fabp7	Fabp7		Fatty acid-l	no|down	0	ENOG4111
P97299	SFRP2_MO	Sfrp2	Sfrp2	20319	Secreted fri	no|no chan	0	ENOG410X
P97298	PEDF_MO	Serpinf1	Serpinf1	20317	Pigment ep	no|no chan	0	COG4826(s
Q9D1M7	FKB11_MO	Fkbp11	Fkbp11	66120	Peptidyl-pr	no|no chan	0	COG0545(g
A0A1Y7VJ(	A0A1Y7VJ(	Snapc1	Snapc1		Small nucle	no|no chan	0	ENOG410Z

E9QQ33	E9QQ33_M	Sh3rf1	Sh3rf1	59009	E3 ubiquiti	no|no chan	0	ENOG410
A0A1D5RL	A0A1D5RL	Wdr59	Wdr59	319481	WD repeat	no|no chan	0	ENOG410
E9QQ30	E9QQ30_M	Eif2ak3	Eif2ak3	13666	PRKR-like	no|no chan	0	ENOG410
E9QQ38	E9QQ38_M	Ddhd1	Ddhd1	114874	DDHD dor	no|no chan	0	ENOG410
Q9D6T0	NOSIP_MO	Nosip	Nosip	66394	Nitric oxid	no|no chan	0	ENOG410
P10833	RRAS_MO	Rras	Rras	20130	Ras-related	no|no chan	0	COG1100(I
P26039	TLN1_MO	Tln1	Tln1	21894	Talin-1	no|no chan	0	ENOG410
Q9D6T9	Q9D6T9_M	Mrps36	Mrps36	66128	Mitochond	no|no chan	0	ENOG410
Q9ESW8	PGPI_MO	Pgpep1	Pgpep1	66522	Pyroglutam	no|no chan	0	COG2039(I
Q6P6J4	ALKB2_MO	Alkbh2	Alkbh2	231642	DNA oxida	no|up	0	COG3145(I
D3YZU6	D3YZU6_M	Haghl	Haghl	68977	Hydroxyacy	no|no chan	0	COG0491(I
Q6P6J0	ADAT2_MO	Adat2	Adat2	66757	tRNA-speci	no|down	0	COG0590(c
Q6P6J9	TXD15_MO	Txndc15	Txndc15	69672	Thioredoxi	no|no chan	0	ENOG4111
A1L3B8	A1L3B8_M	Psmd7	Psmd7	17463	Proteasome	no|no chan	0	COG1310(I
B2RR30	B2RR30_M	Kat2b	Kat2b	18519	Histone ace	yes|up	1	COG5076(c
Q8R4X3	RBM12_MO	Rbm12	Rbm12	75710	RNA-bindi	no|no chan	0	ENOG4111
A0A0A6YW	A0A0A6YW	Usp4	Usp4	22258	Ubiquitin c	no|no chan	0	COG5560(s
A1L3B6	A1L3B6_M	Nol7	Nol7	70078	Nol7 protei	no|no chan	0	ENOG410
Q9CSV6	SFT2C_MO	Sft2d3	Sft2d3	67158	Vesicle trar	no|up	0	COG5102(S
Q3TZ21	Q3TZ21_M	Aurkaip1	Aurkaip1	66077	Small ribos	no|no chan	0	ENOG4111
Q9CY06	Q9CY06_MOUSE	Globin domain-		15122	Globin don	no|no chan	0	COG1018(I
Q3TI79	Q3TI79_M	Dkc1	Dkc1	245474	H/ACA rib	no|no chan	0	COG0130(I
P17183	ENOG_MO	Eno2	Eno2	13807	Gamma-en	no|no chan	0	COG0148(C
P17182	ENOA_MO	Eno1	Eno1	433182; 138	Alpha-enol	no|no chan	0	COG0148(C
O55201	SPT5H_MO	Supt5h	Supt5h	20924	Transcripti	no|no chan	0	COG5164(T
Q9D902	T2EB_MO	Gtf2e2	Gtf2e2	68153	General tra	no|no chan	0	COG5174(g
Q9D4F2	PLPP6_MO	Plpp6	Plpp6	74411	Polyisopren	no|no chan	0	ENOG410
Q3UDQ7	Q3UDQ7_	Afg3l2	Afg3l2	69597	AAA+ ATP	no|no chan	0	COG0465(r
E0CXD2	E0CXD2_M	Etv1	Etv1		Ets variant	no|no chan	0	ENOG410
A0A087WP	A0A087WP	Dhx9	Dhx9	13211	RNA helico	no|no chan	0	COG1643(T
D1MQ07	D1MQ07_M	Kcnn4	Kcnn4	16534	Small cond	no|no chan	0	ENOG410
A0ABA7IW	A0ABA7IW21_MOUSE	Integrator complex sul			Ints8	no|no chan	0	ENOG410
A0A5H1ZR	A0A5H1ZR	Mecom	Mecom		MDS1 and	no|no chan	0	COG5048(T
Q8K0P3	MEAK7_M	Meak7	Meak7	74347	MTOR-asso	no|no chan	0	ENOG410
P48759	PTX3_MO	Ptx3	Ptx3	19288	Pentraxin-r	no|up	0	ENOG410
O35245	PKD2_MO	Pkd2	Pkd2	18764	Polycystin-2	no|no chan	0	ENOG410
A0A0N4SU	A0A0N4SU	Ptpro	Ptpro	19277	Protein tyro	no|down	0	COG5599(I
P48754	BRCA1_MO	Brca1	Brca1	12189	Breast canc	no|no chan	0	ENOG4111
P48755	FOSL1_MO	Fosl1	Fosl1	14283	Fos-related	no|up	0	ENOG4111
Q8VE99	VMA22_MO	Ccdc115	Ccdc115	69668	Coiled-coil	no|no chan	0	ENOG410
Q6PIP8	Q6PIP8_M	Igh	Igh		Igh protein	no|up	0	ENOG410
A0A2X0SF	A0A2X0SF	ECT2	ECT2	13605	ECT2	no|no chan	0	ENOG410
Q3U1T9	DEN1B_M	Dennd1b	Dennd1b	329260	DENN don	yes|up	1	ENOG410

Q8VEJ4	NLE1_MO	Nle1	Nle1	217011	Notchless p	no | no chan	0	ENOG410)
Q99KE1	MAOM_M	Me2	Me2	107029	NAD-deper	no | no chan	0	COG0281(U
Q3TK15	Q3TK15_M	Sh3bp5l	Sh3bp5l	79566	SH3 domain	no | no chan	0	ENOG410Z
Q99KW9	TIP_MOUS	Itfg1	Itfg1	71927	T-cell immu	no | no chan	0	ENOG410)
Q5DU37	ZFY26_MC	Zfyve26	Zfyve26	211978	Zinc finger	no | no chan	0	ENOG410)
P0C605	KGP1_MO	Prkg1	Prkg1	19091	cGMP-depe	no | no chan	0	ENOG410)
Q3U484	Q3U484_M	Ddx3y	Ddx3y	26900	RNA helicc	no | no chan	0	ENOG410)
A0A1L1SU	A0A1L1SU	Scamp2	Scamp2		Secretory cc	no | no chan	0	ENOG410)
Q3UEW2	Q3UEW2_I	Dmtn	Dmtn	13829	HP domain	no | no chan	0	ENOG410)
A2RSB1	A2RSB1_M	Nap1l4	Nap1l4	17955	Nucleosom	no | no chan	0	ENOG410)
Q8BH80	Q8BH80_M	Vapb	Vapb	56491	Vesicle-ass	no | no chan	0	COG5066(C
Q8VE91	RETR1_MC	Retreg1	Retreg1	66270	Reticulophe	no | no chan	0	ENOG410)
P38060	HMGCL_M	Hmgcl	Hmgcl	15356	Hydroxyme	no | no chan	0	COG0119(C
Q7TMM9	TBB2A_MC	Tubb2a	Tubb2a	22151	Tubulin be	no | no chan	0	COG5023(u
Q80U04	PJA2_MOU	Pja2	Pja2	224938	E3 ubiquiti	no | no chan	0	ENOG4112
Q924A2	CIC_MOUS	Cic	Cic	71722	Protein cap	no | no chan	0	ENOG410Y
P70122	SBDS_MOU	Sbds	Sbds	66711	Ribosome n	no | no chan	0	COG1500(F
E9Q580	E9Q580_M	Myo1b	Myo1b		Myosin IB	no | no chan	0	COG5022(C
P70121	ZHX1_MO	Zhx1	Zhx1	22770	Zinc fingers	no | down	0	ENOG410Z
Q3UCQ1	FOXK2_M	Foxk2	Foxk2	68837	Forkhead b	no | no chan	0	COG5025(s
P21126	UBL4A_MC	Ubl4a	Ubl4a	27643; 1001	Ubiquitin-l	no | no chan	0	COG5272(t
A0A991EN	A0A991EN	Sfpq	Sfpq		Splicing fac	no | no chan	0	ENOG410)
P29351	PTN6_MO	Ptpn6	Ptpn6	15170	Tyrosine-pr	no | up	0	COG5599(C
Q9CXY7	Q9CXY7_M	Map2k5	Map2k5	23938	mitogen-ac	no | no chan	0	ENOG410)
Q3TSZ4	Q3TSZ4_M	Galm	Galm	319625	Aldose 1-ep	no | down	0	COG2017(c
E9QPA1	E9QPA1_M	Ric1	Ric1	226089	Protein RIC	no | no chan	0	ENOG410)
Q6P9Q4	FHOD1_M	Fhod1	Fhod1	234686	FH1/FH2 d	no | no chan	0	ENOG410)
Q6P9Q6	FKB15_MC	Fkbp15	Fkbp15	338355	FK506-binc	no | no chan	0	ENOG4112
Q3UUW5	Q3UUW5_I	Ccnh	Ccnh	66671	Cyclin-H	no | no chan	0	COG5333(c
Q3UUW7	Q3UUW7_I	Extl3	Extl3	54616	Exostosin-l	no | no chan	0	ENOG410)
A0A1W2P7	A0A1W2P7	Derl3	Derl3		Derlin	no | no chan	0	COG5291(F
Q9D0J4	ARL2_MOU	Arl2	Arl2	56327	ADP-ribosy	no | no chan	0	ENOG410)
Q9DC40	TELO2_MC	Telo2	Telo2	71718	Telomere le	no | no chan	0	ENOG410)
E9Q5J9	E9Q5J9_M	Tpm3	Tpm3	59069	Tropomyos	no | no chan	0	ENOG410)
E2JF22	PIEZ1_MC	Piezo1	Piezo1	234839	Piezo-type	no | no chan	0	ENOG410Y
Q9CQT0	Q9CQT0_M	Thg1l	Thg1l	66628	tRNA(His)	no | no chan	0	COG4021(g
Q9CQT1	MTNA_MC	Mri1	Mri1	67873	Methylthior	no | no chan	0	COG0182(C
B0QZU1	B0QZU1_M	Pttg1	Pttg1		Securin	no | no chan	0	ENOG410Z
Q9Z0E0	NCDN_MC	Ncdn	Ncdn	26562	Neurochon	no | no chan	0	ENOG410)
Q8C9A2	ENDOV_M	Endov	Endov	338371	Endonuclec	no | no chan	0	COG1515(S
O88384	VTI1B_MC	Vti1b	Vti1b	53612	Vesicle trar	no | no chan	0	ENOG4111
E9PVA8	GCN1_MC	Gcn1	Gcn1	231659	Stalled ribo	no | no chan	0	ENOG410)
Q5I043	UBP28_MC	Usp28	Usp28	235323	Ubiquitin c	yes | up	1	COG5077(I

Q8BS90	BORA_MC	Bora	Bora	77744	Protein aur	no | no chan	0	ENOG410Z
Q69ZF8	MSL2_MOI	Msl2	Msl2	77853	E3 ubiquiti	no | down	0	ENOG410Z
B1AQY9	B1AQY9_M	Septin8	Septin8	20362	Septin	no | no chan	0	COG5019(N
Q8BS95	GPHR_MC	Gpr89	Gpr89	67549	Golgi pH re	no | no chan	0	ENOG410X
Q3TMP8	TM38A_MC	Tmem38a	Tmem38a	74166	Trimeric in	no | no chan	0	ENOG410X
B1AS81	B1AS81_M	St3gal3	St3gal3		ST3 beta-ga	no | no chan	0	ENOG410X
Q69ZF7	CNNM4_M	Cnnm4	Cnnm4	94220	Metal trans	no | no chan	0	COG1253(C
Q99N09	M4A6B_MC	Ms4a6b	Ms4a6b	69774	Membrane-	no | down	0	ENOG4111
D3Z4Z0	D3Z4Z0_M	Tada2b	Tada2b	231151	Transcripti	no | no chan	0	COG5114(t
B1AQY2	B1AQY2_M	Arhgap23	Arhgap23	58996	Rho GTPas	no | no chan	0	ENOG410Y
Q69ZF3	GBA2_MO	Gba2	Gba2	230101	Non-lysoso	no | no chan	0	COG4354(N
H6UWL1	H6UWL1_M	Figla	Figla	26910	Factor in th	no | no chan	0	ENOG4112
Q61753	SERA_MO	Phgdh	Phgdh	236539	D-3-phosph	no | no chan	0	COG0111(I
P35330	ICAM2_MC	Icam2	Icam2	15896	Intercellula	no | no chan	0	ENOG410Z
Q08093	CNN2_MC	Cnn2	Cnn2	12798	Calponin-2	no | no chan	0	COG5199(c
D3YX43	VSI10_MO	Vsig10	Vsig10	231668	V-set and ir	no | no chan	0	ENOG4111
Q3UXD8	Q3UXD8_M	Rps6kb1	Rps6kb1	72508	Ribosomal	no | no chan	0	ENOG410X
Q9JHS9	CWC15_MC	Cwc15	Cwc15	66070	Spliceosom	no | no chan	0	ENOG410Z
A0A1Y7VJ4	A0A1Y7VJ4	Tbca	Tbca		Tubulin-spe	no | no chan	0	ENOG4111
Q9JHS3	LTOR2_MC	Lamtor2	Lamtor2	83409	Ragulator c	no | no chan	0	ENOG4111
E9Q704	E9Q704_M	Pam	Pam		Peptidylgly	no | no chan	0	ENOG410X
B2RRK2	B2RRK2_M	Sdk1	Sdk1	330222	Sidekick ho	no | no chan	0	ENOG410Z
A0A0G2JG	A0A0G2JG	Igkv12-89	Igkv12-89	384411	Immunoglo	no | down	0	ENOG4111
A0AAQ4VI	A0AAQ4VMU6_MOUSE Epidermal growth fa				Eps15l1	no | no chan	0	ENOG410X
A0AAQ4VI	A0AAQ4VMU7_MOUSE Smoothelin OS=Mus				Smtn	no | no chan	0	COG5069(N
Q3U8S0	Q3U8S0_M	Ap3s1	Ap3s1	11777	Adaptor-rel	no | no chan	0	COG5030(e
A2AM97	A2AM97_M	RP23-436K	RP23-436K	110954; 100	Large ribos	no | no chan	0	COG0197(I
P27601	GNA13_MC	Gna13	Gna13	14674	Guanine nu	no | no chan	0	ENOG410X
P27600	GNA12_MC	Gna12	Gna12	14673	Guanine nu	no | no chan	0	ENOG410X
A0A494BBI	A0A494BBI	Scyl1	Scyl1		N-terminal	no | no chan	0	ENOG410X
Q8BGK6	YLAT2_MC	Slc7a6	Slc7a6	330836	Y+L amino	yes | down	1	COG0531(U
A0A0U1RP	A0A0U1RP	Cdipt	Cdipt		CDP-diacyl	no | no chan	0	COG0558(U
Q8VDG5	PPCS_MOI	Ppcs	Ppcs	106564	Phosphopa	no | no chan	0	COG0452(I
A0A1L1SSL	A0A1L1SSL	Atr	Atr	245000	Serine/thre	no | no chan	0	COG5032(I
Q05769	PGH2_MO	Ptgs2	Ptgs2	19225	Prostaglanc	no | up	0	ENOG410X
D3YUL0	D3YUL0_M	Zfand2a	Zfand2a		Zinc finger,	no | up	0	ENOG4111
Q8R3W5	SEN15_MC	Tsen15	Tsen15	66637	tRNA-splic	no | no chan	0	ENOG4112
Q8K1S6	SPIR2_MO	Spire2	Spire2	234857	Protein spir	no | no chan	0	ENOG4111
D3YZ61	D3YZ61_M	C1qtnf3	C1qtnf3	81799	C1q and tu	no | down	0	ENOG410Y
Q3UNG6	Q3UNG6_M	Klk1b21	Klk1b21	16616	Kallikrein 1	no | no chan	0	COG5640(p
E9Q4T1	E9Q4T1_M	Arhgap19	Arhgap19	71085	Rho GTPas	no | no chan	0	ENOG411C
Q8R3W2	Q8R3W2_M	Trappc2b	Trappc2b	66050	Trafficking	no | no chan	0	COG5603(t
A0A0B4J1E	A0A0B4J1E	Gpr39	Gpr39	71111	G protein-c	no | down	0	ENOG410X

A0A0B4J1E	A0A0B4J1E	Uck2	Uck2		uridine/cyt	no | down	0	COG0572(u
A0A0B4J1E	A0A0B4J1E	Fcgr2b	Fcgr2b	14130	Fc receptor	no | down	0	ENOG410Y
P08113	ENPL_MO	Hsp90b1	Hsp90b1	22027	Endoplasm	no | no chan	0	COG0326(C
A2AE98	A2AE98_M	Wdtc1	Wdtc1		WD and tet	no | no chan	0	ENOG410X
A0A0B4J1E	A0A0B4J1E	Ighmbp2	Ighmbp2	20589	Immunoglo	no | no chan	0	COG1112(S
Q498A0	Q498A0_M	Arl5a	Arl5a	75423	ADP-ribosy	no | no chan	0	COG1100(C
D3Z7X0	D3Z7X0_M	Acad12	Acad12	338350	Acyl-Coenz	no | no chan	0	ENOG410X
Q9D6I7	DIK1A_MC	Dipk1a	Dipk1a	67266	Divergent p	no | no chan	0	ENOG411C
Q925E7	2ABD_MO	Ppp2r2d	Ppp2r2d	52432	Serine/thre	no | no chan	0	COG5170(p
Q3UV99	Q3UV99_M	Vps37a	Vps37a	52348	Vacuolar p	no | no chan	0	ENOG4111
Q3U9G9	LBR_MOU	Lbr	Lbr	98386	Delta(14)-st	no | no chan	0	ENOG410X
Q9JIK9	RT34_MOU	Mrps34	Mrps34	79044	Small ribos	no | no chan	0	ENOG4111
P54279	PMS2_MO	Pms2	Pms2	18861	Mismatch r	no | no chan	0	COG0323(T
Q3UDL6	Q3UDL6_M	Aplp2	Aplp2	11804	Amyloid-lik	no | no chan	0	ENOG410Z
Q3UDL9	Q3UDL9_M	Bclaf1	Bclaf1	72567	Bcl-2-assoc	no | no chan	0	ENOG411C
Q922H1	ANM3_MC	Prmt3	Prmt3	71974	Protein arg	no | no chan	0	ENOG410X
P54276	MSH6_MO	Msh6	Msh6	17688	DNA mism	no | no chan	0	COG0249(T
Q80VQ1	LRRC1_MC	Lrrc1	Lrrc1	214345	Leucine-ric	no | no chan	0	COG4886(S
Q9JIK5	DDX21_M	Ddx21	Ddx21	56200	Nucleolar R	no | no chan	0	COG0513(r
P05532	KIT_MOUS	Kit	Kit	16590	Mast/stem	no | down	0	COG0515(c
F8VPM9	F8VPM9_M	Hivep1	Hivep1	110521	Human imr	no | no chan	0	COG5048(T
I6L987	I6L987_MC	Aebp2	Aebp2	11569	AE binding	no | no chan	0	COG5048(C
P62754	RS6_MOUS	Rps6	Rps6	20104	Small ribos	no | no chan	0	COG2125(4
P0CG14	DERPC_M	Derpc	Derpc	214987; 116	Decreased e	no | no chan	0	ENOG410Z
E3WH30	E3WH30_M	Sfr1	Sfr1	67788	Swi5-depen	no | no chan	0	ENOG4112
Q78JE5	FBX22_MC	Fbxo22	Fbxo22	71999	F-box only	no | no chan	0	ENOG411C
P62751	RL23A_MC	Rpl23a	Rpl23a	268449	Large ribos	no | no chan	0	COG0089(C
F8VPM7	F8VPM7_M	Erc1	Erc1	111173	ELKS/RAB	no | no chan	0	ENOG410Y
D3Z1F7	D3Z1F7_M	4833439L19	4833439L19	97820	Putative m	no | no chan	0	ENOG410Y
Q3UY68	Q3UY68_M	Camk1	Camk1	52163	Protein kin	no | no chan	0	ENOG410X
Q6P8J7	KCRS_MO	Ckmt2	Ckmt2	76722	Creatine ki	no | up	0	COG3869(s
E9PZ00	E9PZ00_M	Psap	Psap	19156	Prosaposin	no | up	0	ENOG410X
P97370	AT1B3_MC	Atp1b3	Atp1b3	11933	Sodium/po	no | no chan	0	ENOG4111
Q6ZPJ3	UBE2O_M	Ube2o	Ube2o	217342	(E3-indeper	no | no chan	0	ENOG410X
P97376	FRG1_MO	Frg1	Frg1	14300	Protein FR	no | no chan	0	ENOG411C
P97377	CDK2_MO	Cdk2	Cdk2	12566	Cyclin-depe	no | no chan	0	ENOG410X
P97379	G3BP2_MC	G3bp2	G3bp2	23881	Ras GTPas	no | no chan	0	ENOG410Y
Q6ZPJ0	TEX2_MO	Tex2	Tex2	21763	Testis-expr	no | no chan	0	ENOG410Z
E9QP99	E9QP99_M	Golga3	Golga3	269682	Golgin A3	no | no chan	0	ENOG410Y
E9PXP7	E9PXP7_M	Ston2	Ston2		Stonin-2	no | no chan	0	ENOG410X
Q3TLK4	Q3TLK4_M	Esco2	Esco2	71988	Uncharacte	no | down	0	ENOG410X
C5H0E8	C5H0E8_M	Gm9392	Gm9392	668838	small monc	no | no chan	0	COG1100(I
Q3TVK3	Q3TVK3_M	Dnpep	Dnpep	13437	Aspartyl an	no | no chan	0	COG1362(M

A0A1X7SB'	A0A1X7SB'	Cbll1	Cbll1		E3 ubiquiti	no | no chan	0	ENOG410X
B2RS91	RRN3_MO	Rrn3	Rrn3	106298	RNA polym	no | no chan	0	ENOG410X
Q9CR23	TMEM9_M	Tmem9	Tmem9	66241	Proton-tran	no | down	0	ENOG4111
Q6IWE3	Q6IWE3_M	Gapd	Gapd		glyceraldeh	no | up	0	COG0057(g
Q9CR25	DPH2_MO	Dph2	Dph2	67728	2-(3-amino-	no | no chan	0	COG1736(c
Q9CR24	NUDT8_M	Nudt8	Nudt8	66387	Mitochondı	no | no chan	0	COG0494(T
Q9CR27	WASC3_M(	Washc3	Washc3	67282	WASH com	no | no chan	0	ENOG4111
P16110	LEG3_MO	Lgals3	Lgals3	16854	Galectin-3	no | up	0	ENOG4111
Q9CR29	CCD43_M(	Ccdc43	Ccdc43	52715	Coiled-coil	yes | up	1	ENOG4111
Q542A1	Q542A1_M	Bcap29	Bcap29	12033	B-cell recep	no | no chan	0	COG5374(b
P0C6B7	IGSF6_MO	Igsf6	Igsf6	80719	Immunoglo	no | no chan	0	ENOG410Z
Q9CWS0	DDAH1_M	Ddah1	Ddah1	69219	N(G),N(G)-	no | down	0	COG1834(c
P62325	BTG1_MO	Btg1	Btg1	12226	Protein BT(	no | no chan	0	ENOG410Z
P07356	ANXA2_M	Anxa2	Anxa2	12306	Annexin A2	no | no chan	0	ENOG410X
P62320	SMD3_MO	Snrpd3	Snrpd3	67332	Small nucle	no | no chan	0	COG1958(C
A0A5F8MP	A0A5F8MP	Sh3pxd2b	Sh3pxd2b	268396	SH3 and PX	no | no chan	0	ENOG410Y
A0A5F8MP	A0A5F8MP	Usp25	Usp25	30940	ubiquitinyl	no | no chan	0	COG5077(A
P19324	SERPH_M(	Serpinh1	Serpinh1	12406	Serpin H1	no | no chan	0	COG4826(s
P04919	B3AT_MOI	Slc4a1	Slc4a1	20533	Band 3 anic	no | no chan	0	ENOG410X
Q9D2E2	TOE1_MO	Toe1	Toe1	68276	Target of E	no | no chan	0	ENOG410X
Q8BZG5	MT25B_M(	Mettl25b	Mettl25b	229503	Methyltrans	no | up	0	ENOG410X
A0A0R4J1E	A0A0R4J1E	Tfap2a	Tfap2a	21418	Transcripti	no | no chan	0	ENOG410X
Q9D023	MPC2_MO	Mpc2	Mpc2	70456	Mitochondı	no | no chan	0	ENOG4111
Q9D020	5NT3A_M(	Nt5c3a	Nt5c3a	107569	Cytosolic 5'	no | no chan	0	ENOG410Z
A0A0R4J1E	A0A0R4J1E	Dbn1	Dbn1	56320	Drebrin 1	no | no chan	0	ENOG410Y
Q3UTY6	THSD4_M(	Thsd4	Thsd4	207596	Thrombosp	no | no chan	0	ENOG411(
A2AR99	A2AR99_M	Calcrl	Calcrl	54598	Calcitonin (	no | no chan	0	ENOG410X
E9PYR1	E9PYR1_M	Fbxl18	Fbxl18		F-box and l	no | no chan	0	ENOG410Z
P41105	RL28_MOU	Rpl28	Rpl28	19943	Large ribos	no | no chan	0	ENOG4111
A0A2I3BQ1	A0A2I3BQ1	Vmn1r199	Vmn1r199		Vomeronas	no | no chan	0	ENOG4111
A8Y5B9	A8Y5B9_M	Irf1	Irf1		Interferon r	no | up	0	ENOG4111
Q3U7R1	ESYT1_MC	Esyt1	Esyt1	23943	Extended sy	no | no chan	0	COG5038(F
G3UW82	MYH2_MC	Myh2	Myh2	17882	Myosin-2	no | up	0	COG5022(C
G3UW83	G3UW83_M	Tonsl	Tonsl	72749	Tonsoku-lik	no | no chan	0	COG0666(S
Q00897	A1AT4_MC	Serpina1d	Serpina1d	20703	Alpha-1-an	no | no chan	0	COG4826(s
Q3UC65	RSRP1_MC	Rsrp1	Rsrp1	27981	Arginine/se	no | no chan	0	ENOG410Z
Q0VB14	Q0VB14_M	Irak3	Irak3	73914	Interleukin-	no | no chan	0	COG0515(l
Q3UC67	Q3UC67_M	Acsl5	Acsl5	433256	Long-chain	no | no chan	0	COG1022(r
Q54AA6	Q54AA6_M	Dgat1	Dgat1	13350	O-acyltrans	no | no chan	0	COG5056(C
Q54AA2	Q54AA2_M	Pycard	Pycard	66824	Apoptosis-c	no | up	0	ENOG4111
Q00898	A1AT5_MC	Serpina1e	Serpina1e	20704	Alpha-1-an	no | down	0	COG4826(s
Q00899	TYY1_MOI	Yy1	Yy1	22632	Transcripti	no | no chan	0	COG5048(c
Q4FZC9	SYNE3_M(	Syne3	Syne3	212073	Nesprin-3	no | no chan	0	ENOG4111

Q3UXU0	Q3UXU0_MTsc22d1	Tsc22d1	21807	TSC22 dom	no | no chan		0	ENOG4111
A0A0J9YKI	A0A0J9YKICkm	Ckm		Creatine ki	no | up		0	COG3869(/
D6RHP2	D6RHP2_MErcc4	Ercc4		Excision re	no | no chan		0	COG1948(I
F8WHW8	F8WHW8_ISmim14	Smim14		Small integ	no | down		0	ENOG4112
A0A0A6YX	A0A0A6YXStx6	Stx6		Syntaxin 6	no | no chan		0	ENOG410Z
Q5SX39	MYH4_MCMyh4	Myh4	17884	Myosin-4	no | up		0	COG5022(c
F8WHW6	F8WHW6_IPip5k1c	Pip5k1c	18717	Phosphatid	no | no chan		0	COG5253(S
F8WHW3	F8WHW3_IPip4p1	Pip4p1	219024	Phosphatid	no | no chan		0	ENOG410X
Q62356	FSTL1_MCFstl1	Fstl1	14314	Follistatin-	no | no chan		0	ENOG410Z
Q1XH17	TRI72_MOTrim72	Trim72	434246	Tripartite n	no | up		0	ENOG4111
Q0VDP1	Q0VDP1_MSlc2a13	Slc2a13	239606	Slc2a13 pro	no | down		0	ENOG410X
P13405	RB_MOUSRb1	Rb1	19645	Retinoblast	no | no chan		0	ENOG410X
Q8BVN4	MTEF4_MMterf4	Mterf4	69821	Transcripti	no | no chan		0	ENOG410Y
Q9D8W5	PSD12_MCPsmd12	Psmd12	66997	26S proteas	no | no chan		0	COG5071(2
Q9D8W7	OCAD2_MOciad2	Ociad2	433904	OCIA dom	no | no chan		0	ENOG4111
Q3UHH1	ZSWM8_MZswim8	Zswim8	268721	Zinc finger	no | no chan		0	ENOG410X
E9PVA6	E9PVA6_MGit2	Git2	26431	GIT ArfGA	no | no chan		0	ENOG410X
Q8R2Y8	PTH2_MOPtrh2	Ptrh2	217057	Peptidyl-tR	no | no chan		0	COG1990(T
E9PVA2	E9PVA2_MRrp8	Rrp8	101867	Ribosomal	no | no chan		0	ENOG4111
O70422	TF2H4_MCGtf2h4	Gtf2h4	14885	General tra	no | no chan		0	COG5144(T
O70423	AOC3_MOAoc3	Aoc3	11754	Amine oxid	no | down		0	COG3733(c
Q3UAT2	Q3UAT2_MTram1	Tram1	72265	Translocati	no | no chan		0	ENOG410X
O70421	FZD1_MOFzd1	Fzd1	14362	Frizzled-1	no | no chan		0	ENOG410X
F6TTJ9	F6TTJ9_MTead3	Tead3	21678	Transcripti	no | no chan		0	ENOG410X
P11930	NUD19_MNudt19	Nudt19	110959	Acyl-coenzy	no | no chan		0	ENOG4111
P06745	G6PI_MOUGpi	Gpi	14751	Glucose-6-p	no | no chan		0	COG0166(g
Q6PG95	CRML_MCCramp1	Cramp1	57354	Protein cra	no | no chan		0	ENOG4110
Q3U6T2	Q3U6T2_MNaga	Naga	17939	Alpha-gala	no | no chan		0	ENOG410X
E9Q6D8	CO6_MOUC6	C6		Complemen	no | no chan		0	ENOG410Y
A0A3B2W7	A0A3B2W7Ppard	Ppard		Peroxisome	no | no chan		0	ENOG410X
A0AA74KT	A0AA74KTFhip2a	Fhip2a		FHF compl	no | no chan		0	ENOG410X
E9Q1I3	E9Q1I3_MZc3h12c	Zc3h12c	244871	Zinc finger	no | no chan		0	ENOG410Z
A0AA74KT	A0AA74KTZfp384	Zfp384		Zinc finger	no | no chan		0	COG5048(Z
A3KFM7	CHD6_MOChd6	Chd6	71389	Chromodor	no | no chan		0	COG0553(I
Q8K2W5	Q8K2W5_MEphx1	Ephx1	13849	Epoxide hy	no | no chan		0	COG0596(/
Q7TPW0	Q7TPW0_MRhot1	Rhot1	59040	Arht1 prote	no | up		0	ENOG410X
Q7TPW6	Q7TPW6_MSlc25a20	Slc25a20	57279	Solute carri	no | no chan		0	ENOG410X
A2AP31	A2AP31_MNdufb6	Ndufb6	230075	NADH deh	no | no chan		0	ENOG4111
A0A1L1SQ	A0A1L1SQPoc1a	Poc1a		POC1 centr	no | no chan		0	ENOG410X
A0A286YCl	A0A286YClCamk2g	Camk2g		Calcium/cc	no | no chan		0	ENOG410X
E9Q6D6	ZN277_MCZnf277	Znf277	246196	Zinc finger	no | no chan		0	ENOG410X
Q9D168	INT12_MCInts12	Ints12	71793	Integrator c	no | no chan		0	ENOG410X
P57746	VATD_MCAtp6v1d	Atp6v1d	73834	V-type prot	no | no chan		0	COG1394(I

A2AKQ8	I70A2_MO	Ift70a2	Ift70a2	620631	Intraflagell	no|no chan	0	ENOG410
Q99MY8	ASH1L_MC	Ash1l	Ash1l	192195	Histone-lys	no|no chan	0	COG2940(I
I6L9E1	I6L9E1_MOUSE	Ig-like domain-containing pr			Ig-like dom	no|no chan	0	ENOG4111
I6L9E2	I6L9E2_MOUSE	Ig-like domain-containing pr			Ig-like dom	no|no chan	0	ENOG4111
Q60960	IMA5_MO	Kpna1	Kpna1	16646	Importin su	no|no chan	0	COG5064(i
Q8VDT9	RM50_MO	Mrpl50	Mrpl50	28028	Large ribos	no|no chan	0	ENOG4111
Q60963	PAFA_MO	Pla2g7	Pla2g7	27226	Platelet-act	no|no chan	0	ENOG4111
Q3TL33	Q3TL33_M	Calu	Calu	12321	EF-hand dc	no|no chan	0	ENOG410
Q60967	PAPS1_MC	Papss1	Papss1	23971	Bifunctionc	no|no chan	0	COG2046(C
Q3UGZ4	Q3UGZ4_M	Sptbn2	Sptbn2	20743	Spectrin be	no|no chan	0	COG5069(V
Q9EQI8	RM46_MO	Mrpl46	Mrpl46	67308	Large ribos	no|no chan	0	ENOG4111
Q9EQI5	Q9EQI5_M	Ppbp	Ppbp	57349	Chemokine	no|up	0	ENOG4111
Q3T9H8	Q3T9H8_M	Smug1	Smug1	71726	Uracil-DN	no|no chan	0	ENOG410
E9PWE8	E9PWE8_M	Dpysl3	Dpysl3	22240	Dihydropyr	no|no chan	0	COG0044(I
E9PWE9	E9PWE9_M	Syk	Syk		Tyrosine-pr	no|no chan	0	COG0515(I
E9PYN2	E9PYN2_M	Zfp346	Zfp346		Zinc finger	no|no chan	0	ENOG4111
A2RS22	A2RS22_M	Coro1b	Coro1b	23789	Coronin	no|no chan	0	ENOG410
A2RS23	A2RS23_M	Pepd	Pepd	18624	Xaa-Pro di	no|no chan	0	COG0006(C
D0ESZ5	D0ESZ5_M	Serpine1	Serpine1	18787	Plasminoge	no|up	0	COG4826(T
Q3U944	Q3U944_M	Lman1	Lman1	70361	L-type lecti	no|no chan	0	ENOG410
Q3U417	Q3U417_M	Ppfibp1	Ppfibp1	67533	SAM domai	no|no chan	0	ENOG410
Q8BG60	TXNIP_MC	Txnip	Txnip	56338	Thioredoxi	no|no chan	0	ENOG411C
A2AJK8	A2AJK8_M	Ttc1	Ttc1		Tetratricop	no|no chan	0	ENOG4111
Q9D7M1	GID8_MO	Gid8	Gid8	76425	Glucose-inc	no|no chan	0	ENOG410
B7ZMR0	B7ZMR0_M	Mindy1	Mindy1	75007	Ubiquitin c	no|no chan	0	ENOG411C
D3Z3A8	D3Z3A8_M	Myo9a	Myo9a	270163	Myosin IXa	no|no chan	0	COG5022(C
Q3TH64	Q3TH64_M	Cyb5b	Cyb5b	66427	Cytochrom	no|no chan	0	COG5274(t
Q9D7M8	RPB4_MO	Polr2d	Polr2d	69241	DNA-direc	no|no chan	0	COG5250(p
A2AJK6	CHD7_MO	Chd7	Chd7	320790	Chromodor	no|no chan	0	COG0553(N
D3Z3A0	D3Z3A0_M	Ppp1r2	Ppp1r2		Protein phc	no|up	0	ENOG4111
P70355	P70355_MC	Mela	Mela	17276	Gag-Pol po	no|no chan	0	
Q3UW53	NIBA1_MC	Niban1	Niban1	63913	Protein Nib	no|no chan	0	ENOG411C
Q3UYD0	Q3UYD0_M	Utp14a	Utp14a	72554	U3 small nu	no|no chan	0	COG5644(C
Q921T2	TOIP1_MC	Tor1aip1	Tor1aip1	208263	Torsin-1A-i	no|no chan	0	ENOG4111
P70353	NFYC_MO	Nfyc	Nfyc	18046	Nuclear tra	no|no chan	0	COG5208(v
Q3UX10	TBAL3_MC	Tubal3	Tubal3	238463	Tubulin alp	yes|up	1	COG5023(c
Q05D44	IF2P_MOU	Eif5b	Eif5b	226982	Eukaryotic	no|no chan	0	COG0532(C
Q80V42	CBPM_MC	Cpm	Cpm	70574	Carboxypep	no|down	0	ENOG410
P24547	IMDH2_M	Impdh2	Impdh2	23918	Inosine-5'-n	no|no chan	0	COG0517(I
P24549	AL1A1_MC	Aldh1a1	Aldh1a1	11668	Aldehyde d	no|no chan	0	COG1012(I
Q9ES34	UBE3B_MC	Ube3b	Ube3b	117146	Ubiquitin-p	no|no chan	0	COG5021(c
Q5NCP6	Q5NCP6_M	Tspoap1	Tspoap1		TSPO assoc	no|no chan	0	ENOG410
Q9CZJ1	UTP11_MC	Utp11	Utp11	67205	Probable U	no|no chan	0	COG5223(U

O88712	CTBP1_MC	Ctbp1	Ctbp1	13016	C-terminal-	no | no chan	0	COG0111(C
Q00780	CO8A1_MC	Col8a1	Col8a1	12837	Collagen al	no | down	0	ENOG410Y
Q9CZJ2	HS12B_MC	Hspa12b	Hspa12b	72630	Heat shock	no | no chan	0	COG0443(F
Q91XX1	Q91XX1_M	Pcdhgc3	Pcdhgc3	93706	Protocadhe	no | no chan	0	ENOG410X
Q8BY89	CTL2_MOU	Slc44a2	Slc44a2	68682	Choline tra	no | no chan	0	ENOG410X
Q6NT99	DUS23_MC	Dusp23	Dusp23	68440	Dual specif	no | no chan	0	COG2453(t
Q8BY82	Q8BY82_M	Eif4enif1	Eif4enif1	74203	Eukaryotic	no | no chan	0	ENOG4111
A0A2R8VH	A0A2R8VH	Nup155	Nup155		Nucleopori	no | no chan	0	COG5308(r
Q3TEN9	Q3TEN9_M	Gk	Gk	14933	glycerol kin	no | no chan	0	COG0554(F
P15948	K1B22_MC	Klk1b22	Klk1b22	13646	Kallikrein 1	no | up	0	COG5640(N
G3UX16	G3UX16_M	Dusp7	Dusp7		protein-tyro	no | down	0	COG2453(C
D6RCG4	D6RCG4_M	Ube2w	Ube2w		N-terminal	no | down	0	COG5078(C
A0A0R4J18	A0A0R4J18	Slc35d2	Slc35d2	70484	Solute carri	no | no chan	0	COG5070(S
A0A068BD	A0A068BD	Rgl2	Rgl2	19732	Ral guanin	no | no chan	0	ENOG411C
Q80T21	ATL4_MOU	Adamtsl4	Adamtsl4	229595	ADAMTS-l	no | no chan	0	ENOG411C
P00375	DYR_MOU	Dhfr	Dhfr	13361	Dihydrofol	no | no chan	0	COG0262(c
Q3UJV6	Q3UJV6_M	Uqcr11	Uqcr11	66594	Cytochrom	no | no chan	0	ENOG410X
Q3UJV1	CCD61_MC	Ccdc61	Ccdc61	232933	Centrosom	no | no chan	0	ENOG410Y
A0A0N4SV	A0A0N4SV	Ndufa4	Ndufa4		Ndufa4, mi	no | no chan	0	ENOG4112
O08760	OGG1_MC	Ogg1	Ogg1	18294	N-glycosyla	no | no chan	0	COG0122(C
Q3THL7	Q3THL7_M	Vdac1	Vdac1	22333	Non-selecti	no | no chan	0	ENOG410Z
Q3THL5	Q3THL5_M	Pelo	Pelo	105083	Protein pel	no | no chan	0	COG1537(N
B7ZNA5	B7ZNA5_M	Phc3	Phc3	241915	Phc3 protei	no | no chan	0	ENOG4111
P58929	GMEB2_M	Gmeb2	Gmeb2	229004	Glucocortic	no | no chan	0	ENOG410Z
Q3V1L6	MTMRB_M	Mtmr11	Mtmr11	194126	Myotubula	no | no chan	0	ENOG410X
Q8R151	ZNFX1_MC	Znfx1	Znfx1	98999	NFX1-type	no | no chan	0	COG1112(T
Q6A085	ZN629_MC	Znf629	Znf629	320683	Zinc finger	no | no chan	0	COG5048(A
Q8K4Q8	COL12_MC	Colec12	Colec12	140792	Collectin-1	no | no chan	0	ENOG411C
P49135	ERCC3_MC	Ercc3	Ercc3	13872	General tra	no | no chan	0	COG1061(t
Q8C7I4	Q8C7I4_M	Apol9b	Apol9b	71898	Apolipopro	no | up	0	ENOG410Y
Q8K4Q6	NEIL1_MC	Neil1	Neil1	72774	Endonucle	no | no chan	0	ENOG410Z
C4TGC3	C4TGC3_M	Ankrd27	Ankrd27	245886	VPS9-ankyr	no | no chan	0	COG0666(I
P35456	UPAR_MO	Plaur	Plaur	18793	Urokinase p	no | up	0	ENOG410Z
F6SK53	F6SK53_M	Ncor2	Ncor2		Nuclear rec	no | no chan	0	ENOG410Y
Q8CIW5	PEO1_MO	Twnk	Twnk	226153	Twinkle mt	no | no chan	0	ENOG410X
Q91W53	GOGA7_M	Golga7	Golga7	57437	Golgin sub	no | no chan	0	ENOG4111
Q63870	CO7A1_MC	Col7a1	Col7a1	12836	Collagen al	no | no chan	0	ENOG410X
Q3UA17	Q3UA17_M	Mtch2	Mtch2	56428	Mitochond	no | no chan	0	ENOG410X
Q3UA16	SPC25_MO	Spc25	Spc25	66442	Kinetochor	no | no chan	0	ENOG4111
Q9CQB4	Q9CQB4_M	Uqcrb	Uqcrb	67530	Cytochrom	no | no chan	0	ENOG4111
Q8K3P5	CNOT6_M	Cnot6	Cnot6	104625	CCR4-NOT	no | no chan	0	COG5239(C
H3BL77	H3BL77_M	Gpank1	Gpank1		G patch do	no | up	0	ENOG410Z
E9Q2M9	WDFY4_M	Wdfy4	Wdfy4	545030	WD repeat-	no | no chan	0	ENOG410X

Q3TBC0	Q3TBC0_M	Gvin3	Gvin3	434223	Interferon-i	no|no chan	0 ENOG410Y
A2A4M9	A2A4M9_M	Tmem106a	Tmem106a		Transmemb	no|up	0 ENOG410X
Q3V0F0	RIMB3_MO	Rimbp3	Rimbp3	239731	RIMS-bindi	no|no chan	0 ENOG410X
E9Q0N0	E9Q0N0_M	Itsn1	Itsn1	16443	Intersectin	no|no chan	0 COG5422(N
Q9WVR4	FXR2_MOU	Fxr2	Fxr2	23879	RNA-bindi	no|up	0 ENOG410Z
Q4KL76	Q4KL76_M	Hspe1	Hspe1	15528	10 kDa hea	no|no chan	0 COG0234(E
B1APX2	B1APX2_M	5031439G07	5031439G07	223739	RIKEN cD	no|no chan	0 ENOG410Z
Q9CQB5	CISD2_MO	Cisd2	Cisd2	67006	CDGSH irc	no|no chan	0 ENOG4112
Q9QZN4	FBX6_MOU	Fbxo6	Fbxo6	50762	F-box only	no|no chan	0 ENOG4111
Q8BYC6	TAOK3_M	Taok3	Taok3	330177	Serine/thre	no|no chan	0 ENOG410Y
P10810	CD14_MOU	Cd14	Cd14	12475	Monocyte d	no|no chan	0 ENOG4111
Q9DBB5	IF4E3_MO	Eif4e3	Eif4e3	66892	Eukaryotic	no|up	0 COG5053(N
Q9D1B9	RM28_MO	Mrpl28	Mrpl28	68611	Large ribos	no|no chan	0 ENOG410X
Q3TIJ1	Q3TIJ1_MO	Cops4	Cops4	26891	COP9 signc	no|up	0 ENOG410X
Q63880	EST3A_MO	Ces3a	Ces3a	382053	Carboxylest	yes|down	1 COG2272(C
Q9WTL7	LYPA2_MO	Lypla2	Lypla2	26394	Acyl-protei	no|no chan	0 COG0400(C
P54731	FAF1_MOU	Faf1	Faf1	14084	FAS-associ	no|no chan	0 ENOG410Z
A0A0A6YW	A0A0A6YW	Enah	Enah	13800	ENAH acti	no|no chan	0 ENOG411C
E9Q166	E9Q166_M	Atad2b	Atad2b		ATPase fan	no|no chan	0 COG0464(g
B7ZMR9	B7ZMR9_M	Zfp800	Zfp800	627049	Zinc finger	no|no chan	0 COG5048(F
Q8K1N2	PHLB2_MO	Phldb2	Phldb2	208177	Pleckstrin h	no|no chan	0 ENOG4111
Q64471	GSTT1_MO	Gstt1	Gstt1	14871	Glutathion	no|down	0 COG0625(I
P11499	HS90B_MO	Hsp90ab1	Hsp90ab1	15516	Heat shock	no|no chan	0 COG0326(C
Q8CJ53	CIP4_MOU	Trip10	Trip10	106628	Cdc42-inter	no|no chan	0 ENOG410Z
Q8BTI7	ANR52_MO	Ankrd52	Ankrd52	237615	Serine/thre	no|no chan	0 COG0666(t
D3Z7E5	D3Z7E5_M	Gsk3a	Gsk3a		[tau proteir	no|no chan	0 COG0515(r
D6RIL7	D6RIL7_M	Prdm15	Prdm15		PR domain	no|no chan	0 COG5048(F
A2A5Y4	A2A5Y4_M	Kansl1	Kansl1	76719	KAT8 regul	no|no chan	0 ENOG410Z
A0A0F6PZ	A0A0F6PZ	ATP6	ATP6		ATP syntha	no|no chan	0 COG0356(i
Q3UW57	Q3UW57_M	Cd209d	Cd209d	170779	C-type lecti	no|down	0 ENOG4112
Q8K0V4	CNOT3_M	Cnot3	Cnot3	232791	CCR4-NOT	no|no chan	0 COG5665(C
Q4ACU6	SHAN3_MO	Shank3	Shank3	58234	SH3 and mu	no|no chan	0 COG0666(V
C7C6B2	C7C6B2_M	Elk3	Elk3	13713	Elk3 transc	no|no chan	0 ENOG410Z
A0ABA7IW	A0ABA7IW04_MOUSE	Heterogeneous	nuclear	Hnrnpa2b1		no|no chan	0 COG0724(F
Q3TCC3	Q3TCC3_M	Cytip	Cytip	227929	PDZ domai	no|up	0 ENOG4111
P48774	GSTM5_MO	Gstm5	Gstm5	14866	Glutathion	no|no chan	0 ENOG411C
Q3UDS4	Q3UDS4_M	Sqor	Sqor	59010	FAD/NAD	no|no chan	0 COG0446(i
P48771	CX7A2_MO	Cox7a2	Cox7a2	12866	Cytochrom	no|no chan	0 ENOG4112
A0A087WR	A0A087WR	Adhfe1	Adhfe1		Hydroxyaci	no|down	0 COG1454(c
Q6P572	Q6P572_M	Mtmr2	Mtmr2	77116	Phosphatid	no|no chan	0 ENOG410X
B2RXZ9	B2RXZ9_M	Mylk2	Mylk2	228785	Myosin ligh	no|no chan	0 ENOG410X
Q9WUP4	SR5A3_MO	Srd5a3	Srd5a3	57357	Polyprenal	no|no chan	0 ENOG4111
E0CXB5	E0CXB5_M	Pttg1ip	Pttg1ip		Pituitary tu	no|up	0 ENOG4111

Q8BM75	ARI5B_MC	Arid5b	Arid5b	71371	AT-rich int	no | no chan	0	ENOG411(
Q545I6	Q545I6_M(	Ctsz	Ctsz	64138	cathepsin X	no | up	0	COG4870(T
Q8BM72	HSP13_MC	Hspa13	Hspa13	110920	Heat shock	no | no chan	0	COG0443(F
Q8HWB2	Q8HWB2_I	H2-Q4	H2-Q4	15015	Histocompo	no | up	0	ENOG4111
Q5SWP3	NACAD_M	Nacad	Nacad	192950	NAC-alpha	no | no chan	0	COG1308(r
Q80XH7	Q80XH7_M	Pole	Pole	18973	DNA polym	no | no chan	0	COG0417(C
Q9JJW5	MYOZ2_M	Myoz2	Myoz2	59006	Myozenin-2	no | up	0	ENOG410Z
Q3TTV6	Q3TTV6_M	Tfip11	Tfip11	54723	Tuftelin-int	no | no chan	0	ENOG410X
G3X9C8	G3X9C8_M	Lix1l	Lix1l	280411	Lix1-like	no | no chan	0	ENOG410X
Q9EST3	4ET_MOUS	Eif4enif1	Eif4enif1	74203	Eukaryotic	no | no chan	0	ENOG4111
Q9EST4	PSMG2_M(	Psmg2	Psmg2	107047	Proteasome	no | no chan	0	ENOG410X
A0A0M5KS	A0A0M5KS39_MOUSE	B2 18-5 anti-human bı			B2 18-5 ant	no | no chan	0	ENOG4111
P34884	MIF_MOU	Mif	Mif	17319	Macrophag	no | up	0	ENOG4112
Q9CRB1	Q9CRB1_M	Lgals7	Lgals7	16858	Galectin	no | no chan	0	ENOG4111
Q9CRB0	SNX24_MC	Snx24	Snx24	69226	Sorting nex	no | no chan	0	ENOG4112
B2RX77	B2RX77_M	Polr3c	Polr3c	74414	DNA-direc	no | no chan	0	ENOG410X
Q9CRB2	NHP2_MO	Nhp2	Nhp2	52530	H/ACA rib	no | no chan	0	COG1358(f
Q9CRB9	MIC19_MC	Chchd3	Chchd3	66075	MICOS con	no | no chan	0	ENOG4112
Q9D0M2	CDCA7_M	Cdca7	Cdca7	66953	Cell divisio	no | down	0	ENOG410Z
P33215	NEDD1_M	Nedd1	Nedd1	17997	Protein NE	no | no chan	0	ENOG410Z
F7AL76	F7AL76_M	Chd8	Chd8		Chromodor	no | up	0	COG0553(S
A0A0A0M(	A0A0A0M(	C1d	C1d		Nuclear nu	no | no chan	0	ENOG4111
A0A0A0M(	A0A0A0M(	Gtdc1	Gtdc1		tRNA-queu	yes | down	1	ENOG410Z
A0A0A0M(	A0A0A0M(	Srebf1	Srebf1		Sterol regul	yes | down	1	ENOG410X
P16301	LCAT_MO	Lcat	Lcat	16816	Phosphatid	no | up	0	ENOG410Y
Q61699	HS105_MO	Hsph1	Hsph1	15505	Heat shock	no | no chan	0	COG0443(c
Q3TLP5	ECHD2_M	Echdc2	Echdc2	52430	Enoyl-CoA	no | no chan	0	COG1024(N
Q9D927	Q9D927_M	1810009N0	1810009N0	69099	Uncharacte	no | no chan	0	COG0424(N
Q8VIG1	REST_MO	Rest	Rest	19712	RE1-silenci	no | no chan	0	COG5048(C
Q6PAT0	ADAT3_M	Adat3	Adat3	100113398	Probable in	no | no chan	0	COG0590(F
D3Z2B4	D3Z2B4_M	Xpr1	Xpr1		Solute carri	no | no chan	0	COG5409(X
Q9Z120	TRMB_MC	Mettl1	Mettl1	17299	tRNA (guar	no | no chan	0	COG0220(C
A0A3B2W3	A0A3B2W3	Ift140	Ift140		Intraflagell	no | no chan	0	ENOG410X
Q924C5	ALPK3_M(	Alpk3	Alpk3	116904	Alpha-prot	no | no chan	0	ENOG410Y
Q924C1	XPO5_MO	Xpo5	Xpo5	72322	Exportin-5	no | no chan	0	COG5101(e
Q3UHA3	SPTCS_MC	Spg11	Spg11	214585	Spatacsin	no | no chan	0	ENOG410X
P07607	TYSY_MO	Tyms	Tyms	22171	Thymidylat	no | no chan	0	COG0207(F
Q9QXX8	NUFP1_M(	Nufip1	Nufip1	27275	FMR1-inter	no | no chan	0	ENOG4111
Q69Z38	PEAK1_M(	Peak1	Peak1	244895	Inactive tyr	no | no chan	0	ENOG411(
Q0P648	Q0P648_M	Tbc1d20	Tbc1d20	67231	TBC1 domc	no | no chan	0	ENOG410X
Q3TST4	Q3TST4_M	Grk5	Grk5	14773	G protein-c	no | down	0	ENOG410Y
Q6PFH3	DCA15_M(	Dcaf15	Dcaf15	212123	DDB1- and	no | no chan	0	ENOG410Z
P51655	GPC4_MO	Gpc4	Gpc4	14735	Glypican-4	no | no chan	0	ENOG410X

O54865	GCYB1_M(	Gucy1b1	Gucy1b1	54195	Guanylate (	no | no chan	0	COG2114(T
A0JLN2	A0JLN2_M	Ccne1	Ccne1	12447	Ccne1 prote	no | no chan	0	ENOG410X
Q9DC23	DJC10_MC	Dnajc10	Dnajc10	66861	DnaJ homo	no | no chan	0	COG2214(I
P47199	QOR_MOU	Cryz	Cryz	12972	Quinone ox	no | no chan	0	COG0604(r
E9PYI1	ZN568_MC	Znf568	Znf568	243905	Zinc finger	no | no chan	0	COG5048(t
Q9R0E1	PLOD3_M(	Plod3	Plod3	26433	Multifuncti	no | no chan	0	ENOG410Y
Q9R0E2	PLOD1_M(	Plod1	Plod1	18822	Procollager	no | no chan	0	ENOG410Y
Q69ZX6	MOR2A_M	Morc2a	Morc2a	74522	ATPase MC	no | no chan	0	ENOG411C
A0A0G2JG	A0A0G2JG	Ap1ar	Ap1ar	211556	Adaptor-rel	no | up	0	ENOG4111
Q8BLZ1	Q8BLZ1_M	Acad12	Acad12	338350	Characte	yes | up	1	ENOG410X
A2AH25	A2AH25_M	Arhgap1	Arhgap1	228359	Rho GTPas	no | no chan	0	ENOG410X
A2AH22	AMRA1_M	Ambra1	Ambra1	228361	Activating	no | no chan	0	ENOG410Z
Q69ZX8	ABLM3_M(	Ablim3	Ablim3	319713	Actin-bindi	no | no chan	0	ENOG410X
Q9Z0G0	GIPC1_MC	Gipc1	Gipc1	67903	PDZ domai	no | no chan	0	ENOG410X
Q3TMV8	Q3TMV8_MOUSE	DCN1-like prot		114893	DCN1-like	no | no chan	0	ENOG410X
P49962	SRP09_MO	Srp9	Srp9	27058	Signal reco	no | no chan	0	ENOG4112
Q3TMV7	PYRD1_M(	Pyroxd1	Pyroxd1	232491	Pyridine nu	no | no chan	0	COG0446(p
P24288	BCAT1_M(	Bcat1	Bcat1	12035	Branched-c	no | no chan	0	COG0115(b
Q9DCM0	ETHE1_M(	Ethe1	Ethe1	66071	Persulfide d	no | no chan	0	COG0491(N
Q61733	RT31_MOU	Mrps31	Mrps31	57312	Small ribos	no | no chan	0	ENOG4111
Q61730	IL1AP_MO	Il1rap	Il1rap	16180	Interleukin-	no | no chan	0	ENOG410Z
Q8R1G6	PDLI2_MC	Pdlim2	Pdlim2	213019	PDZ and L	no | no chan	0	ENOG410X
Q80WW9	DDRGK_M	Ddrgk1	Ddrgk1	77006	DDRGK do	no | no chan	0	ENOG4111
Q9DCM7	NACC2_M(	Nacc2	Nacc2	67991	Nucleus acc	no | no chan	0	COG5048(S
B2RWR1	B2RWR1_M	Pcdh20	Pcdh20	219257	Protocadhe	no | no chan	0	ENOG411C
B1AXP6	TOM5_MC	Tomm5	Tomm5	68512	Mitochondr	no | no chan	0	ENOG4111
P52293	IMA1_MOU	Kpna2	Kpna2	16647	Importin su	no | no chan	0	COG5064(r
B2RRM5	B2RRM5_M	Tmed1	Tmed1	17083	Transmemb	no | no chan	0	ENOG410Z
E9QLN4	E9QLN4_M	Trim39	Trim39	79263	Tripartite n	no | down	0	ENOG410Y
A0A494BBI	A0A494BBI	Efemp2	Efemp2		Epidermal (	no | no chan	0	ENOG410Z
Q10470	MGAT3_M	Mgat3	Mgat3	17309	Beta-1,4-mc	no | up	0	ENOG410Y
E9QLN8	E9QLN8_M	Rnaseh1	Rnaseh1	19819	Ribonuclea	no | no chan	0	COG0328(r
Q8K1R3	PNPT1_M(	Pnpt1	Pnpt1	71701	Polyribonuc	no | no chan	0	COG1185(I
E9Q4R1	E9Q4R1_M	Eeig2	Eeig2	329739	EEIG fami	no | no chan	0	ENOG411C
A0A2Y9CZ	A0A2Y9CZ	Ddx11	Ddx11	320209	DEAD/H b	no | no chan	0	COG1199(C
P00493	HPRT_MO	Hprt1	Hprt1	15452	Hypoxanth	no | no chan	0	COG0634(h
O88508	DNM3A_M	Dnmt3a	Dnmt3a	13435	DNA (cytos	no | no chan	0	ENOG410X
O88509	DNM3B_M	Dnmt3b	Dnmt3b	13436	DNA (cytos	no | no chan	0	ENOG410X
A0A1Y7VM	A0A1Y7VM	Fktn	Fktn	246179	Ribitol-5-pl	yes | down	1	ENOG411C
Q4FK49	Q4FK49_M	Ppa1	Ppa1	67895	Inorganic p	no | no chan	0	COG0221(I
C3VPR6	NLRC5_M(	Nlrc5	Nlrc5	434341	Protein NL	no | up	0	ENOG4112
A0A0R4J18	A0A0R4J18	Xrcc6	Xrcc6	14375	X-ray repai	no | no chan	0	ENOG410X
Q3TUI3	Q3TUI3_M	Brd3	Brd3	67382	Bromodom	no | no chan	0	COG5076(C

Q3TWH4	Q3TWH4_I	Rmc1	Rmc1	76482	Uncharacte	no | no chan	0	ENOG410Y
P17426	AP2A1_MC	Ap2a1	Ap2a1	11771	AP-2 compl	no | no chan	0	ENOG410X
P17427	AP2A2_MC	Ap2a2	Ap2a2	11772	AP-2 compl	no | no chan	0	ENOG410X
Q9DAU1	CNPY3_MC	Cnpy3	Cnpy3	72029	Protein can	no | no chan	0	ENOG4111
A0A3B2W7	A0A3B2W7	Taf4b	Taf4b	72504	TATA-box	no | no chan	0	ENOG410X
V9GWU2	V9GWU2_I	Tcf12	Tcf12		Transcripti	no | no chan	0	ENOG410X
Q3UII8	Q3UII8_MC	Mta3	Mta3	116871	Metastasis	no | no chan	0	ENOG410X
Q62426	CYTB_MO	Cstb	Cstb	13014	Cystatin-B	no | no chan	0	ENOG4112
Q8C6B2	RTKN_MC	Rtkn	Rtkn	20166	Rhotekin	no | no chan	0	ENOG410Z
A2A8W8	A2A8W8_M	Mknk1	Mknk1	17346	MAP kinase	no | no chan	0	ENOG410X
P54923	ADPRH_M	Adprh	Adprh	11544	ADP-ribosy	no | no chan	0	ENOG410X
O08599	STXB1_MC	Stxbp1	Stxbp1	20910	Syntaxin-bi	no | no chan	0	COG5158(F
Q99N16	CP4F3_MC	Cyp4f3	Cyp4f3	72054	Cytochrom	no | down	0	COG2124(C
Q8BGF3	DAA10_MC	Dnaaf10	Dnaaf10	103784	Dynein axo	no | no chan	0	ENOG410X
Q8BGF0	Q8BGF0_M	AW551984	AW551984	244810	Expressed s	no | no chan	0	COG2304(v
Q8BGF6	ELMD2_M	Elmod2	Elmod2	244548	ELMO dom	no | no chan	0	ENOG410X
Q4V9X1	Q4V9X1_M	Wdr12	Wdr12	57750	Ribosome b	no | no chan	0	ENOG410X
A0A0G2JF(	A0A0G2JF(	Nbeal2	Nbeal2		Neurobeacl	no | no chan	0	ENOG410X
Q8CH02	SUGP1_MC	Sugp1	Sugp1	70616	SURP and (	no | no chan	0	ENOG410Z
A2AF47	DOC11_MC	Dock11	Dock11	75974	Dedicator c	no | no chan	0	ENOG410X
P62774	MTPN_MC	Mtpn	Mtpn	14489	Myotrophin	no | no chan	0	ENOG4111
D3Z733	D3Z733_M	Rad18	Rad18		RING-type	no | no chan	0	COG5432(F
J3QN89	J3QN89_M	Aamp	Aamp	227290	Angio-asso	no | no chan	0	ENOG410X
Q8K1A0	METL5_MC	Mettl5	Mettl5	75422	rRNA N(6)-	no | no chan	0	COG2263(M
Q8R3U1	PLAT3_MC	Plaat3	Plaat3	225845	Phospholip	no | up	0	ENOG4111
Q8BGT7	SPF30_MO	Smndc1	Smndc1	76479	Survival of	no | no chan	0	ENOG411(
A2AJH3	A2AJH3_M	Nmt2	Nmt2	18108	Glycylpepti	no | no chan	0	COG5092(e
Q4FJL2	Q4FJL2_MC	Rtn1	Rtn1	104001	Reticulon	no | up	0	ENOG410X
Q02257	PLAK_MO	Jup	Jup	16480	Junction pl	no | no chan	0	COG0035(s
P42128	FOXK1_M	Foxk1	Foxk1	17425	Forkhead b	no | no chan	0	COG5025(F
P97313	PRKDC_M	Prkdc	Prkdc	19090	DNA-deper	no | no chan	0	COG5032(F
P97310	MCM2_MC	Mcm2	Mcm2	17216	DNA replic	no | no chan	0	COG1241(F
P97311	MCM6_MC	Mcm6	Mcm6	17219	DNA replic	no | no chan	0	COG1241(F
P97315	CSRP1_MC	Csrp1	Csrp1	13007	Cysteine an	no | no chan	0	ENOG4111
A2AJP9	A2AJP9_MC	Pdp1	Pdp1	381511	[Pyruvate d	no | no chan	0	COG0631(T
Q3TVI8	PBIP1_MO	Pbxip1	Pbxip1	229534	Pre-B-cell l	no | up	0	ENOG410Y
A2AJP5	A2AJP5_MC	Tmem67	Tmem67	329795	Transmemb	no | no chan	0	ENOG410X
Q3U4T8	Q3U4T8_M	Mcm7	Mcm7	17220	DNA replic	no | no chan	0	COG1241(M
Q3TLM7	Q3TLM7_M	Snx16	Snx16	74718	Sorting nex	no | up	0	ENOG410Z
Q5SYD0	MYO1D_M	Myo1d	Myo1d	338367	Unconventi	no | no chan	0	COG5022(c
E9Q6L5	E9Q6L5_M	Atrip	Atrip		ATR intera	no | no chan	0	ENOG411(
E9Q6L4	E9Q6L4_M	Tbc1d1	Tbc1d1	57915	TBC1 domc	no | up	0	ENOG410Y
A0A9S4GQ	A0A9S4GQ	Mcub	Mcub		Calcium un	no | no chan	0	ENOG410Y

Q8K039	K1143_MOUSE	Uncharacterized p		66202	Uncharacte	no | no chan	0	ENOG4112
F6S5X0	F6S5X0_M(	Mrnip	Mrnip		MRN comp	yes | down	1	ENOG4112
A9C471	A9C471_M(	Naip2	Naip2		NLR family	no | no chan	0	ENOG410Y
Q9CWU9	NUP37_M(	Nup37	Nup37	69736	Nucleopori	no | no chan	0	ENOG410X
A0A1Y7VL	A0A1Y7VL	Atp5mj	Atp5mj	70257	ATP syntha	no | no chan	0	ENOG4111
Q9CR09	UFC1_MO	Ufc1	Ufc1	66155	Ubiquitin-f	no | no chan	0	ENOG410Z
A7ISP9	A7ISP9_M(	Eml4	Eml4	78798	EMAP-like	no | no chan	0	COG2319(p
F8VQG7	F8VQG7_M	N4bp2	N4bp2	333789	NEDD4 bi	no | no chan	0	ENOG4110
Q9CR02	TMA16_M(	Tma16	Tma16	66282	Translation	no | no chan	0	ENOG4111
Q9CR00	PSMD9_M(	Psmd9	Psmd9	67151	26S proteas	no | no chan	0	COG0265(c
Q9CWU4	CA052_MOUSE	UPF0690 protein (		66421	UPF0690 p	no | no chan	0	ENOG4111
Q9CWU6	UQCC1_M	Uqcc1	Uqcc1	56046	Ubiquinol-(	no | no chan	0	ENOG4111
P62340	TBPL1_MC	Tbpl1	Tbpl1	237336	TATA box-	no | no chan	0	COG2101(C
P62342	SELT_MOU	Selenot	Selenot	69227	Thioredoxi	no | no chan	0	ENOG4111
A0A5F8MP	A0A5F8MP	Xirp2	Xirp2		Xin actin-b	no | no chan	0	ENOG410Z
E9Q718	E9Q718_M	Plod2	Plod2	26432	procollagen	no | no chan	0	ENOG410Y
P56480	ATPB_MO	Atp5f1b	Atp5f1b	11947	ATP syntha	no | no chan	0	COG0055(F
A0A0J9YV8	A0A0J9YV8	Bcl7b	Bcl7b		B cell CLL/	no | no chan	0	ENOG4111
B7ZN11	B7ZN11_M	Pigw	Pigw	70325	Phosphatid	no | no chan	0	COG5062(F
A0A0G2JH	A0A0G2JH	Ints1	Ints1	68510	Integrator c	no | no chan	0	ENOG410X
Q3UMM7	Q3UMM7_	Adh7	Adh7	11529	Enoyl redu	no | down	0	COG1062(c
A0A0R4J1C	A0A0R4J1C	Mfap3	Mfap3	216760	Microfibrill	no | no chan	0	ENOG410X
A0A0R4J1C	A0A0R4J1C	4933427D1	4933427D1	74477	RIKEN cD	no | down	0	ENOG410Z
A0A0R4J1C	A0A0R4J1C	Ezh1	Ezh1	14055	[histone H3	no | up	0	COG2940(c
P52196	THTR_MO	Tst	Tst	22117	Thiosulfate	no | down	0	COG2897(r
B9EHD0	B9EHD0_M	Lrrc25	Lrrc25	211228	Leucine ricl	no | no chan	0	ENOG4111
Q9D2G2	ODO2_MC	Dlst	Dlst	78920	Dihydrolip	no | no chan	0	COG0508(I
A0A0R4J1C	A0A0R4J1C	Cd68	Cd68	12514	CD68 antig	no | no chan	0	ENOG410X
Q9JI44	DMAP1_M	Dmap1	Dmap1	66233	DNA meth	no | no chan	0	ENOG410X
Q80TY5	VP13B_MC	Vps13b	Vps13b	666173	Intermemb	no | no chan	0	COG5043(V
Q0VBD2	MCM10_M	Mcm10	Mcm10	70024	Protein MC	no | no chan	0	ENOG4110
Q3TXW9	Q3TXW9_M	Sin3b	Sin3b	20467	Histone dec	no | no chan	0	COG5602(F
E9QAD3	E9QAD3_M	Mtf2	Mtf2		Metal respo	no | down	0	ENOG410X
A0A0J9YUI	A0A0J9YUI	Nup205	Nup205		Nucleopori	no | no chan	0	ENOG410X
Q91WE4	CR032_MOUSE	UPF0729 protein (		407819	UPF0729 p	no | no chan	0	ENOG410Y
Q91WE2	PIP30_MO	Psme3ip1	Psme3ip1	102122	PSME3-int	no | no chan	0	ENOG4111
E9QAD4	E9QAD4_M	Ccdc93	Ccdc93	70829	Coiled-coil	no | no chan	0	ENOG410X
Q3V307	Q3V307_M	Pigs	Pigs	276846	GPI transa	no | no chan	0	ENOG410X
Q3UXU8	Q3UXU8_M	Stn1	Stn1	108689	CST compl	no | no chan	0	COG5235(F
A0A0J9YUI	A0A0J9YUI	Hmgb1	Hmgb1		High mobil	no | no chan	0	COG5648(r
Q3URA7	Q3URA7_M	Ccp110	Ccp110	101565	Centriolar c	no | down	0	ENOG410X
B6ZHD0	B6ZHD0_M	Epb41l2	Epb41l2	13822	Erythrocyte	no | no chan	0	ENOG410Y
P63166	SUMO1_M	Sumo1	Sumo1	22218	Small ubiq	no | no chan	0	COG5227(t

Q810A7	DDX42_M	Ddx42	Ddx42	72047	ATP-depen	no|no chan	0	ENOG410
A0A498U6I	A0A498U6I	H2-Eb1	H2-Eb1		Histocomp	no|up	0	ENOG410
Q3TLS3	GDPP1_M	Gdpgp1	Gdpgp1	269952	GDP-D-glu	no|no chan	0	ENOG4111
Q810A3	TTC9C_M	Ttc9c	Ttc9c	70387	Tetratricop	no|no chan	0	ENOG4111
Q9JK24	P2R3C_MC	Ppp2r3c	Ppp2r3c	59032	Serine/thre	no|no chan	0	ENOG410
P63168	DYL1_MO	Dynll1	Dynll1	56455	Dynein ligh	no|no chan	0	ENOG4111
Q9Z2F2	OASL2_M	Oasl2	Oasl2	23962	2'-5'-oligoa	no|up	0	COG5272(
Q3TZU7	Q3TZU7_M	Snx9	Snx9	66616	Sorting nex	no|no chan	0	ENOG410
Q3TZU6	Q3TZU6_M	Rhoj	Rhoj	80837	Rho-related	no|down	0	COG1100(r
Q8BNK1	Q8BNK1_M	B230354K1	B230354K1	105246278	Uncharacte	no|no chan	0	ENOG410
Q9D8U2	TM41A_M	Tmem41a	Tmem41a	66664	Transmemb	no|no chan	0	COG0398(t
A0A494B98	A0A494B98	Btbd9	Btbd9	224671	BTB domai	no|no chan	0	ENOG410
Q9D8U6	MCEM1_M	Mcemp1	Mcemp1	69189	Mast cell-e	no|no chan	0	ENOG4111
Q5PRE5	PRSR1_MC	Proser1	Proser1	212127	Proline and	no|no chan	0	ENOG410
B1ASD4	B1ASD4_M	Mob3c	Mob3c	100465	MOB1, Mp	no|no chan	0	ENOG410
P17225	PTBP1_MC	Ptbp1	Ptbp1	19205	Polypyrimi	no|no chan	0	ENOG410
B1ASD1	B1ASD1_M	Zfx	Zfx	22764	Zinc finger	no|no chan	0	COG5048(
A0A3Q4EC	A0A3Q4EC	Mtch1	Mtch1		Mitochond	no|no chan	0	ENOG410
Q9D8N2	DEN10_M	Dennd10	Dennd10	67894	DENN don	no|no chan	0	ENOG410
A2A464	A2A464_M	Slc9a8	Slc9a8	77031	Sodium/hy	no|no chan	0	COG0025(r
H3BKL1	H3BKL1_M	Tirap	Tirap	117149	Toll/interl	no|no chan	0	ENOG4111
G3UWS7	G3UWS7_M	Cchcr1	Cchcr1	240084	Coiled-coil	no|no chan	0	ENOG410
Q9QWK4	CD5L_MO	Cd5l	Cd5l	11801	CD5 antige	no|up	0	ENOG411
B2RQ14	B2RQ14_M	Inpp5f	Inpp5f	101490	Inositol pol	no|no chan	0	COG5329(
P11688	ITA5_MOU	Itga5	Itga5	16402	Integrin alp	no|no chan	0	ENOG410
O70400	PDLI1_MC	Pdlim1	Pdlim1	54132	PDZ and L	no|no chan	0	ENOG410
E9PVC5	E9PVC5_M	Eif4g1	Eif4g1		Eukaryotic	no|no chan	0	ENOG410
Q3UHJ0	AAK1_MO	Aak1	Aak1	269774	AP2-associ	no|no chan	0	ENOG410
Q80VP1	EPN1_MO	Epn1	Epn1	13854	Epsin-1	no|no chan	0	ENOG410
A2AMM0	CAVN4_M	Cavin4	Cavin4	68016	Caveolae-a	no|up	0	ENOG411
Q60737	CSK21_MC	Csnk2a1	Csnk2a1	12995	Casein kina	no|no chan	0	ENOG410
F6T8X6	F6T8X6_M	Sec16a	Sec16a		SEC16 hom	no|up	0	ENOG4111
Q3UQA7	SELH_MO	Selenoh	Selenoh	72657	Selenoprote	no|no chan	0	ENOG410
A0A1B0GR	A0A1B0GR	Stx3	Stx3		Syntaxin 3	no|no chan	0	COG5074(
Q3TJT6	Q3TJT6_M	Sco1	Sco1	52892	Thioredoxi	no|down	0	COG1999(
Q8C9V1	TB10C_MC	Tbc1d10c	Tbc1d10c	108995	Carabin	no|down	0	ENOG410
Q9D6K8	FUND2_M	Fundc2	Fundc2	67391	FUN14 don	no|no chan	0	ENOG4111
P55097	CATK_MC	Ctsk	Ctsk	13038	Cathepsin I	no|no chan	0	COG4870(c
P55096	ABCD3_M	Abcd3	Abcd3	19299	ATP-bindir	no|no chan	0	COG4178(c
Q9D6K5	SYJ2B_MO	Synj2bp	Synj2bp	24071; 1059	Synaptojan	no|no chan	0	ENOG4111
Q3TR30	Q3TR30_M	4931414P19	4931414P19	74359	Regulator c	no|up	0	ENOG410
Q8K2Q9	SHOT1_M	Shtn1	Shtn1	71653	Shootin-1	no|up	0	ENOG4111
Q8BGA7	INT15_MC	Ints15	Ints15	231868	Integrator c	no|no chan	0	ENOG411

A0AA74KT	A0AA74KT	Tarbp2	Tarbp2		RISC-loadi	no	no chan	0	ENOG410
Q6P9N1	HYCCI_M	Hycc1	Hycc1	84652	Hyccin	no	up	0	ENOG410
Q8K2Q0	COMD9_M	Commd9	Commd9	76501	COMM dor	no	no chan	0	ENOG4111
E9Q1K1	E9Q1K1_M	Cfap97	Cfap97		Cilia- and f	no	no chan	0	ENOG4112
E9Q1K0	E9Q1K0_M	Snx25	Snx25		Sorting nex	no	no chan	0	ENOG410
Q8CB55	Q8CB55_M	Nav1	Nav1	215690	AAA+ ATF	no	no chan	0	ENOG410
E9Q7S1	E9Q7S1_M	Zfp106	Zfp106		Zinc finger	no	no chan	0	ENOG410
A0A494BA	A0A494BA	Slc29a2	Slc29a2		Solute carri	no	no chan	0	ENOG410
B1AUV4	B1AUV4_M	Pramel18	Pramel18		PRAME lik	no	no chan	0	ENOG410
Q69ZN6	GNPTA_M	Gnptab	Gnptab	432486	N-acetylglu	no	no chan	0	ENOG410
A0A1D5RL	A0A1D5RL	Trrap	Trrap		Transformo	no	no chan	0	COG5032(I
Q3TL58	Q3TL58_M	Skp1	Skp1	21402	S-phase kin	no	no chan	0	COG5201(u
Q6NVE8	WDR44_M	Wdr44	Wdr44	72404	WD repeat-	no	no chan	0	ENOG410
Q8VDZ4	ZDHC5_M	Zdhhc5	Zdhhc5	228136	Palmitoyltr	no	no chan	0	COG5273(k
Q91YX0	THMS2_M	Themis2	Themis2	230787	Protein TH	no	up	0	ENOG4110
Q91YX5	LGAT1_M	Lpgat1	Lpgat1	226856	Acyl-CoA:ly	no	no chan	0	COG0204(/
Q61502	E2F5_MOU	E2f5	E2f5	13559	Transcripti	no	no chan	0	ENOG410
Q08642	PADI2_M	Padi2	Padi2	18600	Protein-arg	no	up	0	ENOG410
E9PWK7	E9PWK7_M	Ephb4	Ephb4		receptor pr	no	no chan	0	COG0515(N
A0A338P6E	A0A338P6E	Tango2	Tango2		Transport o	no	no chan	0	COG3332(C
Q3V384	AFG1L_M	Afg1l	Afg1l	215951	AFG1-like	no	no chan	0	COG1485(/
A2RS47	A2RS47_M	Kctd6	Kctd6	71393	Potassium	no	no chan	0	ENOG410
Q3U430	Q3U430_M	Anapc2	Anapc2	99152	Anaphase-p	no	no chan	0	ENOG410
B7ZMP1	XPP3_MOU	Xpnpep3	Xpnpep3	321003	Xaa-Pro an	no	no chan	0	COG0006(C
Q8VE22	RT23_MOU	Mrps23	Mrps23	64656	Small ribos	no	no chan	0	ENOG4111
Q60634	FLOT2_M	Flot2	Flot2	14252	Flotillin-2	no	no chan	0	COG2268(I
Q8VE27	Q8VE27_M	D16Ertd472	D16Ertd472	67102	DNA segme	no	no chan	0	ENOG4111
D3Z125	D3Z125_M	Tpd52	Tpd52		Tumor prot	no	up	0	ENOG4111
Q8BG46	Q8BG46_M	Ankrd10	Ankrd10	102334	Ankyrin rep	yes	down	1	COG0666(I
Q8BG40	KTNB1_M	Katnb1	Katnb1	74187	Katanin p8	no	no chan	0	ENOG410
P62908	RS3_MOUS	Rps3	Rps3	27050	Small ribos	no	no chan	0	COG0092(I
A0A0R4J2B	A0A0R4J2B	Kctd12	Kctd12	239217	Potassium	no	no chan	0	ENOG410
A0A0R4J2B	A0A0R4J2B	Rbbp5	Rbbp5	213464	Retinoblast	no	no chan	0	ENOG410
Q6TEK5	VKORL_M	Vkorc1l1	Vkorc1l1	69568	Vitamin K	no	no chan	0	ENOG4111
Q3UW73	Q3UW73_M	Sgk2	Sgk2	27219	Serine/thre	no	up	0	ENOG410
P70375	FA7_MOUS	F7	F7	14068	Coagulatio	no	up	0	COG5640(I
Q9DB16	CB39L_MC	Cab39l	Cab39l	69008	Calcium-bi	no	no chan	0	ENOG410
P70372	ELAV1_M	Elavl1	Elavl1	15568	ELAV-like	no	no chan	0	ENOG410
P70371	TERF1_M	Terf1	Terf1	21749	Telomeric r	no	no chan	0	ENOG4111
D3YWC2	D3YWC2_M	Arl6ip4	Arl6ip4	65105	ADP-ribosy	no	no chan	0	ENOG4111
Q80V26	IMPA3_M	Bpnt2	Bpnt2	242291	Golgi-resid	no	no chan	0	COG1218(3
Q8R502	LRC8C_M	Lrrc8c	Lrrc8c	100604	Volume-reg	no	no chan	0	COG4886(I
Q80Y98	DDHD2_M	Ddhd2	Ddhd2	72108	Triacylglyc	no	no chan	0	ENOG410

Q9CZH8	CCD77_M(	Ccdc77	Ccdc77	67200	Coiled-coil	no | no chan	0	ENOG410Y
I7HIF3	I7HIF3_M(	Atp1b2	Atp1b2	11932	Sodium/po	no | up	0	ENOG4111
Q9CZH3	PSMG3_M(	Psmg3	Psmg3	66506	Proteasome	no | no chan	0	ENOG410Y
Q9DBV4	MXRA8_M	Mxra8	Mxra8	74761	Matrix rem	no | no chan	0	ENOG4111
Q9CZH7	MXRA7_M	Mxra7	Mxra7	67622	Matrix-rem	no | down	0	ENOG410Y
Q99LI5	ZN281_MC	Znf281	Znf281	226442	Zinc finger	no | no chan	0	COG5048(F
Q7TSC1	PRC2A_M(	Prrc2a	Prrc2a	53761	Protein PRl	no | no chan	0	ENOG410X
F6ZGI7	F6ZGI7_M	Mocs2	Mocs2	17434	Molybdopte	no | no chan	0	COG0314(i
Q9EPS3	GLCE_MO	Glce	Glce	93683	D-glucuron	no | no chan	0	ENOG410X
E9QJS0	E9QJS0_M(	Mrps10	Mrps10	64657	Small ribos	no | no chan	0	COG0051(t
O89023	TPP1_MOU	Tpp1	Tpp1	12751	Tripeptidyl	no | no chan	0	COG4934(p
O89020	AFAM_MC	Afm	Afm	280662	Afamin	no | no chan	0	ENOG410Z
D3Z383	D3Z383_M	Mest	Mest	17294	Mesoderm s	no | down	0	ENOG4111
Q8BSP2	CNDH2_M	Ncaph2	Ncaph2	52683	Condensin-	no | no chan	0	ENOG4111
E9QJS7	E9QJS7_M(	Adgre5	Adgre5	26364	Adhesion G	no | no chan	0	ENOG410X
A5A4Y9	A5A4Y9_M	Ppp1r11	Ppp1r11	76497	E3 ubiquiti	no | up	0	ENOG4112
Q3UJT0	Q3UJT0_M	Tasp1	Tasp1	75812	Taspase, th	no | up	0	COG1446(I
A2AHQ7	A2AHQ7_M	Phka2	Phka2	110094	Phosphoryl	no | no chan	0	ENOG410X
Q9JLN9	MTOR_MC	Mtor	Mtor	56717	Serine/thre	no | no chan	0	COG5032(l
A0A0R4J0Z	A0A0R4J0Z	Pdia4	Pdia4	12304	Protein dist	yes | up	1	COG0526(p
Q99N15	Q99N15_M	Hsd17b10	Hsd17b10	15108	17beta-hyd	no | no chan	0	ENOG410X
Q9JLN6	ADA28_M(	Adam28	Adam28	13522	Disintegrin	no | no chan	0	ENOG410X
F6S7W6	F6S7W6_M	Sik3	Sik3		non-specifi	no | no chan	0	ENOG410X
Q80UK0	SESD1_MC	Sestd1	Sestd1	228071	SEC14 dom	no | no chan	0	ENOG410X
O08749	DLDH_MC	Dld	Dld	13382	Dihydrolip	no | no chan	0	COG1249(c
A0A0G2JE	A0A0G2JE	Sh2b3	Sh2b3		SH2B adap	no | no chan	0	ENOG410Y
O08746	MATN2_M	Matn2	Matn2	17181	Matrilin-2	no | no chan	0	ENOG410X
G3UYU5	G3UYU5_M	Rpp40	Rpp40		Ribonuclea	no | no chan	0	ENOG410Z
A0A0U1RP	A0A0U1RP	Dguok	Dguok		Deoxyguan	no | no chan	0	COG1428(C
Q3THN2	Q3THN2_M	Dera	Dera	232449	deoxyribose	no | no chan	0	COG0274(C
Q9D0F1	NDC80_M(	Ndc80	Ndc80	67052	Kinetochor	no | no chan	0	COG5185(N
G3UYU0	G3UYU0_M	Mex3a	Mex3a	72640	Mex3 RNA	no | no chan	0	ENOG410Z
Q9D0F4	NKAP_MC	Nkap	Nkap	67050	NF-kappa-l	no | no chan	0	ENOG4111
Q7TS68	NSUN6_M(	Nsun6	Nsun6	74455	tRNA (cyto	no | no chan	0	COG0144(C
Q8R179	KBTB4_M(	Kbtbd4	Kbtbd4	67136	Kelch repe	no | no chan	0	ENOG410X
A1L013	A1L013_M(	Cactin	Cactin	70312	Splicing fac	no | no chan	0	ENOG410X
Q6SJQ0	CLM8_MO	Cd300a	Cd300a	217303	CMRF35-lil	no | up	0	ENOG410Z
G3V000	G3V000_M	Otx1	Otx1		Orthodenti	no | no chan	0	ENOG4111
O55142	RL35A_MC	Rpl35a	Rpl35a	57808	Large ribos	no | no chan	0	COG2451(F
Q543P7	Q543P7_M(	Arl3	Arl3	56350	ADP-ribosy	no | no chan	0	ENOG410Y
E9Q512	E9Q512_M	Trip11	Trip11	109181	Thyroid ho	no | no chan	0	ENOG410X
P20029	BIP_MOUS	Hspa5	Hspa5	14828	Endoplasm	no | no chan	0	COG0443(F
D3Z4C0	D3Z4C0_M	Farp2	Farp2		FERM, Rhc	no | down	0	ENOG410X

A2RTA0	A2RTA0_M	Ckm	Ckm	12715	Creatine ki	no | no chan	0	COG3869(/
D3Z4C9	D3Z4C9_M	Uqcc2	Uqcc2		Mitochondı	no | no chan	0	ENOG4111
Q61469	PLPP1_MC	Plpp1	Plpp1	19012	Phospholip	no | down	0	COG0671(S
Q8VI84	NOC3L_M	Noc3l	Noc3l	57753	Nucleolar c	no | no chan	0	COG5117(r
Q91W36	UBP3_MO	Usp3	Usp3	235441	Ubiquitin c	no | no chan	0	ENOG410)
Q91W39	NCOA5_M	Ncoa5	Ncoa5	228869	Nuclear rec	no | no chan	0	ENOG410)
Q63810	CANB1_M	Ppp3r1	Ppp3r1	19058	Calcineurin	no | no chan	0	COG5126(v
Q8C5Q4	GRSF1_MC	Grsf1	Grsf1	231413	G-rich sequ	no | no chan	0	ENOG410Y
Q67FY2	BCL9L_MC	Bcl9l	Bcl9l	80288	B-cell CLL/	no | no chan	0	ENOG410)
Q3UWM4	KDM7A_M	Kdm7a	Kdm7a	338523	Lysine-spec	no | up	0	ENOG410)
A0A9L6JK(	A0A9L6JK(	Irgm2	Irgm2		Immunity-r	no | up	0	ENOG4112
Q4KL52	Q4KL52_M	Zfyve19	Zfyve19	72008	Zfyve19 prc	no | no chan	0	ENOG410)
A0A1L1SQ	A0A1L1SQ	Ahi1	Ahi1	52906	Abelson hel	no | no chan	0	ENOG410)
Q9CXA2	T3HPD_M	L3hypdh	L3hypdh	67217	Trans-L-3-h	no | no chan	0	COG3938(I
E9PY39	E9PY39_M	Gm20431	Gm20431		Predicted g	no | no chan	0	ENOG410)
A0A1Y7VJ	A0A1Y7VJ	Ero1b	Ero1b		Endoplasm	no | up	0	COG5061(T
A0A1D5RL	A0A1D5RL	Iws1	Iws1	73473	IWS1, SUPT	no | no chan	0	COG5139(I
Q9CUX0	Q9CUX0_M	N6amt1	N6amt1	67768	Uncharacte	no | no chan	0	COG2890(c
A0A1D5RL	A0A1D5RL	Wdfy3	Wdfy3	72145	WD repeat	no | no chan	0	ENOG410)
P14152	MDHC_MC	Mdh1	Mdh1	17449	Malate deh	no | no chan	0	COG0039(r
Q3V1N1	MFHA1_M	Mfhas1	Mfhas1	52065	Malignant	no | no chan	0	COG4886(C
P61939	THBG_MC	Serpina7	Serpina7	331535	Thyroxine-l	no | up	0	COG4826(p
Q1A602	Q1A602_M	Actn4	Actn4	60595	Alpha-actir	no | no chan	0	COG5069(r
Q8K262	PLAC9_MC	Plac9	Plac9	211623	Placenta-sp	no | no chan	0	ENOG4111
Q9DBL9	ABHD5_M	Abhd5	Abhd5	67469	1-acylglycei	no | down	0	COG0596(I
Q8K268	ABCF3_MC	Abcf3	Abcf3	27406	ATP-bindir	no | no chan	0	COG0488(S
Q9DBL7	COASY_M	Coasy	Coasy	71743	Bifunctionc	no | no chan	0	COG1019(I
Q9DBL2	GDAP2_M	Gdap2	Gdap2	14547	Ganglioside	no | no chan	0	COG2110(I
P97784	CRY1_MO	Cry1	Cry1	12952	Cryptochro	no | up	0	COG0415(c
A0A2X0SS(	A0A2X0SS(	TIAM2	TIAM2	24001	TIAM2	no | no chan	0	ENOG410)
P97783	AF1Q_MO	Mllt11	Mllt11	56772	Protein AF	no | down	0	ENOG4112
Q8VC16	LRC14_MC	Lrrc14	Lrrc14	223664	Leucine-ric	no | no chan	0	ENOG4111
Q3UFS0	ZY11B_MC	Zyg11b	Zyg11b	414872	Protein zyg	no | no chan	0	ENOG410)
A2ANY6	A2ANY6_M	Mdn1	Mdn1	100019	Midasin	no | no chan	0	COG5271(N
B2RQC6	PYR1_MO	Cad	Cad	69719	Multifuncti	no | no chan	0	COG0540(c
Q8CJ76	Q8CJ76_M	Mllt1	Mllt1	64144	Leukemia-o	no | no chan	0	COG5033(I
Q9CY46	Q9CY46_M	Lsm4	Lsm4	50783	U6 snRNA-	no | no chan	0	ENOG4111
A2ANY8	A2ANY8_M	Ankrd6	Ankrd6		Ankyrin rep	no | no chan	0	COG0666(/
D3Z7C0	D3Z7C0_M	Mrpl40	Mrpl40		Large ribos	no | no chan	0	ENOG4111
Q3UQ71	Q3UQ71_M	Ephx2	Ephx2	13850	AB hydrola	no | down	0	COG1011(C
A6H6K1	A6H6K1_M	Aspn	Aspn	66695	Aspn protei	no | down	0	COG4886(p
Q9WUV0	ORC5_MO	Orc5	Orc5	26429	Origin reco	no | no chan	0	ENOG410)
A0A087WP	A0A087WP	Abi2	Abi2		Abl interac	no | no chan	0	ENOG410Y

Q8K0T4	KATL1_M(	Katnal1	Katnal1	231912	Katanin p6	no	no chan	0	ENOG410)
Q3TDW6	Q3TDW6_I	Apbb2	Apbb2	11787	Amyloid be	yes	down	1	ENOG410Y
Q8K0T2	DC2L1_M(	Dync2li1	Dync2li1	213575	Cytoplasmi	no	no chan	0	ENOG410)
Q8CAA7	PGM2L_M	Pgm2l1	Pgm2l1	70974	Glucose 1,6	no	no chan	0	COG1109(c
Q80XJ6	Q80XJ6_M	Slc25a19	Slc25a19	67283	Mitochondı	no	no chan	0	ENOG410Z
B2RXX9	B2RXX9_M	Myh7	Myh7	140781	Myosin-7	no	up	0	COG5022(C
Q8R1Z9	RN121_MC	Rnf121	Rnf121	75212	E3 ubiquiti	no	up	0	ENOG410)
Q3U0V3	Q3U0V3_M	Ankrd13a	Ankrd13a	68420	Uncharacte	no	no chan	0	ENOG410)
Q80VJ3	DNPH1_M	Dnph1	Dnph1	381101	5-hydroxym	no	no chan	0	ENOG4111
P97434	MPRIP_M(	Mprip	Mprip	26936	Myosin phc	no	no chan	0	ENOG410Z
P97433	ARG28_M(	Arhgef28	Arhgef28	110596	Rho guanin	no	no chan	0	ENOG410)
A0A0R4IZ\	A0A0R4IZ\	Prtn3	Prtn3	19152	Proteinase :	no	up	0	COG5640(c
B1AZP2	DLGP4_M(	Dlgap4	Dlgap4	228836	Disks large-	no	no chan	0	ENOG4111
A0A0R4IZ\	A0A0R4IZ\	Tead4	Tead4	21679	Transcripti	no	no chan	0	ENOG410)
A0A286YC'	A0A286YC'	Kif13b	Kif13b	16554	Kinesin fan	no	no chan	0	COG5059(p
A0A0R4IZ\	A0A0R4IZ\	Thop1	Thop1	50492	Thimet olig	no	no chan	0	COG0339(c
A0A0R4IZ\	A0A0R4IZ\	Dtx2	Dtx2	74198	E3 ubiquiti	no	no chan	0	ENOG4111
P16092	FGFR1_M(	Fgfr1	Fgfr1	14182	Fibroblast (	no	no chan	0	COG0515(N
E9PZQ0	RYR1_MOI	Ryr1	Ryr1	20190	Ryanodine :	no	up	0	ENOG410Y
A0A286YC'	A0A286YC'	Atp9b	Atp9b	50771	Phospholip	no	no chan	0	ENOG410)
A0A0R4IZ\	A0A0R4IZ\	Dus3l	Dus3l	224907	tRNA-dihy	no	no chan	0	COG0042(r
P62488	RPB7_MOI	Polr2g	Polr2g	67710	DNA-direc	no	no chan	0	COG1095(I
G3X9M0	G3X9M0_M	Dap3	Dap3	65111	Small ribos	no	no chan	0	ENOG410)
Q9JL60	GMEB1_M	Gmeb1	Gmeb1	56809	Glucocortic	no	no chan	0	ENOG410Z
A0A1L1SU	A0A1L1SU	Slc37a4	Slc37a4		Solute carri	no	no chan	0	COG2271(t
E9PXK7	E9PXK7_M	Pex11b	Pex11b	18632	Peroxisoma	no	no chan	0	ENOG4111
A2RSF1	A2RSF1_M	Commd1	Commd1	17846	COMM dor	no	no chan	0	ENOG4111
A0A0A0M(	A0A0A0M(	Exo5	Exo5		Exonucleas	no	no chan	0	ENOG4111
Q14BV7	Q14BV7_M	Acbd6	Acbd6	72482	Acyl-CoA-b	no	no chan	0	COG4281(r
A0A2I3BQ	A0A2I3BQ	Nudt18	Nudt18		Nudix hydr	no	no chan	0	ENOG411(
A0A7S8WJ	A0A7S8WJ	Abpbg26	Abpbg26	110187	ABPBG26	no	no chan	0	ENOG4111
P22518	CLK1_MO	Clk1	Clk1	12747	Dual specif	no	no chan	0	ENOG410)
Q9Z100	CPXM1_M	Cpxm1	Cpxm1	56264	Probable cc	no	down	0	ENOG410)
A0A3B2WE	A0A3B2WE	Rhag	Rhag	19743	Ammoniun	no	no chan	0	ENOG410)
Q7TMI3	UHRF2_M(	Uhrf2	Uhrf2	109113	E3 ubiquiti	no	no chan	0	COG3440(u
Q2NL50	Q2NL50_M	Syne1	Syne1	64009	Syne1 prote	no	no chan	0	COG5069(I
A0A3B2WE	A0A3B2WE	Tjp2	Tjp2		Tight juncti	no	no chan	0	ENOG410)
Q9CZC8	SCRN1_M(	Scrn1	Scrn1	69938	Secernin-1	no	no chan	0	COG4690(Z
Q2NL54	Q2NL54_M	Mrgbp	Mrgbp	73247	MRG-bindi	no	no chan	0	ENOG4111
Q3TF84	Q3TF84_M	Lrrc59	Lrrc59	98238	Leucine-ric	no	no chan	0	COG4886(c
Q6PAV2	HERC4_M(	Herc4	Herc4	67345	Probable E	no	no chan	0	COG5184(C
Q3TF87	Q3TF87_M	Dars1	Dars1	226414	Aspartate--	no	no chan	0	COG0017(V
Q3TLR7	DTL_MOU	Dtl	Dtl	76843	Denticleles	no	no chan	0	ENOG410)

Q3TLR8	Q3TLR8_M	Smox	Smox	228608	Spermine o:	no|up	0	ENOG410X
Q05AG7	Q05AG7_M	Nt5dc1	Nt5dc1	319638	5'-nucleotid	no|no chan	0	ENOG410X
Q9D273	MMAB_MO	Mmab	Mmab	77697	Corrinoid a	no|no chan	0	COG2096(c
Q8BXJ2	TREF1_MO	Trerf1	Trerf1	224829	Transcripti	no|no chan	0	ENOG4110
Q9JKY0	CNOT9_M	Cnot9	Cnot9	58184	CCR4-NOT	no|no chan	0	COG5209(c
Q9ERD7	TBB3_MO	Tubb3	Tubb3	22152	Tubulin be	no|no chan	0	COG5023(T
Q9ERD6	RGPS2_MO	Ralgps2	Ralgps2	78255	Ras-specific	no|no chan	0	ENOG410Y
P10493	NID1_MO	Nid1	Nid1	18073	Nidogen-1	no|no chan	0	ENOG410X
Q9Z0H1	WDR46_M	Wdr46	Wdr46	57315	WD repeat-	no|no chan	0	ENOG410X
A0A0N4SV	A0A0N4SV	Fancd2	Fancd2	211651	Fanconi an	no|no chan	0	ENOG410X
Q9CXU9	EIF1B_MO	Eif1b	Eif1b	68969	Eukaryotic	no|no chan	0	COG0023(t
Q8K0C8	COX19_MO	Cox19	Cox19	68033	Cytochrom	no|no chan	0	ENOG4112
A2AIL4	NDUF6_M	Ndufaf6	Ndufaf6	76947	NADH deh	no|no chan	0	COG1562(p
A0A1D5RM	A0A1D5RM	Rassf5	Rassf5		Ras associa	no|no chan	0	ENOG4111
Q6P5D4	CP135_MO	Cep135	Cep135	381644	Centrosom	no|no chan	0	ENOG410X
B2LVG5	B2LVG5_M	Cpsf4	Cpsf4	54188	Cleavage a	no|no chan	0	COG5084(C
Q3TFE2	Q3TFE2_M	Nup62	Nup62	18226	Nucleopori	no|no chan	0	ENOG410X
Q99L27	GMPR2_M	Gmpr2	Gmpr2	105446	GMP reduc	no|no chan	0	COG0516(C
Q8CGF6	WDR47_M	Wdr47	Wdr47	99512	WD repeat-	no|no chan	0	ENOG410Y
Q8C954	Q8C954_M	Tmed5	Tmed5	73130	GOLD dom	no|no chan	0	ENOG410Z
Q99K90	TAB2_MO	Tab2	Tab2	68652	TGF-beta-c	no|no chan	0	ENOG410X
Q3UGT9	Q3UGT9_M	Parvb	Parvb	170736	Parvin, bet	no|no chan	0	ENOG410X
E9PYK0	E9PYK0_M	Unc93b1	Unc93b1	54445	Unc-93 hon	no|no chan	0	ENOG410X
A0PJN6	A0PJN6_M	2310061I04	2310061I04	69662	2310061I04	no|no chan	0	ENOG4111
Q8C140	Q8C140_M	Slc25a13	Slc25a13	50799	EF-hand dc	no|no chan	0	ENOG410X
P51675	CCR1_MO	Ccr1	Ccr1	12768	C-C chemol	yes|up	1	ENOG410X
K3W4P5	K3W4P5_M	Immp2l	Immp2l		IMP2 inner	no|no chan	0	COG0681(I
P56375	ACYP2_MO	Acyp2	Acyp2	75572	Acylphosph	no|up	0	ENOG4112
Q9DAV9	TM38B_MO	Tmem38b	Tmem38b	52076	Trimeric in	no|up	0	ENOG410X
Q8CCJ3	UFL1_MO	Ufl1	Ufl1	67490	E3 UFM1-p	no|no chan	0	ENOG410X
Q91X96	MSS4_MO	Rabif	Rabif	98710	Guanine nu	no|up	0	ENOG4111
B1AQE4	B1AQE4_M	Cyth1	Cyth1		Cytohesin 1	no|no chan	0	COG5307(F
Q69ZZ7	Q69ZZ7_M	mKIAA076	mKIAA076	233744	Spondin-1	no|no chan	0	ENOG410X
Q69ZZ9	K0754_MO	Macf1	Macf1	11426	Microtubul	no|no chan	0	COG5069(F
B1AS46	B1AS46_M	Casz1	Casz1	69743	Castor zinc	no|up	0	ENOG410X
A0A140LH	A0A140LH	Adam9	Adam9	11502	ADAM met	no|no chan	0	ENOG410X
P70168	IMB1_MO	Kpnb1	Kpnb1	16211	Importin su	no|no chan	0	COG5215(I
Q99LE1	RIPL2_MO	Rilpl2	Rilpl2	80291	RILP-like p	no|up	0	ENOG4111
Q80X71	T106B_MO	Tmem106b	Tmem106b	71900	Transmemb	no|no chan	0	ENOG410Z
A0A1W2P7	A0A1W2P7	Sppl2b	Sppl2b		Signal pept	no|down	0	ENOG410Z
O88697	STK16_MO	Stk16	Stk16	20872	Serine/thre	no|no chan	0	ENOG410Y
Q80WQ2	VAC14_MO	Vac14	Vac14	234729	Protein VA	no|no chan	0	ENOG410X
E9Q748	E9Q748_M	Slpi	Slpi		Secretory le	no|no chan	0	ENOG4111

O88693	CEGT_MC	Ugcg	Ugcg	22234	Ceramide g	yes|down	1	COG1215((
Q80WQ6	RHDF2_M	Rhbdf2	Rhbdf2	217344	Inactive rh	no|no chan	0	COG0705(v
Q80WQ8	M18BP_MC	Mis18bp1	Mis18bp1	217653	Mis18-bind	no|no chan	0	ENOG4112
E9Q743	CF251_MO	Cfap251	Cfap251	269701	Cilia- and f	no|no chan	0	ENOG410
A0AAQ4V	A0AAQ4VMQ4_MOUSE	Phosphorylase b kin	Phkb			no|up	0	ENOG410
Q7TN20	Q7TN20_M	Ythdf3	Ythdf3	229096	YTH domai	no|no chan	0	ENOG410
A0AAQ4V	A0AAQ4VMQ7_MOUSE	Poly(rC) binding pro	Pcbp2			no|no chan	0	ENOG410
Q00623	APOA1_M	Apoa1	Apoa1	11806	Apolipopro	no|no chan	0	ENOG410
Q7TN29	SMAP2_M	Smap2	Smap2	69780	Stromal me	no|no chan	0	COG5347(u
Q8BU33	HACL2_M	Ilvbl	Ilvbl	216136	2-hydroxya	no|no chan	0	COG0028(I
A0A0G2JG	A0A0G2JG	Camk2d	Camk2d	108058	Calcium/cc	no|no chan	0	ENOG410
Q6NZL1	Q6NZL1_M	Dhx37	Dhx37	208144	RNA helico	no|no chan	0	COG1643(p
Q8BU30	SYIC_MOU	Iars1	Iars1	105148	Isoleucine--	no|no chan	0	COG0060((
Q9D4J3	DCBD1_M	Dcbld1	Dcbld1	66686	Discoidin, (	no|no chan	0	ENOG411(
Q8BU36	Q8BU36_M	Supt3	Supt3	109115	Transcripti	no|no chan	0	ENOG410
Q3TP69	Q3TP69_M	Gpc6	Gpc6	23888	Glypican-6	no|no chan	0	ENOG410
D3YXK2	SAFB1_MC	Safb	Safb	224903	Scaffold att	no|no chan	0	ENOG4111
D3YXK1	SAMD1_M	Samd1	Samd1	666704	Sterile alph	no|no chan	0	ENOG4111
Q8VHE0	SEC63_MC	Sec63	Sec63	140740	Translocati	no|no chan	0	COG5407(t
A3KMP2	TTC38_MC	Ttc38	Ttc38	239570	Tetratricop	no|no chan	0	ENOG410
A0A0U1RP	A0A0U1RP	Isg20	Isg20	57444	Interferon-s	no|up	0	COG0847(T
Q9CR16	PPID_MOU	Ppid	Ppid	67738	Peptidyl-pr	no|no chan	0	COG0652(I
O88520	SHOC2_M	Shoc2	Shoc2	56392	Leucine-ric	no|no chan	0	COG4886((
A2AUC9	KLH41_M	Klhl41	Klhl41	228003	Kelch-like p	no|up	0	ENOG410
Q8BKW4	ZCHC4_M	Zcchc4	Zcchc4	78796	rRNA N(6)-	no|no chan	0	ENOG410
B9EHJ3	B9EHJ3_M	Tjp1	Tjp1	21872	Tight juncti	no|no chan	0	ENOG410
A2AUC6	A2AUC6_M	Bbs5	Bbs5		Bardet-Biec	no|no chan	0	ENOG410
Q9DBB9	CPN2_MO	Cpn2	Cpn2	71756	Carboxypep	no|no chan	0	COG4886(M
B2CQD6	B2CQD6_M	Fbln1	Fbln1	14114	Fibulin-1	no|no chan	0	ENOG410
P52019	ERG1_MO	Sqle	Sqle	20775	Squalene m	no|no chan	0	COG0654(g
Q9CR13	FMC1_MO	Fmc1	Fmc1	66117	Protein FM	no|no chan	0	ENOG4111
Q4FK24	Q4FK24_M	Usp18	Usp18	24110	ubiquitinyl	no|up	0	COG5077(u
G3X959	G3X959_M	Pphln1	Pphln1	223828	Periphilin 1	no|no chan	0	ENOG4111
Q0GUM3	IRB10_MO	Gm12250	Gm12250	631323	Interferon-g	no|up	0	ENOG4112
Q8VCA8	SCRN2_M	Scrn2	Scrn2	217140	Secernin-2	no|no chan	0	COG4690(d
Q8VCA4	Q8VCA4_M	Cyp4f15	Cyp4f15	106648	Cyp4f15 pr	no|no chan	0	COG2124(M
Q7TPN9	PRR14_MC	Prr14	Prr14	233895	Proline-rich	no|no chan	0	ENOG4111
Q8VDM1	ZGPAT_M	Zgpat	Zgpat	229007	Zinc finger	no|no chan	0	ENOG410
Q61712	DNJC1_M	Dnajc1	Dnajc1	13418	DnaJ homo	no|no chan	0	COG2214(c
W0TYI4	W0TYI4_M	Tcp1	Tcp1	21454	T-complex	no|no chan	0	COG0459(I
Q925I1	ATD3A_M	Atad3a	Atad3a	108888	ATPase fan	no|no chan	0	COG1223(A
D3YX08	D3YX08_M	Tmem109	Tmem109		Transmemb	no|no chan	0	ENOG410
Q61249	IGBP1_MC	Igbp1	Igbp1	18518	Immunoglo	no|no chan	0	ENOG4111

A2AF67	A2AF67_M	Dock11	Dock11		Dedicator c	no|down	0	ENOG410)
A2A8U2	TM201_MC	Tmem201	Tmem201	230917	Transmemb	no|no chan	0	ENOG4112
Q9CQA5	MED4_MC	Med4	Med4	67381	Mediator of	no|no chan	0	ENOG410)
Q8BHD8	PCMD2_M	Pcmtd2	Pcmtd2	245867	Protein-L-is	no|no chan	0	COG2518(C
Q9CQA1	TPPC5_MC	Trappc5	Trappc5	66682	Trafficking	no|no chan	0	COG5128(t
N0E634	N0E634_M	Csnk2b	Csnk2b	13001	Casein kina	no|up	0	COG5041(c
D3Z710	D3Z710_M	Klc3	Klc3		Kinesin ligh	no|no chan	0	COG0457(f
Q8BGD8	COA6_MO	Coa6	Coa6	67892	Cytochrome	no|no chan	0	ENOG4112
Q6P6L8	Q6P6L8_M	Zfp451	Zfp451	98403	Zfp451 prot	no|no chan	0	COG5048(F
A0A0G2JFS	A0A0G2JFS	Atg4a-ps	Atg4a-ps		Cysteine pr	no|no chan	0	ENOG410)
Q9EQ06	DHB11_MC	Hsd17b11	Hsd17b11	114664	Estradiol 17	no|no chan	0	COG1028(r
Q8JZM0	TFB1M_MC	Tfb1m	Tfb1m	224481	Dimethylac	no|no chan	0	COG0030(F
Q8K1N4	SPAS2_MO	Spats2	Spats2	72572	Spermatoge	no|no chan	0	ENOG410Y
Q8JZM6	TIFAB_MC	Tifab	Tifab	212937	TRAF-inter	no|no chan	0	ENOG410Y
Q8JZM7	CDC73_MC	Cdc73	Cdc73	214498	Parafibrom	no|no chan	0	COG5157(c
A0A0R4J22	A0A0R4J22	Neu2	Neu2	23956	exo-alpha-s	no|no chan	0	ENOG410Y
A0A0R4J22	A0A0R4J22	Bscl2	Bscl2	14705	Seipin	no|no chan	0	ENOG4111
A0A0R4J0E	A0A0R4J0E	Fgl1	Fgl1	234199	Fibrinogen-	no|up	0	ENOG410Z
A0A0R4J0E	A0A0R4J0E	Usb1	Usb1	101985	U6 snRNA	no|no chan	0	ENOG4111
U5JE79	U5JE79_MOUSE	Immmunoglobulin heavy chc			Immmunog	no|no chan	0	ENOG4111
A0A0R4J0E	A0A0R4J0E	Ints7	Ints7	77065	Integrator c	no|no chan	0	ENOG410)
P62715	PP2AB_MC	Ppp2cb	Ppp2cb	19053	Serine/thre	no|no chan	0	COG0639((
E9Q4P1	WDFY1_M	Wdfy1	Wdfy1	69368	WD repeat	no|no chan	0	COG2319(c
E9Q4P0	E9Q4P0_M	Kxd1	Kxd1		KxDL moti	no|no chan	0	COG1552(F
A0A087WQ	A0A087WQ	Tns1	Tns1	21961	Tensin 1	no|no chan	0	COG2453(T
A0A087WQ	A0A087WQ	Sgk3	Sgk3		Serum/gluc	no|no chan	0	ENOG410)
A0A087WQ	A0A087WQ	Bzw1	Bzw1		Basic leucir	no|no chan	0	ENOG410)
Q8BSN9	FAKD3_M	Fastkd3	Fastkd3	69577	FAST kinas	no|no chan	0	ENOG4111
Q8CC86	PNCB_MO	Naprt	Naprt	223646	Nicotinate	no|no chan	0	COG1488(F
B1ATS4	B1ATS4_M	Atp2a3	Atp2a3		Calcium-tra	no|no chan	0	COG0474(C
Q5SWZ0	Q5SWZ0_M	Prpsap2	Prpsap2	212627	Phosphorib	no|no chan	0	COG0462(S
Q9CPW7	ZMAT2_M	Zmat2	Zmat2	66492	Zinc finger	no|down	0	ENOG4111
Q3U3C2	Q3U3C2_M	Npc2	Npc2	67963	NPC intrac	no|up	0	ENOG4111
Q8BZF8	PGM5_MC	Pgm5	Pgm5	226041	Phosphoglu	no|up	0	COG0033((
Q8CC88	VWA8_MC	Vwa8	Vwa8	219189	von Willebr	no|no chan	0	ENOG410)
Q9CY64	BIEA_MOU	Blvra	Blvra	109778	Biliverdin r	no|no chan	0	ENOG4111
Q8K010	OPLA_MO	Oplah	Oplah	75475	5-oxoprolin	no|no chan	0	COG0145(5
Q8C263	SKA3_MOU	Ska3	Ska3	219114	SKA compl	no|no chan	0	ENOG4111
Q8VHY0	CSPG4_MC	Cspg4	Cspg4	121021	Chondroitin	no|down	0	ENOG410)
Q9JIG8	PRAF2_MC	Praf2	Praf2	54637	PRA1 famil	no|up	0	ENOG4111
Q9DCG2	CD302_MC	Cd302	Cd302	66205	CD302 anti	no|down	0	ENOG4112
Q0KL01	UBX2B_MC	Ubxn2b	Ubxn2b	68053	UBX doma	no|down	0	ENOG410Y
Q0KL02	TRIO_MOU	Trio	Trio	223435	Triple func	no|no chan	0	ENOG410)

O88983	STX8_MOU	Stx8	Stx8	55943	Syntaxin-8	no|no chan	0	ENOG410Z
O88986	KBL_MOU	Gcat	Gcat	26912	2-amino-3-l	no|no chan	0	COG0156(c
F8VQE9	F8VQE9_M	Agap3	Agap3	213990	ArfGAP wi	no|no chan	0	COG5347(S
A0A5F8MP	A0A5F8MP	Fmn1	Fmn1	14260	Formin 1	no|no chan	0	ENOG410X
Q6PGF5	Q6PGF5_M	Bms1	Bms1	213895	BMS1 homo	no|no chan	0	COG5192(F
A0A0R4J1A	A0A0R4J1A	Tnnt3	Tnnt3	21957	Troponin T	no|up	0	ENOG410X
A0A0R4J1A	A0A0R4J1A	Ssx2ip	Ssx2ip	99167	SSX family	no|up	0	ENOG410X
Q5FW60	MUP20_MO	Mup20	Mup20	381530	Major urinc	no|down	0	ENOG4111
A8E634	A8E634_MO	Slc9a9	Slc9a9	331004	Sodium/hy	no|down	0	COG0025(I
Q6PCQ0	IQCE_MO	Iqce	Iqce	74239	IQ domain-	no|no chan	0	ENOG411C
A0A0R4J1A	A0A0R4J1A	Chml	Chml	12663	Rab proteir	no|up	0	COG5044(C
Q62267	SPR1B_MC	Sprr1b	Sprr1b	20754	Cornifin-B	no|up	0	ENOG410X
A0A0R4J1A	A0A0R4J1A	Gas2l3	Gas2l3	237436	Growth arr	no|no chan	0	ENOG410Z
A0A0R4J1A	A0A0R4J1A	Arid3a	Arid3a	13496	AT-rich int	no|no chan	0	ENOG410Y
P56818	BACE1_MO	Bace1	Bace1	23821	Beta-secret	no|no chan	0	ENOG410X
P49300	CLC10_MC	Clec10a	Clec10a	17312	C-type lecti	no|down	0	ENOG410X
P56812	PDCD5_M	Pdcd5	Pdcd5	56330	Programme	no|no chan	0	COG2118(I
P97333	NRP1_MO	Nrp1	Nrp1	18186	Neuropilin-	no|no chan	0	ENOG410Y
Q8C7R4	UBA6_MO	Uba6	Uba6	231380	Ubiquitin-l	no|no chan	0	COG0476(c
Q3TB15	Q3TB15_M	Appl2	Appl2	216190	DCC-intera	no|no chan	0	ENOG410Z
A0A5F8MP	A0A5F8MP	Septin4	Septin4		Septin-4	no|no chan	0	ENOG4111
Q6ZWX2	Q6ZWX2_N	Tmsb4x	Tmsb4x	19241	Thymosin b	no|no chan	0	ENOG410Y
Q3TB11	Q3TB11_M	Tpp2	Tpp2	22019	tripeptidyl-	no|no chan	0	COG1404(p
A0A494B9U	A0A494B9U	Pde9a	Pde9a		3',5'-cyclic-(	no|no chan	0	ENOG410X
A0A494B9U	A0A494B9U	Pknox1	Pknox1		Pbx/knotte	no|down	0	ENOG410X
Q3UH93	PLXD1_MO	Plxnd1	Plxnd1	67784	Plexin-D1	no|no chan	0	ENOG410Z
D3YYD2	D3YYD2_M	Fam111a	Fam111a	107373	Family with	no|no chan	0	ENOG410Y
Q3UH98	Q3UH98_M	Acap3	Acap3	140500	Arf-GAP w	no|no chan	0	COG5347(h
Q6ZQL9	Q6ZQL9_MOUSE cDNA fis, clone TRACH30				cDNA fis, c	no|up	0	ENOG4112
Q8CIL4	FSAF1_MC	Fsaf1	Fsaf1	66523	40S small su	no|no chan	0	ENOG4112
P21981	TGM2_MC	Tgm2	Tgm2	21817	Protein-glu	no|up	0	ENOG410X
Q9CWW6	PIN4_MOU	Pin4	Pin4	69713	Peptidyl-pr	no|no chan	0	COG0760(U
Q9CWW5	Q9CWW5_	Galk1	Galk1	14635	galactokina	no|no chan	0	COG0153(C
A5CVE4	A5CVE4_M	Stim2	Stim2	116873	Stromal int	no|no chan	0	ENOG410X
A9YZW8	A9YZW8_M	Rassf1	Rassf1	56289	RASSF1A tr	no|no chan	0	ENOG4111
Q8K0V2	DCNL3_M	Dcun1d3	Dcun1d3	233805	DCN1-like	no|no chan	0	ENOG410X
A0A1B0GR	A0A1B0GR	Tubgcp2	Tubgcp2		Gamma-tul	no|no chan	0	ENOG410X
Q9Z2D8	MBD3_MC	Mbd3	Mbd3	17192	Methyl-CpC	no|no chan	0	ENOG4111
Q9Z2D6	MECP2_M	Mecp2	Mecp2	17257	Methyl-CpC	no|no chan	0	ENOG4112
A0A1B0GR	A0A1B0GR	Rbl2	Rbl2	19651	RB transcri	no|up	0	ENOG410X
Q9Z2D1	MTMR2_M	Mtmr2	Mtmr2	77116	Phosphatid	no|no chan	0	ENOG410X
Q3TY06	Q3TY06_M	Pmpca	Pmpca	66865	Mitochondi	no|no chan	0	COG0612(F
Q3TBZ6	Q3TBZ6_M	Necap2	Necap2	66147	NECAP PH	no|no chan	0	ENOG4111

A0A087WN	A0A087WN	Ly6e	Ly6e		Lymphocyt	no | up	0	ENOG4111
G3UWQ7	G3UWQ7_	Prc1	Prc1		Protein reg	no | no chan	0	ENOG410Y
A1A4T2	A1A4T2_M	Ganab	Ganab	14376	Alpha gluc	no | no chan	0	COG1501(v
Q3UP92	Q3UP92_M	Stard7	Stard7	99138	START dom	no | no chan	0	ENOG4111
O70469	DOK2_MC	Dok2	Dok2	13449	Docking pr	no | up	0	ENOG410X
Q6DI95	Q6DI95_M	Tnpo3	Tnpo3	320938	Transportir	no | no chan	0	ENOG410X
B2RQ36	B2RQ36_M	Rufy2	Rufy2	70432	RUN and F	no | no chan	0	ENOG410X
E9PVE2	E9PVE2_M	Lrrc45	Lrrc45		Leucine ricl	no | no chan	0	ENOG410Z
Q6PG57	Q6PG57_M	Mapk11	Mapk11	19094	mitogen-ac	no | no chan	0	ENOG410X
A0A2I3BRI	A0A2I3BRI	Tinf2	Tinf2		Terf1 (TRF	no | no chan	0	ENOG410Z
A0A2I3BRI	A0A2I3BRI	Gm7324	Gm7324		Predicted g	no | no chan	0	ENOG4111
P40237	CD82_MO	Cd82	Cd82	12521	CD82 antig	no | no chan	0	ENOG4111
A2A7H4	A2A7H4_M	Fbxo44	Fbxo44		F-box prote	no | no chan	0	ENOG4111
A0A1R3UC	A0A1R3UC	Klna1	Klna1		Kallikrein c	no | no chan	0	COG5640(p
Q3TAN8	Q3TAN8_M	Tecr	Tecr	106529	Trans-2,3-e	no | up	0	ENOG410X
Q3TJZ7	Q3TJZ7_M	Jpt2	Jpt2	52009	Hematologi	yes | up	1	ENOG4111
Q9D6M3	GHC1_MO	Slc25a22	Slc25a22	68267	Mitochond	no | no chan	0	ENOG410Y
P50295	ARY2_MO	Nat2	Nat2	17961	Arylamine	no | no chan	0	COG2162(N
Q3US29	Q3US29_M	Fdps	Fdps	110196	Farnesyl py	no | no chan	0	COG0142(s
Q8CB77	ELOA1_M	Eloa	Eloa	27224	Elongin-A	no | no chan	0	ENOG411C
A2AW86	A2AW86_M	Ly75	Ly75		Lymphocyt	no | up	0	ENOG410X
Q64151	SEM4C_M	Sema4c	Sema4c	20353	Semaphorir	no | no chan	0	ENOG410X
A2AP78	A2AP78_M	Hmgb3	Hmgb3		High mobil	no | no chan	0	COG5648(C
Q8BK06	FBX9_MO	Fbxo9	Fbxo9	71538	F-box only	no | no chan	0	ENOG410X
Q4FZG9	NUA4L_M	Ndufa4l2	Ndufa4l2	407790	NADH deh	no | up	0	ENOG410X
Q8BK03	MIGA2_M	Miga2	Miga2	108958	Mitoguardi	yes | down	1	ENOG410Y
A2AP74	A2AP74_M	Cd99l2	Cd99l2	171486	CD99 antig	yes | up	1	ENOG410Y
G3UVU2	G3UVU2_M	Sf3a2	Sf3a2	20222	Splicing fac	no | no chan	0	COG5246(S
Q3U181	Q3U181_M	Panx1	Panx1	55991	Pannexin	no | up	0	ENOG410Y
Q3U182	CRTC2_M	Crtc2	Crtc2	74343	CREB-regu	no | no chan	0	ENOG410X
Q3U184	Q3U184_M	Cog8	Cog8	97484	Conserved	no | no chan	0	ENOG410X
Q3U186	SYRM_MO	Rars2	Rars2	109093	Probable a	no | no chan	0	COG0018(p
Q3TL79	Q3TL79_M	Ahsa1	Ahsa1	217737	Activator o	no | no chan	0	COG5580(u
Q9ET80	JPH1_MO	Jph1	Jph1	57339	Junctophili	no | up	0	COG4642(S
O70281	TPST1_MC	Tpst1	Tpst1	22021	Protein-tyr	yes | up	1	ENOG410X
F7CVL0	F7CVL0_M	Dot1l	Dot1l		Histone-lys	no | no chan	0	ENOG410X
Q3TCX3	KHDC4_M	Khdc4	Khdc4	74200	KH homolc	no | no chan	0	ENOG4111
Q3T9D8	Q3T9D8_M	Slc25a32	Slc25a32	69906	Solute carri	no | no chan	0	ENOG410X
A0A0A0MC	A0A0A0MC	Jmjd1c	Jmjd1c	108829	Jumonji dor	no | no chan	0	ENOG410X
A0A0A0MC	A0A0A0MC	Swi5	Swi5		DNA repai	no | no chan	0	ENOG410X
Q8BP40	PPA6_MO	Acp6	Acp6	66659	Lysophospl	no | no chan	0	ENOG410Z
A2RS63	A2RS63_M	Cuedc1	Cuedc1	103841	CUE domai	no | no chan	0	ENOG4111
A0A0A0MC	A0A0A0MC	S100a13	S100a13	20196	Protein S10	no | no chan	0	ENOG410Z

A0A338P6I	A0A338P6I	Heg1	Heg1	77446	Heart devel	no|no chan	0	ENOG410Y
E9PWI3	E9PWI3_M	Mcc	Mcc	328949	Mutated in	no|down	0	ENOG410X
A2RS68	A2RS68_M	Snrnp70	Snrnp70	20637	U1 small nu	no|no chan	0	COG0724(C
A0A0A0MC	A0A0A0MC	Arhgap12	Arhgap12	75415	Rho GTPas	no|no chan	0	ENOG410Z
E9PWI6	E9PWI6_M	Gcc2	Gcc2		GRIP and c	no|no chan	0	ENOG410Y
Q80X19	COEA1_M	Col14a1	Col14a1	12818	Collagen al	no|down	0	ENOG410Y
Q9CYY7	PLD3B_MC	Prelid3b	Prelid3b	66390	PRELI dom	yes|down	1	ENOG410Y
A2AEX0	A2AEX0_M	Kcnd1	Kcnd1	16506	Potassium v	no|no chan	0	COG1226(I
Q545V5	Q545V5_M	Polr2k	Polr2k	17749	DNA-direc	no|no chan	0	COG1996(I
Q9WUP7	UCHL5_M	Uchl5	Uchl5	56207	Ubiquitin c	no|no chan	0	ENOG410X
Q9D7A8	ARMC1_M	Armc1	Armc1	74252	Armadillo r	no|no chan	0	ENOG4111
P62960	YBOX1_M	Ybx1	Ybx1	22608	Y-box-bind	no|no chan	0	COG1278((
P62962	PROF1_MC	Pfn1	Pfn1	18643	Profilin-1	no|no chan	0	ENOG4112
P62965	RABP1_MC	Crabp1	Crabp1	12903	Cellular ret	no|no chan	0	ENOG4111
Q3TKX4	Q3TKX4_M	Ddx52	Ddx52	78394	Probable A	no|no chan	0	ENOG410X
A0A494B9F	A0A494B9F	Wdr4	Wdr4		WD repeat	no|no chan	0	ENOG4111
Q8VE42	ANR49_MC	Ankrd49	Ankrd49	56503	Ankyrin rep	no|no chan	0	COG0666(M
Q99KR7	PPIF_MOU	Ppif	Ppif	105675	Peptidyl-pr	no|no chan	0	COG0652(p
Q3U1M9	Q3U1M9_M	Adgre1	Adgre1	13733	Adhesion G	no|no chan	0	ENOG410X
Q8VE47	UBA5_MO	Uba5	Uba5	66663	Ubiquitin-l	no|no chan	0	COG0476(r
Q9JK38	GNA1_MC	Gnpnat1	Gnpnat1	54342	Glucosamin	no|no chan	0	COG0454(c
P70318	TIAR_MOU	Tial1	Tial1	21843	Nucleolysin	no|no chan	0	ENOG410X
Q8R527	RHOQ_MC	Rhoq	Rhoq	104215	Rho-related	no|down	0	COG1100(I
A0A3Q4EI	A0A3Q4EI	Crim1	Crim1		Cysteine ric	no|down	0	ENOG410Z
Q0VH02	Q0VH02_M	Cdk10	Cdk10	234854	Cyclin-depe	no|no chan	0	ENOG410X
P70313	NOS3_MO	Nos3	Nos3	18127	Nitric oxide	no|down	0	COG0369(s
P70315	WASP_MO	Was	Was	22376	Actin nucle	no|no chan	0	ENOG4111
A2CFB8	A2CFB8_M	Gp1ba	Gp1ba	14723	Glycoprote	no|up	0	COG4886(I
Q62009	POSTN_M	Postn	Postn	50706	Periostin	no|no chan	0	COG2335(I
B2KGA7	B2KGA7_M	Zbtb8os	Zbtb8os	67106	Protein arc	no|no chan	0	COG1371(C
Q5RJV4	Q5RJV4_M	Pgm1	Pgm1	72157	Phosphoglu	no|no chan	0	COG0033(p
Q9R207	NBN_MOU	Nbn	Nbn	27354	Nibrin	no|no chan	0	ENOG410Y
Q9CZV9	Q9CZV9_M	Pacc1	Pacc1	66950	Proton-acti	no|no chan	0	ENOG410X
Q9CZV8	FXL20_MC	Fbxl20	Fbxl20	72194	F-box/LRR	no|no chan	0	ENOG410X
Q497N0	Q497N0_M	Cct6b	Cct6b	12467	Chaperonin	no|down	0	ENOG410X
A0A0J9YM	A0A0J9YM	Sesn3	Sesn3		Sestrin 3	no|no chan	0	ENOG410X
Q91WD1	RPC4_MOU	Polr3d	Polr3d	67065	DNA-direc	no|no chan	0	ENOG410X
Q3KQJ0	TMM69_M	Tmem69	Tmem69	230657	Transmemb	no|no chan	0	ENOG410X
O88200	CLC11_MC	Clec11a	Clec11a	20256	C-type lecti	no|no chan	0	ENOG410Y
Q8CHS7	DRS7C_MC	Dhrs7c	Dhrs7c	68460	Dehydroger	no|up	0	COG1028(I
Q7TQ62	PODN_MC	Podn	Podn	242608	Podocan	no|down	0	COG4886(I
Q9EPQ7	STAR5_MC	Stard5	Stard5	170460	StAR-relate	no|no chan	0	ENOG411C
P53026	RL10A_MC	Rpl10a	Rpl10a	19896	Large ribos	no|no chan	0	COG0081(I

P01132	EGF_MOU	Egf	Egf	13645	Pro-epidern	no	down	0	ENOG410
Q7TSE6	ST38L_MO	Stk38l	Stk38l	232533	Serine/thre	no	no chan	0	ENOG410
Q6S385	Q6S385_M	Plec	Plec	18810	Plectin 10	no	no chan	0	COG5069(I
Q3UJR8	Q3UJR8_M	Btf3	Btf3	218490	Transcripti	no	no chan	0	ENOG4111
Q91WD7	KI18A_MC	Kif18a	Kif18a	228421	Kinesin-lik	no	no chan	0	COG5059(I
Q61382	TRAF4_MC	Traf4	Traf4	22032	TNF recept	no	down	0	ENOG410
Q3U343	Q3U343_M	Zfp622	Zfp622	52521	C2H2-type	no	no chan	0	ENOG410
Q8BJY1	PSMD5_M	Psmd5	Psmd5	66998	26S proteas	no	no chan	0	ENOG410
E9Q9D6	E9Q9D6_M	R3hdm2	R3hdm2	71750	R3H domai	no	no chan	0	ENOG411(
Q8VD31	TPSNR_MC	Tapbpl	Tapbpl	213233	Tapasin-rel	no	up	0	ENOG4111
Q6PFQ7	RASL2_MC	Rasa4	Rasa4	54153	Ras GTPas	no	no chan	0	ENOG410
A0A1Y7VK	A0A1Y7VK	Fancc	Fancc		Fanconi an	no	no chan	0	ENOG411
Q9JK37	MYOZ1_M	Myoz1	Myoz1	59011	Myozenin-1	no	up	0	ENOG4111
A0A0G2JE	A0A0G2JE	Iglc1	Iglc1		Immunoglo	no	no chan	0	ENOG4111
E9QPX6	E9QPX6_M	AW554918	AW554918		Expressed s	no	no chan	0	ENOG4111
Q80X32	CE024_MOUSE	UPF0461 protein (		78521	UPF0461 pı	no	up	0	ENOG4111
E9QPX3	E9QPX3_M	Ndufs4	Ndufs4	17993	NADH deh	no	no chan	0	ENOG4111
A2AJ72	A2AJ72_M	Fubp3	Fubp3	320267	Far upstrea	no	no chan	0	ENOG410
E9QPX1	E9QPX1_M	Col18a1	Col18a1	12822	Collagen, ty	no	no chan	0	ENOG410
B1AQ78	B1AQ78_M	Krt19	Krt19	16669	Keratin, typ	no	up	0	ENOG410
P46656	ADX_MOU	Fdx1	Fdx1	14148	Adrenodoxi	no	no chan	0	COG0633(r
Q3UXJ3	Q3UXJ3_M	Hnrnpll	Hnrnpll	72692	RRM doma	no	no chan	0	ENOG410
Q58FA4	E2F8_MOU	E2f8	E2f8	108961	Transcripti	no	no chan	0	ENOG4111
Q3THH5	Q3THH5_M	Icam1	Icam1	15894	Intercellula	no	up	0	ENOG410
A0S0D6	A0S0D6_M	Hipk1	Hipk1	15257	Homeodom	no	no chan	0	ENOG410
P52480	KPYM_MC	Pkm	Pkm	18746	Pyruvate ki	no	no chan	0	COG0469(I
Q6ZQI3	MLEC_MC	Mlec	Mlec	109154	Malectin	no	no chan	0	ENOG410
A2RTC8	A2RTC8_M	Marchf5	Marchf5	69104	E3 ubiquiti	no	no chan	0	ENOG410
D3Z4A3	D3Z4A3_M	Zfp740	Zfp740		Zinc finger	no	no chan	0	COG5048((
O89001	CBPD_MO	Cpd	Cpd	12874	Carboxypep	no	no chan	0	ENOG410
E9QJU4	E9QJU4_M	Myoz3	Myoz3	170947	Myozenin 3	no	up	0	ENOG410
B0QZL1	B0QZL1_M	Eno1	Eno1		phosphopyı	no	up	0	COG0148((
E9QJU8	E9QJU8_M	Trp53bp2	Trp53bp2	209456	Transformo	no	no chan	0	ENOG410
B0QZL5	B0QZL5_M	Pik3cd	Pik3cd		phosphatid	no	no chan	0	COG5032(p
Q91W18	TDRD3_M	Tdrd3	Tdrd3	219249	Tudor dom	no	no chan	0	ENOG4111
H3BL37	H3BL37_M	Tcof1	Tcof1	21453	Treacle rib	no	no chan	0	ENOG4111
Q4QRK2	Q4QRK2_M	Fech	Fech	14151	Fech protei	no	no chan	0	COG0276((
A1L0X5	A1L0X5_M	Krt78	Krt78	332131	Krt78 prote	no	no chan	0	ENOG4111
Q540J8	Q540J8_M	Nod1	Nod1	107607	NOD1	no	up	0	ENOG410
Q62383	SPT6H_MC	Supt6h	Supt6h	20926	Transcripti	no	no chan	0	COG2183(c
A0A1D5RL	A0A1D5RL	Gramd1b	Gramd1b		GRAM don	no	up	0	ENOG410
Q62384	ZPR1_MO	Zpr1	Zpr1	22687	Zinc finger	no	no chan	0	COG1779(z
A0A286YD	A0A286YD	Eml3	Eml3		Echinodern	no	no chan	0	ENOG410

A0A140LIT	A0A140LIT	Map6	Map6		Microtubul	no | no chan	0	ENOG411(
A0A571BEI	A0A571BEI	Aak1	Aak1		AP2 associc	no | no chan	0	ENOG410Y
A0A140LIT	A0A140LIT	Dhcr7	Dhcr7	13360	7-dehydrocl	no | no chan	0	ENOG410X
Q3TX66	Q3TX66_M	Grn	Grn	14824	Granulins c	no | no chan	0	ENOG410X
E9QK36	E9QK36_M	Wdr62	Wdr62	233064	WD repeat	no | no chan	0	ENOG410Y
Q9DBN9	DDX59_M	Ddx59	Ddx59	67997	Probable A	no | no chan	0	COG0513(I
Q8K1J6	TRNT1_M	Trnt1	Trnt1	70047	CCA tRNA	no | no chan	0	COG0617(C
Q8K1J5	SDE2_MO	Sde2	Sde2	208768	Splicing reg	no | no chan	0	ENOG4111
Q64435	UD16_MO	Ugt1a6	Ugt1a6	94284	UDP-glucu	no | down	0	COG1819(S
Q8K203	NEIL3_MC	Neil3	Neil3	234258	Endonuclec	no | no chan	0	ENOG410Z
E0CX53	E0CX53_M	Tbc1d4	Tbc1d4	210789	TBC1 domc	no | no chan	0	ENOG410Y
Q9DBN5	LONP2_M	Lonp2	Lonp2	66887	Lon protea	no | no chan	0	COG0466(A
Q640P2	ANGL1_M	Angptl1	Angptl1	72713	Angiopoieti	no | up	0	ENOG410Z
Q62120	JAK2_MOU	Jak2	Jak2	16452	Tyrosine-pr	no | no chan	0	COG0515(s
Q00993	UFO_MOU	Axl	Axl	26362	Tyrosine-pr	no | no chan	0	COG0515(c
P54775	PRS6B_MC	Psmc4	Psmc4	23996	26S proteas	no | no chan	0	COG1222(I
A0A1S6GW	A0A1S6GW	Mff	Mff	75734	Mitochondı	no | no chan	0	ENOG410X
B2RQE8	RHG42_M	Arhgap42	Arhgap42	71544	Rho GTPas	no | no chan	0	ENOG410Y
A0A0G2JD	A0A0G2JD	Hax1	Hax1		HCLS1-assc	no | no chan	0	ENOG4112
G3XA59	LRC32_MC	Lrrc32	Lrrc32	434215	Transformi	no | down	0	COG4886(I
G3XA57	RFIP2_MO	Rab11fip2	Rab11fip2	74998	Rab11 fami	no | up	0	ENOG410Z
A0A0R4J05	A0A0R4J05	Acy1	Acy1	109652	N-acyl-alip	no | no chan	0	COG0624(C
Q3U5I9	Q3U5I9_M	Prkab1	Prkab1	19079	5'-AMP-act	no | no chan	0	ENOG410X
A0A0R4J05	A0A0R4J05	Dnmbp	Dnmbp	71972	Dynamin-b	no | no chan	0	ENOG410X
Q80VH1	Q80VH1_M	Snw1	Snw1	66354	SNW doma	no | up	0	ENOG410X
P33435	MMP13_M	Mmp13	Mmp13	17386	Collagenas	no | up	0	ENOG410X
P33434	MMP2_MC	Mmp2	Mmp2	17390	72 kDa typ	no | no chan	0	ENOG410X
F6S062	F6S062_MC	Chka	Chka		ethanolami	no | no chan	0	COG0510(I
A0A087WR	A0A087WR	Mndal	Mndal		Myeloid nu	no | up	0	ENOG410Y
P40936	INMT_MO	Inmt	Inmt	21743	Indolethyla	no | down	0	ENOG4112
E0CXN0	E0CXN0_M	Mst1	Mst1		Macrophag	no | no chan	0	COG5640(g
A0A0M3HI	A0A0M3HI	Lias	Lias	79464	Lipoyl syntl	no | no chan	0	COG0320(C
A0A0M3HI	A0A0M3HI	Atp8a1	Atp8a1	11980	Phospholip	no | no chan	0	ENOG410X
A0A0M3HI	A0A0M3HI	Cadps2	Cadps2		Ca2+-deper	no | no chan	0	ENOG410Y
Q80XL7	MPPE1_M	Mppe1	Mppe1	225651	Metallopho	no | up	0	ENOG410Z
Q80XL9	Q80XL9_M	Fam53c	Fam53c	66306	Fam53c prc	no | down	0	ENOG410Y
P97450	ATP5J_MO	Atp5pf	Atp5pf	11957	ATP syntha	no | no chan	0	ENOG4112
E9PZS8	E9PZS8_M	Cox7a2l	Cox7a2l	20463	Cytochrom	no | no chan	0	ENOG4112
O35226	PSMD4_M	Psmd4	Psmd4	19185	26S proteas	no | no chan	0	COG5148(2
A2CGA5	A2CGA5_M	Birc3	Birc3	11796	Baculoviral	no | no chan	0	ENOG410Y
Q6NV83	SR140_MO	U2surp	U2surp	67958	U2 snRNP-	no | no chan	0	ENOG410X
G5E8B1	G5E8B1_M	Ptpn13	Ptpn13	19249	Tyrosine-pr	no | no chan	0	COG5599(S
O88848	ARL6_MO	Arl6	Arl6	56297	ADP-ribosy	no | no chan	0	COG1100(A

Q3TTJ8	Q3TTJ8_M	Gramd2b	Gramd2b	107022	GRAM don	no	no chan	0	ENOG410
E9PUF7	E9PUF7_M	Arhgef1	Arhgef1	16801	Rho guanin	no	no chan	0	ENOG410
G5E8B9	ZBT11_MC	Zbtb11	Zbtb11	271377	Zinc finger	no	no chan	0	COG5048(I
O88844	IDHC_MO	Idh1	Idh1	15926	Isocitrate d	no	no chan	0	COG0538(i
O88843	CRADD_M	Cradd	Cradd	12905	Death dom	no	no chan	0	ENOG4111
B6YY24	B6YY24_M	Fam91a1	Fam91a1	210998	Skeletal mu	no	no chan	0	ENOG410
P61600	NAA20_M	Naa20	Naa20	67877	N-alpha-ac	no	no chan	0	COG0456(I
P10852	4F2_MOUS	Slc3a2	Slc3a2	17254	Amino acid	no	no chan	0	COG0366(c
Q8JZP9	GA2L1_M	Gas2l1	Gas2l1	78926	GAS2-like p	no	no chan	0	ENOG410
Q3U1G4	Q3U1G4_M	Exoc1	Exoc1	69940	Exocyst cor	no	no chan	0	ENOG410
Q3UEQ9	Q3UEQ9_M	Aass	Aass	30956	Uncharacte	no	no chan	0	COG1748(s
F7AA26	F7AA26_M	Pakap	Pakap		Paralemmi	no	no chan	0	ENOG410
E9PUZ5	E9PUZ5_M	Pick1	Pick1	18693	PRKCA-bir	no	no chan	0	ENOG410
E9PUZ3	E9PUZ3_M	U2af1l4	U2af1l4		U2 small nu	no	no chan	0	ENOG410
Q9Z188	DYR1B_M	Dyrk1b	Dyrk1b	13549	Dual specif	no	down	0	ENOG410
P19536	COX5B_M	Cox5b	Cox5b	12859	Cytochrom	no	no chan	0	ENOG4111
Q3USZ8	DIK2A_M	Dipk2a	Dipk2a	68861	Divergent p	no	no chan	0	ENOG410
B2RX33	B2RX33_M	Steap2	Steap2	74051	Six transme	no	no chan	0	COG2085(C
Q99JF8	PSIP1_MO	Psip1	Psip1	101739	PC4 and SF	no	no chan	0	ENOG410
Q6Y5D8	RHG10_M	Arhgap10	Arhgap10	78514	Rho GTPas	no	no chan	0	ENOG410
A2RSH1	A2RSH1_M	Pde4d	Pde4d	238871	Phosphodie	no	no chan	0	
I3ITR1	I3ITR1_M	AK157302	AK157302	432732	Iron-sulfur	no	no chan	0	COG0316(I
D3Z2N1	D3Z2N1_M	Ankrd9	Ankrd9		Ankyrin rep	no	no chan	0	COG0666(I
Q9D219	BCL9_MO	Bcl9	Bcl9	77578	B-cell CLL/	no	no chan	0	ENOG410
Q6YJU2	Q6YJU2_M	Fetub	Fetub	59083	GUGU alp	no	up	0	ENOG4111
Q9D211	C2AIL_MC	Cdkn2aipnl	Cdkn2aipnl	52626	CDKN2AII	yes	up	1	ENOG4111
Q9JHF7	HPGDS_M	Hpgds	Hpgds	54486	Hematopoi	no	no chan	0	ENOG4111
Q3TF62	Q3TF62_M	Actl6a	Actl6a	56456	Actin-like p	no	no chan	0	COG5277(c
Q3USZ7	Q3USZ7_M	Net1	Net1	56349	Neuroepith	no	no chan	0	COG5422(I
O35955	PSB10_MO	Psmb10	Psmb10	19171	Proteasome	no	up	0	COG0638(f
O35954	PITM1_MC	Pitpnm1	Pitpnm1	18739	Membrane-	no	no chan	0	COG5083(f
Q8VD04	GRAP1_M	Gripap1	Gripap1	54645	GRIP1-assc	no	no chan	0	ENOG410
A0A0N4SV	A0A0N4SV	Asb3	Asb3		Ankyrin rep	no	no chan	0	COG0666(I
A0A0N4SV	A0A0N4SV	Clec1a	Clec1a	243653	C-type lecti	yes	down	1	ENOG410
Q9QXT0	CNPY2_M	Cnpy2	Cnpy2	56530	Protein can	no	no chan	0	ENOG4111
Q9CXW2	RT22_MO	Mrps22	Mrps22	64655	Small ribos	no	no chan	0	ENOG4111
Q9CXW3	CYBP_MO	Cacybp	Cacybp	12301	Calcyclin-b	no	no chan	0	ENOG410
Q9CXW4	RL11_MO	Rpl11	Rpl11	67025	Large ribos	no	no chan	0	COG0094(T
Q99L04	DHRS1_M	Dhrs1	Dhrs1	52585	Dehydroger	no	no chan	0	COG1028(I
Q3U9U5	Q3U9U5_M	Bcap31	Bcap31	27061	B-cell recep	no	no chan	0	COG5374(r
P11672	NGAL_MC	Lcn2	Lcn2	16819	Neutrophil	no	up	0	ENOG4111
Q14C51	PTCD3_M	Ptcd3	Ptcd3	69956	Small ribos	no	no chan	0	ENOG410
Q80VY9	DHX33_M	Dhx33	Dhx33	216877	ATP-depen	no	no chan	0	COG1643(U

Q3UMT1	PP12C_MC	Ppp1r12c	Ppp1r12c	232807	Protein phc	no	no chan	0	COG0666(U
Q9ESP1	SDF2L_MC	Sdf2l1	Sdf2l1	64136	Stromal cel	no	up	0	ENOG410Z
Q3UMT7	Q3UMT7_M	Hnrnpl	Hnrnpl	15388	RRM doma	no	no chan	0	ENOG410X
Q8CCH2	NHLC3_M	Nhlrc3	Nhlrc3	212114	NHL repea	no	no chan	0	ENOG410Z
Q9D9M5	PHOP2_M	Phospho2	Phospho2	73373	Pyridoxal p	no	no chan	0	ENOG4111
B7ZNJ8	B7ZNJ8_M	Rhbdd3	Rhbdd3	279766	Rhbdd3 prc	no	no chan	0	ENOG411C
Q9D9M2	UBP12_MC	Usp12	Usp12	22217	Ubiquitin c	no	no chan	0	ENOG410X
P51612	XPC_MOU	Xpc	Xpc	22591	DNA repai	no	no chan	0	COG5535(r
Q8CD26	S35E1_MO	Slc35e1	Slc35e1	270066	Solute carri	no	no chan	0	ENOG410X
Q2L4X1	5MP1_MUS	Bzw2	Bzw2	66912	eIF5-mimic	no	no chan	0	ENOG410X
Q3TXN0	Q3TXN0_M	Htra2	Htra2	64704	PDZ domai	no	no chan	0	COG0265(F
Q3TXN1	Q3TXN1_M	Ptgr2	Ptgr2	77219	Prostaglanc	no	no chan	0	COG2130(C
A0A3B2WC	A0A3B2WC	Ranbp3	Ranbp3	71810	RAN bindi	no	no chan	0	ENOG4111
Q1KYM0	Q1KYM0_MOUSE	Env polyprotei		17276	Env polypr	no	no chan	0	ENOG4111
P27870	VAV_MOU	Vav1	Vav1	22324	Proto-onco	no	no chan	0	ENOG410X
Q3UHK6	TEN4_MO	Tenm4	Tenm4	23966	Teneurin-4	no	no chan	0	ENOG410Z
Q9D0K0	TBCD7_M	Tbc1d7	Tbc1d7	67046	TBC1 domc	no	no chan	0	ENOG410Y
A0A1B0GQ	A0A1B0GQ	Stim1	Stim1		Stromal int	no	no chan	0	ENOG410X
B7ZCU0	B7ZCU0_M	Abi1	Abi1	11308	Abl interac	no	no chan	0	ENOG410Y
Q8BTG3	T11L1_MO	Tcp11l1	Tcp11l1	320554	T-complex	no	no chan	0	ENOG410Z
A0A140LJF	A0A140LJF	Aldh3a2	Aldh3a2		aldehyde de	no	no chan	0	COG1012(I
A0A140LJF	A0A140LJF	Ryr3	Ryr3		Ryanodine	no	no chan	0	ENOG410Y
A0A068EW	A0A068EW	Pml	Pml	18854	Promyelocy	no	no chan	0	ENOG4111
Q3TMZ1	Q3TMZ1_M	Pycr2	Pycr2	69051	Pyrroline-5	no	no chan	0	COG0345(p
Q8C878	UBA3_MO	Uba3	Uba3	22200	NEDD8-ac	no	no chan	0	COG0476(M
B2RRA5	B2RRA5_M	Rnf135	Rnf135	71956	Ring finger	no	no chan	0	ENOG4111
A0A7G9TM	A0A7G9TM	gag	gag		Gag	no	no chan	0	
A0A0R4J25	A0A0R4J25	Ei24	Ei24	13663	Etoposide i	no	no chan	0	ENOG410X
Q9JHI5	IVD_MOU	Ivd	Ivd	56357	Isovaleryl-C	no	no chan	0	ENOG410X
Q9JHI7	EXOS9_M	Exosc9	Exosc9	50911	Exosome cc	no	no chan	0	COG2123(F
A0A1Z4EA	A0A1Z4EA	B4galnt1	B4galnt1	14421	Beta-1,4-N-	no	no chan	0	ENOG4111
A0A2I3BPC	A0A2I3BPC	Pde1b	Pde1b	18574	Phosphodie	no	up	0	ENOG410X
Q3U8Y1	Q3U8Y1_M	Mrps11	Mrps11	67994	Mitochond	no	no chan	0	COG0100(e
Q3U8Y7	Q3U8Y7_M	Psmc3	Psmc3	19182	AAA+ ATF	no	no chan	0	COG1222(Z
A0A2I3BPC	A0A2I3BPC	Ppp3cc	Ppp3cc	19057	Serine/thre	no	no chan	0	COG0639(F
F8WJ31	F8WJ31_M	Zscan26	Zscan26	432731	Zinc finger	no	no chan	0	
A0A0G2JG	A0A0G2JG	Dclk1	Dclk1		non-specific	no	no chan	0	ENOG410Y
A0A0G2JG	A0A0G2JG	Nub1	Nub1	53312	Negative re	no	no chan	0	ENOG410Z
Q9Z2Y3	HOME1_M	Homer1	Homer1	26556	Homer prot	no	no chan	0	ENOG410X
Q3USB7	PLCL1_MC	Plcl1	Plcl1	227120	Inactive ph	no	no chan	0	ENOG410X
Q9ERR1	NDEL1_M	Ndel1	Ndel1	83431	Nuclear dis	no	no chan	0	ENOG410X
E9QLJ0	E9QLJ0_M	Cmya5	Cmya5	76469	Cardiomyo	no	no chan	0	ENOG410Y
Q6NZB1	ANM6_MC	Prmt6	Prmt6	99890	Protein arg	no	no chan	0	ENOG410X

O54824	IL16_MOU	Il16	Il16	16170	Pro-interleι	no	no chan	0	ENOG411(
O54825	BYST_MOU	Bysl	Bysl	53414	Bystin	no	no chan	0	ENOG410Σ
Q3UUQ7	PGAP1_M(	Pgap1	Pgap1	241062	GPI inosito	no	no chan	0	ENOG410Y
Q9JJQ0	PIGB_MOU	Pigb	Pigb	55981	GPI mannc	no	no chan	0	ENOG410Σ
P59024	FKB14_MC	Fkbp14	Fkbp14	231997	Peptidyl-pr	no	down	0	COG0545(t
A0A2Y9CZ	A0A2Y9CZ	Rab44	Rab44	442827	RAB44, me	no	no chan	0	ENOG410Σ
O88543	CSN3_MOU	Cops3	Cops3	26572	COP9 signc	no	no chan	0	ENOG410Σ
Q8BGH7	C42S2_MO	Cdc42se2	Cdc42se2	72729	CDC42 smc	no	no chan	0	ENOG4112
O88544	CSN4_MOU	Cops4	Cops4	26891	COP9 signc	no	no chan	0	ENOG410Σ
P08607	C4BPA_M(	C4bpa	C4bpa	12269	C4b-bindin	no	no chan	0	ENOG410Σ
B1AX98	B1AX98_M	Lrrc47	Lrrc47	72946	Leucine ricl	no	no chan	0	COG0072(f
G3X972	G3X972_M	Sec24c	Sec24c	218811	SEC24 hom	no	no chan	0	COG5028(s
G3X977	G3X977_M	Itih2	Itih2	16425	Inter-alpha	no	no chan	0	COG2304(s
B1ARC2	B1ARC2_M	Slc39a11	Slc39a11		Zinc transp	no	no chan	0	COG0428(I
Q8VCG9	RFXAP_M	Rfxap	Rfxap	170767	Regulatory	no	no chan	0	ENOG4111
Q3UV38	Q3UV38_M	Tpcn1	Tpcn1	252972	Ion transpo	no	no chan	0	ENOG410Σ
Q99P72	RTN4_MO	Rtn4	Rtn4	68585	Reticulon-4	no	up	0	ENOG410Σ
Q8VCG3	WDR74_M	Wdr74	Wdr74	107071	WD repeat-	no	no chan	0	ENOG410Y
Q8VCG4	CO8G_MC	C8g	C8g	69379	Complemer	no	down	0	ENOG4111
Q9CVB6	ARPC2_M(	Arpc2	Arpc2	76709	Actin-relat	no	no chan	0	ENOG410Y
Q61268	APOC4_M(	Apoc4	Apoc4	11425	Apolipopro	no	no chan	0	ENOG4111
P35821	PTN1_MO	Ptpn1	Ptpn1	19246	Tyrosine-pr	no	no chan	0	COG5599(t
D3YU39	D3YU39_M	Chpt1	Chpt1	212862	Cholinephc	no	no chan	0	COG5050(I
B2KFS7	B2KFS7_M	Klhl42	Klhl42	232539	Kelch dom	no	no chan	0	ENOG411(
Q3UIM5	Q3UIM5_M	Cpt1b	Cpt1b	12895	Carnitine C	no	up	0	ENOG410Σ
Q62469	ITA2_MOU	Itga2	Itga2	16398	Integrin alp	no	no chan	0	ENOG410Σ
Q8BHJ9	SLU7_MOU	Slu7	Slu7	193116	Pre-mRNA	no	no chan	0	ENOG410Σ
Q8BGZ3	DCA12_M(	Dcaf12	Dcaf12	68970	DDB1- and	no	no chan	0	ENOG410Σ
Q4VAI2	Q4VAI2_M	Acp1	Acp1	11431	Low molecι	no	no chan	0	COG0394(I
Q8BHJ5	TBL1R_MC	Tbl1xr1	Tbl1xr1	81004	F-box-like/	no	no chan	0	COG2319(I
A2ALR9	A2ALR9_M	Usp48	Usp48	170707	Ubiquitin c	no	no chan	0	COG5077(t
B1AZ39	B1AZ39_M	Chmp6	Chmp6	208092	Chromatin	no	down	0	ENOG4111
Q53YU5	Q53YU5_M	Nol3	Nol3	78688	Apoptosis r	no	no chan	0	ENOG4111
P15535	B4GT1_M(	B4galt1	B4galt1	14595	Beta-1,4-ga	no	no chan	0	ENOG410Z
P62737	ACTA_MO	Acta2	Acta2	11475	Actin, aorti	no	no chan	0	COG5277((
Q8BPK2	ZCHC3_M(	Zcchc3	Zcchc3	67917	Zinc finger	no	no chan	0	ENOG4112
P15532	NDKA_M(	Nme1	Nme1	18102	Nucleoside	no	no chan	0	COG0105(N
P15533	TR30A_MC	Trim30a	Trim30a	20128	Tripartite n	no	up	0	ENOG410Z
Q8BX05	GLPK5_M(	Gk5	Gk5	235533	Glycerol ki	no	no chan	0	COG0554(p
J3JS86	J3JS86_MO	Cep112	Cep112		Centrosomo	no	no chan	0	ENOG410Z
Q8BX02	KANK2_M	Kank2	Kank2	235041	KN motif a	no	no chan	0	COG0666(p
Q9EQ20	MMSA_MC	Aldh6a1	Aldh6a1	104776	Methylmal	no	down	0	ENOG410Σ
A0A0R4J20	A0A0R4J20	Trmt10a	Trmt10a		tRNA meth	no	no chan	0	ENOG4111

A0A0R4J20	A0A0R4J20	Mocs2	Mocs2	17434	Molybdopte	no | no chan	0	COG0314(M
A0A0R4J0C	A0A0R4J0C	Pck2	Pck2	74551	phosphoen	no | no chan	0	COG1274(C
A0A0R4J0C	A0A0R4J0C	Dlgap5	Dlgap5	218977	DLG associ	no | no chan	0	ENOG4111
A0A0R4J0C	A0A0R4J0C	Qpct	Qpct	70536	Glutaminyl	yes | up	1	ENOG410Y
A0A0R4J0C	A0A0R4J0C	Parpbp	Parpbp	75317	PCNA-inte	no | down	0	ENOG4111
A0A0R4J0C	A0A0R4J0C	Ranbp10	Ranbp10	74334	RAN bindi	no | no chan	0	ENOG410X
Q8C4U3	SFRP1_MC	Sfrp1	Sfrp1	20377	Secreted fri	no | no chan	0	ENOG410X
A0A0R4J0C	A0A0R4J0C	Mtrr	Mtrr	210009	Methionine	no | no chan	0	COG0369(P
A0A338P6Y	A0A338P6Y	Alg3	Alg3		Dol-P-Man	no | no chan	0	ENOG410X
B9EHL0	B9EHL0_M	Mier3	Mier3	218613	Mier3 prote	no | up	0	ENOG410Y
A2AUE1	A2AUE1_M	Dnajc5	Dnajc5		DnaJ homo	no | no chan	0	COG0484(s
Q80UY2	KCMF1_M	Kcmf1	Kcmf1	74287	E3 ubiquiti	no | no chan	0	ENOG410X
Q80UY1	CARME_M	Carnmt1	Carnmt1	67383	Carnosine N	no | no chan	0	ENOG410Z
Q6PGH1	BUD31_MO	Bud31	Bud31	231889	Protein BU	no | no chan	0	COG5132(I
P68404	KPCB_MO	Prkcb	Prkcb	18751	Protein kin	no | no chan	0	ENOG410X
Q3UNI1	Q3UNI1_M	Sirt1	Sirt1	93759	Sirtuin 1	no | no chan	0	COG0846(M
A0A1L1SSF	A0A1L1SSF	Adpgk	Adpgk	72141	ADP-depen	no | no chan	0	ENOG410Z
Q8R3Q6	MIX23_MC	Mix23	Mix23	381045	Protein MI	no | no chan	0	ENOG4111
Q9CY94	PSF3_MOU	Gins3	Gins3	78833	DNA replic	no | no chan	0	COG5138(I
B1ATU5	B1ATU5_M	Dus1l	Dus1l		tRNA-dihy	no | no chan	0	COG0042(T
E9QP36	E9QP36_M	Plekhn1	Plekhn1	231002	Pleckstrin h	yes | up	1	ENOG4111
Q9WV34	MPP2_MO	Mpp2	Mpp2	50997	MAGUK p	no | no chan	0	COG0194(F
A0A0D2X7	A0A0D2X7	Eef1akmt1	Eef1akmt1	68043	EEF1A lysi	no | no chan	0	ENOG4111
Q9CQ88	TSN31_MC	Tspan31	Tspan31	67125	Tetraspanir	yes | up	1	ENOG4111
A8Y5F6	A8Y5F6_M	Pdpn	Pdpn	14726	Podoplanin	no | no chan	0	ENOG4111
Q3U4X8	Q3U4X8_M	Lig1	Lig1	16881	DNA ligase	no | no chan	0	COG1793(M
A0A140T8C	A0A140T8C	Igkv6-17	Igkv6-17	667865	Immunoglo	no | no chan	0	ENOG410Y
Q922R5	P4R3B_MC	Ppp4r3b	Ppp4r3b	104570	Serine/thre	no | no chan	0	ENOG410X
Q61846	MELK_MC	Melk	Melk	17279	Maternal en	no | no chan	0	COG0515(C
Q922R1	PHAF1_MC	Phaf1	Phaf1	234678	Phagosome	no | no chan	0	ENOG410X
Q8R034	APC13_MC	Anapc13	Anapc13	69010	Anaphase-p	no | no chan	0	ENOG4111
Q64127	TIF1A_MC	Trim24	Trim24	21848	Transcripti	no | no chan	0	COG5076(s
Q8R033	LYRM2_MC	Lyrm2	Lyrm2	108755	LYR motif-	no | no chan	0	ENOG4112
Q571D3	Q571D3_M	Zbtb40	Zbtb40	230848	MKIAA047	no | no chan	0	COG5048(c
E9Q6H8	E9Q6H8_M	Plekha5	Plekha5	109135	Pleckstrin h	no | no chan	0	ENOG4111
Q9DA08	SGF29_MC	Sgf29	Sgf29	75565	SAGA-asso	no | no chan	0	ENOG410X
M0QWU0	M0QWU0_	Cxxc4	Cxxc4	319478	CXXC-type	no | down	0	ENOG4111
A0A5F8MP	A0A5F8MP	Qtrt2	Qtrt2	106248	Queuine tR	no | no chan	0	COG0343(I
A0A5F8MP	A0A5F8MP	Fry	Fry		FRY microt	no | no chan	0	ENOG410X
Q8K2P7	S38A1_MO	Slc38a1	Slc38a1	105727	Sodium-cou	no | no chan	0	COG0814(E
A0A0J9YV4	A0A0J9YV4	Slbp	Slbp		Stem-loop b	no | no chan	0	ENOG4112
Q9D2C9	SNPC3_MC	Snapc3	Snapc3	77634	snRNA-act	no | no chan	0	ENOG411C
Q3U2B7	Q3U2B7_M	Atp11b	Atp11b	76295	Phospholip	no | no chan	0	ENOG410X

Q6P2K2	Q6P2K2_M	Acot2	Acot2	171210	Acyl-CoA t	no	up	0	COG1073(I
Q6A0D1	Q6A0D1_M	Emc2	Emc2	66736	ER membr	no	no chan	0	ENOG410Y
Q6A0D4	RFTN1_MO	Rftn1	Rftn1	76438	Raftlin	no	no chan	0	ENOG410Z
A2CF88	A2CF88_M	Alox15	Alox15	11687	Polyunsatu	no	down	0	ENOG410Y
P97864	CASP7_MO	Casp7	Casp7	12369	Caspase-7	no	no chan	0	ENOG410Z
P97868	RBBP6_MO	Rbbp6	Rbbp6	19647	E3 ubiquiti	no	no chan	0	COG5222(I
A2CF85	A2CF85_M	Alox12	Alox12	11684	Arachidono	no	no chan	0	ENOG410Y
B7ZNX2	B7ZNX2_M	Anapc1	Anapc1	17222	Anaphase p	no	no chan	0	ENOG410X
B7ZNX0	B7ZNX0_M	Aff4	Aff4	93736	Aff4 protei	no	no chan	0	ENOG410X
B7ZNX8	B7ZNX8_M	Cep128	Cep128	75216	Centrosom	no	no chan	0	ENOG410X
F6R7Y0	F6R7Y0_M	Slc35a1	Slc35a1		Solute carri	no	no chan	0	COG0697(c
Q6ZWZ2	UB2R2_MO	Ube2r2	Ube2r2	67615	Ubiquitin-c	no	no chan	0	COG5078(T
Q9D0Y8	RM52_MO	Mrpl52	Mrpl52	68836	Large ribos	no	no chan	0	ENOG4111
Q91WA1	TIPIN_MO	Tipin	Tipin	66131	TIMELESS	no	no chan	0	ENOG4111
J3QPR1	J3QPR1_M	Atxn1	Atxn1	20238	Ataxin 1	no	no chan	0	ENOG410X
Q8CB44	GRAM4_M	Gramd4	Gramd4	223752	GRAM dom	no	no chan	0	ENOG410Z
A4UUI2	A4UUI2_M	Gbp2b	Gbp2b	14468	Guanylate l	no	up	0	ENOG410X
F6S185	F6S185_MO	Pik3ip1	Pik3ip1		Phosphoino	no	up	0	ENOG411Z
A6H5X4	PHF11_MO	Phf11	Phf11	219132	PHD finger	no	no chan	0	COG2940(I
Q3UYV9	NCBP1_MO	Ncbp1	Ncbp1	433702	Nuclear cap	no	no chan	0	ENOG410X
B2RS36	B2RS36_MO	Acd	Acd	497652	Adrenocort	no	up	0	ENOG410Y
P11531	DMD_MO	Dmd	Dmd	13405	Dystrophin	no	up	0	COG5069(C
Q8CIN4	PAK2_MO	Pak2	Pak2	224105	Serine/thre	no	no chan	0	ENOG410X
B2RS30	B2RS30_MO	Trip6	Trip6	22051	Thyroid ho	no	no chan	0	ENOG410Y
Q9D8Y7	TP8L2_MO	Tnfaip8l2	Tnfaip8l2	69769	Tumor necr	no	no chan	0	ENOG4111
Q9DC29	ABCB6_MO	Abcb6	Abcb6	74104	ATP-bindin	no	no chan	0	COG5265(i
Q9D3K4	Q9D3K4_M	Spint1	Spint1	20732	Kunitz-type	no	up	0	ENOG410X
Q8C6L5	CGAS_MO	Cgas	Cgas	214763	Cyclic GMP	no	up	0	ENOG410X
A0A1B0GR	A0A1B0GR	Anapc15	Anapc15		Anaphase p	no	no chan	0	ENOG410Y
P61148	FGF1_MO	Fgf1	Fgf1	14164	Fibroblast g	no	no chan	0	ENOG4111
F8WHM5	F8WHM5_	Glg1	Glg1		Golgi appa	no	no chan	0	ENOG410X
A0A140LJ8	A0A140LJ8	Gemin7	Gemin7		Gem nucle	no	no chan	0	ENOG410X
Q60973	RBBP7_MO	Rbbp7	Rbbp7	245688	Histone-bin	no	no chan	0	ENOG410X
H3BKH6	H3BKH6_M	Esd	Esd	13885	S-formylglu	no	no chan	0	COG0627(e
A0A087WN	A0A087WN	Lrrfip1	Lrrfip1		Leucine ric	no	no chan	0	ENOG4111
H3BKH2	H3BKH2_M	Mia2	Mia2		MIA SH3 d	no	no chan	0	ENOG410Z
A2AR76	A2AR76_M	Tank	Tank		TRAF fami	no	no chan	0	ENOG410Y
Q61818	RAI1_MOU	Rai1	Rai1	19377	Retinoic ac	no	no chan	0	ENOG410Z
F6RQ26	F6RQ26_M	Ntm	Ntm		Neurotrimi	no	up	0	ENOG410X
Q78YY6	DJC15_MO	Dnajc15	Dnajc15	66148	DnaJ homo	no	no chan	0	COG2214(I
B2RQ57	B2RQ57_M	Abl2	Abl2	11352	Tyrosine-pr	no	no chan	0	COG0515(S
Q8VEK2	RHBD2_M	Rhbdd2	Rhbdd2	215160	Rhomboid d	no	no chan	0	ENOG411C
Q3U6N9	CH033_MOUSE	UPF0488 protein C		117171	UPF0488 pr	no	no chan	0	ENOG410X

Q8BFY9	TNPO1_M(	Tnpo1	Tnpo1	238799	Transportir	no	no chan	0	ENOG410)
O09110	MP2K3_M(	Map2k3	Map2k3	26397	Dual specif	no	no chan	0	ENOG410)
A0A1R3UC	A0A1R3UC	Klk1b4	Klk1b4	18048	Kallikrein (	no	no chan	0	COG5640(p
Q8BFY6	PEF1_MOU	Pef1	Pef1	67898	Peflin	no	no chan	0	ENOG410Y
Q8BFY7	PIMRE_M(	Pimreg	Pimreg	109212	Protein PIN	no	no chan	0	ENOG4111
A0A668KL	A0A668KL	Esrra	Esrra		Estrogen re	no	no chan	0	ENOG410)
Q3TJX0	Q3TJX0_M	Lcp1	Lcp1	18826	Plastin-2	no	no chan	0	COG5069(C
Q3UN19	Q3UN19_M	Tmod4	Tmod4	50874	Tropomodu	no	no chan	0	ENOG410Y
F6RP29	F6RP29_M(	Rnaseh2a	Rnaseh2a		Ribonuclea	no	no chan	0	COG0164(I
Q8CB16	Q8CB16_M	Palm	Palm	18483	Paralemmi	no	no chan	0	ENOG410Z
O35566	CD151_MC	Cd151	Cd151	12476	CD151 anti	no	no chan	0	ENOG4111
A0A1L1SQ	A0A1L1SQ	Ccdc159	Ccdc159		Coiled-coil	no	no chan	0	ENOG4111
P23188	FURIN_M(	Furin	Furin	18550	Furin	no	no chan	0	COG4935(p
A0A3B2WA	A0A3B2WA	Anks1	Anks1		Ankyrin rep	no	no chan	0	COG0666(I
A0A2R8W6	A0A2R8W6	Pcbp2	Pcbp2		Poly(rC) bir	no	no chan	0	ENOG410)
Q3U3Y1	Q3U3Y1_M	Arel1	Arel1	68497	HECT-type	no	no chan	0	COG5021(U
Q3TCZ2	Q3TCZ2_M	Slc29a1	Slc29a1	63959	Equilibrati	no	down	0	ENOG410Y
A0A3Q4EI	A0A3Q4EI	Zfp521	Zfp521		Zinc finger	no	no chan	0	COG5048(e
Q3TDN2	FAF2_MOU	Faf2	Faf2	76577	FAS-associ	no	no chan	0	ENOG410)
P83887	TBG1_MO	Tubg1	Tubg1	103733	Tubulin ga	no	no chan	0	COG5023(I
D9J303	D9J303_M(	Pdlim5	Pdlim5	56376	ENH isofor	no	up	0	ENOG410)
Q3TDN8	Q3TDN8_M	Bphl	Bphl	68021	AB hydrola	no	no chan	0	ENOG410Y
P83882	RL36A_MC	Rpl36a	Rpl36a	66483; 1998	Large ribos	no	no chan	0	COG1631(I
B9V192	B9V192_M(	Nd5	Nd5		NADH-ubi	no	no chan	0	COG1009(k
P48441	IDUA_MO	Idua	Idua	15932	Alpha-L-id	no	no chan	0	ENOG410)
Q3T9B4	Q3T9B4_M	Rexo2	Rexo2	104444	Exonucleas	no	no chan	0	COG1949(3
Q3UGP8	AG10B_M(	Alg10b	Alg10b	380959	Dol-P-Glc:(	no	no chan	0	ENOG410Y
Q3UGP9	LRC58_MC	Lrrc58	Lrrc58	320184	Leucine-ric	no	up	0	COG4886(C
Q9ET66	PI16_MOU	Pi16	Pi16	74116	Peptidase ii	no	no chan	0	COG2340(S
Q3UGP2	Q3UGP2_M	Fbh1	Fbh1	50755	DNA 3'-5' I	no	no chan	0	COG0210(I
Q923B6	STEA4_MC	Steap4	Steap4	117167	Metalloredu	no	down	0	COG2085(M
A0A0A0M(	A0A0A0M(	Kmt2d	Kmt2d	381022	[histone H3	no	no chan	0	COG2940(I
A0A0A0M(	A0A0A0M(	Slc25a35	Slc25a35		Solute carri	no	down	0	ENOG410)
A2RRJ6	A2RRJ6_M(	Mib1	Mib1	225164	RING-type	no	no chan	0	COG0666(C
A0A338P6F	A0A338P6F	Hmga1	Hmga1	15361	High mobil	no	no chan	0	ENOG410Z
A0A338P6F	A0A338P6F	Robo1	Robo1		Roundabou	no	up	0	ENOG410)
Q6PA06	ATLA2_M(	Atl2	Atl2	56298	Atlastin-2	no	no chan	0	ENOG410)
A0A0A0M(	A0A0A0M(	Asb1	Asb1	65247	Ankyrin rep	no	no chan	0	COG0666(S
G3X8R4	G3X8R4_M	Gtf2h1	Gtf2h1	14884	General tra	no	no chan	0	ENOG410)
A0A0N4SW	A0A0N4SW	Foxj2	Foxj2		Forkhead b	no	no chan	0	COG5025(T
A0A2I3BRJ	A0A2I3BRJ	6030458C11	6030458C11Rik		RIKEN cD	no	no chan	0	ENOG411(
A0A0A0U8	A0A0A0U847_MOUSE	Envelope protein OS=I			Envelope p	no	down	0	ENOG4111
P13020	GELS_MO	Gsn	Gsn	227753	Gelsolin	no	no chan	0	ENOG410)

Q3UYN1	Q3UYN1_M	Plpbp	Plpbp	114863	Pyridoxal p	no|no chan	0	COG0325(c
Q8C196	CPSM_MO	Cps1	Cps1	227231	Carbamoyl-	no|no chan	0	COG0458(c
P70335	ROCK1_M	Rock1	Rock1	19877	Rho-associc	no|no chan	0	ENOG410X
Q3U1C4	SEBP2_MC	Secisbp2	Secisbp2	75420	Selenocyste	no|no chan	0	ENOG410Z
Q8CHI8	EP400_MO	Ep400	Ep400	75560	E1A-bindin	no|no chan	0	COG0553(M
P18911	RARG_MC	Rarg	Rarg	19411	Retinoic ac	no|up	0	ENOG410X
Q921N8	GPS2_MOU	Gps2	Gps2	56310	G protein p	no|no chan	0	ENOG411C
Q99KP6	PRP19_MC	Prpf19	Prpf19	28000	Pre-mRNA	no|no chan	0	ENOG410X
P56581	TDG_MOU	Tdg	Tdg	624784; 216	G/T mismc	no|no chan	0	COG3663(U
Q5NCJ9	Q5NCJ9_M	Uqcr10	Uqcr10	66152	Complex II	no|no chan	0	ENOG410X
P51910	APOD_MC	Apod	Apod	11815	Apolipopro	no|no chan	0	COG3040(C
A2ASZ8	SCMC2_MC	Slc25a25	Slc25a25	227731	Mitochondı	no|no chan	0	ENOG410X
Q6NVE9	PPTC7_MC	Pptc7	Pptc7	320717	Protein phc	no|up	0	COG0631(F
Q9CZT6	CMS1_MO	Cmss1	Cmss1	66497	Protein CM	no|no chan	0	ENOG410X
Q9CZT5	VASN_MO	Vasn	Vasn	246154	Vasorin	no|no chan	0	COG4886(C
Q9CPY1	RM51_MO	Mrpl51	Mrpl51	66493	Large ribos	no|down	0	ENOG4111
P57722	PCBP3_MC	Pcbp3	Pcbp3	59093	Poly(rC)-bi	no|no chan	0	ENOG410X
A2AL76	A2AL76_M	Asph	Asph	65973	Aspartate-b	no|up	0	COG3555(b
A2AC07	A2AC07_M	Cenpx	Cenpx	20892	Centromere	no|no chan	0	ENOG410X
Q3TQQ9	FIRRM_MC	Firrm	Firrm	381306	FIGNL1-in	no|no chan	0	ENOG411C
P57725	SAMN1_M	Samsn1	Samsn1	67742	SAM domai	no|up	0	ENOG410Z
Q7TQ48	SRCA_MO	Srl	Srl	106393	Sarcalumen	no|up	0	ENOG410X
Q5EBG6	HSPB6_MC	Hspb6	Hspb6	243912	Heat shock	no|up	0	ENOG410Y
Q5EBG8	CA050_MOUSE	Uncharacterized p		230696	Uncharacte	no|no chan	0	ENOG4111
A2AQT7	A2AQT7_M	Mmadhc	Mmadhc		Methylmalc	no|no chan	0	ENOG410X
P35700	PRDX1_MC	Prdx1	Prdx1	18477	Peroxiredo	no|no chan	0	COG0450(i
Q3UJP5	CF418_MO	Cfap418	Cfap418	67157	Cilia- and f	no|no chan	0	ENOG4112
P11881	ITPR1_MO	Itpr1	Itpr1	16438	Inositol 1,4,	no|no chan	0	ENOG410X
A0A0N4SV	A0A0N4SV	Lsm5	Lsm5		LSM5 homc	no|no chan	0	COG1958(s
Q811J3	IREB2_MC	Ireb2	Ireb2	64602	Iron-respon	no|no chan	0	COG1048(F
Q60675	LAMA2_M	Lama2	Lama2	16773	Laminin su	no|no chan	0	COG0419(I
P28033	CEBPB_MC	Cebpb	Cebpb	12608	CCAAT/en	no|no chan	0	ENOG410Y
B7ZMT1	B7ZMT1_M	Cdk19	Cdk19	78334	Cdc2l6 prot	no|no chan	0	ENOG410X
Q3U472	Q3U472_M	Cd274	Cd274	60533	PD-L1	no|up	0	ENOG4111
D3Z3K7	D3Z3K7_M	Egr2	Egr2		E3 SUMO-p	no|no chan	0	COG5048(F
A2AJ15	MA1B1_MC	Man1b1	Man1b1	227619	Endoplasm	no|no chan	0	ENOG410X
B7ZMT9	B7ZMT9_M	Chst11	Chst11	58250	Carbohydrc	no|up	0	ENOG4111
A0A571BG	A0A571BG	Dthd1	Dthd1		Death dom	no|no chan	0	ENOG410Z
Q61107	GBP3_MO	Gbp3	Gbp3	55932	Guanylate-	no|up	0	ENOG410X
P24472	GSTA4_MC	Gsta4	Gsta4	14860	Glutathion	no|no chan	0	ENOG4111
Q6A028	SWP70_MC	Swap70	Swap70	20947	Switch-asso	no|no chan	0	ENOG410X
Q8K4K2	TRIB3_MC	Trib3	Trib3	228775	Tribbles ho	no|no chan	0	COG0515(p
A0A8I4P9C	A0A8I4P9C	Rnf213	Rnf213		Ring finger	no|up	0	ENOG410X

A2AHK0	A2AHK0_M	Dgkz	Dgkz	104418	Diacylglyce	no | no chan	0 ENOG410X
B0QZN5	B0QZN5_M	Vamp2	Vamp2		Vesicle-asso	no | no chan	0 COG5143(C
P17879	HS71B_MC	Hspa1b	Hspa1b	15511	Heat shock	no | up	0 COG0443(r
B7ZCJ0	B7ZCJ0_M	Arhgap21	Arhgap21	71435	Rho GTPas	no | no chan	0 ENOG410Y
A0A4W7N	A0A4W7N	N6amt1	N6amt1	67768	N-6 adenin	no | no chan	0 COG2890(M
Q7TSG3	FBX5_MO	Fbxo5	Fbxo5	67141	F-box only	no | no chan	0 ENOG4111
E9QJW0	E9QJW0_M	Mgst1	Mgst1	56615	Microsoma	no | down	0 ENOG4111
Q99J72	ABEC3_M	Apobec3	Apobec3	80287	DNA dC->	no | no chan	0 ENOG4111
Q8K449	ABCA9_M	Abca9	Abca9	217262	ATP-bindir	no | down	0 COG1131(C
Q3TBE9	Q3TBE9_M	Gsdme	Gsdme	54722	Deafness, a	no | no chan	0 ENOG410X
Q8K199	COXM2_M	Cmc2	Cmc2	66531	COX assem	no | no chan	0 ENOG4112
Q8BQ47	CNPY4_M	Cnpy4	Cnpy4	66455	Protein can	no | no chan	0 ENOG4111
E9Q2S7	E9Q2S7_M	Zfp266	Zfp266	77519	Zinc finger	no | down	0 COG5048(S
Q8K190	SAYS1_MC	Saysd1	Saysd1	67509	SAYSvFN d	no | up	0 ENOG4112
Q8K194	SNR27_MC	Snrnp27	Snrnp27	66618	U4/U6.U5 s	no | no chan	0 ENOG410X
A2AWF9	A2AWF9_M	Ptprj	Ptprj	19271	protein-tyro	no | no chan	0 COG5599(c
P14115	RL27A_MC	Rpl27a	Rpl27a	26451	Large ribos	no | no chan	0 COG0200(F
Q8CBI0	Q8CBI0_MOUSE	Uncharacterized		214240	Uncharacte	no | no chan	0 ENOG410X
A0A1L1SQ	A0A1L1SQ	Rps25	Rps25		40S ribosom	no | no chan	0 COG4901(F
A0A1D5RL	A0A1D5RL	Mau2	Mau2	74549	MAU2 chro	no | no chan	0 ENOG410X
P61971	NTF2_MO	Nutf2	Nutf2	68051	Nuclear tra	no | no chan	0 ENOG4111
A0A1D5RL	A0A1D5RL	Rbm34	Rbm34		RNA bindi	no | no chan	0 COG0724(F
P11157	RIR2_MOU	Rrm2	Rrm2	20135	Ribonucleo	no | no chan	0 COG0208(F
A0A286YD	A0A286YD	Cetn3	Cetn3		Centrin 3	no | up	0 COG5126(c
P61979	HNRPK_M	Hnrnpk	Hnrnpk	15387	Heterogene	no | no chan	0 ENOG4111
Q3UD58	Q3UD58_M	Txlna	Txlna	109658	Alpha-taxil	no | no chan	0 ENOG410X
Q3V1B9	Q3V1B9_M	Polr1e	Polr1e	64424	DNA-direc	no | no chan	0 ENOG410X
Q9ERI6	RDH14_M	Rdh14	Rdh14	105014	Retinol deh	no | no chan	0 COG1028(F
Q3UX43	AN13C_M	Ankrd13c	Ankrd13c	433667	Ankyrin rep	no | no chan	0 ENOG410X
Q3THJ3	EIF1A_MC	Eif1ad	Eif1ad	69860	Probable R	no | no chan	0 ENOG4111
Q3U0C0	Q3U0C0_M	Cyth4	Cyth4	72318	Cytohesin 4	no | no chan	0 COG5307(c
Q8BUZ1	ABRA_MO	Abra	Abra	223513	Actin-bindi	no | up	0 ENOG410X
E0CX39	E0CX39_M	Ggact	Ggact		Gamma-glu	no | no chan	0 ENOG4111
P54751	SIA4A_MO	St3gal1	St3gal1	20442	CMP-N-ace	no | up	0 ENOG410X
B2RQG2	B2RQG2_M	Phf3	Phf3	213109	PHD finger	no | no chan	0 ENOG410Z
A0AAA9W	A0AAA9W	Tsr3	Tsr3		18S rRNA c	no | no chan	0 COG2042(p
A0A1S6GW	A0A1S6GW	Ddx39b	Ddx39b	53817	Spliceosom	no | no chan	0 COG0513(c
A0A1S6GW	A0A1S6GW	Birc6	Birc6	12211	UBC core d	no | no chan	0 ENOG410X
A0A1S6GW	A0A1S6GW	Gabarap	Gabarap	56486	Gamma-am	no | no chan	0 ENOG4111
A0A1S6GW	A0A1S6GW	Maff	Maff	17133	BZIP doma	no | no chan	0 ENOG411C
A0A0G2JD	GBP6_MO	Gbp6	Gbp6	100702	Guanylate-	no | up	0 ENOG410X
A0A1S6GW	A0A1S6GW	Uba1	Uba1	22201	Ubiquitin-l	no | no chan	0 COG0476(u
A0A1S6GW	A0A1S6GW	Ncbp2	Ncbp2	68092	Nuclear cap	no | no chan	0 ENOG4111

A0A0R4J07	A0A0R4J07	Uimc1	Uimc1	20184	BRCA1-A c	no | no chan	0	ENOG4111
Q8VID5	RECQ5_M(	Recql5	Recql5	170472	ATP-depen	no | down	0	COG0514((
A0A0R4J07	A0A0R4J07	Suv39h2	Suv39h2	64707	Histone-lys	no | no chan	0	COG2940(u
Q3UVV9	VWA3A_M	Vwa3a	Vwa3a	233813	von Willebr	no | no chan	0	ENOG410Σ
A0A0R4J07	A0A0R4J07	Acbd3	Acbd3	170760	Acyl-Coenz	no | no chan	0	ENOG410Σ
A0A0R4J07	A0A0R4J07	Ubxn4	Ubxn4	67812	UBX doma	no | no chan	0	ENOG410Z
P97470	PP4C_MOI	Ppp4c	Ppp4c	56420	Serine/thre	no | no chan	0	COG0639(r
P97477	AURKA_M	Aurka	Aurka	20878	Aurora kin	no | no chan	0	ENOG410Σ
P97479	MYO7A_M	Myo7a	Myo7a	17921	Unconventi	no | no chan	0	COG5022((
P97478	COQ7_MO	Coq7	Coq7	12850	5-demethox	no | no chan	0	COG2941((
Q3UDY2	Q3UDY2_M	Sgpp1	Sgpp1	81535	Phosphatid	no | no chan	0	ENOG410Z
A2A8L9	A2A8L9_M	Kdm4a	Kdm4a	230674	[histone H3	no | no chan	0	COG5141(c
P36423	THAS_MO	Tbxas1	Tbxas1	21391	Thromboxa	no | no chan	0	COG2124(I
A0A286YCl	A0A286YCl	Syt7	Syt7		Synaptotag	yes | up	1	ENOG410Σ
B2RWU2	B2RWU2_M	Hivep2	Hivep2	15273	Immunodef	no | no chan	0	COG5048(Z
B2RWU3	B2RWU3_M	Fanca	Fanca	14087	Fanconi an	no | no chan	0	ENOG411(
B2RWU4	B2RWU4_M	Cobl	Cobl	12808	Cordon-ble	no | no chan	0	ENOG410Y
B2RWU5	B2RWU5_M	Abca7	Abca7	27403	Abca7 prot	no | no chan	0	COG1131(p
P16054	KPCE_MO	Prkce	Prkce	18754	Protein kin	no | no chan	0	ENOG410Σ
A0A0G2JFI	A0A0G2JFI	Map4	Map4		Microtubul	no | up	0	ENOG4111
G3X9I1	G3X9I1_M	Fam3c	Fam3c	27999	Protein FA	no | no chan	0	ENOG4111
G5E8L8	G5E8L8_M	Map3k2	Map3k2	26405	Mitogen-ac	no | no chan	0	ENOG410Σ
A0A668KL(	A0A668KL(	Nectin1	Nectin1		Nectin cell	no | no chan	0	ENOG410Y
A0A668KL(	A0A668KL(	Map2	Map2		Microtubul	no | no chan	0	ENOG4111
Q8K224	NAT10_M(	Nat10	Nat10	98956	RNA cytidi	no | no chan	0	COG1444((
A2AJY2	A2AJY2_M	Col15a1	Col15a1		Collagen, ty	no | down	0	ENOG410Σ
Q9DBH5	LMAN2_M	Lman2	Lman2	66890	Vesicular ir	no | no chan	0	ENOG410Y
Q8JZR6	S4A8_MOU	Slc4a8	Slc4a8	59033	Electroneut	no | no chan	0	ENOG410Σ
P61620	S61A1_MO	Sec61a1	Sec61a1	53421	Protein trar	no | no chan	0	COG0201(I
G3UZX2	G3UZX2_M	Ube2g2	Ube2g2		E2 ubiquiti	no | no chan	0	ENOG410Y
Q1W5W7	Q1W5W7_I	gag-pol	gag-pol	639390	Putative ga	no | no chan	0	COG2801(I
Q8C436	MT21D_M(	Vcpkmt	Vcpkmt	207965	Protein N-l	no | no chan	0	ENOG4111
A0A1L1SUI	A0A1L1SUI	Sergef	Sergef		Secretion re	no | no chan	0	COG5184(f
B2RX15	B2RX15_M	Ankib1	Ankib1	70797	RBR-type E	no | no chan	0	ENOG410Σ
A6H8H2	DEN4C_M	Dennd4c	Dennd4c	329877	DENN dom	no | no chan	0	ENOG410Σ
P0DW87	ZTRF1_M(	Zftraf1	Zftraf1	125488414;	Zinc finger	no | no chan	0	ENOG410Σ
B7STB7	B7STB7_M	Anxa1	Anxa1	16952	Annexin	no | no chan	0	ENOG410Σ
A0A0A0M(	A0A0A0M(	Camsap1	Camsap1	227634	Calmodulir	no | no chan	0	ENOG4111
Q9QWL7	K1C17_MC	Krt17	Krt17	16667	Keratin, typ	no | no chan	0	ENOG410Y
Q6DFV5	HELZ_MO	Helz	Helz	78455	Probable h	no | no chan	0	COG1112(I
A2RSJ4	BLT3B_MC	Bltp3b	Bltp3b	75089	Bridge-like	no | no chan	0	ENOG410Σ
Q6DFV1	CNDG2_M	Ncapg2	Ncapg2	76044	Condensin-	no | no chan	0	ENOG410Σ
A0A3B2WE	A0A3B2WE	Rpl10a	Rpl10a		Ribosomal	no | no chan	0	COG0081(T

Q6PAR5	GAPD1_M	Gapvd1	Gapvd1	66691	GTPase-act	no	no chan	0	ENOG410
Q6PAR7	Q6PAR7_M	Phf20	Phf20	228829	Phf20 prote	no	up	0	ENOG410
P58069	RASA2_MC	Rasa2	Rasa2	114713	Ras GTPas	no	no chan	0	ENOG410
Q9D236	HTRA3_M	Htra3	Htra3	78558	Serine prote	no	no chan	0	COG0265(S
P58064	RT06_MOU	Mrps6	Mrps6	121022	Small ribos	no	no chan	0	COG0360(I
P62192	PRS4_MOU	Psmc1	Psmc1	19179	26S proteas	no	no chan	0	COG1222(r
Q5FWH6	ARHGF_M	Arhgef15	Arhgef15	442801	Rho guanin	no	down	0	COG5422(r
Q99M07	COA5_MO	Coa5	Coa5	76178	Cytochrom	no	no chan	0	ENOG4111
P62196	PRS8_MOU	Psmc5	Psmc5	19184	26S proteas	no	no chan	0	COG1222(r
Q5FWH2	UNKL_MC	Unkl	Unkl	74154	Putative E3	no	no chan	0	ENOG410
A0A0A6YV	A0A0A6YV	Surf1	Surf1		SURF1-like	no	no chan	0	COG3346(S
Q3TF40	Q3TF40_M	Nono	Nono	53610	RRM doma	no	no chan	0	ENOG410
Q3TC93	H1BP3_MC	Hs1bp3	Hs1bp3	58240	HCLS1-bin	no	no chan	0	ENOG411
Q9R1Y5	HIC1_MOU	Hic1	Hic1	15248	Hypermeth	no	down	0	COG5048(
P47242	PAX9_MO	Pax9	Pax9	18511	Paired box	no	up	0	ENOG4111
Q80YC5	FA12_MOU	F12	F12	58992	Coagulatio	no	up	0	COG5640(T
Q91YJ2	SNX4_MO	Snx4	Snx4	69150	Sorting nex	no	no chan	0	ENOG411C
A2AQK4	A2AQK4_M	Hnmt	Hnmt	140483	Histamine	no	no chan	0	ENOG410Z
Z4YJF5	Z4YJF5_M	Myom1	Myom1		Myomesin 1	no	up	0	ENOG410Z
Q6NSU3	GL8D1_M	Glt8d1	Glt8d1	76485	Glycosyltra	no	no chan	0	ENOG411C
Q3TK92	Q3TK92_M	Arih2	Arih2	23807	RBR-type E	no	no chan	0	ENOG410
A2AIH8	A2AIH8_M	Pir	Pir		Pirin	no	no chan	0	COG1741(p
Q8C3X8	LMF2_MO	Lmf2	Lmf2	105847	Lipase mat	no	no chan	0	ENOG410
Q8K370	ACD10_M	Acad10	Acad10	71985	Acyl-CoA d	no	down	0	COG3173(
P61087	UBE2K_M	Ube2k	Ube2k	53323	Ubiquitin-c	no	no chan	0	COG5078(N
Q3TSR1	Q3TSR1_M	Vps36	Vps36	70160	Vacuolar p	no	no chan	0	ENOG410
P61082	UBC12_MC	Ube2m	Ube2m	22192	NEDD8-co	no	no chan	0	ENOG410
P61080	UB2D1_M	Ube2d1	Ube2d1	216080	Ubiquitin-c	no	no chan	0	COG5078(N
Q3TFA9	Q3TFA9_M	Tmod3	Tmod3	50875	Tropomodu	no	no chan	0	ENOG410
A6H624	A6H624_M	Lysmd1	Lysmd1	217779	LysM and p	no	up	0	ENOG4111
A6H622	A6H622_M	Rasl2-9	Rasl2-9	19428	GTP-bindi	no	no chan	0	ENOG410
Q3UTB8	Q3UTB8_M	Grpel1	Grpel1	17713	GrpE prote	no	no chan	0	COG0576(I
P23798	PCGF2_M	Pcgf2	Pcgf2	22658	Polycomb g	no	no chan	0	ENOG410
Q3UMR5	MCU_MOU	Mcu	Mcu	215999	Calcium un	no	no chan	0	ENOG410
P80316	TCPE_MO	Cct5	Cct5	12465	T-complex	no	no chan	0	COG0459(C
P80314	TCPB_MO	Cct2	Cct2	12461	T-complex	no	no chan	0	COG0459(C
P80315	TCPD_MO	Cct4	Cct4	12464	T-complex	no	no chan	0	COG0459(C
P80313	TCPH_MO	Cct7	Cct7	12468	T-complex	no	no chan	0	COG0459(C
Q9D0R2	SYTC_MOU	Tars1	Tars1	110960	Threonine-	no	no chan	0	COG0441(t
E9PYG5	E9PYG5_M	Parvg	Parvg	64099	Parvin, gan	no	no chan	0	ENOG410
P80318	TCPG_MO	Cct3	Cct3	12462	T-complex	no	no chan	0	COG0459(C
Q3TMX7	QSOX2_M	Qsox2	Qsox2	227638	Sulfhydryl	no	no chan	0	ENOG410
Q6BCN6	Q6BCN6_M	Asb6	Asb6	72323	Ankyrin rep	no	no chan	0	COG0666(V

Q3TXH6	Q3TXH6_M	Smarcd2	Smarcd2	83796	DM2 doma	no | no chan	0	COG5531(U
Q68FM4	Q68FM4_M	Ptprm	Ptprm	19274	protein-tyro	no | up	0	COG5599(S
Q8BTE0	SDHF4_MO	Sdhaf4	Sdhaf4	68002	Succinate d	no | up	0	ENOG4112
Q3U288	ZN710_MO	Znf710	Znf710	209225	Zinc finger	no | no chan	0	COG5048(O
Q8BV49	IFIX_MOU	Pyhin1	Pyhin1	236312	Pyrin and H	no | up	0	ENOG410Y
Q8BTE5	CEBOS_MO	Cebpzos	Cebpzos	68554	Protein CE	no | no chan	0	ENOG410Y
Q99N84	RT18B_MO	Mrps18b	Mrps18b	66973	Small ribos	no | no chan	0	ENOG4111
Q99N87	RT05_MOU	Mrps5	Mrps5	77721	Small ribos	no | no chan	0	COG0098(M
Q3U284	TM231_MO	Tmem231	Tmem231	234740	Transmemb	no | no chan	0	ENOG410X
O35973	PER1_MOU	Per1	Per1	18626	Period circo	no | no chan	0	ENOG410Z
O35972	RM23_MO	Mrpl23	Mrpl23	19935	Large ribos	no | no chan	0	ENOG4111
Q8C850	SCAR3_MO	Scara3	Scara3	219151	Scavenger r	no | down	0	ENOG410Z
E0CYF5	E0CYF5_M	Maf1	Maf1		Repressor o	no | no chan	0	COG5046(F
Q8C854	MYEF2_MO	Myef2	Myef2	17876; 1000	Myelin expr	no | no chan	0	ENOG4110
Q8BIF9	ZN787_MO	Znf787	Znf787	67109	Zinc finger	no | no chan	0	COG5048(F
Q8K0Y2	KT33A_MO	Krt33a	Krt33a	71888	Keratin, typ	no | up	0	ENOG410Y
O55135	IF6_MOUS	Eif6	Eif6	16418	Eukaryotic	no | no chan	0	COG1976(E
D3Z6D2	D3Z6D2_M	Stau2	Stau2	29819	Double-stra	no | no chan	0	ENOG410X
Q9D8B3	CHM4B_M	Chmp4b	Chmp4b	75608	Charged mu	no | no chan	0	ENOG410Y
Q8C0E2	VP26B_MO	Vps26b	Vps26b	69091	Vacuolar p	no | no chan	0	ENOG410X
A0A0U1RP	A0A0U1RP	Mylpf	Mylpf	17907	Fast skeleta	no | up	0	COG5126(T
A0A158SIT	A0A158SIT	Pelp1	Pelp1	75273	Proline-, glu	no | no chan	0	ENOG4110
A0A158SIT	A0A158SIT	Arrb2	Arrb2	216869	Beta-arresti	no | no chan	0	ENOG410X
O35607	BMPR2_MO	Bmpr2	Bmpr2	12168	Bone morph	no | no chan	0	ENOG410X
A0A1W2P7	A0A1W2P7	Gstt2	Gstt2		Glutathione	no | no chan	0	COG0625(E
A0A158SIT	A0A158SIT	Dnase1l1	Dnase1l1	69537	Deoxyribon	no | no chan	0	ENOG410Z
A0A158SIT	A0A158SIT	Fam50a	Fam50a	108160	Ortholog of	no | no chan	0	ENOG410X
A0A158SIT	A0A158SIT	Plxna3	Plxna3	18846	Plexin A3	no | no chan	0	ENOG410X
A0A158SIT	A0A158SIT	Lage3	Lage3	66192	L antigen fa	no | no chan	0	ENOG410Y
A0A158SIT	A0A158SIT	Fam3a	Fam3a	66294	Novel prote	no | no chan	0	
A0A0B4J1N	A0A0B4J1N	Fcgr3	Fcgr3	14131	Fc receptor	no | no chan	0	ENOG410Y
B1ARA3	B1ARA3_M	Rpl26	Rpl26		Large ribos	no | no chan	0	COG0198(E
A2RT57	A2RT57_M	Spg21	Spg21	27965	Maspardin	no | no chan	0	ENOG410X
A0A024QY	A0A024QY	Pten	Pten	19211	Mitochondr	no | no chan	0	COG2453(S
A2AE15	A2AE15_M	Cfp	Cfp	18636	Complemen	no | no chan	0	ENOG410Z
F2Z4B3	F2Z4B3_M	Cdk2ap1	Cdk2ap1		Cyclin depe	no | no chan	0	ENOG4112
Q3TAW4	Q3TAW4_M	Sec23b	Sec23b	27054	Protein tran	no | no chan	0	COG5047(C
Q3TWN8	Q3TWN8_M	Aldh18a1	Aldh18a1	56454	Delta-1-pyr	no | no chan	0	COG0263(C
Q8VCE6	NT5M_MO	Nt5m	Nt5m	103850	5'(3')-deoxyr	no | down	0	ENOG410X
Q3TAW1	Q3TAW1_M	B4galt7	B4galt7	218271	Beta-1,4-ga	no | no chan	0	ENOG410Z
Q3TWN3	CNNM2_M	Cnnm2	Cnnm2	94219	Metal trans	no | no chan	0	COG1253(p
Q8VCE9	PKHH3_M	Plekhh3	Plekhh3	217198	Pleckstrin h	no | down	0	ENOG410Z
A0A0A6YW	A0A0A6YW	Ighv6-5	Ighv6-5		Immunoglo	no | no chan	0	ENOG410Y

Q3TWN7	Q3TWN7_I	Atp6ap1	Atp6ap1	54411	ATPase, H-	no | no chan	0	ENOG410)
G3X914	G3X914_M	Cul5	Cul5	75717	Cullin-5	no | no chan	0	COG5647(s
G3X915	G3X915_M	Lysmd2	Lysmd2	70082	LysM and p	no | no chan	0	ENOG4111
Q9DCW4	ETFB_MO	Etfb	Etfb	110826	Electron trc	no | no chan	0	COG2086(I
B2RXU3	B2RXU3_M	Arl6ip6	Arl6ip6	65103	ADP-ribosy	no | no chan	0	ENOG4111
A0A3B2W8	A0A3B2W8	Vapa	Vapa		Vesicle-assc	no | up	0	COG5066(I
P53986	MOT1_MC	Slc16a1	Slc16a1	20501	Monocarbo	no | no chan	0	ENOG4111
Q3UV16	IPIL2_MOI	Itpripl2	Itpripl2	319622	Inositol 1,4,	no | no chan	0	ENOG4111
Q3UV17	K22O_MO	Krt76	Krt76	77055	Keratin, typ	no | no chan	0	ENOG4111
F6UND7	F6UND7_M	Hck	Hck		Tyrosine-pr	no | up	0	COG0515(I
P29533	VCAM1_M	Vcam1	Vcam1	22329	Vascular ce	no | up	0	ENOG4111
A2BFG8	A2BFG8_M	Zfp971	Zfp971	626848	Zinc finger	no | down	0	COG5048(S
D3YU12	D3YU12_M	Nmral1	Nmral1	67824	NmrA-like	no | no chan	0	COG0702(c
D3YU17	D3YU17_M	Ncln	Ncln	103425	Nicalin	no | no chan	0	ENOG410)
P28776	I23O1_MO	Ido1	Ido1	15930	Indoleamin	no | up	0	ENOG410)
Q8BGX2	TIM29_MC	Timm29	Timm29	69773	Mitochondı	no | no chan	0	ENOG4111
A0A0G2JF	A0A0G2JF	Clca3a2	Clca3a2		Chloride ch	yes | up	1	ENOG410)
B2RWH3	B2RWH3_M	Hist2h2aa1	Hist2h2aa1	319192; 152	Histone H2	no | no chan	0	COG5262(z
A2ALP5	A2ALP5_M	Fgd1	Fgd1	14163	FYVE, Rho	no | no chan	0	ENOG410)
A0A286YC	A0A286YC	Prl3d1	Prl3d1	18775	Growth hor	no | up	0	
Q8BPI1	KTU_MOU	Dnaaf2	Dnaaf2	109065	Protein kin	no | no chan	0	ENOG410)
A0A0R4J26	A0A0R4J26	Osmr	Osmr	18414	Oncostatin	no | no chan	0	ENOG410Y
Q3U3F7	Q3U3F7_M	Tnfrsf23	Tnfrsf23	79201	Tumor necr	no | up	0	ENOG4111
Q8C1I9	Q8C1I9_MOUSE	Centrosomal pro		74201	Centrosomc	no | no chan	0	COG4886(C
A0A0R4J26	A0A0R4J26	Scnm1	Scnm1	69269	Sodium cha	no | no chan	0	ENOG4112
A0A0R4J26	A0A0R4J26	Agpat1	Agpat1	55979	1-acyl-sn-gl	no | no chan	0	COG0204(C
A0A0R4J26	A0A0R4J26	Otud4	Otud4	73945	ubiquitinyl	no | no chan	0	ENOG410Z
J3QMX8	J3QMX8_M	Slc30a6	Slc30a6	210148	Solute carri	no | no chan	0	COG1230(c
J3QMX0	J3QMX0_M	Rpp14	Rpp14	109729085	Ribonuclea	no | no chan	0	COG2030(r
Q570Z8	Q570Z8_M	Picalm	Picalm	233489	MKIAA411	no | no chan	0	ENOG410)
A0A0R4J0A	A0A0R4J0A	Lrp6	Lrp6	16974	Low-density	no | no chan	0	ENOG410)
Q8R5L1	Q8R5L1_M	C1qbp	C1qbp	12261	Complemer	no | no chan	0	ENOG4111
A2AUG1	A2AUG1_M	Fbxw2	Fbxw2		F-box and \	no | up	0	ENOG410)
Q3U4Z7	Q3U4Z7_M	Hdlbp	Hdlbp	110611	K Homologı	no | no chan	0	ENOG410)
E9QP19	E9QP19_M	Trim33	Trim33	94093	Tripartite n	no | no chan	0	ENOG410Z
P53368	8ODP_MO	Nudt1	Nudt1	17766	Oxidized pı	no | no chan	0	COG0494(I
E9Q6J5	BD1L1_MC	Bod1l	Bod1l	665775	Biorientatic	no | no chan	0	ENOG4111
Q8R010	AIMP2_MC	Aimp2	Aimp2	231872	Aminoacyl	no | no chan	0	ENOG411C
Q61823	PDCD4_M	Pdcd4	Pdcd4	18569	Programme	no | no chan	0	ENOG410)
E9Q6J8	E9Q6J8_M	Dmwd	Dmwd		Dystrophia	no | no chan	0	COG2319(S
Q8K057	IFT80_MO	Ift80	Ift80	68259	Intraflagell	no | no chan	0	COG2319(r
B1B1E3	B1B1E3_M	Itgbl1	Itgbl1		Integrin, be	no | no chan	0	ENOG410)
Q9CQE2	TM223_MC	Tmem223	Tmem223	66836	Transmemt	no | down	0	ENOG4111

Q9CQE1	NPS3B_MC	Nipsnap3b	Nipsnap3b	66536	Protein Nip	no | no chan	0	ENOG410X
Q3KQQ1	Q3KQQ1_	Nsfl1c	Nsfl1c	386649	NSFL1 cofc	no | no chan	0	ENOG410Y
Q31206	Q31206_M	H2-T10	H2-T10	15024	Ig-like dom	no | no chan	0	ENOG4111
Q9D7N6	RM30_MO	Mrpl30	Mrpl30	107734	Large ribos	no | no chan	0	ENOG4111
A0A0J9YV2	A0A0J9YV2	Sdad1	Sdad1		Protein SD	no | no chan	0	ENOG410X
F8WI30	F8WI30_M	Snx7	Snx7	76561	Sorting nex	no | no chan	0	COG5391(
A0A0R4J1M	A0A0R4J1M	Art3	Art3	109979	NAD(P)(+)	no | down	0	ENOG4111
Q6P069	SORCN_M	Sri	Sri	109552	Sorcin	no | no chan	0	ENOG410Y
Q3UE03	Q3UE03_M	Cep104	Cep104	230967	TOG doma	no | no chan	0	ENOG410X
P97807	FUMH_MC	Fh	Fh	14194	Fumarate h	no | no chan	0	COG0114(f
A0A2I3BQJ	A0A2I3BQJ	Reep4	Reep4	72549	Receptor ex	no | no chan	0	COG5052(v
A0A2I3BQJ	A0A2I3BQJ	Pced1b	Pced1b		PC-esterase	no | no chan	0	ENOG410Z
B7ZNV4	B7ZNV4_M	Tnnt1	Tnnt1	21955	Troponin T	no | up	0	ENOG410X
A0A494B9I	A0A494B9I	Phf1	Phf1		PHD finger	yes | down	1	ENOG410X
Q8C7V8	CC134_MC	Ccdc134	Ccdc134	76457	Coiled-coil	no | no chan	0	ENOG4111
Q3URK7	Q3URK7_M	Vps39	Vps39	269338	CNH doma	no | no chan	0	ENOG410X
Q8C7V3	UTP15_MC	Utp15	Utp15	105372	U3 small nu	no | no chan	0	ENOG410X
Q61702	ITIH1_MO	Itih1	Itih1	16424	Inter-alpha	no | no chan	0	COG2304(F
F8WGN6	F8WGN6_	Kif21a	Kif21a	16564	Kinesin fan	no | up	0	COG5059(S
Q9JK81	MYG1_MC	Myg1	Myg1	60315	MYG1 exon	no | no chan	0	COG4286(U
P63101	1433Z_MO	Ywhaz	Ywhaz	22631	14-3-3 prote	no | no chan	0	COG5040(t
Q4FJZ3	Q4FJZ3_M	Ero1a	Ero1a	50527	ERO1-like p	no | up	0	COG5061(S
P54320	ELN_MOU	Eln	Eln	13717	Elastin	no | down	0	ENOG4111
A6H5Z3	EXC6B_M	Exoc6b	Exoc6b	75914	Exocyst cor	no | no chan	0	ENOG410X
A2A6J4	A2A6J4_M	Lsp1	Lsp1	16985	Lymphocyte	no | up	0	ENOG410X
Q8CIH9	PUR1_MO	Ppat	Ppat	231327	Amidophos	no | no chan	0	COG0034(g
F8VQ93	F8VQ93_M	Phip	Phip	83946	Pleckstrin h	no | no chan	0	ENOG410Y
F8VQ95	F8VQ95_M	Tacc1	Tacc1	320165	Transformi	no | no chan	0	ENOG410Y
Q8CIH5	PLCG2_M	Plcg2	Plcg2	234779	1-phosphat	no | no chan	0	ENOG410X
Q8BVF7	APH1A_M	Aph1a	Aph1a	226548	Gamma-sec	no | no chan	0	ENOG4111
Q7TS74	CKP2L_M	Ckap2l	Ckap2l	70466	Cytoskeleto	no | no chan	0	ENOG4111
Q8BVF2	PDCL3_M	Pdcl3	Pdcl3	68833	Phosducin-	no | up	0	ENOG4111
P61164	ACTZ_MO	Actr1a	Actr1a	54130	Alpha-cent	no | no chan	0	COG5277((
P61161	ARP2_MO	Actr2	Actr2	66713	Actin-relate	no | no chan	0	COG5277(M
P36895	BMR1A_M	Bmpr1a	Bmpr1a	12166	Bone morpl	no | no chan	0	ENOG410X
Q9D0A3	ARPIN_M	Arpin	Arpin	70420	Arpin	no | no chan	0	ENOG4111
A2A409	A2A409_M	Pax6	Pax6		Paired box	no | no chan	0	ENOG410X
Q9WVG9	MS3L1_MC	Msl3	Msl3	17692	MSL compl	no | no chan	0	ENOG410X
Q9WVD4	CLCN5_M	Clcn5	Clcn5	12728	H(+)/Cl(-)	no | no chan	0	COG0038(M
B2RQ71	B2RQ71_M	Dip2c	Dip2c	208440	Dip2c prote	no | no chan	0	COG0318(
Q5DTK1	CHSS3_MC	Chsy3	Chsy3	78923	Chondroitin	no | no chan	0	ENOG410X
E9QKZ2	E9QKZ2_M	Ipo9	Ipo9	226432	Importin 9	no | no chan	0	COG5657(I
P05977	MYL1_MO	Myl1	Myl1	17901	Myosin ligh	no | up	0	COG5126(C

A3KFU5	A3KFU5_M	Pabpc4	Pabpc4	230721	Polyadenyl	no	no chan	0	ENOG410
P50518	VATE1_M	Atp6v1e1	Atp6v1e1	11973	V-type prot	no	no chan	0	COG1390(
Q6PB44	PTN23_M	Ptpn23	Ptpn23	104831	Tyrosine-pr	no	no chan	0	ENOG410
P50516	VATA_MO	Atp6v1a	Atp6v1a	11964	V-type prot	no	no chan	0	COG1155(
Q3TR55	Q3TR55_M	Smyd3	Smyd3	69726	[histone H3	no	no chan	0	COG2940(
E9Q1A8	E9Q1A8_M	Hbp1	Hbp1	73389	HMG box-	no	up	0	ENOG410
A0A087WR	A0A087WR	Cd22	Cd22		CD22 antig	no	no chan	0	ENOG410
P33609	DPOLA_M	Pola1	Pola1	18968	DNA polyn	no	no chan	0	COG0417(
Q920R0	ALS2_MO	Als2	Als2	74018	Alsin	no	no chan	0	COG4642(
Q5XJY5	COPD_MC	Arcn1	Arcn1	213827	Coatomer s	no	no chan	0	ENOG410
E9Q7U2	E9Q7U2_M	Calcoco1	Calcoco1		Calcium bi	no	no chan	0	ENOG410
P62274	RS29_MOU	Rps29	Rps29	20090	Small ribos	no	no chan	0	COG0199(
Q8BGF7	PAN2_MO	Pan2	Pan2	103135	PAN2-PAN	no	no chan	0	COG0847(
A0A1R3UH	A0A1R3UH	Klk1b3	Klk1b3	18050	Kallikrein	no	up	0	COG5640(
E9Q912	GDS1_MO	Rap1gds1	Rap1gds1	229877	Rap1 GTP	no	no chan	0	ENOG410
Q3TCT4	ENTP7_M	Entpd7	Entpd7	93685	Ectonucleo	no	no chan	0	COG5371(
Q9EQQ9	OGA_MO	Oga	Oga	76055	Protein O-	no	no chan	0	ENOG410
Q9DAT5	MTU1_MO	Trmu	Trmu	72026	Mitochond	no	no chan	0	COG0482(
Q9CTE8	Q9CTE8_M	Alg5	Alg5	66248	dolichyl-ph	no	no chan	0	COG0463(
Q3UGR5	HDHD2_M	Hdhd2	Hdhd2	76987	Haloacid d	no	no chan	0	COG0647(
A0A087WR	A0A087WR	Srrm2	Srrm2		Serine/argi	no	no chan	0	ENOG4111
A0A0N4SU	A0A0N4SU	Naaladl2	Naaladl2	635702	N-acetylate	no	down	0	COG2234(
Q80X59	TMC5B_M	Tmco5b	Tmco5b	75275	Transmemb	no	no chan	0	ENOG4111
Q80X50	UBP2L_M	Ubap2l	Ubap2l	74383	Ubiquitin-c	no	no chan	0	ENOG411
P38585	TTL_MOU	Ttl	Ttl	69737	Tubulin--ty	no	no chan	0	ENOG410
Q6GT24	Q6GT24_M	Prdx6	Prdx6	11758	Peroxiredo	no	no chan	0	COG0450(
Q5UCA9	Q5UCA9_M	Rnase4	Rnase4	58809	Ribonuclea	no	no chan	0	ENOG410
Q8BKI2	TNR6B_M	Tnrc6b	Tnrc6b	213988	Trinucleoti	no	no chan	0	ENOG410
Q01853	TERA_MO	Vcp	Vcp	269523	Transitiona	no	no chan	0	COG0464(
D3Z0Y8	D3Z0Y8_M	Otub2	Otub2	68149	ubiquitinyl	no	down	0	ENOG410
A0A0R4J2D	A0A0R4J2D	Usp37	Usp37	319651	Ubiquitin c	no	no chan	0	ENOG410
A0A0R4J2D	A0A0R4J2D	Mia3	Mia3		MIA SH3 d	no	no chan	0	ENOG410
A0A0A6YX	A0A0A6YX	Taf1a	Taf1a		TATA box-	no	no chan	0	ENOG410
Q921L7	HEMK1_M	Hemk1	Hemk1	69536	MTRF1L re	no	no chan	0	COG2890(
Q921L5	COG2_MO	Cog2	Cog2	76332	Conserved	no	no chan	0	ENOG410
Q3UYL1	Q3UYL1_M	Ubl3	Ubl3	24109	Ubiquitin-l	no	no chan	0	ENOG4111
P09450	JUNB_MO	Junb	Junb	16477	Transcripti	no	no chan	0	ENOG410
P09103	PDIA1_MC	P4hb	P4hb	18453	Protein dis	no	no chan	0	COG0526(
Q80Y32	Q80Y32_M	Hmgxb4	Hmgxb4	70823	HMG box c	no	no chan	0	ENOG411
B2RVT1	B2RVT1_M	Cops9	Cops9	66915	COP9 signo	no	down	0	ENOG410
P56212	ARP19_MC	Arpp19	Arpp19	59046	cAMP-regu	no	no chan	0	ENOG411
Q91YI1	ATG13_M	Atg13	Atg13	51897	Autophagy-	no	no chan	0	ENOG410
Q8CHW4	EI2BE_MC	Eif2b5	Eif2b5	224045	Translation	no	no chan	0	COG1208(

A2AL50	A2AL50_M	Agps	Agps	228061	Alkylglycer	no	no chan	0	COG0277(F
O88792	JAM1_MOU	F11r	F11r	16456	Junctional	no	no chan	0	ENOG410Y
A0A3B2WE	A0A3B2WE	Mea1	Mea1		Male-enhar	no	up	0	ENOG4112
A2ADH1	A2ADH1_M	Magt1	Magt1	67075	Magnesium	no	no chan	0	ENOG410X
D3Z2W0	D3Z2W0_M	Tnip1	Tnip1	57783	TNFAIP3 i	no	no chan	0	ENOG4111
A0A494BA	A0A494BA	Fech	Fech		Ferrochelat	no	no chan	0	COG0276(C
A2AI87	A2AI87_M	Phka1	Phka1		Phosphoryl	no	no chan	0	ENOG410X
P58545	BTBD3_M	Btbd3	Btbd3	228662	BTB/POZ	no	no chan	0	ENOG410X
Q91VD9	NDUS1_M	Ndufs1	Ndufs1	227197	NADH-ubi	no	no chan	0	COG1034(s
E9Q649	GCNT4_M	Gcnt4	Gcnt4	218476	Beta-1,3-ga	no	down	0	ENOG410X
D3YZE7	D3YZE7_M	Edrf1	Edrf1	214764	Erythroid d	no	no chan	0	ENOG410X
E9Q640	E9Q640_M	Rbm26	Rbm26		RNA bindi	no	no chan	0	ENOG4111
Q3UI30	Q3UI30_M	Ewsr1	Ewsr1	14030	RNA-bindi	no	no chan	0	ENOG4111
F6UP77	F6UP77_M	Amdhd2	Amdhd2		N-acetylglu	no	no chan	0	COG1820(C
A2AA85	A2AA85_M	Szrd1	Szrd1		SUZ RNA-l	no	no chan	0	ENOG4111
F6XCT0	F6XCT0_M	Macf1	Macf1		Microtubul	no	no chan	0	COG5069(M
Q99KV1	DJB11_MO	Dnajb11	Dnajb11	67838	DnaJ homo	no	up	0	COG0484(v
Q01705	NOTC1_M	Notch1	Notch1	18128	Neurogenic	no	no chan	0	COG0666(I
Q3V0N8	Q3V0N8_M	Slc12a6	Slc12a6	107723	Solute carri	no	no chan	0	COG0531(c
Q60692	PSB6_MOU	Psmb6	Psmb6	19175	Proteasome	no	no chan	0	ENOG410X
A0A0U1RQ	A0A0U1RQ	Ube2s	Ube2s		E2 ubiquiti	no	no chan	0	COG5078(V
Q8VE80	THOC3_M	Thoc3	Thoc3	73666	THO comp	no	no chan	0	ENOG410X
Q9D7E3	OVCA2_M	Ovca2	Ovca2	246257	Esterase OV	no	no chan	0	ENOG4111
Q9D1M0	SEC13_MO	Sec13	Sec13	110379	Protein SEC	no	no chan	0	ENOG410X
Q6A009	LTN1_MO	Ltn1	Ltn1	78913	E3 ubiquiti	no	no chan	0	COG5219(l
Q61129	CFAI_MOU	Cfi	Cfi	12630	Complemer	no	no chan	0	COG5640(M
Q61127	NAB2_MO	Nab2	Nab2	17937	NGFI-A-bi	no	no chan	0	ENOG410Y
Q05020	APOC2_M	Apoc2	Apoc2	105886299;	Apolipopro	no	no chan	0	ENOG4111
Q61122	NAB1_MO	Nab1	Nab1	17936	NGFI-A-bi	no	no chan	0	ENOG410Y
Q32MD7	Q32MD7_M	Rgs10	Rgs10	67865	Regulator c	no	no chan	0	ENOG410Y
Q9R0X0	MED20_M	Med20	Med20	112694759;	Mediator of	no	no chan	0	ENOG410X
Q9R0X4	ACOT9_M	Acot9	Acot9	56360	Acyl-coenzy	no	no chan	0	ENOG410X
E9QL80	E9QL80_M	Lpgat1	Lpgat1	226856	Lysophosph	no	down	0	COG0204(A
D3Z4E3	D3Z4E3_M	Dhx32	Dhx32	101437	DEAH-box	no	no chan	0	COG1643(k
K4DI61	K4DI61_M	Phf10	Phf10	72057	PHD finger	no	no chan	0	ENOG410Y
Q9EPU4	CPSF1_MC	Cpsf1	Cpsf1	94230	Cleavage ar	no	no chan	0	COG5161(c
A0A571BE	A0A571BE	Zfp827	Zfp827		Zinc finger	no	no chan	0	COG5048(c
Q9EPU0	RENT1_M	Upf1	Upf1	19704	Regulator c	no	no chan	0	COG1112(F
Q7TSI1	PKHM1_M	Plekhm1	Plekhm1	353047	Pleckstrin h	no	no chan	0	ENOG4111
Q80WJ7	LYRIC_MC	Mtdh	Mtdh	67154	Protein LYI	no	no chan	0	ENOG410Y
A0A2L2P5	A0A2L2P5	Dlg2	Dlg2	23859	Discs large	no	up	0	COG0194(M
Q8K3X4	I2BPL_MO	Irf2bpl	Irf2bpl	238330	Probable E	no	no chan	0	ENOG410Z
A2AG22	A2AG22_M	Esx1	Esx1	13984	Extraembry	no	no chan	0	ENOG410Y

Q3V1D3	AMPD1_M	Ampd1	Ampd1	229665	AMP deam	no | up	0	COG1816(S
A0A2I3BS4	A0A2I3BS4	Timm23	Timm23		Translocase	no | no chan	0	COG5596(s
Q9QZV9	NXT1_MO	Nxt1	Nxt1	56488	NTF2-relat	no | no chan	0	ENOG4111
Q3TX25	Q3TX25_M	Aldh3b1	Aldh3b1	67689	Aldehyde d	no | no chan	0	COG1012(V
A0A2I3BS4	A0A2I3BS4	Sorbs3	Sorbs3		Sorbin and	no | no chan	0	ENOG410X
G3UYS3	G3UYS3_M	Mettl14	Mettl14		N6-adenosi	no | no chan	0	ENOG410X
Q9ERK4	XPO2_MO	Cse1l	Cse1l	110750	Exportin-2	no | no chan	0	COG5657(I
P42125	ECI1_MOU	Eci1	Eci1	13177	Enoyl-CoA	no | no chan	0	COG1024(I
A0A0A6YW	A0A0A6YW	Cnih4	Cnih4	98417	Protein cor	no | no chan	0	ENOG4111
A0A1R3UC	A0A1R3UC	Klk1b1	Klk1b1	16623	Kallikrein	no | up	0	COG5640(p
A2CG63	ARI4B_MC	Arid4b	Arid4b	94246	AT-rich int	no | no chan	0	ENOG410Y
B7ZWF1	B7ZWF1_M	Ddx3x	Ddx3x	13205	RNA helico	no | no chan	0	ENOG410X
D3Z7M4	D3Z7M4_M	Igf1	Igf1		Insulin-like	no | no chan	0	ENOG4111
A0A1S6GW	A0A1S6GW	Ddx41	Ddx41	72935	RNA helico	no | no chan	0	ENOG410X
P12804	FGL2_MO	Fgl2	Fgl2	14190	Fibroleukin	no | no chan	0	ENOG410Z
A0A1S6GW	A0A1S6GW	Cnot2	Cnot2	72068	NOT2/NO	no | no chan	0	COG5601(C
A0A1S6GW	A0A1S6GW	Pi4ka	Pi4ka	224020	1-phosphat	no | no chan	0	COG5032(C
P26323	FLI1_MOU	Fli1	Fli1	14247	Friend leuk	no | no chan	0	ENOG410Z
Q04736	YES_MOU	Yes1	Yes1	22612	Tyrosine-pr	no | no chan	0	COG0515(I
Q04735	CDK16_MC	Cdk16	Cdk16	18555	Cyclin-depe	no | up	0	ENOG410X
A0A1S6GW	A0A1S6GW	Hnrnpm	Hnrnpm	76936	RRM doma	no | no chan	0	COG0724(p
A0A1S6GW	A0A1S6GW	Tsfm	Tsfm	66399	Elongation	no | no chan	0	COG0264(A
P53798	FDFT_MO	Fdft1	Fdft1	14137	Squalene sy	no | no chan	0	COG1562(A
A0A0R4J01	A0A0R4J01	Abcc3	Abcc3	76408	ABC-type g	no | no chan	0	COG1132(A
A0A0R4J01	A0A0R4J01	Dus4l	Dus4l	71916	tRNA-dihy	no | no chan	0	COG0042(r
A0A0R4J01	A0A0R4J01	Tcf3	Tcf3	21423	Transcripti	no | no chan	0	ENOG410X
Q790I0	Q790I0_MC	Vars1	Vars1	22321	Valine--tRN	no | no chan	0	COG0525(S
Q68FE6	RIPR1_MC	Ripor1	Ripor1	75687	Rho family-	no | no chan	0	ENOG410Z
A0A0R4J01	A0A0R4J01	Tpmt	Tpmt	22017	Thiopurine	no | no chan	0	ENOG4111
Q8C5W3	TBCEL_MC	Tbcel	Tbcel	272589	Tubulin-sp	no | no chan	0	ENOG411C
Q02085	SNAI1_MC	Snai1	Snai1	20613	Zinc finger	no | no chan	0	ENOG411C
B9EKR4	B9EKR4_M	Prex1	Prex1	277360	Phosphatid	no | no chan	0	ENOG410Z
P97499	TEP1_MOU	Tep1	Tep1	21745	Telomerase	no | no chan	0	ENOG410X
A8IP90	A8IP90_MC	Acsl6	Acsl6	216739	Long-chain	no | up	0	COG1022(A
P97494	GSH1_MO	Gclc	Gclc	14629	Glutamate-	no | no chan	0	ENOG410X
P97496	SMRC1_MC	Smarcc1	Smarcc1	20588	SWI/SNF c	no | no chan	0	COG5259(g
Q80XP8	FA76B_MC	Fam76b	Fam76b	72826	Protein FA	no | no chan	0	ENOG410Y
P97492	RGS14_MC	Rgs14	Rgs14	51791	Regulator c	no | no chan	0	ENOG410Y
G5E8N7	G5E8N7_M	Pnkp	Pnkp	59047	Polynucleot	no | no chan	0	COG0241(I
A0A0G2JFJ	A0A0G2JFJ	Ints3	Ints3		Integrator c	no | no chan	0	ENOG410X
Q3TTN3	Q3TTN3_M	Vdac3	Vdac3	22335	Voltage-de	no | no chan	0	ENOG410Z
F8VPZ9	BICRA_MC	Bicra	Bicra	243842	BRD4-inter	no | no chan	0	ENOG4112
B2RWS6	EP300_MO	Ep300	Ep300	328572	Histone ace	no | no chan	0	COG5076(C

A0A0R4IZ\	A0A0R4IZ\	Sdf2	Sdf2	20316	Stromal cel	no | no chan	0	ENOG410Z
Q3TRV0	Q3TRV0_M	Fiz1	Fiz1	23877	Fiz1 protein	no | no chan	0	COG5048(F
A0A571BD	A0A571BD	Lrp8	Lrp8		Low density	no | no chan	0	ENOG410Y
G3X9K3	BIG1_MOU	Arfgef1	Arfgef1	211673	Brefeldin A	no | no chan	0	COG5307(k
Q8K1F2	Q8K1F2_MOUSE	Anti-VIPase ligh		235952	Anti-VIPase	no | no chan	0	ENOG4111
Q5ND36	Q5ND36_M	Serpinf2	Serpinf2	18816	Serine or cy	no | no chan	0	COG4826(J
A0A0A7NC	A0A0A7NC	Cacnb2	Cacnb2	12296	Voltage-dep	no | up	0	ENOG410X
Q9DBJ3	BI2L1_MO	Baiap2l1	Baiap2l1	66898	BAR/IMD	no | no chan	0	ENOG410Y
P16382	IL4RA_MC	Il4r	Il4r	16190	Interleukin-	no | no chan	0	ENOG4112
Q9CZ52	ANTR1_M	Antxr1	Antxr1	69538	Anthrax tox	no | no chan	0	ENOG410Y
B9EJ29	B9EJ29_MC	Plxna4	Plxna4	243743	Plexin A4	no | no chan	0	ENOG410X
A0A0A0MC	A0A0A0MC	Tm2d1	Tm2d1		TM2 domai	no | no chan	0	ENOG4111
B2RVI6	B2RVI6_M	Cenpp	Cenpp	66336	Centromere	no | no chan	0	ENOG410Y
A0A0A0MC	A0A0A0MC	Mapt	Mapt		Microtubul	no | no chan	0	ENOG4111
A0A0A0MC	A0A0A0MC	Plekhg5	Plekhg5	269608	Pleckstrin h	no | no chan	0	ENOG410X
P58044	IDI1_MOU	Idi1	Idi1	319554	Isopentenyl	no | no chan	0	COG1443(C
P58043	SESN2_MC	Sesn2	Sesn2	230784	Sestrin-2	no | no chan	0	ENOG410X
Q3U4A3	Q3U4A3_M	Cd2bp2	Cd2bp2	70233	GYF domai	no | no chan	0	ENOG4111
Q8BGX0	TRI23_MO	Trim23	Trim23	81003	E3 ubiquiti	no | no chan	0	ENOG410Z
Q7TMC8	FCSK_MO	Fcsk	Fcsk	234730	L-fucose kin	no | no chan	0	ENOG4111
Q3ULL6	Q3ULL6_M	Upf3b	Upf3b	68134	UPF3 regul	no | no chan	0	ENOG4112
A2ACQ1	A2ACQ1_M	Dhx35	Dhx35		RNA helico	no | no chan	0	COG1643(r
Q8BXL7	ARFRP_M	Arfrp1	Arfrp1	76688	ADP-ribosy	no | no chan	0	ENOG410Z
D3Z2J5	D3Z2J5_M	Ncor2	Ncor2		Nuclear rec	no | no chan	0	ENOG410Y
Q80VD1	FA98B_MC	Fam98b	Fam98b	68215	Protein FA	no | no chan	0	ENOG4111
A6H6A9	RBG1L_M	Rabgap1l	Rabgap1l	29809	Rab GTPas	no | no chan	0	COG5210(r
F6WLY0	F6WLY0_M	Irag2	Irag2		Inositol 1,4,	no | down	0	ENOG4112
Q9CXS4	CENPV_M	Cenpv	Cenpv	73139	Centromere	no | no chan	0	COG3791(C
P47911	RL6_MOUS	Rpl6	Rpl6	19988	Large ribos	no | no chan	0	COG2163(J
A8DUK4	A8DUK4_M	Hbb-bs	Hbb-bs	101488143;	Beta-globin	no | no chan	0	COG1018(I
Q80UU9	PGRC2_M	Pgrmc2	Pgrmc2	70804	Membrane-	no | no chan	0	ENOG4111
Q8BZR9	NCBP3_M	Ncbp3	Ncbp3	66874	Nuclear cap	no | no chan	0	ENOG411C
Q99L48	NMD3_MC	Nmd3	Nmd3	97112	60S ribosom	no | no chan	0	COG1499(6
P13542	MYH8_MC	Myh8	Myh8	17885	Myosin-8	no | up	0	COG5022(c
P13541	MYH3_MC	Myh3	Myh3	17883	Myosin-3	yes | up	1	COG5022(c
P49615	CDK5_MO	Cdk5	Cdk5	12568	Cyclin-depe	no | no chan	0	ENOG410X
Q3UTD9	SIM15_MO	Smim15	Smim15	75616	Small integ	no | up	0	ENOG410X
P81069	GABP2_M	Gabpb2	Gabpb2	213054	GA-binding	no | no chan	0	COG0666(r
A0A1W2P6	A0A1W2P6	Myl6	Myl6		Myosin, ligh	no | no chan	0	COG5126(p
Q8BLR5	PSD4_MOU	Psd4	Psd4	215632	PH and SEC	no | up	0	COG5307(p
Q8BLR9	HIF1N_MC	Hif1an	Hif1an	319594	Hypoxia-in	no | no chan	0	ENOG410X
Q7TNC9	I5P1_MOU	Inpp5a	Inpp5a	212111	Inositol pol	no | no chan	0	ENOG410X
Q8BV66	IFI44_MOU	Ifi44	Ifi44	99899	Interferon-i	no | up	0	ENOG410X

P0C7N9	PSMG4_M(	Psmg4	Psmg4	69666	Proteasome	no | no chan	0	ENOG4112
P61202	CSN2_MOI	Cops2	Cops2	12848	COP9 signc	no | no chan	0	COG5159(c
A0A140LJJ1	A0A140LJJ1	Gm44973	Gm44973		Predicted g	no | no chan	0	COG5048(C
A0A140LJJ(	A0A140LJJ(	Wdr45	Wdr45		WD repeat	no | no chan	0	ENOG410X
P61208	ARL4C_M(	Arl4c	Arl4c	320982	ADP-ribosy	no | no chan	0	COG1100((
A0A140LJJ5	A0A140LJJ5	Akap13	Akap13	75547	A kinase an	no | no chan	0	ENOG410X
Q69ZR9	TASOR_M(	Tasor	Tasor	218850	Protein TAS	no | no chan	0	ENOG410X
A0A0U1RQ	A0A0U1RQ	Septin1	Septin1		Septin 1	no | no chan	0	COG5019(V
A0A087WS	A0A087WS	Sh3bgrl2	Sh3bgrl2		SH3 domai	no | no chan	0	ENOG4111
A2AQ56	A2AQ56_M	Tmem62	Tmem62		Transmemb	no | no chan	0	ENOG410X
Q9CPT4	MYDGF_M	Mydgf	Mydgf	28106	Myeloid-de	no | no chan	0	ENOG4112
O35912	EMP3_MO	Emp3	Emp3	13732	Epithelial r	no | up	0	ENOG4111
Q9CPT3	NANP_MC	Nanp	Nanp	67311	N-acylneur	no | no chan	0	COG1011(F
Q5F227	Q5F227_M	Slc36a1	Slc36a1	215335	Slc36a1 pro	no | no chan	0	COG0814(c
A0A2I3BP(	A0A2I3BP(	Hlcs	Hlcs		Holocarbox	no | no chan	0	COG0340(F
Q58A65	JIP4_MOUS	Spag9	Spag9	70834	C-Jun-amin	no | no chan	0	ENOG410X
B1ASY9	B1ASY9_M	Tab3	Tab3	66724	Mitogen-ac	no | no chan	0	ENOG410X
P55264	ADK_MOU	Adk	Adk	11534	Adenosine l	no | no chan	0	COG0524(c
P01029	CO4B_MO	C4b	C4b	625018; 122	Complemer	no | no chan	0	ENOG410X
Q3TGG2	Q3TGG2_M	Atxn2l	Atxn2l	233871	Ataxin 2-lik	no | no chan	0	COG5180(F
F8WJ72	F8WJ72_M(	Cdc45	Cdc45	12544	Cell divisio	no | no chan	0	ENOG410X
A0A140LI5	A0A140LI5	Pnpla6	Pnpla6	50767	Patatin-like	no | no chan	0	COG1752(r
D3Z6F0	D3Z6F0_M	Ifit1bl1	Ifit1bl1	667373	Interferon i	no | no chan	0	COG0457(r
Q60860	LTC4S_MC	Ltc4s	Ltc4s	17001	Leukotriene	no | no chan	0	ENOG4111
F8WJ74	F8WJ74_M(	Gramd1a	Gramd1a		GRAM dom	no | no chan	0	ENOG410X
Q6PE01	SNR40_MC	Snrnp40	Snrnp40	66585	U5 small nu	no | no chan	0	ENOG410X
A0A0A6YY	A0A0A6YY	Impdh2	Impdh2		Inosine mo	no | down	0	
Q69ZR3	Q69ZR3_M	Galnt16	Galnt16	108760	Polypeptide	no | no chan	0	ENOG410X
Q8C0K5	GDC_MOU	Slc25a16	Slc25a16	73132	Solute carri	no | no chan	0	ENOG410Z
Q9ERV1	MKRN2_M	Mkrn2	Mkrn2	67027	E3 ubiquiti	no | no chan	0	COG5084(z
E9PYA3	E9PYA3_M	Hagh	Hagh		Hydroxyacy	no | up	0	COG0491(F
Q8CA72	GAN_MOU	Gan	Gan	209239	Gigaxonin	no | no chan	0	ENOG410X
A0A1W2P8	CE85L_MC	Cep85l	Cep85l	100038725	Centrosome	no | down	0	ENOG410Z
O35623	BET1_MOU	Bet1	Bet1	12068	BET1 homc	no | no chan	0	ENOG4111
O35621	PMM1_MC	Pmm1	Pmm1	29858	Phosphomo	no | no chan	0	COG0561(A
G3X934	G3X934_M	Wdr70	Wdr70	545085	WD repeat-	no | no chan	0	ENOG410X
G3X937	G3X937_M	Slc41a3	Slc41a3	71699	Solute carri	no | no chan	0	ENOG410X
D3Z041	D3Z041_M	Acsl1	Acsl1		Long-chain	no | down	0	COG1022(C
Q7TPD2	F185A_MO	Fam185a	Fam185a	330050	Protein FAI	no | no chan	0	ENOG411C
E9Q8I0	E9Q8I0_M(	Cfh	Cfh	12628	Complemer	no | up	0	ENOG410Y
P70275	SEM3E_M(	Sema3e	Sema3e	20349	Semaphorir	no | up	0	ENOG410X
P70270	RAD54_M(	Rad54l	Rad54l	19366	DNA repai	no | down	0	COG0553(e
P70271	PDLI4_MC	Pdlim4	Pdlim4	30794	PDZ and L	no | no chan	0	ENOG410X

Q8VCK5	KLH20_M(	Klhl20	Klhl20	226541	Kelch-like p	no | no chan	0	ENOG410)
Q32P16	Q32P16_M	Wdr13	Wdr13	73447	WD repeat	no | no chan	0	ENOG410Y
P15864	H12_MOUS	H1-2	H1-2	50708	Histone H1	no | no chan	0	ENOG4112
Q32P12	HROB_MC	Hrob	Hrob	217216	Homologou	no | no chan	0	ENOG4111
Q8K2D8	Q8K2D8_M	Fibp	Fibp	58249	Fibp protei	no | no chan	0	ENOG410Z
P70279	SURF6_MC	Surf6	Surf6	20935	Surfeit locu	no | no chan	0	ENOG4111
P60003	ELOF1_M(	Elof1	Elof1	66126	Transcripti	no | up	0	COG4888(e
Q9DCU6	RM04_MO	Mrpl4	Mrpl4	66163	Large ribos	no | no chan	0	COG0088(r
H3BLR8	H3BLR8_M	Nudt3	Nudt3		diphosphoi	no | no chan	0	COG0494(C
P43346	DCK_MOU	Dck	Dck	13178	Deoxycytid	no | no chan	0	COG1428(I
B2RRE2	B2RRE2_M	Myo18a	Myo18a	360013	Myo18a prc	no | no chan	0	COG5022(S
B2RRE8	B2RRE8_M	Cd2ap	Cd2ap	12488	CD2-associ	no | no chan	0	ENOG410)
Q9CVF5	Q9CVF5_M	Nqo2	Nqo2	18105	Flavodoxin	no | no chan	0	COG2249((
Q3UD06	Q3UD06_M	Atp5f1c	Atp5f1c	11949	ATP syntha	no | no chan	0	COG0224(I
P25916	BMI1_MOU	Bmi1	Bmi1	12151	Polycomb c	no | no chan	0	ENOG410)
P25911	LYN_MOU	Lyn	Lyn	17096	Tyrosine-pr	no | no chan	0	COG0515(I
D3Z1X7	D3Z1X7_M	Zscan21	Zscan21		Zinc finger	no | no chan	0	COG5048(t
A0A0R4J24	A0A0R4J24	Chid1	Chid1	68038	Chitinase d	no | no chan	0	ENOG410)
A0A0R4J24	A0A0R4J24	Suds3	Suds3	71954	Suppressor	no | no chan	0	ENOG410Y
A3KGL9	A3KGL9_M	Hmgn2	Hmgn2		Non-histon	no | down	0	ENOG410Z
A0A0R4J0C	A0A0R4J0C	Eri1	Eri1	67276	Exoribonuc	no | no chan	0	COG5018(r
A0A0R4J0C	A0A0R4J0C	Ino80b	Ino80b	70020	INO80 com	no | no chan	0	ENOG411C
P18760	COF1_MO	Cfl1	Cfl1	12631	Cofilin-1	no | no chan	0	ENOG4112
A0A0R4J0C	A0A0R4J0C	Cpxm2	Cpxm2	55987	Carboxypep	no | no chan	0	ENOG410)
Q99JI4	PSMD6_M(	Psmd6	Psmd6	66413	26S proteas	no | no chan	0	COG5187(I
A0A0R4J0C	A0A0R4J0C	Nsmce1	Nsmce1	67711	Non-structu	no | no chan	0	ENOG4111
A2AUY4	BAZ2B_M(	Baz2b	Baz2b	407823	Bromodom	no | no chan	0	COG5076(p
E9Q4Z2	ACACB_M	Acacb	Acacb	100705	Acetyl-CoA	no | no chan	0	COG4799(c
A0A338P69	A0A338P69	Smpd4	Smpd4		Sphingomy	no | no chan	0	ENOG410Y
A0A0J9YV8	A0A0J9YV8	Rab28	Rab28	100972	Ras-related	no | no chan	0	ENOG410)
O88587	COMT_MC	Comt	Comt	12846	Catechol O	no | no chan	0	COG4122(C
Q9D009	LIPT2_MO	Lipt2	Lipt2	67164	Octanoyl-[c	no | no chan	0	COG0321(C
Q6PGL7	WASC2_M(	Washc2	Washc2	28006	WASH com	no | no chan	0	ENOG411C
A0A0A6YX	A0A0A6YX	Uck2	Uck2		uridine/cyt	no | no chan	0	COG0572(u
Q61805	LBP_MOU	Lbp	Lbp	16803	Lipopolysa	no | up	0	ENOG410Z
A0A0A6YX	A0A0A6YX	Mast4	Mast4		non-specifi	no | no chan	0	ENOG410)
P46737	BRCC3_MC	Brcc3	Brcc3	210766	Lys-63-spec	no | no chan	0	COG1310(e
D3Z0V7	D3Z0V7_M	Tsc22d1	Tsc22d1	21807	TSC22 dom	no | no chan	0	ENOG4112
A0A1B0GT	A0A1B0GT	Bax	Bax		BCL2-assoc	no | no chan	0	ENOG4112
Q059U0	Q059U0_M	Agpat2	Agpat2	67512	1-acyl-sn-gl	no | no chan	0	COG0204(c
E9Q455	E9Q455_M	Tpm1	Tpm1	22003	Tropomyos	no | no chan	0	ENOG410)
Q059U9	Q059U9_M	Akap8	Akap8	56399	A kinase (P	no | no chan	0	ENOG410Y
A0A0D5ZY	A0A0D5ZY72_MOUSE	Immunoglobulin heav			IgE H chair	no | up	0	ENOG4111

Q9WU56	PUS1_MOU	Pus1	Pus1	56361	Pseudourid	no | no chan	0	COG0101(M
E9Q6D3	E9Q6D3_M	Ccdc121rt1	Ccdc121rt1	403180	Coiled-coil	no | no chan	0	ENOG4111
Q8BHN1	TXLNG_M	Txlng	Txlng	353170	Gamma-ta	no | no chan	0	ENOG410X
Q8BHN0	PPM1L_MO	Ppm1l	Ppm1l	242083	Protein pho	no | up	0	COG0631(f
Q8BHN5	RBM45_MO	Rbm45	Rbm45	241490	RNA-bindi	no | no chan	0	ENOG410X
Q8BHN7	RLIG1_MO	Rlig1	Rlig1	68281	RNA ligase	no | no chan	0	ENOG4110
Q8C2T9	Q8C2T9_M	Ik	Ik	24010	Protein Red	no | no chan	0	ENOG410Y
Q8BFT2	HAUS4_MO	Haus4	Haus4	219072	HAUS augm	no | no chan	0	ENOG4111
Q9CQK1	ZNHI3_MO	Znhit3	Znhit3	448850	Zinc finger	no | no chan	0	ENOG4112
D5MCW4	D5MCW4_	Cuta	Cuta	67675	CutA divale	no | no chan	0	COG1324(t
A0A0J9YV0	A0A0J9YV0	Zeb2	Zeb2		Zinc finger	no | no chan	0	ENOG410Z
Q9QZQ1	AFAD_MO	Afdn	Afdn	17356	Afadin	no | no chan	0	ENOG410X
F8WIE5	F8WIE5_M	Hectd1	Hectd1	207304	E3 ubiquiti	no | no chan	0	COG5021(S
Q61292	LAMB2_MO	Lamb2	Lamb2	16779	Laminin su	no | no chan	0	ENOG410X
A0A0R4J1K	A0A0R4J1K	Kcnh2	Kcnh2	16511	Potassium v	no | no chan	0	ENOG410X
A0A0R4J1K	A0A0R4J1K	Cnot4	Cnot4	53621	CCR4-NOT	no | no chan	0	COG5175(C
Q8WUR0	CS012_MOUSE	Protein C19orf12 h		72244	Protein C19	no | no chan	0	ENOG410Y
Q8C796	RCOR2_MO	Rcor2	Rcor2	104383	REST corep	no | no chan	0	ENOG410X
Q811U6	Q811U6_MOUSE	Anti-human Fc gamma rece			Anti-huma	no | no chan	0	ENOG4111
P56873	ZNRD2_M	Znrd2	Znrd2	56390	Protein ZN	no | no chan	0	ENOG4111
P14094	AT1B1_MO	Atp1b1	Atp1b1	11931	Sodium/po	no | up	0	ENOG4111
P97825	JUPI1_MO	Jpt1	Jpt1	15374	Jupiter mic	no | up	0	ENOG410Y
P97390	VPS45_MO	Vps45	Vps45	22365	Vacuolar p	no | no chan	0	COG5158(C
Q80TA9	EPG5_MO	Epg5	Epg5	100502841	Ectopic P g	no | no chan	0	ENOG410X
P97821	CATC_MO	Ctsc	Ctsc	13032	Dipeptidyl	no | up	0	COG4870(E
P97822	AN32E_MO	Anp32e	Anp32e	66471	Acidic leuc	no | no chan	0	ENOG4111
P97823	LYPA1_MO	Lypla1	Lypla1	18777	Acyl-protei	no | no chan	0	COG0400(E
Q8CFT2	SET1B_MO	Setd1b	Setd1b	208043	Histone-lys	no | no chan	0	COG2940(V
Q501J7	PHAR4_MO	Phactr4	Phactr4	100169	Phosphatas	no | no chan	0	ENOG4111
Q80TA6	MTMRC_M	Mtmr12	Mtmr12	268783	Myotubula	no | no chan	0	ENOG410X
Q80TA4	Q80TA4_M	Zfp451	Zfp451	98403	MKIAA170	no | down	0	ENOG4111
Q8BJM7	TYW1_MO	Tyw1	Tyw1	100929	S-adenosyl-	no | no chan	0	COG0731(r
O54774	AP3D1_MO	Ap3d1	Ap3d1	11776	AP-3 compl	no | no chan	0	ENOG410X
A0A1Y7VN	A0A1Y7VN	Pole2	Pole2		DNA polym	no | no chan	0	ENOG410X
B2RUP8	B2RUP8_M	Cln5	Cln5	211286	Bis(monoac	no | no chan	0	ENOG4110
P27512	TNR5_MO	Cd40	Cd40	21939	Tumor necr	no | no chan	0	ENOG4112
Q9JLQ2	GIT2_MOU	Git2	Git2	26431	ARF GTPa	no | no chan	0	ENOG410X
Q8VDI4	Q8VDI4_M	Mlh1	Mlh1	17350	MutL homo	no | no chan	0	COG0323(C
Q6ZQF0	TOPB1_MO	Topbp1	Topbp1	235559	DNA topoi	no | no chan	0	ENOG410X
Q6ZQF7	JADE2_MO	Jade2	Jade2	76901	E3 ubiquiti	no | no chan	0	COG5141(T
Q3UKS3	Q3UKS3_M	Sigirr	Sigirr	24058	Single Ig IL	no | no chan	0	ENOG410Z
H3BJ07	H3BJ07_MO	Golph3l	Golph3l	229593	Golgi phos	no | no chan	0	ENOG410X
Q8BIJ7	RUFY1_MO	Rufy1	Rufy1	216724	RUN and F	no | no chan	0	ENOG410X

Q9CWF6	BBS2_MOU	Bbs2	Bbs2	67378	BBSome co	no|no chan	0	ENOG410X
Q8CIJ3	Q8CIJ3_MO	Eif3b	Eif3b	27979	Eukaryotic	no|no chan	0	COG5354(C
Q9CX66	NOPC1_MO	Nopchap1	Nopchap1	28109	NOP protei	no|up	0	ENOG410Y
Q80VE5	Q80VE5_M	Tbc1d22b	Tbc1d22b	381085	TBC1 doma	no|no chan	0	ENOG410X
Q3UMM1	Q3UMM1_	Tubb6	Tubb6	67951	Tubulin be	no|no chan	0	COG5023(f
Q9CX60	LBH_MOU	Lbh	Lbh	77889	Protein LBI	no|up	0	ENOG4111
P98083	SHC1_MOU	Shc1	Shc1	20416	SHC-transf	no|no chan	0	ENOG410X
Q3V3E3	Q3V3E3_M	Trmt13	Trmt13	229780	tRNA:m(4)X	no|no chan	0	ENOG410Z
M0QWY6	M0QWY6_	Cd200r1	Cd200r1		CD200 rece	no|down	0	ENOG4112
E9PVK4	E9PVK4_M	Ttc21b	Ttc21b	73668	Tetratricop	no|no chan	0	ENOG410X
E9Q0Y4	E9Q0Y4_M	Sipa1	Sipa1	20469	Signal-indu	no|no chan	0	ENOG410X
Q8K3C3	LZIC_MOU	Lzic	Lzic	69151	Protein LZI	no|no chan	0	ENOG410Z
Q3UG80	Q3UG80_M	Vps18	Vps18	228545	Vacuolar p	no|no chan	0	ENOG410X
Q14AR3	Q14AR3_M	Trim14	Trim14	74735	Tripartite n	no|no chan	0	ENOG410Y
Q14AR0	Q14AR0_M	Slirp	Slirp	380773	RRM doma	no|no chan	0	COG0724(c
Q3V389	Q3V389_M	Rab36	Rab36	76877	Ras-related	no|no chan	0	ENOG410X
Q8K2Y3	EVA1B_MO	Eva1b	Eva1b	230752	Protein eva	no|down	0	ENOG4111
Q8K2Y7	RM47_MO	Mrpl47	Mrpl47	74600	Large ribos	no|no chan	0	ENOG4112
Q3TAH3	Q3TAH3_M	Npm3	Npm3	18150	Nucleoplas	no|no chan	0	ENOG4112
Q8R0S2	IQEC1_MO	Iqsec1	Iqsec1	232227	IQ motif ar	no|no chan	0	COG5307(t
D3YZD8	D3YZD8_M	Aamdc	Aamdc	66273	Adipogenes	no|no chan	0	ENOG4112
A0A803ZIZ	A0A803ZIZ	Obsl1	Obsl1		Obscurin-li	no|no chan	0	ENOG410X
B5T0I7	B5T0I7_MO	Apobec3	Apobec3	80287	single-stran	no|no chan	0	ENOG4111
E0CZE0	E0CZE0_M	Nae1	Nae1		NEDD8-ac	no|no chan	0	COG0476(F
A2AGH6	MED12_MO	Med12	Med12	59024	Mediator of	no|no chan	0	ENOG410X
Q7M6X7	Q7M6X7_M	Zfp457	Zfp457	431706	Zinc finger	no|no chan	0	COG5048(F
Q3UXU7	Q3UXU7_M	Vangl1	Vangl1	229658	Vang-like p	no|no chan	0	ENOG410X
Q08857	CD36_MOU	Cd36	Cd36	12491	Platelet gly	no|down	0	ENOG410X
Q8C605	Q8C605_M	Pfkp	Pfkp	56421	ATP-depen	no|up	0	COG0205(C
P62259	1433E_MO	Ywhae	Ywhae	22627	14-3-3 prot	no|no chan	0	COG5040(u
P62257	UBE2H_MO	Ube2h	Ube2h	22214	Ubiquitin-c	no|no chan	0	ENOG410X
P62254	UB2G1_MO	Ube2g1	Ube2g1	67128	Ubiquitin-c	no|no chan	0	COG5078(c
O35407	O35407_MO	Ddr1	Ddr1	12305	receptor pro	no|no chan	0	ENOG410X
Q6P5D3	DHX57_MO	Dhx57	Dhx57	106794	Putative AT	no|no chan	0	COG1643(T
Q9ET22	DPP2_MOU	Dpp7	Dpp7	83768	Dipeptidyl	no|no chan	0	ENOG410Y
Q8K0C9	GMDS_MO	Gmds	Gmds	218138	GDP-mann	no|no chan	0	COG1089(C
A0A087WR	A0A087WR	Agap1	Agap1	347722	ArfGAP wi	no|no chan	0	COG5347(T
P10711	TCEA1_MO	Tcea1	Tcea1	21399	Transcripti	no|no chan	0	
Q9ET26	RN114_MO	Rnf114	Rnf114	81018	E3 ubiquiti	no|no chan	0	ENOG4111
Q8K0C4	CP51A_MO	Cyp51a1	Cyp51a1	13121	Lanosterol	no|down	0	COG2124(r
Q6P5D8	SMHD1_M	Smchd1	Smchd1	74355	Structural r	no|no chan	0	ENOG410X
O35215	DOPD_MO	Ddt	Ddt	13202	D-dopachro	no|no chan	0	ENOG4112
E9Q933	E9Q933_M	Tmem11	Tmem11	216821	Transmemb	no|no chan	0	ENOG410Z

Q3TDB6	Q3TDB6_M	Slc31a1	Slc31a1	20529	Copper trar	no|no chan	0	ENOG4111
Q9CQ37	UBE2T_MO	Ube2t	Ube2t	67196	Ubiquitin-c	no|down	0	COG5078(I
B9EKI5	B9EKI5_M	Cblb	Cblb	208650	E3 ubiquiti	no|no chan	0	ENOG410Y
B9EKI3	TMF1_MO	Tmf1	Tmf1	232286	TATA elem	no|no chan	0	ENOG410Y
Q80X72	LRC15_MC	Lrrc15	Lrrc15	74488	Leucine-ric	no|down	0	COG4886(M
A2A791	ZMYM4_M	Zmym4	Zmym4	67785	Zinc finger	no|no chan	0	ENOG410X
Q9CQ39	MED21_M	Med21	Med21	108098	Mediator of	no|no chan	0	ENOG4111
E9PZD8	E9PZD8_M	Cp	Cp		ferroxidase	no|up	0	COG2132(M
P99026	PSB4_MOU	Psmb4	Psmb4	19172	Proteasome	no|no chan	0	ENOG410Y
P99027	RLA2_MOU	Rplp2	Rplp2	67186	Large ribos	no|no chan	0	COG2058(C
P99024	TBB5_MOU	Tubb5	Tubb5	22154	Tubulin be	no|no chan	0	COG5023(S
P99025	GFRP_MO	Gchfr	Gchfr	320415	GTP cycloh	no|no chan	0	ENOG4111
G5E850	G5E850_M	Cyb5a	Cyb5a	109672	Cytochrom	no|no chan	0	COG5274(c
A0A0N4SU	A0A0N4SU	Nfu1	Nfu1	56748	NFU1 iron-	no|no chan	0	COG0694(r
P99028	QCR6_MO	Uqcrh	Uqcrh	66576	Cytochrom	no|no chan	0	ENOG4112
P99029	PRDX5_M	Prdx5	Prdx5	54683	Peroxiredo	no|up	0	COG0678(p
Q6PB66	LPPRC_MO	Lrpprc	Lrpprc	72416	Leucine-ric	no|no chan	0	ENOG410X
Q9D1Q1	MPH6_MO	Mphosph6	Mphosph6	68533	M-phase ph	no|no chan	0	ENOG4111
Q60604	SCIN_MOU	Scin	Scin	20259	Scinderin	no|no chan	0	ENOG410X
Q9D1Q4	DPM3_MO	Dpm3	Dpm3	68563	Dolichol-pl	no|no chan	0	ENOG4112
Q3V300	KIF22_MO	Kif22	Kif22	110033	Kinesin-lik	no|no chan	0	COG5059(C
Q8BFU3	RN214_MC	Rnf214	Rnf214	235315	RING finge	no|no chan	0	ENOG410Z
O54743	FOXF2_MO	Foxf2	Foxf2	14238	Forkhead b	no|no chan	0	COG5025(I
O54962	BAF_MOU	Banf1	Banf1	23825	Barrier-to-c	no|no chan	0	ENOG4111
P09470	ACE_MOU	Ace	Ace	11421	Angiotensir	no|no chan	0	ENOG410X
Q9ES97	RTN3_MO	Rtn3	Rtn3	20168	Reticulon-3	no|no chan	0	ENOG410X
E9PZP8	E9PZP8_M	Herc1	Herc1	235439	HECT and	no|no chan	0	COG5184(c
Q9CZP3	ZN655_MC	Zfp655	Zfp655	72611	Zinc finger	no|no chan	0	COG5048(A
Q80Y19	RHGBA_M	Arhgap11a	Arhgap11a	228482	Rho GTPas	no|no chan	0	ENOG410Y
A2RSQ0	DEN5B_M	Dennd5b	Dennd5b	320560	DENN don	no|no chan	0	ENOG410X
Q9CZP5	BCS1_MOU	Bcs1l	Bcs1l	66821	Mitochond	no|no chan	0	COG0465(C
D3YWK1	D3YWK1_M	Wipi2	Wipi2		WD repeat	no|no chan	0	ENOG410X
A0A4Y5WV	A0A4Y5WV	Myo18a	Myo18a	360013	Myosin XV	no|no chan	0	COG5022(M
A0A1G5SJ/	A0A1G5SJ/	OTTMUSW	OTTMUSW	621823	Proteasome	no|up	0	ENOG410X
Q8BXV2	BRI3B_MO	Bri3bp	Bri3bp	76809	BRI3-bindi	yes|up	1	ENOG410Z
A2AL36	CNTRL_M	Cntrl	Cntrl	26920	Centriolin	no|no chan	0	ENOG410Z
A0A0G2JF4	A0A0G2JF4	Igkv14-130	Igkv14-130	628072	Immunoglo	yes|down	1	ENOG410Y
Q9CYX7	RRP15_MC	Rrp15	Rrp15	67223	RRP15-like	no|no chan	0	ENOG4111
A0A494BA	A0A494BA	Rpusd1	Rpusd1	106707	RNA pseud	no|up	0	COG0564(c
A2AKG8	FOCAD_M	Focad	Focad	230393	Focadhesin	no|no chan	0	ENOG410Y
A0A494BA	A0A494BA	Ergic1	Ergic1		Endoplasm	no|no chan	0	ENOG410X
Q91YP2	NEUL_MO	Nln	Nln	75805	Neurolysin,	no|no chan	0	COG0339(c
Q8BXW8	Q8BXW8_M	Uvrag	Uvrag	78610	C2 domain-	no|no chan	0	ENOG410Y

Q91YP1	BN3D2_M(	Bcdin3d	Bcdin3d	75284	RNA 5'-mo	no | no chan	0	ENOG410
D3Z2Q3	D3Z2Q3_M	Diablo	Diablo		Direct IAP-	no | no chan	0	ENOG4111
Q3U125	Q3U125_M	Prxl2a	Prxl2a	70564	Peroxiredo	no | no chan	0	ENOG410
Q9JKB3	YBOX3_M	Ybx3	Ybx3	56449	Y-box-bind	no | no chan	0	COG1278(
Q9JKB1	UCHL3_M(	Uchl3	Uchl3	50933	Ubiquitin c	no | no chan	0	ENOG4111
Q3UJL7	Q3UJL7_M	Srm	Srm	20810	PABS doma	no | no chan	0	COG0421(
Q61321	SIX4_MOU	Six4	Six4	20474	Homeobox	no | no chan	0	ENOG410
Q3UJL0	Q3UJL0_M	Gnl2	Gnl2	230737	Nucleolar (	no | no chan	0	COG1161(
Q543N3	Q543N3_M	Lasp1	Lasp1	16796	LIM and SI	no | no chan	0	ENOG4111
Q543N5	Q543N5_M	Clic4	Clic4	29876	Chloride in	no | no chan	0	ENOG410
Q80XK6	ATG2B_M(	Atg2b	Atg2b	76559	Autophagy-	no | no chan	0	ENOG410
Q543N7	Q543N7_M	Pacsin3	Pacsin3	80708	Protein kin	no | up	0	ENOG410
F6RDM4	F6RDM4_M	Gls	Gls		glutaminas	no | down	0	COG2066(
Q9JK23	PSMG1_M(	Psmg1	Psmg1	56088	Proteasome	no | no chan	0	ENOG4111
Q8BYM8	SYCM_MO	Cars2	Cars2	71941	Probable cy	no | down	0	COG0215(
Q14DU1	Q14DU1_M	Klhl11	Klhl11	217194	Kelch-like 1	no | no chan	0	ENOG411(
P01664	KV3AC_MOUSE	Ig kappa chain V-III region (			Ig kappa ch	no | down	0	ENOG4111
P01660	KV3A8_MOUSE	Ig kappa chain V-III region I			Ig kappa ch	no | up	0	ENOG4111
P01662	KV3AA_MOUSE	Ig kappa chain V-III region			Ig kappa ch	no | down	0	ENOG4111
Q4G0F8	UBN1_MO	Ubn1	Ubn1	170644	Ubinuclein-	no | down	0	ENOG410
Q61142	SPIN1_MO	Spin1	Spin1	20729	Spindlin-1	no | no chan	0	ENOG410
A2RTI3	A2RTI3_M	Lgmn	Lgmn	19141	Legumain	no | no chan	0	COG5206(
Q8BLM6	Q8BLM6_M	Nup155	Nup155	170762	Nucleopori	no | no chan	0	COG5308(
D2DFA9	D2DFA9_M	Gprc5b	Gprc5b	64297	G protein-c	no | no chan	0	ENOG410
E9QL65	E9QL65_M	Cog3	Cog3	338337	Conserved (	no | no chan	0	ENOG410
A0A0U1RN	A0A0U1RN	Uri1	Uri1		URI1, prefc	no | no chan	0	ENOG411(
Q3TED0	Q3TED0_M	Slco2a1	Slco2a1	24059	Solute carri	no | no chan	0	ENOG410
Q543M9	Q543M9_M	Trp53rkb	Trp53rkb	76367	non-specifi	no | no chan	0	COG3642(
P54823	DDX6_MC	Ddx6	Ddx6	13209	Probable A	no | no chan	0	ENOG410
P54822	PUR8_MO	Adsl	Adsl	11564	Adenylosuc	no | no chan	0	COG0015(
A0A0G2JG	A0A0G2JG	Igkv12-98	Igkv12-98	435900	Immunoglo	no | down	0	ENOG410
A2AG09	A2AG09_M	Rbm41	Rbm41	237073	RNA bindi	no | no chan	0	ENOG4111
Q3TAS3	Q3TAS3_M	Stx11	Stx11	74732	Stx11 prote	no | no chan	0	COG5074(
P52795	EFNB1_M(	Efnb1	Efnb1	13641	Ephrin-B1	no | up	0	ENOG4111
A0A0G2JG	A0A0G2JG	Il6ra	Il6ra	16194	Interleukin	no | up	0	ENOG4111
A0A1Y7VJI	A0A1Y7VJI	Eif1ad13	Eif1ad13		Eukaryotic	no | no chan	0	COG0361(
Q8CI43	MYL6B_M(	Myl6b	Myl6b	216459	Myosin ligh	no | up	0	COG5126(
A0A286YD	A0A286YD	Adgrf5	Adgrf5		Adhesion G	no | no chan	0	ENOG410
O09030	I13R1_MO	Il13ra1	Il13ra1	16164	Interleukin-	no | no chan	0	ENOG410
P62897	CYC_MOU	Cycs	Cycs	13063	Cytochrom	no | no chan	0	COG3474(
P62892	RL39_MOU	Rpl39	Rpl39	67248	Large ribos	no | no chan	0	COG2167(
A0A571BE	A0A571BE	Ap3b2	Ap3b2	11775	AP-3 compl	no | no chan	0	COG5096(
A0A140LIJ	A0A140LIJ	Fam168a	Fam168a		Family witl	no | no chan	0	ENOG410

A0A140LIJ2	A0A140LIJ2	Tacc2	Tacc2		Transformi	no|no chan	0	ENOG410Y
Q32MF0	Q32MF0_M	Elf4	Elf4	56501	Predicted ge	no|up	0	ENOG4111
P43136	NR2F6_MO	Nr2f6	Nr2f6	13864	Nuclear rec	no|no chan	0	ENOG410X
P43135	COT2_MO	Nr2f2	Nr2f2	11819	COUP tran	yes|down	1	ENOG410X
P42669	PURA_MO	Pura	Pura	19290	Transcripti	no|no chan	0	ENOG410Z
A0JNZ1	A0JNZ1_M	Pik3cb	Pik3cb	74769	phosphatid	no|no chan	0	COG5032(t
Q62148	AL1A2_MO	Aldh1a2	Aldh1a2	19378	Retinal deh	no|down	0	COG1012(p
D3Z7K5	D3Z7K5_M	Paqr7	Paqr7		Progestin a	no|no chan	0	COG1272(C
C6EQJ2	C6EQJ2_M	Anapc10	Anapc10	68999	Anaphase-p	no|no chan	0	COG5156(c
G3XA34	G3XA34_M	Zfp316	Zfp316	54201	Zinc finger	no|up	0	COG5048(r
G3XA30	G3XA30_M	Nsmce4a	Nsmce4a	67872	Non-structu	no|no chan	0	COG5125(r
Q5EBQ0	Q5EBQ0_M	Vdac3	Vdac3	22335	Voltage-dep	no|no chan	0	ENOG410Z
B2RQK7	B2RQK7_M	Synpo2l	Synpo2l	68760	Synaptopoc	no|no chan	0	ENOG410Z
Q3U5C0	Q3U5C0_M	Tbc1d17	Tbc1d17	233204	Rab-GAP T	no|no chan	0	COG5210(C
A0A0R4J03	A0A0R4J03	Hrg	Hrg	94175	Histidine-ri	no|up	0	ENOG4111
A0A0R4J03	A0A0R4J03	Kng1	Kng1	16644	Kininogen	no|up	0	ENOG4111
A0A0R4J03	A0A0R4J03	Cenpk	Cenpk	60411	Centromere	no|no chan	0	ENOG4111
A0A0R4J03	A0A0R4J03	Nefm	Nefm	18040	Neurofilam	no|no chan	0	ENOG410X
A0A0R4J03	A0A0R4J03	Pdxdc1	Pdxdc1	94184	Pyridoxal-d	no|no chan	0	ENOG410X
A0A0R4J03	A0A0R4J03	C9	C9	12279	Complemen	no|down	0	ENOG410X
A0A0R4J03	A0A0R4J03	Trim35	Trim35	66854	Tripartite n	no|down	0	ENOG410Y
Q8R1T1	CHMP7_M	Chmp7	Chmp7	105513	Charged mu	no|no chan	0	ENOG411C
Q8K409	DPOLB_M	Polb	Polb	18970	DNA polym	no|no chan	0	COG1796(C
E9Q2W8	E9Q2W8_M	Borcs5	Borcs5	67774	BLOC-1-rel	no|no chan	0	ENOG410Y
P21614	VTDB_MO	Gc	Gc	14473	Vitamin D-	no|up	0	ENOG4111
Q8K403	Q8K403_M	H2rsp	H2rsp	72275	HAI-2 relat	no|up	0	ENOG411C
Q9CYQ7	NARF_MO	Narf	Narf	67608	Nuclear pre	no|no chan	0	COG4624(r
B2KFX4	B2KFX4_M	Cenps	Cenps	69928	Centromere	no|up	0	ENOG410X
P16015	CAH3_MO	Ca3	Ca3	12350	Carbonic an	no|no chan	0	COG3338(N
Q80XR7	Q80XR7_M	Zfp955a	Zfp955a	77652	Zinc finger	no|down	0	COG5048(S
B9EKT8	B9EKT8_M	Cep162	Cep162	382090	Centrosome	no|no chan	0	ENOG410X
Q9QUR8	SEM7A_MO	Sema7a	Sema7a	20361	Semaphorin	no|no chan	0	ENOG410X
A2ALK8	PTN3_MO	Ptpn3	Ptpn3	545622	Tyrosine-pr	no|no chan	0	COG5599(F
Q2VPA6	HELQ_MO	Helq	Helq	191578	Helicase PO	no|no chan	0	COG1204(p
Q5SX53	Q5SX53_M	Slc25a11	Slc25a11	67863	Mitochondr	no|no chan	0	ENOG410X
A0A571BD	A0A571BD	Xirp1	Xirp1		Xin actin-b	no|up	0	ENOG410Z
Q8BYN3	ITPK1_MO	Itpk1	Itpk1	217837	Inositol-tet	no|no chan	0	ENOG411C
A0A498WG	A0A498WG	Txnl1	Txnl1		Thioredoxin	yes|up	1	ENOG410Y
Q9DBT5	AMPD2_M	Ampd2	Ampd2	109674	AMP deam	no|no chan	0	COG1816(c
Q9DBT3	CCD97_MO	Ccdc97	Ccdc97	52132	Coiled-coil	no|no chan	0	ENOG4111
A0A087WQ	A0A087WQ	Nemp2	Nemp2		Nuclear en	no|up	0	ENOG410Z
A0A087WQ	A0A087WQ	Traf3ip1	Traf3ip1	74019	TRAF3 inte	no|no chan	0	ENOG410Z
A1L361	A1L361_MO	Tbk1	Tbk1	56480	TANK-bind	no|no chan	0	ENOG410X

Q5ND51	Q5ND51_M	Crk	Crk	12928	Adapter mo	no|no chan	0	ENOG411(
Q5ND52	MRM3_MC	Mrm3	Mrm3	67390	rRNA meth	no|no chan	0	COG0566(M
A0A0D4BT	A0A0D4BT	Magp1	Magp1	17150	Microfibril-	no|down	0	ENOG410Y
Q921I1	TRFE_MO	Tf	Tf	22041	Serotransfe	no|up	0	ENOG410X
A0A0A0MC	A0A0A0MC	Mapkbp1	Mapkbp1	26390	Mitogen-ac	no|no chan	0	ENOG410Y
A0A0A0MC	A0A0A0MC	Tuba4a	Tuba4a		Tubulin alp	no|up	0	COG5023(S
Q9CZ30	OLA1_MO	Ola1	Ola1	67059	Obg-like AT	no|no chan	0	COG0012(c
E9PXC0	E9PXC0_M	Srp54c	Srp54c	100101806	Signal recog	no|no chan	0	COG0541(I
E9PXC2	E9PXC2_M	Usf2	Usf2		Upstream t	no|up	0	ENOG4111
P58021	TM9S2_MC	Tm9sf2	Tm9sf2	68059	Transmemb	no|no chan	0	ENOG410X
Q8VEB2	SAV1_MOU	Sav1	Sav1	64010	Protein salv	no|no chan	0	ENOG410Z
P58022	LOXL2_MC	Loxl2	Loxl2	94352	Lysyl oxida	no|no chan	0	ENOG410X
Q8VEB4	PAG15_MC	Pla2g15	Pla2g15	192654	Lysosomal p	no|no chan	0	ENOG410Y
Q8R2Y2	MUC18_MC	Mcam	Mcam	84004	Cell surface	no|down	0	ENOG411(
Q9Z183	PADI4_MC	Padi4	Padi4	18602	Protein-arg	no|no chan	0	ENOG410Z
Q8R0J7	VP37B_MC	Vps37b	Vps37b	330192	Vacuolar p	no|no chan	0	ENOG4111
Q8VHN8	TIRR_MOU	Nudt16l1	Nudt16l1	66911	Tudor-inter	no|no chan	0	ENOG4111
A0A3Q4EC	A0A3Q4EC	Ttc39c	Ttc39c	72747	Tetratricop	no|no chan	0	ENOG410X
Q3TC56	Q3TC56_M	Oxa1l	Oxa1l	69089	Membrane	no|no chan	0	COG0706(F
Q3U1D0	LINES_MC	Lins1	Lins1	72635	Protein Lin	no|no chan	0	ENOG410X
Q8R0J8	GNTK_MC	Idnk	Idnk	75731	Probable gl	no|up	0	COG3265(c
Q3TLZ6	Q3TLZ6_M	Gatb	Gatb	229487	Glutamyl-t	no|no chan	0	COG0064(A
Q3TLZ7	Q3TLZ7_M	Dusp14	Dusp14	56405	Uncharacte	no|up	0	COG2453(F
A0A087WP	A0A087WP	Ktn1	Ktn1	16709	Kinectin 1	no|no chan	0	ENOG410Y
Q8R2Y3	DOLK_MC	Dolk	Dolk	227697	Dolichol ki	no|no chan	0	COG0170(p
G3UWS0	G3UWS0_M	Rpp21	Rpp21		Ribonuclea	no|no chan	0	COG2023(t
P81269	ATF1_MOU	Atf1	Atf1	11908	Cyclic AMP	no|no chan	0	ENOG410Z
A2A5S3	A2A5S3_M	Stau1	Stau1	20853	Staufen dou	no|no chan	0	ENOG410X
Q9QXN3	TRIP4_MO	Trip4	Trip4	56404	Activating s	no|no chan	0	ENOG410X
F6U529	F6U529_M	Gls	Gls		glutaminase	no|no chan	0	COG2066(C
A0A0A0MC	A0A0A0MC	Slc25a14	Slc25a14	20523	Solute carri	no|up	0	ENOG410X
Q9DCM2	GSTK1_MC	Gstk1	Gstk1	76263	Glutathion	no|no chan	0	COG3917(c
Q3UZN6	Q3UZN6_M	Pdcl	Pdcl	67466	Phosducin l	no|no chan	0	ENOG411(
A2A468	A2A468_M	Ncoa3	Ncoa3	17979	Nuclear rec	no|no chan	0	ENOG410X
Q8BZT9	LACC1_MC	Lacc1	Lacc1	210808	Purine nucl	no|no chan	0	COG1496(M
Q9Z1S0	BUB1B_MC	Bub1b	Bub1b	12236	Mitotic che	no|no chan	0	ENOG410X
A0A2X0SY	A0A2X0SY	ARHGAP1	ARHGAP1	73910	ARHGAP1	no|no chan	0	ENOG410X
A0A1R3UC	A0A1R3UC	Klk1b16	Klk1b16	16615	Kallikrein c	no|no chan	0	COG5640(p
A0A1R3UC	A0A1R3UC	Klna6	Klna6		Kallikrein c	no|up	0	COG5640(p
P11031	TCP4_MOU	Sub1	Sub1	20024	Activated R	no|no chan	0	ENOG410X
O70548	TELT_MO	Tcap	Tcap	21393	Telethonin	no|up	0	ENOG4111
P11032	GRAA_MC	Gzma	Gzma	14938	Granzyme A	no|no chan	0	COG5640(F
A6H663	A6H663_M	Bag3	Bag3	29810	BCL2-assoc	no|no chan	0	COG5021(p

O70546	KDM6A_M	Kdm6a	Kdm6a	22289	Lysine-spec	no | no chan	0	COG0457(I
P84309	ADCY5_M	Adcy5	Adcy5	224129	Adenylate c	no | down	0	COG2114(s
F8WJB9	F8WJB9_M	Evl	Evl	14026	Ena/VASP	no | up	0	ENOG410Y
Q3TXD3	Q3TXD3_M	Vat1	Vat1	26949	Enoyl reduc	no | no chan	0	COG0604(C
E9PVC7	E9PVC7_M	Cdk14	Cdk14		Cyclin-depe	no | no chan	0	ENOG410X
E9QA15	E9QA15_M	Cald1	Cald1		Caldesmon	no | no chan	0	ENOG410Z
P61226	RAP2B_MO	Rap2b	Rap2b	74012	Ras-related	no | no chan	0	COG1100(A
A3KGR9	A3KGR9_M	H13	H13		Histocompo	no | up	0	ENOG410X
P61222	ABCE1_MO	Abce1	Abce1	24015	ATP-bindir	no | no chan	0	COG1245(A
Q91WP9	Q91WP9_M	Pygl	Pygl	110095	Alpha-1,4 g	no | no chan	0	COG0058(F
F6V695	F6V695_MO	Arhgef39	Arhgef39		Rho guanin	no | no chan	0	ENOG411C
A2A614	A2A614_MO	Gngt2	Gngt2	14710	Guanine nu	no | up	0	ENOG4112
Q06180	PTN2_MO	Ptpn2	Ptpn2	19255	Tyrosine-pr	no | no chan	0	COG5599(c
G3UXZ5	G3UXZ5_M	Psme1	Psme1		Proteasome	no | up	0	ENOG410X
Q06185	ATP5I_MO	Atp5me	Atp5me	11958	ATP syntha	no | no chan	0	ENOG410X
Q9CQZ6	NDUB3_M	Ndufb3	Ndufb3	66495	NADH deh	no | no chan	0	ENOG4112
Q8R5H1	UBP15_MO	Usp15	Usp15	14479	Ubiquitin c	no | no chan	0	COG5560(I
P47930	FOSL2_MO	Fosl2	Fosl2	14284	Fos-related	no | no chan	0	ENOG4111
O35425	BOK_MOU	Bok	Bok	51800	Bcl-2-relate	no | no chan	0	ENOG4112
O35427	RPC9_MOU	Crcp	Crcp	12909	DNA-direc	no | no chan	0	ENOG4111
Q9CPV4	GLOD4_M	Glod4	Glod4	67201	Glyoxalase	no | no chan	0	ENOG410X
Q9CPV3	RM42_MO	Mrpl42	Mrpl42	67270	Large ribos	no | down	0	ENOG4111
Q9CPV2	APC16_MO	Anapc16	Anapc16	52717	Anaphase-p	no | no chan	0	ENOG4111
Q9CPV1	SKA1_MOU	Ska1	Ska1	66468	SKA compl	no | down	0	ENOG4111
Q60847	COCA1_MO	Col12a1	Col12a1	12816	Collagen al	no | down	0	ENOG4111
D3Z6X6	D3Z6X6_M	Ccn4	Ccn4		Cellular co	yes | up	1	ENOG410Z
A0A2I3BPI	A0A2I3BPI	Khnyn	Khnyn		KH and NY	no | no chan	0	ENOG410Z
E9QNW6	E9QNW6_M	Pafah2	Pafah2	100163	Platelet-act	no | no chan	0	ENOG4111
Q3UGI2	Q3UGI2_M	Gpatch2l	Gpatch2l	70373	G patch do	no | no chan	0	ENOG4111
Q8BIJ6	SYIM_MOU	Iars2	Iars2	381314	Isoleucine--	no | no chan	0	COG0060(c
Q60848	HELLS_MO	Hells	Hells	15201	Lymphocyte	no | no chan	0	COG0553(c
G3UWD2	G3UWD2_	Runx1	Runx1	12394	Runt-relate	no | no chan	0	ENOG4111
Q8C0I4	EPC2_MOU	Epc2	Epc2	227867	Enhancer o	no | no chan	0	ENOG410X
Q8VBW5	BBX_MOU	Bbx	Bbx	70508	HMG box t	no | no chan	0	ENOG410Y
Q3USH5	SFSWA_MO	Sfswap	Sfswap	231769	Splicing fac	no | no chan	0	ENOG410Z
G3UWD8	G3UWD8_	Rchy1	Rchy1	68098	Ring finger	no | no chan	0	ENOG410X
P55200	KMT2A_M	Kmt2a	Kmt2a	214162	Histone-lys	no | no chan	0	COG2940(c
Q9CQZ1	HSBP1_MO	Hsbp1	Hsbp1	68196	Heat shock	no | up	0	ENOG4112
Q3TGI0	Q3TGI0_M	Xrcc1	Xrcc1	22594	BRCT dom	no | no chan	0	ENOG410Z
C7G3P2	C7G3P2_M	Prpf4b	Prpf4b	19134	Serine/thre	no | no chan	0	ENOG410X
H7BX94	H7BX94_M	Knop1	Knop1		Lysine rich	no | no chan	0	ENOG4112
A2ATK9	A2ATK9_M	Fam171a1	Fam171a1	269233	Family with	no | no chan	0	ENOG410Z
O35640	ANXA8_M	Anxa8	Anxa8	11752	Annexin A8	no | no chan	0	ENOG410X

O35643	AP1B1_MC	Ap1b1	Ap1b1	11764	AP-1 compl	no	no chan	0	COG5096(/
X5J4H8	X5J4H8_MOUSE	IgG1 heavy chain VDJ regior			IgG1 heavy	no	up	0	ENOG4111
A0PJ67	A0PJ67_MC	Nsrp1	Nsrp1	237859	Nuclear spe	no	no chan	0	ENOG410Z
O35646	CAN6_MO	Capn6	Capn6	12338	Calpain-6	no	no chan	0	ENOG410X
Q8BWA4	Q8BWA4_I	Tfap4	Tfap4	83383	BHLH dom	no	no chan	0	ENOG4111
Q8C804	SPICE_MC	Spice1	Spice1	212514	Spindle anc	no	no chan	0	ENOG410Y
Q3U829	AP5Z1_MC	Ap5z1	Ap5z1	231855	AP-5 compl	no	no chan	0	ENOG410X
F7C0H6	F7C0H6_M	Cnrip1	Cnrip1	380686	CB1 cannal	no	down	0	ENOG4112
Q8R2S8	CD177_MC	Cd177	Cd177	68891	CD177 anti	no	up	0	ENOG4111
S4R2K0	DEFM_MC	Pdf	Pdf	68023	Peptide def	no	no chan	0	COG0242(F
Q9CQZ5	NDUA6_M	Ndufa6	Ndufa6	67130	NADH deh	no	no chan	0	ENOG4111
Q3TJA9	Q3TJA9_M	Dnaja3	Dnaja3	83945	DnaJ homo	no	no chan	0	COG0484(C
Q8K2F8	LS14A_MO	Lsm14a	Lsm14a	67070	Protein LSN	no	no chan	0	ENOG4112
A0AA74KT	A0AA74KT	Brd7	Brd7		Bromodom	no	no chan	0	ENOG410X
Q3TAS0	Q3TAS0_M	Rgs3	Rgs3	50780	RGS domai	no	no chan	0	ENOG410Y
Q3UV57	Q3UV57_M	Dcaf5	Dcaf5	320808	Uncharacte	no	no chan	0	ENOG410Z
Q8BGI5	PEX26_MC	Pex26	Pex26	74043	Peroxisome	no	no chan	0	ENOG4111
P70700	RPA2_MO	Polr1b	Polr1b	20017	DNA-direc	no	no chan	0	COG0085(C
E9Q7L5	E9Q7L5_M	Maml2	Maml2	270118	Mastermin	no	no chan	0	ENOG4111
Q9DCS9	NDUBA_M	Ndufb10	Ndufb10	68342	NADH deh	no	no chan	0	ENOG4111
D3YU58	D3YU58_M	Anapc7	Anapc7		Anaphase p	no	no chan	0	ENOG410X
E9Q7L1	E9Q7L1_M	Urb2	Urb2	382038	URB2 ribos	no	no chan	0	ENOG410Z
P21619	LMNB2_M	Lmnb2	Lmnb2	16907	Lamin-B2	no	no chan	0	ENOG410Y
Q9DCS2	MTL26_MC	Mettl26	Mettl26	68347	Methyltran	no	down	0	ENOG4111
Q9DCS3	MECR_MC	Mecr	Mecr	26922	Enoyl-[acyl	no	no chan	0	COG0604(c
D3YU56	D3YU56_M	Lemd3	Lemd3		LEM doma	no	no chan	0	ENOG410X
Q61790	LAG3_MO	Lag3	Lag3	16768	Lymphocyt	yes	up	1	ENOG410Z
A2AGS6	A2AGS6_M	Trp53i11	Trp53i11		Tumor prot	no	no chan	0	ENOG4111
B1AXJ1	B1AXJ1_M	Tepsin	Tepsin		TEPSIN, ac	no	no chan	0	ENOG410Z
A0A1L1SSX	A0A1L1SSX	Bud13	Bud13		BUD13 hor	no	no chan	0	ENOG410X
F8VPV3	F8VPV3_M	Zic2	Zic2	22772	Zinc finger	yes	down	1	COG5048(z
Q8R3K3	PTCD2_MC	Ptcd2	Ptcd2	68927	Pentatricop	no	no chan	0	ENOG4111
Q3KNM9	Q3KNM9_	Apoo	Apoo	68316	MICOS con	no	no chan	0	ENOG4112
P28738	KIF5C_MC	Kif5c	Kif5c	16574	Kinesin hec	no	down	0	COG5059(N
Q9D6K9	CERS5_MC	Cers5	Cers5	71949	Ceramide sy	no	no chan	0	COG5058(s
Q8BPM2	M4K5_MO	Map4k5	Map4k5	399510	Mitogen-ac	no	no chan	0	ENOG410X
D3Z793	D3Z793_M	Gtf2a2	Gtf2a2		Transcripti	no	no chan	0	COG5123(T
Q3V2C3	Q3V2C3_M	Six1	Six1	20471	Homeobox	no	down	0	ENOG410X
Q3U3B9	Q3U3B9_M	Syap1	Syap1	67043	BSD domai	no	no chan	0	ENOG410Z
D3Z794	D3Z794_M	Sumo2	Sumo2		Small ubiq	no	down	0	COG5227(s
A0A0R4J0M	A0A0R4J0M	Tbcc	Tbcc	72726	Tubulin-spe	no	no chan	0	ENOG410Y
Q8BW44	Q8BW44_M	Acsl4	Acsl4	50790	long-chain-	no	no chan	0	COG1022(T
A0A0R4J0M	A0A0R4J0M	Atp13a4	Atp13a4	224079	Cation-tran	no	up	0	ENOG410X

Q8R5H6	WASF1_M(	Wasf1	Wasf1	83767	Actin-bindi	no|up	0	ENOG410Z
A0A0R4J0M	A0A0R4J0M	Apof	Apof	103161	Apolipopro	no|up	0	ENOG4112
Q3T992	Q3T992_M	Ubqln1	Ubqln1	56085	Ubiquilin-1	no|no chan	0	COG5272(C
P06728	APOA4_M(	Apoa4	Apoa4	11808	Apolipopro	no|up	0	ENOG4111
A0A0R4J0M	A0A0R4J0M	Vcpip1	Vcpip1	70675	ubiquitinyl	no|no chan	0	ENOG410Z
Q05BF0	Q05BF0_M	Chchd1	Chchd1	66121	Chchd1 pro	no|no chan	0	ENOG410Y
Q9EQ80	NIF3L_MC	Nif3l1	Nif3l1	65102	NIF3-like p	no|no chan	0	COG0327(c
A0A0A6YW	A0A0A6YW	Tbc1d8	Tbc1d8		TBC1 domc	no|no chan	0	COG5210(r
Q921C3	BRWD1_M	Brwd1	Brwd1	93871	Bromodom	no|no chan	0	COG2319(v
A0A1L1SRI	A0A1L1SRI	Cmtm7	Cmtm7		CKLF-like	yes|up	1	ENOG4111
Q6RI63	F120B_MO	Fam120b	Fam120b	67544	Constitutiv	no|no chan	0	ENOG410Z
P68134	ACTS_MO	Acta1	Acta1	11459	Actin, alph	no|up	0	COG5277(c
E9Q4X2	E9Q4X2_M	Uggt2	Uggt2	66435	UDP-gluco	no|no chan	0	ENOG410X
B8JJI9	B8JJI9_MO	Nr2f1	Nr2f1	13865	Nuclear rec	no|down	0	ENOG410X
P00920	CAH2_MO	Ca2	Ca2	12349	Carbonic a	no|no chan	0	COG3338(h
Q8BW41	PMGT2_M	Pomgnt2	Pomgnt2	215494	Protein O-l	no|no chan	0	ENOG410Z
A0A0B4J1I	A0A0B4J1I	Igkv16-104	Igkv16-104	381778	Immunoglo	no|up	0	ENOG410Y
A0A0B4J1I	A0A0B4J1I	Igkv4-68	Igkv4-68	384515	Immunoglo	no|no chan	0	ENOG4111
A0A0B4J1I	A0A0B4J1I	Igkv4-55	Igkv4-55	385253	Immunoglo	no|no chan	0	ENOG4111
P58802	TB10A_MC	Tbc1d10a	Tbc1d10a	103724	TBC1 domc	no|no chan	0	ENOG410X
P58801	RIPK2_MC	Ripk2	Ripk2	192656	Receptor-in	no|no chan	0	COG0515(T
Q3TKM9	Q3TKM9_M	Arpc5	Arpc5	67771	Actin-relat	no|no chan	0	ENOG4111
A0A023J68	A0A023J68	ND3	ND3		NADH-ubi	yes|up	1	COG0838(N
P35550	FBRL_MO	Fbl	Fbl	14113	rRNA 2'-O-	no|no chan	0	COG1889(I
Q05186	RCN1_MO	Rcn1	Rcn1	19672	Reticulocal	no|no chan	0	ENOG410X
P49070	CAMLG_M	Camlg	Camlg	12328	Guided ent	no|no chan	0	ENOG4111
B1B1A8	B1B1A8_M	Mylk	Mylk	107589	Myosin ligh	no|no chan	0	ENOG410X
Q8R050	ERF3A_M(	Gspt1	Gspt1	14852	Eukaryotic	no|no chan	0	COG5256(c
Q9CQI7	RU2B_MO	Snrpb2	Snrpb2	20639	U2 small n	no|no chan	0	ENOG410X
Q544U7	Q544U7_M	Rab27a	Rab27a	11891	Ras-related	no|no chan	0	ENOG410X
O08532	CA2D1_M(	Cacna2d1	Cacna2d1	12293	Voltage-de	no|up	0	ENOG410X
A0A1Y7VP	A0A1Y7VP	Mideas	Mideas		Mitotic dea	no|no chan	0	ENOG4110
A0A1L1STI	A0A1L1STI	Mindy2	Mindy2		Ubiquitin c	no|up	0	ENOG4110
O08539	BIN1_MO	Bin1	Bin1	30948	Myc box-de	no|no chan	0	ENOG410X
O08538	ANGP1_M	Angpt1	Angpt1	11600	Angiopoiet	no|up	0	ENOG410Z
G3X8S2	G3X8S2_M	Brf1	Brf1	72308	B-related fc	no|no chan	0	COG1405(S
A0A494BA	A0A494BA	Csnk1g3	Csnk1g3	70425	Casein kina	no|no chan	0	ENOG410X
D3Z1Z8	D3Z1Z8_M	Stmn1	Stmn1		Stathmin	no|no chan	0	ENOG4111
Q1ERP8	CLM9_MO	Cd300lg	Cd300lg	52685	CMRF35-lil	no|down	0	ENOG4111
Q8BHL5	ELMO2_M	Elmo2	Elmo2	140579	Engulfmen	no|no chan	0	ENOG410X
F6VAN0	ATF6A_M(	Atf6	Atf6	226641	Cyclic AMI	no|no chan	0	ENOG410Z
A0A0R4J1I	A0A0R4J1I	Men1	Men1	17283	Menin	no|no chan	0	ENOG410Z
Q9D2I5	ARMC9_M	Armc9	Armc9	78795	LisH domai	no|no chan	0	ENOG410Y

Q05CL8	LARP7_MC	Larp7	Larp7	28036	La-related p	no|no chan	0 COG5193(c
A0A0R4J1I(	A0A0R4J1I(	Nup210	Nup210	54563	Nucleopori	no|no chan	0 ENOG410X
Q63932	MP2K2_M(	Map2k2	Map2k2	26396	Dual specif	no|no chan	0 ENOG410X
Q3UZQ6	Q3UZQ6_MOUSE	Nucleoporin p5		71844	Nucleopori	no|no chan	0 ENOG410Z
Q05CL2	Q05CL2_M	Proz	Proz	66901	Proz protei	no|no chan	0 COG5640(F
Q8K2Q7	BROX_MC	Brox	Brox	71678	BRO1 dom	yes|up	1 ENOG410Z
Q8CFV9	RIFK_MOU	Rfk	Rfk	54391	Riboflavin	no|no chan	0 COG0196(r
P52633	STAT6_MC	Stat6	Stat6	20852	Signal trans	no|no chan	0 ENOG410X
Q6IR34	GPSM1_M(	Gpsm1	Gpsm1	67839	G-protein-s	no|no chan	0 ENOG410X
Q80SU7	GVIN1_M(	Gvin1	Gvin1	100042856;	Interferon-i	no|no chan	0 ENOG410Y
Q8BYR1	TYW4_MO	Lcmt2	Lcmt2	329504	tRNA wybu	no|no chan	0 ENOG410Y
Q8BYR2	LATS1_MC	Lats1	Lats1	16798	Serine/thre	no|no chan	0 ENOG410X
B2RUR8	OTU7B_M(	Otud7b	Otud7b	229603	OTU doma	no|no chan	0 ENOG410X
B2RUR3	B2RUR3_M	Abcb1b	Abcb1b	18669	ATP-bindir	no|no chan	0 COG1132(F
Q3TK27	Q3TK27_M	Gnl3	Gnl3	30877	Guanine nu	no|no chan	0 COG1161(S
B2RUR0	B2RUR0_M	Fyb1	Fyb1	23880	FYN bindir	no|no chan	0 ENOG410Y
Q5SWR1	Q5SWR1_M	Ap2b1	Ap2b1	71770	AP comple	no|no chan	0 COG5096(r
P27577	ETS1_MOU	Ets1	Ets1	23871	Protein C-e	yes|down	1 ENOG410Z
Q6ZWP4	Q6ZWP4_M	Smarcb1	Smarcb1	20587	SWI/SNF-r	no|no chan	0 ENOG410X
Q8K368	FANCI_M(	Fanci	Fanci	208836	Fanconi an	no|no chan	0 ENOG410X
B8JK07	B8JK07_M(	Cyp4f13	Cyp4f13		Cytochrom	no|no chan	0 COG2124(S
Q8CDD9	LRIF1_MO	Lrif1	Lrif1	321000	Ligand-dep	no|no chan	0 ENOG4112
Q810K9	GXLT2_M(	Gxylt2	Gxylt2	232313	Glucoside x	no|down	0 ENOG410X
P32020	SCP2_MOU	Scp2	Scp2	20280	Sterol carri	no|no chan	0 ENOG410X
Q5U458	DJC11_MO	Dnajc11	Dnajc11	230935	DnaJ homo	no|no chan	0 COG0484(c
Q8CID0	CSR2B_MC	Kat14	Kat14	228714	Cysteine-ric	no|no chan	0 ENOG410Y
Q8VDK4	Q8VDK4_M	Cdh13	Cdh13	12554	Cadherin 1	no|no chan	0 ENOG410X
Q8BSE0	RMD2_MC	Rmdn2	Rmdn2	381110	Regulator c	no|no chan	0 ENOG4112
A0A5F8MP	A0A5F8MP	Trak1	Trak1		Trafficking	no|down	0 ENOG410X
Q8R059	GALE_MO	Gale	Gale	74246	UDP-gluco	no|no chan	0 COG1087(u
Q6PDY0	CC85B_MC	Ccdc85b	Ccdc85b	240514	Coiled-coil	no|no chan	0 ENOG410Y
E0CZ22	MROH1_M	Mroh1	Mroh1	223658	Maestro he	yes|up	1 ENOG410X
Q8K3A2	TRPT1_MC	Trpt1	Trpt1	107328	tRNA 2'-ph	no|no chan	0 COG1859(F
A0A494B92	A0A494B92	Papss2	Papss2	23972	3'-phospho	no|no chan	0 COG2046(r
G3UWY0	G3UWY0_M	Guf1	Guf1		GUF1 hom	no|no chan	0 COG0481(F
Q8K3A9	MEPCE_M	Mepce	Mepce	231803	7SK snRNA	no|no chan	0 ENOG410X
Q3TBR1	Q3TBR1_M	Gskip	Gskip	66787	GSKIP dom	no|no chan	0 ENOG4111
E9PVM1	E9PVM1_M	Inpp4b	Inpp4b	234515	phosphatid	yes|up	1 ENOG410Y
Q8R054	SRPX2_MC	Srpx2	Srpx2	68792	Sushi repea	no|no chan	0 ENOG4110
P48036	ANXA5_M	Anxa5	Anxa5	11747	Annexin A5	no|no chan	0 ENOG410X
P98203	ARVC_MO	Arvcf	Arvcf	11877	Splicing reg	no|no chan	0 ENOG410Y
Q8CI71	VPS50_MO	Vps50	Vps50	73288	Syndetin	no|no chan	0 ENOG410X
Q9WVC3	CAV2_MO	Cav2	Cav2	12390	Caveolin-2	no|no chan	0 ENOG4111

A0A6Q6PU	A0A6Q6PU	Prpf40a	Prpf40a		Pre-mRNA	no	no chan	0	COG5104(e
D3Z5F7	D3Z5F7_M	Gm20521	Gm20521		Predicted g	no	no chan	0	ENOG4111
D3Z5F0	D3Z5F0_M	Cdc42ep5	Cdc42ep5		CDC42 effe	no	no chan	0	ENOG410Z
D3Z5F2	D3Z5F2_M	Kics2	Kics2		KICSTOR s	no	no chan	0	ENOG411(
Q7TQK4	EXOS3_M(	Exosc3	Exosc3	66362	Exosome cc	no	no chan	0	COG1097(I
B7ZNR0	B7ZNR0_M	Ecm1	Ecm1	13601	Ecm1 prote	no	up	0	ENOG4111
A0A0J9YUI	A0A0J9YUI	Specc1	Specc1	432572	Sperm anti	no	down	0	COG5069(M
P26151	FCGR1_M(	Fcgr1	Fcgr1	14129	High affini	no	down	0	ENOG410Y
O54692	ZW10_MO	Zw10	Zw10	26951	Centromere	no	no chan	0	ENOG410X
A2A7P9	A2A7P9_M	Svbp	Svbp	69216	Small vasol	no	no chan	0	ENOG410Y
Q3UI08	Q3UI08_M	Tmem9b	Tmem9b	56786	Transmemb	no	no chan	0	ENOG4111
A2A7P4	A2A7P4_M	Zfp691	Zfp691	195522	Zinc finger	yes	down	1	COG5048(C
Q3UPH1	PRRC1_M(	Prrc1	Prrc1	73137	Protein PRI	no	no chan	0	ENOG410Z
B9EIU3	B9EIU3_M	Atrn	Atrn	11990	Attractin	no	down	0	ENOG410X
Q8K3H0	DP13A_M(	Appl1	Appl1	72993	DCC-intera	no	no chan	0	ENOG410Z
P22907	HEM3_MC	Hmbs	Hmbs	15288	Porphobilir	no	no chan	0	COG0181(T
Q8C622	Q8C622_M	H2bc22	H2bc22	319188	Histone H2	no	no chan	0	ENOG4111
Q3UPH7	ARH40_M(	Arhgef40	Arhgef40	268739	Rho guanin	no	no chan	0	ENOG410X
A0A1B0GR	A0A1B0GR	Tmem160	Tmem160		Transmemb	no	no chan	0	ENOG4111
Q5SUF2	LC7L3_MC	Luc7l3	Luc7l3	67684	Luc7-like p	no	no chan	0	COG5200(c
Q9D600	PSF2_MOU	Gins2	Gins2	272551	DNA replic	no	no chan	0	COG5093(I
E9PWQ3	E9PWQ3_M	Col6a3	Col6a3		Collagen, ty	no	down	0	ENOG411(
Q8VDK1	NIT1_MOU	Nit1	Nit1	27045	Deaminate	no	no chan	0	COG0388(V
Q6P5F9	XPO1_MO	Xpo1	Xpo1	103573	Exportin-1	no	no chan	0	COG5101(c
A0A087WR	A0A087WR	Fat1	Fat1	14107	FAT atypic	no	no chan	0	ENOG410X
Q6P5F6	S39AA_MC	Slc39a10	Slc39a10	227059	Zinc transp	no	no chan	0	COG0428(I
Q6P5F7	TTYH3_M(	Ttyh3	Ttyh3	78339	Protein twe	no	no chan	0	ENOG410Z
O70201	BIRC5_MC	Birc5	Birc5	11799	Baculoviral	yes	down	1	ENOG410Y
E9PZF0	E9PZF0_M	Nme1nme2	Nme1nme2		Nucleoside	no	no chan	0	COG0105(z
Q9JJF9	SPP2A_MC	Sppl2a	Sppl2a	66552	Signal pept	no	no chan	0	ENOG410Z
A9CLV6	A9CLV6_M	Serinc1	Serinc1	56442	Serine inco	no	up	0	ENOG410X
Q80X98	Q80X98_M	Dhx38	Dhx38	64340	RNA helica	no	no chan	0	COG1643(C
Q9CQ18	RNH2C_M	Rnaseh2c	Rnaseh2c	68209	Ribonuclea	no	no chan	0	ENOG4112
Q9JJF3	RIOX1_M(	Riox1	Riox1	71952	Ribosomal	no	no chan	0	ENOG410Y
B2RPV4	B2RPV4_M	Rbm12b2	Rbm12b2	77604	C430048L1(	no	no chan	0	ENOG4111
Q80X95	RRAGA_M	Rraga	Rraga	68441	Ras-related	no	no chan	0	ENOG410X
Q80X90	FLNB_MO	Flnb	Flnb	286940	Filamin-B	no	no chan	0	COG5069(T
Q9CQ10	CHMP3_M	Chmp3	Chmp3	66700	Charged mu	no	no chan	0	COG5491(S
G5E870	TRIPC_MC	Trip12	Trip12	14897	E3 ubiquiti	no	no chan	0	COG5021(A
G5E872	RNPL1_M(	Rnpepl1	Rnpepl1	108657	Aminopept	no	no chan	0	COG0308(S
A2AEP5	A2AEP5_M	Abca5	Abca5		ATP-bindir	no	no chan	0	COG1131((
Q61018	Q61018_M(	Gnai1	Gnai1	14677	Guanine nu	no	no chan	0	ENOG410X
Q6NS82	RETR2_M(	Retreg2	Retreg2	227298	Reticulophe	no	no chan	0	ENOG4111

F8WH69	F8WH69_M	Ncf1	Ncf1	17969	Neutrophil	no | no chan	0	ENOG410Y
F6VQ81	F6VQ81_M	Tpd52l2	Tpd52l2		Tumor prot	no | no chan	0	ENOG4111
D3Z0U4	D3Z0U4_M	Chrac1	Chrac1	93696	Chromatin	no | no chan	0	COG5208(
Q3TA68	Q3TA68_M	Wdr36	Wdr36	225348	WD repeat	no | no chan	0	ENOG410X
Q8BFW7	LPP_MOU	Lpp	Lpp	210126	Lipoma-pre	no | no chan	0	ENOG410Y
Q9CYC6	DCP2_MO	Dcp2	Dcp2	70640	m7GpppN-	no | no chan	0	COG0494(
A0A068BG	A0A068BG	Vps52	Vps52	224705	Vacuolar p	no | no chan	0	ENOG410X
P56528	CD38_MO	Cd38	Cd38	12494	ADP-ribosy	no | no chan	0	ENOG4111
B1AQF4	B1AQF4_M	Dusp3	Dusp3		Dual specif	no | no chan	0	COG2453(
O54946	DNJB6_M	Dnajb6	Dnajb6	23950	DnaJ homo	no | no chan	0	COG0484(
O54941	SMCE1_M	Smarce1	Smarce1	57376	SWI/SNF-r	no | no chan	0	ENOG410Y
Q3UED7	Q3UED7_M	Iigp1c	Iigp1c	240327	Interferon i	no | up	0	ENOG4112
Q8CGY8	OGT1_MO	Ogt	Ogt	108155	UDP-N-ace	no | no chan	0	COG3914(
Q8BHL3	TB10B_MC	Tbc1d10b	Tbc1d10b	68449	TBC1 domc	no | no chan	0	ENOG410X
A2RSS6	A2RSS6_M	Aga	Aga	11593	Aspartylglu	no | no chan	0	COG1446(
Q4VBG1	Q4VBG1_M	Ddx47	Ddx47	67755	RNA helico	no | no chan	0	ENOG410X
Q8CGY6	UN45B_M	Unc45b	Unc45b	217012	Protein unc	no | up	0	ENOG410X
B2RVP5	B2RVP5_M	H2az2	H2az2	77605	Histone H2	no | no chan	0	COG5262(
A0A0G2JF	A0A0G2JF	Pde5a	Pde5a		Phosphodie	no | no chan	0	ENOG410X
A2AL12	A2AL12_M	Hnrnpa3	Hnrnpa3		Heterogene	no | no chan	0	COG0724(
Q8BHL6	FAP24_MC	Faap24	Faap24	101831	Fanconi an	no | up	0	COG5241(
A0A6I8MX	A0A6I8MX	Ldhb	Ldhb	16832	L-lactate de	no | up	0	COG0039(
Q99J64	RBM43_M	Rbm43	Rbm43	71684	RNA-bindi	no | up	0	ENOG4112
Q91YR9	PTGR1_M	Ptgr1	Ptgr1	67103	Prostaglanc	no | no chan	0	COG2130(
Q91YR5	EFNMT_M	Mettl13	Mettl13	71449	eEF1A lysir	no | no chan	0	ENOG410X
Q3TDD8	Q3TDD8_M	Eif4b	Eif4b	75705	RRM doma	no | no chan	0	ENOG4111
Q3TDD9	PPR21_MC	Ppp1r21	Ppp1r21	73825	Protein phc	no | no chan	0	ENOG410Y
Q91YR1	TWF1_MO	Twf1	Twf1	19230	Twinfilin-1	no | no chan	0	ENOG410X
Q3U1T3	BRM1L_M	Brms1l	Brms1l	52592	Breast canc	no | no chan	0	ENOG410Y
E9Q600	E9Q600_M	Loxl4	Loxl4	67573	protein-lysi	no | up	0	ENOG410X
A2AQR0	A2AQR0_M	Gpd2	Gpd2		Glycerol-3-	no | no chan	0	COG0578(
E9Q605	E9Q605_M	Chuk	Chuk	12675	Inhibitor of	no | no chan	0	ENOG410X
Q80YX1	TENA_MC	Tnc	Tnc	21923	Tenascin	no | no chan	0	ENOG410Z
A2AQR4	A2AQR4_M	Zfp64	Zfp64	22722	Zinc finger	no | no chan	0	COG5048(
Q9JKL4	NDUF3_M	Ndufaf3	Ndufaf3	66706	NADH deh	no | no chan	0	ENOG4111
P35761	TTK_MOU	Ttk	Ttk	22137	Dual specif	no | no chan	0	ENOG410X
Q8BJQ2	UBP1_MO	Usp1	Usp1	230484	Ubiquitin c	no | no chan	0	ENOG410X
Q9CXH7	SGO1_MO	Sgo1	Sgo1	72415	Shugoshin 1	no | no chan	0	ENOG4111
P01646	KV5AD_MOUSE	Ig kappa chain V-V region H			Ig kappa ch	no | up	0	ENOG410Y
P01643	KV5AA_MOUSE	Ig kappa chain V-V region M			Ig kappa ch	no | up	0	ENOG410Y
P01642	KV5A9_M	Igkv5-48	Igkv5-48	619846	Immunoglo	no | up	0	ENOG410Y
B1AWB9	B1AWB9_M	Col5a1	Col5a1	12831	Collagen, ty	no | no chan	0	ENOG410X
Q9D7Y9	SLX4I_MO	Slx4ip	Slx4ip	74243	Protein SLX	no | no chan	0	ENOG4111

P01648	KV5AF_MOUSE Ig kappa chain V-V region HIg kappa ch				no | no chan		0	ENOG410Y
P09411	PGK1_MO	Pgk1	Pgk1	18655	Phosphogly	no | up	0	COG0126(p
A0A3Q4EB	A0A3Q4EB	Slc8a1	Slc8a1	20541	Sodium/cal	no | no chan	0	ENOG410X
D6RH77	D6RH77_M	Pld1	Pld1	18805	Phospholip	no | no chan	0	COG1502(c
P70398	USP9X_MC	Usp9x	Usp9x	22284	Ubiquitin c	no | no chan	0	COG5077(u
Q921H9	COA7_MO	Coa7	Coa7	69893	Cytochrom	no | no chan	0	COG0790(V
Q921H8	THIKA_MC	Acaa1a	Acaa1a	113868	3-ketoacyl-(	no | no chan	0	COG0183(c
Q9ESG9	PMYT1_MC	Pkmyt1	Pkmyt1	268930	Membrane-	no | no chan	0	ENOG410X
Q63930	Q63930_MC	Scgb1b27	Scgb1b27	11354	Salivary an	no | no chan	0	ENOG4111
A1Z2X7	A1Z2X7_M	Nphp1	Nphp1	53885	Nephrocyst	no | up	0	ENOG410X
P23298	KPCL_MO	Prkch	Prkch	18755	Protein kin	no | no chan	0	ENOG410X
Q8VD58	EVI2B_MC	Evi2b	Evi2b	216984; 101	Protein EV	no | no chan	0	ENOG4111
Q80ZD3	S2611_MO	Slc26a11	Slc26a11	268512	Sodium-ind	no | down	0	COG0659(r
Q80W94	MOGT2_M	Mogat2	Mogat2	233549	2-acylglycer	no | no chan	0	ENOG410X
A6XA75	A6XA75_M	Treml1	Treml1	71326	Triggering r	no | up	0	ENOG4111
E9QL43	E9QL43_M	Nup42	Nup42	231042	Nucleopori	no | no chan	0	ENOG4111
A0A0G2JE	A0A0G2JE	Cenpa	Cenpa	12615	Histone H3	yes | up	1	COG2036(C
Q3UZQ3	Q3UZQ3_M	Eef1a1	Eef1a1	13627	Elongation	no | down	0	COG5256(T
A0A1D5RM	A0A1D5RM	Psd3	Psd3		Pleckstrin a	no | down	0	COG5307(S
Q99M08	SRALN_MC	Arln	Arln	67704	Sarcoplasm	no | no chan	0	ENOG4111
Q91XL9	OSBL1_MC	Osbpl1a	Osbpl1a	64291	Oxysterol-b	no | no chan	0	ENOG410X
Q3TEZ2	Q3TEZ2_M	Wdr6	Wdr6	83669	tRNA (34-2	no | no chan	0	ENOG410Z
O89086	RBM3_MO	Rbm3	Rbm3	19652	RNA-bindi	no | no chan	0	ENOG410Y
Q91XL3	UXS1_MO	Uxs1	Uxs1	67883	UDP-glucu	no | no chan	0	COG0451(C
Q91XL1	Q91XL1_M	Lrg1	Lrg1	76905	Leucine-ric	no | up	0	COG4886(T
Q99N57	RAF1_MO	Raf1	Raf1	110157	RAF proto-	no | no chan	0	ENOG410Y
P59235	NUP43_MC	Nup43	Nup43	69912	Nucleopori	no | no chan	0	ENOG410Z
A0A3B2W3	A0A3B2W3	Ilrun	Ilrun		Inflammati	no | no chan	0	ENOG4112
A2N494	A2N494_MOUSE Aberrantly recombined kapp				Aberrantly	no | no chan	0	ENOG410Y
Q99104	MYO5A_M	Myo5a	Myo5a	17918	Unconventi	no | no chan	0	COG5022(r
Q4VBD9	GZF1_MO	Gzf1	Gzf1	74533	GDNF-ind	no | down	0	COG5048(t
A2AG68	A2AG68_M	Atp7a	Atp7a	11977	P-type Cu(+	no | no chan	0	COG2217(p
Q9QZR0	RNF25_MC	Rnf25	Rnf25	57751	E3 ubiquiti	no | no chan	0	ENOG4111
A0A0N4SW	A0A0N4SW	C2cd5	C2cd5	74741	C2 calcium	no | no chan	0	ENOG410X
A0A286YD	A0A286YD	Slc30a5	Slc30a5		Zinc transp	no | no chan	0	COG1230(T
P32233	DRG1_MC	Drg1	Drg1	13494	Developme	no | no chan	0	COG1163(C
A0A0G2JG	A0A0G2JG	S100a4	S100a4		S100 calciu	no | no chan	0	ENOG4111
Q3UX26	Q3UX26_M	Pak1ip1	Pak1ip1	68083	P21-activat	no | no chan	0	ENOG410X
Q9CQ79	TXND9_M	Txndc9	Txndc9	98258	Thioredoxi	no | no chan	0	COG0526(F
Q61161	M4K2_MO	Map4k2	Map4k2	26412	Mitogen-ac	no | no chan	0	ENOG410X
Q61160	FADD_MC	Fadd	Fadd	14082	FAS-associ	no | no chan	0	ENOG4112
A9XMT8	A9XMT8_M	Kif19a	Kif19a	286942	Kinesin-lik	no | no chan	0	COG5059(C
A0A1W2P7	A0A1W2P7	Xpot	Xpot	73192	Exportin-T	no | no chan	0	ENOG410X

D3YXX1	D3YXX1_M	Zmynd11	Zmynd11	66505	Zinc finger,	no|no chan	0	ENOG410X
P11416	RARA_MO	Rara	Rara	19401	Retinoic ac	no|no chan	0	ENOG410X
A0A0G2JD	A0A0G2JD	Lmo4	Lmo4		LIM domai	no|down	0	ENOG410Y
Q5SSK3	TEFM_MO	Tefm	Tefm	68550	Transcripti	no|no chan	0	ENOG410X
Q8CJ96	RASF8_MO	Rassf8	Rassf8	71323	Ras associa	no|no chan	0	ENOG410X
B2ZRS3	B2ZRS3_M	Deptor	Deptor	97998	Depdc6-001	no|no chan	0	ENOG4110
Q8VCR3	TM242_MO	Tmem242	Tmem242	70544	Transmemb	no|down	0	ENOG4111
D3Z7I0	D3Z7I0_M	Gtf3c2	Gtf3c2		General tra	no|no chan	0	ENOG410Y
E9QME3	E9QME3_M	Nsl1	Nsl1	381318	NSL1, MIS1	no|no chan	0	ENOG4112
P12849	KAP1_MO	Prkar1b	Prkar1b	19085	cAMP-depe	no|no chan	0	COG0664(f
P26369	U2AF2_MO	U2af2	U2af2	22185	Splicing fac	no|no chan	0	ENOG410X
Q8BIA4	FBXW8_M	Fbxw8	Fbxw8	231672	F-box/WD	no|no chan	0	ENOG4110
Q80U35	ARHGH_M	Arhgef17	Arhgef17	207212	Rho guanin	no|no chan	0	ENOG410X
Q6P1D7	SLX4_MOU	Slx4	Slx4	52864	Structure-sp	no|no chan	0	ENOG410X
A0A1B0GS	A0A1B0GS	Gab1	Gab1	14388	Growth fac	no|no chan	0	ENOG4111
Q8R1V4	TMED4_M	Tmed4	Tmed4	103694	Transmemb	no|no chan	0	ENOG410X
E9Q2Y3	E9Q2Y3_M	Ikbkg	Ikbkg	16151	NF-kappa-I	no|no chan	0	ENOG410Y
Q8K176	Q8K176_M	Taf1d	Taf1d	75316	TATA box-	no|up	0	ENOG4112
Q9CYW4	HDHD3_M	Hdhd3	Hdhd3	72748	Haloacid de	no|no chan	0	COG1011(F
A0A286YC	A0A286YC	Trappc8	Trappc8	75964	Trafficking	no|no chan	0	ENOG410X
A2APM1	A2APM1_M	Cd44	Cd44	12505	CD44 antig	no|no chan	0	ENOG4111
Q80XT2	Q80XT2_M	Abca3	Abca3	27410	Abca3 prot	no|no chan	0	COG1131(S
Q2VR03	Q2VR03_MOUSE	Anti-lipoteichoic acid heavy			Anti-lipotei	no|up	0	ENOG410Y
G5E8J9	G5E8J9_M	Scyl2	Scyl2	213326	SCY1-like 2	no|no chan	0	ENOG410X
G5E8J3	G5E8J3_M	Wdr11	Wdr11	207425	WD repeat	no|no chan	0	ENOG410X
P07903	ERCC1_MO	Ercc1	Ercc1	13870	DNA excisi	no|no chan	0	COG5241(L
P15626	GSTM2_M	Gstm2	Gstm2	14863	Glutathion	no|no chan	0	ENOG4110
P07901	HS90A_MO	Hsp90aa1	Hsp90aa1	15519	Heat shock	no|no chan	0	COG0326(F
A0A494BA	A0A494BA	Pyroxd2	Pyroxd2		Pyridine nu	no|no chan	0	COG1233(p
G5E8J4	G5E8J4_M	Ptpn21	Ptpn21	24000	Tyrosine-pr	no|up	0	COG5599(V
Q8JZX9	BORG1_M	Cdc42ep2	Cdc42ep2	104252	Cdc42 effec	no|no chan	0	ENOG4111
Q8K284	TF3C1_MO	Gtf3c1	Gtf3c1	233863	General tra	no|no chan	0	ENOG410X
Q8JZX4	SPF45_MO	Rbm17	Rbm17	76938	Splicing fac	no|no chan	0	ENOG410X
Q8K288	TP8L1_MO	Tnfaip8l1	Tnfaip8l1	66443	Tumor necr	no|no chan	0	ENOG4111
A0A087WQ	A0A087WQ	Clasp1	Clasp1	76707	CLIP associ	no|no chan	0	ENOG410Z
Q9WUM5	SUCA_MO	Suclg1	Suclg1	56451	Succinate--	no|no chan	0	COG0074(M
E9Q4C1	E9Q4C1_M	Klhl26	Klhl26	234378	Kelch-like 2	no|no chan	0	ENOG410X
A1L341	A1L341_M	Dyrk1a	Dyrk1a	13548	Dual specif	no|no chan	0	ENOG410X
Q8CCE5	Q8CCE5_M	Top2a	Top2a	21973	DNA topoi	no|no chan	0	COG0188(A
Q9QWF0	CAF1A_M	Chaf1a	Chaf1a	27221	Chromatin	no|no chan	0	ENOG410Z
Q3V028	Q3V028_M	Cyld	Cyld	74256	Ubiquitin c	no|no chan	0	ENOG410X
Q8VED9	LEGL_MO	Lgalsl	Lgalsl	216551	Galectin-re	no|no chan	0	ENOG4111
Q8VHL1	SETD7_M	Setd7	Setd7	73251	Histone-lys	no|no chan	0	COG2940(U

Q8VHL0	UT1_MOU	Slc14a1	Slc14a1	108052	Urea transp	no|up	0	COG4413(U
Q8VED2	BL1S4_MO	Bloc1s4	Bloc1s4	117197	Biogenesis (	no|no chan	0	ENOG4111
Q3V023	Q3V023_M	Naaa	Naaa	67111	N-acyletha	no|no chan	0	ENOG4111
Q8BH66	ATLA1_M(	Atl1	Atl1	73991	Atlastin-1	no|no chan	0	ENOG410X
Q3U4E4	Q3U4E4_M	Pstpip2	Pstpip2	19201	F-BAR dom	no|no chan	0	ENOG411(
Q8VED5	K2C79_MC	Krt79	Krt79	223917	Keratin, typ	no|down	0	ENOG410X
A6H6E2	MMRN2_M	Mmrn2	Mmrn2	105450	Multimerin	no|no chan	0	ENOG410Z
Q05421	CP2E1_MC	Cyp2e1	Cyp2e1	13106	Cytochrom	no|down	0	COG2124(c
Q8R0H9	GGA1_MC	Gga1	Gga1	106039	ADP-ribosy	no|no chan	0	ENOG410Y
Q3TC72	FAH2A_M(	Fahd2a	Fahd2a	68126	Oxaloaceta	no|no chan	0	COG0179(s
Q6R891	NEB2_MO	Ppp1r9b	Ppp1r9b	217124	Neurabin-2	no|no chan	0	ENOG410Y
E0CXV8	E0CXV8_M	Phc1	Phc1		Polyhomeo	no|no chan	0	ENOG4111
Q3UPU3	Q3UPU3_M	Ubr2	Ubr2	224826	E3 ubiquiti	yes|up	1	ENOG410X
P14685	PSMD3_M(	Psmd3	Psmd3	22123	26S proteas	no|no chan	0	ENOG410X
Q9CQR7	PEN2_MO	Psenen	Psenen	100042305;	Gamma-sec	no|no chan	0	ENOG4111
P63087	PP1G_MO	Ppp1cc	Ppp1cc	19047	Serine/thre	no|no chan	0	ENOG410X
Q9CQR5	ZMAT5_M	Zmat5	Zmat5	67178	Zinc finger	no|no chan	0	COG5136(C
A0A1G5SL	A0A1G5SL	OTTMUSW	OTTMUSW	100039796	Immunity r	no|up	0	ENOG4112
Q9Z1Q2	ABHGA_M	Abhd16a	Abhd16a	193742	Phosphatid	no|no chan	0	ENOG410X
Q9QXL8	NDK7_MC	Nme7	Nme7	171567	Nucleoside	no|no chan	0	COG0105(F
Q7M753	PANK2_M	Pank2	Pank2	74450	Pantothena	no|no chan	0	COG5146(F
Q99K70	RRAGC_M	Rragc	Rragc	54170	Ras-related	no|no chan	0	ENOG410X
A0A0R4J1T	A0A0R4J1T	Sorbs2	Sorbs2	234214	Sorbin and	no|down	0	ENOG410X
Q0P688	Q0P688_M	Eif4e2	Eif4e2	26987	Eukaryotic	no|no chan	0	COG5053(e
Q91V01	MBOA5_M	Lpcat3	Lpcat3	14792	Lysophosph	no|no chan	0	COG5202(C
P61028	RAB8B_M(	Rab8b	Rab8b	235442	Ras-related	no|no chan	0	ENOG410X
Q9D2V7	CORO7_M	Coro7	Coro7	78885	Coronin-7	no|no chan	0	ENOG410X
P61025	CKS1_MO	Cks1b	Cks1b	54124	Cyclin-depe	no|no chan	0	ENOG4111
P61027	RAB10_MC	Rab10	Rab10	19325	Ras-related	no|no chan	0	ENOG410X
P61793	LPAR1_M(	Lpar1	Lpar1	14745	Lysophosph	no|up	0	ENOG410X
P54116	STOM_MC	Stom	Stom	13830	Stomatin	no|no chan	0	COG0330(T
P85094	ISC2A_MO	Isoc2a	Isoc2a	664994	Isochorism	no|no chan	0	ENOG4111
E9PUR0	E9PUR0_M	Ankhd1	Ankhd1	108857	Ankyrin rep	no|no chan	0	COG0666(c
Q0VBU4	Q0VBU4_M	Ccdc47	Ccdc47	67163	PAT compl	no|no chan	0	ENOG410X
O70569	O70569_M(	rps14	rps14	20044	Small ribos	no|no chan	0	COG0100(I
P13504	IL1R1_MO	Il1r1	Il1r1	16177	Interleukin-	no|no chan	0	ENOG410Z
Q9CZ15	PSF1_MOU	Gins1	Gins1	69270	DNA replic	no|no chan	0	COG5230(L
A0A1W2P6	A0A1W2P6	Zc3h10	Zc3h10		Zinc finger	no|no chan	0	ENOG410Z
A0A068ET	A0A068ETZ5_MOUSE	Gag-Pol polyprotein O			Gag-Pol po	no|no chan	0	
A2AS70	A2AS70_M	Mllt10	Mllt10	17354	Myeloid/ly	no|up	0	COG5141(p
P63330	PP2AA_M(	Ppp2ca	Ppp2ca	19052	Serine/thre	no|no chan	0	COG0639(F
Q7TNG8	LDHD_MC	Ldhd	Ldhd	52815	Probable D	no|no chan	0	COG0277(F
F6UPM1	F6UPM1_M	Mlxip	Mlxip		MLX intera	no|no chan	0	ENOG410X

Q3TMB3	Q3TMB3_M	Fdps	Fdps	110196	Farnesyl py	no | no chan	0	COG0142(s
Q9D832	DNJB4_MC	Dnajb4	Dnajb4	67035	DnaJ homo	no | up	0	COG0484(S
Q9JI11	STK4_MOU	Stk4	Stk4	58231	Serine/thre	no | no chan	0	ENOG410X
Q7TNG5	EMAL2_M	Eml2	Eml2	72205	Echinodern	no | no chan	0	ENOG410X
Q68FC6	Q68FC6_M	Wdr43	Wdr43	72515	Wrd43 prot	no | no chan	0	ENOG410X
F8WGW3	F8WGW3_	Srbd1	Srbd1	78586	S1 RNA bir	no | no chan	0	COG2183(F
A0A0B6VM	A0A0B6VM	HC	HC		MAb 31C6	no | no chan	0	ENOG410Y
P55194	3BP1_MOU	Sh3bp1	Sh3bp1	20401	SH3 domai	no | no chan	0	ENOG410X
E9PU96	E9PU96_M	Urb1	Urb1	207932	URB1 ribos	no | no chan	0	ENOG410X
O35405	PLD3_MOU	Pld3	Pld3	18807	5'-3' exonuc	no | no chan	0	ENOG410X
E9PU93	E9PU93_M	Ash2l	Ash2l	23808	ASH2 like h	no | no chan	0	ENOG410Y
D3YYW8	D3YYW8_M	Rbm42	Rbm42		RNA-bindi	no | no chan	0	ENOG4111
P51569	AGAL_MC	Gla	Gla	11605	Alpha-gala	no | no chan	0	ENOG410X
Q9CWD8	NUBPL_M	Nubpl	Nubpl	76826	Iron-sulfur	no | no chan	0	COG0489(A
A2AQ19	RTF1_MOU	Rtf1	Rtf1	76246	RNA polym	no | no chan	0	COG5296(F
P47955	RLA1_MOU	Rplp1	Rplp1	56040	Large ribos	no | no chan	0	COG2058(F
Q9R1S8	CAN7_MO	Capn7	Capn7	12339	Calpain-7	no | no chan	0	ENOG410X
Q9CPP6	NDUA5_M	Ndufa5	Ndufa5	68202	NADH deh	no | no chan	0	ENOG4111
P31257	HXC10_MC	Hoxc10	Hoxc10	209448	Homeobox	no | down	0	ENOG4111
Q9D8D6	Q9D8D6_M	Csta2	Csta2	76770	2010005H1	no | up	0	ENOG4112
E9QLB2	E9QLB2_M	Lyplal1	Lyplal1	226791	palmitoyl-p	yes | down	1	COG0400(p
B1ASU9	B1ASU9_M	Tlk2	Tlk2	24086	Tousled-lik	no | no chan	0	ENOG410Y
D3Z6Z7	D3Z6Z7_M	Rasal3	Rasal3	320484	RAS protei	no | no chan	0	ENOG410X
E9QLB8	E9QLB8_M	Coq8b	Coq8b	76889	Coenzyme (	no | no chan	0	COG0661(p
Q9D3P8	PLRKT_MC	Plgrkt	Plgrkt	67759	Plasminoge	no | no chan	0	ENOG4111
Q3TJ28	Q3TJ28_M	Chmp1b	Chmp1b	67064	Charged mu	no | no chan	0	COG5491(C
Q3UUI3	THEM4_M	Them4	Them4	75778	Acyl-coenzy	no | no chan	0	ENOG4111
Q3UUI2	Q3UUI2_M	Slc4a1ap	Slc4a1ap	20534	FHA domai	no | no chan	0	ENOG410X
Q3USJ8	FCSD2_MC	Fchsd2	Fchsd2	207278	F-BAR and	no | no chan	0	ENOG410X
Q5XKE0	MYPC2_M	Mybpc2	Mybpc2	233199	Myosin-bin	no | up	0	ENOG4110
P41274	TNFL9_MC	Tnfsf9	Tnfsf9	21950	Tumor necr	no | up	0	ENOG4111
P31786	ACBP_MO	Dbi	Dbi	13167	Acyl-CoA-b	no | no chan	0	COG4281(A
Q9CU65	ZMYM2_M	Zmym2	Zmym2	76007	Zinc finger	no | no chan	0	ENOG410X
E9PV24	FIBA_MOU	Fga	Fga	14161	Fibrinogen	no | up	0	ENOG410Z
Q9R099	TBL2_MOU	Tbl2	Tbl2	27368	Transducin	no | no chan	0	COG2319(F
Q8BI72	CARF_MO	Cdkn2aip	Cdkn2aip	70925	CDKN2A-i	no | no chan	0	ENOG4111
A2ATI9	A2ATI9_M	Gorasp2	Gorasp2	70231	Golgi reass	no | no chan	0	COG5233(C
P37172	ACVR1_MC	Acvr1	Acvr1	11477	Activin rec	no | no chan	0	ENOG410X
E9QJS1	E9QJS1_M	Tyk2	Tyk2	54721	Tyrosine-pr	no | no chan	0	COG0515(t
Q6WIZ9	Q6WIZ9_M	Rnf31	Rnf31	268749	RBR-type E	no | no chan	0	ENOG410X
P07934	PHKG1_M	Phkg1	Phkg1	18682	Phosphoryl	no | no chan	0	ENOG410Y
Q3U379	Q3U379_M	Gpc1	Gpc1	14733	Glypican-1	no | no chan	0	ENOG410X
A0A571BF(	A0A571BF(	Mgam	Mgam	232714	Maltase-glu	no | no chan	0	COG1501(c

Q8BWG8	ARRB1_MO	Arrb1	Arrb1	109689	Beta-arresti	no|no chan	0	ENOG410
D3Z001	D3Z001_M	2310057J18	2310057J18	67719	RIKEN cD	no|no chan	0	ENOG4111
Q3TUE7	Q3TUE7_M	Cep41	Cep41		Rhodanese	no|no chan	0	ENOG410
D6RDC1	D6RDC1_M	Tmem41b	Tmem41b		Transmemb	no|no chan	0	COG0398(S
Q8VD00	SGMR2_M	Tmem97	Tmem97	69071	Sigma intra	no|no chan	0	ENOG4111
E9Q1Z0	E9Q1Z0_M	Krt90	Krt90	239673	Keratin 90	no|no chan	0	ENOG410
Q03265	ATPA_MO	Atp5f1a	Atp5f1a	11946	ATP syntha	no|no chan	0	COG0056(I
P70232	NCHL1_M	Chl1	Chl1	12661	Neural cell	no|no chan	0	ENOG410
P70236	MP2K6_M	Map2k6	Map2k6	26399	Dual specif	no|no chan	0	ENOG410
Q3TJG6	Q3TJG6_M	Ptges3	Ptges3	56351	Prostaglanc	no|no chan	0	ENOG4112
Q8R429	AT2A1_MO	Atp2a1	Atp2a1	11937	Sarcoplasm	no|up	0	COG0474(I
B2RRY4	B2RRY4_M	Pals1	Pals1	56217	Protein PA	no|no chan	0	COG0194(I
A0A0N4SW	A0A0N4SW	Dcun1d2	Dcun1d2	102323	DCN1-like	no|no chan	0	ENOG410
A2AGQ3	A2AGQ3_M	Madd	Madd		MAP kinas	no|no chan	0	ENOG410
B9EKB9	B9EKB9_M	Abcc10	Abcc10	224814	ATP-bindir	no|no chan	0	COG1132(S
P07759	SPA3K_MO	Serpina3k	Serpina3k	20714	Serine prot	no|no chan	0	COG4826(I
O88630	GOSR1_M	Gosr1	Gosr1	53334	Golgi SNA	no|no chan	0	ENOG410
I3PQW8	I3PQW8_M	Tifa	Tifa	211550	TRAF-inter	no|no chan	0	ENOG4111
Q9D172	GAL3A_M	Gatd3	Gatd3	28295	Glutamine	no|no chan	0	COG3155(I
P60229	EIF3E_MO	Eif3e	Eif3e	16341	Eukaryotic	no|no chan	0	ENOG410
Q78JW9	UBFD1_M	Ubfd1	Ubfd1	28018	Ubiquitin d	no|no chan	0	ENOG410
Q3UW71	Q3UW71_M	Ston1	Ston1	77057	Stonin-1	no|no chan	0	ENOG410
A0A571BD	A0A571BD	Prr33	Prr33		Proline rich	no|up	0	ENOG4111
Q9D7P6	ISCU_MOU	Iscu	Iscu	66383	Iron-sulfur	no|no chan	0	COG0822(s
Q8BPC6	IDHG2_MOUSE	Probable isocitra		243996	Probable is	no|down	0	COG0473(I
I6L966	I6L966_MO	Crp	Crp	12944	Pentraxin f	no|up	0	ENOG410
A0A0A6YX	A0A0A6YX	Kctd3	Kctd3		Potassium	no|no chan	0	ENOG410
Q8BPC1	Q8BPC1_MOUSE	Tyrosine-proteir		14302	Tyrosine-pr	no|no chan	0	COG0515(I
Q8R5J9	PRAF3_MO	Arl6ip5	Arl6ip5	65106	PRA1 famil	no|up	0	ENOG4111
Q9D1Q6	ERP44_MO	Erp44	Erp44	76299	Endoplasm	no|no chan	0	COG0526(T
A0MNP4	A0MNP4_M	Skic8	Skic8	66317	Superkiller	no|no chan	0	ENOG410
A2AFK4	A2AFK4_M	Mtg2	Mtg2	52856	Mitochond	yes|down	1	COG0536(S
Q6P3C8	Q6P3C8_M	Hspb2	Hspb2	69253	Heat shock	no|up	0	ENOG410
A2AN08	UBR4_MO	Ubr4	Ubr4	69116	E3 ubiquiti	no|no chan	0	ENOG410
A0A286YE	A0A286YE	Ube2e1	Ube2e1		E2 ubiquiti	no|no chan	0	COG5078(s
A0A2R8VI	A0A2R8VI	Sgsm3	Sgsm3		RUN and T	no|no chan	0	COG5210(Z
Q3UYU7	Q3UYU7_M	Unk	Unk	217331	C3H1-type	no|no chan	0	ENOG410
A8Y5N8	A8Y5N8_M	Plekhf2	Plekhf2		Pleckstrin h	no|no chan	0	ENOG410
Q922Y1	UBXN1_M	Ubxn1	Ubxn1	225896	UBX doma	no|no chan	0	ENOG4111
B7ZP45	B7ZP45_M	Lrig1	Lrig1	16206	Lrig1 prote	yes|down	1	COG4886(I
P53690	MMP14_M	Mmp14	Mmp14	17387	Matrix met	no|no chan	0	ENOG410
E9Q414	APOB_MO	Apob	Apob	238055	Apolipopro	no|up	0	ENOG4111
Q3ULW8	PARP3_MO	Parp3	Parp3	235587	Protein mo	no|up	0	ENOG410

E9Q411	E9Q411_M	Nbas	Nbas	71169	Neuroblast	no	no chan	0	ENOG410Y
E9Q418	E9Q418_M	Tgfbr1	Tgfbr1		TGF-beta r	no	no chan	0	ENOG410X
P27773	PDIA3_MC	Pdia3	Pdia3	14827	Protein dis	no	no chan	0	COG0526(X
F6TS96	F6TS96_M	Tle3	Tle3		Transducin	no	no chan	0	ENOG410X
A0A1Y7VM	A0A1Y7VM	Abcd4	Abcd4		ATP-bindir	no	no chan	0	COG4178(C
Q4VAA9	Q4VAA9_M	Metap1	Metap1	75624	Methionine	no	no chan	0	COG0024(C
A0AAA9W	A0AAA9W	Spi1	Spi1		Spleen focu	no	no chan	0	ENOG410X
Q8BGR9	UBCP1_MC	Ublcp1	Ublcp1	79560	Ubiquitin-l	no	no chan	0	COG5190(S
Q8BGR6	ARL15_MC	Arl15	Arl15	218639	ADP-ribosy	no	no chan	0	ENOG410X
Q4VAA7	SNX33_MC	Snx33	Snx33	235406	Sorting nex	no	no chan	0	ENOG410X
Q8BGR2	LRC8D_MC	Lrrc8d	Lrrc8d	231549	Volume-reg	no	no chan	0	COG4886(U
G3X8Q1	G3X8Q1_M	Cabin1	Cabin1	104248	Calcineurin	no	no chan	0	ENOG410X
G3X8Q0	G3X8Q0_M	Sp1	Sp1	20683	Trans-actin	no	no chan	0	COG5048(v
Q9CZ28	SNF8_MOU	Snf8	Snf8	27681	Vacuolar-sc	no	no chan	0	ENOG410X
Q80SW1	SAHH2_MC	Ahcyl1	Ahcyl1	229709	S-adenosylh	no	no chan	0	COG0499(M
Q80TM9	NISCH_MC	Nisch	Nisch	64652	Nischarin	no	no chan	0	ENOG410X
Q60790	RASA3_MC	Rasa3	Rasa3	19414	Ras GTPase	no	no chan	0	ENOG410Y
Q8CFX3	Q8CFX3_M	Pcdh1	Pcdh1	75599	Protocadhe	no	no chan	0	ENOG410X
A0A075B5J	A0A075B5J	Igkv17-127	Igkv17-127		Immunoglo	no	no chan	0	ENOG4111
Q543F8	Q543F8_M	Orc2	Orc2	18393	Origin reco	no	no chan	0	COG5575(c
D3Z3V2	D3Z3V2_M	St3gal2	St3gal2		Gal-NAc6S	no	no chan	0	ENOG410X
H3BJ40	H3BJ40_M	Arhgef2	Arhgef2	16800	Rho guanin	no	no chan	0	ENOG410X
Q9WV66	MARH7_M	Marchf7	Marchf7	57438	E3 ubiquiti	no	no chan	0	COG5183(C
Q3URU4	Q3URU4_M	Mre11a	Mre11a	17535	Double-strc	no	no chan	0	COG0420(L
P68510	1433F_MO	Ywhah	Ywhah	22629	14-3-3 prote	no	no chan	0	COG5040(c
Q8K341	ATAT_MO	Atat1	Atat1	73242	Alpha-tubu	no	no chan	0	ENOG4111
A0A338P78	A0A338P78	Ube2l3	Ube2l3		E2 ubiquiti	no	no chan	0	COG5078(r
A0A1D5RL	A0A1D5RL	Cherp	Cherp	27967	Calcium ho	no	no chan	0	ENOG410Y
P28867	KPCD_MC	Prkcd	Prkcd	18753	Protein kin	no	no chan	0	ENOG410X
P28862	MMP3_MC	Mmp3	Mmp3	17392	Stromelysin	no	no chan	0	ENOG410X
O88856	TPST2_MC	Tpst2	Tpst2	22022	Protein-tyr	no	no chan	0	ENOG410X
A2RT67	DEND3_M	Dennd3	Dennd3	105841	DENN don	no	no chan	0	ENOG410X
F8WIA3	F8WIA3_M	Gm20604	Gm20604	100859931	Predicted g	no	no chan	0	ENOG4111
A0A5F8MP	A0A5F8MP	Amd1	Amd1		adenosylme	yes	down	1	ENOG410X
A0A5F8MP	A0A5F8MP	Arhgef18	Arhgef18		Rho/Rac gu	no	no chan	0	COG5422(g
A0A5F8MP	A0A5F8MP	Kif1b	Kif1b		Kinesin fan	no	no chan	0	COG5059(c
A0A2I3BQ	A0A2I3BQ	Tmem260	Tmem260		Transmeml	no	no chan	0	ENOG410X
A0A5F8MP	A0A5F8MP	Mical3	Mical3		F-actin mo	no	no chan	0	COG5069(c
A0A0R4J17	A0A0R4J17	Dennd4b	Dennd4b	229541	DENN don	no	no chan	0	ENOG410X
A0A0R4J17	A0A0R4J17	Smarca4	Smarca4	20586	SWI/SNF r	no	no chan	0	COG5076(p
A0A0R4J17	A0A0R4J17	Dlc1	Dlc1	50768	Rho GTPas	no	no chan	0	ENOG410X
Q63918	CAVN2_M	Cavin2	Cavin2	20324	Caveolae-a	no	down	0	ENOG410X
A0A0R4J17	A0A0R4J17	Ccndbp1	Ccndbp1	17151	Cyclin-D1-l	no	no chan	0	ENOG410Z

A0A0R4J17	A0A0R4J17	Ecsit	Ecsit	26940	Evolutiona	no | no chan	0	ENOG411(
A0A0R4J17	A0A0R4J17	Brd9	Brd9	105246	Bromodom	no | no chan	0	ENOG410
Q9WVA4	TAGL2_M(	Tagln2	Tagln2	21346	Transgelin-	no | no chan	0	COG5199(r
Q9JMA1	UBP14_MC	Usp14	Usp14	59025	Ubiquitin c	no | no chan	0	ENOG410
Q9JMA2	TGT_MOU	Qtrt1	Qtrt1	60507	Queuine tR	no | no chan	0	COG0343((
E9M2E8	E9M2E8_M	Il33	Il33	77125	Interleukin-	no | no chan	0	ENOG4111
Q9WVA2	TIM8A_M(	Timm8a1	Timm8a1	30058	Mitochondı	no | no chan	0	ENOG4112
Q9ESL4	M3K20_M(	Map3k20	Map3k20	65964	Mitogen-ac	no | no chan	0	COG0515(I
Q3UK41	Q3UK41_M	Nelfa	Nelfa	24116	HDAg dom	no | no chan	0	ENOG410
Q5DTM8	BRE1A_M(	Rnf20	Rnf20	109331	E3 ubiquiti	no | no chan	0	ENOG410
Q9Z2I0	LETM1_M(	Letm1	Letm1	56384	Mitochondı	no | no chan	0	ENOG410
Q8BHE1	GEMI8_M(	Gemin8	Gemin8	237221	Gem-associ	no | no chan	0	ENOG4111
E9QKD1	E9QKD1_I	Nol8	Nol8	70930	Nucleolar p	no | no chan	0	ENOG410
B1AYC9	B1AYC9_M	Apbb1ip	Apbb1ip	54519	Amyloid be	no | no chan	0	ENOG410
D3Z5H3	D3Z5H3_M	Sec11c	Sec11c		Signal pept	no | no chan	0	COG0681(S
Q9D0Q7	RM45_MO	Mrpl45	Mrpl45	67036	Large ribos	no | no chan	0	ENOG411(
B7ZNP9	B7ZNP9_M	Ddit4	Ddit4	74747	DNA dama	no | no chan	0	ENOG4111
B7ZNP2	B7ZNP2_M	Commd6	Commd6	66200	COMM dor	no | no chan	0	ENOG4111
P0DP26	CALM1_M(	Calm1	Calm1	12315; 1231	Calmodulir	no | no chan	0	COG5126(r
E9Q1G8	E9Q1G8_M	Septin7	Septin7	235072	Septin	no | no chan	0	COG5019(t
Q8R4U7	LUZP1_M(	Luzp1	Luzp1	269593	Leucine zip	no | no chan	0	ENOG410
Q5H8C4	VP13A_MC	Vps13a	Vps13a	271564	Intermembı	no | no chan	0	COG5043(p
B2RQP1	B2RQP1_M	Rnasel	Rnasel	24014	Ribonuclea	no | no chan	0	COG0666(S
P19253	RL13A_MC	Rpl13a	Rpl13a	22121	Large ribos	no | no chan	0	COG0102(s
Q8R0I4	TM2D2_M(	Tm2d2	Tm2d2	69742	TM2 domai	no | no chan	0	ENOG4111
F7AEH4	F7AEH4_M	Rps12	Rps12		40S ribosor	no | no chan	0	COG1358((
Q08890	IDS_MOUS	Ids	Ids	15931	Iduronate 2	no | no chan	0	COG3119(T
Q9D338	RM19_MO	Mrpl19	Mrpl19	56284	Large ribos	no | no chan	0	COG0335(T
P56960	EXOSX_M	Exosc10	Exosc10	50912	Exosome cc	no | no chan	0	COG0349(I
Q80UI7	Q80UI7_M	Clec4a1	Clec4a1	269799	C-type lecti	no | no chan	0	ENOG410
E9PWW9	E9PWW9_I	Rsf1	Rsf1	233532	Remodeling	no | no chan	0	ENOG410
Q9JJD0	THA11_M(	Thap11	Thap11	59016	THAP dom	no | no chan	0	ENOG4111
Q8BGG7	UBS3B_MC	Ubash3b	Ubash3b	72828	Ubiquitin-c	no | no chan	0	ENOG410
O35874	SATT_MOI	Slc1a4	Slc1a4	55963	Neutral am	no | down	0	COG1301(E
Q3T9Z2	Q3T9Z2_M	Grhpr	Grhpr	76238	Glyoxylate	no | no chan	0	COG1052(I
E9PWW6	E9PWW6_I	Zc3h7a	Zc3h7a	106205	Zinc finger	no | no chan	0	ENOG411(
A2AER7	A2AER7_M	Pqbp1	Pqbp1	54633	Polyglutam	no | no chan	0	ENOG4111
Q102J0	Q102J0_M(	Ctbs	Ctbs	74245	Di-N-acety	no | up	0	COG3858(l
Q9CYH5	GFOD2_M	Gfod2	Gfod2	70575	Glucose-frı	no | no chan	0	COG0673(I
Q4VA93	Q4VA93_M	Prkca	Prkca	18750	Protein kin	no | no chan	0	ENOG410
Q9CQ71	RFA3_MOI	Rpa3	Rpa3	68240	Replication	no | no chan	0	ENOG4111
Q9CQ73	PKP2_MOI	Pkp2	Pkp2	67451	Plakophilin	no | no chan	0	ENOG410
Q9CQ75	NDUA2_M	Ndufa2	Ndufa2	17991	NADH deh	no | no chan	0	ENOG4112

J3QNT7	J3QNT7_M	Epn2	Epn2	13855	Epsin 2	no	no chan	0	ENOG410
Q9D1M4	MCA3_MO	Eef1e1	Eef1e1	66143	Eukaryotic	no	no chan	0	COG0625(
Q2M4G4	Q2M4G4_	Ube7ip5	Ube7ip5	140629	Ubiquitin c	no	down	0	COG5113(
A0A2I3BRI	A0A2I3BRI	Nfatc4	Nfatc4		Nuclear fac	no	up	0	ENOG4111
D2CGM4	D2CGM4_	Ifih1	Ifih1	71586	RNA helico	no	no chan	0	COG1111(
Q8BFQ8	GALD1_M	Gatd1	Gatd1	213350	Glutamine	no	no chan	0	ENOG410
G5E814	G5E814_M	Ndufa11	Ndufa11	69875	NADH deh	no	up	0	ENOG4111
Q8BKS6	Q8BKS6_M	Prepl	Prepl	213760	Prolyl endo	no	no chan	0	COG1770(
Q6NXK9	Q6NXK9_	Man1c1	Man1c1	230815	alpha-1,2-M	no	no chan	0	ENOG410
Q99J99	THTM_MC	Mpst	Mpst	246221	3-mercapto	no	no chan	0	COG2897(s
Q8R3A2	CDPF1_M	Cdpf1	Cdpf1	72355	Cysteine-ric	no	no chan	0	ENOG4111
Q3TD14	Q3TD14_M	Pigq	Pigq	14755	Phosphatid	no	down	0	ENOG410
Q6PGK3	Q6PGK3_	2700097O0	2700097O0	72658	RIKEN cD	no	no chan	0	ENOG410
Q3TD13	Q3TD13_M	Itgax	Itgax	16411	VWFA dom	no	up	0	ENOG410
Q3TA40	Q3TA40_M	6430548M0	6430548M0	234797	RIKEN cD	no	no chan	0	ENOG410
A0A1C9ZQ	A0A1C9ZQ	Cd300lf	Cd300lf	246746	CD300 anti	no	no chan	0	ENOG410
A0A1L1SV	A0A1L1SV	Actn4	Actn4		Alpha-actir	no	no chan	0	COG5069(
Q5S006	LRRK2_M	Lrrk2	Lrrk2	66725	Leucine-ric	no	no chan	0	COG4886(
O54927	WSB1_MO	Wsb1	Wsb1	78889	WD repeat	no	no chan	0	ENOG410
O54926	SIVA_MO	Siva1	Siva1	30954	Apoptosis r	no	up	0	ENOG411
A0A6I8MX	A0A6I8MX	Ccdc88a	Ccdc88a		Coiled coil	no	no chan	0	ENOG410
Q91YL3	UCKL1_M	Uckl1	Uckl1	68556	Uridine-cyt	no	no chan	0	COG0035(
A8E4K9	A8E4K9_M	Zfp280b	Zfp280b	64453	Zfp280b pr	no	no chan	0	COG5048(
Q80VA0	GALT7_M	Galnt7	Galnt7	108150	N-acetylgal	no	no chan	0	ENOG410
A0A2R8VK	A0A2R8VK	Larp4	Larp4	207214	La ribonucl	no	no chan	0	ENOG410
Q3UG25	Q3UG25_M	Nfkb2	Nfkb2	18034	RHD doma	no	no chan	0	COG0666(
P23927	CRYAB_M	Cryab	Cryab	12955	Alpha-cryst	no	up	0	ENOG410
Q3TCR9	Q3TCR9_M	Zyx	Zyx	22793	Zyxin	no	no chan	0	ENOG410
Q3TDF8	Q3TDF8_M	Etf1	Etf1	225363	Eukaryotic	no	no chan	0	COG1503(
Q3TDF9	Q3TDF9_M	Nagpa	Nagpa	27426	EGF-like d	no	no chan	0	ENOG410
Q19LI2	A1BG_MO	A1bg	A1bg	117586	Alpha-1B-g	no	up	0	ENOG4111
G3UXL2	G3UXL2_M	Prps1l3	Prps1l3	328099	ribose-phos	no	no chan	0	COG0462(
Q9CXJ1	SYEM_MO	Ears2	Ears2	67417	Nondiscrim	no	no chan	0	COG0008(
P29387	GBB4_MO	Gnb4	Gnb4	14696	Guanine n	no	no chan	0	ENOG410
Q80YV4	PANK4_M	Pank4	Pank4	269614	4'-phospho	no	no chan	0	COG5146(
A0A0N4SV	A0A0N4SV	Tra2a	Tra2a	101214	Transforme	no	no chan	0	COG0724(
E9Q624	E9Q624_M	Zfp644	Zfp644	52397	Zinc finger	no	down	0	COG5048(
Q8BJS8	MTBP_MO	Mtbp	Mtbp	105837	Mdm2-bind	no	no chan	0	ENOG410
Q5SW15	Q5SW15_M	Mettl16	Mettl16		U6 small n	no	no chan	0	COG3129(
Q8BJS4	SUN2_MO	Sun2	Sun2	223697	SUN domai	no	no chan	0	ENOG410
B1AWD8	B1AWD8_	Clta	Clta	12757	Clathrin lig	no	no chan	0	ENOG4111
Q3TSG4	ALKB5_M	Alkbh5	Alkbh5	268420	RNA demet	no	no chan	0	ENOG410
Q3UEB3	PUF60_MC	Puf60	Puf60	67959	Poly(U)-bin	no	no chan	0	ENOG410

A0A1L1SU:	A0A1L1SU:	Thy1	Thy1		Thy-1 mem	no|down	0	ENOG410Y
Q6P7T7	Q6P7T7_M	Rab2b	Rab2b	76338	Rab2b prot	no|up	0	COG1100(C
Q921F4	HNRLL_M	Hnrnpll	Hnrnpll	72692	Heterogene	no|no chan	0	ENOG410X
P48999	LOX5_MO	Alox5	Alox5	11689	Polyunsatu	no|no chan	0	ENOG410Y
Q8BLI4	DSE_MOU	Dse	Dse	212898	Dermatan-s	no|no chan	0	ENOG410Z
Q3UND4	Q3UND4_I	Ptprg	Ptprg	19270	protein-tyrc	no|up	0	COG5599(r
Q9QYM8	CENPH_M	Cenph	Cenph	26886	Centromere	no|up	0	ENOG410Y
A2RRY6	A2RRY6_M	Cthrc1	Cthrc1	68588	Collagen tr	no|no chan	0	ENOG4111
P17563	SBP1_MOU	Selenbp1	Selenbp1	20341	Methanethi	no|no chan	0	ENOG410X
A0A0G2JE:	A0A0G2JE:	Nexn	Nexn	68810	Nexilin	no|no chan	0	ENOG411C
Q00PI9	HNRL2_M	Hnrnpul2	Hnrnpul2	68693	Heterogene	no|no chan	0	ENOG411C
Q9Z0P5	TWF2_MO	Twf2	Twf2	23999	Twinfilin-2	no|no chan	0	ENOG410X
Q9Z1R9	Q9Z1R9_M	Prss1	Prss1	114228	Protease, se	no|up	0	COG5640(p
Q91VJ4	STK38_MC	Stk38	Stk38	106504	Serine/thre	no|no chan	0	ENOG410X
Q91VJ2	CAVN3_M	Cavin3	Cavin3	109042	Caveolae-a:	no|no chan	0	ENOG4111
Q91VJ1	AIM2_MOI	Aim2	Aim2	383619	Interferon-i	no|no chan	0	ENOG410Y
Q3TEX7	Q3TEX7_M	Mfn2	Mfn2	170731	Dynamin-ty	no|no chan	0	COG0699(E
Q3UZ28	Q3UZ28_M	Acin1	Acin1	56215	SAP domai	no|no chan	0	ENOG4111
Q3UWH9	Q3UWH9_MOUSE	Peptidyl-prolyl		66101	Peptidyl-pr	no|no chan	0	COG0652(v
A0A087WS:	A0A087WS:	Stat1	Stat1	20846	Signal trans	no|up	0	ENOG410X
Q61362	CH3L1_MC	Chi3l1	Chi3l1	12654	Chitinase-3	no|up	0	COG3325(c
Q9JKN1	ZNT7_MO	Slc30a7	Slc30a7	66500	Zinc transp	no|up	0	COG1230(C
Q924T2	RT02_MOU	Mrps2	Mrps2	118451	Small ribos	no|no chan	0	COG0052(3
P43247	MSH2_MO	Msh2	Msh2	17685	DNA mism	no|no chan	0	COG0249(t
Q91XS1	MTMR4_M	Mtmr4	Mtmr4	170749	Phosphatid	no|no chan	0	ENOG410X
J7NNX8	J7NNX8_M	Iigp1	Iigp1	60440; 4355	Interferon-g	no|up	0	ENOG4112
Q8CI03	FWCH1_M	Flywch1	Flywch1	224613	FLYWCH-t	no|up	0	ENOG410Y
Q9D6J6	NDUV2_M	Ndufv2	Ndufv2	72900	NADH deh	no|no chan	0	COG1905(N
Q8CI08	SLAI2_MO	Slain2	Slain2	75991	SLAIN mot	no|no chan	0	ENOG410X
A0A494BBI	A0A494BBI	Map3k11	Map3k11		Mitogen-ac	no|no chan	0	COG0515(E
O08967	CYH3_MO	Cyth3	Cyth3	19159	Cytohesin-3	no|no chan	0	COG5307(N
Q8BUB4	WDFY2_M	Wdfy2	Wdfy2	268752	WD repeat	no|no chan	0	COG2319(E
P60335	PCBP1_MC	Pcbp1	Pcbp1	23983	Poly(rC)-bi	no|no chan	0	ENOG410X
A0A286YD	A0A286YD	Zdhhc16	Zdhhc16		Zinc finger,	no|up	0	COG5273(s
A6X919	D19L1_MC	Dpy19l1	Dpy19l1	244745	Protein C-n	no|no chan	0	ENOG410X
D3YXZ5	D3YXZ5_M	Rbm47	Rbm47	245945	RNA-bindi	no|up	0	ENOG410X
Q330K0	Q330K0_M	Ccnq	Ccnq	69109	Cyclin M	no|no chan	0	COG5333(V
D3YXZ3	D3YXZ3_M	Klc2	Klc2		Kinesin ligl	no|no chan	0	COG0457(r
Q3THB4	Q3THB4_M	Ldha	Ldha	16828	L-lactate de	no|up	0	COG0039(C
E9Q5E0	E9Q5E0_M	Mef2d	Mef2d		Myocyte en	no|no chan	0	COG5068(T
P0C7T6	ATX1L_MC	Atxn1l	Atxn1l	52335	Ataxin-1-lil	no|no chan	0	ENOG410X
Q62189	SNRPA_M	Snrpa	Snrpa	53607	U1 small nu	no|no chan	0	ENOG410X
A0A3B2WI	A0A3B2WI	Lclat1	Lclat1		Lysocardiol	no|no chan	0	COG0204(C

Q62187	TTF1_MOUTtf1	Ttf1	22130	Transcripti	no|up		0	COG5147(M
Q18PH1	Q18PH1_MSlamf8	Slamf8	74748	BLAME	yes|up		1	ENOG410Y
Q8VCP8	KAD6_MOAk6	Ak6	102216272	Adenylate k	yes|up		1	COG1936(c
Q18PH8	Q18PH8_MCd48	Cd48	12506	CD48	no|no chan		0	ENOG410Z
Q01320	TOP2A_MOTop2a	Top2a	21973	DNA topoi	no|no chan		0	COG0188(L
Q8R1H0	HOP_MOUHopx	Hopx	74318	Homeodom	no|no chan		0	ENOG410Y
Q61982	NOTC3_M Notch3	Notch3	18131	Neurogenic	no|no chan		0	COG0666(C
A2A591	KRA31_MOKrtap3-1	Krtap3-1	69473	Keratin-ass	no|up		0	ENOG410X
Q3UVN4	Q3UVN4_MJag1	Jag1	16449	Delta-like p	no|no chan		0	ENOG410X
A1L0V6	A1L0V6_M Rigi	Rigi	230073	RNA helico	no|no chan		0	COG1111(c
A1L0V7	A1L0V7_M Rtf2	Rtf2	66404	Replication	no|no chan		0	ENOG4111
A0EQY3	A0EQY3_MNcapd3	Ncapd3	78658	Condensin-	no|no chan		0	ENOG410X
A2AWN8	A2AWN8_MYthdf1	Ythdf1	228994	YTH domai	no|no chan		0	ENOG410Y
Q9CXC3	MGME1_MMgme1	Mgme1	74528	Mitochondi	no|no chan		0	ENOG410X
P41245	MMP9_MOMmp9	Mmp9	17395	Matrix met	no|up		0	ENOG410X
G5E8T6	G5E8T6_MMagi3	Magi3	99470	Membrane-	no|no chan		0	COG5021(U
P41241	CSK_MOU Csk	Csk	12988	Tyrosine-pr	no|no chan		0	COG0515(r
A0A494BA	A0A494BA Sgms1	Sgms1		Phosphatid	no|no chan		0	ENOG410X
Q99MU3	DSRAD_M Adar	Adar	56417	Double-strc	no|no chan		0	ENOG410X
J3QMM7	J3QMM7_MNaxd	Naxd	69225	ATP-depen	no|no chan		0	COG0063(T
A0A0R4J0T	A0A0R4J0T Arfgap3	Arfgap3	66251	ADP-ribosy	no|no chan		0	COG5347(C
A0A0R4J0T	A0A0R4J0T Clec12a	Clec12a	232413	C-type lecti	no|up		0	ENOG410X
A0A0R4J0T	A0A0R4J0T Hscb	Hscb	100900	HscB iron-s	no|no chan		0	COG1076(p
A0A0R4J0T	A0A0R4J0T Ikbkb	Ikbkb	16150	IkappaB ki	no|no chan		0	ENOG410X
A0A0R4J0T	A0A0R4J0T Celf1	Celf1	13046	CUGBP, El	no|no chan		0	ENOG410X
P63321	RALA_MO Rala	Rala	56044	Ras-related	no|no chan		0	COG1100(R
P49222	EPB42_MCEpb42	Epb42	13828	Protein 4.2	no|no chan		0	ENOG410Y
P0DN34	NDUB1_MNdufb1	Ndufb1	102631912	NADH deh	no|no chan		0	ENOG410X
Q9DBP5	KCY_MOUCmpk1	Cmpk1	66588	UMP-CMP	no|no chan		0	COG0563(C
A1L329	A1L329_MNbr1	Nbr1	17966	Neighbor o	no|no chan		0	ENOG4111
A2AFP5	A2AFP5_MRab9	Rab9		RAB9, mem	no|no chan		0	ENOG410X
P43883	PLIN2_MCPlin2	Plin2	11520	Perilipin-2	no|up		0	ENOG410X
E9Q496	E9Q496_M Use1	Use1	67023	Vesicle tran	no|no chan		0	ENOG410Z
A0A1Y7VN	A0A1Y7VNGlrx	Glrx		Glutaredox	no|up		0	COG0695(Y
Q8BQN6	OZF_MOUZnf146	Znf146	26465	Zinc finger	no|no chan		0	COG5048(T
Q3TVV9	Q3TVV9_MGga2	Gga2	74105	ADP-ribosy	no|no chan		0	ENOG410Y
Q3TVV6	Q3TVV6_MHnrnpu	Hnrnpu	51810	Uncharacte	no|no chan		0	ENOG410Y
B1ATB3	B1ATB3_MTmc6	Tmc6	217353	Transmemb	no|no chan		0	ENOG410X
P63328	PP2BA_MCPpp3ca	Ppp3ca	19055	Protein pho	no|no chan		0	COG0639(R
Q3TC14	Q3TC14_MImpa2	Impa2	114663	Inositol-1-n	no|no chan		0	COG0483(i
Q9D4V7	RABL3_MCRabl3	Rabl3	67657	Rab-like pr	no|no chan		0	ENOG410Y
Q8C2E7	WASC5_MWashc5	Washc5	223593	WASH com	no|no chan		0	ENOG410X
Q571A2	Q571A2_M Cul2	Cul2	71745	MKIAA410	no|no chan		0	COG5647(c

Q3UDA4	Q3UDA4_M	Smarcb1	Smarcb1	20587	SWI/SNF-r	no | down	0	ENOG410X
E9Q368	TARB1_MO	Tarbp1	Tarbp1	212728	TAR RNA l	no | no chan	0	COG0566(i
A0A0A6YV	A0A0A6YV	Lin9	Lin9	72568	Protein lin-	no | no chan	0	ENOG410Y
A2A5W4	A2A5W4_M	Glrx2	Glrx2	69367	Glutaredox	no | no chan	0	COG0695(C
Q9JIX8	ACINU_MO	Acin1	Acin1	56215	Apoptotic c	no | no chan	0	ENOG4111
Q3UDA8	Q3UDA8_M	Cpt2	Cpt2	12896	Carnitine C	no | no chan	0	ENOG410X
A0A2K6ED	A0A2K6ED	Itih4	Itih4	16427	Inter alpha	no | up	0	COG2304(v
Q3U9C4	Q3U9C4_M	Mrpl44	Mrpl44	69163	Large ribos	no | no chan	0	ENOG410X
Q45VK7	DYHC2_M	Dync2h1	Dync2h1	110350	Cytoplasmi	no | no chan	0	COG5245(V
P12787	COX5A_M	Cox5a	Cox5a	12858	Cytochrom	no | no chan	0	ENOG4111
Q9CUX1	P52K_MOU	Thap12	Thap12	72981	52 kDa rep	no | no chan	0	ENOG4110
A0A0R4J1R	A0A0R4J1R	Pcbd2	Pcbd2	72562	4a-hydroxy	no | no chan	0	COG2154(4
A0A0R4J1R	A0A0R4J1R	Syvn1	Syvn1	74126	RING-type	no | no chan	0	COG5243(C
Q9D2P4	URM1_MO	Urm1	Urm1	68205	Ubiquitin-r	no | no chan	0	COG5131(t
O70503	DHB12_MO	Hsd17b12	Hsd17b12	56348	Very-long-c	no | no chan	0	COG0300(U
P70451	FER_MOU	Fer	Fer	14158	Tyrosine-pr	no | no chan	0	ENOG410Y
Q9DAP7	ASF1B_MO	Asf1b	Asf1b	66929	Histone ch	no | no chan	0	COG5137(A
Q9JIH2	NUP50_MO	Nup50	Nup50	18141; 7848	Nuclear po	no | no chan	0	ENOG410Y
Q1MWP9	Q1MWP9_	Ehd4	Ehd4	98878	EH-domain	no | no chan	0	ENOG410X
Q9QZH3	PPIE_MOU	Ppie	Ppie	56031	Peptidyl-pr	no | no chan	0	COG0652(c
A0A1G5SL	A0A1G5SL	OTTMUSG	OTTMUSG	103737	Peroxisome	yes | up	1	ENOG410Y
E9PXG9	E9PXG9_M	Vps53	Vps53		Vacuolar p	no | no chan	0	ENOG410X
A0A075B5C	A0A075B5C	Ighv2-5	Ighv2-5		Immunoglo	yes | down	1	ENOG410Y
B2RV73	B2RV73_M	Pofut2	Pofut2	80294	GDP-fucos	no | no chan	0	ENOG410Y
Q9EQU3	TLR9_MOU	Tlr9	Tlr9	81897	Toll-like re	no | no chan	0	COG4886(M
B2RV71	B2RV71_M	Card19	Card19	68480	Uncharacte	no | up	0	ENOG4111
Q3V1N5	Q3V1N5_M	Heca	Heca	380629	Hdc homol	no | no chan	0	ENOG410X
Q9D818	SAPC2_MO	Sapcd2	Sapcd2	72080	Suppressor	no | down	0	ENOG4110
Q9CR08	RPP29_MO	Pop4	Pop4	66161	Ribonuclea	no | no chan	0	ENOG4111
F8WJF7	F8WJF7_M	Traf7	Traf7	224619	TNF recept	no | no chan	0	ENOG410X
Q64726	ZA2G_MO	Azgp1	Azgp1	12007	Zinc-alpha	no | no chan	0	ENOG4111
Q64727	VINC_MO	Vcl	Vcl	22330	Vinculin	no | no chan	0	ENOG410X
Q923G2	RPAB3_MO	Polr2h	Polr2h	245841	DNA-direc	no | no chan	0	ENOG4111
Q923G3	Q923G3_M	Capzb	Capzb	12345	F-actin-cap	no | no chan	0	ENOG410X
A0A140LH	A0A140LH	Bub3	Bub3		Mitotic che	no | no chan	0	ENOG410X
Q99LQ1	MBIP1_MO	Mbip	Mbip	217588	MAP3K12-l	no | no chan	0	ENOG410Y
Q3UM78	Q3UM78_M	Ncoa4	Ncoa4	27057	Nuclear rec	no | no chan	0	ENOG410Y
Q99LQ7	PEDS1_MO	PEDS1	PEDS1	407243	Plasmanyle	no | no chan	0	ENOG410X
P38647	HSPA9_MO	Hspa9	Hspa9	15526	Stress-70 pr	no | no chan	0	COG0443(C
Q9CYJ6	Q9CYJ6_M	Sec61a2	Sec61a2	57743	SEC61 tran	no | no chan	0	COG0201(T
Q9CPR1	RWDD4_M	Rwdd4	Rwdd4	192174	RWD domc	no | no chan	0	ENOG410X
O35465	FKBP8_MO	Fkbp8	Fkbp8	14232	Peptidyl-pr	no | no chan	0	COG0545(C
Q9CPR5	RM15_MO	Mrpl15	Mrpl15	27395	Large ribos	no | no chan	0	COG0200(M

P30412	PPIC_MOU	Ppic	Ppic	19038	Peptidyl-pr	no	no chan	0	ENOG410Z
P30416	FKBP4_MO	Fkbp4	Fkbp4	14228	Peptidyl-pr	no	no chan	0	COG0545(c
P30415	NKTR_MO	Nktr	Nktr	18087	NK-tumor	no	no chan	0	COG0652(s
I7HFI3	I7HFI3_MO	Arhgap32	Arhgap32	330914	GTPase-act	no	no chan	0	ENOG410Y
Q9R1Q6	T176B_MO	Tmem176b	Tmem176b	65963	Transmemb	no	no chan	0	ENOG410Z
A0A1L1SRI	A0A1L1SRI	Cnn1	Cnn1		Calponin 1	no	up	0	COG5199(c
A6PW47	A6PW47_M	Med24	Med24		Mediator o	no	no chan	0	ENOG410Y
P31230	AIMP1_MO	Aimp1	Aimp1	13722	Aminoacyl	no	no chan	0	COG0073(I
A3KMD2	A3KMD2_I	Ranbp6	Ranbp6	240614	RAN bindi	no	no chan	0	ENOG410X
Q3TGM7	Q3TGM7_I	Hbs1l	Hbs1l	56422	HBS1-like p	no	no chan	0	COG5256(c
P50637	TSPO_MO	Tspo	Tspo	12257	Translocato	no	no chan	0	COG3476(t
Q60803	TRAF3_MO	Traf3	Traf3	22031	TNF recept	no	down	0	ENOG410Y
Q3U574	Q3U574_M	Timeless	Timeless	21853	Protein tim	no	no chan	0	ENOG410X
Q60770	STXB3_MO	Stxbp3	Stxbp3	20912	Syntaxin-bi	no	no chan	0	COG5158(p
Q60805	MERTK_M	Mertk	Mertk	17289	Tyrosine-pr	no	no chan	0	COG0515(T
Q8C0M9	ASGL1_MO	Asrgl1	Asrgl1	66514	Isoaspartyl	no	no chan	0	COG1446(c
Q9CX99	GRAP_MO	Grap	Grap	71520	GRB2-relat	no	down	0	ENOG410X
Q80W22	THNS2_MO	Thnsl2	Thnsl2	232078	Threonine s	no	no chan	0	COG0498(t
Q3UHE0	Q3UHE0_M	Ubr2	Ubr2	224826	E3 ubiquiti	no	no chan	0	ENOG410X
Q3UUG6	TBC24_MO	Tbc1d24	Tbc1d24	224617	TBC1 doma	no	no chan	0	ENOG410Z
Q7TNS5	Q7TNS5_M	B630019K0	B630019K0	102941	RIKEN cD	no	no chan	0	ENOG410X
O35685	NUDC_MO	Nudc	Nudc	18221	Nuclear mi	no	no chan	0	ENOG410X
E9PV41	E9PV41_M	Diaph1	Diaph1	13367	Diaphanou	no	no chan	0	ENOG410Y
O35683	NDUA1_M	Ndufa1	Ndufa1	54405	NADH deh	no	no chan	0	ENOG4112
O35682	MYADM_M	Myadm	Myadm	50918	Myeloid-ass	no	no chan	0	ENOG4111
Q7TNE3	SPAG7_MO	Spag7	Spag7	216873	Sperm-asso	no	no chan	0	ENOG4111
A0A2P9DU	A0A2P9DU	Serpina1d	Serpina1d	20703	Serpina1 D	no	no chan	0	COG4826(s
F6WIU1	F6WIU1_M	Kri1	Kri1	215194	Protein KR	no	no chan	0	ENOG410X
D6RDE8	D6RDE8_M	Desi1	Desi1		palmitoyl-p	no	no chan	0	ENOG4111
Q04899	CDK18_MO	Cdk18	Cdk18	18557	Cyclin-depe	no	no chan	0	ENOG410X
P18528	HVM57_MOUSE	Ig heavy chain V region 6.96			Ig heavy ch	no	up	0	ENOG410Y
Q8K2B3	SDHA_MO	Sdha	Sdha	66945	Succinate d	no	no chan	0	COG1053(S
Q3U313	Q3U313_M	Rbm39	Rbm39	170791	RRM doma	no	no chan	0	ENOG410X
Q8VD62	CK068_MO	Bles03	Bles03	107242	UPF0696 p	no	no chan	0	ENOG4112
P70218	M4K1_MO	Map4k1	Map4k1	26411	Mitogen-ac	no	no chan	0	ENOG410X
E5RKB6	E5RKB6_M	Stab1	Stab1	192187	FELE-1	no	down	0	ENOG410X
Q8VCM5	MUL1_MO	Mul1	Mul1	68350	Mitochond	no	no chan	0	ENOG410Y
Q9ER38	TOR3A_MO	Tor3a	Tor3a	30935	Torsin-3A	no	no chan	0	ENOG410X
Q8R404	MIC13_MO	Micos13	Micos13	224904	MICOS con	no	no chan	0	ENOG410Y
Q8R409	HEXI1_MO	Hexim1	Hexim1	192231	Protein HE	no	no chan	0	ENOG4111
Q8CBC4	CNST_MO	Cnst	Cnst	226744	Consortin	no	no chan	0	ENOG411(
O88653	LTOR3_MO	Lamtor3	Lamtor3	56692	Ragulator c	no	no chan	0	ENOG4111
Q6ZQ89	MARH6_M	Marchf6	Marchf6	223455	E3 ubiquiti	no	up	0	COG5183((

P52850	NDST2_M(	Ndst2	Ndst2	17423	Bifunctionc	no | no chan	0	ENOG410
Q8BPA8	DPCD_MC	Dpcd	Dpcd	226162	Protein DP	no | no chan	0	ENOG410
Q8VDI9	ALG9_MO	Alg9	Alg9	102580	Alpha-1,2-r	no | no chan	0	ENOG410
A0A0R4J0I	A0A0R4J0I	Lrp1	Lrp1	16971	Low density	no | no chan	0	ENOG410
Q9DB52	PBIR1_MO	Pabir1	Pabir1	68034	P2R1A-PPF	no | up	0	ENOG410
Q3U3N0	MIER2_M(	Mier2	Mier2	70427	Mesoderm i	no | no chan	0	ENOG410
A0A0R4J0I	A0A0R4J0I	Chtf18	Chtf18	214901	CTF18, chr	no | no chan	0	ENOG410
Q9JI90	RNF14_MC	Rnf14	Rnf14	56736	E3 ubiquiti	no | no chan	0	ENOG410
A3KGV1	ODFP2_M	Odf2	Odf2	18286	Outer dens	no | no chan	0	ENOG4111
A0A0A6YX	A0A0A6YX	Igsf3	Igsf3	78908	Immunoglo	no | no chan	0	ENOG410
A0A0R4J0I	A0A0R4J0I	Erf	Erf	13875	Ets2 repres	no | no chan	0	ENOG410
Q8VDI7	UBAC1_M	Ubac1	Ubac1	98766	Ubiquitin-c	no | no chan	0	ENOG410
A1A549	A1A549_M	Tcf7l1	Tcf7l1	21415	Transcripti	no | no chan	0	ENOG411
Q8R3G9	TSN8_MO	Tspan8	Tspan8	216350	Tetraspanir	no | up	0	ENOG4111
B8JJE0	B8JJE0_MC	Adgrv1	Adgrv1	110789	Adhesion G	no | no chan	0	ENOG410
Q9DAI2	IFT22_MO	Ift22	Ift22	67286	Intraflagell	no | no chan	0	ENOG4111
F6TLV3	F6TLV3_M	Fndc3a	Fndc3a		Fibronectin	no | no chan	0	ENOG410
Q6P3A9	ARL11_MC	Arl11	Arl11	219144	ADP-ribosy	no | no chan	0	COG1100(
Q6P3A8	ODBB_MC	Bckdhb	Bckdhb	12040	2-oxoisoval	no | no chan	0	COG0022(
A2AFM3	A2AFM3_M	Apex2	Apex2	77622	DNA-(apur	no | no chan	0	COG0708(
A2AFM9	A2AFM9_M	Pfkfb1	Pfkfb1	18639	6-phosphof	no | no chan	0	COG0406(
A0A8I4YXJ	A0A8I4YXJ	Bub1	Bub1	12235	BUB1, mito	no | no chan	0	ENOG410
B1AX39	ZCHC7_M(	Zcchc7	Zcchc7	319885	Zinc finger	no | no chan	0	ENOG411
Q9R1T4	SEPT6_MC	Septin6	Septin6	56526	Septin-6	no | no chan	0	COG5019(
Q8K1H7	T11L2_MO	Tcp11l2	Tcp11l2	216198	T-complex	no | no chan	0	ENOG410
B1AU25	B1AU25_M	Aifm1	Aifm1	26926	Apoptosis-i	no | no chan	0	COG0446(
A0A571BF	A0A571BF	Neb	Neb		Nebulin	no | up	0	ENOG410
F6UY95	F6UY95_M	Maml1	Maml1		Mastermin	no | no chan	0	ENOG4111
Q05144	RAC2_MO	Rac2	Rac2	19354	Ras-related	no | up	0	COG1100(
P53811	PIPNB_M(	Pitpnb	Pitpnb	56305	Phosphatid	no | no chan	0	COG5083(
P53810	PIPNA_M(	Pitpna	Pitpna	18738	Phosphatid	no | no chan	0	COG5083(
Q61649	Q61649_MOUSE	Alpha-globin mR		110257	Alpha-glob	no | no chan	0	COG1018(
Q6NXI6	RPRD2_M(	Rprd2	Rprd2	75137	Regulation	no | no chan	0	ENOG410
Q5BKS2	Q5BKS2_M	Ppm1b	Ppm1b	19043	protein-seri	no | no chan	0	COG0631(
P63085	MK01_MO	Mapk1	Mapk1	26413	Mitogen-ac	no | no chan	0	ENOG410
Q3UTZ3	TPC14_MC	Trappc14	Trappc14	231807	Trafficking	no | no chan	0	ENOG411
Q8CGB9	Q8CGB9_M	Ide	Ide	15925	Insulin deg	no | no chan	0	COG1025(
E9Q6B6	E9Q6B6_M	Mapk8ip3	Mapk8ip3	30957	Mitogen-ac	no | no chan	0	ENOG410
Q9CZG9	PDZ11_M(	Pdzd11	Pdzd11	72621	PDZ domai	no | no chan	0	ENOG411
Q9CQM9	GLRX3_M	Glrx3	Glrx3	30926	Glutaredox	no | no chan	0	COG0278(
Q09324	GCNT1_M	Gcnt1	Gcnt1	14537	Beta-1,3-ga	no | no chan	0	ENOG410
A0A1Y7VK	A0A1Y7VK	Nfic	Nfic		Nuclear fac	no | no chan	0	ENOG410
A4IF60	A4IF60_M(	Rhou	Rhou	69581	Rhou prote	no | no chan	0	COG1100(

Q8BFW4	TRI65_MO	Trim65	Trim65	338364	E3 ubiquiti	no|no chan	0	ENOG410
A0AAA9W	A0AAA9W	Ube2i	Ube2i		SUMO-con	no|no chan	0	COG5078(
O08576	RUN3A_M	Rundc3a	Rundc3a	51799	RUN doma	no|up	0	ENOG410
Q4VAC9	PKHG3_M	Plekhg3	Plekhg3	263406	Pleckstrin h	no|no chan	0	ENOG410
Q791N7	RPA12_MC	Polr1h	Polr1h	66136	DNA-direc	no|down	0	COG1594(
A0A494BA	A0A494BA	Cuedc2	Cuedc2	67116	CUE domai	no|no chan	0	ENOG4111
Q3TQD9	Q3TQD9_	Sardh	Sardh	192166	Sarcosine d	no|no chan	0	COG0404(
I6L9H1	I6L9H1_M	Dvl2	Dvl2	13543	Dishevelled	no|no chan	0	ENOG410
B1AUE5	PEX10_MC	Pex10	Pex10	668173	Peroxisome	no|no chan	0	COG5574(
G3X8W2	G3X8W2_	Retnla	Retnla	57262	Resistin like	no|down	0	ENOG410
D3Z1V4	D3Z1V4_M	Pebp1	Pebp1		Phosphatid	no|no chan	0	COG1881(
Q8BGP5	TM127_MC	Tmem127	Tmem127	69470	Transmemb	no|no chan	0	ENOG410
Q8BGP6	S2540_MO	Slc25a40	Slc25a40	319653	Mitochond	no|no chan	0	ENOG410
J3JS23	J3JS23_MO	Zfp62	Zfp62		Zinc finger	no|no chan	0	COG5048(
P08551	NFL_MOU	Nefl	Nefl	18039	Neurofilam	no|no chan	0	ENOG410
Q60787	LCP2_MO	Lcp2	Lcp2	16822	Lymphocyt	no|up	0	ENOG4111
Q8BJL0	SMAL1_M	Smarcal1	Smarcal1	54380	SWI/SNF-r	no|no chan	0	COG0553(
Q80SY5	PR38B_MC	Prpf38b	Prpf38b	66921	Pre-mRNA	no|no chan	0	ENOG410
Q80SY6	ADAL_MC	Mapda	Mapda	75894	N6-Methyl-	no|no chan	0	COG1816(
Q80SY3	VA0D2_M	Atp6v0d2	Atp6v0d2	242341	V-type prot	no|no chan	0	COG1527(
Q62192	CD180_MC	Cd180	Cd180	17079	CD180 anti	no|up	0	COG4886(
O54949	NLK_MOU	Nlk	Nlk	18099	Serine/thre	no|no chan	0	ENOG410
B2RUD1	B2RUD1_	Tmem165	Tmem165	21982	GDT1 fami	no|no chan	0	COG2119(
A0A075B5	A0A075B5	Igkv4-80	Igkv4-80	545848	Immunoglo	no|up	0	ENOG4111
A0A075B5	A0A075B5	Igkv4-90	Igkv4-90	434034	Immunoglo	no|up	0	ENOG4111
Q3URS9	MITOK_M	Ccdc51	Ccdc51	66658	Mitochond	no|no chan	0	ENOG4111
Q8C8N2	SCAI_MOU	Scai	Scai	320271	Protein SC	no|no chan	0	ENOG410
Q8K327	CHAP1_M	Champ1	Champ1	101994	Chromoson	no|no chan	0	ENOG410
Q62513	ZN260_MC	Znf260	Znf260	26466	Zinc finger	no|down	0	COG5048(
Q3TZ63	Q3TZ63_M	Tbc1d13	Tbc1d13	70296	Rab-GAP T	no|no chan	0	COG5210(
A0A1Y7VL	A0A1Y7VL	Lpin1	Lpin1	14245	phosphatid	no|no chan	0	COG5083(
Q62048	PEA15_MC	Pea15	Pea15	18611	Astrocytic p	no|no chan	0	ENOG410
P45591	COF2_MO	Cfl2	Cfl2	12632	Cofilin-2	no|no chan	0	ENOG4112
A0A1Y7VL	A0A1Y7VL	Togaram1	Togaram1		TOG array	no|no chan	0	ENOG410
A0A1I7Q4	A0A1I7Q4	Gatad2a	Gatad2a		GATA zinc	no|no chan	0	ENOG410
P28843	DPP4_MO	Dpp4	Dpp4	13482	Dipeptidyl	no|down	0	COG1506(
P32067	LA_MOUS	Ssb	Ssb	20823	Lupus La p	no|no chan	0	COG5193(
Q6PCM2	INT6_MOU	Ints6	Ints6	18130	Integrator	no|no chan	0	ENOG410
Q6PCM1	KDM3A_M	Kdm3a	Kdm3a	104263	Lysine-spec	no|no chan	0	ENOG410
Q3TNA0	Q3TNA0_	Cyp4b1	Cyp4b1	13120	Cytochrom	no|down	0	COG2124(
A0A0R4J15	A0A0R4J15	Mbd1	Mbd1	17190	Methyl-Cp	no|no chan	0	ENOG4111
Q9JHU4	DYHC1_M	Dync1h1	Dync1h1	13424	Cytoplasmi	no|no chan	0	COG5245(
Q05CH9	Q05CH9_	Rnf123	Rnf123	84585	RING-type	no|no chan	0	ENOG410

A0A0R4J15	A0A0R4J15	Osbpl8	Osbpl8	237542	Oxysterol-b	no | no chan	0	ENOG410
P26443	DHE3_MO	Glud1	Glud1	14661	Glutamate	no | no chan	0	COG0334(c
E9PVQ3	E9PVQ3_M	Spats2l	Spats2l	67198	Spermatoge	no | up	0	ENOG410
Q5SXA7	Q5SXA7_M	Pank3	Pank3	211347	Pantothena	no | no chan	0	COG5146(
Q3UHX9	SPOUT_M	Spout1	Spout1	227695	Putative m	no | no chan	0	COG2106(c
Q3UK61	Q3UK61_M	Oxct1	Oxct1	67041	Succinyl-C	no | no chan	0	COG2057(
Q9JMG1	EDF1_MO	Edf1	Edf1	59022	Endothelial	no | no chan	0	COG1813(
Q3UK65	Q3UK65_M	Cops2	Cops2	12848	COP9 signc	no | up	0	COG5159(c
A4FUU4	A4FUU4_M	Retsat	Retsat	67442	All-trans-re	no | no chan	0	COG1233(t
A0A0J9YU	A0A0J9YU	Tmem248	Tmem248		Transmemb	no | no chan	0	ENOG411
Q3V1Q8	Q3V1Q8_M	Foxg1	Foxg1	15228	Forkhead b	no | no chan	0	COG5025(f
Q14AX6	CDK12_M	Cdk12	Cdk12	69131	Cyclin-depe	no | no chan	0	ENOG410
Q14AX9	Q14AX9_M	Mrc2	Mrc2	17534	Mannose re	no | no chan	0	ENOG410
Q3V1Q5	Q3V1Q5_M	Zbtb2	Zbtb2	381990	Zinc finger	no | no chan	0	COG5048(
Q922B1	MACD1_M	Macrod1	Macrod1	107227	ADP-ribose	no | no chan	0	COG2110(c
G3UYF9	G3UYF9_M	Pfdn6	Pfdn6		Prefoldin su	no | no chan	0	COG1382(
J3QPX1	J3QPX1_M	Rhox5	Rhox5		Reproducti	no | no chan	0	
P11440	CDK1_MO	Cdk1	Cdk1	12534	Cyclin-depe	no | no chan	0	ENOG410
B2RR84	B2RR84_M	Pde3a	Pde3a	54611	Phosphodie	no | up	0	ENOG410
B2RR82	B2RR82_M	Itsn2	Itsn2	20403	Intersectin	no | no chan	0	COG5422(
B2RR83	YTDC2_M	Ythdc2	Ythdc2	240255	3'-5' RNA h	no | no chan	0	COG1643(c
P51683	CCR2_MO	Ccr2	Ccr2	12772	C-C chemol	no | up	0	ENOG410
Q3UFB2	BCD1_MO	Znhit6	Znhit6	229937	Box C/D sr	no | no chan	0	ENOG4111
A0A140T8	A0A140T8	Igkv12-44	Igkv12-44	545851	Immunoglo	no | up	0	ENOG4111
A0A140T8	A0A140T8	Igkv8-19	Igkv8-19		Immunoglo	no | no chan	0	ENOG410
A0A140T8	A0A140T8	Igkv6-15	Igkv6-15	108022	Immunoglo	no | no chan	0	ENOG410
B2RQR1	B2RQR1_M	Notch4	Notch4	18132	Notch gene	no | no chan	0	COG0666(
Q3TZG8	Q3TZG8_M	Nol4l	Nol4l	329540	Nucleolar p	no | no chan	0	ENOG410
Q9D312	K1C20_MC	Krt20	Krt20	66809	Keratin, typ	no | up	0	ENOG410
B5A5B2	B5A5B2_M	Tpsb2	Tpsb2	17229	Tryptase be	no | down	0	COG5640(c
Q3TBV3	Q3TBV3_M	Fam8a1	Fam8a1	97863	RDD doma	no | no chan	0	ENOG4111
Q3TBV5	Q3TBV5_M	Il1rn	Il1rn	16181	Interleukin-	no | up	0	ENOG4111
Q91WB7	UBTD1_M	Ubtd1	Ubtd1	226122	Ubiquitin d	no | no chan	0	ENOG410
E9PZB7	E9PZB7_M	Znrf1	Znrf1	170737	E3 ubiquiti	no | no chan	0	ENOG4111
B2RPR7	B2RPR7_M	Itga2b	Itga2b	16399	Integrin alp	no | no chan	0	ENOG410
Q9CQ54	NDUC2_M	Ndufc2	Ndufc2	68197	NADH deh	no | no chan	0	ENOG410
Q91WB5	Q91WB5_M	Scgb1b27	Scgb1b27	11354	ABPA27	no | no chan	0	ENOG4111
O35855	BCAT2_M	Bcat2	Bcat2	12036	Branched-c	no | no chan	0	COG0115(
O35857	TIM44_MC	Timm44	Timm44	21856	Mitochond	no | no chan	0	ENOG410
A0A2I3BRI	A0A2I3BRI	Pkd1	Pkd1		Polycystin 1	no | up	0	ENOG410
G5E833	G5E833_M	Osbpl5	Osbpl5	79196	Oxysterol-b	no | no chan	0	ENOG410
B1AZA5	TM245_MC	Tmem245	Tmem245	242474	Transmemb	no | no chan	0	ENOG410
A0A286YC	A0A286YC	Ssr1	Ssr1		Translocon	no | no chan	0	ENOG410

Q9D6Y7	MSRA_MO	Msra	Msra	110265	Mitochondı	no | no chan	0	COG0225(I
B2RXX7	B2RXX7_M	Nhs	Nhs	195727	Nhs proteir	yes | down	1	ENOG4111
Q8BFS9	ASND1_M(	Asnsd1	Asnsd1	70396; 1105	Asparagine	no | no chan	0	COG0367(c
Q6NXM5	Q6NXM5_	Brip1os	Brip1os	74038	4632419I22l	no | up	0	ENOG4111
D3Z0Q4	D3Z0Q4_M	Mthfsd	Mthfsd		Methenylte	no | no chan	0	COG0212(5
Q8BFS6	CPPED_M(	Cpped1	Cpped1	223978	Serine/thre	no | no chan	0	ENOG410Z
Q9D6Y9	GLGB_MC	Gbe1	Gbe1	74185	1,4-alpha-g	no | no chan	0	COG0296(C
Q3UQS8	RBM20_M(	Rbm20	Rbm20	73713	RNA-bindi	no | down	0	ENOG410Z
Q3UI43	BABA1_M(	Babam1	Babam1	68251	BRISC and	no | no chan	0	ENOG4111
A2A7T8	A2A7T8_M	Zmym1	Zmym1		Zinc finger,	no | no chan	0	ENOG410X
Q3UQS3	Q3UQS3_M	Pkn3	Pkn3	263803	protein kin	no | no chan	0	ENOG410X
Q8BH35	CO8B_MO	C8b	C8b	110382	Complemer	no | down	0	ENOG410Y
A6MDC5	A6MDC5_M	Cd276	Cd276	102657	CD276 anti	no | no chan	0	ENOG410Z
Q6NSQ5	Q6NSQ5_M	Sp140	Sp140	434484	Sp140 nucl	no | up	0	ENOG4111
Q6NSQ7	LTV1_MOI	Ltv1	Ltv1	353258	Protein LT	no | no chan	0	ENOG410X
A2AI05	NDOR1_M	Ndor1	Ndor1	78797	NADPH-de	no | no chan	0	COG0369(c
A0A3B2WE	A0A3B2WE	Sh3bgr	Sh3bgr		SH3-bindin	no | up	0	ENOG4111
A2AI08	TPRN_MO	Tprn	Tprn	97031	Taperin	no | no chan	0	ENOG410Z
Q6NSQ9	G6PC3_M(	G6pc3	G6pc3	68401	Glucose-6-p	no | no chan	0	ENOG411C
Q3U102	Q3U102_M	Esam	Esam	69524	Ig-like dom	no | down	0	ENOG4111
Q91YN0	FERY3_M(	Ferry3	Ferry3	28040	FERRY enc	no | no chan	0	ENOG410X
Q91YN1	F118A_MO	Fam118a	Fam118a	73225	Protein FA	no | no chan	0	ENOG410Y
Q91YR7	PRP6_MOI	Prpf6	Prpf6	68879	Pre-mRNA	no | no chan	0	ENOG410X
Q3TDX2	Q3TDX2_M	Vps4a	Vps4a	116733	vesicle-fusi	no | no chan	0	ENOG410X
Q91YN9	BAG2_MO	Bag2	Bag2	213539	BAG family	no | no chan	0	ENOG410X
A0A498WC	A0A498WC	Ssh3	Ssh3		protein-seri	no | no chan	0	COG2453(I
Q3TDX8	NB5R4_M(	Cyb5r4	Cyb5r4	266690	Cytochrom	no | up	0	COG5274(I
E9PZQ1	E9PZQ1_M	Ddx60	Ddx60		DExD/H b	no | no chan	0	COG4581(z
Q8K0E9	Q8K0E9_M	Dhdh	Dhdh	71755	Trans-1,2-d	no | no chan	0	COG0673(c
Q3T9X0	GTR9_MO	Slc2a9	Slc2a9	117591	Solute carri	no | no chan	0	COG0477(M
Q6KAQ0	Q6KAQ0_M	Nsmce3	Nsmce3	66647	MFLJ00211	no | no chan	0	ENOG4111
Q8K0E1	KCD15_M(	Kctd15	Kctd15	233107	BTB/POZ	no | no chan	0	ENOG410Z
A0A087WR	A0A087WR	Dock10	Dock10		Dedicator c	no | no chan	0	ENOG410X
A2A5J2	A2A5J2_M(	Zswim1	Zswim1		Zinc finger	no | down	0	ENOG4111
P11862	GAS2_MOI	Gas2	Gas2	14453	Growth arr	no | down	0	ENOG4111
A0A1L1SVI	A0A1L1SVI	Pafah1b2	Pafah1b2		1-alkyl-2-ac	no | no chan	0	ENOG410X
D3YUJ3	CCYL1_M(	Ccnyl1	Ccnyl1	227210	Cyclin-Y-lil	no | no chan	0	ENOG410X
A0A3B0IN	A0A3B0IN	Adib	Adib	12262	Adiponecti	no | up	0	ENOG4111
Q8BJU9	RF1ML_M(	Mtrf1l	Mtrf1l	108853	Peptide cha	no | no chan	0	COG0216(I
A0A1D5RM	A0A1D5RM	Mast3	Mast3	546071	non-specifi	no | no chan	0	ENOG410X
P29416	HEXA_MC	Hexa	Hexa	15211	Beta-hexos	no | no chan	0	COG3525(C
A2AVJ7	A2AVJ7_M	Rrbp1	Rrbp1	81910	Ribosome b	no | no chan	0	ENOG410X
O55057	PDE6D_M	Pde6d	Pde6d	18582	Retinal rod	no | no chan	0	ENOG411C

O55055	TRDMT_M	Trdmt1	Trdmt1	13434	tRNA (cyto	no	no chan	0	COG0270(s
A0A1B0GS	A0A1B0GS	Aldh16a1	Aldh16a1	69748	Aldehyde d	no	no chan	0	COG1012(T
Q91YU6	LZTS2_MC	Lzts2	Lzts2	226154	Leucine zip	no	no chan	0	ENOG4111
H7BWZ9	H7BWZ9_M	Dop1a	Dop1a	320615	DOP1 leuci	no	no chan	0	COG5221(r
Q3TQ21	Q3TQ21_M	Pum1	Pum1	80912	Pumilio ho	no	no chan	0	COG5099(p
P56564	EAA1_MO	Slc1a3	Slc1a3	20512	Excitatory	no	down	0	COG1301(I
P56565	S10A1_MO	S100a1	S100a1	20193	Protein S10	no	no chan	0	ENOG4111
A0A1W2P6	A0A1W2P6	Icosl	Icosl		Icos ligand	no	no chan	0	ENOG410Z
F6TN13	F6TN13_M	Rnpc3	Rnpc3		RNA-bindi	no	no chan	0	ENOG410X
Q9CWV1	MCM8_MC	Mcm8	Mcm8	66634	DNA helic	no	no chan	0	COG1241(c
B0QZX1	B0QZX1_M	Il2rg	Il2rg		Interleukin	no	no chan	0	ENOG4111
G3UXA6	G3UXA6_M	Ptbp3	Ptbp3		Polypyrimi	no	no chan	0	ENOG410X
Q91VL8	TE2IP_MO	Terf2ip	Terf2ip	57321	Telomeric r	no	no chan	0	ENOG4111
A0A087WS	A0A087WS	Pigx	Pigx		Phosphatid	no	no chan	0	ENOG410Y
A0A087WS	A0A087WS	Fn1	Fn1	14268	Fibronectin	no	no chan	0	ENOG410Y
D3YY09	D3YY09_M	Drap1	Drap1	66556	DR1 associ	no	no chan	0	COG5247(p
P24369	PPIB_MOU	Ppib	Ppib	19035	Peptidyl-pr	no	no chan	0	ENOG410Z
Q3UJF5	Q3UJF5_M	Top3b	Top3b	21976	DNA topoi	no	no chan	0	COG0550(I
D3YV77	D3YV77_M	Mvk	Mvk		mevalonate	no	no chan	0	COG1577(r
A0A1D5RL	A0A1D5RL	Slc38a7	Slc38a7		Sodium-cou	no	no chan	0	COG0814(I
A0A286YD	A0A286YD	Grk6	Grk6	26385	G protein-c	no	no chan	0	ENOG410Y
D6RE20	D6RE20_M	Selenoi	Selenoi	28042	Selenoprote	no	up	0	COG5050(p
A6X935	ITIH4_MO	Itih4	Itih4	16427	Inter alpha	no	up	0	COG2304(I
Q56A04	EME2_MO	Eme2	Eme2	193838	Probable cr	no	no chan	0	ENOG4111
Q8BUL6	PKHA1_M	Plekha1	Plekha1	101476	Pleckstrin h	no	no chan	0	ENOG410Z
D3Z632	D3Z632_M	Aktip	Aktip	14339	AKT-intera	no	no chan	0	ENOG410Z
A0A0U1RP	A0A0U1RP	Cib1	Cib1	23991	Calcium an	no	no chan	0	COG5126(r
Q3U0U3	Q3U0U3_M	Mad2l1bp	Mad2l1bp	66591	MAD2L1-b	no	no chan	0	ENOG4111
Q8R216	SIR4_MOU	Sirt4	Sirt4	75387	NAD-deper	no	no chan	0	COG0846(C
P56387	DYLT3_M	Dynlt3	Dynlt3	67117	Dynein ligh	no	no chan	0	ENOG4111
P56382	ATP5E_M	Atp5f1e	Atp5f1e	67126	ATP syntha	no	no chan	0	ENOG410Y
P51863	VA0D1_M	Atp6v0d1	Atp6v0d1	11972	V-type prot	no	no chan	0	COG1527(A
P56380	AP4A_MO	Nudt2	Nudt2	66401	Bis(5'-nucle	no	no chan	0	COG0494(C
A0A1W2P7	A0A1W2P7	Cdc34	Cdc34		Cell divisio	no	no chan	0	COG5078(T
A2NW55	A2NW55_MOUSE	E8 variable heavy chain (F			E8 variable	no	no chan	0	ENOG4111
P56389	CDD_MOU	Cda	Cda	72269	Cytidine de	no	up	0	COG0295(c
Q9D1F3	EOLA1_M	Eola1	Eola1	68618	Protein EO	no	no chan	0	ENOG4111
Q8BK30	NDUV3_M	Ndufv3	Ndufv3	78330	NADH deh	no	up	0	ENOG410Y
P21460	CYTC_MO	Cst3	Cst3	13010	Cystatin-C	no	up	0	ENOG4112
E9Q5G3	KIF23_MO	Kif23	Kif23	71819	Kinesin-lik	no	no chan	0	COG5059(c
B7ZWN9	B7ZWN9_M	Senp6	Senp6	215351	Senp6 prote	no	no chan	0	COG5160(c
P10518	HEM2_MC	Alad	Alad	17025	Delta-amin	no	no chan	0	COG0113(c
B7ZWN1	B7ZWN1_M	Kif20b	Kif20b	240641	Kif20b prot	no	no chan	0	COG5059(c

B1ARR4	B1ARR4_M	Car6	Car6		Carbonic a	no|up	0	COG3338(I
A0A0R4J09	A0A0R4J09	2210016L21	2210016L21	72357	Protein CU	no|no chan	0	ENOG410Y
A0A0R4J09	A0A0R4J09	Zfp326	Zfp326	54367	DBIRD cor	no|no chan	0	ENOG4111
Q3UVL4	VPS51_MO	Vps51	Vps51	68505	Vacuolar p	no|no chan	0	ENOG410X
A0A0R4J09	A0A0R4J09	Tgfbr3	Tgfbr3	21814	Transformi	no|no chan	0	ENOG410X
Q8VCV1	AB17C_MC	Abhd17c	Abhd17c	70178	Alpha/beta	no|no chan	0	COG1073(M
A0A0R4J09	A0A0R4J09	Pole3	Pole3	59001	DNA polym	no|no chan	0	ENOG4111
F2Z470	F2Z470_M	Hmgcr	Hmgcr		3-hydroxy-3	no|no chan	0	COG1257()
Q9JM76	ARPC3_MC	Arpc3	Arpc3	56378	Actin-relate	no|no chan	0	ENOG4111
A0A0R4J09	A0A0R4J09	Manba	Manba	110173	Beta-mann	no|no chan	0	COG3250(p
Q8R1J3	ZCHC9_M	Zcchc9	Zcchc9	69085	Zinc finger	no|no chan	0	COG5082(z
Q99KJ0	UBX2A_M	Ubxn2a	Ubxn2a	217379	UBX doma	no|no chan	0	ENOG410Y
Q3UPP8	CEP63_MC	Cep63	Cep63	28135	Centrosom	no|no chan	0	ENOG4111
E1U8D0	MTCL2_M	Mtcl2	Mtcl2	320706	Microtubul	no|no chan	0	ENOG410X
Q8R373	CLMP_MO	Clmp	Clmp	71566	CXADR-lik	no|down	0	ENOG4111
Q80U72	SCRIB_MC	Scrib	Scrib	105782	Protein scri	no|no chan	0	COG4886(p
A2AWL7	MGAP_MC	Mga	Mga	29808	MAX gene-	no|no chan	0	ENOG410X
H3BKM2	H3BKM2_M	Zc3hc1	Zc3hc1		Zinc finger,	no|no chan	0	ENOG410Z
O88888	APBA3_MC	Apba3	Apba3	57267	Amyloid-be	yes|up	1	ENOG410X
A0A494BA	A0A494BA	Nme4	Nme4		Nucleoside	no|no chan	0	COG0105(N
G5E8V9	ARFP1_MC	Arfip1	Arfip1	99889	Arfaptin-1	no|no chan	0	ENOG410Y
G5E8V8	G5E8V8_M	Phax	Phax	56698	Phosphoryl	no|no chan	0	ENOG411C
F8VPR1	F8VPR1_M	Lmod1	Lmod1	93689	Leiomodin	no|no chan	0	ENOG410Y
G5E8V4	G5E8V4_M	Nadsyn1	Nadsyn1	78914	Glutamine-	no|up	0	COG0388(U
F8VPR5	F8VPR5_M	Crebbp	Crebbp	12914	histone ace	no|no chan	0	COG5076(I
Q3TTF2	Q3TTF2_M	Ubxn8	Ubxn8	108159	UBX doma	no|no chan	0	ENOG410X
P58389	PTPA_MO	Ptpa	Ptpa	110854	Serine/thre	no|no chan	0	COG5057(I
Q9DBR7	MYPT1_M	Ppp1r12a	Ppp1r12a	17931	Protein pho	no|no chan	0	COG0666(S
Q9DBR1	XRN2_MO	Xrn2	Xrn2	24128	5'-3' exorib	no|no chan	0	COG5049(S
A0A0R4J0V	A0A0R4J0V	Jmy	Jmy	57748	Junction-m	no|no chan	0	ENOG4111
A0A0R4J0V	A0A0R4J0V	Polr2a	Polr2a	20020	DNA-direc	no|no chan	0	COG0086(p
Q6PFY1	FCSD1_MC	Fchsd1	Fchsd1	319262	F-BAR and	yes|up	1	ENOG410X
E9Q9A9	OAS2_MO	Oas2	Oas2	246728	2'-5'-oligoa	no|no chan	0	COG5272(I
A0A0R4J0V	A0A0R4J0V	Ints10	Ints10	70885	Integrator c	no|no chan	0	ENOG410X
E0CX98	E0CX98_M	Tmbim6	Tmbim6		Bax inhibit	no|no chan	0	ENOG4111
Q3UNZ8	QORL2_M	Cryzl2	Cryzl2	226527	Quinone ox	no|down	0	COG0604(M
Q9D8Z2	TRIA1_MC	Triap1	Triap1	69076	TP53-regul	no|no chan	0	ENOG4111
A0ABA7IX	A0ABA7IXF2_MOUSE	DNA-damage inducibl	Ddi2			no|no chan	0	ENOG410X
Q3UNZ1	Q3UNZ1_M	Ghdc	Ghdc	80860	GH3 doma	no|no chan	0	ENOG410X
C9VZF2	C9VZF2_M	Pfkfb4	Pfkfb4	270198	6-phosphof	no|no chan	0	COG0406(T
A0A087WQ	A0A087WQ	Morf4l1	Morf4l1		Mortality fc	no|no chan	0	ENOG410X
P97946	VEGFD_M	Vegfd	Vegfd	14205	Vascular er	no|no chan	0	ENOG4111
P97760	RPB3_MO	Polr2c	Polr2c	20021	DNA-direc	no|no chan	0	COG0202(I

P97765	WBP2_MO	Wbp2	Wbp2	22378	WW domai	no | no chan	0	ENOG4111
Q9Z103	ADNP_MC	Adnp	Adnp	11538	Activity-dep	no | no chan	0	ENOG410X
Q8R0G9	NU133_MC	Nup133	Nup133	234865	Nuclear po	no | no chan	0	ENOG410X
Q8C2G3	Q8C2G3_M	Ttc4	Ttc4	72354	Cns1/TTC4	no | no chan	0	COG0457(F
Q8VEH8	ERLEC_MO	Erlec1	Erlec1	66753	Endoplasm	no | no chan	0	ENOG410X
Q8VEH6	ZNG1_MO	Zng1	Zng1	226043	Zinc-regula	no | no chan	0	COG0523(c
Q8VEH5	EPMIP_MO	Epm2aip1	Epm2aip1	77781	EPM2A-int	no | no chan	0	ENOG410X
Q76N33	STALP_MO	Stambpl1	Stambpl1	76630	AMSH-like	no | no chan	0	COG1310(C
Q8R2Z5	VWA1_MO	Vwa1	Vwa1	246228	von Willebr	no | no chan	0	ENOG410X
Q3TVT6	Q3TVT6_M	Pcyox1	Pcyox1	66881	Prenylcyste	no | no chan	0	ENOG410Y
Q8R0L9	TADA3_MO	Tada3	Tada3	101206	Transcripti	no | no chan	0	ENOG410X
Q3TC33	CC127_MO	Ccdc127	Ccdc127	67433	Coiled-coil	no | no chan	0	ENOG410Z
P36371	TAP2_MOU	Tap2	Tap2	21355	Antigen pep	no | up	0	COG1132(I
P11276	FINC_MOU	Fn1	Fn1	14268	Fibronectin	no | no chan	0	ENOG410Y
A2A8Z1	OSBL9_MO	Osbpl9	Osbpl9	100273	Oxysterol-b	no | no chan	0	ENOG410X
Q8BTG6	SPRNG_MO	Spring1	Spring1	76792	SREBP regu	no | no chan	0	ENOG4110
Q9CQV1	TIM16_MO	Pam16	Pam16	66449	Mitochondr	no | no chan	0	ENOG4112
Q9CQV4	RETR3_MO	Retreg3	Retreg3	67998	Reticulopho	no | no chan	0	ENOG4110
Q9CQV5	RT24_MOU	Mrps24	Mrps24	64660	Small ribos	no | no chan	0	ENOG4111
H7BX83	H7BX83_M	Ggnbp2	Ggnbp2		Gametogen	no | no chan	0	ENOG410X
Q9D2R6	COA3_MO	Coa3	Coa3	52469	Cytochrom	no | no chan	0	ENOG4111
Q9Z108	STAU1_MO	Stau1	Stau1	20853	Double-str	no | no chan	0	ENOG410X
Q9D2R0	AACS_MO	Aacs	Aacs	78894	Acetoacetyl	no | no chan	0	COG0365(C
V9GZK9	V9GZK9_M	Slp	Slp	625018	Sex-limited	no | no chan	0	ENOG410X
A4QPC5	A4QPC5_M	Cma1	Cma1	17228	Chymase 1,	no | down	0	COG5640(t
Q05DA2	Q05DA2_M	Sec62	Sec62	69276	Translocati	no | no chan	0	COG5232(t
Q3UG98	NAT9_MO	Nat9	Nat9	66176	Alpha/beta	no | no chan	0	ENOG4111
Q9D786	HAUS5_MO	Haus5	Haus5	71909	HAUS augm	no | no chan	0	ENOG410Z
A4QPC9	A4QPC9_M	Fancg	Fancg	60534	Fancg prote	no | down	0	ENOG4111
H7BX88	H7BX88_M	Crat	Crat		Carnitine a	no | no chan	0	ENOG410X
Q5XJE5	LEO1_MO	Leo1	Leo1	235497	RNA polym	no | no chan	0	ENOG410X
A0A087WP	A0A087WP	Resf1	Resf1		Retroeleme	no | no chan	0	ENOG4111
E9PUV9	E9PUV9_M	Card10	Card10	105844	Caspase rec	no | no chan	0	COG0194(F
P14576	SRP54_MO	Srp54	Srp54	24067; 6651	Signal reco	no | no chan	0	COG0541(I
P59470	RPC2_MOU	Polr3b	Polr3b	70428	DNA-direc	no | no chan	0	COG0085(s
Q8C2Q3	RBM14_MO	Rbm14	Rbm14	56275	RNA-bindi	no | no chan	0	ENOG4111
Q3TLR3	Q3TLR3_M	Trim32	Trim32	69807	RING-type	no | no chan	0	ENOG410X
A0A2S0DF	A0A2S0DFG6_MOUSE	Anti-HIV fusion pepti			Anti-HIV fu	no | no chan	0	ENOG4111
Q496R6	Q496R6_M	Aven	Aven	74268	Aven protei	no | no chan	0	ENOG4111
Q8QZZ7	TPRKB_MO	Tprkb	Tprkb	69786	EKC/KEO	no | no chan	0	ENOG4111
A0A075B5S	A0A075B5S	Ighv7-1	Ighv7-1		Immunoglo	no | no chan	0	ENOG410Y
A0A1B0GQ	A0A1B0GQ	Saa2	Saa2		Serum amyl	no | up	0	ENOG410Y
Q8WTY4	CPIN1_MO	Ciapin1	Ciapin1	109006	Anamorsin	no | no chan	0	COG5636(C

Q99LW6	YAF2_MOU	Yaf2	Yaf2	67057	YY1-associ	no|up	0	ENOG4111
Q3TE95	Q3TE95_M	Rcn2	Rcn2	26611	EF-hand dc	no|no chan	0	ENOG410X
A0A140LH	A0A140LH	Spcs2	Spcs2		Signal pept	no|no chan	0	ENOG4111
F7BGR7	F7BGR7_M	Gm21992	Gm21992	102902673	Predicted g	no|no chan	0	ENOG4110
A0A140LH	A0A140LH	Med25	Med25	75613	Mediator of	no|no chan	0	ENOG410Z
Q3TH44	Q3TH44_M	Nudt15	Nudt15	214254	Nudix hydr	no|no chan	0	COG1051(N
P50431	GLYC_MO	Shmt1	Shmt1	20425	Serine hydr	no|no chan	0	COG0112(S
Q64701	RBL1_MOU	Rbl1	Rbl1	19650	Retinoblast	no|no chan	0	ENOG410X
D3YYS6	D3YYS6_M	Mgll	Mgll		Monoglycer	no|down	0	COG2267(c
Q64707	U2AFL_MO	Zrsr2-ps1	Zrsr2-ps1	22183	U2 small nu	no|down	0	ENOG410Z
Q923A2	SPDLY_MO	Spdl1	Spdl1	70385	Protein Spi	no|no chan	0	ENOG4110
O35448	PPT2_MOU	Ppt2	Ppt2	54397	Lysosomal t	no|no chan	0	COG1075(C
O35443	O35443_M	NG1	NG1	106512	Uncharacte	no|no chan	0	ENOG4111
Q2HZ94	Q2HZ94_M	Mrc1	Mrc1	17533	Macrophag	no|down	0	ENOG410X
Q9QYS9	QKI_MOU	Qki	Qki	19317	KH domain	no|no chan	0	COG5176(S
O35445	RNF5_MO	Rnf5	Rnf5	54197	E3 ubiquiti	no|up	0	ENOG4111
Q3V3R4	ITA1_MOU	Itga1	Itga1	109700	Integrin alp	no|down	0	ENOG410Y
Q3V3R1	C1TM_MO	Mthfd1l	Mthfd1l	270685	Monofuncti	no|no chan	0	COG0190(C
Q8BJ05	ZC3HE_MO	Zc3h14	Zc3h14	75553	Zinc finger	no|no chan	0	ENOG410Y
Z4YLT1	Z4YLT1_M	Inip	Inip		SOSS comp	no|no chan	0	ENOG4111
Q9D270	ZDH21_MO	Zdhhc21	Zdhhc21	68268	Palmitoyltr	no|no chan	0	COG5273(S
Q8BJ03	COX15_MO	Cox15	Cox15	226139	Heme A syn	yes|up	1	COG1612(C
P84104	SRSF3_MO	Srsf3	Srsf3	20383	Serine/argi	no|no chan	0	ENOG4111
Q3TJ69	Q3TJ69_M	Serpinb9b	Serpinb9b	20706	Serpin dom	no|up	0	COG4826(C
A3KMF4	A3KMF4_M	Aff1	Aff1	17355	AF4/FMR2	yes|down	1	ENOG410X
F7BE84	F7BE84_M	Ankrd50	Ankrd50		Ankyrin rep	no|no chan	0	COG0666(S
Q3TM92	Q3TM92_M	Anapc4	Anapc4	52206	Anaphase-p	no|no chan	0	ENOG410X
Q8BVU5	NUDT9_M	Nudt9	Nudt9	74167	ADP-ribose	no|up	0	ENOG410X
Q9D8H7	OMA1_MO	Oma1	Oma1	67013	Metalloend	no|no chan	0	COG0501(F
P60882	MEGF8_M	Megf8	Megf8	269878	Multiple ep	no|no chan	0	ENOG410Y
Q8K3M5	CABL2_MO	Cables2	Cables2	252966	CDK5 and	yes|down	1	ENOG4110
A0A0G2JF	A0A0G2JF	Fubp1	Fubp1		Far upstrea	no|no chan	0	ENOG410X
E9PV68	E9PV68_M	Nabp2	Nabp2		Nucleic aci	no|up	0	ENOG4111
E9PV69	E9PV69_M	Cacul1	Cacul1	78832	CDK2 asso	no|no chan	0	COG5647(r
A0A1W2P8	A0A1W2P8	Mcm9	Mcm9		DNA helic	no|no chan	0	COG1241(t
Q06EZ3	Q06EZ3_M	Soat1	Soat1	20652	O-acyltrans	no|no chan	0	COG5056(A
Q9R059	FHL3_MOU	Fhl3	Fhl3	14201	Four and a	no|no chan	0	ENOG410X
F8WJD6	F8WJD6_M	Mex3b	Mex3b	108797	Mex3 RNA	no|down	0	ENOG410Y
F6RJ39	F6RJ39_M	Acin1	Acin1		Apoptotic c	no|no chan	0	ENOG4111
Q68FG3	SPT2_MOU	Spty2d1	Spty2d1	101685	Protein SPT	no|no chan	0	ENOG410Y
E9Q7F2	RN169_MO	Rnf169	Rnf169	108937	E3 ubiquiti	no|no chan	0	ENOG4111
Q8BND5	QSOX1_MO	Qsox1	Qsox1	104009	Sulfhydryl o	no|up	0	ENOG410X
A0A0N4SW	A0A0N4SW	Tmem168	Tmem168	101118	Transmemb	no|no chan	0	ENOG410Y

Q8BIP0	SYDM_MC	Dars2	Dars2	226539	Aspartate--	no | no chan	0	COG0173(c
A0A286YEI	A0A286YEI	Rps24	Rps24		40S ribosom	no | no chan	0	COG2004(s
Q78XR0	TPC6A_MC	Trappc6a	Trappc6a	67091	Trafficking	no | no chan	0	ENOG410Y
O88673	DGKA_MC	Dgka	Dgka	13139	Diacylglyce	no | up	0	ENOG410X
A2AVY9	A2AVY9_M	Tgif2	Tgif2	228839	TGFB-indu	yes | down	1	ENOG410X
P58404	STRN4_MC	Strn4	Strn4	97387	Striatin-4	no | no chan	0	ENOG410X
Q3TPD9	Q3TPD9_M	Maoa	Maoa	17161	Amine oxid	no | no chan	0	ENOG410X
A0A0A6YX	A0A0A6YX	Rpl31	Rpl31		Large ribos	no | no chan	0	COG2097((
Q32NY4	CNNM3_M	Cnnm3	Cnnm3	94218	Metal trans	no | no chan	0	COG1253(C
A0A0R4J0K	A0A0R4J0K	Cd84	Cd84	12523	CD84 antig	no | no chan	0	ENOG410Z
Q8R3E3	WIPI1_MO	Wipi1	Wipi1	52639	WD repeat	no | no chan	0	ENOG410X
A0A0R4J0K	A0A0R4J0K	Ckap5	Ckap5	75786	Cytoskeleto	no | no chan	0	ENOG410X
A0A0R4J0K	A0A0R4J0K	Selenof	Selenof		Selenoprote	no | no chan	0	ENOG4111
A0A0R4J0K	A0A0R4J0K	Spred3	Spred3	101809	Sprouty-rel	no | no chan	0	ENOG4111
Q9DB77	QCR2_MO	Uqcrc2	Uqcrc2	67003	Cytochrom	no | no chan	0	COG0612(p
Q9DB73	NB5R1_MC	Cyb5r1	Cyb5r1	72017	NADH-cyto	no | no chan	0	COG0543(f
Q8BZ71	STAC3_MC	Stac3	Stac3	237611	SH3 and cy	no | up	0	ENOG410Y
A0A0R4J0K	A0A0R4J0K	Ap5m1	Ap5m1	74385	AP-5 compl	no | no chan	0	ENOG410X
Q9DB70	FUND1_M	Fundc1	Fundc1	72018	FUN14 dom	no | no chan	0	ENOG4112
B8JJC1	B8JJC1_MC	Tdp1	Tdp1	104884	Tyrosyl-DN	no | no chan	0	ENOG410X
A0A338P6U	A0A338P6U	Arid1b	Arid1b		AT-rich int	no | no chan	0	ENOG410Y
Q80UE4	Q80UE4_M	Epb41l2	Epb41l2	13822	Epb4.1l2 pr	no | no chan	0	ENOG410Y
B9EHX4	B9EHX4_M	Atm	Atm	11920	Serine-prot	no | no chan	0	ENOG410X
Q9CR26	VTA1_MO	Vta1	Vta1	66201	Vacuolar p	no | no chan	0	ENOG4111
A0A2I3BR8	A0A2I3BR8	Nt5dc2	Nt5dc2		5'-nucleotid	no | no chan	0	ENOG410X
F7CB97	F7CB97_M	Clip1	Clip1		CAP-GLY c	no | no chan	0	COG5244(C
Q8VC34	RPAP2_MC	Rpap2	Rpap2	231571	Putative RN	no | no chan	0	ENOG410Z
Q8VC30	TKFC_MO	Tkfc	Tkfc	225913	Triokinase/	no | no chan	0	COG2376(I
Q3U335	Q3U335_M	Stk24	Stk24	223255	non-specific	no | no chan	0	ENOG410X
Q9CXU1	MED31_M	Med31	Med31	67279	Mediator of	no | up	0	COG5088(C
Q61666	HIRA_MO	Hira	Hira	15260	Protein HIR	no | no chan	0	ENOG410X
D3YWT1	D3YWT1_M	Hnrnph3	Hnrnph3	432467	Heterogene	no | no chan	0	ENOG410Z
A0A1Y7VK	A0A1Y7VK	Crppa	Crppa	75847	D-ribitol-5-	no | down	0	COG1211(C
G3X8U3	QNG1_MC	QNG1	QNG1	70153	Queuosine	no | no chan	0	ENOG410X
Q4VAE3	TMM65_M	Tmem65	Tmem65	74868	Transmemb	no | no chan	0	ENOG4111
P20108	PRDX3_M	Prdx3	Prdx3	11757	Thioredoxin	no | no chan	0	COG0450(e
Q4VAE8	Q4VAE8_M	Ndufb4	Ndufb4	68194	NADH deh	no | no chan	0	ENOG4112
G3X8U8	G3X8U8_M	Parg	Parg	26430	poly(ADP-r	no | no chan	0	ENOG410X
A0A494BA	A0A494BA	Scd2	Scd2		Stearoyl-Co	no | no chan	0	COG1398(c
Q8C1S0	MED19_M	Med19	Med19	381379	Mediator of	no | no chan	0	ENOG4111
D6RFU9	D6RFU9_M	Sypl1	Sypl1		Synaptophy	no | no chan	0	ENOG4111
Q3U3H5	Q3U3H5_M	Galk2	Galk2	69976	N-acetylgal	no | no chan	0	COG0153(F
O89104	SYPL2_MC	Sypl2	Sypl2	17306	Synaptophy	no | up	0	ENOG4111

Q3TQB2	FXRD1_M(	Foxred1	Foxred1	235169	FAD-depen	no	no chan	0	ENOG410X
O89100	GRAP2_M(	Grap2	Grap2	17444	GRB2-relat	no	no chan	0	ENOG410X
O89103	C1QR1_M(	Cd93	Cd93	17064	Complemer	no	no chan	0	ENOG410Z
D3YVP9	D3YVP9_M	Gstm6	Gstm6		Glutathion	no	no chan	0	ENOG411(
Q9QXV1	CBX8_MO	Cbx8	Cbx8	30951	Chromobox	no	no chan	0	ENOG410X
Q8BJH1	ZC21A_M(	Zc2hc1a	Zc2hc1a	67306	Zinc finger	no	no chan	0	ENOG4111
P30993	C5AR1_M(	C5ar1	C5ar1	12273	C5a anaphy	no	up	0	ENOG410Y
A0A075B5N	A0A075B5N	Igkv6-13	Igkv6-13		Immunoglo	no	up	0	ENOG410Y
A0A075B5N	A0A075B5N	Igkv8-27	Igkv8-27		Immunoglo	no	no chan	0	ENOG410Y
A0A075B5N	A0A075B5N	Igkv8-28	Igkv8-28		Immunoglo	no	up	0	ENOG410Y
Q499F3	Q499F3_M	Tlr3	Tlr3	142980	Toll-like re	no	no chan	0	COG4886(T
B2RUH3	B2RUH3_M	Msrb1	Msrb1	27361	Methionine	no	no chan	0	COG0229(S
B2RUH2	B2RUH2_M	Abtb3	Abtb3	74007	BTB (POZ)	no	no chan	0	ENOG410X
D3Z3Z3	D3Z3Z3_M	Cacnb3	Cacnb3	12297	Voltage-dep	no	no chan	0	ENOG410X
A0A0J9YSF	A0A0J9YSF	Zfp729b	Zfp729b		Zinc finger	no	no chan	0	COG5048(U
A8Y5J3	A8Y5J3_M(	Ints11	Ints11		Integrator c	no	no chan	0	COG1236(r
Q3UYY2	Q3UYY2_M	Rad17	Rad17	19356	AAA+ ATP	no	no chan	0	COG0470(L
A0A0J9YTF	A0A0J9YTF	Ncbp2as2	Ncbp2as2		Ncbp2 anti	no	no chan	0	ENOG410Y
A8Y5J9	A8Y5J9_M(	Tmem39b	Tmem39b		Transmemb	no	no chan	0	ENOG410X
A0A2R8VI:	A0A2R8VI:	Gfus	Gfus		GDP-L-fuc	no	no chan	0	COG0451(r
A9E3L2	A9E3L2_M	Fkbp1b	Fkbp1b	14226	peptidylpro	no	no chan	0	COG0545(t
J3QK52	J3QK52_M	Noc2l	Noc2l	57741	NOC2 like	no	no chan	0	COG5604(r
Q542Q3	Q542Q3_M	Gtse1	Gtse1	29870	G2 and S pl	no	no chan	0	ENOG410Z
Q9DBC0	SELO_MO	Selenoo	Selenoo	223776	Protein ade	no	no chan	0	COG0397(U
Q0VGZ0	Q0VGZ0_M	Gtf2a1	Gtf2a1	83602	Gtf2a1 prot	no	up	0	ENOG4111
A2A6T1	CDR2L_M(	Cdr2l	Cdr2l	237988	Cerebellar (	no	no chan	0	ENOG410X
Q80ZS3	RT26_MOU	Mrps26	Mrps26	99045	Small ribos	no	no chan	0	ENOG4111
Q9WV27	AT1A4_M(	Atp1a4	Atp1a4	27222	Sodium/po	no	up	0	COG0474(F
F8VQ72	F8VQ72_M	Map3k1	Map3k1	26401	Mitogen-ac	no	no chan	0	ENOG410X
A2AHZ2	A2AHZ2_M	Siah1b	Siah1b	20438	E3 ubiquiti	no	up	0	ENOG410X
F8VQ75	F8VQ75_M	Kif13a	Kif13a	16553	Kinesin fan	no	no chan	0	COG5059(T
A2RT28	A2RT28_M	Mars2	Mars2	212679	Methionine	no	no chan	0	COG0143(I
B2RT41	B2RT41_M	Zfc3h1	Zfc3h1	216345	Zinc finger,	no	no chan	0	ENOG410X
Q2PZL6	FAT4_MOU	Fat4	Fat4	329628	Protocadhe	no	no chan	0	ENOG410X
P62627	DLRB1_M(	Dynlrb1	Dynlrb1	67068	Dynein ligh	no	no chan	0	ENOG4111
A0A5F8MP	A0A5F8MP	Itga6	Itga6		Integrin alp	no	no chan	0	ENOG410X
B1AVD1	XPP2_MOU	Xpnpep2	Xpnpep2	170745	Xaa-Pro an	no	down	0	COG0006(E
Q8VI78	PLA1A_M(	Pla1a	Pla1a	85031	Phospholip	yes	up	1	ENOG411(
A0A0J9YVI	A0A0J9YVI	Tmem129	Tmem129		Transmemb	no	no chan	0	ENOG410X
Q5BL07	PEX1_MOU	Pex1	Pex1	71382	Peroxisoma	no	no chan	0	COG0464(E
A0A0R4J13	A0A0R4J13	Phyhd1	Phyhd1	227696	Phytanoyl-(	no	no chan	0	ENOG410X
A0A494B8Y	A0A494B8Y	Yif1a	Yif1a		Protein YIF	no	up	0	COG5197(r
A0A0R4J13	A0A0R4J13	Btd	Btd	26363	Biotinidase	no	no chan	0	COG0388(E

Q91VS8	FARP2_MC	Farp2	Farp2	227377	FERM, ARI	no | no chan	0	ENOG410X
Q05CJ7	Q05CJ7_M	Ctr9	Ctr9	22083	Ctr9 protei	no | no chan	0	COG0457(F
A0A0R4J13	A0A0R4J13	Arsb	Arsb	11881	Arylsulfata	no | no chan	0	COG3119(I
A0A0R4J13	A0A0R4J13	Msrb3	Msrb3	320183	Peptide-me	no | no chan	0	COG0229(I
Q9WVM1	RGAP1_MC	Racgap1	Racgap1	26934	Rac GTPas	no | no chan	0	ENOG410X
Q3UHZ5	LMOD2_M	Lmod2	Lmod2	93677	Leiomodin-	no | no chan	0	ENOG410Y
E9PVS0	E9PVS0_M	Ifrd1	Ifrd1		Interferon-	no | no chan	0	ENOG410X
Q9QZE7	TSNAX_M	Tsnax	Tsnax	53424	Translin-as	no | no chan	0	COG2178(t
Q9QZE5	COPG1_M	Copg1	Copg1	54161	Coatomer s	no | no chan	0	COG5240(c
A0A1Y7VJZ	A0A1Y7VJZ	Gstz1	Gstz1	14874	maleylacetc	no | no chan	0	COG0625(C
A0A286YD	A0A286YD	Nolc1	Nolc1	70769	Nucleolar c	no | no chan	0	ENOG4111
B2RSR7	B2RSR7_M	Gpd1l	Gpd1l	333433	Glycerol-3-	no | no chan	0	COG0240(e
B2RSR0	B2RSR0_M	Cyp4v3	Cyp4v3	102294	Cytochrome	no | up	0	COG2124(A
Q02614	S30BP_MO	Sap30bp	Sap30bp	57230	SAP30-binc	no | no chan	0	ENOG4111
Q3U7D2	Q3U7D2_M	Rpl15	Rpl15	66480	Ribosomal	no | no chan	0	COG1632(F
E9QAT3	E9QAT3_M	Depdc5	Depdc5	277854	DEP domai	no | no chan	0	ENOG410X
E9QAT0	E9QAT0_M	Fmr1	Fmr1	14265	Fragile X m	no | no chan	0	ENOG410Z
Q3TU79	Q3TU79_M	Dnajb1	Dnajb1	81489	J domain-cc	no | no chan	0	COG0484(v
B7ZNL9	B7ZNL9_M	Tatdn2	Tatdn2	381801	TatD DNa	no | no chan	0	COG0084(t
P58242	ASM3B_MC	Smpdl3b	Smpdl3b	100340	Acid sphing	no | no chan	0	ENOG410X
E9QAT9	E9QAT9_M	Trim54	Trim54		Tripartite n	no | no chan	0	ENOG410Y
Q14AZ4	Q14AZ4_M	Oas1f	Oas1f	243262	2'-5' oligoac	yes | up	1	COG5272(u
Q3URQ7	MTHSD_M	Mthfsd	Mthfsd	234814	Methenylte	no | no chan	0	COG0212(5
Q3URQ0	TEX10_MC	Tex10	Tex10	269536	Testis-expre	no | no chan	0	ENOG410Y
Q8K4Z3	NNRE_MC	Naxe	Naxe	246703	NAD(P)H-I	no | no chan	0	COG0062(C
Q6ZWV7	RL35_MOU	Rpl35	Rpl35	66489	Large ribos	no | no chan	0	COG0255(5
A0A2X0SZ	A0A2X0SZ	PIK3R2	PIK3R2	18709	PIK3R2	no | no chan	0	ENOG410X
A0A068BFI	A0A068BFI	Rab11b	Rab11b	19326	RAS oncoge	no | no chan	0	COG1100(I
Q8K4Z5	SF3A1_MO	Sf3a1	Sf3a1	67465	Splicing fac	no | no chan	0	ENOG410X
Q9JJ78	TOPK_MC	Pbk	Pbk	52033	Lymphokin	no | no chan	0	COG0515(C
A2ARP1	VIP1_MOU	Ppip5k1	Ppip5k1	327655	Inositol hex	yes | up	1	ENOG410X
B2RY19	B2RY19_M	Cgnl1	Cgnl1	68178	Cgnl1 prote	no | no chan	0	ENOG410Y
A0A140T8S	A0A140T8S	Dolpp1	Dolpp1	57170	Dolichyl py	no | no chan	0	COG0671(M
A2ARP8	A2ARP8_M	Map1a	Map1a	17754	Microtubul	no | no chan	0	ENOG410X
E9PZ73	E9PZ73_M	Tead1	Tead1		TEA domai	no | no chan	0	ENOG410X
A0A6Q6PK	A0A6Q6PK	Tpgs1	Tpgs1		Tubulin po	no | no chan	0	ENOG4111
Q8BHS3	RBM22_MC	Rbm22	Rbm22	66810	Pre-mRNA	no | no chan	0	ENOG410X
Q3UUX7	CD046_MOUSE	Uncharacterized p		75939	Uncharacte	no | down	0	ENOG4111
D6REH1	D6REH1_M	Pxmp2	Pxmp2		Peroxisoma	no | down	0	ENOG4111
Q9D666	SUN1_MO	Sun1	Sun1	77053	SUN domai	no | no chan	0	ENOG410Y
Q5SUH5	Q5SUH5_M	Lsm11	Lsm11	72290	U7 snRNP-	no | no chan	0	ENOG4111
Q5SUH6	Q5SUH6_M	Clint1	Clint1	216705	Clathrin in	no | no chan	0	ENOG410X
Q9JME7	TPC2L_MC	Trappc2l	Trappc2l	59005	Trafficking	no | up	0	ENOG4111

Q3UK06	Q3UK06_M	Cyp4f16	Cyp4f16	70101	Uncharacte	no|no chan	0	COG2124(-
Q8K3K8	OPTN_MC	Optn	Optn	71648	Optineurin	no|up	0	ENOG4102
Q9CYL5	GAPR1_M	Glipr2	Glipr2	384009	Golgi-assoc	no|no chan	0	COG2340(I
B2KF50	B2KF50_M	Bltp3a	Bltp3a	224648	Bridge-like	no|no chan	0	ENOG410X
E9PZL9	E9PZL9_M	Uvssa	Uvssa		UV-stimula	no|no chan	0	ENOG410X
A0A0N4SW	A0A0N4SW	Zfp746	Zfp746	69228	Zinc finger	no|no chan	0	ENOG4111
Q545D8	Q545D8_M	Prg2	Prg2	19074	Proteoglyca	no|down	0	ENOG410X
Q99PU8	DHX30_M	Dhx30	Dhx30	72831	ATP-depen	no|no chan	0	COG1643(u
Q3V214	Q3V214_M	Polr2e	Polr2e	66420	DNA-direc	no|no chan	0	COG2012(I
Q5HZJ0	RNC_MOU	Drosha	Drosha	14000	Ribonuclea	no|no chan	0	COG0571(I
Q6NVG1	LPCT4_MC	Lpcat4	Lpcat4	99010	Lysophosph	no|no chan	0	ENOG410X
P25206	MCM3_MC	Mcm3	Mcm3	17215	DNA replic	no|no chan	0	COG1241(T
Q3TD55	Q3TD55_M	Fbxl8	Fbxl8	50788	F-box domc	no|up	0	ENOG4111
Q6PGG6	GNL3L_M	Gnl3l	Gnl3l	237107	Guanine nu	no|no chan	0	COG1161(F
Q3TIU4	PDE12_MC	Pde12	Pde12	211948	2',5'-phosph	no|no chan	0	COG5239(C
Q8C4B4	U119B_MC	Unc119b	Unc119b	106840	Protein unc	no|no chan	0	ENOG410Y
Q6PGG2	GMIP_MO	Gmip	Gmip	78816	GEM-intera	no|no chan	0	ENOG410X
A2A7Z4	A2A7Z4_M	Btf3l4	Btf3l4	70533	Transcripti	no|no chan	0	ENOG4111
Q6ZPT1	KLHL9_M	Klhl9	Klhl9	242521	Kelch-like p	no|no chan	0	ENOG410X
Q8BM80	Q8BM80_M	Tmem175	Tmem175	72392	Endosomal	no|no chan	0	ENOG4102
Q9CZX9	EMC4_MO	Emc4	Emc4	68032	ER membra	no|no chan	0	ENOG4111
P30355	AL5AP_MC	Alox5ap	Alox5ap	11690	Arachidono	no|no chan	0	ENOG4111
Q8BH15	CNO10_M	Cnot10	Cnot10	78893	CCR4-NOT	no|no chan	0	ENOG410X
A2RSY7	A2RSY7_M	Prf1	Prf1	18646	Perforin 1 (	no|no chan	0	ENOG410X
Q9CZX0	ELP3_MOU	Elp3	Elp3	74195	Elongator c	no|no chan	0	COG1243(F
A2RSY1	KANL3_M	Kansl3	Kansl3	226976	KAT8 regul	no|no chan	0	ENOG410X
Q9CZX5	PINX1_MC	Pinx1	Pinx1	72400	PIN2/TERF	no|no chan	0	ENOG4111
Q3UR91	Q3UR91_M	Pum2	Pum2	80913	Pumilio RN	no|no chan	0	COG5099(N
Q9D8Z6	ATGA1_M	Atg101	Atg101	68118	Autophagy-	no|no chan	0	ENOG410X
Q8BMI0	FBX38_MC	Fbxo38	Fbxo38	107035	F-box only	no|no chan	0	ENOG410X
Q922Q1	MARC2_M	Mtarc2	Mtarc2	67247	Mitochondr	no|no chan	0	COG3217(p
Q61169	GATA6_M	Gata6	Gata6	14465	Transcripti	no|up	0	COG5641(T
Q5DTJ9	MYPN_MC	Mypn	Mypn	68802	Myopalladi	no|up	0	ENOG411C
Q3TCN5	Q3TCN5_M	Gorasp1	Gorasp1	74498	PDZ GRAS	no|no chan	0	COG5233(p
Q91YH6	VATB1_M	Atp6v1b1	Atp6v1b1	110935	V-type prot	no|no chan	0	COG1156(F
Q3TCN2	PLBL2_MC	Plbd2	Plbd2	71772	Putative ph	no|no chan	0	ENOG410X
B2RXW4	B2RXW4_M	Cop1	Cop1	26374	Ring finger	no|no chan	0	ENOG410X
Q3TRG2	Q3TRG2_M	Map3k20	Map3k20	65964	Protein kin	no|up	0	COG0515(F
B2RXW1	B2RXW1_M	Hal	Hal	15109	Histidine a	yes|up	1	COG2986(H
Q6PDK8	DTX4_MO	Dtx4	Dtx4	207521	E3 ubiquiti	no|no chan	0	ENOG4111
A0A087WP	A0A087WP	Abhd17a	Abhd17a		Abhydrolas	no|no chan	0	COG1073(k
Q149U7	Q149U7_M	Ccl8	Ccl8	20307	C-C motif c	no|no chan	0	ENOG410Y
E9Q3Q6	E9Q3Q6_M	Alcam	Alcam	11658	CD166 anti	no|no chan	0	ENOG4102

A2BDN9	A2BDN9_M	1810030O0	1810030O0	69155	Uncharacte	no	no chan		0	ENOG4111
A0AAR0Q(	A0AAR0Q0P9_MOUSE	Lipid droplet-associat	Ldah			no	no chan		0	ENOG4102
B7ZP34	B7ZP34_M	Rft1	Rft1	328370	Protein RF	no	no chan		0	ENOG410Y
Q8BJW5	NOL11_M(	Nol11	Nol11	68979	Nucleolar p	no	no chan		0	ENOG4111
Q8BJW6	EIF2A_MC	Eif2a	Eif2a	229317	Eukaryotic	no	no chan		0	COG5354(N
Q8BJW7	EME1_MO	Eme1	Eme1	268465	Crossover ju	no	no chan		0	ENOG4111
Q9D7S9	CHMP5_M	Chmp5	Chmp5	76959	Charged mu	yes	up		1	ENOG410X
A2NTK0	A2NTK0_M	Lilra6	Lilra6	18726	PIRA3	no	no chan		0	ENOG4111
Q3UT32	Q3UT32_M	Zfyve1	Zfyve1	217695	FYVE-type	no	no chan		0	ENOG410X
Q99J19	SIM11_MO	Smim11	Smim11	68936	Small integ	no	no chan		0	ENOG4102
O35161	CELR1_M(	Celsr1	Celsr1	12614	Cadherin E	no	no chan		0	ENOG410X
Q5RJH6	SMG7_MO	Smg7	Smg7	226517	Nonsense-n	no	no chan		0	ENOG410X
Q5RJH2	MCTP2_M(	Mctp2	Mctp2	244049	Multiple C2	no	no chan		0	ENOG410X
P56546	CTBP2_M(	Ctbp2	Ctbp2	13017	C-terminal-	no	no chan		0	COG0111(E
Q5RJH9	Q5RJH9_M	Sppl3	Sppl3	74585	Signal pept	yes	up		1	ENOG410X
D3Z4S6	D3Z4S6_M	Tmem132a	Tmem132a		Transmemb	no	no chan		0	ENOG410X
A0A0G2JG	A0A0G2JG	Crybg1	Crybg1		Crystallin b	no	no chan		0	ENOG4110
A2AB59	RHG27_M(	Arhgap27	Arhgap27	544817	Rho GTPas	no	no chan		0	ENOG4102
D3Z4S3	PTRD1_M(	Ptrhd1	Ptrhd1	69709	Putative pe	no	no chan		0	ENOG4111
Q9QYI3	DNJC7_M(	Dnajc7	Dnajc7	56354	DnaJ homo	no	no chan		0	COG0484(c
Q9QYI5	DNJB2_M(	Dnajb2	Dnajb2	56812	DnaJ homo	no	no chan		0	COG0484(N
Q9CQM5	TXD17_M(	Txndc17	Txndc17	52700	Thioredoxi	no	no chan		0	ENOG4111
Q9QYI6	DNJB9_M(	Dnajb9	Dnajb9	27362	DnaJ homo	no	up		0	COG0484(N
B1AQR8	B1AQR8_M	Lgals9	Lgals9		Galectin	no	no chan		0	ENOG4111
Q99LJ7	RCBT2_M(	Rcbtb2	Rcbtb2	105670	RCC1 and I	no	no chan		0	COG5184(c
Q8BTV1	TUSC3_MC	Tusc3	Tusc3	80286	Dolichyl-di	no	no chan		0	ENOG410X
B0R0Y8	B0R0Y8_M	Sh3kbp1	Sh3kbp1		SH3-domai	no	no chan		0	ENOG410X
Q8BTV2	CPSF7_MC	Cpsf7	Cpsf7	269061	Cleavage ar	no	no chan		0	ENOG4111
Q91XF0	PNPO_MC	Pnpo	Pnpo	103711	Pyridoxine-	no	no chan		0	COG0259(C
P31001	DESM_MC	Des	Des	13346	Desmin	no	up		0	ENOG410X
Q61411	RASH_MO	Hras	Hras	15461	GTPase HR	no	no chan		0	COG1100(T
Q3UMU9	HDGR2_M	Hdgfl2	Hdgfl2	15193	Hepatoma-	no	no chan		0	ENOG4111
Q80YR2	FHI2B_MC	Fhip2b	Fhip2b	239170	FHF compl	no	no chan		0	ENOG410X
Q80YR6	CTIP_MOU	Rbbp8	Rbbp8	225182	DNA endon	no	no chan		0	ENOG4102
Q80YR7	CLSPN_M(	Clspn	Clspn	269582	Claspin	no	down		0	ENOG410Y
Q80YR4	ZN598_MC	Znf598	Znf598	213753	E3 ubiquiti	no	no chan		0	COG5236(z
A0A0A6YW	A0A0A6YW	Slamf7	Slamf7	75345	SLAM fami	no	no chan		0	ENOG4111
Q99LE3	LYSM3_M(	Lysmd3	Lysmd3	80289	LysM and p	no	no chan		0	ENOG4112
O88696	CLPP_MOU	Clpp	Clpp	53895	ATP-depen	no	no chan		0	COG0740(C
D3Z656	D3Z656_M	Synj1	Synj1	104015	phosphoinc	no	no chan		0	COG5411(f
P39039	MBL1_MO	Mbl1	Mbl1	17194	Mannose-b	no	no chan		0	ENOG410X
Q99MR8	MCCA_MC	Mccc1	Mccc1	72039	Methylcrot	no	no chan		0	COG4770(c
P70404	IDHG1_M(	Idh3g	Idh3g	15929	Isocitrate d	no	no chan		0	COG0473(3

Q9ERE7	MESD_MC	Mesd	Mesd	67943	LRP chaper	no | no chan	0	ENOG4111
P70402	MYBPH_M	Mybph	Mybph	53311	Myosin-bin	no | up	0	ENOG410Y
Q99MR1	GGYF1_M	Gigyf1	Gigyf1	57330	GRB10-inte	no | no chan	0	ENOG4111
Q9ERE8	TLRN1_M	Tlnrd1	Tlnrd1	80889	Talin rod d	no | no chan	0	ENOG410X
Q99MR3	S12A9_MO	Slc12a9	Slc12a9	83704	Solute carri	no | no chan	0	COG0531(C
Q3TGR2	Q3TGR2_M	Fgb	Fgb	110135	Fibrinogen	yes | up	1	ENOG410Z
Q99MR6	SRRT_MO	Srrt	Srrt	83701	Serrate RN	no | no chan	0	ENOG410X
P21440	MDR3_MC	Abcb4	Abcb4	18670	Phosphatid	no | no chan	0	COG1132(I
P21447	MDR1A_M	Abcb1a	Abcb1a	18671	ATP-depen	no | up	0	COG1132(I
A0JLY3	A0JLY3_M	F9	F9	14071	Coagulatio	yes | up	1	COG5640(t
Q8R238	SDSL_MO	Sdsl	Sdsl	257635	Serine dehy	no | no chan	0	COG1171(T
H3BJW0	H3BJW0_M	Ccn2	Ccn2		CCN family	no | no chan	0	ENOG4111
A0PJC8	A0PJC8_M	Zbtb33	Zbtb33	56805	Zbtb33 pro	no | no chan	0	COG5048(I
Q08288	LYAR_MO	Lyar	Lyar	17089	Cell growth	no | no chan	0	ENOG411C
O88441	MTX2_MO	Mtx2	Mtx2	53375	Metaxin-2	no | no chan	0	ENOG410X
P29699	FETUA_M	Ahsg	Ahsg	11625	Alpha-2-HS	no | no chan	0	ENOG4111
Q8BG51	MIRO1_M	Rhot1	Rhot1	59040	Mitochondı	no | no chan	0	ENOG410X
Q8VCT4	EST1D_M	Ces1d	Ces1d	104158	Carboxyles	no | down	0	COG2272(C
F2Z456	F2Z456_M	Cyb5r3	Cyb5r3		NADH-cyt	no | no chan	0	COG0543(C
Q8VCT3	AMPB_MC	Rnpep	Rnpep	215615	Aminopept	no | no chan	0	COG0308(M
Q3U422	Q3U422_M	Ndufv3	Ndufv3	78330	NADH:ubi	no | no chan	0	ENOG410Y
Q8R355	Q8R355_M	Lpxn	Lpxn	107321	Lpxn protei	no | no chan	0	ENOG410X
Q3UIB5	Q3UIB5_M	Dhrs4	Dhrs4	28200	NADPH-de	no | no chan	0	COG1028(I
A0A6I8MW	A0A6I8MW	Mtx1	Mtx1	17827	Metaxin-1	no | no chan	0	ENOG411C
Q9Z1X2	PTSS2_MO	Ptdss2	Ptdss2	27388	Phosphatid	no | no chan	0	ENOG410X
F6RJV6	F6RJV6_M	Lancl2	Lancl2		LanC (bact	no | no chan	0	ENOG410X
Q80U56	AVL9_MO	Avl9	Avl9	78937	Late secret	no | no chan	0	ENOG410Y
Q80U58	PUM2_MO	Pum2	Pum2	80913	Pumilio ho	no | no chan	0	COG5099(p
H3BLE5	H3BLE5_M	Kmt5b	Kmt5b		[histone H4	no | down	0	ENOG410X
Q8R1L4	ERD23_M	Kdelr3	Kdelr3	105785	ER lumen p	no | no chan	0	COG5196(v
Q7TN22	TXD16_M	Txndc16	Txndc16	70561	Thioredoxi	no | no chan	0	COG0526(S
A0A2X0TV	A0A2X0TV	ARAP3	ARAP3	106952	ARAP3	no | no chan	0	COG5347(r
Q5RKZ7	MOCS1_M	Mocs1	Mocs1	56738	Molybdenu	no | no chan	0	COG0315(T
A6MDD2	A6MDD2_	Ptprn	Ptprn	19275	Protein tyr	no | no chan	0	COG5599(C
A6MDD3	A6MDD3_	Cd109	Cd109	235505	CD109 anti	no | no chan	0	ENOG410X
G5E8P1	BRD1_MO	Brd1	Brd1	223770	Bromodom	no | no chan	0	COG5076(p
Q9D187	CIA2B_MC	Ciao2b	Ciao2b	68523	Cytosolic ir	no | down	0	COG5133(F
A0A0R4J0P	A0A0R4J0P	Parn	Parn	74108	Poly(A)-spe	no | no chan	0	ENOG410X
A0A0R4J0P	A0A0R4J0P	Slc22a18	Slc22a18	18400	Solute carri	no | down	0	ENOG4111
A0A0R4J0P	A0A0R4J0P	Pstpip1	Pstpip1	19200	Proline-seri	no | no chan	0	ENOG410X
A0A0R4J0P	A0A0R4J0P	Haspin	Haspin	14841	non-specifi	no | no chan	0	COG5072(g
A0A0R4J0P	A0A0R4J0P	Acad8	Acad8	66948	Acyl-Coenz	no | no chan	0	COG1960(V
E9Q9C6	E9Q9C6_M	Fcgbp	Fcgbp	215384	Fc fragmen	no | up	0	ENOG410X

Q91ZJ0	MUS81_MO	Mus81	Mus81	71711	Crossover ju	no | no chan	0	COG1948(c
E9Q9C3	E9Q9C3_M	Afdn	Afdn	17356	Afadin, adh	no | no chan	0	ENOG410X
E9Q9C0	E9Q9C0_M	Ablim1	Ablim1	226251	Actin-bindi	no | no chan	0	ENOG410X
E9Q4M2	E9Q4M2_M	Lipe	Lipe		Hormone-se	no | down	0	COG0657(c
E9Q4M4	E9Q4M4_M	Chchd6	Chchd6	66098	Coiled-coil-	no | no chan	0	ENOG4112
Q8K1X4	NCKPL_M	Nckap1l	Nckap1l	105855	Nck-associa	no | no chan	0	ENOG410X
E9Q4M7	E9Q4M7_M	Egfl7	Egfl7		EGF-like do	yes | down	1	ENOG410X
Q03145	EPHA2_MO	Epha2	Epha2	13836	Ephrin type	no | no chan	0	COG0515(C
Q921Q3	ALG1_MO	Alg1	Alg1	208211	Chitobiosyl	no | no chan	0	COG0438(S
P97927	LAMA4_M	Lama4	Lama4	16775	Laminin su	no | down	0	ENOG410Z
Q8CJG0	AGO2_MO	Ago2	Ago2	239528	Protein arg	no | no chan	0	ENOG410X
P82350	SGCA_MO	Sgca	Sgca	20391	Alpha-sarco	no | up	0	ENOG411C
Q8C2A2	SEN54_MO	Tsen54	Tsen54	76265	tRNA-splic	no | no chan	0	ENOG4111
Q3TY78	Q3TY78_M	Gnal	Gnal	14680	Guanine nu	no | no chan	0	ENOG410X
A0A5F8MQ	A0A5F8MQ	Prx	Prx		Periaxin	no | down	0	ENOG410Z
Q8BU31	RAP2C_MO	Rap2c	Rap2c	72065	Ras-related	no | no chan	0	COG1100(C
Q3U4K6	Q3U4K6_M	Cilk1	Cilk1	56542	Protein kin	yes | down	1	ENOG410X
Q99JG7	TNIP2_MO	Tnip2	Tnip2	231130	TNFAIP3-i	no | up	0	ENOG410X
E9PX62	E9PX62_M	Hdac7	Hdac7	56233	Histone dec	no | no chan	0	COG0123(r
Q9CQT5	POMP_MO	Pomp	Pomp	66537	Proteasome	no | no chan	0	ENOG410X
E9PX66	E9PX66_M	Supt7l	Supt7l		SPT7-like, S	no | no chan	0	ENOG410Y
P11214	TPA_MOU	Plat	Plat	18791	Tissue-type	no | up	0	COG5640(F
Q3UDE2	TTL12_MO	Ttll12	Ttll12	223723	Tubulin--ty	no | no chan	0	ENOG410X
E9PX68	E9PX68_M	Slc4a1ap	Slc4a1ap	20534	Solute carri	no | no chan	0	ENOG410X
Q5M8M3	Q5M8M3_M	Trmt112	Trmt112	67674	Multifuncti	no | no chan	0	ENOG4111
F8VPT6	F8VPT6_M	Evi5	Evi5	14020	Ecotropic v	no | no chan	0	ENOG410Y
F8VPT7	F8VPT7_M	Sik2	Sik2	235344	non-specifi	no | no chan	0	ENOG410X
B2RU82	B2RU82_M	Slc30a9	Slc30a9	109108	Proton-coup	no | up	0	COG0053(c
Q3TFU7	Q3TFU7_M	Bad	Bad	12015	Bcl2 antago	no | no chan	0	ENOG410Y
P10922	H10_MOUS	H1-0	H1-0	14958	Histone H1	no | no chan	0	ENOG4112
A0A1B0GS	A0A1B0GS	Arfip2	Arfip2	76932	ADP-ribosy	no | no chan	0	ENOG410Y
Q91V64	ISOC1_MO	Isoc1	Isoc1	66307	Isochorism	no | no chan	0	ENOG4111
Q05DC4	Q05DC4_M	Plpp2	Plpp2	50784	Ppap2c pro	no | no chan	0	COG0671(C
H7BX60	H7BX60_M	Pot1b	Pot1b	72836	Protection	no | no chan	0	ENOG410Z
Q3TFU8	Q3TFU8_M	Tmed9	Tmed9	67511	GOLD dom	no | no chan	0	ENOG410X
Q0VEE0	Q0VEE0_M	Nt5e	Nt5e	23959	5'-nucleotid	no | no chan	0	COG0737(5
Q8CFA1	IRAK2_MO	Irak2	Irak2	108960	Interleukin-	no | no chan	0	COG0515(I
F6V3Y9	F6V3Y9_M	Atg2a	Atg2a		Autophagy	no | no chan	0	ENOG410X
D3YVM8	D3YVM8_M	Bloc1s1	Bloc1s1		Biogenesis	no | no chan	0	ENOG4111
Q3UTN3	Q3UTN3_M	Sp2	Sp2	78912	C2H2-type	no | no chan	0	COG5048(p
Q3URV1	BROMI_M	Tbc1d32	Tbc1d32	544696	Protein bro	no | no chan	0	ENOG410X
D3YVM2	D3YVM2_M	Tmem59	Tmem59		Transmemb	no | no chan	0	ENOG410X
Q566I6	Q566I6_MO	C1ra	C1ra	50909	complemen	no | down	0	COG5640(g

Q8QZX2	HAUS3_M(	Haus3	Haus3	231123	HAUS augr	no | no chan	0	ENOG4111
P59041	DJC30_MC	Dnajc30	Dnajc30	66114	DnaJ homo	no | no chan	0	COG0484((
B9EJ80	PDZD8_M	Pdzd8	Pdzd8	107368	PDZ domai	no | no chan	0	ENOG410Z
Q7TMY7	IPO8_MOU	Ipo8	Ipo8	320727	Importin-8	no | no chan	0	COG5656(\
Q7TMY2	Q7TMY2_M	Acadsb	Acadsb	66885	Short/bran	no | no chan	0	COG1960(\
A2ACG7	A2ACG7_M	Rpn2	Rpn2		Dolichyl-di	no | no chan	0	ENOG410X
Q6DFZ1	GBF1_MO	Gbf1	Gbf1	107338	Golgi-speci	no | no chan	0	COG5307(c
P40336	VP26A_MC	Vps26a	Vps26a	30930	Vacuolar p	no | no chan	0	ENOG410X
Q8BTI8	SRRM2_M(	Srrm2	Srrm2	75956	Serine/argi	no | no chan	0	ENOG4111
Q6PAM0	AAKB2_M(	Prkab2	Prkab2	108097	5'-AMP-act	no | no chan	0	ENOG410X
P40338	VHL_MOU	Vhl	Vhl	22346	von Hippel-	no | no chan	0	ENOG4111
O35317	PBX3_MO	Pbx3	Pbx3	18516	Pre-B-cell l	no | no chan	0	ENOG410X
O35316	SC6A6_MC	Slc6a6	Slc6a6	21366	Sodium- an	no | no chan	0	COG0733(N
A2A699	F1712_MO	Fam171a2	Fam171a2	217219	Protein FA	no | down	0	ENOG410Z
Q6ZQ38	CAND1_M	Cand1	Cand1	71902	Cullin-asso	no | no chan	0	ENOG410X
Q9CWJ9	PUR9_MO	Atic	Atic	108147	Bifunctionc	no | no chan	0	COG0138(b
F8VQJ3	F8VQJ3_M	Lamc1	Lamc1	226519	Laminin, gc	no | no chan	0	ENOG410X
Q9CWJ3	KTBL1_M(	Katnbl1	Katnbl1	72425	KATNB1-li	no | no chan	0	ENOG4111
Q7M739	Q7M739_M	Tpr	Tpr	108989	Nucleoprot	no | no chan	0	ENOG410X
Q78IS1	TMED3_M	Tmed3	Tmed3	66111	Transmemb	no | no chan	0	ENOG4111
Q8C0Q9	RPGF5_M(	Rapgef5	Rapgef5	217944	Rap guanin	no | no chan	0	ENOG410X
Q9EPR4	S23A2_MO	Slc23a2	Slc23a2	54338	Solute carri	no | no chan	0	COG2233(p
E9PV87	TALD3_M(	Talpid3	Talpid3	76967	Protein TA	no | no chan	0	ENOG4112
Q8R2N0	TAP26_MC	Ccdc59	Ccdc59	52713	Thyroid tra	no | no chan	0	ENOG4111
Q7TNI6	Q7TNI6_M	Tom1l1	Tom1l1	71943	Jerry protei	no | no chan	0	ENOG410Y
A2ATS4	A2ATS4_M	Slc43a1	Slc43a1	72401	Solute carri	no | no chan	0	ENOG410Y
Q9D853	EFMT2_M(	Eef1akmt2	Eef1akmt2	72096	EEF1A lysi	no | no chan	0	ENOG4111
Q91XU0	WRIP1_MC	Wrnip1	Wrnip1	78903	ATPase WF	no | no chan	0	COG2256(F
Q91WN4	KMO_MO	Kmo	Kmo	98256	Kynurenine	no | no chan	0	COG0654(c
Q9D850	DIESL_MC	Tmem68	Tmem68	72098	DGAT1/2-	no | no chan	0	COG0204(A
A0A1D8RI	A0A1D8RI	Rasef	Rasef	242505	Testis-speci	no | up	0	ENOG410X
Q91WN1	DNJC9_M(	Dnajc9	Dnajc9	108671	DnaJ homo	no | no chan	0	COG2214(T
A0A494B9X	A0A494B9X	Nt5c2	Nt5c2	76952	Cytosolic p	no | no chan	0	ENOG410X
Q14AA1	Q14AA1_M	Dok3	Dok3	27261	Docking pr	no | no chan	0	ENOG410X
P47877	IBP2_MOU	Igfbp2	Igfbp2	16008	Insulin-like	no | up	0	ENOG4111
E9Q8D1	E9Q8D1_M	Zmynd8	Zmynd8	228880	Zinc finger,	no | no chan	0	ENOG410X
E9Q8D0	DJC21_MC	Dnajc21	Dnajc21	78244	DnaJ homo	no | no chan	0	ENOG410X
E9Q1P8	I2BP2_MO	Irf2bp2	Irf2bp2	270110	Interferon r	no | no chan	0	ENOG411(
Q2TBA3	MALT1_M(	Malt1	Malt1	240354	Mucosa-ass	no | no chan	0	COG4249(p
Q9ER72	SYCC_MO	Cars1	Cars1	27267	Cysteine--tI	no | no chan	0	COG0215((
Q3U2X6	Q3U2X6_M	Pdcd7	Pdcd7	50996	Programme	no | no chan	0	ENOG4112
A0A0N4SW	A0A0N4SW	Rab11fip5	Rab11fip5	52055	RAB11 fam	no | no chan	0	ENOG410X
E9Q7D5	ARHG5_M	Arhgef5	Arhgef5	54324	Rho guanin	no | no chan	0	COG5422(p

B2RXS3	B2RXS3_M	Scaper	Scaper	244891	S phase cyc	no | no chan	0	ENOG410
G3X956	G3X956_M	Supt16	Supt16	114741	FACT comp	no | no chan	0	COG5406(
Q8BIR2	ASTE1_MC	Aste1	Aste1	66595	Single-strar	no | no chan	0	ENOG4111
Q91WM3	U3IP2_MO	Rrp9	Rrp9	27966	U3 small nu	no | no chan	0	ENOG410
A0A2I3BP(	A0A2I3BP(	Zc3h13	Zc3h13	67302	Zinc finger	no | no chan	0	ENOG4111
F7D3W5	F7D3W5_N	Gm45837	Gm45837		Phosphodie	no | no chan	0	ENOG410
P58468	SLX9_MOU	Slx9	Slx9	108707	Ribosome b	no | no chan	0	ENOG410
Q3TWJ4	Q3TWJ4_M	Actr3	Actr3	74117	Actin-relate	no | no chan	0	COG5277(
Q3TG12	Q3TG12_M	Farsb	Farsb	23874	Phenylalan	no | no chan	0	COG0072(
P55284	CADH5_M	Cdh5	Cdh5	12562	Cadherin-5	no | no chan	0	ENOG410
P55288	CAD11_M(	Cdh11	Cdh11	12552	Cadherin-1	no | no chan	0	ENOG410
P58466	CTDS1_M(	Ctdsp1	Ctdsp1	227292	Carboxy-ter	no | no chan	0	COG5190(
Q8R3C0	MCMBP_M	Mcmbp	Mcmbp	210711	Mini-chrom	no | no chan	0	ENOG410
D3YUP5	D3YUP5_M	Exoc3l2	Exoc3l2		Exocyst cor	no | no chan	0	COG5173(
D3YUP1	D3YUP1_M	Carm1	Carm1		Histone-arg	no | no chan	0	ENOG410
Q8R3C6	RBM19_M(	Rbm19	Rbm19	74111	Probable R	no | no chan	0	ENOG410
Q9DB10	EMRE_MC	Smdt1	Smdt1	69029	Essential M	yes | down	1	ENOG411
Q9DB15	RM12_MO	Mrpl12	Mrpl12	56282	Large ribos	no | no chan	0	COG0222(
Q71RI9	KAT3_MO	Kyat3	Kyat3	229905	Kynurenine	no | no chan	0	COG0436(
D3YUP8	D3YUP8_M	Ing3	Ing3	71777	Inhibitor of	no | no chan	0	COG5034(
A0A338P6V	A0A338P6V	Ttc3	Ttc3		RING-type	no | no chan	0	ENOG411(
Q80UK7	SAS6_MOU	Sass6	Sass6	72776	Spindle ass	no | no chan	0	ENOG410
Q6P3E7	HDA10_M(	Hdac10	Hdac10	170787	Polyamine	no | no chan	0	COG0123(
B9EHZ5	B9EHZ5_M	Pals2	Pals2	56524	Membrane	no | no chan	0	COG0194(
P11983	TCPA_MO	Tcp1	Tcp1	21454	T-complex	no | no chan	0	COG0459(
Q9D7W5	MED8_MC	Med8	Med8	80509	Mediator of	no | no chan	0	ENOG410
A2AN61	A2AN61_M	Ciz1	Ciz1	68379	CDKN1A i	no | no chan	0	ENOG4111
P18872	GNAO_M(	Gnao1	Gnao1	14681	Guanine nu	no | no chan	0	ENOG410
O70152	DPM1_MC	Dpm1	Dpm1	13480	Dolichol-ph	no | no chan	0	COG0463(
Q04857	CO6A1_M(	Col6a1	Col6a1	12833	Collagen al	no | down	0	ENOG410
Q6PJN8	DALD3_M	Dalrd3	Dalrd3	67789	DALR anti	no | no chan	0	COG0018(
A0A1S6GW	A0A1S6GW	Nsun2	Nsun2	28114	tRNA (cyto	no | no chan	0	COG0144(
A0A068BE	A0A068BE	Tap1	Tap1	21354	ABC-type a	no | up	0	COG1132(
P23116	EIF3A_MC	Eif3a	Eif3a	13669	Eukaryotic	no | no chan	0	ENOG410
Q61609	S20A1_MO	Slc20a1	Slc20a1	20515	Sodium-dep	no | no chan	0	COG0306(
Q6PIJ4	NFRKB_M	Nfrkb	Nfrkb	235134	Nuclear fac	no | no chan	0	ENOG410
F6SPK0	F6SPK0_M	Ube2j1	Ube2j1		E2 ubiquiti	no | no chan	0	COG5078(
A0A1Y7VK	A0A1Y7VK	Isca2	Isca2		Iron-sulfur	no | no chan	0	COG0316(
Q8BHT6	B3GLT_M(	B3glct	B3glct	381694	Beta-1,3-glu	no | no chan	0	ENOG410
A2AIV6	A2AIV6_M	Snapc4	Snapc4		Small nucle	no | no chan	0	COG5147(
Q569Z5	DDX46_M	Ddx46	Ddx46	212880	Probable A	no | no chan	0	ENOG410
Q8K4A2	Q8K4A2_M	Pcdhgc5	Pcdhgc5	93708	Protocadhe	no | up	0	ENOG410
Q569Z6	TR150_MO	Thrap3	Thrap3	230753	Thyroid hor	no | no chan	0	ENOG410

Q6PDL0	DC1L2_MC	Dync1li2	Dync1li2	234663	Cytoplasmi	no | no chan	0	ENOG410X
P17918	PCNA_MO	Pcna	Pcna	18538	Proliferatin	no | no chan	0	COG0592(I
B1AUY7	B1AUY7_M	Naa10	Naa10		N-terminal	no | no chan	0	COG0456(N
Q8BGT5	ALAT2_MC	Gpt2	Gpt2	108682	Alanine am	no | no chan	0	COG0436(S
Q8BGT6	MILK1_MC	Micall1	Micall1	27008	MICAL-like	no | up	0	COG5069(C
P17665	COX7C_M	Cox7c	Cox7c	654359; 128	Cytochrom	no | no chan	0	ENOG4112
Q8BGT0	OSTM1_M	Ostm1	Ostm1	14628	Osteopetro	no | no chan	0	ENOG4111
Q3UNV7	Q3UNV7_M	Mlf2	Mlf2	30853	Myeloid leu	no | no chan	0	ENOG4111
Q8BPE4	TM177_MC	Tmem177	Tmem177	66343	Transmemb	no | up	0	ENOG4111
A0A217FL5	A0A217FL5	Slc35d1	Slc35d1		Nucleotide	no | up	0	COG5070(Y
Q8VDM6	HNRL1_M	Hnrnpul1	Hnrnpul1	232989	Heterogene	no | no chan	0	ENOG410X
Q3U3J1	Q3U3J1_M	Bckdha	Bckdha	12039	2-oxoisoval	no | no chan	0	COG1071(E
Q3U3J3	Q3U3J3_M	Polrmt	Polrmt	216151	DNA-direc	no | no chan	0	COG5108(I
Q05816	FABP5_MC	Fabp5	Fabp5	16592	Fatty acid-l	no | no chan	0	ENOG4111
B2RUJ6	B2RUJ6_M	Rapgef6	Rapgef6	192786	Rap guanin	no | no chan	0	ENOG410X
B2RUJ9	B2RUJ9_M	Pde4dip	Pde4dip	83679	Pde4dip pr	no | no chan	0	ENOG4112
Q62266	SPR1A_MC	Sprr1a	Sprr1a	20753	Cornifin-A	no | up	0	ENOG410X
Q62261	SPTB2_MC	Sptbn1	Sptbn1	20742	Spectrin be	no | no chan	0	COG5069(S
P18293	ANPRA_M	Npr1	Npr1	18160	Atrial natri	no | no chan	0	COG2114(I
Q8BYZ1	ABI3_MOU	Abi3	Abi3	66610	ABI gene fa	no | no chan	0	ENOG410Y
Q8BG95	MYPT2_MC	Ppp1r12b	Ppp1r12b	329251	Protein pho	no | up	0	COG0666(r
Q3TV70	NR2CA_M	Nr2c2ap	Nr2c2ap	75692	Nuclear rec	no | up	0	ENOG4111
A0A0J9YT1	A0A0J9YT1	Ube2a	Ube2a		Ubiquitin-c	no | no chan	0	COG5078(-
P46061	RAGP1_MC	Rangap1	Rangap1	19387	Ran GTPas	no | no chan	0	COG5238(F
Q8CGC6	RBM28_MC	Rbm28	Rbm28	68272	RNA-bindi	no | no chan	0	ENOG410X
A8Y5H7	S14L1_MO	Sec14l1	Sec14l1	74136	SEC14-like	no | no chan	0	ENOG410X
Q5NCE8	MRS2_MO	Mrs2	Mrs2	380836	Magnesium	no | no chan	0	ENOG410X
Q80ZQ9	ABITM_MC	Abitram	Abitram	230234	Protein Abi	no | no chan	0	ENOG4111
P98063	BMP1_MO	Bmp1	Bmp1	12153	Bone morpl	no | no chan	0	ENOG410Z
P98064	MASP1_MC	Masp1	Masp1	17174	Mannan-bi	no | no chan	0	COG5640(N
A2A997	A2A997_M	C8a	C8a	230558	Complemer	no | no chan	0	ENOG410Y
A0A0G2JE	A0A0G2JE	Rbm8a	Rbm8a		RNA-bindi	no | no chan	0	ENOG4111
B1AVY7	KI16B_MO	Kif16b	Kif16b	16558	Kinesin-like	no | no chan	0	COG5059(u
Q3UAA9	Q3UAA9_M	Actb	Actb	11461	Actin, cytop	no | up	0	COG5277(g
F8VQ54	F8VQ54_M	Dicer1	Dicer1	192119	Endoribonu	no | no chan	0	COG1111(C
A2AHX9	A2AHX9_M	Bcl2l1	Bcl2l1		Bcl-2-like p	no | no chan	0	ENOG4112
Q7TSV4	PGM2_MO	Pgm2	Pgm2	66681	Phosphope	no | no chan	0	COG1109(C
Q7TSV9	ENKD1_M	Enkd1	Enkd1	102124	Enkurin do	no | no chan	0	ENOG4111
A0A0R4J11	A0A0R4J11	Utp25	Utp25	215193	U3 small nu	no | no chan	0	ENOG410X
A0A0R4J11	A0A0R4J11	Igsf8	Igsf8	140559	Immunoglo	no | no chan	0	ENOG410X
Q8BIU1	Q8BIU1_M	Chdh	Chdh	218865	Glucose-me	no | no chan	0	COG2303(c
Q91VU8	PTOV1_MC	Ptov1	Ptov1	84113	Prostate tu	no | down	0	ENOG410Z
A0A9L6H7	A0A9L6H7	Tet1	Tet1		Methylcyto	no | no chan	0	ENOG410X

P24668	MPRD_MC	M6pr	M6pr	17113	Cation-dep	no | no chan	0	ENOG4111
E9PVU0	E9PVU0_M	Myo6	Myo6		Unconventi	no | no chan	0	COG5022(I
A0A1D5RL	A0A1D5RL	Zfp1	Zfp1	22640	Zinc finger	no | no chan	0	COG5048(I
D3Z5N8	D3Z5N8_M	Plekhg2	Plekhg2	101497	Pleckstrin h	no | no chan	0	ENOG410X
A1BN54	A1BN54_M	Actn1	Actn1	109711	Alpha-actin	no | no chan	0	COG5069(M
A0A2I3BQ	A0A2I3BQ	Sall2	Sall2	50524	Spalt like tr	no | no chan	0	ENOG410Z
Q8K4X7	PLCD_MO	Agpat4	Agpat4	68262	1-acyl-sn-gl	no | no chan	0	COG0204(C
Q9D0K1	PEX13_MC	Pex13	Pex13	72129	Peroxisoma	no | no chan	0	ENOG410X
Q7TS98	Q7TS98_MOUSE	Anti-colorectal carcinoma li			Anti-colore	no | up	0	ENOG4111
Q91WS7	DEPD7_M	Depdc7	Depdc7	211896	DEP domai	no | no chan	0	ENOG410Z
B7ZNJ0	B7ZNJ0_M	Adam17	Adam17	11491	A disintegri	no | no chan	0	ENOG410X
Q3U7F6	Q3U7F6_M	Zfp422	Zfp422	67255	C2H2-type	no | down	0	COG5048(F
G3UYV7	G3UYV7_M	Rps28	Rps28		Small ribos	no | no chan	0	COG2053(F
B7ZNJ6	B7ZNJ6_M	Spata13	Spata13	219140	Spata13 prc	yes | up	1	ENOG410X
Q9WV02	RBMX_MC	Rbmx	Rbmx	19655	RNA-bindi	no | no chan	0	ENOG4111
E0CY18	E0CY18_M	Zfand2b	Zfand2b		Zinc finger,	no | no chan	0	ENOG4111
P60764	RAC3_MO	Rac3	Rac3	170758	Ras-related	no | no chan	0	COG1100(e
H3BJJ1	H3BJJ1_MC	Gmnn	Gmnn		Geminin	no | up	0	ENOG410X
P60766	CDC42_MC	Cdc42	Cdc42	12540	Cell divisio	no | no chan	0	COG1100(e
B2RQV1	B2RQV1_M	Naa25	Naa25	231713	N-alpha-ac	no | no chan	0	ENOG410X
B9EKC1	B9EKC1_M	Pask	Pask	269224	non-specifi	no | no chan	0	COG0515(I
A0A0G2JD	A0A0G2JD	Ighv8-12	Ighv8-12		Immunoglo	no | down	0	ENOG4111
A0A140T8C	A0A140T8C	Milr1	Milr1	380732	Mast cell in	no | up	0	ENOG4111
A0A0G2JD	A0A0G2JD	Adgrl2	Adgrl2		Adhesion G	no | no chan	0	ENOG410X
Q8CAK1	CAF17_MC	Iba57	Iba57	216792	Putative trc	no | no chan	0	COG0354(I
E9PZ16	E9PZ16_M	Hspg2	Hspg2	15530	Perlecan (h	no | no chan	0	ENOG410X
Q3TZK4	Q3TZK4_M	Immt	Immt	76614	MICOS con	no | up	0	ENOG410Y
Q6DVA0	LEMD2_M	Lemd2	Lemd2	224640	LEM doma	no | no chan	0	ENOG410X
Q9Z247	FKBP9_MC	Fkbp9	Fkbp9	27055	Peptidyl-pr	no | no chan	0	COG0545(U
E9QMX4	E9QMX4_M	Rps6kc1	Rps6kc1	320119	non-specifi	no | no chan	0	ENOG410X
Q3TBJ0	Q3TBJ0_M	Fbxo42	Fbxo42	213499	F-box domc	no | no chan	0	ENOG410Y
A0A668KL	A0A668KL	Nkrf	Nkrf		NF-kappaB	no | no chan	0	COG5048(C
Q6NZQ4	PAXI1_MC	Paxip1	Paxip1	55982	PAX-intera	no | no chan	0	ENOG410X
Q8VI59	PCX3_MO	Pcnx3	Pcnx3	104401	Pecanex-lik	no | no chan	0	ENOG410X
Q5XF89	AT133_MC	Atp13a3	Atp13a3	224088	Polyamine-	no | no chan	0	ENOG410X
P81122	IRS2_MOU	Irs2	Irs2	384783	Insulin rece	no | no chan	0	ENOG410Z
E9Q0W6	E9Q0W6_M	Ablim2	Ablim2	231148	Actin-bindi	no | no chan	0	ENOG410X
Q3UK24	Q3UK24_M	Wdr37	Wdr37	207615	WD repeat-	no | no chan	0	ENOG410X
Q3UPI1	GAK1B_M	Gask1b	Gask1b	68659	Golgi-assoc	no | down	0	ENOG410Y
Q9JMK2	KC1E_MO	Csnk1e	Csnk1e	27373	Casein kina	no | no chan	0	ENOG410X
E9Q2B2	E9Q2B2_M	Ncor1	Ncor1		Nuclear rec	no | no chan	0	ENOG410Y
Q9JMK0	B4GT5_MC	B4galt5	B4galt5	56336	Beta-1,4-ga	no | no chan	0	ENOG410Z
Q99JI1	MSTN1_M	Mustn1	Mustn1	66175	Musculoske	no | up	0	ENOG410Y

P47810	WEE1_MO	Wee1	Wee1	22390	Wee1-like p	no | no chan	0	ENOG410
P47811	MK14_MO	Mapk14	Mapk14	26416	Mitogen-ac	no | no chan	0	ENOG410
B9EKC2	B9EKC2_M	Abcc4	Abcc4	239273	ATP-bindir	no | no chan	0	COG1132(i
B9EKC3	B9EKC3_M	Arhgap5	Arhgap5	11855	Rho GTPas	no | no chan	0	ENOG410
D3YUL8	D3YUL8_M	Inpp4a	Inpp4a		phosphatid	no | no chan	0	ENOG410
G3X9X1	KBTB2_M	Kbtbd2	Kbtbd2	210973	Kelch repec	no | no chan	0	ENOG410
D2EAC2	ZBED6_M	Zbed6	Zbed6	667118	Zinc finger	no | no chan	0	ENOG410
Q91XB2	Q91XB2_M	Mrpl3	Mrpl3	94062	Large ribos	no | no chan	0	COG0087(
Q3TRA2	Q3TRA2_M	Ust	Ust	338362	Uronyl 2-su	no | no chan	0	ENOG410
Q9D1K2	VATF_MO	Atp6v1f	Atp6v1f	66144	V-type prot	no | no chan	0	COG1436(
Q68ED7	CRTC1_M	Crtc1	Crtc1	382056	CREB-regu	no | no chan	0	ENOG410
Q3V231	Q3V231_M	Cnot7	Cnot7	18983	CCR4-NOT	no | no chan	0	COG5228(
D3YZR6	D3YZR6_M	Zbtb7b	Zbtb7b		Zinc finger	no | down	0	COG5048(
A0A1B0GR	A0A1B0GR	Rtp4	Rtp4		Receptor tr	no | no chan	0	ENOG4111
Q6ZPV2	INO80_M	Ino80	Ino80	68142	Chromatin-	no | no chan	0	ENOG410
D3YZR1	D3YZR1_M	Tssc4	Tssc4		U5 small nu	no | no chan	0	ENOG411
Q3TD71	Q3TD71_M	Scamp1	Scamp1	107767	Secretory c	no | no chan	0	ENOG410
Q99J77	SIAS_MOU	Nans	Nans	94181	N-acetylne	no | no chan	0	COG2089(s
P11404	FABPH_M	Fabp3	Fabp3	14077	Fatty acid-l	no | up	0	ENOG4111
A0A338P6J	A0A338P6J	Sec22a	Sec22a		SEC22 hom	no | no chan	0	COG5143(
P14434	HA2B_MO	H2-Aa	H2-Aa	14960	H-2 class II	no | no chan	0	ENOG410
Q3UD53	Q3UD53_M	Carhsp1	Carhsp1	52502	CSD domai	no | no chan	0	COG1278(
Q3UI84	Q3UI84_M	Rfc4	Rfc4	106344	Replication	no | no chan	0	COG0470(
Q8BMK1	METL2_M	Mettl2	Mettl2	52686	tRNA N(3)-	no | no chan	0	ENOG410
Q8BH79	ANO10_M	Ano10	Ano10	102566	Anoctamin-	no | no chan	0	ENOG410
P20491	FCERG_M	Fcer1g	Fcer1g	14127	High affini	no | no chan	0	ENOG410
Q8BMK4	CKAP4_M	Ckap4	Ckap4	216197	Cytoskeleto	no | no chan	0	ENOG4111
Q8BH73	QPCTL_M	Qpctl	Qpctl	67369	Glutaminyl	no | no chan	0	ENOG410
P22892	AP1G1_M	Ap1g1	Ap1g1	11765	AP-1 compl	no | no chan	0	ENOG410
Q8BH75	RNF41_M	Rnf41	Rnf41	67588	E3 ubiquiti	yes | down	1	ENOG410
P25322	CCND1_M	Ccnd1	Ccnd1	12443	G1/S-speci	no | no chan	0	ENOG410
Q91YJ5	IF2M_MOU	Mtif2	Mtif2	76784	Translation	no | no chan	0	COG0532(
Q91XU3	PI42C_MO	Pip4k2c	Pip4k2c	117150	Phosphatid	no | no chan	0	COG5253(
Q91YJ3	THYN1_M	Thyn1	Thyn1	77862	Thymocyte	no | no chan	0	COG2947(
Q9CQR6	PPP6_MOU	Ppp6c	Ppp6c	67857	Serine/thre	no | no chan	0	COG0639(
A2AKM2	HACD4_M	Hacd4	Hacd4	66775	Very-long-c	no | up	0	COG5198(
A2A5N2	A2A5N2_M	Ywhab	Ywhab	54401	14-3-3 prot	no | no chan	0	COG5040(
Q3UQ84	SYTM_MO	Tars2	Tars2	71807	Threonine--	no | no chan	0	COG0441(
Q3T9T2	Q3T9T2_M	Noc4l	Noc4l	100608	CCAAT-bir	no | no chan	0	ENOG410
P23949	TISD_MOU	Zfp36l2	Zfp36l2	12193	mRNA dec	no | no chan	0	COG5063(z
Q3TCH7	CUL4A_M	Cul4a	Cul4a	99375	Cullin-4A	no | no chan	0	COG5647(
E9PWY9	E9PWY9_M	Farsa	Farsa		Phenylalan	no | no chan	0	COG0016(
Q9JJN6	CNBP1_M	Ctnnbip1	Ctnnbip1	67087	Beta-cateni	no | no chan	0	ENOG4111

Q9CQ92	FIS1_MOU	Fis1	Fis1	66437	Mitochondı	no|no chan	0	ENOG4111
Q9CQ91	NDUA3_M	Ndufa3	Ndufa3	66091	NADH deh	no|no chan	0	ENOG4112
A3KGJ7	A3KGJ7_M	Cep250	Cep250	16328	Centrosom	no|no chan	0	ENOG410
A0A1L1ST(	A0A1L1ST(	Higd1a	Higd1a		HIG1 dom	no|no chan	0	ENOG4112
P62717	RL18A_MC	Rpl18a	Rpl18a	76808	Large ribos	no|no chan	0	COG2157(r
A0A3B2W4	A0A3B2W4	Cyp39a1	Cyp39a1	56050	Cytochrom	no|up	0	COG2124(i
F6VRP8	F6VRP8_M	Lgals3bp	Lgals3bp		Lectin, gala	no|up	0	ENOG410
Q8BRF7	SCFD1_MC	Scfd1	Scfd1	76983	Sec1 family	no|no chan	0	COG5158(c
Q6PD28	2A5B_MOI	Ppp2r5b	Ppp2r5b	225849	Serine/thre	no|up	0	ENOG410
A0A087WS	A0A087WS	Gm13889	Gm13889	620695	Predicted g	no|up	0	
O55013	TPPC3_MC	Trappc3	Trappc3	27096	Trafficking	no|no chan	0	ENOG410
Q6PD21	SHB_MOU	Shb	Shb	230126	SH2 domai	no|no chan	0	ENOG410
F8WI37	F8WI37_M	Kmt2c	Kmt2c	231051	[histone H3	no|no chan	0	COG2940(S
A4GE64	A4GE64_M	Ctnna3	Ctnna3	216033	Alpha-T-ca	no|no chan	0	ENOG410
F6RND9	F6RND9_M	Mprip	Mprip		Myosin pho	no|up	0	ENOG410
Q33DR3	DLP1_MO	Pdss2	Pdss2	71365	All trans-p	no|no chan	0	COG0142(M
Q33DR2	DPS1_MOI	Pdss1	Pdss1	56075	All trans-p	no|no chan	0	COG0142(M
A2A7W1	A2A7W1_M	Cd300ld4	Cd300ld4	100043123;	CD300 mol	no|no chan	0	ENOG410
Q9DCE9	IRGM3_M	Igtp	Igtp	16145	Immunity-r	no|up	0	ENOG4112
Q6ZPY7	KDM3B_M	Kdm3b	Kdm3b	277250	Lysine-spec	no|no chan	0	ENOG410
Q3TER9	Q3TER9_M	Brip1	Brip1	237911	Helicase A	no|no chan	0	COG1199(I
P31532	SAA4_MOI	Saa4	Saa4	20211	Serum amyl	no|no chan	0	ENOG4111
Q6NXW0	Q6NXW0_	Akt3	Akt3	23797	non-specifi	no|no chan	0	ENOG410
Q99LH4	ZN672_MC	Znf672	Znf672	319475	Zinc finger	no|no chan	0	COG5048(c
A0A159AK	A0A159AK	Fads3	Fads3	60527	Fatty acid c	no|no chan	0	ENOG410
Q3UJB0	SF3B2_MO	Sf3b2	Sf3b2	319322	Splicing fac	no|no chan	0	COG5182(S
Q61475	DAF1_MO	Cd55	Cd55	13136	Complemer	no|no chan	0	ENOG410
Q61478	Q61478_M	Ybx3	Ybx3	56449	DbpA muri	no|down	0	COG1278(I
Q924Z4	CERS2_MC	Cers2	Cers2	76893	Ceramide s	no|no chan	0	COG5058(c
Q924Z5	TRAM2_M	Tram2	Tram2	170829	Translocati	no|no chan	0	ENOG410
Q924Z6	XPO6_MO	Xpo6	Xpo6	74204	Exportin-6	no|no chan	0	COG5101(I
Q07243	MTF1_MO	Mtf1	Mtf1	17764	Metal regul	no|no chan	0	COG5048(I
Q9D7P9	SPB12_MO	Serpinb12	Serpinb12	71869	Serpin B12	yes|down	1	COG4826(p
Q8CEG5	CC28B_MC	Ccdc28b	Ccdc28b	66264	Coiled-coil	no|up	0	ENOG4111
Q80YP6	ZIK1_MOI	Zik1	Zik1	22775	Zinc finger	no|no chan	0	COG5048(r
P29452	CASP1_MC	Casp1	Casp1	12362	Caspase-1	no|up	0	ENOG410
Q9D2V5	AAR2_MO	Aar2	Aar2	68295	Protein AA	no|no chan	0	ENOG410
A0A2C9F2	A0A2C9F2	Rin1	Rin1		Ras and Ra	no|no chan	0	ENOG410
Q04447	KCRB_MO	Ckb	Ckb	12709	Creatine ki	no|no chan	0	COG3869(I
P62830	RL23_MOI	Rpl23	Rpl23	65019	Large ribos	no|no chan	0	COG0093(I
P39053	DYN1_MC	Dnm1	Dnm1	13429	Dynamin-1	no|no chan	0	COG0699(I
Q9QZZ6	DERM_MC	Dpt	Dpt	56429	Dermatopo	no|down	0	ENOG4111
Q5SRY7	FBW1B_M	Fbxw11	Fbxw11	103583	F-box/WD	no|no chan	0	ENOG410

P09581	CSF1R_MC	Csf1r	Csf1r	12978	Macrophag	no|no chan	0	COG0515(S
A0A0U1RP	A0A0U1RP	1110004F10	1110004F10Rik		Small acidi	no|up	0	ENOG4111
D3YXP6	D3YXP6_M	Pmvk	Pmvk		Phosphome	no|no chan	0	ENOG4111
P70428	EXT2_MO	Ext2	Ext2	14043	Exostosin-2	no|no chan	0	ENOG410X
P70424	ERBB2_MC	Erbb2	Erbb2	13866	Receptor ty	no|no chan	0	ENOG410X
Q9ERG0	LIMA1_MC	Lima1	Lima1	65970	LIM domai	no|no chan	0	ENOG411C
Q8K2I1	FNTB_MO	Fntb	Fntb	110606	Protein farn	no|no chan	0	COG5029(b
P51829	ADCY7_M	Adcy7	Adcy7	11513	Adenylate c	no|no chan	0	COG2114(F
A0A1W2P7	A0A1W2P7	Ptprk	Ptprk		protein-tyro	no|no chan	0	COG5599(M
Q8K2I3	FMO2_MC	Fmo2	Fmo2	55990	Dimethylar	no|no chan	0	COG2072(b
Q9QYK7	RNF11_MC	Rnf11	Rnf11	29864	RING finge	no|no chan	0	ENOG4112
Q9QYK5	H6ST1_MC	Hs6st1	Hs6st1	50785	Heparan-su	no|no chan	0	ENOG410X
Q3TJL8	Q3TJL8_M	Pdia6	Pdia6	71853	Protein disu	yes|up	1	COG0526(p
A0A1U6UR	A0A1U6UR	Nfat5	Nfat5	54446	Nuclear fac	no|no chan	0	ENOG4111
F2Z433	F2Z433_M	Rad51c	Rad51c		DNA repai	no|no chan	0	COG0468(C
Q9DD24	TCAL9_MC	Tceal9	Tceal9	22381	Transcripti	no|no chan	0	ENOG4111
Q8BWR4	UBP40_MC	Usp40	Usp40	227334	Ubiquitin c	no|no chan	0	COG5077(F
Q8BWR2	PITH1_MC	Pithd1	Pithd1	66193	PITH domo	no|no chan	0	ENOG4111
A0A6I8MW	A0A6I8MW	Msantd2	Msantd2		Myb/SANT	no|no chan	0	ENOG4111
Q8R1N0	ZN830_MC	Znf830	Znf830	66983	Zinc finger	no|no chan	0	ENOG4111
A0A6I8MW	A0A6I8MW	Fndc1	Fndc1		Fibronectin	no|no chan	0	ENOG410Z
Q501J2	ANKMT_M	Antkmt	Antkmt	214917	Adenine nu	no|no chan	0	COG0500(F
P46471	PRS7_MOU	Psmc2	Psmc2	19181	26S proteas	no|no chan	0	COG1222(r
P09055	ITB1_MOU	Itgb1	Itgb1	16412	Integrin bet	no|no chan	0	ENOG410X
A0A5E1	A0A5E1_M	Igfbp7	Igfbp7	29817	Insulin-like	no|down	0	ENOG4111
A0A5E3	A0A5E3_M	LOC100046	LOC100046793		LOC100046	yes|up	1	ENOG4111
A2AWP6	A2AWP6_M	Arhgef10l	Arhgef10l		Rho guanin	no|no chan	0	ENOG410Z
A2AM05	CNTLN_M	Cntln	Cntln	338349	Centlein	no|no chan	0	ENOG410X
A0A087WP	A0A087WP	Atg9a	Atg9a		Autophagy-	no|no chan	0	ENOG410X
Q8C3Y4	KNTC1_M	Kntc1	Kntc1	208628	Kinetochor	no|no chan	0	ENOG410Y
A0A0R4J0R	A0A0R4J0R	Vamp8	Vamp8	22320	Vesicle-ass	no|no chan	0	COG5143(p
A0A0R4J0R	A0A0R4J0R	Isg20l2	Isg20l2	229504	Interferon s	no|no chan	0	COG0847(M
A0A0R4J0R	A0A0R4J0R	Jak3	Jak3	16453	Tyrosine-pr	no|no chan	0	COG0515(p
A0A087WQ	A0A087WQ	Xrn1	Xrn1	24127	5'-3' exoribo	no|no chan	0	COG5049(T
Q9D081	ALG14_MC	Alg14	Alg14	66789	UDP-N-ace	no|up	0	ENOG4111
Q9WTX8	MD1L1_MC	Mad1l1	Mad1l1	17120	Mitotic spir	no|no chan	0	ENOG410Z
Q9WTX2	PRKRA_M	Prkra	Prkra	23992	Interferon-i	no|no chan	0	ENOG410X
Q6KAR6	EXOC3_M	Exoc3	Exoc3	211446	Exocyst cor	no|no chan	0	COG5173(r
D3Z7Q2	SIM20_MO	Smim20	Smim20	66278	Small integ	no|no chan	0	ENOG410Y
Q9CWU2	ZDH13_MC	Zdhhc13	Zdhhc13	243983	Palmitoyltr	no|no chan	0	COG5273(c
A0A494BB2	A0A494BB2	Ptprcap	Ptprcap		Protein tyro	no|up	0	ENOG4111
Q91YV1	Q91YV1_M	Acvrl1	Acvrl1	11482	receptor pro	no|no chan	0	ENOG410X
P26231	CTNA1_MC	Ctnna1	Ctnna1	12385	Catenin alp	no|no chan	0	ENOG410X

Q920G8	MFRN1_M	Slc25a37	Slc25a37	67712; 6746	Mitoferrin-	no | down	0	ENOG410
D3YTQ9	D3YTQ9_M	Rps15	Rps15	20054	Small ribos	no | no chan	0	COG0185(
E9Q309	CE350_MO	Cep350	Cep350	74081	Centrosome	no | no chan	0	ENOG410
Q9ERY9	ERG28_MO	Erg28	Erg28	58520	Ergosterol l	no | no chan	0	ENOG4111
D3YTQ3	D3YTQ3_M	Hnrnpdl	Hnrnpdl	50926	Heterogene	no | down	0	ENOG4111
P63005	LIS1_MOU	Pafah1b1	Pafah1b1	18472	Platelet-act	no | no chan	0	ENOG410
A0A2K6EE	A0A2K6EE	Styx	Styx		Serine/thre	no | no chan	0	COG2453(
Q9CQZ0	ORML2_M	Ormdl2	Ormdl2	66844	ORM1-like	no | no chan	0	COG5081(
P14602	HSPB1_MO	Hspb1	Hspb1	15507	Heat shock	no | up	0	ENOG410
Q31106	Q31106_M	H2-DMa	H2-DMa	14998	H2-M alpha	no | no chan	0	ENOG410
Q99P69	NUF2_MO	Nuf2	Nuf2	66977	Kinetochor	no | no chan	0	ENOG410
A0A0G2JFI	A0A0G2JFI	Ppp1cc	Ppp1cc		Serine/thre	yes | down	1	ENOG410
G5E8R4	G5E8R4_M	Ppp6r3	Ppp6r3	52036	Protein pho	no | no chan	0	ENOG410
Q99P65	S29A3_MO	Slc29a3	Slc29a3	71279	Equilibrati	no | no chan	0	ENOG410
Q6PR54	RIF1_MOU	Rif1	Rif1	51869	Telomere-a	no | no chan	0	ENOG410
G5E8R8	G5E8R8_M	Ubxn7	Ubxn7	224111	UBX doma	no | no chan	0	ENOG410
A2ALA4	A2ALA4_M	Med22	Med22		Mediator of	no | no chan	0	ENOG410
Q99LT0	DPY30_MO	Dpy30	Dpy30	66310	Protein dpy	no | no chan	0	ENOG4112
Q0VBM3	Q0VBM3_M	Prl3c1	Prl3c1	27372	Growth hor	no | no chan	0	ENOG410
Q91V83	TTI1_MOU	Tti1	Tti1	75425	TELO2-inte	no | no chan	0	ENOG410
Q8CFC7	CLASR_MO	Clasrp	Clasrp	53609	CLK4-asso	no | no chan	0	COG0303(
Q3UBU9	Q3UBU9_M	Fkbp3	Fkbp3	30795	peptidylpro	no | no chan	0	COG0545(
F6V8M6	F6V8M6_M	Atxn2	Atxn2		Ataxin 2	yes | up	1	COG5180(
E9PXY8	E9PXY8_M	Usp7	Usp7	252870	Ubiquitin c	no | no chan	0	COG5077(
Q8QZV7	INT13_MO	IntS13	IntS13	71177	Integrator c	no | no chan	0	ENOG410
Q9CZ96	ZCRB1_MO	Zcrb1	Zcrb1	67197	Zinc finger	no | no chan	0	COG0724(
Q6QI06	RICTR_MO	Rictor	Rictor	78757	Rapamycin	no | no chan	0	ENOG410
A0A075B5V	A0A075B5V	Ighv1-54	Ighv1-54		Immunoglo	no | down	0	ENOG4111
Q8VEL2	MTMRE_M	Mtmr14	Mtmr14	97287	Phosphatid	no | no chan	0	ENOG410
Q3U4M0	Q3U4M0_MOUSE	TSC22 domain		78829	TSC22 dom	no | no chan	0	ENOG4112
O09106	HDAC1_M	Hdac1	Hdac1	433759	Histone dec	no | no chan	0	COG0123(
A0A5F8MQ	A0A5F8MQ	Kat6b	Kat6b	54169	histone ace	no | no chan	0	COG5027(
Q7TMW6	CIAO3_MO	Ciao3	Ciao3	67563	Cytosolic ir	no | no chan	0	COG4624(
A0A5F8MQ	A0A5F8MQ	Mrtfa	Mrtfa		Myocardin	no | no chan	0	ENOG411
H3BIU1	H3BIU1_M	Anapc15	Anapc15	75430	Anaphase p	no | no chan	0	ENOG410
Q3UM18	LSG1_MOU	Lsg1	Lsg1	224092	Large subu	no | no chan	0	COG1161(
Q6P5G6	UBXN7_M	Ubxn7	Ubxn7	224111	UBX doma	no | no chan	0	ENOG410
P21956	MFGM_MO	Mfge8	Mfge8	17304	Lactadherin	no | up	0	ENOG4111
O35375	NRP2_MO	Nrp2	Nrp2	18187	Neuropilin-	no | no chan	0	ENOG410
Q9CPX7	RT16_MOU	Mrps16	Mrps16	66242	Small ribos	no | no chan	0	COG0228(
Q9CPX6	ATG3_MO	Atg3	Atg3	67841	Ubiquitin-l	no | no chan	0	ENOG410
A0A3B0IP0	A0A3B0IP0	C1qa	C1qa	12259	Complemer	no | up	0	ENOG410
Q9CR98	F136A_MO	Fam136a	Fam136a	66488	Protein FA	no | no chan	0	ENOG4111

A0A384DV	A0A384DV	Ifnar1	Ifnar1		Interferon (	no|up	0	ENOG4111
Q8VDC1	FYCO1_M(	Fyco1	Fyco1	17281	FYVE and	no|no chan	0	ENOG410X
M0QWF6	M0QWF6_	Macir	Macir		Macrophag	no|no chan	0	ENOG4111
O88952	LIN7C_MC	Lin7c	Lin7c	22343	Protein lin-	no|no chan	0	ENOG410X
F6U2C2	F6U2C2_M	Atxn2	Atxn2		Ataxin 2	no|no chan	0	COG5180(s
O88958	GNPI1_M(	Gnpda1	Gnpda1	26384	Glucosamii	no|no chan	0	COG0363(c
Q9QXD8	LIMD1_M(	Limd1	Limd1	29806	LIM domai	no|no chan	0	ENOG410Y
Q6PCZ4	MAGE1_M	Magee1	Magee1	107528	Melanoma-	no|no chan	0	ENOG4111
Q9QZK2	BCAR3_M(	Bcar3	Bcar3	29815	Breast canc	no|up	0	ENOG410X
A0A5F8MP	A0A5F8MP	Ptk2	Ptk2		Focal adhes	no|no chan	0	ENOG410Z
H7BX49	H7BX49_M	Wdr90	Wdr90	106618	WD repeat	no|no chan	0	ENOG410X
Q3UHC4	Q3UHC4_M	Kif3b	Kif3b	16569	Kinesin-like	no|no chan	0	COG5059(5
Q8R2P1	KLH25_M(	Klhl25	Klhl25	207952	Kelch-like	no|no chan	0	ENOG410X
Q3UHC2	LRRK1_M(	Lrrk1	Lrrk1	233328	Leucine-ric	no|no chan	0	COG4886(s
Q03402	CRIS3_MO	Crisp3	Crisp3	11572	Cysteine-ric	no|no chan	0	COG2340(s
Q5M8N4	D39U1_M(	Sdr39u1	Sdr39u1	654795	Epimerase	no|no chan	0	COG1090(I
Q64444	CAH4_MO	Ca4	Ca4	12351	Carbonic a	no|down	0	COG3338(I
A0A1W2P8	A0A1W2P8	Csrp2	Csrp2	13008	Cysteine an	no|no chan	0	ENOG4111
E9PYU8	E9PYU8_M	Smurf1	Smurf1	75788	E3 ubiquiti	no|no chan	0	COG5021(I
Q9CQ19	MYL9_MO	Myl9	Myl9	98932	Myosin regi	no|down	0	COG5126(S
Q8CCX9	Q8CCX9_M	Mrpl39	Mrpl39	27393	TGS domai	no|no chan	0	COG0441(c
Q8K1K1	Q8K1K1_M	Gemin4	Gemin4	276919	Gem (Nucl	no|no chan	0	ENOG410X
P15261	INGR1_M(	Ifngr1	Ifngr1	15979	Interferon g	no|up	0	ENOG4112
Q78S06	SYS1_MOU	Sys1	Sys1	66460	Protein SYS	no|up	0	ENOG4111
Q9CQ66	DYT2B_M(	Dynlt2b	Dynlt2b	66061	Dynein ligh	no|no chan	0	ENOG4111
Q99LS3	SERB_MOU	Psph	Psph	100678	Phosphoser	no|no chan	0	COG0560(p
Q3TH01	Q3TH01_M	H2-K1	H2-K1	14972	Histocomp	no|no chan	0	ENOG4111
Q3TH04	Q3TH04_M	Hspa8	Hspa8	15481	Heat shock	no|no chan	0	COG0443(c
Q91WL8	WWOX_M	Wwox	Wwox	80707	WW domai	no|no chan	0	COG5021(u
A0A494B9Z	A0A494B9Z	Bbs1	Bbs1		Bardet-Biec	no|no chan	0	ENOG410Y
Q80X82	SYMPK_M	Sympk	Sympk	68188	Symplekin	no|no chan	0	ENOG410X
P58196	PLS4_MOU	Plscr4	Plscr4	235527	Phospholip	no|no chan	0	ENOG410X
O35594	IFT81_MO	Ift81	Ift81	12589	Intraflagell	no|no chan	0	ENOG410X
O35593	PSDE_MO	Psmd14	Psmd14	59029	26S proteas	no|no chan	0	COG1310(r
E9Q8J5	E9Q8J5_M(	Spata31e5	Spata31e5	210962	Predicted g	no|up	0	
O70126	AURKB_M	Aurkb	Aurkb	20877	Aurora kinc	no|no chan	0	ENOG410X
A0A1L1SQ	A0A1L1SQ	Ate1	Ate1		Arginyl-tRN	yes|up	1	COG2935(M
O35598	ADA10_M(	Adam10	Adam10	11487	Disintegrin	no|no chan	0	ENOG410X
E9Q1R7	E9Q1R7_M	Tiam1	Tiam1	21844	T cell lymp	no|no chan	0	ENOG410X
A0A0B6VS(	A0A0B6VS(	Klk1	Klk1	16612	Kallikrein 1	no|up	0	COG5640(p
Q9CX86	ROA0_MO	Hnrnpa0	Hnrnpa0	77134	Heterogene	no|no chan	0	ENOG410Y
A0A2I3BPS	A0A2I3BPS	Cdkn3	Cdkn3	72391	protein-tyrc	yes|down	1	COG2453(I
A0A2I3BPS	A0A2I3BPS	Otulinl	Otulinl	223433	OTU deubi	no|no chan	0	ENOG410Y

Q9Z2I8	SUCB2_MC	Suclg2	Suclg2	20917	Succinate--(	no | no chan	0	COG0045(I
E9QNY8	E9QNY8_M	Sacs	Sacs		Sacsin	no | no chan	0	ENOG410X
A0A1D5RL	A0A1D5RL	Il12rb1	Il12rb1	16161	Interleukin	no | no chan	0	ENOG410Y
Q8VBY2	KKCC1_M	Camkk1	Camkk1	55984	Calcium/cc	no | no chan	0	ENOG410Y
Q3USR5	Q3USR5_M	Fbxo2	Fbxo2	230904	F-box only	no | no chan	0	ENOG4111
Q8BVQ5	PPME1_M(	Ppme1	Ppme1	72590	Protein pho	no | no chan	0	COG0596(S
Q6NZR5	SKI2_MOU	Skic2	Skic2	108077	Superkiller	no | no chan	0	COG4581(I
Q3U047	Q3U047_M	Pigt	Pigt	78928	GPI transa	no | no chan	0	ENOG410X
Q60710	SAMH1_M	Samhd1	Samhd1	56045	Deoxynucle	no | up	0	COG1078(M
Q3USR8	Q3USR8_M	Ctcf	Ctcf	13018	C2H2-type	no | no chan	0	COG5048(I
Q60715	P4HA1_MC	P4ha1	P4ha1	18451	Prolyl 4-hy	no | up	0	ENOG410X
P04627	ARAF_MO	Araf	Araf	11836	Serine/thre	no | no chan	0	ENOG410Y
A0A338P6I	A0A338P6I	Gm56451	Gm56451		Predicted g	no | no chan	0	
K7TDG8	K7TDG8_MOUSE	IgM heavy chain variable r			IgM heavy	no | up	0	ENOG4111
Q61599	GDIR2_MC	Arhgdib	Arhgdib	11857	Rho GDP-c	yes | up	1	ENOG4111
A0A1L1SSV	A0A1L1SSV	Taok2	Taok2		non-specifi	no | no chan	0	ENOG410Y
Q61592	GAS6_MO	Gas6	Gas6	14456	Growth arr	no | no chan	0	ENOG410Z
Q8CG19	LTBP1_MC	Ltbp1	Ltbp1	268977	Latent-tran	no | no chan	0	ENOG410Z
P51125	ICAL_MOU	Cast	Cast	12380	Calpastatin	yes | up	1	ENOG4111
Q61043	NIN_MOU	Nin	Nin	18080	Ninein	no | no chan	0	ENOG410Z
Q61595	KTN1_MO	Ktn1	Ktn1	16709	Kinectin	no | no chan	0	ENOG410Y
G5E861	SCLT1_MC	Sclt1	Sclt1	67161	Sodium cha	no | no chan	0	ENOG4111
Q99PN3	TRI26_MO	Trim26	Trim26	22670	Tripartite n	no | no chan	0	ENOG410Z
Q3UFR4	Q3UFR4_M	Slc1a5	Slc1a5	20514	Amino acid	no | down	0	COG1301(s
Q8BP00	IQCB1_MC	Iqcb1	Iqcb1	320299	IQ calmodu	no | no chan	0	COG5022(V
Q5SV54	Q5SV54_M	Pard3b	Pard3b	72823	Ortholog of	no | no chan	0	ENOG410Z
Q8K2H2	OTU6B_M	Otud6b	Otud6b	72201	Deubiquiti	no | no chan	0	COG5539(c
P13634	CAH1_MO	Ca1	Ca1	12346	Carbonic a	no | no chan	0	COG3338(c
A0A0U1RN	A0A0U1RN	Dock7	Dock7	67299	Dedicator c	no | no chan	0	ENOG410X
Q8K2H4	ACAP1_MC	Acap1	Acap1	216859	Arf-GAP w	no | no chan	0	COG5347(c
Q8VC70	RBMS2_MC	Rbms2	Rbms2	56516	RNA-bindi	no | no chan	0	ENOG410X
C5H7S3	C5H7S3_M	Abt1	Abt1	30946	Activator o	no | no chan	0	ENOG4111
P35288	RAB23_MC	Rab23	Rab23	19335	Ras-related	no | no chan	0	ENOG411C
Q9CZA6	NDE1_MO	Nde1	Nde1	67203	Nuclear dis	no | no chan	0	ENOG410X
Q544C3	Q544C3_M	Stard3	Stard3	59045	StAR-relate	no | no chan	0	ENOG411C
P35282	RAB21_MC	Rab21	Rab21	216344	Ras-related	no | no chan	0	ENOG410X
Q569X8	Q569X8_M	Nup107	Nup107	103468	Nuclear po	no | no chan	0	ENOG410X
Q544C9	Q544C9_M	Lsm1	Lsm1	67207	U6 snRNA-	no | no chan	0	ENOG4112
P35285	RB22A_MC	Rab22a	Rab22a	19334	Ras-related	no | no chan	0	ENOG410Y
D3YWX2	D3YWX2_I	Ylpm1	Ylpm1	56531	YLP motif c	no | no chan	0	ENOG410Y
P05214	TBA3_MOU	Tuba3a	Tuba3a	22144; 2214	Tubulin alp	no | no chan	0	COG5023(c
A0A1Y7VM	A0A1Y7VM	Rock2	Rock2		Rho-associc	no | no chan	0	ENOG410X
G3X8Y3	G3X8Y3_M	Naa15	Naa15	74838	N(alpha)-ac	no | no chan	0	ENOG410X

P05213	TBA1B_MO	Tuba1b	Tuba1b	22143	Tubulin alp	no|no chan	0	COG5023(c
Q8BHZ0	CYRIA_MO	Cyria	Cyria	76820	CYFIP-rela	no|no chan	0	ENOG410X
G3X8Y8	G3X8Y8_M	Tlr2	Tlr2	24088	Toll-like rec	no|no chan	0	COG4886(C
Q8BR63	F177A_MO	Fam177a1	Fam177a1	73385; 1001	Protein FAI	no|no chan	0	ENOG4112
Q8VDS4	RPR1A_MO	Rprd1a	Rprd1a	225283	Regulation	no|no chan	0	ENOG410X
P28798	GRN_MOU	Grn	Grn	14824	Progranulin	no|no chan	0	ENOG410X
Q9D9V3	ECHD1_M	Echdc1	Echdc1	52665	Ethylmalon	no|no chan	0	COG1024(r
Q8VDS8	STX18_MO	Stx18	Stx18	71116	Syntaxin-18	no|no chan	0	ENOG410X
Q3UGC7	EI3JA_MO	Eif3j1	Eif3j1	78655	Eukaryotic	no|no chan	0	ENOG4111
A0AA74KU	A0AA74KU	Kctd9	Kctd9		Potassium c	no|up	0	COG1357(p
Q9DB30	PHKG2_M	Phkg2	Phkg2	68961	Phosphoryl	no|no chan	0	ENOG410Y
A0AA74KU	A0AA74KU	Tnks2	Tnks2		Poly [ADP-	no|no chan	0	COG0666(p
Q3UGC1	Q3UGC1_M	Strip1	Strip1	229707	Striatin-int	no|no chan	0	ENOG410X
Q9DB34	CHM2A_M	Chmp2a	Chmp2a	68953	Charged mu	yes|up	1	COG5491(c
Q3UGC8	Q3UGC8_M	Pcca	Pcca	110821	Propionyl-C	no|no chan	0	COG4770(C
Q99M95	Q99M95_M	St7	St7	64213	ST7 protein	no|no chan	0	ENOG410X
Q62245	SOS1_MOU	Sos1	Sos1	20662	Son of seven	no|no chan	0	ENOG410X
A0A0N4SV	A0A0N4SV	Dysf	Dysf	26903	Dysferlin	no|no chan	0	ENOG410X
Q3U590	Q3U590_M	C2cd2l	C2cd2l	71764	C2 domain-	no|no chan	0	ENOG410Z
Q5SWD9	TSR1_MOU	Tsr1	Tsr1	104662	Pre-rRNA-p	no|no chan	0	COG5177(T
Q8BJL1	FBX30_MO	Fbxo30	Fbxo30	71865	F-box only	no|no chan	0	ENOG410X
A0A1L1SV	A0A1L1SV	Parp16	Parp16		Poly [ADP-	no|no chan	0	ENOG410Z
P32883	RASK_MO	Kras	Kras	16653	GTPase KR	no|no chan	0	COG1100(v
D3Z198	D3Z198_M	Mrps17	Mrps17		Mitochondr	no|no chan	0	ENOG4111
A0A0J9YTV	A0A0J9YTV	Rbpj	Rbpj	19664	Recombina	no|no chan	0	ENOG410X
D3Z197	D3Z197_M	Nsd3	Nsd3		Nuclear rec	no|no chan	0	COG2940(C
F7DF62	F7DF62_M	Arrb1	Arrb1		Arrestin, be	no|up	0	ENOG410X
A0A023J6F	A0A023J6F	COX2	COX2		Cytochrom	no|no chan	0	COG1622(S
A0A068BEI	A0A068BEI	Angptl4	Angptl4	57875	Angiopoieti	no|up	0	ENOG410Z
A0A068BEI	A0A068BEI	Daxx	Daxx	13163	Death dom	no|no chan	0	ENOG4111
C0LLJ0	IMA8_MOU	Kpna7	Kpna7	381686	Importin su	yes|down	1	COG5064(T
A0A0G2JE	A0A0G2JE	Sh3glb1	Sh3glb1		Endophilin	no|no chan	0	
A0A1I7Q4	A0A1I7Q4	Cenpq	Cenpq	83815	Centromere	no|no chan	0	ENOG410Y
A0A2R8VH	A0A2R8VH	Tns2	Tns2		Tensin 2	no|no chan	0	ENOG410Y
Q91VW5	GOGA4_M	Golga4	Golga4	54214	Golgin sub	no|no chan	0	ENOG410X
Q3TNK3	Q3TNK3_M	Gpx7	Gpx7	67305	Glutathion	no|no chan	0	COG0386(C
Q3U319	BRE1B_MO	Rnf40	Rnf40	233900	E3 ubiquiti	no|no chan	0	ENOG410Y
Q7TQ95	LNP_MOU	Lnpk	Lnpk	69605	Endoplasm	no|no chan	0	ENOG4111
Q91VW3	SH3L3_MO	Sh3bgrl3	Sh3bgrl3	73723	SH3 domain	no|no chan	0	ENOG4111
Q59J92	Q59J92_MO	Galnt17	Galnt17	212996	Polypeptide	no|up	0	ENOG410X
Q3U5H9	Q3U5H9_M	Rrp7a	Rrp7a	74778	Ribosomal	no|no chan	0	ENOG410Y
Q5EBP9	Q5EBP9_M	Trim28	Trim28	21849	Tripartite m	no|no chan	0	ENOG411C
Q5RIM6	Q5RIM6_M	Ncor1	Ncor1	20185	Nuclear rec	no|no chan	0	ENOG410Y

Q5EBP3	ARMC5_M	Armc5	Armc5	233912	Armadillo r	no | no chan	0	ENOG410>
A4FUS1	A4FUS1_M	Rps16	Rps16	20055; 1000	Small ribos	no | no chan	0	COG0103(t
B2RSV4	B2RSV4_M	Sf3b3	Sf3b3	101943	Splicing fac	no | no chan	0	ENOG410>
E9PY85	E9PY85_M	Rsl24d1	Rsl24d1		Probable ri	no | no chan	0	COG2075(F
Q8BGB4	Q8BGB4_M	Bcam	Bcam	57278	Ig-like dom	no | no chan	0	ENOG4111
P30306	MPIP2_MC	Cdc25b	Cdc25b	12531	M-phase in	no | no chan	0	COG5105(c
B7ZNH7	B7ZNH7_M	Col14a1	Col14a1	12818	Collagen, ty	no | down	0	ENOG410Y
F6YC79	F6YC79_M	Lmf1	Lmf1		Lipase mat	no | no chan	0	ENOG410>
E9QKL0	E9QKL0_M	Rad54l2	Rad54l2	81000	RAD54 like	no | no chan	0	COG0553(r
Q99P31	HPBP1_MC	Hspbp1	Hspbp1	66245	Hsp70-bind	no | no chan	0	ENOG410Y
Q9QZA0	CAH5B_MC	Ca5b	Ca5b	56078	Carbonic an	no | down	0	COG3338(c
P49182	HEP2_MO	Serpind1	Serpind1	15160	Heparin co	no | up	0	COG4826(t
Q9D0I9	SYRC_MO	Rars1	Rars1	104458	Arginine--t	no | no chan	0	COG0018(c
Q9D0I6	WSDU1_M	Wdsub1	Wdsub1	72137	WD repeat,	no | no chan	0	ENOG410>
Q9D0I4	STX17_MC	Stx17	Stx17	67727	Syntaxin-17	no | no chan	0	COG5325(S
Q80ZW2	THEM6_M	Them6	Them6	223626	Protein TH	no | up	0	ENOG4111
Q3ULN7	Q3ULN7_M	Dazap2	Dazap2	23994	DAZ-assoc	no | down	0	ENOG4111
B0LAA9	B0LAA9_M	Pgk1	Pgk1	18655	Phosphogly	no | no chan	0	COG0126(p
A0A0G2JD	A0A0G2JD	Rabggtb	Rabggtb		Rab gerany	no | no chan	0	COG5029(I
A0A0G2JD	A0A0G2JD	Mef2c	Mef2c		Myocyte en	no | down	0	COG5068(i
A2AM29	AF9_MOUS	Mllt3	Mllt3	70122	Protein AF-	yes | up	1	COG5033(r
A0A140T8V	A0A140T8V	Col6a5	Col6a5	665033	Collagen, ty	no | down	0	ENOG410Y
Q8R2H9	PHOP1_MC	Phospho1	Phospho1	237928	Phosphoeth	no | up	0	ENOG4111
Q6ZWX6	IF2A_MOU	Eif2s1	Eif2s1	13665	Eukaryotic	no | no chan	0	COG1093(t
Q8K3W3	CASC3_MC	Casc3	Casc3	192160	Protein CAS	no | no chan	0	ENOG410Z
P25799	NFKB1_MC	Nfkb1	Nfkb1	18033	Nuclear fac	no | no chan	0	COG0666(I
F7BQE4	F7BQE4_M	Arhgef10	Arhgef10		Rho guanin	no | no chan	0	ENOG410Z
P49452	CENPC_M	Cenpc	Cenpc	12617	Centromere	no | no chan	0	ENOG4112
Q8VI75	IPO4_MOU	Ipo4	Ipo4	75751	Importin-4	no | no chan	0	ENOG410>
A0A1R3UF	A0A1R3UF	Klna3	Klna3		Kallikrein c	no | up	0	COG5640(p
Q66JV6	Q66JV6_M	Orc6	Orc6	56452	Origin reco	no | no chan	0	ENOG4111
Q812G0	MGT4A_M	Mgat4a	Mgat4a	269181	Alpha-1,3-n	no | no chan	0	ENOG410>
Q3UF10	Q3UF10_M	Ogfr	Ogfr	72075	Opioid grov	no | no chan	0	ENOG4111
A2A4B1	A2A4B1_M	Lpin3	Lpin3		phosphatid	no | no chan	0	COG5083(N
G5E890	G5E890_M	Rfx3	Rfx3	19726	Transcripti	no | no chan	0	ENOG410>
G5E896	G5E896_M	Edc4	Edc4	234699	Enhancer o	no | no chan	0	ENOG410>
G5E897	PLGT3_MC	Poglut3	Poglut3	68304	Protein O-g	no | no chan	0	ENOG410>
G5E895	G5E895_M	Akr1b10	Akr1b10	67861	Aldo-keto r	yes | up	1	COG0656(T
G5E898	G5E898_M	Ppl	Ppl	19041	Periplakin	no | no chan	0	ENOG410Y
Q99PQ2	TRI11_MO	Trim11	Trim11	94091	E3 ubiquiti	no | no chan	0	ENOG410Y
Q8BM85	TBCK_MO	Tbck	Tbck	271981	TBC domai	no | no chan	0	ENOG410Y
Q6PGC1	DHX29_MC	Dhx29	Dhx29	218629	ATP-depen	no | no chan	0	COG1643(I
Q91WC9	DGLB_MC	Daglb	Daglb	231871	Diacylglyce	no | no chan	0	ENOG410>

Q99J10	CTU1_MO'	Ctu1	Ctu1	233189	Cytoplasmi	no|no chan	0	COG0037(I
D6RGR4	D6RGR4_M	Wdr83	Wdr83		WD repeat	no|no chan	0	ENOG410)
Q91Z31	PTBP2_MC	Ptbp2	Ptbp2	56195	Polypyrimi	no|no chan	0	ENOG410)
Q9D1E6	TBCB_MO'	Tbcb	Tbcb	66411	Tubulin-fol	no|no chan	0	ENOG410Y
G3X9Z0	G3X9Z0_M	Zwilch	Zwilch	68014	Protein zwi	no|no chan	0	ENOG410Y
G3X9Z4	PCF11_MC	Pcf11	Pcf11	74737	Pre-mRNA	no|no chan	0	ENOG410Y
Q8C9B9	DIDO1_M(	Dido1	Dido1	23856	Death-indu	no|no chan	0	ENOG411(
Q80XM9	S66A2_MO	Slc66a2	Slc66a2	66943	Solute carri	no|no chan	0	ENOG4111
F6WJB7	F6WJB7_M	Usp34	Usp34		ubiquitinyl	no|no chan	0	COG5077(M
D3YZP9	CCDC6_M(	Ccdc6	Ccdc6	76551	Coiled-coil	no|no chan	0	ENOG410)
H3BK50	H3BK50_M	Ppp2r1b	Ppp2r1b		Protein phc	no|up	0	ENOG410)
P63213	GBG2_MO	Gng2	Gng2	14702	Guanine nu	no|no chan	0	ENOG4112
B2RY56	RBM25_M(	Rbm25	Rbm25	67039	RNA-bindi	no|no chan	0	ENOG410Z
Q6P4T3	Q6P4T3_M	Eya3	Eya3	14050	Eyes absent	no|no chan	0	ENOG410)
Q6P4T2	U520_MOU	Snrnp200	Snrnp200	320632	U5 small nu	no|no chan	0	COG1204(I
Q6P4T1	SNX19_MC	Snx19	Snx19	102607	Sorting nex	no|no chan	0	ENOG410)
B2RY59	B2RY59_M	Neurl4	Neurl4	216860	Neurl4 prot	no|no chan	0	ENOG410)
G5E8C4	G5E8C4_M	Tmtc3	Tmtc3	237500	dolichyl-ph	no|no chan	0	COG0457(T
D3Z2E3	D3Z2E3_M	Reps1	Reps1		RalBP1 ass	no|no chan	0	ENOG410Z
Q54AE3	Q54AE3_M	Gnb3	Gnb3	14695	GTP-bindir	no|down	0	ENOG410)
O88852	TSYL1_MC	Tspyl1	Tspyl1	22110	Testis-speci	no|down	0	ENOG410)
D3Z2E8	D3Z2E8_M	Calhm2	Calhm2		Calcium ho	no|no chan	0	ENOG410)
Q3TCJ1	ABRX2_M(	Abraxas2	Abraxas2	109359	BRISC com	no|no chan	0	ENOG410Z
Q3TCJ7	Q3TCJ7_M	Samhd1	Samhd1	56045	Deoxynucle	no|no chan	0	COG1078(M
Q3TCJ8	CCD69_M(	Ccdc69	Ccdc69	52570	Coiled-coil	no|no chan	0	ENOG4111
Q91YD3	DCP1A_M(	Dcp1a	Dcp1a	75901	mRNA-deca	no|no chan	0	ENOG410)
G5E8C2	G5E8C2_M	Kbtbd7	Kbtbd7	211255	Kelch repea	no|down	0	ENOG411(
A2A716	A2A716_M(	Osbpl7	Osbpl7		Oxysterol-b	no|no chan	0	ENOG410)
G5E8C3	G5E8C3_M	Gprc5a	Gprc5a	232431	G protein-c	no|no chan	0	ENOG410Y
E9Q3M3	E9Q3M3_M	Dctn1	Dctn1	13191	Dynactin su	no|no chan	0	COG5244(i
Q80XC2	TRM61_M(	Trmt61a	Trmt61a	328162	tRNA (ader	no|no chan	0	COG2519(t
Q80XC3	US6NL_M(	Usp6nl	Usp6nl	98910	USP6 N-ter	no|no chan	0	COG5210(t
Q9JJL8	SYSM_MO'	Sars2	Sars2	71984	Serine--tRN	no|no chan	0	COG0172(c
B9EKE7	B9EKE7_M	Tmed8	Tmed8	382620	Tmed8 prot	no|no chan	0	ENOG410)
Q80XC6	NRDE2_M	Nrde2	Nrde2	217827	Nuclear exc	no|no chan	0	ENOG410)
P47878	IBP3_MOU	Igfbp3	Igfbp3	16009	Insulin-like	no|down	0	ENOG4111
O35744	CHIL3_MC	Chil3	Chil3	12655	Chitinase-li	no|up	0	COG3325(E
B2RXS4	PLXB2_M(	Plxnb2	Plxnb2	140570	Plexin-B2	no|no chan	0	ENOG4111
O35295	PURB_MO	Purb	Purb	19291	Transcripti	no|no chan	0	ENOG410Z
B1AWL2	ZN462_MC	Znf462	Znf462	242466	Zinc finger	no|no chan	0	COG5048(u
Q6PD03	2A5A_MOU	Ppp2r5a	Ppp2r5a	226849	Serine/thre	no|no chan	0	ENOG410)
O55033	NCK2_MO	Nck2	Nck2	17974	Cytoplasmi	no|no chan	0	ENOG410)
F8WI18	F8WI18_M(	Pip5k1a	Pip5k1a		Phosphatid	no|no chan	0	COG5253(S

Q8C8R3	ANK2_MO	Ank2	Ank2	109676	Ankyrin-2	no	no chan	0	COG0666(r
D6RH99	D6RH99_M	Tmem50b	Tmem50b		Transmemb	no	no chan	0	ENOG410Y
Q3U1S6	Q3U1S6_M	Csde1	Csde1	229663	Cold shock	no	no chan	0	ENOG410Z
Q3TN34	MILK2_MC	Micall2	Micall2	231830	MICAL-like	no	no chan	0	COG5069(t
O35126	ATN1_MO	Atn1	Atn1	13498	Atrophin-1	no	no chan	0	ENOG410Z
O35127	C10_MOUS	Grcc10	Grcc10	14790	Protein C10	no	no chan	0	ENOG4111
Q5DU41	LRC8B_MC	Lrrc8b	Lrrc8b	433926	Volume-reg	no	no chan	0	COG4886(Z
Q52L66	Q52L66_M	Ubl7	Ubl7	69459	Ubiquitin-l	no	no chan	0	ENOG410Y
Q9CPV7	ZDHC6_M	Zdhhc6	Zdhhc6	66980	Palmitoyltr	no	no chan	0	COG5273(Z
E9PUA5	E9PUA5_M	Kifc5b	Kifc5b	16580	Kinesin-like	no	no chan	0	COG5059(c
E9PUA2	E9PUA2_M	Tut7	Tut7	214290	Terminal u	no	no chan	0	COG5260(c
O35129	PHB2_MO	Phb2	Phb2	12034	Prohibitin-2	no	no chan	0	COG0330(l
Q8BH57	WDR48_M	Wdr48	Wdr48	67561	WD repeat-	no	no chan	0	ENOG410X
Q8BH58	TIPRL_MC	Tiprl	Tiprl	226591	TIP41-like p	no	no chan	0	ENOG410X
Q8BH59	S2512_MO	Slc25a12	Slc25a12	78830	Electrogeni	no	no chan	0	ENOG410X
Q05C56	Q05C56_M	Add3	Add3	27360	Add3 protei	no	no chan	0	COG0235(c
Q91XB0	TREX1_MC	Trex1	Trex1	22040	Three-prim	no	no chan	0	ENOG4111
Q3TWT2	Q3TWT2_M	Memo1	Memo1	76890	Protein ME	no	no chan	0	COG1355(U
Q3U254	Q3U254_M	Emilin1	Emilin1	100952	EMILIN-1	no	down	0	ENOG4111
Q69ZA1	CDK13_MC	Cdk13	Cdk13	69562	Cyclin-depe	no	no chan	0	ENOG410X
Q99LN9	DOHH_MC	Dohh	Dohh	102115	Deoxyhypu	no	no chan	0	COG1413(C
P70196	TRAF6_MC	Traf6	Traf6	22034	TNF recept	no	no chan	0	ENOG410X
P70195	PSB7_MOU	Psmb7	Psmb7	19177	Proteasome	no	no chan	0	COG0638(T
Q8BTR5	DUS28_MC	Dusp28	Dusp28	67446	Dual specif	no	up	0	COG2453(c
P70191	TRAF5_MC	Traf5	Traf5	22033	TNF recept	no	no chan	0	ENOG410Z
A2AQD5	A2AQD5_M	Itprid2	Itprid2		ITPR interc	no	no chan	0	ENOG410Z
A2AVP4	A2AVP4_M	Rc3h2	Rc3h2	319817	RING-type	no	no chan	0	ENOG410Y
Q4TVN0	Q4TVN0_M	Tbc1d15	Tbc1d15	66687	TBC1 domc	no	no chan	0	COG5210(T
D3YV13	D3YV13_M	Dtx3	Dtx3		E3 ubiquiti	no	no chan	0	ENOG410Z
A0ELI5	A0ELI5_M	Edc3	Edc3	353190	Enhancer o	no	no chan	0	COG0062(N
A7YB12	A7YB12_M	Ankrd54	Ankrd54	223690	Lyn-interac	no	no chan	0	COG0666(g
Q9EPK7	XPO7_MO	Xpo7	Xpo7	65246	Exportin-7	no	no chan	0	ENOG410X
Q00547	HMMR_MC	Hmmr	Hmmr	15366	Hyaluronar	no	no chan	0	ENOG4111
S4R2I9	S4R2I9_MC	Zfp532	Zfp532		Zinc finger	no	no chan	0	COG5048(C
Q5SX74	Q5SX74_M	P4ha2	P4ha2	18452	procollagen	no	up	0	ENOG410X
Q01149	CO1A2_MC	Col1a2	Col1a2	12843	Collagen al	no	no chan	0	ENOG410X
P62855	RS26_MOU	Rps26	Rps26	27370	Small ribos	no	no chan	0	COG4830(F
A0A140LIF	IRGM2_MC	Irgm2	Irgm2	54396	Immunity-r	no	up	0	ENOG4112
Q3TNH0	Q3TNH0_M	Tmpo	Tmpo	21917	Thymopoie	no	no chan	0	ENOG4111
Q01147	CREB1_MC	Creb1	Creb1	12912	Cyclic AMF	no	no chan	0	ENOG410Z
Q3U0S1	Q3U0S1_M	Parp12	Parp12	243771	Poly [ADP-	no	no chan	0	ENOG410Z
A0A0U1RP	A0A0U1RP	Atxn2l	Atxn2l	233871	Ataxin 2-lik	no	up	0	COG5180(V
A0A3Q4EF	A0A3Q4EF	Rnf138	Rnf138		E3 ubiquiti	no	up	0	ENOG410Y

P70441	NHRF1_M(	Nherf1	Nherf1	26941	Na(+)/H(+	no|no chan	0	ENOG411(
P70445	4EBP2_MC	Eif4ebp2	Eif4ebp2	13688	Eukaryotic	no|no chan	0	ENOG4112
P70444	BID_MOU:	Bid	Bid	12122	BH3-intera(	no|no chan	0	ENOG410Z
Q80W37	SPN1_MOU	Snupn	Snupn	66069	Snurportin-	no|no chan	0	ENOG410X
E9Q5M3	E9Q5M3_M	S100pbp	S100pbp		S100P-bind	no|no chan	0	ENOG410X
Q7TNP2	2AAB_MO	Ppp2r1b	Ppp2r1b	73699	Serine/thre	no|no chan	0	ENOG410X
Q9QYE6	GOGA5_M	Golga5	Golga5	27277	Golgin sub	no|no chan	0	ENOG410X
Q8BWP8	B4GA1_M(	B4gat1	B4gat1	108902	Beta-1,4-gl	no|no chan	0	ENOG410X
Q925B0	PAWR_MC	Pawr	Pawr	114774	PRKC apop	no|no chan	0	ENOG4111
Q8VCX5	MICU1_M(	Micu1	Micu1	216001	Calcium up	no|no chan	0	ENOG410Z
Q80U95	UBE3C_M(	Ube3c	Ube3c	100763	Ubiquitin-p	no|no chan	0	COG5021(S
Q80U93	NU214_MC	Nup214	Nup214	227720	Nuclear po	no|no chan	0	ENOG410Y
Q9DCD9	Q9DCD9_MOUSE	Ig-like domain-		16061	Ig-like dom	no|up	0	ENOG410Z
Q8BT07	CEP55_MC	Cep55	Cep55	74107	Centrosom(	no|no chan	0	ENOG4112
Q9DCD5	TJAP1_MO	Tjap1	Tjap1	74094	Tight juncti	no|no chan	0	ENOG410Z
Q8CBY1	SMAG1_M	Samd4a	Samd4a	74480	Protein Sm(	no|no chan	0	ENOG410X
Q8CBY0	GATC_MC	Gatc	Gatc	384281	Glutamyl-t	no|no chan	0	ENOG4111
Q8BVJ1	Q8BVJ1_M	Rxylt1	Rxylt1	216395	Transmemb	no|down	0	ENOG410Y
P27659	RL3_MOU:	Rpl3	Rpl3	27367	Large ribos	no|no chan	0	COG0087(X
P28301	LYOX_MC	Lox	Lox	16948	Protein-lysi	no|no chan	0	ENOG411(
A0A494BB/	A0A494BB/	Fau	Fau		FAU ubiqu	no|no chan	0	ENOG4112
A0A0G2JG	A0A0G2JG	Zgrf1	Zgrf1		Zinc finger,	no|no chan	0	COG1112(i
Q9DBX3	SUSD2_MC	Susd2	Susd2	71733	Sushi doma	no|no chan	0	ENOG410Y
Q9DBX1	RGCC_MC	Rgcc	Rgcc	66214	Regulator c	no|no chan	0	ENOG4111
Q9CQI9	MED30_M(	Med30	Med30	69790	Mediator o	no|no chan	0	ENOG411(
E9Q9G8	E9Q9G8_M	Ift122	Ift122	81896	Intraflagell	no|no chan	0	COG2319(I
Q8C3W1	CA198_MOUSE	Uncharacterized p		69551	Uncharacte	no|no chan	0	ENOG4111
B8JJ84	B8JJ84_MO	Stxbp6	Stxbp6		Syntaxin bi	no|no chan	0	ENOG410Z
Q9WTZ0	UXT_MOU	Uxt	Uxt	22294	Protein UX	no|no chan	0	ENOG4111
Q3U1Z8	Q3U1Z8_M	Ubr7	Ubr7	66622	UBR-type d	no|down	0	ENOG410X
D3YUI6	D3YUI6_M	Cfap298	Cfap298		Cilia and fl	no|no chan	0	ENOG410X
A0A0A6YW	A0A0A6YW	Flvcr1	Flvcr1	226844	Feline leuk(	no|no chan	0	ENOG410X
B2RVL4	B2RVL4_M	Bbc3	Bbc3	170770	BCL2 bindi	no|down	0	ENOG4111
Q546F8	Q546F8_M	Exosc8	Exosc8	69639	Ribosomal	no|no chan	0	COG2123(S
O08692	NGP_MOU	Ngp	Ngp	18054	Neutrophil	no|up	0	ENOG4111
A0A494BB4	A0A494BB4	Cd5	Cd5		CD5 antige	no|down	0	ENOG4111
P19157	GSTP1_MC	Gstp1	Gstp1	14870	Glutathion	no|no chan	0	ENOG4111
Q5SS55	Q5SS55_M(	Ehbp1	Ehbp1		EH domain	no|no chan	0	COG5069(N
B1ART1	B1ART1_M	Vps13d	Vps13d		Vacuolar p	no|no chan	0	COG5043(V
Q8R0F3	SUMF1_M(	Sumf1	Sumf1	58911	Formylglyci	no|no chan	0	COG1262(S
D3YTS3	D3YTS3_M	D630045J12	D630045J12Rik		RIKEN cD	no|no chan	0	ENOG410X
B1AXD8	AKIR2_MC	Akirin2	Akirin2	433693	Akirin-2	no|no chan	0	ENOG4111
D3YTS1	D3YTS1_M	Sec11a	Sec11a	56529	Signal pept	no|no chan	0	COG0681(I

Q8R0F6	ILKAP_MO	Ilkap	Ilkap	67444	Integrin-lin	no | no chan	0	COG0631(p
Q8R0F8	FAHD1_M	Fahd1	Fahd1	68636	Oxaloaceta	no | no chan	0	COG0179(S
Q571I4	PRAG1_MO	Prag1	Prag1	244418	Inactive tyr	no | no chan	0	ENOG4110
Q8VHR5	P66B_MOU	Gatad2b	Gatad2b	229542	Transcripti	no | no chan	0	ENOG410Z
P63024	VAMP3_M	Vamp3	Vamp3	22319	Vesicle-ass	no | no chan	0	COG5143(F
Q922G0	S2536_MOU	Slc25a36	Slc25a36	192287	Solute carri	no | no chan	0	ENOG410X
Q922G2	FA76A_MO	Fam76a	Fam76a	230789	Protein FAI	no | no chan	0	ENOG410Y
P63028	TCTP_MO	Tpt1	Tpt1	22070	Translation	no | no chan	0	ENOG4111
Q9CQX8	KGD4_MC	Kgd4	Kgd4	66128	Alpha-keto	no | no chan	0	ENOG410X
Q9CQX4	PAF15_MC	Pclaf	Pclaf	68026	PCNA-asso	no | no chan	0	ENOG410Y
Q9CQX6	Q9CQX6_M	Naa12	Naa12	117903916;	N-terminal	no | no chan	0	COG0456(c
Q8BG48	ST17B_MO	Stk17b	Stk17b	98267	Serine/thre	no | no chan	0	ENOG410X
F6WR04	F6WR04_M	Ctss	Ctss	13040	Cathepsin S	no | up	0	COG4870(M
B0V2N1	PTPRS_MC	Ptprs	Ptprs	19280	Receptor-ty	no | no chan	0	COG5599(F
A0A668KL	A0A668KL	Ccdc50	Ccdc50	67501	Coiled-coil	no | no chan	0	ENOG4111
Q3UJ81	NEPR1_MO	Cnep1r1	Cnep1r1	382030	Nuclear env	no | no chan	0	ENOG4111
Q3TFQ8	Q3TFQ8_M	Pygb	Pygb	110078	Alpha-1,4 g	no | no chan	0	COG0058(S
Q3TFQ1	SPRY7_MC	Sprya7	Spryd7	66674	SPRY domc	no | no chan	0	ENOG410X
Q3UMB5	SMCR8_MO	Smcr8	Smcr8	237782	Guanine nu	no | no chan	0	ENOG410X
Q8CFE8	Q8CFE8_M	Fbxw17	Fbxw17	109082	F-box and W	no | up	0	ENOG4111
Q3UIH7	Q3UIH7_M	Rabggta	Rabggta	56187	Geranylger	no | no chan	0	COG5536((
P59941	SIR6_MOU	Sirt6	Sirt6	50721	NAD-deper	no | no chan	0	COG0846(F
Q3UBS3	Q3UBS3_M	Hp	Hp	15439	Haptoglobi	no | up	0	COG5640(F
Q8CFE2	HPF1_MOU	Hpf1	Hpf1	72612	Histone PA	no | no chan	0	ENOG410X
Q8CFE6	S38A2_MO	Slc38a2	Slc38a2	67760	Sodium-cou	no | no chan	0	COG0814(M
Q3TVN6	Q3TVN6_M	Pf4	Pf4	56744	C-X-C moti	no | no chan	0	ENOG4111
A0A075B5Y	A0A075B5Y	Ighv1-80	Ighv1-80		Immunoglo	no | no chan	0	ENOG4111
A2ACC6	A2ACC6_M	Ralgapb	Ralgapb	228850	Ral GTPas	no | no chan	0	ENOG4110
E9Q3U5	E9Q3U5_M	Naa16	Naa16		N(alpha)-a	no | no chan	0	ENOG410X
O09126	SEM4D_M	Sema4d	Sema4d	20354	Semaphorir	no | no chan	0	ENOG410X
Q5FWX6	Q5FWX6_M	Prkd3	Prkd3	75292	Serine/thre	no | no chan	0	ENOG410X
Q3V2E3	Q3V2E3_M	Uqcc3	Uqcc3	107197	Ubiquinol-c	no | no chan	0	ENOG4111
Q8VEN2	PLET1_MC	Plet1	Plet1	76509	Placenta-ex	no | up	0	ENOG4111
Q6ZQ73	CAND2_M	Cand2	Cand2	67088	Cullin-asso	no | no chan	0	ENOG410X
Q6ZQ74	Q6ZQ74_M	Daam1	Daam1	208846	MKIAA066	no | no chan	0	ENOG410X
D3YYM6	D3YYM6_M	Rps5	Rps5		Small ribos	no | no chan	0	COG0049(A
P33766	FPR1_MOU	Fpr1	Fpr1	14293	fMet-Leu-P	no | up	0	ENOG410Y
E0CYV0	E0CYV0_M	Pcmt1	Pcmt1	18537	Protein-L-is	no | no chan	0	COG2518(F
A2A9V9	A2A9V9_M	Kctd2	Kctd2		Potassium c	no | up	0	ENOG410X
E0CYV4	E0CYV4_M	Desi2	Desi2		Deubiquiti	yes | up	1	ENOG4111
B2RTM2	B2RTM2_M	Ucp3	Ucp3	22229	Putative mi	no | up	0	ENOG410X
B2RTM0	B2RTM0_M	Hist2h4	Hist2h4	319155; 319	Histone H4	no | no chan	0	
Q1JPR8	Q1JPR8_M	Tppp3	Tppp3	67971	Tubulin po	no | up	0	ENOG4111

Q9CR78	MSD3_MO	Msantd3	Msantd3	66665	Myb/SANT	no	no chan	0	ENOG410
O35350	CAN1_MO	Capn1	Capn1	12333	Calpain-1 c	no	no chan	0	ENOG410
Q9CPZ6	ORML3_M	Ormdl3	Ormdl3	66612	ORM1-like	no	down	0	COG5081(
Q9QXB9	DRG2_MC	Drg2	Drg2	13495	Developme	no	no chan	0	COG1163(
P07309	TTHY_MO	Ttr	Ttr	22139	Transthyret	no	no chan	0	COG2351(
F8VQN6	F8VQN6_M	Arhgef12	Arhgef12	69632	Rho guanin	no	no chan	0	COG5422(
A0A1B0GT	A0A1B0GT	Kcnn2	Kcnn2	140492	Potassium i	no	up	0	ENOG410
H7BX23	H7BX23_M	P4ha3	P4ha3	320452	procollagen	no	no chan	0	ENOG410
H7BX26	H7BX26_M	Cep170	Cep170	545389	Centrosom	no	no chan	0	ENOG411
A0A0R4J1Z	A0A0R4J1Z	Pou2f1	Pou2f1	18986	POU doma	no	no chan	0	ENOG410
Q9D2X8	CH076_MOUSE	Uncharacterized p		75758	Uncharacte	no	no chan	0	ENOG411
F8WIR1	F8WIR1_M	Ctsd	Ctsd		Cathepsin I	no	no chan	0	ENOG410
G3UYM7	G3UYM7_	Dtnb	Dtnb		Dystrobrevi	no	down	0	ENOG410
A0A0R4J1Z	A0A0R4J1Z	Tmem33	Tmem33	67878	Transmemb	no	no chan	0	ENOG4111
P10639	THIO_MO	Txn	Txn	22166	Thioredoxi	no	no chan	0	COG0526(
Q8R2R9	AP3M2_M	Ap3m2	Ap3m2	64933	AP-3 compl	no	no chan	0	ENOG410
O70325	GPX4_MO	Gpx4	Gpx4	625249	Phospholip	no	no chan	0	COG0386(
Q8R2R6	MTG1_MC	Mtg1	Mtg1	212508	Mitochondı	no	no chan	0	COG1161(
P33587	PROC_MO	Proc	Proc	19123	Vitamin K-	no	no chan	0	COG5640(
O70494	SP3_MOUS	Sp3	Sp3	20687	Transcripti	no	up	0	COG5048(
P10630	IF4A2_MO	Eif4a2	Eif4a2	13682	Eukaryotic	no	no chan	0	COG0513(
O70492	SNX3_MO	Snx3	Snx3	54198	Sorting nex	no	no chan	0	COG5391(
B9EI38	B9EI38_M	Reps2	Reps2	194590	RALBP1 as	no	down	0	ENOG410
Q3UAS2	Q3UAS2_M	Capza1	Capza1	12340	F-actin-cap	no	no chan	0	ENOG410
B2RSI3	B2RSI3_M	Parp14	Parp14	547253	Poly [ADP-	no	up	0	COG2110(
A6PWY4	WDR76_M	Wdr76	Wdr76	241627	WD repeat-	no	no chan	0	ENOG410
Q2TPA8	HSDL2_M	Hsdl2	Hsdl2	72479	Hydroxyste	no	no chan	0	COG1028(
Q9DAM7	TM263_MC	Tmem263	Tmem263	103266	Transmemb	no	no chan	0	ENOG4111
Q3TXX8	Q3TXX8_M	Wasl	Wasl	73178	N-WASP	no	no chan	0	ENOG4111
Q7TNM8	Q7TNM8_	Scgb2b27	Scgb2b27	233099	Salivary an	no	no chan	0	ENOG4111
Q3TMH2	SCRN3_M	Scrn3	Scrn3	74616	Secernin-3	no	no chan	0	COG4690(
B0FTY3	B0FTY3_M	Nudcd3	Nudcd3	209586	NudC-like	no	no chan	0	ENOG410
Q4VAA2	CDV3_MO	Cdv3	Cdv3	321022	Protein CD	no	no chan	0	ENOG410
Q3TXX3	ZFY27_MC	Zfyve27	Zfyve27	319740	Protrudin	no	no chan	0	ENOG4111
P62082	RS7_MOUS	Rps7	Rps7	20115	Small ribos	no	no chan	0	ENOG410
Q9D898	ARP5L_M	Arpc5l	Arpc5l	74192	Actin-relat	no	no chan	0	ENOG4111
Q3TB82	PKHF1_M	Plekhf1	Plekhf1	72287	Pleckstrin h	no	no chan	0	ENOG410
Q8BXV4	Q8BXV4_M	Ecd	Ecd	70601	Protein ecd	no	no chan	0	ENOG410
B2RRS4	B2RRS4_M	Gtpbp3	Gtpbp3	70359	GTP bindir	no	no chan	0	COG0486(
A0A1L1SQ	A0A1L1SQ	Rnf26	Rnf26		Ring finger	no	no chan	0	ENOG410
A0A7N9VS	AFG2B_M	Afg2b	Afg2b	214616	ATPase fan	no	no chan	0	COG0464(
Q8R480	NUP85_M	Nup85	Nup85	445007	Nuclear po	no	no chan	0	ENOG410
O70145	NCF2_MO	Ncf2	Ncf2	17970	Neutrophil	no	no chan	0	ENOG4111

Q8N7N5	DCAF8_M(	Dcaf8	Dcaf8	98193	DDB1- and	no | no chan	0	ENOG410
A0A1L1SRI	A0A1L1SRI	Senp8	Senp8	71599	SUMO pep	no | no chan	0	ENOG4111
Q3TZR8	Q3TZR8_M	Zhx2	Zhx2	387609	Zinc finger	no | no chan	0	ENOG410
Q3TZR9	Q3TZR9_M	Atf7	Atf7	223922	Cyclic AMI	no | no chan	0	ENOG4111
F8WHP8	F8WHP8_M	Atp5mf	Atp5mf		ATP syntha	no | no chan	0	ENOG4111
Q99M31	HSP7E_MC	Hspa14	Hspa14	50497	Heat shock	no | no chan	0	COG0443(c
A0A571BF	A0A571BF	Fam83h	Fam83h		Family with	no | up	0	ENOG410
Q14B65	Q14B65_M	Cep95	Cep95	320162	Ccdc45 pro	no | up	0	ENOG411(
Q6ZPF4	FMNL3_M	Fmnl3	Fmnl3	22379	Formin-like	no | no chan	0	ENOG410
A0A3Q4EF	A0A3Q4EF	Srsf7	Srsf7		Serine and	no | no chan	0	ENOG4111
E9Q852	E9Q852_M	Afdn	Afdn		Afadin, adh	no | no chan	0	ENOG410
A0A087WN	A0A087WN	Ly6c1	Ly6c1	17067	Lymphocyt	no | up	0	ENOG4111
A3KML5	A3KML5_M	Atp6v0c	Atp6v0c	11984; 1000	V-type prot	no | no chan	0	COG0636(I
Q3TJ84	Q3TJ84_M(	Paip2	Paip2	67869	Polyadenyl	no | no chan	0	ENOG410
A3KML3	A3KML3_M	Ywhaq	Ywhaq	22630	14-3-3 prote	no | no chan	0	COG5040((
H3BKE1	H3BKE1_M	Plch1	Plch1		Phosphoinc	no | up	0	ENOG410
Q3TJ87	Q3TJ87_M(	Nabp1	Nabp1	109019	SOSS comp	no | no chan	0	ENOG4111
A0A338P6K	A0A338P6K	Myh11	Myh11		Myosin-11	no | down	0	COG5022(I
Q91XK1	Q91XK1_M	Tcf4	Tcf4	21413	Transcripti	no | down	0	ENOG410
P51141	DVL1_MO	Dvl1	Dvl1	13542	Segment po	no | no chan	0	ENOG410
Q8CG73	FTM_MOU	Rpgrip1l	Rpgrip1l	244585	Protein fan	no | up	0	ENOG410
A0A338P6K	QSER1_M(	Qser1	Qser1	99003	Glutamine	no | down	0	ENOG410
Q8CG71	P3H2_MOU	P3h2	P3h2	210530	Prolyl 3-hy	no | down	0	ENOG410
Q8CG76	ARK72_M(	Akr7a2	Akr7a2	110198	Aflatoxin B	no | no chan	0	COG0667(r
B1AR17	B1AR17_M	Chd3	Chd3	216848	DNA helic	no | no chan	0	COG0553(
Q3UMB9	WASC4_M(	Washc4	Washc4	319277	WASH com	no | no chan	0	ENOG410
B1AR13	CISD3_MC	Cisd3	Cisd3	217149	CDGSH irc	no | no chan	0	ENOG411
Q99PL6	UBXN6_M	Ubxn6	Ubxn6	66530	UBX doma	no | no chan	0	ENOG410
Q8BP60	Q8BP60_M	Nxf1	Nxf1	53319	Nuclear RN	no | no chan	0	ENOG410
P06797	CATL1_M(	Ctsl	Ctsl	13039	Procatheps	no | no chan	0	COG4870(g
D3Z0F5	D3Z0F5_M	Cops6	Cops6		COP9 signc	no | no chan	0	COG1310((
Q3TR89	Q3TR89_M	Cog1	Cog1	16834	Conserved	no | no chan	0	ENOG410
E9QN63	E9QN63_M	Asap1	Asap1		ArfGAP wi	no | no chan	0	COG5347(I
Q3U6Q5	Q3U6Q5_M	Lyn	Lyn	17096	Tyrosine-pr	no | no chan	0	COG0515(S
Q3UQN9	Q3UQN9_	Tada2a	Tada2a	217031	Uncharacte	no | no chan	0	COG5114(N
B7FAT9	B7FAT9_M	Gprc5c	Gprc5c	70355	G protein-c	no | no chan	0	ENOG411(
A2A7A7	A2A7A7_M	H6pd	H6pd	100198	Hexose-6-p	no | no chan	0	COG0364(I
Q3UQN2	FCHO2_M	Fcho2	Fcho2	218503	F-BAR dom	no | no chan	0	ENOG410
P70295	AUP1_MO	Aup1	Aup1	11993	Lipid dropl	no | no chan	0	ENOG410
P70297	STAM1_M(	Stam	Stam	20844	Signal trans	no | no chan	0	ENOG410
P70290	EM55_MO	Mpp1	Mpp1	17524	55 kDa eryt	no | no chan	0	COG0194(s
Q3UTR5	Q3UTR5_M	Pycr1	Pycr1	209027	Pyrroline-5	no | no chan	0	COG0345(s
Q52KC5	Q52KC5_M	Chchd5	Chchd5	66170	Coiled-coil	no | no chan	0	ENOG4111

Q8BHX3	BOREA_M	Cdca8	Cdca8	52276	Borealin	no | no chan	0	ENOG4111
O88746	TOM1_MC	Tom1	Tom1	21968	Target of M	no | no chan	0	ENOG410Y
Q8CHZ9	MTF1A_M	Mterf1a	Mterf1a	545725	Transcripti	no | down	0	ENOG410X
A2AKV6	A2AKV6_M	Alkbh3	Alkbh3		AlkB homo	no | no chan	0	COG3145(I
A0A1Y7VM	A0A1Y7VM	E2f3	E2f3		E2F transcr	no | no chan	0	ENOG410X
P57791	FACE2_MC	Rce1	Rce1	19671	CAAX pren	no | no chan	0	ENOG410Y
A0ZV96	A0ZV96_M	Oscp1	Oscp1	230751	Organic sol	no | no chan	0	ENOG411C
Q6PJ18	Q6PJ18_M	Tpm2	Tpm2	22004	Tropomyos	no | no chan	0	ENOG410X
Q8BPY9	FIGL1_MC	Fignl1	Fignl1	60530	Fidgetin-lik	no | no chan	0	COG0464(z
Q8C1Y8	CCZ1_MO	Ccz1	Ccz1	231874	Vacuolar fu	no | no chan	0	ENOG410X
Q3TW51	Q3TW51_M	Snd1	Snd1	56463	Staphyloco	no | no chan	0	COG1525(r
Q60996	2A5G_MO	Ppp2r5c	Ppp2r5c	26931	Serine/thre	no | no chan	0	ENOG410X
Q3UGE1	Q3UGE1_M	Trappc12	Trappc12	217449	Uncharacte	no | no chan	0	ENOG410X
Q149L6	DJB14_MO	Dnajb14	Dnajb14	70604	DnaJ homo	no | down	0	COG0484(p
Q9DAA6	EXOS1_MC	Exosc1	Exosc1	66583	Exosome cc	no | no chan	0	COG1096(I
Q61024	ASNS_MO	Asns	Asns	27053	Asparagine	no | no chan	0	COG0367(c
E9PWB2	E9PWB2_M	Csnk1a1	Csnk1a1		non-specifi	no | no chan	0	ENOG410X
Q8CI12	SMTL2_MC	Smtnl2	Smtnl2	276829	Smoothelin	no | up	0	COG5069(S
A0A0N4SV	A0A0N4SV	Rarres2	Rarres2	71660	Retinoic ac	no | up	0	ENOG410Z
A2AD03	A2AD03_M	Chm	Chm	12662	Rab proteir	no | no chan	0	COG5044(r
B2RUF9	B2RUF9_M	Olfm4	Olfm4	380924	Olfm4 prot	no | up	0	ENOG410Z
Q9D7J4	COX20_MC	Cox20	Cox20	66359	Cytochrom	no | no chan	0	ENOG4111
B1AWS8	B1AWS8_M	Tek	Tek	21687	receptor pr	no | no chan	0	COG0515(u
Q9D7J9	ECHD3_M	Echdc3	Echdc3	67856	Enoyl-CoA	no | no chan	0	ENOG410X
A2AJN7	S4A11_MO	Slc4a11	Slc4a11	269356	Solute carri	no | no chan	0	ENOG410X
Q8C145	S39A6_MO	Slc39a6	Slc39a6	106957	Zinc transp	no | no chan	0	COG0428(c
Q08EE0	Q08EE0_M	Bod1	Bod1	69556	2310022M1	no | no chan	0	ENOG4111
Q3UYC1	Q3UYC1_M	Mvd	Mvd	192156	Diphospho	no | no chan	0	COG3407(I
Q08EE6	Q08EE6_M	Tsku	Tsku	244152	Tsku protei	no | no chan	0	COG4886(t
Q921Y2	IMP3_MOU	Imp3	Imp3	102462	U3 small nu	no | no chan	0	COG0522(N
Q5BL18	Q5BL18_M	Matr3	Matr3	17184	Matrin 3	no | no chan	0	ENOG410X
A8Y5T6	A8Y5T6_M	Pars2	Pars2	230577	proline--tR	no | no chan	0	COG0442(C
E9Q6Z3	E9Q6Z3_M	Nemp1	Nemp1		Nuclear en	no | no chan	0	ENOG410Z
F6QSC4	F6QSC4_M	Mgl2	Mgl2		Macrophag	no | down	0	ENOG410X
B1AVU1	B1AVU1_M	Prkx	Prkx	19108	Protein kin	no | up	0	ENOG410X
Q14DP3	Q14DP3_M	Lars2	Lars2	102436	leucine--tR	no | no chan	0	COG0495(I
Q8BN51	Q8BN51_M	Tk2	Tk2	57813	Thymidine	no | up	0	COG1428(v
Q3TEK6	Q3TEK6_M	Dapp1	Dapp1	26377	Dual adapt	no | up	0	ENOG410X
Q3U2P1	SC24A_MC	Sec24a	Sec24a	77371	Protein trar	no | no chan	0	COG5028(S
Q7TSZ3	Q7TSZ3_M	Lars1	Lars1	107045	leucine--tR	no | no chan	0	COG0495(S
Q8BY35	FGD2_MO	Fgd2	Fgd2	26382	FYVE, Rho	no | no chan	0	ENOG410X
A0A0J9YVI	A0A0J9YVI	Klrg2	Klrg2		Killer cell l	no | down	0	ENOG410X
Q7TSZ8	NACC1_M	Nacc1	Nacc1	66830	Nucleus ac	no | down	0	COG5048(s

Q9EPJ9	ARFG1_MO	Arfgap1	Arfgap1	228998	ADP-ribosy	no | no chan	0	COG5347(I
A2BG75	A2BG75_M	Nfib	Nfib	18028	Nuclear fac	no | no chan	0	ENOG410Y
A0A991EQ	A0A991EQ	Nploc4	Nploc4		NPL4 homo	no | no chan	0	COG5100(N
A0A991EQ	A0A991EQ	Rptor	Rptor		Regulatory	no | no chan	0	ENOG410X
H3BKY1	H3BKY1_M	Glmp	Glmp		Glycosylate	no | no chan	0	ENOG4111
P02816	PIP_MOUS	Pip	Pip	18716	Prolactin-ir	no | no chan	0	ENOG4111
Q91YZ5	Q91YZ5_M	Pex2	Pex2	19302	Peroxisome	no | no chan	0	ENOG411C
A0A286YD	A0A286YD	Smc5	Smc5	226026	Structural r	no | no chan	0	COG1196(s
Q3THW7	Q3THW7_I	Eif3h	Eif3h	68135	Eukaryotic	no | no chan	0	ENOG410X
A0A6G7MA	A0A6G7MA	Xaf1	Xaf1	327959	Xaf1	no | up	0	ENOG411C
F6ZFT1	F6ZFT1_M	Ndufab1	Ndufab1		Acyl carrier	no | no chan	0	COG0236(C
H3BJN3	H3BJN3_M	Cux1	Cux1	13047	Protein CAS	no | no chan	0	ENOG410X
Q8R107	PRLD1_MO	Prelid1	Prelid1	66494	PRELI dom	no | no chan	0	ENOG4111
Q8R105	VP37C_MO	Vps37c	Vps37c	107305	Vacuolar p	no | no chan	0	ENOG4111
P42232	STA5B_MO	Stat5b	Stat5b	20851	Signal trans	no | no chan	0	ENOG410X
P42230	STA5A_MO	Stat5a	Stat5a	20850	Signal trans	no | no chan	0	ENOG410X
Q0VGT1	Q0VGT1_M	Diaph3	Diaph3	56419	Diaphanou	no | no chan	0	ENOG410Y
Q0VGT2	GLI2_MOU	Gli2	Gli2	14633	Zinc finger	no | down	0	COG5048(S
Q5SUR0	PUR4_MOU	Pfas	Pfas	237823	Phosphorib	no | no chan	0	COG0047(p
A0A571BEY	A0A571BEY	Zdhhc3	Zdhhc3		Palmitoyltr	no | no chan	0	
Q5SSZ5	TENS3_MO	Tns3	Tns3	319939	Tensin-3	no | no chan	0	COG2453(I
Q66JT1	LRC8E_MO	Lrrc8e	Lrrc8e	72267	Volume-reg	no | up	0	COG4886(C
Q8C5X6	Q8C5X6_M	Fen1	Fen1	14156	Flap endon	no | no chan	0	COG0258(C
Q9D394	RUFY3_MO	Rufy3	Rufy3	52822	Protein RU	no | no chan	0	ENOG410X
A0A023J5X	A0A023J5X	CYTB	CYTB		Cytochrom	no | no chan	0	COG1290(C
Q3UF75	Q3UF75_M	Parva	Parva	57342	Parvin, alpl	no | no chan	0	ENOG410X
Q3UPE3	RMI2_MOU	Rmi2	Rmi2	223970	RecQ-medi	no | no chan	0	ENOG4112
Q6GQX2	NCK5L_MO	Nckap5l	Nckap5l	380969	Nck-associ	no | no chan	0	ENOG410X
Q540E6	Q540E6_M	Rheb	Rheb	19744	RHEB-like	no | no chan	0	COG1100(r
A0A1Y7VJ1	A0A1Y7VJ1	Zfyve21	Zfyve21		Zinc finger,	no | no chan	0	ENOG4111
Q148S3	Q148S3_MO	Nob1	Nob1	67619	RNA-bindi	no | no chan	0	COG1439(N
G3X9D6	G3X9D6_M	Apon	Apon	28194	Apolipopro	no | up	0	ENOG4111
Q3V277	Q3V277_M	Zbtb1	Zbtb1	268564	Zinc finger	no | no chan	0	COG5048(T
A0A2I3BRX	A0A2I3BRX	Mettl6	Mettl6		tRNA N(3)-	no | no chan	0	ENOG410X
A0A2I3BRX	A0A2I3BRX	Ralgapa1	Ralgapa1		Ral GTPase	no | no chan	0	ENOG410X
Q9EQM6	DGCR8_M	Dgcr8	Dgcr8	94223	Microproce	no | no chan	0	ENOG410X
A0A1B0GR	A0A1B0GR	Sirt3	Sirt3		NAD-deper	no | no chan	0	COG0846(N
Q99J39	DCMC_MO	Mlycd	Mlycd	56690	Malonyl-Co	no | no chan	0	COG1593(t
Q99J36	THUM1_M	Thumpd1	Thumpd1	233802	THUMP do	no | no chan	0	COG1818(T
Q9D1G3	HHATL_M	Hhatl	Hhatl	74770	Protein-cys	no | up	0	COG1696(N
Q9DBE9	SPB1_MOU	Ftsj3	Ftsj3	56095	pre-rRNA 2	no | no chan	0	COG0293(S
Q9DBE8	ALG2_MO	Alg2	Alg2	56737	Alpha-1,3/1	no | no chan	0	COG0438(U
Q9JJ94	SSNA1_MO	Ssna1	Ssna1	68475	Microtubul	no | no chan	0	ENOG4111

A0JNU3	LPP60_MO	Aspg	Aspg	104816	60 kDa lyso	no|up	0	COG0252(I
D3YZV3	D3YZV3_M	Gsta2	Gsta2		Glutathion	no|down	0	ENOG4111
Q3UCI6	Q3UCI6_M	Ptcd1	Ptcd1	71799	Pentatricop	no|no chan	0	ENOG410Z
Q6ZPR6	IBTK_MOU	Ibtk	Ibtk	108837	Inhibitor of	no|no chan	0	COG5184(T
Q6ZPR5	NSMA3_M	Smpd4	Smpd4	77626	Sphingomy	no|no chan	0	ENOG410Y
G5E889	G5E889_M	Mto1	Mto1	68291	Protein MT	no|no chan	0	COG0445(M
B2RQZ3	B2RQZ3_M	Slc41a2	Slc41a2	338365	Solute carri	no|down	0	ENOG410X
Q99PP2	ZN318_MC	Znf318	Znf318	57908	Zinc finger	no|no chan	0	ENOG4111
Q59IX2	Q59IX2_M	Orc1	Orc1	18392	Origin reco	no|no chan	0	COG1474(I
P63271	SPT4A_MC	Supt4h1a	Supt4h1a	20922	Transcripti	no|no chan	0	COG5204(T
B2RY70	B2RY70_M	Cmip	Cmip	74440	4933407C0	no|no chan	0	ENOG410X
B2RR03	B2RR03_M	Gp1bb	Gp1bb	14724	Glycoprote	no|no chan	0	COG4886(M
Q9DAX9	APBP2_MC	Appbp2	Appbp2	66884	Amyloid pr	no|no chan	0	COG0457(C
O55236	MCE1_MO	Rngtt	Rngtt	24018	mRNA-cap	no|no chan	0	COG5226(S
Q8BXA5	CLP1L_MC	Clptm1l	Clptm1l	218335	Lipid scram	no|no chan	0	ENOG410X
O55234	PSB5_MOU	Psmb5	Psmb5	19173	Proteasome	no|no chan	0	ENOG410X
A2AKI5	A2AKI5_M	Itgav	Itgav		Integrin alp	no|no chan	0	ENOG410X
O55239	NNMT_MC	Nnmt	Nnmt	18113	Nicotinami	no|down	0	ENOG4112
P01898	HA10_MOU	H2-Q10	H2-Q10	15007	H-2 class I	no|no chan	0	ENOG4111
Q3TDP6	Q3TDP6_M	Trpc4ap	Trpc4ap	56407	Short transi	no|no chan	0	ENOG410Z
Q3TCD4	Q3TCD4_M	Eci2	Eci2	23986	Enoyl-Coen	no|no chan	0	COG4281(I
D3Z286	D3Z286_M	Tmppe	Tmppe	100504715	Transmemb	no|no chan	0	COG1408(r
Q3TDP3	Q3TDP3_M	Agpat5	Agpat5	52123	Phospholip	no|no chan	0	COG0204(p
B2RXQ3	B2RXQ3_M	Slf2	Slf2	226151	Family with	yes|up	1	ENOG410Y
B2RXQ2	B2RXQ2_M	Ppfia1	Ppfia1	233977	Ppfia1 prot	no|no chan	0	ENOG410X
A0A087WR	A0A087WR	Faim	Faim		Fas apopto	no|no chan	0	ENOG410X
A0A087WR	A0A087WR	Pcbp4	Pcbp4		Poly(rC) bir	no|no chan	0	ENOG410X
B2RXQ4	B2RXQ4_M	Nek1	Nek1	18004	non-specifi	no|no chan	0	ENOG410Y
A0A1L1SS1	A0A1L1SS1	Keap1	Keap1		Kelch-like I	no|no chan	0	ENOG410X
Q64337	SQSTM_M	Sqstm1	Sqstm1	18412	Sequestoso	no|no chan	0	ENOG410X
B1AZI6	THOC2_M	Thoc2	Thoc2	331401	THO comp	no|no chan	0	ENOG410X
A0A0N4SU	A0A0N4SU	Magi1	Magi1	14924	Membrane	no|no chan	0	COG5021(I
Q9CPZ8	COXM1_M	Cmc1	Cmc1	67899	COX assem	no|no chan	0	ENOG4111
E9PZJ8	ASCC3_MC	Ascc3	Ascc3	77987	Activating	no|no chan	0	COG1204(c
B1AW21	B1AW21_M	Mtm1	Mtm1	17772	Myotubula	no|no chan	0	ENOG410X
E9PZJ4	E9PZJ4_M	Ndst3	Ndst3	83398	[heparan su	no|no chan	0	ENOG410X
B9EKG5	B9EKG5_M	Gm4841	Gm4841	225594	IRG-type G	no|up	0	ENOG4112
P47856	GFPT1_M	Gfpt1	Gfpt1	14583	Glutamine-	no|no chan	0	COG0449(C
P47857	PFKAM_M	Pfkm	Pfkm	18642	ATP-depen	no|up	0	COG0205(C
A0A498WC	A0A498WC	Tm9sf3	Tm9sf3		Transmemb	no|no chan	0	ENOG410X
B9VU57	B9VU57_MUSMC	Fv1	gammaretro	14349	Fv1 gamma	no|down	0	ENOG410Z
B9EKG3	B9EKG3_M	Ggh	Ggh	14590	folate gam	no|no chan	0	ENOG410X
B5B2U4	B5B2U4_M	Nfatc3	Nfatc3	18021	Nuclear fac	no|no chan	0	ENOG4111

A2AIW0	ENTR1_M(	Entr1	Entr1	68112	Endosome-(	no | no chan	0	ENOG4111
Q6PFE3	RA54B_MC	Rad54b	Rad54b	623474	DNA repai	no | no chan	0	COG0553(c
Q0P6I6	Q0P6I6_M(	Ccdc91	Ccdc91	67015	Coiled-coil	no | no chan	0	ENOG4111
A6H697	A6H697_M	Kctd17	Kctd17	72844	Kctd17 pro	no | up	0	ENOG410X
Q9D710	TMX2_MO	Tmx2	Tmx2	66958	Thioredoxi	no | no chan	0	ENOG410X
Q9ESK4	ING2_MO	Ing2	Ing2	69260	Inhibitor o	no | no chan	0	COG5034(I
Q9D1D4	TMEDA_M	Tmed10	Tmed10	68581	Transmemb	no | no chan	0	ENOG410X
Q9ESK9	RBCC1_M(	Rb1cc1	Rb1cc1	12421	RB1-induci	no | no chan	0	ENOG410X
Q9R194	CRY2_MO	Cry2	Cry2	12953	Cryptochro	no | no chan	0	COG0415(A
A0A1L1SU	A0A1L1SU	1700017B05	1700017B05Rik		RIKEN cD	no | no chan	0	ENOG410Y
Q9CWN7	CNO11_M(	Cnot11	Cnot11	52846	CCR4-NOT	no | no chan	0	ENOG410X
Q9R190	MTA2_MO	Mta2	Mta2	23942	Metastasis-	no | no chan	0	ENOG410X
A0A1W2P6	A0A1W2P6	Stab2	Stab2		Stabilin 2	no | no chan	0	ENOG411(
Q8CE46	PUS7L_MC	Pus7l	Pus7l	78895	Pseudourid	no | no chan	0	COG0585(c
E9PW03	E9PW03_M	Gpaa1	Gpaa1		GPI ancho	no | no chan	0	ENOG410X
Q99LD9	EI2BB_MC	Eif2b2	Eif2b2	217715	Translation	no | no chan	0	COG1184(I
O54984	GET3_MO	Get3	Get3	56495	ATPase GE	no | no chan	0	COG0003(A
Q3SYK5	Q3SYK5_M	Abl1	Abl1	11350	Tyrosine-pr	no | no chan	0	COG0515(M
Q9Z175	LOXL3_M(	Loxl3	Loxl3	16950	Lysyl oxida	no | no chan	0	ENOG410X
Q9Z179	SHCBP_M(	Shcbp1	Shcbp1	20419	SHC SH2 d	no | no chan	0	ENOG410X
Q60865	CAPR1_M(	Caprin1	Caprin1	53872	Caprin-1	no | no chan	0	ENOG411(
Q60520	SIN3A_MC	Sin3a	Sin3a	20466	Paired amp	no | no chan	0	COG5602(I
P60122	RUVB1_M(	Ruvbl1	Ruvbl1	56505	RuvB-like 1	no | no chan	0	COG1224(r
J7H264	J7H264_M(	Slc4a10	Slc4a10	94229	Anion exch	no | no chan	0	ENOG410X
Q3UCV8	OTUL_MC	Otulin	Otulin	432940	Ubiquitin t	no | no chan	0	ENOG4111
Q6P8N8	Q6P8N8_M	Clpx	Clpx	270166	Caseinolyti	no | no chan	0	COG1219(A
Q5U4F6	DC2I2_MC	Dync2i2	Dync2i2	71820	Cytoplasmi	no | up	0	ENOG410Z
A2AVR6	A2AVR6_M	Acot11	Acot11	329910	Acyl-CoA t	no | no chan	0	COG1607(I
P35980	RL18_MOU	Rpl18	Rpl18	19899	Large ribos	no | no chan	0	COG1727(I
Q543X9	Q543X9_M	Tob2	Tob2	57259	Transducer	no | no chan	0	ENOG410Z
C4PFH5	C4PFH5_M	Psmc3ip	Psmc3ip	19183	Homologou	no | no chan	0	ENOG410X
Q9DC77	SMPX_MO	Smpx	Smpx	66106	Small musc	yes | up	1	ENOG4112
P70460	VASP_MO	Vasp	Vasp	22323	Vasodilato	no | no chan	0	ENOG411(
Q9DC70	NDUS7_M	Ndufs7	Ndufs7	75406	NADH deh	no | no chan	0	COG0377(M
D3YXL1	D3YXL1_M	Ntpcr	Ntpcr		Nucleoside-	no | no chan	0	COG1618(I
A0A0A6YY	A0A0A6YY	Pcdh7	Pcdh7	54216	Protocadhe	no | no chan	0	ENOG410X
Q8K2C6	SIR5_MOU	Sirt5	Sirt5	68346	NAD-deper	no | no chan	0	COG0846(S
P62876	RPAB5_M(	Polr2l	Polr2l	66491	DNA-direc	no | no chan	0	COG1644(I
F8VPY2	F8VPY2_M	Taf5	Taf5	226182	TATA-box	no | no chan	0	ENOG410X
P62874	GBB1_MO	Gnb1	Gnb1	14688	Guanine nu	no | no chan	0	ENOG410X
A0PJE6	A0PJE6_M(	Pccb	Pccb	66904	Propionyl-C	no | no chan	0	COG4799(M
Q5NBY9	PATZ1_M(	Patz1	Patz1	56218	POZ (BTB)	no | no chan	0	COG5048(v
A3QWY0	A3QWY0_M	Mbtps1	Mbtps1	56453	Membrane-	no | up	0	COG1404(I

Q11011	PSA_MOU!	Npepps	Npepps	19155	Puromycin-	no | no chan	0	COG0308(C
Q6NY09	Q6NY09_M	Lnpep	Lnpep	240028	Lnpep prot	no | up	0	COG0308(p
Q9QYG0	NDRG2_M	Ndrg2	Ndrg2	29811	Protein ND	no | no chan	0	ENOG410X
Q9R0H0	ACOX1_M	Acox1	Acox1	11430	Peroxisoma	no | no chan	0	COG1960(p
A4FTZ0	A4FTZ0_M	Yod1	Yod1	226418	Ubiquitin t	no | no chan	0	COG5539(t
B0QZP8	B0QZP8_M	Syngr2	Syngr2	20973	Synaptogyri	no | no chan	0	ENOG4111
D3Z4U2	D3Z4U2_M	Gtpbp10	Gtpbp10		GTP-bindir	no | no chan	0	COG0536(A
A2AH98	A2AH98_M	Tmem164	Tmem164		Transmemb	no | no chan	0	ENOG4111
B1AV66	B1AV66_M	Yipf6	Yipf6		Protein YIF	no | no chan	0	COG5080(f
Q7TNV0	DEK_MOU	Dek	Dek	110052	Protein DE	no | no chan	0	ENOG410X
Q7TNV1	TLC3B_MC	Tlcd3b	Tlcd3b	68952	Ceramide sy	no | down	0	ENOG410X
D3Z4U8	D3Z4U8_M	Dcaf11	Dcaf11		DDB1 and	no | no chan	0	ENOG410X
Q69ZC8	GPAM1_M	Gpalpp1	Gpalpp1	67467	GPALPP m	no | no chan	0	ENOG4111
A0AA74KS	A0AA74KS	Kat7	Kat7		histone ace	no | no chan	0	COG5027(j
Q3UID0	Q3UID0_M	Smarcc2	Smarcc2	68094	SWI/SNF r	no | no chan	0	COG5259(S
J3QM81	J3QM81_M	Ankzf1	Ankzf1	52231	Ankyrin rep	no | no chan	0	ENOG410X
J3QM82	J3QM82_M	Phlpp2	Phlpp2	244650	PH domain	no | down	0	COG4886(A
Q99KR3	LACB2_MC	Lactb2	Lactb2	212442	Endoribonu	no | no chan	0	COG0491(F
Q6P1H6	ANKL2_M	Ankle2	Ankle2	71782	Ankyrin rep	no | no chan	0	ENOG410X
A2AWT6	A2AWT6_M	Ubtf	Ubtf	21429	Upstream b	no | no chan	0	COG5648(h
A2AWT5	A2AWT5_M	Ubtf	Ubtf	21429	Upstream b	no | no chan	0	COG5648(c
Q3UZB8	Q3UZB8_M	Gtf2h3	Gtf2h3	209357	General tra	no | up	0	COG5242(T
Q8R1B4	EIF3C_MC	Eif3c	Eif3c	56347	Eukaryotic	no | no chan	0	ENOG410X
B2RRH9	B2RRH9_M	Gmps	Gmps	229363	GMP synth	no | no chan	0	COG0519(C
Q9Z2X2	PSD10_MC	Psmd10	Psmd10	53380	26S proteas	no | no chan	0	COG0666(e
A0A494BB(	A0A494BB(	Fads1	Fads1		Fatty acid c	no | no chan	0	ENOG410X
P39098	MA1A2_M(	Man1a2	Man1a2	17156	Mannosyl-c	no | no chan	0	ENOG410X
P49282	NRAM2_M	Slc11a2	Slc11a2	18174	Natural res	no | no chan	0	COG1914(e
Q3UNB9	Q3UNB9_M	Steap1	Steap1	70358	Ferric oxid	no | no chan	0	ENOG4111
J3QMG7	J3QMG7_M	Adgra2	Adgra2		Adhesion G	no | down	0	COG4886(r
Q80UP2	Q80UP2_M	Brd1	Brd1	223770	Brd1 protei	no | down	0	COG5076(b
B3V097	B3V097_M(	Cstf2	Cstf2	108062	BetaCstF-6	no | no chan	0	ENOG410X
B9EHC7	B9EHC7_M	Casq2	Casq2	12373	Calsequestr	no | up	0	ENOG4111
E9Q284	E9Q284_M	Coil	Coil	12812	Coilin	no | no chan	0	ENOG4112
E9Q286	ICE1_MOU	Ice1	Ice1	218333	Little elong	no | down	0	ENOG4111
Q78RX3	SIM12_MO	Smim12	Smim12	80284	Small integ	no | no chan	0	ENOG4112
Q3TWE8	Q3TWE8_M	Polr3e	Polr3e	26939	DNA-direc	no | no chan	0	ENOG410Y
A0A494BB(	A0A494BB(	Snx15	Snx15		Sorting nex	no | no chan	0	ENOG4111
E9QM90	E9QM90_M	Relch	Relch	227446	RAB11 binc	no | no chan	0	ENOG410Y
Q9WU81	G6PT3_MC	Slc37a2	Slc37a2	56857	Glucose-6-p	no | no chan	0	COG2271(M
D3YTU2	D3YTU2_M	Ogfod1	Ogfod1		Prolyl 3-hyc	no | no chan	0	COG3751(c
Q922E6	FAKD2_M	Fastkd2	Fastkd2	75619	FAST kinas	no | no chan	0	ENOG410Z
O70493	SNX12_MC	Snx12	Snx12	55988	Sorting nex	no | no chan	0	COG5391(T

A0A3Q4EC	A0A3Q4EC	Map4k3	Map4k3	225028	Mitogen-ac	no | no chan	0	ENOG410
Q8BHC7	RHBL4_MC	Rhbdd1	Rhbdd1	76867	Rhomboid-	no | no chan	0	ENOG411C
Q8BHC4	DCAKD_M	Dcakd	Dcakd	68087	Dephospho	no | no chan	0	COG0237(C
Q8BHC2	Q8BHC2_M	Ist1	Ist1	71955	IST1 homol	no | no chan	0	ENOG410Y
Q8R2R1	POMT1_M	Pomt1	Pomt1	99011	Protein O-r	no | no chan	0	COG1928(c
Q8BHC0	LYVE1_MC	Lyve1	Lyve1	114332	Lymphatic	no | no chan	0	ENOG4112
A2A8R7	A2A8R7_M	Tut4	Tut4	230594	Terminal u	no | no chan	0	COG5260(c
Q3UDK3	Q3UDK3_I	Rfc5	Rfc5	72151	AAA+ ATP	no | no chan	0	COG0470(S
A2A8R0	A2A8R0_M	Zfyve9	Zfyve9	230597	Zinc finger	no | up	0	ENOG410
A0A0G2JF	A0A0G2JF	Igkv19-93	Igkv19-93	692161	Immunoglo	no | down	0	ENOG410Y
D3Z767	D3Z767_M	Ttc33	Ttc33		Tetratricop	no | no chan	0	ENOG4111
D3Z769	D3Z769_M	Lin37	Lin37		Lin-37 DRE	no | no chan	0	ENOG4111
A0A0J9YQ	A0A0J9YQ	Rnf187	Rnf187		Ring finger	no | down	0	ENOG4111
Q3UY32	Q3UY32_M	Snapin	Snapin	20615	SNARE-ass	no | no chan	0	ENOG4111
Q3UBQ4	Q3UBQ4_I	Actb	Actb	11461	Actin, cyto	no | no chan	0	COG5277(c
A2A542	A2A542_M	Cacnb1	Cacnb1	12295	Voltage-dep	no | up	0	ENOG410
Q3UJ69	Q3UJ69_M	Zfp148	Zfp148	22661	C2H2-type	no | no chan	0	COG5048(F
Q9JI91	ACTN2_MC	Actn2	Actn2	11472	Alpha-actir	no | up	0	COG5069(F
A2A547	A2A547_M	Rpl19	Rpl19	19921	Ribosomal	no | no chan	0	COG2147(r
Q64524	H2B2E_MC	H2bc21	H2bc21	319190	Histone H2	no | no chan	0	ENOG4111
Q80TR8	DCAF1_MC	Dcaf1	Dcaf1	321006	DDB1- and	no | no chan	0	ENOG410
Q64522	H2A2B_MC	H2ac21	H2ac21	621893	Histone H2	no | no chan	0	COG5262(p
Q566C3	Q566C3_M	Gpt	Gpt	76282	alanine tra	no | no chan	0	COG0436(c
Q8QZR4	OAF_MOU	Oaf	Oaf	102644	Out at first	no | no chan	0	ENOG410
Q3TCE7	Q3TCE7_M	Arpc1b	Arpc1b	11867	Actin-relate	no | no chan	0	ENOG410
P45700	MA1A1_MC	Man1a1	Man1a1	17155	Mannosyl-c	no | no chan	0	ENOG410
Q9DBZ5	EIF3K_MC	Eif3k	Eif3k	73830	Eukaryotic	no | no chan	0	ENOG410
Q3U4Q5	Q3U4Q5_M	Hdac6	Hdac6	15185	UBP-type d	no | no chan	0	COG0123(i
Q7TMS6	Q7TMS6_M	Gltp	Gltp	56356	Glycolipid	no | no chan	0	ENOG410Y
Q9QWZ1	RAD1_MO	Rad1	Rad1	19355	Cell cycle c	no | no chan	0	ENOG410Y
Q6PAL7	AHDC1_M	Ahdc1	Ahdc1	230793	Transcripti	no | no chan	0	ENOG411C
Q6PAL5	Q6PAL5_M	Garre1	Garre1	233103	DUF4745 d	no | no chan	0	ENOG411C
Q6PAL3	Q6PAL3_M	Ap3s2	Ap3s2	11778	Adaptor-rel	no | no chan	0	COG5030(A
P31311	HXA11_MC	Hoxa11	Hoxa11	15396	Homeobox	no | no chan	0	ENOG4111
D3YYK8	D3YYK8_M	Mapre2	Mapre2		Microtubul	no | no chan	0	COG5217(A
Q6ZQ58	LARP1_MC	Larp1	Larp1	73158	La-related p	no | no chan	0	COG5193(I
A2A9P6	A2A9P6_M	Cdk11b	Cdk11b	12537	Cyclin depe	no | no chan	0	ENOG410
D3YYK0	D3YYK0_M	Abhd11	Abhd11	68758	sn-1-specifi	no | no chan	0	COG0596(p
B2X2D7	B2X2D7_M	Fut8	Fut8	53618	ASL1/FUT	no | no chan	0	ENOG410Y
P11589	MUP2_MO	Mup2	Mup2	17841; 1000	Major urinc	no | down	0	ENOG4111
Q3UH07	Q3UH07_M	Sfmbt2	Sfmbt2	353282	Scm-like wi	no | no chan	0	ENOG410Y
Q3UH06	RREB1_MC	Rreb1	Rreb1	68750	Ras-respon	no | no chan	0	COG5048(V
Q9CR58	KMCP1_M	Slc25a30	Slc25a30	67554	Kidney mit	no | no chan	0	ENOG410

Q9CR59	G45IP_MO	Gadd45gip	Gadd45gip	102060	Large ribos	no | no chan	0	ENOG410
D3YXD7	D3YXD7_	Slc39a14	Slc39a14		Solute carri	no | no chan	0	COG0428(z
F8VQL9	F8VQL9_M	Ncor2	Ncor2	20602	Nuclear rec	no | no chan	0	ENOG410
A0A494BA	A0A494BA	Arap3	Arap3		ArfGAP wi	no | up	0	COG5347(c
Q9CR55	CP087_MOUSE	UPF0547 protein C		66714	UPF0547 p	no | no chan	0	ENOG4111
Q9CR56	KBRS2_MC	Nkiras2	Nkiras2	71966	NF-kappa-l	no | no chan	0	ENOG411
Q9CR57	RL14_MOU	Rpl14	Rpl14	67115	Large ribos	no | no chan	0	COG2163((
Q9D706	RPAP3_MC	Rpap3	Rpap3	71919	RNA polym	no | no chan	0	ENOG4111
A0A5F8MP	CTSRT_MC	C2cd6	C2cd6	73463	Cation cha	no | no chan	0	ENOG411
Q9Z1E3	IKBA_MO	Nfkbia	Nfkbia	18035	NF-kappa-l	yes | up	1	COG0666(C
Q8BZB2	COAC_MC	Ppcdc	Ppcdc	66812	Phosphopa	no | no chan	0	COG0452(C
B7ZN64	B7ZN64_M	Siglecf	Siglecf	233186	Sialic acid l	no | down	0	ENOG410
P07361	A1AG2_MC	Orm2	Orm2	18406	Alpha-1-ac	no | up	0	ENOG4111
F8WIP8	F8WIP8_M	Spp1	Spp1	20750	Secreted ph	no | up	0	ENOG4111
P62313	LSM6_MO	Lsm6	Lsm6	78651	U6 snRNA-	no | no chan	0	COG1958(I
B7ZN63	B7ZN63_M	Prelid2	Prelid2	77619	PRELI dom	no | no chan	0	ENOG4111
P62311	LSM3_MO	Lsm3	Lsm3	67678	U6 snRNA-	no | no chan	0	ENOG4111
Q3UIJ1	Q3UIJ1_M	Exd2	Exd2	97827	3'-5' exonuc	no | no chan	0	ENOG410
Q8C761	DC2I1_MC	Dync2i1	Dync2i1	217935	Cytoplasmi	no | no chan	0	ENOG411
H7BX02	H7BX02_M	Rc3h1	Rc3h1		RING-type	no | no chan	0	ENOG410
Q05DI7	Q05DI7_M	Fam89b	Fam89b	17826	Family with	no | no chan	0	ENOG411
P61750	ARF4_MO	Arf4	Arf4	11843	ADP-ribosy	no | no chan	0	COG1100(F
Q05DI2	Q05DI2_M	Inpp5k	Inpp5k	19062	Inpp5k pro	no | no chan	0	COG5411(r
E9PYQ4	E9PYQ4_M	Prol1	Prol1	17830	Proline rich	no | no chan	0	
Q54A37	Q54A37_M	Akr1c13	Akr1c13	27384	Dihydrodio	no | no chan	0	COG0656(r
Q78H73	Q78H73_M	Slc35c1	Slc35c1	228368	Solute carri	no | no chan	0	ENOG410
Q3TMN9	Q3TMN9_	Herpud1	Herpud1	64209	Ubiquitin-l	no | no chan	0	ENOG410
A0A0J9YU	A0A0J9YU	Med13l	Med13l	76199	Mediator o	no | no chan	0	ENOG410
B9EID1	B9EID1_M	Coq4	Coq4	227683	Ubiquinon	no | no chan	0	COG5031(C
A2AW73	A2AW73_M	Sema6d	Sema6d	214968	Sema doma	no | up	0	ENOG410
Q9CYA0	CREL2_MC	Creld2	Creld2	76737	Protein dis	yes | up	1	ENOG411
Q14B01	Q14B01_M	Rnf113a2	Rnf113a2	66381	Ring finger	no | no chan	0	COG5152(F
P84091	AP2M1_MC	Ap2m1	Ap2m1	11773	AP-2 compl	no | no chan	0	ENOG410
Q8BIX3	AL14E_MC	Arl14ep	Arl14ep	212772	ARL14 effe	no | no chan	0	ENOG4111
P84096	RHOG_MC	Rhog	Rhog	56212	Rho-related	no | no chan	0	COG1100(s
Q99M51	NCK1_MO	Nck1	Nck1	17973	SH2/SH3 a	no | no chan	0	ENOG410
D6REU0	D6REU0_M	Mindy3	Mindy3		Ubiquitin c	no | no chan	0	ENOG410
Q99M54	CDCA3_M	Cdca3	Cdca3	14793	Cell divisio	no | no chan	0	ENOG410
Q99NA9	PCGF6_MC	Pcgf6	Pcgf6	71041	Polycomb g	no | no chan	0	ENOG410
G3UZZ3	G3UZZ3_M	Dnm2	Dnm2		dynamin G'	no | no chan	0	COG0699(M
O70306	TBX15_MC	Tbx15	Tbx15	21384	T-box trans	no | no chan	0	ENOG410
K7XUH1	K7XUH1_	S1pr1	S1pr1	13609	Sphingosin	no | no chan	0	ENOG410
O70309	ITB5_MOU	Itgb5	Itgb5	16419	Integrin be	no | no chan	0	ENOG410

Q9EQ61	PESC_MOUPes1		Pes1	64934	Pescadillo h	no	no chan	0	COG5163((
Q9CZ91	SRFB1_MCSrfbp1		Srfbp1	67222	Serum resp	no	no chan	0	ENOG4111
B1AR74	B1AR74_M Bcas3		Bcas3		BCAS3 mic	no	no chan	0	ENOG410X
P25118	TNR1A_M(Tnfrsf1a		Tnfrsf1a	21937	Tumor necr	no	no chan	0	ENOG4111
Q3TAE8	ALG6_MO Alg6		Alg6	320438	Dolichyl py	no	no chan	0	ENOG410X
P17742	PPIA_MOUPpia		Ppia	268373	Peptidyl-pr	no	no chan	0	COG0652(c
Q8BP47	SYNC_MO Nars1		Nars1	70223	Asparagine	no	no chan	0	COG0017(c
Q8K2T8	PAF1_MOUPaf1		Paf1	54624	RNA polym	no	no chan	0	ENOG410Y
P05480	SRC_MOUSrc		Src	20779	Proto-onco	no	no chan	0	COG0515(c
E9Q8N1	E9Q8N1_MTtn		Ttn	22138	Titin	no	up	0	ENOG410X
Q9D0D3	PAPD1_M(Mtpap		Mtpap	67440	Poly(A) RN	no	no chan	0	COG5260(S
E9Q8N5	E9Q8N5_MClasp2		Clasp2	76499	CLIP associ	no	no chan	0	ENOG410Z
O70165	FCN1_MO Fcn1		Fcn1	14133	Ficolin-1	no	no chan	0	ENOG410Z
Q6ZPI0	JADE1_MCJade1		Jade1	269424	Protein Jad	no	no chan	0	COG5141(f
A2A7G6	A2A7G6_MMad2l2		Mad2l2		Mitotic spi	no	down	0	ENOG4111
Q9DD16	NUD22_M(Nudt22		Nudt22	68323	Uridine dip	no	no chan	0	ENOG410Y
Q9CQK7	RWDD1_MRwdd1		Rwdd1	66521	RWD domc	no	no chan	0	ENOG410X
O88720	O88720_M(Rbm6		Rbm6	19654	RNA bindi	no	no chan	0	ENOG4111
Q544G5	Q544G5_MDag1		Dag1	13138	Dystroglyc	no	no chan	0	ENOG410X
O88727	O88727_M(Erdr1x		Erdr1x	170942	Erythroid d	no	no chan	0	
A2ADE0	A2ADE0_MPlekhm2		Plekhm2	69582	Pleckstrin h	no	no chan	0	ENOG410Z
Q3U2J9	Q3U2J9_M(Gmcl1		Gmcl1	23885	BTB domai	no	no chan	0	ENOG410X
A0A654ICI	A0A654ICIGja1		Gja1	14609	Gap junctic	no	down	0	ENOG411(
Q3TYA6	MPP8_MO Mphosph8		Mphosph8	75339	M-phase ph	no	no chan	0	COG0666(p
B1AUS0	B1AUS0_MNxf3		Nxf3		Nuclear RN	no	no chan	0	ENOG410X
Q3U3P6	Q3U3P6_MAdgrg3		Adgrg3	54672	G-protein c	no	up	0	ENOG410X
Q9EQS3	MYCBP_M Mycbp		Mycbp	56309	c-Myc-bind	no	no chan	0	ENOG4112
P51163	HEM4_MCUros		Uros	22276	Uroporphyı	no	no chan	0	COG1587(s
Q61009	SCRB1_MCScarb1		Scarb1	20778	Scavenger r	no	no chan	0	ENOG410X
O19455	O19455_MOUSE Major histocomp			14961	Major histo	no	no chan	0	ENOG410Y
Q61554	FBN1_MO Fbn1		Fbn1	14118	Fibrillin-1	no	down	0	ENOG410X
Q61555	FBN2_MO Fbn2		Fbn2	14119	Fibrillin-2	no	down	0	ENOG410X
P45952	ACADM_MAcadm		Acadm	11364	Medium-ch	no	no chan	0	COG1960(c
Q61550	RAD21_M(Rad21		Rad21	19357	Double-strc	no	no chan	0	ENOG410X
A2AA71	A2AA71_MSec24a		Sec24a	77371	SEC24 hom	no	no chan	0	COG5028(t
D3Z3B4	D3Z3B4_MNrf1		Nrf1		Nuclear res	no	no chan	0	ENOG410X
B1AT92	B1AT92_M Grb2		Grb2		Growth fac	no	no chan	0	ENOG410X
A0A0G2JE	A0A0G2JEAntxr2		Antxr2		Anthrax to	no	no chan	0	ENOG410Y
Q8K2Y9	CCM2_MOCcm2		Ccm2	216527	Cerebral ca	no	no chan	0	ENOG410Z
Q3U1J4	DDB1_MODdb1		Ddb1	13194	DNA dama	no	no chan	0	ENOG410X
Q3U912	Q3U912_MSphk2		Sphk2	56632	DAGKc do	no	no chan	0	COG1597(2
Q3UF95	Q3UF95_MBag6		Bag6	224727	Large proli	no	no chan	0	ENOG410X
O54790	MAFG_MCMafg		Mafg	17134	Transcripti	no	no chan	0	ENOG4111

Q5NCC3	TRI41_MO	Trim41	Trim41	211007	E3 ubiquiti	no	no chan	0	ENOG4111
Q08EK4	Q08EK4_M	Krt77	Krt77	406220	Keratin 77	no	up	0	ENOG4111
Q3UYA5	Q3UYA5_M	Atg4c	Atg4c	242557	Cysteine pr	no	no chan	0	ENOG410
Q8C163	EXOG_MO	Exog	Exog	208194	Nuclease E	no	no chan	0	COG1864(s
A0A0G2JE	A0A0G2JE	Stard3nl	Stard3nl	76205	STARD3 N	no	no chan	0	ENOG411
Q921W0	CHM1A_M	Chmp1a	Chmp1a	234852	Charged m	no	no chan	0	COG5491(
B2KGF0	B2KGF0_M	Rpe	Rpe	66646	Ribulose-p	no	no chan	0	COG0036(r
Q80V76	Q80V76_M	Pygo2	Pygo2	68911	Pygopus 2	no	no chan	0	ENOG4112
A0A0G2JE	A0A0G2JE	Slc35f6	Slc35f6		Solute carri	no	up	0	COG0697(c
Q05CR3	Q05CR3_M	Plec	Plec	18810	Plec1 prote	no	no chan	0	COG5069(
Q04690	NF1_MOU	Nf1	Nf1	18015	Neurofibro	no	no chan	0	ENOG410
Q04692	SMRCD_M	Smarcad1	Smarcad1	13990	SWI/SNF-r	no	no chan	0	ENOG410
F2Z3X6	F2Z3X6_M	Pdpk1	Pdpk1	18607	3-phospho	no	no chan	0	ENOG410
E9PW15	E9PW15_M	Suz12	Suz12	52615	SUZ12 poly	no	no chan	0	ENOG410
Q9D6N4	Q9D6N4_M	Ttn	Ttn	22138	Ig-like dom	no	no chan	0	ENOG410
Q3UJY3	Q3UJY3_M	Utp4	Utp4	21771	Uncharacte	no	no chan	0	ENOG410
P00329	ADH1_MO	Adh1	Adh1	11522; 1102	Alcohol de	no	no chan	0	COG1062(r
A0A0N4SV	A0A0N4SV	Dnajb8	Dnajb8		DnaJ heat s	no	up	0	COG0484(c
O08842	GFRA2_M	Gfra2	Gfra2	14586	GDNF fam	no	no chan	0	ENOG410
O08848	RO60_MO	RO60	RO60	20822	RNA-bindi	no	no chan	0	ENOG410
Q9QZM4	TR10B_MC	Tnfrsf10b	Tnfrsf10b	21933	Tumor necr	no	no chan	0	ENOG4111
E9QQ25	E9QQ25_M	Speg	Speg	11790	SPEG comp	no	up	0	ENOG410
Q9QZM0	UBQL2_M	Ubqln2	Ubqln2	54609	Ubiquilin-2	no	no chan	0	COG5272(s
B7ZND8	B7ZND8_M	Tex9	Tex9	21778	Testis expre	no	no chan	0	ENOG411
Q3THQ1	Q3THQ1_M	Spop	Spop	20747	Speckle-typ	no	down	0	ENOG410
Q6ZWN5	RS9_MOUS	Rps9	Rps9	76846	Small ribos	no	no chan	0	COG0522(
G3UYH0	G3UYH0_M	Foxa2	Foxa2		Forkhead b	no	no chan	0	COG5025(
Q3THQ5	Q3THQ5_M	Stip1	Stip1	20867	Stress-indu	no	no chan	0	ENOG410
Q3THQ7	Q3THQ7_M	Pold3	Pold3	67967	DNA polyn	no	no chan	0	ENOG410
Q8C570	RAE1L_MO	Rae1	Rae1	66679	mRNA exp	no	no chan	0	ENOG410
Q3UKC8	Q3UKC8_M	Gng5	Gng5	14707	Guanine nu	no	no chan	0	ENOG4112
Q0VGR5	Q0VGR5_M	Cdk5rap2	Cdk5rap2	214444	CDK5 regu	no	no chan	0	ENOG4112
P35428	HES1_MOU	Hes1	Hes1	15205	Transcripti	no	no chan	0	ENOG4111
Q8K382	DEN1A_M	Dennd1a	Dennd1a	227801	DENN dom	no	no chan	0	ENOG410
Q3UKC1	TAXB1_MO	Tax1bp1	Tax1bp1	52440	Tax1-bindi	no	no chan	0	ENOG410
Q8R123	FAD1_MO	Flad1	Flad1	319945	FAD synth	no	up	0	COG0175(
Q8K387	UBP45_MC	Usp45	Usp45	77593	Ubiquitin c	no	no chan	0	COG5560(
E0CY72	E0CY72_M	Alkbh1	Alkbh1		AlkB homo	no	no chan	0	COG3145(
E0CY73	E0CY73_M	Lrmda	Lrmda	76633	Leucine ric	no	up	0	COG4886(
P42703	LIFR_MOU	Lifr	Lifr	16880	Leukemia i	no	up	0	ENOG410
Q9D0D5	T2EA_MO	Gtf2e1	Gtf2e1	74197	General tra	no	no chan	0	COG1675(
Q542Y0	Q542Y0_M	Nqo1	Nqo1	18104	NAD(P)H c	no	no chan	0	COG2249(c
G3UWH9	G3UWH9_	Cog4	Cog4		Conserved	no	no chan	0	ENOG410

Q3U5N6	Q3U5N6_M	Ms4a4c	Ms4a4c	64380	Membrane-	no | up	0	ENOG4112
Q14A90	Q14A90_M	Dus2	Dus2	66369	Dihydrouri	no | no chan	0	COG0042(C
Q3U5N2	Q3U5N2_M	Ifit1	Ifit1	15957	Interferon-i	no | no chan	0	COG0457(r
Q14A95	Q14A95_M	Rbm7	Rbm7	67010	RNA bindi	no | no chan	0	ENOG4112
Q9CQF6	ADPPT_M	Aasdhppt	Aasdhppt	67618	L-aminoadi	no | no chan	0	COG2091(4
Q9CQF7	Q9CQF7_M	Pfdn1	Pfdn1	67199	Prefoldin s	no | no chan	0	ENOG4111
Q8VI33	TAF9_MOU	Taf9	Taf9	108143	Transcripti	no | no chan	0	COG5094(F
A0A172Q3	A0A172Q3	Fap	Fap	14089	Fibroblast	no | no chan	0	COG1506(E
Q91W61	FXL15_MC	Fbxl15	Fbxl15	68431	F-box/LRR	no | no chan	0	ENOG4111
Q5F271	Q5F271_M	Plek	Plek	56193	Pleckstrin	no | no chan	0	ENOG4102
Q148Q4	Q148Q4_M	Dhrs9	Dhrs9	241452	Dehydroger	no | up	0	ENOG410Y
Q3UPG3	Q3UPG3_M	Gpsm2	Gpsm2	76123	G-protein-s	no | no chan	0	ENOG410X
Q9D9G3	CHIC2_MC	Chic2	Chic2	74277	Cysteine-ric	no | down	0	ENOG4111
P31725	S10A9_MO	S100a9	S100a9	20202	Protein S10	no | up	0	ENOG4111
P11352	GPX1_MO	Gpx1	Gpx1	14775	Glutathion	no | no chan	0	COG0386(g
A0A1D5RL	A0A1D5RL	Kifc3	Kifc3	16582	Kinesin-lik	no | no chan	0	COG5059(E
E9PY43	E9PY43_M	Erich1	Erich1	234086	Glutamate	no | up	0	ENOG410Y
P97287	MCL1_MO	Mcl1	Mcl1	17210	Induced my	no | no chan	0	ENOG4112
Q9D1A2	CNDP2_M	Cndp2	Cndp2	66054	Cytosolic n	no | no chan	0	COG0624(s
P61327	MGN_MO	Magoh	Magoh	17149	Protein ma	no | no chan	0	ENOG4111
G3X9F6	G3X9F6_M	Sh3tc1	Sh3tc1	231147	SH3 domai	no | no chan	0	ENOG410Y
E9Q137	TX264_MC	Tex264	Tex264	21767	Testis-expr	no | no chan	0	ENOG4111
Q3TA88	Q3TA88_M	Sh3kbp1	Sh3kbp1	58194	SH3 domai	no | no chan	0	ENOG410X
Q641K1	CBPC1_MC	Agtpbp1	Agtpbp1	67269	Cytosolic c	no | up	0	COG2866(s
Q9DBG7	SRPRA_MC	Srpra	Srpra	67398	Signal reco	no | no chan	0	COG0552(I
Q9DBG5	PLIN3_MC	Plin3	Plin3	66905	Perilipin-3	no | no chan	0	ENOG410X
A0A087WQ	A0A087WQ	Msh3	Msh3	17686	DNA mism	no | no chan	0	COG0249(T
Q8R4Y8	RTTN_MO	Rttn	Rttn	246102	Rotatin	no | no chan	0	ENOG410X
P98192	GNPAT_M	Gnpat	Gnpat	14712	Dihydroxya	no | no chan	0	COG2937(S
P23611	IRF8_MOU	Irf8	Irf8	15900	Interferon r	no | no chan	0	ENOG410X
P54071	IDHP_MO	Idh2	Idh2	269951	Isocitrate d	no | no chan	0	COG0538(s
B2RR29	B2RR29_M	Ptpn7	Ptpn7	320139	protein-tyr	no | no chan	0	COG5599(N
Q3TT62	Q3TT62_M	St6galnac4	St6galnac4	20448	ST6 N-acet	no | no chan	0	ENOG410X
A0A1C7CY	A0A1C7CY	Pls3	Pls3		Plastin-3	no | no chan	0	COG5069(C
Q9CY34	UB2FA_MC	Ube2f	Ube2f	67921	NEDD8-co	no | no chan	0	ENOG410X
Q6P3Y5	Z280C_MC	Znf280c	Znf280c	208968	Zinc finger	no | no chan	0	COG5048(I
B2RR26	B2RR26_M	Nhlrc2	Nhlrc2	66866	NHL repea	no | no chan	0	ENOG410X
A0A1D5RL	A0A1D5RL	Pard6a	Pard6a		Par-6 famil	no | no chan	0	ENOG410X
Q9CQS2	NOP10_MC	Nop10	Nop10	66181	H/ACA rib	no | no chan	0	COG2260(r
Q8JZL3	THTPA_M	Thtpa	Thtpa	105663	Thiamine-t	no | no chan	0	ENOG4111
D3Z2A4	D3Z2A4_M	Rrm2b	Rrm2b		Ribonucleo	no | no chan	0	COG0208(S
Q64311	NTAN1_M	Ntan1	Ntan1	18203	Protein N-t	no | no chan	0	ENOG410X
Q64317	DLX1_MO	Dlx1	Dlx1	13390	Homeobox	no | no chan	0	ENOG4102

Q3TDR0	Q3TDR0_M	Kdsr	Kdsr	70750	3-dehydrosp	no	no chan	0	ENOG410Y
Q64318	ZEB1_MOU	Zeb1	Zeb1	21417	Zinc finger	no	no chan	0	ENOG4110
Q99JX4	EIF3M_MC	Eif3m	Eif3m	98221	Eukaryotic	no	no chan	0	ENOG410X
O35709	ENC1_MO	Enc1	Enc1	13803	Ectoderm-n	no	no chan	0	ENOG410X
Q9CX34	SGT1_MOU	Sugt1	Sugt1	67955	Protein SGT	no	no chan	0	COG5091(S
O35704	SPTC1_MC	Sptlc1	Sptlc1	268656	Serine palm	no	no chan	0	COG0156(E
P48722	HS74L_MC	Hspa4l	Hspa4l	18415	Heat shock	no	up	0	COG0443(p
A2APB8	TPX2_MOU	Tpx2	Tpx2	72119	Targeting p	no	no chan	0	ENOG4110
Q4VA53	PDS5B_MC	Pds5b	Pds5b	100710	Sister chron	no	no chan	0	ENOG410X
Q53VR2	Q53VR2_MOUSE VH region (Fragment) OS=M				VH region	no	no chan	0	ENOG410Z
Q4QY64	ATAD5_MC	Atad5	Atad5	237877	ATPase fam	no	no chan	0	ENOG410Y
Q8C1L7	Q8C1L7_MOUSE 40S ribosomal pr			66481	40S ribosom	no	no chan	0	ENOG4112
P31938	MP2K1_MC	Map2k1	Map2k1	26395	Dual specif	no	no chan	0	ENOG410X
Q8BM41	Q8BM41_M	Lsm14b	Lsm14b	241846	LSM family	no	up	0	ENOG4112
Q5U415	Q5U415_M	Akr1b1	Akr1b1	11677	Aldo-keto r	no	no chan	0	COG0656(r
Q5XJZ3	Q5XJZ3_M	Grwd1	Grwd1	101612	Glutamate-	no	no chan	0	ENOG410X
Q99KD5	UN45A_MC	Unc45a	Unc45a	101869	Protein unc	no	no chan	0	ENOG410X
A0A3B2W4	A0A3B2W4	Emilin2	Emilin2		Elastin mic	no	no chan	0	ENOG410Z
Q3TFH8	Q3TFH8_M	Pgm3	Pgm3	109785	Phosphoace	no	no chan	0	COG1109(I
P50172	DHI1_MOU	Hsd11b1	Hsd11b1	15483	11-beta-hyd	no	down	0	COG1028(S
Q80YA7	DPP8_MOU	Dpp8	Dpp8	74388	Dipeptidyl	no	no chan	0	COG1506(Z
E9PUE1	E9PUE1_M	R3hcc1	R3hcc1		R3H and cc	no	no chan	0	ENOG4110
P33267	CP2F2_MC	Cyp2f2	Cyp2f2	13107	Cytochrom	no	down	0	COG2124(M
E9PUE8	E9PUE8_M	Abcc9	Abcc9	20928	ATP-bindir	no	no chan	0	COG1132(c
A0A1L1SU	A0A1L1SU	Acp3	Acp3		acid phosph	yes	up	1	ENOG410Z
A0A0A0MC	A0A0A0MC	Ttc28	Ttc28		Tetratricop	no	no chan	0	COG0457(F
A0A0A0MC	A0A0A0MC	Krtcap2	Krtcap2	66059	Keratinocy	no	no chan	0	ENOG4111
Q5DU02	UBP22_MC	Usp22	Usp22	216825	Ubiquitin c	no	no chan	0	ENOG410X
A0A0A0MC	A0A0A0MC	Atg7	Atg7	74244	Ubiquitin-l	no	no chan	0	COG0476(s
Q8CE64	ZN276_MC	Znf276	Znf276	57247	Zinc finger	no	no chan	0	COG5048(I
Q9Z150	Q9Z150_M	Zbtb12	Zbtb12	193736	Zinc finger	no	no chan	0	COG5048(A
P15327	PMGE_MC	Bpgm	Bpgm	12183	Bisphospho	no	no chan	0	COG0588(C
Q8BMA5	NPAT_MO	Npat	Npat	244879	Protein NP	no	no chan	0	ENOG4111
A0A3B2WE	A0A3B2WE	Polr2m	Polr2m		Polymerase	no	up	0	
Q8BMA6	SRP68_MO	Srp68	Srp68	217337	Signal recog	no	no chan	0	ENOG410X
P30681	HMGB2_M	Hmgb2	Hmgb2	97165	High mobil	no	no chan	0	COG5648(C
Q8BH95	ECHM_MC	Echs1	Echs1	93747	Enoyl-CoA	no	no chan	0	COG1024(I
Q8R3P9	SLF1_MOU	Slf1	Slf1	105377	SMC5-SMC	no	no chan	0	ENOG410X
P70663	SPRL1_MC	Sparcl1	Sparcl1	13602	SPARC-like	no	no chan	0	ENOG4110
P70665	SIAE_MOU	Siae	Siae	22619	Sialate O-a	no	no chan	0	ENOG410X
Q5U4D9	THOC6_M	Thoc6	Thoc6	386612	THO comp	no	no chan	0	ENOG410X
Q99LB6	MAT2B_M	Mat2b	Mat2b	108645	Methionine	no	no chan	0	COG1091(I
P18406	CCN1_MO	Ccn1	Ccn1	16007	CCN family	no	no chan	0	ENOG4111

Q3TET5	Q3TET5_M	Pmpcb	Pmpcb	73078	Mitochondı	no | no chan	0	COG0612(I
G3UXW9	G3UXW9_	Gps1	Gps1	209318	G protein p	no | no chan	0	ENOG410X
Q9QZX7	SRR_MOU	Srr	Srr	27364	Serine racei	no | no chan	0	COG1171(I
Q9JKR6	HYOU1_M	Hyou1	Hyou1	12282	Hypoxia up	no | no chan	0	COG0443(C
Q5SYL3	BLTP2_MC	Bltp2	Bltp2	72503	Bridge-like	no | no chan	0	ENOG410X
Q8R3P6	INT14_MC	Ints14	Ints14	69882	Integrator c	no | no chan	0	ENOG410X
A0A0N4SV	A0A0N4SV	Chn2	Chn2		Chimerin 2	no | up	0	ENOG4111
Q8R3P0	ACY2_MO	Aspa	Aspa	11484	Aspartoacy	no | no chan	0	COG2988(M
P29341	PABP1_MC	Pabpc1	Pabpc1	18458	Polyadenyl	no | no chan	0	ENOG410X
P58742	AAAS_MO	Aaas	Aaas	223921	Aladin	no | no chan	0	ENOG410X
B2WTI1	B2WTI1_M	Jmjd6	Jmjd6	107817	Bifunctionc	no | no chan	0	ENOG410X
Q3U6Q1	Q3U6Q1_M	Wbp11	Wbp11	60321	WW domai	no | no chan	0	ENOG4111
Q8R5A6	TB22A_MC	Tbc1d22a	Tbc1d22a	223754	TBC1 domc	no | no chan	0	ENOG410X
Q9DC50	OCTC_MC	Crot	Crot	74114	Peroxisoma	no | no chan	0	ENOG410Z
Q9DC51	GNAI3_M	Gnai3	Gnai3	14679	Guanine nı	no | no chan	0	ENOG410X
Q9DC53	CPNE8_M	Cpne8	Cpne8	66871	Copine-8	no | down	0	ENOG410X
Q6P9P6	KIF11_MO	Kif11	Kif11	16551	Kinesin-lik	no | no chan	0	COG5059(I
P09528	FRIH_MO	Fth1	Fth1	14319	Ferritin hec	no | no chan	0	COG1528(S
Q3U6Q4	PI3R6_MO	Pik3r6	Pik3r6	104709	Phosphoinc	no | up	0	ENOG411C
E9PYL4	E9PYL4_M	Clcn7	Clcn7	26373	Chloride ch	no | no chan	0	COG0038(c
E9PYL2	PRR12_MC	Prr12	Prr12	233210	Proline-rich	no | no chan	0	ENOG410Y
A0JLQ7	A0JLQ7_M	Pcid2	Pcid2	234069	PCI domair	no | no chan	0	COG5600(I
Q8VC65	NRM_MO	Nrm	Nrm	106582	Nurim	no | no chan	0	ENOG4111
P49935	CATH_MC	Ctsh	Ctsh	13036	Pro-catheps	no | no chan	0	COG4870(M
B1AQZ2	B1AQZ2_M	Kif3a	Kif3a	16568	Kinesin-lik	no | no chan	0	COG5059(A
Q99N12	Q99N12_M	Dusp19	Dusp19	68082	Dual specif	no | up	0	COG2453(r
A0A1D5RM	A0A1D5RM	Scoc	Scoc		Short coilec	no | up	0	ENOG4111
Q99N11	DUS22_MC	Dusp22	Dusp22	105352	Dual specif	no | no chan	0	COG2453(c
Q61941	NNTM_M	Nnt	Nnt	18115	NAD(P) trc	no | no chan	0	COG1282(T
Q9JJZ2	TBA8_MO	Tuba8	Tuba8	53857	Tubulin alp	no | up	0	COG5023(T
Q3UIJ9	MYZAP_M	Myzap	Myzap	102371	Myocardial	no | down	0	ENOG410Z
P46412	GPX3_MO	Gpx3	Gpx3	14778	Glutathion	no | no chan	0	COG0386(I
Q8R2R3	AAGAB_M	Aagab	Aagab	66939	Alpha- and	no | no chan	0	ENOG410X
Q61768	KINH_MO	Kif5b	Kif5b	16573	Kinesin-1 h	no | no chan	0	COG5059(I
P46414	CDN1B_M	Cdkn1b	Cdkn1b	12576	Cyclin-depe	no | no chan	0	ENOG410X
A0A1W2P7	A0A1W2P7	Slc1a6	Slc1a6		Amino acid	no | up	0	COG1301(I
Q80WT5	AFTIN_M	Aftph	Aftph	216549	Aftiphilin	no | no chan	0	ENOG4111
A0A1W2P7	A0A1W2P7	H3c14	H3c14	15077	H3 clustere	no | no chan	0	COG2036(S
Q8K2J7	RELL1_MC	Rell1	Rell1	100532	RELT-like	no | no chan	0	ENOG4111
A2AM67	A2AM67_M	Sh2d5	Sh2d5	230863	Vascular SI	no | no chan	0	ENOG4111
A0AAQ4V	A0AAQ4VMV6_MOUSE	Transcription initiat	Taf1			no | no chan	0	COG5076(p
A0AAQ4V	A0AAQ4VMV9_MOUSE	LIM domain only 7 (	Lmo7			no | down	0	ENOG410Z
Q7TPR4	ACTN1_M	Actn1	Actn1	109711	Alpha-actir	no | no chan	0	COG5069(I

A0A1Y7VJ5	A0A1Y7VJ5	Cinp	Cinp		Cyclin depe	no | up	0	ENOG4111
P27612	PLAP_MOU	Plaa	Plaa	18786	Phospholip	no | no chan	0	ENOG410X
A0A494BBI	A0A494BBI	Pold4	Pold4	69745	Polymerase	no | up	0	ENOG410X
E9QLQ7	E9QLQ7_M	Rab29	Rab29	226422	Ras-related	no | down	0	ENOG410Y
E9QLQ3	E9QLQ3_M	Pde3b	Pde3b	18576	Phosphodie	no | no chan	0	ENOG410X
Q9WTV7	RNF12_MO	Rlim	Rlim	19820	E3 ubiquiti	no | down	0	ENOG4112
D3YUM1	D3YUM1_M	Ndufv1	Ndufv1		NADH deh	no | no chan	0	COG1894(N
A0A0A6YW	A0A0A6YW	Ankrd39	Ankrd39	109346	Ankyrin rep	yes | down	1	COG0666(i
Q8R3V5	SHLB2_MO	Sh3glb2	Sh3glb2	227700	Endophilin	no | no chan	0	ENOG410X
Q8R3V2	Q8R3V2_M	Aimp2	Aimp2	231872	Aminoacyl	no | up	0	ENOG4110
A0A087WQ	A0A087WQ	Casp8	Casp8	12370	Caspase 8	no | no chan	0	ENOG410Z
B9EHE8	B9EHE8_M	R3hdm1	R3hdm1	226412	R3hdm1 pro	no | no chan	0	ENOG410Z
A0A0B4J1F	A0A0B4J1F	Stoml1	Stoml1	69106	Stomatin-li	no | no chan	0	COG0330(u
A0A0B4J1F	A0A0B4J1F	Itga11	Itga11	319480	Integrin alp	no | up	0	ENOG410Y
P08121	CO3A1_MO	Col3a1	Col3a1	12825	Collagen al	no | no chan	0	ENOG410X
P62869	ELOB_MOU	Elob	Elob	67673	Elongin-B	no | no chan	0	ENOG4111
Q3TWG5	Q3TWG5_M	Dync1li1	Dync1li1	235661	Dynein ligh	no | no chan	0	ENOG410X
Q8BHX1	HAUS1_MO	Haus1	Haus1	225745	HAUS augm	no | no chan	0	ENOG4111
Q8BWT5	DIP2A_MO	Dip2a	Dip2a	64451	Disco-inter	no | no chan	0	COG0318(p
Q8BWT1	THIM_MOU	Acaa2	Acaa2	52538	3-ketoacyl-C	no | no chan	0	COG0183(p
Q3UDM3	Q3UDM3_M	Ptges	Ptges	64292	Prostagland	no | no chan	0	ENOG4111
P67778	PHB1_MOU	Phb1	Phb1	18673	Prohibitin 1	no | no chan	0	COG0330(F
A0A7N9VR	A0A7N9VR	Ahnak2	Ahnak2		AHNAK nu	no | no chan	0	ENOG410X
E0CXX6	E0CXX6_M	Mpnd	Mpnd		MPN doma	no | no chan	0	COG1310(C
Q8BHA0	IN80C_MO	Ino80c	Ino80c	225280	INO80 com	no | no chan	0	COG5195(I
Q8BHA3	DTD2_MOU	Dtd2	Dtd2	328092	D-aminoac	no | no chan	0	COG1490(F
Q62418	DBNL_MOU	Dbnl	Dbnl	13169	Drebrin-lik	no | no chan	0	ENOG410X
Q8BHA9	PXYP1_MO	Pxylp1	Pxylp1	235534	2-phosphox	no | no chan	0	ENOG410Y
A0A0U1RP	A0A0U1RP	Fes	Fes		Tyrosine-pr	no | no chan	0	ENOG410Y
Q9CZ09	MET18_MO	Mettl18	Mettl18	69962	Histidine pr	no | down	0	ENOG4111
D3Z742	D3Z742_M	Mb21d2	Mb21d2	239796	Mab-21 dor	no | no chan	0	ENOG410X
A0A498WC	A0A498WC	Nmrk1	Nmrk1		Nicotinami	no | up	0	COG0572(n
F8VPL2	F8VPL2_M	Pik3c2a	Pik3c2a	18704	Phosphatid	no | no chan	0	COG5032(F
Q2VPQ9	EAF6_MOU	Meaf6	Meaf6	70088	Chromatin	no | no chan	0	ENOG4111
Q8BGC0	HTSF1_MO	Htatsf1	Htatsf1	72459	17S U2 SnR	no | no chan	0	ENOG410Y
A0A0R4J29	A0A0R4J29	Tgm1	Tgm1	21816	Protein-glu	no | no chan	0	ENOG410X
A0A0R4J29	A0A0R4J29	Fastkd5	Fastkd5	380601	FAST kinas	no | no chan	0	ENOG4111
Q8R1G2	CMBL_MO	Cmbl	Cmbl	69574	Carboxyme	no | no chan	0	COG0412(c
A0A0R4J0X	A0A0R4J0X	Arhgef7	Arhgef7	54126	Rho guanin	no | up	0	ENOG410X
Q91ZR4	NEK8_MOU	Nek8	Nek8	140859	Serine/thre	no | no chan	0	ENOG410Y
A0A0R4J0X	A0A0R4J0X	Serpina1c	Serpina1c	20702	Serine (or c	no | no chan	0	COG4826(s
A0A0R4J0X	A0A0R4J0X	Alas1	Alas1	11655	5-aminolev	no | no chan	0	COG0156(p
E9Q9K8	E9Q9K8_M	Akap6	Akap6	238161	A kinase an	no | no chan	0	ENOG410X

P97346	NXN_MOU	Nxn	Nxn	18230	Nucleoredo	no|no chan	0	ENOG410Z
Q9JI78	NGLY1_M	Ngly1	Ngly1	59007	Peptide-N(4	no|no chan	0	ENOG410X
Q5F1Z8	Q5F1Z8_M	Gm2a	Gm2a	14667	Gm2a prote	no|no chan	0	ENOG4111
Q8CFI7	RPB2_MOU	Polr2b	Polr2b	231329	DNA-direc	no|no chan	0	COG0085(I
E9PXS4	E9PXS4_M	Ptp4a3	Ptp4a3	19245	Protein tyro	no|no chan	0	ENOG4111
Q3U4S1	Q3U4S1_M	Snx1	Snx1	56440	Sorting nex	no|no chan	0	COG5391(i
Q3TVJ8	Q3TVJ8_M	Ssr4	Ssr4	20832	Translocon	no|no chan	0	ENOG4111
Q3TVJ9	Q3TVJ9_M	B3gnt2	B3gnt2	53625	Hexosyltran	no|no chan	0	ENOG410Z
O09167	RL21_MOU	Rpl21	Rpl21	675278; 199	Large ribos	no|no chan	0	COG2139((
O09165	CASQ1_MO	Casq1	Casq1	12372	Calsequestr	no|up	0	ENOG4111
O09164	SODE_MO	Sod3	Sod3	20657	Extracellul	no|down	0	COG2032(g
Q99M71	EPDR1_MO	Epdr1	Epdr1	105298	Mammalian	no|no chan	0	ENOG4112
Q7TMQ7	WDR91_M	Wdr91	Wdr91	101240	WD repeat-	no|no chan	0	ENOG410X
Q571M5	Q571M5_M	Itch	Itch	16396	HECT-type	no|no chan	0	COG5021(I
Q3TLJ5	Q3TLJ5_M	Cdk5rap3	Cdk5rap3	80280	CDK5 regu	no|no chan	0	ENOG410X
Q3TLJ6	Q3TLJ6_M	Coq3	Coq3	230027	Ubiquinone	no|no chan	0	COG2227(N
Q2KHK6	GSDC2_MO	Gsdmc2	Gsdmc2	331063	Gasdermin-	no|up	0	ENOG4111
Q9DAR7	DCPS_MO	Dcps	Dcps	69305	m7GpppX	no|no chan	0	COG5075(I
Q9CR39	WIPI3_MO	Wdr45b	Wdr45b	66840	WD repeat	no|no chan	0	ENOG410X
Q922M3	BACD3_MO	Kctd10	Kctd10	330171	BTB/POZ d	no|no chan	0	ENOG410X
A0A5F8MP	A0A5F8MP	Exoc6	Exoc6		Exocyst cor	no|no chan	0	ENOG410X
O88935	SYN1_MOU	Syn1	Syn1	20964	Synapsin-1	no|up	0	ENOG410X
Q9JLI8	SART3_MO	Sart3	Sart3	53890	Squamous c	no|no chan	0	ENOG410X
Q09XV5	CHD8_MO	Chd8	Chd8	67772	Chromodor	no|no chan	0	COG0553(I
A0A5F8MP	A0A5F8MP	Agt	Agt		Angiotensir	no|up	0	COG4826(A
F8VQB6	MYO10_MO	Myo10	Myo10	17909	Unconventi	no|no chan	0	COG5022(C
A0A6B9EQ	A0A6B9EQ	Ppp3cb	Ppp3cb	19056	Serine/thre	no|no chan	0	COG0639(g
A0A0R4J1F	A0A0R4J1F	Wdhd1	Wdhd1	218973	WD repeat	no|no chan	0	ENOG410X
P04925	PRIO_MOU	Prnp	Prnp	19122	Major prior	no|no chan	0	ENOG410Y
Q78IK2	ATPMK_M	Atp5mk	Atp5mk	66477	ATP syntha	no|no chan	0	ENOG410X
P62331	ARF6_MOU	Arf6	Arf6	11845	ADP-ribosy	no|no chan	0	ENOG410X
P62334	PRS10_MO	Psmc6	Psmc6	67089	26S proteas	no|no chan	0	COG1222(S
Q78IK4	MIC27_MO	Apool	Apool	68117	MICOS con	no|no chan	0	ENOG4112
Q3UJ44	Q3UJ44_M	Capg	Capg	12332	Gelsolin-lik	no|no chan	0	ENOG410X
Q922M5	CDA7L_MO	Cdca7l	Cdca7l	217946	Cell divisio	no|no chan	0	ENOG410X
Q0VBK2	K2C80_MO	Krt80	Krt80	74127	Keratin, typ	yes|up	1	ENOG4111
Q0VBK8	Q0VBK8_M	Cdk6	Cdk6	12571	Cyclin-depe	no|no chan	0	ENOG410X
A0A1L1SR9	A0A1L1SR9	Ano8	Ano8		Anoctamin	no|up	0	ENOG410X
B2RSM5	B2RSM5_M	Afap1l1	Afap1l1	106877	Actin filam	no|no chan	0	ENOG4111
Q60992	VAV2_MO	Vav2	Vav2	22325	Guanine nu	no|no chan	0	ENOG410X
Q6DID7	WLS_MOU	Wls	Wls	68151	Protein wnt	no|no chan	0	ENOG410X
Q6DID5	PWP3A_MO	Pwwp3a	Pwwp3a	68114	PWWP dom	no|no chan	0	ENOG4111
Q14AI6	RUSD3_MO	Rpusd3	Rpusd3	101122	Mitochondr	no|no chan	0	COG0564(C

P41133	ID3_MOUS	Id3	Id3	15903	DNA-bindi	no|down	0	ENOG4112
I7ENE7	I7ENE7_M	Uchl1	Uchl1	22223	Ubiquitin c	yes|down	1	ENOG4111
Q3TXT7	Q3TXT7_M	Ruvbl2	Ruvbl2	20174	RuvB-like h	no|no chan	0	COG1224(p
A0A0J9YU(	A0A0J9YU(	Mettl27	Mettl27		Methyltrans	no|no chan	0	COG0500(r
Q3TML6	Q3TML6_M	Eif2s3x	Eif2s3x	26905	protein-syn	no|no chan	0	COG5257(t
Q5U628	Q5U628_M	Rab2b	Rab2b	76338	RAB2B, me	no|no chan	0	COG1100(I
Q6ZQK4	Q6ZQK4_M	Ppp2r5c	Ppp2r5c	26931	MKIAA004	yes|up	1	ENOG410X
E9Q092	E9Q092_M	Habp2	Habp2		Hyaluronic	no|up	0	COG5640(z
A2A6A1	GPTC8_MO	Gpatch8	Gpatch8	237943	G patch do	no|no chan	0	ENOG410Z
Q8BK77	Q8BK77_M	Tor1aip2	Tor1aip2	240832	Uncharacte	no|no chan	0	ENOG4111
P30276	CCNB2_MO	Ccnb2	Ccnb2	12442	G2/mitotic	no|no chan	0	COG5024(A
Q6P5U7	NWD2_MO	Nwd2	Nwd2	319807	NACHT an	no|no chan	0	COG2319(I
P30275	KCRU_MO	Ckmt1	Ckmt1	12716	Creatine ki	no|up	0	COG3869(A
Q61025	IFT20_MO	Ift20	Ift20	55978	Intraflagell	no|down	0	ENOG4111
B2RRW5	B2RRW5_M	Fem1c	Fem1c	240263	Fem-1 hom	no|no chan	0	COG0666(Z
F8WHT3	F8WHT3_M	Prrc2b	Prrc2b	227723	Proline-rich	no|no chan	0	ENOG411(
F8WHT2	F8WHT2_M	Ssh1	Ssh1	231637	protein-seri	no|no chan	0	COG2453(S
A0A2I3BPY	A0A2I3BPY	Rai14	Rai14	75646	Retinoic ac	no|no chan	0	COG0666(I
Q9Z2C4	MTMR1_M	Mtmr1	Mtmr1	53332	Phosphatid	no|no chan	0	ENOG410X
Q8K205	Q8K205_M	Pop1	Pop1	67724	Processing	no|no chan	0	ENOG410X
B1ASK8	B1ASK8_M	Ube2j2	Ube2j2	140499	E2 ubiquiti	no|no chan	0	ENOG410Z
Q14B21	Q14B21_M	Mrpl9	Mrpl9	78523	Large ribos	no|no chan	0	ENOG4111
Q8BIZ6	SNIP1_MO	Snip1	Snip1	76793	Smad nucle	no|no chan	0	ENOG4111
P37040	NCPR_MO	Por	Por	18984	NADPH--c	no|no chan	0	COG0369(C
Q8C0Y2	Q8C0Y2_M	Add2	Add2	11519	Class II ald	no|no chan	0	COG0235(C
A0A0M3KI	A0A0M3KL49_MOUSE	Fab4201 heavy chain (			Fab4201 he	no|no chan	0	ENOG4111
Q3USX5	Q3USX5_M	Irak4	Irak4	266632	Interleukin-	no|no chan	0	COG0515(s
Q9D3B1	HACD2_M	Hacd2	Hacd2	70757	Very-long-c	no|no chan	0	COG5198(I
Q8C6E0	CFA36_MO	Cfap36	Cfap36	216618	Cilia- and f	no|no chan	0	ENOG4111
P49722	PSA2_MOU	Psma2	Psma2	19166	Proteasome	no|no chan	0	ENOG410X
Q3U026	Q3U026_M	Mogs	Mogs	57377	Mannosyl-c	no|no chan	0	ENOG410X
Q3U024	Q3U024_M	Txndc11	Txndc11	106200	Thioredoxi	no|no chan	0	COG0526(r
Q0VDQ8	Q0VDQ8_M	Klhdc1	Klhdc1	271005	Kelch dom	no|no chan	0	ENOG410Y
Q8R2V2	IFFO2_MO	Iffo2	Iffo2	212632	Intermedia	no|no chan	0	COG1012(c
P60867	RS20_MOU	Rps20	Rps20	67427	Small ribos	no|no chan	0	COG0051(e
Q03137	EPHA4_MO	Epha4	Epha4	13838	Ephrin type	no|no chan	0	COG0515(C
Q99PH4	Q99PH4_M	Aldh3a2	Aldh3a2	11671	Aldehyde d	no|no chan	0	COG1012(I
A0A2I3BR(	A0A2I3BR(	Adcy6	Adcy6	11512	Adenylate c	no|no chan	0	COG2114(N
Q9D6H2	IFT25_MO	Ift25	Ift25	72938	Intraflagell	no|no chan	0	ENOG4111
P50580	PA2G4_MO	Pa2g4	Pa2g4	18813	Proliferatio	no|no chan	0	COG0024(I
Q8VC90	ZDH12_MO	Zdhhc12	Zdhhc12	66220	Palmitoyltr	no|no chan	0	COG5273(V
A2AMH4	A2AMH4_M	Slc44a1	Slc44a1	100434	Choline tra	no|no chan	0	ENOG410X
Q3U6U7	Q3U6U7_M	Wars1	Wars1	22375	Tryptophan	no|up	0	COG0180(t

J3QNE8	J3QNE8_M	Slc38a10	Slc38a10		Solute carri	no|no chan	0	COG0814(F
Q64105	SPRE_MOU	Spr	Spr	20751	Sepiapterin	no|no chan	0	ENOG4111
Q8K2V6	IPO11_MO	Ipo11	Ipo11	76582	Importin-11	no|no chan	0	COG5657(c
Q7TPV0	Q7TPV0_M	Gtf2h2	Gtf2h2	23894	General tra	no|no chan	0	COG5151(T
Q3TJQ2	Q3TJQ2_M	Slc35b2	Slc35b2	73836	Adenosine 3	no|no chan	0	COG0697(G
Q9DA69	IFT43_MO	Ift43	Ift43	76411	Intraflagell	no|no chan	0	ENOG4111
E9Q8L9	E9Q8L9_M	Rab11fip1	Rab11fip1	75767	RAB11 fam	no|no chan	0	ENOG410Z
O35066	KIF3C_MC	Kif3c	Kif3c	16570	Kinesin-like	no|no chan	0	COG5059(s
Q9EQQ2	YIPF5_MO	Yipf5	Yipf5	67180	Protein YIP	no|no chan	0	COG5080(y
A0A1Y7VM	A0A1Y7VM	Vrk1	Vrk1		non-specific	no|no chan	0	ENOG410X
A0A1G5SJJ	A0A1G5SJJ	OTTMUSP	OTTMUSP	15944	Irgm1	no|up	0	ENOG4111
P57759	ERP29_MC	Erp29	Erp29	67397	Endoplasm	no|no chan	0	ENOG4111
Q99J93	IFM2_MOU	Ifitm2	Ifitm2	80876	Interferon-i	no|no chan	0	ENOG4112
Q3TW96	UAP1L_MC	Uap1l1	Uap1l1	227620	UDP-N-ace	no|no chan	0	COG4284(p
Q3TW90	Q3TW90_M	Psmd8	Psmd8	57296	26S proteas	no|no chan	0	ENOG410X
E3UVT8	E3UVT8_M	Slc4a7	Slc4a7	218756	Anion exch	no|no chan	0	ENOG410X
Q9DB91	MED29_M	Med29	Med29	67224	Mediator of	no|no chan	0	ENOG4111
Q9DB90	SMG9_MO	Smg9	Smg9	71997	Nonsense-m	no|no chan	0	ENOG410X
A0A0A6YX	A0A0A6YX	Naa15	Naa15		N(alpha)-ac	no|no chan	0	ENOG410X
Q9DB96	NGDN_MC	Ngdn	Ngdn	68966	Neuroguidi	no|no chan	0	ENOG4111
Q9EQH4	TAF8_MOU	Taf8	Taf8	63856	Transcripti	no|no chan	0	ENOG410Z
Q9EQH2	ERAP1_MC	Erap1	Erap1	80898	Endoplasm	no|no chan	0	COG0308(c
A2AQ07	TBB1_MOU	Tubb1	Tubb1	545486	Tubulin bet	no|no chan	0	COG5023(T
E9PWF0	E9PWF0_M	Thbs3	Thbs3		Thrombosp	no|no chan	0	ENOG410X
Q99J95	CDK9_MO	Cdk9	Cdk9	107951	Cyclin-depe	no|no chan	0	ENOG410X
Q149H7	Q149H7_M	Cenpu	Cenpu	71876	Centromere	no|no chan	0	ENOG410Z
P29758	OAT_MOU	Oat	Oat	18242	Ornithine a	no|no chan	0	COG4992(A
Q8BG17	NOL12_MC	Nol12	Nol12	97961	Nucleolar p	no|no chan	0	ENOG410X
Q8BG15	CTSL2_MC	Ctdspl2	Ctdspl2	329506	CTD small	no|no chan	0	COG5190(T
Q8BYB9	PGLT1_MC	Poglut1	Poglut1	224143	Protein O-g	no|no chan	0	ENOG410X
A0A571BG	A0A571BG	Abr	Abr		Active brea	no|no chan	0	ENOG410X
A0A3Q4EB	A0A3Q4EB	Thumpd2	Thumpd2		THUMP do	no|no chan	0	COG1041(F
Q3UW40	Q3UW40_M	Rpl24	Rpl24	68193	Large ribos	no|no chan	0	COG2075(v
P70343	CASP4_MC	Casp4	Casp4	12363	Caspase-4	no|up	0	ENOG410Z
P70340	SMAD1_M	Smad1	Smad1	17125	Mothers ag	no|no chan	0	ENOG410X
P31324	KAP3_MO	Prkar2b	Prkar2b	19088	cAMP-depe	no|no chan	0	COG0664(k
P70349	HINT1_MC	Hint1	Hint1	15254	Adenosine 5	no|no chan	0	COG0537(v
Q8C108	Q8C108_M	Polr3f	Polr3f	70408	DNA-direc	no|no chan	0	COG5111(c
Q9ES28	ARHG7_M	Arhgef7	Arhgef7	54126	Rho guanin	no|no chan	0	ENOG410X
Q61686	CBX5_MO	Cbx5	Cbx5	12419	Chromobox	no|no chan	0	ENOG4111
Q61687	ATRX_MO	Atrx	Atrx	22589	Transcripti	no|no chan	0	COG0553(c
Q5NCM9	Q5NCM9_I	B9d1	B9d1		B9 domain-	yes|down	1	ENOG410X
A2ASA8	IPIL1_MOU	Itpripl1	Itpripl1	73338	Inositol 1,4,	no|no chan	0	ENOG4111

Q8CGN4	BCOR_MO	Bcor	Bcor	71458	BCL-6 core	no | no chan	0	ENOG410Y
Q544E8	Q544E8_M	Rab37	Rab37	58222	Rab37	no | no chan	0	ENOG410Z
O88708	ORC4_MO	Orc4	Orc4	26428	Origin reco	no | no chan	0	ENOG410X
Q91XV3	BASP1_MC	Basp1	Basp1	70350	Brain acid s	yes | down	1	ENOG4112
Q3TYS2	CYBC1_M(	Cybc1	Cybc1	217370	Cytochrom	no | no chan	0	ENOG4111
Q921Y4	MFSD5_M	Mfsd5	Mfsd5	106073	Molybdate-	no | up	0	ENOG410X
Q8BY71	HAT1_MO	Hat1	Hat1	107435	Histone ace	no | no chan	0	ENOG410X
P28665	MUG1_MC	Mug1	Mug1	17836	Murinoglob	no | down	0	COG2373(c
P28666	MUG2_MC	Mug2	Mug2	17837	Murinoglob	no | no chan	0	ENOG410X
P28667	MRP_MOU	Marcksl1	Marcksl1	17357	MARCKS-r	no | no chan	0	ENOG410Z
Q9WTX6	CUL1_MO	Cul1	Cul1	26965	Cullin-1	no | no chan	0	COG5647(F
O89079	COPE_MO	Cope	Cope	59042	Coatomer s	no | no chan	0	ENOG410X
Q91XY9	Q91XY9_M	Pcdhga10	Pcdhga10	93722	Protocadhe	no | no chan	0	ENOG410X
Q9QZ06	TOLIP_MC	Tollip	Tollip	54473	Toll-interac	no | no chan	0	ENOG410X
A0A0R4J19	A0A0R4J19	Bst1	Bst1	12182	ADP-ribosy	no | up	0	ENOG4111
A0A0R4J19	A0A0R4J19	Rprd1b	Rprd1b	70470	Regulation	no | no chan	0	ENOG410X
A0A0R4J19	A0A0R4J19	Spag9	Spag9	70834	Sperm asso	no | no chan	0	ENOG410X
P04104	K2C1_MOU	Krt1	Krt1	16678	Keratin, typ	no | no chan	0	ENOG410Y
A0A3Q4L2	A0A3Q4L2	Nubp2	Nubp2		Nucleotide	no | no chan	0	COG0489(C
Q66JR8	Q66JR8_M	Ptms	Ptms		Eukaryotic	no | no chan	0	COG0231(I
P26952	IL3RA_MC	Il3ra	Il3ra	16188	Interleukin	no | no chan	0	ENOG4111
Q3UYC0	PPM1H_M	Ppm1h	Ppm1h	319468	Protein pho	no | no chan	0	ENOG410X
Q8C142	ARH_MOU	Ldlrap1	Ldlrap1	100017	Low density	no | no chan	0	ENOG410Z
G3UX26	G3UX26_M	Vdac2	Vdac2		Voltage-dep	no | no chan	0	ENOG410Z
O08797	O08797_M	Serpinb9	Serpinb9	20723	SPI6	no | up	0	COG4826(s
O08796	EF2K_MO	Eef2k	Eef2k	13631	Eukaryotic	no | no chan	0	ENOG410Y
O08795	GLU2B_M	Prkcsh	Prkcsh	19089	Glucosidas	no | no chan	0	ENOG410X
Q3TKQ3	Q3TKQ3_M	Exosc2	Exosc2	227715	Uncharacte	no | no chan	0	COG1097(F
A0A0N4SV	A0A0N4SV	Amn1	Amn1		Antagonist	no | no chan	0	ENOG410Z
Q3U0J6	Q3U0J6_M	Mutyh	Mutyh	70603	Adenine DI	no | no chan	0	COG1194(c
Q3U0J8	TBD2B_M	Tbc1d2b	Tbc1d2b	67016	TBC1 doma	no | no chan	0	COG5210(c
H3BJB4	H3BJB4_M	Rnf141	Rnf141	67150	RING finge	no | no chan	0	ENOG410X
Q3THS2	Q3THS2_M	Wdr75	Wdr75	73674	WD repeat-	no | no chan	0	ENOG410Y
A0AAQ4VI	A0AAQ4VMY7_MOUSE	Dihydropyrimidinase	Dpysl2			no | no chan	0	COG0044(r
Q8K4P0	WDR33_M	Wdr33	Wdr33	74320	pre-mRNA	no | no chan	0	COG2319(I
Q3THS6	METK2_M	Mat2a	Mat2a	232087	S-adenosylr	no | no chan	0	COG0192(F
Q80V94	AP4E1_MC	Ap4e1	Ap4e1	108011	AP-4 compl	no | no chan	0	ENOG410X
Q9WV89	STXB4_MC	Stxbp4	Stxbp4	20913	Syntaxin-bi	no | no chan	0	ENOG411C
A2BE93	A2BE93_M	Set	Set		SET nuclea	no | no chan	0	ENOG410X
Q80ZI6	LRSM1_M	Lrsam1	Lrsam1	227738	E3 ubiquiti	no | no chan	0	COG4886(r
Q80ZI7	Q80ZI7_M	Igh	Igh	16061	Igh protein	no | up	0	ENOG410Z
Q0VGP5	Q0VGP5_M	Lnx2	Lnx2	140887	Ligand of n	no | no chan	0	ENOG410Y
P36916	GNL1_MO	Gnl1	Gnl1	14670	Guanine nu	no | no chan	0	COG1161(C

Q62084	PP14B_MC	Ppp1r14b	Ppp1r14b	18938	Protein phc	no	no chan	0	ENOG4111
Q62087	PON3_MO	Pon3	Pon3	269823	Serum para	no	no chan	0	ENOG4111
Q62086	PON2_MO	Pon2	Pon2	330260	Serum para	no	no chan	0	ENOG4111
A2BH40	ARI1A_MC	Arid1a	Arid1a	93760	AT-rich int	no	no chan	0	ENOG410Y
A0A0J9YU	A0A0J9YU	Ccnd2	Ccnd2		Cyclin D2	no	no chan	0	ENOG410X
Q66JX5	CEP43_MC	Cep43	Cep43	75296	Centrosome	no	no chan	0	ENOG4111
E9QNG6	E9QNG6_I	Snx13	Snx13	217463	Sorting nex	no	no chan	0	ENOG410X
Q8R2Y0	ABHD6_M	Abhd6	Abhd6	66082	Monoacylgl	no	no chan	0	ENOG410X
A0A8Q0KE	A0A8Q0KE	Cibar1	Cibar1	68099	CBY1 inter	no	no chan	0	ENOG410Z
Q3UDW8	HGNAT_M	Hgsnat	Hgsnat	52120	Heparan-al	no	no chan	0	COG4299(I
Q3V100	Q3V100_M	Mpi	Mpi	110119	Mannose-6-	no	no chan	0	COG1482(r
H3BL81	H3BL81_M	Chtf8	Chtf8		CTF8, chro	no	no chan	0	ENOG4111
F6YVP7	F6YVP7_M	Rps18-ps6	Rps18-ps6		Small ribos	no	no chan	0	COG0099(I
Q5F259	AN13B_MC	Ankrd13b	Ankrd13b	268445	Ankyrin rep	no	no chan	0	ENOG410X
Q5F258	Q5F258_M	Git1	Git1		GIT ArfGA	no	no chan	0	ENOG410X
Q812A5	PRR5_MOI	Prr5	Prr5	109270	Proline-rich	no	no chan	0	ENOG410X
Q3UA06	PCH2_MO	Trip13	Trip13	69716	Pachytene c	no	no chan	0	COG0464(s
E9QK62	E9QK62_M	Ngef	Ngef	53972	Neuronal g	no	up	0	COG5422(N
Q9JHI2	ADAT1_M	Adat1	Adat1	30947	tRNA-speci	no	no chan	0	ENOG410X
B2RWW2	B2RWW2_I	Golgb1	Golgb1	224139	Golgb1 pro	no	no chan	0	ENOG410X
Q91Z92	B3GT6_MC	B3galt6	B3galt6	117592	Beta-1,3-ga	no	no chan	0	ENOG410X
Q91Z96	BMP2K_M	Bmp2k	Bmp2k	140780	BMP-2-indu	no	no chan	0	ENOG410Y
P26043	RADI_MO	Rdx	Rdx	19684	Radixin	no	no chan	0	ENOG410X
P26040	EZRI_MOI	Ezr	Ezr	22350	Ezrin	no	no chan	0	ENOG410X
P26041	MOES_MC	Msn	Msn	17698	Moesin	no	no chan	0	ENOG410X
Q9D1C2	CBY1_MOI	Cby1	Cby1	73739	Protein chil	no	no chan	0	ENOG4111
L0CL36	L0CL36_M	Tlr4	Tlr4	21898	Toll-like re	no	no chan	0	COG4886(p
E9Q150	E9Q150_M	Dvl3	Dvl3		Dishevelled	no	no chan	0	ENOG410Y
Q3TQY2	Q3TQY2_M	Dctn4	Dctn4	67665	Dynactin su	no	no chan	0	ENOG410Z
Q564E8	Q564E8_M	Rpl4	Rpl4	67891	Large ribos	no	no chan	0	COG0088(C
A0A087WQ	A0A087WQ	Pign	Pign	27392	GPI ethanc	no	no chan	0	COG1524(S
D3YZZ5	D3YZZ5_M	Tmed7	Tmed7	66676	Transmemb	no	no chan	0	ENOG4111
P54728	RD23B_MC	Rad23b	Rad23b	19359	UV excisior	no	no chan	0	COG5272(s
Q9DBA6	TYSD1_MC	Tysnd1	Tysnd1	71767	Peroxisoma	no	no chan	0	COG0265(N
B2RR42	B2RR42_M	Dzip1l	Dzip1l	72507	DAZ intera	no	down	0	ENOG4111
Q9CY16	RT28_MOI	Mrps28	Mrps28	66230	Small ribos	no	no chan	0	ENOG4111
Q3UNC0	Q3UNC0_I	Gmppa	Gmppa	69080	Mannose-1-	no	no chan	0	COG1208(r
E9PZ97	E9PZ97_M	Blm	Blm	12144	DNA 3'-5' h	no	no chan	0	COG0514(c
E9Q971	E9Q971_M	Yju2b	Yju2b		Probable sp	no	no chan	0	COG5134(C
Q3TZ34	Q3TZ34_M	Inpp5e	Inpp5e	64436	Inositol pol	no	no chan	0	COG5411(C
A0A0G2JD	A0A0G2JD	Anapc5	Anapc5	59008	Anaphase-p	no	no chan	0	ENOG410Y
Q8K0G5	EIPR1_MC	Eipr1	Eipr1	380752	EARP and	no	no chan	0	ENOG410X
F7AVN4	F7AVN4_M	Hmg20b	Hmg20b		High mobil	no	no chan	0	COG5648(C

Q3TDT0	Q3TDT0_M	Trim3	Trim3	55992	RING-type	no	no chan	0	ENOG410
Q3TI69	Q3TI69_M	Apobec3	Apobec3	80287	single-stran	no	down	0	ENOG4111
Q3UC32	Q3UC32_M	Arpc5	Arpc5	67771	Actin-relat	no	no chan	0	ENOG4111
Q3TI65	Q3TI65_M	Mtfp1	Mtfp1	67900	Mitochond	no	up	0	ENOG4111
Q8BM39	PRP18_MC	Prpf18	Prpf18	67229	Pre-mRNA	no	no chan	0	ENOG410
Q3TI62	Q3TI62_M	Cct6a	Cct6a	12466	T-complex	no	no chan	0	ENOG410
P97406	P97406_MC	D17H6S56E	D17H6S56E	110956	Envelope p	no	no chan	0	
E9Q3G8	NU153_MC	Nup153	Nup153	218210	Nucleopori	no	no chan	0	ENOG411
E0CXE7	E0CXE7_M	Ctc1	Ctc1	68964	CST compl	no	no chan	0	ENOG410
E9Q3G7	E9Q3G7_M	Atp11a	Atp11a	50770	Phospholip	no	no chan	0	COG0474(c
D3YTL1	D3YTL1_M	Dnal1	Dnal1		Dynein, ax	no	no chan	0	COG4886(p
Q9CYZ6	REX1B_MC	Rex1bd	Rex1bd	66462	Required fc	no	no chan	0	ENOG4111
Q02013	AQP1_MO	Aqp1	Aqp1	11826	Aquaporin-	no	no chan	0	COG0580(I
Q9CYZ2	TPD54_MC	Tpd52l2	Tpd52l2	66314	Tumor prot	no	no chan	0	ENOG4111
Q91YT7	YTHD2_M	Ythdf2	Ythdf2	213541	YTH domai	no	no chan	0	ENOG410
Q9JJV2	PROF2_MC	Pfn2	Pfn2	18645	Profilin-2	no	no chan	0	ENOG411
A2AU61	A2AU61_M	Raly	Raly		HnRNP-ass	no	no chan	0	COG0724(C
Q545N8	Q545N8_M	Pmm2	Pmm2	54128	Phosphomo	no	no chan	0	COG0561(.
Q9QUG9	GRP2_MO	Rasgrp2	Rasgrp2	19395	RAS guanyl	no	no chan	0	ENOG410
A0A571BD	A0A571BD	Nav3	Nav3		Neuron nav	no	no chan	0	ENOG410
Q9QUG2	POLK_MC	Polk	Polk	27015	DNA polyn	no	no chan	0	COG0389(I
O88845	AKA10_MC	Akap10	Akap10	56697	A-kinase ar	no	no chan	0	ENOG410
P23336	GGTA1_M	Ggta1	Ggta1	14594	N-acetyllac	no	up	0	ENOG4111
Q9ESW4	AGK_MOI	Agk	Agk	69923	Acylglycero	no	no chan	0	COG1597(e
Q640Q5	PAN3_MO	Pan3	Pan3	72587	PAN2-PAN	no	no chan	0	ENOG410
Q5U472	Q5U472_M	Igh	Igh		Igh protein	no	no chan	0	ENOG410
Q5U3L0	Q5U3L0_M	Sh3bp2	Sh3bp2	24055	SH3-domai	no	no chan	0	ENOG4111
P50114	S100B_MO	S100b	S100b	20203	Protein S10	no	no chan	0	ENOG411
Q3TFF0	Q3TFF0_M	Dnaja2	Dnaja2	56445	DnaJ homo	no	no chan	0	COG0484(r
F6WMJ3	F6WMJ3_M	Arhgef6	Arhgef6	73341	Rac/Cdc42	no	no chan	0	ENOG410
B2RX66	B2RX66_M	Taok1	Taok1	216965	non-specifi	no	no chan	0	ENOG410
E9Q296	E9Q296_M	Acox3	Acox3		Acyl-coenz	no	no chan	0	COG1960(i
Q9CRC6	BORC7_MC	Borcs7	Borcs7	66439	BLOC-1-rel	no	no chan	0	ENOG410
A2AEG2	A2AEG2_M	Ofd1	Ofd1	237222	OFD1, cent	no	down	0	ENOG4111
Q9JL35	HMGN5_M	Hmgn5	Hmgn5	50887	High mobil	no	no chan	0	ENOG410
Q60GT3	Q60GT3_M	Galnt12	Galnt12	230145	Polypeptid	no	no chan	0	ENOG410
A0A0A0M	A0A0A0M	A430005L1	A430005L14Rik		RIKEN cD	no	no chan	0	ENOG410
P16374	ZFP60_MC	Zfp60	Zfp60	22718	Zinc finger	no	no chan	0	COG5048(Z
Q9D937	CK098_MOUSE	Uncharacterized p		66276	Uncharacte	no	no chan	0	ENOG410
P15306	TRBM_MC	Thbd	Thbd	21824	Thrombom	no	up	0	ENOG410
Q9Z130	HNRDL_M	Hnrnpdl	Hnrnpdl	50926	Heterogene	no	no chan	0	ENOG4111
Q7TML3	S35F2_MO	Slc35f2	Slc35f2	72022	Solute carri	no	down	0	COG0697(T
Q52KP0	Q52KP0_M	Rpl38	Rpl38	67671; 6256	Large ribos	no	no chan	0	ENOG411

E0CYA8	E0CYA8_M	Tmem120b	Tmem120b		Transmemb	no | no chan	0	ENOG410X
E0CYA9	E0CYA9_M	Pole4	Pole4		Polymerase	yes | down	1	ENOG4112
E0CYA3	E0CYA3_M	Ephb3	Ephb3		Ephrin type	no | no chan	0	COG0515(F
A0A9S4GT	A0A9S4GT	Zcchc14	Zcchc14		Zinc finger,	no | no chan	0	ENOG410X
O35988	SDC4_MOU	Sdc4	Sdc4	20971	Syndecan-4	no | no chan	0	ENOG4111
Q2KHP2	Q2KHP2_M	Mmp19	Mmp19	58223	Matrix meta	yes | up	1	ENOG410X
Q8VCL2	SCO2_MOU	Sco2	Sco2	100126824	Protein SCO	no | no chan	0	COG1999(r
A2A9I0	A2A9I0_MO	Gosr2	Gosr2	56494	Golgi SNAR	no | no chan	0	ENOG4111
O35984	PBX2_MOU	Pbx2	Pbx2	18515	Pre-B-cell l	no | no chan	0	ENOG410X
Q9QXY6	EHD3_MO	Ehd3	Ehd3	57440	EH domain	no | no chan	0	ENOG410X
Q07797	LG3BP_MO	Lgals3bp	Lgals3bp	19039	Galectin-3-	no | no chan	0	ENOG410Y
A0A0N4SV	A0A0N4SV	Klrb1b	Klrb1b		Killer cell l	no | no chan	0	ENOG410X
O55091	IMPCT_MO	Impact	Impact	16210	Protein IMP	no | no chan	0	COG1739(p
A2AIS9	A2AIS9_MO	Arrdc1	Arrdc1		Arrestin do	no | no chan	0	ENOG4110
Q3TZX8	NOL9_MO	Nol9	Nol9	74035	Polynucleot	no | no chan	0	COG1341(N
D3YXH8	D3YXH8_M	Itgb2l	Itgb2l	16415	Integrin bet	no | up	0	ENOG410X
D3YXH9	D3YXH9_M	Tatdn3	Tatdn3		TatD DNas	no | down	0	COG0084(z
Q6RUT7	UQCC4_MO	Uqcc4	Uqcc4	214489	Ubiquinol-c	no | up	0	ENOG410X
A0A1W2P7	A0A1W2P7	Pcbd1	Pcbd1		Pterin-4-alp	no | down	0	COG2154(F
Q9DC33	HM20A_MO	Hmg20a	Hmg20a	66867	High mobil	no | no chan	0	COG5648(h
Q6P9R1	DDX51_MO	Ddx51	Ddx51	69663	ATP-depen	no | no chan	0	ENOG410X
Q6P9R2	OXSR1_MO	Oxsr1	Oxsr1	108737	Serine/thre	no | no chan	0	ENOG410X
O88396	GRPE2_MO	Grpel2	Grpel2	17714	GrpE prote	no | no chan	0	COG0576(O
E9Q5K9	YTDC1_MO	Ythdc1	Ythdc1	231386	YTH domai	no | no chan	0	ENOG4111
E9Q5K8	E9Q5K8_M	Itgam	Itgam		Integrin alp	no | no chan	0	ENOG410Z
Q8CCM6	TIM21_MO	Timm21	Timm21	67105	Mitochondr	no | no chan	0	ENOG4111
A2RRK3	A2RRK3_M	Slk	Slk	20874	non-specific	no | no chan	0	ENOG410X
E9PYN1	E9PYN1_M	Cadm1	Cadm1	54725	Cell adhesi	no | no chan	0	ENOG4111
O88398	AVIL_MOU	Avil	Avil	11567	Advillin	no | down	0	ENOG410X
Q8VCY6	UTP6_MOU	Utp6	Utp6	216987	U3 small nu	no | no chan	0	COG5191(U
Q7TNZ4	Q7TNZ4_M	Gpld1	Gpld1	14756	Phosphatid	no | no chan	0	ENOG4111
P27808	MGAT1_M	Mgat1	Mgat1	17308	Alpha-1,3-m	no | no chan	0	ENOG410X
A0AA74KT	A0AA74KT	Jun	Jun		Transcripti	no | up	0	ENOG410X
P49919	CDN1C_M	Cdkn1c	Cdkn1c	12577	Cyclin-depe	no | no chan	0	ENOG410X
B3VQI8	B3VQI8_M	Cyba	Cyba	13057	Cytochrom	no | up	0	ENOG4111
Q9DCN7	RNFT1_MO	Rnft1	Rnft1	76892	E3 ubiquiti	no | no chan	0	ENOG410X
Q8R1F1	NIBA2_MO	Niban2	Niban2	227737	Protein Nib	no | no chan	0	ENOG4110
D3YU80	D3YU80_M	Cirbp	Cirbp		Cold-induci	yes | down	1	COG0724(r
P14234	FGR_MOU	Fgr	Fgr	14191	Tyrosine-pr	no | no chan	0	COG0515(r
Q00651	ITA4_MOU	Itga4	Itga4	16401	Integrin alp	no | up	0	ENOG410X
Q8BNU0	ARMC6_M	Armc6	Armc6	76813	Armadillo r	no | no chan	0	ENOG410X
A0AAQ4VI	A0AAQ4VMT1_MOUSE	phosphatidylinositol-	Mtmr3			no | no chan	0	ENOG410X
Q80WV3	CHST2_MO	Chst2	Chst2	54371	Carbohydrc	no | no chan	0	ENOG4111

P20664	PRI1_MOU	Prim1	Prim1	19075	DNA primo	no|no chan	0	COG1467(I
D6RHA9	D6RHA9_M	Tmtc4	Tmtc4		Transmemb	no|no chan	0	ENOG410X
Q8BU00	IKZF5_MO	Ikzf5	Ikzf5	67143	Zinc finger	yes|up	1	COG5048(F
Q8BU05	Q8BU05_M	Polr3k	Polr3k	67005	DNA-direc	yes|up	1	COG1594(I
B5B2N2	B5B2N2_M	Nfatc1	Nfatc1	18018	Nuclear fac	no|no chan	0	ENOG411C
Q9WTP2	SPY4_MOU	Spry4	Spry4	24066	Protein spr	no|no chan	0	ENOG410Z
P0DPE0	EFMT4_MO	Eef1akmt4	Eef1akmt4	110599584;	EEF1A lysi	no|no chan	0	ENOG410X
Q9WTP7	KAD3_MO	Ak3	Ak3	56248	GTP:AMP p	no|no chan	0	COG0563(s
Q9WTP6	KAD2_MO	Ak2	Ak2	11637	Adenylate k	no|no chan	0	COG0563(C
Q8BHT7	RGP1_MO	Rgp1	Rgp1	242406	RAB6A-GE	no|no chan	0	ENOG410Y
A0ABA7IX	A0ABA7IXJ6_MOUSE		Sin3A associated protei	Sap130		no|no chan	0	ENOG410Z
Q4FK74	Q4FK74_M	Atp5f1d	Atp5f1d	66043	ATP syntha	no|no chan	0	COG0355(F
Q4FK79	Q4FK79_M	Ssr3	Ssr3	67437	Translocon	no|no chan	0	ENOG410X
A0A2R3XZ	A0A2R3XZ	Sting1	Sting1	72512	Stimulator	no|no chan	0	ENOG4111
Q8VCB1	NDC1_MO	Ndc1	Ndc1	72787	Nucleopori	no|no chan	0	ENOG411C
Q9D8X5	CNOT8_M	Cnot8	Cnot8	69125	CCR4-NOT	no|no chan	0	COG5228(Z
P43404	ZAP70_MO	Zap70	Zap70	22637	Tyrosine-pr	no|no chan	0	COG0515(I
Q99JB2	STML2_MO	Stoml2	Stoml2	66592	Stomatin-li	no|no chan	0	COG0330(I
O08584	KLF6_MOU	Klf6	Klf6	23849	Krueppel-li	no|no chan	0	COG5048(T
Q99JB0	KLF7_MOU	Klf7	Klf7	93691	Krueppel-li	no|no chan	0	COG5048(s
O08582	GTPB1_MO	Gtpbp1	Gtpbp1	14904	GTP-bindi	no|no chan	0	COG5258(T
O08583	THOC4_MO	Alyref	Alyref	21681	THO comp	no|no chan	0	ENOG4111
Q6KCD5	NIPBL_MO	Nipbl	Nipbl	71175	Nipped-B-l	no|no chan	0	ENOG410X
Q922D8	C1TC_MOU	Mthfd1	Mthfd1	108156	C-1-tetrahy	no|no chan	0	COG0190(C
A2A8V8	A2A8V8_M	Srrm1	Srrm1		Serine/argi	no|no chan	0	ENOG4111
Q80VP0	TCPR1_MO	Tecpr1	Tecpr1	70381	Tectonin be	no|no chan	0	ENOG410Y
Q80VP5	MTRFR_M	Mtrfr	Mtrfr	72650	Mitochondr	no|no chan	0	COG1186(I
F8VPN4	F8VPN4_M	Agl	Agl	77559	Glycogen d	no|up	0	COG3408(C
Q5SQA2	Q5SQA2_M	Tle1	Tle1	21885	Transducin	no|no chan	0	ENOG410X
Q8BGA5	KRR1_MO	Krr1	Krr1	52705	KRR1 smal	no|no chan	0	COG1094(I
Q8BHG9	CGBP1_MO	Cggbp1	Cggbp1	106143	CGG triple	no|no chan	0	ENOG4111
Q62432	SMAD2_M	Smad2	Smad2	17126	Mothers ag	no|no chan	0	ENOG410X
P62746	RHOB_MO	Rhob	Rhob	11852	Rho-related	no|up	0	COG1100((
P62743	AP2S1_MO	Ap2s1	Ap2s1	232910	AP-2 compl	no|no chan	0	COG5030((
D3Z723	D3Z723_M	Nfyb	Nfyb		Nuclear tra	no|no chan	0	COG2036(C
P62748	HPCL1_MO	Hpcal1	Hpcal1	53602	Hippocalci	no|no chan	0	COG5126(Y
A0A668KL	A0A668KL	Tmem94	Tmem94	71947	Transmemb	no|no chan	0	ENOG410X
A0A0R4J0Z	A0A0R4J0Z	Gtpbp6	Gtpbp6	107999	GTP bindi	no|no chan	0	COG2262(C
E9Q9M0	E9Q9M0_M	Cep290	Cep290	216274	Centrosom	no|no chan	0	ENOG410X
F8WHY8	F8WHY8_M	Mta1	Mta1	116870	Metastasis	no|no chan	0	ENOG410X
E9Q9M8	PWP2B_MO	Pwwp2b	Pwwp2b	101631	PWWP dom	no|no chan	0	ENOG410X
Q9JI58	RAE1D_MO	Raet1d	Raet1d	56554	Retinoic ac	no|no chan	0	ENOG4111
P97369	NCF4_MO	Ncf4	Ncf4	17972	Neutrophil	no|up	0	ENOG411C

P97364	SPS2_MOU	Sephs2	Sephs2	20768	Selenide, w	yes|down	1	COG0709(S
P97363	SPTC2_MC	Sptlc2	Sptlc2	20773	Serine palm	no|no chan	0	COG0156(I
P97360	ETV6_MO	Etv6	Etv6	14011	Transcripti	no|no chan	0	ENOG4111
Q9QWV9	CCNT1_M	Ccnt1	Ccnt1	12455	Cyclin-T1	no|no chan	0	COG5333(I
Q9DB42	ZN593_MC	Znf593	Znf593	68040	Zinc finger	no|no chan	0	COG5112(z
E9QP84	E9QP84_M	Med16	Med16	216154	Mediator of	no|no chan	0	ENOG410Z
Q5ICG5	Q5ICG5_M	Acsl6	Acsl6	216739	Long-chain	no|up	0	COG1022(I
Q3TLL0	Q3TLL0_M	Spidr	Spidr	224008	DNA repai	no|no chan	0	ENOG410Y
Q3U0N4	Q3U0N4_M	Taf1c	Taf1c	21341	TATA box-	no|no chan	0	ENOG411C
Q9DB40	MED27_M	Med27	Med27	68975	Mediator of	no|no chan	0	ENOG410Z
Q3TLL4	Q3TLL4_M	Hgs	Hgs	15239	Hepatocyte	no|no chan	0	ENOG410X
Q8BGU5	CCNY_MC	Ccny	Ccny	67974	Cyclin-Y	no|no chan	0	ENOG410X
E9Q6M7	E9Q6M7_M	Pum1	Pum1	80912	Pumilio ho	no|no chan	0	COG5099(e
Q8R086	SUOX_MC	Suox	Suox	211389	Sulfite oxid	no|down	0	COG2041(T
Q571C7	BDP1_MO	Bdp1	Bdp1	544971	Transcripti	no|no chan	0	COG5118(I
Q8R084	Q8R084_M	Ugt2b1	Ugt2b1	71773	UDP-glucu	no|up	0	COG1819(T
Q8R081	HNRPL_M	Hnrnpl	Hnrnpl	15388	Heterogene	no|no chan	0	ENOG410X
A0A0R4J0F	A0A0R4J0F	Gnptg	Gnptg	214505	N-acetylglu	no|no chan	0	ENOG410X
Q9R1C6	DGKE_MC	Dgke	Dgke	56077	Diacylglyce	no|no chan	0	ENOG410X
Q9CR14	FANCL_M	Fancl	Fancl	67030	E3 ubiquiti	no|no chan	0	ENOG410Y
B2CY77	B2CY77_M	Rpsa	Rpsa	16785	40S ribosom	no|no chan	0	COG0052(C
Q9CWT6	DDX28_M	Ddx28	Ddx28	71986	Probable A	no|no chan	0	ENOG410X
Q9CR11	YETS4_MC	Yeats4	Yeats4	64050	YEATS dom	no|no chan	0	COG5033(u
B2RS80	B2RS80_M	Ggcx	Ggcx	56316	Vitamin K-	no|no chan	0	ENOG410X
Q8BSN5	PHF23_MC	Phf23	Phf23	78246	PHD finger	no|no chan	0	ENOG4111
Q3U2G2	Q3U2G2_M	Hspa4	Hspa4	15525	Heat shock	no|no chan	0	COG0443(I
A0A5F8MP	A0A5F8MP	Fnbp1l	Fnbp1l		Formin-bin	no|no chan	0	ENOG410X
Q9Z306	S22A4_MO	Slc22a4	Slc22a4	30805	Solute carri	no|down	0	COG0477(I
B7ZN28	B7ZN28_M	Papln	Papln	170721	Papilin, pro	no|up	0	ENOG410X
M0QWN7	M0QWN7_	Usp35	Usp35	244144	ubiquitinyl	no|up	0	ENOG410X
Q3U2G9	Q3U2G9_M	Nagk	Nagk	56174	N-acetyl-D	no|no chan	0	COG2971(I
F6T2S0	F6T2S0_M	Fech	Fech		Ferrochelat	no|no chan	0	COG0276(C
A0A0R4J1D	A0A0R4J1D	Cpne2	Cpne2	234577	Copine II	no|no chan	0	ENOG410X
A0A0R4J1D	A0A0R4J1D	Cbfa2t2	Cbfa2t2	12396	CBFA2/RU	no|up	0	ENOG410X
Q5FW52	MLIP_MO	Mlip	Mlip	69642	Muscular L	no|up	0	ENOG4111
Q8C2S7	AMGO3_M	Amigo3	Amigo3	320844	Amphoterin	no|down	0	ENOG410Y
A0A0R4J1D	A0A0R4J1D	Fgd3	Fgd3	30938	FYVE, Rho	no|no chan	0	ENOG410X
Q3UE92	Q3UE92_M	Xpnpep1	Xpnpep1	170750	X-prolyl an	no|no chan	0	COG0006(p
Q6P2L7	GOLM2_M	Golm2	Golm2	319996	Protein GO	no|no chan	0	ENOG4111
Q3UTX5	Q3UTX5_M	Cd209b	Cd209b	69165	C-type lecti	no|down	0	ENOG410Y
Q9D1Z3	ACKMT_M	Atpsckmt	Atpsckmt	68073	ATP syntha	no|no chan	0	ENOG4111
B2RSC8	B2RSC8_M	Nedd4	Nedd4	17999	E3 ubiquiti	no|no chan	0	COG5021(I
Q9D287	SPF27_MO	Bcas2	Bcas2	68183	Pre-mRNA	no|no chan	0	ENOG411C

Q8CCP0	NEMF_MC	Nemf	Nemf	66244	Ribosome q	no|no chan	0	COG1293(I
A2ATY4	A2ATY4_M	Zbtb34	Zbtb34	241311	Zinc finger	no|down	0	COG5048(C
Q14CH1	MOCOS_M	Mocos	Mocos	68591	Molybdenu	no|no chan	0	COG3217(N
Q14CH7	SYAM_MO	Aars2	Aars2	224805	Alanine--tR	no|no chan	0	COG0013(C
Q3TXV4	Q3TXV4_M	Rab31	Rab31	106572	RAB31, mer	no|no chan	0	ENOG410Y
Q9D6N1	CAH13_MC	Ca13	Ca13	71934	Carbonic an	yes|up	1	COG3338(C
H3BJ97	H3BJ97_MC	Tinagl1	Tinagl1		Tubulointe	no|no chan	0	ENOG410Y
Q3UDR8	YIPF3_MO	Yipf3	Yipf3	28064	Protein YIF	no|no chan	0	ENOG4111
Q3UKZ0	Q3UKZ0_M	Rbfa	Rbfa	68731	Ribosome-b	no|no chan	0	ENOG4111
A0A411P8U	A0A411P8U	Mbp	Mbp	17196	Myelin basi	no|down	0	ENOG4111
G3UW90	G3UW90_M	Trps1	Trps1	83925	Transcripti	no|no chan	0	COG5641(S
Q91WD4	CG025_MOUSE	UPF0415 protein (		105351	UPF0415 pr	no|no chan	0	ENOG410Z
Q91WD5	NDUS2_M	Ndufs2	Ndufs2	226646	NADH deh	no|no chan	0	COG0649(N
G3UW94	G3UW94_M	U2af1	U2af1	108121	U2 small nu	no|no chan	0	ENOG410Z
P36552	HEM6_MC	Cpox	Cpox	12892	Oxygen-dep	no|no chan	0	COG0408(H
Q0VGM9	RTEL1_MC	Rtel1	Rtel1	269400	Regulator c	no|no chan	0	COG1199(S
Q0VB06	Q0VB06_M	Necap1	Necap1	67602	Adaptin ea	no|no chan	0	ENOG4111
Q3UM97	Q3UM97_M	Eapp	Eapp	66266	E2F-associc	no|no chan	0	ENOG4110
Q3UC72	Q3UC72_M	Gdi2	Gdi2	14569	Rab GDP d	no|no chan	0	COG5044(F
Q3UM95	Q3UM95_M	Vipas39	Vipas39	104799	Spermatoge	no|no chan	0	ENOG410X
Q6PDJ1	CAHD1_M	Cachd1	Cachd1	320508	VWFA and	yes|down	1	ENOG410X
A2AGA4	RHBL2_MC	Rhbdl2	Rhbdl2	230726	Rhomboid-	no|no chan	0	COG0705(F
Q8BK58	HBAP1_MC	Hspbap1	Hspbap1	66667	HSPB1-assc	no|no chan	0	ENOG410X
Q9Z2A9	GGT5_MO	Ggt5	Ggt5	23887	Glutathion	no|down	0	COG0405(U
A3KMJ8	A3KMJ8_M	Vbp1	Vbp1	22327	Vbp1 prote	no|no chan	0	ENOG410X
Q0VDW9	Q0VDW9_	Abcg1	Abcg1	11307	ATP-bindir	no|no chan	0	COG1131(F
Q8BVI4	DHPR_MC	Qdpr	Qdpr	110391	Dihydropte	no|no chan	0	ENOG4111
Q8BVI5	STX16_MC	Stx16	Stx16	228960	Syntaxin-16	no|no chan	0	COG5325(S
B0R0U1	B0R0U1_M	Mettl8	Mettl8		tRNA N(3)-	no|no chan	0	ENOG410Y
B9EI57	B9EI57_MC	Sltm	Sltm	66660	SAFB-like,	no|no chan	0	ENOG4111
O70439	STX7_MOU	Stx7	Stx7	53331	Syntaxin-7	no|no chan	0	COG5325(I
E9PVB6	E9PVB6_M	Limk1	Limk1	16885	LIM domai	no|no chan	0	COG0515(F
O70435	PSA3_MOU	Psma3	Psma3	19167	Proteasome	no|no chan	0	ENOG410X
P11679	K2C8_MOU	Krt8	Krt8	16691	Keratin, typ	no|no chan	0	ENOG410Y
P60840	ENSA_MO	Ensa	Ensa	56205	Alpha-endc	no|no chan	0	ENOG4112
O70433	FHL2_MOU	Fhl2	Fhl2	14200	Four and a	no|no chan	0	ENOG410X
P60843	IF4A1_MO	Eif4a1	Eif4a1	13681	Eukaryotic	no|no chan	0	COG0513(e
P11928	OAS1A_MC	Oas1a	Oas1a	246730; 239	2'-5'-oligoac	no|no chan	0	COG5272(i
Q2M4J2	Q2M4J2_M	Plcb1	Plcb1	18795	Phosphoinc	no|no chan	0	ENOG410X
Q8BKN5	GCP5_MO	Tubgcp5	Tubgcp5	233276	Gamma-tul	no|no chan	0	ENOG410Z
Q3U6K8	Q3U6K8_M	Vdac1	Vdac1	22333	Non-selecti	no|no chan	0	ENOG410Z
Q3TAA7	S11IP_MOU	Stk11ip	Stk11ip	71728	Serine/thre	no|no chan	0	ENOG410X
Q8BW75	AOFB_MO	Maob	Maob	109731	Amine oxid	no|no chan	0	ENOG410X

Q9D6J5	NDUB8_M	Ndufb8	Ndufb8	67264	NADH deh	no | no chan	0 ENOG4111
P55086	PAR2_MO	F2rl1	F2rl1	14063	Proteinase-	no | no chan	0 ENOG4111
Q3TAA8	Q3TAA8_M	Pigu	Pigu	228812	Phosphatid	no | no chan	0 ENOG410
Q5HZY7	Q5HZY7_M	Atp6v1g1	Atp6v1g1	66290	V-type prot	no | no chan	0 ENOG4111
Q0VFY6	Q0VFY6_M	Zdhhc17	Zdhhc17	320150	Palmitoyltr	no | no chan	0 COG5273(
Q3UQD0	Q3UQD0_	Tomm40	Tomm40	53333	Mitochondı	no | no chan	0 ENOG410
Q8C9W3	ATS2_MO	Adamts2	Adamts2	216725	A disintegri	no | down	0 ENOG410
Q6ZPE2	MTMR5_M	Sbf1	Sbf1	77980	Myotubula	no | no chan	0 ENOG410
A9XX86	A9XX86_M	Mgl2	Mgl2	216864	Macrophag	no | down	0 ENOG410
P51437	CAMP_MC	Camp	Camp	12796	Cathelicidi	no | up	0 ENOG4111
O35516	NOTC2_M	Notch2	Notch2	18129	Neurogenic	no | no chan	0 COG0666(
P51432	PLCB3_MC	Plcb3	Plcb3	18797	1-phosphat	no | no chan	0 ENOG410
Q5SVX3	Q5SVX3_M	Ddx56	Ddx56	52513	RNA helico	no | no chan	0 ENOG410
B1AUW6	B1AUW6_	Irak1	Irak1	16179	non-specifi	no | no chan	0 COG0515(
Q60972	RBBP4_MC	Rbbp4	Rbbp4	19646	Histone-bir	no | no chan	0 ENOG410
Q3TDI5	Q3TDI5_M	Pex19	Pex19	19298	Peroxisoma	no | no chan	0 ENOG4111
Q3TDI7	Q3TDI7_M	3110040N1	3110040N1	67290	Uncharacte	no | no chan	0 ENOG4112
Q8BPS4	GP180_MC	Gpr180	Gpr180	58245	Integral me	no | no chan	0 ENOG410
I6L9F0	I6L9F0_MC	Tec	Tec	21682	Tyrosine-pr	no | no chan	0 COG0515(
Q3TL44	NLRX1_M	Nlrx1	Nlrx1	270151	NLR family	no | no chan	0 ENOG410
Q61510	TRI25_MO	Trim25	Trim25	217069	E3 ubiquiti	no | no chan	0 ENOG410
E9PWD4	E9PWD4_	Trmt1	Trmt1	212528	tRNA (guai	no | no chan	0 COG1867(I
Q99L00	HAUS8_M	Haus8	Haus8	76478	HAUS augr	no | no chan	0 ENOG4112
Q91W86	VPS11_MO	Vps11	Vps11	71732	Vacuolar p	no | no chan	0 ENOG410
Q91W87	Q91W87_M	Cyp2d22	Cyp2d22	56448	Cytochrom	no | no chan	0 COG2124(
Q545U2	Q545U2_M	Timm17a	Timm17a	21854	Mitochondı	no | down	0 COG5596(
F7CFT1	F7CFT1_M	Zfand1	Zfand1		Zinc finger,	no | no chan	0 ENOG4111
Q3U957	Q3U957_M	Derl2	Derl2	116891	Derlin	no | no chan	0 COG5291(I
Q8BG79	C19L2_MO	Cwf19l2	Cwf19l2	244672	CWF19-like	no | no chan	0 ENOG410
Q8VE19	MIOS_MO	Mios	Mios	252875	GATOR2 c	no | no chan	0 ENOG410
Q8VE18	SMG8_MO	Smg8	Smg8	74133	Nonsense-n	no | no chan	0 ENOG410
P62918	RL8_MOU	Rpl8	Rpl8	26961	Large ribos	no | no chan	0 COG0090((
P70362	UFD1_MO	Ufd1	Ufd1	22230	Ubiquitin r	no | no chan	0 COG5140(I
Q8C120	SH3R3_MC	Sh3rf3	Sh3rf3	237353	E3 ubiquiti	no | up	0 ENOG410
Q921S7	RM37_MO	Mrpl37	Mrpl37	56280	Large ribos	no | no chan	0 ENOG410
A3KGG8	A3KGG8_	Knstrn	Knstrn	51944	Kinetochor	no | no chan	0 ENOG4111
Q3U1N2	SRBP2_MC	Srebf2	Srebf2	20788	Sterol regul	no | no chan	0 ENOG410
P62911	RL32_MO	Rpl32	Rpl32	19951	Large ribos	no | no chan	0 COG1717((
Q8C129	LCAP_MO	Lnpep	Lnpep	240028	Leucyl-cysti	no | no chan	0 COG0308(
P62915	TF2B_MO	Gtf2b	Gtf2b	229906	Transcripti	no | no chan	0 COG1405((
Q80Y84	KDM5B_M	Kdm5b	Kdm5b	75605	Lysine-spec	no | no chan	0 ENOG410
E9Q6P4	E9Q6P4_M	Tubgcp6	Tubgcp6		Tubulin, ga	no | no chan	0 ENOG410
Q3ULG4	Q3ULG4_	Psmd4	Psmd4	19185	26S proteas	no | no chan	0 COG5148(2

E9PYK3	PARP4_MC	Parp4	Parp4	328417	Protein mo	no	no chan	0	ENOG410
Q8R570	SNP47_MC	Snap47	Snap47	67826	Synaptoson	no	no chan	0	ENOG410
O88271	CFDP1_MC	Cfdp1	Cfdp1	23837	Craniofacic	no	no chan	0	ENOG4111
A2ADA5	PUSL1_MC	Pusl1	Pusl1	433813	tRNA pseu	no	no chan	0	COG0101(r
A0A1G5SJI	A0A1G5SJI	OTTMUSW	OTTMUSW	20555	Schlafen fa	no	up	0	ENOG4111
O89053	COR1A_M	Coro1a	Coro1a	12721	Coronin-1A	no	no chan	0	ENOG410
A0A0U1RN	A0A0U1RN	Tufm	Tufm		Tu translat	yes	up	1	COG0050(T
O89051	ITM2B_MC	Itm2b	Itm2b	16432	Integral me	no	no chan	0	ENOG410
O89050	MKLN1_M	Mkln1	Mkln1	27418	Muskelin	no	no chan	0	ENOG410
Q9EPL8	IPO7_MOU	Ipo7	Ipo7	233726	Importin-7	no	no chan	0	COG5656(I
Q9EPL2	CSTN1_MC	Clstn1	Clstn1	65945	Calsyntenir	no	no chan	0	ENOG410
Q9EPL4	METL9_MC	Mettl9	Mettl9	59052	Protein-L-h	no	no chan	0	ENOG4110
Q3UJU9	RMD3_MC	Rmdn3	Rmdn3	67809	Regulator c	no	no chan	0	ENOG4110
P16951	ATF2_MOU	Atf2	Atf2	11909	Cyclic AMF	no	no chan	0	ENOG4111
Q8CGC7	SYEP_MOU	Eprs1	Eprs1	107508	Bifunctionc	no	no chan	0	COG0442(I
Q197W7	Q197W7_M	Man2a2	Man2a2	140481	Alpha-man	no	no chan	0	ENOG410
P68040	RACK1_M	Rack1	Rack1	14694	Small ribos	no	no chan	0	ENOG410
Q8CGC4	LS14B_MO	Lsm14b	Lsm14b	241846	Protein LSM	no	no chan	0	ENOG4112
Q8BJD1	ITIH5_MO	Itih5	Itih5	209378	Inter-alpha	no	no chan	0	COG2304(A
Q5SFM8	RBM27_MC	Rbm27	Rbm27	225432	RNA-bindi	no	no chan	0	ENOG410
O08800	SPB8_MOU	Serpinb8	Serpinb8	20725	Serpin B8	no	up	0	COG4826(c
O08807	PRDX4_M	Prdx4	Prdx4	53381	Peroxiredo	no	no chan	0	COG0450(F
O08804	O08804_M	Serpinb6b	Serpinb6b	20708	NK13	no	no chan	0	COG4826(c
P58281	OPA1_MO	Opa1	Opa1	74143	Dynamin-li	no	no chan	0	COG0699(C
P58283	RN216_MC	Rnf216	Rnf216	108086	E3 ubiquiti	no	no chan	0	ENOG410
Q3U0D9	HACE1_M	Hace1	Hace1	209462	E3 ubiquiti	no	no chan	0	COG5021(s
Q7TS72	IP3KC_MC	Itpkc	Itpkc	233011	Inositol-tris	no	no chan	0	ENOG410
Q9D0A0	DET1_MO	Det1	Det1	76375	DET1 hom	no	up	0	ENOG410
A0A140LIC	A0A140LIC	Tmem147	Tmem147		BOS compl	no	no chan	0	ENOG4110
Q3UXI9	Q3UXI9_M	Ilf2	Ilf2	67781	DZF domai	no	no chan	0	ENOG410
Q6A097	Q6A097_M	Ip6k1	Ip6k1	27399	Kinase	no	no chan	0	ENOG410
F6TWM7	F6TWM7_M	Fxyd5	Fxyd5		FXYD dom	no	no chan	0	ENOG4111
E9Q509	E9Q509_M	Pklr	Pklr	18770	Pyruvate ki	no	no chan	0	COG0469(c
Q8CIV2	MBRL_MO	Tmem259	Tmem259	216157	Membralin	no	no chan	0	ENOG410
E9Q507	E9Q507_M	Gon4l	Gon4l	76022	GON-4-like	no	no chan	0	ENOG410
Q8CIV8	TBCE_MO	Tbce	Tbce	70430	Tubulin-sp	no	no chan	0	ENOG410
A2AAN0	A2AAN0_M	Exoc7	Exoc7	53413	Exocyst cor	no	no chan	0	ENOG410
P27005	S10A8_MO	S100a8	S100a8	20201	Protein S10	no	up	0	ENOG4111
A0A0G2JEI	A0A0G2JEI	Crip1	Crip1	12925	Cysteine-ric	no	no chan	0	ENOG4111
Q3UVS6	Q3UVS6_M	Flrt2	Flrt2	399558	Fibronectin	no	no chan	0	ENOG4111
Q91W29	COX42_MC	Cox4i2	Cox4i2	84682	Cytochrom	no	no chan	0	ENOG4111
Q9WVQ5	MTNB_MC	Apip	Apip	56369	Methylthio	no	no chan	0	COG0235(F
Q3UPC8	Q3UPC8_M	Zfp592	Zfp592	233410	C2H2-type	no	no chan	0	COG5048(S

Q4KL41	Q4KL41_M	Tomm20	Tomm20	67952	Mitochondı	no	no chan	0	ENOG4111
A0A1Z3MF	A0A1Z3MF	Ifi203a	Ifi203a	15950	Ifi203a	no	up	0	ENOG410Y
A0A1Y7VJ	A0A1Y7VJ	Dtna	Dtna	13527	Dystrobrevi	no	up	0	ENOG410X
Q8R3C7	Q8R3C7_M	Ddx19b	Ddx19b	234733	RNA helicc	no	no chan	0	ENOG410X
P14148	RL7_MOUS	Rpl7	Rpl7	19989	Large ribos	no	no chan	0	COG1841(r
E9PY08	E9PY08_M	Adamts1	Adamts1		ADAM met	no	no chan	0	ENOG410X
P61922	GABT_MO	Abat	Abat	268860	4-aminobut	no	no chan	0	COG0160(E
Q3TXN3	Q3TXN3_M	Mgat2	Mgat2	217664	Alpha-1,6-r	no	no chan	0	ENOG410X
P61924	COPZ1_M	Copz1	Copz1	56447	Coatomer s	no	no chan	0	COG5541((
P19123	TNNC1_M	Tnnc1	Tnnc1	21924	Troponin C	no	up	0	COG5126(s
Q9QZI9	SERC3_MC	Serinc3	Serinc3	26943	Serine incoı	no	no chan	0	ENOG410X
Q9D6W8	BORC6_M	Borcs6	Borcs6	71923	BLOC-1-rel	no	no chan	0	ENOG4111
Q9DBC7	KAP0_MO	Prkar1a	Prkar1a	19084	cAMP-depe	no	no chan	0	COG0664(t
Q9ET78	JPH2_MOU	Jph2	Jph2	59091	Junctophili	no	up	0	COG4642(S
P97797	SHPS1_MC	Sirpa	Sirpa	19261	Tyrosine-pr	no	no chan	0	ENOG410Y
Q8BKY8	MTEF2_M	Mterf2	Mterf2	74238	Transcripti	no	no chan	0	ENOG410X
K0BWC3	K0BWC3_M	Palld	Palld	72333	Palladin isc	no	no chan	0	ENOG410(
P67984	RL22_MOU	Rpl22	Rpl22	19934	Large ribos	no	no chan	0	ENOG4111
Q8K1I7	WIPF1_MC	Wipf1	Wipf1	215280	WAS/WAS	no	no chan	0	ENOG4111
A0A338P69	A0A338P69	Yeats2	Yeats2		YEATS don	no	no chan	0	COG5033(r
Q3TQE0	Q3TQE0_M	Hk3	Hk3	212032	hexokinase	no	up	0	COG5026(k
F6XDW8	F6XDW8_M	Usp32	Usp32		ubiquitinyl	no	no chan	0	COG5560(u
Q8CJ69	BMPER_M	Bmper	Bmper	73230	BMP-bindi	no	no chan	0	ENOG410X
A0A2R8W6	A0A2R8W6	Cd47	Cd47		Leukocyte s	no	no chan	0	ENOG4111
S4R1W1	S4R1W1_M	Gapdhrt	Gapdhrt		Glyceraldel	no	up	0	COG0057(g
Q59J78	NDUF2_M	Ndufaf2	Ndufaf2	75597	NADH deh	no	no chan	0	ENOG4111
D3Z7B5	D3Z7B5_M	Cip2a	Cip2a		Cell prolife	no	no chan	0	ENOG411(
A0A0A6YV	A0A0A6YV	Adrm1b	Adrm1b	100043022	Adhesion re	no	no chan	0	ENOG410X
Q3UG85	Q3UG85_M	Ank	Ank	11732	Progressive	no	no chan	0	ENOG410Z
Q3TDV8	Q3TDV8_M	Zfp444	Zfp444	72667	Zinc finger	yes	down	1	COG5048(A
E9Q3E2	E9Q3E2_M	Synpo	Synpo	104027	Synaptopoc	no	no chan	0	ENOG4111
E9Q3E1	AL3B2_MC	Aldh3b2	Aldh3b2	621603	Aldehyde d	no	down	0	COG1012(r
Q99JT6	TLCD1_M	Tlcd1	Tlcd1	68385	TLC domai	no	no chan	0	ENOG410Z
P50285	FMO1_MC	Fmo1	Fmo1	14261	Flavin-cont	no	down	0	COG2072(M
A2A799	A2A799_M	Zfp979	Zfp979	112422	Zinc finger	yes	down	1	COG5048(T
Q9ERS4	PKHA3_M	Plekha3	Plekha3	83435	Pleckstrin h	no	up	0	ENOG410Y
P48760	FOLC_MO	Fpgs	Fpgs	14287	Folylpolygl	no	down	0	COG0285(E
Q9JJT0	RCL1_MOU	Rcl1	Rcl1	59028	RNA 3'-terr	no	no chan	0	COG0430(p
P97425	ECP2_MOU	Ear2	Ear2	13587	Eosinophil	no	down	0	ENOG4111
P97426	ECP1_MOU	Ear1	Ear1	13586	Eosinophil	no	down	0	ENOG4111
P97427	DPYL1_M	Crmp1	Crmp1	12933	Dihydropyr	no	no chan	0	COG0044(F
P97429	ANXA4_M	Anxa4	Anxa4	11746	Annexin A4	no	no chan	0	ENOG410X
O35218	CPSF2_MC	Cpsf2	Cpsf2	51786	Cleavage aı	no	no chan	0	COG1236(N

E0CXC2	E0CXC2_M	Gm28043	Gm28043		E3 ubiquiti	no|no chan	0	ENOG410Z
Q8BGS7	CEPT1_MC	Cept1	Cept1	99712	Choline/etl	no|no chan	0	COG5050(e
O88874	CCNK_MC	Ccnk	Ccnk	12454	Cyclin-K	no|no chan	0	COG5333(A
Q91X78	ERLN1_M(	Erlin1	Erlin1	226144	Erlin-1	no|no chan	0	ENOG410X
P19788	MGP_MOU	Mgp	Mgp	17313	Matrix Gla	no|up	0	ENOG410Y
A2APF7	A2APF7_M	Zbp1	Zbp1	58203	Z-DNA bin	no|up	0	ENOG4111
O88878	ZFAN5_M(	Zfand5	Zfand5	22682	AN1-type z	no|down	0	ENOG4111
O88879	APAF_MO	Apaf1	Apaf1	11783	Apoptotic p	no|no chan	0	ENOG410X
G3X9B1	G3X9B1_M	Heatr1	Heatr1	217995	HEAT repe	no|no chan	0	ENOG410X
Q8C460	ERI3_MOU	Eri3	Eri3	140546	ERI1 exoril	no|no chan	0	COG5018(F
Q8BGS2	BOLA2_M(	Bola2	Bola2	66162	BolA-like p	no|no chan	0	COG0271(M
G5E8A7	G5E8A7_M	Samd4b	Samd4b	233033	Sterile alph	no|no chan	0	ENOG410X
A0A571BD	A0A571BD	Zfp869	Zfp869		Zinc finger	no|no chan	0	COG5048(Z
Q9DBE0	CSAD_MO	Csad	Csad	246277	Cysteine su	no|no chan	0	COG0076(c
Q9D0F7	Q9D0F7_M	Pus3	Pus3		Pseudourid	no|no chan	0	COG0101(F
E9PUY1	E9PUY1_M	Mrps12	Mrps12		Small ribos	no|no chan	0	COG0048(I
Q91X72	HEMO_M(	Hpx	Hpx	15458	Hemopexin	no|up	0	ENOG410X
Q3TFD2	PCAT1_M(	Lpcat1	Lpcat1	210992	Lysophosph	no|no chan	0	ENOG410X
Q5XJV7	SETD5_M(	Setd5	Setd5	72895	Histone-lys	no|no chan	0	ENOG410Z
Q9CRA8	EXOS5_M(	Exosc5	Exosc5	27998	Exosome cc	no|no chan	0	COG0689(f
Q9CRA9	FGOP2_M(	Fgfr1op2	Fgfr1op2	67529	FGFR1 onc	no|no chan	0	ENOG410X
Q9CRA4	MSMO1_M	Msmo1	Msmo1	66234	Methylsterc	no|no chan	0	COG3000(f
Q9CRA5	GOLP3_M(	Golph3	Golph3	66629	Golgi phos	no|no chan	0	ENOG410X
Q9CRA7	ATP5S_MC	Dmac2l	Dmac2l	68055	ATP syntha	no|no chan	0	ENOG411(
Q9JL56	GDE1_MC	Gde1	Gde1	56209	Glycerophc	no|no chan	0	COG0584(s
O35326	SRSF5_MO	Srsf5	Srsf5	20384	Serine/argi	no|no chan	0	COG0724(M
F6UIS1	F6UIS1_M(	Ergic3	Ergic3		Endoplasm	no|no chan	0	ENOG410X
A2RSE4	A2RSE4_M	Ube2b	Ube2b	22210	Ubiquitin-c	no|no chan	0	COG5078(C
Q05AH6	SPNDC_M	Spindoc	Spindoc	68229	Spindlin int	no|no chan	0	ENOG4111
P61588	RND3_MC	Rnd3	Rnd3	74194	Rho-related	no|no chan	0	COG1100(F
Q3U646	Q3U646_M	Asah1	Asah1	11886	Acid ceram	no|no chan	0	ENOG410X
A2A9K3	A2A9K3_M	Cep85	Cep85	70012	Centrosom	no|no chan	0	ENOG410Z
Q52KR3	PRUN2_M(	Prune2	Prune2	353211	Protein pru	no|up	0	ENOG410Z
Q545G0	Q545G0_M	Psmb3	Psmb3	26446	Proteasome	no|no chan	0	ENOG410X
Q8VE11	MTMR6_M	Mtmr6	Mtmr6	219135	Phosphatid	no|no chan	0	ENOG410X
A8DUP7	A8DUP7_M	Hbbt1	Hbbt1	100503605	Beta-globin	no|no chan	0	COG1018(I
A2AQL0	A2AQL0_M	Stk39	Stk39	53416	non-specifi	no|no chan	0	ENOG410X
Q69Z43	Q69Z43_M	Nek9	Nek9	217718	non-specifi	no|no chan	0	ENOG410Y
G5E902	G5E902_M	Slc25a3	Slc25a3	18674	Solute carri	no|no chan	0	ENOG410X
Q8BKJ9	SIR7_MOU	Sirt7	Sirt7	209011	NAD-deper	no|no chan	0	COG0846(r
G5E905	G5E905_M	Zfp871	Zfp871	208292	Zinc finger	no|no chan	0	COG5048(F
Q9QXW9	LAT2_MOU	Slc7a8	Slc7a8	50934	Large neutr	no|no chan	0	COG0531(F
Q9QXW2	FBXW5_M	Fbxw5	Fbxw5	30839	F-box/WD	no|no chan	0	ENOG410X

Q9QXW0	FBXL6_MC	Fbxl6	Fbxl6	30840	F-box/LRR	no | no chan	0	ENOG410
Q05A36	MEX3C_M	Mex3c	Mex3c	240396	RNA-bindi	no | no chan	0	ENOG410
Q05A37	Q05A37_M	Has1	Has1	15116	hyaluronan	no | up	0	COG1215(
Q5U452	Q5U452_M	Trp53rka	Trp53rka	381406	non-specifi	no | down	0	COG3642(
Q3TK29	Q3TK29_M	Trap1	Trap1	68015	Histidine ki	no | no chan	0	COG0326(
A0A183ZRI	A0A183ZRI	Ece1	Ece1	230857	Endothelin-	no | no chan	0	COG3590(
P51660	DHB4_MO	Hsd17b4	Hsd17b4	15488	Peroxisoma	no | no chan	0	COG2030(
P03958	ADA_MOU	Ada	Ada	11486	Adenosine	no | no chan	0	COG1816(
K3W4Q8	K3W4Q8_M	Bsg	Bsg	12215	Basigin	no | no chan	0	ENOG4111
K3W4Q9	K3W4Q9_M	Gopc	Gopc		Golgi assoc	no | no chan	0	ENOG410
P51667	MLRV_MC	Myl2	Myl2	17906	Myosin regu	no | no chan	0	COG5126(
P51885	LUM_MOU	Lum	Lum	17022	Lumican	no | down	0	COG4886(
K7Q7T7	K7Q7T7_M	Rac1	Rac1	19353	Ras-related	no | no chan	0	COG1100(
P51881	ADT2_MO	Slc25a5	Slc25a5	11740	ADP/ATP	no | no chan	0	ENOG410
A0A158SIS	A0A158SIS	Trp53	Trp53	22059	Cellular tur	no | no chan	0	ENOG410
Q8BLY7	HPS6_MOU	Hps6	Hps6	20170	BLOC-2 co	no | no chan	0	ENOG411
Q8BLY2	SYTC2_MC	Tars3	Tars3	272396	Threonine--	no | no chan	0	COG0441(
Q9D2D7	ZN687_MC	Znf687	Znf687	78266	Zinc finger	no | no chan	0	COG5048(
E9PYH0	E9PYH0_M	Vcan	Vcan	13003	Versican co	no | no chan	0	ENOG410
E9PYH2	E9PYH2_M	Acot7	Acot7	70025	Acyl-CoA t	no | no chan	0	COG1607(
A8DIL0	A8DIL0_M	Lgals8	Lgals8	56048	Galectin	no | no chan	0	ENOG4111
E9PYH6	SET1A_MC	Setd1a	Setd1a	233904	Histone-lys	no | no chan	0	COG2940(
P17047	LAMP2_M	Lamp2	Lamp2	16784	Lysosome-a	no | no chan	0	ENOG410
B7ZDF5	B7ZDF5_M	Hdac5	Hdac5	15184	Histone dec	no | no chan	0	COG0123(
Q69ZY2	Q69ZY2_M	Endod1	Endod1	71946	MKIAA083	no | no chan	0	ENOG4111
Q3TUX3	Q3TUX3_M	Psma5	Psma5	26442	Proteasome	no | no chan	0	COG0638(
B1AV01	B1AV01_M	Mid1	Mid1	17318	E3 ubiquiti	no | up	0	ENOG410
Q8BTZ5	ANR46_MC	Ankrd46	Ankrd46	68839	Ankyrin rep	no | no chan	0	COG0666(
Q8BTZ7	GMPPB_M	Gmppb	Gmppb	331026	Mannose-1-	no | no chan	0	COG1208(
Q8BS40	CPTP_MO	Cptp	Cptp	79554	Ceramide-1	no | no chan	0	ENOG4111
P83917	CBX1_MO	Cbx1	Cbx1	12412	Chromobox	no | no chan	0	ENOG4111
Q8BS45	IFT56_MO	Ift56	Ift56	264134	Intraflagell	no | no chan	0	ENOG410
B7ZCR6	B7ZCR6_M	Zfp512b	Zfp512b	269401	Zinc finger	no | no chan	0	COG5048(
Q9D2Y4	MLKL_MC	Mlkl	Mlkl	74568	Mixed linea	no | up	0	COG0515(
Q99KS6	ADPRM_M	Adprm	Adprm	66358	Manganese-	no | no chan	0	COG1409(
Q8CBQ5	P4K2B_MC	Pi4k2b	Pi4k2b	67073	Phosphatid	no | no chan	0	ENOG410
E9Q777	E9Q777_M	Akap11	Akap11	219181	A kinase an	no | no chan	0	ENOG410
H7BX14	H7BX14_M	Tsen34	Tsen34		tRNA-splic	no | no chan	0	COG1676(
Q8BNW9	KBTBB_M	Kbtbd11	Kbtbd11	74901	Kelch repea	no | down	0	ENOG411
A0AAQ4VI	A0AAQ4VMR7_MOUSE	Macrophage-express			Mpeg1	no | up	0	ENOG410
Q99KS2	NGRN_MC	Ngrn	Ngrn	83485	Neugrin	yes | down	1	ENOG411
Q9Z2Z9	GFPT2_MC	Gfpt2	Gfpt2	14584	Glutamine-	no | no chan	0	COG0449(
Q9D8L4	Q9D8L4_MOUSE	Ig-like domain-containing p			Ig-like dom	no | no chan	0	ENOG410

Q8BU20	Q8BU20_M	Ndufb5	Ndufb5	66046	NADH deh	no|no chan	0	ENOG4111
P28474	ADHX_MO	Adh5	Adh5	11532	Alcohol deh	no|no chan	0	COG1062(I
Q8BU22	Q8BU22_M	Kif2c	Kif2c	73804	Kinesin-like	no|no chan	0	COG5059(r
E9QNL5	E9QNL5_M	Sult1a1	Sult1a1	20887	Sulfotransfe	no|down	0	ENOG4111
P63276	RS17_MOU	Rps17	Rps17	20068	Small ribos	no|no chan	0	COG1383(I
Q14BI5	Q14BI5_MO	Myom2	Myom2	17930	Myomesin 2	no|up	0	ENOG410Z
Q6NZM9	HDAC4_M	Hdac4	Hdac4	208727	Histone dec	no|no chan	0	COG0123(I
Q8K119	Q8K119_M	Pms1	Pms1	227099	PMS1 homo	no|no chan	0	COG0323(T
F6SFF5	F6SFF5_MO	Coq9	Coq9		Ubiquinone	no|no chan	0	COG5590(u
A0A1L1SSC	A0A1L1SSC	Ttc13	Ttc13		Tetratricop	no|no chan	0	COG0457(T
O88512	AP1G2_MO	Ap1g2	Ap1g2	11766	AP-1 compl	no|no chan	0	ENOG410X
P68181	KAPCB_M	Prkacb	Prkacb	18749	cAMP-depe	no|no chan	0	ENOG410X
Q9WTR1	TRPV2_MO	Trpv2	Trpv2	22368	Transient r	no|no chan	0	ENOG411C
A0A0A6YW	A0A0A6YW	Map4k4	Map4k4	26921	non-specific	no|no chan	0	ENOG410X
E9QM77	E9QM77_M	Atxn2	Atxn2	20239	Ataxin 2	no|no chan	0	COG5180(s
A2AMQ5	A2AMQ5_M	Cds2	Cds2	110911	Phosphatid	no|no chan	0	COG0575(p
P59048	PDRG1_M	Pdrg1	Pdrg1	68559	p53 and DN	no|no chan	0	ENOG4112
P17439	GBA1_MO	Gba1	Gba1	14466	Lysosomal a	no|no chan	0	COG5520(g
Q7TPM9	MTMRA_M	Mtmr10	Mtmr10	233315	Myotubular	no|no chan	0	ENOG410X
Q8R2U2	NEPRO_M	Nepro	Nepro	212547	Nucleolus c	no|no chan	0	ENOG4111
Q9DCL4	MET15_MO	Mettl15	Mettl15	76894	12S rRNA N	no|no chan	0	COG0275(S
Q9DCL2	CIA2A_MO	Ciao2a	Ciao2a	68250	Cytosolic ir	no|no chan	0	COG5133(p
A0A345IT9	A0A345IT9	env	env		Envelope p	no|no chan	0	ENOG4111
Q9DCL9	PUR6_MO	Paics	Paics	67054	Bifunctiona	no|no chan	0	COG0152(S
Q6P1F5	Q6P1F5_M	Hip1r	Hip1r	29816	Huntingtin	no|no chan	0	ENOG410X
Q8BHE0	PRR11_MO	Prr11	Prr11	270906	Proline-rich	no|no chan	0	ENOG4112
A2A8T9	A2A8T9_M	Efcab14	Efcab14		EF-hand ca	no|no chan	0	ENOG411C
P42337	PK3CA_MO	Pik3ca	Pik3ca	18706	Phosphatid	no|no chan	0	COG5032(p
Q6IRU2	TPM4_MO	Tpm4	Tpm4	326618	Tropomyos	no|no chan	0	ENOG410X
Q8BHE8	MAIP1_MO	Maip1	Maip1	68115	m-AAA pro	no|no chan	0	ENOG4111
Q8CH18	CCAR1_MO	Ccar1	Ccar1	67500	Cell divisio	no|no chan	0	ENOG410Z
A0A0G2JFI	A0A0G2JFI	Frrs1	Frrs1	20321	Ferric-chel	no|no chan	0	ENOG410X
P28293	CATG_MO	Ctsg	Ctsg	13035	Cathepsin G	no|up	0	COG5640(o
A2ALW5	DJC25_MO	Dnajc25	Dnajc25	72429	DnaJ homo	no|no chan	0	COG0484(I
A0A0G2JFI	A0A0G2JFI	Znhit6	Znhit6		Zinc finger,	yes|down	1	ENOG4111
B2RXY7	B2RXY7_M	Cbr1	Cbr1	12408	Carbonyl re	no|no chan	0	COG1028(C
A0A0R4J0D	A0A0R4J0D	Stt3b	Stt3b	68292	dolichyl-dip	no|no chan	0	COG1287(r
A0A0R4J0D	A0A0R4J0D	Saraf	Saraf	67887	Store-opera	no|no chan	0	ENOG4111
A0A0R4J0D	A0A0R4J0D	Mon1a	Mon1a	72825	Vacuolar fu	no|no chan	0	ENOG410X
Q3UND0	SKAP2_MO	Skap2	Skap2	54353	Src kinase-a	no|up	0	ENOG410Z
D3YZ57	D3YZ57_M	Fyn	Fyn		Tyrosine-pr	no|no chan	0	COG0515(c
D3YZ54	D3YZ54_M	Hacl1	Hacl1		2-hydroxya	no|no chan	0	COG0028(c
A0FK58	A0FK58_M	Klra2	Klra2	16633	Killer cell l	no|up	0	ENOG410X

Q8R0Y6	AL1L1_MC	Aldh1l1	Aldh1l1	107747	Cytosolic 1(	no | down	0	COG1012(t
Q5M9J2	Q5M9J2_M	Hint2	Hint2	68917	Histidine tr	no | no chan	0	COG0537(h
Q02248	CTNB1_M(	Ctnnb1	Ctnnb1	12387	Catenin bet	no | no chan	0	COG0035(s
P97302	BACH1_M(	Bach1	Bach1	12013	Transcripti	no | no chan	0	ENOG410Z
P97304	RPAC2_M(	Polr1d	Polr1d	20018	DNA-direc	no | no chan	0	COG1761(M
Q3U4W5	Q3U4W5_M	Rab6a	Rab6a	19346	Ras-related	no | no chan	0	ENOG410X
A0A087WP	A0A087WP	Apbb1	Apbb1	11785	Amyloid be	no | no chan	0	ENOG410Y
A2AJQ2	A2AJQ2_M	Dpy19l4	Dpy19l4		Dpy-19 like	no | no chan	0	ENOG410X
Q5SWY7	FA83G_M(	Fam83g	Fam83g	69640	Protein FAI	no | no chan	0	ENOG410Y
Q9WV55	VAPA_MO	Vapa	Vapa	30960	Vesicle-ass	no | no chan	0	COG5066(I
Q8C259	Q8C259_M	Slc66a3	Slc66a3	217430	Solute carri	no | no chan	0	ENOG4111
F8VPP8	F8VPP8_M	Zc3h7b	Zc3h7b	20286	Zinc finger	no | no chan	0	ENOG410Y
Q8VHZ7	IMP4_MOU	Imp4	Imp4	27993	U3 small nu	no | no chan	0	COG2136(g
Q3TLN7	Q3TLN7_M	Car9	Car9	230099	Carbonic an	no | up	0	COG3338(s
Q6PAJ1	BCR_MOU	Bcr	Bcr	110279	Breakpoint	no | no chan	0	ENOG410X
A0A023J61	A0A023J61	COX1	COX1		Cytochrome	no | up	0	COG0843(C
Q8K009	AL1L2_MC	Aldh1l2	Aldh1l2	216188	Mitochondr	no | no chan	0	COG1012(r
Q9WU28	PFD5_MO	Pfdn5	Pfdn5	56612	Prefoldin su	no | no chan	0	COG1730(M
Q3ULZ3	Q3ULZ3_M	Psat1	Psat1	107272	Phosphoser	no | no chan	0	COG1932(C
A0A0G2JF1	A0A0G2JF1	Gsap	Gsap		Gamma-sec	no | up	0	ENOG4112
B1AVH4	B1AVH4_M	Coro2a	Coro2a	107684	Coronin	no | up	0	ENOG410X
B1AVH7	TBD2A_M(	Tbc1d2	Tbc1d2	381605	TBC1 domc	no | up	0	COG5210(I
M0QWP1	M0QWP1_	Agrn	Agrn	11603	Agrin	no | no chan	0	ENOG410Y
B2RTE3	B2RTE3_M	Prpf38a	Prpf38a	230596	Pre-mRNA	no | no chan	0	ENOG410X
Q4LDD4	ARAP1_M(	Arap1	Arap1	69710	Arf-GAP w	no | no chan	0	COG5347(s
B2RTE7	B2RTE7_M	Klhl17	Klhl17	231003	Kelch-like 1	no | no chan	0	ENOG410X
Q9CX97	WDR55_M	Wdr55	Wdr55	67936	WD repeat-	no | no chan	0	ENOG410Z
B2RTE5	B2RTE5_M	Ccdc102a	Ccdc102a	234582	Coiled-coil	no | no chan	0	ENOG410X
Q8K337	I5P2_MOU	Inpp5b	Inpp5b	16330	Type II ino	no | no chan	0	COG5411(C
A0A5F8MP	A0A5F8MP	Acp2	Acp2		Lysosomal	no | no chan	0	ENOG410Z
Q9Z329	ITPR2_MO	Itpr2	Itpr2	16439	Inositol 1,4,	no | no chan	0	ENOG410X
F8WIZ7	F8WIZ7_M	Osbpl6	Osbpl6	99031	Oxysterol-b	no | no chan	0	ENOG410X
Q8BSL7	ARF2_MO	Arf2	Arf2	11841	ADP-ribosy	no | no chan	0	COG1100(t
A0A0U1RN	A0A0U1RN	Ctf1	Ctf1	13019	Cardiotropl	no | no chan	0	ENOG4111
B7ZN02	B7ZN02_M	Jcad	Jcad	240185	Junctional	no | no chan	0	ENOG411C
Q9DA19	CIR1_MOU	Cir1	Cir1	66935	Corepressor	no | no chan	0	ENOG410Y
Q8C788	Q8C788_M	Snx18	Snx18	170625	Sorting nex	no | no chan	0	ENOG410X
A0A3Q4EC	A0A3Q4EC	Kctd1	Kctd1		Potassium	no | down	0	ENOG410Z
A0A0R4J1B	A0A0R4J1B	Dennd5a	Dennd5a	19347	DENN don	no | no chan	0	ENOG410X
A0A0R4J1B	A0A0R4J1B	Tnnt3	Tnnt3	21957	Troponin T	no | up	0	ENOG410X
Q3UMJ4	Q3UMJ4_M	Gtf2f2	Gtf2f2	68705	General tra	no | no chan	0	COG5090(f
F7A1B4	F7A1B4_M	Eng	Eng	13805	Endoglin	no | no chan	0	ENOG410Y
Q9D103	IFM1_MOU	Ifitm1	Ifitm1	68713	Interferon-i	no | no chan	0	ENOG4112

Q5RL20	Q5RL20_M	Mrpl43	Mrpl43	94067	Large ribos	no	no chan	0	ENOG4111
Q9JI39	ABCBA_M	Abcb10	Abcb10	56199	ATP-bindir	no	no chan	0	COG1132(I
A0A1G5SK	A0A1G5SK	OTTMUSP	OTTMUSP	327978	Schlafen fa	no	no chan	0	ENOG410Z
N0E4C0	N0E4C0_M	Csnk2b	Csnk2b	13001	Casein kina	no	no chan	0	COG5041(c
B2RXQ8	B2RXQ8_M	Wdr82	Wdr82	77305	WD repeat	no	no chan	0	ENOG410X
A7YY80	A7YY80_M	Epb41l3	Epb41l3	13823	130kDa Pro	no	no chan	0	ENOG410Y
Q3U7U3	FBX7_MOU	Fbxo7	Fbxo7	69754	F-box only	no	no chan	0	ENOG410X
E9QAE4	E9QAE4_M	Nsd1	Nsd1	18193	Nuclear rec	no	no chan	0	COG2940(A
Q80V53	CHSTE_MO	Chst14	Chst14	72136	Carbohydra	no	no chan	0	ENOG4111
C0HKD8	MFA1A_M	Mfap1a	Mfap1a	100034361;	Microfibrill	no	no chan	0	ENOG410Y
A0A494B9T	A0A494B9T	Cxxc5	Cxxc5		CXXC fing	no	no chan	0	ENOG411C
P58137	ACOT8_M	Acot8	Acot8	170789	Acyl-coenzy	no	no chan	0	COG1946(c
F8WGA9	F8WGA9_M	Nek4	Nek4		non-specifi	no	no chan	0	ENOG410Y
Q923W1	TGS1_MOU	Tgs1	Tgs1	116940	Trimethylg	no	no chan	0	ENOG410X
Q8CG70	P3H3_MOU	P3h3	P3h3	14789	Prolyl 3-hy	no	no chan	0	ENOG410X
Q3TB02	Q3TB02_M	Bloc1s5	Bloc1s5	17828	Biogenesis	no	no chan	0	ENOG4111
S4R165	S4R165_MO	Ank3	Ank3		Ankyrin 3, e	no	no chan	0	COG0666(A
Q549X4	Q549X4_M	Rab27b	Rab27b	80718	small mono	no	no chan	0	ENOG410X
Q9CWV6	PKRI1_MO	Prkrip1	Prkrip1	66801	PRKR-inter	no	no chan	0	ENOG4111
Q9CWV0	MASU1_M	Malsu1	Malsu1	75593	Mitochondr	no	no chan	0	COG0799(F
Q8CDC7	ZBTB9_MO	Zbtb9	Zbtb9	474156	Zinc finger	no	down	0	COG5048(c
D3YYE1	D3YYE1_M	Anp32a	Anp32a		Acidic leuci	no	no chan	0	ENOG4111
D3YYE2	D3YYE2_M	Eef2kmt	Eef2kmt		Eukaryotic	no	no chan	0	ENOG410Z
Q3UC14	Q3UC14_M	Sfxn2	Sfxn2	94279	Sidoreflexin	no	no chan	0	ENOG410Y
Q8CIM8	INT4_MOU	Ints4	Ints4	101861	Integrator c	no	no chan	0	ENOG410X
Q4VA32	Q4VA32_M	Acot13	Acot13	66834	Thioesteras	no	no chan	0	COG2050(t
Q9Z2G0	FEM1B_M	Fem1b	Fem1b	14155	Protein fem	no	no chan	0	COG0666(M
Q3UDP0	WDR41_M	Wdr41	Wdr41	218460	WD repeat-	no	no chan	0	ENOG410Z
Q9D8V0	SPP_MOUS	Hm13	Hm13	14950	Minor histo	no	no chan	0	ENOG410X
Q3TZZ7	ESYT2_MO	Esyt2	Esyt2	52635	Extended sy	no	no chan	0	ENOG410X
Q8BK35	NOP53_MO	Nop53	Nop53	68077	Ribosome b	no	no chan	0	ENOG4111
B2ZAC8	B2ZAC8_M	Tcf25	Tcf25	66855	Nuclear loc	no	no chan	0	ENOG410X
Q5XK33	Q5XK33_M	Sdhc	Sdhc	66052	Succinate d	no	no chan	0	COG2009(s
P49769	PSN1_MOU	Psen1	Psen1	19164	Presenilin-1	no	no chan	0	ENOG410X
Q5PRF0	HTR5A_M	Heatr5a	Heatr5a	320487	HEAT repe	no	no chan	0	ENOG410X
Q5SX40	MYH1_MO	Myh1	Myh1	17879	Myosin-1	no	up	0	COG5022(c
Q3TPJ7	MIDN_MO	Midn	Midn	59090	Midnolin	no	down	0	ENOG410X
A0A3Q4EC	A0A3Q4EC	Zfp472	Zfp472		Zinc finger	no	down	0	COG5048(C
Q8BVK9	SP110_MO	Sp110	Sp110	109032	Sp110 nucle	no	no chan	0	ENOG4111
Q63829	COMD3_M	Commd3	Commd3	12238	COMM dor	no	no chan	0	ENOG4111
Q9D8Z1	ASCC1_MO	Ascc1	Ascc1	69090	Activating s	no	no chan	0	ENOG4111
P13439	UMPS_MO	Umps	Umps	22247	Uridine 5'-r	no	no chan	0	COG0284(C
Q3UHU5	MTCL1_M	Mtcl1	Mtcl1	68617	Microtubul	no	no chan	0	ENOG410X

A0A087WN	A0A087WN	Fam168b	Fam168b		Myelin-assc	no|no chan	0	ENOG410Y
G3UZP7	G3UZP7_M	H2-D1	H2-D1		Histocompc	no|up	0	ENOG4111
Q8R2Z0	ADIPL_MC	C1qtnf12	C1qtnf12	67389	Adipolin	no|no chan	0	ENOG411C
Q3UHU8	Q3UHU8_M	Gtf2i	Gtf2i	14886	General tra	no|no chan	0	ENOG411C
Q6DI86	FAKD1_M	Fastkd1	Fastkd1	320720	FAST kinas	no|no chan	0	ENOG410Z
Q6DI85	Q6DI85_M	Kctd18	Kctd18	51960	Kctd18 pro	no|no chan	0	ENOG410Z
A0A1L1SR5	A0A1L1SR5	Calml4	Calml4	75600	Calmodulir	no|up	0	COG5126(c
Q9CU62	SMC1A_MC	Smc1a	Smc1a	24061	Structural r	no|no chan	0	COG1196(c
E9PVD3	PCD16_MC	Dchs1	Dchs1	233651	Protocadhe	no|no chan	0	ENOG410X
Q50HX2	Q50HX2_M	Rab14	Rab14	68365	Ras-related	no|no chan	0	COG1100(-
Q9D842	APLF_MO	Aplf	Aplf	72103	Aprataxin c	no|no chan	0	ENOG4112
A0A2X0SSI	A0A2X0SSI	ARHGAP1.	ARHGAP1.	76117	Rho GTPas	no|no chan	0	ENOG410Z
Q8K2Z8	UB2Q2_MC	Ube2q2	Ube2q2	109161	Ubiquitin-c	no|no chan	0	ENOG410X
Q8K2R5	ZN668_MC	Znf668	Znf668	244219	Zinc finger	no|no chan	0	COG5048(I
Q9D6L8	PPIL3_MO	Ppil3	Ppil3	70225	Peptidyl-pr	no|no chan	0	COG0652(M
Q6PB97	FXL19_MC	Fbxl19	Fbxl19	233902	F-box/LRR	no|no chan	0	ENOG410Z
Q5U430	UBR3_MO	Ubr3	Ubr3	68795	E3 ubiquiti	no|no chan	0	ENOG410X
P51410	RL9_MOUS	Rpl9	Rpl9	20005	Large ribos	no|no chan	0	COG0097(T
E9Q1L2	E9Q1L2_M	Klhl5	Klhl5	71778	Kelch-like 5	no|no chan	0	ENOG410X
Q6ZPG3	Q6ZPG3_M	Usp38	Usp38	74841	ubiquitinyl	no|no chan	0	ENOG410X
Q3US15	Q3US15_M	Nsdhl	Nsdhl	18194	NAD(P)-de	no|no chan	0	COG0451(M
E9Q1L7	E9Q1L7_M	Dnajc28	Dnajc28	246738	DnaJ heat s	no|down	0	ENOG410Z
Q3US17	ZNF48_MC	Znf48	Znf48	233887	Zinc finger	no|down	0	COG5048(T
E9Q1L5	E9Q1L5_M	Senp7	Senp7	66315	SUMO1/se	no|no chan	0	COG5160(S
A2ARA0	A2ARA0_M	Tfpi	Tfpi		Tissue facto	no|no chan	0	ENOG410X
P84075	HPCA_MO	Hpca	Hpca	15444	Neuron-spe	no|no chan	0	COG5126(s
P97742	CPT1A_MC	Cpt1a	Cpt1a	12894	Carnitine C	no|no chan	0	ENOG410X
Q3UJQ2	Q3UJQ2_M	Hmgcs1	Hmgcs1	208715	Hydroxyme	no|down	0	COG3425(h
Q8CB62	CNTRB_M	Cntrob	Cntrob	216846	Centrobin	no|no chan	0	ENOG4111
A2CEE5	A2CEE5_M	Rps6ka6	Rps6ka6		Ribosomal	no|no chan	0	ENOG410X
P84078	ARF1_MO	Arf1	Arf1	11840	ADP-ribosy	no|no chan	0	COG1100(C
Q3LR78	Q3LR78_M	Zbtb38	Zbtb38	245007	CtBP-interc	no|no chan	0	COG5048(t
A0A1L1SQ'	A0A1L1SQ'	Ss18l2	Ss18l2	26901	SS18, nBAF	no|no chan	0	ENOG4112
Q60914	Q60914_MC	Zfp87	Zfp87	170763	KRAB-zinc	no|up	0	COG5048(I
Q3U3L3	Q3U3L3_M	Serpinb6a	Serpinb6a	20719	Serpin B6	no|no chan	0	COG4826(s
Q8CAS9	PARP9_MC	Parp9	Parp9	80285	Protein mo	no|up	0	COG2110(M
Q8K0H5	TAF10_MC	Taf10	Taf10	24075	Transcripti	no|no chan	0	COG5162(T
Q3TDK6	ROGDI_M	Rogdi	Rogdi	66049	Protein rog	no|no chan	0	ENOG410Y
Q8VDY4	EFCB7_MC	Efcab7	Efcab7	230500	EF-hand ca	no|no chan	0	COG5126(k
Q91YY4	ATPF2_MC	Atpaf2	Atpaf2	246782	ATP syntha	no|no chan	0	COG5387(A
Q9CTN4	RHBT3_MC	Rhobtb3	Rhobtb3	73296	Rho-related	no|no chan	0	COG1100(C
A0PK78	A0PK78_M	Ccdc25	Ccdc25	67179	Coiled-coil	no|no chan	0	ENOG410Z
Q149D1	Q149D1_M	Thnsl1	Thnsl1	208967	Threonine s	no|no chan	0	COG0498(I

P48410	ABCD1_M(	Abcd1	Abcd1	11666	ATP-bindir	no | no chan	0	COG4178(i
A2RS58	A2RS58_M(	Crkl	Crkl	12929	V-crk sarco	no | no chan	0	ENOG411(
Q9Z2L7	CRLF3_MC	Crlf3	Crlf3	54394	Cytokine re	no | no chan	0	ENOG411(
Q5JCS9	B3GN3_M(	B3gnt3	B3gnt3	72297	N-acetyllac	no | no chan	0	ENOG410Z
F6UKN5	F6UKN5_M	Septin2	Septin2		Septin 2	no | up	0	COG5019(s
A2RS53	A2RS53_M(	Tmed2	Tmed2	56334	Transmemb	no | no chan	0	ENOG410X
A0A1B0GS	A0A1B0GS	Tti2	Tti2	234138	TELO2 inte	no | no chan	0	ENOG4111
Q3TKY6	CWC27_M(	Cwc27	Cwc27	67285	Spliceosom	no | no chan	0	COG0652(C
Q3TZS3	Q3TZS3_M	Itga7	Itga7	16404	Integrin alp	no | down	0	ENOG410X
Q8VE38	OXND1_M	Oxnad1	Oxnad1	218885	Oxidoreduc	no | no chan	0	COG0543(c
Q3U425	Q3U425_M	Pom121	Pom121	107939	Nuclear env	no | no chan	0	ENOG4112
Q3U1L8	Q3U1L8_M	Sidt2	Sidt2	214597	SID1 transr	no | no chan	0	ENOG410X
D3Z136	D3Z136_M	Elovl5	Elovl5		Elongation	no | no chan	0	ENOG410X
Q8BG55	GP171_MC	Gpr171	Gpr171	229323	G-protein c	no | no chan	0	ENOG410Y
S4R2A9	S4R2A9_M(	Sec31a	Sec31a		Protein trar	no | down	0	ENOG410X
Q5NCI0	URGCP_M	Urgcp	Urgcp	72046	Up-regulate	no | no chan	0	ENOG410Y
Q5F283	Q5F283_M(	Plscr3	Plscr3	70310	Phospholip	no | up	0	ENOG410X
Q3UYK8	Q3UYK8_M	Rab3gap1	Rab3gap1	226407	Rab3 GTPa	no | no chan	0	ENOG410Y
P70302	STIM1_MC	Stim1	Stim1	20866	Stromal int	no | no chan	0	ENOG410X
P70303	PYRG2_M(	Ctps2	Ctps2	55936	CTP syntha	no | no chan	0	COG0504(C
A0A0A6YX	A0A0A6YX	Ighv8-8	Ighv8-8		Immunoglo	no | no chan	0	ENOG4111
Q9CZW4	ACSL3_MC	Acsl3	Acsl3	74205	Fatty acid C	no | no chan	0	COG1022(A
Q544I4	Q544I4_M(	Padi1	Padi1	18599	Protein-arg	no | down	0	ENOG410Z
Q9CZW2	CENPN_M	Cenpn	Cenpn	72155	Centromere	no | no chan	0	ENOG4111
Q544I1	Q544I1_M(	Ndrg3	Ndrg3	29812	Protein ND	no | no chan	0	ENOG410X
Q3U4G3	XXLT1_M(	Xxylt1	Xxylt1	268880	Xyloside xy	no | no chan	0	ENOG410X
E9Q6R7	E9Q6R7_M	Utrn	Utrn	22288	Utrophin	no | no chan	0	COG5069(Y
Q6TKP2	Q6TKP2_M	Npcd	Npcd	504193	Neuronal p	no | no chan	0	ENOG410Z
A2AL85	A2AL85_M	Asph	Asph	65973	Aspartate-b	no | no chan	0	COG3555(b
P57716	NICA_MO	Ncstn	Ncstn	59287	Nicastrin	no | no chan	0	ENOG410X
Q9DCI9	RM32_MO	Mrpl32	Mrpl32	75398	Large ribos	no | no chan	0	COG0333(5
Q91VA6	PDIP2_MC	Poldip2	Poldip2	67811	Polymerase	no | no chan	0	COG2967(A
Q91VA7	Q91VA7_M	Idh3b	Idh3b	170718	Isocitrate d	no | no chan	0	COG0473(3
A0A0U1RN	A0A0U1RN	B9d2	B9d2		B9 domain-	no | no chan	0	ENOG410Z
O89032	SPD2A_M(	Sh3pxd2a	Sh3pxd2a	14218	SH3 and PX	no | no chan	0	ENOG410Y
Q3U2X7	Q3U2X7_M	Ccn3	Ccn3	18133	CCN family	no | no chan	0	ENOG4111
A0A0U1RN	A0A0U1RN	Csnk1g1	Csnk1g1	214897	Casein kina	no | no chan	0	ENOG410X
Q04187	Q04187_M(	L factor typ	L factor type I (LF1)		Large T ant	no | down	0	
A0A068BD	A0A068BD	Rxrb	Rxrb	20182	Putative ret	no | no chan	0	ENOG410X
Q811E7	Q811E7_M	Myo1f	Myo1f	17916	Myosin IF	no | no chan	0	COG5022(T
Q543E2	Q543E2_M	Slc25a15	Slc25a15	18408	Solute carri	no | no chan	0	ENOG410X
O08750	NFIL3_MC	Nfil3	Nfil3	18030	Nuclear fac	no | no chan	0	ENOG410Z
A0A0R4J19	A0A0R4J19	Robo4	Robo4	74144	Roundabou	no | no chan	0	ENOG410Y

O08759	UBE3A_M(	Ube3a	Ube3a	22215	Ubiquitin-p	no|no chan	0 COG5021(r
A0A0G2JE8	A0A0G2JE8	Csn2	Csn2		Beta-casein	no|up	0 ENOG4111
Q8C6B9	AROS_MO	Rps19bp1	Rps19bp1	66538	Active regu	no|no chan	0 ENOG4112
A2AD84	A2AD84_M	Stk26	Stk26		non-specifi	no|up	0 ENOG410X
A2ABY3	A2ABY3_M	Pcyt2	Pcyt2	68671	ethanolami	no|no chan	0 COG0615(e
Q9D0G0	RT30_MOU	Mrps30	Mrps30	59054	Large ribos	no|no chan	0 ENOG410Z
A0A0U1RP	A0A0U1RP	Spns1	Spns1		Protein spir	no|up	0 COG0477(V
A0A571BE	A0A571BE	Ralgds	Ralgds		Ral guanin	no|up	0 ENOG410Z
Q505B1	Q505B1_M	Rpl17	Rpl17	319195	Large ribos	no|no chan	0 COG0091(T
A0A0U1RP	A0A0U1RP	Arl6ip1	Arl6ip1		ADP-ribosy	no|no chan	0 ENOG410Z
Q5U5I3	Q5U5I3_M	Mrps18a	Mrps18a	68565	Mitochondr	no|no chan	0 COG0238(I
Q80ZM5	Q80ZM5_M	H1f10	H1f10	243529	H1 histone	no|down	0 ENOG4112
E9Q526	E9Q526_M	C2cd3	C2cd3		C2 calcium	no|no chan	0 ENOG410X
P35486	ODPA_MC	Pdha1	Pdha1	18597	Pyruvate de	no|no chan	0 COG1071(I
Q9CQG1	CHAC2_M(	Chac2	Chac2	68044	Putative gl	no|no chan	0 COG3703(c
D3Z394	D3Z394_M	Wdr54	Wdr54		WD repeat-	no|no chan	0 ENOG410X
D3Z4B2	D3Z4B2_M	Napg	Napg		Gamma-sol	no|no chan	0 ENOG410Z
Q8VI93	OAS3_MO	Oas3	Oas3	246727	2'-5'-oligoa	no|up	0 COG5272(C
H3BL44	H3BL44_M	Atp2c1	Atp2c1		Calcium-tr	no|no chan	0 COG0474(I
Q8VI94	OASL1_M(	Oasl1	Oasl1	231655	2'-5'-oligoa	no|up	0 COG5272(r
A2AWA9	RBGP1_M(	Rabgap1	Rabgap1	227800	Rab GTPas	no|no chan	0 COG5210(p
E9Q0C1	E9Q0C1_M	Sh3kbp1	Sh3kbp1		SH3-domai	no|no chan	0 ENOG410X
E9PY28	E9PY28_M	Btbd2	Btbd2		BTB domai	no|up	0 ENOG410X
F6SMY7	F6SMY7_M	Mycbp2	Mycbp2		RCR-type E	no|no chan	0 ENOG410X
A0A1D5RL	A0A1D5RL	Myo9b	Myo9b		Myosin IXb	no|no chan	0 COG5022(I
A0A8X8M4	A0A8X8M4	Fggy	Fggy	75578	FGGY vari	no|no chan	0 COG1069(r
Q3U0F3	Q3U0F3_M	Itm2c	Itm2c	64294	Integral me	no|no chan	0 ENOG410X
A0A2I3BS1	A0A2I3BS1	Nsmce2	Nsmce2	68501	E3 SUMO-p	no|no chan	0 COG5627(r
A0A140LIS	A0A140LIS	Arglu1	Arglu1		Arginine ar	no|no chan	0 ENOG4111
A0A0J9YUV	A0A0J9YUV	Scn8a	Scn8a	20273	Sodium cha	no|no chan	0 ENOG410X
Q64442	DHSO_MC	Sord	Sord	20322	Sorbitol del	no|no chan	0 COG1063(I
A0A2I3BQ9	A0A2I3BQ9	Ubr5	Ubr5	70790	Ubiquitin p	no|no chan	0 COG5021(N
Q8K274	KT3K_MO	Fn3krp	Fn3krp	238024	Ketosamine	no|no chan	0 COG3001(I
Q8K273	EMC5_MO	Mmgt1	Mmgt1	236792	ER membr	no|no chan	0 ENOG4111
Q9DBM2	ECHP_MO	Ehhadh	Ehhadh	74147	Peroxisoma	no|no chan	0 COG1250(I
P54099	DPOG1_M	Polg	Polg	18975	DNA polyn	no|no chan	0 COG0749(I
P54763	EPHB2_M(	Ephb2	Ephb2	13844	Ephrin type	no|no chan	0 COG0515(I
Q9WTI7	MYO1C_M	Myo1c	Myo1c	17913	Unconventi	no|no chan	0 COG5022(C
A0A2R8W6	A0A2R8W6	Prph	Prph	19132	Peripherin	no|down	0 ENOG410Y
A2ANZ2	A2ANZ2_M	C9orf72	C9orf72		C9orf72, m	no|no chan	0 ENOG4111
Q9CY58	SERB1_MC	Serbp1	Serbp1	66870	SERPINE1	no|no chan	0 ENOG410Y
Q99LL5	PWP1_MO	Pwp1	Pwp1	103136	Periodic try	no|no chan	0 ENOG410X
F6Z7L1	F6Z7L1_M(	Tnrc18	Tnrc18		Trinucleoti	no|no chan	0 ENOG410X

Q0R0H6	Q0R0H6_M	A4galt	A4galt	239559	Lactosylcer	no | no chan	0	ENOG4111
A0A0R4J04	A0A0R4J04	Prmt5	Prmt5	27374	Protein arg	no | no chan	0	ENOG410
P26883	FKB1A_M	Fkbp1a	Fkbp1a	14225	Peptidyl-pr	no | no chan	0	COG0545(
A0A0R4J04	A0A0R4J04	Bfar	Bfar	67118	Bifunction	no | no chan	0	COG2802(
A0A0R4J04	A0A0R4J04	Mettl3	Mettl3	56335	mRNA m(6	no | no chan	0	COG4725(
A0A0R4J04	A0A0R4J04	Cebpz	Cebpz	12607	CCAAT/en	no | no chan	0	COG5593(
A0A0R4J04	A0A0R4J04	Luc7l	Luc7l	66978	Luc7-like	no | no chan	0	COG5200(
A0A0R4J04	A0A0R4J04	Rrp36	Rrp36	224823	rRNA biog	no | no chan	0	ENOG4111
E9Q3C1	C2CD2_M	C2cd2	C2cd2	207781	C2 domain-	no | no chan	0	ENOG411
A0A0R4J0C	A0A0R4J0C	Mettl25	Mettl25	216292	Methyltran	no | up	0	ENOG410
Q3UQ44	IQGA2_M	Iqgap2	Iqgap2	544963	Ras GTPas	no | no chan	0	COG5261(
A2A5Z6	SMUF2_M	Smurf2	Smurf2	66313	E3 ubiquiti	no | no chan	0	COG5021(
E0CXA9	E0CXA9_M	Mob4	Mob4	19070	MOB-like p	no | no chan	0	ENOG410
Q8CIR9	Q8CIR9_M	3	3	17174	Serine prot	no | up	0	COG5640(
Q8K0U4	HS12A_M	Hspa12a	Hspa12a	73442	Heat shock	no | no chan	0	COG0443(
P97449	AMPN_M	Anpep	Anpep	16790	Aminopept	no | no chan	0	COG0308(
O35239	PTN9_MO	Ptpn9	Ptpn9	56294	Tyrosine-pr	no | no chan	0	COG5599(
Q9JJZ6	KLF13_M	Klf13	Klf13	50794	Krueppel-li	no | no chan	0	COG5048(
P97443	SMYD1_M	Smyd1	Smyd1	12180	Histone-lys	no | up	0	COG2940(
B2RXY0	B2RXY0_M	Katnip	Katnip	233865	KATNIP d	no | up	0	ENOG410
A0A0M3HI	A0A0M3HI	Txnrd2	Txnrd2		thioredoxin	no | no chan	0	COG1249(
P97447	FHL1_MO	Fhl1	Fhl1	14199	Four and a	no | up	0	ENOG410
A0A0R4IZ	A0A0R4IZ	Ncan	Ncan	13004	Neurocan	no | no chan	0	ENOG410
G5E8C6	G5E8C6_M	Vill	Vill	22351	Villin-like	no | no chan	0	ENOG410
A0A0R4IZ	A0A0R4IZ	Def6	Def6	23853	Differentia	no | no chan	0	ENOG410
A0A0R4IZ	A0A0R4IZ	Med6	Med6	69792	Mediator o	no | no chan	0	COG5097(
A0A0R4IZ	A0A0R4IZ	Nherf2	Nherf2	65962	Na(+)/H(+	no | no chan	0	ENOG4111
Q545J3	Q545J3_M	Hgfac	Hgfac	54426	Hepatocyte	no | no chan	0	COG5640(
A2AED8	A2AED8_M	Spata6	Spata6		Spermatoge	no | down	0	ENOG4111
Q711L0	Q711L0_M	Spp2	Spp2	75396	Secreted ph	no | no chan	0	ENOG410
Q6NV99	Q6NV99_M	Haus6	Haus6	230376	HAUS augr	no | no chan	0	ENOG4111
Q3U057	Q3U057_M	Cpsf3	Cpsf3	54451	Cleavage a	no | no chan	0	COG1236(
Q8C440	Q8C440_M	Tmcc3	Tmcc3	319880	Transmemb	no | no chan	0	ENOG410
Q91ZE0	TMLH_MC	Tmlhe	Tmlhe	192289	Trimethylly	no | no chan	0	COG2175(
A6H619	PHRF1_M	Phrf1	Phrf1	101471	PHD and R	no | no chan	0	ENOG410
Q06770	CBG_MOU	Serpina6	Serpina6	12401	Corticoster	no | up	0	COG4826(
A6H611	MIPEP_M	Mipep	Mipep	70478	Mitochond	no | no chan	0	COG0339(
P16332	MUTA_MC	Mmut	Mmut	17850	Methylmal	no | no chan	0	COG1884(
P16330	CN37_MO	Cnp	Cnp	12799	2',3'-cyclic-	no | no chan	0	ENOG4111
Q9R118	HTRA1_M	Htra1	Htra1	56213	Serine prot	no | up	0	COG0265(
K0J1C6	K0J1C6_M	Extl2	Extl2	58193	Alpha-1,4-	no | no chan	0	ENOG410
B2RVL6	ZCH24_M	Zcchc24	Zcchc24	71918	Zinc finger	no | no chan	0	ENOG410
A0A0A0M	A0A0A0M	Macf1	Macf1		Microtubul	no | no chan	0	COG5069(

Q6DFU8	Q6DFU8_MZfp948	Zfp948	381066	Zinc finger	no | down		0	COG5048(I
G5DDB7	G5DDB7_MPlcd1	Plcd1	18799	Phosphoinc	no | no chan		0	ENOG410X
P62141	PP1B_MOUPpp1cb	Ppp1cb	19046	Serine/thre	no | no chan		0	ENOG410X
Q9JJ06	C1GLT_MOC1galt1	C1galt1	94192	Glycoprote	no | no chan		0	ENOG410Y
Q7TMH5	Q7TMH5_MMrpl13	Mrpl13	68537	Mrpl13 pro	no | no chan		0	COG0102(T
D3YZ89	D3YZ89_MMtfr1	Mtfr1		Mitochondı	no | no chan		0	ENOG4111
Q0P5W1	VPS8_MOUVps8	Vps8	209018	Vacuolar p	no | no chan		0	ENOG410X
Q0P5W2	Q0P5W2_MTmx4	Tmx4	52837	Thioredoxi	no | no chan		0	ENOG4111
Q9D4C5	EAF1_MOUEaf1	Eaf1	74427	ELL-associ	no | no chan		0	ENOG4111
Q0P5W9	Q0P5W9_MRfx5	Rfx5	53970	Rfx5 protei	no | up		0	ENOG410X
Q3TN61	Q3TN61_MRhoa	Rhoa	11848	Transformi	no | no chan		0	COG1100(C
Q8C2R7	PIGM_MO Pigm	Pigm	67556	GPI mannc	no | no chan		0	ENOG410X
P09926	SURF2_MCSurf2	Surf2	20931	Surfeit locu	no | no chan		0	ENOG4111
O35490	BHMT1_M Bhmt	Bhmt	12116	Betaine--hc	no | down		0	COG0646(I
A2AQN4	A2AQN4_MAcss2	Acss2		Propionate-	no | no chan		0	COG0365(I
A8DUN2	A8DUN2_MHbbt1	Hbbt1	100503605	Beta-globin	no | no chan		0	COG1018(I
Q9JKX4	AATF_MO Aatf	Aatf	56321	Protein AA'	no | no chan		0	ENOG410X
Q9JKX6	NUDT5_M Nudt5	Nudt5	53893	ADP-sugar	no | no chan		0	COG0494(c
O35943	FRDA_MOFxn	Fxn	14297	Frataxin, m	no | no chan		0	COG1965(f
A0A0N4SV	A0A0N4SV Etnk1	Etnk1		ethanolami	no | no chan		0	COG0510(I
Q80YD1	SUV3_MOUSupv3l1	Supv3l1	338359	ATP-depen	no | no chan		0	ENOG410X
Q9CXT7	TM192_MCTmem192	Tmem192	73067	Transmemb	no | no chan		0	ENOG4111
Q69Z66	MYSM1_M Mysm1	Mysm1	320713	Deubiquiti	no | no chan		0	COG1310(C
E9QPD7	E9QPD7_MPcx	Pcx	18563	Pyruvate ca	no | down		0	COG1038(C
Q69Z61	PWP2A_MOPwwp2a	Pwwp2a	70802	PWWP dom	no | up		0	ENOG4111
P13595	NCAM1_MNcam1	Ncam1	17967	Neural cell	no | no chan		0	ENOG410X
Q3UMW7	MAPK3_M Mapkapk3	Mapkapk3	102626	MAP kinas	no | no chan		0	ENOG410X
A0A140T8N	A0A140T8NIgkv6-32	Igkv6-32	434039	Immunoglo	no | no chan		0	ENOG410Y
Q99L13	3HIDH_MOHibadh	Hibadh	58875	3-hydroxyis	no | no chan		0	COG2084(I
Q91Y57	SIG12_MO Siglec12	Siglec12	83382	Sialic acid-l	no | up		0	ENOG410Y
Q8CCI5	RYBP_MO Rybp	Rybp	56353	RING1 and	no | no chan		0	ENOG4111
P17751	TPIS_MOUTpi1	Tpi1	21991	Triosephos	no | up		0	COG0149(t
E9PYJ7	E9PYJ7_MOPitpnm2	Pitpnm2		Phosphatid	yes | down		1	COG5083(I
Q9D708	S10AG_MCS100a16	S100a16	67860	Protein S10	no | no chan		0	ENOG4111
Q3TR93	Q3TR93_MDecr2	Decr2	26378	Peroxisoma	no | no chan		0	COG1028(t
E9PYJ9	E9PYJ9_MOLdb3	Ldb3	24131	LIM domai	no | up		0	ENOG410X
Q6NXY1	TBC31_MCTbc1d31	Tbc1d31	210544	TBC1 domc	no | no chan		0	ENOG410Y
Q91X83	METK1_M Mat1a	Mat1a	11720	S-adenosylr	no | down		0	COG0192(C
B7ZCT1	B7ZCT1_MRgs19	Rgs19	56470	Regulator c	no | no chan		0	ENOG410Y
B1AQD9	B1AQD9_MUnc119	Unc119	22248	Protein unc	no | up		0	ENOG410Y
Q91X84	CRTC3_MOCrtc3	Crtc3	70461	CREB-regu	no | no chan		0	ENOG410X
Q9CY97	SSU72_MO Ssu72	Ssu72	68991	RNA polym	no | no chan		0	COG5211(I
Q3U741	Q3U741_MDdx17	Ddx17	67040	RNA helicc	no | no chan		0	COG0513(t

Q91X88	PMGT1_M	Pomgnt1	Pomgnt1	68273	Protein O-l	no | no chan	0	ENOG410Y
E0CYE2	E0CYE2_M	Impdh1	Impdh1		Inosine-5'-n	no | no chan	0	COG0517(g
A0A140LH'	A0A140LH'	Mrpl48	Mrpl48	52443	Mitochondı	no | no chan	0	ENOG4111
Q8BTX9	HSDL1_M(	Hsdl1	Hsdl1	72552	Inactive hy	no | no chan	0	COG0300(I
Q99LD8	DDAH2_M	Ddah2	Ddah2	51793	Putative hy	no | no chan	0	ENOG4111
P70178	SIX5_MOU	Six5	Six5	20475	Homeobox	no | no chan	0	ENOG410X
Q8BTX6	Q8BTX6_M	Mtrex	Mtrex	72198	MTREX he	no | no chan	0	COG4581(r
O88685	PRS6A_MC	Psmc3	Psmc3	19182	26S proteas	no | no chan	0	COG1222(2
Q9JHH9	COPZ2_M(	Copz2	Copz2	56358	Coatomer s	no | no chan	0	COG5541(v
Q00612	G6PD1_M(	G6pdx	G6pdx	14381	Glucose-6-p	no | no chan	0	COG0364(C
Q9JHH6	CBPB2_MC	Cpb2	Cpb2	56373	Carboxypep	no | up	0	COG2866(r
A0A0G2JG	A0A0G2JG	Shisa5	Shisa5		Protein shis	yes | up	1	ENOG410Y
Q9Z2X1	HNRPF_M	Hnrnpf	Hnrnpf	98758	Heterogene	no | no chan	0	ENOG410Z
Q7TN37	TRPM4_M(	Trpm4	Trpm4	68667	Transient r	no | no chan	0	ENOG410X
A0A0G2JG	A0A0G2JG	Crlf2	Crlf2	57914	Cytokine re	no | down	0	ENOG4111
P14901	HMOX1_M	Hmox1	Hmox1	15368	Heme oxyg	no | no chan	0	COG5398(I
A0A0G2JG	A0A0G2JG	Nras	Nras		GTPase NF	no | no chan	0	COG1100(C
B1AXS5	B1AXS5_M	Zmym3	Zmym3	56364	Zinc finger,	no | no chan	0	ENOG410X
I3PQX0	I3PQX0_MOUSE	DUF4605 domai		70617	DUF4605 d	no | no chan	0	ENOG4112
Q5XKN4	JAGN1_M(	Jagn1	Jagn1	67767	Protein jag	no | no chan	0	ENOG4111
E9QLK3	E9QLK3_M	Brat1	Brat1	231841	BRCA1-ass	no | no chan	0	ENOG411C
A0A0U1RP	A0A0U1RP	Selenos	Selenos	109815	Selenoprote	no | no chan	0	ENOG4112
K3W4S6	K3W4S6_M	Gyg1	Gyg1		glycogenin	no | up	0	COG5597(C
A0A1W2P7	A0A1W2P7	Asf1a	Asf1a		Anti-silenci	no | no chan	0	COG5137(S
Q9ERS2	NDUAD_M	Ndufa13	Ndufa13	67184	NADH deh	no | no chan	0	ENOG4111
Q3USA5	Q3USA5_M	Fstl5	Fstl5	213262	Follistatin-	no | no chan	0	ENOG410Y
Q9ERS5	PKHA2_M(	Plekha2	Plekha2	83436	Pleckstrin h	no | no chan	0	ENOG410Z
D3YXD1	D3YXD1_M	Ube2e2	Ube2e2		E2 ubiquiti	no | no chan	0	COG5078(Y
Q80UX8	ABHDD_M	Abhd13	Abhd13	68904	Protein AB	no | no chan	0	COG1073(S
Q546X9	Q546X9_M	Atxn3	Atxn3	110616	ubiquitinyl	no | no chan	0	ENOG4111
B1AXI9	B1AXI9_M	Cep131	Cep131	12009	5-azacytidi	no | no chan	0	ENOG410Y
O88532	ZFR_MOU	Zfr	Zfr	22763	Zinc finger	no | no chan	0	ENOG410X
O88531	PPT1_MOU	Ppt1	Ppt1	19063	Palmitoyl-p	no | no chan	0	COG1075(T
P62878	RBX1_MO	Rbx1	Rbx1	56438	E3 ubiquiti	no | no chan	0	COG5194(T
D3Z090	D3Z090_M	Csf1	Csf1		Colony stin	no | up	0	ENOG410Z
Q4FK31	Q4FK31_M	Mid1ip1	Mid1ip1	68041	Mid1ip1 pr	no | down	0	ENOG4111
B1ARB3	B1ARB3_M	Pecam1	Pecam1	18613	Platelet/en	no | no chan	0	ENOG4111
Q4FK36	Q4FK36_M	Dstn	Dstn	56431	Destrin	no | no chan	0	ENOG4112
Q8VCF0	MAVS_MC	Mavs	Mavs	228607	Mitochondı	no | no chan	0	ENOG410Y
Q8VCF1	CANT1_M(	Cant1	Cant1	76025	Soluble calc	no | up	0	ENOG410X
Q9DCJ1	LST8_MOU	Mlst8	Mlst8	56716	Target of rc	no | no chan	0	ENOG410X
D3YX10	D3YX10_M	Spsb2	Spsb2		SplA/ryanc	no | no chan	0	ENOG410X
Q9DCJ5	NDUA8_M	Ndufa8	Ndufa8	68375	NADH deh	no | no chan	0	ENOG4111

Q61706	Q61706_MOUSE	RNA-directed DNA polymer			RNA-direct	no | no chan	0	ENOG410Z
Q61704	ITIH3_MO	Itih3	Itih3	16426	Inter-alpha	no | up	0	COG2304(F
Q9DCJ9	NPL_MOU	Npl	Npl	74091	N-acetylne	no | down	0	COG0329(C
Q3UIL5	Q3UIL5_M	Maged1	Maged1	94275	MAGE don	no | no chan	0	ENOG4111
L7N463	L7N463_M	Cyp2d34	Cyp2d34	223706	Cytochrom	no | up	0	COG2124(e
L7N466	L7N466_M	Mthfsl	Mthfsl	100039707	5-formyltet	no | up	0	COG0212(S
Q9CQF8	RT63_MOU	Mrpl57	Mrpl57	67840	Large ribos	no | no chan	0	ENOG410X
Q8CH77	NAV1_MO	Nav1	Nav1	215690	Neuron nav	no | no chan	0	ENOG410Z
Q8BHK9	ERC6L_MO	Ercc6l	Ercc6l	236930	DNA excisi	no | no chan	0	ENOG410X
Q9CQF3	CPSF5_MC	Nudt21	Nudt21	68219	Cleavage ar	no | no chan	0	ENOG410X
Q9CQF0	RM11_MO	Mrpl11	Mrpl11	66419	Large ribos	no | no chan	0	COG0080(T
Q6QLQ4	CLC7A_MO	Clec7a	Clec7a	56644	C-type lecti	no | up	0	ENOG410X
Q62470	ITA3_MOU	Itga3	Itga3	16400	Integrin alp	no | no chan	0	ENOG410Z
Q8BHK3	S36A2_MO	Slc36a2	Slc36a2	246049	Proton-cou	no | down	0	COG0814(f
Q9CQF4	MRES1_MO	Mtres1	Mtres1	67851	Mitochondr	no | no chan	0	ENOG4111
Q8BX13	UBE3D_M	Ube3d	Ube3d	70348	E3 ubiquiti	no | no chan	0	ENOG4111
Q04519	ASM_MOU	Smpd1	Smpd1	20597	Sphingomy	no | no chan	0	ENOG410Y
D3Z1C5	D3Z1C5_M	Ldb1	Ldb1		LIM domai	no | no chan	0	ENOG410Y
Q8JZL1	I17RD_MC	Il17rd	Il17rd	171463	Interleukin	no | no chan	0	ENOG411C
A0A0R4J23	A0A0R4J23	Septin10	Septin10	103080	Septin	no | no chan	0	COG5019(F
A0A0R4J0F	A0A0R4J0F	Osbpl10	Osbpl10	74486	Oxysterol-b	no | no chan	0	ENOG410X
Q91ZX6	SENP2_MO	Senp2	Senp2	75826	Sentrin-spe	no | no chan	0	COG5160(k
P62700	YPEL5_MO	Ypel5	Ypel5	383295	Protein yip	no | no chan	0	ENOG4111
A0A0R4J0F	A0A0R4J0F	Gak	Gak	231580	Cyclin G as	no | no chan	0	ENOG410Y
P62702	RS4X_MOU	Rps4x	Rps4x	20102	Small ribos	no | no chan	0	COG1471((
A0A1B0GX	A0A1B0GX	Nebl	Nebl		Nebulette	no | no chan	0	ENOG4111
D3YUG3	D3YUG3_M	Rps19	Rps19		Small ribos	no | no chan	0	COG2238(F
A0A1L1SSA	A0A1L1SSA	Tmem205	Tmem205		Transmemb	no | no chan	0	ENOG4111
B8JJN2	B8JJN2_MO	C2	C2		Complemen	no | no chan	0	COG5640(t
Q08376	ZBT14_MC	Zbtb14	Zbtb14	22666	Zinc finger	no | no chan	0	COG5048(I
B8JJN0	B8JJN0_MO	Gm20547	Gm20547		Complemen	no | no chan	0	COG5640(t
A0A0A6YW	A0A0A6YW	Rcor3	Rcor3	214742	REST corep	no | no chan	0	ENOG410X
Q8R5A0	SMYD2_M	Smyd2	Smyd2	226830	N-lysine me	no | no chan	0	COG2940(I
D3YW25	D3YW25_M	Ninj1	Ninj1	18081	Ninjurin 1	no | down	0	ENOG4111
Q8BVF6	Q8BVF6_M	Pvr	Pvr	52118	Ig-like dom	no | no chan	0	ENOG4111
Q3UNJ3	Q3UNJ3_M	Fkbp10	Fkbp10	14230	peptidylpro	no | no chan	0	COG0545(M
A0A1L1SSA	A0A1L1SSA	Cep164	Cep164	214552	Centrosom	no | no chan	0	ENOG4111
Q8R035	ICT1_MOU	Mrpl58	Mrpl58	68572	Large ribos	no | no chan	0	COG1186(T
Q3TKD1	Q3TKD1_M	Rfc3	Rfc3	69263	AAA+ ATP	no | no chan	0	COG0470(S
A8Y5G9	A8Y5G9_M	Lsm10	Lsm10		U7 snRNP-	no | no chan	0	COG1958(s
Q571G4	LIN54_MO	Lin54	Lin54	231506	Protein lin-	no | no chan	0	ENOG411C
Q9JJE4	PAQR4_M	Paqr4	Paqr4	76498	Progestin a	no | no chan	0	COG1272(c
Q8VHX6	FLNC_MO	Flnc	Flnc	68794	Filamin-C	no | no chan	0	COG5069(U

Q497V6	BAHD1_M	Bahd1	Bahd1	228536	Bromo adja	no | no chan	0	ENOG410>
Q544R5	Q544R5_M	Tardbp	Tardbp	230908	TAR DNA-	no | no chan	0	ENOG410Z
Q71FD7	FBLI1_MO	Fblim1	Fblim1	74202	Filamin-bir	no | no chan	0	ENOG410Y
A0A1L1STI	A0A1L1STI	Mtfr2	Mtfr2		Mitochondı	no | no chan	0	ENOG4112
A0A1L1STI	A0A1L1STI	Tbrg1	Tbrg1		Transformi	no | up	0	ENOG410>
Q922M7	ASHWN_MOUSE	Ashwin OS=Mu		98404	Ashwin	no | no chan	0	ENOG4111
Q544R8	Q544R8_M	Trappc4	Trappc4	60409	Trafficking	no | no chan	0	COG5122(t
O88990	ACTN3_M	Actn3	Actn3	11474	Alpha-actir	no | up	0	COG5069(s
A0A5F8MP	A0A5F8MP	Mpz	Mpz	17528	Myelin prot	no | down	0	ENOG4111
Q9D071	MMS19_M	Mms19	Mms19	72199	MMS19 nuc	no | no chan	0	ENOG410>
Q3U2C9	Q3U2C9_M	Phf12	Phf12	268448	PHD finger	yes | down	1	ENOG410>
Q9D074	MGRN1_M	Mgrn1	Mgrn1	17237	E3 ubiquiti	no | no chan	0	ENOG410>
Q8BUY8	GASP2_M	Gprasp2	Gprasp2	245607	G-protein c	no | no chan	0	ENOG410>
Q9Z1M4	KS6B2_MC	Rps6kb2	Rps6kb2	58988	Ribosomal	no | no chan	0	ENOG410>
Q3U2C5	RN149_MC	Rnf149	Rnf149	67702	E3 ubiquiti	no | up	0	ENOG4112
Q0VBA4	Q0VBA4_M	Rpl22l1	Rpl22l1	68028	Ribosomal	no | no chan	0	ENOG4111
P49312	ROA1_MO	Hnrnpa1	Hnrnpa1	15382	Heterogene	no | no chan	0	COG0724(e
Q8VH37	HDAC8_M	Hdac8	Hdac8	70315	Histone dec	no | no chan	0	COG0123(c
P97855	G3BP1_MC	G3bp1	G3bp1	27041	Ras GTPas	no | no chan	0	ENOG410Y
Q80TZ9	RERE_MO	Rere	Rere	68703	Arginine-gl	no | no chan	0	ENOG410Z
P97329	KI20A_MC	Kif20a	Kif20a	19348	Kinesin-lik	no | no chan	0	COG5059(I
O09061	PSB1_MOU	Psmb1	Psmb1	19170	Proteasome	no | no chan	0	COG0638(C
P97858	S35B1_MO	Slc35b1	Slc35b1	110172	Solute carri	no | no chan	0	COG0697(I
Q9JI13	SAS10_MO	Utp3	Utp3	65961	Something	no | no chan	0	ENOG410>
Q5SXC4	Q5SXC4_M	Vezf1	Vezf1	22344	Vascular er	no | no chan	0	COG5048(I
Q8BKP2	Q8BKP2_M	Zfp189	Zfp189	230162	Zinc finger	no | no chan	0	COG5048(-
A0A385JBX	A0A385JBX	Atg5	Atg5	11793	Autophagy	no | no chan	0	ENOG410>
A0A2I3BQ	A0A2I3BQ	Stk3	Stk3		Serine/thre	no | no chan	0	ENOG410>
Q8VDP3	MICA1_M	Mical1	Mical1	171580	[F-actin]-m	no | no chan	0	COG5069(V
B1AYL1	SCN7A_M	Scn7a	Scn7a	20272	Sodium cha	no | down	0	ENOG410Y
Q78YZ6	SCOC_MO	Scoc	Scoc	56367	Short coilec	no | up	0	ENOG4111
A0A494B9	A0A494B9	Mxi1	Mxi1		MAX interc	no | no chan	0	ENOG410>
Q6ZWY3	RS27L_MO	Rps27l	Rps27l	67941	Small ribos	no | no chan	0	COG2051(4
A0A494B9	A0A494B9	Ptar1	Ptar1	72351	Protein pre	no | no chan	0	ENOG411C
E9QAG2	E9QAG2_M	Phf6	Phf6		PHD finger	no | no chan	0	ENOG410>
D3YYC3	D3YYC3_M	Setdb1	Setdb1	84505	SET domai	no | no chan	0	COG2940(p
A0A077S2U	A0A077S2U	Lyz2	Lyz2	17105	lysozyme	no | up	0	ENOG4111
Q6ZQM8	UD17_MO	Ugt1a7	Ugt1a7	394432	UDP-glucu	no | no chan	0	COG1819(c
Q3UKV4	Q3UKV4_M	Dpf2	Dpf2	19708	Zinc finger	no | no chan	0	ENOG410Y
Q3URN2	Q3URN2_M	Alg8	Alg8	381903	Alpha-1,3-g	no | no chan	0	ENOG410>
Q542D8	Q542D8_M	Mbnl3	Mbnl3	171170	C3H1-type	no | no chan	0	ENOG410Z
Q542D9	Q542D9_M	Tfrc	Tfrc	22042	Transferrin	no | no chan	0	COG2234(p
P21995	EMB_MOU	Emb	Emb	13723	Embigin	no | no chan	0	ENOG4112

Q9CWX4	RUSD4_MO	Rpusd4	Rpusd4	71989	Pseudourid	no	no chan	0	COG0564(U
P63154	CRNL1_MO	Crnkl1	Crnkl1	66877	Crooked ne	no	no chan	0	ENOG410X
A0A2I3BQ	A0A2I3BQ	Lrch1	Lrch1	380916	Leucine-ric	no	no chan	0	COG4886(F
A0AAA9W	A0AAA9W	Celf2	Celf2		CUGBP Elc	no	no chan	0	ENOG410X
A0A1B0GR	A0A1B0GR	Rcn3	Rcn3		Reticulocal	no	no chan	0	ENOG410X
A0A1B0GR	A0A1B0GR	Mob2	Mob2	101513	MOB kinas	no	no chan	0	ENOG410X
B1ASA5	B1ASA5_M	Zfp362	Zfp362	230761	Zinc finger	yes	down	1	COG5048(S
A0A1B0GR	A0A1B0GR	Taldo1	Taldo1		Transaldolc	no	no chan	0	COG0176(T
Q8K215	LYRM4_MO	Lyrm4	Lyrm4	380840	LYR motif-	no	no chan	0	ENOG4112
Q9Z2E1	MBD2_MC	Mbd2	Mbd2	17191	Methyl-CpG	no	no chan	0	ENOG410Z
Q3TZX3	S2533_MOI	Slc25a33	Slc25a33	70556	Solute carri	no	no chan	0	ENOG410X
A0A2I3BRS	A0A2I3BRS	Klf10	Klf10		Kruppel-lik	no	no chan	0	COG5048(c
A0A1B0GR	A0A1B0GR	Irx5	Irx5		Iroquois ho	no	no chan	0	ENOG410X
Q8BVE8	NSD2_MO	Nsd2	Nsd2	107823	Histone-lys	no	no chan	0	COG2940(c
Q9CPR7	SIKE1_MO	Sike1	Sike1	66641	Suppressor	no	no chan	0	ENOG4111
Q9D8X2	CC124_MO	Ccdc124	Ccdc124	234388	Coiled-coil	no	no chan	0	ENOG410Z
A0A0X1KC	A0A0X1KC	Emc10	Emc10	69683	ER membrc	no	no chan	0	ENOG4111
A0A3Q4EC	A0A3Q4EC	Commd10	Commd10		COMM dor	no	no chan	0	ENOG4111
A0A087WN	A0A087WN	Eloc	Eloc	67923	Elongin-C	no	no chan	0	ENOG4112
O70472	TM131_MC	Tmem131	Tmem131	56030	Transmemb	no	no chan	0	ENOG410Z
O70475	UGDH_MO	Ugdh	Ugdh	22235	UDP-gluco	no	up	0	COG1004(U
O70479	BACD2_MO	Tnfaip1	Tnfaip1	21927	BTB/POZ c	no	no chan	0	ENOG410X
B1AY10	NFX1_MO	Nfx1	Nfx1	74164	Transcripti	no	no chan	0	ENOG410X
F6TB64	F6TB64_MO	Ubn2	Ubn2		Ubinuclein	no	no chan	0	ENOG411C
Q3UP87	ELNE_MO	Elane	Elane	50701	Neutrophil	no	up	0	COG5640(s
B2RSG2	B2RSG2_M	Nthl1	Nthl1	18207	Endonuclec	no	no chan	0	COG0177(e
Q8BFP9	PDK1_MO	Pdk1	Pdk1	228026	[Pyruvate d	no	no chan	0	COG0642(M
Q62130	PTN14_MC	Ptpn14	Ptpn14	19250	Tyrosine-pr	no	no chan	0	COG5599(p
P55772	ENTP1_MO	Entpd1	Entpd1	12495	Ectonucleo	no	no chan	0	COG5371(c
A0A1L7NR	A0A1L7NR	Sorbs1	Sorbs1	20411	C-Cbl assoc	no	no chan	0	ENOG4111
Q8C4Q6	AIDA_MO	Aida	Aida	108909	Axin intera	no	no chan	0	ENOG410Y
Q9D1L9	LTOR5_MO	Lamtor5	Lamtor5	68576	Ragulator c	no	no chan	0	ENOG4111
P40201	CHD1_MO	Chd1	Chd1	12648	Chromodor	no	no chan	0	COG0553(T
A0AA74KT	A0AA74KT	Ado	Ado		2-aminoeth	no	no chan	0	ENOG4111
Q8C9S4	CC186_MO	Ccdc186	Ccdc186	213993	Coiled-coil	no	no chan	0	ENOG411C
Q3UQD5	Q3UQD5_I	Ncoa6	Ncoa6	56406	Nuclear rec	no	no chan	0	ENOG410X
D3YZC9	D3YZC9_M	Sf1	Sf1		Splicing fac	no	no chan	0	COG5176(r
O35551	RABE1_MO	Rabep1	Rabep1	54189	Rab GTPas	no	no chan	0	ENOG410X
D1FNM8	D1FNM8_I	Atp2b4	Atp2b4	381290	Calcium-trc	no	no chan	0	ENOG410X
Q8R4N0	CLYBL_MO	Clybl	Clybl	69634	Citramalyl-	no	no chan	0	COG2301(c
E9Q8V6	E9Q8V6_M	Dennd4a	Dennd4a	102442	DENN don	no	down	0	ENOG410Y
P30561	AHR_MOU	Ahr	Ahr	11622	Aryl hydroc	no	no chan	0	ENOG410X
Q4FZF3	DDX49_MO	Ddx49	Ddx49	234374	Probable A	no	no chan	0	ENOG410X

Q9R1Q8	TAGL3_M(	Tagln3	Tagln3	56370	Transgelin-	no|up	0	COG5199(I
Q8C9E8	CAHM6_M	Calhm6	Calhm6	215900	Calcium ho	no|up	0	ENOG4111
Q4VBX4	Q4VBX4_M	Ube2v2	Ube2v2	70620	Ubiquitin-c	no|no chan	0	ENOG4111
Q62210	BIRC2_MC	Birc2	Birc2	11797	Baculoviral	no|no chan	0	ENOG410Y
Q60930	VDAC2_M	Vdac2	Vdac2	22334	Non-selecti	no|no chan	0	ENOG410Z
Q60936	COQ8A_M	Coq8a	Coq8a	67426	Atypical ki	no|up	0	COG0661(I
Q9D9Z5	DDA1_MC	Dda1	Dda1	66498	DET1- and	no|up	0	ENOG4112
Q149F3	ERF3B_M(	Gspt2	Gspt2	14853	Eukaryotic	no|no chan	0	COG5256(I
E9PWH2	E9PWH2_M	Card6	Card6	239319	Caspase rec	no|down	0	ENOG410X
Q149F1	RUSD2_M(	Rpusd2	Rpusd2	271842	Pseudourid	no|no chan	0	COG0564(p
Q03734	SPA3M_M(	Serpina3m	Serpina3m	20717	Serine prot	no|up	0	COG4826(s
B1B0E8	B1B0E8_M	Zrsr2	Zrsr2	22184	Zinc finger	no|no chan	0	ENOG410Z
Q80TE0	RPAP1_M(	Rpap1	Rpap1	68925	RNA polym	no|no chan	0	ENOG410X
A0A0A0M(	A0A0A0M(	Actr5	Actr5	109275	Actin-relat	no|no chan	0	ENOG410X
A0A0A0M(	A0A0A0M(	Sos2	Sos2	20663	SOS Ras/R	no|no chan	0	ENOG410X
A0A0A0M(	A0A0A0M(	Map3k6	Map3k6	53608	mitogen-ac	no|no chan	0	ENOG410X
A0A0A0M(	A0A0A0M(	Afg2a	Afg2a	57815	vesicle-fusi	no|no chan	0	COG0464(I
Q8BKJ2	Q8BKJ2_M	Rbms3	Rbms3	207181	RRM doma	no|up	0	ENOG410X
Q99PB4	Q99PB4_M	Maged2	Maged2	80884	Mage-d2	no|no chan	0	ENOG4111
Q545Q2	Q545Q2_M	Surf4	Surf4	20932	Surfeit locu	no|no chan	0	COG2259(c
Q00422	GABPA_M	Gabpa	Gabpa	14390	GA-binding	no|no chan	0	ENOG410Z
Q00420	GABP1_M(	Gabpb1	Gabpb1	14391	GA-binding	no|no chan	0	COG0666(p
A0A2I3BRI	A0A2I3BRI	Hnrnpc	Hnrnpc		Heterogene	no|up	0	COG0724(c
Q9JHP7	PLGT2_M(	Poglut2	Poglut2	72050	Protein O-g	no|no chan	0	ENOG410X
P70324	TBX3_MOI	Tbx3	Tbx3	21386	T-box trans	no|no chan	0	ENOG410X
Q3UYI5	RGL3_MO	Rgl3	Rgl3	71746	Ral guanin	no|up	0	ENOG410Z
A3KG93	A3KG93_M	Kdm1a	Kdm1a	99982	Lysine-spec	no|no chan	0	ENOG410X
Q8BG28	B3GL2_MC	B3galnt2	B3galnt2	97884	UDP-GalN	no|no chan	0	ENOG410Z
Q3TJ01	Q3TJ01_M(	Rtcb	Rtcb	28088	RNA-splici	no|no chan	0	COG1690(C
A0A0R4J2A	A0A0R4J2A	Tktl2	Tktl2	74419	transketola	no|no chan	0	COG0021(t
A0A0R4J2A	A0A0R4J2A	Ssh2	Ssh2	237860	protein-seri	no|no chan	0	COG2453(t
A0A0R4J2A	A0A0R4J2A	Ccser2	Ccser2	72972	Coiled-coil	no|up	0	ENOG410X
B7ZMW4	B7ZMW4_I	Pald1	Pald1	27355	Paladin	no|no chan	0	ENOG410Y
B7ZMW6	B7ZMW6_I	Cd163	Cd163	93671	Cd163 prot	no|down	0	ENOG410X
Q5NCK8	Q5NCK8_I	Mapk9	Mapk9	26420	Stress-activ	no|no chan	0	ENOG410X
Q3UEM7	Q3UEM7_I	Fgg	Fgg	99571	Fibrinogen	no|up	0	ENOG410Z
P47757	CAPZB_M(	Capzb	Capzb	12345	F-actin-cap	no|up	0	ENOG410X
Q60778	IKBB_MOI	Nfkbib	Nfkbib	18036	NF-kappa-l	no|no chan	0	COG0666(p
B9EJR8	DAAF5_M(	Dnaaf5	Dnaaf5	433956	Dynein axo	no|no chan	0	ENOG410X
Q9CZU4	ERAL1_M(	Eral1	Eral1	57837	GTPase Era	no|no chan	0	COG1159(A
Q9CZU6	CISY_MOU	Cs	Cs	12974	Citrate synt	no|no chan	0	COG0372(c
Q1MX40	Q1MX40_M	Prkcd	Prkcd	18753	Protein kin	no|no chan	0	ENOG410X
Q80Y44	DDX10_M(	Ddx10	Ddx10	77591	Probable A	no|no chan	0	COG0513(M

Q8CHP8	PGP_MOU	Pgp	Pgp	67078	Glycerol-3-	no | no chan	0	COG0647(r
A0A494BA	A0A494BA	Ccdc40	Ccdc40	207607	Coiled-coil	no | no chan	0	ENOG410
A0A0G2JF1	A0A0G2JF1	Ccdc12	Ccdc12		Coiled-coil	no | down	0	ENOG4111
A2AC19	A2AC19_M	Suv39h1	Suv39h1	20937	Histone-lys	no | down	0	COG2940(r
P52927	HMGA2_M	Hmga2	Hmga2	15364	High mobil	no | up	0	ENOG410
A2AC16	A2AC16_M	Dcxr	Dcxr	67880	Dicarbonyl	no | no chan	0	ENOG410
Q8CHP5	PYM1_MO	Pym1	Pym1	78428	Partner of	no | no chan	0	ENOG4111
Q3TEA8	HP1B3_MC	Hp1bp3	Hp1bp3	15441	Heterochro	no | no chan	0	ENOG4112
Q91VC3	IF4A3_MO	Eif4a3	Eif4a3	192170; 434	Eukaryotic	no | no chan	0	COG0513(I
Q91VC7	PP14A_MC	Ppp1r14a	Ppp1r14a	68458	Protein pho	no | no chan	0	ENOG4111
Q91VC9	GHITM_M	Ghitm	Ghitm	66092	Growth hor	no | no chan	0	ENOG410
Q3UJQ1	Q3UJQ1_M	Kat5	Kat5	81601	Histone ace	no | no chan	0	COG5027(k
A2AQU8	A2AQU8_M	Srxn1	Srxn1	76650	sulfiredoxir	no | up	0	COG5119(s
A0A0N4SV	A0A0N4SV	Eif4g3	Eif4g3		Eukaryotic	no | no chan	0	ENOG410
O08734	BAK_MOU	Bak1	Bak1	12018	Bcl-2 homo	no | no chan	0	ENOG4112
O08736	CASPC_MC	Casp12	Casp12	12364	Caspase-12	no | up	0	ENOG410Z
O08738	CASP6_MC	Casp6	Casp6	12368	Caspase-6	no | no chan	0	ENOG410Z
Q543K2	Q543K2_M	Ccs	Ccs	12460	Superoxide	no | no chan	0	COG2032(I
P32848	PRVA_MO	Pvalb	Pvalb	19293	Parvalbumi	no | down	0	COG5126(v
D3Z3J6	D3Z3J6_M	Paip1	Paip1	218693	Polyadenyl	no | no chan	0	ENOG411C
P28028	BRAF_MO	Braf	Braf	109880	Serine/thre	no | no chan	0	ENOG410Y
Q5PT53	NTCP7_M	Slc10a7	Slc10a7	76775	Sodium/bil	no | no chan	0	COG0385(I
Q61112	CAB45_MC	Sdf4	Sdf4	20318	45 kDa calc	no | no chan	0	ENOG410
Q505D7	OPA3_MO	Opa3	Opa3	403187	Optic atrop	yes | up	1	ENOG4111
A0A0J9YU	A0A0J9YU	Dusp11	Dusp11		Dual specif	no | no chan	0	COG5226(M
P46660	AINX_MO	Ina	Ina	226180	Alpha-inter	no | no chan	0	ENOG410
P46662	MERL_MC	Nf2	Nf2	18016	Merlin	no | up	0	ENOG410
Q6A058	ARMX2_M	Armcx2	Armcx2	67416	Armadillo r	no | down	0	ENOG4111
Q3V340	Q3V340_M	Adap2	Adap2	216991	Arf-GAP w	no | up	0	COG5347(p
A2AHJ4	BRWD3_M	Brwd3	Brwd3	382236	Bromodom	no | no chan	0	COG2319(p
P27046	MA2A1_M	Man2a1	Man2a1	17158	Alpha-man	no | no chan	0	ENOG410
P27048	RSMB_MO	Snrpb	Snrpb	20638	Small nucle	no | no chan	0	COG1958(M
E9QJT2	E9QJT2_M	Naf1	Naf1	234344	H/ACA rib	no | no chan	0	ENOG4111
E9QJT5	E9QJT5_M	Acyp1	Acyp1		Acylphosph	no | no chan	0	ENOG4112
P06330	HVM51_MOUSE	Ig heavy chain V region AC3			Ig heavy ch	no | up	0	ENOG4111
E9QJT6	E9QJT6_M	Tent4b	Tent4b	214627	Terminal n	no | no chan	0	COG5260(r
P06339	HA15_MO	H2-T23	H2-T23	15040	H-2 class I	no | up	0	ENOG4111
P68372	TBB4B_MC	Tubb4b	Tubb4b	227613	Tubulin be	no | no chan	0	COG5023(g
Q3TXS7	PSMD1_M	Psmd1	Psmd1	70247	26S proteas	no | no chan	0	COG5116(2
Q4KL82	Q4KL82_M	Rfc2	Rfc2	19718	Replication	no | no chan	0	COG0470(I
E9Q0A3	E9Q0A3_M	Arhgef11	Arhgef11		Rho guanin	no | no chan	0	COG5422(c
E9Q0A4	E9Q0A4_M	Kif21b	Kif21b		Kinesin fan	no | no chan	0	COG5059(z
E9Q0A7	E9Q0A7_M	Oxr1	Oxr1	170719	Oxidation r	no | no chan	0	ENOG4111

E9QK04	E9QK04_M	Neo1	Neo1	18007	Neogenin	no | no chan	0	ENOG410Z
P61965	WDR5_MC	Wdr5	Wdr5	140858	WD repeat-	no | no chan	0	ENOG410X
P61967	AP1S1_MO	Ap1s1	Ap1s1	11769	AP-1 compl	no | no chan	0	COG5030(U
P61961	UFM1_MO	Ufm1	Ufm1	67890	Ubiquitin-f	no | no chan	0	ENOG4111
P61963	DCAF7_M(	Dcaf7	Dcaf7	71833	DDB1- and	no | no chan	0	ENOG410X
Q62392	PHLA1_M(	Phlda1	Phlda1	21664	Pleckstrin h	no | down	0	ENOG4111
Q9QZU9	UB2L6_MC	Ube2l6	Ube2l6	56791	Ubiquitin/I	no | up	0	COG5078(c
Q8BUY5	TIDC1_MC	Timmdc1	Timmdc1	76916	Complex I c	no | no chan	0	ENOG4111
Q99ME9	GTPB4_M(	Gtpbp4	Gtpbp4	69237	GTP-bindir	no | no chan	0	COG1084(I
A0A140LIU	A0A140LIU	Cox7a1	Cox7a1	12865	Cytochrome	no | up	0	ENOG410Z
Q9DD18	DTD1_MO	Dtd1	Dtd1	66044	D-aminoac	no | no chan	0	COG1490(F
A0A571BEI	A0A571BEI	Mybpc1	Mybpc1		Myosin bin	no | up	0	ENOG411C
A0A1R3UD	A0A1R3UD	Klk1b11	Klk1b11	16613	Kallikrein c	no | up	0	COG5640(p
Q9WTK7	STK11_MC	Stk11	Stk11	20869	Serine/thre	no | no chan	0	COG0515(C
Q64429	CP1B1_MC	Cyp1b1	Cyp1b1	13078	Cytochrome	no | no chan	0	COG2124(S
Q8K212	PACS1_MC	Pacs1	Pacs1	107975	Phosphofur	no | no chan	0	ENOG410X
A0A087WQ	A0A087WQ	Hdac4	Hdac4		Histone dec	no | no chan	0	COG0123(k
Q8K1E0	STX5_MOU	Stx5	Stx5	56389	Syntaxin-5	no | no chan	0	ENOG410Y
Q8CJ26	NRADD_M	Nradd	Nradd	67169	Death dom	no | no chan	0	ENOG4111
Q8CJ27	ASPM_MO	Aspm	Aspm	12316	Abnormal s	no | no chan	0	ENOG410X
E9PV44	E9PV44_M	Atp5if1	Atp5if1		ATPase inh	no | no chan	0	ENOG4112
A0A0G2JD	A0A0G2JD	Rps27	Rps27		40S ribosom	no | no chan	0	COG2051(F
A0A0G2JD	A0A0G2JD	Myl3	Myl3		Myosin ligh	no | up	0	COG5126(F
A0A1S6GW	A0A1S6GW	Man2c1	Man2c1	73744	alpha-mani	no | no chan	0	COG0383(c
D3Z512	D3Z512_M	Dnase2a	Dnase2a		Deoxyribon	no | no chan	0	ENOG4111
A0A1S6GW	A0A1S6GW	Dnaja4	Dnaja4	58233	DnaJ homo	no | up	0	COG0484(3
A0A1S6GW	A0A1S6GW	Atp6v1b2	Atp6v1b2	11966	Vacuolar p	no | no chan	0	COG1156(3
A0A1S6GW	A0A1S6GW	Mrps9	Mrps9	69527	28S ribosom	no | no chan	0	COG0103(3
A0A0R4J06	A0A0R4J06	Scly	Scly	50880	Selenocyste	no | no chan	0	COG1104(M
Q3UVW5	Q3UVW5_I	Slc6a3	Slc6a3	13162	Transporter	no | no chan	0	COG0733(T
A0A0R4J06	A0A0R4J06	Atg4b	Atg4b	66615	Cysteine pr	no | no chan	0	ENOG410X
Q3U5F4	YRDC_MC	Yrdc	Yrdc	230734	Threonylca	no | no chan	0	COG0009(s
A0A0R4J06	A0A0R4J06	Hps1	Hps1	192236	HPS1, biog	no | no chan	0	ENOG410Y
P97464	EXT1_MO	Ext1	Ext1	14042	Exostosin-1	no | no chan	0	ENOG410X
P97465	DOK1_MC	Dok1	Dok1	13448	Docking pr	no | no chan	0	ENOG410X
Q9WUU9	GANP_MC	Mcm3ap	Mcm3ap	54387	Germinal-c	no | no chan	0	COG5079(F
Q3UDV5	Q3UDV5_I	Plscr1	Plscr1	22038	Phospholip	no | up	0	ENOG410X
Q3UL30	Q3UL30_M	Cmtm6	Cmtm6	67213	MARVEL d	no | no chan	0	ENOG4111
Q3UL31	Q3UL31_M	Zc3h11a	Zc3h11a	70579	Zinc finger	no | no chan	0	ENOG411C
B2RWV6	B2RWV6_M	Styk1	Styk1	243659	Serine/thre	no | no chan	0	COG0515(p
A0A0R4IZ	A0A0R4IZ	Fnbp4	Fnbp4	55935	Formin bin	no | no chan	0	ENOG4111
E9Q9I2	E9Q9I2_M	Dlg5	Dlg5	71228	Discs large	no | no chan	0	ENOG410X
Q9JJX7	TYDP2_M(	Tdp2	Tdp2	56196	Tyrosyl-DN	no | no chan	0	ENOG410X

O88839	ADA15_MO	Adam15	Adam15	11490	Disintegrin	no	no chan	0	ENOG410
D6RGN2	D6RGN2_I	Plcd4	Plcd4		Phosphoinc	no	no chan	0	ENOG410
Q9D198	SYF2_MOU	Syf2	Syf2	68592	Pre-mRNA-	no	no chan	0	ENOG410
E9LV85	E9LV85_M	Sun1	Sun1	77053	Unc84-like	no	up	0	ENOG410
Q2TBE6	P4K2A_MO	Pi4k2a	Pi4k2a	84095	Phosphatid	no	no chan	0	ENOG410
B2RX06	B2RX06_M	Slit3	Slit3	20564	Slit homolo	no	no chan	0	COG4886(
I7DM66	I7DM66_M	Dhrs7b	Dhrs7b	216820	Dehydroger	no	no chan	0	COG1028(
B9EJ70	B9EJ70_MO	Dock8	Dock8	76088	Dedicator c	no	no chan	0	ENOG410
A6H630	ARMT1_M	Armt1	Armt1	73419	Damage-co	no	no chan	0	ENOG410
O70591	PFD2_MOU	Pfdn2	Pfdn2	18637	Prefoldin su	no	no chan	0	ENOG4111
B2RX08	B2RX08_M	Sptb	Sptb	20741	Spectrin be	no	no chan	0	COG5069(
Q9JL90	Q9JL90_MOUSE Nebulin (Fragment) OS=Mu		Nebulin			no	no chan	0	ENOG410
Q80UP8	S20A2_MO	Slc20a2	Slc20a2	20516	Sodium-dep	yes	down	1	COG0306(
A0A0A0MC	A0A0A0MC	Ano1	Ano1	101772	Anoctamin	no	no chan	0	ENOG410
Q6DFW4	NOP58_MO	Nop58	Nop58	55989	Nucleolar p	no	no chan	0	COG1498(
A0A0A0MC	A0A0A0MC	Gapdh	Gapdh	14433	Glyceraldel	no	no chan	0	COG0057(
B1ATI9	B1ATI9_MO	Gas7	Gas7	14457	Growth arr	no	no chan	0	ENOG410
Q8C2L6	T161B_MO	Tmem161b	Tmem161b	72745	Transmemb	no	no chan	0	ENOG410
Q8BJR1	Q8BJR1_M	Etv5	Etv5	104156	Ets variant	no	no chan	0	ENOG410
A0A0R3P9	A0A0R3P9	Ndufa9	Ndufa9		NADH deh	no	no chan	0	COG0702(
F6ZBR8	F6ZBR8_M	Wiz	Wiz		Widely-inte	no	no chan	0	
A0A0R3P9	A0A0R3P9	Uqcc5	Uqcc5	66487	Ubiquinol-c	no	no chan	0	ENOG411
Q5FWI3	CEIP2_MO	Cemip2	Cemip2	83921	Cell surface	no	no chan	0	ENOG410
Q3TLU9	Q3TLU9_M	Cenpi	Cenpi	102920	Centromere	no	up	0	ENOG410
Q99JP4	CDC26_MO	Cdc26	Cdc26	668450; 664	Anaphase-p	no	no chan	0	ENOG4111
Q99JP7	GGT7_MO	Ggt7	Ggt7	207182	Glutathion	no	no chan	0	COG0405(
Q99JP6	HOME3_M	Homer3	Homer3	26558	Homer prot	no	no chan	0	ENOG411
Q3TI02	Q3TI02_MO	Adipor1	Adipor1	72674	Adiponecti	no	no chan	0	COG1272(
P50247	SAHH_MO	Ahcy	Ahcy	269378; 116	Adenosylho	no	no chan	0	COG0499(
Q8BXK4	SAMTR_M	Samtor	Samtor	101148	S-adenosylr	no	no chan	0	ENOG410
O35969	GAMT_MO	Gamt	Gamt	14431	Guanidinoa	no	no chan	0	ENOG410
A2AQH4	BCORL_M	Bcorl1	Bcorl1	320376	BCL-6 core	no	no chan	0	ENOG411
Q9CXV1	DHSD_MO	Sdhd	Sdhd	66925	Succinate d	no	up	0	ENOG4111
Q9JKZ2	SC5A3_MO	Slc5a3	Slc5a3	53881	Sodium/my	no	no chan	0	COG4146(
O35963	RB33B_MO	Rab33b	Rab33b	19338	Ras-related	no	no chan	0	ENOG410
Q9CXV9	DCNL5_M	Dcun1d5	Dcun1d5	76863	DCN1-like	no	no chan	0	ENOG410
Q9QXS1	PLEC_MO	Plec	Plec	18810	Plectin	no	no chan	0	COG5069(
Q9R1Z7	PTPS_MOU	Pts	Pts	19286	6-pyruvoyl	no	no chan	0	COG0720(
Q3UTC0	Q3UTC0_M	Pramel17	Pramel17	545662	PRAME lik	no	up	0	ENOG410
Q9CWY3	SETD6_MO	Setd6	Setd6	66083	N-lysine me	no	no chan	0	ENOG410
P23780	BGAL_MO	Glb1	Glb1	12091	Beta-galact	no	no chan	0	COG1874(
Q8CCG5	Q8CCG5_M	Ralb	Ralb	64143	Ras-related	no	no chan	0	COG1100(
E9PYD1	E9PYD1_M	Fam98c	Fam98c	73833	Family with	no	no chan	0	ENOG4111

B9EJ77	B9EJ77_M(	Tanc1	Tanc1	66860	Tanc1 prot(	no | no chan	0	COG0666(I
A2AH75	A2AH75_M	Kif1b	Kif1b	16561	Kinesin fan	no | no chan	0	COG5059(r
Q9Z0L0	TPBG_MO	Tpbg	Tpbg	21983	Trophoblas	no | no chan	0	COG4886(C
Q8BTF5	Q8BTF5_M	Lig4	Lig4	319583	DNA ligase	no | no chan	0	COG1793(I
F2WWK5	F2WWK5_I	Fads2	Fads2	56473	Acyl-CoA 6	no | down	0	ENOG410X
D7F2B6	D7F2B6_M	Raet1e	Raet1e	379043	Retinoic ac	no | no chan	0	ENOG4111
Q99LZ3	SLD5_MOU	Gins4	Gins4	109145	DNA replic	no | no chan	0	COG5086(C
Q99N96	RM01_MO	Mrpl1	Mrpl1	94061	Large ribos	no | no chan	0	COG0081(I
Q8BV57	SRCRL_M(	Ssc5d	Ssc5d	269855	Soluble sca	no | no chan	0	ENOG410X
Q3U297	Q3U297_M	Zer1	Zer1	227693	Protein zer-	no | no chan	0	ENOG410X
Q99N92	RM27_MO	Mrpl27	Mrpl27	622268; 940	Large ribos	no | no chan	0	COG0211(S
Q99N93	RM16_MO	Mrpl16	Mrpl16	94063	Large ribos	no | no chan	0	COG0197(r
E9QPU1	E9QPU1_M	Vwf	Vwf	22371	von Willebr	no | up	0	ENOG411C
Q9JL95	PRG3_MO	Prg3	Prg3	53856	Proteoglyca	no | down	0	ENOG410Y
A0A3B2W3	A0A3B2W3	Zfp523	Zfp523		Zinc finger	no | no chan	0	COG5048(i
P70158	ASM3A_M(	Smpdl3a	Smpdl3a	57319	Cyclic GMI	no | no chan	0	ENOG410X
Q8C845	Q8C845_M	Efhd2	Efhd2	27984	EF hand dc	no | no chan	0	ENOG4111
Q06138	CAB39_MC	Cab39	Cab39	12283	Calcium-bi	no | no chan	0	ENOG410X
A0A811BIT	A0A811BIT	Cldnd1	Cldnd1	224250	Claudin doi	no | no chan	0	ENOG411C
Q80T69	RSBN1_M(	Rsbn1	Rsbn1	229675	Lysine-spec	no | no chan	0	ENOG410X
P29037	TBP_MOU	Tbp	Tbp	21374	TATA-box-	no | no chan	0	COG2101(C
O55126	NIPS2_MO	Nipsnap2	Nipsnap2	14467	Protein Nip	no | up	0	ENOG410X
Q9D3W4	GPN3_MO	Gpn3	Gpn3	68080	GPN-loop (	no | no chan	0	ENOG410X
O55125	NIPS1_MO	Nipsnap1	Nipsnap1	18082	Protein Nip	no | no chan	0	ENOG410X
E9QNH7	E9QNH7_I	Acbd5	Acbd5	74159	Acyl-CoA-b	no | no chan	0	COG4281(S
Q8R0Y8	S2542_MO	Slc25a42	Slc25a42	73095	Mitochondr	no | up	0	ENOG410Z
Q9D8Y8	ING5_MOU	Ing5	Ing5	66262	Inhibitor of	no | no chan	0	COG5034(I
Q8C6P8	ZFP57_MC	Zfp57	Zfp57	22715	Zinc finger	no | no chan	0	COG5048(N
P05132	KAPCA_M	Prkaca	Prkaca	18747	cAMP-depe	no | no chan	0	ENOG410X
Q8C0D5	EFL1_MOU	Efl1	Efl1	101592	Elongation	no | no chan	0	COG0480((
Q3USC0	Q3USC0_M	Heatr3	Heatr3	234549	HEAT repe	no | no chan	0	ENOG410Y
Q8C0D0	TRUB1_M(	Trub1	Trub1	72133	Pseudourid	no | no chan	0	COG0130((
Q8K3G7	Q8K3G7_M	Rtn4	Rtn4	68585	Reticulon	no | no chan	0	ENOG410X
A0A0A6YY	A0A0A6YY	Cyb561d2	Cyb561d2		Cytochrom	no | no chan	0	ENOG4111
Q3TP13	Q3TP13_M	Slc27a1	Slc27a1	26457	long-chain-	no | no chan	0	ENOG410X
Q9ERU9	RBP2_MOU	Ranbp2	Ranbp2	19386	E3 SUMO-p	no | no chan	0	COG0652(I
Q8C0D9	CEP68_MC	Cep68	Cep68	216543	Centrosom	no | down	0	ENOG4112
Q3TGB1	Q3TGB1_M	Stambp	Stambp	70527	MPN doma	no | no chan	0	COG1310(S
O54833	CSK22_MC	Csnk2a2	Csnk2a2	13000	Casein kina	no | no chan	0	ENOG410X
O54830	PR7A1_MC	Prl7a1	Prl7a1	19113	Prolactin-7	no | up	0	
A0A1W2P7	A0A1W2P7	Rab3ip	Rab3ip	216363	RAB3A inte	no | up	0	ENOG410X
O54834	RHG06_M(	Arhgap6	Arhgap6	11856	Rho GTPas	no | no chan	0	ENOG410Y
D3YXF5	CO7_MOU	C7	C7	109828	Complemer	no | down	0	ENOG410Y

Q0VDN7	RUND1_M	Rundc1	Rundc1	217201	RUN doma	no|no chan	0	ENOG410)
A0A0B4J1N	A0A0B4J1N	Ighv1-76	Ighv1-76	16061	Immunoglo	no|no chan	0	ENOG4111
A2AN96	A2AN96_M	Rbbp9	Rbbp9	26450	Retinoblast	no|no chan	0	ENOG4111
O88554	PARP2_MC	Parp2	Parp2	11546	Poly [ADP-	no|no chan	0	ENOG410)
Q8VCD5	MED17_M	Med17	Med17	234959	Mediator of	no|no chan	0	ENOG410)
E9QM38	E9QM38_M	Slc12a2	Slc12a2	20496	Solute carri	no|no chan	0	COG0531(f
P01592	IGJ_MOUS	Jchain	Jchain	16069	Immunoglo	no|up	0	ENOG410Y
D3Z079	D3Z079_M	Stxbp5	Stxbp5	78808	Syntaxin bi	no|no chan	0	ENOG410)
Q3U878	Q3U878_M	Dazap1	Dazap1	70248	RRM doma	no|no chan	0	ENOG410Y
Q9QZB7	ARP10_MC	Actr10	Actr10	56444	Actin-relate	no|no chan	0	COG5277(I
G3X963	G3X963_M	Atad2	Atad2	70472	ATPase fan	no|no chan	0	COG0464(s
G3X961	G3X961_M	Klhdc4	Klhdc4	234825	Kelch dom	no|no chan	0	ENOG410)
G3X960	G3X960_M	Syde1	Syde1	71709	Synapse def	no|no chan	0	ENOG410)
A0AA74KS	A0AA74KS	Hdgf	Hdgf		Heparin bi	no|no chan	0	ENOG4111
Q3UIR3	DTX3L_M	Dtx3l	Dtx3l	209200	E3 ubiquiti	no|up	0	ENOG4111
P53994	RAB2A_M	Rab2a	Rab2a	59021	Ras-related	no|no chan	0	COG1100(s
P53996	CNBP_MO	Cnbp	Cnbp	12785	CCHC-type	no|no chan	0	COG5082(s
P35831	PTN12_MC	Ptpn12	Ptpn12	19248	Tyrosine-pr	no|no chan	0	COG5599(t
E9PUH6	E9PUH6_M	Crls1	Crls1		cardiolipin	no|no chan	0	COG0558(c
P35385	HSPB7_MC	Hspb7	Hspb7	29818	Heat shock	no|up	0	ENOG410Y
Q9DCH2	POP7_MO	Pop7	Pop7	74097	Ribonuclea	no|no chan	0	ENOG4111
E9Q7K9	E9Q7K9_M	Mettl17	Mettl17		Methyltran	no|no chan	0	COG5459(3
Q9DCH6	ZFAN6_M	Zfand6	Zfand6	65098	AN1-type z	no|no chan	0	ENOG4111
P22366	MYD88_M	Myd88	Myd88	17874	Myeloid dif	no|no chan	0	ENOG410Z
A0A0G2JF	A0A0G2JF	Rufy3	Rufy3	52822	RUN and F	no|no chan	0	ENOG410)
B2RWI2	B2RWI2_M	Lactb	Lactb	80907	Lactamase,	no|no chan	0	COG1680(I
A0A286YD	A0A286YD	Vti1a	Vti1a		Vesicle trar	no|no chan	0	ENOG4111
Q3U3E2	F117B_MO	Fam117b	Fam117b	72750	Protein FAI	no|no chan	0	ENOG4111
B1AUN2	B1AUN2_M	Eif2b3	Eif2b3	108067	Translation	no|no chan	0	COG1208(f
A0A0R4J21	A0A0R4J21	Dhx34	Dhx34	71723	DExH-box	no|no chan	0	COG1643(c
A0A0R4J21	A0A0R4J21	Gpatch11	Gpatch11	53951	G patch do	no|no chan	0	ENOG411(
Q8R3N6	THOC1_M	Thoc1	Thoc1	225160	THO comp	no|no chan	0	ENOG410)
Q8R5C5	ACTY_MO	Actr1b	Actr1b	226977	Beta-centra	no|no chan	0	COG5277(/
Q8R3N1	NOP14_M	Nop14	Nop14	75416	Nucleolar p	no|no chan	0	ENOG410)
Q8C3K0	Q8C3K0_M	Lgals12	Lgals12	56072	Galectin	yes|down	1	ENOG4111
Q8JZN7	MIRO2_M	Rhot2	Rhot2	214952	Mitochondi	no|no chan	0	ENOG410)
Q9EQ32	BCAP_MO	Pik3ap1	Pik3ap1	83490	Phosphoinc	no|up	0	ENOG411(
Q8JZN5	ACAD9_M	Acad9	Acad9	229211	Complex I c	no|no chan	0	COG1960(c
D3YZ98	D3YZ98_M	Pik3c3	Pik3c3		Phosphatid	no|no chan	0	COG5032(r
B9EHM1	B9EHM1_M	Zc3hav1l	Zc3hav1l	209032	Zinc finger	no|no chan	0	ENOG4111
Q9D0L7	ARM10_M	Armc10	Armc10	67211	Armadillo r	no|no chan	0	ENOG4112
A2BI80	A2BI80_M	Ebf4	Ebf4		Early B cell	no|no chan	0	ENOG410)
O70445	BARD1_M	Bard1	Bard1	12021	BRCA1-ass	no|no chan	0	ENOG410Z

D3YUE4	F151B_MO	Fam151b	Fam151b	73942	Protein FAI	no | no chan	0	ENOG410Z
P21550	ENOB_MC	Eno3	Eno3	13808	Beta-enolas	no | no chan	0	COG0148(C
D3YW42	D3YW42_M	Ccdc9	Ccdc9		Coiled-coil	no | no chan	0	ENOG4111
Q5SWU9	ACACA_M	Acaca	Acaca	107476	Acetyl-CoA	no | no chan	0	COG4799(c
Q3TVB5	Q3TVB5_MOUSE	RNA-binding m		209003	RRM doma	no | up	0	ENOG4111
B1ATV5	B1ATV5_M	Bbs7	Bbs7	71492	Bardet-Biec	no | no chan	0	ENOG410X
Q6GQT9	NOMO1_M	Nomo1	Nomo1	211548	BOS compl	no | no chan	0	ENOG410X
P58854	GCP3_MO	Tubgcp3	Tubgcp3	259279	Gamma-tul	no | no chan	0	ENOG410X
Q3UYR3	Q3UYR3_M	Cbfb	Cbfb	12400	Core-bindir	no | no chan	0	ENOG410Z
Q3UDK1	TRAD1_M	Trafd1	Trafd1	231712	TRAF-type	no | up	0	ENOG4111
Q6GQT1	A2MG_MC	A2m	A2m	232345	Alpha-2-mc	no | up	0	ENOG410X
A0A023J65	A0A023J65	ATP8	ATP8		ATP syntha	no | no chan	0	ENOG4111
Q8BQR4	KANL2_M	Kansl2	Kansl2	69612	KAT8 regul	no | no chan	0	ENOG411C
Q6GQT6	SCAP_MO	Scap	Scap	235623	Sterol regul	no | no chan	0	ENOG410X
Q9WU62	INCE_MO	Incenp	Incenp	16319	Inner centr	no | no chan	0	ENOG410X
A0A0R4J1E	A0A0R4J1E	Hif1a	Hif1a	15251	Hypoxia-in	no | up	0	ENOG410Y
E9Q6K1	E9Q6K1_M	Ercc2	Ercc2		DNA 5'-3' h	no | no chan	0	COG1199(I
B1B1D8	B1B1D8_M	Mrpl2	Mrpl2		Mitochondi	no | no chan	0	COG0090(C
E9Q6K3	E9Q6K3_M	Pibf1	Pibf1	52023	Progesteron	no | no chan	0	ENOG410Y
P10126	EF1A1_MC	Eef1a1	Eef1a1	13627; 2355	Elongation	no | no chan	0	COG5256(T
Q571E4	GALNS_M	Galns	Galns	50917	N-acetylgal	no | no chan	0	COG3119(g
Q9CQD4	CH1B2_MC	Chmp1b2	Chmp1b2	67028	Charged mu	no | up	0	COG5491(f
A0A1L1ST(	A0A1L1ST(	Syne1	Syne1	64009	Spectrin rep	no | no chan	0	COG5069(M
Q9CQD1	RAB5A_M	Rab5a	Rab5a	271457	Ras-related	no | no chan	0	ENOG410Y
Q3U2A5	Q3U2A5_M	D030056L2	D030056L2	225995	RIKEN cD	no | no chan	0	ENOG4111
A0A0R4J0I	A0A0R4J0I	Acad11	Acad11	102632	Acyl-CoA d	no | no chan	0	COG3173(I
A0A2I3BQ(	A0A2I3BQ(	Ankrd28	Ankrd28		Ankyrin rep	no | no chan	0	COG0666(I
P41317	MBL2_MO	Mbl2	Mbl2	17195	Mannose-b	no | no chan	0	ENOG410X
Q9Z1K5	ARI1_MOU	Arih1	Arih1	23806	E3 ubiquiti	no | no chan	0	ENOG410X
A0A0R4J0I	A0A0R4J0I	Serpina3k	Serpina3k	20714	Serine (or c	no | down	0	COG4826(s
C0HK79	ARXS1_MC	Arxes1	Arxes1	76219; 7697	Adipocyte-r	no | no chan	0	ENOG411C
A0A0R4J1N	A0A0R4J1N	Dpysl4	Dpysl4	26757	Dihydropyr	no | no chan	0	COG0044(A
A0A0R4J1N	A0A0R4J1N	Apoc3	Apoc3	11814	Apolipopro	no | no chan	0	ENOG4111
A0A0R4J1N	A0A0R4J1N	Arsk	Arsk	77041	Arylsulfata	no | no chan	0	COG3119(t
A0A0R4J1N	A0A0R4J1N	Ank1	Ank1	11733	Ankyrin 1, e	no | no chan	0	COG0666(c
Q8VHC3	SELM_MO	Selenom	Selenom	114679	Selenoprote	no | no chan	0	ENOG4112
A0A0R4J1N	A0A0R4J1N	Tfam	Tfam	21780	Transcripti	no | no chan	0	COG5648(c
A0A0R4J1N	A0A0R4J1N	Rdh13	Rdh13	108841	Retinol deh	no | no chan	0	COG1028(-
Q9D051	ODPB_MC	Pdhb	Pdhb	68263	Pyruvate de	no | no chan	0	COG0022(r
I3RSH5	I3RSH5_M	Wwp2	Wwp2	66894	E3 ubiquiti	no | no chan	0	COG5021(c
A0A384DV	A0A384DV	Stfa1	Stfa1		Stefin A1	no | up	0	ENOG4112
A0A384DV	A0A384DV	Pde10a	Pde10a	23984	Phosphodie	no | no chan	0	ENOG410X
Q3UE37	UBE2Z_M	Ube2z	Ube2z	268470	Ubiquitin-c	no | no chan	0	COG5078(Y

Q60597	ODO1_MC	Ogdh	Ogdh	18293	2-oxoglutar	no	no chan	0	COG0567(2
A0A338P6S	A0A338P6S	Abi3bp	Abi3bp	320712	ABI family	no	down	0	ENOG410Y
A0A1Y7VN	A0A1Y7VN	Dhrs7	Dhrs7		Dehydroger	no	no chan	0	COG0300(Y
P97872	FMO5_MC	Fmo5	Fmo5	14263	Flavin-cont	no	no chan	0	COG2072(F
Q14AQ2	Q14AQ2_M	Rilp	Rilp	280408	Rab interac	yes	up	1	ENOG4111
A0A494B9H	A0A494B9H	Rhod	Rhod		Ras homolo	no	no chan	0	COG1100(N
A0A494B9H	A0A494B9H	Sil1	Sil1		Nucleotide	no	no chan	0	ENOG410Y
P20801	TNNC2_M	Tnnc2	Tnnc2	21925	Troponin C	no	up	0	COG5126(t
Q14AQ4	Q14AQ4_M	Samd8	Samd8	67630	Sterile alph	no	no chan	0	ENOG410X
Q3UKP5	Q3UKP5_M	Xab2	Xab2	67439	Pre-mRNA-	no	no chan	0	ENOG410X
F8WGM5	F8WGM5_	Stxbp2	Stxbp2		Syntaxin-bi	no	no chan	0	COG5158(S
Q9JK92	HSPB8_MC	Hspb8	Hspb8	80888	Heat shock	no	up	0	ENOG410Y
Q3UY35	Q3UY35_M	Chst1	Chst1	76969	Sulfotransf	no	up	0	ENOG411C
Q9D0T1	NH2L1_M	Snu13	Snu13	20826	NHP2-like	no	no chan	0	COG1358((
A6H5Y3	METH_MC	Mtr	Mtr	238505	Methionine	no	no chan	0	COG1410(r
Q9CPN8	IF2B3_MO	Igf2bp3	Igf2bp3	140488	Insulin-like	no	no chan	0	ENOG410Z
B2RT97	B2RT97_M	Psmd13	Psmd13	23997	26S proteas	no	no chan	0	ENOG410X
P14733	LMNB1_M	Lmnb1	Lmnb1	16906	Lamin-B1	no	no chan	0	ENOG410Y
Q9CX53	GEMI6_M	Gemin6	Gemin6	67242	Gem-associ	no	no chan	0	ENOG4111
Q8C6M1	UBP20_MC	Usp20	Usp20	74270	Ubiquitin c	no	no chan	0	COG5560(N
P61406	EST1A_MC	Smg6	Smg6	103677	Telomerase	no	no chan	0	ENOG410X
F8WHL2	F8WHL2_M	Copa	Copa		Coatomer s	no	no chan	0	ENOG410X
A0A087WN	A0A087WN	Agfg1	Agfg1	15463	ArfGAP wi	no	no chan	0	COG5347(F
Q8BVG4	DPP9_MO	Dpp9	Dpp9	224897	Dipeptidyl	no	no chan	0	COG1506(c
Q0PD62	Q0PD62_M	Rab3b	Rab3b	69908	Ras-related	no	no chan	0	ENOG410Z
A2AR02	PPIG_MOU	Ppig	Ppig	228005	Peptidyl-pr	no	no chan	0	COG0652(F
A0A1L1SR1	A0A1L1SR1	Ift46	Ift46		Intraflagell	yes	down	1	ENOG410Z
E9PVH4	E9PVH4_M	Plk4	Plk4		Serine/thre	no	down	0	ENOG410X
A2A432	CUL4B_M	Cul4b	Cul4b	72584	Cullin-4B	no	no chan	0	COG5647(r
Q3UHQ6	DOP1B_M	Dop1b	Dop1b	70028	Protein DO	no	no chan	0	COG5221(I
O70456	1433S_MOU	Sfn	Sfn	55948	14-3-3 prot	no	no chan	0	COG5040(T
Q8BL80	RHG22_M	Arhgap22	Arhgap22	239027	Rho GTPas	no	no chan	0	ENOG410X
Q8BL86	MBLC2_M	Mblac2	Mblac2	72852	Acyl-coenzy	no	no chan	0	COG0491(Z
B2RSE6	B2RSE6_M	Tfb2m	Tfb2m	15278	rRNA aden	no	no chan	0	COG0030(c
A0A1B0GR	A0A1B0GR	Ptpre	Ptpre	19267	Receptor-ty	no	no chan	0	COG5599(e
Q91VM9	IPYR2_MO	Ppa2	Ppa2	74776	Inorganic p	no	no chan	0	COG0221(N
Q8C9M2	CCD15_M	Ccdc15	Ccdc15	245902	Coiled-coil	no	no chan	0	ENOG410Z
Q3TJY1	Q3TJY1_M	Mcrs1	Mcrs1	51812	Microspher	no	no chan	0	ENOG410X
Q3TR40	Q3TR40_M	Thbs1	Thbs1	21825	Thrombosp	no	up	0	ENOG410X
D3Z0Z4	D3Z0Z4_M	Abcg3	Abcg3		ATP bindin	no	no chan	0	COG1131(S
Q6PB51	CC117_MO	Ccdc117	Ccdc117	104479	Coiled-coil	no	no chan	0	ENOG410Y
Q3UFK7	Q3UFK7_M	Fam210b	Fam210b	67017	DUF1279 d	no	no chan	0	ENOG4112
E9Q8T1	E9Q8T1_M	Tacc2	Tacc2	57752	Transformi	no	no chan	0	ENOG410Y

A0A087WR	A0A087WR	Edem3	Edem3	66967	alpha-1,2-M	no | no chan	0	ENOG410
Q920Q8	NS1BP_MC	Ivns1abp	Ivns1abp	117198	Influenza v	no | no chan	0	ENOG410
Q3UFK8	FRMD8_M	Frmd8	Frmd8	67457	FERM dom	no | no chan	0	ENOG410
Q3U6Y0	Q3U6Y0_M	Stk25	Stk25	59041	non-specifi	no | no chan	0	ENOG410
P15066	JUND_MO	Jund	Jund	16478	Transcripti	no | up	0	ENOG410
P62267	RS23_MOU	Rps23	Rps23	66475	Small ribos	no | no chan	0	COG0048(
Q9JIG7	CCD22_MC	Ccdc22	Ccdc22	54638	Coiled-coil	no | no chan	0	ENOG410
Q91YU9	Q91YU9_M	Prdx5	Prdx5	54683	thioredoxin	no | up	0	COG0678(
Q91YU8	SSF1_MOU	Ppan	Ppan	235036	Suppressor	no | no chan	0	ENOG410
Q9D0R8	LSM12_MC	Lsm12	Lsm12	268490	Protein LSM	no | no chan	0	ENOG410
A0A0N4SU	A0A0N4SU	Pum3	Pum3	52874	Pumilio RN	no | no chan	0	ENOG410
Q3TCU5	Q3TCU5_M	Tapbp	Tapbp	21356	Tapasin	no | up	0	ENOG4111
Q3TCU2	Q3TCU2_M	Ppp1r13l	Ppp1r13l	333654	SH3 domai	no | no chan	0	ENOG410
Q8K0L9	ZBT20_MC	Zbtb20	Zbtb20	56490	Zinc finger	no | no chan	0	COG5048(
Q91YU3	MSD4_MO	Msantd4	Msantd4	78100	Myb/SANT	no | no chan	0	ENOG4111
G3UVV4	G3UVV4_M	Hk1	Hk1	15275	Hexokinase	no | no chan	0	COG5026(
Q9DAU9	ZN654_MC	Znf654	Znf654	72020	Zinc finger	no | no chan	0	COG5048(
E9PWN8	E9PWN8_M	Glt8d2	Glt8d2		Glycosyltra	no | no chan	0	ENOG411
A0AAG1F6	A0AAG1F6P4_MOUSE	Ubiquitin carboxyl-ter	Usp24			no | no chan	0	COG5077(
E9Q3Z5	E9Q3Z5_M	Svil	Svil		Supervillin	no | no chan	0	ENOG410
Q07417	ACADS_M	Acads	Acads	11409	Short-chain	no | no chan	0	COG1960(
A0A338P6	A0A338P6	Hmga1	Hmga1		High mobil	no | no chan	0	ENOG410
A0A0A0M	A0A0A0M	Gcdh	Gcdh	270076	Glutaryl-C	no | no chan	0	COG1960(
A2RS96	A2RS96_M	Tmed5	Tmed5	73130	Transmemb	no | no chan	0	ENOG410
Q52KE7	CCNL1_M	Ccnl1	Ccnl1	56706	Cyclin-L1	no | no chan	0	COG5333(
Q8BZK0	Q8BZK0_M	Hps3	Hps3	12807	Hermansky	no | no chan	0	ENOG410
P58681	TLR7_MOU	Tlr7	Tlr7	170743	Toll-like re	no | no chan	0	COG4886(
P12388	PAI2_MOU	Serpinb2	Serpinb2	18788	Plasminoge	no | up	0	COG4826(
P58682	TLR8_MOU	Tlr8	Tlr8	170744	Toll-like re	no | no chan	0	COG4886(
A0A2I3BR	A0A2I3BR	Eny2	Eny2		Transcripti	no | no chan	0	ENOG4111
P12382	PFKAL_M	Pfkl	Pfkl	18641	ATP-depen	no | up	0	COG0205(
Q8C180	FRS2_MOU	Frs2	Frs2	327826	Fibroblast	no | no chan	0	ENOG410
Q921M7	CYRIB_MC	Cyrib	Cyrib	223601	CYFIP-rela	no | no chan	0	ENOG410
Q80Y20	ALKB8_M	Alkbh8	Alkbh8	67667	tRNA (carb	no | no chan	0	COG3145(
Q3ULI5	Q3ULI5_M	Ppp1r10	Ppp1r10	52040	Serine/thre	no | no chan	0	ENOG410
A2ASY0	A2ASY0_M	Taf3	Taf3		TATA-box	no | no chan	0	ENOG410
D3YWF6	D3YWF6_M	Otub1	Otub1		ubiquitinyl	no | no chan	0	ENOG410
O54734	OST48_MC	Ddost	Ddost	13200	Dolichyl-di	no | no chan	0	ENOG410
A0A3B2WE	A0A3B2WE	Dennd1c	Dennd1c	70785	DENN don	no | no chan	0	ENOG410
Q9CZS1	AL1B1_MC	Aldh1b1	Aldh1b1	72535	Aldehyde d	no | no chan	0	COG1012(
O88783	FA5_MOU	F5	F5	14067	Coagulatio	no | up	0	ENOG4111
Q5SVR0	TBC9B_MC	Tbc1d9b	Tbc1d9b	76795	TBC1 domc	no | no chan	0	COG5210(
Q6QD59	SEC20_MC	Bnip1	Bnip1	224630	Vesicle trar	no | no chan	0	ENOG4111

A0A0G2JF:	A0A0G2JF:	Crip2	Crip2	68337	Cysteine-ric	no	no chan	0	ENOG410:
A0A0G2JF:	A0A0G2JF:	Klhl7	Klhl7	52323	Kelch-like '	no	up	0	ENOG410:
Q9D385	AR2BP_MC	Arl2bp	Arl2bp	107566	ADP-ribosy	no	no chan	0	ENOG4111
D3YY91	LLR1_MOU	Lrr1	Lrr1	69706	Leucine-ric	no	up	0	COG4886(p
Q5GQ64	Q5GQ64_M	Sncg	Sncg	20618	Gamma-syn	no	no chan	0	ENOG4111
Q91VE0	S27A4_MO	Slc27a4	Slc27a4	26569	Long-chain	no	no chan	0	ENOG410:
P04187	GRAB_MC	Gzmb	Gzmb	14939	Granzyme l	no	up	0	COG5640(T
P04184	KITH_MO	Tk1	Tk1	21877	Thymidine	no	down	0	COG1435(T
Q8BQX5	TMCO6_M	Tmco6	Tmco6	71983	Transmemb	no	no chan	0	ENOG4112
A2AVA0	SVEP1_MC	Svep1	Svep1	64817	Sushi, von '	no	no chan	0	ENOG410:
E9Q652	E9Q652_M	Rgs12	Rgs12	71729	Regulator c	no	no chan	0	ENOG410
P35737	DMB_MOU	H2-DMb1	H2-DMb1	14999	Class II his	no	up	0	ENOG410
Q922X9	ANM7_MC	Prmt7	Prmt7	214572	Protein arg	no	no chan	0	COG0500(M
A2ABU4	MYOM3_M	Myom3	Myom3	242702	Myomesin-:	no	up	0	ENOG410Z
O08715	AKAP1_M	Akap1	Akap1	11640	A-kinase ar	no	no chan	0	ENOG410Z
Q8VE70	PDC10_MC	Pdcd10	Pdcd10	56426	Programme	no	no chan	0	ENOG410:
P83741	WNK1_MC	Wnk1	Wnk1	232341	Serine/thre	no	no chan	0	ENOG410:
D3Z3H0	D3Z3H0_M	Ino80e	Ino80e		INO80 com	no	no chan	0	ENOG4111
Q6A037	N4BP1_MC	N4bp1	N4bp1	80750	NEDD4-bi	no	no chan	0	ENOG410Z
Q505F1	NR2C1_M	Nr2c1	Nr2c1	22025	Nuclear rec	no	no chan	0	ENOG410:
Q8BT60	CPNE3_M	Cpne3	Cpne3	70568	Copine-3	no	no chan	0	ENOG410:
Q91WS0	CISD1_MC	Cisd1	Cisd1	52637	CDGSH irc	no	no chan	0	ENOG4112
Q9QZ83	Q9QZ83_M	Actg1	Actg1	11465	Actin, cytop	no	no chan	0	COG5277(r
Q91YT8	TM63A_M	Tmem63a	Tmem63a	208795	CSC1-like p	no	no chan	0	COG5594(\
P05366	SAA1_MOU	Saa1	Saa1	20208	Serum amyl	no	up	0	ENOG410
A2RTL5	RSRC2_MC	Rsrc2	Rsrc2	208606	Arginine/se	no	no chan	0	ENOG4111
P17809	GTR1_MO	Slc2a1	Slc2a1	20525	Solute carri	no	up	0	COG0477(r
A0A0G2JE	A0A0G2JE	Ttc14	Ttc14		Tetratricop	no	no chan	0	ENOG410:
Q3TL95	Q3TL95_M	Psma4	Psma4	26441	Proteasome	no	no chan	0	COG0638(T
Q7TSF4	LR75A_MC	Lrrc75a	Lrrc75a	192976	Leucine-ric	no	up	0	ENOG410Z
E9QJV4	E9QJV4_M	Prpf39	Prpf39	328110	Pre-mRNA	no	no chan	0	ENOG410:
H3BL08	H3BL08_M	Cers6	Cers6	241447	Ceramide s	no	no chan	0	COG5058(I
Q8R1Q9	RBSK_MO	Rbks	Rbks	71336	Ribokinase	no	down	0	COG0524(p
Q3U614	Q3U614_M	Xylt2	Xylt2	217119	Xylosyltran	no	no chan	0	ENOG410:
Q8K183	PDXK_MC	Pdxk	Pdxk	216134	Pyridoxal k	no	no chan	0	COG2240(I
H3BL05	H3BL05_M	Zfp428	Zfp428		Zinc finger	no	no chan	0	ENOG4111
A2AG36	A2AG36_M	Pigo	Pigo	56703	GPI ethanc	no	no chan	0	COG1524(r
A0A1G5SJ	A0A1G5SJ	OTTMUSW	OTTMUSW	245240	Immunity r	no	no chan	0	ENOG4112
P52760	RIDA_MO	Rida	Rida	15473	2-iminobut	no	no chan	0	COG0251(I
A2AG30	A2AG30_M	Dnajb5	Dnajb5	56323	DnaJ heat s	no	up	0	COG0484(/
D3Z687	D3Z687_M	0610010K1	0610010K1	104457	RIKEN cD	no	no chan	0	ENOG4111
A0A494BBI	A0A494BBI	Fas	Fas		Tumor necr	no	down	0	ENOG4112
A0A1D5RL	A0A1D5RL	Capn15	Capn15	50817	Calpain 15	no	no chan	0	ENOG410:

Q3THK3	T2FA_MOU	Gtf2f1	Gtf2f1	98053	General tra	no|up	0	ENOG410Z
Q9ERH4	NUSAP_M	Nusap1	Nusap1	108907	Nucleolar c	no|no chan	0	ENOG4111
Q6PAL0	BEND3_M	Bend3	Bend3	331623	BEN doma	no|no chan	0	ENOG410Y
Q9CQM2	ERD22_M	Kdelr2	Kdelr2	66913	ER lumen p	no|no chan	0	COG5196(M
A2NUE7	A2NUE7_MOUSE	V(Kappa) gene		672450	V(Kappa) g	no|up	0	ENOG410Y
Q5U5M8	BL1S3_MO	Bloc1s3	Bloc1s3	232946	Biogenesis	no|no chan	0	ENOG4111
O09043	NAPSA_M	Napsa	Napsa	16541	Napsin-A	no|no chan	0	ENOG410Z
O09047	C3AR_MO	C3ar1	C3ar1	12267	C3a anaphy	no|up	0	ENOG410Y
A0JNY3	A0JNY3_M	Gphn	Gphn	268566	Gephyrin	no|no chan	0	COG0521(m
A0JNY7	A0JNY7_M	Eif4g2	Eif4g2	13690	Eukaryotic	no|no chan	0	ENOG410Z
A2CG76	A2CG76_M	Ehmt2	Ehmt2	110147	Euchromat	no|no chan	0	COG2940(C
Q3UFV9	Q3UFV9_M	Dnajc3	Dnajc3	100037258	DnaJ homo	no|no chan	0	COG0484(c
Q8K1G2	LBN_MOU	Evc2	Evc2	68525	Limbin	no|no chan	0	ENOG4111
B7ZWE5	B7ZWE5_M	Bcl10	Bcl10	12042	B-cell leuke	no|no chan	0	ENOG4111
B2RQF6	B2RQF6_M	A830018L1	A830018L1	320492	A830018L1	no|up	0	ENOG4111
G3X9G4	G3X9G4_M	Dnm2	Dnm2		dynamin G	no|no chan	0	COG0699(I
Q8CE96	TRM6_MO	Trmt6	Trmt6	66926	tRNA (ader	no|no chan	0	ENOG410Z
A0A1S6GW	A0A1S6GW	Psmf1	Psmf1	228769	Proteasome	no|no chan	0	ENOG4111
A0A1S6GW	A0A1S6GW	Tmem258	Tmem258	69038	Transmemb	no|no chan	0	ENOG4111
A0A1S6GW	A0A1S6GW	Rtca	Rtca	66368	RNA 3'-terr	no|no chan	0	COG0430(C
Q6R5N8	TLR13_MC	Tlr13	Tlr13	279572	Toll-like re	yes|up	1	COG4886(F
A0A1S6GW	A0A1S6GW	Bud23	Bud23	66138	18S rRNA (	no|no chan	0	COG0500(u
A0A1S6GW	A0A1S6GW	Exosc3	Exosc3	66362	Ribosomal	no|no chan	0	COG1097(I
A0A1S6GW	A0A1S6GW	Lrpap1	Lrpap1	16976	Alpha-2-m	no|no chan	0	ENOG410Y
E9QMH7	E9QMH7_	Ikbip	Ikbip	67454	IKBKB inte	no|no chan	0	ENOG410Y
A0A0R4J00	A0A0R4J00	Pald1	Pald1	27355	Paladin	no|down	0	ENOG410Y
A0A0R4J00	A0A0R4J00	Mfsd3	Mfsd3	69572	Major facili	no|no chan	0	COG0477(c
A0A0R4J00	A0A0R4J00	Lims1	Lims1	110829	LIM and se	no|no chan	0	ENOG410Z
A0A0R4J00	A0A0R4J00	Kctd5	Kctd5	69259	Potassium	no|no chan	0	ENOG410Z
A0A0R4J00	A0A0R4J00	Hdac2	Hdac2	15182	Histone dec	no|no chan	0	COG0123(F
A0A0R4J00	A0A0R4J00	Actr6	Actr6	67019	ARP6 actin	no|no chan	0	ENOG410Z
A2BDX3	MOCS3_M	Mocs3	Mocs3	69372	Adenylyltra	no|no chan	0	COG0607(C
Q91WC1	POTE1_M	Pot1	Pot1	101185	Protection	no|no chan	0	ENOG410Z
A2A8M8	A2A8M8_M	Pramel11	Pramel11		PRAME lik	no|up	0	
P33174	KIF4_MOU	Kif4	Kif4	16571	Chromoson	no|no chan	0	COG5059(v
A0A338P6F	A0A338P6F	Osbpl11	Osbpl11		Oxysterol-b	no|no chan	0	ENOG410Z
O88811	STAM2_M	Stam2	Stam2	56324	Signal trans	no|no chan	0	ENOG410Z
Q08509	EPS8_MOU	Eps8	Eps8	13860	Epidermal	no|no chan	0	ENOG410Z
P16045	LEG1_MO	Lgals1	Lgals1	16852	Galectin-1	no|no chan	0	ENOG4111
A0A0G2JFI	A0A0G2JFI	Kmt5a	Kmt5a	67956	[histone H4	no|down	0	COG2940(s
A0A498WC	A0A498WC	Phf21a	Phf21a	192285	PHD finger	no|no chan	0	ENOG4111
A0A494BA	A0A494BA	Pggt1b	Pggt1b		Protein ger	no|no chan	0	COG5029(I
Q9CXD6	MCUR1_M	Mcur1	Mcur1	76137	Mitochond	no|no chan	0	ENOG4111

B9EKS7	B9EKS7_M	Pgs1	Pgs1	74451	CDP-diacyl	no | no chan	0	COG1502((
Q8C405	Q8C405_M	Sp100	Sp100	20684	Nuclear an	no | up	0	ENOG4111
Q8C407	YIPF4_MO	Yipf4	Yipf4	67864	Protein YIF	no | no chan	0	ENOG4111
G3X9H5	G3X9H5_M	Htt	Htt	15194	Huntingtin	no | no chan	0	ENOG410X
G3X9H7	G3X9H7_M	Mtss1	Mtss1	211401	MTSS I-BA	no | no chan	0	ENOG410X
Q5KU40	Q5KU40_M	Mkrn1	Mkrn1	54484	RING-type	no | up	0	ENOG410X
Q3TIC8	Q3TIC8_M	Uqcrc1	Uqcrc1	22273	Cytochrome	no | no chan	0	COG0612((
P12970	RL7A_MOU	Rpl7a	Rpl7a	27176	Large ribos	no | no chan	0	COG1358(f
A0A668KL	A0A668KL	Clic5	Clic5	224796	Chloride in	no | up	0	ENOG410Z
V9GWW1	V9GWW1_	Zfp280d	Zfp280d	235469	Zinc finger	no | no chan	0	COG5048(t
Q8JZU2	TXTP_MO	Slc25a1	Slc25a1	13358	Tricarboxyl	no | no chan	0	ENOG410Y
Q8JZU0	NUD13_M	Nudt13	Nudt13	67725	NAD(P)H p	no | no chan	0	COG2816(N
Q4U2R1	HERC2_M	Herc2	Herc2	15204	E3 ubiquiti	no | no chan	0	COG5184(A
Q8JZU6	PXDC1_M	Pxdc1	Pxdc1	66895	PX domain	no | no chan	0	ENOG411C
Q8BGS0	MAK16_M	Mak16	Mak16	67920	Protein MA	no | no chan	0	COG5129(N
E9PXL0	E9PXL0_M	Fam222b	Fam222b	216971	Family with	no | no chan	0	ENOG410Y
A0A0A0MC	A0A0A0MC	Tdrd7	Tdrd7	100121	Tudor dom	no | no chan	0	ENOG410X
A0A0A0MC	A0A0A0MC	Ralgapa2	Ralgapa2	241694	Ral GTPas	no | no chan	0	ENOG410X
A0A0A0MC	A0A0A0MC	9530068E07	9530068E07Rik		RIKEN cD	no | no chan	0	ENOG410X
Q99N91	RM34_MO	Mrpl34	Mrpl34	94065	Large ribos	no | no chan	0	COG0230(S
P58058	NADK_MO	Nadk	Nadk	192185	NAD kinas	no | no chan	0	COG0061((
P58059	RT21_MOU	Mrps21	Mrps21	66292	Small ribos	no | no chan	0	ENOG4111
D3Z2K2	D3Z2K2_M	Mrps14	Mrps14	64659	Mitochondr	no | no chan	0	COG0199(F
Q9D7B7	GPX8_MO	Gpx8	Gpx8	69590	Probable gl	no | no chan	0	COG0386(N
Q99JR1	SFXN1_MO	Sfxn1	Sfxn1	14057	Sideroflexir	no | no chan	0	ENOG410Y
I7GPR1	I7GPR1_M	Traf3ip2	Traf3ip2	103213	TRAF3 inte	no | no chan	0	ENOG4111
A0A0A6YV	A0A0A6YV	Tmco1	Tmco1		Calcium lo	no | no chan	0	ENOG410X
A2AQJ8	A2AQJ8_M	Ganc	Ganc	76051	Glucosidas	no | no chan	0	COG1501(k
Q8CEW9	Q8CEW9_N	Sf3b5	Sf3b5	66125	Splicing fac	no | no chan	0	ENOG4111
Q8BJ90	ZN771_MC	Znf771	Znf771	244216	Zinc finger	no | no chan	0	COG5048(A
Q9CXP8	GBG10_M	Gng10	Gng10	14700	Guanine nu	no | down	0	ENOG4112
A2AT37	RENT2_M	Upf2	Upf2	326622	Regulator c	no | no chan	0	ENOG410X
Q14C26	Q14C26_M	Emc7	Emc7	73024	Endoplasm	no | no chan	0	ENOG4111
Q99NB8	UBQL4_M	Ubqln4	Ubqln4	94232	Ubiquilin-4	no | no chan	0	COG5272(u
A6X8Z5	RHG31_M	Arhgap31	Arhgap31	12549	Rho GTPas	no | no chan	0	ENOG410Z
Q99K28	ARFG2_M	Arfgap2	Arfgap2	77038	ADP-ribosy	no | no chan	0	COG5347(u
Q99K23	UFSP2_MC	Ufsp2	Ufsp2	192169	Ufm1-speci	no | no chan	0	ENOG410X
Q0VEJ0	CEP76_MC	Cep76	Cep76	225659	Centrosome	no | down	0	ENOG410X
P62317	SMD2_MO	Snrpd2	Snrpd2	107686	Small nucle	no | no chan	0	COG1958(S
A6H659	A6H659_M	Pag1	Pag1	94212	Pag1 protei	yes | up	1	ENOG4111
Q924W5	SMC6_MO	Smc6	Smc6	67241	Structural r	no | no chan	0	COG1196(E
P70333	HNRH2_M	Hnrnph2	Hnrnph2	56258	Heterogene	no | no chan	0	ENOG410Z
P62315	SMD1_MO	Snrpd1	Snrpd1	20641	Small nucle	no | no chan	0	COG1958((

Q541Z2	Q541Z2_M	Fnta	Fnta	14272	Protein farı	no|no chan	0	COG5536(c
Q7TNB8	SBNO2_M(	Sbno2	Sbno2	216161	Protein stra	no|no chan	0	ENOG410)
P61216	TAF13_MC	Taf13	Taf13	99730	Transcripti	no|no chan	0	COG5248(T
Q921N6	DDX27_M	Ddx27	Ddx27	228889	Probable A	no|no chan	0	COG0513(S
Q6NS46	RRP5_MOI	Pdcd11	Pdcd11	18572	Protein RR	no|no chan	0	COG0539(t
P61211	ARL1_MOI	Arl1	Arl1	104303	ADP-ribosy	no|no chan	0	COG1100(p
Q9Z0N2	IF2H_MOU	Eif2s3y	Eif2s3y	26908	Eukaryotic	no|no chan	0	COG5257(C
B1AS37	B1AS37_M	Ssbp3	Ssbp3		Single-strar	no|no chan	0	ENOG410)
Q8BNS1	Q8BNS1_M	Ube3a	Ube3a	22215	HECT dom	no|no chan	0	COG5021(i
Q8BWY4	MBLC1_M(	Mblac1	Mblac1	330216	Metallo-bet	no|up	0	ENOG4111
Q3UR70	TGFA1_M(	Tgfbrap1	Tgfbrap1	73122	Transformi	no|no chan	0	ENOG410)
Q8BTD8	Q8BTD8_M	Pitpnc1	Pitpnc1	71795	Cytoplasmi	no|no chan	0	COG5083(p
Q99LX5	MMTA2_M	Mmtag2	Mmtag2	67862	Multiple m	no|no chan	0	ENOG4112
Q99LX0	PARK7_M(	Park7	Park7	57320	Parkinson c	no|no chan	0	COG0693(I
O35900	LSM2_MOI	Lsm2	Lsm2	27756	U6 snRNA-	no|no chan	0	COG1958(I
A0A0R4J08	A0A0R4J08	Ndufaf1	Ndufaf1	69702	Complex I i	no|no chan	0	ENOG410)
A0A1L1SU	A0A1L1SU	Tube1	Tube1	71924	Tubulin, ep	no|no chan	0	COG5023(t
Q9CY28	GTPB8_M(	Gtpbp8	Gtpbp8	66067	GTP-bindir	no|no chan	0	COG0218(N
Q8BIG4	FBX28_MC	Fbxo28	Fbxo28	67948	F-box only	no|no chan	0	ENOG410Y
O55100	SNG1_MO	Syngr1	Syngr1	20972	Synaptogyri	no|no chan	0	ENOG4111
Q9D8C4	IN35_MOU	Ifi35	Ifi35	70110	Interferon-i	no|up	0	ENOG4111
Q9D8C6	MED11_M	Med11	Med11	66172	Mediator o	no|no chan	0	ENOG4111
O55106	STRN_MO	Strn	Strn	268980	Striatin	no|no chan	0	ENOG410)
Q9CY27	TECR_MO	Tecr	Tecr	106529	Very-long-c	no|no chan	0	ENOG410)
Q60876	4EBP1_MC	Eif4ebp1	Eif4ebp1	13685	Eukaryotic	no|no chan	0	ENOG4112
Q8BU88	RM22_MO	Mrpl22	Mrpl22	216767	Large ribos	no|no chan	0	COG0091(S
Q9D8C9	Q9D8C9_M	Pnp2	Pnp2	667034	Purine nucl	no|no chan	0	COG0005(T
P55258	RAB8A_M(	Rab8a	Rab8a	17274	Ras-related	no|no chan	0	ENOG410)
Q3U8X1	Q3U8X1_M	Eif3f	Eif3f	66085	Eukaryotic	no|no chan	0	COG1310(I
P59017	B2L13_MO	Bcl2l13	Bcl2l13	94044	Bcl-2-like p	no|no chan	0	ENOG410)
P59016	VP33B_MC	Vps33b	Vps33b	233405	Vacuolar p	no|no chan	0	COG5158(T
Q80Y56	RBNS5_M(	Rbsn	Rbsn	78287	Rabenosyn-	no|no chan	0	ENOG410)
A6P3E3	A6P3E3_M	Ncoa2	Ncoa2	17978	Nuclear rec	no|no chan	0	ENOG410)
Q99L20	GSTT3_M(	Gstt3	Gstt3	103140	Glutathion	no|no chan	0	COG0625(I
Q8C0J2	A16L1_MO	Atg16l1	Atg16l1	77040	Autophagy-	no|no chan	0	ENOG410)
Q80Y55	BSDC1_M(	Bsdc1	Bsdc1	100383	BSD domai	no|no chan	0	ENOG411(
A6P3E4	A6P3E4_M	Mapk8	Mapk8	26419	Stress-activ	no|no chan	0	ENOG410)
Q8CA44	Q8CA44_M	Dmac2	Dmac2	66349	Distal mem	no|no chan	0	ENOG4111
Q8CD10	MICU2_M(	Micu2	Micu2	68514	Calcium up	no|no chan	0	ENOG410Z
Q8CD17	Q8CD17_M	Ap4m1	Ap4m1	11781	AP-4 compl	no|no chan	0	ENOG410)
Q8CD15	RIOX2_M(	Riox2	Riox2	67014	Ribosomal	no|no chan	0	ENOG410Y
Q8CD14	Q8CD14_M	Ddx54	Ddx54	71990	RNA helico	no|no chan	0	ENOG410)
A0PJ18	A0PJ18_M(	Il18	Il18	16173	Interleukin-	no|no chan	0	ENOG410Z

P56695	WFS1_MO	Wfs1	Wfs1	22393	Wolframin	no | up	0	ENOG4111
Q8CD19	LANC3_M	Lancl3	Lancl3	236285	LanC-like p	no | no chan	0	ENOG410
Q5F2E7	NUFP2_M	Nufip2	Nufip2	68564	FMR1-inter	no | no chan	0	ENOG411
A0A068EW	A0A068EWW2_MOUSE Gag polyprotein OS=				Gag polypr	no | no chan	0	ENOG410
A2AX52	CO6A4_M	Col6a4	Col6a4	68553	Collagen al	no | no chan	0	ENOG410
G3X908	G3X908_M	Dctd	Dctd	320685	Deoxycytid	no | no chan	0	COG2131(
Q32P04	Q32P04_M	Krt5	Krt5	110308	Keratin, typ	no | up	0	ENOG410
Q9D9H8	CB069_MOUSE Mitochondrial pro			73467	Mitochond	no | no chan	0	ENOG411
Q3TUB5	Q3TUB5_M	Nol10	Nol10	217431	Nucleolar p	no | no chan	0	ENOG410
Q3U816	Q3U816_M	Htatip2	Htatip2	53415	Oxidoreduc	no | no chan	0	COG0702(
Q9DCV4	RMD1_MC	Rmdn1	Rmdn1	66302	Regulator c	no | no chan	0	ENOG410
Q3UIP2	Q3UIP2_M	Pcolce	Pcolce	18542	Procollager	no | no chan	0	ENOG410
P24270	CATA_MO	Cat	Cat	12359	Catalase	no | no chan	0	COG0753(
Q03963	E2AK2_M	Eif2ak2	Eif2ak2	19106	Interferon-i	no | up	0	ENOG410
Q9D967	MGDP1_M	Mdp1	Mdp1	67881	Magnesium	no | no chan	0	ENOG4111
A2BFF7	A2BFF7_M	Dync1i2	Dync1i2	13427	Cytoplasmi	no | no chan	0	ENOG410
Q61553	FSCN1_M	Fscn1	Fscn1	14086	Fascin	no | no chan	0	ENOG410
Q8BGY7	F210A_MO	Fam210a	Fam210a	108654	Protein FA	no | no chan	0	ENOG4111
A0A0G2JF	A0A0G2JF	Ptbp2	Ptbp2		Polypyrimi	no | no chan	0	ENOG410
A0A0R4J0B	A0A0R4J0B	Sh3gl3	Sh3gl3	20408	SH3-domai	no | down	0	ENOG410
Q8C0D7	ING4_MO	Ing4	Ing4	28019	Inhibitor of	no | no chan	0	COG5034(
P09671	SODM_MC	Sod2	Sod2	20656	Superoxide	no | no chan	0	COG0605(
A0A0R4J27	A0A0R4J27	Ndufa12	Ndufa12	66414	NADH deh	no | no chan	0	ENOG4111
A0A0R4J0B	A0A0R4J0B	Mark2	Mark2	13728	non-specifi	no | no chan	0	ENOG410
A0A0R4J0B	A0A0R4J0B	Plbd1	Plbd1	66857	Phospholip	no | up	0	ENOG410
A0A0R4J0B	A0A0R4J0B	Ulk1	Ulk1	22241	Serine/thre	no | no chan	0	ENOG410
A0A0R4J0B	A0A0R4J0B	Cmas	Cmas	12764	N-acylneur	no | no chan	0	COG1083(
A0A0R4J0B	A0A0R4J0B	Rusf1	Rusf1	233913	RUS family	no | no chan	0	ENOG410
A0A0R4J0B	A0A0R4J0B	Abcg2	Abcg2	26357	Broad subst	no | no chan	0	COG1131(
A0A0R4J27	A0A0R4J27	Tmem70	Tmem70	70397	Transmemb	no | no chan	0	ENOG4111
D3YW62	D3YW62_M	Snx10	Snx10		Sorting nex	no | up	0	COG5391(
Q8CHQ0	FBX4_MO	Fbxo4	Fbxo4	106052	F-box only	no | no chan	0	ENOG410
Q8R5M0	GIPC3_MC	Gipc3	Gipc3	209047	PDZ domai	no | no chan	0	ENOG410
A2AFF6	A2AFF6_M	Stag2	Stag2	20843	Cohesin sul	no | no chan	0	COG5537(
E9PYG6	E9PYG6_M	Rasa1	Rasa1	218397	RAS p21 pr	no | no chan	0	ENOG410
O88574	SAP30_MO	Sap30	Sap30	60406	Histone dec	no | no chan	0	ENOG4111
A2AUF7	A2AUF7_M	Rabepk	Rabepk		Rab9 effect	no | no chan	0	ENOG410
A0A0J9YT	A0A0J9YT	Septin11	Septin11		Septin	no | no chan	0	COG5019(
A0A494BA	A0A494BA	Ankrd1	Ankrd1		Ankyrin rep	no | down	0	COG0666(
H9KV00	H9KV00_M	Son	Son		Son DNA b	no | no chan	0	ENOG410
Q99JY0	ECHB_MO	Hadhb	Hadhb	231086	Trifunction	no | no chan	0	COG0183(
H9KV07	H9KV07_M	Nphp3	Nphp3		Nephrocyst	no | no chan	0	COG0457(
Q61838	PZP_MOU	Pzp	Pzp	11287	Pregnancy	no | no chan	0	COG2373(

A1ILG8	A1ILG8_M	Vps13c	Vps13c	320528	Chorein	no|no chan	0	COG5043(I
Q3ULP8	Q3ULP8_M	Pus7	Pus7	78697	TRUD dom	no|no chan	0	COG0585(I
E9Q6E9	SIMC1_MC	Simc1	Simc1	319719	SUMO-inte	yes|down	1	ENOG410X
P35505	FAAA_MO	Fah	Fah	14085	Fumarylace	no|no chan	0	COG0179(I
E9Q6E5	E9Q6E5_M	Srsf11	Srsf11	69207	Serine and	no|no chan	0	ENOG410Y
Q922Q2	RIOK1_MC	Riok1	Riok1	71340	Serine/thre	no|no chan	0	COG1718(p
A0A0D5ZY	A0A0D5ZY64_MOUSE	IgE L chain kappa OS			IgE L chain	no|up	0	ENOG4111
Q3ULP2	Q3ULP2_M	Wdr20	Wdr20	69641	Uncharacte	no|no chan	0	COG2319(I
A0A991ELI	A0A991ELI	Ptprg	Ptprg	19270	protein-tyr	no|no chan	0	COG5599(p
Q8CHK3	MBOA7_M	Mboat7	Mboat7	77582	Lysophosph	no|no chan	0	COG5202(r
Q9CQJ6	DENR_MC	Denr	Denr	68184	Density-reg	no|no chan	0	COG0023(c
Q9CQJ4	RING2_MC	Rnf2	Rnf2	19821	E3 ubiquiti	no|no chan	0	ENOG410X
Q9CQJ2	PIHD1_MC	Pih1d1	Pih1d1	68845	PIH1 doma	no|no chan	0	ENOG410X
F8WID5	F8WID5_M	Tpm1	Tpm1		Tropomyos	no|down	0	ENOG410X
A0A0R4J1L	A0A0R4J1L	Zkscan3	Zkscan3	72739	Zinc finger	no|no chan	0	COG5048(c
A0A0U1RN	A0A0U1RN	Maz	Maz		MYC-assoc	no|no chan	0	COG5048(c
A0A0R4J1L	A0A0R4J1L	Tnk2	Tnk2	51789	non-specifi	no|no chan	0	ENOG410X
A0A5F8MP	A0A5F8MP	Rapgef1	Rapgef1	107746	Rap guanin	no|no chan	0	ENOG410X
A0A0U1RN	A0A0U1RN	Bckdk	Bckdk		Protein-seri	no|no chan	0	COG0642(c
Q9D2N9	VP33A_MC	Vps33a	Vps33a	77573	Vacuolar p	no|no chan	0	COG5158(V
Q70IV5	SYNEM_M	Synm	Synm	233335	Synemin	no|up	0	ENOG4111
Q0VEW4	Q0VEW4_M	Plp2	Plp2	18824	Proteolipid	no|no chan	0	ENOG4111
P97819	PLPL9_MC	Pla2g6	Pla2g6	53357	85/88 kDa	no|no chan	0	COG3621(I
Q5SWW4	MED13_M	Med13	Med13	327987	Mediator of	no|no chan	0	ENOG410X
Q6XPS7	Q6XPS7_M	Tha1	Tha1	71776	L-threonine	no|no chan	0	COG2008(A
A0A1Y7VN	A0A1Y7VN	Glrx5	Glrx5		Glutaredox	no|no chan	0	COG0278(Y
P97813	PLD2_MO	Pld2	Pld2	18806	Phospholip	no|no chan	0	COG1502(f
A0A2I3BQ	A0A2I3BQ	Tmem184b	Tmem184b	223693	Transmemb	no|no chan	0	ENOG410X
Q8C7U7	GALT6_MC	Galnt6	Galnt6	207839	Polypeptide	no|up	0	ENOG410X
Q8C7U1	N4BP3_MC	N4bp3	N4bp3	212706	NEDD4-bi	no|no chan	0	ENOG410X
Q3U7K7	Q3U7K7_M	Trim21	Trim21	20821	RING-type	no|no chan	0	ENOG4111
A0A2I3BQ	A0A2I3BQ	Zfp219	Zfp219		Zinc finger	yes|down	1	COG5048(t
Q6ZQA6	IGSF3_MO	Igsf3	Igsf3	78908	Immunoglo	no|no chan	0	ENOG410Y
A2A6K0	A2A6K0_M	Tnni2	Tnni2		Troponin I,	no|up	0	ENOG410Y
Q9QZL0	RIPK3_MC	Ripk3	Ripk3	56532	Receptor-in	no|no chan	0	COG0515(s
P14719	ILRL1_MO	Il1rl1	Il1rl1	17082	Interleukin-	no|no chan	0	ENOG411(
Q542H2	Q542H2_M	Psma7	Psma7	26444	Proteasome	no|no chan	0	ENOG410X
B2RS09	B2RS09_M	Brd2	Brd2	14312	Bromodom	no|no chan	0	COG5076(t
Q8BVA2	TM222_MC	Tmem222	Tmem222	52174	Transmemb	no|no chan	0	ENOG4111
A0A1B0GR	A0A1B0GR	Wrn	Wrn		DNA 3'-5' h	no|no chan	0	COG0514(e
G3UWZ0	G3UWZ0_M	Baz1a	Baz1a	217578	Bromodom	no|no chan	0	COG5076(C
G3UWZ1	G3UWZ1_M	Pear1	Pear1		Platelet enc	yes|down	1	ENOG410X
A0A1B0GR	A0A1B0GR	Rnf170	Rnf170	77733	Ring finger	no|no chan	0	ENOG410X

A2AMU9	A2AMU9_M	Mrto4	Mrto4	69902	Ribosome a	no | no chan	0	COG0244(S
Q5SXG1	Q5SXG1_M	Mink1	Mink1		non-specifi	no | no chan	0	ENOG410X
Q8BL66	EEA1_MO	Eea1	Eea1	216238	Early endos	no | no chan	0	ENOG4111
Q8BL63	Q8BL63_M	Pigk	Pigk	329777	GPI-ancho	no | no chan	0	COG5206(p
A2A7S7	A2A7S7_M	Yars1	Yars1	107271	Tyrosine--t	no | no chan	0	COG0162(E
P40240	CD9_MOU	Cd9	Cd9	12527	CD9 antige	no | up	0	ENOG4111
Q8K2X7	Q8K2X7_M	Xpa	Xpa	22590	Xeroderma	no | no chan	0	COG5145(L
Q3U1V3	Q3U1V3_M	Grk2	Grk2	110355	G protein-c	no | no chan	0	ENOG410Y
Q8K2X8	TF2H5_MC	Gtf2h5	Gtf2h5	66467	General tra	no | no chan	0	ENOG4112
Q6PB75	PAPD7_MC	Tent4a	Tent4a	210106	Terminal n	no | down	0	COG5260(L
A2A7S8	NHSL3_MC	Nhsl3	Nhsl3	97130	NHS-like p	no | no chan	0	ENOG410Y
D3YZG8	MTD2L_M	Mthfd2l	Mthfd2l	665563	Bifunctiona	no | no chan	0	COG0190(C
Q9JM13	RABX5_MC	Rabgef1	Rabgef1	56715	Rab5 GDP,	no | no chan	0	ENOG410Y
Q9JM14	NT5C_MO	Nt5c	Nt5c	50773	5'(3')-deoxy	no | no chan	0	ENOG410X
Q920S3	GATD1_M	Gatad1	Gatad1	67210	GATA zinc	no | no chan	0	ENOG410X
Q5U421	Q5U421_M	Mapk14	Mapk14	26416	Mitogen-ac	no | no chan	0	ENOG410X
E9Q8Z6	E9Q8Z6_M	Ctnnd1	Ctnnd1	12388	Catenin del	no | no chan	0	ENOG410Y
P33611	DPOA2_M	Pola2	Pola2	18969	DNA polym	no | no chan	0	COG5214(L
P33610	PRI2_MOU	Prim2	Prim2	19076	DNA prima	no | no chan	0	COG2219(L
P16254	SRP14_MO	Srp14	Srp14	20813	Signal reco	no | no chan	0	ENOG4111
P22935	RABP2_MC	Crabp2	Crabp2	12904	Cellular ret	no | no chan	0	ENOG4111
Q8BSA9	TYW3_MO	Tyw3	Tyw3	209584	tRNA wybu	no | no chan	0	COG1590(t
P62242	RS8_MOUS	Rps8	Rps8	20116	Small ribos	no | no chan	0	COG2007(r
P62245	RS15A_MC	Rps15a	Rps15a	267019	Small ribos	no | no chan	0	COG0096(C
F8WHN4	F8WHN4_M	Ralgapb	Ralgapb	228850	Ral GTPase	no | no chan	0	ENOG411C
Q62193	RFA2_MOU	Rpa2	Rpa2	19891	Replication	no | no chan	0	COG5235(M
A2AFQ0	A2AFQ0_M	Huwe1	Huwe1	59026	HECT-type	no | no chan	0	COG5021(E
E9Q3X4	E9Q3X4_M	Hps5	Hps5	246694	Hermansky	no | no chan	0	ENOG4111
Q3UGS4	MCRI1_MC	Mcrip1	Mcrip1	192173	Mapk-regul	no | no chan	0	ENOG4112
E9Q3X0	E9Q3X0_M	Mvp	Mvp	78388	Major vault	no | no chan	0	ENOG410Z
Q3TDA7	Q3TDA7_M	Pacsin2	Pacsin2	23970	Protein kin	no | no chan	0	ENOG410X
Q3TCW3	Q3TCW3_M	Fuca1	Fuca1	71665	Alpha-L-fu	no | no chan	0	COG3669(c
Q3TDA1	Q3TDA1_M	Osbpl2	Osbpl2	228983	Oxysterol-b	no | no chan	0	ENOG410X
Q3TCW5	Q3TCW5_M	Nrbp1	Nrbp1	192292	Protein kin	no | no chan	0	ENOG410X
Q9DAW9	CNN3_MO	Cnn3	Cnn3	71994	Calponin-3	no | no chan	0	COG5199(C
Q9CQ25	MZT2_MO	Mzt2	Mzt2	72083	Mitotic-spi	no | no chan	0	ENOG410Y
Q9CQ22	LTOR1_MC	Lamtor1	Lamtor1	66508	Ragulator c	no | no chan	0	ENOG4112
A0A087WR	A0A087WR	Nucks1	Nucks1	98415	Nuclear cas	no | up	0	ENOG410X
Q9CQ21	MCTS2_MC	Mcts2	Mcts2	66405	Malignant	no | no chan	0	COG2016(E
Q9JJC9	HERP2_MC	Herpud2	Herpud2	80517	Homocystei	no | no chan	0	ENOG410Z
Q9CQ28	DPH6_MO	Dph6	Dph6	66632	Diphthine-	no | no chan	0	COG2102(A
Q6PE54	DHX40_MC	Dhx40	Dhx40	67487	Probable A	no | no chan	0	COG1643(S
Q80X45	KISHB_MC	Tmem167b	Tmem167b	67495	Protein kish	no | no chan	0	ENOG4112

A0A0U1RN	A0A0U1RN	Calca	Calca		Calcitonin/	yes|up	1	ENOG4111
Q8BFZ3	ACTBL_M(	Actbl2	Actbl2	238880	Beta-actin-:	no|no chan	0	COG5277(r
Q3TRJ7	Q3TRJ7_M	Sh3gl1	Sh3gl1	20405	Endophilin	no|no chan	0	ENOG410X
V9GX91	V9GX91_M	Nav2	Nav2	78286	Neuron nav	no|up	0	ENOG410X
G3X9W4	G3X9W4_M	Nr2c2	Nr2c2	22026	Nuclear rec	no|no chan	0	ENOG410X
D3Z0X5	D3Z0X5_M	Phldb1	Phldb1		Pleckstrin h	no|no chan	0	ENOG411(
Q3TRJ1	Q3TRJ1_M	Vps35	Vps35	65114	Vacuolar p:	no|no chan	0	ENOG410X
Q3U6C7	Q3U6C7_M	Sucla2	Sucla2	20916	Succinate-C	no|no chan	0	COG0045(S
Q8BFZ9	ERLN2_M(	Erlin2	Erlin2	244373	Erlin-2	no|no chan	0	ENOG410X
O54752	O54752_M(	Naglu	Naglu	27419	Naglu	no|no chan	0	ENOG410X
Q921K8	TCAF2_MC	Tcaf2	Tcaf2	232748	TRPM8 chc	no|down	0	ENOG410Y
Q80Y09	Q80Y09_M	Pdcd6ip	Pdcd6ip	18571	Pdcd6ip prc	no|down	0	ENOG410X
D3YWH4	D3YWH4_I	Tm9sf1	Tm9sf1		Transmemb	no|no chan	0	ENOG410X
P47738	ALDH2_M	Aldh2	Aldh2	11669	Aldehyde d	no|no chan	0	COG1012(p
A2RSP9	A2RSP9_M	Reck	Reck	53614	Reck protei	no|no chan	0	ENOG410Y
Q3TFK5	GPTC4_M(	Gpatch4	Gpatch4	66614	G patch do	no|no chan	0	ENOG4111
A0A0G2JF!	A0A0G2JF!	Tet2	Tet2	214133	Methylcyto	no|no chan	0	ENOG410X
Q8CHT0	AL4A1_MC	Aldh4a1	Aldh4a1	212647	Delta-1-pyr	no|no chan	0	COG1012(I
Q60676	PPP5_MOU	Ppp5c	Ppp5c	19060	Serine/thre	no|no chan	0	COG0639(s
Q8CHT3	INT5_MOU	Ints5	Ints5	109077	Integrator c	no|no chan	0	ENOG411(
Q3U661	Q3U661_M	Tox4	Tox4	268741	TOX high r	no|no chan	0	COG5648(r
Q9JMG2	C1GLC_M(	C1galt1c1	C1galt1c1	59048	C1GALT1-s	no|no chan	0	ENOG410Y
A2AKX3	SETX_MO	Setx	Setx	269254	Probable h	no|no chan	0	COG1112(N
E9Q7K1	E9Q7K1_M	Guk1	Guk1	14923	guanylate k	no|no chan	0	COG0194(N
A0A2I3BPV	A0A2I3BPV	Ndrg2	Ndrg2		Protein ND	yes|down	1	ENOG410X
Q6PDR1	Q6PDR1_M	Lipa	Lipa	16889	Lipase	no|no chan	0	COG0596(I
A0A654ICX	A0A654ICX	Gjb3	Gjb3	14620	Gap junctic	no|up	0	ENOG410Y
Q2M2Q8	Q2M2Q8_M	Msi2	Msi2	76626	Msi2h prot	no|no chan	0	ENOG410Y
Q3TVW6	Q3TVW6_I	Lmcd1	Lmcd1	30937	LIM and cy	no|up	0	ENOG410Z
P35979	RL12_MOU	Rpl12	Rpl12	269261	Large ribos	no|no chan	0	COG0080(I
Q4FJR4	Q4FJR4_M	Pdk3	Pdk3	236900	Protein-seri	no|no chan	0	COG0642(A
Q4FJR0	Q4FJR0_M	Nudt4	Nudt4	71207	diphosphoi	no|no chan	0	COG0494(c
Q9D8P4	RM17_MO	Mrpl17	Mrpl17	27397	Large ribos	no|no chan	0	COG0203(S
A0A0N4SV	A0A0N4SV	Arhgdib	Arhgdib		Rho, GDP c	yes|up	1	ENOG4111
Q9DC71	RT15_MOU	Mrps15	Mrps15	66407	Small ribos	no|no chan	0	COG0184(I
Q4FJR9	Q4FJR9_M	Isg15	Isg15	100038882	G1p2 prote	no|up	0	COG5272(A
Q8BYL4	SYYM_MO	Yars2	Yars2	70120	Tyrosine--tI	no|no chan	0	COG0162(C
Q99KW3	TARA_MO	Triobp	Triobp	110253	TRIO and I	no|no chan	0	ENOG410Z
Q3U489	Q3U489_M	Ak4	Ak4	11639	Adenylate k	no|up	0	COG0563(U
Q8VE95	CH082_MOUSE	UPF0598 protein (		223665	UPF0598 pr	no|up	0	ENOG4111
P28571	SC6A9_MC	Slc6a9	Slc6a9	14664	Sodium- an	no|no chan	0	COG0733(T
Q3U487	HECD3_M	Hectd3	Hectd3	76608	E3 ubiquiti	no|no chan	0	COG5021(U
P01675	KV6A1_MOUSE	Ig kappa chain V-VI region X			Ig kappa ch	no|up	0	ENOG4111

Q01730	RSU1_MOU	Rsu1	Rsu1	20163	Ras suppres	no	no chan	0	COG4886(F
Q3U480	Q3U480_M	Nop9	Nop9	67842	Nucleolar p	no	no chan	0	ENOG410Y
D4Q8F9	D4Q8F9_M	B3gat3	B3gat3	72727	Galactosylg	no	no chan	0	ENOG410X
Q6P264	Q6P264_M	Fn3k	Fn3k	63828	protein-ribu	no	no chan	0	COG3001(F
Q61151	2A5E_MOU	Ppp2r5e	Ppp2r5e	26932	Serine/thre	no	no chan	0	ENOG410X
Q8BLN5	LSS_MOUS	Lss	Lss	16987	Lanosterol	no	no chan	0	COG1657(S
P08249	MDHM_M	Mdh2	Mdh2	17448	Malate deh	no	no chan	0	COG0039(C
Q9QYP6	AZI2_MOU	Azi2	Azi2	27215	5-azacytidi	no	no chan	0	ENOG4111
A0A1Y7VL	A0A1Y7VL	Ttc7b	Ttc7b		Tetratricop	no	no chan	0	ENOG410X
Q9D3D0	TTPAL_MO	Ttpal	Ttpal	76080	Alpha-toco	no	no chan	0	ENOG410X
Q9R0Y5	KAD1_MO	Ak1	Ak1	11636	Adenylate k	no	no chan	0	COG0563(A
D3Z4D7	D3Z4D7_M	Mfap5	Mfap5		Microfibrill	no	no chan	0	ENOG410Y
Q61102	ABCB7_MO	Abcb7	Abcb7	11306	Iron-sulfur	no	no chan	0	COG5265(f
K4DI77	K4DI77_M	Wdr81	Wdr81		WD repeat	no	no chan	0	ENOG410X
A0A571BE	A0A571BE	Lcor	Lcor		Ligand dep	no	no chan	0	ENOG4111
B7ZCM9	B7ZCM9_M	Gm28042	Gm28042		Phospholip	no	no chan	0	ENOG410X
Q3TEE6	Q3TEE6_M	Fermt3	Fermt3	108101	PH domain	no	no chan	0	ENOG410X
Q7TSH6	SCAF4_MO	Scaf4	Scaf4	224432	SR-related	no	down	0	ENOG410Z
Q7TSH3	ZN516_MO	Znf516	Znf516	329003	Zinc finger	no	no chan	0	COG5048(F
Q7TSH2	KPBB_MO	Phkb	Phkb	102093	Phosphoryl	no	no chan	0	ENOG410X
F6QCI0	F6QCI0_M	Taf15	Taf15		TATA-box	no	no chan	0	ENOG4111
P27784	CCL6_MOU	Ccl6	Ccl6	20305	C-C motif c	no	down	0	ENOG4111
A0A1D5RL	A0A1D5RL	Cdc42bpa	Cdc42bpa		non-specifi	no	no chan	0	ENOG410X
O08992	SDCB1_MO	Sdcbp	Sdcbp	53378	Syntenin-1	no	no chan	0	ENOG410Y
O08997	ATOX1_M	Atox1	Atox1	11927	Copper tra	no	no chan	0	COG2608(F
Q8CI51	PDLI5_MO	Pdlim5	Pdlim5	56376	PDZ and L	no	no chan	0	ENOG410X
Q8BUE4	FSP1_MOU	Aifm2	Aifm2	71361	Ferroptosis	no	no chan	0	COG1252(N
Q9QZQ8	H2AY_MO	Macroh2a1	Macroh2a1	26914	Core histon	no	no chan	0	COG5262(r
A0A286YD	A0A286YD	Atxn2	Atxn2		Ataxin 2	no	up	0	COG5180(s
P62880	GBB2_MO	Gnb2	Gnb2	14693	Guanine nu	no	no chan	0	ENOG410X
Q3V1G4	OLM2B_M	Olfml2b	Olfml2b	320078	Olfactomec	no	no chan	0	ENOG410Z
H3BKC5	H3BKC5_M	Lsm5	Lsm5		LSM5 homo	no	no chan	0	COG1958((
Q4V9V3	Q4V9V3_M	Elovl1	Elovl1	54325	Elongation	no	no chan	0	ENOG410X
Q3TX36	Q3TX36_M	Parp1	Parp1	11545	Poly [ADP-	no	no chan	0	ENOG410X
P62889	RL30_MOU	Rpl30	Rpl30	19946	Large ribos	no	no chan	0	COG1911((
A0A158RF	A0A158RF	Paip2b	Paip2b	232164	Poly(A) bin	no	down	0	ENOG4111
A0A158RF	A0A158RF	Raver1	Raver1	71766; 1005	Ribonucleo	no	no chan	0	ENOG410Z
Q3THE8	Q3THE8_M	Fkrp	Fkrp	243853	Ribitol-5-pl	no	no chan	0	ENOG410X
Q8K4F6	NSUN5_M	Nsun5	Nsun5	100609	28S rRNA (	no	no chan	0	COG0144(p
Q3THE7	Q3THE7_M	Aldh9a1	Aldh9a1	56752	4-trimethyl	no	no chan	0	COG1012(F
Q3THE2	ML12B_MO	Myl12b	Myl12b	67938	Myosin reg	no	no chan	0	COG5126(F
Q3UFT3	GARE1_M	Garem1	Garem1	381126	GRB2-asso	no	up	0	ENOG410X
Q8VDB2	ALG12_MO	Alg12	Alg12	223774	Dol-P-Man	no	no chan	0	ENOG410X

Q91WE6	CDKAL_M	Cdkal1	Cdkal1	68916	Threonylca	no | no chan	0	COG0621((
Q91WU5	AS3MT_M(	As3mt	As3mt	57344	Arsenite me	no | no chan	0	ENOG410X
Q62179	SEM4B_M(	Sema4b	Sema4b	20352	Semaphorin	no | no chan	0	ENOG410X
Q62178	SEM4A_M(	Sema4a	Sema4a	20351	Semaphorin	no | no chan	0	ENOG410X
Q5SSH8	NEUFC_M	Cyb5d2	Cyb5d2	192986	Neuferricin	no | up	0	ENOG4111
G3XA08	G3XA08_M	Zfp26	Zfp26	22688	Zinc finger	no | no chan	0	COG5048(I
A0A1S6GW	A0A1S6GW	Stt3a	Stt3a	16430	Dolichyl-di	no | no chan	0	COG1287(c
A0A1S6GW	A0A1S6GW	Paxbp1	Paxbp1	67367	GCF C-term	no | no chan	0	ENOG410Y
A2ANP1	A2ANP1_M	Fam219a	Fam219a	71901	2310028H24	no | down	0	ENOG410Y
P26350	PTMA_MC	Ptma	Ptma	19231	Prothymosi	no | down	0	ENOG410Z
Q3UVK0	ERMP1_M(	Ermp1	Ermp1	226090	Endoplasm	no | no chan	0	COG2234(p
A0A0R4J02	A0A0R4J02	Auh	Auh	11992	AU RNA bi	no | no chan	0	COG1024(p
A0A0R4J02	A0A0R4J02	Xrcc4	Xrcc4	108138	X-ray repai	no | no chan	0	ENOG410Y
A0A0R4J02	A0A0R4J02	Rcbtb1	Rcbtb1	71330	Regulator c	no | no chan	0	COG5184(A
A0A0R4J02	A0A0R4J02	Acod1	Acod1	16365	Aconitate d	no | up	0	COG2079(M
Q8R1S0	COQ6_MC	Coq6	Coq6	217707	Ubiquinone	no | no chan	0	COG0654(z
Q8VIG6	TRAIP_MC	Traip	Traip	22036	E3 ubiquiti	no | down	0	ENOG410Y
Q8R1S9	S38A4_MO	Slc38a4	Slc38a4	69354	Sodium-cou	no | no chan	0	COG0814(z
B2RXC8	B2RXC8_M	Ppp2r3a	Ppp2r3a	235542	3222402P14	no | no chan	0	ENOG410X
E9PZX7	E9PZX7_M	Cdk4	Cdk4		Cyclin-depe	no | no chan	0	ENOG410X
B2KFY0	B2KFY0_M	Serinc2	Serinc2	230779	Serine inco	no | no chan	0	ENOG410X
B2RXC1	TPC11_MC	Trappc11	Trappc11	320714	Trafficking	no | no chan	0	ENOG410X
E9PX91	E9PX91_M	Pisd	Pisd	320951	Phosphatid	no | no chan	0	COG0688(I
B2RXC6	B2RXC6_M	Polr3a	Polr3a	218832	DNA-direc	no | no chan	0	COG0086(t
A0A498WC	A0A498WC	Synpo2	Synpo2		Synaptopoc	no | up	0	ENOG410Z
B2RWR2	B2RWR2_M	Lpcat2	Lpcat2	270084	Lpcat2 prot	no | no chan	0	ENOG410X
A0A571BD	A0A571BD	Rab3il1	Rab3il1		RAB3A inte	no | no chan	0	ENOG410X
Q6P8M1	TATD1_M(	Tatdn1	Tatdn1	69694	Deoxyribon	no | no chan	0	COG0084(Z
Q9D154	ILEUA_M(	Serpinb1a	Serpinb1a	66222	Leukocyte e	no | up	0	COG4826(s
G3X9J0	SI1L3_MO	Sipa1l3	Sipa1l3	74206	Signal-indu	no | no chan	0	ENOG410X
V9GWU5	V9GWU5_I	Tpd52l2	Tpd52l2		Tumor prot	no | up	0	ENOG4111
A0A087WQ	A0A087WQ	Kif1a	Kif1a		Kinesin fan	no | no chan	0	COG5059(T
Q8K1A6	C2D1A_M(	Cc2d1a	Cc2d1a	212139	Coiled-coil	no | no chan	0	ENOG410X
A0A0A6YW	A0A0A6YW	Rapgef2	Rapgef2		Rap guanin	no | no chan	0	
A0A1Y7VN	A0A1Y7VN	Dcaf4	Dcaf4	73828	DDB1 and	no | no chan	0	ENOG410X
Q8BUN5	SMAD3_M	Smad3	Smad3	17127	Mothers ag	no | no chan	0	ENOG410X
A0A0A0M(	A0A0A0M(	Meis1	Meis1		Meis home	no | no chan	0	ENOG410X
A0A1Y7VN	A0A1Y7VN	Mark3	Mark3	17169	non-specifi	no | no chan	0	ENOG410X
E9PXB0	E9PXB0_M	Pnpla8	Pnpla8		Patatin-like	no | no chan	0	ENOG410X
Q5Y5T1	ZDH20_M(	Zdhhc20	Zdhhc20	75965	Palmitoyltr	no | no chan	0	COG5273(j
A0A0G2JE	A0A0G2JE	Lrrfip2	Lrrfip2		Leucine-ric	no | no chan	0	ENOG4111
Q7TMB8	CYFP1_M(	Cyfip1	Cyfip1	20430	Cytoplasmi	no | no chan	0	ENOG410X
A2ACP1	TT39A_MC	Ttc39a	Ttc39a	230603	Tetratricop	no | no chan	0	ENOG410X

Q8VEA8	RAB7B_M(	Rab7b	Rab7b	226421	Ras-related	no	no chan	0	ENOG410)
Q8R0K4	CC137_MO	Ccdc137	Ccdc137	67291	Coiled-coil	no	no chan	0	ENOG4111
Q8R0K9	E2F4_MOU	E2f4	E2f4	104394	Transcripti	no	no chan	0	ENOG410)
P06151	LDHA_MC	Ldha	Ldha	16828	L-lactate de	no	no chan	0	COG0039((
Q80VC6	TSAP1_MC	Trnau1ap	Trnau1ap	71787	tRNA selen	no	no chan	0	COG0724(S
Q3UL79	Q3UL79_MOUSE Ferritin OS=Mu			14325	Ferritin	no	no chan	0	COG1528(S
F6QYS7	F6QYS7_M	Amot	Amot		Angiomotir	no	no chan	0	ENOG410)
Q3UG02	Q3UG02_M	Dusp6	Dusp6	67603	Dual specif	no	no chan	0	COG2453((
Q7TSG1	CE120_MO	Cep120	Cep120	225523	Centrosom	no	no chan	0	ENOG410)
Q3TMS3	Q3TMS3_M	Xylb	Xylb	102448	Carbohydr	no	no chan	0	COG1070((
Q9CXR6	Q9CXR6_M	Mis18a	Mis18a	66578	Protein Mis	no	no chan	0	ENOG4112
B2RTP7	B2RTP7_M	Krt2	Krt2	16681	Krt2 protei	no	no chan	0	ENOG410Y
Q8BZU6	Q8BZU6_M	Tnfrsf21	Tnfrsf21	94185	Tumor necr	no	down	0	ENOG410Y
A4QPD3	A4QPD3_M	Rel	Rel	19696	Reticuloen	no	no chan	0	ENOG410)
Q9Z1T6	FYV1_MOU	Pikfyve	Pikfyve	18711	1-phosphat	no	no chan	0	COG0459(b
A0A0R4J1V	A0A0R4J1V	Asb8	Asb8	78541	Ankyrin rep	no	no chan	0	COG0666(I
A0A0R4J1V	A0A0R4J1V	Cdc23	Cdc23	52563	CDC23 cell	no	no chan	0	ENOG410)
Q9Z1T1	AP3B1_MC	Ap3b1	Ap3b1	11774	AP-3 compl	no	no chan	0	COG5096(b
Q3UMQ2	Q3UMQ2_	Dr1	Dr1	13486	Protein Dr1	no	no chan	0	COG5150(I
Q8K135	K319L_MC	Kiaa0319l	Kiaa0319l	100317	Dyslexia-as	no	no chan	0	ENOG410)
Q8BP43	Q8BP43_M	Tpm1	Tpm1	22003	Tropomyos	no	up	0	ENOG410)
Q9CRY7	GDPD1_M	Gdpd1	Gdpd1	66569	Lysophosph	no	no chan	0	COG0584((
E9PUQ8	DGKD_M(	Dgkd	Dgkd	227333	Diacylglyce	no	no chan	0	ENOG410)
Q9CRC9	GNPI2_M(	Gnpda2	Gnpda2	67980	Glucosami	no	no chan	0	COG0363((
E9PUQ7	E9PUQ7_M	Trmt2a	Trmt2a	15547	tRNA (urac	no	no chan	0	COG2265(c
E9PUQ3	E9PUQ3_M	Nkapd1	Nkapd1	270156	NKAP dom	no	no chan	0	ENOG4112
O70551	SRPK1_M(	Srpk1	Srpk1	20815	SRSF protei	no	no chan	0	ENOG410)
Q6NXX6	Q6NXX6_I	Atp6v0a1	Atp6v0a1	11975	V-type prot	no	no chan	0	COG1269(p
Q9Z0H8	CLIP2_MO	Clip2	Clip2	269713	CAP-Gly do	no	no chan	0	COG5244((
Q91X21	K2013_MO	Kiaa2013	Kiaa2013	77034	Uncharacte	no	no chan	0	ENOG410Y
Q8BV13	CSN7B_M(	Cops7b	Cops7b	26895	COP9 signc	no	no chan	0	ENOG410)
F8WGT1	F8WGT1_M	Ahcyl2	Ahcyl2	74340	S-adenosylh	no	no chan	0	COG0499(S
Q69ZQ1	MYORG_M	Myorg	Myorg	329828	Myogenesis	no	no chan	0	COG1501(N
P39876	TIMP3_MC	Timp3	Timp3	21859	Metallopro	no	up	0	ENOG411(
Q69ZQ2	ISY1_MOU	Isy1	Isy1	57905	Pre-mRNA-	no	no chan	0	ENOG410)
Q924L1	LTMD1_M	Letmd1	Letmd1	68614	LETM1 dor	no	no chan	0	ENOG410Z
A0A087WS	A0A087WS	Minar1	Minar1	209743	Membrane	no	up	0	ENOG410Z
Q9CWA5	Q9CWA5_I	Nme3	Nme3	79059	Nucleoside	no	down	0	COG0105(p
O35382	EXOC4_M	Exoc4	Exoc4	20336	Exocyst cor	no	no chan	0	ENOG410Z
Q9CPW3	RM54_MO	Mrpl54	Mrpl54	66047	Large ribos	no	no chan	0	ENOG4112
A2AQ41	A2AQ41_M	Fnbp1	Fnbp1	14269	Formin bin	no	no chan	0	ENOG410Z
O35385	PPE2_MOU	Ppef2	Ppef2	19023	Serine/thre	no	up	0	COG0639(I
O35386	PAHX_MC	Phyh	Phyh	16922	Phytanoyl-(	no	no chan	0	COG5285(p

Q8HW98	IGLO5_M(	Iglon5	Iglon5	210094	IgLON fam	no|no chan	0	ENOG411(
Q9CPW9	MAP12_M(	Metap1d	Metap1d	66559	Methionine	no|no chan	0	COG0024(F
O35435	PYRD_MO	Dhodh	Dhodh	56749	Dihydrooro	no|no chan	0	COG0167(C
A2A9C3	SZT2_MOU	Szt2	Szt2	230676	KICSTOR c	no|no chan	0	ENOG410X
E9QNT5	E9QNT5_M	Taf12	Taf12		Transcripti	no|no chan	0	COG5624(T
Q9Z2R9	E2AK1_M(	Eif2ak1	Eif2ak1	15467	Eukaryotic	no|no chan	0	ENOG410Y
Q14BA9	Q14BA9_M	Spc24	Spc24	67629	Kinetochor	no|no chan	0	ENOG4111
Q8VBT6	APOBR_M	Apobr	Apobr	171504	Apolipopro	no|no chan	0	ENOG410X
Q8VBT1	TXLNB_M	Txlnb	Txlnb	378431	Beta-taxilir	no|up	0	ENOG410X
Q8VBT0	TMX1_MO	Tmx1	Tmx1	72736	Thioredoxir	no|no chan	0	ENOG4111
Q3TM09	Q3TM09_M	Nup88	Nup88	19069	Nuclear po	no|no chan	0	ENOG410Y
Q3TM08	Q3TM08_M	Sumf2	Sumf2	67902	Sulfatase-m	no|no chan	0	COG1262(S
Q6PE15	ABHDA_M	Abhd10	Abhd10	213012	Palmitoyl-p	no|no chan	0	COG0596(c
Q9D3S3	SNX29_M(	Snx29	Snx29	74478	Sorting nex	yes|down	1	ENOG410X
Q8VBT9	ASPC1_MC	Aspscr1	Aspscr1	68938	Tether cont	no|no chan	0	ENOG410X
Q60855	RIPK1_MC	Ripk1	Ripk1	19766	Receptor-in	no|no chan	0	COG0515(c
A0A0A6YY	A0A0A6YY	Camsap2	Camsap2	67886	Calmodulir	no|no chan	0	ENOG411(
Q8R2G6	CCD80_M(	Ccdc80	Ccdc80	67896	Coiled-coil	no|no chan	0	ENOG410X
A0A0A6YY	A0A0A6YY	Pfkfb3	Pfkfb3	170768	6-phosphof	no|no chan	0	COG0406(F
P0DOV2	IFI4_MOU	Ifi204	Ifi204	15951	Interferon-c	no|up	0	ENOG410Y
P0DOV1	IFI5B_MO	Ifi211	Ifi211	381308	Interferon-c	no|up	0	ENOG410Y
D3YXB7	D3YXB7_M	Spg7	Spg7		SPG7, para	no|no chan	0	COG0465(Y
Q3UUJ4	STRAA_M(	Strada	Strada	72149	STE20-rela	no|no chan	0	ENOG410X
K4PWM5	K4PWM5_l	Diaph2	Diaph2	54004	MDia3 isof	no|no chan	0	ENOG410Y
O35657	NEUR1_M	Neu1	Neu1	18010	Sialidase-1	no|no chan	0	COG4409(s
O35654	DPOD2_M	Pold2	Pold2	18972	DNA polyn	no|no chan	0	COG1311(I
G3X922	G3X922_M	Dnajc13	Dnajc13	235567	DnaJ heat s	no|no chan	0	ENOG410X
D3Z030	D3Z030_M	Carmil1	Carmil1	68732	Capping pr	no|no chan	0	ENOG410Y
G3X920	G3X920_M	Armc8	Armc8	74125	Armadillo r	no|no chan	0	COG5369(A
G3X926	G3X926_M	Rpf2	Rpf2	67239	Ribosome p	no|no chan	0	COG5106(r
Q3U832	Q3U832_M	Ccnt2	Ccnt2	72949	Cyclin-like	no|no chan	0	COG5333(e
G3X924	G3X924_M	Plvap	Plvap	84094	Plasmalem	no|no chan	0	ENOG410Y
B1ARD4	B1ARD4_M	Rad51d	Rad51d	19364	RAD51 par	no|up	0	COG0468(F
B1ARD6	SLFN9_MC	Slfn9	Slfn9	237886	Schlafen fa	no|no chan	0	ENOG410Z
A2AMY5	A2AMY5_M	Ubap2	Ubap2		Ubiquitin-c	no|no chan	0	ENOG411(
F2Z4A9	F2Z4A9_M	Smarca2	Smarca2	67155	SWI/SNF r	no|no chan	0	COG5076(C
Q7TPE5	S7A6O_MC	Slc7a6os	Slc7a6os	66432	Probable R	no|no chan	0	ENOG4111
P70268	PKN1_MO	Pkn1	Pkn1	320795	Serine/thre	no|no chan	0	ENOG410X
Q8VCH6	DHC24_M(	Dhcr24	Dhcr24	74754	Delta(24)-st	no|no chan	0	COG0277(r
Q8VCH7	RDH10_M(	Rdh10	Rdh10	98711	Retinol deh	no|no chan	0	COG1028(r
Q08024	PEBB_MO	Cbfb	Cbfb	12400	Core-bindir	no|no chan	0	ENOG410Z
Q9DCT2	NDUS3_M	Ndufs3	Ndufs3	68349	NADH deh	no|no chan	0	COG0852(N
Q9DCT1	AKCL2_M(	Akr1e2	Akr1e2	56043	1,5-anhydrc	no|no chan	0	COG0656(N

Q547N0	Q547N0_M	Brms1	Brms1	107392	BRMS1	no	no chan	0	ENOG410Y
Q9JHF5	Q9JHF5_M	Tcirg1	Tcirg1	27060	V-type prot	no	no chan	0	COG1269(A
B2RRF3	B2RRF3_M	Suco	Suco	226551	AI848100 p	no	no chan	0	ENOG4111
Q5KU39	VPS41_MO	Vps41	Vps41	218035	Vacuolar p	no	no chan	0	ENOG410X
Q9WU63	HEBP2_MO	Hebp2	Hebp2	56016	Heme-bind	no	no chan	0	ENOG410Z
B1AUB6	B1AUB6_M	Nfia	Nfia		CCAAT-bo	no	no chan	0	ENOG410Y
D3Z780	D3Z780_M	Eif2b4	Eif2b4		Translation	no	no chan	0	COG1184(F
A0A0R4J25	A0A0R4J25	Unc13d	Unc13d	70450	Unc-13 hon	no	no chan	0	ENOG410Z
A0A0R4J25	A0A0R4J25	Psmb9	Psmb9	16912	Proteasome	no	up	0	ENOG410X
P60330	ESPL1_MO	Espl1	Espl1	105988	Separin	no	no chan	0	COG5155(e
A0A0R4J25	A0A0R4J25	Xpo4	Xpo4	57258	Exportin-4	no	no chan	0	ENOG410X
A0A0R4J0L	A0A0R4J0L	Mrps35	Mrps35	232536	Mitochondi	no	no chan	0	ENOG410Z
E9Q9W4	S27A6_MO	Slc27a6	Slc27a6	225579	Long-chain	no	no chan	0	ENOG410X
A0A0R4J0L	A0A0R4J0L	Ccdc174	Ccdc174	232236	Coiled-coil	no	up	0	ENOG410Y
Q8VDF2	UHRF1_MO	Uhrf1	Uhrf1	18140	E3 ubiquiti	no	down	0	COG3440(V
A0A0R4J25	A0A0R4J25	Syncrip	Syncrip	56403	Synaptotag	no	no chan	0	ENOG410X
Q3U3A6	Q3U3A6_M	Slc9a1	Slc9a1	20544	Sodium/hy	no	no chan	0	COG0025(S
A2AUX3	A2AUX3_M	Dab2ip	Dab2ip	69601	Disabled 2	no	no chan	0	ENOG410X
E9Q4Y4	E9Q4Y4_M	Cep192	Cep192	70799	Centrosome	no	no chan	0	ENOG411C
Q9D1R2	KTI12_MO	Kti12	Kti12	100087	Protein KT	no	no chan	0	COG4088(F
Q8R3J4	MTEF3_MO	Mterf3	Mterf3	66410	Transcripti	no	down	0	ENOG410X
Q8VIB3	SIA10_MO	St3gal6	St3gal6	54613	Type 2 lact	no	down	0	ENOG410X
A0A0B4J1J	A0A0B4J1J	Ighv9-3	Ighv9-3		Immunoglo	no	no chan	0	ENOG4111
O88597	BECN1_MO	Becn1	Becn1	56208	Beclin-1	no	no chan	0	ENOG410X
A0A0B4J1J	A0A0B4J1J	Ighv10-1	Ighv10-1	16050	Immunoglo	no	no chan	0	ENOG410Y
O88593	PGRP1_MO	Pglyrp1	Pglyrp1	21946	Peptidoglyc	no	up	0	ENOG4111
P60060	SC61G_MO	Sec61g	Sec61g	20335	Protein trar	no	up	0	ENOG4111
Q6IM74	Q6IM74_M	Rtn2	Rtn2	20167	Reticulon	no	up	0	ENOG410X
F7BJB9	MORC3_M	Morc3	Morc3	338467	MORC fam	no	no chan	0	ENOG411C
Q7TT18	MCAF1_MO	Atf7ip	Atf7ip	54343	Activating	no	no chan	0	ENOG410X
A0A1L1ST2	A0A1L1ST2	Clasp2	Clasp2		CLIP associ	no	no chan	0	ENOG410Z
P60487	PLPP_MOU	Pdxp	Pdxp	57028	Chronophir	no	no chan	0	COG0647(e
P35569	IRS1_MOU	Irs1	Irs1	16367	Insulin rece	no	down	0	ENOG410Z
Q059T9	Q059T9_M	Prpf4	Prpf4	70052	PRP4 pre-m	no	no chan	0	ENOG410X
P35561	KCNJ2_MO	Kcnj2	Kcnj2	16518	Inward rect	no	no chan	0	ENOG410X
Q059T5	Q059T5_M	Mgat5	Mgat5	107895	alpha-1,6-m	no	up	0	ENOG410X
P35564	CALX_MO	Canx	Canx	12330	Calnexin	no	no chan	0	ENOG410X
P00687	AMY1_MO	Amy1	Amy1	11722	Alpha-amy	no	up	0	COG0366(h
O08528	HXK2_MO	Hk2	Hk2	15277	Hexokinase	no	no chan	0	COG5026(p
O08529	CAN2_MO	Capn2	Capn2	12334	Calpain-2 c	no	no chan	0	ENOG410X
A2ADR8	A2ADR8_M	Ppp1r8	Ppp1r8	100336	Protein phc	no	no chan	0	ENOG410X
G3X8R5	G3X8R5_M	Qrich1	Qrich1	69232	Glutamine-	no	no chan	0	ENOG410X
G3X8R0	G3X8R0_M	Reep5	Reep5	13476	Receptor ex	no	no chan	0	COG5052(r

G3X8R1	G3X8R1_M	Kptn	Kptn	70394	Kaptin	no	no chan	0 ENOG411(
Q3UZR8	Q3UZR8_M	Eif2b1	Eif2b1	209354	Translation	no	no chan	0 COG1184(t
Q7TN02	MED26_M	Med26	Med26	70625	Mediator of	no	no chan	0 COG1594(\
A0A0R4J1J	A0A0R4J1J	Pnkd	Pnkd	56695	Paroxysmal	no	no chan	0 ENOG410Y
Q6A0A9	F120A_MO	FAM120A	FAM120A	218236	Constitutiv	no	no chan	0 ENOG410X
E9QA62	LMOD3_M	Lmod3	Lmod3	320502	Leiomodin-	no	no chan	0 ENOG410Y
Q6A0A2	LAR4B_M(	Larp4b	Larp4b	217980	La-related		no	no chan	0 ENOG4111
D3YVY9	D3YVY9_M	Dscc1	Dscc1		Sister chron	no	no chan	0 ENOG411(
Q6IR21	Q6IR21_M	Aptx	Aptx	66408	Aprataxin	no	no chan	0 ENOG411(
P97384	ANX11_M(	Anxa11	Anxa11	11744	Annexin A1	no	no chan	0 ENOG410X
P97386	DNLI3_M(	Lig3	Lig3	16882	DNA ligase	no	no chan	0 COG1793(I
Q3TY95	Q3TY95_M	Akt2	Akt2	11652	non-specifi	no	no chan	0 ENOG410X
D3Z3S7	D3Z3S7_M	Mcf2l	Mcf2l		Mcf.2 trans	no	up	0 ENOG410X
E9QP62	E9QP62_M	Lims1	Lims1	110829	LIM and se	no	no chan	0 ENOG410X
B2RUS7	B2RUS7_M	Abcc8	Abcc8	20927	ATP-bindir	no	no chan	0 COG1132((
F6XC25	F6XC25_M	Cc2d1b	Cc2d1b		Coiled-coil	no	no chan	0 ENOG410X
Q5XFZ0	CR021_MOUSE	UPF0711 protein (		68046	UPF0711 pr	no	no chan	0 ENOG4112
Q91WZ8	DTBP1_M(	Dtnbp1	Dtnbp1	94245	Dysbindin	no	no chan	0 ENOG4111
A0A494B9I	A0A494B9I	Akap8l	Akap8l		A kinase an	no	no chan	0 ENOG410Y
Q9D0R9	WDR89_M	Wdr89	Wdr89	72338	WD repeat-	no	no chan	0 ENOG410Z
F8WGI9	F8WGI9_M	Vldlr	Vldlr	22359	Very low de	no	up	0 ENOG410Y
Q3U7M5	Q3U7M5_M	Abhd4	Abhd4	105501	(Lyso)-N-ac	no	no chan	0 COG0596(t
H3BJ38	H3BJ38_M(	Pde4dip	Pde4dip		Phosphodie	no	no chan	0 ENOG410Z
Q80ZZ0	Q80ZZ0_M	Ehd1	Ehd1	13660	EH-domain	no	no chan	0 ENOG410X
H3BJ30	H3BJ30_M(	Cpsf6	Cpsf6	432508	Cleavage ar	no	no chan	0 ENOG410Y
Q8CDG8	Q8CDG8_M	Lztfl1	Lztfl1	93730	Leucine zip	no	no chan	0 ENOG410Z
H3BJ37	H3BJ37_M(	Comtd1	Comtd1		Catechol-O	no	no chan	0 COG4122(I
P32037	GTR3_MO	Slc2a3	Slc2a3	20527	Solute carri	no	up	0 COG0477(s
Q9CX11	UTP23_MC	Utp23	Utp23	78581	rRNA-proc	no	no chan	0 COG1412(I
Q8CIE4	PAR10_MC	Parp10	Parp10	671535	Protein mo	no	up	0 ENOG411(
Q3TYD4	ARSG_MO	Arsg	Arsg	74008	Arylsulfata	no	no chan	0 COG3119(p
Q3TYD2	Q3TYD2_M	Nf1	Nf1	18015	Neurofibro	no	no chan	0 ENOG410X
A0A5F8MP	A0A5F8MP	Sacm1l	Sacm1l		Phosphatid	no	no chan	0 COG5329(S
A0A2I3BQ	A0A2I3BQ	Dgkh	Dgkh		Diacylglyce	no	up	0 ENOG410X
M0QWX4	M0QWX4_	Senp1	Senp1	223870	SUMO1/se	no	no chan	0 COG5160(-
Q3U2A8	SYVM_MO	Vars2	Vars2	68915	Valine--tRN	no	no chan	0 COG0525(c
Q61559	FCGRN_M	Fcgrt	Fcgrt	14132	IgG recepto	no	down	0 ENOG4111
P48024	EIF1_MOU	Eif1	Eif1	20918	Eukaryotic	no	no chan	0 COG0023((
Q3UK98	Q3UK98_M	Cbx6	Cbx6	494448	Chromobox	no	no chan	0 ENOG4112
F6S1R2	F6S1R2_M(	Ahsa2	Ahsa2		AHA1, acti	no	no chan	0 COG5580(A
Q8K3B1	FBSP1_MC	Fbxo45	Fbxo45	268882	F-box/SPR	no	no chan	0 ENOG410X
E9Q0X4	E9Q0X4_M	Col16a1	Col16a1		Collagen, ty	no	no chan	0 ENOG4111
Q6A0E5	Q6A0E5_M	mKIAA002	mKIAA002	19210	Phosphatid	no	no chan	0 ENOG410X

Q61001	LAMA5_M	Lama5	Lama5	16776	Laminin su	no|no chan	0	ENOG410
P21278	GNA11_M	Gna11	Gna11	14672	Guanine nu	no|no chan	0	ENOG410
Q3V441	Q3V441_M	Ptpn18	Ptpn18	19253	Protein tyro	no|no chan	0	COG5599(
B7XGA6	B7XGA6_M	Alpl	Alpl	11647	Alkaline ph	no|down	0	COG1785(
Q3V1Z5	Q3V1Z5_M	Rps4l	Rps4l	66184	40S ribosom	no|no chan	0	COG1471((
B7ZNS9	B7ZNS9_M	Cfd	Cfd	11537	Complemer	no|no chan	0	COG5640(
Q3V449	Q3V449_M	Nmnat1	Nmnat1	66454	Nicotinami	no|no chan	0	COG1057(
E9Q1D6	E9Q1D6_M	Ltbp2	Ltbp2		Latent tran	no|no chan	0	ENOG410
A2A7Q5	A2A7Q5_M	P3h1	P3h1	56401	procollagen	no|no chan	0	ENOG410
E9Q1D5	E9Q1D5_M	Tgfb1i1	Tgfb1i1		Transformi	no|no chan	0	ENOG410
A2A7Q9	RN19B_M	Rnf19b	Rnf19b	75234	E3 ubiquiti	no|no chan	0	ENOG410
Q5RKP4	Q5RKP4_M	Rpn1	Rpn1	103963	Dolichyl-di	no|no chan	0	ENOG410
B2RY84	B2RY84_M	Kif14	Kif14	381293	Kif14 prote	no|no chan	0	COG5059(
Q9JM73	SRF_MOU	Srf	Srf	20807	Serum resp	no|no chan	0	COG5068(
Q9D6J3	YJU2_MOU	Yju2	Yju2	72886	Splicing fac	yes|down	1	COG5134(
B9EIV8	B9EIV8_M	Slc12a7	Slc12a7	20499	Solute carri	no|no chan	0	COG0531(
Q8BN21	VRK2_MO	Vrk2	Vrk2	69922	Serine/thre	no|no chan	0	ENOG410
A0A140T8J	A0A140T8J	Hebp1	Hebp1	15199	Heme-bind	no|down	0	ENOG4111
A0A1L1SR	A0A1L1SR	Ampd3	Ampd3		AMP deam	no|no chan	0	COG1816(
Q8BHL7	C42S1_MO	Cdc42se1	Cdc42se1	57912	CDC42 smc	no|no chan	0	ENOG4112
A0A2R8W6	A0A2R8W6	Dnal4	Dnal4		Dynein, axc	no|no chan	0	ENOG4111
B9EIV6	B9EIV6_M	Pram1	Pram1	378460	PML-RAR	no|up	0	ENOG410
Q9D361	SNR48_MC	Snrnp48	Snrnp48	67797	U11/U12 sr	no|up	0	ENOG4111
A0A1B0GR	A0A1B0GR	Chd9	Chd9		DNA helicc	no|no chan	0	COG0553(
P12032	TIMP1_MC	Timp1	Timp1	21857	Metallopro	no|no chan	0	ENOG411
E9Q447	E9Q447_M	Sptan1	Sptan1		Spectrin alp	no|down	0	COG5069(
Q6P5E3	Q6P5E3_M	Sall1	Sall1	58198	Sal-like 1 (I	no|no chan	0	ENOG410
Q6P5E4	UGGG1_M	Uggt1	Uggt1	320011	UDP-gluco	no|no chan	0	ENOG410
E9Q3V9	E9Q3V9_M	Adgrl1	Adgrl1		Adhesion G	no|no chan	0	ENOG410
Q6P5E8	DGKQ_M	Dgkq	Dgkq	110524	Diacylglyce	no|no chan	0	ENOG410
Q9EQT6	SYT13_MO	Syt13	Syt13	80976	Synaptotag	no|no chan	0	ENOG411
Q9WUB3	PYGM_MC	Pygm	Pygm	19309	Glycogen p	no|up	0	COG0058(
Q9WUB0	HOIL1_MC	Rbck1	Rbck1	24105	RanBP-type	no|no chan	0	ENOG410
Q9CQ00	DMAC1_M	Dmac1	Dmac1	66928	Distal mem	no|no chan	0	ENOG410
Q7TQE6	MACOI_M	Maco1	Maco1	66146	Macoilin	no|no chan	0	ENOG410
Q9CQ06	RM24_MO	Mrpl24	Mrpl24	67707	Large ribos	no|no chan	0	COG0198(
Q9JJA7	CCNL2_M	Ccnl2	Ccnl2	56036	Cyclin-L2	no|no chan	0	COG5333(
P29595	NEDD8_M	Nedd8	Nedd8	18002	Ubiquitin-l	no|no chan	0	COG5272(
Q8BKT3	GCFC2_M	Gcfc2	Gcfc2	330361	Intron Larg	no|no chan	0	ENOG410
B7ZW94	B7ZW94_M	Fam120c	Fam120c	207375	Family with	no|no chan	0	ENOG410
Q9JHQ0	ANXA9_M	Anxa9	Anxa9	71790	Annexin A	no|no chan	0	ENOG410
Q8BKT8	HAUS7_M	Haus7	Haus7	73738	HAUS augr	no|no chan	0	ENOG4111
A0A2I3BR	A0A2I3BR	Spcs1	Spcs1	69019	Signal pept	no|no chan	0	ENOG4112

Q07456	AMBP_MC	Ambp	Ambp	11699	Protein AM	yes|up	1	ENOG410
P50543	S10AB_MO	S100a11	S100a11	20195	Protein S10	no|no chan	0	ENOG410
Q9D1P4	CHRD1_M	Chordc1	Chordc1	66917	Cysteine an	no|no chan	0	ENOG410
P50544	ACADV_M	Acadvl	Acadvl	11370	Very long-c	no|no chan	0	COG1960(
P55065	PLTP_MO	Pltp	Pltp	18830	Phospholip	no|no chan	0	ENOG410
G3X9Q3	G3X9Q3_M	Arhgap45	Arhgap45	70719	Rho GTPas	no|no chan	0	ENOG410
G3X9Q0	G3X9Q0_M	Mbnl1	Mbnl1	56758	Musclebline	no|no chan	0	ENOG410
G3X9Q1	G3X9Q1_M	Itga7	Itga7	16404	Integrin alp	no|no chan	0	ENOG410
Q6I6G8	HECW2_M	Hecw2	Hecw2	329152	E3 ubiquiti	no|no chan	0	COG5021(r
O54950	AAKG1_M	Prkag1	Prkag1	19082	5'-AMP-act	no|no chan	0	COG0517(
P51943	CCNA2_M	Ccna2	Ccna2	12428	Cyclin-A2	no|no chan	0	COG5024(
P51944	CCNF_MO	Ccnf	Ccnf	12449	Cyclin-F	no|no chan	0	COG5024(
Q8CE90	MP2K7_M	Map2k7	Map2k7	26400	Dual specif	no|no chan	0	ENOG410
Q60749	KHDR1_M	Khdrbs1	Khdrbs1	20218	KH domain	no|no chan	0	COG5176(r
P51949	MAT1_MO	Mnat1	Mnat1	17420	CDK-activ	no|no chan	0	COG5220(r
Q5NCQ5	DPH1_MO	Dph1	Dph1	116905	2-(3-amino-	no|no chan	0	COG1736(c
P37804	TAGL_MO	Tagln	Tagln	21345	Transgelin	no|down	0	COG5199(
P47713	PA24A_MC	Pla2g4a	Pla2g4a	18783	Cytosolic pl	no|no chan	0	ENOG410
Q922F6	Q922F6_MOUSE Uncharacterized protein (Frc				Uncharacte	no|no chan	0	COG0326(
A2AI57	A2AI57_M	Trpm7	Trpm7	58800	non-specifi	no|no chan	0	ENOG410
A2AI52	A2AI52_M	Usp8	Usp8	84092	ubiquitinyl	no|no chan	0	ENOG410
P49813	TMOD1_M	Tmod1	Tmod1	21916	Tropomodu	no|up	0	ENOG410
Q8BRQ9	Q8BRQ9_M	Sfxn5	Sfxn5	94282	Sidoreflexir	no|no chan	0	ENOG410
P49817	CAV1_MO	Cav1	Cav1	12389	Caveolin-1	no|no chan	0	ENOG4111
D0EW11	D0EW11_M	Mcfd2	Mcfd2	193813	Multiple co	no|no chan	0	ENOG411
P35951	LDLR_MO	Ldlr	Ldlr	16835	Low-density	no|no chan	0	ENOG410
E9Q616	E9Q616_M	Ahnak	Ahnak	66395	AHNAK nu	no|no chan	0	ENOG410
D3YY50	D3YY50_M	Cutc	Cutc		Copper hon	no|no chan	0	COG3142(c
A0A0N4SV	A0A0N4SV	Tmsb10	Tmsb10		Thymosin b	no|no chan	0	ENOG410
F5BFH0	F5BFH0_M	Clec2d	Clec2d	93694	C-type lecti	no|no chan	0	ENOG410
Q9CXI3	MOXD1_M	Moxd1	Moxd1	59012	DBH-like m	no|down	0	ENOG410
Q9CXI0	COQ5_MO	Coq5	Coq5	52064	2-methoxy-	no|no chan	0	COG2226(
P14869	RLA0_MO	Rplp0	Rplp0	11837	Large ribos	no|no chan	0	COG0244(
B7ZMY4	B7ZMY4_M	Adap1	Adap1	231821	Adap1 prot	no|no chan	0	COG5347(c
Q8BQ33	TICRR_MC	Ticrr	Ticrr	77011	Treslin	no|no chan	0	ENOG410
Q8BQ30	PPR18_MC	Ppp1r18	Ppp1r18	76448	Phostensin	no|no chan	0	ENOG410
A0A1B0GS	A0A1B0GS	Srek1	Srek1		Splicing reg	no|down	0	ENOG410
D3Z3L3	D3Z3L3_M	Trim12c	Trim12c	319236	Tripartite m	no|up	0	ENOG410
Q921I6	SH3B4_MC	Sh3bp4	Sh3bp4	98402	SH3 domain	no|no chan	0	ENOG4111
P70387	HFE_MOU	Hfe	Hfe	15216	Hereditary	no|down	0	ENOG4111
Q8C166	CPNE1_M	Cpne1	Cpne1	266692	Copine-1	no|no chan	0	ENOG410
A0A3Q4EB	A0A3Q4EB	Fam98a	Fam98a		Family with	no|no chan	0	ENOG4111
Q99KU0	VMP1_MO	Vmp1	Vmp1	75909	Vacuole me	no|up	0	ENOG410

P01654	KV3A1_MOUSE Ig kappa chain V-III region F				Ig kappa ch	no|up	0	ENOG4111
Q921I9	EXOS4_M(	Exosc4	Exosc4	109075	Exosome cc	no|no chan	0	COG0689(S
P06684	CO5_MOU	C5	C5	15139	Complemer	no|no chan	0	ENOG410)
Q07832	PLK1_MO	Plk1	Plk1	18817	Serine/thre	no|no chan	0	ENOG410)
P08226	APOE_MC	Apoe	Apoe	11816	Apolipopro	no|up	0	ENOG4111
P08228	SODC_MC	Sod1	Sod1	20655	Superoxide	no|no chan	0	COG2032(N
Q8CDX7	Q8CDX7_M	Coa8	Coa8	68020	Cytochrom	no|no chan	0	ENOG4111
Q80ZE3	SIG10_MO	Siglec10	Siglec10	243958	Sialic acid-l	yes|down	1	ENOG410Y
A0A1D5RM	A0A1D5RM	Alms1	Alms1	236266	ALMS1, cer	no|no chan	0	ENOG4111
Q9JJY4	DDX20_M	Ddx20	Ddx20	53975	Probable A	no|no chan	0	COG0513(T
A2RTH5	A2RTH5_M	Lcmt1	Lcmt1	30949	Leucine car	no|no chan	0	ENOG410Y
A0A8Z1SL5	A0A8Z1SL5	Renbp	Renbp	19703	N-acylgluc	no|no chan	0	COG2942(r
A5D6P2	A5D6P2_M	Pard3	Pard3	93742	Par-3 (Parti	no|no chan	0	ENOG410Z
A5D6P3	A5D6P3_M	Abcc1	Abcc1	17250	Multidrug r	no|no chan	0	COG1132(c
A5D6P6	A5D6P6_M	Twist2	Twist2	13345	Twist basic	no|no chan	0	ENOG4111
E9QL53	E9QL53_M	Cit	Cit	12704	Citron Rho-	no|no chan	0	ENOG410)
Q3UZ58	Q3UZ58_M	Fdxr	Fdxr	14149	NADPH:ad	no|no chan	0	COG0493(r
P59222	SREC2_MC	Scarf2	Scarf2	224024	Scavenger r	no|no chan	0	ENOG410Y
P54818	GALC_MO	Galc	Galc	14420	Galactocer	no|no chan	0	ENOG410)
A0A1L1SQ	A0A1L1SQ	Zbtb44	Zbtb44		Zinc finger	no|no chan	0	COG5048(A
Q62371	DDR2_MC	Ddr2	Ddr2	18214	Discoidin d	no|no chan	0	ENOG410)
Q8CI78	RMND1_M	Rmnd1	Rmnd1	66084	Required fc	no|no chan	0	COG1723(F
Q4VBE8	WDR18_M	Wdr18	Wdr18	216156	WD repeat-	no|no chan	0	COG2319(F
Q5SXE4	Q5SXE4_M	Ksr1	Ksr1		Kinase sup	no|no chan	0	ENOG410Y
Q9QZS3	NUMB_M(	Numb	Numb	18222	Protein nur	no|no chan	0	ENOG410)
Q8CI70	LRC20_MC	Lrrc20	Lrrc20	216011	Leucine-ric	no|up	0	ENOG4111
Q9QZS8	SH2D3_M(	Sh2d3c	Sh2d3c	27387	SH2 domai	no|no chan	0	ENOG410)
Q8CI75	DI3L2_MO	Dis3l2	Dis3l2	208718	DIS3-like e	no|no chan	0	COG0557(F
A0A0G2JG	A0A0G2JG	Krit1	Krit1		KRIT1, ank	no|no chan	0	ENOG410Z
A0A023J5Z	A0A023J5Z	ND1	ND1		NADH-ubi	no|no chan	0	COG1005(N
O09005	DEGS1_M(	Degs1	Degs1	13244	Sphingolipi	no|no chan	0	ENOG410)
Q9ERL9	GCYA1_M	Gucy1a1	Gucy1a1	60596	Guanylate	no|no chan	0	COG2114(C
Q61176	ARGI1_MC	Arg1	Arg1	11846	Arginase-1	no|no chan	0	COG0010(E
Q61171	PRDX2_M	Prdx2	Prdx2	21672	Peroxiredo	no|no chan	0	COG0450(c
A0A077K84	A0A077K84	Alg11	Alg11	207958	GDP-Man:	no|no chan	0	COG0438(I
Q62159	RHOC_MC	Rhoc	Rhoc	11853	Rho-related	no|no chan	0	COG1100(T
Q62158	TRI27_MO	Trim27	Trim27	19720	Zinc finger	no|no chan	0	ENOG410Z
P45377	ALD2_MO	Akr1b8	Akr1b8	14187	Aldose redu	no|up	0	COG0656(C
P45376	ALDR_MC	Akr1b1	Akr1b1	11677	Aldo-keto r	no|no chan	0	COG0656(r
A0A8T9ITI	A0A8T9ITI	ECM1	ECM1		Extracellul	yes|up	1	ENOG4111
B7ZWA9	B7ZWA9_M	Zbtb7a	Zbtb7a	16969	Zbtb7a pro	no|no chan	0	COG5048(F
A0A0G2JD	A0A0G2JD	Map4	Map4		Microtubul	no|down	0	ENOG4111
C6EQI4	C6EQI4_MOUSE ASL1/Clec4a2 fusion protei				ASL1/Clec4	no|no chan	0	ENOG410Z

E9QMD3	E9QMD3_I	Zfhx3	Zfhx3	11906	Zinc finger	no	down	0	ENOG410X
G3XA25	G3XA25_M	Acat2	Acat2		acetyl-CoA	no	no chan	0	COG0183(c
Q80XN0	BDH_MOU	Bdh1	Bdh1	71911	D-beta-hyd	no	no chan	0	ENOG410Y
A3KCG1	A3KCG1_M	Orai1	Orai1	109305	Calcium rel	no	no chan	0	ENOG4110
A0A0J9YUJ	A0A0J9YUJ	Gsn	Gsn		Gelsolin	no	up	0	ENOG410X
Q3UVI9	Q3UVI9_M	Abcf2	Abcf2	27407	ABC transp	no	no chan	0	COG0488(M
Q8C5H8	NADK2_M	Nadk2	Nadk2	68646	NAD kinas	no	no chan	0	ENOG410X
A2A4P4	A2A4P4_M	Gm27029	Gm27029		Predicted g	no	no chan	0	COG2872(s
A2A4P0	DHX8_MC	Dhx8	Dhx8	217207	ATP-depen	no	no chan	0	COG1643(I
Q8K411	PREP_MO	Pitrm1	Pitrm1	69617	Presequenc	no	no chan	0	COG1026(p
H3BLL2	H3BLL2_M	Atpaf1	Atpaf1	230649	ATP syntha	no	no chan	0	ENOG4111
Q8R3Y8	I2BP1_MO	Irf2bp1	Irf2bp1	272359	Interferon r	no	no chan	0	ENOG410Z
A0A494BA	A0A494BA	Rela	Rela		V-rel reticu	no	no chan	0	ENOG410X
Q78J03	MSRB2_M(	Msrb2	Msrb2	76467	Methionine	no	no chan	0	COG0229()
A0A2R8VK	A0A2R8VK	Cd47	Cd47		Leukocyte s	no	no chan	0	ENOG4111
Q9DBU5	RNF6_MO	Rnf6	Rnf6	74132	E3 ubiquiti	no	down	0	ENOG4112
A0A668KL	A0A668KL	Capn3	Capn3		Calpain-3	no	up	0	ENOG410X
Q8JZY4	MRPP3_M(	Prorp	Prorp	66132	Mitochond	no	no chan	0	ENOG4111
Q9D173	TOM7_MC	Tomm7	Tomm7	66169	Mitochond	no	no chan	0	ENOG4112
Q91ZM2	SH2B1_MC	Sh2b1	Sh2b1	20399	SH2B adap	no	no chan	0	ENOG4110
Q9DBU8	Q9DBU8_M	Ap3m1	Ap3m1	55946	AP-3 compl	no	no chan	0	ENOG410X
Q9EQY0	ERN1_MO	Ern1	Ern1	78943	Serine/thre	no	no chan	0	COG0515(M
A2CG35	A2CG35_M	Rab12	Rab12		RAB12, me	no	no chan	0	ENOG410X
A1L353	A1L353_M(	Tgfbi	Tgfbi	21810	Transformi	no	no chan	0	COG2335(I
Q8K1C9	LRC41_MC	Lrrc41	Lrrc41	230654	Leucine-ric	no	no chan	0	ENOG410Y
A0A075F5C	A0A075F5C	Hsf1	Hsf1	15499	Heat shock	no	no chan	0	COG5169(T
E9Q4D5	E9Q4D5_M	Rem2	Rem2	140743	Rad and ge	no	no chan	0	COG1100(u
Q3UNW1	Q3UNW1_	1810034E14	1810034E14	66503	Uncharacte	no	up	0	
Q8K1C0	ANGE2_M	Angel2	Angel2	52477	Protein ang	no	no chan	0	COG5239(S
Q3TTL0	CC038_MOUSE	Uncharacterized p		67609	Uncharacte	no	no chan	0	ENOG4111
Q4FJK0	Q4FJK0_M	Decr1	Decr1	67460	2,4-dienoyl-	no	no chan	0	COG1028(A
Q9D281	NXP20_M(	Fam114a1	Fam114a1	68303	Protein No	no	no chan	0	ENOG4110
Q9CPN9	Q9CPN9_M	Prss3b	Prss3b	67373	Serine prot	no	up	0	COG5640(M
P04444	HBBZ_MO	Hbb-bh1	Hbb-bh1	15132	Hemoglobi	no	no chan	0	COG1018(S
Q8K2C7	OS9_MOU	Os9	Os9	216440	Protein OS-	no	no chan	0	ENOG410X
Q9D289	TPC6B_MC	Trappc6b	Trappc6b	78232	Trafficking	no	no chan	0	ENOG410Y
A0A0B4J1C	FCG3A_M(	Fcgr4	Fcgr4	246256	Low affinit	no	up	0	ENOG4111
Q3U4B1	Q3U4B1_M	Ssbp4	Ssbp4	76900	Single stran	no	no chan	0	ENOG410X
Q4FJK5	Q4FJK5_M	Casp9	Casp9	12371	Casp9 prote	no	no chan	0	ENOG410Z
A0A3Q4EC	A0A3Q4EC	Gfer	Gfer		Sulfhydryl c	no	no chan	0	COG5054(t
A2A5R2	BIG2_MOU	Arfgef2	Arfgef2	99371	Brefeldin A	no	no chan	0	COG5307(r
Q3TC47	Q3TC47_M	Bin3	Bin3	57784	BAR domai	no	no chan	0	ENOG4111
Q3TC46	PATL1_MC	Patl1	Patl1	225929	Protein PA	no	no chan	0	ENOG4110

Q99JN2	KLH22_M(	Klhl22	Klhl22	224023	Kelch-like	no	no chan	0	ENOG410
A6H6B9	A6H6B9_M	Zbtb17	Zbtb17	22642	Zinc finger	no	down	0	COG5048(
A0A3B0IT(	A0A3B0IT(	Adia	Adia	12260	Adiponecti	no	up	0	ENOG4111
P63094	GNAS2_M	Gnas	Gnas	14683	Guanine n	no	no chan	0	ENOG410
Q3UG22	Q3UG22_M	Tgfbr2	Tgfbr2	21813	TGF-beta r	no	down	0	ENOG410
E0CXI7	E0CXI7_M	Ephx4	Ephx4		Epoxide hy	no	no chan	0	ENOG410
A0A1L1ST	A0A1L1ST	Nos2	Nos2		Nitric oxid	no	up	0	COG0369(
Q9QXM0	ABHD2_M	Abhd2	Abhd2	54608	Monoacylgl	no	no chan	0	COG0429(
Q8K2C8	GPAT4_M(	Gpat4	Gpat4	102247	Glycerol-3-	no	no chan	0	COG0204(
Q9D2U5	NAA38_M(	Naa38	Naa38	78304	N-alpha-ac	no	no chan	0	ENOG410
Q8CII2	CD123_MC	Cdc123	Cdc123	98828	Translation	no	no chan	0	ENOG410
Q8CEC6	PPWD1_M	Ppwd1	Ppwd1	238831	Peptidylpro	no	no chan	0	ENOG410
Q8CCF0	PRP31_MC	Prpf31	Prpf31	68988	U4/U6 sma	no	no chan	0	COG1498(
Q9Z1R3	APOM_MC	Apom	Apom	55938	Apolipopro	no	no chan	0	ENOG4111
Q8JZR4	EAA5_MO	Slc1a7	Slc1a7	242607	Excitatory	no	no chan	0	COG1301(
P54103	DNJC2_M(	Dnajc2	Dnajc2	22791	DnaJ homo	no	no chan	0	COG5269(
A0A1R3UC	A0A1R3UC	Klna12	Klna12		Kallikrein	no	up	0	COG5640(
Q3UTI7	Q3UTI7_M	Pin1	Pin1	23988	Peptidyl-pr	no	no chan	0	COG0760(
P13516	ACOD1_M	Scd1	Scd1	20249	Acyl-CoA d	no	down	0	COG1398(
Q3TFZ4	Q3TFZ4_M	Zfp7	Zfp7	223669	Zinc finger	no	down	0	COG5048(
O70579	PM34_MO	Slc25a17	Slc25a17	20524	Peroxisoma	no	no chan	0	ENOG410
Q3UGQ2	Q3UGQ2_	Pias4	Pias4	59004	E3 SUMO-	no	no chan	0	ENOG410
Q3TSA1	Q3TSA1_M	Cdyl	Cdyl	12593	Chromodor	no	no chan	0	COG1024(
P63325	RS10_MOU	Rps10	Rps10	67097	Small ribos	no	no chan	0	COG5045(
O70570	PIGR_MO	Pigr	Pigr	18703	Polymeric i	no	up	0	ENOG4112
O70571	PDK4_MO	Pdk4	Pdk4	27273	[Pyruvate d	no	up	0	COG0642(
O70572	NSMA_MC	Smpd2	Smpd2	20598	Sphingomy	no	no chan	0	ENOG410
Q91ZF0	DJC24_MO	Dnajc24	Dnajc24	99349	DnaJ homo	no	no chan	0	COG0484(
Q9CRW3	CB076_MOUSE	UPF0538 protein (		73103	UPF0538 p	no	no chan	0	ENOG4111
F8WJA0	F8WJA0_M	Ddx24	Ddx24	27225	ATP-depen	no	no chan	0	ENOG410
B2RX13	B2RX13_M	Pxdn	Pxdn	69675	Peroxidasin	no	no chan	0	ENOG410
Q9D824	FIP1_MOU	Fip1l1	Fip1l1	66899	Pre-mRNA	no	no chan	0	COG5213(
Q7TMX5	SHQ1_MO	Shq1	Shq1	72171	Protein SH	no	no chan	0	ENOG410
Q9D820	PRXD1_M	Prorsd1	Prorsd1	67939	Prolyl-tRN	no	no chan	0	COG3760(
Q69ZS0	PZRN3_M(	Pdzrn3	Pdzrn3	55983	E3 ubiquiti	no	no chan	0	ENOG4111
Q3TXE6	Q3TXE6_M	Rnf4	Rnf4	19822	RING-type	no	down	0	ENOG410
Q9D828	Q9D828_M	Tfg	Tfg	21787	PB1 domai	no	no chan	0	ENOG410
A0A140LH	A0A140LH	Nr1h2	Nr1h2		Nuclear rec	no	no chan	0	ENOG410
Q3TS44	Q3TS44_M	Psma1	Psma1	26440	Proteasome	no	no chan	0	COG0638(
A0A140LH	A0A140LH	Sirt2	Sirt2		NAD-deper	no	no chan	0	COG0846(
Q504M2	PDP2_MO	Pdp2	Pdp2	382051	[Pyruvate d	no	no chan	0	COG0631(-
Q3UCF2	Q3UCF2_M	Cdc5l	Cdc5l	71702	Cell divisio	no	no chan	0	COG5147(
Q3UCF3	Q3UCF3_M	AA467197	AA467197	433470	Normal mu	no	up	0	ENOG410

Q6ZQ84	Q6ZQ84_M	mKIAA061	mKIAA061	26554	MKIAA061	no|no chan	0	COG5647(c
B9EJ46	B9EJ46_MC	Tlr1	Tlr1	21897	Toll-like rec	no|no chan	0	COG4886(c
P59764	DOCK4_M	Dock4	Dock4	238130	Dedicator c	no|no chan	0	ENOG410X
Q9CPQ8	ATP5L_MC	Atp5mg	Atp5mg	27425	ATP syntha	no|no chan	0	ENOG4111
A6PW90	A6PW90_M	Pramel12	Pramel12		PRAME lik	no|no chan	0	ENOG4111
Q9R1T2	SAE1_MOU	Sae1	Sae1	56459	SUMO-acti	no|no chan	0	COG0476(T
Q9CPQ3	TOM22_M	Tomm22	Tomm22	223696	Mitochondr	no|no chan	0	ENOG4112
Q9CPQ1	COX6C_M	Cox6c	Cox6c	12864	Cytochrom	no|no chan	0	ENOG4112
Q811C3	Q811C3_MOUSE Imunoglobulin gamma-3 kap				Imunoglobu	no|no chan	0	ENOG4111
F8WJ40	F8WJ40_M	Kmt2b	Kmt2b	75410	Histone-lys	no|no chan	0	COG2940(S
A0A0G2JG	A0A0G2JG	Atp1a3	Atp1a3	232975	Sodium/po	no|no chan	0	COG0474(A
F6M3K1	F6M3K1_M	Zfp703	Zfp703	353310	Zeppo1	no|no chan	0	ENOG4111
O55143	AT2A2_MC	Atp2a2	Atp2a2	11938	Sarcoplasm	no|no chan	0	COG0474(P
E9QNV2	E9QNV2_M	Med23	Med23	70208	Mediator of	no|no chan	0	ENOG410Z
Q8C0N2	GPAT3_M	Gpat3	Gpat3	231510	Glycerol-3-	no|no chan	0	COG0204(C
Q3UUH0	Q3UUH0_M	Asl	Asl	109900	Argininosu	no|no chan	0	COG0165(c
Q8VBV7	CSN8_MOU	Cops8	Cops8	108679	COP9 signa	no|no chan	0	ENOG410X
P51682	CCR5_MOU	Ccr5	Ccr5	12774	C-C chemol	no|up	0	ENOG410X
O54890	ITB3_MOU	Itgb3	Itgb3	16416	Integrin be	no|no chan	0	ENOG410X
O35671	ITBP1_MO	Itgb1bp1	Itgb1bp1	16413	Integrin be	no|no chan	0	ENOG4111
Q99KN2	CIAO1_MC	Ciao1	Ciao1	26371	Probable cy	no|no chan	0	ENOG410X
Q8BI66	ZN526_MC	Znf526	Znf526	210172	Zinc finger	no|no chan	0	COG5048(F
Q9JLI2	Q9JLI2_MC	Col5a3	Col5a3	53867	Collagen ty	no|no chan	0	ENOG410X
Q548F2	Q548F2_M	Gda	Gda	14544	Guanine de	no|no chan	0	COG0402(c
Q8VCN5	CGL_MOU	Cth	Cth	107869	Cystathioni	no|up	0	COG0626(c
P31428	DPEP1_MC	Dpep1	Dpep1	13479	Dipeptidas	no|no chan	0	COG2355(E
Q32P49	Q32P49_M	Mier1	Mier1	71148	Mesoderm i	no|no chan	0	ENOG410Y
G4XVC4	G4XVC4_M	Fam110a	Fam110a	73847	Serine threc	yes|up	1	ENOG4111
S4R2J9	S4R2J9_MC	Prrc2c	Prrc2c	226562	Proline-rich	no|no chan	0	ENOG4111
P70245	EBP_MOU	Ebp	Ebp	13595	3-beta-hydr	no|no chan	0	ENOG411C
Q3TAR6	Q3TAR6_M	Vrk3	Vrk3	101568	Protein kin	no|no chan	0	ENOG410X
A2BFB0	A2BFB0_M	Mlx	Mlx	21428	MAX-like p	no|no chan	0	ENOG410Z
B2RRX1	B2RRX1_M	Actb	Actb	11461	Actin, cytop	no|no chan	0	COG5277(A
Q61781	K1C14_MC	Krt14	Krt14	16664	Keratin, typ	no|up	0	ENOG410Y
P07742	RIR1_MOU	Rrm1	Rrm1	20133	Ribonucleo	no|no chan	0	COG0209(F
Q8BNY6	NCS1_MOU	Ncs1	Ncs1	14299	Neuronal c	no|no chan	0	COG5126(V
Q00690	LYAM2_M	Sele	Sele	20339	E-selectin	no|no chan	0	ENOG410X
L7N209	L7N209_M	Hbs1l	Hbs1l	56422	Hbs1-like (S	no|no chan	0	ENOG410X
Q3U3C7	Q3U3C7_M	Taf5l	Taf5l	102162	TFIID subu	no|no chan	0	ENOG410X
Q8VDD5	MYH9_MC	Myh9	Myh9	17886	Myosin-9	no|no chan	0	COG5022(T
Q04592	PCSK5_MC	Pcsk5	Pcsk5	18552	Proprotein	no|no chan	0	COG4935(E
Q3U3C9	GSE1_MOU	Gse1	Gse1	382034	Genetic sup	no|no chan	0	ENOG410Z
Q8VDD8	WASH1_M	Washc1	Washc1	68767	WASH com	no|no chan	0	ENOG410Y

A0A0R4J0N	A0A0R4J0N	Pdcd2	Pdcd2	18567	Programme	no | no chan	0	ENOG410X
Q3T981	Q3T981_M	Cox10	Cox10	70383	Uncharacte	no | down	0	COG0109(C
Q8R3H7	HS2ST_MC	Hs2st1	Hs2st1	23908	Heparan su	no | no chan	0	ENOG410X
Q8R5I6	GSTM4_M	Gstm4	Gstm4	14865	Glutathion	no | up	0	ENOG4110
Q8C3E0	Q8C3E0_M	Zfp384	Zfp384	269800	C2H2-type	no | down	0	COG5048(I
P04202	TGFB1_M(	Tgfb1	Tgfb1	21803	Transformi	no | no chan	0	ENOG410X
A0A0R4J0N	A0A0R4J0N	Mtus1	Mtus1	102103	Mitochondı	no | up	0	ENOG4111
Q9D0M3	CY1_MOU	Cyc1	Cyc1	66445	Cytochrom	no | no chan	0	COG2857(c
A0A338P6F	A0A338P6F	Lztr1	Lztr1		Leucine-zip	no | no chan	0	ENOG410Y
A0A338P6F	A0A338P6F	Acap2	Acap2	78618	Arf-GAP w	no | no chan	0	COG5347(I
A0A0A6YW	A0A0A6YW	Usp19	Usp19	71472	ubiquitinyl	no | no chan	0	COG5560(r
Q8C7D2	CRBN_MC	Crbn	Crbn	58799	Protein cere	no | no chan	0	ENOG410X
B9EHS4	B9EHS4_M	Tecpr2	Tecpr2	104859	Tectonin be	no | no chan	0	ENOG410Z
Q6P3B2	UBAD1_M	Ubald1	Ubald1	207740	UBA-like d	no | no chan	0	ENOG4111
A0A571BEI	A0A571BEI	Sh3d19	Sh3d19		SH3 domai	no | no chan	0	ENOG4111
A0A087WQ	A0A087WQ	Ly96	Ly96	17087	Lymphocyt	no | no chan	0	ENOG4111
A0A0B4J1F	A0A0B4J1F	Igkv1-135	Igkv1-135	243420	Immunoglo	no | no chan	0	ENOG410Z
A0A0B4J1F	A0A0B4J1F	Igkv2-137	Igkv2-137	692187	Immunoglo	no | up	0	ENOG410Z
A0A286YE	A0A286YE	Spast	Spast	50850	Spastin	no | no chan	0	COG0464(N
Q7TME2	SPAG5_M(	Spag5	Spag5	54141	Sperm-asso	yes | down	1	ENOG410Y
Q7TT37	ELP1_MOI	Elp1	Elp1	230233	Elongator c	no | no chan	0	COG5290(p
P19228	CASA1_M(	Csn1s1	Csn1s1	12990	Alpha-S1-c	no | up	0	ENOG4111
P35235	PTN11_M(	Ptpn11	Ptpn11	19247	Tyrosine-pr	no | no chan	0	COG5599(A
Q3ULT0	Q3ULT0_M	Orc3	Orc3	50793	Origin reco	no | no chan	0	ENOG410X
Q922U1	PRPF3_MC	Prpf3	Prpf3	70767	U4/U6 sma	no | no chan	0	ENOG410X
E9Q6A6	E9Q6A6_M	Col6a6	Col6a6		Collagen, ty	no | down	0	ENOG410Y
E9Q6A7	E9Q6A7_M	Bptf	Bptf	207165	Bromodom	no | no chan	0	COG5076(c
Q8R046	Q8R046_M	Ms4a4b	Ms4a4b	60361	Ms4a4b pro	no | no chan	0	ENOG4112
Q3UGN1	Q3UGN1_	Gng7	Gng7	14708	Guanine nu	no | up	0	ENOG4112
Q8BHS6	ARMX3_M	Armcx3	Armcx3	71703	Armadillo r	no | no chan	0	ENOG4112
Q9CQN3	TOM6_MC	Tomm6	Tomm6	66119	Mitochondı	no | no chan	0	ENOG4111
A0A6G7NF	A0A6G7NF	Vkorc1	Vkorc1	27973	vitamin-K-	no | no chan	0	ENOG4111
A0A1L1STF	A0A1L1STF	Ilf3	Ilf3	16201	Interleukin	no | no chan	0	ENOG410X
O08547	SC22B_MO	Sec22b	Sec22b	20333	Vesicle-traf	no | no chan	0	COG5143(I
A0A1L1STF	A0A1L1STF	Idh3a	Idh3a		Isocitrate d	no | no chan	0	COG0473(z
A0A1Y7VM	A0A1Y7VM	Med10	Med10		Mediator of	no | no chan	0	ENOG4111
G3X8P6	G3X8P6_M	Txnrd3	Txnrd3		thioredoxin	no | no chan	0	COG1249(N
G3X8P9	G3X8P9_M	Aox1	Aox1	11761	aldehyde ox	no | no chan	0	COG4630(v
A0A494BA	A0A494BA	Slc25a45	Slc25a45		Solute carri	no | up	0	ENOG410X
Q497Z1	Q497Z1_M	Trim47	Trim47	217333	Trim47 pro	no | no chan	0	ENOG4111
Q3TQG9	Q3TQG9_	Zfp607a	Zfp607a	545938	Zinc finger	no | no chan	0	COG5048(f
Q6PCN7	HLTF_MO	Hltf	Hltf	20585	Helicase-lik	no | no chan	0	ENOG410X
A0A0R4J1F	A0A0R4J1F	Foxp1	Foxp1	108655	Forkhead b	no | no chan	0	COG5025(I

D3YVW2	D3YVW2_I	Golim4	Golim4	73124	Golgi integ	no | no chan	0	ENOG410
P52624	UPP1_MOU	Upp1	Upp1	22271	Uridine pho	no | up	0	COG2820(U
P27546	MAP4_MO	Map4	Map4	17758	Microtubul	no | no chan	0	ENOG4111
P14069	S10A6_MO	S100a6	S100a6	20200	Protein S10	no | up	0	ENOG410Z
B2RUM8	B2RUM8_I	Ddx18	Ddx18	66942	ATP-depen	no | no chan	0	COG0513(S
P10649	GSTM1_M	Gstm1	Gstm1	14862	Glutathion	no | no chan	0	ENOG411C
A0A075B5I	A0A075B5I	Igkv2-112	Igkv2-112		Immunoglo	no | down	0	ENOG410Z
E9QP49	E9QP49_M	Ehbp1l1	Ehbp1l1	114601	EH domain	no | no chan	0	COG5069(e
A0A075B5I	A0A075B5I	Igkv9-124	Igkv9-124		Immunoglo	no | up	0	ENOG410Y
Q8R121	ZPI_MOUS	Serpina10	Serpina10	217847	Protein Z-d	no | up	0	COG4826(I
P61804	DAD1_MC	Dad1	Dad1	13135	Dolichyl-di	no | no chan	0	ENOG4111
D3Z3Q3	D3Z3Q3_M	Smtn	Smtn		Smoothelin	no | no chan	0	COG5069(M
E9QP46	E9QP46_M	Syne2	Syne2		Spectrin rep	no | no chan	0	COG5069(T
E9QP44	E9QP44_M	Arap2	Arap2	212285	ArfGAP wi	no | no chan	0	COG5347(p
Q9D0P8	IFT27_MO	Ift27	Ift27	67042	Intraflagell	no | no chan	0	ENOG4111
Q8C9M6	Q8C9M6_MOUSE	Large ribosoma		66845	Large ribos	no | no chan	0	COG0267(S
Q8K352	SASH3_MC	Sash3	Sash3	74131	SAM and SI	no | up	0	ENOG410Y
E0CY81	E0CY81_M	Gm44505	Gm44505		Predicted re	no | no chan	0	COG5048(I
Q8R124	KLH36_MC	Klhl36	Klhl36	234796	Kelch-like p	yes | up	1	ENOG410
Q6ZWQ7	SPCS3_MO	Spcs3	Spcs3	76687	Signal pept	no | no chan	0	ENOG411C
Q505Q3	Q505Q3_M	Fuca2	Fuca2	66848	Alpha-L-fu	no | up	0	COG3669(-
Q810L3	CHFR_MO	Chfr	Chfr	231600	E3 ubiquiti	no | no chan	0	COG5243(I
Q80YC2	Q80YC2_M	Hsp90ab1	Hsp90ab1	15516	Hsp90ab1 p	no | up	0	COG0326(-
B8JK39	ITA9_MOU	Itga9	Itga9	104099	Integrin alp	no | up	0	ENOG410
Q80ZX0	Q80ZX0_M	Sec24b	Sec24b	99683	SEC24 hom	no | no chan	0	COG5028(I
M0QWZ1	M0QWZ1_	Fam193a	Fam193a		Family with	no | no chan	0	ENOG410Z
B2RS43	B2RS43_M	D17H6S53I	D17H6S53I	114585	DNA segme	no | no chan	0	ENOG4111
B2RS41	B2RS41_M	Aldh5a1	Aldh5a1	214579	Succinate-s	no | no chan	0	COG1012(c
Q8CIG3	KDM1B_M	Kdm1b	Kdm1b	218214	Lysine-spec	no | no chan	0	ENOG410
Q9CX30	YIF1B_MO	Yif1b	Yif1b	77254	Protein YIF	no | no chan	0	COG5197(y
A0A5F8MP	A0A5F8MP	Prdm11	Prdm11		PR domain	no | no chan	0	
F2Z3U3	F2Z3U3_M	Raph1	Raph1	77300	Ras associa	no | no chan	0	ENOG410
A0A2R8VH	A0A2R8VH	Zfp385a	Zfp385a	29813	Zinc finger	no | no chan	0	ENOG410Y
Q7TSQ8	PDPR_MO	Pdpr	Pdpr	319518	Pyruvate de	no | no chan	0	COG0404(T
A0A087WN	A0A087WN	Srcap	Srcap		Snf2-relatec	no | no chan	0	COG0553(I
Q9JMB0	GKAP1_M	Gkap1	Gkap1	56278	G kinase-ar	no | no chan	0	ENOG410Y
Q0PD10	Q0PD10_M	Rab43	Rab43	69834	Rab41	no | up	0	ENOG410
E0CZ31	E0CZ31_M	Pias3	Pias3		Protein inh	no | no chan	0	ENOG410
Q5DTL8	Q5DTL8_I	Sel1l	Sel1l	20338	MKIAA413	no | no chan	0	COG0790(S
E9PVN6	E9PVN6_I	S2bpcox16	S2bpcox16	105940408	Cytochrom	no | no chan	0	ENOG4112
P97503	NKX32_M	Nkx3-2	Nkx3-2	12020	Homeobox	no | no chan	0	ENOG410
D3Z5G7	D3Z5G7_I	Ces1b	Ces1b	382044	Carboxylic	no | up	0	COG2272(C
E9QKG3	E9QKG3_I	Trmt11	Trmt11	73681	tRNA (guai	no | no chan	0	COG1041(r

E9QKG2	E9QKG2_M	Katna1	Katna1		Katanin p6	no|no chan	0	ENOG410X
E9QKG8	E9QKG8_M	Hjurp	Hjurp	381280	Holliday ju	no|down	0	ENOG4111
Q8C586	Q8C586_M	Rbpms	Rbpms	19663	RRM doma	no|no chan	0	ENOG410X
B7ZNQ8	B7ZNQ8_M	Calm4	Calm4	80796	Calm4 prot	no|up	0	COG5126(C
P26638	SYSC_MOU	Sars1	Sars1	20226	Serine--tRN	no|no chan	0	COG0172(C
A0A0A1HA	A0A0A1HA	Marcks	Marcks	17118	Myristoylat	no|no chan	0	ENOG4111
Q3UFM5	NOM1_MO	Nom1	Nom1	433864	Nucleolar M	no|no chan	0	ENOG410X
A1L3P4	SL9A6_MO	Slc9a6	Slc9a6	236794	Sodium/hy	no|no chan	0	COG0025(r
Q6ZPY2	SMG5_MO	Smg5	Smg5	229512	Nonsense-n	no|no chan	0	ENOG410X
E9Q1F6	E9Q1F6_M	Tnn	Tnn		Tenascin N	no|no chan	0	ENOG410Z
P0CW03	LY6C2_MC	Ly6c2	Ly6c2	100041546	Lymphocyt	no|up	0	ENOG4111
Q9JM58	CRLF1_MC	Crlf1	Crlf1	12931	Cytokine re	no|no chan	0	ENOG410Z
Q63844	MK03_MO	Mapk3	Mapk3	26417	Mitogen-ac	no|no chan	0	ENOG410X
Q9JJ28	FLII_MOU	Flii	Flii	14248	Protein flig	no|no chan	0	COG4886(C
Q6ZPY5	ZN507_MC	Znf507	Znf507	668501	Zinc finger	no|no chan	0	COG5048(T
P19246	NFH_MOU	Nefh	Nefh	380684	Neurofilam	no|no chan	0	ENOG410X
B2RQQ7	B2RQQ7_M	Cdc42bpb	Cdc42bpb	217866	non-specifi	no|no chan	0	COG0515(F
B2RQQ5	B2RQQ5_M	Map1b	Map1b	17755	Microtubul	no|no chan	0	ENOG410X
Q8CAB8	CAST2_MC	Castor2	Castor2	80909	Cytosolic a	no|no chan	0	ENOG4111
Q9CS42	PRPS2_MC	Prps2	Prps2	110639	Ribose-pho	no|no chan	0	COG0462(F
B2RQQ8	B2RQQ8_M	Col4a2	Col4a2	12827	Collagen, ty	no|down	0	ENOG410X
F8WHB7	F8WHB7_M	Clec10a	Clec10a	17312	C-type lecti	no|down	0	ENOG410X
P97351	RS3A_MOU	Rps3a	Rps3a	20091	Small ribos	no|no chan	0	COG1890(F
A0A171KX	A0A171KX	Prmt1	Prmt1		type I prote	no|no chan	0	ENOG410X
A0A494B93	A0A494B93	Slc37a1	Slc37a1		Solute carri	no|up	0	COG2271(C
O35887	CALU_MO	Calu	Calu	12321	Calumenin	no|no chan	0	ENOG410X
A0A087WR	A0A087WR	Cyp11a1	Cyp11a1		Cholesterol	no|up	0	COG2124(T
Q6P5G3	MBTD1_M	Mbtd1	Mbtd1	103537	MBT doma	no|no chan	0	ENOG410Z
E9PWP6	E9PWP6_M	Rims1	Rims1		Regulating	yes|down	1	ENOG410Z
Q6P5G0	MK04_MO	Mapk4	Mapk4	225724	Mitogen-ac	no|up	0	ENOG410X
Q0VF80	Q0VF80_M	Dusp4	Dusp4	319520	Dual specif	no|no chan	0	COG2453(C
Q9JJG9	NOA1_MC	Noa1	Noa1	56412	Nitric oxide	no|no chan	0	COG1161(F
Q9CQ69	QCR8_MO	Uqcrq	Uqcrq	22272	Cytochrom	no|no chan	0	ENOG4112
Q9CYK1	SYWM_MC	Wars2	Wars2	70560	Tryptophar	no|no chan	0	COG0180(I
Q545Y5	Q545Y5_M	Cd74	Cd74	16149	CD74 antig	no|up	0	ENOG410Z
B9EKJ7	B9EKJ7_M	Tnks1bp1	Tnks1bp1	228140	Tankyrase 1	no|no chan	0	ENOG410Z
Q9CQ60	6PGL_MO	Pgls	Pgls	66171	6-phosphog	no|no chan	0	COG0363(A
B9EKJ3	B9EKJ3_M	Mon2	Mon2	67074	Protein MC	no|no chan	0	ENOG410X
B2RPW2	B2RPW2_M	Mief1	Mief1	239555	Smith-Mag	no|no chan	0	ENOG411C
Q3U6G0	Q3U6G0_M	Cybb	Cybb	13058	Cytochrom	no|up	0	ENOG410X
Q3U6G1	Q3U6G1_M	Blvrb	Blvrb	233016	NAD(P)-bi	no|no chan	0	ENOG4111
Q5D1E7	ZC12A_MC	Zc3h12a	Zc3h12a	230738	Endoribonu	no|no chan	0	ENOG410Z
G5E869	ZN142_MC	Znf142	Znf142	77264	Zinc finger	no|no chan	0	COG5048(S

G5E866	G5E866_M	Sf3b1	Sf3b1	81898	Splicing fac	no | no chan	0	COG5181(S
Q3U6G8	Q3U6G8_M	Morf4l2	Morf4l2	56397	Mortality fc	no | no chan	0	ENOG410X
A2AES3	A2AES3_M	Otud5	Otud5		ubiquitinyl	no | no chan	0	ENOG410X
P41731	CD63_MOU	Cd63	Cd63	12512	CD63 antig	no | up	0	ENOG4111
Q8BKR5	PPR37_MC	Ppp1r37	Ppp1r37	232947	Protein phc	no | no chan	0	ENOG410X
Q3TIZ8	Q3TIZ8_M	Golm1	Golm1	105348	Golgi mem	no | no chan	0	ENOG4111
Q9D1R9	RL34_MOU	Rpl34	Rpl34	619547; 684	Large ribos	no | no chan	0	COG2174((
Q60760	GRB10_MC	Grb10	Grb10	14783	Growth fac	no | no chan	0	ENOG410Z
Q9D6F9	TBB4A_MC	Tubb4a	Tubb4a	22153	Tubulin be	no | down	0	COG5023(p
Q6URW6	MYH14_MC	Myh14	Myh14	71960	Myosin-14	no | no chan	0	COG5022(v
Q9D1R1	T126B_MO	Tmem126b	Tmem126b	68472	Complex I c	no | no chan	0	ENOG4111
Q3TIZ5	Q3TIZ5_M	Apbb2	Apbb2	11787	Amyloid be	no | no chan	0	ENOG410Y
Q6NXH3	GPBP1_MC	Gpbp1	Gpbp1	73274	Vasculin	no | no chan	0	ENOG410Y
Q6NXH2	MANEA_M	Manea	Manea	242362	Glycoprote	no | no chan	0	ENOG410X
A2ASS6	TITIN_MC	Ttn	Ttn	22138	Titin	no | up	0	
Q9R0A0	PEX14_MC	Pex14	Pex14	56273	Peroxisoma	no | no chan	0	ENOG4111
Q8BML1	MICA2_MC	Mical2	Mical2	320878	[F-actin]-m	no | no chan	0	COG5069(C
P30658	CBX2_MO	Cbx2	Cbx2	12416	Chromobox	no | no chan	0	ENOG4111
B2RVQ7	B2RVQ7_M	Zfp706	Zfp706	68036	Zinc finger	no | no chan	0	ENOG4112
Q8BML9	SYQ_MOU	Qars1	Qars1	97541	Glutamine-	no | no chan	0	COG0008(C
Q8VI38	GBGT1_M	Gbgt1	Gbgt1	227671	Globoside c	no | no chan	0	ENOG4111
P39428	TRAF1_MC	Traf1	Traf1	22029	TNF recept	no | no chan	0	ENOG410Y
P39429	TRAF2_MC	Traf2	Traf2	22030	TNF recept	no | no chan	0	ENOG4111
D3Z2R5	SELN_MO	Selenon	Selenon	74777	Selenoprote	no | no chan	0	ENOG410X
A2AKD7	A2AKD7_M	Snta1	Snta1	20648	Syntrophin,	no | up	0	ENOG410X
A2AKD6	A2AKD6_M	Pxmp4	Pxmp4		Peroxisoma	no | no chan	0	ENOG410Z
Q9JJN5	CBPN_MO	Cpn1	Cpn1	93721	Carboxypep	no | no chan	0	ENOG410X
Q3TDE6	Q3TDE6_M	Pck2	Pck2	74551	phosphoeno	no | no chan	0	COG1274(e
B2CC69	B2CC69_M	Irf5	Irf5	27056	Interferon r	no | up	0	ENOG410X
Q811S7	UBIP1_MC	Ubp1	Ubp1	22221	Upstream-b	no | no chan	0	ENOG410X
Q543S0	Q543S0_M	Prelp	Prelp	116847	LRRNT dor	no | down	0	COG4886(I
E9Q634	MYO1E_M	Myo1e	Myo1e	71602	Unconventi	no | no chan	0	COG5022(Y
Q9CXK8	NIP7_MOU	Nip7	Nip7	66164	60S ribosom	no | no chan	0	COG1374(p
P20722	BMP6_MO	Bmp6	Bmp6	12161	Bone morpl	no | no chan	0	ENOG410X
P11835	ITB2_MOU	Itgb2	Itgb2	16414	Integrin be	no | up	0	ENOG410X
A2AJ88	PLPL7_MC	Pnpla7	Pnpla7	241274	Patatin-like	no | no chan	0	COG1752(F
P39689	CDN1A_M	Cdkn1a	Cdkn1a	12575	Cyclin-depe	no | up	0	ENOG410X
Q99KK7	DPP3_MOU	Dpp3	Dpp3	75221	Dipeptidyl	no | no chan	0	ENOG410X
Q6PFX9	TNKS1_MC	Tnks	Tnks	21951	Poly [ADP-	no | no chan	0	COG0666(F
Q9D7X8	GGCT_MC	Ggct	Ggct	110175	Gamma-glu	no | up	0	ENOG4111
Q3V0K9	PLSI_MOU	Pls1	Pls1	102502	Plastin-1	no | no chan	0	COG5069(r
A0A1B0GS	A0A1B0GS	Ing1	Ing1		Inhibitor of	no | no chan	0	COG5034(T
Q6PFX6	CAD24_MC	Cdh24	Cdh24	239096	Cadherin-2	no | no chan	0	ENOG410X

Q99KK9	SYHM_MC	Hars2	Hars2	70791	Histidine--t	no | no chan	0	COG0124(p
A0A1B0GT	A0A1B0GT	Gys1	Gys1		Glycogen [s	no | up	0	COG0438(C
Q3UTB2	Q3UTB2_M	A630001G2	A630001G2	319997	RIKEN cD	no | up	0	ENOG4111
A0A0U1RQ	A0A0U1RQ	Tnrc6a	Tnrc6a		Trinucleoti	no | no chan	0	ENOG4111
Q8C9V6	Q8C9V6_M	Mpdu1	Mpdu1	24070	Mannose-P	no | no chan	0	ENOG4111
P09405	NUCL_MC	Ncl	Ncl	17975	Nucleolin	no | no chan	0	ENOG410X
Q921G6	LRCH4_MC	Lrch4	Lrch4	231798	Leucine-ric	no | no chan	0	COG4886(C
Q921G7	ETFD_MO	Etfdh	Etfdh	66841	Electron tr	no | no chan	0	COG0644(c
Q5NCS9	MID49_MC	Mief2	Mief2	237781	Mitochondr	no | no chan	0	ENOG410X
O88413	TULP3_MC	Tulp3	Tulp3	22158	Tubby-rela	no | no chan	0	ENOG410X
A2RRZ9	A2RRZ9_M	Tlr12	Tlr12	384059	Toll-like re	no | up	0	COG4886(F
Q99MP8	BRAP_MO	Brap	Brap	72399	BRCA1-ass	no | no chan	0	ENOG410X
Q9D2V8	MFS10_MC	Mfsd10	Mfsd10	68294	Major facili	no | no chan	0	ENOG410X
P08207	S10AA_MC	S100a10	S100a10	20194	Protein S10	no | no chan	0	ENOG410Y
P08752	GNAI2_MC	Gnai2	Gnai2	14678	Guanine nu	no | no chan	0	ENOG410X
P29621	SPA3C_MC	Serpina3c	Serpina3c	16625	Serine prot	no | up	0	COG4826(s
Q8BQY8	HUS1_MO	Hus1	Hus1	15574	Checkpoint	no | no chan	0	ENOG410Z
D3Z4H6	D3Z4H6_M	Tia1	Tia1		Cytotoxic g	no | no chan	0	ENOG410X
Q9R0C8	VAV3_MO	Vav3	Vav3	57257	Guanine nu	no | no chan	0	ENOG410X
A0A1D5RM	A0A1D5RM	Cx3cl1	Cx3cl1		C-X3-C mo	no | no chan	0	ENOG4111
A0A1D5RM	A0A1D5RM	Elmo3	Elmo3	234683	Engulfmen	yes | up	1	ENOG410X
P52430	PON1_MO	Pon1	Pon1	18979	Serum para	no | up	0	ENOG4111
P52431	DPOD1_M	Pold1	Pold1	18971	DNA polym	no | no chan	0	COG0417(r
P52432	RPAC1_MC	Polr1c	Polr1c	20016	DNA-direc	no | no chan	0	COG0202(c
Q9Z0W3	NU160_MC	Nup160	Nup160	59015	Nuclear po	no | no chan	0	ENOG410Y
D6RJJ7	D6RJJ7_MC	Usp21	Usp21	30941	ubiquitinyl	no | no chan	0	ENOG410X
Q56A08	GPKOW_M	Gpkow	Gpkow	209416	G-patch do	no | no chan	0	ENOG410Y
Q3UZ39	LRRF1_MC	Lrrfip1	Lrrfip1	16978	Leucine-ric	no | no chan	0	ENOG4111
A0A2R8VH	A0A2R8VH	Csf2rb2	Csf2rb2	12984	Colony stir	no | no chan	0	ENOG4112
Q3UWW9	Q3UWW9_	Psmd11	Psmd11	69077	26S proteas	no | no chan	0	COG5159(v
D3YY36	D3YY36_M	Inhca	Inhca		Inhibitor of	no | no chan	0	ENOG410X
Q0PD48	Q0PD48_M	Rab9	Rab9	56382	Rab9A	no | no chan	0	ENOG410X
Q924S8	SPRE1_MC	Spred1	Spred1	114715	Sprouty-rel	no | no chan	0	ENOG4112
Q924S7	SPRE2_MC	Spred2	Spred2	114716	Sprouty-rel	no | no chan	0	ENOG410Y
Q0PD42	Q0PD42_M	Rab13	Rab13	68328	Ras-related	no | no chan	0	ENOG410X
Q62311	TAF6_MO	Taf6	Taf6	21343	Transcripti	no | no chan	0	COG5095(T
Q8BPU7	ELMO1_M	Elmo1	Elmo1	140580	Engulfmen	no | no chan	0	ENOG410X
A0A0H2UH	A0A0H2UH	Ehmt1	Ehmt1	77683	Euchromat	no | no chan	0	COG2940(A
A2AG58	BCLA3_MC	Bclaf3	Bclaf3	382252	BCLAF1 an	no | no chan	0	ENOG4112
Q8BI18	Q8BI18_MC	Loxhd1	Loxhd1	240411	PLAT dom	no | no chan	0	
E9QK89	E9QK89_M	Mdc1	Mdc1	240087	Mediator of	no | no chan	0	ENOG4111
Q9QY93	DCTP1_MC	Dctpp1	Dctpp1	66422	dCTP pyrop	no | no chan	0	ENOG4111
E9QK83	E9QK83_M	Utp20	Utp20	70683	UTP20 sma	no | no chan	0	ENOG410X

Q792Z1	Q792Z1_M	Try10	Try10	436522	trypsin	no	up	0	COG5640(S
Q3THA0	Q3THA0_M	Eif3g	Eif3g	53356	Eukaryotic	no	no chan	0	ENOG410Y
A0A3Q4EF	A0A3Q4EF	Cmtr1	Cmtr1		Cap-specifi	no	no chan	0	ENOG410X
Q3THA4	Q3THA4_M	Dnttip1	Dnttip1	76233	Deoxynucle	no	no chan	0	ENOG410X
A0A023J5X	A0A023J5X	ND4	ND4		NADH-ubi	no	no chan	0	COG1008(s
A0A023J5X	A0A023J5X	ND2	ND2		NADH-ubi	no	no chan	0	COG1007(N
Q3U0X6	Q3U0X6_M	Zfp217	Zfp217	228913	Zinc finger	no	no chan	0	COG5048(p
E9Q5D6	E9Q5D6_M	Ranbp9	Ranbp9	56705	Ran-bindin	no	no chan	0	ENOG410X
A0A1W2P7	A0A1W2P7	Tmem19	Tmem19		Transmemb	no	no chan	0	ENOG410X
A0A1W2P7	A0A1W2P7	Max	Max		Max protei	no	no chan	0	ENOG4111
Q9D6S7	RRFM_MC	Mrrf	Mrrf	67871	Ribosome-r	no	no chan	0	COG0233(F
B7ZWC0	B7ZWC0_M	Ndrg1	Ndrg1	17988	Protein ND	no	up	0	ENOG410X
D3Z7V3	D3Z7V3_M	Slmap	Slmap	83997	Sarcolemm	no	no chan	0	COG1716(f
B7ZWC4	B7ZWC4_M	Igf2r	Igf2r	16004	Insulin-like	no	no chan	0	ENOG410Z
Q02526	ZFP41_MC	Zfp41	Zfp41	22701	Zinc finger	no	no chan	0	COG5048(F
A2ANT9	A2ANT9_M	Anks6	Anks6		Ankyrin rep	no	no chan	0	COG0666(A
Q8VCS6	MED9_MC	Med9	Med9	192191	Mediator of	no	no chan	0	ENOG4111
D3Z598	D3Z598_M	Ltbp4	Ltbp4		Latent tran	no	no chan	0	ENOG410Y
Q8VCS3	XYLK_MO	Fam20b	Fam20b	215015	Glycosamir	no	no chan	0	ENOG410X
Q01339	APOH_MC	Apoh	Apoh	11818	Beta-2-glyc	no	no chan	0	ENOG410X
A2NW56	A2NW56_MOUSE E8 variable light chain (Frc				E8 variable	no	no chan	0	ENOG4111
Q8C4J7	TBL3_MOI	Tbl3	Tbl3	213773	Transducin	no	no chan	0	ENOG410X
G3UY52	G3UY52_M	Usp9x	Usp9x		Ubiquitin s	no	no chan	0	COG5077(F
Q8K354	CBR3_MOI	Cbr3	Cbr3	109857	Carbonyl re	no	no chan	0	COG1028(g
Q80WG5	LRC8A_MC	Lrrc8a	Lrrc8a	241296	Volume-reg	no	no chan	0	COG4886(c
Q6P1E1	ZMIZ1_MC	Zmiz1	Zmiz1	328365	Zinc finger	no	no chan	0	ENOG410X
B2RRT9	B2RRT9_M	Zranb2	Zranb2	53861	Zinc finger	no	no chan	0	ENOG4111
E9Q2X6	E9Q2X6_M	Smc4	Smc4		Structural r	no	no chan	0	COG1196(r
H3BLJ7	H3BLJ7_M	Cux1	Cux1		Protein CA	no	up	0	ENOG410X
Q02788	CO6A2_MC	Col6a2	Col6a2	12834	Collagen al	no	down	0	ENOG410X
Q9CYT6	CAP2_MOI	Cap2	Cap2	67252	Adenylyl cy	no	up	0	ENOG410X
A2ALF2	A2ALF2_M	Dnajc8	Dnajc8	68598	DnaJ (Hsp4	no	no chan	0	ENOG411C
G5E8U9	G5E8U9_M	Bnip2	Bnip2	12175	BCL2/aden	no	no chan	0	ENOG410Y
Q9D113	DNLZ_MC	Dnlz	Dnlz	52838	DNL-type z	no	no chan	0	ENOG4112
P41251	NRAM1_M	Slc11a1	Slc11a1	18173	Natural res	no	up	0	COG1914(F
A0A668KL	A0A668KL	Akap12	Akap12	83397	A kinase (P	no	no chan	0	ENOG411C
B1B506	B1B506_M	Sell	Sell	20343	L-selectin	no	down	0	ENOG410Y
H3BK13	H3BK13_M	Hmbox1	Hmbox1		Homeobox	no	no chan	0	ENOG4111
Q8K297	GT251_MC	Colgalt1	Colgalt1	234407	Procollager	no	no chan	0	ENOG410X
Q8K299	SCAR5_MC	Scara5	Scara5	71145	Scavenger r	no	no chan	0	ENOG4111
Q8K298	ANLN_MC	Anln	Anln	68743	Anillin	no	no chan	0	ENOG411C
Q9DBW3	NATD1_M	Natd1	Natd1	24083	Protein NA	no	no chan	0	ENOG4112
Q3TQC7	Q3TQC7_M	Entpd5	Entpd5	12499	nucleoside	no	no chan	0	COG5371(i

A0A0A6YW	A0A0A6YW	Ighv5-15	Ighv5-15		Immunoglo	no|no chan	0	ENOG410Y
Q9JIF7	COPB_MO	Copb1	Copb1	70349	Coatomer s	no|no chan	0	COG5096(T
A1L333	A1L333_M(	Ddx5	Ddx5	13207	Probable A	no|no chan	0	COG0513(p
B7ZJH7	B7ZJH7_M	Sigmar1	Sigmar1	18391	Sigma non-	no|no chan	0	ENOG410X
Q9DBG9	TX1B3_MC	Tax1bp3	Tax1bp3	76281	Tax1-bindi	no|no chan	0	ENOG4111
Q8BMQ8	MON1B_M	Mon1b	Mon1b	270096	Vacuolar fu	no|no chan	0	ENOG410X
Q3TC66	Q3TC66_M	Riok3	Riok3	66878	Serine/thre	no|no chan	0	COG1718(s
Q8VHK1	CSKI2_MO	Caskin2	Caskin2	140721	Caskin-2	no|no chan	0	COG0666(g
Q3TC60	Q3TC60_M	1700123O2(	1700123O2(	58248	Uncharacte	no|no chan	0	ENOG4111
Q8VEE4	RFA1_MOI	Rpa1	Rpa1	68275	Replication	no|no chan	0	COG1599(L
Q3UG43	Q3UG43_M	Oard1	Oard1	106821	Macro dom	no|no chan	0	ENOG4111
Q9JIY0	PKHO1_M	Plekho1	Plekho1	67220	Pleckstrin h	no|no chan	0	ENOG411(
A0A3Q4EC	A0A3Q4EC	Esco1	Esco1		Establishm	no|no chan	0	ENOG410X
E0CXW2	E0CXW2_M	Adck5	Adck5		AarF doma	no|down	0	COG0661(I
Q99JH1	RP25L_MC	Rpp25l	Rpp25l	69961	Ribonuclea	no|no chan	0	ENOG4111
A0A0A6YV	A0A0A6YV	Tlr5	Tlr5	53791	Toll-like rec	no|no chan	0	COG4886(s
Q80ZV0	RNH2B_M	Rnaseh2b	Rnaseh2b	67153	Ribonuclea	no|no chan	0	ENOG410Y
Q9CQS5	RIOK2_MC	Riok2	Riok2	67045	Serine/thre	no|no chan	0	COG0478(S
Q9CQS4	S2546_MOI	Slc25a46	Slc25a46	67453	Mitochondr	no|no chan	0	ENOG410X
Q9CQS3	FIBIN_MC	Fibin	Fibin	67606	Fin bud ini	no|no chan	0	ENOG410Z
Q1HL32	Q1HL32_M	Smc3	Smc3	13006	Structural r	no|no chan	0	COG1196(A
P11247	PERM_MC	Mpo	Mpo	17523	Myeloperox	no|up	0	ENOG410X
Q9CQS9	HAUS2_MC	Haus2	Haus2	66296	HAUS augr	no|no chan	0	ENOG410Y
Q9CQS8	SC61B_MO	Sec61b	Sec61b	66212	Protein trar	no|no chan	0	ENOG4111
A0A8I4RSN	A0A8I4RSN	Cacna1s	Cacna1s		Voltage-dep	no|up	0	ENOG410X
Q9Z1P6	NDUA7_M	Ndufa7	Ndufa7	66416	NADH deh	no|no chan	0	ENOG410X
Q9QXK3	COPG2_M	Copg2	Copg2	54160	Coatomer s	no|no chan	0	COG5240(T
A0A0R4J1S	A0A0R4J1S	Csgalnact1	Csgalnact1	234356	Hexosyltrar	no|no chan	0	ENOG410X
P06537	GCR_MOU	Nr3c1	Nr3c1	14815	Glucocortic	no|no chan	0	ENOG410X
A0A0R4J1S	A0A0R4J1S	Prpf40b	Prpf40b	54614	Pre-mRNA	no|no chan	0	COG5104(I
Q91V35	Q91V35_M	Ptpra	Ptpra	19262	Receptor-ty	no|no chan	0	COG5599(-
Q91V36	NRBP2_MC	Nrbp2	Nrbp2	223649	Nuclear rec	no|up	0	ENOG410X
Q99K46	UBP11_MC	Usp11	Usp11	236733	Ubiquitin c	no|no chan	0	COG5560(s
Q9ESY9	GILT_MOU	Ifi30	Ifi30	65972	Gamma-int	no|no chan	0	ENOG4111
Q8BKH7	SIN1_MOU	Mapkap1	Mapkap1	227743	Target of rc	no|no chan	0	ENOG410X
E9PUU4	E9PUU4_M	Gemin5	Gemin5	216766	Gem nuclec	no|no chan	0	ENOG410X
F6TCF9	F6TCF9_M	Bag1	Bag1		BAG family	no|no chan	0	ENOG4111
A0A087WC	A0A087WC	Skp2	Skp2	27401	S-phase kin	no|down	0	ENOG410Z
Q9D8Y1	T126A_MC	Tmem126a	Tmem126a	66271	Transmemb	no|no chan	0	ENOG4111
P62305	RUXE_MC	Snrpe	Snrpe	20643	Small nucle	no|no chan	0	COG1958(c
P13707	GPDA_MC	Gpd1	Gpd1	14555	Glycerol-3-	no|down	0	COG0240(c
Q9CZ04	CSN7A_MC	Cops7a	Cops7a	26894	COP9 signc	no|no chan	0	ENOG410X
B2RV69	B2RV69_M	Tmem199	Tmem199	195040	Transmemb	no|no chan	0	ENOG4111

E9PXF8	MTMRD_M	Sbf2	Sbf2	319934	Myotubular	no|no chan	0	ENOG410X
A0A0R4J1C	A0A0R4J1C	Tcf7l2	Tcf7l2	21416	Transcripti	no|no chan	0	ENOG411C
B1AQJ2	UBP36_MC	Usp36	Usp36	72344	Ubiquitin c	no|no chan	0	ENOG410X
E9QA45	E9QA45_M	Gtpbp2	Gtpbp2		GTP bindir	no|no chan	0	COG5258(C
F8WJG3	F8WJG3_M	Tra2b	Tra2b	20462	Transforme	no|no chan	0	ENOG411C
P12265	BGLR_MO	Gusb	Gusb	110006	Beta-glucur	no|no chan	0	COG3250(I
Q3UM63	Q3UM63_M	Slc30a1	Slc30a1	22782	Zinc transp	no|no chan	0	COG1230(U
Q8BTN4	Q8BTN4_M	Uba7	Uba7	74153	Ubiquitin-c	no|no chan	0	COG0476(s
Q99LR1	ABD12_MC	Abhd12	Abhd12	76192	Lysophosph	no|no chan	0	COG1073(I
A0A087WS	A0A087WS	Eef1b2	Eef1b2		Eukaryotic	no|no chan	0	COG2092(r
Q9CPS6	HINT3_MC	Hint3	Hint3	66847	Adenosine	no|no chan	0	ENOG4111
Q9CPS7	PNO1_MO	Pno1	Pno1	66249	RNA-bindi	no|no chan	0	COG1094(M
Q6PHZ5	RB15B_MC	Rbm15b	Rbm15b	109095	Putative RN	no|no chan	0	ENOG410X
A2AUM0	A2AUM0_M	Eif2ak4	Eif2ak4	27103	non-specific	no|no chan	0	COG0124(I
A0A0R4J09	A0A0R4J09	Cd1d1	Cd1d1	12479	CD1d1 anti	no|no chan	0	ENOG4111
P47968	RPIA_MOU	Rpia	Rpia	19895	Ribose-5-ph	no|no chan	0	COG0120(p
P47964	RL36_MOU	Rpl36	Rpl36	54217	Large ribos	no|no chan	0	COG5051(6
Q9CWE0	MFR1L_MC	Mtfr1l	Mtfr1l	76824	Mitochondr	no|no chan	0	ENOG4111
P47962	RL5_MOUS	Rpl5	Rpl5	100503670	Large ribos	no|no chan	0	COG0256(T
P47963	RL13_MOU	Rpl13	Rpl13	270106	Large ribos	no|no chan	0	COG4352(c
Q3TP92	CNEP1_MC	Ctdnep1	Ctdnep1	67181	CTD nucle	no|no chan	0	COG5190(F
Q9D8U0	IFRD2_MC	Ifrd2	Ifrd2	15983	Interferon-r	no|no chan	0	ENOG410X
Q60819	I15RA_MO	Il15ra	Il15ra	16169	Interleukin-	no|up	0	ENOG410Z
Q3U568	Q3U568_M	Mafk	Mafk	17135	BZIP doma	no|up	0	ENOG4111
E9QLA5	E9QLA5_M	Inf2	Inf2	70435	Inverted for	no|no chan	0	ENOG410X
B1AST7	B1AST7_M	Mast2	Mast2	17776	non-specific	no|no chan	0	ENOG410X
D3Z7F1	D3Z7F1_M	Cyp2s1	Cyp2s1		Cytochrom	no|no chan	0	COG2124(r
P50608	FMOD_MC	Fmod	Fmod	14264	Fibromodu	no|down	0	COG4886(S
Q3TGJ9	Q3TGJ9_M	Gsn	Gsn	227753	Gelsolin	no|no chan	0	ENOG410X
M4TKR7	M4TKR7_M	Insr	Insr	16337	Tyrosine-pr	no|no chan	0	COG0515(F
Q8C0L8	COG5_MC	Cog5	Cog5	238123	Conserved	no|no chan	0	ENOG410X
G3UWC2	G3UWC2_I	Naalad2	Naalad2	72560	N-acetylate	no|no chan	0	COG2234(S
Q14BF8	Q14BF8_M	Trappc1	Trappc1	245828	Trafficking	no|no chan	0	ENOG4111
E9PV38	E9PV38_M	Ces2g	Ces2g	72361	Carboxylic	no|down	0	COG2272(I
Q8R2K3	Q8R2K3_M	Ssbp1	Ssbp1	381760	Single-strar	no|no chan	0	COG0629(s
O35691	PININ_MC	Pnn	Pnn	18949	Pinin	no|no chan	0	ENOG411C
Q7TND5	RPF1_MOU	Rpf1	Rpf1	70285	Ribosome p	no|no chan	0	COG2136(u
Q3TXG1	Q3TXG1_M	Map3k7	Map3k7	26409	Mitogen-ac	no|no chan	0	COG0515(C
Q7TND3	Q7TND3_M	Ap4b1	Ap4b1	67489	AP complex	no|no chan	0	COG5096(S
Q3TJD4	Q3TJD4_M	Atp5pb	Atp5pb	11950	ATP syntha	no|no chan	0	ENOG4111
Q3TAP1	Q3TAP1_M	Ppm1d	Ppm1d	53892	PPM-type p	no|no chan	0	COG0631(T
Q3TJD7	PDLI7_MC	Pdlim7	Pdlim7	67399	PDZ and L	no|no chan	0	ENOG410X
Q3TAP4	AP5B1_MC	Ap5b1	Ap5b1	381201	AP-5 compl	no|no chan	0	ENOG410Y

Q8VD72	TTC8_MOI	Ttc8	Ttc8	76260	Tetratricop	no | down	0	ENOG410Z
Q3U300	Q3U300_M	Bcl3	Bcl3	12051	BCL3 trans	no | up	0	COG0666(M
Q921W4	QORL1_M	Cryzl1	Cryzl1	66609	Quinone ox	no | no chan	0	COG0604(M
Q3U304	Q3U304_M	Pdcd1lg2	Pdcd1lg2	58205	Pdcd1lg2 pr	no | no chan	0	ENOG4111
Q8VD75	HIP1_MOU	Hip1	Hip1	215114	Huntingtin	no | no chan	0	ENOG410X
E9Q8A3	E9Q8A3_M	Pi4kb	Pi4kb		Phosphatid	no | no chan	0	ENOG410X
Q8R411	MYCT1_M	Myct1	Myct1	68632	Myc target	no | down	0	ENOG4111
P56183	RRP1_MOU	Rrp1	Rrp1	18114	Ribosomal	no | no chan	0	ENOG4111
Q3UIZ4	Q3UIZ4_M	Cxxc1	Cxxc1	74322	CXXC-type	no | no chan	0	ENOG410Y
P70224	GIMA1_M	Gimap1	Gimap1	16205	GTPase IM	no | no chan	0	ENOG410Y
P70227	ITPR3_MO	Itpr3	Itpr3	16440	Inositol 1,4,	no | no chan	0	ENOG410X
O88627	S28A2_MO	Slc28a2	Slc28a2	269346	Sodium/nu	no | no chan	0	COG1972()
Q60949	TBCD1_M	Tbc1d1	Tbc1d1	57915	TBC1 domc	no | no chan	0	ENOG410Y
B2RRZ7	B2RRZ7_M	Tradd	Tradd	71609	Tumor necr	no | no chan	0	ENOG4111
E9Q7C4	E9Q7C4_M	Prr14l	Prr14l	215476	Proline rich	no | down	0	ENOG4111
Q3TI84	Q3TI84_M	Cdc16	Cdc16	69957	Uncharacte	no | no chan	0	ENOG410X
E9Q7C1	E9Q7C1_M	Med15	Med15	94112	Mediator of	no | no chan	0	ENOG410Y
E9Q7C0	E9Q7C0_M	1700030K0	1700030K09Rik		RIKEN cD	no | no chan	0	ENOG4111
Q3U9N9	MOT10_M	Slc16a10	Slc16a10	72472	Monocarbo	no | no chan	0	ENOG410X
Q3UH60	DIP2B_MC	Dip2b	Dip2b	239667	Disco-inter	no | no chan	0	COG0318(V
Q7TN90	Q7TN90_M	Polm	Polm	54125	DNA-direc	no | no chan	0	COG1796(L
P07724	ALBU_MO	Alb	Alb	11657	Albumin	no | no chan	0	ENOG410Z
Q3U3M9	Q3U3M9_M	Clec4n	Clec4n	56620	C-type lecti	no | no chan	0	ENOG410Z
A0A217FL8	A0A217FL8	Ccdc85c	Ccdc85c	668158	Coiled-coil	no | no chan	0	ENOG410Y
A0A0R4J0F	A0A0R4J0F	Fndc3b	Fndc3b	72007	Fibronectin	no | no chan	0	ENOG410X
Q8BPB5	FBLN3_M	Efemp1	Efemp1	216616	EGF-conta	no | down	0	ENOG410Y
A0A0R4J0F	A0A0R4J0F	Ncapd2	Ncapd2	68298	Condensin	no | no chan	0	COG5098(E
A0A0A6YX	A0A0A6YX	Prkar2a	Prkar2a		cAMP-depe	no | no chan	0	COG0664(E
A0A0A6YX	A0A0A6YX	Ighv15-2	Ighv15-2		Immunoglo	no | up	0	ENOG4111
Q8R3F9	STPAP_MC	Tut1	Tut1	70044	Speckle targ	no | up	0	COG5260(E
Q8R3F5	FABD_MO	Mcat	Mcat	223722	Malonyl-Co	no | no chan	0	COG0331(M
Q4VBD2	TAPT1_MC	Tapt1	Tapt1	231225	Transmemb	no | no chan	0	ENOG410X
Q9CQ65	MTAP_MC	Mtap	Mtap	66902	S-methyl-5'	no | no chan	0	COG0005(A
Q8R5K4	NOL6_MO	Nol6	Nol6	230082	Nucleolar p	no | no chan	0	ENOG410X
Q8CBC7	TRM7_MO	Ftsj1	Ftsj1	54632	tRNA (cytic	no | no chan	0	COG0293(E
B8JJD3	B8JJD3_M	Fance	Fance	72775	Fanconi an	no | down	0	ENOG4112
A0A338P6F	A0A338P6F	Gm49601	Gm49601		Septin	no | no chan	0	COG5019(C
Q80UF7	TCAM1_M	Ticam1	Ticam1	106759	TIR domain	yes | up	1	ENOG410Y
Q3TWW5	Q3TWW5_	Wbp4	Wbp4	22380	WW domai	no | no chan	0	ENOG4111
Q80U76	Q80U76_M	Rrs1	Rrs1	59014	Ribosome b	no | no chan	0	COG5225(r
Q3TWW8	SRSF6_MO	Srsf6	Srsf6	67996	Serine/argi	no | no chan	0	COG0724(p
A2AK40	A2AK40_M	Slc30a4	Slc30a4	22785	Solute carri	no | no chan	0	COG1230(C
P53808	PPCT_MO	Pctp	Pctp	18559	Phosphatid	no | no chan	0	ENOG4111

P0DJF2	PT117_MO	Pet117	Pet117	100048644	Protein PE	no|no chan	0	ENOG410
Q5BKP5	Q5BKP5_M	Ddx50	Ddx50	94213	RNA helico	no|no chan	0	COG0513(I
P13864	DNMT1_M	Dnmt1	Dnmt1	13433	DNA (cytos	no|no chan	0	COG0270(C
E9Q401	RYR2_MO	Ryr2	Ryr2	20191	Ryanodine	yes|up	1	ENOG410
E9Q6C9	E9Q6C9_M	Entrep3	Entrep3	68521	Endosomal	no|down	0	ENOG410
D3YWS7	D3YWS7_M	Fuom	Fuom	69064	L-fucose m	no|no chan	0	COG4154(I
D3YWS8	D3YWS8_M	Leng8	Leng8	232798	Leukocyte r	no|no chan	0	ENOG410
E9Q6C2	E9Q6C2_M	C1s1	C1s1	50908	Complemer	no|no chan	0	COG5640(I
Q6P549	SHIP2_MO	Inppl1	Inppl1	16332	Phosphatid	no|no chan	0	COG5411(
A0A494BA	A0A494BA	Ostf1	Ostf1		Osteoclast-	no|no chan	0	ENOG410
O35604	NPC1_MO	Npc1	Npc1	18145	NPC intrac	no|no chan	0	ENOG410
Q9CQL4	RM20_MO	Mrpl20	Mrpl20	66448	Large ribos	no|no chan	0	COG0292(I
Q9CQL5	RM18_MO	Mrpl18	Mrpl18	67681	Large ribos	no|no chan	0	ENOG4111
Q9CQL6	RM35_MO	Mrpl35	Mrpl35	66223	Large ribos	no|no chan	0	ENOG4111
Q9CQL7	MOFA1_M	Mrfap1	Mrfap1	67568	MORF4 far	no|no chan	0	ENOG4111
Q9CQL0	MT21A_M	Mettl21A	Mettl21A	67099	Protein N-l	no|no chan	0	ENOG4111
Q66VB7	Q66VB7_M	Gm1553	Gm1553	432480	Lacrein	no|down	0	
B1AUF1	B1AUF1_M	Ski	Ski	20481	Ski oncoger	no|no chan	0	ENOG410
B1AW58	B1AW58_M	Camk1d	Camk1d	227541	Calcium/cc	no|no chan	0	ENOG410
Q6PDI5	ECM29_M	Ecpas	Ecpas	230249	Proteasome	no|no chan	0	ENOG410
Q3TQP7	Q3TQP7_M	Acat1	Acat1	110446	Acetyl-CoA	no|no chan	0	COG0183(c
Q4VAB2	Q4VAB2_M	Gpn1	Gpn1	74254	GPN-loop	no|no chan	0	ENOG410
Q8BGS1	E41L5_MO	Epb41l5	Epb41l5	226352	Band 4.1-lil	no|no chan	0	ENOG410
F8VPR2	F8VPR2_M	Fmnl2	Fmnl2	71409	Formin-like	no|no chan	0	
Q4VAB6	Q4VAB6_M	Igh	Igh		Igh protein	no|up	0	ENOG410
G3X8V5	G3X8V5_M	Obi1	Obi1	72486	ORC ubiqu	no|no chan	0	ENOG4111
D3YVU4	D3YVU4_M	Kdm2b	Kdm2b		[histone H3	no|no chan	0	ENOG410
D3YVU1	D3YVU1_M	Usp16	Usp16		ubiquitinyl	no|no chan	0	COG5560(c
D3YVU0	D3YVU0_M	Usp46	Usp46		Ubiquitin c	no|up	0	ENOG410
Q5XG69	F169A_MO	Fam169a	Fam169a	320557	Soluble lam	no|no chan	0	ENOG410
A2A6S1	A2A6S1_M	Tmem104	Tmem104		Transmemb	no|up	0	ENOG410
P23492	PNPH_MO	Pnp	Pnp	18950	Purine nucl	no|no chan	0	COG0005(
Q80SX0	Q80SX0_M	Stard4	Stard4	170459	StAR relate	no|no chan	0	ENOG4111
A0A075B5	A0A075B5	Igkv5-39	Igkv5-39		Immunoglo	no|up	0	ENOG410
A0A075B5	A0A075B5	Igkv4-58	Igkv4-58		Immunoglo	no|no chan	0	ENOG4111
Q9CXD9	LRC17_MC	Lrrc17	Lrrc17	74511	Leucine-ric	no|no chan	0	COG4886(c
A0A075B5	A0A075B5	Igkv4-63	Igkv4-63		Immunoglo	no|up	0	ENOG4111
Q3V0T8	Q3V0T8_M	Ggps1	Ggps1	14593	Geranylger	no|no chan	0	COG0142(
Q8BYU6	TOIP2_MC	Tor1aip2	Tor1aip2	240832	Torsin-1A-i	no|no chan	0	ENOG4111
A2A6Q5	CDC27_M	Cdc27	Cdc27	217232	Cell divisio	no|no chan	0	COG0457(r
Q9WV52	PLEK2_M	Plek2	Plek2	27260	Pleckstrin-2	no|no chan	0	ENOG410
Q8K339	KIN17_MC	Kin	Kin	16588	DNA/RNA	no|no chan	0	ENOG410
F6ZV59	F6ZV59_M	Hnrnpd	Hnrnpd		Heterogene	no|no chan	0	COG0724(r

Q1XID4	Q1XID4_M	Atp6ap2	Atp6ap2	70495	Renin recep	no | up	0	ENOG4111
A2A6Q8	A2A6Q8_M	Myl4	Myl4		Myosin, ligl	no | no chan	0	COG5126(C
Q9CTW2	Q9CTW2_M	Lipe	Lipe	16890	Hormone-se	no | no chan	0	COG0657(c
Q8CIA5	S35B4_MO	Slc35b4	Slc35b4	58246	Nucleotide	no | no chan	0	ENOG410X
B8JK56	B8JK56_M(	Adcy3	Adcy3	104111	Adenylate c	no | no chan	0	COG2114(t
Q8C7H1	MMAA_M(	Mmaa	Mmaa	109136	Methylmalc	no | no chan	0	COG1703(I
Q62059	CSPG2_M(	Vcan	Vcan	13003	Versican co	no | no chan	0	ENOG410Z
F8VQ29	F8VQ29_M	Iqgap3	Iqgap3	404710	IQ motif cc	no | no chan	0	COG5261(C
A0A2R8VK	A0A2R8VK	Polr3h	Polr3h		DNA-direc	no | no chan	0	COG1095(I
Q7TSS2	UB2Q1_M(	Ube2q1	Ube2q1	70093	Ubiquitin-c	no | no chan	0	ENOG410X
Q6ZQJ5	DNA2_MC	Dna2	Dna2	327762	DNA replic	no | no chan	0	COG1112(T
A0A0R4J16	A0A0R4J16	Foxp4	Foxp4	74123	Forkhead b	no | no chan	0	COG5025(z
Q05CI8	Q05CI8_M(	Kyat1	Kyat1	70266	Ccbl1 prote	no | no chan	0	COG0436(C
Q6PCL9	PAPOG_M	Papolg	Papolg	216578	Poly(A) pol	no | no chan	0	COG5186(p
A0A0R4J16	A0A0R4J16	Klhl40	Klhl40	72330	Kelch-like 4	no | up	0	ENOG410X
Q148Z6	Q148Z6_M	Gpr107	Gpr107	277463	G protein-c	no | no chan	0	ENOG410X
E0CZ58	E0CZ58_M	Prg4	Prg4	96875	Proteoglyca	no | up	0	ENOG410X
Q3UAG2	Q3UAG2_M	Pgd	Pgd	110208	6-phosphog	no | no chan	0	COG0362(C
E9PVP0	E9PVP0_M	Stard10	Stard10		StAR relate	no | no chan	0	ENOG411C
A1A4A7	A1A4A7_M	Pgam5	Pgam5	72542	Serine/thre	no | no chan	0	ENOG410X
Q91VE6	MK67I_MC	Nifk	Nifk	67949	MKI67 FHA	no | no chan	0	ENOG4111
Q3UP21	Q3UP21_M	Lrrc42	Lrrc42	77809	Leucine-ric	no | no chan	0	ENOG410Y
A0A2I3BQ9	A0A2I3BQ9	Gm55359	Gm55359		Predicted g	no | up	0	ENOG410Z
D3Z5I9	D3Z5I9_M(	Rbm33	Rbm33	381626	RNA bindin	no | no chan	0	ENOG410X
D3Z5I6	D3Z5I6_M(	Iqsec2	Iqsec2		IQ motif ar	no | no chan	0	COG5307(S
D3Z5I1	D3Z5I1_M(	Zc3hav1	Zc3hav1	78781	Zinc finger	no | no chan	0	ENOG410Y
Q6ZWS7	Q6ZWS7_M	Camk2g	Camk2g	12325	calcium/ca	no | no chan	0	ENOG410X
Q8BWM0	PGES2_M(	Ptges2	Ptges2	96979	Prostaglanc	no | no chan	0	ENOG410X
E9QAQ5	E9QAQ5_M	Gsk3b	Gsk3b	56637	Glycogen sy	no | no chan	0	COG0515(F
Q9CYD3	CRTAP_M(	Crtap	Crtap	56693	Cartilage-a	no | no chan	0	ENOG410Y
Q920I9	WDR7_MC	Wdr7	Wdr7	104082	WD repeat-	no | no chan	0	ENOG410Y
B9EK92	B9EK92_M	Rasip1	Rasip1	69903	Ras interac	no | no chan	0	COG5022(F
B9EK90	B9EK90_M	Snrk	Snrk	20623	non-specifi	no | up	0	ENOG410X
Q3UFC9	Q3UFC9_M	Plekhb2	Plekhb2	226971	PH domain	no | no chan	0	ENOG4111
A0A0E4B3(	A0A0E4B3(	HC	HC		MAb 44B1 l	no | up	0	ENOG410Y
B2RQS1	B2RQS1_M	Strn3	Strn3	94186	Striatin, cal	no | no chan	0	ENOG410X
B9EIZ9	B9EIZ9_M(	Nlrp9b	Nlrp9b	243874	NLR family	no | no chan	0	ENOG410Z
B2RQS6	B2RQS6_M	Dhx36	Dhx36	72162	RNA helico	no | no chan	0	COG1643(S
A0A140T8N	A0A140T8N	Igkv6-23	Igkv6-23	637227	Immunoglo	no | up	0	ENOG410Y
B9EIZ7	B9EIZ7_M(	Coro6	Coro6	216961	Coronin	yes | up	1	ENOG410X
Q14A47	Q14A47_M	Tma7	Tma7	66167	Coiled-coil	no | no chan	0	ENOG410Z
F8WHD8	F8WHD8_M	Synj2	Synj2	20975	phosphoinc	no | no chan	0	COG5411(S
Q3TZF1	Q3TZF1_M	Acvr1b	Acvr1b	11479	receptor pr	no | up	0	ENOG410X

Q91VP7	TM101_MC	Tmem101	Tmem101	76547	Transmemt	no	no chan	0	ENOG410
H3BKQ2	H3BKQ2_M	Iftap	Iftap		Intraflagell	no	no chan	0	ENOG4112
A0A0R4J14	A0A0R4J14	Cluh	Cluh	74148	Clustered m	no	no chan	0	ENOG410
Q3TWN5	Q3TWN5_	Hnrnpu	Hnrnpu	51810	Uncharacte	no	no chan	0	ENOG410
A0A494B95	A0A494B95	Nedd4l	Nedd4l		HECT-type	no	no chan	0	COG5021(r
B2RPU7	B2RPU7_M	Klf16	Klf16	118445	Kruppel-lik	no	no chan	0	COG5048(-
Q9CQ45	NENF_MC	Nenf	Nenf	66208	Neudesin	no	no chan	0	ENOG4111
B2RPU5	B2RPU5_M	Traf3ip3	Traf3ip3	215243	TRAF3 inte	no	no chan	0	ENOG410
Q9CQ40	RM49_MO	Mrpl49	Mrpl49	18120	Large ribos	no	no chan	0	ENOG4111
Q9CQ43	DUT_MOU	Dut	Dut	110074	Deoxyuridi	no	no chan	0	COG0756(
O35864	CSN5_MO	Cops5	Cops5	26754	COP9 signc	no	no chan	0	COG1310(
E9PWV3	E9PWV3_M	Rps6ka1	Rps6ka1	20111	Ribosomal	no	no chan	0	ENOG410
Q9CQ48	NUDC2_M	Nudcd2	Nudcd2	52653	NudC dom	no	no chan	0	ENOG410
E9PWV4	E9PWV4_M	Cdin1	Cdin1	399568	CDAN1-int	yes	down	1	ENOG410
B2RPU8	B2RPU8_M	Zbed5	Zbed5	71970	SCAN dom	no	no chan	0	ENOG410
B9EKL9	B9EKL9_M	Eml1	Eml1	68519	Eml1 prote	no	no chan	0	COG2319(v
A0A9L6HJI	A0A9L6HJI	Lrrcc1	Lrrcc1	71710	Leucine ricl	no	up	0	COG4886(j
P97762	RP9_MOUS	rp9	rp9	55934	Retinitis pi	no	no chan	0	ENOG410
B2KF05	BRPF3_MC	Brpf3	Brpf3	268936	Bromodom	no	down	0	COG5076(I
B9EKL6	B9EKL6_M	Ptp4a1	Ptp4a1	19243; 4340	Protein tyrc	no	no chan	0	ENOG4111
P49586	PCY1A_M	Pcyt1a	Pcyt1a	13026	Choline-ph	no	no chan	0	COG0615(c
Q6PGH0	UBTD2_M	Ubtd2	Ubtd2	327900	Ubiquitin d	no	no chan	0	ENOG410
Q3U6E9	Q3U6E9_M	Tubgcp4	Tubgcp4	51885	Gamma-tul	no	no chan	0	ENOG410
Q6NXJ0	WWC2_MC	Wwc2	Wwc2	52357	Protein WW	no	no chan	0	COG5021(F
E3SRG8	E3SRG8_M	Smad4	Smad4	17128	Mothers ag	no	no chan	0	ENOG410
Q3TIX9	UBP39_MC	Usp39	Usp39	28035	Ubiquitin c	no	no chan	0	ENOG410
Q76MZ3	2AAA_MO	Ppp2r1a	Ppp2r1a	51792	Serine/thre	no	no chan	0	ENOG410
E9Q186	E9Q186_M	Fcf1	Fcf1		FCF1 rRNA	no	no chan	0	COG1412(s
A2A7U2	A2A7U2_M	Zmym6	Zmym6	100177	Zinc finger	no	no chan	0	ENOG410
A2A7U3	A2A7U3_M	Tmem35b	Tmem35b		Transmemt	yes	up	1	ENOG4111
Q3TA31	Q3TA31_M	Fig4	Fig4	103199	SAC domai	no	no chan	0	COG5329(F
Q99J87	DHX58_M	Dhx58	Dhx58	80861	ATP-depen	no	up	0	COG1111(h
Q3UN88	Q3UN88_M	Mcpt4	Mcpt4	17227	Mast cell p	no	down	0	COG5640(e
A2RSV5	A2RSV5_M	Isl1	Isl1	16392	Insulin gen	no	no chan	0	ENOG410
Q6IQX7	CHSS2_MC	Chpf	Chpf	74241	Chondroiti	no	no chan	0	ENOG410
B9EJX8	B9EJX8_M	Ppil1	Ppil1	68816	Peptidyl-pr	no	no chan	0	COG0652(
P47754	CAZA2_M	Capza2	Capza2	12343	F-actin-cap	no	no chan	0	ENOG410
B9EJX5	B9EJX5_M	Taf2	Taf2	319944	Transcripti	no	no chan	0	ENOG410
P47758	SRPRB_MC	Srprb	Srprb	20818	Signal reco	no	no chan	0	COG2229(c
A2RSV8	A2RSV8_M	Cox4i1	Cox4i1	12857	Cytochrom	no	no chan	0	ENOG4111
D3YWN6	D3YWN6_	Donson	Donson		Downstrea	yes	down	1	ENOG410
Q58DZ5	Q58DZ5_M	Mrps33	Mrps33	14548	Small ribos	no	no chan	0	ENOG4111
P20918	PLMN_MC	Plg	Plg	18815	Plasminoge	no	up	0	COG5640(I

A2AKB9	DCA10_MO	Dcaf10	Dcaf10	242418	DDB1- and	no|no chan	0	ENOG410X
Q91YM4	FAKD4_M	Tbrg4	Tbrg4	21379	FAST kinas	no|no chan	0	ENOG4111
P83870	PHF5A_MO	Phf5a	Phf5a	68479	PHD finger	no|no chan	0	ENOG4111
P83877	TXN4A_M	Txnl4a	Txnl4a	27366	Thioredoxi	no|no chan	0	ENOG4111
A0A6I8MX	A0A6I8MX	Ago1	Ago1	236511	Argonaute	no|no chan	0	ENOG410X
Q5SVJ0	Q5SVJ0_M	Camk2b	Camk2b	12323	calcium/ca	no|up	0	ENOG410X
A0A6I8MX	A0A6I8MX	Tbc1d25	Tbc1d25	209815	Ornithine c	no|no chan	0	COG5210(M
Q8BXZ1	TMX3_MO	Tmx3	Tmx3	67988	Protein dis	no|no chan	0	COG0526(A
Q6GU23	Q6GU23_M	Stat3	Stat3	20848	Signal trans	no|no chan	0	ENOG410X
Q9WUF3	C8AP2_MC	Casp8ap2	Casp8ap2	26885	CASP8-assc	no|no chan	0	ENOG410Y
Q3TDG9	Q3TDG9_M	Stx12	Stx12	100226	t-SNARE c	no|no chan	0	COG5325(F
Q99KQ4	NAMPT_M	Nampt	Nampt	59027	Nicotinami	no|up	0	COG1488(M
A2A5K6	ZN335_MC	Znf335	Znf335	329559	Zinc finger	no|no chan	0	COG5048(r
O70230	ZN143_MC	Znf143	Znf143	20841	Zinc finger	no|no chan	0	COG5048(F
Q8K0D5	EFGM_MC	Gfm1	Gfm1	28030	Elongation	no|no chan	0	COG0480(C
Q9EQX4	AIF1L_MO	Aif1l	Aif1l	108897	Allograft in	no|no chan	0	ENOG4111
E9Q3R9	E9Q3R9_M	Zbtb21	Zbtb21	114565	Zinc finger	no|up	0	COG5048(I
Q3U466	Q3U466_M	Cln6	Cln6	76524	Ceroid-lipo	no|no chan	0	ENOG410Z
A0A1L1SVI	A0A1L1SVI	Rbpms2	Rbpms2		RNA bindi	no|no chan	0	ENOG410X
Q07763	CD244_MC	Cd244	Cd244	18106	Natural kil	no|up	0	ENOG410Y
Q8VEH3	ARL8A_MO	Arl8a	Arl8a	68724	ADP-ribosy	no|no chan	0	ENOG410X
A0A1Y7VK	A0A1Y7VK	Fancm	Fancm		Fanconi an	no|up	0	COG1111(F
Q1PG84	Q1PG84_M	Rsl1d1	Rsl1d1	66409	PBK1	no|no chan	0	ENOG4111
A2IXM7	A2IXM7_MOUSE	Anti-CD30 moab Ki-4 scFv			Anti-CD30	no|no chan	0	ENOG4111
P14824	ANXA6_M	Anxa6	Anxa6	11749	Annexin A6	no|no chan	0	ENOG410X
D6RET6	D6RET6_M	Gpatch1	Gpatch1		G patch do	no|no chan	0	ENOG410Y
A0A1B0GS	A0A1B0GS	Folr2	Folr2		Folate rece	no|down	0	ENOG4111
Q99KI3	EMC3_MO	Emc3	Emc3	66087	ER membra	no|no chan	0	ENOG410X
Q99KI0	ACON_MC	Aco2	Aco2	11429	Aconitate h	no|no chan	0	COG1048(U
Q7TT23	DAAF9_MO	Dnaaf9	Dnaaf9	228602	Dynein axo	no|no chan	0	ENOG410X
O54917	E2F6_MOU	E2f6	E2f6	50496	Transcripti	no|no chan	0	ENOG410X
A0A1W2P6	A0A1W2P6	Sgta	Sgta		Small gluta	no|no chan	0	COG0457(r
O54918	B2L11_MO	Bcl2l11	Bcl2l11	12125	Bcl-2-like p	no|no chan	0	ENOG410Y
Q3SXD3	HDDC2_M	Hddc2	Hddc2	69692	5'-deoxynuc	no|no chan	0	COG1896(M
A2RTT4	A2RTT4_M	Ube2n	Ube2n	93765	E2 ubiquiti	no|no chan	0	COG5078(r
Q8BLH7	HIRP3_MC	Hirip3	Hirip3	233876	HIRA-inter	yes|down	1	ENOG4111
A0A0G2JG	A0A0G2JG	Smarcd3	Smarcd3	66993	SWI/SNF r	no|no chan	0	COG5531(c
O88477	IF2B1_MO	Igf2bp1	Igf2bp1	140486	Insulin-like	no|no chan	0	ENOG410Z
A0A140LJ0	A0A140LJ0	Zzef1	Zzef1	195018	Zinc finger,	no|no chan	0	ENOG410X
A0A0G2JE	A0A0G2JE	Arhgap29	Arhgap29		Rho GTPas	no|no chan	0	ENOG410Y
A0A0G2JE	A0A0G2JE	Supt20	Supt20		SPT20 SAG	no|no chan	0	ENOG410Y
B7ZCC4	B7ZCC4_M	Atxn7l2	Atxn7l2		Ataxin 7-lik	no|no chan	0	ENOG4111
Q60590	A1AG1_MO	Orm1	Orm1	18405	Alpha-1-ac	no|up	0	ENOG4111

A0A1D5RM	A0A1D5RM	Dock9	Dock9	105445	Dedicator c	no|no chan	0	ENOG410X
A0A0G2JE	A0A0G2JE	Syde2	Syde2		Synapse def	no|no chan	0	ENOG410X
Q60598	SRC8_MOU	Cttn	Cttn	13043	Src substrat	no|no chan	0	ENOG410X
A0A1D5RM	A0A1D5RM	Pxn	Pxn		Paxillin	no|no chan	0	ENOG410X
Q0PD66	Q0PD66_M	Rab1b	Rab1b	76308	small monc	no|no chan	0	ENOG410X
P59266	FITM2_MC	Fitm2	Fitm2	228859	Acyl-coenzy	no|no chan	0	ENOG4111
Q3UZ18	ICE2_MOU	Ice2	Ice2	93697	Little elong	no|no chan	0	ENOG410Y
P70695	F16P2_MO	Fbp2	Fbp2	14120	Fructose-1,(	no|up	0	COG0158(I
P70697	DCUP_MC	Urod	Urod	22275	Uroporphyı	no|no chan	0	COG0407(C
P70698	PYRG1_M(	Ctps1	Ctps1	51797	CTP syntha	no|no chan	0	COG0504(C
P70699	LYAG_MC	Gaa	Gaa	14387	Lysosomal (	no|no chan	0	COG1501(C
Q3UZ17	Q3UZ17_M	Luc7l2	Luc7l2	192196	RNA-bindi	no|no chan	0	COG5200(S
Q8BMA3	CNKR3_M	Cnksr3	Cnksr3	215748	Connector (	no|no chan	0	ENOG411C
Q3B7Z2	OSBP1_MC	Osbp	Osbp	76303	Oxysterol-b	no|no chan	0	ENOG410X
Q8CF66	LTOR4_M(	Lamtor4	Lamtor4	66096	Ragulator c	no|no chan	0	ENOG4112
Q8CF60	ZN263_MC	Znf263	Znf263	74120	Zinc finger	no|down	0	COG5048(l
Q4KMS1	Q4KMS1_M	Trim44	Trim44	80985	Tripartite n	no|no chan	0	ENOG4111
P27790	CENPB_M	Cenpb	Cenpb	12616	Major centr	no|no chan	0	ENOG411C
D3YV64	D3YV64_M	Ecm1	Ecm1		Extracellul	no|up	0	ENOG4111
Q61425	HCDH_MC	Hadh	Hadh	15107	Hydroxyacy	no|no chan	0	COG1250(T
P43274	H14_MOUS	H1-4	H1-4	50709	Histone H1	no|no chan	0	ENOG4112
P43275	H11_MOUS	H1-1	H1-1	80838	Histone H1	no|no chan	0	ENOG4112
P43276	H15_MOUS	H1-5	H1-5	56702	Histone H1	no|no chan	0	ENOG4112
P43277	H13_MOUS	H1-3	H1-3	14957	Histone H1	no|no chan	0	ENOG4112
B1AZ14	B1AZ14_M	Cobll1	Cobll1		Cobl-like 1	no|no chan	0	ENOG4111
Q8CI32	BAG5_MO	Bag5	Bag5	70369	BAG family	no|no chan	0	ENOG4111
Q8CI33	C19L1_MO	Cwf19l1	Cwf19l1	72502	CWF19-like	no|no chan	0	ENOG410X
P18654	KS6A3_MC	Rps6ka3	Rps6ka3	110651	Ribosomal	no|no chan	0	ENOG410X
Q56A13	Q56A13_M	Commd7	Commd7	99311	Commd7 pı	no|no chan	0	ENOG4111
Q3U0Z8	Q3U0Z8_M	Sufu	Sufu	24069	Suppressor	no|no chan	0	ENOG410X
D3Z600	D3Z600_M	Pex5	Pex5		Peroxisoma	no|no chan	0	ENOG410X
Q8BZ03	KPCD2_M	Prkd2	Prkd2	101540	Serine/thre	no|no chan	0	ENOG410X
A0A3Q4EC	A0A3Q4EC	Ss18	Ss18		SS18, subun	no|no chan	0	ENOG4112
D0EX61	D0EX61_M	Mbnl2	Mbnl2	105559	Musclebline	no|no chan	0	ENOG410Z
E9Q5F9	SETD2_MC	Setd2	Setd2	235626	Histone-lys	no|no chan	0	COG2940(c
Q02819	NUCB1_M	Nucb1	Nucb1	18220	Nucleobinc	no|no chan	0	ENOG410X
Q99JR6	NMNA3_M	Nmnat3	Nmnat3	74080	Nicotinami	no|no chan	0	COG1057(C
B7ZWM7	B7ZWM7_	Ankrd11	Ankrd11	77087	Ankrd11 pr	no|no chan	0	ENOG411C
A0A2I3BQ	A0A2I3BQ	Ermard	Ermard		ER membrc	no|no chan	0	ENOG4111
Q3UVM5	Q3UVM5_	Map3k3	Map3k3	26406	Mitogen-ac	no|no chan	0	ENOG410X
Q9EPK2	XRP2_MO	Rp2	Rp2	19889	Protein XR	no|no chan	0	ENOG410Y
A0A0R4J08	A0A0R4J08	F3	F3	14066	Tissue factc	no|up	0	ENOG4111
Q8VCQ8	Q8VCQ8_M	Cald1	Cald1	109624	Caldesmon	no|no chan	0	ENOG410Z

A0A0R4J08	A0A0R4J08	Olfml3	Olfml3	99543	Olfactomec	no | no chan	0	ENOG4102
A0A0R4J08	A0A0R4J08	Uap1	Uap1	107652	UDP-N-ace	no | no chan	0	COG4284(u
Q8VCQ3	NRBF2_MO	Nrbf2	Nrbf2	641340	Nuclear rec	no | no chan	0	ENOG4111
A0A0R4J08	A0A0R4J08	Acadl	Acadl	11363	Long-chain	no | no chan	0	ENOG410X
F2Z461	HERC6_MO	Herc6	Herc6	67138	E3 ISG15--p	no | up	0	COG5184(A
Q8VIM9	IRGQ_MO	Irgq	Irgq	210146	Immunity-r	no | no chan	0	ENOG4111
Q3UPQ8	Q3UPQ8_M	Zhx3	Zhx3	320799	Zinc fingers	no | no chan	0	ENOG4102
G3UY70	G3UY70_M	L3mbtl2	L3mbtl2	214669	Lethal(3)mo	no | no chan	0	ENOG4102
Q8CBB9	RSAD2_MO	Rsad2	Rsad2	58185	S-adenosylm	no | up	0	ENOG410X
Q6P1G0	HEAT6_MO	Heatr6	Heatr6	217026	HEAT repe	no | no chan	0	ENOG410X
P41233	ABCA1_MO	Abca1	Abca1	11303	Phospholip	no | no chan	0	COG1131(r
P41230	KDM5C_M	Kdm5c	Kdm5c	20591	Lysine-spec	no | no chan	0	ENOG410X
A0A0R4J0A	A0A0R4J0A	Flt1	Flt1	14254	receptor pro	no | no chan	0	COG0515(I
O88895	HDAC3_M	Hdac3	Hdac3	15183	Histone dec	no | no chan	0	COG0123(I
F8VPQ2	ARI4A_MO	Arid4a	Arid4a	238247	AT-rich int	no | no chan	0	ENOG410Y
A0A0R4J0U	A0A0R4J0U	Trmt1l	Trmt1l	98685	TRMT1-like	no | no chan	0	COG1867(C
Q9D136	OGFD3_M	Ogfod3	Ogfod3	66179	2-oxoglutar	no | no chan	0	ENOG4102
A0A0R4J0U	A0A0R4J0U	Ppp4r2	Ppp4r2	232314	Protein pho	no | no chan	0	ENOG411C
P46938	YAP1_MOU	Yap1	Yap1	22601	Transcripti	no | no chan	0	COG5021(e
A0A0R4J0U	A0A0R4J0U	Commd5	Commd5	66398	COMM dor	no | no chan	0	ENOG4111
Q99JT9	MTND_MO	Adi1	Adi1	104923	Acireductor	no | no chan	0	COG1791(C
A0A087WQ	A0A087WQ	Tmem237	Tmem237		Transmemb	no | no chan	0	ENOG4112
Q9DBQ7	PACE1_MO	Scyl3	Scyl3	240880	Protein-asso	no | no chan	0	ENOG410X
A0A494B9E	A0A494B9E	Sdhaf2	Sdhaf2		Succinate d	no | no chan	0	COG2938(t
O08601	MTP_MOU	Mttp	Mttp	17777	Microsomal	no | no chan	0	ENOG410Y
O08600	NUCG_MO	Endog	Endog	13804	Endonuclec	no | no chan	0	COG1864(I
A7VMV2	A7VMV2_M	Cryl1	Cryl1	68631	L-gulonate	no | no chan	0	COG1250(V
P97770	THUM3_M	Thumpd3	Thumpd3	14911	tRNA (guan	no | no chan	0	COG0116(S
Q3UZH3	Q3UZH3_M	Mthfd1	Mthfd1	108156	C-1-tetrahy	no | no chan	0	COG0190(C
Q8BMS9	RASF2_MO	Rassf2	Rassf2	215653	Ras associa	no | no chan	0	ENOG410Y
Q8VEG0	CCD71_MO	Ccdc71	Ccdc71	72454	Coiled-coil	no | no chan	0	ENOG410Y
P26262	KLKB1_MO	Klkb1	Klkb1	16621	Plasma kall	no | no chan	0	COG5640(c
Q8VEG6	CNO6L_M	Cnot6l	Cnot6l	231464	CCR4-NOT	no | no chan	0	COG5239(I
E9Q9T6	E9Q9T6_M	Ddx55	Ddx55	67848	ATP-depen	no | no chan	0	ENOG410X
Q8BMS1	ECHA_MO	Hadha	Hadha	97212	Trifunction	no | no chan	0	COG1250(C
A2ACJ2	FP100_MO	Faap100	Faap100	71885	Fanconi an	no | no chan	0	ENOG410Y
Q3TVW1	Q3TVW1_M	Spart	Spart	229285	MIT domai	no | no chan	0	ENOG4102
A0A1W2P8	A0A1W2P8	Reep3	Reep3	28193	Receptor ex	no | no chan	0	COG5052(C
A9C491	A9C491_MO	Cul7	Cul7	66515	Cullin 7	no | no chan	0	ENOG410X
B2RXM7	B2RXM7_M	Sarnp	Sarnp	625193; 661	SAP domain	no | no chan	0	ENOG4111
B2RXM2	B2RXM2_M	Gm6793	Gm6793	627828	EG627828 p	no | no chan	0	COG0724(I
Q3U9A8	Q3U9A8_M	Sh3bgrl	Sh3bgrl	56726	SH3 domain	no | no chan	0	ENOG4111
Q80VJ2	SRA1_MOU	Sra1	Sra1	24068	Steroid rece	no | no chan	0	ENOG4112

Q3UG63	Q3UG63_M	Ascc2	Ascc2	75452	CUE domai	no	no chan	0	ENOG410
A0A2K6ED	A0A2K6ED	Dpep2	Dpep2		Dipeptidas	no	no chan	0	COG2355(
Q9CQ02	COMD4_M	Commd4	Commd4	66199	COMM dor	no	no chan	0	ENOG4111
Q9CQQ8	LSM7_MO	Lsm7	Lsm7	66094; 1000	U6 snRNA-	no	no chan	0	ENOG4111
A2A8E2	A2A8E2_M	Czib	Czib		CXXC mot	no	no chan	0	ENOG4111
A0A2K6ED	A0A2K6ED	Nudcd1	Nudcd1	67429	NudC dom	no	no chan	0	ENOG410
Q9CQQ4	GEMI2_M	Gemin2	Gemin2	66603	Gem-associ	no	no chan	0	ENOG410
Q9CQQ0	SMIM8_M	Smim8	Smim8	66291	Small integ	no	no chan	0	ENOG4111
A0A0R4J1C	A0A0R4J1C	Fbxl12	Fbxl12	30843	F-box and l	no	no chan	0	ENOG410
H7BX95	H7BX95_M	Srsf1	Srsf1		Serine/argi	no	no chan	0	COG0724(
H7BX91	H7BX91_M	Aig1	Aig1		Androgen-i	no	no chan	0	ENOG4111
Q9D799	FMT_MOU	Mtfmt	Mtfmt	69606	Methionyl-t	no	no chan	0	COG0223(
H7BX99	H7BX99_M	F2	F2		Prothrombi	no	up	0	COG5640(
A0A0R4J1C	A0A0R4J1C	Enpp1	Enpp1	18605	Ectonucleo	no	no chan	0	COG1524(
Q9D2Q3	PAAT_MO	Paat	Paat	68277	ATPase PA	no	no chan	0	ENOG4111
Q9D2Q2	TRM44_M	Trmt44	Trmt44	78890	Probable tF	no	no chan	0	ENOG410
A0A2X0SY	A0A2X0SY	ARHGAP2	ARHGAP2	268970	ARHGAP2	no	up	0	ENOG410
A2AS45	A2AS45_M	Pkp4	Pkp4		Plakophilin	no	no chan	0	ENOG410
A0A087WP	A0A087WP	Dstyk	Dstyk		Dual serine	no	up	0	COG0515(
A0A075B5F	A0A075B5F	Ighv2-2	Ighv2-2		Immunoglo	no	no chan	0	ENOG410
A0A075B5F	A0A075B5F	Igkv3-1	Igkv3-1		Immunoglo	no	down	0	ENOG4111
A0A075B5F	A0A075B5F	Ighg2b	Ighg2b		Immunoglo	no	up	0	ENOG410
A0A075B5F	A0A075B5F	Ighg3	Ighg3		Immunoglo	no	up	0	ENOG410
Q566J7	Q566J7_M	Igh	Igh		Igh protein	no	down	0	ENOG410
A0A075B5F	A0A075B5F	Ighm	Ighm		Immunoglo	no	up	0	ENOG4111
A0A1B0GC	A0A1B0GC	Kdelr1	Kdelr1		ER lumen p	no	no chan	0	COG5196(
F8WGR0	F8WGR0_M	Add1	Add1	11518	Adducin 1	no	no chan	0	COG0235(
A0A1B0GC	A0A1B0GC	Irf2	Irf2		Interferon r	no	no chan	0	ENOG4111
Q69ZW3	EHBP1_M	Ehbp1	Ehbp1	216565	EH domain	no	no chan	0	COG5069(
P61294	RAB6B_M	Rab6b	Rab6b	270192	Ras-related	no	no chan	0	ENOG410
B1AQH6	B1AQH6_M	Med1	Med1	19014	Mediator o	no	no chan	0	ENOG410
P61290	PSME3_M	Psme3	Psme3	19192	Proteasome	no	no chan	0	ENOG410
Q64735	CR1L_MO	Cr1l	Cr1l	12946	Complemer	no	no chan	0	ENOG410
Q64737	PUR2_MO	Gart	Gart	14450	Trifunction	no	no chan	0	COG0150(
Q3UM44	Q3UM44_M	Vamp5	Vamp5	53620	V-SNARE c	no	no chan	0	COG5143(
Q3UM45	PP1R7_MC	Ppp1r7	Ppp1r7	66385	Protein phc	no	no chan	0	COG4886(
D3YYT1	D3YYT1_M	Glyr1	Glyr1	74022	Cytokine-lil	no	no chan	0	COG2084(
D3YYT0	D3YYT0_M	Cdh2	Cdh2		Cadherin-2	no	no chan	0	ENOG410
P59729	RIN3_MO	Rin3	Rin3	217835	Ras and Ra	no	no chan	0	ENOG410
A0A087WS	A0A087WS	Tent5a	Tent5a	212943	polynucleot	no	no chan	0	ENOG410
Q923F1	Q923F1_M	Clns1a	Clns1a	12729	Methyloson	no	no chan	0	ENOG4111
Q8CC21	TTC19_MC	Ttc19	Ttc19	72795	Tetratricop	no	no chan	0	COG0457(
B2RTJ2	B2RTJ2_M	Kctd21	Kctd21	622320	Potassium c	no	no chan	0	ENOG410

O35459	ECH1_MO	Ech1	Ech1	51798	Delta(3,5)-I	no|no chan	0	ENOG410)
A2AQ25	SKT_MOU	Skt	Skt	208618	Sickle tail p	no|no chan	0	ENOG410Z
O35454	CLCN6_M(	Clcn6	Clcn6	26372	H(+)/Cl(-) (	no|no chan	0	COG0038(C
P21279	GNAQ_M(	Gnaq	Gnaq	14682	Guanine nu	no|no chan	0	ENOG410)
Q9CWG9	BL1S2_MO	Bloc1s2	Bloc1s2	73689	Biogenesis (	no|no chan	0	ENOG4111
Q9CWG8	NDUF7_M	Ndufaf7	Ndufaf7	73694	Protein arg	no|no chan	0	COG1565(N
O35450	FKBPL_M(	Fkbpl	Fkbpl	56299	FK506-binc	no|no chan	0	COG0457(c
O35451	ATF6B_M(	Atf6b	Atf6b	12915	Cyclic AMF	no|no chan	0	ENOG410Z
B2RTJ8	B2RTJ8_M(	Noct	Noct	12457	Nocturnin	yes|up	1	COG5239(S
Q9R1P3	PSB2_MOU	Psmb2	Psmb2	26445	Proteasome	no|no chan	0	COG0638(r
P31809	CEAM1_M	Ceacam1	Ceacam1	26365	Cell adhesi	no|up	0	ENOG410Y
Q3U548	Q3U548_M	Ugp2	Ugp2	216558	UTP--gluco	no|up	0	COG4284(F
Q3USU4	Q3USU4_M	Cyp7b1	Cyp7b1	13123	25-hydroxy(	no|no chan	0	COG2124(s
P31750	AKT1_MO	Akt1	Akt1	11651	RAC-alpha	no|no chan	0	ENOG410)
O55187	CBX4_MO	Cbx4	Cbx4	12418	E3 SUMO-p	no|no chan	0	ENOG410Z
Q8R2M2	TDIF2_MC	Dnttip2	Dnttip2	99480	Deoxynucle	no|no chan	0	ENOG410Z
Q3UHD6	SNX27_M(	Snx27	Snx27	76742	Sorting nex	no|no chan	0	ENOG410)
Q3UHD2	GFOD1_M	Gfod1	Gfod1	328232	Glucose-fru	no|no chan	0	COG0673(u
E9PV59	E9PV59_M	Inafm1	Inafm1	66300	InaF motif	yes|down	1	ENOG4112
E9PV58	E9PV58_M	Tnpo2	Tnpo2	212999	Transportir	no|no chan	0	ENOG410)
Q548B0	Q548B0_M	Rcc1l	Rcc1l	94254	RCC1-like (	yes|up	1	COG5184(F
P16406	AMPE_MC	Enpep	Enpep	13809	Glutamyl a	no|no chan	0	COG0308(i
Q8CD92	TTC27_MC	Ttc27	Ttc27	74196	Tetratricop	no|no chan	0	ENOG410)
Q9R045	ANGL2_M	Angptl2	Angptl2	26360	Angiopoieti	no|no chan	0	ENOG410Z
Q6P7W5	SEN2_MOU	Tsen2	Tsen2	381802	tRNA-splic	no|no chan	0	COG1676(s
P12242	UCP1_MOU	Ucp1	Ucp1	22227	Mitochondi	no|up	0	ENOG410)
P12246	SAMP_MO	Apcs	Apcs	20219	Serum amyl	no|up	0	ENOG410Y
Q68FD7	FNIP1_MC	Fnip1	Fnip1	216742	Folliculin-i	no|no chan	0	ENOG410Z
P70202	LXN_MOU	Lxn	Lxn	17035	Latexin	no|no chan	0	ENOG4111
P70207	PLXA2_M(	Plxna2	Plxna2	18845	Plexin-A2	no|no chan	0	ENOG410Z
P70206	PLXA1_M(	Plxna1	Plxna1	18844	Plexin-A1	no|no chan	0	ENOG410)
A0AA74KT	A0AA74KT	Chtop	Chtop		Chromatin	no|no chan	0	ENOG4111
Q8K2C9	HACD3_M	Hacd3	Hacd3	57874	Very-long-c	no|no chan	0	COG5198(S
Q3TJJ5	Q3TJJ5_M(	Prkci	Prkci	18759	Protein kin(	no|no chan	0	ENOG410Z
Q04887	SOX9_MO	Sox9	Sox9	20682	Transcripti(	no|no chan	0	ENOG410)
A0A171EBI	A0A171EBI	Rap1gap2	Rap1gap2		RAP1 GTP(	no|no chan	0	ENOG410)
Q8BNE1	TCAF1_M(	Tcaf1	Tcaf1	77574	TRPM8 chc	no|down	0	ENOG410Y
A0A0N4SW	A0A0N4SW	Sinhcaf	Sinhcaf		SIN3-HDA(	no|no chan	0	
P16546	SPTN1_M(	Sptan1	Sptan1	20740	Spectrin alp	no|no chan	0	COG5069(C
A0A2I3BPN	A0A2I3BPN	Taf4	Taf4		TATA-box	no|no chan	0	ENOG410)
Q9DB60	PXL2B_M(	Prxl2b	Prxl2b	66469	Prostamide	no|no chan	0	ENOG410Y
A0A0R4J0J	A0A0R4J0J	Recql4	Recql4	79456	DNA 3'-5' h	no|no chan	0	COG0514(I
A0A0R4J0J	A0A0R4J0J	Ints9	Ints9	210925	Integrator (	no|no chan	0	COG1236(c

A0A0R4J0J(	A0A0R4J0J(	Thoc5	Thoc5	107829	THO comp	no|no chan	0	ENOG410Y
A0A0R4J0J8	A0A0R4J0J8	Specc1l	Specc1l	74392	Cytospin-A	no|no chan	0	COG5069(T
P04247	MYG_MOU	Mb	Mb	17189	Myoglobin	no|no chan	0	COG1018(T
A3KGW5	GT253_MC	Cercam	Cercam	99151	Inactive gly	no|up	0	ENOG410X
A1A553	A1A553_M	Stk10	Stk10	20868	non-specifi	no|no chan	0	ENOG410X
D3YUS5	D3YUS5_M	Rasal2	Rasal2		RAS protei	no|no chan	0	ENOG410X
D3YUS1	D3YUS1_M	Prkag2	Prkag2		Protein kin	no|no chan	0	COG0517(C
A2AN37	A2AN37_M	Uck1	Uck1	22245	Uridine-cyt	no|no chan	0	COG0572(t
Q8C708	CP054_MOUSE	Transmembrane p		101602	Transmemb	no|no chan	0	ENOG4111
Q9WU79	PROD_MC	Prodh	Prodh	19125	Proline deh	no|no chan	0	COG0506(E
Q3U890	Q3U890_M	Hars1	Hars1	15115	Histidine--t	no|no chan	0	COG0124(E
G3X982	AOXC_MC	Aox3	Aox3	71724	Aldehyde o	no|down	0	COG4630(x
Q3U896	Q3U896_M	Mtmr9	Mtmr9	210376	Myotubula	no|no chan	0	ENOG410X
D3Z0M5	D3Z0M5_M	Gnb1l	Gnb1l		Guanine nu	no|no chan	0	COG2319(c
G3X987	GIMA9_M	Gimap9	Gimap9	317758	GTPase IM	no|down	0	ENOG4111
Q9WU78	PDC6I_MC	Pdcd6ip	Pdcd6ip	18571	Programme	no|no chan	0	ENOG410X
D3Z0M9	D3Z0M9_M	Ddx23	Ddx23	74351	RNA helico	no|no chan	0	ENOG410X
Q5SPW0	VPS54_MO	Vps54	Vps54	245944	Vacuolar p	no|no chan	0	ENOG410X
Q61655	DD19A_M	Ddx19a	Ddx19a	13680	ATP-depen	no|no chan	0	ENOG410X
Q545E6	Q545E6_M	Tsn	Tsn	22099	Translin	no|no chan	0	ENOG410X
P35585	AP1M1_M	Ap1m1	Ap1m1	11767	AP-1 compl	no|no chan	0	ENOG410X
A0A0A6YX	A0A0A6YX	Ighv1-18	Ighv1-18		Immunoglo	no|up	0	ENOG4111
A0PJK9	A0PJK9_M	5730455P16	5730455P16	70591	Protein Njn	no|no chan	0	ENOG410Z
Q8CGA0	PPM1F_M	Ppm1f	Ppm1f	68606	Protein phc	no|no chan	0	COG0631(r
P35278	RAB5C_M	Rab5c	Rab5c	19345	Ras-related	no|no chan	0	ENOG410Y
Q8R016	BLMH_MC	Blmh	Blmh	104184	Bleomycin l	no|no chan	0	COG3579(c
P35276	RAB3D_M	Rab3d	Rab3d	19340	Ras-related	no|no chan	0	ENOG410Z
A0A1G5SJ\	A0A1G5SJ\	OTTMUSW	OTTMUSW	20556	Schlafen fa	no|up	0	ENOG4111
Q3TQI7	TLS1_MOUSE	Splicing factor C9o		227707	Splicing fac	no|up	0	ENOG410X
Q8CHC8	ADNP2_M	Adnp2	Adnp2	240442	Activity-de	no|down	0	ENOG410Z
G3X8T2	G3X8T2_M	Zc3h18	Zc3h18	76014	Zinc finger	no|no chan	0	ENOG4111
G3X8T3	G3X8T3_M	Ctsa	Ctsa	19025	Carboxype	no|no chan	0	COG2939(c
A0A1Y7VM	A0A1Y7VM	Kidins220	Kidins220	77480	Kinase D-i	no|no chan	0	COG0666(r
G3X8T9	G3X8T9_M	Serpina3n	Serpina3n	20716	Serine (or c	no|up	0	COG4826(s
I6L9I7	I6L9I7_MC	Cbx3	Cbx3	12417	Cbx3 protei	no|no chan	0	ENOG4111
Q8VDH1	FBX21_MC	Fbxo21	Fbxo21	231670	F-box only	no|no chan	0	COG2912(E
P28271	ACOHC_M	Aco1	Aco1	11428	Cytoplasmi	no|no chan	0	COG1048(c
O89112	LANC1_M	Lancl1	Lancl1	14768	Glutathion	no|no chan	0	ENOG410X
O89113	IER5_MOU	Ier5	Ier5	15939	Immediate	no|no chan	0	ENOG4111
Q8K4H1	KFA_MOU	Afmid	Afmid	71562	Kynurenine	no|up	0	COG0657(p
Q80TN4	DJC16_MC	Dnajc16	Dnajc16	214063	DnaJ homo	no|no chan	0	COG0484(E
D3YVS7	D3YVS7_M	Cystm1	Cystm1		Cysteine-ric	no|up	0	ENOG410Y
Q3U0B3	DHR11_M	Dhrs11	Dhrs11	192970	Dehydroge	no|no chan	0	COG4221(s

Q8CG72	ADPRS_M(	Adprs	Adprs	100206	ADP-ribosy	no|no chan	0	COG1397(/
Q80SZ6	Q80SZ6_M	Nxf7	Nxf7	170722	Nuclear RN	no|no chan	0	ENOG410)
Q5K4Q9	Q5K4Q9_M	Tmt1a	Tmt1a	70152	AAM-B pro	no|down	0	ENOG4111
Q8BYW9	EOGT_MC	Eogt	Eogt	101351	EGF domai	no|no chan	0	ENOG410Z
A2AJG0	A2AJG0_M	Rpp38	Rpp38	227522	Ribonuclea	no|no chan	0	COG1358(p
Q3TKH5	Q3TKH5_M	Zcchc8	Zcchc8	70650	Zinc finger	no|no chan	0	ENOG410)
A0A0J9YT(	A0A0J9YT(	Eif2b4	Eif2b4		Eukaryotic	no|down	0	COG1184(t
P53395	ODB2_MO	Dbt	Dbt	13171	Lipoamide	no|no chan	0	COG0508(g
Q9EQG9	CERT_MO	Cert1	Cert1	68018	Ceramide tı	no|no chan	0	ENOG410)
Q9WV38	GTR5_MO	Slc2a5	Slc2a5	56485	Solute carri	no|no chan	0	COG0477(F
Q6VGS5	DAPLE_M	Ccdc88c	Ccdc88c	68339	Protein Da	no|no chan	0	ENOG410Y
Q3URR4	Q3URR4_M	Sgsh	Sgsh	27029	N-sulfogluc	no|no chan	0	COG3119(T
A2BE28	LAS1L_MC	Las1l	Las1l	76130	Ribosomal	no|no chan	0	ENOG4112
Q9WV35	ABEC2_M(	Apobec2	Apobec2	11811	C->U-editi	no|up	0	ENOG4111
F8VQ07	F8VQ07_M	Albfm1	Albfm1	622307	Albumin su	no|down	0	ENOG4111
P48379	RFX2_MO	Rfx2	Rfx2	19725	DNA-bindi	no|no chan	0	ENOG410)
F8VQ05	F8VQ05_M	Fryl	Fryl	72313	FRY like tr	no|no chan	0	ENOG410)
B2RT77	B2RT77_M	Polr1f	Polr1f	28071	DNA-direc	no|no chan	0	ENOG4111
P42932	TCPQ_MO	Cct8	Cct8	12469	T-complex	no|no chan	0	ENOG410)
Q3U2W7	Q3U2W7_M	Kras	Kras	16653	GTPase KR	no|no chan	0	COG1100(N
Q3U2W2	Q3U2W2_M	Mybbp1a	Mybbp1a	18432	Myb-bindin	no|no chan	0	ENOG410Y
B1AVZ0	UPP_MOU	Uprt	Uprt	331487	Uracil phos	no|no chan	0	COG0035(N
A0A0R4J14	A0A0R4J14	Pcgf1	Pcgf1	69837	Polycomb g	no|down	0	ENOG410)
B8JJX7	B8JJX7_MC	Smn1	Smn1		Survival mo	no|no chan	0	ENOG4111
A0A0R4J14	A0A0R4J14	Cdc6	Cdc6	23834	Cell divisio	no|no chan	0	COG1474(I
P26450	P85A_MOU	Pik3r1	Pik3r1	18708	Phosphatid	no|no chan	0	ENOG410)
Q9D6Z0	ALKB7_M(	Alkbh7	Alkbh7	66400	Alpha-keto	no|no chan	0	COG3145(F
D3Z5K6	D3Z5K6_M	Dnajc19	Dnajc19	67713	Mitochondı	no|no chan	0	COG2214(F
E9QQ96	E9QQ96_M	Obscn	Obscn	380698	Obscurin, c	no|up	0	ENOG410)
A0A5F8MP	A0A5F8MP	Tnxb	Tnxb	81877	Tenascin X	no|no chan	0	ENOG410Z
A4FUV6	A4FUV6_M	Met	Met	17295	Hepatocyte	no|no chan	0	COG0515(F
A4FUV8	A4FUV8_M	Gzmk	Gzmk	14945	Gzmk prote	yes|down	1	COG5640(p
A2BEG5	A2BEG5_M	Kdm4c	Kdm4c	76804	[histone H3	no|up	0	COG5141(C
Q9CRI6	Q9CRI6_M	Zfp511	Zfp511	69752	C2H2-type	yes|up	1	ENOG4111
Q4V9W2	SR1IP_MO	Srek1ip1	Srek1ip1	67288	Protein SRI	yes|up	1	ENOG4112
E9QAS4	E9QAS4_M	Chd4	Chd4		DNA helicc	no|no chan	0	COG0553(/
P58252	EF2_MOUS	Eef2	Eef2	13629	Elongation	no|no chan	0	COG0480(C
Q5SVT2	Q5SVT2_M	Trim16	Trim16	94092	Tripartite n	no|no chan	0	ENOG411(
E9PWG6	E9PWG6_M	Ncapg	Ncapg	54392	Non-SMC c	no|no chan	0	COG5218(N
Q4V9W8	Q4V9W8_M	Rab4b	Rab4b	19342	Ras-related	no|no chan	0	ENOG410)
M0QW57	M0QW57_M	Jsrp1	Jsrp1	71912	Junctional s	no|up	0	ENOG4111
E9QAS9	E9QAS9_M	Fmr1	Fmr1		Fragile X m	no|no chan	0	ENOG410Z
E9QKC6	E9QKC6_M	Trim2	Trim2	80890	Tripartite n	no|no chan	0	ENOG410)

Q3UXZ9	KDM5A_M	Kdm5a	Kdm5a	214899	Lysine-spec	no | no chan	0	ENOG410
Q3UKJ7	SMU1_MO	Smu1	Smu1	74255	WD40 repe	no | no chan	0	ENOG410
G3UYG6	G3UYG6_M	Gigyf2	Gigyf2	227331	GRB10 inte	no | no chan	0	ENOG4111
Q91WT4	DJC17_MO	Dnajc17	Dnajc17	69408	DnaJ homo	no | no chan	0	COG2214(
A0AA74KC	A0AA74KC	Sf3b2	Sf3b2		Splicing fac	no | no chan	0	COG5182(
J3QNW4	J3QNW4_M	Trappc13	Trappc13		Trafficking	no | no chan	0	ENOG410
B9EIX2	B9EIX2_M	Cep170b	Cep170b	217882	AW555464	no | up	0	ENOG410
A2AJI0	MA7D1_M	Map7d1	Map7d1	245877	MAP7 dom	no | no chan	0	ENOG410
Q9JJ66	CDC20_M	Cdc20	Cdc20	107995	Cell divisio	no | no chan	0	ENOG410
Q9JM96	BORG4_M	Cdc42ep4	Cdc42ep4	56699	Cdc42 effec	no | no chan	0	ENOG4111
Q8R4R6	NUP35_M	Nup35	Nup35	69482	Nucleopori	no | no chan	0	ENOG4111
Q8BN82	S17A5_MO	Slc17a5	Slc17a5	235504	Sialin	yes | down	1	ENOG410
Q8CC12	CDAN1_M	Cdan1	Cdan1	68968	Codanin-1	no | no chan	0	ENOG410
Q8BFR4	GNS_MOU	Gns	Gns	75612	N-acetylglu	no | no chan	0	COG3119(s
A0A0J9YU	A0A0J9YU	Stard13	Stard13		StAR relate	no | up	0	ENOG410
Q9CXJ4	MITOS_M	Abcb8	Abcb8	74610	Mitochondi	no | no chan	0	COG1132((
Q3TPX4	EXOC5_M	Exoc5	Exoc5	105504	Exocyst cor	no | no chan	0	ENOG410
Q14A61	Q14A61_M	Cenpl	Cenpl	70454	Centromere	no | no chan	0	ENOG410
E9Q2A6	E9Q2A6_M	Ptk2b	Ptk2b	19229	non-specifi	no | up	0	ENOG410
E0CZ72	E0CZ72_M	Kif2a	Kif2a		Kinesin-lik	no | no chan	0	COG5059(c
Q3TBW2	RM10_MO	Mrpl10	Mrpl10	107732	Large ribos	no | no chan	0	ENOG4111
Q3UPL0	SC31A_MC	Sec31a	Sec31a	69162	Protein trar	no | no chan	0	ENOG410
P56959	FUS_MOU	Fus	Fus	233908	RNA-bindi	no | no chan	0	ENOG4111
B2RPS1	B2RPS1_M	Rab5b	Rab5b	19344	small monc	no | no chan	0	ENOG410
Q3UI55	Q3UI55_M	Lrp5	Lrp5	16973	Low-densit	no | no chan	0	ENOG4111
A0A0N4SU	A0A0N4SU	Brpf1	Brpf1	78783	Bromodom	no | no chan	0	COG5076(I
Q3V206	Q3V206_M	Ptrh1	Ptrh1	329384	Peptidyl-tR	no | no chan	0	COG0193(
P12815	PDCD6_M	Pdcd6	Pdcd6	18570	Programme	no | no chan	0	ENOG410
G5E825	G5E825_M	Arhgef25	Arhgef25	52666	Rho guanin	no | no chan	0	ENOG410
G5E829	AT2B1_MC	Atp2b1	Atp2b1	67972	Plasma mer	no | no chan	0	ENOG410
P41778	PBX1_MO	Pbx1	Pbx1	18514	Pre-B-cell l	no | no chan	0	ENOG410
A2AEW0	A2AEW0_M	Tfe3	Tfe3	209446	Transcripti	no | no chan	0	ENOG410
Q99PV0	PRP8_MO	Prpf8	Prpf8	192159	Pre-mRNA	no | no chan	0	COG5178(
A0A2I3BR	A0A2I3BR	AI182371	AI182371		Expressed s	no | no chan	0	ENOG410
Q6NXL1	Q6NXL1_M	Sec24d	Sec24d	69608	SEC24 hom	no | no chan	0	COG5028(
Q9D6Z1	NOP56_M	Nop56	Nop56	67134	Nucleolar p	no | no chan	0	COG1498(
Q3TIV9	Q3TIV9_M	Ap1s2	Ap1s2	108012	AP comple	no | no chan	0	COG5030(
Q91Z40	GBP7_MO	Gbp7	Gbp7	229900	Guanylate-	no | up	0	ENOG410
Q9D1N9	RM21_MO	Mrpl21	Mrpl21	353242	Large ribos	no | no chan	0	ENOG4111
Q3TA13	Q3TA13_M	Synpo	Synpo	104027	Synaptopoc	no | up	0	ENOG4111
Q3TIV5	ZC3HF_M	Zc3h15	Zc3h15	69082	Zinc finger	no | no chan	0	COG5252(
Q6PGF7	EXOC8_M	Exoc8	Exoc8	102058	Exocyst cor	no | no chan	0	ENOG410
Q3TIV6	Q3TIV6_M	Kars1	Kars1	85305	Lysine--tR	no | no chan	0	COG1190(

Q91Z49	UIF_MOUS	Fyttd1	Fyttd1	69823	UAP56-inte	no|no chan	0	ENOG4111
Q8BFR5	EFTU_MO	Tufm	Tufm	233870	Elongation	no|no chan	0	COG0050(T
J3QNW0	J3QNW0_M	Dnmt1	Dnmt1	13433	DNA (cytos	no|up	0	COG0270(C
B8QI34	B8QI34_MO	Ppfia2	Ppfia2	327814	Liprin-alph	no|up	0	ENOG410X
Q3UD01	A7L3B_MO	Atxn7l3b	Atxn7l3b	382423	Ataxin-7-lik	no|no chan	0	ENOG4111
Q3UD03	Q3UD03_M	Npepl1	Npepl1	228961	Cytosol ami	no|no chan	0	COG0260(A
Q6ZPU9	KBP_MOU	Kifbp	Kifbp	72320	KIF-bindin	no|no chan	0	ENOG410X

KO_id	KO_name	paths	pfam	go	subloc	nr	kegg_genes	tf_id
K17086	TM9SF2_4		PF02990.21	GO:003303	plasma mem	NP_598608	mmu:99237	
K18066	TYW5		PF13621.12	GO:010252	cytoplasmic	NP_001032	mmu:68736	
DNR(transcription, DNA-depende			PF04538.17	GO:009007	cytoplasmic	NP_001162	mmu:66684	
XMV(Ectropic viral integration si			PF05399.16	GO:000557	mitochondr	NP_001028	mmu:14017	
K18681	DIS3L	mmu03018(	PF00773.24	GO:001679	mitochondr	BAD32588.	mmu:213550	
K18189	TACO1		PF01709.25	GO:003361	mitochondr	NP_081622	mmu:70207	
K09275	TFCP2		PF04516.20	GO:000097	cytoplasmic	BAE28214.	mmu:21422	CP2
K13377	TGFB3	mmu05200(	PF00688.24	GO:003288	ER	P17125.1(R	mmu:21809	
TYR(-)			PF02093.21	GO:004671	cytoplasmic	AMK48512	mmu:118567843	
K01834	PGAM, gpr	mmu05230(	PF00300.27	GO:004643	cytoplasmic	NP_061358	mmu:56012	
K21418	HMOX2	mmu04978(	PF01126.25	GO:000449	cytoplasmic	NP_001129	mmu:15369	
SH0(Pinin, desmosome associated			PF05918.17	GO:200026	cytoplasmic	NP_031492	mmu:11800	
K06751	MHC1	mmu05165(	PF00129.23	GO:000989	ER	NP_001185	mmu:15018	
K11499	CENPF		PF10481.14	GO:000810	cytoplasmic	NP_001074	mmu:108000	
K09561	STUB1, CH	mmu04141(	PF04564.21	GO:004590	cytoplasmic	NP_062693	mmu:56424	
K13706	ABHD14		PF12697.13	GO:008009	cytoplasmic	NP_083907	mmu:76491	
K01254	LTA4H	mmu00590(	PF01433.26	GO:000548	cytoplasmic	NP_032543	mmu:16993	
WD5(Lymphocyte antigen 86)			PF02221.20	GO:003210	extracellulo	NP_034875	mmu:17084	
K03906	F13B	mmu04610(	PF00084.26	GO:004423	extracellulo	NP_112441	mmu:14060	
5KF(Immunity-related GTPase fan			PF05049.19	GO:003563	cytoplasmic	NP_001039	mmu:620913	
K10085	EDEM2	mmu04141(	PF01532.26	GO:003315	Golgi appa	NP_663512	mmu:108687	
K06641	CHEK2	mmu04115(	PF00069.31	GO:004423	cytoplasmic	NP_001412	mmu:50883	
K06491	NCAM, CD	mmu05020(	PF07679.22	GO:004858	plasma mem	NP_001297	mmu:17967	
K01874	MARS, met	mmu00970(	PF09334.17	GO:001710	mitochondr	NP_001165	mmu:216443	
K24790	POSTN		PF02469.27	GO:001922	extracellulo	NP_001300	mmu:50706	
K25792	JMJD8		PF13621.12	GO:000989	ER	BAE28331.	mmu:72106	
K18739	BICD	mmu03250(	PF09730.14	GO:001593	cytoplasmic	NP_084067	mmu:76895	
K02963	RP-S18, MR	mmu03010(	PF01084.26	GO:001593	mitochondr	NP_081102	mmu:68735	
K05867	CDC25C	mmu05170(	PF00581.25	GO:011003	nuclear	NP_033990	mmu:12532	
K11857	USP47		PF19718.4(	GO:200123	nuclear	NP_001344	mmu:74996	
K05863	SLC25A4S,	mmu05208(	PF00153.32	GO:200123	cytoplasmic	NP_031476	mmu:11739	
K11841	USP10, UBP3		PF00443.35	GO:002262	cytoplasmic	EDL11619.	mmu:22224	
7ZQ(PleXin Domain containing)			PF01437.31	GO:001602	plasma mem	XP_006497	mmu:67448	
K14349	NCEH1, A	mmu04979(	PF07859.19	GO:000578	ER	NP_848887	mmu:320024	
K05868	CCNB1	mmu05170(	PF00134.28	GO:004863	cytoplasmic	NP_758505	mmu:268697	
K09534	DNAJC14		PF14901.11	GO:000810	cytoplasmic	BAC32320.	mmu:74330	
K12587	MTR3, EX(	mmu03018(	PF01138.27	GO:008009	nuclear	NP_082550	mmu:72544	
K05724	FGD5_6		PF00621.25	GO:000573	nuclear	BAD32435.	mmu:13998	
Chromosome 16 open reading frame			PF00919.26	GO:005134	mitochondr	NP_080152	mmu:66971	
K05734	PAK4	mmu04012(	PF00069.31	GO:004885	cytoplasmic	BAC98108.	mmu:70584	
K20161	DENND2		PF02141.26	GO:005503	nuclear	NP_001001	mmu:76954	
K10666	RNF5_185	mmu04141(	PF00097.31	GO:008009	cytoplasmic	NP_001277	mmu:193670	

K11310	GTF3C4, KAT12		PF12657.13	GO:000367	cytoplasmic	BAE29737.1	mmu:269252
K03419	CHPF2	mmu00532(	PF05679.22	GO:003020	ER	NP_598674	mmu:100910
K23285	GULP1, CE	mmu04148(	PF00640.29(	PID:Phospl	cytoplasmic	ABG91070.	mmu:70676
K04403	MAP3K7IP	mmu04010(	PF00481.26	GO:000578	cytoplasmic	NP_079885	mmu:66513
K10370	TMOD	mmu04820(	PF03250.19	GO:009951	cytoplasmic	NP_001033	mmu:50876
K04004	CFH, HF1	mmu04610(	PF00084.26	GO:004858	extracellula	AAA37415.	mmu:12628
K25703	VEZT		PF12632.12	GO:009859	nuclear	NP_766126	mmu:215008
K03911	SERPINC1,	mmu04610(	PF00079.25	GO:000759	extracellula	NP_001406	mmu:11905
K17702	SIPA1L2, SI	mmu04015(	PF11881.13	GO:000573	cytoplasmic	NP_001344	mmu:244668
K17543	PXK		PF00787.29	GO:000810	cytoplasmic	NP_001347	mmu:218699
K07876	RAB35, RA	mmu04144(	PF00071.28	GO:000810	cytoplasmic	NP_001013	mmu:77407
K08509	SNAP29	mmu04140(	PF12352.13	GO:000810	cytoplasmic	NP_075837	mmu:67474
K00710	GALNT	mmu00514(	PF00535.33	GO:003024	ER	NP_056551	mmu:14425
K02260	COX17	mmu04714(	PF05051.18	GO:000548	cytoplasmic	NP_001017	mmu:12856
K04743	CHST7	mmu00532(	PF00685.33	GO:003020	Golgi appa	NP_068361	mmu:60322
K02267	COX6B	mmu05208(	PF02297.22	GO:199035	cytoplasmic	NP_001138	mmu:110323
K02219	CKS1	mmu05200(	PF01111.24	GO:006157	cytoplasmic	NP_001119	mmu:66197
K11836	USP5_13, UBP14		PF00443.35	GO:000989	nuclear	NP_038728	mmu:22225
K25874	KIRREL, NEPH		PF13927.12	GO:009859	lysosomal	NP_001164	mmu:170643
K24169	THADA, ARMC13		PF10350.15	GO:000578	cytoplasmic	NP_001413	mmu:240174
K06826	NID	mmu04820(	PF07474.17	GO:003101	lysosomal	NP_032721	mmu:18074
K05757	ARPC1A_B	mmu04530(	PF23414.1(I	GO:009859	cytoplasmic	NP_062741	mmu:56443
K13050	PCSK9	mmu04979(	PF18459.6(I	GO:000425	extracellula	AAP31672.1	mmu:100102
K04482	RAD51	mmu03440(	PF08423.17	GO:001922	cytoplasmic	NP_035364	mmu:19361
			PF21975.1(ASNSD1-SE		nuclear	NP_001340	mmu:110599589
)N7Q(Cell cycle progression 1)				GO:004212	cytoplasmic	NP_001273	mmu:72278
K00670	NAA30, MAK3		PF00583.31	GO:003124	cytoplasmic	NP_081768	mmu:70646
K23617	ACSF2		PF00501.34	GO:000663	mitochondr	NP_722502	mmu:264895
K08341	GABARAP	mmu04137(	PF02991.22	GO:000810	cytoplasmic	NP_001181	mmu:93739
K00665	FASN	mmu01100(	PF00698.26	GO:004745	cytoplasmic	NP_032014	mmu:14104
K01764	HCCS	mmu00860(	PF01265.22	GO:004322	cytoplasmic	NP_001317	mmu:15159
K04679	MADHIP, S	mmu04144(	PF11979.13	GO:000810	cytoplasmic	NP_775568	mmu:218441
K06688	UBE2C, UE	mmu04120(	PF00179.31	GO:199023	cytoplasmic	NP_081061	mmu:68612
K10405	KIFC1, ncd	mmu04814(	PF00225.29	GO:009951	cytoplasmic	NP_001182	mmu:100502766
K18202	AGXT2L2	mmu00310(	PF00202.27	GO:007027	cytoplasmic	NP_082674	mmu:72947
K25216	UBAC2		PF00627.37	GO:003288	ER	NP_081137	mmu:68889
K24022	PLEKHO		PF00169.35	GO:000692	cytoplasmic	NP_694759	mmu:102595
K04441	P38	mmu05208(	PF00069.31	GO:003563	cytoplasmic	NP_038899	mmu:29857
K00710	GALNT	mmu00514(	PF00535.33	GO:003024	ER	NP_001153	mmu:14423
K17767	AIP, XAP2	mmu04934(	PF23322.1(I	GO:000810	cytoplasmic	NP_001263	mmu:11632
K07192	FLOT	mmu04910(	PF01145.30	GO:005080	cytoplasmic	NP_001397	mmu:14251
K06057	NUMBL	mmu04330(	PF06311.17	GO:002260	cytoplasmic	NP_035080	mmu:18223
K13176	THOC7	mmu03013(	PF05615.18	GO:001593	cytoplasmic	NP_001346	mmu:66231

K05750	NCKAP1, N	mmu04810(	PF09735.14	GO:005112	cytoplasmic	AAI27068.1	mmu:50884	
K6G(RPA interacting protein)			PF14766.11	GO:003450	cytoplasmic	NP_001239	mmu:69723	
K22937	LYST, CHS1		PF02138.23	GO:000810	cytoplasmic	NP_034878	mmu:17101	
QFW(Family with sequence similar			PF06625.16	(DUF1151:P	cytoplasmic	XP_021011	mmu:108168726	
K10392	KIF1	mmu04814(	PF00225.29	GO:190195	cytoplasmic	XP_036012	mmu:16562	
)AQ3(Tumor protein p63 regulated)			PF12456.14	GO:000548	cytoplasmic	NP_080664	mmu:67808	
K27488	TMEM43	mmu04820(	PF07787.17	GO:000563	peroxisoma	NP_083042	mmu:74122	
XPCC(ran binding protein)			PF00622.36	GO:003251	cytoplasmic	XP_006757	mmu:223918	
COMM domain containing 5)			PF13855.12	GO:000998	cytoplasmic	NP_077156	mmu:67144	
K10407	KLC	mmu05020(	PF13424.12	GO:000573	cytoplasmic	XP_021041	mmu:74764	
K16483	POC5			GO:000557	cytoplasmic	NP_080449	mmu:67463	
K06525	ITGB4, CD	mmu05165(	PF00362.24	GO:000716	plasma mer	XP_006532	mmu:192897	
K17404	MRPS25		PF05047.22	GO:001593	cytoplasmic	BAD21364.	mmu:64658	
K12186	FAM125	mmu04144(	PF10240.14	GO:001602	cytoplasmic	NP_780393	mmu:72543	
K22808	KANK		PF12796.13	GO:003304	cytoplasmic	NP_001391	mmu:107351	
			PF00103.25	GO:000514	ER	NP_035248	mmu:18812	
K25129	AHCTF1, E	mmu03013(	PF16687.11	GO:001593	nuclear	EDL13164.	mmu:22674	TF_others
K11593	ELF2C, AGO		PF02171.23	GO:003577	cytoplasmic	NP_700451	mmu:214150	
K00943	tmk, DTYM	mmu01100(	PF02223.23	GO:000921	mitochondr	NP_001099	mmu:21915	
K14283	TACC3, maskin		PF05010.19	GO:000367	cytoplasmic	NP_001035	mmu:21335	
K25006	RBM24_38		PF00076.28	GO:008009	mitochondr	BAE38891.	mmu:56190	
K10436	MAPRE		PF03271.22	GO:003111	cytoplasmic	NP_031922	mmu:13589	
K01348	PLAU	mmu05202(	PF00089.32	GO:000167	extracellulo	NP_032899	mmu:18792	
K04742	CHST12	mmu00532(	PF03567.20	GO:003020	cytoplasmic	BAE37408.	mmu:59031	
ZG21(Retroviral envelope protein)					ER	XP_030107	mmu:115489417	
Z7NH(-)			PF19532.5(I	GO:003308	ER	XP_006501	mmu:229473	
K04497	GRO, TLE	mmu04310(	PF03920.20	GO:003250	nuclear	XP_006511	mmu:21887	
K18327	REXO4, REX4		PF00929.31	GO:009030	nuclear	AAH60147.	mmu:227656	
K01065	AOAH		PF20825.2(S	GO:000663	extracellulo	AAI50768.1	mmu:27052	
K10203	ELOVL6	mmu01040(	PF01151.23	GO:000578	ER	XP_036046	mmu:170439	
Y9KV(transmembrane and coiled-co			PF10267.14	GO:000548	cytoplasmic	PNJ83439.1	mmu:319880	
K08516	YKT6	mmu04140(	PF13774.12	GO:000810	cytoplasmic	NP_062635	mmu:56418	
K23567	EMC6, TMEM93		PF07019.17	GO:000810	plasma mer	NP_001014	mmu:66048	
K23226	SATB2		PF16557.11	GO:008009	nuclear	NP_001345	mmu:21271	Homeobox
XUV5(zinc finger, CCHC domain co			PF13917.11	GO:004316	nuclear	EDL33600.	mmu:67966	
K02296	ARNTL, BM	mmu04728(	PF14598.12	GO:009008	nuclear	NP_001361	mmu:11865	bHLH
ZYMI(Chromosome 11 open reading			PF08925.16	GO:004316	nuclear	NP_001186	mmu:70984	
K06566	IFITM		PF04505.17	GO:001922	cytoplasmic	NP_001028	mmu:213002	
K04883	KCNAB2		PF00248.27	GO:000403	mitochondr	XP_015858	mmu:16498	
K10450	KLHL12, C3IP1		PF01344.31	GO:004820	cytoplasmic	NP_001408	mmu:24075	ZBTB
K18625	SHROOM		PF08687.16	GO:003243	cytoplasmic	NP_001035	mmu:208431	
CNJ(Fat storage-inducing transmem			PF10261.15	GO:001712	ER	NP_081084	mmu:68680	
K18422	MOV10		PF13087.12	GO:003577	mitochondr	XP_051021	mmu:17454	

Nucleoporin 43kDa)			PF01135.25	GO:199023	cytoplasmic	NP_898849	mmu:319263	
K27818	RIC8		PF10165.14	GO:000508	cytoplasmic	NP_001013	mmu:237422	
YRGB(ig heavy chain V-III region V			PF07686.23	GO:000548	lysosomal	DBA12464.1(TPA: IGHV7-3*01, pa		
K18624	MAEA, EMP		PF10607.14	GO:004846	cytoplasmic	NP_001008	mmu:59003	
K06553	VPREB, CD179a		PF07686.23(V-set:Immu		extracellula	AAA97383.	mmu:22363	
K06553	VPREB, CD179a		PF07686.23	GO:000367	ER	DBA12471.	mmu:22362	
K06553	VPREB, CD179a		PF07686.23	GO:000548	ER	DBA12474.	mmu:22363	
K06553	VPREB, CD179a		PF07686.23	GO:000367	ER	DBA12469.	mmu:22362	
K18695	GPCPD1	mmu05231(	PF03009.23	GO:000665	cytoplasmic	NP_001036	mmu:74182	
K14165	K14165		PF00782.25	GO:009008	ER	Q66GT5.1(	mmu:66461	
K01134	ARSA	mmu04142(	PF00884.28	GO:001989	extracellula	NP_033843	mmu:11883	
K25807	KLHDC2		PF24681.1(I	GO:199023	nuclear	XP_031213	mmu:69554	
K17255	GDI1_2		PF00996.24	GO:000810	cytoplasmic	NP_034403	mmu:14567	
K01131	STS	mmu00140(	PF00884.28	GO:004212	extracellula	NP_033319	mmu:20905	
K04528	SNCA	mmu05010(	PF01387.22	GO:005104	cytoplasmic	NP_001035	mmu:20617	
K12886	HNRNPK	mmu05203(	PF00013.35	GO:190216	nuclear	NP_001288	mmu:15387	
K13162	PCBP2	mmu04216(	PF00013.35	GO:005068	nuclear	NP_001122	mmu:18521	
K19480	ANO5, GD	mmu04148(	PF04547.17	GO:000522	cytoplasmic	NP_808362	mmu:233246	
Z2X0(-)			PF17687.6(I	GO:000579	cytoplasmic	NP_080156	mmu:668661	
K17920	SNX5_6_32	mmu04144(	PF00787.29	GO:000810	cytoplasmic	NP_081274	mmu:72183	
K12833	SF3B14	mmu03040(	PF00076.28	GO:002260	cytoplasmic	NP_001002	mmu:66055	
K05218	P2RX4	mmu04080(	PF00864.24	GO:000526	plasma mer	XP_006530	mmu:18438	
K04429	TAO	mmu04010(	PF00069.31	GO:005149	cytoplasmic	NP_001157	mmu:381921	
K16634	RNH1		PF13516.12	GO:005112	cytoplasmic	BAE26275.	mmu:107702	
K13625	FTL	mmu04978(	PF00210.30	GO:000032	cytoplasmic	BAE29597.	mmu:14325	
N1Z(N-myc (And STAT) interactor			PF07292.18	GO:008009	cytoplasmic	NP_001135	mmu:64685	
K23705	SASH1		PF12485.13	GO:190187	cytoplasmic	NP_780364	mmu:70097	
K05858	PLCB	mmu04750(	PF00388.25	GO:000578	cytoplasmic	NP_001342	mmu:18798	
K15262	BCP1, BCCIP		PF13862.11	GO:190291	cytoplasmic	NP_079668	mmu:66165	
K17573	LKAP, MARF1		PF19687.5(	GO:004885	cytoplasmic	Q8BJ34.3(R	mmu:223989	
K16309	MASTL, GW		PF00069.31	GO:009859	cytoplasmic	NP_080255	mmu:67121	
K13180	INTS6L, DDX26B		PF15300.11	GO:001618	cytoplasmic	EDL42146.	mmu:236790	
K19363	LITAF	mmu04142(	PF10601.15	GO:008009	nuclear	NP_064364	mmu:56722	zf-LITAF-li
K09569	FKBP2		PF00254.34	GO:001602	ER	NP_001159	mmu:14227	
K03017	RPB9, POL	mmu03420(	PF02150.21	GO:003277	nuclear	NP_001099	mmu:69920	
K09259	ANK3, AN	mmu05205(	PF00023.36	GO:003202	cytoplasmic	XP_036011	mmu:11735	
K20050	LRP3_10_12			GO:003802	cytoplasmic	ABS83531.1	mmu:65107	
K17868	DPH7, RRT2		PF24807.1(	GO:000573	cytoplasmic	NP_080320	mmu:67228	
K19674	WDR35, IFT121		PF24797.1(I	GO:004590	cytoplasmic	EDK98417.	mmu:74682	
Dermatopontin)			PF10609.15	GO:000810	cytoplasmic	NP_036085	mmu:26425	
K13526	ATP13A2		PF00122.25	GO:003315	plasma mer	XP_006539	mmu:74772	
K11375	ELP4		PF05625.17	GO:000802	cytoplasmic	NP_076365	mmu:77766	
K13523	AGPAT3_4	mmu00564(	PF16076.10	GO:000563	ER	NP_001391	mmu:28169	

K09560	ST13		PF18253.6(I	GO:001707	cytoplasmic	NP_001405	mmu:70356	
K04646	CLTC	mmu04721(	PF00637.26	GO:000810	cytoplasmic	XP_008995	mmu:67300	
K22990	TOR1		PF06309.17	GO:003450	ER	NP_598434	mmu:30934	
K13708	DOCK1	mmu04666(	PF23554.2(	GO:000548	cytoplasmic	NP_001028	mmu:330662	
K10435	MAP1LC	mmu04137(	PF02991.22	GO:009951	cytoplasmic	AAI10309.1	mmu:67443	
K03250	EIF3E, IN	mmu05160(	PF09440.15	GO:002260	nuclear	AAC53346.	mmu:16341	
K17419	MRPL38		PF01161.25	GO:001593	mitochondr	NP_077139	mmu:60441	
K16808	NUMA1		PF21670.2(I	GO:003111	nuclear	NP_001390	mmu:101706	
K24643	PRDM10		PF00096.32	GO:008009	nuclear	NP_001355	mmu:38206	zf-C2H2
K25476	CREG		PF13883.13	GO:003575	ER	NP_035934	mmu:433375	
K07207	TSC2	mmu05165(	PF03542.21	GO:005116	cytoplasmic	NP_001273	mmu:22084	
K07845	RRAD, RAD1		PF00071.28	GO:001707	cytoplasmic	O88667.1(R	mmu:56437	
K10152	CCND3	mmu05165(	PF00134.28	GO:001922	cytoplasmic	NP_001075	mmu:12445	
K11217	JAK1	mmu04550(	PF07714.23	GO:000810	cytoplasmic	NP_666257	mmu:16451	
K15590	TFEB	mmu04137(	PF15951.10	GO:008009	nuclear	BAC37377.	mmu:21425	bHLH
K24633	ANKMY2, ZMYND20,		PF12796.13	GO:004316	cytoplasmic	NP_666145	mmu:217473	
S9J(PEST proteolytic signal contair			PF15473.11	GO:004322	cytoplasmic	BAE38553.	mmu:76302	
K01197	hya	mmu04142(	PF01630.24	GO:003288	lysosomal	BAC39851.	mmu:15587	
K02432	FZD1_7, fz	mmu05165(	PF01534.23	GO:001922	plasma mer	NP_032083	mmu:14369	
K11321	BRD8	mmu03082(	PF00439.30	GO:003124	nuclear	NP_084423	mmu:78656	
K15296	NAPA, SN	mmu04721(	PF14938.12	GO:000548	cytoplasmic	NP_080174	mmu:108124	
K25442	NCOA7	mmu04142(	PF07534.21	GO:000548	nuclear	NP_001345	mmu:211329	
K26728	ZNF292		PF00096.32	GO:008009	nuclear	NP_038917	mmu:30046	zf-C2H2
K17332	NFATC2, N	mmu04921(	PF16179.11	GO:000097	cytoplasmic	NP_001278	mmu:18019	RHD
K16855	NUDT16	mmu01100(	PF22327.2(	GO:004643	cytoplasmic	NP_083661	mmu:75686	
K24787	RUNDC3		PF02759.24(RUN:RUN		nuclear	NP_001334	mmu:242819	
K06013	STE24	mmu00900(	PF16491.10	GO:200073	ER	NP_766288	mmu:230709	
K25838	MYO19	mmu04814(	PF00063.27	GO:003202	cytoplasmic	NP_079690	mmu:66196	
K00109	L2HGDH	mmu00650(	PF01266.30	GO:004754	mitochondr	NP_663418	mmu:217666	
Y3Q0(CRAL TRIO domain protein)			PF00650.25	GO:000548	cytoplasmic	NP_084006	mmu:76763	
K11291	NASP		PF13424.12	GO:003472	cytoplasmic	NP_058057	mmu:50927	
XSX1(ankyrin repeat domain 40)			PF13637.12(Ank_4:Ank		cytoplasmic	NP_082075	mmu:71452	
YTCM(pleckstrin homology domain			PF00169.35(PH:PH don		cytoplasmic	NP_766229	mmu:226245	
K22610	RSBN1, KDM9			GO:004316	nuclear	NP_001074	mmu:242860	
K06553	VPREB, CD179a		PF07686.23	GO:000382	lysosomal	DBA12461.	mmu:22362	
K24181	LRBA		PF15787.11	GO:000582	cytoplasmic	NP_001071	mmu:80877	
K02266	COX6A	mmu05208(	PF02046.20	GO:001545	mitochondr	NP_034073	mmu:12862	
K14379	ACP5	mmu05323(	PF00149.33	GO:001922	lysosomal	NP_001095	mmu:11433	
K17520	CLEC3B		PF00059.27	GO:003028	extracellulo	P43025.2(R	mmu:21922	
K02266	COX6A	mmu05208(	PF02046.20	GO:004322	mitochondr	NP_031774	mmu:12861	
SHF(chromosome X open reading f			PF04669.18	GO:000573	cytoplasmic	NP_080588	mmu:67683	
K27082	SORC2		PF15902.11	GO:009859	plasma mer	BAC41477.	mmu:81840	
K01880	GARS, glyS	mmu00970(	PF03129.25	GO:003042	cytoplasmic	NP_851009	mmu:353172	

K09228	KRAB		PF13465.12	GO:000097	cytoplasmic	NP_001361	mmu:23595	zf-C2H2
K09554	CDC37	mmu04151(	PF08565.16	GO:009859	cytoplasmic	NP_058022	mmu:12539	
K19469	FTO		PF12933.13	GO:003577	cytoplasmic	NP_036066	mmu:26383	
K04855	CACNA1H	mmu04010(	PF00520.37	GO:000526	cytoplasmic	NP_067390	mmu:58226	
K17554	PPP1R13B, ASPP1		PF21801.3(	GO:009719	cytoplasmic	NP_035755	mmu:21981	
K13163	MSL1		PF15275.11	GO:007248	nuclear	NP_082998	mmu:74026	
K23600	TARDBP, T	mmu05014(	PF00076.28	GO:009008	nuclear	ACV52544.	mmu:230908	
K20406	NPRL3, NF	mmu04150(	PF03666.18	GO:000989	cytoplasmic	NP_853547	mmu:17168	
Yae1 domain containing 1)			PF00248.27	GO:000810	cytoplasmic	XP_006516	mmu:116852	
K13411	DUOX, TH	mmu04918(	PF03098.20	GO:009859	plasma mer	NP_001349	mmu:214593	
K01539	ATP1A	mmu04971(	PF00122.25	GO:000578	cytoplasmic	NP_659149	mmu:11928	
K13822	HBA	mmu05144(	PF00042.28	GO:000534	cytoplasmic	BAC36159.1	mmu:15122	
Family with sequence similarity 117,			PF00096.32	GO:000097	nuclear	NP_001028	mmu:31974	zf-C2H2
K08077	NUDT14		PF00293.34	GO:001679	cytoplasmic	NP_001303	mmu:66174	
K10427	DCTN5	mmu05022(	PF21711.3(I	GO:000328	cytoplasmic	NP_001032	mmu:59288	
K27408	SPTA		PF00435.27	GO:003243	cytoplasmic	NP_035595	mmu:20739	
K00759	APRT, apt	mmu01100(	PF00156.33	GO:000759	cytoplasmic	NP_033828	mmu:11821	
K07909	RAB17	mmu04148(	PF00071.28	GO:000810	cytoplasmic	NP_001153	mmu:19329	
K01112	PHPT1		PF05005.20	GO:000548	cytoplasmic	NP_083569	mmu:75454	
K01969	MCCC2, ac	mmu00280(	PF01039.27	GO:000367	mitochondr	NP_084302	mmu:78038	
K14077	TPCN2	mmu04972(	PF00520.37	GO:000526	cytoplasmic	AAI41196.1	mmu:233979	
K12796	ERBIN, ER	mmu04621(	PF23598.1(I	GO:000810	cytoplasmic	NP_001005	mmu:59079	
K17777	TIM9		PF02953.20	GO:001602	cytoplasmic	NP_001020	mmu:30056	
K20792	NAA15_16		PF12569.14	GO:003124	cytoplasmic	NP_080108	mmu:66897	
K09228	KRAB		PF13465.12	GO:000097	cytoplasmic	NP_001001	mmu:40806	zf-C2H2
K10320	FBXO46		PF12937.12(F-box-like:I		mitochondr	EDL23110.	mmu:243867	
K22414	POLDIP3		PF00076.28	GO:001593	nuclear	NP_848742	mmu:73826	
K24309	TET2_3		PF12851.13	GO:008009	nuclear	NP_001334	mmu:194388	
K24943	TTC39		PF10300.15	GO:008009	cytoplasmic	NP_081514	mmu:69863	
K22990	TOR1		PF06309.17	GO:000810	ER	NP_659133	mmu:30931	
K17350	TSPAN9		PF00335.26	GO:004239	plasma mer	NP_001343	mmu:109246	
K12827	SF3A3, SAF	mmu03040(	PF11931.14	GO:002260	nuclear	EDL30356.	mmu:75062	
K17779	TIM10B		PF02953.20	GO:000810	cytoplasmic	NP_001346	mmu:14356	
K17926	SNX14		PF02194.20	GO:001602	ER	Q8BHY8.2(	mmu:244962	
K16938	SEPT3_9_1	mmu05100(	PF00735.24	GO:003243	cytoplasmic	XP_006533	mmu:53860	
K06729	PTGFRN, CD315		PF07686.23	GO:000989	ER	NP_035327	mmu:19221	
K24964	KIAA1109		PF25040.1(I	GO:000531	plasma mer	NP_766267	mmu:229227	
XS2I(transmembrane and coiled-coi			PF23565.1(	GO:004518	plasma mer	NP_766625	mmu:272538	
K14427	SLC12A4_6, KCC1_3		PF00324.27	GO:000682	cytoplasmic	NP_001240	mmu:20498	
K03231	EEF1A	mmu03013(	PF00009.33	GO:004316	cytoplasmic	NP_031932	mmu:13628	
K16938	SEPT3_9_1	mmu05100(	PF00735.24(Septin:Sept		cytoplasmic	NP_001355	mmu:24050	
K13575	SLC4A4, N	mmu04964(	PF00955.27	GO:009859	cytoplasmic	NP_001346	mmu:54403	
)247(inositol 1,4,5-trisphosphate rec			PF20266.3(	GO:190204	ER	NP_001001	mmu:414801	

K08831	SRPK2		PF00069.31	GO:003506	nuclear	NP_001346	mmu:20817	
K05752	C3ORF10, I	mmu04810(Regulation		GO:000806	cytoplasmic	NP_001182	mmu:101314	
K10435	MAP1LC	mmu04137(	PF02991.22	GO:009951	cytoplasmic	NP_001248	mmu:66734	
K00036	G6PD, zwf	mmu05230(	PF02781.21	GO:008009	cytoplasmic	BAE38077.	mmu:14381	
K13177	DDX1		PF00270.35	GO:000810	cytoplasmic	NP_598801	mmu:104721	
K12373	HEXA_B	mmu01100(	PF00728.28	GO:008009	lysosomal	NP_034552	mmu:15212	
K21098	DLG3	mmu05165(	PF00625.27	GO:000810	cytoplasmic	NP_001171	mmu:53310	
K10848	ERCC4, XP	mmu03420(	PF02732.21	GO:008009	cytoplasmic	NP_056584	mmu:50505	
KS1Q(Transmembrane protein 87A)			PF06814.19	GO:007126	ER	NP_001103	mmu:211499	
K03246	EIF3I		PF24805.1(	GO:002260	cytoplasmic	NP_061269	mmu:54709	
K13577	SLC25A10,	mmu04964(	PF00153.32	GO:001572	mitochondr	NP_038798	mmu:27376	
K08450	ADGRG1, GPR56		PF18587.6(	GO:005084	extracellulo	EDL11154.	mmu:14766	
K13341	PEX7, PTS	mmu04146(	PF00400.38	GO:003444	peroxisoma	NP_001155	mmu:18634	
K15027	EIF2D		PF17832.7(	GO:002260	cytoplasmic	XP_006529	mmu:16865	
K10375	TPM4	mmu04260(	PF00261.25	GO:009951	cytoplasmic	NP_001258	mmu:326618	
K11471	ASXL	mmu03083(	PF13919.11	GO:008009	nuclear	NP_001035	mmu:228790	
K03662	ATPeVS1,	mmu05165(	PF05827.17	GO:000810	plasma mer	BAE40053.	mmu:54411	
K20794	NAA40, NAT4		PF00583.31	GO:006173	cytoplasmic	XP_053416	mmu:70999	
K07753	PECR	mmu04146(	PF00106.31	GO:000663	peroxisoma	BAE22624.	mmu:111175	
K24724	AES, TLE5		PF03920.20	GO:008009	mitochondr	NP_001263	mmu:14797	
K26519	SCIMP		PF15050.11	GO:001922	plasma mer	NP_001038	mmu:327957	
K10695	RNF1_2	mmu03083(	PF16207.11	GO:008009	nuclear	AIC84004.1	mmu:19763	
K24928	TTC5		PF16669.11	GO:000989	nuclear	BAC32087.	mmu:219022	
K06528	LAMP1_2,	mmu04140(	PF01299.22	GO:000810	ER	NP_034814	mmu:16783	
K19989	TNFAIP2, EXOC3L3		PF06046.19	GO:003015	cytoplasmic	AAA72044.	mmu:21928	
K17707	DOCK5		PF23554.2(	GO:004590	cytoplasmic	NP_808448	mmu:68813	
K23449	TRDN	mmu04260(	PF05279.16	GO:000578	plasma mer	AAL33878.	mmu:76757	
K22156	JADE3		PF13832.12	GO:003124	nuclear	NP_001393	mmu:382207	
K11829	RCOR1, C	mmu05016(	PF20878.2(	GO:000548	nuclear	AAH42731.	mmu:21786	MYB
K11658	BAZ1B, WS	mmu03082(	PF10537.14	GO:190521	nuclear	NP_035844	mmu:22385	
K11542	CASC5, KN	mmu04110(	PF19221.5(	GO:000810	cytoplasmic	NP_083893	mmu:76464	
K10424	DCTN2	mmu05022(	PF04912.19	GO:000810	cytoplasmic	NP_081427	mmu:69654	
K11221	STAT2	mmu05165(	PF01017.26	GO:001922	cytoplasmic	BAE31965.	mmu:20847	STAT
K19475	WIPF	mmu05135(	PF02205.27	GO:000573	cytoplasmic	NP_922922	mmu:68524	
K26269	SFMBT	mmu03083(	PF02820.24	GO:005124	nuclear	NP_001160	mmu:54650	
ZJGQ(Zinc finger protein)				GO:009951	nuclear	NP_001123	mmu:22680	TF_others
K24806	ZBTB35, ZNF131		PF00651.37	GO:000097	cytoplasmic	EDL18331.	mmu:72465	ZBTB
K12403	AP4S1	mmu04142(	PF01217.26	GO:000810	cytoplasmic	NP_068356	mmu:11782	
K01800	maiA, GST	mmu00350(	PF02798.26	GO:001975	mitochondr	NP_034493	mmu:14874	
22WA(ArfGAP with FG repeats)			PF01412.23	GO:002260	cytoplasmic	BAE42210.	mmu:231801	
K12363	RASGRP4	mmu05200(	PF00617.24	GO:000548	cytoplasmic	NP_660131	mmu:233046	
K12462	ARHGDI, I	mmu04962(	PF02115.23	GO:005104	cytoplasmic	NP_001007	mmu:192662	
K12189	VPS25, EAI	mmu04144(	PF05871.18	GO:003251	nuclear	NP_081052	mmu:28084	

K20091	CHD2		PF00176.29	GO:008009	nuclear	NP_001074	mmu:244059	
.NG4(SR-related CTD-associated fo			PF23030.1(	GO:002260	cytoplasmic	EDL04241.	mmu:72193	
K17790	TIM22		PF02466.24	GO:000810	cytoplasmic	NP_062792	mmu:56322	
K07401	K07401		PF10262.15	GO:000989	cytoplasmic	NP_079835	mmu:103742	
K05726	BCAR1, CA	mmu04670(	PF12026.14	GO:004858	cytoplasmic	BAE39759.	mmu:12927	
K27759	MTNAP1			GO:003204	cytoplasmic	NP_001348	mmu:276852	
Family with sequence similarity 3, m			PF00248.27	GO:000403	cytoplasmic	NP_001013	mmu:432720	
K10596	UBE4A	mmu04120(	PF10408.14	GO:000815	cytoplasmic	NP_001333	mmu:140630	
K15254	HELB		PF13604.12	GO:008009	cytoplasmic	NP_536694	mmu:117599	
K01099	INPP5B_F	mmu04070(	PF22669.2(	GO:003126	cytoplasmic	NP_796189	mmu:320634	
K27588	RHBDF	mmu04668(	PF12595.13	GO:000425	cytoplasmic	NP_034247	mmu:13650	
K09518	DNAJB12	mmu04141(	PF09320.16	GO:001602	nuclear	NP_001361	mmu:56709	
K17434	MRPL53		PF10780.14	GO:001593	mitochondr	NP_081020	mmu:68499	
K12472	EPS15	mmu04144(	PF12763.14	GO:000810	cytoplasmic	XP_006502	mmu:13858	
K00653	CDY		PF00378.25	GO:014003	nuclear	BAE24934.	mmu:75796	
K24834	GLIPR1		PF00188.32	GO:000557	plasma mem	NP_082884	mmu:73690	
K07526	SRGAP	mmu04360(	PF00620.32	GO:003444	cytoplasmic	NP_001074	mmu:14270	
K23387	GET4	mmu03060(	PF04190.19	GO:000810	cytoplasmic	NP_080545	mmu:67604	
K18161	NDUFAF4	mmu04714(	PF06784.16	GO:000548	cytoplasmic	NP_081018	mmu:68493	
K09391	E2F7_8		PF02319.26	GO:006071	nuclear	NP_001345	mmu:52679	E2F
K06553	VPREB, CD179a		PF07686.23	GO:000548	cytoplasmic	AAL09421.	mmu:22363	
YDSX(chromosome 2 open reading f			PF02862.22	GO:002260	cytoplasmic	AAI16305.1	mmu:207352	
K23286	NEBL1_2		PF16057.10	GO:000557	cytoplasmic	NP_775620	mmu:269198	
K17307	FBLN1_2	mmu04820(	PF07645.21	GO:005084	extracellula	NP_032018	mmu:14115	
K03236	EIF1A		PF01176.25	GO:008009	cytoplasmic	NP_001004	mmu:66235	
XR06(Sodium hydrogen exchanger)			PF06309.17	GO:000563	cytoplasmic	NP_666227	mmu:227612	
K26735	KEA4_5_6, TMCO3, yb		PF00999.27	GO:002289	ER	NP_758486	mmu:234076	
.54J(major urinary protein)			PF00061.29	GO:009008	ER	NP_001039	mmu:17841	
.54J(major urinary protein)			PF00061.29	GO:009008	ER	NP_001334	mmu:100041687	
K12409	GNE	mmu00520(	PF02350.25	GO:001920	cytoplasmic	XP_006538	mmu:50798	
K10899	RECQL		PF00270.35	GO:007110	nuclear	NP_001191	mmu:19691	
K13863	SLC7A1, A	mmu05206(	PF13520.12	GO:004858	plasma mem	NP_001288	mmu:11987	
K10636	AMFR, GP	mmu04141(	PF18442.6(	GO:000578	extracellula	NP_035917	mmu:23802	
K00016	LDH, ldh	mmu05230(	PF00056.29	GO:005128	cytoplasmic	BAE41969.	mmu:16828	
K14079	PAPD4, GLD2		PF22600.2(	GO:007104	cytoplasmic	NP_001348	mmu:100715	
K13118	DGCR14		PF09751.14	GO:000613	nuclear	BAC40400.	mmu:27886	
K00164	OGDH, suc	mmu01200(	PF00676.26(E1_dh:Deh		mitochondr	NP_001074	mmu:239017	
K01227	ENGASE	mmu00511(	PF03644.19	GO:001678	cytoplasmic	NP_766161	mmu:217364	
K06685	MOB1, Ma	mmu04390(	PF03637.22	GO:000548	cytoplasmic	NP_001101	mmu:68473	
K02925	RP-L3e, RP	mmu05171(	PF00297.28	GO:001593	mitochondr	NP_001157	mmu:66211	
K18041	PTP4A		PF00102.32	GO:001679	cytoplasmic	NP_001098	mmu:19244	
K05668	ABCC5	mmu02010(	PF00664.28	GO:003197	cytoplasmic	NP_038818	mmu:27416	
K25858	ZNF410			GO:008009	cytoplasmic	KAI257196	mmu:52708	

K11267	PDS5	mmu04110(	PF20168.4(I	GO:004423	nuclear	NP_001074	mmu:71521	
K20161	DENND2		PF02141.26	GO:000810	cytoplasmic	AAI45600.1	mmu:209773	
K08758	APOA2	mmu04979(	PF04711.18	GO:004643	ER	ABV02576.	mmu:11807	
K11489	PCGF5	mmu03083(	PF16207.11	GO:008009	cytoplasmic	AAI45592.1	mmu:76073	
SPT2, Suppressor of Ty, domain con			PF21276.3(Z	GO:004316	nuclear	BAD32545.	mmu:26963	zf-C2H2
K20129	ANKFY1		PF12796.13	GO:000554	cytoplasmic	AAI45584.1	mmu:11736	ZBTB
K18271	RBP4		PF00061.29	GO:009008	cytoplasmic	NP_001152	mmu:19662	
K24639	ZBED3			GO:001922	cytoplasmic	BAE22645.	mmu:72114	
K14800	TSR2		PF10273.14	GO:001607	nuclear	NP_001158	mmu:69499	
XQWI(Cytochrome B561, N terminc			PF09786.14	GO:000579	Golgi appa	NP_848740	mmu:72649	
K04707	CBL	mmu04012(	PF02262.21	GO:000688	cytoplasmic	NP_031645	mmu:12402	
K11448	KDM6B, JMJD3		PF02373.28	GO:008009	cytoplasmic	NP_001017	mmu:216850	
K02974	RP-S24e, Rl	mmu05171(	PF01282.24	GO:003204	cytoplasmic	BAE24039.	mmu:225058	
K17584	PCIF1		PF12237.13	GO:000989	nuclear	NP_001361	mmu:228866	
K00383	GSR, gor	mmu01100(	PF07992.20	GO:003459	mitochondr	NP_034474	mmu:14782	
K17501	PPM1E, POPX1		PF00481.26	GO:004423	cytoplasmic	NP_796141	mmu:320472	
K16495	PCDHGA		PF00028.23	GO:004823	plasma mer	BAC26363.	mmu:93717	
K23354	LIMS1_2, PINCH1_2		PF00412.28	GO:000591	cytoplasmic	ABV30674.	mmu:110829	
K01908	ACSS3, prp	mmu00640(	PF00501.34	GO:001564	mitochondr	NP_001136	mmu:380660	
K12186	FAM125	mmu04144(	PF10240.14	GO:004323	nuclear	NP_082893	mmu:73711	
K25470	CFAP20		PF05018.18	GO:002260	cytoplasmic	NP_001003	mmu:14894	
XPB2(HID1 domain containing)			PF12722.12	GO:000573	cytoplasmic	EDL34478.	mmu:217310	
F4U(SR-related CTD-associated fa			PF23030.1(S	GO:003277	nuclear	NP_001008	mmu:233208	
K14408	CSTF3, RN	mmu03015(	PF05843.20	GO:009030	nuclear	NP_663504	mmu:228410	
K25375	BPIFA, SPLUNC		PF01273.31	GO:004322	extracellula	NP_032979	mmu:19194	
K14399	CLP1, HER	mmu03015(	PF16575.11	GO:001922	cytoplasmic	NP_001009	mmu:98985	
K00710	GALNT	mmu00514(	PF00535.33	GO:003024	cytoplasmic	BAE30483.	mmu:108148	
K18458	ACY3		PF24827.1(	GO:000404	cytoplasmic	NP_001289	mmu:71670	
K11650	SMARCD	mmu04714(	PF02201.23	GO:003506	nuclear	NP_114030	mmu:83797	
K07918	RAB32		PF00071.28	GO:000810	cytoplasmic	NP_080681	mmu:67844	
YJ0T(chromosome 5 open reading fr			PF22834.2(Polo_box_4:		cytoplasmic	NP_001107	mmu:67392	
K07921	RAB34		PF00071.28	GO:000858	cytoplasmic	NP_001152	mmu:19376	
K15731	CTDSP		PF03031.24	GO:000989	cytoplasmic	XP_006512	mmu:69274	
K16762	CEP57		PF14073.11	GO:000573	cytoplasmic	XP_036011	mmu:103268	
K21356	MFN1	mmu04621(	PF04799.18	GO:000167	cytoplasmic	NP_077162	mmu:67414	
K16643	SERPINE2	mmu04610(	PF00079.25	GO:005080	extracellula	NP_033281	mmu:20720	
K18616	AFAP1		PF00169.35	GO:003243	nuclear	EDL37501.	mmu:70292	
K03908	PROS1	mmu04148(	PF00054.30	GO:003250	ER	NP_035303	mmu:19128	
K00106	XDH	mmu00983(	PF20256.3(I	GO:004340	cytoplasmic	EDL38417.	mmu:22436	
K18467	VPS29	mmu04144(	PF12850.12	GO:000810	cytoplasmic	KAF640437	mmu:56433	
K16535	FOPNL, FOR20		PF09398.16(FOP_dime		cytoplasmic	XP_052052	mmu:66086	
K07359	CAMKK2	mmu04921(	PF00069.31	GO:001922	cytoplasmic	NP_001186	mmu:207565	
K01893	NARS, asnS	mmu00970(	PF00152.25	GO:003563	mitochondr	NP_705819	mmu:244141	

JPF0249 protein)			PF04794.17	GO:000028	cytoplasmic	NP_081216	mmu:69101
K22429	FGF21	mmu04010(	PF00167.24	GO:001922	extracellulo	NP_064397	mmu:56636
29XH(Chromosome 3 open reading frame 33)				GO:008009	cytoplasmic	NP_808524	mmu:329659
K10429	MAP1	mmu03250(	PF23415.1(	GO:003111	extracellulo	EDL28891.	mmu:270058
K04445	RPS6KA5, I	mmu04010(	PF00069.31	GO:001922	cytoplasmic	EDL18894.	mmu:73086
K25743	TWSG1		PF04668.17	GO:004213	extracellulo	NP_075540	mmu:65960
K07206	TSC1	mmu05165(	PF04388.17	GO:003514	nuclear	NP_001276	mmu:64930
K07874	RAB1A	mmu04140(	PF00071.28	GO:000810	cytoplasmic	NP_001003	mmu:19324
K07936	RAN	mmu03008(	PF00071.28	GO:005116	nuclear	NP_001029	mmu:19384
K15732	CTDP1, FCP1		PF09309.15	GO:000647	cytoplasmic	BAE33777.	mmu:67655
K23843	MICU3		PF13499.12	GO:000548	cytoplasmic	AAI25420.1	mmu:78506
K21456	GSS	mmu01240(	PF03917.23	GO:001003	cytoplasmic	NP_032206	mmu:14854
RK6(glia maturation factor)			PF00241.25	GO:005112	cytoplasmic	XP_013204	mmu:63985
2205(Transmembrane protein 14A)			PF03647.18	GO:001922	mitochondr	NP_001347	mmu:66154
K24498	SORL1		PF00057.23	GO:003315	plasma mer	NP_035566	mmu:20660
K07604	KRT1	mmu04915(	PF00038.26	GO:009951	nuclear	NP_034792	mmu:16663
K08342	ATG4	mmu04140(	PF03416.26	GO:003472	cytoplasmic	NP_705811	mmu:235040
K24754	WDR73			GO:009859	cytoplasmic	NP_082302	mmu:71968
K24848	IFIT2		PF13431.12	GO:000578	cytoplasmic	NP_001342	mmu:15958
K18723	GLE1	mmu03013(	PF07817.19	GO:001593	nuclear	NP_083199	mmu:74412
K05676	ABCD2, AI	mmu02010(	PF06472.20	GO:003444	mitochondr	NP_036124	mmu:26874
K24993	PSPC1		PF00076.28	GO:008009	nuclear	NP_079958	mmu:66645
K20358	GOLGA2		PF15070.11	GO:000810	cytoplasmic	Q921M4.3(	mmu:99412
K12196	VPS4	mmu03250(	PF00004.35	GO:000699	cytoplasmic	NP_033216	mmu:20479
K21289	PIK3CG	mmu04725(	PF19710.4(	GO:001922	cytoplasmic	BAE23366.	mmu:30955
K06027	NSF, SEC1	mmu04727(	PF00004.35	GO:000810	cytoplasmic	NP_032766	mmu:18195
conserved protein)			PF02586.19	GO:008009	cytoplasmic	NP_001405	mmu:232210
V4F(-)				GO:000573	cytoplasmic	AAH24394.1(Polymerase (RNA) I p	
K08583	CAPNS1, CAPN4		PF00036.39	GO:004212	cytoplasmic	NP_033925	mmu:12336
K15516	FMR		PF12235.13	GO:003577	cytoplasmic	XP_006151	mmu:23879
K24553	DPY19L		PF10034.15	GO:000578	ER	NP_848819	mmu:233115
K14678	LOXL1		PF01186.22	GO:003399	extracellulo	NP_034859	mmu:16949
KPS(Serum amyloid A-like 1)				GO:000828	cytoplasmic	NP_084509	mmu:78935
K12026	TRIM56		PF13445.12	GO:001922	cytoplasmic	NP_958761	mmu:384309
K14209	SLC36A, P/	mmu04974(	PF01490.24	GO:000534	plasma mer	NP_758493	mmu:234967
K20294	COG7		PF10191.15	GO:000810	cytoplasmic	EDL17254.	mmu:233824
K07931	RABL2		PF00071.28	GO:000548	cytoplasmic	NP_081093	mmu:68708
K19937	RAB3GAP2		PF14656.11	GO:190306	cytoplasmic	XP_006497	mmu:98732
K21894	CYGB		PF00042.28	GO:003444	cytoplasmic	NP_084482	mmu:114886
K04407	MAP4K4, I	mmu04010(	PF00069.31	GO:003563	nuclear	XP_006496	mmu:26921
K06107	EPB41		PF05902.18	GO:002260	cytoplasmic	NP_906273	mmu:269587
K04428	MAP3K4, M	mmu04010(	PF19431.4(	GO:006071	cytoplasmic	NP_036078	mmu:26407
K12173	BRE, BRCC	mmu03440(	PF06113.17	GO:008009	cytoplasmic	KAF64460	mmu:107976

K01425	glsA, GLS	mmu04724(	PF04960.20	GO:005128	mitochondr	NP_001074	mmu:14660	
K03939	NDUFS6	mmu05208(	PF10276.14	GO:004858	mitochondr	NP_035018	mmu:407785	
K12566	SGCB	mmu05410(	PF04790.18	GO:001601	cytoplasmic	NP_036020	mmu:24051	
K12564	SGCG	mmu05410(	PF04790.18	GO:004873	cytoplasmic	NP_036022	mmu:24053	
K12563	SGCD	mmu05410(	PF04790.18	GO:004323	cytoplasmic	NP_036021	mmu:24052	
K27702	SPIN		PF02513.24	GO:004239	cytoplasmic	NP_848868	mmu:270624	
YPIH(kiaa1467)			PF23727.1(I	GO:000579	cytoplasmic	AAI37675.1	mmu:74525	
K26598	HSPA2	mmu04612(	PF00012.26	GO:009008	cytoplasmic	ACJ64499.1	mmu:15512	
K17776	MTX	mmu04137(	PF10568.15	GO:000810	cytoplasmic	NP_001156	mmu:382793	
K00746	CSGALNA	mmu00532(	PF05679.22	GO:003020	ER	NP_001349	mmu:78752	
RER1 retention in endoplasmic retic			PF03248.18	GO:000810	Golgi appa	NP_001034	mmu:67830	
K05360	TXNDC12	mmu00480(	PF13899.12	GO:200123	ER	NP_079610	mmu:66073	
K11664	VPS72, TCI	mmu03082(	PF05764.18	GO:008009	nuclear	NP_033362	mmu:21427	
K19943	ZWINT		PF15556.11	GO:200024	cytoplasmic	NP_001280	mmu:52696	
K03283	HSPA1_6_8	mmu04915(	PF00012.26	GO:000573	cytoplasmic	NP_077327	mmu:15481	
K09647	IMP1	mmu03060(	PF10502.14	GO:000425	cytoplasmic	NP_082536	mmu:66541	
K00799	GST, gst	mmu00980(	PF01124.23	GO:000663	ER	NP_079845	mmu:66447	
K07882	RAB3A	mmu04721(	PF00071.28	GO:000810	cytoplasmic	NP_001116	mmu:19339	
K04354	PPP2R2	mmu05165(	PF24814.1(\	GO:001631	cytoplasmic	EFB22778.1	mmu:71978	
K07277	SAM50, TO	mmu04137(	PF01103.29	GO:002260	cytoplasmic	NP_848729	mmu:68653	
K17477	FARP1		PF00621.25	GO:190560	cytoplasmic	NP_598843	mmu:223254	
K11757	PBRM1, PB	mmu03082(	PF00439.30(Bromodom		nuclear	NP_001346	mmu:66923	HMG
K11840	USP9_24		PF25010.1(	GO:000823	cytoplasmic	NP_683745	mmu:107868	
K05749	CYFIP	mmu04810(	PF05994.16	GO:008009	cytoplasmic	NP_001239	mmu:76884	
K24049	MPP3_7		PF00625.27	GO:000592	cytoplasmic	NP_001074	mmu:75739	
K20474	RINT1, TIP20		PF04437.19	GO:000810	cytoplasmic	AAI45268.1	mmu:72772	
K06559	LY75, CD205		PF00059.27	GO:000989	lysosomal	BAE26137.1	mmu:17076	
K00615	E2.2.1.1, tkt	mmu00030(	PF02779.29	GO:000028	cytoplasmic	NP_033414	mmu:21881	
K03263	EIF5A		PF21485.3(I	GO:000374	cytoplasmic	NP_001003	mmu:276770	
K20898	GBP5	mmu04621(	PF02263.25	GO:000810	cytoplasmic	EDL12061.	mmu:229898	
K07604	KRT1	mmu04915(	PF00038.26	GO:002260	cytoplasmic	NP_034790	mmu:16661	
K15029	EIF3L		PF10255.15	GO:000548	cytoplasmic	NP_660121	mmu:223691	
K17296	TSPAN14		PF00335.26	GO:000810	plasma mer	NP_001303	mmu:52588	
K13984	TXNDC5, I	mmu04141(	PF00085.26	GO:000645	cytoplasmic	NP_001276	mmu:105245	
K25165	HELZ2		PF13087.12	GO:003577	cytoplasmic	XP_006500	mmu:229003	
K17258	ITFG3		PF23727.1(I	GO:000557	plasma mer	NP_001361	mmu:106581	
K12831	SF3B4, SAP	mmu03040(	PF00076.28	GO:001921	nuclear	XP_032010	mmu:107701	
K27315	MPZL1		PF07686.23	GO:001602	cytoplasmic	NP_001001	mmu:68481	
K20071	ARHGAP26, GRAF		PF16746.10	GO:001602	cytoplasmic	AAI41395.1	mmu:71302	
K06236	COL1A	mmu05165(	PF01391.23	GO:000810	extracellula	AAB29425.	mmu:12842	
K06553	VPREB, CD179a		PF07686.23(V-set:Immu		lysosomal	DBA12444.	mmu:22362	
K23698	SLA		PF00017.30	GO:008009	cytoplasmic	AAH52655.	mmu:77799	
K12589	RRP42, EX	mmu03018(	PF01138.27	GO:007104	nuclear	AAH52656.	mmu:66446	

YRGB(immunoglobulin heavy varia			PF07686.23(V-set:Immu		ER	DBA12450.1(TPA: IGHV6-6*01, pa	
K11351	NDUFB11	mmu05208(	PF10183.14	GO:002260'	mitochondr	NP_001345	mmu:104130
K14801	TSR4		PF04194.18	GO:000573'	mitochondr	NP_080825	mmu:68079
26M5(N-acetyltransferase 6 (GCN5-			PF00583.31	GO:003421	cytoplasmic	NP_062724	mmu:56441
K10352	MYH9s	mmu04530(	PF01576.24	GO:003243	cytoplasmic	BAE27998.1	mmu:77579
K12604	CNOT1, N(	mmu03018(	PF04054.21(Not1:CCR4		cytoplasmic	XP_036009	mmu:234594
K17390	CDCA5	mmu05206(	PF09666.15	GO:000548	nuclear	NP_080686	mmu:67849
K02427	rlmE, rrmJ, ftsJ		PF01728.25	GO:002260'	mitochondr	NP_080786	mmu:68017
K19387	CAVIN1, PTRF		PF15237.11	GO:009859	cytoplasmic	NP_033012	mmu:19285
K23335	GID4		PF09783.14	GO:000989	cytoplasmic	NP_080033	mmu:66771
K11142	LAP3	mmu00330(	PF00883.26	GO:000417'	mitochondr	NP_077754	mmu:66988
K01314	F10	mmu04610(	PF00089.32	GO:000425	extracellulo	NP_001229	mmu:14058
K16816	PNPLA2, A	mmu04714(	PF01734.28	GO:008009	cytoplasmic	NP_001157	mmu:66853
XPTT(-)					mitochondr	XP_006531208.3(genetic suppressor	
K05732	ARHGAP3	mmu04611(	PF19518.5(I	GO:003514	cytoplasmic	NP_766327	mmu:232906
XRCG(USO1 vesicle docking protei			PF04869.20	GO:004323	cytoplasmic	NP_062363	mmu:56041
K05012	CLCN3_4_	mmu04613(	PF00654.25	GO:000802	cytoplasmic	EDL01088.	mmu:12727
K11305	MYST3, KA	mmu04550(	PF01853.24	GO:008009	nuclear	NP_001074	mmu:244349
K03512	POLL	mmu03450(	PF14792.12	GO:001683	nuclear	NP_001317	mmu:56626
K15464	BNIP3	mmu04140(	PF06553.17	GO:003202	cytoplasmic	NP_033890	mmu:12176
K03917	F13A1	mmu04610(	PF00927.28	GO:001814	cytoplasmic	BAE20463.1	mmu:74145
K11462	EED	mmu03083(	PF24817.1(\	GO:001922	nuclear	AAC53302.	mmu:13626
K04508	TBL1	mmu04310(	PF00400.38	GO:008009	nuclear	XP_028629	mmu:21372
K02537	MAD2	mmu04110(	PF02301.23	GO:008009	cytoplasmic	BAE35726.1	mmu:56150
K02355	fusA, GFM, EFG		PF00009.33	GO:003563	mitochondr	NP_001258	mmu:320806
K16822	FRMD6	mmu04390(	PF00373.23	GO:000810	ER	NP_082403	mmu:319710
K08864	TLK		PF00069.31	GO:190227	cytoplasmic	NP_766252	mmu:228012
K16726	ANKRD17, MASK		PF12796.13	GO:190024	nuclear	XP_006535	mmu:81702
K24769	TCF20		PF13771.12	GO:000097'	nuclear	NP_001107	mmu:21411
K06731	BST2, CD3	mmu03250(	PF16716.10	GO:000486	cytoplasmic	NP_932763	mmu:69550
K16735	ITGB1BP2		PF04968.17	GO:000716	cytoplasmic	NP_038740	mmu:26549
K13192	RBM26		PF00076.28	GO:000613	nuclear	XP_036014	mmu:74213
K00869	MVK, mval	mmu00900(	PF00288.32	GO:000449	cytoplasmic	NP_076045	mmu:17855
K07764	ADAMTS4		PF05986.19	GO:000417	plasma mer	EDL39113.	mmu:240913
K06570	LY9, CD229		PF13927.12	GO:000989'	plasma mer	NP_032560	mmu:17085
K14944	NOVA		PF00013.35	GO:004866	cytoplasmic	NP_001025	mmu:384569
K07583	PUS10		PF21238.2(I	GO:003111	cytoplasmic	NP_001028	mmu:74467
ZR2P(Zinc finger protein-like 1)				GO:004322	cytoplasmic	NP_001343	mmu:81909
K22641	TTYH		PF04906.18	GO:000522	plasma mer	NP_444503	mmu:117160
K01834	PGAM, gpr	mmu05230(	PF00300.27	GO:001922	cytoplasmic	NP_075907	mmu:18648
K20638	ARHGAP1'	mmu04530(	PF03114.23	GO:000688'	cytoplasmic	NP_001116	mmu:70497
K00364	guaC, GMF	mmu01100(	PF00478.31	GO:004317	cytoplasmic	NP_079784	mmu:66355
K07432	ALG13	mmu00510(	PF04101.22	GO:000578	cytoplasmic	Q9D8C3.2(	mmu:67574

Y9BS(Zinc finger CCCH-type contai			PF22623.2(z	GO:000989	nuclear	NP_941033	mmu:330474	
K13191	RBM16, SCAF8		PF04818.19	GO:008009	cytoplasmic	BAC65733.1	mmu:106583	
K01402	MMP8		PF00413.30	GO:000425	plasma mer	NP_032637	mmu:17394	
K03233	EEF1G	mmu05134(	PF00647.24	GO:000578	cytoplasmic	KAF156865	mmu:67160	
K23296	ITFG2		PF15907.11	GO:000226	cytoplasmic	XP_029334	mmu:101142	
K11765	ARID2	mmu03082(	PF01388.27	GO:003472	cytoplasmic	NP_780460	mmu:77044	ARID
XPZC(transmembrane protein 161B			PF10268.14	GO:200123	plasma mer	NP_663572	mmu:234371	
K13348	MPV17	mmu04146(	PF04117.17	GO:008009	mitochondr	NP_001281	mmu:17527	
K12591	RRP6, EXC	mmu03018(	PF01612.26	GO:007104	nuclear	BAB30844.1	mmu:50912	
TJK(coiled-coil domain containing			PF15027.12	GO:000557	ER	NP_780307	mmu:57895	
K09228	KRAB		PF13465.12	GO:000097	nuclear	BAB25286.1	mmu:22750	zf-C2H2
TGA(family with sequence similari			PF14977.11	(FAM194:F/	cytoplasmic	NP_001074	mmu:545527	
K18340	AEN, ISG20L1			GO:000931	nuclear	BAC36333.1	mmu:68048	
K08486	STX1B_2_3	mmu04130(	PF00804.31	GO:000802	cytoplasmic	NP_001272	mmu:13852	
ZR2A(Transmembrane protein 214)			PF10151.14	GO:009951	nuclear	NP_653108	mmu:68796	
K06622	CDKN2C, I	mmu04934(	PF12796.13	GO:003250	cytoplasmic	BAE34001.1	mmu:12580	
K15437	AIMP1, ARC1		PF01588.26	GO:004212	cytoplasmic	BAC40045.1	mmu:13722	
K08753	FABP4, aP2	mmu03320(	PF00061.29	GO:008009	cytoplasmic	AAH02148.	mmu:11770	
K01889	FARSA, ph	mmu00970(	PF01409.25	GO:000643	peroxisoma	NP_077236	mmu:69955	
K11519	DERL1	mmu04141(	PF04511.20	GO:000810	ER	BAE28291.1	mmu:67819	
K23205	THAP4		PF08768.17	GO:001975	nuclear	NP_080196	mmu:67026	THAP
K14558	PWP2, UTF	mmu03008(	PF24817.1(V	GO:002260	mitochondr	AAI28481.1	mmu:110816	
K19403	MCPH1		PF12258.13	GO:000810	nuclear	BAE28292.1	mmu:244329	
K24849	IFIT3		PF14559.12	GO:003563	cytoplasmic	AAH03804.	mmu:15959	
K12028	TRIM59		PF13445.12	GO:000578	cytoplasmic	NP_080139	mmu:66949	
umor necrosis factor receptor super			PF00023.36	(Ank:Ankyri	nuclear	BAC27191.1	mmu:268301	
K17920	SNX5_6_32	mmu04144(	PF09325.15	GO:000810	cytoplasmic	BAE39459.1	mmu:69178	
K03652	MPG	mmu03410(	PF02245.21	GO:000582	nuclear	NP_034952	mmu:268395	
M4G(huntingtin interacting protei			PF19026.7(U	GO:005082	cytoplasmic	NP_080594	mmu:67693	
K11251	H2A	mmu05322(	PF01661.27	GO:000810	nuclear	BAC37288.1	mmu:404634	
K25202	IPO13, RAN	mmu03013(	PF18786.7(I	GO:003450	cytoplasmic	BAE29208.1	mmu:230673	
K13370	HSD17B8	mmu00140(	PF13561.12	GO:000663	mitochondr	AIC84005.1	mmu:14979	
VC9(zinc finger)			PF04438.22	GO:004316	cytoplasmic	AAH19440.	mmu:29805	
K03238	EIF2S2		PF01873.23	GO:002260	nuclear	NP_080306	mmu:67204	
K09228	KRAB		PF13465.12	GO:000097	cytoplasmic	BAE26431.1	mmu:74352	zf-C2H2
K03539	RPP1, RPP3	mmu03008(	PF01876.22	GO:000454	cytoplasmic	NP_062301	mmu:54364	
K07606	VIM	mmu05169(	PF00038.26	GO:009951	cytoplasmic	AAA40555.	mmu:22352	
K19199	SETD3		PF09273.16	GO:000647	cytoplasmic	NP_082538	mmu:52690	
K15148	MED7		PF05983.16	GO:008009	nuclear	NP_001098	mmu:66213	
K03990	C3	mmu05167(	PF07678.20	GO:004340	ER	NP_033908	mmu:12266	
K10994	RAD9A	mmu04218(	PF04139.18	GO:200123	cytoplasmic	NP_035367	mmu:19367	
K12813	DHX16	mmu03040(	PF07717.22	GO:004508	nuclear	NP_081263	mmu:69192	
K20122	ARHGAP4		PF00620.32	GO:004863	cytoplasmic	NP_001155	mmu:171207	

K10597	UBE4B, UF	mmu04141(	PF10408.14(Ufd2P_core		nuclear	XP_029331.	mmu:63958
K14966	HCFC1	mmu03083(	PF24681.1(I	GO:008009	mitochondr	XP_006527	mmu:15161
K06548	SN, SIGLE(	mmu04514(	PF13895.12	GO:200123	extracellulo	NP_035556	mmu:20612
K20180	VPS16	mmu04140(	PF04841.19	GO:000810	cytoplasmic	NP_085036	mmu:80743
K03262	EIF5		PF01873.23	GO:002260	cytoplasmic	NP_775539	mmu:217869
K07575	MCTS, TMA20		PF17832.7(I	GO:002260	cytoplasmic	NP_081178	mmu:68995
K02137	ATPeF0O,	mmu05208(	PF00213.23	GO:000526	mitochondr	NP_613063	mmu:28080
K02144	ATPeV1H	mmu05165(	PF03224.20	GO:003131	cytoplasmic	NP_001297	mmu:108664
K03537	POP5	mmu03008(	PF01900.24	GO:003320	cytoplasmic	NP_080674	mmu:117109
K23978	IAH1		PF00657.28	GO:001678	extracellulo	NP_080623	mmu:67732
UCZ(Leptin receptor overlapping)			PF04133.19	GO:003251	plasma mer	BAE32780.	mmu:68192
K19415	PHF8, JHDM1F		PF17811.6(J	GO:003506	nuclear	NP_001106	mmu:320595
K22525	MKL		PF02755.20	GO:008009	nuclear	XP_006522	mmu:239719
K23789	RNF145		PF13705.11	GO:000548	cytoplasmic	NP_001350	mmu:74315
K05310	PIGG, GPI	mmu00563(	PF19316.4(I	GO:005126	ER	NP_001297	mmu:433931
K17089	ANXA3		PF00191.25	GO:000690	cytoplasmic	NP_001407	mmu:11745
)UEV(oxidoreductase)					cytoplasmic	NP_803415	mmu:52013
K14567	UTP14	mmu03008(	PF04615.18	GO:003204	nuclear	AAI41103.1	mmu:195434
K01759	GLO1, glo/	mmu00620(	PF00903.31	GO:008009	cytoplasmic	NP_001107	mmu:109801
K16469	CROCC			GO:000810	cytoplasmic	XP_036860	mmu:230872
K23028	SPECC1		PF00307.36	GO:001602	nuclear	NP_001268	mmu:432572
K10477	BTBD1_2		PF08005.17	GO:003577	cytoplasmic	NP_666305	mmu:83962 ZBTB
K10382	DST		PF00435.27	GO:002260	ER	NP_001263	mmu:13518
K01166	RNASET2		PF00445.23	GO:003389	cytoplasmic	NP_001351	mmu:100037283
K16942	SEPT2	mmu05100(	PF00735.24	GO:009951	cytoplasmic	NP_001153	mmu:18000
K26390	TNFAIP8		PF05527.16	GO:000367	cytoplasmic	NP_598892	mmu:106869
K25552	CFAP47		PF24507.1(Ig_CFAP65_		cytoplasmic	NP_001355	mmu:636104
K18953	NSMAF, F/	mmu04071(	PF02138.23	GO:003425	cytoplasmic	NP_035075	mmu:18201
K04680	ID1	mmu04390(	PF00010.31	GO:003292	cytoplasmic	NP_001355	mmu:15901 bHLH
K11848	USP20_33		PF00443.35	GO:001922	cytoplasmic	NP_001070	mmu:170822
K17499	PPM1G, PP2CG		PF00481.26	GO:001679	cytoplasmic	KAF61056(	mmu:14208
K22644	HVCN1, HV1		PF16799.10	GO:000526	cytoplasmic	NP_001035	mmu:74096
K25605	BCL7	mmu03082(	PF04714.18	GO:190494	cytoplasmic	NP_001334	mmu:12055
K09228	KRAB		PF01352.32	GO:000097	cytoplasmic	NP_001161	mmu:239546
K11581	SGOL2			GO:003304	cytoplasmic	NP_950172	mmu:68549
K01303	APEH		PF19283.4(/	GO:000425	mitochondr	NP_001397	mmu:235606
K12475	DAB2	mmu04144(	PF21792.3(I	GO:005104	cytoplasmic	NP_075607	mmu:13132
K14950	ATP13A1, SPF1		PF00122.25	GO:000810	cytoplasmic	NP_573487	mmu:170759
K13855	SLC4A2, AI	mmu04971(	PF00955.27	GO:004213	cytoplasmic	NP_001240	mmu:20535
K26497	TMEM230		PF05915.17	GO:000802	ER	NP_001135	mmu:70612
K08823	CLK2_3		PF04144.18	GO:004348	nuclear	NP_001296	mmu:105943584
K11539	CBR4	mmu01212(	PF13561.12	GO:000663	mitochondr	NP_663570	mmu:234309
YE0(immunoglobulin heavy variab			PF07686.23	GO:000961	cytoplasmic	BAV89191.1(single-chain variable f	

K15182	RDBP, NEl	mmu03250(	PF00076.28	GO:000802:	nuclear	P19426.2(R	mmu:27632
K16537	PCM1		PF15717.10	GO:003315'	cytoplasmic	AAI39034.1	mmu:18536
K17340	FBLN5		PF07645.21	GO:004316'	extracellulo	NP_001348	mmu:23876
K18469	TBC1D5		PF00566.25	GO:000548:	cytoplasmic	NP_001272	mmu:72238
K20306	TRAPPC9, TRS120		PF08626.16	GO:004885	cytoplasmic	XP_006521	mmu:76510
K02992	RP-S7, MRI	mmu03010(	PF00177.27	GO:002260'	mitochondr	NP_079581	mmu:50529
K00784	rnz		PF13691.12	GO:014005:	peroxisoma	NP_075968	mmu:68626
K06751	MHC1	mmu05165(	PF00129.23	GO:001922:	cytoplasmic	NP_038847	mmu:14991
K13499	CHSY	mmu00532(	PF05679.22	GO:000548:	mitochondr	AAI28568.1	mmu:269941
K25839	NDFIP		PF10176.14	GO:190187:	cytoplasmic	XP_028747	mmu:65113
K03993	MASP2	mmu04610(	PF00089.32	GO:000425:	lysosomal	NP_001003	mmu:17175
K20868	ATG16L2	mmu04140(	PF08614.16	GO:002260'	cytoplasmic	NP_001104	mmu:73683
K20732	GPNMB		PF20433.3(I	GO:004213'	plasma mer	BAE42560.1	mmu:93695
2243(Transmembrane protein 167A)			PF06842.17	GO:000810	ER	EDL00977.1	mmu:66074
K22556	MANF, ARMET		PF20145.4(/	GO:003250:	nuclear	AAH48616.	mmu:74840
K06699	PSME4	mmu03050(	PF16507.11	GO:004239:	nuclear	NP_598774	mmu:103554
K06553	VPREB, CD179a		PF07686.23(V-set:Immu		plasma mer	CAA75911.1	mmu:22364
K18763	LARP4		PF05383.23	GO:002260	cytoplasmic	NP_001271	mmu:207214
ΥQB2(immunoglobulin kappa varia			PF07686.23	GO:000237'	plasma mer	CAB46315.1(immunoglobulin light	
K06553	VPREB, CD179a		PF07686.23(V-set:Immu		extracellulo	CAB46304.1	mmu:22364
K20804	RNF34		PF23632.1(S	GO:200123	cytoplasmic	NP_085041	mmu:80751
K09291	TPR, MLP1	mmu03013(	PF07926.17	GO:003315'	cytoplasmic	NP_598541	mmu:108989
K08780	CPA3	mmu04972(	PF00246.29	GO:004322	plasma mer	NP_031779	mmu:12873
K06556	CD200		PF00047.31	GO:003411:	plasma mer	NP_034948	mmu:17470
K02949	RP-S11e, Rl	mmu05171(	PF16205.10	GO:003204'	cytoplasmic	NP_001006	mmu:27207
K16731	GOLGA1		PF01465.25	GO:000579	cytoplasmic	AAH80733.	mmu:76899
K17406	MRPS27		PF10037.15	GO:001593:	mitochondr	NP_776118	mmu:218506
K02981	RP-S2e, RP!	mmu05171(	PF00333.26	GO:001593:	cytoplasmic	XP_057623:	mmu:16898
K01302	CPQ		PF04389.22	GO:000032:	ER	NP_061225	mmu:54381
K14191	DIM1		PF00398.26	GO:200023	nuclear	BAE26964.1	mmu:66254
ΥF12(family with sequence similarit			PF24630.1(I	GO:000989:	ER	NP_598824	mmu:105203
K16488	SDCCAG8		PF15964.10	GO:003001'	cytoplasmic	AAH25138.	mmu:76816
Ƶ94Y(Chromosome 18 open reading			PF15303.11	GO:004322'	nuclear	NP_848785	mmu:212163
K01519	rdgB, ITPA	mmu01100(	PF01725.21	GO:000619:	cytoplasmic	NP_080198	mmu:16434
K17849	HECTD4		PF00632.30	GO:001978'	cytoplasmic	NP_852086	mmu:269700
K08014	RAPGEF3,	mmu04670(	PF00617.24	GO:003315'	cytoplasmic	NP_001171	mmu:223864
K11544	DSN1		PF08202.16	GO:190304'	nuclear	NP_080129	mmu:66934
Υ9KV(Dysferlin, limb girdle muscul			PF10267.14	GO:000578'	cytoplasmic	XP_006506:	mmu:330401
K13997	PDHX	mmu01100(	PF00198.28	GO:000663'	mitochondr	NP_780303	mmu:27402
K07605	KRT2		PF00038.26	GO:009951:	mitochondr	NP_149064	mmu:110310
K27818	RIC8		PF10165.14	GO:000548:	cytoplasmic	NP_444424	mmu:101489
K25772	PAXX		PF15384.11	GO:001989'	extracellulo	XP_030105	mmu:227622
K20793	NAA50, NAT5		PF00583.31	GO:003124:	cytoplasmic	NP_001334	mmu:72117

K06817	PODXL	mmu05132(	PF06365.17	GO:009859	extracellula	BAA86912.	mmu:27205	
K01082	cysQ, MET:	mmu00920(	PF00459.30	GO:004648	mitochondr	NP_001334	mmu:23827	
K09173	RFX1_2_3		PF04589.18	GO:008009	nuclear	NP_033081	mmu:19724	RFX
K24086	EIF4H		PF00076.28	GO:009007	cytoplasmic	BAE20955.	mmu:22384	
K00939	adk, AK	mmu01240(	PF00406.28	GO:001920	cytoplasmic	XP_021041	mmu:11636	
K18708	FAM103A1		PF15320.11	GO:000548	nuclear	XP_036009	mmu:100043059	
K06562	LAMP3, CI	mmu04142(	PF01299.22	GO:000989	ER	NP_796330	mmu:239739	
YNB1(antigen binding)			PF07654.21	GO:000548	plasma mer	AAH10327.1(Ighg protein [Mus mu		
K03103	MINPP1	mmu00562(	PF00328.27	GO:001679	mitochondr	BAC31265.2	mmu:17330	
K15202	GTF3C5, TFC1		PF09734.15	GO:000018	nuclear	NP_683730	mmu:70239	
K24990	RBMS		PF00076.28	GO:000573	cytoplasmic	NP_001135	mmu:56878	
K03135	TAF11	mmu03022(	PF04719.20	GO:008009	nuclear	NP_001366	mmu:68776	
K11095	SNRPC	mmu03040(	PF06220.18	GO:002260	cytoplasmic	NP_001400	mmu:20630	
					nuclear	BAE28621.1(unnamed protein prod		
K06554	IGLL1, IGI	mmu05340(	PF07654.21	GO:000588	cytoplasmic	P01844.1(R	mmu:16136	
K01008	selD, SEPH	mmu00450(	PF02769.27	GO:001602	cytoplasmic	NP_001349	mmu:109079	
K14684	SLC25A23S		PF00153.32	GO:000534	cytoplasmic	NP_766273	mmu:229731	
ZFEH(family with sequence similari			PF15068.11	GO:003243	cytoplasmic	NP_083934	mmu:76566	
K17422	MRPL41		PF09809.14	GO:001593	mitochondr	NP_001026	mmu:107733	
R9S(OCIA domain containing 1)			PF07051.17	GO:200073	cytoplasmic	NP_075918	mmu:68095	
XP3F(DENN MADD domain conta			PF09794.14	GO:005503	cytoplasmic	NP_001127	mmu:211922	
K12469	EHD2	mmu04144(	PF18150.6(I	GO:000810	cytoplasmic	NP_001406	mmu:259300	
K15156	MED14, RC	mmu04919(	PF08638.16	GO:006026	nuclear	NP_001041	mmu:26896	
K14849	RRP1		PF05997.17	GO:008009	nuclear	NP_082520	mmu:72462	
K06727	FCRL, IRTA, CD307		PF13927.12	GO:000989	plasma mer	AAH16551.	mmu:80891	
K25174	RNF220		PF15926.10	GO:008009	cytoplasmic	NP_001297	mmu:66743	
K25436	POLR1G	mmu03020(	PF08208.16	GO:003088	cytoplasmic	NP_665821	mmu:70333	
K01044	CES1	mmu00983(	PF00135.34	GO:000581	ER	NP_031980	mmu:13884	
K18753	ZFP36L	mmu04218(	PF04553.17	GO:004559	cytoplasmic	NP_031590	mmu:12192	zf-CCCH
K15280	SLC35C2		PF03151.22	GO:001593	plasma mer	XP_057359	mmu:228875	
K18162	NDUFAF5	mmu04714(	PF08241.18	GO:002260	mitochondr	NP_081369	mmu:69487	
ESF1, nucleolar pre-rRNA processin			PF25121.1(I	GO:009030	nuclear	NP_001074	mmu:66580	
K08628	ADAMTS14		PF19030.6(	GO:000417	plasma mer	AAI57954.1	mmu:237360	
K05633	WWP1, AII	mmu04120(	PF00632.30	GO:004423	nuclear	AAI50813.1	mmu:107568	
K02905	RP-L29e, R	mmu05171(	PF01779.22	GO:002262	cytoplasmic	NP_001311	mmu:19944	
K05636	LAMB1	mmu05165(	PF00053.31	GO:003444	plasma mer	AAI50810.1	mmu:16777	
K08333	PIK3R4, VI	mmu04136(	PF22956.2(	GO:000810	cytoplasmic	AAI57948.1	mmu:75669	
K25813	CCDC88B			GO:000578	extracellula	AAI57947.1	mmu:78317	
K15503	ANKRD44		PF12796.13	GO:000557	cytoplasmic	XP_030110	mmu:329154	
K04430	MAP2K4, M	mmu04012(	PF00069.31	GO:001922	cytoplasmic	NP_001025	mmu:26398	
K25752	ZPBP		PF07354.18	GO:003250	cytoplasmic	NP_001172	mmu:53604	
K04581	GLP1R	mmu04024(	PF00002.29	GO:009008	plasma mer	AAI45627.1	mmu:14652	
GJP(cyclic nucleotide binding dom			PF00027.35(cNMP_bin		cytoplasmic	NP_001358	mmu:435772	

)29H(cytochrome-b5 reductase activ			PF05334.18(DUF719:Pr		nuclear	XP_006534	mmu:67726	
K22210	DGLUCY	mmu00470(	PF14336.11	GO:003197	mitochondr	EDL18891.	mmu:217830	
K06103	CASK		PF00069.31	GO:009008	cytoplasmic	AAI45621.1	mmu:12361	
K14297	NUP98, AD	mmu03013(	PF12110.13	GO:005116	nuclear	NP_001274	mmu:269966	
K23353	PDLIM1_2	mmu04820(	PF15936.10	GO:003243	cytoplasmic	NP_001361	mmu:53318	
K25163	WAPL	mmu04110(	PF07814.18	GO:003304	nuclear	NP_001288	mmu:218914	
XQ3P(chromosome 10 open reading			PF08427.15	GO:009858	cytoplasmic	NP_938038	mmu:71617	
K24510	VWA5		PF08487.16	GO:000557	cytoplasmic	NP_001139	mmu:67776	
K04443	MAPKAPK	mmu04010(	PF00069.31	GO:008009	cytoplasmic	DAC77328.	mmu:17164	
K06751	MHC1	mmu05165(	PF00129.23	GO:009857	ER	NP_001001	mmu:14972	
K07836	RAP1B	mmu04010(	PF00071.28	GO:005104	cytoplasmic	NP_001007	mmu:215449	
.KMN(motile sperm domain contai			PF00635.32	GO:000989	cytoplasmic	BAE24140.	mmu:70380	
ıcute-phase response)			PF00089.32	GO:000425	extracellula	NP_001409	mmu:14940	
›rotease)			PF00089.32	GO:000425	extracellula	NP_034504	mmu:14943	
K24611	AMMECR1, AMMECR		PF01871.22	GO:004323	nuclear	NP_001229	mmu:225339	
K11670	TEPT, INO	mmu03082(	PF24245.1(I	GO:009951	nuclear	BAB26816.1	mmu:69714	
K12384	SCARB2, L	mmu04142(	PF01130.26	GO:000810	extracellula	NP_031670	mmu:12492	
K16682	AJUBA, LIM	mmu04390(	PF00412.28	GO:000810	cytoplasmic	NP_034720	mmu:16475	
K07968	B4GALT3	mmu01100(	PF13733.12	GO:000367	Golgi appa	NP_065604	mmu:57370	
XQ0M(G patch domain containing			PF01585.28	GO:000548	nuclear	AAI21830.1	mmu:242691	
K13335	PEX16	mmu04146(	PF08610.15	GO:003303	peroxisoma	NP_660104	mmu:18633	
JPF0176 protein)			PF23949.1(TSTD2_N:T		cytoplasmic	NP_001342	mmu:272027	
K10083	LMAN2L, VIPL		PF03388.20	GO:000810	mitochondr	NP_001297	mmu:214895	
ZUGY(filamin A interacting protein			PF09727.14	GO:000573	cytoplasmic	NP_001035	mmu:78749	
K22824	WTAP		PF17098.10	GO:001602	cytoplasmic	NP_001107	mmu:60532	
K25388	KIFAP3, K	mmu04814(	PF05804.18	GO:000810	cytoplasmic	NP_001292	mmu:16579	
K20908	GBP6		PF02263.25	GO:002000	cytoplasmic	NP_001411	mmu:236573	
K14308	NUP54, NU	mmu03013(	PF13874.11	GO:003450	cytoplasmic	NP_899248	mmu:269113	
K19539	SECISBP2, SBP2		PF01248.31	GO:000645	nuclear	NP_808276	mmu:70354	
K14794	RRP12		PF08161.18	GO:009030	nuclear	NP_955518	mmu:107094	
K09571	FKBP4_5	mmu04915(	PF00254.34	GO:000645	cytoplasmic	NP_034350	mmu:14229	
K23333	RMND5		PF10607.14	GO:199023	cytoplasmic	NP_001192	mmu:68477	
K15166	MED23		PF11573.13	GO:008009	cytoplasmic	BAC98122.	mmu:70208	
K11478	JARID2, JM	mmu03083(	PF02373.28	GO:000810	nuclear	XP_006516	mmu:16468	ARID
K24733	LRWD1		PF23215.1(\	GO:004239	mitochondr	EDL19321.	mmu:71735	
K01511	ENTPD5_6	mmu01100(	PF01150.23	GO:001707	ER	NP_001341	mmu:12497	
K05487	IL36G, IL1	mmu04060(	PF00340.25	GO:000512	cytoplasmic	NP_705731	mmu:215257	
Competence protein)			PF12836.13	GO:001602	nuclear	BAD32517.	mmu:67484	
K01265	map		PF00557.29	GO:000417	nuclear	XP_017169	mmu:56307	
K25790	EMSY		PF03735.19	GO:008009	nuclear	XP_006507	mmu:233545	
K10408	DNAH	mmu05014(	PF12774.13	GO:002260	cytoplasmic	NP_001074	mmu:327954	
K07119	PTGR3, ZA	mmu00590(	PF00107.32	GO:005078	peroxisoma	NP_666202	mmu:225791	
DNA-dependent RNA polymerase c			PF08711.16	GO:001607	nuclear	NP_079893	mmu:66526	

'B1Z(transmembrane and coiled-co			PF05277.18	GO:000557	cytoplasmic	NP_001292	mmu:77056	
K01530	E7.6.2.1		PF16212.11	GO:001602	ER	NP_001074	mmu:54667	
K13502	STX4	mmu04962(	PF00804.31	GO:003577	cytoplasmic	NP_033320	mmu:20909	
K06512	LILR, CD8!	mmu04380(	PF13895.12	GO:000759	extracellula	NP_038560	mmu:14728	
KR7V(Transmembrane protein 19)			PF14705.12	GO:000815	nuclear	NP_001346	mmu:73112	
K00949	thiN, TPK1	mmu01240(	PF04263.21	GO:000677	cytoplasmic	NP_038889	mmu:29807	
K20353	SEC16		PF12931.13	GO:004820	cytoplasmic	NP_694765	mmu:227648	
K01044	CES1	mmu00983(	PF00135.34	GO:000663	ER	NP_659179	mmu:234564	
K00799	GST, gst	mmu00980(	PF02798.26	GO:000578	cytoplasmic	NP_080948	mmu:68312	
K11252	H2B	mmu05322(	PF00125.30	GO:003472	nuclear	AAI15793.1	mmu:319185	
K24335	CRELD		PF07645.21	GO:004316	plasma mer	NP_598691	mmu:171508	
SG0(Transmembrane protein 186)				GO:002260	mitochondr	NP_079984	mmu:66690	
K24387	L3MBTL	mmu03083(	PF02820.24	GO:000989	nuclear	NP_001346	mmu:23733	zf-C2HC
K17784	MICOS10, MINOS1, M		PF04418.17	GO:004428	peroxisoma	NP_001156	mmu:433771	
K06553	VPREB, CD179a		PF07686.23	GO:000548	cytoplasmic	AFW17460.	mmu:22362	
K06244	LAMB3	mmu05165(	PF23219.1(I	GO:000367	nuclear	BAC37486.1	mmu:16780	
K13438	RETN		PF06954.16	GO:000512	extracellula	NP_853627	mmu:245195	
Tumor necrosis factor receptor supe			PF00561.26	GO:001678	peroxisoma	BAE32818.1	mmu:68607	
K12388	SORT1	mmu04979(	PF15902.11	GO:000810	lysosomal	NP_064356	mmu:20661	
K07198	PRKAA, AI	mmu04714(	PF00069.31	GO:004732	cytoplasmic	AAH86695.	mmu:105787	
K23576	IGFBP4		PF00086.23	GO:008009	cytoplasmic	CAA57272.	mmu:16010	
K13348	MPV17	mmu04146(	PF04117.17	GO:002260	mitochondr	NP_898993	mmu:234384	
K07249	ALDH1A	mmu00830(	PF00171.27	GO:006000	cytoplasmic	NP_444310	mmu:56847	
K16481	PCNT		PF10495.14	GO:003315	cytoplasmic	NP_001269	mmu:18541	
K06566	IFITM		PF04505.17	GO:005160	cytoplasmic	NP_079654	mmu:66141	
K03132	TAF7	mmu03022(	PF04658.18	GO:003506	cytoplasmic	BAC35898.1	mmu:24074	
K01858	INO1, ISYN	mmu00562(	PF07994.18	GO:000989	cytoplasmic	NP_076116	mmu:71780	
'NU7(DNA-binding protein HGH1			PF04063.19(DUF383:D		cytoplasmic	NP_067530	mmu:59053	
K11982	RNF115_126		PF13639.12	GO:008009	cytoplasmic	NP_653111	mmu:70294	
K26401	TOM1, TOM1L1_2		PF00790.25	GO:001602	cytoplasmic	NP_694720	mmu:216810	
K05730	VAV	mmu04664(	PF00621.25	GO:000690	cytoplasmic	BAE30926.1	mmu:22324	
K13806	DAGL	mmu04723(	PF01764.32	GO:004634	ER	AAI12415.1	mmu:269060	
K11251	H2A	mmu05322(	PF16211.10	GO:007082	nuclear	NP_034566	mmu:15270	
F9W(chromosome 1 open reading f			PF07798.16	GO:004323	mitochondr	NP_079791	mmu:66365	
K03691	POFUT	mmu00514(	PF10250.15	GO:003606	Golgi appa	NP_536711	mmu:140484	
K11654	SMARCA5,	mmu03082(	PF00176.29	GO:190521	nuclear	NP_444354	mmu:93762	
CDC42 effector protein (Rho GTPas			PF12796.13	GO:003315	cytoplasmic	NP_082559	mmu:72565	
K06094	LLGL	mmu05165(	PF08366.18	GO:005104	cytoplasmic	NP_001152	mmu:16897	
K23164	RTN4IP1		PF13602.12	GO:005112	mitochondr	NP_570962	mmu:170728	
K17256	IGFALS, A	mmu04935(	PF13855.12	GO:000761	extracellula	NP_032366	mmu:16005	
PPX(CDC42 effector protein (Rho			PF14957.11	GO:002260	cytoplasmic	NP_081495	mmu:104445	
K22378	RNF181		PF13639.12	GO:000367	cytoplasmic	NP_001318	mmu:66510	
K19605	EVC1	mmu04340(	Hedgehog s	GO:009859	cytoplasmic	NP_067267	mmu:59056	

K01325	KLK1_2	mmu04614(	PF00089.32	GO:000425	extracellul	NP_034774	mmu:16618	
K08823	CLK2_3		PF00069.31	GO:001707	nuclear	EDL15234.	mmu:12748	
K01265	map		PF00557.29	GO:000417	cytoplasmi	NP_062622	mmu:56307	
K23500	SFXN1_3		PF03820.22	GO:000534	cytoplasmi	NP_001349	mmu:94280	
K17885	MTCH		PF00153.32	GO:200123	plasma me	NP_001304	mmu:56428	
K23314	WRAP53, TCAB1		PF00400.38	GO:000548	cytoplasmi	NP_001351	mmu:216853	
78B(myeloid cell nuclear differenti			PF02760.21	GO:003210	nuclear	ACD47030.	mmu:226695	
K03938	NDUFS5	mmu05208(	PF10200.14	GO:002260	cytoplasmi	NP_001025	mmu:595136	
2B3Y(Transmembrane protein 179B)				GO:001660	extracellul	EDL33366.	mmu:67706	
.7TV(chromosome 1 open reading f			PF15855.10(DUF4726:I		cytoplasmi	NP_001334	mmu:68920	
K17083	FERMT2, KIND2		PF18124.7(I	GO:003243	cytoplasmi	NP_666166	mmu:218952	
K08144	SLC2A6, GLUT6		PF00083.30	GO:000989	plasma me	NP_766247	mmu:227659	
K15686	MEX3, RKHD		PF00013.35	GO:000989	cytoplasmi	NP_941017	mmu:237400	
K14324	SAP18	mmu03013(	PF06487.18	GO:000371	cytoplasmi	NP_001028	mmu:100041953	
K04077	groEL, HSP	mmu04940(	PF00118.29	GO:004423	mitochondr	NP_001343	mmu:15510	
K23949	SAMD9, SAMD9L			GO:000989	cytoplasmi	NP_034286	mmu:209086	
K16198	YWHAG_I	mmu04114(	PF00244.26	GO:000810	cytoplasmi	NP_001026	mmu:22628	
K15424	PPP4R1		PF02985.28	GO:000573	cytoplasmi	NP_001107	mmu:70351	
K14943	MBNL		PF22628.2(zf-CCCH_1C		cytoplasmi	NP_001347	mmu:105559	
K05849	SLC8A, NC	mmu04974(	PF03160.20	GO:004354	ER	XP_006515	mmu:110893	
K19202	SAP30	mmu05169(	PF13866.11	GO:008009	nuclear	NP_001074	mmu:50724	
K09502	DNAJA1	mmu04141(	PF01556.23	GO:200123	cytoplasmi	NP_001158	mmu:15502	
K17065	DNM1L	mmu04621(	PF01031.25(Dynamin_I		cytoplasmi	NP_001392	mmu:74006	
K09852	RASSF3		PF16517.10	GO:000573	cytoplasmi	NP_620406	mmu:192678	
K04973	TRPV4	mmu04750(	PF00520.37	GO:000526	cytoplasmi	XP_035300	mmu:63873	
)1K4(NEDD4 binding protein)			PF13671.12(AAA_33:A		cytoplasmi	NP_598659	mmu:100637	
K00920	PIP4K2	mmu04070(	PF01504.24	GO:001922	cytoplasmi	NP_473392	mmu:108083	
K23345	GLMN		PF08568.16	GO:004213	cytoplasmi	NP_001155	mmu:170823	
K17261	CAP1_2, SRV2		PF08603.16	GO:000166	cytoplasmi	AAC37610.	mmu:12331	
K13190	RBM15		PF07744.19	GO:008009	nuclear	NP_001039	mmu:229700	
K12900	SRSF10, FU	mmu03040(	PF00076.28	GO:000024	nuclear	NP_001177	mmu:14105	
K06104	AMOTL	mmu04530(	PF12240.13	GO:000592	cytoplasmi	XP_006510	mmu:75723	
K08959	CSNK1D	mmu04390(	PF00069.31	GO:003292	cytoplasmi	NP_001268	mmu:104318	
.YE0(immunoglobulin heavy variab			PF07686.23(V-set:Immu		cytoplasmi	UNP35762.1(immunoglobulin heav		
.YE0(immunoglobulin heavy variab			PF07686.23(V-set:Immu		cytoplasmi	DBA12423.1(TPA: IGHV1-43*01, p		
K15135	MED18		PF09637.15	GO:008009	nuclear	NP_080315	mmu:67219	
.YE0(immunoglobulin heavy variab			PF07686.23(V-set:Immu		extracellul	DBA12425.1(TPA: IGHV1-39*01, p		
Complement component 3a recepto			PF11976.14	GO:008009	cytoplasmi	NP_035030	mmu:18020	
K00799	GST, gst	mmu00980(	PF13409.12	GO:005127	cytoplasmi	NP_034492	mmu:14873	
K01681	ACO	mmu01100(Metabolic p		GO:005089	cytoplasmi	KAF645149	mmu:11429	
K26787	MARVELD1		PF01284.28	GO:004322	ER	NP_899018	mmu:277010	
K09106	USF		PF00010.31	GO:004423	nuclear	CAA64627.	mmu:22278	bHLH
K15500	PPP6R2, SAPS2		PF04499.20	GO:003577	nuclear	XP_006521	mmu:71474	

K16682	AJUBA, LI!	mmu04390(	PF00412.28	GO:003577	cytoplasmic	NP_997095	mmu:101543
K08524	RXRA, NR:	mmu04151(	PF00104.35(Hormone_r		nuclear	XP_031225	mmu:20181
K11722	BRD4		PF00439.30	GO:190303	nuclear	NP_001273	mmu:57261
K01539	ATP1A	mmu04971(	PF00122.25	GO:001922	cytoplasmic	EDL39027.	mmu:98660
36R(This is a non-secretory ribonuc			PF00074.25	GO:000961	extracellulo	NP_444341	mmu:93719
K17932	SNX21		PF00787.29	GO:000810	cytoplasmic	NP_598685	mmu:101113
K13113	UBL5, HUB1			GO:009030	cytoplasmic	KAB04001S	mmu:66177
K11546	PMF1		PF03980.19	GO:000860	cytoplasmic	NP_080204	mmu:67037
K07249	ALDH1A	mmu00830(	PF00171.27	GO:000548	cytoplasmic	NP_036051	mmu:26358
K04659	THBS2S	mmu05165(	PF05735.19	GO:001922	plasma mer	NP_035712	mmu:21828
K13120	FAM32A		PF08555.15	GO:000557	nuclear	NP_080731	mmu:67922
559(NFAT activating protein with ITAM motif				GO:008009	cytoplasmic	AAI50489.1	mmu:74039
K13293	PDE4	mmu04928(	PF00233.25	GO:000921	cytoplasmic	NP_899668	mmu:18577
K23583	KPNA3_4	mmu03013(	PF00514.29	GO:003450	cytoplasmic	NP_001014	mmu:16648
K15042	KPNA5_6	mmu03013(	PF00514.29	GO:005116	cytoplasmic	NP_032494	mmu:16650
K20366	ERGIC2, ERV41		PF07970.17	GO:000578	Golgi appa	NP_080444	mmu:67456
K27834	MIF4GD, SLIP1		PF02854.25	GO:008009	cytoplasmic	NP_001230	mmu:69674
WXF(Yae1 domain containing 1)				GO:000573	cytoplasmic	BAB31592.1	mmu:67008
K12404	GGA	mmu04142(	PF00790.25	GO:000810	cytoplasmic	EDL34503.	mmu:260302
YE0(Is involved in NO detoxificati			PF07686.23	GO:000548	cytoplasmic	CAH04483.1(immunoglobulin heav	
K05718	ITGAL, CD	mmu04670(	PF00092.34	GO:000690	extracellulo	AAI45095.1	mmu:16408
K08955	YME1		PF01434.24	GO:000417	mitochondr	NP_038799	mmu:27377
K14619	TCN2	mmu04980(	PF01122.25	GO:000989	extracellulo	NP_001123	mmu:21452
K10407	KLC	mmu05020(	PF13374.12	GO:000573	cytoplasmic	XP_036013	mmu:16593
K14309	NUP93, NI	mmu03013(	PF04097.20	GO:003450	nuclear	EDL11120.	mmu:71805
XP22(chromosome 1 open reading fr			PF07406.16	GO:000690	Golgi appa	ERE92302.	mmu:99650
VAP(small integral membrane protein 7)				GO:000557	plasma mer	NP_765984	mmu:66818
K19737	PRMT9		PF22528.1(I	GO:001627	cytoplasmic	NP_001074	mmu:102182
K08504	BET1	mmu04130(SNARE int		GO:000810	cytoplasmic	NP_061212	mmu:54399
K01149	TRIR			GO:009030	cytoplasmic	NP_081036	mmu:68544
MWE(-)			PF15006.11	GO:000548	cytoplasmic	NP_001298	mmu:67326
K08513	VAMP4	mmu04130(	PF00957.27	GO:000810	cytoplasmic	NP_001334	mmu:53330
K10625	UBR1		PF18995.5(I	GO:004858	cytoplasmic	NP_033487	mmu:22222
XT8W(5-nucleotidase domain conta			PF05761.20	GO:001679	mitochondr	NP_780540	mmu:103466
K01363	CTSB	mmu04621(	PF00112.29	GO:000962	lysosomal	NP_031824	mmu:13030
K03671	TXN, trxA	mmu04621(	PF00085.26	GO:001972	cytoplasmic	NP_064297	mmu:56551
K11673	ACTR8, AR	mmu03082(	PF00022.25	GO:008009	cytoplasmic	EDL24744.	mmu:56249
K02202	CDK7	mmu03420(	PF00069.31	GO:199023	cytoplasmic	NP_034004	mmu:12572
K10084	EDEM1	mmu04141(	PF01532.26	GO:003315	Golgi appa	BAE30594.	mmu:192193
K00509	PTGS1, CO	mmu04611(	PF03098.20	GO:004590	lysosomal	NP_032995	mmu:19224
K01940	argG, ASS1	mmu00220(	PF20979.2(	GO:001564	cytoplasmic	NP_031520	mmu:11898
K17339	ATL		PF02263.25	GO:002260	cytoplasmic	NP_001156	mmu:109168
K17778	TIM10		PF02953.20	GO:001602	cytoplasmic	NP_001248	mmu:30059

K07830	RRAS2, TC	mmu04137(	PF00071.28	GO:190397	cytoplasmic	NP_001013	mmu:66922	
K17780	TIM8		PF02953.20	GO:000810	cytoplasmic	NP_038925	mmu:30057	
K17781	TIM13		PF02953.20	GO:000548	cytoplasmic	NP_038923	mmu:30055	
K11584	PPP2R5	mmu05165(	PF01603.26	GO:001922	nuclear	XP_021041	mmu:21770	
K18498	DUSP9, MI	mmu04010(	PF00782.25	GO:001679	cytoplasmic	CAD22884.	mmu:75590	
K05723	FGD4		PF00621.25	GO:004340	nuclear	NP_001288	mmu:224014	
K09377	CSRP	mmu04820(	PF00412.28	GO:000810	cytoplasmic	NP_001185	mmu:13009	
K24155	DMXL, DM	mmu04142(	PF12234.13	GO:001972	cytoplasmic	NP_001074	mmu:240283	
K17496	TIM50		PF03031.24	GO:000810	mitochondr	NP_079892	mmu:66525	
K17610	CHP, CHP1		PF13499.12	GO:003288	cytoplasmic	CAH67880	mmu:56398	
Hydrolase)			PF13344.12	GO:000665	mitochondr	NP_659064	mmu:214932	
K13200	STRBP		PF07528.20	GO:004885	cytoplasmic	NP_001342	mmu:20744	
K06271	TLN	mmu04015(	PF21896.2(	GO:000520	cytoplasmic	XP_036011	mmu:70549	
K02138	ATPeF0D,	mmu05208(	PF05873.17	GO:000526	cytoplasmic	NP_001404	mmu:71679	
K03165	TOP3	mmu03440(	PF01131.25	GO:003320	mitochondr	NP_033436	mmu:21975	
K15683	NFXL1, OZFP		PF01422.22	GO:008009	nuclear	NP_598682	mmu:10097	zf-NF-X1
K11257	FUT11		PF00852.26	GO:000367	Golgi appa	NP_082704	mmu:73068	
K17785	IMMT, MIC60		PF09731.14	GO:009879	mitochondr	NP_083949	mmu:76614	
K04347	GNG12	mmu04724(	PF00631.27	GO:000367	mitochondr	CAG95535	mmu:14701	
KWIP(GTPase, IMAP family memb			PF04548.22	GO:000578	cytoplasmic	NP_001070	mmu:243374	
K04520	APP	mmu04726(	PF12925.12	GO:004885	ER	NP_001185	mmu:11820	
K05087	IGF1R, CD	mmu04730(	PF07714.23	GO:000740	plasma mer	NP_034643	mmu:16001	
K12816	CDC40, PR	mmu03040(	PF00400.38	GO:004856	cytoplasmic	NP_082155	mmu:71713	
K14325	RNPS1	mmu03013(	PF00076.28	GO:000018	nuclear	XP_022372	mmu:19826	
K17701	SIPA1L1, E	mmu04015(	PF11881.13	GO:004858	cytoplasmic	NP_001394	mmu:217692	
K08958	CSNK1G	mmu04340(	PF00069.31	GO:000548	cytoplasmic	NP_001153	mmu:103236	
K25805	FEM1A_C		PF00023.36	GO:000582	nuclear	NP_789799	mmu:216622	
K22286	APOC1	mmu04979(	PF04691.17	GO:001922	ER	NP_001103	mmu:11812	
K06095	MPDZ, MU	mmu04530(	PF00595.29	GO:000592	cytoplasmic	NP_034950	mmu:17475	
K17277	EPS8		PF08416.19	GO:000548	cytoplasmic	EDL18055.	mmu:98845	
K14806	DDX31, DBP7		PF00270.35	GO:001688	nuclear	NP_001028	mmu:227674	
nvolved in mRNA degradation. Hyd			PF00575.29	GO:000989	nuclear	NP_694800	mmu:619605	
K02148	ATPeV1C,	mmu05165(	PF03223.20	GO:004696	cytoplasmic	BAE40727.	mmu:66335	
The purine nucleoside phosphorylas			PF08079.17	GO:001593	nuclear	NP_079709	mmu:66229	
I9Z(RAN guanine nucleotide releas			PF04603.18	GO:005116	cytoplasmic	NP_067304	mmu:57785	
K12389	BTS, CLN3	mmu04142(	PF02487.23	GO:004239	cytoplasmic	AAH47120.	mmu:12752	
K07897	RAB7A	mmu04137(	PF00071.28	GO:007266	cytoplasmic	NP_001280	mmu:19349	
K00791	miaA, TRIT	mmu01100(	PF01715.23	GO:003563	nuclear	NP_080149	mmu:66966	
K06251	VTN	mmu05165(	PF00045.24	GO:001922	ER	NP_035837	mmu:22370	
K15516	FMR		PF12235.13	GO:003577	nuclear	NP_001106	mmu:14359	
K00629	GPAT1_2	mmu00564(	PF19277.4(	GO:008009	cytoplasmic	XP_036017	mmu:14732	
K05127	PTK7, CCK4		PF13927.12	GO:004858	plasma mer	NP_780377	mmu:71461	
K01294	CPE	mmu04940(	PF00246.29	GO:000810	ER	NP_038522	mmu:12876	

K23588	MLLT6_10, AF17_10		PF13832.12	GO:000367	nuclear	NP_647472	mmu:246198	
K14946	RBFOX, FOX		PF12414.13	GO:004590	cytoplasmic	NP_444334	mmu:93686	
K09253	RELB	mmu04010(	PF16181.10	GO:003292	nuclear	NP_033072	mmu:19698	RHD
K10837	PSTK	mmu00970(	PF08433.16	GO:000645	cytoplasmic	NP_001034	mmu:214580	
K13121	FRA10AC1		PF09725.14	GO:001679	nuclear	NP_001074	mmu:70567	
K23884	WAC		PF00397.32	GO:008009	nuclear	NP_694725	mmu:225131	
K11432	PRDM2, RI	mmu00310(	PF21549.2(I	GO:008009	nuclear	NP_001074	mmu:11059	zf-C2H2
Y5E9(coiled-coil domain containing			PF15188.11	GO:000557	cytoplasmic	XP_052600	mmu:68597	
K14006	SEC23	mmu04141(	PF04811.21	GO:000548	cytoplasmic	ERE73132.	mmu:20334	
K00742	GCNT2	mmu00601(	PF02485.27	GO:003411	Golgi appa	NP_573482	mmu:14538	
K22647	MINDY3_4		PF13898.11	GO:004423	cytoplasmic	NP_001136	mmu:330323	
K15710	SHPRH		PF00176.29	GO:003472	nuclear	NP_001071	mmu:268281	
K13755	PDE1	mmu05032(	PF00233.25	GO:004299	cytoplasmic	NP_001342	mmu:18573	
K06237	COL4A	mmu05165(	PF01391.23	GO:006130	extracellulo	CAA26132.	mmu:12826	
K17491	SMEK, PPI	mmu04922(	PF04802.21	GO:008009	cytoplasmic	XP_031213	mmu:68734	
K08675	PRSS15, PIM1		PF05362.19	GO:000425	mitochondr	NP_083058	mmu:74142	
K14455	GOT2	mmu04975(	PF00155.27	GO:003017	mitochondr	NP_034455	mmu:14719	
K14454	GOT1	mmu01100(	PF00155.27	GO:000962	cytoplasmic	NP_034454	mmu:14718	
K08773	RALBP1	mmu05200(	PF00620.32	GO:001922	cytoplasmic	BAE26405.	mmu:19765	
K26152	SLC25A51_52		PF00153.32	GO:001593	cytoplasmic	BAE27432.	mmu:230125	
K22558	COMMD2		PF07258.19	GO:000573	cytoplasmic	NP_780304	mmu:52245	
K18424	ENPP4	mmu00230(	PF01663.27	GO:004227	cytoplasmic	NP_001344	mmu:224794	
K04725	XIAP, BIR(	mmu04120(	PF00653.27	GO:008009	cytoplasmic	NP_001288	mmu:11798	
K15181	TH1L, NEI	mmu03250(	PF04858.18	GO:000802	nuclear	BAE35440.	mmu:57314	
K24064	SNTB	mmu05410(	PF23012.2(S	GO:009951	mitochondr	BAE20762.	mmu:20650	
K12305	ENTPD4, I	mmu04142(	PF01150.23	GO:000918	cytoplasmic	BAC32317.	mmu:67464	
K20774	FAM175A,	mmu03440(	PF21125.3(I	GO:008009	nuclear	NP_765993	mmu:70681	
K00738	MGAT4A_	mmu00510(	PF04666.18	GO:000578	Golgi appa	NP_666038	mmu:103534	
K13105	PRCC	mmu05202(	PF10253.14	GO:001660	nuclear	NP_291051	mmu:94315	
K10840	CETN2	mmu03420(	PF13499.12	GO:002260	cytoplasmic	XP_027975	mmu:26370	
K25566	ISLR		PF13855.12	GO:000557	extracellulo	NP_001182	mmu:26968	
K14376	PAP	mmu03015(	PF20750.3(I	GO:003563	nuclear	XP_012423	mmu:18789	
K03965	NDUFB9	mmu05208(	PF05347.21	GO:002260	mitochondr	NP_075661	mmu:66218	
K17283	LTF		PF00405.22	GO:000425	lysosomal	NP_032548	mmu:17002	TF_others
K00081	CBR2	mmu00980(	PF00106.31	GO:001932	mitochondr	NP_031647	mmu:12409	
K11471	ASXL	mmu03083(	PF13919.11	GO:008009	nuclear	NP_001257	mmu:75302	
K02085	APC	mmu05165(	PF16629.10	GO:003042	cytoplasmic	NP_001389	mmu:11789	
K20897	GBP2	mmu04621(	PF02263.25	GO:000810	ER	NP_034390	mmu:14469	
K23313	TEN1		PF15490.12	GO:003304	cytoplasmic	NP_081383	mmu:69535	
K10645	MIB	mmu05417(	PF06701.19	GO:000548	cytoplasmic	XP_030109	mmu:76580	
K08772	SLC27A3, F	mmu04931(	PF00501.34	GO:001564	ER	AAI41135.1	mmu:26568	
K06230	GLI3	mmu05200(	PF23561.1(z	GO:003510	nuclear	NP_032156	mmu:14634	zf-C2H2
YBAR(Complement factor)			PF00084.26	GO:004858	extracellulo	AAI41137.1	mmu:214403	

K06676	BRRN1, BRN1, CAPH		PF05786.19	GO:003304	nuclear	NP_659067	mmu:215387	
K02730	PSMA6	mmu05020(	PF00227.31	GO:004423	cytoplasmic	NP_036098	mmu:26443	
K03234	EEF2	mmu04152(	PF00009.33	GO:008009	cytoplasmic	BAC26203.1	mmu:13629	
K02977	RP-S27Ae, 1	mmu04120(	PF00240.29	GO:003204	cytoplasmic	NP_001029	mmu:78294	
K03028	PSMD2, RF	mmu05020(	PF17781.7(I	GO:000989	nuclear	NP_598862	mmu:21762	
K07911	RAB20		PF00071.28	GO:000810	cytoplasmic	NP_035357	mmu:19332	
K07910	RAB18		PF00071.28	GO:005116	cytoplasmic	NP_851415	mmu:19330	
accharopine dehydrogenase)			PF03435.24	GO:001602	peroxisoma	BAE26412.1	mmu:109232	
K07912	RAB24		PF00071.28	GO:001678	cytoplasmic	NP_001015	mmu:19336	
K11222	STAT4	mmu04658(	PF01017.26	GO:001922	nuclear	P42228.1(R	mmu:20849	STAT
KWKI(selenoprotein K)			PF10961.13	GO:000578	cytoplasmic	AAF27311.1	mmu:80795	
K24635	SMYD5, ZMYND23		PF00856.33	GO:000988	cytoplasmic	NP_659167	mmu:232187	
YNYQ(LRRN4 C-terminal like)			PF00041.27	GO:000557	plasma mer	NP_001013	mmu:68852	
K07193	SH2B, APS	mmu04722(	PF08916.16	GO:003243	cytoplasmic	NP_001289	mmu:23921	
K01596	E4.1.1.32, p	mmu04910(	PF00821.23	GO:000167	cytoplasmic	BAC26903.1	mmu:18534	
K20161	DENND2		PF02141.26	GO:000557	cytoplasmic	NP_082386	mmu:72121	
ZNS0(Family with sequence similari			PF22749.2(	GO:005125	ER	NP_612185	mmu:68675	
K16820	WWTR1, T.	mmu04390(	PF00397.32	GO:001922	cytoplasmic	NP_598545	mmu:97064	
K13403	MTHFD2	mmu01240(	PF02882.25	GO:001678	mitochondr	NP_032664	mmu:17768	
K12192	CHMP2B	mmu05014(	PF03357.26	GO:000810	cytoplasmic	XP_036015	mmu:68942	
K08588	BAP1, UCH	mmu03083(	PF01088.27	GO:008009	nuclear	EDL24802.	mmu:104416	
K20293	COG6, COD2		PF20653.3(	GO:000810	cytoplasmic	NP_080501	mmu:67542	
K19676	IFT172		PF24762.1(	GO:000858	mitochondr	NP_080574	mmu:67661	
208Z(churchill domain containing 1			PF06573.16	GO:000548	nuclear	NP_996257	mmu:211151	
24Y3(Chromosome 6 open reading fi			PF17065.10	GO:000998	cytoplasmic	AAH27313.	mmu:67912	
K20254	PLIN4	mmu03320(	PF03036.21	GO:000573	cytoplasmic	EDL23717.	mmu:57435	
K01374	CTSO	mmu04142(	PF00112.29	GO:000417	lysosomal	AAH98219.	mmu:229445	
18NJ(Trophinin associated protein)				GO:000573	cytoplasmic	AAI00406.1	mmu:78733	
K24144	NHS		PF15273.11	GO:003250	cytoplasmic	NP_001392	mmu:215819	
K18806	KIF7	mmu05200(	PF00225.29	GO:000858	cytoplasmic	B7ZNG0.1(	mmu:16576	
21G3(Transmembrane protein 88)				GO:000548	cytoplasmic	NP_080191	mmu:67020	
K14823	EBP2, EBNA1BP2		PF05890.17	GO:003068	nuclear	NP_081208	mmu:69072	
K14824	ERB1, BOP1		PF08145.17	GO:000044	nuclear	BAE40096.1	mmu:12181	
K13707	ABHD15			GO:003433	extracellulo	NP_080461	mmu:67477	
K10751	CHAF1B		PF24105.1(I	GO:003472	nuclear	NP_001406	mmu:110749	
K23818	SRPX		PF13778.12	GO:200123	ER	NP_058607	mmu:51795	
K14559	MPP10	mmu03008(	PF04006.17	GO:003204	nuclear	NP_080759	mmu:67973	
K15201	GTP3C3, TFC4		PF07719.23	GO:003277	cytoplasmic	EDL00008.	mmu:98488	
K12893	SRSF4_5_6,	mmu03040(	PF00076.28	GO:003311	nuclear	Q8VE97.1(I	mmu:57317	
K02874	RP-L14, MI	mmu03010(	PF00238.24	GO:001593	mitochondr	NP_081008	mmu:68463	
K14169	CTU2, NCS	mmu04122(	PF10288.14	GO:003422	cytoplasmic	NP_722470	mmu:66965	
K05606	MCEE, epi	mmu00630(	PF13669.12	GO:000663	mitochondr	NP_082902	mmu:73724	
K05135	IL10RB, CI	mmu05163(	PF09294.16	GO:190185	plasma mer	XP_015857	mmu:16155	

K22182	TXNRD	mmu05200(	PF07992.20	GO:004428	cytoplasmic	NP_001035	mmu:50493	
K04457	PPM1A, PP	mmu04010(	PF00481.26	GO:003315	cytoplasmic	NP_032936	mmu:19042	
K01107	INPP1	mmu04070(	PF00459.30	GO:001679	cytoplasmic	NP_032410	mmu:16329	
K13139	INTS2		PF14750.11	GO:009030	cytoplasmic	AAI18008.1	mmu:70422	
K00002	AKR1A1, a	mmu01240(	PF00248.27	GO:000810	cytoplasmic	NP_067448	mmu:58810	
K17582	MKI67		PF08065.17	GO:190227	nuclear	NP_001074	mmu:17345	
K20371	NUCB		PF00036.39	GO:009008	extracellulo	NP_001123	mmu:53322	
K09408	FOXO3	mmu04917(	PF16675.10	GO:001922	nuclear	NP_001363	mmu:56484	Fork head
K17807	TAM41, MMP37		PF09139.17	GO:000665	mitochondr	NP_081170	mmu:68971	
K15433	CGI99, CLE7, RLLM1		PF10036.14	GO:005134	cytoplasmic	NP_080804	mmu:68045	
K12824	TCERG1, C	mmu03040(	PF01846.25	GO:014029	nuclear	NP_001034	mmu:56070	
K04409	PAK1	mmu04012(	PF00069.31	GO:003111	cytoplasmic	NP_001344	mmu:18479	
K22188	CLCC1		PF05934.16	GO:000578	ER	NP_001171	mmu:229725	
K13044	HNRNPABD		PF00076.28	GO:003577	nuclear	NP_001041	mmu:15384	
K08873	SMG1	mmu03015(	PF15785.10	GO:003577	mitochondr	NP_001026	mmu:233789	
K11308	MYST1, MOF, KAT8		PF01853.24	GO:008009	cytoplasmic	BAE20930.1	mmu:67773	
K05627	BAIAP2, IR	mmu04810(	PF08397.16	GO:005080	cytoplasmic	NP_001032	mmu:108100	
ZE8W(Transmembrane protein 123)			PF05283.16	GO:000989	extracellulo	NP_598500	mmu:71929	
K05021	CLIC1		PF22441.2((	GO:000548	cytoplasmic	BAE39798.1	mmu:114584	
K18441	CYTH	mmu04144(	PF01369.25	GO:001989	cytoplasmic	OBS60344.1	mmu:19158	
YE2(Small integral membrane pro			PF15875.10	GO:000548	plasma mer	NP_001157	mmu:68859	
K13221	WDR77, MEP50		PF24807.1(	GO:008009	cytoplasmic	NP_081708	mmu:70465	
K26544	SEC14, SEC14L		PF00650.25	GO:008009	cytoplasmic	NP_653103	mmu:67815	
K21407	APMAP		PF03088.22	GO:001678	Golgi appa	NP_082253	mmu:71881	
K22071	FDX2		PF00111.32	GO:002260	mitochondr	NP_001034	mmu:68165	
K09646	SCPEP1		PF00450.28	GO:001975	lysosomal	NP_083299	mmu:74617	
K08956	AFG3	mmu05017(	PF01434.24	GO:000417	mitochondr	NP_473411	mmu:114896	
K06062	PCAF, KAT	mmu04330(	PF06466.16	GO:003514	cytoplasmic	BAE29392.1	mmu:14534	
K09313	CUTL		PF02376.21	GO:008009	nuclear	NP_001278	mmu:13047	Homeobox
K15141	MED28		PF11594.13	GO:006026	cytoplasmic	NP_080171	mmu:66999	
K14822	CGR1		PF03879.19	GO:000557	nuclear	NP_076220	mmu:108673	
K07604	KRT1	mmu04915(	PF00038.26	GO:000810	cytoplasmic	NP_034794	mmu:16668	
K12735	PPIL4		PF00160.26	GO:000557	nuclear	BAB28194.1	mmu:67418	
K26420	FHIP1, FAM160A		PF10257.15	GO:000810	mitochondr	NP_001229	mmu:74349	
K05857	PLCD	mmu01100(	PF00388.25	GO:003250	mitochondr	NP_690026	mmu:72469	
VT9(LLP homolog, long-term synaj			PF10169.15	GO:004858	nuclear	NP_001404	mmu:66225	
K12471	EPN	mmu04144(	PF01417.26	GO:001602	cytoplasmic	BAE34326.1	mmu:71889	
K00627	DLAT, acel	mmu01100(	PF00198.28	GO:000599	mitochondr	NP_663589	mmu:235339	
K21989	TMEM63, CSC1		PF13967.11	GO:000526	ER	NP_001400	mmu:224807	
K15113	SLC25A28_37, MFRN		PF00153.32	GO:000004	cytoplasmic	NP_660138	mmu:246696	
K17295	TSPAN6		PF00335.26	GO:001602	plasma mer	XP_029409	mmu:56496	
K13094	RBM5_10		PF01585.28	GO:002260	nuclear	NP_001346	mmu:83486	
K02921	RP-L37Ae,	mmu05171(	PF01780.25	GO:002262	cytoplasmic	NP_000989	mmu:19981	

K09592	EGLN, HP	mmu05200(	PF13640.12	GO:008009	nuclear	XP_031197	mmu:112405	
K09558	BAG4	mmu04668(	PF02179.21	GO:000810	cytoplasmic	NP_080397	mmu:67384	
K25543	GLB1L		PF01301.25	GO:004423	lysosomal	XP_011237	mmu:74577	
Protein phosphatase 2, regulatory su			PF13855.12	GO:000699	cytoplasmic	NP_001297	mmu:70144	
K07879	RAB4A, RA	mmu04144(	PF00071.28	GO:000810	cytoplasmic	NP_033029	mmu:19341	
K25710	NPTXR		PF00354.24	GO:003303	Golgi appa	NP_109614	mmu:73340	
K02216	CHEK1	mmu05170(	PF00069.31	GO:000699	cytoplasmic	NP_001397	mmu:12649	
K24421	NRAP	mmu04820(	PF00880.23	GO:001716	cytoplasmic	NP_032759	mmu:18175	
K12820	DHX15, PR	mmu03040(	PF07717.22	GO:004858	cytoplasmic	NP_031865	mmu:13204	
K01092	E3.1.3.25, II	mmu04070(	PF00459.30	GO:001679	cytoplasmic	O55023.1(R	mmu:114663	
K17278	PGRMC1_2	mmu04080(	PF00173.33	GO:000578	ER	NP_058063	mmu:53328	
K25668	FEZ2		PF07763.18	GO:000989	cytoplasmic	NP_001272	mmu:225020	
K06553	VPREB, CD179a		PF07654.21	GO:000548	lysosomal	AAH04786.	mmu:22363	
K01509	ENTPD2	mmu01100(	PF01150.23	GO:004643	extracellula	NP_033979	mmu:12496	
K16848	IQGAP1	mmu04810(	PF00616.24	GO:004340	cytoplasmic	NP_057930	mmu:29875	
K13084	TMEM55	mmu04070(	PF09788.14	GO:001679	cytoplasmic	NP_082540	mmu:72519	
K17302	COPB2, SEC27		PF04053.20	GO:000810	cytoplasmic	NP_056642	mmu:50797	
K11493	RCC1		PF00415.24	GO:004239	cytoplasmic	NP_001184	mmu:100088	
K21392	AEBP1		PF00246.29	GO:000097	ER	NP_033766	mmu:11568	
K07962	ARL13B, ARL2L1		PF00025.27	GO:000810	cytoplasmic	NP_080853	mmu:68146	
K17252	CLU	mmu04610(	PF01093.22	GO:200123	ER	NP_001409	mmu:12759	
Y8WT(NCK interacting protein witl			PF09431.15	GO:002260	cytoplasmic	NP_109654	mmu:80987	
XQN5(UPF0378 protein)			PF00071.28	GO:003563	nuclear	NP_001019	mmu:227624	
K23957	FMNL	mmu04820(	PF02181.28	GO:002260	cytoplasmic	NP_001390	mmu:57778	
K17921	SNX7_30		PF00787.29	GO:000989	cytoplasmic	NP_766056	mmu:209131	
K14568	EMG1, NE	mmu03008(	PF03587.19	GO:003204	nuclear	NP_038564	mmu:14791	
K09573	FKBP7		PF00254.34	GO:000578	ER	NP_034352	mmu:14231	
K12641	LMNA	mmu05410(	PF00038.26	GO:200123	nuclear	NP_001002	mmu:16905	
K02999	RPA1, POL	mmu03020(	PF04998.22	GO:003288	nuclear	NP_033114	mmu:20019	
K15224	BAZ2A, TII	mmu03082(	PF01429.25	GO:014003	nuclear	XP_011241	mmu:11684	MBD
K16808	NUMA1			GO:003111	cytoplasmic	NP_001390	mmu:101706	
K05748	WASF2	mmu05231(	PF02205.27	GO:004846	cytoplasmic	NP_001406	mmu:242687	
K13887	CORO2		PF08953.17	GO:003243	cytoplasmic	NP_780693	mmu:235431	
K03984	SERPINA1	mmu04610(	PF00079.25	GO:000695	extracellula	NP_033270	mmu:20701	
K24629	UBAP1		PF22567.2(l	GO:000810	cytoplasmic	NP_001342	mmu:67123	
K22700	ZCCHC2		PF00098.28	GO:000573	cytoplasmic	NP_001116	mmu:227449	
K15306	RANBP1	mmu05203(	PF00638.24	GO:005116	cytoplasmic	NP_035369	mmu:19385	
K15465	BNIP3L	mmu04137(	PF06553.17	GO:000578	cytoplasmic	BAE40370.1	mmu:12177	
K12569	EMD	mmu05410(	PF03020.21	GO:003315	nuclear	NP_031953	mmu:13726	
K13088	ELAVL1, H	mmu04152(	PF00076.28	GO:005116	cytoplasmic	BAC40744.1	mmu:15568	
K12862	PLRG1, PR	mmu03040(	PF00400.38	GO:003450	nuclear	NP_058064	mmu:53317	
K11317	TADA1, STAF42		PF12767.12	GO:003124	nuclear	NP_084521	mmu:27878	
K04659	THBS2S	mmu05165(	PF05735.19	GO:200018	extracellula	NP_035711	mmu:21826	

K02161	BCL2	mmu05022(	PF00452.24	GO:200123	cytoplasmic	NP_033871	mmu:12043	
K10590	TRIP12	mmu04120(Ubiquitin m		GO:000020	mitochondr	AAY14755.	mmu:14897	
K10105	LIPT1	mmu01240(	PF21948.1(I	GO:004322	mitochondr	BAC33210.	mmu:623661	
K26226	PPFIA		PF00536.37(SAM_1:SAI		nuclear	XP_006508	mmu:233977	
K10754	RFC1	mmu03030(	PF08519.17	GO:008009	nuclear	NP_001334	mmu:19687	
K15173	TTF2	mmu04918(	PF00176.29	GO:003277	nuclear	NP_001013	mmu:74044	
K07370	BTK	mmu04664(	PF07714.23	GO:001989	cytoplasmic	NP_038510	mmu:12229	
K15338	GEN1, YEN1		PF00867.23	GO:001710	nuclear	NP_796305	mmu:209334	
XQM0(Transmembrane protein 115)			PF08551.15	GO:004322	ER	NP_062678	mmu:56395	
K20135	F8A			GO:000989	cytoplasmic	NP_032004	mmu:14070	
K23952	VMA21		PF09446.15	GO:002260	peroxisoma	NP_001074	mmu:67048	
K22075	BOLA3		PF01722.23	GO:003197	cytoplasmic	NP_001350	mmu:78653	
K08768	PLIN1	mmu03320(	PF03036.21	GO:000578	cytoplasmic	NP_001106	mmu:103968	
K15272	SLC35A1_2_3		PF04142.21	GO:001593	ER	KAI259936	mmu:22232	
K21434	ANKRD2	mmu04820(	PF00023.36	GO:009951	nuclear	NP_064417	mmu:56642	
K16536	HOOK3	mmu04814(	PF05622.17	GO:000024	cytoplasmic	NP_997542	mmu:320191	
K24594	RSRC1			GO:005116	nuclear	NP_001343	mmu:66880	
K09329	PRRX, PMX		PF00046.35	GO:000858	nuclear	NP_001020	mmu:18933	Homeobox
K03024	RPC7, POL	mmu04623(	PF11705.13	GO:003277	nuclear	NP_001280	mmu:69870	
K08593	SENP3		PF02902.25	GO:000989	nuclear	NP_001157	mmu:80886	
TEU(zinc finger protein 395)			PF15997.11	GO:000097	nuclear	NP_950194	mmu:38091	TF_others
K10356	MYO1	mmu04814(	PF00063.27	GO:009951	cytoplasmic	NP_848534	mmu:246177	
K24736	WDR1, AIP1		PF00400.38	GO:004521	cytoplasmic	NP_035845	mmu:22388	
K12307	MSFD8, CI	mmu04142(Lysosome)		GO:003250	plasma mer	NP_082416	mmu:72175	
K10425	DCTN3	mmu05014(	PF07426.16	GO:009859	cytoplasmic	NP_058586	mmu:53598	
K12852	EFTUD2	mmu03040(	PF00009.33	GO:004316	nuclear	NP_001103	mmu:20624	
K11152	RDH11	mmu00830(	PF00106.31	GO:000403	extracellulo	NP_067532	mmu:17252	
XSM4(Ectonucleoside triphosphate			PF03637.22	GO:000548	cytoplasmic	NP_766045	mmu:208228	
K09097	ARNT	mmu05208(	PF14598.12	GO:008009	nuclear	NP_001032	mmu:11863	bHLH
K03833	selB, EEFSEC		PF21131.3(	GO:000645	cytoplasmic	NP_075547	mmu:65967	
K13566	NIT2, yafV	mmu00250(	PF00795.27	GO:010600	mitochondr	NP_075664	mmu:52633	
K13336	PEX3	mmu04146(	PF04882.17	GO:004357	peroxisoma	NP_064345	mmu:56535	
K21852	DOCK6_7_8		PF06920.19	GO:003023	cytoplasmic	NP_796004	mmu:319899	
K00286	proC	mmu00330(	PF14748.11	GO:001649	mitochondr	NP_079688	mmu:66194	
K12488	ASAP	mmu04666(	PF01412.23	GO:000573	cytoplasmic	NP_001004	mmu:211914	
K13208	ELAVL2_3_4		PF00076.28	GO:008009	cytoplasmic	XP_036019	mmu:15569	
YQB2(immunoglobulin kappa varia			PF07686.23	GO:000237	extracellulo	CAB46311.1(immunoglobulin light		
K10885	XRCC5, KI	mmu03450(	PF03731.21	GO:003210	cytoplasmic	NP_033559	mmu:22596	
XRNB(Zinc finger protein 609)				GO:003308	nuclear	AAI50684.1	mmu:214812	
)155(Transmembrane protein 248)				GO:001602	cytoplasmic	NP_081103	mmu:68742	
K10428	DCTN6	mmu05022(	PF00132.30	GO:000367	cytoplasmic	NP_001280	mmu:22428	
K13161	HNRNPR		PF00076.28	GO:000989	cytoplasmic	BAE30898.	mmu:74326	
K17095	ANXA7_11	mmu05014(	PF00191.25	GO:009008	cytoplasmic	NP_001361	mmu:11750	

K05664	ABCB11	mmu02010(	PF00664.28	GO:008009	cytoplasmic	NP_001350	mmu:27413	
K11377	ELP6		PF09807.15	GO:000945	nuclear	NP_001074	mmu:72341	
8M9(coiled-coil domain containing			PF15374.11	(CCDC71L:	cytoplasmic	NP_001156	mmu:72123	
TUV(Transmembrane protein 254)			PF14934.11	GO:000557	Golgi appa	NP_080955	mmu:66039	
K03008	RPB11, PO	mmu03420(	PF13656.11	GO:003088	cytoplasmic	CAC18368.	mmu:20022	
K14571	RIX7, NVL	mmu03008(	PF00004.35	GO:000367	nuclear	NP_080447	mmu:67459	
K10788	EPX, EPO	mmu05310(	PF03098.20	GO:001922	extracellula	NP_031972	mmu:13861	
K12076	DLG1	mmu05165(	PF00625.27	GO:000810	cytoplasmic	XP_006521	mmu:13383	
K24262	SPARC		PF10591.15	GO:007149	extracellula	XP_050013	mmu:20692	
K08805	DCLK1_2		PF00069.31	GO:003450	cytoplasmic	EDL15373.	mmu:70762	
K23961	EPB41L1_2_3		PF05902.18	GO:009859	nuclear	NP_001278	mmu:13821	
K06553	VPREB, CD179a		PF07686.23	GO:000548	plasma mer	DBA12440.	mmu:22363	
K19414	PHF2, JHDM1E		PF17811.6(J	GO:003506	nuclear	NP_035208	mmu:18676	
K24183	NBEA		PF15787.11	GO:001989	cytoplasmic	NP_085098	mmu:26422	
K08574	CAPN5		PF00648.27	GO:000417	cytoplasmic	NP_001288	mmu:12337	
K24931	TTC17		PF07719.23	GO:002260	ER	XP_006500	mmu:74569	
K25731	VPS35L			GO:000810	cytoplasmic	NP_082091	mmu:71517	
K10482	BTBD10		PF16017.11	GO:001922	cytoplasmic	XP_021047	mmu:68815	
K14590	CMTR2, FTSJD1, AFT		PF01728.25	GO:000815	cytoplasmic	NP_666327	mmu:234728	
K22382	WDR26		PF00400.38	GO:199023	nuclear	NP_663489	mmu:226757	
K19827	MACF1		PF00435.27	GO:000810	cytoplasmic	XP_030108	mmu:11426	
K07598	DSG3		PF00028.23	GO:000367	plasma mer	BAC26246.	mmu:13512	
K20463	OSBPL3_6_7, ORP3_6_		PF01237.23	GO:003243	nuclear	NP_001334	mmu:71720	
K15691	RFWD3		PF23419.1(	GO:004858	cytoplasmic	NP_666330	mmu:234736	
K24536	ZZZ3		PF00249.37	GO:003124	nuclear	NP_001274	mmu:10894	MYB
K06478	PTPRC, CD	mmu04666(	PF00102.32	GO:004209	plasma mer	NP_001104	mmu:19264	
K00029	maeB	mmu03320(	PF03949.21	GO:001922	cytoplasmic	NP_032641	mmu:17436	
K01623	ALDO	mmu00051(	PF00274.25	GO:004643	cytoplasmic	NP_001170	mmu:11674	
K08336	ATG12	mmu04140(	PF04110.19	GO:003472	cytoplasmic	NP_080493	mmu:67526	
SP8(Cas scaffolding protein family			PF08824.15	GO:001922	cytoplasmic	NP_001263	mmu:320664	
K01623	ALDO	mmu00051(	PF00274.25	GO:004643	cytoplasmic	NP_001290	mmu:11676	
K08023	LTBP2_3_4		PF07645.21	GO:002260	extracellula	AAI41006.1	mmu:108075	
K17879	NUDT7	mmu04146(	PF00293.34	GO:000578	peroxisoma	NP_077757	mmu:67528	
K23583	KPNA3_4	mmu03013(	PF00514.29	GO:003450	cytoplasmic	AQS27613.	mmu:16649	
K11457	PHC2, EDI	mmu03083(	PF16616.10	GO:004239	nuclear	CAQ52299.	mmu:54383	
K14529	RPP14		PF01900.24	GO:000454	cytoplasmic	NP_080214	mmu:67053	
K26608	TMEM120		PF07851.18	GO:002260	cytoplasmic	NP_766129	mmu:215210	
K19951	TBC1D8_9		PF00566.25	GO:000367	cytoplasmic	NP_001074	mmu:245638	
K06063	SNW1, SKI	mmu04330(	PF02731.20	GO:004423	nuclear	NP_079783	mmu:66354	
K07296	ADIPOQ,	mmu04152(	PF00386.27	GO:003250	ER	SOR70272.1	mmu:11450	
K03164	TOP2	mmu01524(	PF00521.26	GO:008009	nuclear	NP_033435	mmu:21974	
K01280	TPP2		PF12580.14	GO:000417	cytoplasmic	NP_033444	mmu:22019	
K23553	PGAP3, PER1		PF04080.19	GO:000665	extracellula	NP_001028	mmu:320655	

K01641	HMGCS	mmu00650(	PF08540.16	GO:000663'	cytoplasmic	BAE27285.1	mmu:208715	
K17795	TIM17		PF02466.24	GO:000810	cytoplasmic	BAE29896.1	mmu:21855	
K03898	KNG, BK	mmu04750(	PF00031.27	GO:000759	extracellulo	NP_001095	mmu:385643	
K11806	DCAF13, WDSOF1		PF04158.19	GO:001922	nuclear	NP_941008	mmu:223499	
K20119	BIN2		PF21532.2(I	GO:004299	cytoplasmic	D3Z6Q9.1(	mmu:668218	
K12311	MAN2B1, I	mmu04142(	PF01074.27	GO:000032	lysosomal	NP_034894	mmu:17159	
K00587	ICMT, STE	mmu00900(	PF04140.19	GO:000810	ER	NP_598549	mmu:57295	
K01411	NRD1		PF16187.10	GO:005104	cytoplasmic	NP_001334	mmu:230598	
K24135	MORC		PF17942.8(	GO:004239	nuclear	AAI32154.1	mmu:75746	
K00898	PDK2_3_4	mmu05415(	PF10436.14	GO:001922	mitochondr	NP_598428	mmu:18604	
K17436	MRPL55		PF09776.14	GO:001593	mitochondr	NP_001289	mmu:67212	
K23294	PACS2		PF10254.14	GO:000548	cytoplasmic	NP_001278	mmu:217893	
K00411	UQCRFS1,	mmu05208(	PF02921.19	GO:004323	mitochondr	NP_079986	mmu:66694	
K07605	KRT2		PF00038.26	GO:009951	mitochondr	NP_034797	mmu:16679	
K03963	NDUFB7	mmu05208(	PF05676.18	GO:003197	cytoplasmic	NP_080119	mmu:66916	
K26683	GOLT1, GOT1		PF04178.17	GO:000810	extracellulo	NP_080148	mmu:66964	
T3A(PDGFA associated protein 1)			PF10252.14	GO:000367	cytoplasmic	XP_031194	mmu:231887	
K21970	NSUN4		PF01189.23	GO:007013	cytoplasmic	NP_082418	mmu:72181	
K10685	UBLE1B, S	mmu04120(	PF00899.27	GO:190187	cytoplasmic	NP_057891	mmu:50995	
K04706	PIAS1	mmu04120(	PF14324.12	GO:003243	cytoplasmic	NP_062637	mmu:56469	zf-MIZ
K04515	CAMK2	mmu04750(	PF00069.31	GO:004858	cytoplasmic	NP_001125	mmu:12322	
K01409	OSGEP, KAE1, QRI7		PF00814.31	GO:199023	cytoplasmic	NP_598437	mmu:66246	
)5JD(Protein of unknown function (			PF12576.13	GO:003250	peroxisoma	BAC30266.1	mmu:70209	
3DA(SPHK1 interactor, AKAP don			PF05716.18	GO:000573	nuclear	NP_001392	mmu:77629	
K08064	NFYA, HA	mmu04612(	PF02045.20	GO:000097	nuclear	NP_001334	mmu:18044	NF-YA
K18615	RCSD1, CAPZIP		PF15255.11	GO:004323	cytoplasmic	NP_848708	mmu:226594	
K20642	ARHGAP22_24_25		PF00620.32	GO:004858	cytoplasmic	AAI38753.1	mmu:232201	
K10137	ZMAT3	mmu04115(	PF12874.13	GO:000810	nuclear	NP_033543	mmu:22401	zf-C2H2
K00671	NMT		PF02799.21	GO:000810	cytoplasmic	NP_032733	mmu:18107	
K14003	PREB, SEC	mmu04141(	PF00400.38	GO:000810	cytoplasmic	NP_057912	mmu:50907	
K14299	SEH1	mmu03013(	PF00400.38	GO:005116	nuclear	NP_001034	mmu:72124	
K22384	GET1, WR	mmu03060(	PF04420.19	GO:000810	ER	NP_997184	mmu:71446	
K16219	NTMT1, METTL11A, N		PF05891.18	GO:000647	cytoplasmic	NP_001343	mmu:66617	
K22999	TOR2A		PF06309.17	GO:000645	ER	NP_690013	mmu:30933	
K04630	GNAI	mmu04730(	PF00503.26	GO:004858	cytoplasmic	NP_001116	mmu:14677	
K13100	CWC22		PF02847.23	GO:007100	nuclear	NP_001277	mmu:80744	
K25867	TPPP		PF05517.18	GO:009951	cytoplasmic	NP_878259	mmu:72948	
G9C(SPRY domain containing 4)			PF00622.36	GO:004323	mitochondr	NP_079992	mmu:66701	
K22653	NPTN		PF13927.12	GO:004340	ER	NP_001280	mmu:20320	
K02437	gcvH, GCSl	mmu00630(	PF01597.25	GO:000652	mitochondr	NP_080848	mmu:68133	
XNTY(Zinc finger protein 330)			PF06524.17	GO:004322	cytoplasmic	NP_663575	mmu:30932	
K09272	SSRP1, POB3		PF21103.3(I	GO:190227	nuclear	AAH42502.	mmu:20833	HMG
K10635	RNF111		PF15303.11	GO:008009	nuclear	NP_001361	mmu:93836	

K18170	LYRM7, MZM1		PF05347.21	GO:002260	cytoplasmic	XP_021032	mmu:75530	
K01939	purA, ADS	mmu01240(	PF00709.27	GO:000401	cytoplasmic	NP_031448	mmu:11566	
K09406	FOXM	mmu04218(	PF00250.24	GO:008009	nuclear	NP_032047	mmu:14235	Fork head
K01514	PRUNE, PI	mmu00230(	PF02833.19	GO:003111	cytoplasmic	NP_775482	mmu:229589	
Z48(May have a role in the cell cyc			PF01133.22	GO:004323	nuclear	NP_001029	mmu:13877	
K17590	CDCA2		PF15276.11	GO:003304	nuclear	NP_001103	mmu:108912	
K15294	CPLX1_2	mmu04721(	PF05835.17	GO:000688	nuclear	OBS65475.1	mmu:12890	
K07940	ARF5	mmu04144(	PF00025.27	GO:000810	mitochondr	NP_001077	mmu:11844	
K18617	AIF1		PF21008.3(	GO:001491	cytoplasmic	ABK96804.	mmu:11629	
K16063	PIAS2	mmu04120(	PF14324.12	GO:008009	mitochondr	NP_001157	mmu:17344	zf-MIZ
K04645	CLTB, LCB	mmu04721(	PF01086.22	GO:009964	cytoplasmic	BAE39600.	mmu:74325	
K10642	DZIP3		PF18738.6(I	GO:001989	cytoplasmic	NP_001103	mmu:224170	
K24388	ZC3H8		PF18345.7(	GO:008009	cytoplasmic	BAE21298.	mmu:57432	
K13676	GXYLT	mmu00514(	PF01501.26	GO:000367	Golgi appa	NP_001333	mmu:223827	
K22066	BOLA1		PF01722.23	GO:003299	mitochondr	NP_081251	mmu:69168	
K12757	MYL12	mmu04530(	PF13499.12	GO:003243	cytoplasmic	NP_001128	mmu:67268	
K23569	EMC8_9		PF03665.18	GO:000810	cytoplasmic	NP_149158	mmu:85308	
K12160	SUMO, SM	mmu03013(	PF11976.14	GO:003450	cytoplasmic	EDL31801.	mmu:170930	
XPEV(cleft lip and palate)			PF05602.17	GO:000989	plasma mer	NP_062623	mmu:56457	
K11170	DHRSX		PF00106.31	GO:000989	extracellula	Q8VBZ0.2(	mmu:236082	
K23332	RSPRY1		PF00622.36	GO:000367	extracellula	NP_001346	mmu:67610	
YEVU(immunoglobulin superfamily			PF07679.22	GO:000150	extracellula	NP_001156	mmu:242050	
K09228	KRAB		PF13465.12	GO:000097	cytoplasmic	AAI39792.1	mmu:63057	zf-C2H2
K00849	galK	mmu00052(	PF10509.15	GO:004643	cytoplasmic	AAH50151.	mmu:14635	
K12884	HNRNPC	mmu03040(	PF00076.28	GO:009030	nuclear	BAE31679.	mmu:15381	
K19679	IFT74			GO:008009	nuclear	NP_080595	mmu:67694	
K07435	CYP20A		PF00067.27	GO:004316	ER	NP_084289	mmu:77951	
K24220	MYH1s	mmu04814(	PF00063.27	GO:001645	cytoplasmic	NP_001074	mmu:544791	
K22555	TBC1D23		PF19430.4(TBC1D23_C		cytoplasmic	NP_001345	mmu:67581	
K06553	VPREB, CD179a		PF07686.23	GO:000237	cytoplasmic	ANG60751.	mmu:22364	
K03024	RPC7, POL	mmu04623(	PF11705.13	GO:001922	nuclear	NP_001074	mmu:67486	
K05089	PDGFRB, (	mmu05165(	PF07714.23	GO:003197	plasma mer	BAE22283.	mmu:18596	
K22078	PGGHG, ATHL1		PF03632.21	GO:001592	cytoplasmic	NP_663362	mmu:212974	
JHV(SRR1 domain containing)			PF07985.18	GO:000573	cytoplasmic	NP_001346	mmu:70118	
K08118	BGN	mmu04820(	PF13855.12	GO:005084	extracellula	BAE42709.	mmu:12111	
K20194	HPS4		PF19031.6(I	GO:003303	cytoplasmic	BAE42710.	mmu:192232	
K07526	SRGAP	mmu04360(	PF00620.32	GO:000573	cytoplasmic	XP_006506	mmu:259302	
)A7R(PWWP domain); ENOG41127			PF00855.23	GO:003111	cytoplasmic	BAE37140.	mmu:29877	
K04437	FLNA	mmu04010(	PF00630.25	GO:003243	cytoplasmic	NP_034357	mmu:192176	
K00920	PIP4K2	mmu04070(	PF01504.24	GO:001922	cytoplasmic	NP_032871	mmu:18718	
XQKW(CBP80 20-dependent transla			PF02854.25	GO:000018	nuclear	NP_001395	mmu:269037	
K08515	VAMP7	mmu04130(	PF00957.27	GO:003288	extracellula	NP_035645	mmu:20955	
K23327	HIKESHI		PF05603.17	GO:003450	cytoplasmic	NP_001385	mmu:67669	

K11322	EPC	mmu03082(	PF06752.17	GO:008009	nuclear	NP_081773	mmu:13831	
K11275	H1_5		PF00538.25	GO:003472	nuclear	XP_021036	mmu:107970	
K02433	gatA, QRSI	mmu00970(	PF01425.27	GO:003164	cytoplasmic	NP_001074	mmu:76563	
K00600	glyA, SHM	mmu01240(	PF00464.25	GO:008009	mitochondr	NP_082506	mmu:108037	
?0RS(coactosin-like 1 (Dictyostelium			PF00241.25	GO:009951	nuclear	NP_082347	mmu:72042	
K06226	SMO	mmu05200(	PF01534.23	GO:000959	plasma mer	NP_795970	mmu:319757	
K15429	TRM5, TRMT5		PF02475.23	GO:000817	cytoplasmic	EDL36512.	mmu:76357	
K11092	SNRPA1	mmu03040(	PF14580.12	GO:007100	nuclear	NP_067311	mmu:68981	
K08180	SLC16A3	mmu05230(	PF07690.21	GO:009859	plasma mer	NP_001033	mmu:80879	
K21918	KCTD8_12_16		PF23110.1(	GO:002260	cytoplasmic	NP_780638	mmu:207474	
K14638	SLC15A3_4, PHT, NPF		PF00854.27	GO:001564	cytoplasmic	NP_075531	mmu:65221	
K25816	CCAR2, DI	mmu04310(	PF14443.11	GO:200123	cytoplasmic	NP_666167	mmu:219158	
K22594	POGZ		PF03184.25	GO:002260	nuclear	NP_766271	mmu:229584	
K24996	STEEP1			GO:000810	nuclear	NP_001101	mmu:77644	
K00231	PPOX, hem	mmu01240(	PF01593.30	GO:003197	mitochondr	NP_032937	mmu:19044	
K24031	TMPO		PF11560.13	GO:001602	nuclear	NP_035735	mmu:21917	
K19671	WDR19, IFT144		PF23389.1(	GO:003510	mitochondr	NP_001346	mmu:213081	
K13182	DDX39		PF00270.35	GO:001593	cytoplasmic	BAE43010.	mmu:68278	
K13170	PNISR, SFRS18		PF15996.10	GO:001660	nuclear	AAI41032.1	mmu:66625	
Jbiquitin-conjugating enzyme E2)			PF14572.12	GO:000815	mitochondr	NP_080640	mmu:67763	
K06531	NECTIN2,	mmu03266(	PF08205.18	GO:000285	plasma mer	NP_033016	mmu:19294	
K15499	PPP6R1, SAPS1		PF04499.20	GO:000557	nuclear	AAI41040.1	mmu:243819	
K00308	PAOX	mmu04146(	PF01593.30	GO:000659	mitochondr	CAJ18113.1	mmu:212503	
K06557	PROCR, CI	mmu04610(	PF16497.10	GO:005124	plasma mer	NP_035301	mmu:19124	
Z9YG(transmembrane protein 176A			PF04103.21	GO:001602	plasma mer	NP_001091	mmu:66058	
K15183	ELL	mmu03250(	PF10390.15	GO:008009	cytoplasmic	NP_031950	mmu:13716	
K04718	SPHK	mmu04666(	PF00781.29	GO:005080	cytoplasmic	NP_001165	mmu:20698	
Hydrolyzes cAMP to 5'-AMP. Plays c			PF13465.12	GO:004671	nuclear	NP_001155	mmu:67778	zf-C2H2
K11725	LHPP	mmu00190(	PF13242.12	GO:001679	cytoplasmic	NP_083885	mmu:76429	
K17443	HOXA10	mmu05202(	PF00046.35	GO:003510	nuclear	NP_032289	mmu:15395	Homeobox
K10631	TOPORS		PF00097.31	GO:000810	nuclear	NP_598858	mmu:106021	
K16805	FAM83D, CHICA		PF07894.18	GO:190248	cytoplasmic	NP_082251	mmu:71878	
K12823	DDX5, DB	mmu05202(	PF00270.35	GO:005116	cytoplasmic	BAE21355.	mmu:13207	
K19953	GRTP1, TBC1D6, MSB		PF00566.25	GO:003023	cytoplasmic	NP_001026	mmu:66790	
K20876	NEK7	mmu04621(	PF00069.31	GO:008009	cytoplasmic	NP_067618	mmu:59125	
K20875	NEK6		PF00069.31	GO:004858	cytoplasmic	NP_001153	mmu:59126	
K15200	GTF3C2		PF24807.1(	GO:003277	nuclear	BAE23772.	mmu:71752	
K09583	PDIA5		PF00085.26	GO:000645	ER	NP_082571	mmu:72599	
Y78B(myeloid cell nuclear differenti			PF02760.21	GO:008009	nuclear	ASD34094.	mmu:226695	
K09550	PFDN4		PF01920.25	GO:000989	cytoplasmic	NP_001186	mmu:109054	
.6JK(odorant binding); ENOG4111I			PF00061.29	GO:000554	extracellulo	AIA65153.1	mmu:18249	
YR8R(CTTNBP2 N-terminal like)			PF09727.14	GO:003243	cytoplasmic	BAC98169.	mmu:80281	
K08594	SENP5		PF19722.4(	GO:007013	nuclear	NP_001344	mmu:320213	

K26978	ZNF526_574		PF00096.32	GO:000097	nuclear	NP_001161	mmu:23297	zf-C2H2
K10611	RBX2, ROC	mmu04120(	PF12678.13	GO:000827	cytoplasmic	NP_001100	mmu:19823	
K18438	PDE10	mmu04024(cAMP signa		GO:000921	cytoplasmic	XP_017172	mmu:23984	
K25493	DKK3		PF04706.18	GO:008009	extracellula	NP_001347	mmu:50781	
K19177	NUS1	mmu00900(	PF01255.24	GO:005104	Golgi appa	NP_084526	mmu:52014	
K01076	ABHD17		PF12146.15	GO:009859	plasma mer	NP_001014	mmu:226016	
K10420	DYNLT1_3	mmu05132(	PF03645.18	GO:003058	cytoplasmic	NP_001186	mmu:100310872	
K24825	ARMC7		PF00514.29	GO:001607	cytoplasmic	NP_808446	mmu:276905	
K11498	CENPE, KI	mmu04814(	PF00225.29	GO:000810	cytoplasmic	NP_776123	mmu:229841	
K16769	CKAP2		PF15297.11	GO:003111	cytoplasmic	NP_001004	mmu:80986	
K21842	EFR3		PF21052.3(I	GO:000810	cytoplasmic	XP_052603	mmu:76740	
K26934	ATP11	mmu04148(	PF16212.11	GO:014032	ER	NP_001001	mmu:320940	
K22125	MYOF		PF00168.36	GO:009859	cytoplasmic	NP_001289	mmu:226101	
K16551	AKAP9, AKAP350		PF10495.14	GO:003111	cytoplasmic	NP_919444	mmu:100986	
K10377	SYNC1		PF00038.26	GO:000573	cytoplasmic	XP_006503	mmu:68828	
K08869	ADCK, ABC1		PF03109.22	GO:000548	mitochondr	NP_001264	mmu:72113	
K13823	HBB	mmu05144(	PF00042.28	GO:003172	cytoplasmic	BAG16710.	mmu:101488143	
K17922	SNX8, MVP1		PF00787.29	GO:000810	cytoplasmic	BAE33864.1	mmu:231834	
K24814	HEATR5, LAA1		PF20210.4(I	GO:000810	cytoplasmic	NP_001074	mmu:320473	
K00565	RNMT	mmu03015(	PF03291.22	GO:004423	nuclear	NP_001347	mmu:67897	
K14407	CSTF2, RN	mmu03015(	PF14327.11	GO:009030	cytoplasmic	NP_112539	mmu:83410	
YP91(Transmembrane protein 135)			PF15982.10	GO:004239	peroxisoma	EDL06791.	mmu:72759	
K09278	RUNX2, AI	mmu04928(	PF00853.24	GO:001707	cytoplasmic	XP_007530	mmu:12393	Runt
K25219	SYNRG		PF12763.14	GO:000810	nuclear	Q5SV85.1(F	mmu:217030	
K07953	SAR1	mmu04141(	PF00025.27	GO:003315	extracellula	NP_079811	mmu:66397	
K15370	PLCL2		PF00388.25	GO:001922	cytoplasmic	NP_038908	mmu:224860	
K05220	P2RX7	mmu04621(	PF00864.24	GO:000526	cytoplasmic	AAI28304.1	mmu:18439	
neiotic nuclear divisions 1 homolog			PF03962.21	GO:190304	nuclear	NP_084073	mmu:76915	
K13902	CST6		PF00031.27	GO:003041	extracellula	AAH27680.	mmu:73720	
K12627	LSM8	mmu03018(	PF01423.28	GO:000613	nuclear	NP_001029	mmu:76522	
K05858	PLCB	mmu04750(	PF08703.15	GO:000959	cytoplasmic	NP_808236	mmu:18796	
SUS(AhpC TSA antioxidant enzym			PF13911.12	GO:000989	mitochondr	BAE26709.1	mmu:66129	
K23562	EMC1		PF07774.18	GO:000810	ER	AAI29805.1	mmu:230866	
K01895	ACSS1_2, a	mmu00640(	PF00501.34	GO:001564	mitochondr	BAE34365.1	mmu:68738	
K08823	CLK2_3		PF00069.31	GO:008009	nuclear	BAE39405.1	mmu:102414	
K20408	WDR24, SE	mmu04150(	PF24807.1(	GO:000810	mitochondr	NP_776102	mmu:268933	
YCNM(TSC22 domain family, meml			PF01166.23	GO:008009	cytoplasmic	NP_001074	mmu:72033	TSC22
XSI8(chromosome 1 open reading fr			PF14778.11	GO:000810	plasma mer	NP_663486	mmu:226499	
K14638	SLC15A3_4, PHT, NPF		PF00854.27	GO:001564	plasma mer	NP_598656	mmu:100561	
K13809	CMPK2	mmu01240(	PF02223.23	GO:000921	mitochondr	NP_065582	mmu:22169	
K06706	CD248, CD164L1		PF14670.12	GO:000989	cytoplasmic	AAK84664.	mmu:70445	
K13155	SNRNP35		PF00076.28	GO:000613	nuclear	NP_083808	mmu:76167	
K20092	CHD1L		PF00176.29	GO:003563	nuclear	NP_080815	mmu:68058	

K20710	ARHGEF37		PF00621.25	GO:000573'	cytoplasmic	A1IGU4.1(I	mmu:328967	
K01116	PLCG1	mmu04750(	PF00388.25	GO:004643	cytoplasmic	XP_021012	mmu:18803	
K14819	DUSP12, YVH1		PF00782.25	GO:001679	nuclear	NP_075662	mmu:80915	
)7NW(sec1 family domain containir			PF00995.28	GO:007170	cytoplasmic	BAE25466.1	mmu:212986	
K05012	CLCN3_4_	mmu04613(	PF00654.25	GO:000802	cytoplasmic	NP_001403	mmu:12725	
K03695	clpB	mmu04213(	PF07724.20	GO:003085	mitochondr	NP_001404	mmu:20480	
K09458	fabF, OXSM	mmu01240(	PF00109.32	GO:000663	mitochondr	AAI18052.1	mmu:71147	
K16184	AKT1S1, Pl	mmu04140(	PF15798.10	GO:001920'	cytoplasmic	NP_001240	mmu:67605	
K06590	ITGB7	mmu04672(	PF00362.24	GO:009863	plasma mer	NP_038594	mmu:16421	
K00235	SDHB, SDH	mmu05208(	PF13085.11	GO:000916	cytoplasmic	ABD77133.	mmu:67680	
K08853	AAK				cytoplasmic	NP_001365	mmu:269774	
.3F5(Family with sequence similarit			PF16297.11	GO:004323	nuclear	NP_001018	mmu:66158	
K25092	SAFB		PF00076.28	GO:004280	nuclear	EDL38173.	mmu:224902	
K02901	RP-L27e, R	mmu05171(	PF01777.24	GO:004222	cytoplasmic	NP_000979	mmu:19942	
K14085	ALDH7A1	mmu00053(	PF00171.27	GO:000403	mitochondr	NP_613066	mmu:110695	
K03131	TAF6	mmu03022(	PF07571.19	GO:003124	nuclear	NP_666204	mmu:225895	
XSTA(CWC25 spliceosome-associat			PF12542.13	GO:007100	nuclear	NP_080462	mmu:67480	
K03372	SLC33A1, A	mmu00604(	PF13000.13	GO:001593	Golgi appa	NP_001258	mmu:11416	
K15507	MRM1, PET56		PF00588.24	GO:003197	mitochondr	NP_663408	mmu:217038	
K01001	ALG7	mmu00510(	PF00953.26	GO:000548	Golgi appa	NP_031901	mmu:13478	
K09433	ETV3		PF00178.28	GO:003250	nuclear	NP_001076	mmu:27049	ETS
.US8(collagen, type VIII, alpha 1)			PF00061.29	GO:000677	cytoplasmic	NP_035384	mmu:19659	
K24242	NT5C3	mmu01100(	PF05822.18	GO:000367	cytoplasmic	NP_001096	mmu:68106	
K17654	TRMT10C, MRPP1		PF01746.26	GO:007013	mitochondr	NP_083368	mmu:52575	
K19024	AP5S1		PF15001.12	GO:004323	cytoplasmic	NP_001277	mmu:69596	
K15272	SLC35A1_2_3		PF04142.21	GO:001593	ER	BAC30858.1	mmu:229782	
K17588	PREX2		PF00621.25	GO:003003	cytoplasmic	NP_083801	mmu:109294	
K12585	DIS3, RRP4	mmu03018(	PF00773.24	GO:001679	nuclear	NP_082591	mmu:72662	
K11543	MIS12		PF05859.17	GO:002260'	cytoplasmic	NP_080269	mmu:67139	
K06839	SLIT2	mmu04360(	PF13855.12	GO:004867	plasma mer	XP_006503	mmu:20563	
K06272	ILK	mmu05213(	PF07714.23	GO:001922	cytoplasmic	NP_001155	mmu:16202	
K17637	EXOC2, SE	mmu05132(	PF15469.12	GO:000810	cytoplasmic	NP_001347	mmu:66482	
K20281	GCC1		PF01465.25	GO:000579	cytoplasmic	NP_001414	mmu:74375	
K27390	HCFC2	mmu05168(	PF24681.1(I	GO:004508'	mitochondr	NP_001074	mmu:67933	
K14156	CHK	mmu05231(	PF01633.27	GO:000664	cytoplasmic	NP_031718	mmu:12651	
K00613	GATM	mmu00330(	Arginine an	GO:001003	mitochondr	NP_080237	mmu:67092	
eucine Rich Repeat)			PF13855.12	GO:000557	cytoplasmic	EDL28007.	mmu:66606	
K27397	PHF14		PF00628.35	GO:008009	nuclear	NP_001161	mmu:75725	
K08508	SNAP23	mmu04611(	PF00835.24	GO:005104	cytoplasmic	NP_001171	mmu:20619	
K01080	PLPP1_2_3	mmu05231(	PF01569.27	GO:001922	plasma mer	NP_542122	mmu:67916	
K22808	KANK		PF12796.13	GO:003304	cytoplasmic	AIC84023.1	mmu:80880	
K27761	GIMAP4		PF04548.22	GO:003563	cytoplasmic	NP_778155	mmu:107526	
K08055	B2M	mmu05170(	PF07654.21	GO:001097	ER	NP_033865	mmu:12010	

K08126	OGN		PF13855.12	GO:003250	extracellula	NP_032786	mmu:18295	
raB family)			PF01963.22(TraB_PrgY		cytoplasmic	NP_001365	mmu:67976	
K20915	TP53BP1, P	mmu04621(	PF09038.16	GO:000810	nuclear	NP_001277	mmu:27223	
K15180	COBRA1, N	mmu03250(	PF06209.18	GO:000989	cytoplasmic	NP_067368	mmu:58202	
K12898	HNRNPF_H		PF00076.28	GO:004348	nuclear	NP_001349	mmu:59013	
K02098	SPIR		PF16474.11	GO:003608	cytoplasmic	XP_006526	mmu:68166	
21VM(chromosome 19 open reading			PF17740.6(I	GO:004323	nuclear	NP_001361	mmu:66367	
K17766	TOM34		PF00515.34	GO:000810	cytoplasmic	NP_001278	mmu:67145	
K19748	GORAB, SC	mmu04115(	PF04949.18	GO:000858	cytoplasmic	NP_849214	mmu:98376	
K17768	TOM70	mmu04137(	PF00515.34	GO:000810	plasma mer	BAE36547.1	mmu:28185	
K13094	RBM5_10		PF01585.28	GO:008009	nuclear	NP_663602	mmu:236732	
Cold shock domain containing E1, R			PF05743.18	GO:000548	cytoplasmic	NP_001035	mmu:54122	
K24064	SNTB	mmu05410(	PF23012.2(S	GO:000585	mitochondr	NP_001398	mmu:20649	
K11376	ELP5, IKI1		PF10483.15	GO:000945	cytoplasmic	NP_061210	mmu:54351	
K04464	MAPK7	mmu04010(	PF00069.31	GO:200123	cytoplasmic	NP_001277	mmu:23939	
K26156	KIF18B	mmu04814(	PF00225.29	GO:009951	cytoplasmic	NP_932063	mmu:70218	
K27320	MSTO1, MST		PF10644.15	GO:004322	extracellula	NP_659147	mmu:229524	
K16734	SAC3D1, SHD1		PF03399.22	GO:002260	cytoplasmic	A6H687.1(F	mmu:66406	
21JM(Brain protein I3)			PF10164.14	GO:003109	extracellula	NP_001157	mmu:55950	
K15595	CDK17, PCTK2		PF00069.31	GO:003563	cytoplasmic	XP_036011	mmu:237459	
K09650	PARL, PSARL, PCP1		PF01694.27	GO:200123	mitochondr	NP_001005	mmu:381038	
K27274	GTF2IRD1		PF02946.19	GO:000097	nuclear	AAH83150.	mmu:57080	GTF2I
K07189	PPP1R3	mmu04931(	PF03370.18	GO:001679	nuclear	NP_536712	mmu:140491	
K03105	SRP19	mmu03060(	PF01922.23	GO:000578	cytoplasmic	BAB27165.1	mmu:66384	
K06269	PPP1C	mmu04750(	PF00149.33	GO:200123	cytoplasmic	NP_114074	mmu:19045	
K12367	DOCK2	mmu04062(	PF23554.2(	GO:005104	cytoplasmic	NP_203538	mmu:94176	
K03626	EGD2, NA	mmu04928(	PF01849.23	GO:004863	nuclear	NP_001106	mmu:17938	
K05411	IRF3	mmu05165(	PF10401.14	GO:008009	cytoplasmic	NP_058545	mmu:54131	IRF
K13886	CORO1B_1C_6		PF08953.17	GO:000810	cytoplasmic	BAE41150.1	mmu:23790	
K02187	CASP3	mmu05020(	PF00656.27	GO:003202	cytoplasmic	NP_001271	mmu:12367	
K13339	PEX6, PXA	mmu04146(	PF00004.35	GO:000810	peroxisoma	NP_663463	mmu:224824	
K03522	fixB, etfA		PF01012.27	GO:003444	mitochondr	NP_663590	mmu:110842	
K14406	CSTF1	mmu03015(	PF00400.38	GO:009030	cytoplasmic	XP_052040	mmu:67337	
K03954	NDUFA10	mmu05208(	PF01712.25	GO:001913	mitochondr	NP_077159	mmu:67273	
K10655	TRIM63, M	mmu04820(	PF13445.12	GO:009951	cytoplasmic	NP_001034	mmu:433766	
YJCN(epithelial cell proliferation)				GO:004322	cytoplasmic	NP_001366	mmu:80748	
K12183	TSG101, ST	mmu03250(	PF05743.18	GO:000810	cytoplasmic	NP_068684	mmu:22088	
K12874	AQR	mmu03040(	PF16399.11	GO:001688	nuclear	NP_001277	mmu:11834	
K24692	ZNF638		PF00076.28	GO:008009	nuclear	NP_001393	mmu:18139	
K10374	TPM2	mmu04260(	PF00261.25	GO:009951	cytoplasmic	NP_001288	mmu:22004	
K19942	GAS8, DRC4		PF13851.11	GO:000858	cytoplasmic	NP_061343	mmu:104346	
K22910	VIRMA		PF15912.10	GO:000815	cytoplasmic	NP_001333	mmu:66185	
K14618	MMACHC	mmu04980(	PF16690.10	GO:000923	cytoplasmic	NP_080238	mmu:67096	

K12794	CARD9	mmu04621(	PF00619.27	GO:008009	cytoplasmic	NP_001032	mmu:332579	
K06551	SIRPA_B1_	mmu04148(	PF07654.21	GO:000690	plasma mer	NP_001166	mmu:100038947	
K10290	FBXO3		PF04379.20	GO:000960	cytoplasmic	NP_997598	mmu:57443	
K23751	TRIOBP		PF00169.35	GO:004438	cytoplasmic	NP_001019	mmu:110253	
K26935	TMEM30, (	mmu04148(	PF03381.20	GO:199053	ER	NP_598479	mmu:69981	
21QW(Cystatin B (Stefin B))			PF00031.27	GO:000582	cytoplasmic	P35174.2(R	mmu:268885	
K10276	FBXL10_11	mmu03083(	PF16866.11	GO:003292	nuclear	NP_001001	mmu:225876	
K13210	FUBP		PF00013.35	GO:003577	nuclear	XP_034354	mmu:16549	
K20173	RILPL1		PF11461.13	GO:004299	cytoplasmic	NP_067405	mmu:75695	
K14820	BRX1, BRIX1		PF04427.23	GO:009030	nuclear	NP_080672	mmu:67832	
K02740	PSMB8, LM	mmu03050(	PF00227.31	GO:000417	cytoplasmic	AAA75035.	mmu:16913	
K14713	SLC39A7, K	mmu05010(	PF02535.28	GO:001972	extracellulo	NP_001071	mmu:14977	
K24609	LRIG2	mmu04360(	PF13855.12	GO:005104	plasma mer	EDL07570.	mmu:269473	
K02186	CASP2	mmu04210(	PF00656.27	GO:200123	mitochondr	NP_031636	mmu:12366	
K26273	SBNO, SNO, FGT1		PF13872.12	GO:004239	nuclear	NP_001074	mmu:243272	
K12184	VPS28	mmu04144(	PF03997.17	GO:000810	cytoplasmic	NP_080118	mmu:66914	
K06084	LMO7, FB>	mmu04520(	PF15949.10	GO:009859	nuclear	XP_006519	mmu:380928	
.YQN(-)			PF15054.11	GO:008009	cytoplasmic	XP_021006	mmu:136856708	
K26501	OSTC	mmu04141(	PF04756.19	GO:199023	ER	NP_001102	mmu:66357	
K23918	NEXN		PF07679.22	GO:000699	cytoplasmic	AEM53921.	mmu:68810	
.YCK(Family with sequence similar			PF06388.16	GO:003304	mitochondr	NP_081618	mmu:70186	
K01513	ENPP1_3, (	mmu01100(	PF01663.27	GO:004643	plasma mer	NP_598766	mmu:209558	
K10771	APEX1	mmu03410(	PF03372.29	GO:000578	cytoplasmic	NP_033817	mmu:11792	
K09171	NFIX		PF00859.23	GO:000690	nuclear	NP_001357	mmu:18032	MH1
K14285	NXT1_2, P	mmu03008(	PF02136.26	GO:001593	cytoplasmic	NP_001154	mmu:237082	
K11111	TERF2, TRF2		PF08558.16	GO:000810	cytoplasmic	NP_033379	mmu:21750	MYB
K11128	GAR1, NO	mmu03008(	PF04410.20	GO:007258	nuclear	NP_080854	mmu:68147	
)GZM(GC-rich promoter binding p			PF15337.11	GO:008009	nuclear	NP_001355	mmu:77110	TF_others
K13024	PPIP5K, VI	mmu04070(	PF00328.27(His_Phos_2		nuclear	NP_001392	mmu:227399	
K16728	CEP152, ASL			GO:000024	cytoplasmic	BAD32338.	mmu:99100	
.RN8(DiGeorge syndrome critical r			PF07324.16(DGCR6:Di		cytoplasmic	EDL77918.	mmu:13353	
K10371	TNNI1	mmu04814(	PF00992.25	GO:005501	cytoplasmic	XP_034369	mmu:21952	
K12560	FCGR2B, C	mmu04666(	PF13895.12	GO:004340	plasma mer	NP_034317	mmu:14130	
K09428	ELF1_2_4		PF00178.28	GO:008009	nuclear	NP_001277	mmu:69257	ETS
K10839	RAD23, HR	mmu04141(	PF00627.37	GO:003243	cytoplasmic	NP_001284	mmu:19358	
K11169	DHRS13		PF00106.31	GO:000557	extracellulo	NP_899109	mmu:70451	
K20823	NAA35, MAK10		PF04112.18	GO:003124	cytoplasmic	NP_001371	mmu:78689	
K23691	PKN2	mmu04621(	PF00069.31	GO:004521	cytoplasmic	NP_848769	mmu:109333	
Hydrolase of the alpha beta-hydrola			PF20408.4(Abhydrolase		cytoplasmic	NP_001343	mmu:75623	
.MJZ(caspase activity and apoptosis			PF15335.11	GO:000691	cytoplasmic	BAE35881.1	mmu:67770	
K03341	SEPSECS	mmu00970(	PF05889.19	GO:000645	cytoplasmic	NP_766078	mmu:211006	
K01634	SGPL1, DP	mmu04071(	PF00282.25	GO:000578	Golgi appa	BAE29269.1	mmu:20397	
K13356	FAR	mmu04146(	PF07993.18	GO:000663	peroxisoma	NP_001347	mmu:67420	

K11984	SART1, HA	mmu03040(	PF03343.18	GO:190303	nuclear	NP_058578	mmu:20227	
K10421	CLIP1, RSN	mmu04150(	PF01302.31	GO:003111	nuclear	NP_062739	mmu:56430	
K03364	CDH1, FZF	mmu04120(	PF24807.1(	GO:003243	nuclear	CAH67792	mmu:56371	
K14556	DIP2, UTP	mmu03008(	PF00400.38	GO:003204	nuclear	NP_780761	mmu:269470	
K04683	TFDP1	mmu03083(	PF08781.15	GO:000097	cytoplasmic	NP_001278	mmu:21781	E2F
K08057	CALR	mmu05170(	PF00262.25	GO:005116	ER	BAE22866.	mmu:12317	
K03259	EIF4E	mmu04910(	PF01652.23	GO:005116	cytoplasmic	NP_031943	mmu:13684	
QB6(NOP16 nucleolar protein hon			PF09420.15	GO:004227	nuclear	NP_848720	mmu:28126	
K04699	SOCS6_7	mmu04917(	Prolactin si	GO:000268	cytoplasmic	EDL16880.	mmu:54607	
K13169	AKAP17, SFRS17		PF25015.1(	RBD_AKAF	nuclear	NP_001405	mmu:118568424	
K09880	mtnC, ENC	mmu00270(	PF00702.32	GO:000548	cytoplasmic	NP_001156	mmu:67870	
K06709	KDM4, JMJD2, JHDM		PF02373.28	GO:001922	cytoplasmic	EDL38165.	mmu:193796	
S7F(LIM domain containing 2)			PF00412.28	GO:002260	cytoplasmic	NP_001343	mmu:67803	
K21843	TTC7		PF19440.5(	GO:003250	cytoplasmic	NP_082915	mmu:225049	
K11276	NPM1		PF03066.21	GO:005116	cytoplasmic	NP_032748	mmu:18148	
K14634	TIGAR	mmu05230(	PF00300.27	GO:003202	cytoplasmic	NP_795977	mmu:319801	
K12600	SKI3, TTC	mmu03018(	PF00515.34	GO:000018	nuclear	NP_001074	mmu:218343	
K14845	RAI1, DOM3Z		PF08652.17	GO:008009	cytoplasmic	NP_001157	mmu:112403	
K03941	NDUFS8	mmu05208(	PF00037.32	GO:000916	mitochondr	BAE22408.	mmu:225887	
K13194	ADARB		PF02137.24	GO:005134	nuclear	XP_006513	mmu:110532	
K06184	ABCF1		PF00005.33	GO:001688	cytoplasmic	AAH46965.	mmu:224742	
K00802	SMS	mmu00270(	PF01564.23	GO:000930	cytoplasmic	NP_033240	mmu:20603	
ZVJE(Glutathione S-transferase, C-t			PF13679.12	GO:000573	cytoplasmic	EDL12187.	mmu:67553	
K05755	ARPC4	mmu04530(	PF05856.17	GO:002260	cytoplasmic	NP_001069	mmu:68089	
K08023	LTBP2_3_4		PF07645.21	GO:000810	extracellul	NP_032546	mmu:16998	
K16761	CEP44		PF15007.11	GO:002260	cytoplasmic	EDL28605.	mmu:382010	
K20645	ARHGAP30		PF00620.32	GO:004322	cytoplasmic	SPT35701.1	mmu:226652	
K15213	TAF1B		PF20645.2(	GO:002260	nuclear	NP_065639	mmu:21340	
K05605	HIBCH	mmu00410(	PF16113.10	GO:001679	mitochondr	NP_666220	mmu:227095	
K09594	FLCN, BHI	mmu05211(	PF16692.10	GO:004340	cytoplasmic	NP_001258	mmu:216805	
K07928	RAB40		PF00071.28	GO:000802	cytoplasmic	NP_631893	mmu:224624	
K03163	TOP1		PF01028.25	GO:003292	nuclear	NP_033434	mmu:21969	
K01796	E5.1.99.4, A	mmu00120(	PF02515.22	GO:000663	peroxisoma	NP_032563	mmu:17117	
K01285	PRCP	mmu04614(	PF05577.18	GO:000032	lysosomal	NP_082519	mmu:72461	
K16469	CROCC		PF15035.11	GO:000810	extracellul	NP_742120	mmu:230872	
K11205	GCLM	mmu01240(	PF00248.27	GO:200123	mitochondr	NP_032155	mmu:14630	
K15192	BTAF1, MOT1		PF12054.13	GO:001707	nuclear	BAF41982.1	mmu:107182	
K20634	ARHGAP9		PF00620.32	GO:000548	cytoplasmic	NP_001272	mmu:216445	
K11430	EZH2	mmu03083(	PF21358.2(	GO:000810	nuclear	BAD90359.	mmu:14056	
K10598	PPIL2, CY(	mmu04120(	PF00160.26	GO:000810	cytoplasmic	EDK97443.	mmu:66053	
K20307	TRAPPC10, TRS130		PF23036.1(	GO:199007	cytoplasmic	NP_001074	mmu:216131	
K14842	NSA2		PF01201.27	GO:003068	nuclear	NP_067527	mmu:59050	
K26095	SKA2		PF16740.10	GO:003111	cytoplasmic	NP_079653	mmu:66140	

K00586	DPH5		PF00590.25	GO:004423	cytoplasmic	NP_081469	mmu:69740	
K15258	PARP6_8		PF00644.27	GO:000563	mitochondr	ABV44383.	mmu:52552	
K23080	CASTOR1	mmu04150(	PF13840.12	GO:004222	extracellulo	NP_082298	mmu:71962	
K03108	SRP72	mmu03060(	PF08492.17	GO:000578	nuclear	NP_079967	mmu:66661	
K10142	STEAP3, T	mmu04115(	PF03807.23	GO:200123	cytoplasmic	XP_006529	mmu:68428	
K11099	SNRPG, SM	mmu03040(	PF01423.28	GO:000548	nuclear	NP_001035	mmu:68011	
K01915	glnA, GLUl	mmu04724(	PF00120.29	GO:009008	cytoplasmic	NP_032157	mmu:14645	
K11098	SNRPF, SM	mmu03040(	PF01423.28	GO:000548	nuclear	NP_001005	mmu:69878	
54J(major urinary protein)			PF00061.29	GO:009008	extracellulo	P04939.1(R	mmu:17842	
K02953	RP-S13e, R	mmu05171(	PF08069.17	GO:003311	mitochondr	NP_001001	mmu:68052	
K12349	ASAH2	mmu04071(	PF04734.18	GO:200123	ER	AAI45017.1	mmu:54447	
K20778	RAD51AP1		PF15696.10	GO:008009	nuclear	NP_033039	mmu:19362	
K14701	SLC26A2, D	mmu05208(	PF00916.26	GO:009859	cytoplasmic	NP_031911	mmu:13521	
K24144	NHS		PF15273.11	GO:003250	cytoplasmic	NP_001157	mmu:100042480	
K01872	AARS, alaS	mmu00970(	PF01411.24	GO:008009	cytoplasmic	BAE34378.	mmu:234734	
K07375	TUBB	mmu05020(	PF00091.31	GO:009951	cytoplasmic	XP_028912	mmu:73710	
K15277	SLC35B3, PAPST2		PF08449.17	GO:000534	peroxisoma	XP_021036	mmu:108652	
K20873	NEK3		PF00069.31	GO:008009	cytoplasmic	NP_035978	mmu:23954	
K23687	RIN2_3		PF23268.1(I	GO:200121	cytoplasmic	NP_083000	mmu:74030	
K18660	ACSF3	mmu00280(	PF00501.34	GO:000663	mitochondr	NP_659181	mmu:257633	
K08489	STX16	mmu04130(	PF05739.25	GO:000810	cytoplasmic	AAH94436.	mmu:228960	
Retrotransposon protein)			PF06817.19	GO:004671	cytoplasmic	AAH94435.	mmu:118568345	
K05060	IL6ST, GP1	mmu05167(	PF06328.17	GO:001922	plasma mer	AEZ35214.	mmu:16195	
K07521	UNC5	mmu04360(	PF17217.9(I	GO:200123	cytoplasmic	BAE28019.	mmu:107449	
K23970	FRMD4		PF11819.13	GO:000592	cytoplasmic	NP_001334	mmu:209630	
Glioma tumor suppressor candidate			PF00415.24	GO:000810	cytoplasmic	NP_776292	mmu:108911	
K06558	MSR1, CD2	mmu04145(	PF00530.23	GO:000810	plasma mer	P30204.3(R	mmu:20288	
RK6(glia maturation factor)			PF00241.25	GO:005112	cytoplasmic	NP_001034	mmu:63986	
K05872	NFKBIE	mmu05235(	PF12796.13	GO:190165	cytoplasmic	NP_001291	mmu:18037	
K09851	RASSF2_4	mmu04392(	PF00788.29	GO:000998	cytoplasmic	NP_835146	mmu:213391	
K19500	ANO6, TM	mmu04148(	PF04547.17	GO:000759	cytoplasmic	NP_001240	mmu:105722	
K02602	CPEB, ORI	mmu04114(	PF16367.11	GO:000989	cytoplasmic	KAI456118	mmu:67579	
K06671	STAG1_2, S	mmu04110(	PF24571.1(I	GO:004322	cytoplasmic	XP_021027	mmu:20842	
K16510	RPS6KA4, I	mmu04010(	PF00069.31	GO:001922	cytoplasmic	NP_064308	mmu:56613	
K10990	RMI1, BRA	mmu03460(	PF16099.11	GO:003320	cytoplasmic	NP_001161	mmu:74386	
K09428	ELF1_2_4		PF00178.28	GO:000097	nuclear	NP_001273	mmu:13709	ETS
K03434	PIGL	mmu00563(	PF02585.22	GO:000665	ER	NP_001034	mmu:327942	
K01413	MMP12		PF00413.30	GO:005116	ER	NP_032631	mmu:17381	
K07048	PTER, php		PF02126.24	GO:003250	cytoplasmic	NP_032987	mmu:19212	
K02209	MCM5, CD	mmu03030(	PF00493.29	GO:003126	nuclear	NP_001289	mmu:17218	
K17929	SNX17		PF18116.7(S	GO:000810	cytoplasmic	NP_710147	mmu:266781	
K08880	STK19		PF10494.14	GO:000016	cytoplasmic	NP_062315	mmu:54402	
K02212	MCM4, CD	mmu03030(	PF00493.29	GO:190231	nuclear	NP_032591	mmu:17217	

K07953	SAR1	mmu04141(	PF00025.27	GO:003315	cytoplasmic	NP_001007	mmu:20224	
K06106	CTTN, EM	mmu04530(	PF02218.20	GO:003315	nuclear	NP_032251	mmu:15163	
K27864	BANP		PF10523.15	GO:003450	nuclear	NP_058092	mmu:53325	
K23569	EMC8_9		PF03665.18	GO:000810	cytoplasmic	NP_035056	mmu:18117	
K11856	USP43		PF00443.35	GO:004423	cytoplasmic	NP_001277	mmu:216835	
K02576	NDST1	mmu00534(	PF12062.14	GO:000858	Golgi appa	NP_001335	mmu:15531	
K22530	ATAD1, MSP1		PF00004.35	GO:000810	ER	NP_001244	mmu:67979	
K03494	ST3GAL4	mmu00601(	PF00777.23	GO:004858	Golgi appa	NP_033204	mmu:20443	
K14835	NOP2		PF01189.23	GO:004212	nuclear	NP_620086	mmu:110109	
K10866	RAD50	mmu03440(	PF13476.12	GO:001922	nuclear	NP_033038	mmu:19360	
K20222	IPO5, KPN	mmu03013(	PF18829.7(I	GO:003315	cytoplasmic	NP_001403	mmu:70572	
K23923	GCA		PF00036.39	GO:000573	cytoplasmic	NP_663498	mmu:227960	
K22564	COMMD8		PF22838.1((	GO:000565	cytoplasmic	NP_848714	mmu:27784	
K13780	SLC7A5, LA	mmu05230(	PF13520.12	GO:001922	plasma mer	BAE25012.1	mmu:20539	
K06474	CD34	mmu04514(	PF06365.17	GO:001922	extracellula	BAE39431.1	mmu:12490	
K10768	ALKBH6		PF13532.12	GO:003005	cytoplasmic	NP_932144	mmu:233065	
K01831	PTGIS, CY	mmu00590(	PF00067.27	GO:000449	extracellula	NP_032994	mmu:19223	
K25730	VPS26C		PF03643.20	GO:000810	cytoplasmic	NP_031860	mmu:13185	
K17635	RGL1, RGI	mmu04014(	PF00617.24	GO:001602	cytoplasmic	NP_001333	mmu:19731	
K10727	CDT1	mmu04110(	PF08839.17	GO:190304	nuclear	NP_080290	mmu:67177	
K03251	EIF3D		PF05091.18	GO:000374	cytoplasmic	NP_001004	mmu:55944	
Cerebellar degeneration-related prot			PF00096.32	GO:190227	nuclear	NP_001333	mmu:10926	zf-C2H2
K25004	MTHFR	mmu01523(	PF21895.1(I	GO:000652	cytoplasmic	NP_001155	mmu:17769	
K24832	IQCJ		PF10148.15	GO:004870	cytoplasmic	NP_001106	mmu:100505386	
K24686	ZFAND3		PF01428.22	GO:004316	nuclear	NP_001343	mmu:21769	
K25100	SPEN, SHA	mmu04330(	PF20809.2(I	GO:008009	nuclear	NP_062737	mmu:56381	
K23951	DYM		PF09742.14	GO:000573	cytoplasmic	NP_082003	mmu:69190	
K13373	HSD17B7	mmu00100(	PF00106.31	GO:000512	extracellula	NP_034606	mmu:15490	
K00809	DHPS, dys		PF01916.23	GO:000548	cytoplasmic	NP_001034	mmu:330817	
K07602	DSC3		PF00028.23	GO:002260	extracellula	NP_031908	mmu:13507	
K18402	PHF20		PF16660.10	GO:003124	nuclear	XP_006520	mmu:239510	
K23456	CFAP410, C21ORF2		PF12799.13	GO:002260	mitochondr	BAE31826.1	mmu:67884	
K26125	MMS22L		PF14910.11	GO:000802	cytoplasmic	NP_955761	mmu:212377	
K05164	IL17RA, CI	mmu04936(	PF16556.11	GO:001922	plasma mer	NP_032385	mmu:16172	
KQAH(MIT, microtubule interactin			PF16565.11	GO:001602	cytoplasmic	NP_001343	mmu:69028	
K07609	NES		PF00038.26	GO:000512	cytoplasmic	NP_057910	mmu:18008	
K21358	ULK3		PF00069.31	GO:000858	cytoplasmic	NP_082171	mmu:71742	
K14553	UTP18	mmu03008(	PF23414.1(I	GO:003204	nuclear	NP_001013	mmu:217109	
K03014	RPABC2, R	mmu03420(	Nucleotide	GO:000367	nuclear	XP_037702	mmu:69833	
K04426	MAP3K5, A	mmu05208(	PF13281.12	GO:001922	cytoplasmic	NP_032606	mmu:26408	
K22803	SMC5		PF02463.25	GO:003506	cytoplasmic	NP_001239	mmu:226026	
K07955	ARL8	mmu05132(	PF00025.27	GO:000810	cytoplasmic	BAB24358.1	mmu:67166	
K06674	SMC2		PF02463.25	GO:190136	nuclear	NP_001288	mmu:14211	

K25509	CRYBG		PF00030.24	GO:003250	cytoplasmic	NP_777273	mmu:224273	
K05738	MYL1	mmu04814(	PF13405.12	GO:009951	cytoplasmic	XP_011236	mmu:17901	
K06689	UBE2D, UI	mmu04141(	PF00179.31	GO:008009	cytoplasmic	XP_010846	mmu:66105	
K00940	ndk, NME	mmu01240(	PF00334.24	GO:000921	peroxisoma	EDL09034.	mmu:54369	
K21767	TBCD		PF12612.13	GO:000592	cytoplasmic	EDL34842.	mmu:108903	
K07964	HPSE	mmu00531(	PF03662.20	GO:000810	ER	EDL20282.	mmu:15442	
K13137	STRAP, UNRIP		PF00400.38	GO:008009	cytoplasmic	NP_035629	mmu:20901	
K17392	IGF2BP2		PF00013.35	GO:199024	cytoplasmic	EDK97620.	mmu:319765	
K04361	EGFR, ERI	mmu05208(	PF07714.23	GO:006008	plasma mer	NP_997538	mmu:13649	
K18647	ANP32B		PF14580.12	GO:003315	cytoplasmic	BAE23087.	mmu:67628	
K02932	RP-L5e, RP	mmu05171(	PF17144.9(I	GO:001710	cytoplasmic	XP_006534	mmu:100503670	
K07831	MRAS	mmu04137(	PF00071.28(Ras:Ras far		cytoplasmic	XP_023591	mmu:17532	
K19721	COL5AS	mmu04974(	PF01391.23	GO:000520	extracellulo	AAH43696.	mmu:12832	
K16860	PLD3_4	mmu00564(	PF13918.12	GO:000695	ER	NP_849242	mmu:104759	
K05744	LIMK2	mmu04810(	PF07714.23	GO:001922	cytoplasmic	NP_034848	mmu:16886	
K08803	DAPK	mmu04140(	PF00069.31	GO:200123	cytoplasmic	NP_001177	mmu:13144	
K02310	DFFA, DFI	mmu04210(	PF09033.15	GO:006107	nuclear	NP_001020	mmu:13347	
K24270	TES		PF06297.19	GO:000367	cytoplasmic	EDL13893.	mmu:21753	
K12312	MAN2B2	mmu00511(	PF01074.27	GO:000032	extracellulo	NP_032576	mmu:17160	
K22461	P3H4		PF23557.1(	GO:000367	mitochondr	BAC26365.	mmu:66180	
K03084	SHIP1, INP	mmu04664(	PF22669.2(I	GO:009951	cytoplasmic	NP_034696	mmu:16331	
K04487	iscS, NFS1	mmu01240(	PF00266.25	GO:003017	mitochondr	AAH10586.	mmu:18041	
K24020	PLEKHH		PF00784.22	GO:004299	cytoplasmic	NP_001344	mmu:213556	
K11374	ELP2		PF00400.38	GO:000802	mitochondr	NP_067423	mmu:58523	
K04660	DCN	mmu04820(	PF13855.12	GO:190418	extracellulo	NP_001177	mmu:13179	
K11279	NAP1L1, NRP		PF00956.23	GO:003472	nuclear	NP_056596	mmu:53605	
K01939	purA, ADS	mmu01240(	PF00709.27	GO:000401	cytoplasmic	NP_031447	mmu:11565	
K08118	BGN	mmu04820(	PF13855.12	GO:005084	extracellulo	NP_031568	mmu:12111	
K10746	EXO1	mmu03430(	PF00867.23	GO:001710	nuclear	NP_036142	mmu:26909	
K19323	ATXN10	mmu05017(	PF09759.15	GO:002260	cytoplasmic	NP_058539	mmu:54138	
K24318	SCARF1	mmu04148(	PF00053.31	GO:005113	extracellulo	AAH80288.	mmu:380713	
K12486	SMAP	mmu04144(	PF01412.23	GO:200036	cytoplasmic	NP_082810	mmu:98366	
K08753	FABP4, aP	mmu03320(	PF00061.29	GO:008009	cytoplasmic	NP_077717	mmu:11770	
:ardiotrophin 1)			PF00096.32	GO:004356	cytoplasmic	BAC97922.	mmu:23390	zf-C2H2
K23497	TRUB2		PF01509.23	GO:007013	mitochondr	NP_663495	mmu:227682	
K17917	SNX1_2	mmu04144(	PF09325.15	GO:004885	cytoplasmic	AAH06960.	mmu:67804	
ZDGT(Protein kinase (CAMP-deper			PF02827.21	GO:003315	cytoplasmic	XP_047626	mmu:18769	
K20130	RABEP2		PF03528.20	GO:004299	cytoplasmic	NP_085043	mmu:70314	
XPV4(Family with sequence similarity 91, meml				GO:000557	cytoplasmic	XP_029326	mmu:228869	
K02154	ATPeV0A,	mmu05165(	PF01496.24	GO:009877	extracellulo	NP_035726	mmu:21871	
K12864	CTNNBL1	mmu03040(	PF08216.17	GO:000237	cytoplasmic	BAE27033.	mmu:66642	
K26253	FBRS	mmu03083(	PF15336.11(Auts2:Autis		cytoplasmic	NP_034313	mmu:14123	
K00710	GALNT	mmu00514(	PF00535.33	GO:003024	Golgi appa	NP_056552	mmu:14426	

K09250	CNBP		PF00098.28	GO:000828	nuclear	NP_001342	mmu:12785	
K01322	PREP	mmu04614(	PF02897.21	GO:000425	cytoplasmic	NP_035286	mmu:19072	
K09115	MNT, ROX		PF00010.31	GO:200123	nuclear	XP_021032	mmu:17428	bHLH
K15410	EEF1D		PF00736.24	GO:004518	nuclear	NP_001272	mmu:66656	
5I2(IGv); ENOG4111CF7(IGv); EN			PF07686.23(V-set:Immu		cytoplasmic	CAA41828.1(IgK light chain V regi		
K08080	CMAH	mmu00520(	PF13483.12	GO:000449	cytoplasmic	XP_011242	mmu:12763	
K17072	IFI47	mmu04668(	PF05049.19	GO:000578	cytoplasmic	BAE33853.	mmu:15953	
K09403	FOXJ2_3		PF00250.24	GO:000097	nuclear	NP_001344	mmu:23070	Fork head
K11429	SUV420H	mmu00310(	PF00856.33	GO:008009	nuclear	NP_001108	mmu:232811	
Z9V(chromosome 5 open reading fr			PF17716.6(I	GO:000557	cytoplasmic	NP_666042	mmu:106064	
K18633	MZT1, GIP1, GIP2		PF12554.13	GO:002260	cytoplasmic	NP_780454	mmu:76789	
K10400	KIF15	mmu04814(	PF00225.29	GO:009951	cytoplasmic	NP_034750	mmu:209737	
K14311	NUP188	mmu03013(	PF21093.3(	GO:003450	mitochondr	EDL08456.	mmu:227699	
YDRZ(family with sequence similar			PF13289.12	GO:001660	cytoplasmic	NP_001273	mmu:109229	
Y3UN(IAO AO transport system AT			PF07156.20	GO:003032	extracellulo	NP_766420	mmu:240334	
K16539	PLK1S1			GO:001989	cytoplasmic	NP_001028	mmu:228730	
XR8P(LMBR1 domain containing 2)			PF04791.21	GO:001602	Golgi appa	NP_001405	mmu:320506	
Rhodanese domain protein)			PF00581.25	GO:001678	mitochondr	NP_084116	mmu:77032	
K21123	SRSF9, SFR	mmu03040(	PF00076.28	GO:003311	cytoplasmic	NP_079849	mmu:108014	
YT3F(Transmembrane protein 119)			PF15724.10	GO:001922	ER	NP_001412	mmu:231633	
J5W(Transcription factor 19)			PF00498.31	GO:001922	nuclear	NP_001157	mmu:106795	
K13132	GEMIN4			GO:002260	nuclear	BAE24559.	mmu:276919	
K07837	RAP2A		PF00071.28	GO:000810	cytoplasmic	NP_001179	mmu:76108	
K23793	PHLDA		PF00169.35	GO:001602	cytoplasmic	NP_038778	mmu:27280	
K19875	MYOT	mmu04820(	PF07679.22	GO:009863	cytoplasmic	NP_001028	mmu:58916	
K25753	SELENOP		PF04592.20	GO:001995	extracellulo	NP_001036	mmu:20363	
K12891	SRSF2_8, SI	mmu03040(	PF00076.28	GO:003506	nuclear	NP_001009	mmu:20382	
K10297	FBXO11		PF13229.12	GO:008009	cytoplasmic	NP_001074	mmu:225055	
K01446	PGRP		PF01510.30	GO:000959	plasma mer	ABM92420.	mmu:57757	
K01648	ACLY	mmu00020(	PF24948.1((	GO:000663	cytoplasmic	NP_001186	mmu:104112	
K16795	PAFAH1B2	mmu00565(	PF13472.12	GO:000384	cytoplasmic	NP_001344	mmu:18476	
K27568	BLZF1		PF08227.16	GO:003303	nuclear	BAE42289.	mmu:66352	
K18621	ANLN		PF00169.35(PH:PH don		cytoplasmic	XP_011240	mmu:68743	
K20917	GSDMD	mmu04621(	PF04598.17	GO:000810	cytoplasmic	NP_081236	mmu:69146	
K02214	CDC7	mmu04110(	PF00069.31	GO:008009	cytoplasmic	NP_001258	mmu:12545	
K23767	S100A14			GO:004858	cytoplasmic	NP_001156	mmu:66166	
Y78B(ddb1 and cul4 associated facto			PF02760.21	GO:003210	nuclear	ASD34085.	mmu:226691	
K04001	SERPING1	mmu04610(	PF00079.25	GO:000759	extracellulo	NP_001406	mmu:12258	
K08756	FABP7	mmu03320(	PF00061.29	GO:003210	cytoplasmic	EDL92888.	mmu:12140	
K02176	SFRP2	mmu04310(	PF01392.28	GO:200123	extracellulo	NP_033170	mmu:20319	
K19614	SERPINF1,	mmu04310(	PF00079.25	GO:003202	extracellulo	NP_035470	mmu:20317	
K09576	FKBP11		PF00254.34	GO:004323	ER	NP_077131	mmu:66120	
K15208	SNAPC1		PF09808.14	GO:003277	cytoplasmic	NP_001405	mmu:75627	

K12171	SH3RF, POSH		PF00018.34	GO:200123	cytoplasmic	NP_067481	mmu:59009	
K20409	WDR59, SE	mmu04150(	PF24807.1(	GO:000810	cytoplasmic	NP_795897	mmu:319481	
K08860	EIF2AK3, I	mmu04137(	PF00069.31	GO:003288	extracellula	NP_034251	mmu:13666	
K13619	DDHD1		PF02862.22	GO:000665	cytoplasmic	NP_789815	mmu:114874	
K13125	NOSIP		PF15906.11	GO:001922	cytoplasmic	NP_079809	mmu:66394	
K07829	RRAS	mmu04137(	PF00071.28	GO:004858	cytoplasmic	NP_033127	mmu:20130	
K06271	TLN	mmu04015(	PF21896.2(	GO:009859	cytoplasmic	XP_006537	mmu:21894	
K17414	MRPS36		PF10937.13	GO:001975	mitochondr	NP_001177	mmu:66128	
K01304	pcp		PF01470.22	GO:004423	plasma mer	NP_075706	mmu:66522	
K10859	ALKBH2		PF13532.12	GO:005121	nuclear	NP_001346	mmu:231642	
Beta-lactamase domain protein)			PF16123.11	GO:001679	cytoplasmic	NP_001258	mmu:68977	
K15441	TAD2, ADAT2		PF14437.12	GO:004316	cytoplasmic	NP_080024	mmu:66757	
K25389	TXNDC15		PF00085.26	GO:009859	ER	NP_780359	mmu:69672	
K03038	PSMD7, RF	mmu05020(	PF13012.11	GO:000367	ER	NP_034947	mmu:17463	
K06062	PCAF, KA7	mmu04330(	PF06466.16	GO:001989	cytoplasmic	NP_064389	mmu:18519	
K24526	RBM12		PF00076.28	GO:000548	cytoplasmic	BAC28911.1	mmu:75710	
K11835	USP4_11, UBP12		PF00443.35	GO:000810	cytoplasmic	NP_001298	mmu:22258	
K27687	NOL7		PF08157.16	GO:003204	nuclear	NP_076043	mmu:70078	
SFT2 domain containing)			PF04178.17	GO:000810	cytoplasmic	NP_080282	mmu:67158	
K16830	AURKAIP1		PF08213.16	GO:199023	mitochondr	EDL15041.	mmu:66077	
K13822	HBA	mmu05144(	PF00042.28	GO:000534	cytoplasmic	BAB27381.1	mmu:15122	
K11131	DKC1, NO	mmu03008(	PF08068.17	GO:008009	nuclear	BAE39967.1	mmu:245474	
K01689	ENO1_2_3	mmu04820(	PF00113.28	GO:000918	cytoplasmic	NP_001342	mmu:13807	
K01689	ENO1_2_3	mmu04820(	PF00113.28	GO:200123	cytoplasmic	NP_001020	mmu:433182	
K15172	SUPT5H, Sl	mmu03250(	PF00467.35	GO:008009	nuclear	NP_038704	mmu:20924	
K03137	TFIIE2, GT	mmu05203(	PF02186.21	GO:000635	nuclear	NP_001161	mmu:68153	
K22904	PLPP6		PF01569.27	GO:004239	cytoplasmic	NP_083198	mmu:74411	
K08956	AFG3	mmu05017(	PF01434.24	GO:006000	cytoplasmic	BAE29204.1	mmu:69597	
K09431	ETV1	mmu05202(	PF04621.18	GO:008009	nuclear	XP_036013	mmu:14009	ETS
K13184	DHX9		PF00035.32	GO:000962	cytoplasmic	EDL39437.	mmu:13211	
K04945	KCNN4, K	mmu04970(	PF02888.21	GO:004340	ER	EDL24340.	mmu:16534	
K13145	INTS8			GO:001618	cytoplasmic	NP_001153	mmu:72656	
K22410	PRDM16	mmu04714(	PF00096.32(zf-C2H2:Zi		cytoplasmic	NP_001357	mmu:70673	zf-C2H2
XR8F(TLD domain-containing prot			PF07534.21	GO:006034	cytoplasmic	NP_083159	mmu:74347	
K25724	PTX3		PF00354.24	GO:008009	plasma mer	NP_033013	mmu:19288	
K04986	PKD2		PF08016.18	GO:009859	cytoplasmic	NP_032887	mmu:18764	
K18035	PTPRO		PF00102.32	GO:003250	extracellula	NP_001157	mmu:19277	
K10605	BRCA1	mmu03440(	PF12820.12	GO:004423	cytoplasmic	NP_033894	mmu:12189	
K04502	FOSL1	mmu04310(	PF00170.26	GO:001922	nuclear	NP_034365	mmu:14283	TF_bZIP
K23543	CCDC115		PF21730.2(	GO:002260	cytoplasmic	NP_081435	mmu:69668	
K06554	IGLL1, IGI	mmu05340(	PF07654.21	GO:000548	plasma mer	AAH31470.	mmu:16136	
K20704	ECT2, ARHGEF31		PF00621.25	GO:003315	nuclear	SPT35752.1	mmu:13605	
K20160	DENND1		PF02141.26	GO:000810	cytoplasmic	NP_001159	mmu:329260	

K14855	RSA4, NLE1		PF00400.38	GO:004864	cytoplasmic	AAH18399.	mmu:217011	
K00027	ME2, sfcA,	mmu01200(	PF03949.21	GO:001922	mitochondr	NP_663469	mmu:107029	
K23739	SH3BP5		PF05276.19	GO:001920	cytoplasmic	BAE39330.1	mmu:79566	
K17257	ITFG1		PF23122.1(	GO:000557	extracellulo	NP_082283	mmu:71927	
K19027	ZFYVE26		PF01363.27	GO:001989	cytoplasmic	NP_001008	mmu:211978	
K07376	PRKG1	mmu04970(	PF00069.31	GO:001922	cytoplasmic	NP_001013	mmu:19091	
K17642	DDX3Y		PF00270.35	GO:003577	cytoplasmic	BAE32549.1	mmu:26900	
K19995	SCAMP		PF04144.18	GO:000810	cytoplasmic	NP_001365	mmu:24044	
K27697	DMTN		PF16182.10	GO:003288	cytoplasmic	BAE28799.1	mmu:13829	
K11282	NAP1L4, NAP2		PF00956.23	GO:003472	nuclear	NP_032698	mmu:17955	
K10707	VAPB, ALS	mmu04979(	PF00635.32	GO:000578	cytoplasmic	NP_062780	mmu:56491	
K23880	RETREG		PF24456.1(I	GO:000367	ER	NP_001030	mmu:66270	
K01640	HMGCL, h	mmu00650(	PF00682.25	GO:003197	peroxisoma	NP_032280	mmu:15356	
K07375	TUBB	mmu05020(	PF00091.31	GO:009951	cytoplasmic	XP_037004	mmu:22151	
K10634	PJA2		PF13639.12	GO:000761	cytoplasmic	NP_001020	mmu:224938	
K20225	CIC	mmu05017(	PF16090.11	GO:004885	nuclear	NP_001289	mmu:71722	HMG
K14574	SDO1, SBD	mmu03008(	PF01172.24	GO:003028	cytoplasmic	NP_075737	mmu:66711	
K10356	MYO1	mmu04814(	PF00063.27	GO:009951	cytoplasmic	NP_001277	mmu:17912	
K24879	ZHX1		PF00046.35	GO:000989	nuclear	BAD90299.	mmu:22770	Homeobox
K09404	FOXK	mmu03083(	PF00250.24	GO:008009	cytoplasmic	NP_001074	mmu:68837	Fork head
K23388	UBL4	mmu03060(	PF17840.6(	GO:000810	cytoplasmic	NP_001265	mmu:27643	
K13219	SFPQ, PSF		PF00076.28	GO:200123	nuclear	XP_036020	mmu:71514	
K05697	PTPN6, SH	mmu05235(	PF00102.32	GO:004213	cytoplasmic	NP_038573	mmu:15170	
K04463	MAP2K5, M	mmu05418(	PF00069.31	GO:003563	cytoplasmic	BAB29020.1	mmu:23938	
K01785	galM, GAL	mmu00052(	PF01263.26	GO:001932	cytoplasmic	BAE36531.1	mmu:319625	
K20476	RIC1		PF07064.18	GO:000989	mitochondr	NP_001074	mmu:226089	
K23938	FHOD1	mmu05132(	PF02181.28	GO:003243	cytoplasmic	NP_808367	mmu:234686	
K17478	FKBP15, WAFL		PF23649.1(I	GO:001562	cytoplasmic	NP_001038	mmu:338355	
K06634	CCNH	mmu03420(	PF16899.10	GO:003280	nuclear	BAE23508.1	mmu:66671	
K02370	EXTL3	mmu00534(	PF09258.16	GO:001922	cytoplasmic	NP_001347	mmu:54616	
K13989	DERL2_3	mmu04141(	PF04511.20	GO:003315	extracellulo	NP_001345	mmu:70377	
K07943	ARL2		PF00025.27	GO:003111	cytoplasmic	NP_062696	mmu:56327	
K11137	TELO2, TE	mmu04150(	PF10193.14	GO:004423	cytoplasmic	NP_001157	mmu:71718	
K09290	TPM3	mmu04820(	PF00261.25	GO:009951	nuclear	NP_071709	mmu:59069	
K22128	PIEZO1_2, FAM38		PF24871.1(I	GO:004239	ER	E2JF22.1(R	mmu:234839	
K10761	THG1		PF14413.11	GO:002260	cytoplasmic	NP_001277	mmu:66628	
K08963	mtnA	mmu00270(	PF01008.23	GO:000548	cytoplasmic	NP_080699	mmu:67873	
K06635	PTTG	mmu04114(	PF04856.18	GO:000486	cytoplasmic	NP_001412	mmu:30939	
K27819	NCDN	mmu04724(	PF05536.17	GO:006024	cytoplasmic	NP_001411	mmu:26562	
K21813	ENDOV		PF04493.20	GO:000573	cytoplasmic	NP_001158	mmu:338371	
K08493	VTI1	mmu04130(	PF12352.13	GO:000810	cytoplasmic	O88384.1(R	mmu:53612	
XPSD(GCN1 general control of ami			PF24987.1(I	GO:002262	cytoplasmic	NP_766307	mmu:231659	
K21122	USP28		PF00443.35	GO:000962	nuclear	NP_780691	mmu:235323	

K16831	BORA		PF15280.11	GO:009022	cytoplasmic	NP_780474	mmu:77744	
K13164	MSL2		PF16685.11	GO:008009	cytoplasmic	BAD32486.	mmu:77853	
K16939	SEPT6_8_1	mmu05100(	PF00735.24	GO:003315	cytoplasmic	XP_006532	mmu:20362	
K22193	GPR89, GPHR		PF12430.13	GO:000810	ER	NP_080505	mmu:67549	
K24013	TMEM38, TRIC		PF05197.18	GO:000526	extracellulo	NP_653117	mmu:74166	
K00781	ST3GAL3	mmu01100(	PF00777.23	GO:004322	cytoplasmic	XP_051044	mmu:20441	
K16302	CNNM		PF01595.25	GO:003450	plasma mer	NP_291048	mmu:94220	
K22191	MS4A5_6_7		PF04103.21	GO:003198	plasma mer	NP_081485	mmu:69774	
K15127	TADA2B		PF22941.1(	GO:003124	nuclear	NP_001163	mmu:23115	MYB
K20315	ARHGAP21_23		PF00620.32	GO:000716	nuclear	Q69ZH9.2(	mmu:58996	
K17108	GBA2	mmu00600(	PF04685.19	GO:003111	cytoplasmic	BAD32491.	mmu:230101	
K09066	FIGLA		PF00010.31	GO:000097	cytoplasmic	NP_036143	mmu:26910	bHLH
K00058	serA, PHGI	mmu00270(	PF02826.25	GO:000548	cytoplasmic	NP_058662	mmu:236539	
K06523	ICAM2, CD	mmu04650(	PF03921.20	GO:009859	plasma mer	NP_034624	mmu:15896	
ore-binding factor beta subunit)			PF00402.23	GO:003243	cytoplasmic	NP_031751	mmu:12798	
I6E(V-set and immunoglobulin dor			PF13927.12	GO:001602	plasma mer	NP_001028	mmu:231668	
K04688	RPS6KB	mmu05200(	PF00069.31	GO:200123	cytoplasmic	BAE22625.	mmu:72508	
K12863	CWC15	mmu03040(	PF04889.17	GO:007100	nuclear	NP_075642	mmu:66070	
K17292	TBCA		PF02970.22	GO:002260	cytoplasmic	CAH67875	mmu:21371	
K20398	LAMTOR2	mmu04150(	PF03259.23	GO:004340	mitochondr	NP_001099	mmu:83409	
K24006	PAM		PF03712.20	GO:000449	ER	NP_001407	mmu:18484	
K16353	SDK		PF00041.27	GO:005080	cytoplasmic	AAI38454.1	mmu:330222	
RRF(IGv); ENOG410YQB2(immur			PF07686.23(V-set:Immu		plasma mer	CAB46310.1(immunoglobulin light		
K12472	EPS15	mmu04144(	PF12763.14	GO:000588	cytoplasmic	XP_006509	mmu:13859	
Microtubule associated monoxygena			PF00307.36	GO:000699	cytoplasmic	NP_001412	mmu:29856	
K12399	AP3S	mmu04142(	PF01217.26	GO:009951	cytoplasmic	NP_001039	mmu:11777	
K02866	RP-L10e, R	mmu05171(	PF00252.24	GO:000582	cytoplasmic	NP_001162	mmu:110954	
K04639	GNA13	mmu04072(	PF00503.26	GO:003175	cytoplasmic	NP_034433	mmu:14674	
K04346	GNA12	mmu04010(	PF00503.26	GO:000759	cytoplasmic	NP_034432	mmu:14673	
K08876	SCYL1		PF00069.31	GO:003250	cytoplasmic	NP_001348	mmu:78891	
K13872	SLC7A6		PF13520.12	GO:000534	plasma mer	XP_006531	mmu:330836	
K00999	CDIPT	mmu04070(	PF01066.26	GO:000665	extracellulo	NP_080914	mmu:52858	
K01922	PPCS, COA	mmu01240(	PF04127.22	GO:000663	cytoplasmic	NP_080770	mmu:106564	
K06640	ATR	mmu05165(	PF25032.1(	GO:000810	nuclear	NP_063917	mmu:245000	
K11987	PTGS2, CO	mmu05022(	PF03098.20	GO:200123	lysosomal	NP_035328	mmu:19225	
K24685	ZFAND2		PF01428.22	GO:000989	cytoplasmic	NP_001153	mmu:100494	
K15324	TSEN15		PF09631.16	GO:009030	nuclear	Q8R3W5.1(	mmu:66637	
K02098	SPIR		PF16474.11	GO:003608	cytoplasmic	NP_758491	mmu:234857	
K24236	C1QTNF3		PF00386.27	GO:004340	extracellulo	NP_001191	mmu:81799	
K01325	KLK1_2	mmu04614(	PF00089.32	GO:000425	lysosomal	NP_034772	mmu:16616	
K20640	ARHGAP19		PF00620.32	GO:004858	cytoplasmic	NP_001156	mmu:71085	
K20301	TRAPPC2, TRS20		PF04628.19	GO:004322	cytoplasmic	NP_001404	mmu:66050	
K08412	GPR39		PF00001.26	GO:003577	plasma mer	NP_081953	mmu:71111	

K00876	udk, UCK	mmu00983(	PF00485.24	GO:000921	cytoplasmic	NP_001404	mmu:80914	
K12560	FCGR2B, C	mmu04666(	PF13895.12	GO:004340	plasma mer	NP_001070	mmu:14130	
K09487	HSP90B, TI	mmu04915(	PF00183.23	GO:000810	ER	AAH10445.	mmu:22027	
K11807	WDTC1, DCAF9		PF24814.1(	GO:008009	cytoplasmic	NP_001397	mmu:230796	
K19036	IGHMBP2		PF13086.12	GO:008009	cytoplasmic	NP_033238	mmu:20589	
K07949	ARL5A		PF00025.27	GO:000810	cytoplasmic	NP_892039	mmu:75423	
KNTS(acyl-CoA dehydrogenase); CC			PF00441.29	GO:003444	mitochondr	NP_848914	mmu:338350	
ANJ(family with sequence similarit			PF12260.14	GO:000557	ER	NP_080338	mmu:67266	
K04354	PPP2R2	mmu05165(	PF24814.1(	GO:001920	cytoplasmic	BAC98196.2	mmu:52432	
K12185	VPS37	mmu04144(	PF07200.18	GO:000810	mitochondr	BAE23372.1	mmu:52348	
K19532	LBR	mmu00100(	PF01222.22	GO:003085	nuclear	NP_001408	mmu:98386	
K17412	MRPS34		PF16053.10	GO:001593	cytoplasmic	NP_075749	mmu:79044	
K10858	PMS2	mmu03430(	PF08676.16	GO:008009	cytoplasmic	NP_032912	mmu:18861	
K08117	APLP2		PF12925.12	GO:000486	ER	BAE29207.1	mmu:11804	
K13087	BCLAF1		PF15440.11	GO:200123	nuclear	BAE29048.1	mmu:72567	
K11436	PRMT3		PF22528.1(I	GO:000647	cytoplasmic	EDL22978.	mmu:71974	
K08737	MSH6	mmu05200(	PF00488.27	GO:003506	nuclear	NP_034960	mmu:17688	
steroid receptor RNA activator)			PF23598.1(I	GO:009859	cytoplasmic	NP_001139	mmu:214345	
K16911	DDX21		PF00270.35	GO:008009	nuclear	NP_062426	mmu:56200	
K05091	KIT, SCFR,	mmu04916(	PF07714.23	GO:000989	extracellulo	NP_001116	mmu:16590	
K09239	HIVEP		PF00096.32	GO:000582	nuclear	NP_031798	mmu:11052	zf-C2H2
K17452	AEBP2	mmu03083(	Polycomb r	GO:000989	nuclear	NP_001005	mmu:11569	
K02991	RP-S6e, RP	mmu05171(	PF01092.24	GO:002262	nuclear	NP_001001	mmu:20104	
ZRNR(-)				GO:000565	extracellulo	NP_001365	mmu:116621581	
K10992	SFR1		PF10376.14	GO:003306	nuclear	AAH24403.	mmu:67788	
K10302	FBXO22	mmu05132(	PF10442.14	GO:000989	nuclear	NP_082325	mmu:71999	
K02893	RP-L23Ae,	mmu05171(	PF03939.18	GO:004323	cytoplasmic	NP_000975	mmu:268449	
K19878	ERC2, CAST		PF10174.14	GO:000810	cytoplasmic	NP_444434	mmu:238988	
YBU1(-)			PF15302.11	GO:000573	cytoplasmic	NP_001239	mmu:97820	
K08794	CAMK1	mmu04921(	PF00069.31	GO:003315	cytoplasmic	NP_598687	mmu:52163	
K00933	E2.7.3.2	mmu00330(	PF00217.24	GO:000659	mitochondr	NP_001121	mmu:76722	
K12382	PSAP, SGP	mmu04142(	PF05184.20	GO:000810	cytoplasmic	NP_001139	mmu:19156	
K01540	ATP1B, CD	mmu04971(	PF00287.24	GO:000810	peroxisoma	NP_031528	mmu:11933	
K10581	UBE2O	mmu04120(	PF23046.1(t	GO:004323	nuclear	NP_776116	mmu:217342	
K13122	FRG1		PF06229.18	GO:009030	nuclear	XP_028624	mmu:14300	
K02206	CDK2	mmu05165(	PF00069.31	GO:004423	cytoplasmic	NP_904326	mmu:12566	
K24983	G3BP2		PF02136.26	GO:000548	cytoplasmic	NP_001074	mmu:23881	
Z3SZ(Testis expressed 2)				GO:004323	cytoplasmic	NP_938034	mmu:21763	
YGSW(Golgin A3)				GO:003015	cytoplasmic	NP_001334	mmu:269682	
K20067	STON		PF12016.13	GO:000802	cytoplasmic	EDL18947.	mmu:108800	
K11268	ESCO, ECO	mmu04110(Cell cycle)		GO:000810	nuclear	BAE38788.1	mmu:71988	
K04353	RAP1A	mmu04530(	PF00071.28	GO:004340	cytoplasmic	XP_045727	mmu:109905	
K01267	DNPEP		PF02127.20	GO:000417	mitochondr	NP_001104	mmu:13437	

K15685	CBLL1		PF18408.7(z	GO:199023	cytoplasmic	NP_001409	mmu:104836	
K15216	RRN3, TIFIA		PF05327.17	GO:200123	mitochondr	NP_001034	mmu:106298	
K26238	TMEM9		PF05434.16	GO:000810	ER	NP_001153	mmu:66241	
K00134	GAPDH, g	mmu01100(	PF02800.25	GO:004643	cytoplasmic	AAT40729.	mmu:14433	
K17866	DPH2		PF01866.22	GO:000367	cytoplasmic	NP_080620	mmu:67728	
K18665	NUDT8		PF00293.34	GO:000663	cytoplasmic	NP_079805	mmu:66387	
K18463	CCDC53	mmu04144(	PF10152.14	GO:005112	cytoplasmic	NP_080346	mmu:67282	
K06831	LGALS3		PF00337.29	GO:200123	cytoplasmic	P16110.3(R	mmu:16854	
JC7(Coiled-coil domain containing 43)				GO:000557	nuclear	NP_080194	mmu:52715	
-cell receptor-associated protein)			PF05529.17	GO:000810	extracellulo	NP_001157	mmu:12033	
Z30T(immunoglobulin superfamily member 6)				GO:000557	plasma mer	NP_109616	mmu:80719	
K01482	DDAH, ddaH		PF19420.4(I	GO:001922	mitochondr	NP_081269	mmu:69219	
K14443	TOB	mmu03018(	PF07742.17	GO:003250	mitochondr	NP_001253	mmu:12226	
K17092	ANXA2	mmu05132(	PF00191.25	GO:003238	cytoplasmic	NP_001396	mmu:12306	
K11088	SNRPD3, S	mmu05322(	PF01423.28	GO:000024	nuclear	XP_033788	mmu:67332	
K24032	SH3PXD2		PF00018.34	GO:000810	cytoplasmic	XP_006514	mmu:268396	
K11849	USP25	mmu04657(	PF00443.35	GO:000989	nuclear	XP_006523	mmu:30940	
K09501	SERPINH1, HSP47		PF00079.25	GO:004885	ER	NP_001104	mmu:12406	
K06573	SLC4A1, AI	mmu04966(	PF00955.27	GO:000810	cytoplasmic	NP_035533	mmu:20533	
K13202	TOE1		PF04857.26	GO:001679	cytoplasmic	NP_080930	mmu:68276	
XPIU(Ribosomal RNA adenine dim			PF13679.12	GO:000557	cytoplasmic	NP_705790	mmu:229503	
K09176	TFAP2A_B		PF03299.19	GO:003250	nuclear	NP_001288	mmu:21418	
K22139	MPC2	mmu05415(	PF03650.18	GO:003577	mitochondr	NP_081706	mmu:70456	
K24242	NT5C3	mmu01100(	PF05822.18	GO:004643	ER	NP_001385	mmu:107569	
K25391	DBN1		PF00241.25	GO:005080	cytoplasmic	NP_001170	mmu:56320	
K23377	THSD4, AD	mmu04350(	PF19030.6(	GO:000417	extracellulo	NP_001035	mmu:207596	
K04577	CALCRL	mmu04080(	PF00002.29	GO:000810	plasma mer	NP_061252	mmu:54598	
K10284	FBXL18		PF19729.6(I	GO:000815	mitochondr	EDL19083.	mmu:231863	
K02903	RP-L28e, R	mmu05171(	PF01778.22	GO:001593	mitochondr	NP_033107	mmu:19943	
K04614	V1R		PF03402.20	GO:003802	extracellulo	NP_598974	mmu:171247	
K09444	IRF1	mmu04917(	PF00605.23	GO:003265	nuclear	XP_030885	mmu:16362	IRF
Phosphoinositide-3-kinase, regulator			PF00168.36	GO:001989	cytoplasmic	NP_035973	mmu:23943	
K24220	MYH1s	mmu04814(	PF00063.27	GO:001645	cytoplasmic	NP_001034	mmu:17882	
K09257	NFKBIL2		PF00023.36	GO:004239	cytoplasmic	NP_898914	mmu:72749	
K03984	SERPINA1	mmu04610(	PF00079.25	GO:000695	extracellulo	NP_033272	mmu:20703	
Z5YZ(chromosome 1 open reading f			PF17069.10	GO:006500	nuclear	NP_076154	mmu:27981	
K04732	IRAK3	mmu04722(	PF00069.31	GO:003210	cytoplasmic	NP_082955	mmu:73914	
K01897	ACSL, fadD	mmu01100(	PF00501.34	GO:001564	ER	NP_082252	mmu:433256	
K11155	DGAT1	mmu00830(	PF03062.24	GO:000810	cytoplasmic	NP_034176	mmu:13350	
K12799	PYCARD, /	mmu05164(	PF00619.27	GO:200123	cytoplasmic	NP_075747	mmu:66824	
K03984	SERPINA1	mmu04610(	PF00079.25	GO:000695	extracellulo	NP_033273	mmu:20704	
K09201	YY	mmu03082(	PF13465.12	GO:003315	cytoplasmic	XP_021034	mmu:22632	zf-C2H2
K21761	SYNE3	mmu04820(	PF10541.14	GO:002260	cytoplasmic	NP_001036	mmu:212073	

.PNZ(TSC22 domain family, memb			PF01166.23	GO:008009	cytoplasmic	NP_033392	mmu:21807	TSC22
K27382	CKM	mmu00330(	PF00217.24	GO:000659	cytoplasmic	XP_017444	mmu:12715	
K10848	ERCC4, XP	mmu03420(	Nucleotide	GO:008009	nuclear	BAE23706.	mmu:50505	
27PU(chromosome 4 open reading f			PF11027.13	GO:007168	cytoplasmic	EDL37743.	mmu:68552	
K08498	STX6	mmu04130(	PF09177.16	GO:000810	cytoplasmic	XP_006529	mmu:58244	
K24220	MYH1s	mmu04814(	PF00063.27	GO:000810	cytoplasmic	NP_034985	mmu:17884	
K00889	PIP5K	mmu05231(	PF01504.24	GO:000690	cytoplasmic	NP_001280	mmu:18717	
K13084	TMEM55	mmu04070(	PF09788.14	GO:002260	cytoplasmic	NP_001297	mmu:219024	
K23912	FSTL1		PF23564.1(I	GO:003015	extracellula	NP_032073	mmu:14314	
K12036	TRIM72		PF00622.36	GO:005115	cytoplasmic	NP_001073	mmu:434246	
K08150	SLC2A13, ITR		PF00083.30	GO:003425	plasma mer	AAI19588.1	mmu:239606	
K06618	RB1	mmu05200(	PF08934.15	GO:008009	cytoplasmic	NP_033055	mmu:19645	
K15031	MTERF		PF02536.19	GO:002260	mitochondr	NP_835152	mmu:69821	
K03035	PSMD12, R	mmu05020(	PF22241.2(I	GO:000573	cytoplasmic	NP_080170	mmu:66997	
.YF8(OCIA domain containing 2)			PF07051.17	GO:001602	cytoplasmic	NP_081226	mmu:433904	
K25207	ZSWIM8		PF21055.3(	GO:000989	cytoplasmic	NP_082272	mmu:268721	
K12487	GIT2	mmu05135(	PF12205.13	GO:004858	cytoplasmic	NP_001334	mmu:26431	
K04794	PTH2, PTRH2		PF01981.21	GO:001602	extracellula	NP_001092	mmu:217057	
K14850	RRP8		PF05148.21	GO:003506	nuclear	NP_598712	mmu:101867	
K03144	TFIIH4, G1	mmu03420(	PF03849.19	GO:003280	nuclear	NP_001400	mmu:14885	
K00276	AOC3, AO	mmu00360(	PF01179.26	GO:000578	plasma mer	NP_033805	mmu:11754	
K14010	TRAM1	mmu04141(	PF03798.21	GO:000810	ER	BAE30232.	mmu:72265	
K02432	FZD1_7, fz	mmu05165(	PF01534.23	GO:200123	plasma mer	NP_067432	mmu:14362	
K09448	TEAD	mmu04390(	PF17725.7(	GO:005505	nuclear	AAC12263.	mmu:21678	TEA
K13355	NUDT19	mmu04146(	Peroxisome	GO:000663	peroxisoma	NP_149071	mmu:110959	
K01810	GPI, pgi	mmu01250(	PF00342.24	GO:004643	cytoplasmic	NP_032181	mmu:14751	
)SYD(Crm, cramped-like (Drosophila))					nuclear	XP_006524	mmu:57354	
K01204	NAGA	mmu04142(	PF16499.11	GO:001613	lysosomal	BAE31642.	mmu:17939	
K03995	C6	mmu04610(	PF01823.24	GO:000268	plasma mer	XP_021039	mmu:12274	
K04504	NR1C2, PP	mmu04148(	PF00105.24	GO:008009	nuclear	NP_001398	mmu:19015	
K26421	FHIP2, FAM160B		PF10257.15(RAI16-like:		cytoplasmic	EDL01780.	mmu:226252	
K18668	ZC3H12, MCPIP		PF11977.14	GO:003577	cytoplasmic	NP_001156	mmu:244871	
K23480	ZNF362_384, LIN-29		PF13465.12	GO:005116	nuclear	XP_031239	mmu:26980	zf-C2H2
K14436	CHD6		PF00176.29	GO:001707	nuclear	NP_775544	mmu:71389	
K01253	EPHX1	mmu00980(	PF06441.18	GO:000663	ER	AAH29636.	mmu:13849	
K07870	RHOT1, AI	mmu04137(	PF08355.17	GO:005080	cytoplasmic	AAH52882.	mmu:59040	
K15109	SLC25A20_	mmu04714(	PF00153.32	GO:001587	cytoplasmic	AAH52871.	mmu:57279	
K03962	NDUFB6	mmu05208(	PF09782.14	GO:002260	cytoplasmic	NP_001028	mmu:230075	
K16482	POC1		PF00400.38	GO:002260	cytoplasmic	BAC38167.	mmu:70235	
K04515	CAMK2	mmu04750(	PF00069.31	GO:000810	extracellula	XP_017710	mmu:12325	
XQ2B(Zinc finger protein)			PF12756.13	GO:000367	cytoplasmic	NP_766163	mmu:24619	zf-C2H2
K13149	INTS12		PF00628.35	GO:009030	nuclear	NP_082203	mmu:71793	
K02149	ATPeV1D,	mmu05165(	PF01813.22	GO:000810	cytoplasmic	NP_076210	mmu:73834	

K19683	TTC30, DYF1		PF07719.23	GO:009951	cytoplasmic	NP_001074	mmu:620631	
K06101	ASH1L	mmu00310(	PF00856.33	GO:008009	nuclear	NP_619620	mmu:192195	
K06554	IGLL1, IGI	mmu05340(	PF07654.21	GO:004316	extracellula	AAH55906.	mmu:16136	
K06554	IGLL1, IGI	mmu05340(	PF07654.21	GO:000237	extracellula	AAH55911.	mmu:16136	
K23940	KPNA1	mmu03013(	PF00514.29	GO:003450	cytoplasmic	XP_021064	mmu:16646	
K17431	MRPL50		PF10501.15	GO:001593	mitochondr	NP_848718	mmu:28028	
K01062	PLA2G7, P.	mmu00565(	PF03403.19	GO:003444	ER	NP_001412	mmu:27226	
K23901	CALU		PF00036.39	GO:000578	ER	BAE38959.	mmu:12321	
K13811	PAPSS	mmu00230(	PF01583.26	GO:000933	cytoplasmic	NP_035993	mmu:23971	
K23932	SPTBN2	mmu05017(	PF00435.27	GO:001636	cytoplasmic	BAE28063.	mmu:20743	
K17427	MRPL46		PF11788.14	GO:001593	mitochondr	NP_075820	mmu:67308	
K10029	PPBP, CXC	mmu04062(	PF00048.25	GO:001922	extracellula	NP_076274	mmu:57349	
K10800	SMUG1	mmu03410(	PF03167.24	GO:001679	nuclear	BAE43042.	mmu:71726	
Dihydroorotase)			PF01979.26	GO:003250	cytoplasmic	NP_001278	mmu:22240	
K05855	SYK	mmu04664(	PF07714.23	GO:005116	cytoplasmic	XP_006516	mmu:20963	
K26542	ZNF346, JAZ		PF12874.13	GO:001989	nuclear	NP_036147	mmu:26919	
K13886	CORO1B_1C_6		PF08953.17	GO:003243	cytoplasmic	NP_001399	mmu:23789	
K14213	PEPD		PF00557.29	GO:000417	cytoplasmic	NP_032846	mmu:18624	
K03982	SERPINE1,	mmu04115(	PF00079.25	GO:200123	extracellula	ACX48997.	mmu:18787	
K10080	LMAN1, EI	mmu04141(	PF03388.20	GO:000810	mitochondr	BAE30539.	mmu:70361	
K27096	PPFIBP		PF00536.37	GO:000557	cytoplasmic	BAE32618.	mmu:67533	
K20910	TXNIP	mmu04621(	PF00339.34	GO:005116	cytoplasmic	NP_001009	mmu:56338	
K24926	TTC1		PF00515.34	GO:000557	cytoplasmic	NP_598556	mmu:66827	
K23338	GID8		PF10607.14	GO:000548	cytoplasmic	NP_001276	mmu:76425	
K01309	MINDY1_2		PF04424.18	GO:010100	cytoplasmic	NP_955769	mmu:75007	
K10360	MYO9	mmu04814(	PF00063.27	GO:001645	nuclear	NP_766606	mmu:270163	
K23490	CYB5		PF00173.33	GO:000812	cytoplasmic	BAE40334.	mmu:66427	
K03012	RPB4, POL	mmu03420(	PF03874.21	GO:004423	nuclear	NP_001102	mmu:69241	
K14437	CHD7		PF00176.29	GO:009008	nuclear	NP_001264	mmu:320790	
K16833	PPP1R2, IPP2		PF04979.19	GO:001920	cytoplasmic	EDK97747.	mmu:66849	
			PF02093.21	GO:004671	nuclear	AAB06450.	mmu:118567641	
2BB(family with sequence similarity 129, memb				GO:001056	cytoplasmic	NP_071301	mmu:63913	
K14567	UTP14	mmu03008(	PF04615.18	GO:003204	nuclear	BAE22282.	mmu:72554	
K23001	TOR1AIP		PF05609.18	GO:003450	nuclear	Q921T2.3(R	mmu:208263	
K08066	NFYC, HA	mmu04612(	PF00125.30	GO:000097	nuclear	NP_001041	mmu:18046	NF-YC
K07374	TUBA	mmu04530(	PF00091.31	GO:000520	cytoplasmic	NP_001029	mmu:238463	
K03243	EIF5B		PF00009.33	GO:002260	nuclear	NP_938045	mmu:226982	
K01296	CPM		PF00246.29	GO:001602	plasma mer	NP_081744	mmu:70574	
K00088	IMPDH, gu	mmu01100(	PF00478.31	GO:000916	cytoplasmic	NP_035960	mmu:23918	
K07249	ALDH1A	mmu00830(	PF00171.27	GO:001922	cytoplasmic	NP_001348	mmu:11668	
K10588	UBE3B	mmu04120(	PF00632.30	GO:000367	cytoplasmic	NP_001411	mmu:117146	
K19922	TSPOAP1, BZRAP1, RI		PF07653.23	GO:000526	cytoplasmic	NP_766037	mmu:207777	
K14769	UTP11		PF03998.19	GO:003204	cytoplasmic	NP_080307	mmu:67205	

K04496	CTBP	mmu05200(	PF02826.25	GO:008009	cytoplasmic	NP_038530	mmu:13016	
K23455	COL8A	mmu04974(	PF01391.23	GO:000520	extracellula	NP_031765	mmu:12837	
Heat shock protein)				GO:004316	cytoplasmic	NP_082582	mmu:72630	
K16497	PCDHGC		PF00028.23	GO:005080	plasma mer	NP_291059	mmu:93706	
K15377	SLC44A2_4	mmu05231(	PF04515.18	GO:001585	plasma mer	NP_690021	mmu:68682	
K14165	K14165		PF22784.1(I	GO:002260	cytoplasmic	NP_081001	mmu:68440	
K18728	EIF4ENIF1		PF10477.14	GO:005116	cytoplasmic	BAC30999.1	mmu:74203	
K14312	NUP155, N	mmu03013(	PF03177.20	GO:003450	cytoplasmic	NP_573490	mmu:170762	
K00864	glpK, GK	mmu03320(	PF00370.27	GO:008009	cytoplasmic	BAE41209.1	mmu:14933	
K01325	KLK1_2	mmu04614(	PF00089.32	GO:000425	lysosomal	NP_034244	mmu:13646	
K04459	DUSP, MK	mmu04148(	PF00782.25	GO:001679	cytoplasmic	XP_025771	mmu:235584	
K10688	UBE2W, U	mmu04120(	PF00179.31	(UQ_con:U	cytoplasmic	XP_006495	mmu:66799	
K15281	SLC35D1_2_3		PF03151.22	GO:001593	ER	NP_001001	mmu:70484	
K17636	RGL2	mmu04014(	PF00617.24	GO:001064	plasma mer	AIC83998.1	mmu:19732	
K23369	ADAMTSL4		PF19030.6(	GO:000548	extracellula	NP_001288	mmu:229595	
K00287	DHFR, fol	mmu01240(	PF00186.24	GO:008009	cytoplasmic	NP_034179	mmu:13361	
K00420	QCR10, UQ	mmu05208(	PF08997.15	GO:004322	ER	EDL31547.	mmu:66594	
K16755	CCDC61			GO:002260	cytoplasmic	NP_001028	mmu:232933	
K03948	NDUFA4	mmu05208(	PF06522.17	GO:003241	cytoplasmic	EDM15077	mmu:17992	
K03660	OGG1	mmu03410(	PF07934.18	GO:000962	nuclear	NP_035087	mmu:18294	
K05862	VDAC1	mmu05208(	PF01459.28	GO:004343	cytoplasmic	BAE40179.1	mmu:22333	
K06965	PELO, DO	mmu03015(	PF03463.20	GO:199053	cytoplasmic	BAE27128.1	mmu:105083	
K11458	PHC3, EDF	mmu03083(	PF00536.37	GO:004239	nuclear	NP_001159	mmu:241915	
K24505	GMEB		PF01342.26	GO:004280	cytoplasmic	NP_001406	mmu:22900	SAND
K18085	MTMR10_11_12		PF06602.19	GO:001679	cytoplasmic	NP_852074	mmu:194126	
K27391	ZNFX1	mmu04622(	PF13087.12	GO:003577	nuclear	NP_001028	mmu:98999	TF_others
Armadillo repeat containing, X-link			PF13465.12	GO:000097	cytoplasmic	NP_001349	mmu:32068	zf-C2H2
K10062	COLEC12	mmu04145(	PF01391.23	GO:004858	plasma mer	NP_569716	mmu:140792	
K10843	ERCC3, XP	mmu03420(	PF16203.11	GO:000810	cytoplasmic	NP_598419	mmu:13872	
K14480	APOL		PF05461.16	GO:004423	cytoplasmic	NP_001162	mmu:71898	
K10567	NEIL1	mmu03410(	PF09292.15	GO:000390	nuclear	NP_082623	mmu:72774	
K20175	ANKRD27		PF00023.36	GO:000810	cytoplasmic	BAH79262.	mmu:245886	
K03985	PLAUR, CI	mmu04610(	PF00021.27	GO:200123	extracellula	NP_035243	mmu:18793	
K06065	NCOR2, SM	mmu04330(	Notch signaling pathw		cytoplasmic	XP_030110	mmu:20602	
K17680	PEO1	mmu05017(	PF13481.12	GO:003421	mitochondr	NP_722491	mmu:226153	
K25777	GOLGA7, GCP16		PF10256.14	GO:007265	mitochondr	NP_001035	mmu:57437	
K16628	COL7A	mmu04974(	PF01391.23	GO:000520	extracellula	NP_031764	mmu:12836	
K17885	MTCH		PF00153.32	GO:200123	plasma mer	BAE30498.1	mmu:56428	
K11550	SPBC25, SPC25		PF08234.17	GO:004584	nuclear	NP_001186	mmu:66442	
K00417	QCR7, UQ	mmu05208(	PF02271.21	GO:000367	mitochondr	NP_080495	mmu:67530	
K12603	CNOT6, C	mmu03018(	PF03372.29	GO:008009	cytoplasmic	NP_001277	mmu:104625	
ZSRX(G patch domain and ankyrin			PF12796.13	GO:009715	nuclear	NP_001122	mmu:81845	
K24777	WDFY4		PF02138.23	GO:000578	cytoplasmic	XP_006519	mmu:545030	

Y9GN(Up-regulator of cell proliferation)				GO:003563	cytoplasmic	BAE42394.1	mmu:434223	
K25049	TMEM106A		PF21002.2(	GO:003578	cytoplasmic	NP_001346	mmu:217203	
K26225	RIMBP3		PF07653.23	GO:003015	cytoplasmic	NP_001028	mmu:239731	
K20045	ITSN		PF16652.11	GO:009008	cytoplasmic	XP_006522	mmu:16443	
K15516	FMR		PF12235.13	GO:003577	cytoplasmic	Q9WVR4.1	mmu:23879	
K04078	groES, HSPE1		PF00166.26	GO:000645	cytoplasmic	NP_032329	mmu:15528	
ZRJN(kiaa0930)			PF09741.14(DUF2045:U		mitochondr	BAE29080.1	mmu:223739	
K23882	CISD2		PF10660.14	GO:000989	ER	NP_080178	mmu:67006	
K10100	FBXO6	mmu04141(	PF04300.19	GO:199023	lysosomal	NP_001157	mmu:50762	
K04429	TAO	mmu04010(	PF00069.31	GO:004858	cytoplasmic	NP_001074	mmu:330177	
K04391	CD14	mmu04010(	PF12799.13	GO:003202	extracellulo	NP_033971	mmu:12475	
NatA auxiliary subunit)			PF01652.23	GO:009007	peroxisoma	NP_080105	mmu:66892	
K02902	RP-L28, MF	mmu03010(	PF00830.24	GO:001593	mitochondr	NP_077189	mmu:68611	
K12178	COPS4, CSN4		PF22241.2(I	GO:000802	cytoplasmic	BAE39855.1	mmu:26891	
K15743	CES3_5		PF00135.34	GO:001678	lysosomal	AAH48380.	mmu:382053	
K06130	LYPLA2	mmu00564(	PF02230.22	GO:000663	cytoplasmic	NP_001405	mmu:26394	
K20703	FAF1		PF21021.3(I	GO:200123	cytoplasmic	NP_032009	mmu:14084	
K05746	ENAH, ME	mmu04810(	PF00568.29	GO:003243	cytoplasmic	NP_001076	mmu:13800	
K22531	ATAD2		PF00004.35(AAA:ATPa		nuclear	NP_001349	mmu:320817	
Regulator of G-protein signaling)			PF16624.10	GO:003101	nuclear	NP_001398	mmu:62704	zf-C2H2
K23794	PHLDB		PF00169.35	GO:000810	nuclear	XP_006521	mmu:208177	
K00799	GST, gst	mmu00980(	PF02798.26	GO:001649	cytoplasmic	NP_032211	mmu:14871	
K04079	HSP90A, ht	mmu04915(	PF00183.23	GO:008009	cytoplasmic	ELK00257.1	mmu:15516	
K07196	TRIP10, CI	mmu04910(	PF00611.28	GO:009951	cytoplasmic	EDL38257.1	mmu:106628	
K15504	ANKRD52		PF12796.13	GO:003304	cytoplasmic	NP_766378	mmu:237615	
K08822	GSK3A	mmu04062(	PF00069.31	GO:009951	cytoplasmic	NP_001026	mmu:606496	
K24647	PRDM15		PF21549.2(I	GO:008009	cytoplasmic	NP_001390	mmu:11460	zf-C2H2
K18400	KANSL1		PF15275.11(PEHE:PEH		nuclear	NP_001359	mmu:76719	
K02126	ATPeF0A, I	mmu05208(	PF00119.25	GO:000962	extracellulo	AKC99164.	mmu:17705	
K06563	CLEC4L_M	mmu05162(	PF00059.27	GO:001922	plasma mer	NP_570974	mmu:170779	
K12580	CNOT3, N	mmu03018(	PF04065.20	GO:003577	nuclear	NP_666288	mmu:232791	
K15009	SHANK	mmu04724(	PF12796.13	GO:003514	cytoplasmic	Q4ACU6.3(	mmu:58234	
K09430	ELK3		PF00178.28	GO:000097	nuclear	CBA13447.1	mmu:13713	ETS
K13158	HNRNPA2	mmu05014(	PF00076.28	GO:199024	nuclear	KAG32780	mmu:53379	
KMM(cytohesin 1 interacting prote			PF00595.29	GO:000573	cytoplasmic	BAE42034.1	mmu:227929	
K00799	GST, gst	mmu00980(	PF00043.30	GO:009875	cytoplasmic	NP_034490	mmu:14866	
K22470	SQOR	mmu00920(	PF07992.20	GO:190136	mitochondr	BAE29187.1	mmu:59010	
K02270	COX7A	mmu05208(	PF02238.20	GO:002260	mitochondr	NP_034075	mmu:12866	
K11173	ADHFE1		PF00465.25	GO:004322	mitochondr	NP_780445	mmu:76187	
K18081	MTMR1_2	mmu04070(	PF06602.19	GO:005080	cytoplasmic	NP_001359	mmu:77116	
K00907	MYLK	mmu04971(	PF00069.31	GO:003202	cytoplasmic	AAI58042.1	mmu:228785	
K12345	SRD5A3	mmu00510(	PF02544.21	GO:000578	ER	NP_065636	mmu:57357	
K26099	PTTG1IP			GO:200123	plasma mer	BAC40985.1	mmu:108705	

K25857	ARID5		PF01388.27	GO:008009	nuclear	NP_076087	mmu:71371	ARID
K08568	CTSZ	mmu04142(	PF00112.29	GO:000417	lysosomal	NP_071720	mmu:64138	
K09491	STCH		PF00012.26	GO:000645	ER	NP_084477	mmu:110920	
K06751	MHC1	mmu05165(	PF00129.23	GO:000989	lysosomal	NP_001137	mmu:15015	
nascent polypeptide-associated com			PF01849.23	GO:000810	cytoplasmic	NP_001075	mmu:192950	
K02324	POLE	mmu03030(	PF08490.17	GO:004423	nuclear	AAH48166.	mmu:18973	
K26050	MYOZ	mmu04820(	PF05556.16	GO:004885	cytoplasmic	NP_067478	mmu:59006	
K13103	TFIP11		PF07842.17	GO:000548	nuclear	NP_061253	mmu:54723	
K16673	LIX1L		PF14954.12	GO:000573	mitochondr	Q8BQ89.2(	mmu:280411	
K18728	EIF4ENIF1		PF10477.14	GO:005116	cytoplasmic	NP_001160	mmu:74203	
K11876	PSMG2, PAC2		PF09754.15	GO:004584	nuclear	NP_598899	mmu:107047	
K06553	VPREB, CD179a		PF07686.23	GO:001972	cytoplasmic	ALD84362.	mmu:22362	
K07253	MIF	mmu00360(	PF01187.23	GO:200123	cytoplasmic	NP_034928	mmu:17319	
K10092	LGALS7		PF00337.29	GO:000573	cytoplasmic	NP_001389	mmu:16858	
K17941	SNX22_24		PF00787.29	GO:000810	cytoplasmic	NP_001008	mmu:69226	
K03023	RPC3, POL	mmu04623(	PF05645.19	GO:001922	cytoplasmic	NP_083201	mmu:74414	
K11129	NHP2, NO	mmu03008(	PF01248.31	GO:001593	cytoplasmic	NP_080907	mmu:52530	
K17563	CHCHD3		PF05300.16	GO:001989	nuclear	NP_079612	mmu:66075	
K23408	CDCA7, CDCA7L, JPO		PF10497.14	GO:008009	cytoplasmic	NP_080142	mmu:66953	
K16547	NEDD1		PF24807.1(	GO:000024	cytoplasmic	NP_032708	mmu:17997	
K04494	CHD8, HEl	mmu04310(	PF23078.2(	GO:003506	nuclear	XP_011243	mmu:67772	
K12592	C1D, LRP1	mmu03018(	PF04000.20	GO:000989	cytoplasmic	NP_001317	mmu:57316	
ZWWX(glycosyltransferase-like dom			PF12038.13	GO:008009	cytoplasmic	BAC25967.	mmu:227835	
K07197	SREBP1, SI	mmu04152(	PF00010.31	GO:009008	cytoplasmic	NP_001345	mmu:20787	bHLH
K00650	LCAT	mmu04979(	PF02450.21	GO:003435	lysosomal	NP_032516	mmu:16816	
K09485	HSP110	mmu04141(	PF00012.26	GO:200123	cytoplasmic	NP_038587	mmu:15505	
Non-specific O-methyltransferase th			PF00378.25	GO:003444	mitochondr	NP_081004	mmu:52430	
Maf-like protein); ENOG410XS7T(o			PF02545.19	GO:001678	mitochondr	NP_081215	mmu:69099	
K09222	REST	mmu05016(	PF13465.12	GO:008009	nuclear	NP_035393	mmu:19712	zf-C2H2
K15442	TAD3, ADAT3		PF00383.29	GO:000825	cytoplasmic	NP_001094	mmu:100113398	
K24195	XPR1, PHO1		PF03124.20	GO:005506	cytoplasmic	NP_035403	mmu:19775	
K03439	trmB, METTL1, TRM8		PF02390.23	GO:199023	nuclear	NP_034922	mmu:17299	
K19672	IFT140		PF24762.1(	GO:000858	cytoplasmic	NP_001397	mmu:106633	
K08868	ALPK		PF02816.24	GO:005501	nuclear	NP_473426	mmu:116904	
K14289	XPO5	mmu03013(	PF19273.5(	GO:001593	cytoplasmic	NP_082474	mmu:72322	
K19026	SPG11	mmu05014(	PF14649.11	GO:004867	cytoplasmic	NP_663506	mmu:214585	
K00560	thyA, TYM	mmu01523(	PF00303.24	GO:000921	cytoplasmic	NP_067263	mmu:22171	
K23308	NUFIP1		PF10453.14	GO:002260	nuclear	NP_038773	mmu:27275	
K17538	PEAK1, SGK269		PF00069.31	GO:002260	cytoplasmic	NP_766512	mmu:244895	
K20372	TBC1D20, GYP8		PF00566.25	GO:003238	cytoplasmic	NP_077158	mmu:67231	
K08291	GRK4_5_6	mmu04062(	PF00069.31	GO:004858	cytoplasmic	NP_061357	mmu:14773	
K11791	DCAF15		PF14939.11	GO:000020	cytoplasmic	NP_001344	mmu:212123	
K08110	GPC4	mmu04310(	PF01153.25	GO:000989	extracellulo	NP_032176	mmu:14735	

K12319	GUCY1B	mmu04970(	PF00211.25	GO:000401	cytoplasmic	NP_059497	mmu:54195	
K06626	CCNE	mmu05165(	PF00134.28	GO:008009	cytoplasmic	AAH03499.	mmu:12447	
K09530	DNAJC10	mmu04141(	PF00085.26	GO:001922	ER	NP_077143	mmu:66861	
K00344	qor, CRYZ		PF00107.32	GO:000827	cytoplasmic	NP_001344	mmu:12972	
K09228	KRAB		PF13465.12	GO:004859	cytoplasmic	NP_001028	mmu:24390	zf-C2H2
K13646	PLOD3	mmu00514(	PF03171.26	GO:000810	ER	NP_036092	mmu:26433	
K00473	PLOD1	mmu00310(	PF03171.26	GO:003250	ER	NP_035252	mmu:18822	
K24135	MORC		PF13589.12	GO:008009	cytoplasmic	BAD32320.	mmu:74522	
HXC(Adaptor-related protein com			PF15745.10	GO:005112	cytoplasmic	NP_001333	mmu:211556	
NTS(acyl-CoA dehydrogenase); COG3173(Am				GO:003444	cytoplasmic	BAC30711.	mmu:338350	
K18470	ARHGAP1, CDC42GA		PF00620.32	GO:000004	cytoplasmic	NP_001139	mmu:228359	
K17985	AMBRA1	mmu04137(	PF24814.1(	GO:008009	cytoplasmic	EDL27576.	mmu:228361	
K07520	ABLIM	mmu04360(	PF16182.10	GO:003243	cytoplasmic	BAD32318.	mmu:319713	
K20056	GIPC, SEMCAP		PF25083.1(	GO:000810	cytoplasmic	NP_061241	mmu:67903	
K17822	DCUN1D1_2		PF03556.21	GO:190187	cytoplasmic	BAE38332.	mmu:114893	
K03109	SRP9	mmu03060(	PF05486.17	GO:000578	cytoplasmic	NP_036188	mmu:27058	
K24426	PYROXD1		PF07992.20	GO:190136	cytoplasmic	NP_898988	mmu:232491	
K00826	E2.6.1.42, il	mmu00290(	PF01063.24	GO:190160	cytoplasmic	NP_031558	mmu:12035	
K17725	ETHE1	mmu00920(	PF00753.33	GO:005031	mitochondr	NP_001350	mmu:66071	
K17410	MRPS31		PF15433.11	GO:001593	mitochondr	NP_065585	mmu:57312	
K04723	IL1RAP	mmu04750(	PF01582.25	GO:190560	plasma mer	EDK97692.	mmu:16180	
K23353	PDLIM1_2	mmu04820(	PF00595.29	GO:003243	cytoplasmic	NP_001240	mmu:213019	
K23344	DDRGK1		PF09756.15	GO:000810	ER	NP_084108	mmu:77006	
K10486	BTBD14		PF00651.37	GO:200123	nuclear	NP_001032	mmu:67991	ZBTB
K16498	PCDHD1		PF00028.23	GO:004316	plasma mer	AAI50666.1	mmu:219257	
K17773	TOM5		PF10642.14	GO:000810	cytoplasmic	NP_001093	mmu:68512	
K15043	KPNA2_7	mmu03013(	PF00514.29	GO:005116	cytoplasmic	NP_034785	mmu:16647	
K20348	TMED1		PF01105.29	GO:003802	ER	NP_034874	mmu:17083	
K12015	TRIM39, BTR		PF00622.36	GO:200123	cytoplasmic	BAD90223.	mmu:79263	
K19866	EFEMP2		PF07645.21	GO:000810	extracellulo	NP_001361	mmu:58859	
K00737	MGAT3	mmu00510(	PF04724.18	GO:000810	Golgi appa	Q10470.2(R	mmu:17309	
K03469	rnhA, RNA	mmu03030(	PF00075.30	GO:003204	nuclear	NP_035405	mmu:19819	
K00962	pnp, PNPT	mmu03018(	PF01138.27	GO:007104	mitochondr	NP_082145	mmu:71701	
G47(Pfam:Eeig1)			PF10358.15(NT-C2:N-t		nuclear	NP_001157	mmu:329739	
K11273	DDX11, CI	mmu04110(	PF06733.21	GO:003206	cytoplasmic	NP_001003	mmu:320209	
K00760	hprT, hpt, I	mmu01100(	PF00156.33	GO:004643	cytoplasmic	NP_038584	mmu:15452	
K17398	DNMT3A	mmu04148(	PF22855.1(	GO:008009	cytoplasmic	NP_001258	mmu:13435	
K17399	DNMT3B	mmu00270(	PF17980.7(	GO:008009	cytoplasmic	XP_006498	mmu:13436	
K19872	FKTN	mmu00515(	PF19737.4(	GO:000578	ER	XP_006538	mmu:246179	
K01507	ppa	mmu00190(	PF00719.24	GO:000367	cytoplasmic	NP_080714	mmu:67895	
K22615	NLRC5, NOD4		PF18461.7(	GO:008009	cytoplasmic	NP_001028	mmu:434341	
K10884	XRCC6, KU	mmu03450(	PF03731.21	GO:008009	nuclear	NP_001398	mmu:14375	
K11721	BRD3		PF00439.30	GO:000810	nuclear	NP_001107	mmu:67382	

K24763	RMC1		PF07035.17	GO:000989	cytoplasmic	BAE35292.1	mmu:76482	
K11824	AP2A	mmu05016(	PF01602.26	GO:003243	cytoplasmic	NP_031484	mmu:11771	
K11824	AP2A	mmu05016(	PF01602.26	GO:000802	cytoplasmic	NP_031485	mmu:11772	
K22816	CNPY3_4		PF11938.13	GO:000578	ER	NP_082341	mmu:72029	
K03129	TAF4	mmu05016(	PF05236.20	GO:008009	mitochondr	NP_001093	mmu:72504	
K15603	TCF4_12		PF00010.31	GO:008009	nuclear	XP_006831	mmu:21406	bHLH
K11660	MTA	mmu03082(	PF17226.7(	GO:200073	cytoplasmic	NP_001164	mmu:11687	zf-GATA
K13907	CSTA_B		PF00031.27	GO:000989	cytoplasmic	NP_031819	mmu:13014	
K24024	RTKN		PF08174.17	GO:000367	cytoplasmic	NP_001129	mmu:20166	
K04372	MKNK, MI	mmu04010(	PF00069.31	GO:008009	nuclear	NP_001272	mmu:17346	
K01245	ADPRH		PF03747.19	GO:001679	cytoplasmic	NP_031440	mmu:11544	
K15292	STXBP1, M	mmu04721(	PF00995.28	GO:001989	cytoplasmic	NP_001027	mmu:20910	
K00490	CYP4F		PF00067.27	GO:003444	ER	NP_077764	mmu:72054	
K24761	WDR92, DNAAF10		PF00400.38	GO:002260	cytoplasmic	NP_849240	mmu:103784	
von Willebrand factor, type A)			PF08487.16	GO:003250	cytoplasmic	EDL25489.	mmu:244810	
K23538	ELMOD		PF04727.18	GO:005068	plasma mer	NP_001164	mmu:244548	
K14863	WDR12, YTM1		PF00400.38	GO:000046	nuclear	NP_001185	mmu:57750	
K23286	NEBL1_2		PF02138.23	GO:000367	cytoplasmic	AAI72118.1	mmu:235627	
K13096	SF4, SUGP1		PF01805.25	GO:000557	cytoplasmic	NP_001355	mmu:70616	
K21853	DOCK9_10_11		PF06920.19	GO:003250	cytoplasmic	NP_001009	mmu:75974	
U90(Myotrophin)			PF13637.12	GO:004423	cytoplasmic	XP_006861	mmu:14489	
K10627	RAD18		PF02037.33	GO:008009	nuclear	NP_001368	mmu:58186	
K24725	AAMP		PF00400.38	GO:001481	cytoplasmic	NP_001177	mmu:227290	
K24418	METTL5		PF05175.20	GO:004323	nuclear	NP_083556	mmu:75422	
K16817	PLA2G16	mmu04014(	PF04970.19	GO:000578	cytoplasmic	NP_001349	mmu:225845	
K12839	SMNDC1, S	mmu03040(	PF06003.18	GO:008009	cytoplasmic	NP_001343	mmu:76479	
K00671	NMT		PF02799.21	GO:004322	cytoplasmic	NP_001277	mmu:18108	
K20721	RTN1		PF02453.23	GO:002260	cytoplasmic	NP_703187	mmu:104001	
K10056	JUP, PG	mmu04820(	PF00514.29	GO:003315	cytoplasmic	NP_034723	mmu:16480	
K09404	FOXK	mmu03083(	PF00250.24	GO:008009	nuclear	NP_951031	mmu:17425	Fork head
K06642	PRKDC	mmu03450(	PF20500.4(I	GO:004858	ER	NP_035289	mmu:19090	
K02540	MCM2	mmu03030(	PF00493.29	GO:190231	cytoplasmic	BAE27124.1	mmu:17216	
K02542	MCM6	mmu03030(	PF00493.29	GO:003126	cytoplasmic	NP_032593	mmu:17219	
K09377	CSRP	mmu04820(	PF00412.28	GO:003103	cytoplasmic	NP_001244	mmu:13007	
K01102	PDP		PF00481.26	GO:001679	mitochondr	XP_021077	mmu:381511	
YAJH(pre-B-cell leukemia homeobox interacting				GO:009951	nuclear	NP_666243	mmu:229534	
K19348	TMEM67, MKS3		PF09773.14	GO:008009	plasma mer	XP_006538	mmu:329795	
K02210	MCM7, CD	mmu03030(	PF00493.29	GO:001922	nuclear	NP_032594	mmu:17220	
K17928	SNX16		PF00787.29	GO:003131	cytoplasmic	NP_001120	mmu:74718	
K10356	MYO1	mmu04814(	PF00063.27	GO:009951	cytoplasmic	NP_796364	mmu:338367	
K10905	ATRIP	mmu03460(	Fanconi an	GO:014003	nuclear	NP_766362	mmu:235610	
K18341	TBC1D1	mmu04152(	PF00566.25	GO:009008	nuclear	NP_001276	mmu:57915	
K22829	MCUB		PF04678.18	GO:199024	mitochondr	NP_080055	mmu:66815	

289D(kiaa1143)			PF15377.11(DUF4604:I		nuclear	NP_079695	mmu:66202	
227Z(chromosome 5 open reading fr			PF15749.10	GO:000810	mitochondr	EDL33721.	mmu:68067	
K12807	NAIP, BIR(	mmu04621(	PF00653.27	GO:000959	cytoplasmic	AAI16627.1	mmu:17948	
K14302	NUP37	mmu03013(	PF00400.38	GO:001593	cytoplasmic	NP_081467	mmu:69736	
K18193	MP68, MLQ		PF08039.16	GO:000575	cytoplasmic	EDL18604.	mmu:70257	
K12165	UFC1		PF08694.16	GO:006165	cytoplasmic	NP_001347	mmu:66155	
K15420	EML4	mmu05200(	PF23409.1(I	GO:003111	cytoplasmic	NP_001366	mmu:78798	
K15720	N4BP2		PF25124.1(I	GO:001920	nuclear	XP_006504	mmu:333789	
K14860	TMA16		PF11176.13	GO:004322	nuclear	NP_079741	mmu:66282	
K06693	PSMD9, RF	mmu05020(	PF18265.6(	GO:009008	cytoplasmic	NP_080276	mmu:67151	
NBN(Chromosome 1 open reading			PF15559.11	GO:000565	nuclear	NP_079831	mmu:66421	
K17662	CBP3, UQCC		PF03981.17	GO:002260	mitochondr	NP_061376	mmu:56046	
K03120	TBP, tbp	mmu05165(	PF00352.27	GO:000921	nuclear	NP_001069	mmu:237336	
K22366	SELENOT		PF10262.15	GO:009008	ER	NP_001014	mmu:69227	
K27381	XIRP	mmu04820(	PF08043.17	GO:003243	cytoplasmic	NP_001077	mmu:241431	
K13645	PLOD2	mmu00310(	PF03171.26	GO:001649	ER	NP_001136	mmu:26432	
K02133	ATPeF1B, /	mmu05208(	PF00006.31	GO:000526	mitochondr	AAH37127.	mmu:11947	
K25605	BCL7	mmu03082(	PF04714.18	GO:003304	cytoplasmic	XP_020752	mmu:12054	
K05283	PIGW	mmu00563(	PF06423.18	GO:000810	plasma mer	NP_001071	mmu:70325	
K13138	INTS1		PF22929.2(I	GO:004858	nuclear	BAF03197.1	mmu:68510	
K13951	ADH1_7	mmu00980(	PF08240.17	GO:000152	cytoplasmic	BAE26071.	mmu:11529	
K25407	MFAP3		PF13927.12	GO:000989	ER	NP_663401	mmu:216760	
K21765	MNR		PF15718.10	GO:002260	nuclear	NP_001348	mmu:74477	
K17451	EZH1	mmu03083(	PF21358.2(I	GO:008009	nuclear	NP_001390	mmu:14055	
K01011	TST, MPST	mmu04122(	PF00581.25	GO:001593	peroxisoma	NP_033463	mmu:22117	
DR8(Leucine rich repeat containin			PF13855.12	GO:000573	extracellulo	NP_694714	mmu:211228	
K00658	DLST, sucB	mmu00310(	PF00198.28	GO:003197	mitochondr	NP_084501	mmu:78920	
K06501	CD68	mmu04142(	PF01299.22	GO:000268	extracellulo	BAA23738.	mmu:12514	
K11324	DMAP1, SV	mmu03082(	PF05499.17	GO:003315	cytoplasmic	NP_001405	mmu:66233	
K19526	VPS13B		PF12624.14	GO:006144	cytoplasmic	NP_796125	mmu:666173	
K10736	MCM10		PF24863.1(z	GO:004316	nuclear	EDL07958.	mmu:70024	
K23213	SIN3B		PF16879.10	GO:000371	cytoplasmic	BAE34795.	mmu:20467	
K11485	MTF2, PCL	mmu03083(	PF14061.12	GO:004239	nuclear	XP_004402	mmu:17765	
K14310	NUP205, N	mmu03013(	PF11894.14	GO:000699	mitochondr	EDL13668.	mmu:70699	
Y1I3(Chromosome 18 open reading			PF14975.11	GO:000578	ER	NP_001001	mmu:407819	
INN(Family with sequence similar			PF10187.14	GO:000989	nuclear	NP_001350	mmu:102122	
XRRE(Coiled-coil domain containir			PF09762.14	GO:000810	cytoplasmic	NP_084231	mmu:70829	
K05291	PIGS	mmu00563(	PF10510.14	GO:004423	ER	NP_958808	mmu:276846	
K23312	STN1		PF09170.16	GO:008009	cytoplasmic	XP_006526	mmu:108689	
K10802	HMGB1	mmu04140(	PF09011.16	GO:004508	nuclear	CAH72778(	mmu:15289	HMG
K16453	CCP110, CEP110		PF16025.10	GO:190372	cytoplasmic	BAE24781.	mmu:101565	
K23961	EPB41L1_2_3		PF05902.18	GO:002260	cytoplasmic	BAG84712.	mmu:13822	
K12160	SUMO, SM	mmu03013(	PF11976.14	GO:003315	nuclear	NP_001005	mmu:22218	

K12835	DDX42, SF	mmu03040(	PF00270.35	GO:000810	cytoplasmic	NP_082350	mmu:72047		
K06752	MHC2	mmu05323(	PF00969.24	GO:002260	cytoplasmic	NP_034512	mmu:14969		
K15630	VTC2			GO:000508	cytoplasmic	NP_848867	mmu:269952		
K24930	TTC9		PF13432.12(TPR_16:Te		cytoplasmic	NP_081688	mmu:70387		
K11583	PPP2R3	mmu05165(	PF17958.7(I	GO:001922	cytoplasmic	NP_067504	mmu:59032		
K10418	DYNLL	mmu04962(	PF01221.23	GO:009951	cytoplasmic	NP_001003	mmu:56455		
K14608	OASL	mmu05165(	PF10421.14	GO:009040	cytoplasmic	NP_035984	mmu:23962		
K17923	SNX9_18_3	mmu05132(	PF10456.14	GO:003608	cytoplasmic	BAE34110.1	mmu:66616		
K07864	RHOJ, TCL	mmu05132(	PF00071.28	GO:000716	cytoplasmic	BAE34111.1	mmu:80837		
YPY4(-)					cytoplasmic	BAC38923.1	mmu:14349		
K27310	TMEM41		PF09335.16	GO:000557	plasma mer	NP_079969	mmu:66664		
K10481	BTBD9		PF00651.37	GO:005080	cytoplasmic	XP_006524	mmu:22467	ZBTB	
9J7(chromosome 19 open reading fr			PF03954.19	GO:001602	plasma mer	NP_081261	mmu:69189		
ZVUA(proline and serine rich 1)			PF14771.11(DUF4476:D		cytoplasmic	NP_775558	mmu:212127		
XSM4(MOB kinase activator)			PF03637.22	GO:000548	cytoplasmic	NP_780517	mmu:100465		
K13218	PTBP1, PTB		PF13893.12	GO:009008	nuclear	NP_001070	mmu:19205		
K27541	ZFX_Y		PF04704.18	GO:005125	nuclear	NP_001037	mmu:22764	zf-C2H2	
K17885	MTCH		PF00153.32	GO:000810	plasma mer	AAH23135.	mmu:56462		
Z0E9(family with sequence similarit			PF08616.15	GO:000810	cytoplasmic	NP_080713	mmu:67894		
K14724	SLC9A8, NHE8		PF00999.27	GO:004864	cytoplasmic	NP_001291	mmu:77031		
K05403	TIRAP	mmu05161(	PF13676.11	GO:008009	cytoplasmic	NP_001171	mmu:117149		
K16760	CCHCR1		PF07111.17	GO:005116	cytoplasmic	XP_011244	mmu:240084		
K25412	CD5L		PF00530.23	GO:000425	extracellulo	NP_033820	mmu:11801		
K21798	SAC2, INPI	mmu04070(	PF02383.23	GO:001922	cytoplasmic	NP_848756	mmu:101490		
K06484	ITGA5, CD	mmu05165(	PF20806.3(I	GO:003411	plasma mer	EDL03922.1	mmu:16402		
K23353	PDLIM1_2_	mmu04820(	PF15936.10	GO:003243	cytoplasmic	NP_058557	mmu:54132		
K03260	EIF4G	mmu05416(	PF02854.25	GO:003288	cytoplasmic	NP_001390	mmu:208643		
K08853	AAK		PF00069.31	GO:005104	cytoplasmic	NP_001035	mmu:269774		
K12471	EPN	mmu04144(	PF01417.26	GO:006102	cytoplasmic	NP_001239	mmu:13854		
K27815	CAVIN4, MURC		PF15237.11	GO:009859	cytoplasmic	NP_080785	mmu:68016		
K03097	CSNK2A	mmu04137(	PF00069.31	GO:008009	cytoplasmic	NP_001403	mmu:12995		
K20353	SEC16			GO:004820	cytoplasmic	XP_011237	mmu:227648		
Y6GD(chromosome 11 open reading			PF10262.15	GO:000579	cytoplasmic	NP_001028	mmu:72657		
K08486	STX1B_2_3	mmu04130(	PF00804.31	GO:000688	cytoplasmic	NP_689344	mmu:20908		
K07152	SCO1		PF02630.20	GO:000810	cytoplasmic	BAE39409.1	mmu:52892		
K19944	TBC1D10		PF00566.25	GO:004340	peroxisoma	NP_848765	mmu:108995		
K24308	FUNDC2		PF04930.20	GO:000554	nuclear	NP_080402	mmu:67391		
K01371	CTSK	mmu05323(	PF00112.29	GO:200123	lysosomal	NP_031828	mmu:13038		
K05677	ABCD3, PM	mmu02010(	PF06472.20	GO:003444	mitochondr	NP_033017	mmu:19299		
K20057	SYNJ2BP, OMP25		PF00595.29	GO:000810	cytoplasmic	NP_079568	mmu:24071		
YVIZ(Chromosome 14 open reading			PF15394.11	GO:000989	cytoplasmic	BAE37200.1	mmu:74359		
HDU(kiaa1598)				GO:009951	cytoplasmic	BAD90258.	mmu:71653		
39P(chromosome 7 open reading fr			PF14964.11	GO:000557	cytoplasmic	NP_766314	mmu:231868		

K18420	TARBP2		PF00035.32	GO:005068	cytoplasmic	NP_033345	mmu:21357	
K21844	FAM126		PF09790.15	GO:000810	cytoplasmic	NP_444320	mmu:84652	
K22565	COMMD9		PF20923.2(	GO:004259	cytoplasmic	NP_083911	mmu:76501	
2B5C(kiaa1430)			PF13879.11	GO:003250	cytoplasmic	NP_001344	mmu:66756	
K17887	SNX25, MDM1		PF08628.17(Nexin_C:Sc		ER	NP_001359	mmu:102141	
K16776	NAV1		PF23092.1(I	GO:000367	cytoplasmic	BAC29566.1	mmu:215690	
K24691	ZNF106		PF24814.1(	GO:000367	nuclear	NP_035873	mmu:20402	
K15014	SLC29A1_2	mmu05034(	PF01733.24	GO:190379	extracellula	XP_030106	mmu:13340	
YW74(-); ENOG4111FCX(leucine rich repeat cc				GO:004212	cytoplasmic	XP_030109	mmu:381535	
K08239	GNPTAB	mmu04142(	PF11380.13	GO:000810	extracellula	NP_001004	mmu:432486	
K08874	TRRAP	mmu03082(	PF20206.4(Tra1_ring:T		cytoplasmic	XP_006504	mmu:100683	
K03094	SKP1, CBF	mmu04120(	PF01466.24	GO:000582	cytoplasmic	BAE38934.1	mmu:21402	
K20241	WDR44, RAB11BP		PF00400.38	GO:006049	cytoplasmic	NP_001292	mmu:72404	
K20030	ZDHHC5_8		PF01529.25	GO:000810	extracellula	EDL27297.1	mmu:228136	
)SKT(thymocyte selection associated			PF12736.12	GO:000695	cytoplasmic	AAH13712.	mmu:230787	
K13514	LPGAT1	mmu00564(	PF16076.10	GO:004592	ER	NP_001408	mmu:226856	
K04682	E2F4_5	mmu04218(	PF16421.10	GO:008009	nuclear	NP_031918	mmu:13559	E2F
K01481	E3.5.3.15		PF03068.20	GO:001922	cytoplasmic	NP_032838	mmu:18600	
K05113	EPHB4, HT	mmu04360(	PF07714.23	GO:200052	plasma mer	NP_034274	mmu:13846	
Chromosome 22 open reading frame			PF05742.17	GO:000810	cytoplasmic	EDK97516.	mmu:27883	
K18798	AFG1, LACE1		PF03969.22	GO:003563	mitochondr	NP_665686	mmu:215951	
K21916	KCTD6		PF02214.28	GO:000858	cytoplasmic	NP_001292	mmu:71393	
K03349	APC2, ANA	mmu04110(	PF00888.28	GO:003304	cytoplasmic	EDL08226.1	mmu:99152	
K01262	pepP		PF00557.29	GO:000417	mitochondr	NP_001334	mmu:321003	
K17402	MRPS23		PF10484.15	GO:001593	cytoplasmic	NP_077136	mmu:64656	
K07192	FLOT	mmu04910(	PF01145.30	GO:000810	cytoplasmic	NP_001035	mmu:14252	
10T8(chromosome 21 open reading f			PF06937.16	GO:003250	cytoplasmic	AAH19957.	mmu:67102	
1M9H(tumor protein)			PF04201.20	GO:004212	cytoplasmic	EDL05191.1	mmu:21985	
Gv)			PF12796.13(Ank_2:Ank		cytoplasmic	NP_001161	mmu:102334	
K18643	KATNB1		PF00400.38	GO:003111	cytoplasmic	NP_083081	mmu:74187	
K02985	RP-S3e, RP	mmu05171(	PF00189.25	GO:003111	cytoplasmic	XP_032748	mmu:27050	
K21918	KCTD8_12_16		PF23110.1(I	GO:009859	cytoplasmic	NP_808383	mmu:239217	
K14961	RBBP5, SW	mmu04934(	PF24817.1(	GO:004239	nuclear	BAE42295.1	mmu:213464	
K05357	VKORC1	mmu01240(	PF07884.19	GO:000677	peroxisoma	NP_081397	mmu:69568	
K13303	SGK2	mmu04151(	PF00069.31	GO:009910	cytoplasmic	BAE23044.1	mmu:27219	
K01320	F7	mmu04610(	PF00089.32	GO:000425	plasma mer	NP_034302	mmu:14068	
K08272	CAB39, MC	mmu04152(	PF08569.17	GO:001920	cytoplasmic	NP_001347	mmu:69008	
K13088	ELAVL1, H	mmu04152(	PF00076.28	GO:005116	cytoplasmic	NP_001102	mmu:15568	
K11110	TERF1, TRF1		PF08558.16	GO:008009	cytoplasmic	NP_033378	mmu:21749	MYB
1ZMS(ADP-ribosylation-like factor			PF10500.14	GO:009030	nuclear	EDL19604.1	mmu:65105	
K15759	IMPAD1, II	mmu04070(	PF00459.30	GO:003250	ER	NP_808398	mmu:242291	
K22038	LRRC8		PF12534.13	GO:000578	ER	NP_598658	mmu:100604	
K16545	DDHD2		PF02862.22	GO:000763	cytoplasmic	AAH46229.	mmu:72108	

K16757	CCDC77			GO:000557	cytoplasmic	NP_001368	mmu:67200	
K01540	ATP1B, CD	mmu04971(	PF00287.24	GO:000367	cytoplasmic	CAI52017.1	mmu:11932	
K11877	PSMG3, PAC3		PF10178.14	GO:005113	cytoplasmic	NP_001343	mmu:66506	
K24402	MXRA8		PF07686.23	GO:009859	ER	NP_077225	mmu:74761	
K24401	MXRA7			GO:000557	extracellula	NP_080556	mmu:67622	
Rab interacting lysosomal protein-li			PF13465.12	GO:004859	nuclear	NP_001153	mmu:22644	zf-C2H2
KQRI(proline-rich coiled-coil 2A)			PF07001.16	GO:000573	cytoplasmic	NP_064411	mmu:53761	
nterferon (alpha, beta and omega) r			PF02597.26	GO:000677	cytoplasmic	NP_001413178.1(molybdopterin syr		
K01793	GLCE	mmu00534(	PF06662.19	GO:000828	Golgi appa	NP_001355	mmu:93683	
K02946	RP-S10, MR	mmu03010(	PF00338.27	GO:001593	cytoplasmic	NP_898909	mmu:64657	
K01279	TPP1, CLN	mmu04142(	PF09286.16	GO:000425	lysosomal	NP_034036	mmu:12751	
K25349	AFM		PF00273.25	GO:000810	extracellula	NP_660128	mmu:280662	
.G38(Mesoderm specific transcript l			PF00561.26	GO:000578	ER	NP_001239	mmu:17294	
K11490	NCAPH2		PF16858.11	GO:003304	nuclear	NP_001108	mmu:52683	
K08446	ADGRE5, CD97		PF00002.29	GO:000548	plasma mer	NP_036055	mmu:26364	
K17553	PPP1R11		PF07491.16	GO:005124	cytoplasmic	EDL23313.	mmu:76497	
K08657	TASP1		PF01112.23	GO:000417	nuclear	NP_001153	mmu:75812	
K07190	PHKA_B	mmu04922(	PF00723.27	GO:199023	mitochondr	NP_001171	mmu:110094	
K07203	MTOR, FR	mmu05022(	PF02259.29	GO:000548	mitochondr	EDL14819.	mmu:56717	
K09582	PDIA4, ER	mmu04141(	PF00085.26	GO:000645	ER	NP_033917	mmu:12304	
K08683	HSD17B10	mmu00280(	PF00106.31	GO:003444	mitochondr	NP_058043	mmu:15108	
K08614	ADAM28		PF01421.25	GO:000417	extracellula	NP_034212	mmu:13522	
K19009	SIK3		PF00069.31	GO:003510	cytoplasmic	XP_006510	mmu:70661	
KPZN(SEC14 and spectrin domains			PF24915.1(S	GO:007027	cytoplasmic	NP_780674	mmu:228071	
K00382	DLD, lpd, p	mmu01240(	PF07992.20	GO:005128	mitochondr	NP_031887	mmu:13382	
K12459	SH2B1_3	mmu04722(	PF08916.16	GO:001922	cytoplasmic	BAE43063.	mmu:16923	
K19467	MATN		PF00092.34	GO:000367	extracellula	NP_001345	mmu:17181	
K14530	RPP40	mmu03008(	PF08584.16	GO:003320	cytoplasmic	NP_001413	mmu:208366	
K00904	DGUOK	mmu01100(	PF01712.25	GO:000921	cytoplasmic	EDK99085.	mmu:27369	
K01619	deoC, DER	mmu00030(	PF01791.15	GO:000413	cytoplasmic	BAE40164.	mmu:232449	
K11547	NDC80, HI	mmu04110(	PF24487.1(I	GO:004858	cytoplasmic	NP_075783	mmu:67052	
K15686	MEX3, RKHD		PF00013.35	GO:003577	cytoplasmic	NP_001025	mmu:72640	
K25931	NKAP		PF06047.16	GO:008009	nuclear	NP_080213	mmu:67050	
K21971	NSUN6		PF01189.23	GO:000817	cytoplasmic	EDL08077.	mmu:74455	
K10472	KBTBD4		PF00651.37(BTB:BTB/I		cytoplasmic	NP_001298	mmu:67136	ZBTB
K25058	CACTIN		PF10312.14	GO:001922	nuclear	EDL31428.	mmu:70312	
K06719	CD300		PF07686.23	GO:004592	extracellula	NP_739564	mmu:217303	
K09326	OTX1	mmu04550(	PF00046.35	GO:004322	nuclear	VCW79133.	mmu:18423	Homeobox
K02917	RP-L35Ae,	mmu05171(	PF01247.24	GO:001593	cytoplasmic	NP_001123	mmu:57808	
K07944	ARL3		PF00025.27	GO:000810	cytoplasmic	NP_062692	mmu:56350	
K23368	TRIP11			GO:004885	cytoplasmic	NP_082722	mmu:109181	
K09490	HSPA5, BII	mmu05020(	PF00012.26	GO:001989	ER	NP_001156	mmu:14828	
K06082	FARP2, FR	mmu04520(	PF09380.16	GO:000585	cytoplasmic	ERE82032.	mmu:227377	

K27382	CKM	mmu00330(	PF00217.24	GO:000659	cytoplasmic	NP_031736	mmu:12715
K17682	MNF1		PF20180.4(	GO:009008	mitochondr	NP_001351	mmu:67267
K01080	PLPP1_2_3	mmu05231(	PF01569.27	GO:009859	plasma mer	NP_032929	mmu:19012
K14834	NOC3		PF03914.23	GO:003015	nuclear	NP_067290	mmu:57753
K11986	USP3		PF00443.35	GO:003577	cytoplasmic	NP_659186	mmu:235441
KPV4(nuclear pore complex protein)				GO:000548	cytoplasmic	EDL06441.	mmu:228869
K06268	PPP3R, CN	mmu05020(	PF13499.12	GO:005116	cytoplasmic	NP_000936	mmu:19058
K24984	GRSF1		PF00076.28	GO:000989	mitochondr	NP_848815	mmu:231413
K22649	BCL9, BCL9L		PF11502.13	GO:008009	nuclear	NP_001408	mmu:80288
K11445	KDM7A, JHDM1D		PF17811.6(	GO:004239	nuclear	NP_001028	mmu:338523
K14139	IRGM, LR(	mmu04621(	PF05049.19	GO:000810	cytoplasmic	EDL07757.	mmu:54396
K24778	ZFYVE19, ANCHR		PF01363.27	GO:000548	cytoplasmic	NP_001158	mmu:72008
KVKN(Abelson helper integration s			PF00400.38	GO:000810	cytoplasmic	AAH55400.	mmu:52906
K18384	L3HYPDH	mmu00330(	PF05544.16	GO:001685	cytoplasmic	NP_080314	mmu:67217
K20656	TMEM189	mmu00565(	PF10520.15	GO:000663	peroxisoma	XP_060140	mmu:407243
K10976	ERO1LB	mmu04141(	PF04137.20	GO:000645	ER	AAI11901.1	mmu:67475
K17498	SPN1, IWS1		PF08711.16	GO:001593	nuclear	XP_006526	mmu:73473
K19589	N6AMT1			GO:000647	nuclear	BAB28948.2	mmu:67768
K22262	WDFY3, A	mmu04140(	PF02138.23	GO:000563	cytoplasmic	XP_006535	mmu:72145
K00025	MDH1	mmu01100(	PF02866.23	GO:000548	cytoplasmic	NP_032644	mmu:17449
Cytoskeleton associated protein)			PF13855.12	GO:004858	cytoplasmic	NP_001074	mmu:52065
K20734	SERPINA7	mmu04918(	PF00079.25	GO:005138	extracellulo	NP_001369	mmu:331535
K05699	ACTN1_4	mmu04530(	PF00435.27	GO:003243	cytoplasmic	NP_113863	mmu:60595
8J4(placenta-specific 9)			PF15205.11	GO:000557	extracellulo	NP_997112	mmu:211623
K13699	ABHD5, C(	mmu04923(	PF00561.26	GO:008009	cytoplasmic	NP_080455	mmu:67469
K06158	ABCF3		PF00005.33	GO:003563	cytoplasmic	NP_038880	mmu:27406
K02318	COASY	mmu01240(	PF01121.26	GO:001677	ER	NP_001292	mmu:71743
K24997	GDAP2		PF13716.11	GO:190170	cytoplasmic	NP_001397	mmu:14547
K02295	CRY	mmu04710(	PF03441.19	GO:003292	nuclear	NP_031797	mmu:12952
K16847	TIAM2		PF23014.2(	GO:001097	cytoplasmic	SPT35807.1	mmu:24001
CHF(myeloid lymphoid or mixed-li			PF15017.11	GO:004239	cytoplasmic	NP_001398	mmu:56772
FCX(Actin-related protein)			PF13516.12	GO:000988	extracellulo	NP_001345	mmu:223664
K10350	ZYG11		PF22964.2(	GO:000989	cytoplasmic	NP_001028	mmu:414872
K14572	MDN1, RE	mmu03008(	PF07728.20	GO:005116	nuclear	XP_006537	mmu:100019
K11540	CAD	mmu01240(	PF02786.22	GO:000759	cytoplasmic	NP_076014	mmu:69719
K15187	MLLT1_3,	mmu03250(	PF03366.22	GO:000802	nuclear	AAN03013.	mmu:64144
K12623	LSM4	mmu03018(	PF01423.28	GO:003577	cytoplasmic	NP_001099	mmu:50783
K21435	ANKRD6		PF00023.36	GO:000810	cytoplasmic	XP_036019	mmu:140577
K17421	MRPL40		PF09812.14	GO:001593	nuclear	XP_036015	mmu:18100
K08726	EPHX2	mmu05208(	PF00561.26	GO:001564	peroxisoma	BAE25172.	mmu:13850
K08120	ASPN		PF13855.12	GO:003028	cytoplasmic	NP_001165	mmu:66695
K02607	ORC5	mmu04110(	PF14630.12	GO:008009	cytoplasmic	NP_036089	mmu:26429
K05751	ABI2	mmu04810(	PF07815.19	GO:001712	cytoplasmic	NP_001391	mmu:329165

K07767	KATNA1		PF00004.35	GO:004280	cytoplasmic	EDL05851.	mmu:231912	
K04530	APBB2, FE65L		PF00640.29	GO:000578	cytoplasmic	NP_001297	mmu:11787	
K10417	DYNC2LI	mmu04962(	PF05783.17	GO:002260	cytoplasmic	NP_758460	mmu:213575	
K11809	PGM2L1	mmu00500(	PF02878.22	GO:004322	cytoplasmic	NP_081905	mmu:70974	
K15108	SLC25A19, DNC, TPC1		PF00153.32	GO:001602	cytoplasmic	AAH46767.	mmu:67283	
K17751	MYH6_7	mmu04820(	PF00063.27	GO:003243	cytoplasmic	AAI58071.1	mmu:140781	
K15698	RNF121_175		PF13639.12	GO:003650	cytoplasmic	NP_083487	mmu:75212	
K21437	ANKRD13		PF11904.13	GO:014003	cytoplasmic	BAE33748.	mmu:68420	
K22928	DNPH1		PF05014.21	GO:004643	cytoplasmic	NP_997044	mmu:381101	
K23792	MPRIP		PF00169.35	GO:003243	nuclear	NP_036157	mmu:26936	
K21072	ARHGEF2	mmu05135(	PF17838.7(I	GO:003003	cytoplasmic	NP_001395	mmu:110596	
K01350	PRTN3		PF00089.32	GO:000425	plasma mer	NP_035308	mmu:19152	
JKV(Discs, large (Drosophila) hom			PF03359.18	GO:003042	cytoplasmic	NP_666240	mmu:228836	
K09448	TEAD	mmu04390(	PF17725.7(	GO:008009	nuclear	BAA13519.	mmu:21679	TEA
K17914	KIF13	mmu04814(	PF00225.29	GO:001707	cytoplasmic	XP_006518	mmu:16554	
K01392	THOP1	mmu04614(	PF01432.26	GO:000417	cytoplasmic	NP_073144	mmu:50492	
K06058	DTX	mmu04330(	PF18102.6(I	GO:000548	cytoplasmic	NP_076231	mmu:74198	
K04362	FGFR1, CD	mmu05230(	PF07714.23	GO:001922	extracellulo	NP_034336	mmu:14182	
K04961	RYR1	mmu05020(	PF21119.3(I	GO:000526	cytoplasmic	NP_033135	mmu:20190	
K01530	E7.6.2.1		PF16212.11	GO:001602	cytoplasmic	NP_056620	mmu:50771	
K05544	DUS3		PF01207.24	GO:000294	cytoplasmic	NP_659107	mmu:224907	
K03015	RPB7, POL	mmu03420(	PF00575.29	GO:003577	cytoplasmic	NP_001015	mmu:67710	
K17408	DAP3, MRPS29		PF10236.15	GO:001593	mitochondr	NP_075370	mmu:65111	
K24505	GMEB		PF01342.26	GO:000097	cytoplasmic	NP_001116	mmu:56809	SAND
K08171	SLC37A4	mmu04973(	PF07690.21	GO:001560	ER	NP_001344	mmu:14385	
K13352	PEX11B	mmu04146(	PF05648.19	GO:000548	peroxisoma	NP_001155	mmu:18632	
K22557	COMMD1, MURR1		PF17221.8(	GO:003315	cytoplasmic	NP_653097	mmu:17846	
K17815	EXO5		PF09810.15	GO:001679	nuclear	NP_001153	mmu:73172	
K27751	ACBD6		PF00887.24	GO:000573	cytoplasmic	NP_082526	mmu:72482	
K17817	NUDT18, MTH3		PF00293.34	GO:000918	cytoplasmic	NP_001347	mmu:213484	
K25468	SCGB2B		PF09252.15	GO:000557	extracellulo	NP_840093	mmu:110187	
K23561	CLK1_4	mmu05134(	PF00069.31	GO:008009	nuclear	NP_001036	mmu:12747	
K08638	CPXM1		PF00246.29	GO:000823	ER	NP_062670	mmu:56264	
K06580	SLC42A, RHAG, RHBC		PF00909.26	GO:003537	Golgi appa	XP_006523	mmu:19743	
K15713	UHRF2, NIRF		PF12148.14	GO:004239	nuclear	NP_659122	mmu:109113	
K19326	SYNE1	mmu04820(	PF00435.27	GO:006049	cytoplasmic	AAI11035.1	mmu:64009	
K06098	TJP2, ZO2	mmu04530(	PF00595.29	GO:190560	nuclear	NP_001363	mmu:21873	
K14358	SCRN		PF03577.20	GO:001602	cytoplasmic	NP_001404	mmu:69938	
K11343	MRGBP	mmu03082(	PF07904.18	GO:003124	nuclear	AAI10994.1	mmu:73247	
lual adaptor of phosphotyrosine an			PF13855.12	GO:001602	cytoplasmic	BAE41014.	mmu:98238	
K10615	HERC4	mmu04120(	PF00415.24	GO:000858	cytoplasmic	NP_084390	mmu:67345	
K22503	DARS1	mmu00970(	PF00152.25	GO:001710	cytoplasmic	BAE41011.	mmu:226414	
K11790	DTL, CDT2, DCAF2		PF00400.38	GO:008009	mitochondr	NP_084042	mmu:76843	

K12259	SMOX, PA(	mmu00410(	PF01593.30	GO:000573'	cytoplasmic	NP_001171	mmu:228608	
KQAV(ig heavy chain V-III region V			PF05761.20	GO:004316'	cytoplasmic	NP_795942	mmu:319638	
K00798	MMAB, pd	mmu01240(	PF01923.23	GO:003563'	mitochondr	NP_084232	mmu:77697	
K26583	TRERF1		PF13912.12	GO:000989	nuclear	EDL23550.	mmu:22482	zf-C2H2
K12606	RCD1, CN(	mmu03018(	PF04078.19	GO:001922	cytoplasmic	NP_001006	mmu:58184	
K07375	TUBB	mmu05020(	PF00091.31	GO:009951	cytoplasmic	XP_030616	mmu:22152	
K24023	RALGPS		PF00617.24	GO:000573'	cytoplasmic	NP_001153	mmu:78255	
K06826	NID	mmu04820(	PF07474.17	GO:009863	lysosomal	NP_035047	mmu:18073	
K14768	UTP7, WDR46		PF08149.16	GO:003204	nuclear	NP_065628	mmu:57315	
K10891	FANCD2	mmu03460(	PF14631.11	GO:004858	nuclear	BAE33341.	mmu:211651	
K03113	EIF1, SUI1		PF01253.27	GO:000374	cytoplasmic	NP_001001	mmu:68969	
K18183	COX19	mmu04714(	PF06747.18	GO:003361'	cytoplasmic	NP_932097	mmu:68033	
K18163	NDUFAF6	mmu04714(	PF00494.24	GO:002260'	mitochondr	NP_001078	mmu:76947	
K08015	RASSF5, R/	mmu04670(	PF16517.10	GO:000548	cytoplasmic	NP_061220	mmu:54354	
K16461	CEP135, CEP4			GO:002260'	cytoplasmic	EDL37906.	mmu:381644	
K14404	CPSF4, YTI	mmu05164(	PF00642.30	GO:009030	cytoplasmic	XP_042542'	mmu:54188	
K14306	NUP62, NS	mmu03013(	PF05064.18	GO:003288	cytoplasmic	NP_444304	mmu:18226	
K00364	guaC, GMF	mmu01100(	PF00478.31	GO:004643	cytoplasmic	NP_818773	mmu:105446	
K24746	WDR47		PF00400.38	GO:003111	cytoplasmic	NP_852065	mmu:99512	
K14825	TMED5, ERP2, ERP4		PF01105.29	GO:003802	ER	BAC31408.	mmu:73130	
K04404	MAP3K7IP	mmu04010(	PF02845.22	GO:001922	cytoplasmic	NP_001346	mmu:68652	
K06275	PARV	mmu04510(	PF00307.36	GO:002260	cytoplasmic	NP_573395	mmu:170736	
K26150	UNC93B		PF05978.22	GO:000810	cytoplasmic	EDL32974.	mmu:54445	
RAP(chromosome 6 open reading f			PF10184.16	GO:004323	peroxisoma	AAI17036.1	mmu:69662	
K15105	SLC25A12_13, AGC		PF00153.32	GO:000916	cytoplasmic	NP_001171	mmu:50799	
K04176	CCR1, CD1	mmu04062(	PF00001.26	GO:001922	plasma mer	NP_034042	mmu:12768	
K09648	IMP2	mmu03060(Protein exp		GO:004323	mitochondr	XP_036013	mmu:93757	
K01512	acyP	mmu00620(	PF00708.24	GO:001678'	cytoplasmic	NP_083620	mmu:75572	
K24013	TMEM38, TRIC		PF05197.18	GO:000526'	ER	NP_082329	mmu:52076	
K22755	UFL1		PF09743.14	GO:000578	nuclear	NP_001342	mmu:67490	
K19952	RABIF, MSS4, DSS4		PF04421.21	GO:000810	cytoplasmic	NP_663485	mmu:98710	
K18441	CYTH	mmu04144(	PF01369.25	GO:005105	cytoplasmic	NP_001365	mmu:19157	
K24427	SPON1		PF06468.19	GO:000989	plasma mer	BAD32299.	mmu:233744	
K19827	MACF1		PF00435.27(Spectrin:Sp		cytoplasmic	NP_001388	mmu:11426	
K25209	CASZ1			GO:008009	nuclear	XP_006539	mmu:69743	TF_others
K06834	ADAM9	mmu04148(	PF01421.25	GO:005104	plasma mer	NP_001257	mmu:11502	
K14293	KPNB1, IP	mmu03013(	PF13513.12	GO:005116	cytoplasmic	NP_032405	mmu:16211	
K25403	RILPL2		PF11461.13	GO:004299	cytoplasmic	NP_084535	mmu:80291	
K25048	TMEM106B		PF07092.18	GO:003134	cytoplasmic	NP_082268	mmu:71900	
K09597	SPPL2B		PF04258.18	GO:000417	cytoplasmic	XP_011241	mmu:73218	
K08856	STK16		PF00069.31	GO:008009	cytoplasmic	NP_001264	mmu:20872	
K15305	VAC14, TA	mmu05203(	PF11916.13	GO:000548	cytoplasmic	NP_666328	mmu:234729	
K23638	SLPI, WFDC4		PF00095.26	GO:003041	cytoplasmic	AAC53394.	mmu:20568	

K00720	UGCG	mmu00600(	PF13506.12	GO:003250	Golgi appa	NP_035803	mmu:22234	
K27588	RHBDF	mmu04668(	PF12595.13	GO:000810	cytoplasmic	NP_001161	mmu:217344	
20B5(MIS18 binding protein 1)			PF09133.15	GO:000557	cytoplasmic	NP_766166	mmu:21765	MYB
K24228	WDR66, CFAP251		PF00400.38(WD40:WD		cytoplasmic	NP_001357	mmu:269701	
K07190	PHKA_B	mmu04922(	PF00723.27	GO:199023	cytoplasmic	NP_001390	mmu:102093	
K20102	YTHDF		PF04146.21	GO:199024	cytoplasmic	AAH52631.	mmu:229096	
K13162	PCBP2	mmu04216(	PF00013.35	GO:008009	cytoplasmic	XP_006981	mmu:18521	
K08757	APOA1	mmu04975(	PF01442.23	GO:003435	extracellulo	NP_033822	mmu:11806	
K12486	SMAP	mmu04144(	PF01412.23	GO:000573	cytoplasmic	NP_598477	mmu:69780	
K11259	ILVBL, HACL2		PF02776.23	GO:003444	ER	XP_030100	mmu:216136	
K04515	CAMK2	mmu04750(	PF00069.31	GO:003563	cytoplasmic	NP_001308	mmu:108058	
K14780	DHX37, DHR1		PF23362.1(I	GO:200024	nuclear	NP_976064	mmu:208144	
K01870	IARS, ileS	mmu00970(	PF00133.28	GO:001710	cytoplasmic	NP_742012	mmu:105148	
K24794	DCBLD		PF00431.26	GO:003802	Golgi appa	NP_079981	mmu:66686	
K11313	SUPT3H, Sl	mmu05202(	PF02269.22	GO:003124	nuclear	BAC40048.	mmu:109115	
K08112	GPC6		PF01153.25	GO:006034	extracellulo	BAE37868.	mmu:23888	
K25092	SAFB		PF00076.28	GO:000367	nuclear	NP_001156	mmu:224903	
K26266	SAMD1		PF21524.3(S	GO:003444	nuclear	NP_001074	mmu:666704	
K09540	SEC63, DN	mmu04141(	PF02889.21	GO:000810	Golgi appa	NP_694695	mmu:140740	
K24942	TTC38				cytoplasmic	NP_001028	mmu:239570	
K12579	ISG20		PF00929.31	GO:003577	nuclear	EDL07082.	mmu:57444	
K05864	PPID, CYP	mmu04217(	PF00160.26	GO:000962	cytoplasmic	NP_080628	mmu:67738	
K19613	SHOC2, SU	mmu04014(	PF13855.12	GO:004858	cytoplasmic	NP_001161	mmu:56392	
K10473	KBTBD5_10		PF01344.31	GO:004212	cytoplasmic	NP_001074	mmu:22800	ZBTB
K25177	ZCCHC4		PF10237.15	GO:003116	nuclear	EDL37665.	mmu:78796	
K05701	TJP1, ZO1	mmu04530(	PF00595.29	GO:190560	cytoplasmic	NP_001157	mmu:21872	
K16748	BBS5		PF07289.17	GO:009859	cytoplasmic	XP_008836	mmu:72569	
K13023	CPN2		PF13855.12	GO:001602	extracellulo	NP_082180	mmu:71756	
K17307	FBLN1_2	mmu04820(	PF07645.21	GO:000810	extracellulo	NP_034310	mmu:14114	
K00511	SQLE, ERG	mmu00100(	PF08491.16	GO:000548	ER	NP_033296	mmu:20775	
SA8(chromosome 7 open reading frame 55)				GO:003361	mitochondr	NP_079639	mmu:66117	
K11846	USP18_41		PF00443.35	GO:003210	nuclear	CAJ18436.1	mmu:24110	
K21872	PPHLN1		PF11488.13	GO:000810	cytoplasmic	NP_001403	mmu:223828	
25KF(Immunity-related GTPase fan			PF05049.19	GO:004858	cytoplasmic	NP_001128	mmu:631323	
K14358	SCRN		PF03577.20	GO:004423	cytoplasmic	NP_666139	mmu:217140	
K00490	CYP4F		PF00067.27	GO:006223	ER	NP_001333	mmu:106648	
V15(Mitochondrial inner membran			PF15386.11	GO:000565	cytoplasmic	NP_001403	mmu:233895	
Z4ZI(Zinc finger CCCH-type with C			PF01585.28	GO:005134	nuclear	EDL07411.	mmu:22900	TF_others
K09521	DNAJC1	mmu04141(	PF00226.36	GO:005108	ER	NP_001177	mmu:13418	MYB
K09493	CCT1, TCP1		PF00118.29	GO:000024	cytoplasmic	BAO48049.	mmu:21454	
K17681	ATAD3A_B		PF12037.13	GO:000961	cytoplasmic	NP_849534	mmu:108888	
XXDM(transmembrane protein 109)			PF14965.11	GO:001602	ER	BAE29844.	mmu:68539	
K17606	IGBP1, TA	mmu04140(	PF04177.18	GO:200123	cytoplasmic	NP_032810	mmu:18518	

K21853	DOCK9_10_11		PF06920.19	GO:003250	cytoplasmic	XP_006541	mmu:75974	
23RR(transmembrane protein 201)			PF10476.14	GO:000701	extracellulo	NP_001271	mmu:230917	
K15146	MED4	mmu04919(	PF10018.14	GO:006026	nuclear	NP_080395	mmu:67381	
Catalyzes the methyl esterification o			PF01135.25	GO:000827	cytoplasmic	NP_705822	mmu:245867	
K20280	TRAPPC5, TRS31		PF04051.21	GO:004820	mitochondr	NP_001102	mmu:66682	
K03115	CSNK2B	mmu04137(	PF01214.23	GO:001920	cytoplasmic	CCI79672.1	mmu:13001	
K10407	KLC	mmu05020(	PF13424.12	GO:001989	cytoplasmic	XP_029335	mmu:232943	
K18179	COA6	mmu04714(	PF02297.22	GO:002260	cytoplasmic	NP_778152	mmu:67892	
K25176	ZNF451		PF18479.7(I	GO:008009	cytoplasmic	AAH62154.	mmu:98403	
K08342	ATG4	mmu04140(	PF03416.26	GO:003472	cytoplasmic	EDL07595.	mmu:666468	
K25779	HSD17B11	mmu00140(	PF00106.31	GO:000578	ER	NP_444492	mmu:114664	
K15266	TFB1M		PF00398.26	GO:003088	mitochondr	NP_666186	mmu:22448	TF_others
Y9WN(Spindlin family, member 4)			PF07139.16	GO:000573	cytoplasmic	NP_001405	mmu:72572	
K23835	TIFB		PF00498.31	GO:001922	cytoplasmic	NP_001162	mmu:212937	
K15175	CDC73		PF16050.10	GO:008009	nuclear	NP_001074	mmu:214498	
K12357	NEU2_3_4	mmu00600(	PF13088.12	GO:190350	cytoplasmic	EDL40157.	mmu:23956	
K19365	BSCL2		PF06775.19	GO:004544	ER	NP_001129	mmu:14705	
K25536	FGL1		PF00147.23	GO:200123	extracellulo	NP_663569	mmu:234199	
K23093	USB1		PF09749.15	GO:001679	nuclear	NP_598715	mmu:101985	
IYE0(immunoglobulin heavy variab			PF07686.23	GO:000548	cytoplasmic	AGN33424.1(immmunoglobulin he		
K13144	INTS7		PF24436.1(I	GO:000931	nuclear	NP_848747	mmu:77065	
K04382	PPP2C	mmu05165(	PF00149.33	GO:008009	cytoplasmic	NP_001009	mmu:19053	
K23299	WDFY1		PF00400.38	GO:000554	cytoplasmic	NP_001104	mmu:69368	
K20818	KXD1, BORCS4		PF00240.29	GO:007238	cytoplasmic	XP_049983	mmu:75620	
K18080	TNS		PF10409.15	GO:002260	cytoplasmic	NP_001276	mmu:21961	
K13304	SGK3	mmu04151(	PF00069.31	GO:200123	cytoplasmic	EDL14293.	mmu:170755	
XR08(kiaa1551)			PF02020.24	GO:000573	mitochondr	XP_006245	mmu:66882	
K27755	FASTKD3		PF08368.17	GO:000548	mitochondr	XP_006517	mmu:69577	
K00763	pncB, NAP	mmu01240(	PF17767.7(	GO:000548	cytoplasmic	NP_766195	mmu:223646	
K05853	ATP2A	mmu04972(	PF00122.25	GO:000578	ER	NP_001156	mmu:53313	
Specifically methylates the adenine i			PF14572.12	GO:000815	cytoplasmic	NP_001157	mmu:212627	
K12848	SNU23	mmu03040(	PF12874.13	GO:000548	nuclear	NP_001073	mmu:66492	
K13443	NPC2	mmu04979(	PF02221.20	GO:004323	extracellulo	BAE32866.	mmu:67963	
K15636	PGM5		PF24947.1(I	GO:003243	cytoplasmic	NP_778178	mmu:226041	
K24512	VWA8		PF07728.20	GO:003563	mitochondr	NP_082182	mmu:219189	
K00214	BLVRA, bv	mmu00860(	PF09166.15	GO:000677	cytoplasmic	NP_080954	mmu:109778	
K01469	OPLAH, O	mmu00480(	PF02538.19	GO:003563	cytoplasmic	XP_006521	mmu:75475	
K26096	SKA3			GO:003111	cytoplasmic	NP_941007	mmu:219114	
K08115	CSPG4		PF16184.11	GO:001922	plasma mer	NP_620570	mmu:121021	
K20392	PRAF2		PF03208.24	GO:001250	plasma mer	NP_613068	mmu:54637	
K06722	CD302		PF00059.27	GO:000989	Golgi appa	NP_001277	mmu:66205	
K24347	UBXN2B		PF08059.18	GO:000548	cytoplasmic	NP_080810	mmu:68053	
K08810	TRIO		PF16609.10	GO:005080	cytoplasmic	Q0KL02.3(I	mmu:223435	

K08501	STX8	mmu04130(	PF05739.25	GO:000810	cytoplasmic	NP_061238	mmu:55943	
K00639	kbl, GCAT	mmu00260(	PF00155.27	GO:003017	mitochondr	NP_038875	mmu:26912	
K12491	AGAP	mmu04144(	PF01412.23	GO:005116	cytoplasmic	NP_631892	mmu:347722	
K10367	FMN1		PF02181.28	GO:003510	cytoplasmic	XP_006498	mmu:14260	
K14569	BMS1	mmu03008(	PF04950.18	GO:003204	nuclear	NP_919320	mmu:213895	
K12046	TNNT3	mmu04814(	PF00992.25	GO:003103	cytoplasmic	XP_028642	mmu:21957	
K06085	SSX2IP, AD	mmu04520(	PF11559.13	GO:002260	cytoplasmic	NP_001240	mmu:99167	
54J(major urinary protein)			PF00061.29	GO:009008	extracellula	NP_001012	mmu:381530	
K14725	SLC9A9, NHE9		PF00999.27	GO:007184	cytoplasmic	AAI53826.1	mmu:331004	
K24677	IQCE	mmu04340(	PF00612.33	GO:009859	cytoplasmic	NP_083109	mmu:74239	
K23460	CHM, CHML		PF00996.24	GO:004323	cytoplasmic	NP_067325	mmu:12663	
XUVY(Cross-linked envelope protei			PF02389.20	GO:000573	cytoplasmic	XP_017175	mmu:20754	
K24627	GAS2, GAS2L		PF02187.23	GO:009951	cytoplasmic	NP_001028	mmu:237436	
K25856	ARID3		PF01388.27	GO:005125	nuclear	NP_001275	mmu:13496	ARID
K04521	BACE1	mmu05010(	PF00026.29	GO:000578	lysosomal	NP_035922	mmu:23821	
K06721	CLEC10A, CLECSF13,		PF03954.19	GO:009770	plasma mer	NP_034926	mmu:17312	
K06875	PDCD5, TFAR19		PF01984.25	GO:003315	cytoplasmic	NP_062720	mmu:56330	
K06724	NRP1, CD3	mmu05171(	PF00431.26	GO:004867	ER	NP_032763	mmu:18186	
K10699	UBE1L2, U	mmu04120(	PF10585.14	GO:003563	cytoplasmic	NP_766300	mmu:231380	
K20132	APPL2, DIP13B		PF16746.10	GO:005116	cytoplasmic	BAE42499.1	mmu:216190	
K16943	SEPT4	mmu04215(	PF15548.11	(DUF4655:D	nuclear	NP_001411	mmu:18952	
K05764	TMSB4	mmu04810(	PF01290.25	GO:001922	cytoplasmic	NP_001001	mmu:19241	
K01280	TPP2		PF12580.14	GO:000417	cytoplasmic	BAE42503.1	mmu:22019	
K13761	PDE9	mmu00230(	PF00233.25	GO:000921	cytoplasmic	NP_001397	mmu:18585	
K24891	PKNOX		PF16493.11	GO:004864	nuclear	NP_001398	mmu:18771	Homeobox
K06822	PLXND		PF08337.17	GO:003042	ER	NP_080652	mmu:67784	
K24274	FAM111A		PF13365.12	GO:000626	cytoplasmic	NP_001333	mmu:107373	
K12489	ACAP	mmu04144(	PF16746.10	GO:001250	cytoplasmic	BAE27959.1	mmu:140500	
K14139	IRGM, LRG	mmu04621(	PF05049.19	GO:000810	cytoplasmic	BAC87666.1	mmu:54396	
20YJ(Chromosome 1 open reading fr			PF15375.11	GO:003204	nuclear	NP_079891	mmu:66523	
K05625	TGM2	mmu05016(	PF00927.28	GO:003514	cytoplasmic	NP_033399	mmu:21817	
K09579	PIN4		PF13616.12	GO:009030	nuclear	NP_001388	mmu:69713	
K00849	galK	mmu00052(	PF10509.15	GO:004643	cytoplasmic	BAB26864.1	mmu:14635	
K18196	STIM2	mmu04020(	PF16533.10	GO:000526	cytoplasmic	BAD32461.	mmu:116873	
K09850	RASSF1	mmu05219(	PF16517.10	GO:190187	cytoplasmic	NP_001230	mmu:56289	
K17823	DCUN1D3		PF03556.21	GO:008009	cytoplasmic	NP_001157	mmu:233805	
K16569	TUBGCP2, GCP2		PF04130.18	GO:000705	cytoplasmic	NP_001272	mmu:74237	
K11591	MBD3	mmu03082(	PF14048.11	GO:200073	nuclear	NP_038623	mmu:17192	MBD
K11588	MECP2		PF01429.25	GO:005089	nuclear	NP_034918	mmu:17257	MBD
K16332	RBL2	mmu05165(	PF01858.22	GO:000367	nuclear	NP_001268	mmu:19651	
K18081	MTMR1_2	mmu04070(	PF06602.19	GO:005080	cytoplasmic	NP_076347	mmu:77116	
K01412	PMPCA, MAS2		PF00675.25	GO:000417	mitochondr	BAE34758.1	mmu:66865	
K20069	NECAP1_2		PF07933.20	GO:000810	cytoplasmic	BAE33757.1	mmu:66147	

K06846	LY6D_E_F_G6_H		PF00087.27	GO:003054	extracellula	AAH37143.	mmu:17069	
K16732	PRC1, ASE1, MAP65		PF03999.17	GO:000828	cytoplasmic	NP_001361	mmu:233406	
K05546	GANAB	mmu04141(	PF01055.32	GO:000578	Golgi appa	NP_032086	mmu:14376	
K24141	STARD7		PF01852.25	GO:004885	cytoplasmic	BAE25505.1	mmu:99138	
K20234	DOK2		PF02174.23	GO:003015	cytoplasmic	NP_034201	mmu:13449	
K15436	TRPO3, MT	mmu03013(	PF24139.1(	GO:003450	cytoplasmic	AAH75678.	mmu:320938	
K12482	RUFY1_2, I	mmu04144(	PF02759.24	GO:000573	cytoplasmic	BAD32473.	mmu:70432	
K25401	LRRC45		PF13516.12	GO:000573	cytoplasmic	NP_705773	mmu:217366	
K04441	P38	mmu05208(	PF00069.31	GO:001922	cytoplasmic	AAH57211.	mmu:19094	
K11112	TINF2, TIN2		PF14973.11	GO:003304	cytoplasmic	NP_001391	mmu:28113	
GI6(RNA binding motif protein)			PF00076.28	GO:008009	nuclear	NP_001239	mmu:19656	
K06509	KAI1, CD8	mmu04115(	PF00335.26	GO:000557	plasma mer	NP_001129	mmu:12521	
K10103	FBXO44		PF04300.19	GO:199023	lysosomal	NP_001155	mmu:230903	
K01325	KLK1_2	mmu04614(	PF00089.32	GO:000425	lysosomal	SFW93232.1	mmu:16624	
K10258	TER, TSC1	mmu01040(	PF02544.21	GO:000663	Golgi appa	BAE42630.1	mmu:106529	
K27587	JPT, HN		PF17054.10	GO:004858	cytoplasmic	BAE39348.1	mmu:52009	
K15107	SLC25A18_22, GC		PF00153.32	GO:009008	cytoplasmic	NP_001171	mmu:68267	
K00622	nat	mmu00232(	PF00797.23	GO:004856	cytoplasmic	NP_001162	mmu:17961	
K00787	FDPS	mmu00900(	PF00348.22	GO:008009	cytoplasmic	BAE24508.1	mmu:110196	
K15076	ELOA, TCEB3		PF06881.16	GO:000802	nuclear	NP_038764	mmu:27224	
K06559	LY75, CD205		PF00059.27	GO:000989	lysosomal	NP_038853	mmu:17076	
K06521	SEMA4, CD	mmu04360(	PF01403.25	GO:003514	lysosomal	EDL14495.1	mmu:20353	
K11296	HMGB3		PF09011.16(HMG_box_		nuclear	XP_029329	mmu:15354	HMG
K10295	FBXO9		PF19270.4(I	GO:199023	cytoplasmic	NP_001074	mmu:71538	
K03948	NDUFA4	mmu05208(	PF06522.17	GO:000575	Golgi appa	NP_001092	mmu:407790	
K27289	MIGA, FAM73		PF10265.14	GO:006034	ER	EDL08464.1	mmu:108958	
K06520	CD99, MIC	mmu04670(	PF12301.13	GO:005090	extracellula	EDL26580.1	mmu:171486	
K12826	SF3A2, SAF	mmu03040(	PF16835.10(SF3A2:Pre-		cytoplasmic	XP_021560	mmu:20222	
K03443	PANX1	mmu04148(	PF00876.23	GO:000526	ER	BAE33619.1	mmu:55991	
K16333	CRTC2, TC	mmu04922(	PF12885.12	GO:002260	cytoplasmic	NP_083157	mmu:74343	
K20295	COG8		PF04124.18	GO:000810	cytoplasmic	NP_631975	mmu:97484	
K01887	RARS, argS	mmu00970(	PF00750.25	GO:001602	cytoplasmic	NP_852071	mmu:109093	
K26216	AHSA1, AHA1		PF09229.17	GO:000645	cytoplasmic	BAE38913.1	mmu:217737	
K19530	JPH		PF02493.26	GO:000578	cytoplasmic	NP_065629	mmu:57339	
K01021	TPST		PF13469.12	GO:000814	Golgi appa	NP_001123	mmu:22021	
K11427	DOT1L, D(	mmu05202(	PF08123.19	GO:008009	nuclear	AAQ82850.	mmu:208266	
K25780	KHDC4, BLOM7		PF22675.2(I	GO:002260	mitochondr	NP_083090	mmu:74200	
K15115	SLC25A32, MFT		PF00153.32	GO:001593	mitochondr	BAE43085.1	mmu:69906	
K11449	JMJD1C	mmu05202(	PF22987.3(	GO:000338	cytoplasmic	NP_997104	mmu:108829	
K10991	SWI5, SAE3		PF07061.16	GO:003306	nuclear	XP_021049	mmu:72931	
K14395	ACP6		PF00328.27	GO:001679	mitochondr	NP_062774	mmu:66659	
S1H(CUE domain containing 1)			PF02845.22	GO:000367	cytoplasmic	AAH24476.	mmu:103841	
K23765	S100A11_13, S100C		PF01023.25	GO:004212	extracellula	AQS27595.1	mmu:20196	

K24472	HEG	mmu04520(	PF00008.33	GO:005134	plasma mer	XP_006522	mmu:77446	
K26734	MCC	mmu04310(	PF10506.14	GO:004212	nuclear	NP_001078	mmu:328949	
K11093	SNRP70	mmu03040(	PF12220.14	GO:001921	nuclear	NP_033250	mmu:20637	
K20636	ARHGAP12_27		PF16618.10	GO:001032	cytoplasmic	XP_006526	mmu:75415	
K20282	GCC2	mmu05132(	PF16704.10	GO:003315	cytoplasmic	NP_081651	mmu:70297	
K08133	COL14A	mmu04974(	PF00092.34	GO:004863	extracellulo	EDL29274.	mmu:12818	
YBNZ(Slowmo homolog)			PF04707.20	GO:000686	cytoplasmic	NP_079807	mmu:66390	
K04891	KCND1, KV4.1		PF00520.37	GO:009859	plasma mer	NP_032449	mmu:16506	
K03009	RPABC4, R	mmu03420(	PF03604.18	GO:003088	nuclear	NP_075616	mmu:17749	
K05610	UCHL5, UC	mmu03082(	PF01088.27	GO:000858	nuclear	NP_062508	mmu:56207	
K17306	ARMC1			GO:004322	cytoplasmic	NP_083116	mmu:74252	
K09276	YBX1, NSEP1		PF00313.28	GO:000810	nuclear	NP_035862	mmu:22608	CSD
K05759	PFN	mmu05014(	PF00235.25	GO:003514	cytoplasmic	NP_035202	mmu:18643	
K17337	CRABP1		PF00061.29	GO:000686	cytoplasmic	NP_001099	mmu:12903	
K14779	DDX52, ROK1		PF00270.35	GO:000367	nuclear	BAE39019.	mmu:78394	
K15443	TRM82, WDR4		PF24817.1(	GO:003626	nuclear	NP_067297	mmu:57773	
K21439	ANKRD49		PF12796.13	GO:008009	nuclear	NP_001335	mmu:56503	
K09565	PPIF	mmu05208(	PF00160.26	GO:200123	mitochondr	NP_598845	mmu:105675	
K04591	ADGRE1, EMR1		PF00002.29	GO:000989	plasma mer	BAE33466.	mmu:13733	
K12164	UBA5, UBE1DC1		PF00899.27	GO:004858	cytoplasmic	NP_079968	mmu:66663	
K00621	GNPNAT1	mmu00520(	PF00583.31	GO:000548	cytoplasmic	NP_001404	mmu:54342	
K13201	TIA1, TIAL1		PF00076.28	GO:003577	cytoplasmic	NP_001013	mmu:21843	
K07194	RHOQ, TC	mmu04910(	PF00071.28	GO:004423	cytoplasmic	NP_445974	mmu:104215	
K24518	CRIM1		PF19442.4(	GO:003041	cytoplasmic	NP_056615	mmu:50766	
K02449	CDK10		PF00069.31	GO:004858	cytoplasmic	AAH17131.	mmu:234854	
K13242	NOS3	mmu04915(	PF02898.20	GO:001649	cytoplasmic	NP_032739	mmu:18127	
K05747	WAS	mmu05231(	PF00568.29	GO:009951	cytoplasmic	NP_033541	mmu:22376	
K06261	GP1BA, CD	mmu04512(	PF13855.12	GO:000759	plasma mer	AAC53320.	mmu:14723	
K24790	POSTN		PF02469.27	GO:001922	extracellulo	NP_001355	mmu:50706	
K24956	ZBTB8OS		PF01951.21	GO:007266	cytoplasmic	NP_080246	mmu:67106	
K01835	pgm	mmu00052(	PF24947.1(I	GO:000925	cytoplasmic	AAH86490.	mmu:72157	
K10867	NBN, NBS	mmu03440(	PF08599.15	GO:000810	nuclear	NP_038780	mmu:27354	
K25860	PACC1		PF15122.11	GO:003470	cytoplasmic	BAB28038.1	mmu:66950	
K10268	FBXL2_20		PF12799.13	GO:000989	cytoplasmic	XP_053425	mmu:72194	
K09498	CCT6		PF00118.29	GO:000645	cytoplasmic	NP_033969	mmu:12467	
K10141	SESN1_3	mmu04115(	PF04636.18	GO:001922	cytoplasmic	NP_084537	mmu:75747	
K03026	RPC4, POL	mmu04623(	PF05132.19	GO:001922	nuclear	NP_001157	mmu:67065	
XW1Y(transmembrane protein 69)			PF11911.13	GO:000557	cytoplasmic	NP_808338	mmu:230657	
K17521	CLEC11A		PF00059.27	GO:004212	extracellulo	NP_033157	mmu:20256	
K11167	DHRS7C		PF00106.31	GO:190316	extracellulo	NP_001013	mmu:68460	
K25037	PODN		PF13855.12	GO:000828	extracellulo	NP_001272	mmu:242608	
)0B5(nudix (nucleoside diphosphate			PF01852.25	GO:001591	cytoplasmic	NP_075866	mmu:170460	
K02865	RP-L10Ae,	mmu05171(	PF00687.26	GO:004323	nuclear	P53026.3(R	mmu:19896	

K04357	EGF	mmu05208(	PF00058.23	GO:002192	plasma mer	NP_034243	mmu:13645		
K08790	STK38, NDR		PF00069.31	GO:000989	cytoplasmic	XP_030111	mmu:232533		
K10388	PLEC	mmu04820(	PF00681.25	GO:001922	cytoplasmic	AAR95675.	mmu:18810		
K01527	EGD1, BTF3		PF01849.23	GO:190555	cytoplasmic	NP_001008	mmu:218490		
K10401	KIF18A	mmu04814(	PF00225.29	GO:000810	cytoplasmic	NP_647464	mmu:228421		
K09848	TRAF4	mmu05200(	PF02176.24	GO:001922	cytoplasmic	NP_033449	mmu:22032		
K14816	REI1		PF12756.13	GO:000579	nuclear	BAE32946.	mmu:52521		
K06692	PSMD5		PF10508.15	GO:006500	cytoplasmic	NP_542121	mmu:66998		
)6PI(R3H domain containing 2); EN			PF01424.28	GO:004323	nuclear	XP_006514	mmu:71750		
SQB(TAP binding protein-like)			PF07686.23	GO:002260	ER	NP_663366	mmu:213233		
K17630	RASA4, CA	mmu04014(	PF00168.36	GO:001602	cytoplasmic	NP_598675	mmu:54153		
K10890	FANCC	mmu03460(	PF02106.20	GO:004423	cytoplasmic	EDL16217.	mmu:14088		
K26050	MYOZ	mmu04820(	PF05556.16	GO:008009	cytoplasmic	NP_067483	mmu:59011		
K06554	IGLL1, IGI	mmu05340(	PF07654.21	GO:001972	cytoplasmic	P01843.1(R	mmu:16136		
J99(kiaa1328)			PF15369.11(KIAA1328:		cytoplasmic	NP_001392	mmu:225289		
J4R(Chromosome 5 open reading fr			PF17724.6(DUF5568:Fo		nuclear	NP_851795	mmu:78521		
K03937	NDUFS4	mmu05208(	PF04800.17	GO:001922	mitochondr	NP_035017	mmu:17993		
K13210	FUBP		PF00013.35	GO:000989	cytoplasmic	NP_001277	mmu:320267		
K06823	COL18A	mmu04974(	PF06482.17	GO:000150	extracellulo	XP_006513	mmu:12822		
K07604	KRT1	mmu04915(	PF00038.26	GO:009859	cytoplasmic	NP_032497	mmu:16669		
K22070	FDX1, ADX		PF00111.32	GO:003197	mitochondr	NP_032022	mmu:14148		
K13159	HNRNPL			GO:000548	nuclear	BAE22570.	mmu:72692		
K09391	E2F7_8		PF02319.26	GO:006071	nuclear	NP_001013	mmu:10896	E2F	
K06490	ICAM1, CD	mmu05164(	PF03921.20	GO:200123	plasma mer	BAE40221.	mmu:15894		
K08826	HIPK	mmu04218(	Cellular sen	GO:008009	cytoplasmic	ABB92152.1	mmu:15257		
K00873	PK, pyk	mmu05165(	PF00224.27	GO:004643	cytoplasmic	XP_036010	mmu:18746		
KNU5(malectin)			PF11721.14	GO:000578	ER	NP_780612	mmu:109154		
K10660	5-Mar	mmu04137(	PF12906.13	GO:000827	cytoplasmic	NP_001029	mmu:69104		
Calcium channel, voltage-dependent			PF13465.12	GO:008009	nuclear	XP_030104	mmu:68744	zf-C2H2	
K07752	CPD		PF00246.29	GO:001602	plasma mer	NP_031780	mmu:12874		
K26050	MYOZ	mmu04820(	PF05556.16	GO:003143	cytoplasmic	NP_579941	mmu:170947		
K01689	ENO1_2_3	mmu04820(	PF03952.21	GO:200123	cytoplasmic	NP_001366	mmu:433182		
K16823	TP53BP2, A	mmu04390(	PF21801.3(	GO:190011	cytoplasmic	NP_775554	mmu:209456		
K00922	PIK3CA_B	mmu05208(	PF00454.33	GO:001922	cytoplasmic	NP_032866	mmu:18707		
K18404	TDRD3		PF08585.18	GO:007110	nuclear	NP_766193	mmu:219249		
K14562	TCOF1	mmu03008(	PF03546.19	GO:008009	nuclear	NP_001185	mmu:21453		
K01772	hemH, FEC	mmu01240(	PF00762.24	GO:001922	cytoplasmic	AAH96770.	mmu:14151		
K07605	KRT2		PF00038.26	GO:009951	mitochondr	AAI27242.1	mmu:332131		
K08727	NOD1, CA	mmu04621(	PF05729.18	GO:000959	cytoplasmic	NP_001164	mmu:107607		
K11292	SUPT6H, SPT6		PF14633.12	GO:008009	nuclear	NP_033323	mmu:20926		
KRQQ(Protein of unknown functior			PF16016.11	GO:012002	cytoplasmic	XP_060227	mmu:235283		
K06874	ZPR1		PF22794.1(j	GO:003315	cytoplasmic	NP_035882	mmu:22687		
K18596	EML3		PF23414.1(I	GO:009022	cytoplasmic	NP_001361	mmu:225898		

K10432	MAP6		PF21410.2(I	GO:009951	cytoplasmic	CAA75049.	mmu:17760
K08853	AAK		PF15282.11(BMP2K_C		cytoplasmic	XP_036022	mmu:269774
K00213	DHCR7	mmu00100(	PF01222.22	GO:001613	peroxisoma	XP_006508	mmu:13360
K23879	GRN		PF00396.23	GO:000578	extracellula	BAE35050.	mmu:14824
K21762	WDR62		PF24780.1(	GO:004212	cytoplasmic	BAE32697.	mmu:233064
K19466	DDX59		PF00270.35	GO:000367	cytoplasmic	NP_080776	mmu:67997
K00974	cca		PF01743.26	GO:004280	mitochondr	NP_001229	mmu:70047
K25059	SDE2		PF22781.2(	GO:000716	nuclear	NP_666055	mmu:208768
K00699	UGT	mmu00980(	PF00201.24	GO:000578	ER	NP_659545	mmu:94284
K10569	NEIL3	mmu03410(	PF06839.17	GO:000390	nuclear	NP_666320	mmu:234258
K17902	TBC1D4, A	mmu04931(	PF00566.25	GO:007149	cytoplasmic	XP_006518	mmu:210789
K01338	lon		PF05362.19	GO:000425	cytoplasmic	NP_080103	mmu:66887
K25356	ANGPTL1, ANG3		PF00147.23	GO:011016	ER	NP_082609	mmu:72713
K04447	JAK2	mmu04550(	PF07714.23	GO:001922	cytoplasmic	NP_001041	mmu:16452
K05115	AXL, UFO	mmu01521(	PF07714.23	GO:000690	extracellula	NP_033491	mmu:26362
K03063	PSMC4, RP	mmu05020(	PF00004.35	GO:000182	cytoplasmic	NP_036004	mmu:23996
K22076	MFF		PF05644.16	GO:000802	peroxisoma	AQS27591.	mmu:75734
K20651	ARHGAP42, GRAF3		PF16746.10	GO:004590	cytoplasmic	NP_082099	mmu:71544
K16220	HAX1			GO:001922	cytoplasmic	NP_035956	mmu:23897
K26316	LRRC32	mmu04350(	PF13855.12	GO:004213	extracellula	NP_001106	mmu:434215
K12484	RAB11FIP1	mmu04144(	PF00168.36	GO:003288	cytoplasmic	G3XA57.1(	mmu:74998
K14677	ACY1	mmu00220(	PF01546.33	GO:000367	cytoplasmic	NP_001263	mmu:109652
K07199	PRKAB	mmu04530(	PF16561.11	GO:000663	cytoplasmic	BAE32089.	mmu:19079
K20705	DNMBP, ARHGEF36,		PF14604.12	GO:002260	cytoplasmic	NP_082305	mmu:71972
K06063	SNW1, SKI	mmu04330(Notch signa		GO:008009	nuclear	AAH49245.	mmu:66354
K07994	MMP13	mmu04928(	PF00413.30	GO:000425	ER	NP_032633	mmu:17386
K01398	MMP2	mmu05219(	PF00413.30	GO:000150	ER	NP_032636	mmu:17390
K14156	CHK	mmu05231(	PF01633.27	GO:004428	cytoplasmic	EDL32959.	mmu:12660
Y78B(myeloid cell nuclear differenti			PF02760.21	GO:004592	nuclear	NP_001361	mmu:100040462
K00562	INMT, TEM	mmu00380(	PF01234.23	GO:009861	cytoplasmic	NP_033375	mmu:21743
K23441	MST1	mmu04020(	PF00051.23	GO:000425	plasma mer	NP_032269	mmu:15235
K03644	lipA, LIAS,	mmu01240(	PF04055.27	GO:000663	cytoplasmic	NP_001297	mmu:79464
K14802	DRS2, ATP	mmu04148(	PF16212.11	GO:199053	cytoplasmic	NP_001271	mmu:11980
K19933	CADPS		PF06292.23	GO:000810	cytoplasmic	XP_036022	mmu:320405
K23362	MPPE1, PG	mmu00563(	PF00149.33	GO:001602	ER	NP_001344	mmu:225651
YEWD(-)				GO:003450	nuclear	AAH44896.	mmu:66306
K02131	ATPeF0F6,	mmu05208(	PF05511.16	GO:000526	mitochondr	NP_001289	mmu:11957
K02270	COX7A	mmu05208(Chemical c		GO:002260	mitochondr	NP_001153	mmu:20463
K03029	PSMD4, RP	mmu05020(	PF13519.12	GO:002262	nuclear	NP_032977	mmu:19185
K16060	BIRC2_3	mmu04120(	PF00653.27	GO:001922	cytoplasmic	XP_006509	mmu:11796
K12842	SR140	mmu03040(	PF01805.25	GO:000557	nuclear	NP_001108	mmu:67958
K02374	PTPN13, F	mmu04210(	PF00595.29	GO:001922	plasma mer	NP_035334	mmu:19249
K07951	ARL6, BBS3		PF00025.27	GO:000858	cytoplasmic	NP_062639	mmu:56297

KRQQ(GRAM domain containing);			PF02893.26	GO:009908	cytoplasmic	BAE36327.1	mmu:107022	
K12330	ARHGEF1	mmu05163(	PF09128.16	GO:004393	cytoplasmic	EDL24303.	mmu:16801	
K10498	ZBTB11		PF13465.12	GO:008009	nuclear	NP_766614	mmu:27137	ZBTB
K00031	IDH1, IDH	mmu05230(	PF00180.25	GO:001922	cytoplasmic	NP_001104	mmu:15926	
K02832	RAIDD		PF00619.27	GO:200123	cytoplasmic	NP_001317	mmu:12905	
KT0G(Family with sequence similar			PF14648.11	GO:000810	cytoplasmic	BAC65579.1	mmu:210998	
K17972	NAA20, NAT3		PF00583.31	GO:003124	cytoplasmic	NP_001135	mmu:67877	
K06519	SLC3A2, M	mmu04216(	PF16028.10	GO:004877	Golgi appa	NP_001154	mmu:17254	
K24627	GAS2, GAS2L		PF02187.23	GO:003111	cytoplasmic	NP_001177	mmu:78926	
K19983	EXOC1, SEC3		PF20654.3(S	GO:000548	cytoplasmic	XP_021018	mmu:69940	
K14157	AASS	mmu00310(	PF16653.10	GO:000573	mitochondr	NP_038958	mmu:30956	
K16519	PALM2AKAP2		PF03285.20	GO:002260	cytoplasmic	NP_001371	mmu:677884	
K24062	PICK1		PF06456.18	GO:009008	cytoplasmic	NP_001039	mmu:18693	
K12836	U2AF1	mmu03040(	PF00642.30	GO:000613	cytoplasmic	XP_038937	mmu:233073	
K08825	DYRK1		PF00069.31	GO:008009	nuclear	EDL24152.	mmu:13549	
K02265	COX5B	mmu05208(	PF01215.24	GO:001545	mitochondr	P19536.1(R	mmu:12859	
K25757	DIPK2		PF12260.14	GO:001602	extracellulo	NP_001028	mmu:68861	
K14738	STEAP2	mmu04978(	PF03807.23	GO:000682	cytoplasmic	NP_001096	mmu:74051	
K25057	PSIP1, LED	mmu03250(	PF11467.13	GO:014029	nuclear	NP_598709	mmu:101739	
K13736	ARHGAP1	mmu05100(	PF16746.10	GO:000367	cytoplasmic	NP_084389	mmu:78514	
K13293	PDE4	mmu04928(	PF00233.25	GO:190189	cytoplasmic	NP_035186	mmu:238871	
K22063	ISCA1		PF01521.26	GO:002260	mitochondr	EDL32648.	mmu:69046	
K25208	ANKRD9			GO:000815	mitochondr	EDL18647.	mmu:74251	
K22649	BCL9, BCL9L		PF11502.13	GO:005125	cytoplasmic	NP_084209	mmu:77578	
K23411	FETUB		PF00031.27	GO:000989	extracellulo	AAN34519.	mmu:59083	
WEJ(CDKN2A interacting protein			PF11952.13(XTBD:XRN		cytoplasmic	NP_084252	mmu:52626	
K04097	HPGDS	mmu00980(	PF14497.11	GO:000663	cytoplasmic	NP_062328	mmu:54486	
K11340	ACTL6A, II	mmu04714(	PF00022.25	GO:003506	cytoplasmic	BAE41036.1	mmu:56456	
K20683	ARHGEF3_8		PF00621.25	GO:005145	cytoplasmic	NP_001040	mmu:56349	
K02733	PSMB10, M	mmu03050(	PF00227.31	GO:001977	cytoplasmic	NP_038668	mmu:19171	
K24069	PITPNM		PF02121.25	GO:009859	cytoplasmic	NP_032877	mmu:18739	
K27567	GRIPAP1			GO:003315	cytoplasmic	NP_997553	mmu:54645	
K10325	ASB3		PF12796.13	GO:000810	nuclear	NP_076395	mmu:65257	
K10069	CLEC1A			GO:000998	plasma mer	NP_001334	mmu:243653	
K22936	CNPY1_2		PF11938.13	GO:000989	ER	NP_064337	mmu:56530	
K17401	MRPS22		PF10245.14	GO:001593	mitochondr	NP_079761	mmu:64655	
K04507	CACYBP, S	mmu04310(	PF09032.16	GO:190169	cytoplasmic	NP_033916	mmu:12301	
K02868	RP-L11e, R	mmu05171(	PF00281.24	GO:000810	cytoplasmic	NP_000966	mmu:67025	
K11163	DHRS1		PF00106.31	GO:000403	peroxisoma	NP_081095	mmu:52585	
K14009	BCAP31, B	mmu05165(	PF05529.17	GO:003315	ER	BAE30571.1	mmu:27061	
K21129	LCN2	mmu04657(	PF00061.29	GO:001922	ER	NP_032517	mmu:16819	
K17659	PTCD3		PF22330.2(I	GO:001593	mitochondr	NP_081551	mmu:69956	
K17820	DHX33	mmu04621(	PF07717.22	GO:008009	nuclear	NP_848144	mmu:216877	

K17457	PPP1R12C,	mmu04810(	PF15898.10	GO:003243	cytoplasmic	NP_084110	mmu:232807	
ZBU3(stromal cell-derived factor)			PF02815.25	GO:001602	mitochondr	NP_071719	mmu:64136	
K13159	HNRNPL		PF13893.12	GO:003577	nuclear	BAE26011.	mmu:15388	
ZCFR(NHL repeat containing 3)			PF01436.27	GO:000988	lysosomal	NP_766089	mmu:212114	
K13248	PHOSPHO	mmu01240(	PF06888.18	GO:001678	cytoplasmic	NP_082797	mmu:73373	
)0KJ(Rhomboid domain containing)			PF01694.27	GO:000989	extracellulo	NP_001277	mmu:279766	
K11842	USP12_46		PF00443.35	GO:000417	cytoplasmic	NP_035799	mmu:22217	
K10838	XPC	mmu03420(	PF10405.15	GO:008009	nuclear	NP_033557	mmu:22591	
K15283	SLC35E1		PF03151.22	GO:000534	ER	NP_808434	mmu:270066	
XR08(Basic leucine zipper and W2)			PF02020.24	GO:008009	cytoplasmic	NP_080116	mmu:66912	
K08669	HTRA2, PR	mmu04215(	PF13365.12	GO:000425	peroxisoma	NP_062726	mmu:64704	
K13949	PTGR2, ZA	mmu00590(	PF16884.10	GO:000663	cytoplasmic	BAE34884.	mmu:77219	
K15304	RANBP3	mmu05166(	PF00638.24	GO:005116	nuclear	NP_001239	mmu:71810	
.5RF(Envelope)			PF00429.24	GO:004316	cytoplasmic	ABD14434.	mmu:108167918	
K05730	VAV	mmu04664(	PF00621.25	GO:000690	cytoplasmic	NP_035821	mmu:22324	
K24473	TENM, ODZ		PF25023.1(	GO:004859	cytoplasmic	XP_036008	mmu:23966	
K20396	TBC1D7	mmu04150(	PF00566.25	GO:008009	cytoplasmic	NP_001239	mmu:67046	
K16059	STIM1	mmu04611(	PF16533.10	GO:000578	nuclear	XP_008590	mmu:20866	
K23649	ABI1	mmu05132(	PF07815.19	GO:003243	cytoplasmic	NP_001317	mmu:11308	
K25628	TCP11		PF05794.18	GO:000557	cytoplasmic	NP_796164	mmu:320554	
K00128	ALDH	mmu01240(	Biosynthesis	GO:000548	peroxisoma	NP_001318	mmu:11671	
K04963	RYR3	mmu04970(	PF21119.3(I	GO:003202	cytoplasmic	XP_017172	mmu:20192	
K10054	PML, TRIM	mmu05164(	PF12126.13	GO:200123	cytoplasmic	NP_835188	mmu:18854	
K00286	proC	mmu00330(	PF14748.11	GO:000695	mitochondr	NP_598466	mmu:69051	
K10686	UBA3, UBE	mmu04120(	PF00899.27	GO:014029	cytoplasmic	NP_035796	mmu:22200	
K16272	RNF135		PF00622.36	GO:003577	cytoplasmic	NP_082295	mmu:71956	
			PF00607.25	GO:004316	Golgi appa	QNN81289	mmu:118568284	
K10134	EI24	mmu04115(	PF07264.17	GO:003315	cytoplasmic	AAC52483.	mmu:13663	
K00253	IVD, ivd	mmu00280(	PF00441.29	GO:003444	mitochondr	NP_062800	mmu:56357	
K03678	RRP45, EX	mmu03018(	PF01138.27	GO:007104	nuclear	NP_062266	mmu:50911	
K00725	B4GALNT	mmu00604(	PF00535.33	GO:004323	ER	NP_082015	mmu:14421	
K13755	PDE1	mmu05032(	PF00233.25	GO:000411	cytoplasmic	NP_001272	mmu:18574	
K02948	RP-S11, MR	mmu03010(	PF00411.24	GO:002262	mitochondr	NP_080774	mmu:67994	
K03065	PSMC3, RP	mmu05020(	PF00004.35	GO:003577	cytoplasmic	XP_048216	mmu:19182	
K04348	PPP3C, CN	mmu05020(	PF00149.33	GO:005104	cytoplasmic	AAH46617.	mmu:19057	
K09230	SCAN		PF13465.12	GO:008009	cytoplasmic	NP_001013	mmu:43273	zf-C2H2
K08805	DCLK1_2		PF00069.31	GO:003003	cytoplasmic	NP_001344	mmu:13175	
ZK3P(Negative regulator of ubiquit			PF18037.7(I	GO:000989	nuclear	NP_001292	mmu:53312	
K15010	HOMER	mmu04724(	PF00568.29	GO:005080	cytoplasmic	NP_671705	mmu:26556	
K15375	PLCL1, PRI	mmu04727(	PF00388.25	GO:000989	cytoplasmic	NP_001108	mmu:227120	
K16739	NDEL1		PF04880.18	GO:003315	cytoplasmic	NP_076157	mmu:83431	
K24480	CMYA5		PF00041.27	GO:000548	cytoplasmic	NP_076310	mmu:76469	
K11437	PRMT6		PF22528.1(I	GO:008009	nuclear	NP_849222	mmu:99890	

K22628	IL16	mmu04060(	PF00595.29	GO:001922	cytoplasmic	XP_006507	mmu:16170
K14797	ENP1, BYSL		PF05291.16	GO:003068	cytoplasmic	NP_058555	mmu:53414
K05294	PGAP1	mmu00563(	PF07819.20	GO:000810	ER	NP_001156	mmu:241062
K05286	PIGB	mmu00563(	PF03901.23	GO:000665	cytoplasmic	NP_001397	mmu:55981
K09577	FKBP14		PF00254.34	GO:000578	ER	NP_705801	mmu:231997
XPUI(Rho GTPase activating protei			PF00071.28	GO:190169	cytoplasmic	XP_006524	mmu:442827
K12177	COPS3, CSN3		PF22788.2(	GO:190216	cytoplasmic	NP_036121	mmu:26572
2ATR(CDC42 small effector)				GO:002260	cytoplasmic	NP_001033	mmu:72729
K12178	COPS4, CSN4		PF22241.2(I	GO:000802	cytoplasmic	NP_036131	mmu:26891
K04002	C4BPA	mmu04610(	PF00084.26	GO:003044	mitochondr	NP_001406	mmu:12269
at cell differentiation)			PF13855.12	GO:000643	cytoplasmic	NP_957678	mmu:72946
K14007	SEC24	mmu04141(	PF04811.21	GO:000810	cytoplasmic	NP_766184	mmu:218811
K19015	ITIH2, SHAP		PF06668.17	GO:003020	extracellula	BAE21837.1	mmu:16425
K14717	SLC39A11,	mmu05010(	PF02535.28	GO:001602	Golgi appa	EDL34404.1	mmu:69806
K08063	RFXAP	mmu04612(	PF15289.11	GO:000097	nuclear	NP_573494	mmu:170767
K16896	TPCN1	mmu04020(	PF00520.37	GO:000526	plasma mer	BAE23435.1	mmu:252972
K20720	RTN4	mmu05010(	PF02453.23	GO:007266	cytoplasmic	NP_918943	mmu:68585
K14841	NSA1, WDR74		PF00400.38	GO:000017	nuclear	NP_598900	mmu:107071
K03999	C8G	mmu05171(	PF00061.29	GO:001984	extracellula	NP_081338	mmu:69379
K05758	ARPC2	mmu04530(	PF04045.19	GO:000806	cytoplasmic	NP_001344	mmu:76709
K25362	APOC4		PF15119.11	GO:003435	extracellula	NP_031411	mmu:11425
K05696	PTPN1, PT	mmu05208(	PF00102.32	GO:200123	cytoplasmic	NP_035331	mmu:19246
K00994	CHPT1, CP	mmu05231(	PF01066.26	GO:000665	plasma mer	NP_001140	mmu:212862
)X8D(One of the primary rRNA bin			PF01344.31	GO:000020	cytoplasmic	NP_001074	mmu:232539
K19523	CPT1B	mmu04148(	PF00755.25	GO:000657	cytoplasmic	BAE27481.1	mmu:12895
K06481	ITGA2, CD	mmu05165(	PF00092.34	GO:190515	ER	NP_032422	mmu:16398
K12819	SLU7	mmu03040(	PF11708.13	GO:003303	nuclear	EDL33860.1	mmu:193116
K11803	DCAF12		PF23760.1(I	GO:000989	nuclear	NP_081169	mmu:68970
K14394	ACP1	mmu05208(	PF01451.26	GO:001679	cytoplasmic	NP_067305	mmu:11431
K04508	TBL1	mmu04310(	PF00400.38	GO:008009	nuclear	BAC29294.1	mmu:81004
K11858	USP48		PF00443.35	GO:000417	nuclear	NP_001334	mmu:170707
K12195	CHMP6, VI	mmu03250(	PF03357.26	GO:000699	cytoplasmic	NP_001078	mmu:208092
1BHK(nucleolar protein 3 (apoptosi			PF00619.27	GO:200123	cytoplasmic	NP_084428	mmu:78688
K07966	B4GALT1	mmu00052(	PF13733.12	GO:200024	Golgi appa	NP_071641	mmu:14595
K12313	ACTA2	mmu04270(	PF00022.25	GO:009951	cytoplasmic	NP_001026	mmu:11475
K27162	ZCCHC3	mmu04623(	PF23058.1(I	GO:000959	nuclear	NP_780335	mmu:67917
K00940	ndk, NME	mmu01240(	PF00334.24	GO:000167	cytoplasmic	NP_001365	mmu:18102
ZW0W(Major role in the synthesis o			PF13445.12	GO:001989	cytoplasmic	NP_001344	mmu:20128
K19583	GK5		PF00370.27	GO:000548	cytoplasmic	EDL20949.1	mmu:235533
K16767	CEP112		PF14846.11	GO:003150	cytoplasmic	EDL34342.1	mmu:76380
K22808	KANK		PF12796.13	GO:008009	cytoplasmic	NP_663586	mmu:235041
K00140	mmsA, iolA	mmu00280(	PF00171.27	GO:000449	mitochondr	NP_598803	mmu:104776
K15445	TRMT10, TRM10, RG9		PF01746.26	GO:005080	nuclear	NP_001335	mmu:108943

K03635	MOCS2B, n	mmu01240(	PF02391.22	GO:000677	cytoplasmic	NP_001106	mmu:17434	
K01596	E4.1.1.32, p	mmu04910(	PF00821.23	GO:009008	mitochondr	BAC26991.1	mmu:74551	
K16804	DLGAP5, DLG7		PF03359.18	GO:000367	cytoplasmic	NP_653136	mmu:218977	
K00683	QPCT		PF04389.22	GO:000367	extracellulo	NP_081731	mmu:70536	
K27412	PARPBP			GO:000989	mitochondr	NP_083525	mmu:75317	
K23334	RANBP9_10, RANBPM		PF00622.36	GO:000508	cytoplasmic	AAR01221.	mmu:74334	
K02166	SFRP1	mmu04310(	PF01392.28	GO:006034	extracellulo	NP_038862	mmu:20377	
K00597	MTRR	mmu04980(	PF00667.26	GO:003058	cytoplasmic	NP_001295	mmu:210009	
K03845	ALG3	mmu00510(	PF05208.19	GO:005292	cytoplasmic	XP_006521	mmu:208624	
Y9DJ(Mesoderm induction early res			PF19426.4(	GO:000989	cytoplasmic	NP_766181	mmu:21861	MYB
K09525	DNAJC5	mmu04141(	PF00226.36	GO:000548	cytoplasmic	XP_048297	mmu:13002	
K22376	KCMF1, FIGC		PF05605.18	GO:003197	cytoplasmic	NP_062689	mmu:74287	
K19787	CARNMT1	mmu00340(	PF07942.18	GO:004280	cytoplasmic	NP_080396	mmu:67383	
K12873	BUD31, G1	mmu03040(	PF01125.22	GO:001921	cytoplasmic	NP_001039	mmu:231889	
K19662	PRKCB	mmu04750(	PF00069.31	GO:009008	cytoplasmic	NP_001303	mmu:18751	
K11411	SIRT1, SIR2	mmu04152(	PF02146.23	GO:005128	cytoplasmic	NP_001153	mmu:93759	
K08074	ADPGK	mmu01200(	PF04587.20	GO:004603	ER	NP_082397	mmu:72141	
KXF(coiled-coil domain containin			PF09774.14	GO:004323	cytoplasmic	NP_941047	mmu:381045	
K10734	GINS3		PF22466.2(	GO:007110	nuclear	NP_084474	mmu:78833	
K05542	DUS1		PF01207.24	GO:004316	mitochondr	NP_001404	mmu:68730	
GEF(pleckstrin homology domain			PF00169.35	GO:000989	cytoplasmic	NP_001008	mmu:231002	
K24048	MPP2_6		PF00625.27	GO:000548	cytoplasmic	NP_001401	mmu:50997	
K22855	EEF1AKMT1, EFM5		PF10237.15	GO:000647	cytoplasmic	AAH51925.	mmu:68043	
K17356	TSPAN13_31		PF00335.26	GO:000557	plasma mer	NP_080258	mmu:67125	
K16778	PDPN		PF05808.16	GO:004423	extracellulo	NP_001277	mmu:14726	
K10747	LIG1	mmu03030(	PF01068.26	GO:003563	nuclear	AAI38542.1	mmu:16881	
YS8R(IGv); ENOG410YQB2(immur			PF07686.23	GO:000237	extracellulo	CAA75914.1(variable region of imn		
K17491	SMEK, PPI	mmu04922(	PF04802.21	GO:008009	cytoplasmic	BAC98154.1	mmu:104570	
K08799	MELK		PF00069.31	GO:000016	cytoplasmic	EDL02426.	mmu:17279	
K27411	PHAF1		PF03676.19	GO:000810	cytoplasmic	NP_663579	mmu:234678	
K12456	APC13, AN	mmu04110(	PF05839.16	GO:199023	cytoplasmic	NP_852059	mmu:69010	
K08881	TRIM24, TIF1A		PF00439.30	GO:008009	cytoplasmic	NP_659542	mmu:21848	
277T(LYR motif containing 2)			PF05347.21	GO:000699	mitochondr	NP_780573	mmu:108755	
K10512	ZBTB40		PF13465.12	GO:008009	nuclear	BAD90181.	mmu:23084	ZBTB
K23797	PLEKHA4_5_6_7		PF00169.35	GO:007027	nuclear	NP_659169	mmu:109135	
K11364	SGF29		PF07039.17	GO:003506	nuclear	NP_083615	mmu:75565	
K03344	IDAX	mmu04310(	PF02008.25	GO:000367	cytoplasmic	XP_028616	mmu:319478	
K15407	QTRT2, QTRTD1		PF01702.25	GO:000815	cytoplasmic	XP_006521	mmu:106248	
K27804	FRY		PF19421.4(	GO:000989	cytoplasmic	XP_006504	mmu:320365	
K14990	SLC38A1, S	mmu04724(	PF01490.24	GO:005104	plasma mer	NP_001159	mmu:105727	
K18710	SLBP		PF15247.11	GO:000548	cytoplasmic	BAE20786.1	mmu:20492	
K15210	SNAPC3		PF12251.13	GO:003277	nuclear	NP_084225	mmu:77634	
K26934	ATP11	mmu04148(	PF16209.10	GO:199053	cytoplasmic	BAE33225.1	mmu:76295	

K01068	ACOT1_2_	mmu01040(	PF08840.17	GO:000663	mitochondr	AAH64469.	mmu:171210	
K23563	EMC2, TTC35		PF22890.2(	GO:000810	cytoplasmic	NP_080012	mmu:66736	
ZCAE(raftlin lipid raft linker 1)			PF15250.11	GO:000810	cytoplasmic	NP_852062	mmu:76438	
K00460	ALOX15	mmu04726(	PF00305.24	GO:004340	cytoplasmic	XP_006532	mmu:11687	
K04397	CASP7	mmu05022(	PF00656.27	GO:004352	cytoplasmic	NP_031637	mmu:12369	
K10624	RBBP6		PF08783.17	GO:008009	nuclear	NP_035377	mmu:19647	
K00458	ALOX12	mmu04750(	PF00305.24	GO:003444	ER	NP_031466	mmu:11684	
K03348	APC1, ANA	mmu04110(	PF20518.4(	GO:003304	nuclear	AAI45493.1	mmu:17222	
K15185	AFF4	mmu03250(	PF05110.18	GO:000802	nuclear	NP_001350	mmu:93736	AF-4
K16460	CEP128			GO:000810	cytoplasmic	NP_861536	mmu:75216	
K15272	SLC35A1_2_3		PF04142.21	GO:001593	Golgi appa	XP_006537	mmu:24060	
K02207	UBE2R, UE	mmu04120(	PF00179.31	GO:000016	cytoplasmic	NP_001121	mmu:67615	
K17433	MRPL52		PF18699.6(	GO:001593	mitochondr	NP_081127	mmu:68836	
K10998	TIPIN, CSM3, SWI3		PF07962.17	GO:000548	nuclear	NP_001311	mmu:66131	
K23616	ATXN1_1L	mmu05017(	PF12547.13	GO:005116	nuclear	NP_001186	mmu:20238	
K23554	GRAMD4		PF02893.26	GO:006220	nuclear	Q8CB44.1(I	mmu:223752	
K20897	GBP2	mmu04621(	PF02263.25	GO:004858	cytoplasmic	NP_034389	mmu:14468	
2B5B(Phosphoinositide-3-kinase inte			PF00051.23	GO:000417	cytoplasmic	EDL40408.	mmu:216505	
Lactoylglutathione lyase)			PF13771.12	GO:004316	nuclear	NP_001406	mmu:219132	
K12882	NCBP1, CB	mmu03013(	PF09090.17	GO:005116	nuclear	NP_001028	mmu:433702	
K11114	ACD, TPP1		PF10341.16	GO:003510	cytoplasmic	NP_001012	mmu:497652	
K10366	DMD	mmu05410(	PF00435.27	GO:004239	cytoplasmic	NP_031894	mmu:13405	
K04410	PAK2	mmu04012(	PF00069.31	GO:200123	cytoplasmic	NP_796300	mmu:224105	
K12792	TRIP6	mmu04621(	PF00412.28	GO:000512	cytoplasmic	NP_035769	mmu:22051	
K26390	TNFAIP8		PF05527.16	GO:000268	cytoplasmic	NP_081482	mmu:69769	
K05661	ABCB6	mmu02010(	PF16185.10	GO:000578	cytoplasmic	NP_076221	mmu:74104	
K15619	SPINT1	mmu05202(	PF00014.28	GO:004671	extracellula	BAB30697.1	mmu:20732	
K17834	CGAS, MB2	mmu04623(	PF03281.20	GO:008009	nuclear	NP_775562	mmu:214763	
K25228	APC15, AN	mmu04110(Cell cycle);		GO:003304	extracellula	XP_051048	mmu:75430	
K18496	FGF1	mmu04010(	PF00167.24	GO:003214	cytoplasmic	NP_034327	mmu:14164	
K06816	GLG1, ESL	mmu04514(	PF00839.22	GO:000810	ER	NP_033175	mmu:20340	
K13135	GEMIN7, SIP3		PF11095.14	GO:002260	cytoplasmic	NP_081465	mmu:69731	
K11659	RBBP7	mmu03082(	PF00400.38	GO:200073	nuclear	NP_033057	mmu:245688	
K01070	frmB, ESD,	mmu01200(	PF00756.26	GO:004428	mitochondr	NP_001272	mmu:13885	
K26434	LRRFIP		PF09738.14	GO:004356	nuclear	XP_006529	mmu:16978	
K23703	MIA2, CTAGE5			GO:000810	ER	XP_030102	mmu:338320	
K12650	TANK	mmu05022(	PF12845.12	GO:009008	mitochondr	NP_001361	mmu:21353	
K19749	RAI1		PF13771.12	GO:004863	cytoplasmic	NP_001032	mmu:19377	
K06774	HNT		PF07679.22(I-set:Immu		cytoplasmic	XP_006510	mmu:235106	
K09535	DNAJC15			GO:000810	cytoplasmic	NP_079660	mmu:66148	
K08887	ABL2	mmu04012(	PF07714.23	GO:003563	cytoplasmic	NP_001129	mmu:11352	
K09652	RHBDD2, RHBDL7		PF01694.27	GO:000425	Golgi appa	NP_666114	mmu:215160	
XW3G(Chromosome 8 open reading			PF15393.11(DUF4615:I		cytoplasmic	NP_001334	mmu:117171	

K18752	TNPO1, IP	mmu03013(	PF13513.12	GO:003450	cytoplasmic	XP_021035.	mmu:238799	
K04432	MAP2K3, M	mmu04664(	PF00069.31	GO:001922	cytoplasmic	NP_032954	mmu:26397	
K05451	NGFA	mmu04750(	PF00089.32	GO:000425	lysosomal	NP_035045	mmu:18048	
K23905	PEF1		PF13499.12	GO:008009	cytoplasmic	NP_080717	mmu:67898	
M05(Family with sequence similari			PF07326.16	GO:000557	cytoplasmic	NP_001405	mmu:109212	
K08552	NR3B1, ESRRA		PF00105.24	GO:008009	mitochondr	XP_031245	mmu:26379	
K17276	LCP1, PLS2		PF00307.36	GO:003243	cytoplasmic	BAE39375.1	mmu:18826	
K10370	TMOD	mmu04820(	PF03250.19	GO:002260	cytoplasmic	NP_057921	mmu:50874	
K10743	RNASEH2/	mmu03030(	PF01351.24	GO:000629	cytoplasmic	NP_081463	mmu:69724	
K16519	PALM2AKAP2		PF03285.20	GO:000810	cytoplasmic	BAC29683.1	mmu:18483	
K06537	CD151, TSPAN24		PF00335.26	GO:001602	plasma mer	NP_001104	mmu:12476	
4KW(coiled-coil domain containing 159)					peroxisoma	XP_029337	mmu:67119	
K01349	FURIN, PC	mmu03250(	PF00082.27	GO:000425	plasma mer	NP_001074	mmu:18550	
K21413	ANKS1		PF12796.13	GO:000989	cytoplasmic	NP_001361	mmu:224650	
K13162	PCBP2	mmu04216(	PF00013.35	GO:008009	cytoplasmic	XP_005652	mmu:18521	
K22370	AREL1		PF00632.30	GO:003210	extracellulo	BAE32654.1	mmu:68497	
K15014	SLC29A1_2	mmu05034(	PF01733.24	GO:000759	ER	NP_001186	mmu:63959	
K24916	ZNF521, EVI3		PF13894.12	GO:000097	cytoplasmic	BAC38132.1	mmu:22520	zf-C2H2
K18726	FAF2, UBXD8		PF22566.2(U	GO:000810	cytoplasmic	NP_848484	mmu:76577	
K10389	TUBG	mmu05165(	PF00091.31	GO:000024	cytoplasmic	EDL03891.1	mmu:103733	
K19867	PDLIM5_6	mmu04820(	PF00595.29	GO:003243	nuclear	NP_001177	mmu:56376	
K18399	BPHL		PF00561.26	GO:004423	mitochondr	BAC37185.1	mmu:68021	
K02929	RP-L44e, R	mmu05171(	PF00935.25	GO:004423	cytoplasmic	NP_001029	mmu:19982	
K03883	ND5	mmu05208(	PF00361.26	GO:000916	ER	ACM24395.	mmu:17721	
K01217	IDUA	mmu04142(	PF01229.23	GO:003510	lysosomal	NP_032351	mmu:15932	
K13288	orn, REX2,	mmu03008(	PF00929.31	GO:000829	mitochondr	BAE43110.1	mmu:104444	
K03850	ALG10	mmu00510(	PF04922.18	GO:003250	ER	NP_001028	mmu:380959	
Guanine nucleotide-binding protein			PF12799.13	GO:000998	cytoplasmic	NP_796067	mmu:320184	
K20412	PI16, CRISP9, CD364		PF00188.32	GO:001474	extracellulo	NP_076223	mmu:74116	
K10300	FBH1, FBXO18		PF00580.27	GO:200123	mitochondr	BAE28165.1	mmu:50755	
K19876	STEAP4		PF03807.23	GO:000682	cytoplasmic	NP_473439	mmu:117167	
K09187	MLL2, ALR	mmu04934(	PF00628.35	GO:008009	nuclear	NP_001028	mmu:381022	
K15117	SLC25A34_35, OAC1		PF00153.32	GO:003109	cytoplasmic	NP_001404	mmu:71998	
K10645	MIB	mmu05417(	PF12796.13	GO:005104	cytoplasmic	AAI31663.1	mmu:225164	
K09282	HMGA1			GO:009039	nuclear	NP_001160	mmu:111241	
K06753	ROBO1	mmu04360(	PF07679.22	GO:001922	cytoplasmic	XP_006523	mmu:19876	
K17339	ATL		PF02263.25	GO:002260	cytoplasmic	NP_062691	mmu:56298	
K10323	ASB1		PF07525.21	GO:199023	extracellulo	EDL40022.1	mmu:65247	
K03141	TFIIH1, GT	mmu03420(	PF08567.17	GO:003280	nuclear	NP_001278	mmu:14884	
K09403	FOXJ2_3		PF00250.24	GO:008009	nuclear	NP_068699	mmu:60611	Fork head
09E9(chromosome 5 open reading fr			PF12640.12(UPF0489:U		cytoplasmic	NP_001344	mmu:77877	
5RF(Envelope)			PF00429.24	GO:004316	cytoplasmic	AIW39926.1	mmu:105245342	
K05768	GSN	mmu04810(	PF00626.27	GO:003288	extracellulo	NP_666232	mmu:227753	

K06997	yggS, PROSC		PF01168.26	GO:004316	mitochondr	BAE22181.1	mmu:114863	
K01948	CPS1	mmu01100(	PF02786.22	GO:000573	mitochondr	NP_001074	mmu:227231	
K04514	ROCK1	mmu04530(	PF00069.31	GO:000301	cytoplasmic	NP_033097	mmu:19877	
K19539	SECISBP2, SBP2		PF01248.31	GO:004866	nuclear	NP_083555	mmu:75420	
K11320	EP400	mmu03082(	PF15790.10	GO:008009	cytoplasmic	Q8CHI8.3(I	mmu:75560	
K08529	RARG, NR1B3		PF00105.24	GO:003510	nuclear	NP_001398	mmu:19411	THR-like
K15307	GPS2	mmu05166(	PF15991.10	GO:008009	nuclear	NP_001344	mmu:56310	
K10599	PRPF19, PF	mmu04120(	PF24814.1(V	GO:000810	cytoplasmic	NP_598890	mmu:28000	
K20813	TDG	mmu03410(	PF03167.24	GO:008009	nuclear	NP_766140	mmu:21665	
K00419	QCR9, UCF	mmu05208(	PF05365.17	GO:004322	mitochondr	NP_932096	mmu:66152	
K03098	APOD		PF08212.18	GO:003315	extracellulo	NP_001288	mmu:11815	
K14684	SLC25A23S		PF00153.32	GO:004858	cytoplasmic	NP_001277	mmu:227731	
K17508	PTC7, PPTC7		PF07228.17	GO:001679	mitochondr	NP_796216	mmu:320717	
K14784	CMS1		PF14617.12	GO:003299	nuclear	NP_079875	mmu:66497	
K24500	VASN		PF13855.12	GO:001602	ER	NP_647468	mmu:246154	
K17432	MRPL51		PF10244.14	GO:001593	mitochondr	NP_079871	mmu:66493	
K21444	PCBP3_4		PF00013.35	GO:000989	cytoplasmic	NP_067543	mmu:59093	
K00476	ASPH	mmu04260(	PF05279.16	GO:000548	ER	NP_001171	mmu:65973	
K15360	STRA13, CI	mmu03460(	PF09415.16	GO:002260	nuclear	NP_001334	mmu:20892	
)VTC(Chromosome 1 open reading f			PF14868.11	GO:001990	cytoplasmic	NP_958752	mmu:381306	
K23707	SAMSN1		PF12485.13	GO:000989	cytoplasmic	NP_075869	mmu:67742	
XYGB(EH-domain containing)			PF00350.29	GO:001487	Golgi appa	NP_001334	mmu:106393	
K09545	HSPB6		PF00525.23	GO:000645	mitochondr	NP_001012	mmu:243912	
ERT(chromosome 1 open reading f			PF10504.14	GO:000367	cytoplasmic	NP_001012	mmu:230696	
K26006	MMADHC	mmu04980(	PF10229.14	GO:000573	mitochondr	EDL26886.1	mmu:109129	
K13279	PRDX1	mmu05146(	PF00578.27	GO:000578	cytoplasmic	NP_035164	mmu:18477	
K25226	C8ORF37		PF14996.11	GO:000573	cytoplasmic	NP_080281	mmu:67157	
K04958	ITPR1	mmu04750(	PF08709.17	GO:009008	cytoplasmic	NP_034715	mmu:16438	
K12624	LSM5	mmu03018(	PF01423.28	GO:000613	cytoplasmic	EDL88057.1	mmu:66373	
K22416	IREB2		PF00330.25	GO:000962	cytoplasmic	NP_073146	mmu:64602	
K05637	LAMA1_2	mmu05165(	PF00054.30	GO:005080	plasma mer	NP_032507	mmu:16773	
K10048	CEBPB	mmu04148(	PF07716.20	GO:008009	extracellulo	NP_034013	mmu:12608	TF_bZIP
K02208	CDK8_11		PF00069.31	GO:007122	cytoplasmic	NP_001161	mmu:78334	
K06745	PDL1, CD2	mmu05235(	PF13927.12	GO:004213	ER	NP_068693	mmu:60533	
K12496	EGR2	mmu04625(	PF11928.13	GO:005116	cytoplasmic	NP_001334	mmu:13654	zf-C2H2
K23741	MAN1B, M	mmu04141(	PF01532.26	GO:190169	Golgi appa	NP_001025	mmu:227619	
K01017	CHST11	mmu00532(	PF03567.20	GO:003510	Golgi appa	XP_031205	mmu:58250	
ZN6X(Death domain containing 1)			PF00531.28(Death:Dea		cytoplasmic	NP_001164	mmu:100322896	
K20899	GBP1_3_4_	mmu04621(	PF02263.25	GO:004858	cytoplasmic	XP_006501	mmu:55932	
K00799	GST, gst	mmu00980(	PF00043.30	GO:007124	cytoplasmic	NP_034487	mmu:14860	
K20072	SWAP70		PF00169.35	GO:003288	cytoplasmic	NP_033328	mmu:20947	
K19518	TRIB3	mmu04931(	PF00069.31	GO:005134	cytoplasmic	NP_780302	mmu:228775	
K22754	RNF213		PF20173.3(ZnF_RZ-typ		nuclear	NP_001035	mmu:672511	

K00901	dgkA, DGK	mmu05231(	PF00609.24	GO:000665	cytoplasmic	NP_001160	mmu:104418	
K13504	VAMP2	mmu04970(	PF00957.27	GO:002260	cytoplasmic	XP_032768	mmu:22318	
K03283	HSPA1_6_8	mmu04915(	PF00012.26	GO:003111	cytoplasmic	NP_034608	mmu:15511	
K20315	ARHGAP21_23		PF00620.32	GO:001562	nuclear	NP_001074	mmu:71435	
K19589	N6AMT1		PF05175.20	GO:000647	nuclear	EDK98331.	mmu:67768	
K10292	FBXO5, EM	mmu04114(	PF22191.2(I	GO:008009	mitochondr	NP_080271	mmu:67141	
K00799	GST, gst	mmu00980(	PF01124.23	GO:000578	mitochondr	NP_001334	mmu:56615	
K18750	APOBEC3	mmu03250(	PF18782.7(	GO:003577	cytoplasmic	Q99J72.3(R	mmu:80287	
K05651	ABCA9	mmu02010(	PF00005.33	GO:000686	ER	NP_671753	mmu:217262	
K22146	GSDME, D	mmu04623(	PF04598.17	GO:200123	cytoplasmic	AAI32304.1	mmu:54722	
K18172	CMC2		PF08583.15	GO:004323	cytoplasmic	NP_081120	mmu:66531	
K22816	CNPY3_4		PF11938.13	GO:190547	extracellulo	NP_848727	mmu:66455	
K09228	KRAB		PF13465.12	GO:008009	cytoplasmic	AAH61209.	mmu:77519	zf-C2H2
27HA(SAYSVFN motif domain cont			PF10260.14	GO:003109	mitochondr	NP_080485	mmu:67509	
K12846	SNRNP27	mmu03040(	PF08648.17	GO:009030	extracellulo	NP_079941	mmu:66618	
K05698	PTPRJ, DE	mmu05208(	PF00102.32	GO:005134	plasma mer	NP_001129	mmu:19271	
K02900	RP-L27Ae,	mmu05171(	PF00828.25	GO:002262	cytoplasmic	XP_050022	mmu:26451	
K24681	DISP2			GO:000998	plasma mer	BAC29264.1	mmu:214240	
K02975	RP-S25e, RI	mmu05171(	PF03297.21	GO:000557	cytoplasmic	KAH05196(	mmu:75617	
K11266	MAU2	mmu04110(	PF10345.15	GO:000367	nuclear	NP_001161	mmu:74549	
QUX(nuclear transport factor)			PF02136.26	GO:003315	cytoplasmic	NP_001007	mmu:68051	
K14837	NOP12		PF00076.28	GO:009030	nuclear	NP_001361	mmu:52202	
K10808	RRM2	mmu00983(	PF00268.27	GO:006173	cytoplasmic	NP_033130	mmu:20135	
K16466	CETN3, CDC31		PF13499.12	GO:004316	cytoplasmic	KAB03652(	mmu:12626	
K12886	HNRNPK	mmu05203(	PF00013.35	GO:190216	nuclear	NP_001305	mmu:15387	
K26808	TXLN		PF09728.14	GO:004211	cytoplasmic	NP_001005	mmu:109658	
K03005	RPA49, PO	mmu03020(	PF06870.17	GO:002260	nuclear	NP_073722	mmu:64424	
K11162	RDH14		PF00106.31	GO:000403	mitochondr	NP_076186	mmu:105014	
K21437	ANKRD13		PF11904.13	GO:003543	cytoplasmic	NP_001013	mmu:433667	
K15025	EIF1AD		PF01176.25	GO:000557	nuclear	NP_081512	mmu:69860	
K18441	CYTH	mmu04144(	PF01369.25	GO:004858	cytoplasmic	NP_082471	mmu:72318	
KR7V(actin-binding Rho activating			PF14705.12	GO:005116	cytoplasmic	NP_780665	mmu:223513	
K19761	GGACT		PF06094.17	GO:001684	cytoplasmic	NP_001412	mmu:223267	
K00780	ST3GAL1	mmu00604(	PF00777.23	GO:007023	Golgi appa	NP_001399	mmu:20442	
ZCN4(SPOC domain containing 1);			PF07744.19	GO:003277	nuclear	NP_001074	mmu:213109	
K09140	TSR3		PF04034.18	GO:003111	cytoplasmic	XP_036016	mmu:68327	
K12812	DDX39B, U	mmu03013(	PF00270.35	GO:001593	cytoplasmic	AQS27607.1	mmu:53817	
K10586	BIRC6, BRU	mmu04140(	PF12356.13	GO:200123	cytoplasmic	AQS27609.1	mmu:12211	
K08341	GABARAP	mmu04137(	PF02991.22	GO:000810	cytoplasmic	AQS27601.1	mmu:56486	
K09037	MAFF_G_K		PF03131.22	GO:004322	nuclear	AQS27604.1	mmu:17133	
K20908	GBP6		PF02263.25	GO:002000	cytoplasmic	A0A0G2JD	mmu:100702	
K03178	UBE1, UBA	mmu04120(	PF10585.14	GO:000016	cytoplasmic	AQS27600.1	mmu:22201	
K12883	NCBP2, CB	mmu03013(	PF00076.28	GO:005116	nuclear	AQS27606.1	mmu:68092	

K20775	UIMC1, RA	mmu03440(	PF18282.7(I	GO:008009	nuclear	NP_001346	mmu:20184	
K10902	RECQL5		PF06959.16	GO:008009	nuclear	XP_006532	mmu:170472	
K11419	SUV39H, C	mmu00310(	PF00856.33	GO:008009	cytoplasmic	NP_073561	mmu:64707	
K24509	VWA3		PF13768.12(VWA_3:vo		nuclear	NP_808365	mmu:233813	
K23935	ACBD3	mmu05132(	PF13897.11	GO:000548	nuclear	Q8BMP6.3(	mmu:170760	
K24348	UBXN1_4	mmu04141(	PF00789.26	GO:001602	cytoplasmic	NP_080666	mmu:67812	
K15423	PPP4C	mmu04922(	PF00149.33	GO:001922	cytoplasmic	NP_001092	mmu:56420	
K11481	AURKA	mmu04114(	PF00069.31	GO:000810	cytoplasmic	NP_001278	mmu:20878	
K10359	MYO7A, U	mmu04814(	PF00063.27	GO:000810	cytoplasmic	NP_001243	mmu:17921	
K06134	coq7	mmu01240(	PF03232.19	GO:000449	mitochondr	NP_034070	mmu:12850	
K04716	SGPP1	mmu04071(	PF01569.27	GO:000664	cytoplasmic	NP_109675	mmu:81535	
K06709	KDM4, JMJD2, JHDM		PF02373.28	GO:003506	nuclear	NP_001413	mmu:230674	
K01832	TBXAS1, C	mmu04611(	PF00067.27	GO:000449	ER	NP_035669	mmu:21391	
K19907	SYT7		PF00168.36	GO:000802	plasma mer	XP_060242	mmu:54525	
K09239	HIVEP		PF00096.32	GO:000097	nuclear	AAI50705.1	mmu:15273	zf-C2H2
K10888	FANCA	mmu03460(	PF15865.11	GO:004212	cytoplasmic	AAI50706.1	mmu:14087	
K18623	COBL		PF09469.16	GO:003514	nuclear	AAI45119.1	mmu:12808	
K05645	ABCA7	mmu02010(	PF00005.33	GO:000810	extracellula	AAI50709.1	mmu:27403	
K18050	PRKCE	mmu04530(	PF00069.31	GO:000578	cytoplasmic	NP_035234	mmu:18754	
K10431	MAP4		PF00418.24(Tubulin-bi		nuclear	XP_029337	mmu:17758	
19K(Family with sequence similarit			PF15711.10	GO:000548	mitochondr	EDL13839.	mmu:27999	
K04420	MAP3K2, M	mmu04010(	PF00069.31	GO:003563	cytoplasmic	NP_036076	mmu:26405	
K06081	NECTIN1,	mmu03266(	PF08205.18(C2-set_2:CI		ER	XP_034346	mmu:58235	
K10430	MAP2		PF08377.15	GO:190195	cytoplasmic	XP_036018	mmu:17756	
K14521	NAT10, KR	mmu03008(	PF13718.12	GO:008009	nuclear	NP_694766	mmu:98956	
K08135	COL15A	mmu04974(	PF06482.17	GO:000520	extracellula	BAE21987.	mmu:12819	
K10082	LMAN2, V	mmu04141(	PF03388.20	GO:003303	extracellula	NP_080104	mmu:66890	
K13859	SLC4A8		PF00955.27	GO:000810	cytoplasmic	NP_067505	mmu:59033	
K10956	SEC61A	mmu04141(	PF00344.25	GO:000578	ER	NP_001126	mmu:53421	
K04555	UBE2G2, U	mmu04141(	PF00179.31	GO:003315	cytoplasmic	XP_049641	mmu:22213	
Retrotransposon protein)			PF06817.19	GO:004671	Golgi appa	ABD97982.	mmu:118568634	
K21806	VCPKMT, METTL21D		PF10294.15	GO:000647	cytoplasmic	NP_001028	mmu:207965	
K15421	SERGEF		PF00415.24	GO:006034	cytoplasmic	XP_030098	mmu:27414	
K11967	ANKIB1		PF01485.27	GO:000989	cytoplasmic	AAI45512.1	mmu:70797	
K20163	DENND4		PF02141.26(DENN:DE		cytoplasmic	NP_001392	mmu:329877	
XVP0(NADH dehydrogenase (ubiqu			PF21362.3(	GO:004316	nuclear	NP_001344	mmu:125488414	
K17091	ANXA1		PF00191.25	GO:000690	cytoplasmic	ACG75704.	mmu:16952	
K17493	CAMSAP		PF08683.16	GO:002260	cytoplasmic	NP_001263	mmu:227634	
K07604	KRT1	mmu04915(	PF00038.26	GO:009951	cytoplasmic	NP_034793	mmu:16667	
K25168	HELZ		PF13087.12	GO:003577	cytoplasmic	NP_001355	mmu:78455	
XQGP(LAS1-like (S. cerevisiae))			PF24917.1(I	GO:000686	cytoplasmic	NP_083442	mmu:75089	
K11492	NCAPG2, LUZP5		PF12422.13	GO:003304	cytoplasmic	NP_598523	mmu:76044	
K02865	RP-L10Ae,	mmu05171(	PF00687.26	GO:001593	nuclear	XP_005005	mmu:19896	

K27566	GAPVD1		PF00616.24(RasGAP:G'		cytoplasmic	NP_001343	mmu:66691	
K18402	PHF20		PF12618.13	GO:003124	nuclear	AAH60121.	mmu:228829	
K08053	RASA2, GA	mmu04010(	PF00168.36	GO:001602	cytoplasmic	NP_444498	mmu:114713	
K08785	HTRA3		PF13365.12	GO:000425	extracellula	NP_084403	mmu:78558	
K02990	RP-S6, MRI	mmu03010(	PF01250.22	GO:002262	mitochondr	NP_536704	mmu:121022	
K03062	PSMC1, RP	mmu05165(	PF00004.35	GO:003640	nuclear	NP_001039	mmu:19179	
K20687	ARHGEF15		PF00621.25	GO:003250	cytoplasmic	NP_808234	mmu:442801	
K18178	COA5, PET	mmu04714(	PF10203.14	GO:000699	cytoplasmic	NP_001182	mmu:76178	
K03066	PSMC5, RP	mmu05020(	PF00004.35	GO:003640	cytoplasmic	NP_001248	mmu:19184	
K23047	UNKL		PF23261.1(z	GO:004322	cytoplasmic	NP_001183	mmu:74154	
K14998	SURF1, SHY1		PF02104.20	GO:001545	peroxisoma	XP_006497	mmu:20930	
K13214	NONO, NRB54		PF00076.28	GO:200123	nuclear	BAE41058.1	mmu:53610	
K24094	HS1BP3		PF00787.29	GO:000578	cytoplasmic	NP_067404	mmu:58240	
K24384	HIC		PF13465.12	GO:008009	nuclear	NP_001091	mmu:15248	ZBTB
K09382	PAX1_9		PF00292.24	GO:007149	nuclear	NP_035171	mmu:18511	PAX
K01328	F12	mmu04610(	PF00089.32	GO:000425	extracellula	AAH49867.	mmu:58992	
K17919	SNX4	mmu04144(	PF00787.29	GO:000810	cytoplasmic	NP_542124	mmu:69150	
K00546	HNMT	mmu00340(	PF13489.12	GO:005138	cytoplasmic	NP_536710	mmu:140483	
K24495	MYOM	mmu04820(	PF07679.22	GO:000548	cytoplasmic	NP_001077	mmu:17929	
)6K3(LTV1 homolog (S. cerevisiae))			PF01501.26	GO:000579	Golgi appa	NP_001159	mmu:76485	
K11969	ARIH2		PF01485.27	GO:000989	cytoplasmic	NP_001344	mmu:23807	
K06911	PIR		PF02678.22	GO:003022	cytoplasmic	NP_081429	mmu:69656	
KPYW(transmembrane and coiled-c			PF06762.19	GO:004423	ER	NP_849250	mmu:105847	
K11729	ACAD10		PF01636.29	GO:003444	mitochondr	NP_082313	mmu:71985	
K04649	UBE2K, HI	mmu04120(	PF00179.31	GO:003243	cytoplasmic	NP_001072	mmu:53323	
K12190	VPS36, EAI	mmu04144(	PF04157.22	GO:003251	nuclear	NP_081614	mmu:70160	
K10579	UBE2M, UI	mmu04120(	PF00179.31	GO:006165	cytoplasmic	NP_001076	mmu:22192	
K06689	UBE2D, UI	mmu04141(	PF00179.31	GO:004438	cytoplasmic	NP_001033	mmu:216080	
K10370	TMOD	mmu04820(	PF03250.19	GO:009951	cytoplasmic	BAE27611.1	mmu:50875	
.TII(peptidoglycan-binding, domain			PF01476.26	GO:000565	mitochondr	NP_694761	mmu:217779	
K07936	RAN	mmu03008(	PF00071.28	GO:005116	nuclear	NP_033054	mmu:19428	
K03687	GRPE		PF01025.24	GO:000810	mitochondr	BAE24062.1	mmu:17713	
K11460	PCGF2, RN	mmu03083(	PF16207.11	GO:200123	nuclear	NP_001156	mmu:22658	
K20858	MCU	mmu05020(	PF04678.18	GO:009008	peroxisoma	NP_001028	mmu:215999	
K09497	CCT5		PF00118.29	GO:009951	cytoplasmic	NP_031663	mmu:12465	
K09494	CCT2		PF00118.29	GO:009951	cytoplasmic	NP_001345	mmu:12461	
K09496	CCT4		PF00118.29	GO:009951	cytoplasmic	NP_033967	mmu:12464	
K09499	CCT7		PF00118.29	GO:009951	cytoplasmic	NP_031664	mmu:12468	
K01868	TARS, thrS	mmu00970(	PF00587.30	GO:000643	cytoplasmic	NP_149065	mmu:110960	
K06275	PARV	mmu04510(	PF00307.36	GO:002260	cytoplasmic	NP_001155	mmu:64099	
K09495	CCT3, TRIC5		PF00118.29	GO:009951	cytoplasmic	NP_033966	mmu:12462	
K10758	QSOX		PF18371.6(I	GO:000645	mitochondr	NP_001289	mmu:227638	
K10328	ASB6		PF00023.36	GO:000573	cytoplasmic	AAH19192.	mmu:72323	

K11650	SMARCD	mmu04714(	PF02201.23	GO:003506	nuclear	BAE34940.1	mmu:83796	
K05693	PTPRM	mmu04520(	PF00102.32	GO:000647	plasma mer	NP_001344	mmu:19274	
25HM(chromosome 6 open reading f			PF07896.17	GO:002260	mitochondr	NP_080779	mmu:68002	
Cold shock domain containing E1, R			PF13465.12	GO:008009	nuclear	NP_001139	mmu:20922	zf-C2H2
Y78B(G protein-coupled receptor as			PF02760.21	GO:003210	nuclear	NP_778191	mmu:236312	
Y42A(-)				GO:000557	plasma mer	NP_001170	mmu:68554	
K16174	MRPS18B, I	mmu05203(	PF01084.26	GO:001593	cytoplasmic	NP_080154	mmu:66973	
K02988	RP-S5, MRI	mmu03010(	PF21251.2(I	GO:001593	mitochondr	NP_084239	mmu:77721	
K19362	TMEM231		PF10149.14	GO:003250	Golgi appa	NP_001028	mmu:234740	
K21944	PER1	mmu04710(	PF12114.13	GO:003292	cytoplasmic	NP_001152	mmu:18626	
K02892	RP-L23, MF	mmu03010(	PF00276.25	GO:001593	mitochondr	NP_035418	mmu:19935	
Z17S(scavenger receptor class A mer			PF01391.23	GO:000520	Golgi appa	NP_766192	mmu:219151	
K25817	MAF1		PF09174.15	GO:000989	nuclear	XP_006241	mmu:68877	
K24992	MYEF2		PF00076.28	GO:000097	cytoplasmic	NP_001155	mmu:17876	TF_others
HAUS augmin-like complex, subuni			PF00096.32	GO:008009	cytoplasmic	NP_001013	mmu:67109	zf-C2H2
K07604	KRT1	mmu04915(	PF00038.26	GO:009951	cytoplasmic	NP_082259	mmu:71888	
K03264	EIF6	mmu03008(	PF01912.23	GO:005116	cytoplasmic	NP_034709	mmu:16418	
K17597	STAU		PF00035.32	GO:005080	nuclear	NP_001333	mmu:29819	
K12194	CHMP4A_I	mmu03250(	PF03357.26	GO:000810	cytoplasmic	NP_001386	mmu:75608	
K18466	VPS26A_B	mmu04144(	PF03643.20	GO:000810	cytoplasmic	NP_001100	mmu:69091	
K12758	MYLPF	mmu04670(	PF13499.12	GO:000237	cytoplasmic	NP_001334	mmu:17907	
K16913	PELP1, MNAR		PF08166.18	GO:008009	nuclear	BAB23754.1	mmu:75273	
K04439	ARRB	mmu04010(	PF00339.34	GO:004340	cytoplasmic	NP_001258	mmu:216869	
K04671	BMPR2	mmu04550(	PF00069.31	GO:004590	plasma mer	NP_031587	mmu:12168	
K00799	GST, gst	mmu00980(	PF02798.26	GO:004322	cytoplasmic	XP_011241	mmu:14872	
K11995	DNASE1L		PF03372.29	GO:000578	ER	Q9D7J6.3(F	mmu:69537	
K13119	FAM50, XAP5		PF04921.20	GO:008009	nuclear	NP_613073	mmu:108160	
K06820	PLXNA	mmu04360(	PF08337.17	GO:004867	ER	NP_032909	mmu:18846	
K15902	LAGE3, PCC1		PF09341.15	GO:000815	cytoplasmic	NP_079686	mmu:66192	
			PF15711.10	GO:001973	Golgi appa	NP_001300	mmu:66294	
K06472	FCGR2A, C	mmu05171(	PF13895.12	GO:000270	plasma mer	NP_001391	mmu:14131	
K02898	RP-L26e, R	mmu05171(	PF16906.11	GO:001593	cytoplasmic	KAI258133	mmu:19941	
K19367	SPG21	mmu04144(	PF00561.26	GO:000548	cytoplasmic	NP_001344	mmu:27965	
K01110	PTEN	mmu05208(	PF10409.15	GO:001679	nuclear	DAA64595.	mmu:19211	
K15412	CFP	mmu05168(	PF00090.25	GO:003010	extracellulo	NP_032849	mmu:18636	
K26454	CDK2AP1	mmu03082(	PF09806.14	GO:001922	cytoplasmic	NP_001387	mmu:13445	
K14006	SEC23	mmu04141(	PF04811.21	GO:000548	cytoplasmic	BAE42552.1	mmu:27054	
K12657	ALDH18A1	mmu00330(	PF00696.33	GO:001920	mitochondr	BAE35228.1	mmu:56454	
K01081	E3.1.3.5	mmu01100(	PF06941.18	GO:000916	mitochondr	NP_598790	mmu:103850	
K00733	B4GALT7	mmu00534(	PF02709.20	GO:003020	Golgi appa	NP_666157	mmu:218271	
K16302	CNNM		PF01595.25	GO:009859	ER	NP_291047	mmu:94219	
Z45E(pleckstrin homology domain c			PF00784.22	GO:000585	cytoplasmic	NP_666142	mmu:217198	
YRGB(IGv); ENOG410YMG4(imm			PF07686.23(V-set:Immu		plasma mer	DBA12451.1(TPA: IGHV6-5*01, pa		

K03662	ATPeVS1, /	mmu05165(	PF05827.17	GO:000810	plasma mer	NP_061264	mmu:54411	
K10612	CUL5	mmu04120(	PF00888.28	GO:004438	cytoplasmic	EDL25800.	mmu:75717	
.KU1(peptidoglycan-binding, doma			PF01476.26(LysM:LysM		cytoplasmic	NP_081585	mmu:70082	
K03521	fixA, etfB		PF01012.27	GO:000548	cytoplasmic	NP_080971	mmu:110826	
.N8A(ADP-ribosylation-like factor (			PF15062.11	GO:003109	mitochondr	AAH19550.	mmu:65103	
K06096	VAPA	mmu04979(	PF00635.32	GO:003003	cytoplasmic	EDL38296.	mmu:30960	
K08179	SLC16A1	mmu04148(	PF07690.21	GO:009008	lysosomal	NP_033222	mmu:20501	
.FAS(Inositol 1,4,5-trisphosphate re			PF20266.3(	GO:000557	Golgi appa	NP_001028	mmu:319622	
K07605	KRT2		PF00038.26	GO:009951	mitochondr	EDL04019.	mmu:77055	
K08893	HCK	mmu04062(	PF07714.23	GO:000690	cytoplasmic	NP_034537	mmu:15162	
K06527	VCAM1, CI	mmu04670(	PF13927.12	GO:003411	plasma mer	P29533.1(R	mmu:22329	
;ushi-repeat containing protein, X-l			PF13465.12	GO:000097	cytoplasmic	XP_036018	mmu:11548	zf-C2H2
oxygenase)			PF05368.20	GO:000557	nuclear	NP_001277	mmu:67824	
K26499	NCLN		PF04389.22	GO:000367	ER	XP_017169	mmu:103425	
K00463	IDO, INDO	mmu01240(	PF01231.23	GO:004213	cytoplasmic	NP_032350	mmu:15930	
.K8C(Chromosome 19 open reading			PF10171.14	GO:000810	cytoplasmic	NP_848734	mmu:69773	
K05030	CLCA3_4	mmu04972(	PF01835.25	GO:000522	cytoplasmic	BAE22045.	mmu:80797	
K11251	H2A	mmu05322(	PF16211.10	GO:000078	nuclear	NP_001035	mmu:319192	
K05720	FGD1	mmu04810(	PF00621.25	GO:002260	cytoplasmic	XP_006528	mmu:14163	
			PF00103.25	GO:000512	extracellulo	NP_001192	mmu:18775	
K19751	DNAAF2, KTU, PF13		PF08190.17	GO:001003	cytoplasmic	NP_081545	mmu:109065	
K05057	OSMR	mmu04151(	PF00041.27	GO:000512	plasma mer	NP_001297	mmu:18414	
K05159	TNFRSF23		PF00020.24	GO:200123	extracellulo	NP_077252	mmu:79201	
K16717	CEP97		PF14580.12	GO:009022	cytoplasmic	BAC25489.	mmu:74201	
K24827	SCNM1		PF15803.10	GO:004316	nuclear	BAB29077.1	mmu:69269	
K13509	AGPAT1_2	mmu00564(	PF01553.26	GO:000665	ER	NP_001156	mmu:55979	
K13718	OTUD4		PF02338.25	GO:009008	nuclear	NP_001074	mmu:73945	
K14693	SLC30A6, ZNT6		PF01545.26	GO:001602	extracellulo	NP_001239	mmu:210148	
K22540	HTD2	mmu01212(	PF01575.25	GO:000663	mitochondr	NP_001335	mmu:109729085	
K20044	PICALM		PF07651.22	GO:003425	mitochondr	BAD90216.	mmu:233489	
K03068	LRP5_6	mmu05022(	PF00058.23	GO:000810	ER	NP_032540	mmu:16974	
K15414	C1QBP		PF02330.21	GO:007013	mitochondr	NP_031599	mmu:12261	
K10261	FBXW2, MD6		PF00400.38(WD40:WD		cytoplasmic	NP_001393	mmu:30050	
XQFV(High density lipoprotein bin			PF00013.35	GO:003435	cytoplasmic	NP_001288	mmu:110611	
K08883	TRIM33, TIF1G		PF00439.30	GO:000827	nuclear	NP_444400	mmu:94093	
K17816	NUDT1, MTH1		PF00293.34	GO:003197	mitochondr	NP_001343	mmu:17766	
K27262	BOD1L1		PF05205.17	GO:000815	nuclear	NP_001074	mmu:665775	
K15438	AIMP2		PF18569.7(	GO:003250	cytoplasmic	NP_001165	mmu:231872	
K16865	PDCD4	mmu05205(	PF02847.23	GO:005134	cytoplasmic	NP_001161	mmu:18569	
K24731	DMWD		PF00400.38	GO:000573	cytoplasmic	NP_001361	mmu:13401	
K19678	IFT80		PF23335.1(I	GO:000810	cytoplasmic	NP_080917	mmu:68259	
XP60(integrin, beta)			PF23105.1(I	GO:009863	cytoplasmic	BAE43329.	mmu:223272	
K27342	TMEM223		PF06979.18	GO:003361	Golgi appa	NP_080067	mmu:66836	

KSWP(Nipsnap homolog)			PF07978.19	GO:004323	mitochondr	NP_079899	mmu:66536
K14012	NSFL1C, U	mmu04141(	PF08059.18	GO:199073	cytoplasmic	AAI06102.1	mmu:386649
K06751	MHC1	mmu05165(	PF00129.23	GO:000989	extracellulo	AAA39695.	mmu:15024
K02907	RP-L30, MF	mmu03010(	PF00327.25	GO:001593	mitochondr	NP_001343	mmu:107734
K14856	SDA1, SDAD1		PF08158.18	GO:005116	nuclear	NP_766301	mmu:231452
K17921	SNX7_30		PF00787.29	GO:000989	cytoplasmic	NP_083931	mmu:76561
K00775	ART3		PF01129.24	GO:001677	extracellulo	CAC84541.	mmu:109979
K23924	SRI		PF00036.39	GO:003577	cytoplasmic	NP_001074	mmu:109552
K16458	CEP104		PF21040.3((	GO:004322	cytoplasmic	BAE29108.	mmu:230967
K01679	E4.2.1.2B, fı	mmu01100(	PF00206.25	GO:008009	mitochondr	NP_034339	mmu:14194
K17338	REEP1_2_3_4		PF03134.24	GO:002260	ER	XP_006519	mmu:72549
ZZG9(PC-esterase domain containing)					extracellulo	EDL87106.	mmu:239647
K10372	TNNT1	mmu04814(	PF00992.25	GO:003103	cytoplasmic	AAI45450.1	mmu:21955
K11467	PHF1, PCL	mmu03083(	PF18104.7(	GO:004239	nuclear	XP_031204	mmu:21652
K25417	CCDC134		PF15002.11	GO:000578	ER	NP_766016	mmu:76457
K20183	VPS39, VAI	mmu04140(	PF10366.15	GO:001602	cytoplasmic	BAE24681.	mmu:269338
K14549	UTP15	mmu03008(	PF09384.15	GO:003204	cytoplasmic	NP_849249	mmu:105372
K19014	ITIH1, SHAP		PF06668.17	GO:003020	extracellulo	NP_032432	mmu:16424
K24185	KIF21	mmu04814(	PF00225.29	GO:009951	nuclear	NP_001102	mmu:16564
K25877	MYG1		PF03690.18	GO:000762	mitochondr	NP_068359	mmu:60315
K16197	YWHAB_Q	mmu04114(	PF00244.26	GO:003288	cytoplasmic	NP_001240	mmu:22631
K10950	ERO1L	mmu04141(	PF04137.20	GO:000578	ER	AAH25102.	mmu:50527
K14211	ELN	mmu04974(	Protein dige	GO:003003	extracellulo	NP_031951	mmu:13717
K19985	EXOC6, SEC15		PF04091.18	GO:000810	cytoplasmic	NP_796051	mmu:75914
K14957	LSP1	mmu04625(	PF02029.20	GO:000367	cytoplasmic	NP_001258	mmu:16985
K00764	purF, PPAT	mmu00250(	PF13522.11	GO:000759	cytoplasmic	BAE28301.	mmu:231327
K11797	PHIP, DCAF14		PF00400.38	GO:200123	nuclear	NP_001074	mmu:83946
K14281	TACC1		PF05010.19	GO:003015	nuclear	NP_796063	mmu:320165
K05859	PLCG2	mmu04750(	PF00388.25	GO:004643	cytoplasmic	EDL11583.	mmu:234779
K06172	APH1	mmu04330(	PF06105.17	GO:000578	ER	NP_666246	mmu:226548
K16769	CKAP2		PF15297.11	GO:007268	cytoplasmic	NP_853620	mmu:70466
.H24(Phosducinlike)			PF02114.22	GO:001922	cytoplasmic	NP_081126	mmu:68833
K16575	ACTR1, AR	mmu05014(	PF00022.25	GO:003563	cytoplasmic	NP_001003	mmu:54130
K17260	ACTR2, AR	mmu04530(	PF00022.25	GO:008009	cytoplasmic	NP_001095	mmu:66713
K04673	BMPR1A, A	mmu04390(	PF00069.31	GO:003510	extracellulo	NP_033888	mmu:12166
.JJD(Chromosome 15 open reading f			PF10574.14	GO:002260	cytoplasmic	NP_081696	mmu:70420
K08031	PAX6	mmu04950(	PF00292.24	GO:008009	nuclear	CAC80515.	mmu:18508 PAX
K18403	MSL3		PF05712.18(	MRG:MRG	nuclear	NP_001357	mmu:17692
K05012	CLCN3_4_	mmu04613(	PF00654.25	GO:000802	cytoplasmic	NP_001230	mmu:12728
K24909	DIP2C		PF00501.34(	AMP-bindi	cytoplasmic	NP_001074	mmu:208440
K13499	CHSY	mmu00532(	PF05679.22	GO:003020	mitochondr	NP_001074	mmu:78923
K20224	IPO9, RAN	mmu03013(	PF25018.1(I	GO:003450	cytoplasmic	NP_722469	mmu:226432
K05738	MYL1	mmu04814(	PF13405.12	GO:009951	cytoplasmic	NP_001407	mmu:17901

K13126	PABPC	mmu03018(	PF00076.28	GO:000989	cytoplasmic	XP_006503	mmu:230721	
K02150	ATPeV1E,	mmu05165(	PF01991.23	GO:009859	cytoplasmic	NP_031536	mmu:11973	
K18040	PTPN23		PF03097.24	GO:000810	cytoplasmic	NP_001074	mmu:104831	
K02145	ATPeV1A,	mmu05165(	PF00006.31	GO:000526	cytoplasmic	NP_001345	mmu:11964	
K11426	SMYD	mmu00310(	PF00856.33	GO:000810	cytoplasmic	BAE37175.	mmu:69726	
K21644	HBP1		PF08517.17	GO:004851	nuclear	EDL36902.	mmu:73389	HMG
K06467	CD22, SIGI	mmu04662(	PF24518.1(I	GO:001922	ER	EDL23981.	mmu:12483	
K02320	POLA1	mmu03030(	PF00136.26	GO:190231	nuclear	NP_032918	mmu:18968	
K04575	ALS2	mmu05014(	PF00415.24	GO:000810	cytoplasmic	EDL00123.	mmu:74018	
K20471	COPD, ARCN1, RET2		PF00928.26	GO:003303	mitochondr	NP_666097	mmu:213827	
K25828	CALCOCO1		PF07888.16	GO:005125	cytoplasmic	NP_080468	mmu:67488	
K02980	RP-S29e, RI	mmu05171(	PF00253.27	GO:001593	mitochondr	NP_001001	mmu:20090	
K12571	PAN2	mmu03018(	PF20770.3(I	GO:003577	nuclear	NP_598753	mmu:103135	
K01325	KLK1_2	mmu04614(	PF00089.32	GO:000425	lysosomal	NP_032719	mmu:18050	
K27817	RAP1GDS1		PF00514.29	GO:000810	cytoplasmic	NP_001035	mmu:229877	
K14643	ENTPD7		PF01150.23	GO:000921	extracellulo	NP_444333	mmu:93685	
K15719	NCOAT, M	mmu04931(	PF07555.19	GO:009008	nuclear	NP_076288	mmu:76055	
K21027	TRMU, SLM3		PF03054.22	GO:003422	mitochondr	NP_082339	mmu:72026	
K00729	ALG5	mmu00510(	PF00535.33	GO:000578	Golgi appa	BAB23015.	mmu:66248	
elicase)			PF13242.12	GO:001678	cytoplasmic	NP_001034	mmu:76987	
K13172	SRRM2, SRM300		PF08312.17	GO:000372	nuclear	AAH92355.	mmu:75956	
K01301	NAALAD			GO:000557	cytoplasmic	NP_001313	mmu:635702	
425(transmembrane and coiled-coil			PF14992.11	GO:000557	cytoplasmic	NP_083508	mmu:75275	
K26545	UBAP2		PF12478.13	GO:005113	nuclear	NP_001159	mmu:74383	
K06047	TTL		PF03133.20	GO:004863	cytoplasmic	NP_081468	mmu:69737	
K11188	PRDX6	mmu00480(	PF00578.27	GO:008009	cytoplasmic	NP_031479	mmu:11758	
K16632	RNASE4		PF00074.25	GO:000454	ER	AAV31936.	mmu:58809	
K18412	TNRC6, GW182		PF16608.10	GO:000989	nuclear	NP_659061	mmu:213988	
K13525	VCP, CDC4	mmu04141(	PF00004.35	GO:003577	cytoplasmic	NP_009057	mmu:269523	
K09603	OTUB2		PF10275.15	GO:000989	nuclear	NP_001171	mmu:68149	
K11850	USP26_29_37		PF00443.35	GO:000417	nuclear	EDL00328.	mmu:319651	
K23704	MIA3, TANGO1		PF07653.23	GO:000810	Golgi appa	Q8BI84.2(R	mmu:338366	
K15212	TAF1A, SL1		PF14929.12	GO:003277	nuclear	NP_001264	mmu:21339	
K02493	hemK, prmC, HEMK		PF17827.6(I	GO:004322	mitochondr	NP_598745	mmu:69536	
K20289	COG2		PF12022.14	GO:000810	cytoplasmic	NP_084022	mmu:76332	
N2D(ubiquitin-like 3)			PF13881.12	GO:004323	cytoplasmic	BAE22201.	mmu:24109	
K09028	JUNB	mmu04935(	PF03957.18	GO:000962	nuclear	NP_032442	mmu:16477	TF_bZIP
K09580	PDIA1, P4I	mmu04141(	PF00085.26	GO:000578	ER	EDL34766.	mmu:18453	
K11298	HMGXB4, HMG2L1		PF13775.11	GO:190494	nuclear	NP_821136	mmu:70823	HMG
XYIZ(myeloma overexpressed 2)			PF15004.11	GO:004212	cytoplasmic	NP_001102	mmu:66915	
K27491	ENSA, ARPP19		PF04667.22	GO:008009	cytoplasmic	NP_001253	mmu:59046	
K08331	ATG13	mmu04136(	PF10033.14(ATG13:Au		cytoplasmic	NP_001342	mmu:51897	
K03240	EIF2B5	mmu05168(	PF25084.1(I	GO:008009	cytoplasmic	NP_758469	mmu:224045	

K00803	AGPS, agpS	mmu00565(	PF02913.24	GO:000578	peroxisoma	NP_766254	mmu:228061	
K06089	F11R, JAM1	mmu04530(	PF13927.12	GO:000810	plasma mer	NP_766235	mmu:16456	
20JT(male-enhanced antigen 1)			PF06910.16	GO:000573	cytoplasmic	NP_001037	mmu:17256	
K19478	MAGT1, TU	mmu04141(	PF04756.19	GO:000815	mitochondr	EDL14066.	mmu:67075	
K23829	TNIP1, ABIN1		PF16516.10	GO:009008	cytoplasmic	NP_001258	mmu:57783	
K01772	hemH, FEC	mmu01240(	PF00762.24	GO:001922	mitochondr	BAC39290.	mmu:14151	
K07190	PHKA_B	mmu04922(	PF00723.27	GO:199023	mitochondr	NP_032858	mmu:18679	
K10478	BTBD3_6		PF08005.17	GO:003003	nuclear	XP_052039	mmu:22866	ZBTB
K03934	NDUFS1	mmu05208(	PF00384.28	GO:003197	mitochondr	NP_001153	mmu:227197	
K09663	GCNT4	mmu00512(	PF02485.27	GO:000237	Golgi appa	NP_001159	mmu:218476	
XSWW(Chromosome 10 open readir			PF23788.1(I	GO:008009	nuclear	NP_001394	mmu:214764	
K13192	RBM26		PF00076.28(RRM_1:RN		nuclear	NP_001355	mmu:74213	
K13209	EWSR1	mmu05202(	PF00641.23	GO:008009	nuclear	BAE27676.	mmu:14030	
K01443	nagA, AMD	mmu00520(	PF01979.26	GO:001975	mitochondr	XP_036016	mmu:245847	
N0B(SUZ RNA binding domain co			PF12752.12(SUZ:SUZ d		cytoplasmic	NP_001264	mmu:213491	
K19827	MACF1		PF00307.36	GO:000810	cytoplasmic	XP_036019	mmu:11426	
K09517	DNAJB11	mmu04141(	PF01556.23	GO:000645	ER	NP_001177	mmu:67838	
K02599	NOTCH1	mmu05165(	PF00008.33	GO:009859	plasma mer	NP_032740	mmu:18128	
K14427	SLC12A4_6, KCC1_3		PF00324.27	GO:003042	cytoplasmic	BAE21466.	mmu:107723	
K02738	PSMB6	mmu05020(	PF00227.31	GO:000417	cytoplasmic	NP_032972	mmu:19175	
K10583	UBE2S, E2I	mmu04120(	PF00179.31	GO:000578	cytoplasmic	EDL31265.	mmu:77891	
K12880	THOC3	mmu03013(	PF24807.1(V	GO:001593	mitochondr	NP_082873	mmu:73666	
JYH(ovarian cancer-associated gen			PF03959.19	GO:000573	mitochondr	NP_081412	mmu:246257	
K14004	SEC13	mmu03013(	PF00400.38	GO:005116	cytoplasmic	NP_077168	mmu:110379	
K22377	LTN1		PF22999.2(I	GO:000548	cytoplasmic	NP_001074	mmu:78913	
K01333	CFI	mmu04610(	PF00089.32	GO:000425	plasma mer	NP_031712	mmu:12630	
K22388	NAB		PF04905.18	GO:000989	nuclear	NP_032694	mmu:17937	
K22287	APOC2	mmu04979(	PF05355.16	GO:000810	extracellulo	NP_001264	mmu:11813	
K22388	NAB		PF04905.18	GO:000989	nuclear	NP_001407	mmu:17936	
K16449	RGS		PF00615.24	GO:000548	nuclear	NP_080694	mmu:67865	
K13528	MED20		PF08612.16	GO:006026	cytoplasmic	NP_064432	mmu:56771	
K17361	ACOT9		PF03061.28	GO:000663	mitochondr	NP_062710	mmu:56360	
K13514	LPGAT1	mmu00564(	PF16076.10	GO:004592	ER	NP_001128	mmu:226856	
K18994	DHX32		PF07717.22	GO:000367	cytoplasmic	AAH22920.	mmu:101437	
K22197	BAF45A, PI	mmu03082(	PF00628.35	GO:008009	nuclear	NP_077212	mmu:72057	
K14401	CPSF1, CF1	mmu03015(	PF03178.21	GO:000557	nuclear	NP_444423	mmu:94230	
K27104	ZNF827		PF13909.12(zf-H2C2_5:		nuclear	XP_029336	mmu:62267	zf-C2H2
K14326	UPF1, REN	mmu03013(	PF09416.16	GO:007104	cytoplasmic	NP_001116	mmu:19704	
K23282	PLEKHM1	mmu04140(	PF13901.12	GO:000810	cytoplasmic	NP_898855	mmu:353047	
K26435	MTDH, AEG1		PF15686.10	GO:000578	ER	NP_080278	mmu:67154	
K12075	DLG2	mmu05165(	PF00595.29(PDZ:PDZ (		cytoplasmic	XP_030098	mmu:23859	
K22383	IRF2BP		PF11261.13	GO:005125	cytoplasmic	NP_665835	mmu:238330	
K18491	ESX1	mmu04550(	PF00046.35	GO:004859	nuclear	XP_006528	mmu:13984	Homeobox

K01490	AMPD	mmu01100(	PF19326.5(	GO:000582	cytoplasmic	NP_001028	mmu:229665	
K17794	TIM23		PF02466.24	GO:000810	cytoplasmic	NP_058593	mmu:53600	
K14285	NXT1_2, P	mmu03008(	PF02136.26	GO:001593	nuclear	NP_001103	mmu:56488	
K00129	ALDH3	mmu00980(	PF00171.27	GO:000403	cytoplasmic	NP_080592	mmu:67689	
K23709	SORBS3		PF14604.12	GO:001716	cytoplasmic	NP_035496	mmu:20410	
K23960	METTL14		PF05063.20	GO:008009	nuclear	CAH67890	mmu:210529	
K18423	CSE1, CAS,	mmu03013(	PF03378.21	GO:003450	cytoplasmic	NP_076054	mmu:110750	
K13238	ECI1, DCI	mmu00071(	PF00378.25	GO:003444	mitochondr	NP_034153	mmu:13177	
K20368	CNIH, ERV14		PF03311.19	GO:000512	extracellulo	XP_015848	mmu:98417	
K01325	KLK1_2	mmu04614(	PF00089.32	GO:000425	lysosomal	NP_034775	mmu:16623	
K19195	ARID4B		PF08169.17	GO:008009	nuclear	NP_919238	mmu:94246	ARID
K11594	DDX3X, b	mmu05203(	PF00270.35	GO:003577	cytoplasmic	AAI72016.1	mmu:13205	
K05459	IGF1	mmu04750(	PF00049.23	GO:005104	extracellulo	NP_001104	mmu:16000	
K13116	DDX41, AI	mmu04623(	PF00270.35	GO:008009	cytoplasmic	AQS27628.	mmu:72935	
K25537	FGL2		PF00147.23	GO:005068	plasma mer	NP_032039	mmu:14190	
K12605	CNOT2, N	mmu03018(	PF04153.24	GO:003577	cytoplasmic	NP_001032	mmu:72068	
K00888	PI4KA	mmu04070(	PF19274.4(	GO:000548	cytoplasmic	NP_001360	mmu:224020	
K09436	FLI1	mmu05202(	PF00178.28	GO:000097	nuclear	NP_032052	mmu:14247	ETS
K05705	YES1	mmu04520(	PF07714.23	GO:009951	cytoplasmic	EDL37396.	mmu:22612	
K08820	CDK16, PCTK1		PF00069.31	GO:009008	cytoplasmic	XP_021008	mmu:18555	
K12887	HNRNPM	mmu03040(	PF00076.28	GO:005104	cytoplasmic	AQS27631.	mmu:76936	
K02357	tsf, TSFM		PF00889.24	GO:008009	mitochondr	AQS27625.	mmu:66399	
K00801	FDFT1	mmu00100(	PF00494.24	GO:000548	cytoplasmic	NP_001347	mmu:14137	
K05667	ABCC3	mmu02010(	PF00664.28	GO:001516	plasma mer	NP_083876	mmu:76408	
K05545	DUS4		PF01207.24	GO:000945	cytoplasmic	NP_082278	mmu:71916	
K09063	TCF3, E2A	mmu05202(	PF00010.31	GO:003315	nuclear	NP_001157	mmu:21423	bHLH
K01873	VARS, valS	mmu00970(	PF00133.28	GO:003563	cytoplasmic	NP_001411	mmu:22321	
K24818	RIPOR, FAM65		PF15903.10	GO:003315	mitochondr	AAH79880.	mmu:75687	
K00569	TPMT, tpm	mmu00983(	PF05724.17	GO:000680	cytoplasmic	NP_058065	mmu:22017	
K22449	TBCEL		PF14580.12	GO:002260	cytoplasmic	NP_001344	mmu:272589	
K05707	SNAI1	mmu04520(	PF00096.32	GO:200123	cytoplasmic	CAA47675.	mmu:20613	zf-C2H2
K12365	PREX1	mmu04062(	PF00621.25	GO:003042	cytoplasmic	AAI51075.1	mmu:277360	
K11127	TEP1		PF05731.17	GO:003563	cytoplasmic	NP_033377	mmu:21745	
K01897	ACSL, fadD	mmu01100(	PF00501.34	GO:001564	ER	BAF82006.1	mmu:216739	
K11204	GCLC	mmu01240(	PF03074.21	GO:200123	cytoplasmic	NP_034425	mmu:14629	
K11649	SMARCC	mmu04714(	PF16496.10	GO:003506	nuclear	NP_033237	mmu:20588	MYB
YE53(Metallophosphoesterase 1)			PF16046.10	GO:001660	cytoplasmic	NP_789806	mmu:72826	
K17706	RGS14	mmu04015(	PF02196.21	GO:005116	cytoplasmic	NP_058038	mmu:51791	
K08073	PNKP	mmu03410(	PF08645.17	GO:008009	mitochondr	XP_006541	mmu:59047	
K13140	INTS3		PF10189.15	GO:000962	cytoplasmic	BAE28188.	mmu:229543	
K15041	VDAC3	mmu05208(	PF01459.28	GO:000802	cytoplasmic	BAE36292.	mmu:22335	
K25612	BICRA, GL	mmu03082(	PF15249.11	GO:004212	cytoplasmic	NP_001074	mmu:243842	
K04498	EP300, CRI	mmu03083(	PF08214.17	GO:008009	nuclear	NP_808489	mmu:328572	

ZBU3(small integral membrane prot			PF02815.25	GO:000645'	ER	BAB22144.2	mmu:20316	
Heparan sulfate 2-o-sulfotransferase			PF00096.32	GO:014029'	cytoplasmic	NP_001103	mmu:23877	zf-C2H2
K20052	LRP8, APOER2		PF00058.23	GO:009859	cytoplasmic	PNI71454.1	mmu:16975	
K18442	ARFGEF, I	mmu04144(	PF01369.25	GO:000810	cytoplasmic	NP_001095	mmu:211673	
K06553	VPREB, CD179a		PF07686.23	GO:000237	cytoplasmic	AAM64201.	mmu:22362	
K03983	SERPINF2,	mmu04610(	PF00079.25	GO:008009	extracellula	NP_032904	mmu:18816	
K04863	CACNB2	mmu04010(	PF00625.27	GO:000526	cytoplasmic	NP_001393	mmu:12296	
K20127	BAIAP2L1, IRTKS		PF08397.16	GO:002260'	cytoplasmic	NP_080109	mmu:66898	
K05071	IL4R, CD12	mmu04658(	PF09238.16	GO:190303'	lysosomal	NP_001008	mmu:16190	
K20909	ANTXR	mmu04621(	PF05586.16	GO:000989'	cytoplasmic	NP_473382	mmu:69538	
K06820	PLXNA	mmu04360(	PF08337.17	GO:004395	plasma mer	NP_786926	mmu:243743	
INTX(TM2 domain containing 1)			PF05154.21	GO:000493	cytoplasmic	NP_444387	mmu:94043	
K11508	CENPP		PF13096.11	GO:003408	nuclear	NP_079771	mmu:66336	
K04380	MAPT, TAU	mmu04010(	PF00418.24	GO:004867	cytoplasmic	XP_036012	mmu:17762	
K19464	PLEKHG5	mmu05200(	PF00621.25	GO:000989	cytoplasmic	NP_001412	mmu:269608	
K01823	idi, IDI	mmu00900(	PF00293.34	GO:000548	cytoplasmic	P58044.1(R	mmu:319554	
K20394	SESN2	mmu04115(	PF04636.18	GO:003288	cytoplasmic	NP_659156	mmu:230784	
K13099	CD2BP2, PPP1R59		PF02213.21	GO:000613	cytoplasmic	BAE32530.1	mmu:70233	
K07963	TRIM23, ARFD1		PF00025.27	GO:000810	cytoplasmic	EDL18499.	mmu:81003	
K05305	FUK	mmu00520(	PF07959.18	GO:190335	cytoplasmic	EDL11476.	mmu:234730	
K14328	UPF3, REN	mmu03013(	PF03467.21	GO:008009	nuclear	NP_080849	mmu:68134	
K13117	DHX35		PF07717.22	GO:001678'	cytoplasmic	NP_001359	mmu:71715	
K07952	ARFRP1		PF00025.27	GO:003250	cytoplasmic	NP_001159	mmu:76688	
K06065	NCOR2, SM	mmu04330(	PF00249.37(Myb_DNA		cytoplasmic	XP_030110	mmu:20602	MYB
K15434	FAM98B		PF10239.14	GO:000647	nuclear	EDL27895.	mmu:68215	
minichromosome maintenance comp			PF00566.25	GO:000810	nuclear	NP_038890	mmu:29809	
2CA2(Lymphoid-restricted membran			PF05781.17	GO:001602	nuclear	BAE38908.1	mmu:16970	
K27583	CENPV		PF04828.22	GO:003304	mitochondr	NP_082724	mmu:73139	
K02934	RP-L6e, RP	mmu05171(	PF01159.24	GO:003646	nuclear	NP_001408	mmu:19988	
K13823	HBB	mmu05144(	PF00042.28	GO:000534	cytoplasmic	NP_001188	mmu:101488143	
K17278	PGRMC1_2	mmu04080(	PF00173.33	GO:000004	ER	NP_081834	mmu:70804	
)MQP(chromosome 17 open reading			PF10309.14	GO:005116	nuclear	NP_080094	mmu:66874	
K07562	NMD3	mmu03008(	PF04981.18	GO:003288	cytoplasmic	NP_598548	mmu:97112	
K24220	MYH1s	mmu04814(	PF00063.27	GO:001645	cytoplasmic	NP_796343	mmu:17885	
K24220	MYH1s	mmu04814(	PF00063.27	GO:001645	cytoplasmic	XP_021031	mmu:17883	
K02090	CDK5	mmu04360(	PF00069.31	GO:004867	cytoplasmic	NP_001129	mmu:12568	
XUYP(Chromosome 5 open reading			PF15086.11	GO:000557	plasma mer	NP_001041	mmu:75616	
K09454	GABPB		PF12796.13	GO:008009	nuclear	NP_084161	mmu:21305	TF_others
K12751	MYL6	mmu04530(	PF00036.39	GO:004316	cytoplasmic	XP_008763	mmu:17904	
K12494	PSD	mmu04144(	PF01369.25	GO:009859	cytoplasmic	NP_001342	mmu:215632	
K18055	HIF1AN		PF13621.12	GO:000548	cytoplasmic	NP_795932	mmu:319594	
K01106	INPP5A	mmu04070(	PF22669.2(I	GO:001679	cytoplasmic	NP_001102	mmu:212111	
XP4J(Interferon-induced protein)				GO:000573'	mitochondr	NP_598632	mmu:99899	

K11878	PSMG4, PAC4		PF16093.10	GO:006500	cytoplasmic	NP_001094	mmu:69666	
K12176	COPS2, CSN2, TRIP15		PF01399.33	GO:008009	cytoplasmic	NP_001026	mmu:12848	
Gem (Nuclear organelle) associated			PF00096.32	GO:008009	nuclear	NP_001405	mmu:66056	zf-C2H2
K22991	WDR45, W	mmu04140(	PF21032.3(I	GO:000810	cytoplasmic	XP_021008	mmu:54636	
K07945	ARL4		PF00025.27	GO:004316	cytoplasmic	NP_001095	mmu:320982	
K16529	ARHGEF1	mmu04928(	PF17838.7(I	GO:005116	cytoplasmic	XP_006541	mmu:75547	
K21873	FAM208A		PF12509.13	GO:003250	nuclear	Q69ZR9.2(I	mmu:218850	
K13737	SEPT1	mmu05100(	PF00735.24	GO:005104	cytoplasmic	EDL17514.	mmu:54204	
K23740	SH3BGR		PF04908.21	GO:000573	cytoplasmic	EDL26465.	mmu:212531	
XNV4(transmembrane protein 62)			PF24394.1(	GO:001678	cytoplasmic	NP_780494	mmu:96957	
26BF(chromosome 19 open reading f			PF10572.14	GO:001922	ER	NP_543027	mmu:28106	
K26257	EMP3	mmu04350(	PF00822.25	GO:000691	extracellulo	NP_001139	mmu:13732	
K01097	NANP	mmu00520(	PF00702.32	GO:001679	cytoplasmic	NP_080362	mmu:67311	
K14209	SLC36A, PA	mmu04974(	PF01490.24	GO:009859	plasma mer	NP_694779	mmu:215335	
K01942	HLCS	mmu00780(	PF03099.24(BPL_LplA_		mitochondr	NP_001346	mmu:110948	
K20317	SPAG9, JIP4		PF19056.6(	GO:001922	cytoplasmic	Q58A65.2(F	mmu:70834	
K12793	MAP3K7IP	mmu04621(	PF02845.22	GO:000548	cytoplasmic	BAD90370.	mmu:66724	
K00856	ADK, adoK	mmu01100(	PF00294.29	GO:003292	cytoplasmic	NP_598840	mmu:11534	
K03989	C4	mmu05171(	PF07678.20	GO:004299	ER	XP_006523	mmu:12268	
K23625	ATXN2_2L	mmu05014(	PF14438.12	GO:000548	cytoplasmic	NP_001334	mmu:233871	
K06628	CDC45	mmu04110(	PF02724.19	GO:007110	nuclear	NP_001155	mmu:12544	
K14676	NTE, NRE	mmu00564(	PF24179.1(	GO:000578	ER	NP_001116	mmu:50767	
K14217	IFIT1	mmu05160(	PF07719.23	GO:004441	cytoplasmic	NP_001103	mmu:667373	
K00807	LTC4S	mmu00590(	PF01124.23	GO:000578	extracellulo	NP_032547	mmu:17001	
XRQQ(Cell Division)			PF02893.26	GO:004423	nuclear	NP_082174	mmu:52857	
K12857	SNRNP40,	mmu03040(	PF00400.38	GO:007100	cytoplasmic	NP_079921	mmu:66585	
K00088	IMPDH, gu	mmu01100(	PF00478.31	GO:000925	cytoplasmic	XP_031200	mmu:23918	
K00710	GALNT	mmu00514(	PF00535.33	GO:003024	cytoplasmic	BAD32383.	mmu:108760	
K15084	SLC25A16, GDA, LEU		PF00153.32	GO:001593	mitochondr	NP_780403	mmu:73132	
K15687	MKRN		PF18345.7(	GO:008009	cytoplasmic	NP_075779	mmu:67027	zf-CCCH
K01069	gloB, gloC,	mmu00620(	PF16123.11	GO:001679	mitochondr	Q99KB8.2(	mmu:14651	
K10453	KLHL16, GAN		PF01344.31	GO:199023	cytoplasmic	XP_055476	mmu:20923	ZBTB
K16766	CEP85		PF24555.1(	GO:000573	cytoplasmic	NP_001191	mmu:100038725	
K08504	BET1	mmu04130(	SNARE inte	GO:000810	cytoplasmic	NP_033878	mmu:12068	
K17497	PMM	mmu01240(	PF03332.19	GO:001967	cytoplasmic	NP_038900	mmu:29858	
K24752	WDR70		PF00400.38	GO:004323	nuclear	NP_001074	mmu:545085	
K15122	SLC41A		PF01769.21	GO:000367	peroxisoma	NP_082144	mmu:71699	
K01897	ACSL, fadD	mmu01100(	PF00501.34	GO:001564	cytoplasmic	XP_030099	mmu:14081	
)HBQ(Family with sequence similar			PF13349.12(DUF4097:I		mitochondr	NP_808537	mmu:330050	
K04004	CFH, HF1	mmu04610(	PF00084.26	GO:004858	extracellulo	NP_034018	mmu:12628	
K06840	SEMA3	mmu04360(	PF01403.25	GO:004858	extracellulo	NP_035478	mmu:20349	
K10875	RAD54L, R	mmu03440(	PF00176.29	GO:190136	cytoplasmic	NP_001116	mmu:19366	
K23353	PDLIM1_2	mmu04820(	PF00595.29	GO:003243	mitochondr	NP_062290	mmu:30794	

K10457	KLHL20, KLEIP		PF01344.31	GO:000810	cytoplasmic	EDL39323.	mmu:22654	ZBTB
K24739	WDR13		PF24817.1(	GO:004323	mitochondr	NP_001385	mmu:73447	
K11275	H1_5		PF00538.25	GO:003472	nuclear	NP_056601	mmu:50708	
K27803	HROB		PF15072.12	GO:000367	nuclear	NP_705772	mmu:217216	
K26161	FIBP		PF05427.16	GO:001660	cytoplasmic	NP_001240	mmu:58249	
.G0Y(surfeit locus protein)			PF04935.17	GO:002260	nuclear	NP_033324	mmu:20935	
K25829	ELOF1, ELF1		PF05129.18	GO:000802	cytoplasmic	NP_001098	mmu:66126	
K02926	RP-L4, MR	mmu03010(	PF00573.27	GO:001593	mitochondr	NP_075656	mmu:66163	
K07766	E3.6.1.52		PF00293.34	GO:004643	cytoplasmic	XP_024622	mmu:56409	
K00893	DCK	mmu01100(	PF01712.25	GO:001913	cytoplasmic	NP_031858	mmu:13178	
K10362	MYO18	mmu04814(	PF00063.27	GO:003288	cytoplasmic	NP_001278	mmu:360013	
K13738	CD2AP	mmu05100(	PF00018.34	GO:002260	cytoplasmic	AAI38375.1	mmu:12488	
K08071	NQO2		PF02525.22	GO:000548	mitochondr	BAB25683.1	mmu:18105	
K02136	ATPeF1G, .	mmu05208(	PF00231.24	GO:000526	mitochondr	NP_065640	mmu:11949	
K11459	PCGF4, BM	mmu05202(	PF16207.11	GO:200123	ER	NP_001403	mmu:12151	
K05854	LYN	mmu04664(	PF07714.23	GO:001922	cytoplasmic	NP_001104	mmu:17096	
K09230	SCAN		PF02023.22	GO:000097	nuclear	AAB03786.	mmu:22697	
K17525	CHID1		PF00704.34	GO:000577	ER	AAH61063.	mmu:68038	
K19201	SUDS3, SAP45		PF08598.16	GO:008009	nuclear	XP_021018	mmu:71954	
K11300	HMGN2		PF01101.23	GO:000989	nuclear	XP_051047	mmu:15331	
K18416	THEX1, ERI1		PF00929.31	GO:003600	nuclear	NP_080343	mmu:67276	
K11666	INO80B, Z	mmu03082(	PF04795.17	GO:008009	nuclear	AAH19383.	mmu:70020	
K05765	CFL	mmu05170(	PF00241.25	GO:003243	nuclear	XP_021007	mmu:12631	
K08639	CPXM2		PF00246.29	GO:000823	extracellulo	NP_061355	mmu:55987	
K03037	PSMD6, RP	mmu05020(	PF10602.15	GO:002262	cytoplasmic	NP_079826	mmu:66413	
K22817	NSMCE1, NSE1		PF07574.19	GO:199023	mitochondr	XP_021023	mmu:67711	
K26167	BAZ2B	mmu03082(	PF00439.30	GO:004316	nuclear	NP_001001	mmu:40782	MBD
K01946	ACACB	mmu00640(	PF08326.17	GO:004863	ER	NP_598665	mmu:100705	
K12353	SMPD4	mmu00600(	PF14724.11	GO:003014	cytoplasmic	XP_006522	mmu:77626	
K07915	RAB28		PF00071.28	GO:005104	cytoplasmic	NP_001297	mmu:100972	
K00545	COMT	mmu00350(	PF01596.23	GO:003323	ER	NP_001104	mmu:12846	
K23735	LIPT2, LIP	mmu01240(	PF21948.1(	GO:000989	peroxisoma	NP_080286	mmu:67164	
K18462	FAM21	mmu04144(	PF15255.11	GO:005112	nuclear	NP_080861	mmu:28006	
K00876	udk, UCK	mmu00983(	PF00485.24	GO:000921	cytoplasmic	NP_001404	mmu:80914	
K05399	LBP	mmu04620(	PF02886.23	GO:000959	ER	NP_032515	mmu:16803	
K08789	MAST, IREH1		PF08926.16	GO:003563	nuclear	XP_030103	mmu:328329	
K11864	BRCC3, BR	mmu03440(	PF18110.6(	GO:008009	cytoplasmic	NP_001159	mmu:210766	
251N(Nuclear transcription factor Y			PF01166.23	GO:008009	cytoplasmic	NP_001297	mmu:21807	TSC22
K02159	BAX	mmu05020(	PF00452.24	GO:200123	cytoplasmic	NP_001398	mmu:12028	
K13509	AGPAT1_2	mmu00564(	PF01553.26	GO:000665	ER	NP_080488	mmu:67512	
K10375	TPM4	mmu04260(	PF00261.25	GO:003243	cytoplasmic	XP_006511	mmu:326618	
K16525	AKAP8		PF04988.17	GO:000810	nuclear	XP_006524	mmu:56399	
K06752	MHC2	mmu05323(	PF07654.21	GO:001973	plasma mer	BAQ55489.	mmu:14969	

K06173	truA, PUS1		PF01416.25	GO:005125	mitochondr	NP_062674	mmu:56361	
.9U4(Coiled-coil domain containing			PF14988.11	(DUF4515:I	cytoplasmic	NP_997163	mmu:403180	
K26808	TXLN		PF09728.14	GO:000367	cytoplasmic	NP_849266	mmu:353170	
K17506	PPM1L, PP2CE		PF00481.26	GO:000548	ER	NP_848841	mmu:242083	
K25081	RBM45		PF00076.28	GO:003015	cytoplasmic	NP_700454	mmu:241490	
)N6G(chromosome 12 open reading			PF17720.7(I	GO:003563	cytoplasmic	NP_780337	mmu:68281	
K13109	IK, RED, RER		PF07808.18	GO:000810	nuclear	BAC40070.1	mmu:24010	
K16587	HAUS4, AUG4		PF14735.11	GO:002260	cytoplasmic	NP_663437	mmu:219072	
K23309	ZNHIT3		PF21373.2(	GO:003303	cytoplasmic	NP_001005	mmu:448850	
K03926	cutA		PF03091.20	GO:000810	plasma mer	NP_081224	mmu:67675	
K23560	ZEB2	mmu05206(	PF13465.12	GO:003514	nuclear	NP_001276	mmu:24136	zf-C2H2
K05702	AF6, MLLT	mmu04530(	PF00788.29	GO:000810	nuclear	NP_034936	mmu:17356	
K12231	HECTD1		PF00632.30	GO:000367	cytoplasmic	NP_659037	mmu:207304	
K06243	LAMB2	mmu05165(	PF00053.31	GO:004867	extracellulo	NP_001398	mmu:16779	
K04905	KCNH2, KV11.1		PF00520.37	GO:004239	plasma mer	NP_001281	mmu:16511	
K10643	CNOT4, N	mmu03018(	PF14570.12	GO:005123	cytoplasmic	NP_001157	mmu:53621	
K23168	MPAN		PF20721.2(	GO:000578	cytoplasmic	NP_001078	mmu:72244	
K26242	RCOR2_3		PF20878.2(I	GO:000989	nuclear	NP_001307	mmu:10438	MYB
K06553	VPREB, CD179a		PF07686.23	GO:000237	mitochondr	AAO18226.	mmu:22363	
.RNZ(Sjogren syndrome scleroderm			PF06677.18	(Auto_anti-]	cytoplasmic	NP_065237	mmu:56390	
K01540	ATP1B, CD	mmu04971(	PF00287.24	GO:000810	plasma mer	NP_033851	mmu:11931	
K27587	JPT, HN		PF17054.10	GO:000573	cytoplasmic	NP_032284	mmu:15374	
K12479	VPS45	mmu04144(	PF00995.28	GO:000802	cytoplasmic	NP_038869	mmu:22365	
K23883	EPG5	mmu04140(	Autophagy	GO:001604	cytoplasmic	NP_001182	mmu:100502841	
K01275	CTSC	mmu04142(	PF00112.29	GO:000425	lysosomal	NP_034112	mmu:13032	
K18648	ANP32E		PF12799.13	GO:004239	cytoplasmic	BAC36147.1	mmu:66471	
K06128	LYPLA1	mmu05231(	PF02230.22	GO:000578	cytoplasmic	NP_032892	mmu:18777	
K11422	SETD1, SE	mmu00310(	PF00856.33	GO:004205	nuclear	NP_001035	mmu:208043	
K17594	PHACTR		PF02755.20	GO:000548	cytoplasmic	BAC33611.1	mmu:100169	
K18085	MTMR10_11_12		PF06602.19	GO:001679	cytoplasmic	NP_766546	mmu:268783	
K25176	ZNF451		PF11560.13	GO:000382	cytoplasmic	BAC65823.1	mmu:98403	
K15449	TYW1		PF00258.30	GO:000548	ER	NP_001015	mmu:100929	
K12396	AP3D	mmu04142(	PF01602.26	GO:009951	nuclear	NP_031486	mmu:11776	
K02325	POLE2	mmu03030(	PF04042.21	GO:199023	mitochondr	NP_035263	mmu:18974	
K12390	CLN5	mmu04142(	PF15014.11	GO:000578	ER	NP_001028	mmu:211286	
K03160	TNFRSF5,	mmu04672(	PF00020.24	GO:003265	plasma mer	NP_035741	mmu:21939	
K12487	GIT2	mmu05135(	PF12205.13	GO:006062	cytoplasmic	NP_062808	mmu:26431	
K08734	MLH1	mmu03460(	PF16413.10	GO:003238	cytoplasmic	AAH21815.	mmu:17350	
K10728	TOPBP1	mmu03440(	PF00533.33	GO:000810	nuclear	NP_795953	mmu:235559	
K22155	JADE2		PF13832.12	GO:008009	nuclear	NP_001344	mmu:76901	
K05169	SIGIRR		PF01582.25	GO:001602	cytoplasmic	NP_001341	mmu:24058	
K15620	GOLPH3, GPP34		PF05719.16	GO:003303	cytoplasmic	EDL38823.	mmu:229593	
K12482	RUFY1_2, I	mmu04144(	PF02759.24	GO:000810	cytoplasmic	NP_766145	mmu:216724	

K16747	BBS2		PF14783.12	GO:000810	mitochondr	NP_080392	mmu:67378	
K03253	EIF3B		PF08662.17	GO:002260	cytoplasmic	AAH23767.	mmu:27979	
K26142	NOPCHAP1		PF15370.11	GO:002260	nuclear	NP_080855	mmu:28109	
K20360	TBC1D22, GYP1		PF00566.25	GO:003023	cytoplasmic	NP_941049	mmu:381085	
K07375	TUBB	mmu05020(	PF00091.31	GO:000520	cytoplasmic	NP_080749	mmu:67951	
.VQ7(limb bud and heart developm			PF15317.11	GO:200073	cytoplasmic	NP_084275	mmu:77889	
K06279	SHC1	mmu05226(	PF00640.29	GO:004340	cytoplasmic	NP_001106	mmu:20416	
K15446	TRM13, CCDC76		PF05206.20	GO:000817	cytoplasmic	BAE20598.1	mmu:229780	
K21668	CD200R	mmu05167(	PF07686.23	GO:000989	extracellulo	NP_067300	mmu:57781	
K19673	TTC21B, IFT139B		PF25060.1(	GO:000858	cytoplasmic	NP_001277	mmu:73668	
K08013	SIPA1	mmu04670(	PF02145.21	GO:000338	cytoplasmic	NP_001157	mmu:20469	
ZY5B(leucine zipper and CTNNBIP			PF06384.16	GO:000962	cytoplasmic	NP_001343	mmu:69151	
K20181	VPS18, PEI	mmu04140(	PF05131.20	GO:000810	cytoplasmic	BAE28329.1	mmu:228545	
K12004	TRIM14		PF00622.36	GO:009858	cytoplasmic	EDL02365.	mmu:74735	
K25095	SLIRP		PF00076.28	GO:008009	mitochondr	NP_081234	mmu:380773	
K07922	RAB36		PF00071.28	GO:003563	cytoplasmic	BAE20645.1	mmu:76877	
K27569	EVA1		PF14851.11	GO:000557	plasma mei	NP_742157	mmu:230752	
K17428	MRPL47, NCM1		PF06984.18	GO:001593	mitochondr	NP_083293	mmu:74600	
K11278	NPM3		PF03066.21	GO:004239	cytoplasmic	BAE33994.1	mmu:18150	
K12495	IQSEC	mmu04144(	PF01369.25	GO:190189	nuclear	NP_001395	mmu:232227	
20BN(adipogenesis associated, Mth9			PF04430.19	GO:003277	plasma mei	NP_001171	mmu:66273	
K19574	OBSL1		PF07679.22	GO:002260	cytoplasmic	NP_849215	mmu:98733	
K18750	APOBEC3	mmu03250(	PF18782.7(	GO:003577	cytoplasmic	ACH89410.	mmu:80287	
K04532	NAE1, APF	mmu05010(	PF00899.27	GO:004352	cytoplasmic	NP_001413	mmu:234664	
K15162	MED12	mmu04919(	PF12145.13	GO:004423	cytoplasmic	NP_067496	mmu:59024	
K09228	KRAB		PF13465.12	GO:000097	cytoplasmic	DAA01851.	mmu:43170	zf-C2H2
K04510	VANGL	mmu04310(	PF06638.16	GO:004347	cytoplasmic	BAE22466.1	mmu:229658	
K06259	CD36	mmu04975(	PF01130.26	GO:190148	ER	NP_001153	mmu:12491	
K00850	pfkA, PFK	mmu00052(	PF00365.25	GO:004643	cytoplasmic	NP_001278	mmu:56421	
K06630	YWHAE	mmu04114(	PF00244.26	GO:003315	cytoplasmic	NP_001006	mmu:22627	
K10576	UBE2H, UI	mmu04120(	PF00179.31	GO:000020	cytoplasmic	NP_001025	mmu:22214	
K10575	UBE2G1, U	mmu04141(	PF00179.31	GO:004438	cytoplasmic	NP_001074	mmu:67128	
K05124	DDR1, NEP, CD167a		PF07714.23	GO:006130	ER	AAB28212.	mmu:12305	
K13026	DHX57		PF07717.22	GO:000367	cytoplasmic	NP_001157	mmu:106794	
K01276	DPP7		PF05577.18	GO:000417	lysosomal	NP_114031	mmu:83768	
K01711	gmd, GMD	mmu00520(	PF16363.11	GO:001967	cytoplasmic	NP_666153	mmu:218138	
K12491	AGAP	mmu04144(	PF01412.23	GO:000810	cytoplasmic	NP_001032	mmu:347722	
K03145	TFIIS		PF07500.19	GO:004423	nuclear	NP_035671	mmu:21399	
K15697	RNF114		PF05605.18	GO:000020	cytoplasmic	NP_001347	mmu:81018	
K05917	CYP51	mmu00100(	PF00067.27	GO:000449	ER	NP_064394	mmu:13121	
K23113	SMCHD1		PF22899.2(	GO:008009	cytoplasmic	NP_083163	mmu:74355	
K10028	DDT		PF01187.23	GO:001922	cytoplasmic	NP_034157	mmu:13202	
Z9AV(Transmembrane protein 11)			PF14972.11	GO:000699	cytoplasmic	NP_001161	mmu:216821	

K14686	SLC31A1, C	mmu04978(	PF04145.20	GO:003000	cytoplasmic	BAE41687.1	mmu:20529	
K13960	UBE2T, HS	mmu03460(	PF00179.31	GO:003551	nuclear	NP_001265	mmu:67196	
K22517	CBLB	mmu04012(	PF02262.21	GO:004213	cytoplasmic	NP_001028	mmu:208650	
K20286	TMF1		PF12325.13	GO:000578	nuclear	NP_001074	mmu:23228	TF_others
MIS18 binding protein 1)			PF13855.12	GO:001906	extracellula	NP_083249	mmu:74488	
K24675	ZMYM2_3_4		PF12012.13	GO:002260	cytoplasmic	NP_001107	mmu:67785	
K15152	MED21, SRB7		PF11221.13	GO:008009	nuclear	NP_079591	mmu:108098	
K13624	CP	mmu04216(	PF07731.20	GO:004706	ER	NP_001396	mmu:12870	
K02736	PSMB4	mmu05020(	PF00227.31	GO:003210	cytoplasmic	NP_032971	mmu:19172	
K02943	RP-LP2, RF	mmu05171(	PF00428.24	GO:001593	cytoplasmic	NP_001347	mmu:67186	
K07375	TUBB	mmu05020(	PF00091.31	GO:003577	cytoplasmic	NP_001027	mmu:22154	
X10(GTP cyclohydrolase I feedbacl			PF06399.18	GO:002260	cytoplasmic	NP_796131	mmu:320415	
K23490	CYB5		PF00173.33	GO:000663	cytoplasmic	NP_001335	mmu:109672	
K22074	NFU1, HIRIP5		PF08712.16	GO:002260	mitochondr	NP_001164	mmu:56748	
K00416	QCR6, UQ(	mmu05208(	PF02320.22	GO:000812	cytoplasmic	NP_079917	mmu:66576	
K11187	PRDX5	mmu04146(	PF08534.16	GO:000578	mitochondr	NP_036151	mmu:54683	
K17964	LRPPRC		PF13041.12	GO:009951	mitochondr	NP_082509	mmu:72416	
K12593	MPHOSPH	mmu03018(	PF10175.14	GO:009030	nuclear	NP_081034	mmu:68533	
K05768	GSN	mmu04810(	PF00626.27	GO:000806	cytoplasmic	BAD32573.	mmu:20259	
K09659	DPM3	mmu00510(	PF08285.16	GO:000578	ER	NP_081043	mmu:68563	
K10403	KIF22	mmu04914(	PF00225.29	GO:009951	nuclear	NP_663563	mmu:110033	
ZK0J(ring finger protein 214)			PF24525.1(	GO:001978	cytoplasmic	NP_001348	mmu:235315	
K09399	FOXF		PF00250.24	GO:008009	nuclear	CAA72035.	mmu:14238	Fork head
K21870	BANF		PF02961.22	GO:001922	nuclear	NP_001033	mmu:23825	
K01283	ACE, CD14	mmu05171(	PF01401.24	GO:000167	ER	NP_997507	mmu:11421	
K20723	RTN3	mmu05010(	PF02453.23	GO:002260	cytoplasmic	NP_001003	mmu:20168	
K10594	HERC1	mmu04120(	PF00415.24	GO:000989	cytoplasmic	NP_663592	mmu:235439	
Allows the formation of correctly ch			PF13465.12	GO:005125	cytoplasmic	NP_082574	mmu:72611	zf-C2H2
K20635	ARHGAP11		PF00620.32	GO:004322	nuclear	NP_852081	mmu:228482	
K20164	DENND5		PF02141.26	GO:190588	cytoplasmic	NP_796166	mmu:320560	
K08900	BCS1		PF08740.16	GO:000810	cytoplasmic	NP_001292	mmu:66821	
K17908	WIPI1_2, A	mmu04136(	PF21032.3(I	GO:000040	cytoplasmic	XP_020032	mmu:74781	
K10362	MYO18	mmu04814(	PF00063.27(Myosin_he		nuclear	XP_036012	mmu:360013	
K06697	PSME2	mmu04612(	PF02252.23	GO:000548	cytoplasmic	NP_001268	mmu:621823	
ZDYG(bri3 binding protein)			PF14965.11	GO:003196	ER	NP_084028	mmu:76809	
K16770	CNTRL		PF14580.12(LRR_9:Leu		cytoplasmic	NP_001366	mmu:26920	
K06553	VPREB, CD179a		PF07686.23	GO:000237	extracellula	CAB46155.1	mmu:22363	
R78(ribosomal RNA processing 15			PF07890.17	GO:001607	nuclear	NP_080317	mmu:67223	
coiled-coil domain containing 40)			PF00849.27	GO:003111	cytoplasmic	NP_001344	mmu:106707	
K27498	FOCAD		PF11229.14	GO:000557	cytoplasmic	XP_036019	mmu:230393	
K20365	ERGIC1		PF07970.17	GO:000578	cytoplasmic	KAF608105	mmu:67458	
K01393	NLN	mmu04614(	PF01432.26	GO:000417	mitochondr	NP_083723	mmu:75805	
K21249	UVRAG	mmu04140(	PF10186.14	GO:000578	cytoplasmic	BAC31454.1	mmu:78610	

K25191	BCDIN3D		PF06859.18	GO:000989	cytoplasmic	NP_083512	mmu:75284	
K10522	DIABLO, S	mmu04215(	PF09057.15	GO:003563	mitochondr	NP_075721	mmu:66593	
YFY9(endothelial cell adhesion mol			PF13911.12	GO:004322	extracellul	NP_001303	mmu:70564	
K06099	CSDA, ZOI	mmu04530(	PF00313.28	GO:200123	nuclear	NP_620817	mmu:56449	CSD
K05609	UCHL3, YUH1		PF01088.27	GO:007149	cytoplasmic	NP_057932	mmu:50933	
K00797	speE, SRM,	mmu00270(	PF01564.23	GO:000659	cytoplasmic	BAE27138.	mmu:20810	
K15615	SIX4	mmu05202(	PF16878.10	GO:000810	nuclear	NP_035512	mmu:20474	Homeobox
K14537	NUG2, GN	mmu03008(	PF08153.17	GO:003563	nuclear	BAE27145.	mmu:230737	
K24420	LASP1		PF00880.23	GO:004322	cytoplasmic	NP_034818	mmu:16796	
K05024	CLIC4		PF22441.2(	GO:003000	ER	NP_038913	mmu:29876	
K17906	ATG2	mmu04136(	PF13329.13	GO:003472	nuclear	NP_083930	mmu:76559	
K20123	PACSIN		PF00611.28	GO:005104	cytoplasmic	NP_001276	mmu:80708	
K01425	glsA, GLS	mmu04724(	PF04960.20	GO:005128	mitochondr	AAM00020.	mmu:14660	
K11875	PSMG1, DSCR2, PAC1		PF16094.10	GO:002192	cytoplasmic	NP_062410	mmu:56088	
K01883	CARS, cysS	mmu00970(	PF01406.26	GO:003563	mitochondr	NP_001346	mmu:71941	
K10449	KLHL11		PF00651.37(BTB:BTB/I		cytoplasmic	NP_766153	mmu:21719	ZBTB
K06553	VPREB, CD179a		PF07686.23	GO:000237	cytoplasmic	P01664.1(R	mmu:22364	
K06553	VPREB, CD179a		PF07686.23	GO:000237	cytoplasmic	P01660.1(R	mmu:22363	
K06553	VPREB, CD179a		PF07686.23	GO:000237	mitochondr	P01662.1(R	mmu:22363	
K17492	UBN		PF14075.11	GO:003472	nuclear	NP_080942	mmu:170644	
K27702	SPIN		PF02513.24	GO:003506	nuclear	NP_001269	mmu:20729	
K01369	LGMN	mmu04142(	PF01650.23	GO:001922	extracellul	NP_001365	mmu:19141	
K14312	NUP155, N	mmu03013(	PF08801.17	GO:003450	plasma mer	BAC31782.	mmu:170762	
K04619	GPRC5B		PF00003.28	GO:001922	ER	NP_001182	mmu:64297	
K20290	COG3, SEC34		PF20671.3(	GO:003303	cytoplasmic	NP_796355	mmu:338337	
K17560	URI1		PF02996.22	GO:200123	ER	XP_006539	mmu:19777	
K14345	SLCO2A		PF03137.26	GO:009859	plasma mer	NP_201571	mmu:24059	
K08851	TP53RK, PRPK, BUD3		PF06293.20	GO:004316	cytoplasmic	NP_076304	mmu:76367	
K12614	DDX6, RCl	mmu03018(	PF00270.35	GO:003577	cytoplasmic	EDL25604.	mmu:13209	
K01756	purB, ADSI	mmu01240(	PF00206.25	GO:000401	cytoplasmic	NP_033764	mmu:11564	
YQB2(immunoglobulin kappa varia			PF07686.23(V-set:Immu		extracellul	CAB46309.1(immunoglobulin light		
.F4W(RNA binding motif protein 4			PF00076.28	GO:000613	nuclear	NP_001277	mmu:237073	
K08487	STX11	mmu04130(	PF00804.31	GO:000810	cytoplasmic	AAI17540.1	mmu:74732	
K05463	EFNB	mmu04360(	PF00812.23	GO:000716	ER	NP_034240	mmu:13641	
K05055	IL6R, CD12	mmu05171(	PF09240.16	GO:001922	lysosomal	NP_001297	mmu:16194	
K03236	EIF1A		PF01176.25	GO:002260	cytoplasmic	NP_001171	mmu:666806	
K12751	MYL6	mmu04530(	PF13499.12	GO:001470	cytoplasmic	NP_758463	mmu:216459	
K08458	ADGRF5, GPR116		PF00002.29	GO:001922	cytoplasmic	NP_001391	mmu:224792	
K05076	IL13RA1, C	mmu05200(	PF18001.7(I	GO:000548	plasma mer	NP_598751	mmu:16164	
K08738	CYC	mmu05020(	PF00034.26	GO:003197	cytoplasmic	NP_031834	mmu:13063	
K02924	RP-L39e, R	mmu05171(	PF00832.25	GO:002262	mitochondr	NP_000991	mmu:67248	
K12397	AP3B	mmu04142(	PF01602.26	GO:004323	cytoplasmic	XP_006540	mmu:11775	
XR79(Family with sequence similari			PF14944.11	GO:000989	cytoplasmic	XP_020142	mmu:319604	

K14282	TACC2		PF05010.19	GO:000367	cytoplasmic	BAE26610.1	mmu:57752	
K09428	ELF1_2_4		PF12310.13	GO:008009	nuclear	AAI09161.1	mmu:56501	ETS
K08549	NR2F6, EAR2		PF00104.35	GO:008009	nuclear	NP_034280	mmu:13864	RXR-like
K08548	NR2F2, TFCOUP2		PF00104.35	GO:008009	nuclear	CAA54096.	mmu:11819	RXR-like
K21772	PURA		PF04845.19	GO:008009	nuclear	NP_001387	mmu:19290	TF_others
K00922	PIK3CA_B	mmu05208(	PF00454.33	GO:200123	cytoplasmic	AAI27074.1	mmu:74769	
K07249	ALDH1A	mmu00830(	PF00171.27	GO:003510	cytoplasmic	NP_033048	mmu:19378	
K25039	PAQR7, MI	mmu05207(	PF03006.26	GO:003802	cytoplasmic	NP_001272	mmu:71904	
K03357	APC10, AN	mmu04110(	PF03256.22	GO:199023	cytoplasmic	ACD47054.	mmu:68999	
K09228	KRAB		PF13465.12	GO:008009	nuclear	NP_059495	mmu:54201	zf-C2H2
K22825	NSMCE4, NSE4		PF08743.15	GO:199023	nuclear	NP_001156	mmu:67872	
K15041	VDAC3	mmu05208(	PF01459.28	GO:000802	cytoplasmic	NP_001185	mmu:22335	
ZJ02(kiaa1467)			PF00595.29	GO:005113	cytoplasmic	NP_001297	mmu:68760	
K19945	TBC1D17	mmu04137(	PF00566.25	GO:000810	cytoplasmic	BAE32159.1	mmu:233204	
K23410	HRG			GO:003362	extracellula	NP_444406	mmu:94175	
K03898	KNG, BK	mmu04750(	PF00031.27	GO:004213	extracellula	NP_001095	mmu:16644	
K11503	CENPK		PF11802.13	GO:002260	cytoplasmic	NP_068562	mmu:60411	
K04573	NEF3, NF-	mmu05014(	PF00038.26	GO:009951	cytoplasmic	NP_032717	mmu:18040	
KYFU(pyridoxal-dependent decarbo			PF22937.1(I	GO:000548	cytoplasmic	NP_001277	mmu:94184	
K04000	C9	mmu05171(	PF01823.24	GO:002260	ER	AAH11137.	mmu:12279	
K12012	TRIM35		PF00622.36	GO:000548	cytoplasmic	NP_084255	mmu:66854	
K15053	CHMP7	mmu03272(	PF03357.26	GO:000810	cytoplasmic	NP_598839	mmu:105513	
K02330	POLB	mmu05203(	PF14792.12	GO:009951	cytoplasmic	NP_035260	mmu:18970	
K20819	BORCS5, LOH12CR1		PF10158.14	GO:000587	mitochondr	NP_001163	mmu:67774	
K12258	GC		PF00273.25	GO:000548	extracellula	NP_032122	mmu:14473	
ORNT(cDNA 2200002D01 gene); EN			PF15761.10	GO:000557	nuclear	AAM54028.	mmu:72275	
netallo-sulfur cluster assembly)			PF02906.19	GO:009951	cytoplasmic	NP_080548	mmu:67608	
K11511	APITD1, C	mmu03460(	PF15630.12	GO:190304	nuclear	AAI38927.1	mmu:69928	
K01672	CA	mmu00910(	PF00194.26	GO:001615	cytoplasmic	NP_031632	mmu:12350	
solute carrier family 25)			PF13465.12	GO:008009	nuclear	BAC27823.1	mmu:77652	zf-C2H2
K16809	CEP162			GO:002260	cytoplasmic	AAI51105.1	mmu:382090	
K06529	SEMA7, CD	mmu04360(	PF01403.25	GO:004867	lysosomal	NP_035482	mmu:20361	
K18027	PTPN3, PTPH1		PF00102.32	GO:001679	cytoplasmic	XP_006538	mmu:545622	
K19178	HELQ		PF21099.2(I	GO:008009	nuclear	NP_001074	mmu:191578	
K15104	SLC25A11, OGC		PF00153.32	GO:001572	mitochondr	NP_077173	mmu:67863	
K27381	XIRP	mmu04820(	PF08043.17	GO:003243	cytoplasmic	XP_030100	mmu:22437	
K00913	ITPK1	mmu04070(	PF05770.16	GO:009859	cytoplasmic	NP_766172	mmu:217837	
YQ2G(thioredoxin-like protein)			PF06201.19	GO:000582	cytoplasmic	XP_028713	mmu:53382	
K01490	AMPD	mmu01100(	PF19326.5(	GO:004846	cytoplasmic	NP_001333	mmu:109674	
.QQ2(Coiled-coil domain containin			PF09747.14	GO:004323	nuclear	NP_083047	mmu:52132	
K27355	NEMP, TMEM194		PF10225.14	GO:000563	plasma mer	EDK99977.	mmu:227094	
K19680	TRAF3IP1, IFT54		PF17749.6(	GO:000858	nuclear	XP_006529	mmu:74019	
K05410	TBK1	mmu05022(	PF18394.6(	GO:008009	cytoplasmic	EDL24421.1	mmu:56480	

K04438	CRK, CRK	mmu04012(	PF07653.23	GO:003288	cytoplasmic	NP_598417	mmu:12928	
K20095	RNMTL1		PF00588.24	GO:004280	mitochondr	NP_899086	mmu:67390	
K25406	MFAP2		PF05507.16	GO:004859	extracellulo	AJT39497.1	mmu:17150	
K14736	TF	mmu04978(	PF00405.22	GO:001922	lysosomal	NP_598738	mmu:22041	
K21763	MAPKBP1		PF24780.1(	GO:003210	cytoplasmic	NP_036071	mmu:26390	
K07374	TUBA	mmu04530(	PF00091.31	GO:000520	cytoplasmic	XP_007445	mmu:22145	
K19788	OLA1		PF06071.19	GO:003563	cytoplasmic	NP_080218	mmu:67059	
K03106	SRP54, ffh	mmu03060(	PF00448.28	GO:000578	cytoplasmic	NP_001093	mmu:100101806	
K09106	USF		PF00010.31	GO:008009	nuclear	XP_025224	mmu:22282	bHLH
K17086	TM9SF2_4		PF02990.21	GO:000810	Golgi appa	NP_542123	mmu:68059	
K16686	SAV1, Sav	mmu04390(	PF00397.32	GO:008009	cytoplasmic	NP_071311	mmu:64010	
K00280	LOXL2_3_4		PF01186.22	GO:000578	plasma mer	NP_201582	mmu:94352	
K06129	LYPLA3	mmu04148(	PF02450.21	GO:000548	extracellulo	NP_598553	mmu:192654	
K06534	MCAM, CD146		PF13927.12	GO:000989	plasma mer	NP_075548	mmu:84004	
K24669	PADI4, PA	mmu04613(	PF03068.20	GO:003472	cytoplasmic	NP_035191	mmu:18602	
K12185	VPS37	mmu04144(	PF07200.18	GO:004323	cytoplasmic	NP_808544	mmu:330192	
K16867	NUDT16L1	mmu05205(	PF22327.2(	GO:000989	nuclear	NP_080115	mmu:66911	
K24943	TTC39		PF10300.15	GO:003250	nuclear	NP_082617	mmu:72747	
K03217	yidC, spoIII	mmu03060(	PF02096.25	GO:000573	mitochondr	BAE42101.	mmu:69089	
K22533	LINS1		PF14694.11	GO:000815	cytoplasmic	NP_690028	mmu:72635	
K00851	idnK, gntK	mmu00030(	PF01202.28	GO:003563	cytoplasmic	NP_932121	mmu:75731	
K02434	gatB, PET1	mmu00970(	PF02934.20	GO:003563	mitochondr	BAE38646.	mmu:229487	
K14165	K14165		PF00782.25	GO:001679	mitochondr	XP_021033	mmu:56405	
K17975	KTN1		PF05104.17	GO:000810	ER	NP_001334	mmu:16709	
K00902	DOLK	mmu00510(	N-Glycan b	GO:000578	ER	NP_808316	mmu:227697	
K03540	RPR2, RPP21		PF04032.21	GO:000454	cytoplasmic	KAF636522	mmu:67676	
K09053	ATF1	mmu05202(	PF00170.26	GO:009057	nuclear	NP_001397	mmu:11908	TF_bZIP
K17597	STAU		PF16482.11	GO:000810	cytoplasmic	BAE26678.	mmu:20853	
K23398	TRIP4		PF23135.1(	GO:008009	nuclear	NP_001344	mmu:56404	
K01425	glsA, GLS	mmu04724(	PF04960.20	GO:004428	cytoplasmic	XP_031507	mmu:14660	
K15106	SLC25A14_30		PF00153.32	GO:004858	cytoplasmic	NP_001277	mmu:20523	
K13299	GSTK1	mmu00980(	PF01323.26	GO:003015	peroxisoma	NP_083831	mmu:76263	
)DRD(phosducin)			PF02114.22	GO:000858	cytoplasmic	BAE21820.	mmu:67466	
K11256	NCOA3, A(	mmu05200(	PF16665.10	GO:200073	nuclear	NP_001361	mmu:17979	
K05810	LACC1, yfi	mmu00270(	PF02578.20	GO:001922	cytoplasmic	NP_766076	mmu:210808	
K06637	BUB1B, BU	mmu05166(	PF08311.18	GO:000810	nuclear	NP_033903	mmu:12236	
K20639	ARHGAP18_28_40		PF00620.32	GO:002260	cytoplasmic	SPT35689.1	mmu:73910	
K01339	KLK16		PF00089.32	GO:000425	lysosomal	NP_032480	mmu:16615	
K01325	KLK1_2	mmu04614(	PF00089.32	GO:000425	lysosomal	SFW93237.	mmu:16619	
K25815	SUB1		PF02229.22	GO:008009	nuclear	NP_001398	mmu:20024	PC4
K19879	TCAP	mmu04820(	PF09470.15	GO:003514	cytoplasmic	NP_035670	mmu:21393	
K01352	GZMA	mmu04080(	PF00089.32	GO:000425	extracellulo	NP_034500	mmu:14938	
K09557	BAG3		PF02179.21	GO:003243	cytoplasmic	AAI45766.1	mmu:29810	

K11447	UTX, KDM	mmu05202(	PF21322.2(I	GO:200123	cytoplasmic	NP_001297	mmu:22289	
K08045	ADCY5	mmu04750(	PF16214.10	GO:009008	cytoplasmic	NP_001012	mmu:224129	
K23487	EVL	mmu04015(	PF00568.29	GO:003243	cytoplasmic	NP_001156	mmu:14026	
K23167	VAT1		PF13602.12	GO:003304	cytoplasmic	BAE34983.1	mmu:26949	
K08821	CDK14, PF	mmu05202(	PF00069.31	GO:003563	cytoplasmic	NP_001411	mmu:18647	
K12327	CALD1	mmu04270(	PF02029.20(Caldesmon		cytoplasmic	WEL28135.	mmu:109624	
K07838	RAP2B		PF00071.28	GO:000810	cytoplasmic	NP_001116	mmu:74012	
K09595	HM13		PF04258.18	GO:000417	ER	XP_008993	mmu:14950	
K06174	ABCE1, Rli1		PF00005.33	GO:005116	cytoplasmic	NP_001035	mmu:24015	
K00688	PYG, glgP	mmu01100(	PF00343.26	GO:000818	cytoplasmic	AAH13636.	mmu:110095	
K23798	ARHGEF39		PF00621.25	GO:000508	mitochondr	XP_027470	mmu:230098	
K04549	GNGT2	mmu04724(	PF00631.27	GO:001678	cytoplasmic	NP_001033	mmu:14710	
K18026	PTPN2, PT	mmu04630(	PF00102.32	GO:004340	cytoplasmic	NP_001120	mmu:19255	
K06696	PSME1	mmu04612(	PF02252.23	GO:000989	cytoplasmic	NP_035319	mmu:19186	
K02129	ATPeF0E, A	mmu04714(	PF05680.17	GO:000526	mitochondr	NP_031533	mmu:11958	
K03959	NDUFB3	mmu05208(	PF08122.17	GO:002260	cytoplasmic	NP_079873	mmu:66495	
K21343	USP15	mmu04137(	PF00443.35	GO:200123	cytoplasmic	NP_081880	mmu:14479	
K09030	FOSL2	mmu04380(	PF00170.26	GO:008009	nuclear	NP_032063	mmu:14284	TF_bZIP
K02561	BOK	mmu04215(	PF00452.24	GO:200123	cytoplasmic	NP_058058	mmu:51800	
K25304	RPC9, CRC	mmu03020(	PF03874.21	GO:003042	cytoplasmic	NP_031787	mmu:12909	
XP3J(glyoxalase domain containing			PF21701.3((	GO:004323	mitochondr	NP_080305	mmu:67201	
K17423	MRPL42		PF10210.14	GO:001593	mitochondr	NP_001346	mmu:67270	
K25229	APC16, AN	mmu04110(	PF17256.7(A	GO:199023	cytoplasmic	NP_001124	mmu:52717	
K26094	SKA1		PF07160.18	GO:003111	cytoplasmic	NP_001157	mmu:66468	
K08132	COL12A	mmu04974(	PF00041.27	GO:003250	plasma mer	Q60847.3(R	mmu:12816	
K22471	CCN4, WIS	mmu04310(	PF00219.23	GO:001922	extracellulo	XP_020144	mmu:22402	
ZNK1(stress granule assembly)			PF11977.14	GO:000548	cytoplasmic	EDL36230.1	mmu:219094	
K01062	PLA2G7, P.	mmu00565(	PF03403.19	GO:000578	cytoplasmic	NP_001272	mmu:100163	
K17569	GPATCH2			GO:004323	nuclear	BAE28226.1	mmu:70373	
K01870	IARS, ileS	mmu00970(	PF00133.28	GO:000216	mitochondr	NP_941055	mmu:381314	
K19001	HELLS, DDM1		PF00176.29	GO:200123	cytoplasmic	NP_032260	mmu:15201	
K08367	RUNX1, AI	mmu04530(	PF00853.24	GO:190303	mitochondr	NP_001104	mmu:12394	Runt
K11322	EPC	mmu03082(	PF06752.17	GO:003124	nuclear	NP_766251	mmu:227867	
K21643	BBX, HBP2		PF09667.15	GO:004885	nuclear	EDK98123.	mmu:70508	HMG
ZQGI(splicing factor, suppressor of			PF09750.14	GO:003311	nuclear	NP_758480	mmu:231769	
K10144	RCHY1, PII	mmu04115(	PF14599.11	GO:003243	cytoplasmic	NP_001258	mmu:68098	
K09186	MLL1	mmu04934(	PF00856.33	GO:003292	nuclear	P55200.3(R	mmu:214162	
K19765	HSBP1		PF06825.17	GO:001989	nuclear	NP_077181	mmu:68196	
K10803	XRCC1	mmu03410(	PF01834.22	GO:004423	nuclear	BAE40568.1	mmu:22594	
K08827	PRPF4B		PF00069.31	GO:000024	nuclear	EDL40894.1	mmu:19134	
20AA(Chromosome 16 open reading			PF15477.11	GO:004323	nuclear	NP_075686	mmu:66356	
ZV9V(family with sequence similari			PF20771.2(I	GO:002260	ER	NP_001074	mmu:269233	
K17096	ANXA8		PF00191.25	GO:007234	cytoplasmic	NP_038501	mmu:11752	

K12392	AP1B1	mmu04142(	PF01602.26	GO:000810	cytoplasmic	NP_031480	mmu:11764	
YE0(immunoglobulin heavy variab			PF07686.23	GO:000548	cytoplasmic	CCQ13178.1(IgG1 heavy chain VD		
K13206	CCDC55		PF09745.14	GO:005125	nuclear	AAH16601.	mmu:237859	
K08575	CAPN6		PF00648.27	GO:000417	cytoplasmic	NP_031629	mmu:12338	
K09108	TFAP4	mmu05205(	PF00010.31	GO:008009	nuclear	BAC35263.	mmu:83383	bHLH
K16490	SPICE1		PF15678.10	GO:002260	cytoplasmic	NP_001403	mmu:212514	
K19025	AP5Z1, SPG48		PF25154.1(	GO:004323	cytoplasmic	NP_766313	mmu:231855	
23ZA(Cannabinoid receptor interact			PF15043.11	GO:000573	cytoplasmic	EDL40678.	mmu:380686	
K06552	CD177, PRV1		PF00021.27	GO:009859	plasma mer	NP_081138	mmu:68891	
K01462	PDF, def		PF01327.26	GO:004212	mitochondr	NP_080789	mmu:68023	
K03950	NDUFA6	mmu05208(	PF05347.21	GO:002260	mitochondr	NP_080263	mmu:67130	
K09504	DNAJA3	mmu05203(	PF01556.23	GO:000810	mitochondr	NP_001128	mmu:83945	
K18749	LSM14, RAP55, SCD6		PF12701.13	GO:003577	nuclear	NP_080224	mmu:67070	
K11723	BRD7	mmu03082(	PF12024.13(DUF3512:I		nuclear	NP_001363	mmu:26992	
K07524	RGS3	mmu04360(	PF00615.24	GO:004858	cytoplasmic	XP_006538	mmu:50780	
K11800	DCAF5, BCRP2		PF00400.38	GO:004592	mitochondr	BAC65843.	mmu:320808	
K13340	PEX26	mmu04146(	PF07163.17	GO:000810	peroxisoma	NP_083006	mmu:74043	
K03002	RPA2, POL	mmu03020(	PF00562.33	GO:000699	nuclear	NP_033112	mmu:20017	
K06061	MAML	mmu05165(Human pap		GO:008009	mitochondr	NP_776137	mmu:270118	
K03966	NDUFB10	mmu05208(	PF10249.14	GO:002260	cytoplasmic	NP_080960	mmu:68342	
K03354	APC7, ANA	mmu04110(	PF07719.23	GO:001096	cytoplasmic	EDL19679.	mmu:56317	
K14862	URB2		PF10441.14	GO:004323	cytoplasmic	NP_001025	mmu:382038	
K07611	LMNB	mmu04210(	PF00038.26	GO:003450	nuclear	P21619.2(R	mmu:16907	
J4H(Chromosome 16 open reading			PF06080.18	GO:000998	cytoplasmic	NP_080962	mmu:68347	
K07512	MECR, NR	mmu01100(	PF00107.32	GO:000663	mitochondr	NP_079573	mmu:26922	
K19410	LEMD3		PF09402.16	GO:001602	nuclear	NP_001074	mmu:380664	
K06565	LAG3, CD223		PF13895.12	GO:001922	plasma mer	NP_032505	mmu:16768	
H6J(tumor protein p53 inducible p			PF14936.11	GO:000557	peroxisoma	EDL27617.	mmu:277414	
K27312	TEPSIN		PF01417.26	GO:001989	cytoplasmic	EDL34727.	mmu:78777	
K13106	BUD13, CWC26		PF09736.14	GO:007027	nuclear	KAF610233	mmu:215051	
K06235	ZIC2		PF18366.7(z	GO:003292	nuclear	NP_033600	mmu:22772	zf-C2H2
K17711	PTCD2		PF10037.15	GO:005125	mitochondr	NP_081149	mmu:68927	
K24625	APOO		PF09769.14	GO:000578	cytoplasmic	AAI07209.1	mmu:68316	
K10396	KIF5	mmu05020(	PF00225.29	GO:000810	cytoplasmic	BAC41428.	mmu:16574	
K23727	CERS5_6, I	mmu04071(	PF03798.21	GO:003250	ER	NP_082291	mmu:71949	Homeobox
K08833	MAP4K5, KHS1		PF00069.31	GO:003563	cytoplasmic	NP_958927	mmu:399510	
K03123	TFIIA2, GT	mmu05203(	PF02268.21	GO:006026	nuclear	TKC48816.	mmu:235459	
K15614	SIX1	mmu05202(	PF16878.10	GO:000810	cytoplasmic	NP_033215	mmu:20471	Homeobox
ZPSV(Synapse associated protein 1)			PF03909.22	GO:001922	cytoplasmic	BAE32869.	mmu:67043	
K12160	SUMO, SM	mmu03013(	PF11976.14	GO:000989	nuclear	NP_001005	mmu:170930	
K21766	TBCC		PF07986.17	GO:002260	cytoplasmic	NP_848472	mmu:72726	
K01897	ACSL, fadD	mmu01100(	PF00501.34	GO:009008	cytoplasmic	BAC35758.	mmu:50790	
K14951	ATP13A3_4_5		PF00122.25	GO:001508	cytoplasmic	NP_001158	mmu:224079	

K05753	WASF1	mmu05231(	PF02205.27	GO:004858	cytoplasmic	NP_001366	mmu:83767	
K25363	APOF		PF15148.11	GO:003435	extracellulo	AAH10815.	mmu:103161	
K04523	UBQLN, D	mmu04141(	PF23195.1(l	GO:000962	cytoplasmic	BAE43132.1	mmu:56085	
K08760	APOA4	mmu04979(	PF01442.23	GO:003444	extracellulo	NP_031494	mmu:11808	
K11861	VCPIP1, VCIP135		PF19437.4(	GO:002260	cytoplasmic	NP_775619	mmu:70675	
Y5R6(coiled-coil-helix-coiled-coil-he			PF06747.18	GO:001593	cytoplasmic	AAH49582.	mmu:66121	
linuclear metal center protein, YbgI			PF01784.23	GO:000989	mitochondr	NP_075364	mmu:65102	
K19951	TBC1D8_9		PF02893.26	GO:003023	cytoplasmic	NP_001344	mmu:54610	
K11798	BRWD1_3		PF00400.38	GO:002260	nuclear	NP_660107	mmu:93871	
IAF(coiled-coil domain containing 159)				GO:000512	cytoplasmic	AAH26773.	mmu:102545	
ZBE9(family with sequence similarity 120B)				GO:003250	nuclear	NP_001344	mmu:67544	
K10354	ACTA1	mmu04814(	PF00022.25	GO:003243	cytoplasmic	KAF725445	mmu:11459	
K11718	HUGT	mmu04141(	PF18404.7(	GO:000578	ER	NP_001074	mmu:66435	
K08547	NR2F1, TFCOUP1		PF00104.35	GO:008009	cytoplasmic	NP_001334	mmu:13865	
K18245	CA2	mmu04971(	PF00194.26	GO:009008	cytoplasmic	NP_001344	mmu:12349	
K18207	POMGNT2	mmu00515(	PF04577.19	GO:000578	ER	NP_001276	mmu:215494	
K06553	VPREB, CD179a		PF07686.23	GO:000237	extracellulo	CAB46298.1	mmu:22362	
CF7(IGv); ENOG41115I2(IGv); EN			PF07686.23(V-set:Immu		extracellulo	CAB46136.1(immunoglobulin light		
K06553	VPREB, CD179a		PF07686.23(V-set:Immu		extracellulo	CAB46140.1	mmu:22362	
K19944	TBC1D10		PF00566.25	GO:008009	cytoplasmic	NP_598784	mmu:103724	
K08846	RIPK2	mmu04621(	PF07714.23	GO:000270	cytoplasmic	NP_620402	mmu:192656	
K05754	ARPC5	mmu04530(	PF04699.19	GO:000520	cytoplasmic	BAE39116.1	mmu:67771	
K03880	ND3	mmu05208(	PF00507.24	GO:000916	ER	AHG32156.	mmu:17718	
K14563	NOP1, FBL	mmu03008(	PF01269.23	GO:000945	nuclear	NP_032017	mmu:14113	
K23898	RCN1		PF00036.39	GO:000578	ER	NP_033063	mmu:19672	
K22385	CAMLG		PF14963.11	GO:000810	cytoplasmic	NP_031622	mmu:12328	
K00907	MYLK	mmu04971(	PF07679.22	GO:003243	cytoplasmic	NP_647461	mmu:107589	
K03267	ERF3, GSP	mmu03015(	PF00009.33	GO:002262	cytoplasmic	NP_666178	mmu:14852	
K11094	SNRPB2	mmu03040(	PF00076.28	GO:003646	nuclear	NP_067310	mmu:20639	
K07885	RAB27A		PF00071.28	GO:000810	cytoplasmic	NP_001288	mmu:11891	
K04858	CACNA2D	mmu04010(	PF08473.17	GO:000526	ER	NP_001104	mmu:12293	
K26582	MIDEAS, ELMSAN1		PF01448.29	GO:000989	nuclear	NP_001156	mmu:238317	
K01309	MINDY1_2		PF04424.18	GO:010100	cytoplasmic	EDL26208.	mmu:235461	
K12562	AMPH	mmu04666(	PF03114.23	GO:009951	cytoplasmic	NP_033798	mmu:30948	
K05465	ANGPT1	mmu04010(	PF00147.23	GO:190560	plasma mer	NP_033770	mmu:11600	
K15196	BRF1, GTF3B		PF00382.24	GO:008009	nuclear	NP_082469	mmu:72308	
K08958	CSNK1G	mmu04340(	PF12605.13	GO:000548	cytoplasmic	NP_074046	mmu:70425	
K04381	STMN1	mmu04010(	PF00836.24	GO:003111	cytoplasmic	XP_031234	mmu:16765	
K16663	CD300LG, NEPMUCIN		PF07686.23	GO:009859	plasma mer	NP_001154	mmu:52685	
K18985	ELMO2, CI	mmu05135(	PF11841.14	GO:000690	cytoplasmic	NP_997589	mmu:140579	
K09054	ATF6A	mmu05022(	PF00170.26	GO:000578	nuclear	NP_001074	mmu:22664	TF_bZIP
K14970	MEN1, MN	mmu04934(	PF05053.19	GO:200073	nuclear	NP_001161	mmu:17283	
K22864	ARMC9		PF23138.1(	GO:000858	cytoplasmic	NP_084460	mmu:78795	

K15191	LARP7		PF08777.17	GO:008009	nuclear	NP_613059	mmu:28036	
K14314	NUP210, G	mmu03013(	PF22963.3(I	GO:001593	plasma mer	AAH52468.	mmu:54563	
K04369	MAP2K2, M	mmu05208(	PF00069.31	GO:009951	cytoplasmic	NP_075627	mmu:26396	
K14307	NUPL1, NU	mmu03013(	PF15967.10	GO:003315	cytoplasmic	BAE21799.1	mmu:71844	
K24467	PROZ		PF00089.32	GO:000425	cytoplasmic	NP_001344	mmu:66901	
ZHPX(BRO1 domain and CAAX m			PF03097.24	GO:003146	mitochondr	NP_001344	mmu:71678	
K00861	RFK, FMN	mmu01240(	PF01687.23	GO:000016	cytoplasmic	NP_062310	mmu:54391	
K11225	STAT6	mmu05161(	PF14596.11	GO:001989	cytoplasmic	NP_033310	mmu:20852	STAT
K15839	GPSM1, AG	mmu05030(	PF02188.22	GO:000989	cytoplasmic	NP_700459	mmu:67839	
Y9GN(phosphorylase kinase)			PF05879.18	GO:003563	cytoplasmic	NP_001229	mmu:100042856	
K15451	PPM2, LCMT2, TYW4		PF04072.20	GO:000151	mitochondr	NP_808514	mmu:329504	
K08791	LATS1_2, W	mmu04390(	PF00069.31	GO:003288	cytoplasmic	NP_034820	mmu:16798	
K11860	OTUD7A_B		PF02338.25	GO:008009	nuclear	NP_001020	mmu:229603	
K05658	ABCB1, CD	mmu02010(	PF00664.28	GO:014032	peroxisoma	NP_035205	mmu:18669	
K14538	NUG1, GN	mmu03008(	PF08701.16	GO:008009	nuclear	BAE39318.1	mmu:30877	
K17698	FYB, ADAI	mmu05135(	PF14603.12	GO:000810	nuclear	XP_006520	mmu:23880	
K11825	AP2B1	mmu05016(	PF01602.26	GO:000810	cytoplasmic	NP_001030	mmu:71770	
K02678	ETS1, pnt	mmu05200(	PF19525.4(I	GO:004864	nuclear	NP_035938	mmu:23871	ETS
K11648	SMARCB1,	mmu03250(	PF04855.17	GO:003506	nuclear	NP_003064	mmu:20587	
K10895	FANCI	mmu03460(	PF14678.12	GO:190187	cytoplasmic	NP_666058	mmu:208836	
K00490	CYP4F		PF00067.27	GO:000449	ER	EDL40279.1	mmu:170716	
K23220	LRIF1		PF15741.10	GO:000022	cytoplasmic	NP_001034	mmu:321000	
K13676	GXYLT	mmu00514(	PF01501.26	GO:000367	Golgi appa	NP_941014	mmu:232313	
K08764	SCP2, SCPX	mmu00120(	PF22691.2(T	GO:003444	peroxisoma	NP_035457	mmu:20280	
K09531	DNAJC11		PF11875.14	GO:006102	cytoplasmic	NP_766292	mmu:230935	
K24537	KAT14		PF00583.31	GO:003124	cytoplasmic	NP_852082	mmu:228714	
K06808	CDH13		PF00028.23	GO:000591	plasma mer	AAH21628.	mmu:12554	
26JJ(family with sequence similarity			PF21033.3(I	GO:009951	cytoplasmic	NP_001355	mmu:381110	
K15369	TRAK1		PF04849.18	GO:000810	cytoplasmic	EDL09161.1	mmu:67095	
K01784	galE, GALE	mmu00052(	PF16363.11	GO:001932	cytoplasmic	NP_001343	mmu:74246	
K16758	CCDC85B		PF10226.14	GO:004592	nuclear	NP_001387	mmu:240514	
K24816	MROH1, HEATR7A		PF23210.1(I	GO:000573	cytoplasmic	XP_011243	mmu:223658	
K10669	TRPT1, TPT1		PF01885.22	GO:009030	cytoplasmic	NP_705825	mmu:107328	
K13811	PAPSS	mmu00230(	PF01583.26	GO:000759	cytoplasmic	NP_001188	mmu:23972	
K21594	GUF1		PF00009.33	GO:000016	cytoplasmic	NP_766299	mmu:231279	
K15190	MEPCE, BCDIN3		PF06859.18	GO:008009	nuclear	NP_659162	mmu:231803	
QBG(GSK3B interacting protein)			PF05303.17	GO:003367	cytoplasmic	NP_848728	mmu:66787	
K01109	INPP4	mmu04070(	Phosphatid	GO:010601	cytoplasmic	NP_001284	mmu:234515	
K19408	SRPX2		PF13778.12	GO:000548	plasma mer	EDL20413.1	mmu:68792	
K16646	ANXA5		PF00191.25	GO:009008	cytoplasmic	NP_033803	mmu:11747	
Y21Q(SHC (Src homology 2 domain			PF00514.29	GO:002260	cytoplasmic	NP_001258	mmu:11877	
K23288	VPS50		PF10475.15	GO:000810	cytoplasmic	BAC98268.1	mmu:73288	
K12958	CAV2	mmu05020(	PF01146.22	GO:003288	cytoplasmic	NP_058596	mmu:12390	

K12821	PRPF40, PF	mmu03040(	PF01846.25	GO:002260	nuclear	NP_061255	mmu:56194	
K14396	PABPN1, P	mmu05164(	PF00452.24	GO:200123	cytoplasmic	XP_048300	mmu:54196	
ZF7J(CDC42 effector protein (Rho C			PF00786.33	GO:000548	cytoplasmic	NP_067429	mmu:58804	
K23297	C12ORF66		PF09404.15	GO:000810	cytoplasmic	NP_001346	mmu:270802	
K03681	RRP40, EX	mmu03018(	PF21262.3(I	GO:008009	nuclear	NP_079789	mmu:66362	
K23867	ECM1		PF05782.16	GO:008009	extracellulo	AAI45382.1	mmu:13601	
K23028	SPECC1		PF00307.36	GO:001602	nuclear	NP_001025	mmu:432572	
K06498	FCGR1A, C	mmu04666(	PF13895.12	GO:005123	plasma mer	NP_034316	mmu:14129	
K11578	ZW10, DSL1		PF20665.3(	GO:000810	cytoplasmic	NP_036169	mmu:26951	
K23357	SVBP		PF15674.10	GO:000810	mitochondr	NP_001034	mmu:69216	
K26237	TMEM9B		PF05434.16	GO:004858	ER	BAE27698.	mmu:56786	
Cerebellar degeneration-related prot			PF00096.32	GO:000548	nuclear	NP_001139	mmu:19552	zf-C2H2
ZN4R(Proline-rich coiled-coil 1)			PF01931.24	GO:000573	cytoplasmic	NP_082723	mmu:73137	
K24332	ATRN		PF24981.1(I	GO:004863	mitochondr	AAI41055.1	mmu:11990	
K08733	APPL1, DII	mmu05200(	PF16746.10	GO:003288	cytoplasmic	NP_660256	mmu:72993	
K01749	hemC, HMI	mmu01240(	PF01379.25	GO:003202	cytoplasmic	NP_038579	mmu:15288	
K11252	H2B	mmu05322(	PF00125.30	GO:000078	nuclear	BAC36440.	mmu:319188	
K24316	ARHGEF40		PF22697.2(	GO:000573	cytoplasmic	NP_937892	mmu:268739	
VX5(Transmembrane protein 160)				GO:003109	mitochondr	XP_031241	mmu:69094	
cytochrome b5 domain containing 2			PF03194.20	GO:002260	nuclear	XP_024428	mmu:67684	
K10733	GINS2, PSF2		PF25005.1(I	GO:007110	cytoplasmic	NP_849187	mmu:272551	
K06238	COL6A	mmu05165(	PF00092.34	GO:000931	extracellulo	NP_001229	mmu:12835	
K11206	NIT1, ybeM		PF00795.27	GO:001679	mitochondr	NP_036179	mmu:27045	
K14290	XPO1, CRM	mmu03008(	PF08767.17	GO:003315	cytoplasmic	XP_051020	mmu:103573	
K16506	FAT1_2_3		PF00028.23	GO:000716	plasma mer	XP_006509	mmu:14107	
K14716	SLC39A10,	mmu05010(	PF02535.28	GO:004858	extracellulo	NP_001343	mmu:227059	
K22641	TTYH		PF04906.18	GO:000522	plasma mer	NP_780483	mmu:78339	
K08731	BIRC5	mmu05161(	PF00653.27	GO:009008	cytoplasmic	NP_033819	mmu:11799	
K00940	ndk, NME	mmu01240(	PF00334.24	GO:008009	cytoplasmic	NP_001018	mmu:18103	
K09596	SPPL2A		PF04258.18	GO:000417	plasma mer	NP_075709	mmu:66552	
K23544	SERINC1		PF03348.21	GO:000665	plasma mer	NP_062734	mmu:56442	
K12815	DHX38, PR	mmu03040(	PF07717.22	GO:003563	nuclear	NP_848467	mmu:64340	
K10745	RNASEH2(	mmu03030(	PF08615.16	GO:000629	nuclear	NP_080892	mmu:68209	
K16914	RIOX1, NO66		PF21233.3(I	GO:005125	mitochondr	NP_076122	mmu:71952	
K24526	RBM12		PF00076.28	GO:000557	ER	AAI37621.1	mmu:77604	
K16185	RRAGA_B	mmu04140(	PF04670.18	GO:001989	cytoplasmic	NP_001030	mmu:68441	
K27392	FLNB	mmu04510(	PF00630.25	GO:003243	cytoplasmic	NP_001074	mmu:286940	
K12193	VPS24, CHI	mmu04217(	PF03357.26	GO:000699	cytoplasmic	NP_001348	mmu:66700	
K10590	TRIP12	mmu04120(	PF00632.30	GO:000020	cytoplasmic	NP_598736	mmu:14897	
K09605	RNPEPL1		PF01433.26	GO:000417	cytoplasmic	NP_852070	mmu:108657	
K05648	ABCA5	mmu02010(	PF12698.13	GO:190306	ER	BAE23713.	mmu:217265	
K04630	GNAI	mmu04730(	PF00503.26	GO:200123	cytoplasmic	AAB01732.	mmu:14677	
K23880	RETREG		PF24456.1(I	GO:004349	ER	NP_739561	mmu:227298	

K08011	NCF1, P47I	mmu05208(	PF00018.34	GO:00016	cytoplasmic	NP_001272	mmu:17969	
M9H(stress granule assembly)			PF04201.20	GO:00057	cytoplasmic	NP_001360	mmu:66314	
K11656	CHRAC1, C	mmu03082(	PF00808.29	GO:00347	nuclear	EDL29411.	mmu:93696	
K14554	UTP21, WD	mmu03008(	PF04192.17	GO:00486	nuclear	NP_001103	mmu:225348	
K16676	LPP		PF00412.28	GO:00324	cytoplasmic	NP_001139	mmu:210126	
K12613	DCP2	mmu03018(	PF05026.18	GO:00710	cytoplasmic	NP_081766	mmu:70640	
K20298	VPS52		PF20655.3(	GO:00486	cytoplasmic	AIC84002.1	mmu:224705	
K01242	CD38	mmu04970(	PF02267.23	GO:00900	plasma mer	NP_031672	mmu:12494	
K17614	DUSP3, VH	mmu04010(	PF00782.25	GO:00064	nuclear	XP_006534	mmu:72349	
K09512	DNAJB6		PF00226.36	GO:00081	nuclear	NP_001033	mmu:23950	
K11651	SMARCE1	mmu04714(	PF00505.25	GO:00350	cytoplasmic	NP_065643	mmu:57376	HMG
5KF(centrosomal protein 104kDa)			PF05049.19	GO:00200	cytoplasmic	NP_001028	mmu:240327	
K09667	OGT	mmu00514(	PF13844.12	GO:00329	cytoplasmic	NP_631883	mmu:108155	
K19944	TBC1D10		PF00566.25	GO:00058	cytoplasmic	NP_653105	mmu:68449	
K01444	AGA, aspG	mmu04142(	PF01112.23	GO:00003	lysosomal	NP_001005	mmu:11593	
K14777	DDX47, RRP3		PF00270.35	GO:19013	nuclear	NP_080636	mmu:67755	
K21991	UNC45		PF11701.14	GO:00064	cytoplasmic	Q8CGY6.1(	mmu:217012	
inc finger, CCHC domain containir			PF00125.30	GO:00007	nuclear	NP_001016	mmu:77605	
K13762	PDE5	mmu04022(	PF00233.25	GO:00421	cytoplasmic	NP_001396	mmu:242202	
K12741	HNRNPA1	mmu05014(	PF00076.28	GO:00903	nuclear	AAH62198.	mmu:229279	
K10898	FAAP24	mmu03460(	PF17949.7(I	GO:00061	nuclear	NP_848758	mmu:101831	
K00016	LDH, ldh	mmu05230(	PF00056.29	GO:00428	cytoplasmic	NP_001303	mmu:16832	
541(ig heavy chain V-III region VH26-like)				GO:00971	cytoplasmic	NP_001135	mmu:71684	
K13948	PTGR1, LT	mmu00590(	PF16884.10	GO:00066	cytoplasmic	NP_080244	mmu:67103	
K25166	EEF1AKNMT, METTI		PF13649.12	GO:00058	cytoplasmic	NP_659126	mmu:71449	
K03258	EIF4B	mmu04151(	PF00076.28	GO:00037	cytoplasmic	BAE41664.	mmu:75705	
K17562	PPP1R21, CCDC128, K		PF10212.14	GO:00319	nuclear	NP_082934	mmu:73825	
K08870	TWF		PF00241.25	GO:00995	cytoplasmic	NP_032997	mmu:19230	
K19196	BRMS1		PF08598.16	GO:00001	nuclear	NP_001032	mmu:52592	
K00280	LOXL2_3_4		PF01186.22	GO:00310	extracellula	NP_001157	mmu:67573	
K00111	glpA, glpD	mmu00564(	PF01266.30	GO:00054	mitochondr	NP_001139	mmu:14571	
K04467	IKBKA, IK	mmu05208(	PF18397.6(I	GO:00054	cytoplasmic	NP_001155	mmu:12675	
K06252	TN	mmu05165(	PF00041.27	GO:00192	plasma mer	NP_001356	mmu:21923	
ydrolase, family 31)			PF13465.12	GO:00800	cytoplasmic	NP_033590	mmu:22722	zf-C2H2
K09008	NDUFAF3	mmu04714(	PF04430.19	GO:00226	mitochondr	NP_075736	mmu:66706	
K08866	TTK, MPS1	mmu04110(	PF00069.31	GO:00081	cytoplasmic	P35761.1(R	mmu:22137	
K11832	USP1	mmu03460(	PF00443.35	GO:00058	cytoplasmic	NP_001288	mmu:230484	
K11580	SGOL1	mmu04114(	PF07558.16	GO:00198	nuclear	NP_082508	mmu:72415	
K06553	VPREB, CD179a		PF07686.23	GO:00023	cytoplasmic	P01646.1(R	mmu:22362	
K06553	VPREB, CD179a		PF07686.23	GO:00023	cytoplasmic	P01643.1(R	mmu:22364	
S8R(RNA binding motif protein 6)			PF07686.23	GO:00023	extracellula	P01642.1(RecName: Full=Ig kappa		
K19721	COL5AS	mmu04974(	PF01391.23	GO:00433	extracellula	NP_056549	mmu:12831	
ZEV(SLX4 interacting protein)			PF15744.10(UPF0492:U		nuclear	NP_001033	mmu:74243	

K06553	VPREB, CD179a		PF07686.23	GO:000237	cytoplasmic	P01648.1(R	mmu:22363
K00927	PGK, pgk	mmu01100(	PF00162.24	GO:004643	cytoplasmic	NP_032854	mmu:18655
K05849	SLC8A, NC	mmu04974(	PF03160.20	GO:005134	ER	NP_001273	mmu:20541
K01115	PLD1_2	mmu04724(	PF00614.28	GO:004643	cytoplasmic	NP_001355	mmu:18805
K11840	USP9_24		PF25010.1(	GO:004867	cytoplasmic	P70398.2(R	mmu:22284
K18180	COA7, SEL	mmu04714(	PF08238.18	GO:000557	cytoplasmic	NP_081526	mmu:69893
K07513	ACAA1	mmu00280(	PF00108.29	GO:003444	mitochondr	NP_001408	mmu:113868
K06633	PKMYT	mmu04914(	PF00069.31	GO:011003	cytoplasmic	NP_075545	mmu:268930
DIV(secretoglobin, family 1B, mem			PF01099.22	GO:000573	cytoplasmic	AAB97170.	mmu:11354
K19657	NPHP1		PF00018.34	GO:000592	cytoplasmic	ABM47411.	mmu:53885
K18051	PRKCH	mmu04750(	PF00069.31	GO:001060	cytoplasmic	EDL36503.	mmu:18755
K16854	EVI2B, CD361			GO:004885	plasma mer	NP_001070	mmu:101488212
K14708	SLC26A11		PF00916.26	GO:009859	ER	XP_006533	mmu:268512
K14457	MOGAT2,	mmu00561(	PF03982.18	GO:000665	Golgi appa	NP_803231	mmu:233549
K14363	TLT1			GO:003802	extracellulo	NP_001276	mmu:71326
K14321	NUPL2, NI	mmu03013(	PF18345.7(	GO:001593	nuclear	NP_694732	mmu:231042
K11495	CENPA		PF00125.30	GO:009868	cytoplasmic	EDL37288.	mmu:12615
K03231	EEF1A	mmu03013(	PF00009.33	GO:002262	cytoplasmic	BAE21802.	mmu:13627
K12494	PSD	mmu04144(	PF01369.25	GO:009859	cytoplasmic	XP_036009	mmu:234353
K26467	ALN	mmu04020(	PF17696.6(	GO:000557	Golgi appa	NP_077781	mmu:67704
K20174	OSBPL1_2, ORP1_2		PF01237.23	GO:001602	cytoplasmic	NP_997413	mmu:64291
K24737	WDR6		PF00400.38	GO:000989	cytoplasmic	BAE41106.	mmu:83669
K13186	RBM3		PF00076.28	GO:001593	cytoplasmic	NP_001159	mmu:19652
K08678	UXS1, uxs	mmu00520(	PF16363.11	GO:001932	mitochondr	NP_080706	mmu:67883
K25431	LRG1		PF13855.12	GO:000526	extracellulo	NP_084072	mmu:76905
K04366	RAF1	mmu05208(	PF00069.31	GO:200123	cytoplasmic	EDK99533.	mmu:110157
K14305	NUP43	mmu03013(	PF24796.1(	GO:001593	cytoplasmic	NP_663752	mmu:69912
245W(ubiquitin-associated (UBA) T			PF16158.11	GO:000989	cytoplasmic	NP_001069	mmu:224647
K06553	VPREB, CD179a		PF07686.23	GO:000237	cytoplasmic	AAA38822.	mmu:22364
K10357	MYO5	mmu04814(	PF00063.27	GO:005148	cytoplasmic	NP_034994	mmu:17918
K22402	GZF1		PF13465.12	GO:000097	nuclear	NP_001342	mmu:74533 ZBTB
K17686	copA, ctpA	mmu04978(	PF00403.32	GO:003202	cytoplasmic	NP_001103	mmu:11977
K10640	RNF25, AO7		PF05773.27	GO:004323	cytoplasmic	NP_067288	mmu:57751
XQDE(kiaa0528)			PF23025.1(	GO:009859	cytoplasmic	XP_017177	mmu:74741
K14692	SLC30A5_7, ZNT5_7, M		PF01545.26	GO:000578	cytoplasmic	EDL00783.	mmu:69048
K06944	DRG1, RBG1		PF16897.10	GO:003111	cytoplasmic	NP_001009	mmu:13494
K23761	S100A4		PF01023.25	GO:004205	cytoplasmic	NP_001397	mmu:20198
K14830	MAK11, PAK1IP1		PF24814.1(	GO:004858	nuclear	BAE22737.	mmu:68083
Replication protein A3, 14kDa)			PF00085.26	GO:000573	cytoplasmic	NP_001404	mmu:98258
K04414	MAP4K2, C	mmu04010(	PF00069.31	GO:004858	cytoplasmic	NP_033032	mmu:26412
K02373	FADD	mmu05022(	PF01335.27	GO:007066	cytoplasmic	NP_034305	mmu:14082
K26157	KIF19	mmu04814(	PF00225.29	GO:000367	nuclear	NP_001096	mmu:286942
K14288	XPOT	mmu03013(	PF19282.5(I	GO:001593	cytoplasmic	NP_001074	mmu:73192

K23218	ZMYND11, BS69		PF23461.1(Z	GO:200123	nuclear	NP_001334	mmu:66505	
K08527	RARA, NR	mmu05200(	PF00105.24	GO:000690	nuclear	NP_001170	mmu:19401	THR-like
K24099	LMO4		PF00412.28	GO:003514	cytoplasmic	KAF381966	mmu:16911	
K17658	TEFM		PF12836.13	GO:003277	mitochondr	NP_899098	mmu:68550	
K09855	RASSF7_8		PF21712.3(I	GO:000998	cytoplasmic	NP_082036	mmu:71323	
K20402	DEPTOR	mmu04140(	PF00610.27	GO:200123	cytoplasmic	NP_663445	mmu:97998	
.SBW(Transmembrane protein 242)			PF07096.16	GO:003361	peroxisoma	NP_081733	mmu:70544	
K15200	GTF3C2		PF00400.38	GO:003277	nuclear	NP_001020	mmu:71752	
K11545	NSL1, MIS14, DC8		PF08641.17	GO:190304	nuclear	NP_941056	mmu:381318	
K04739	PRKAR	mmu04910(	PF00027.35	GO:001922	cytoplasmic	NP_001240	mmu:19085	
K12837	U2AF2	mmu03040(	PF00076.28	GO:000024	nuclear	NP_001192	mmu:22185	
K10264	FBXW8	mmu04120(	PF24807.1(V	GO:003243	nuclear	BAC35527.1	mmu:231672	
K20689	ARHGEF17		PF19056.6(V	GO:000699	cytoplasmic	NP_001074	mmu:207212	
K10484	BTBD12, SI	mmu03460(	PF09494.16	GO:008009	cytoplasmic	NP_803423	mmu:52864	ZBTB
K09593	GAB1	mmu04012(	PF00169.35	GO:001922	cytoplasmic	NP_001288	mmu:14388	
K20346	TMED4_9_11		PF01105.29	GO:003802	ER	NP_598781	mmu:103694	
K07210	IKBKG, IK	mmu05208(	PF11577.13	GO:200123	cytoplasmic	NP_001154	mmu:16151	
K15215	TAF1D		PF15333.11	GO:008009	nuclear	AAH27813.	mmu:75316	
Hydrolase)			PF00702.32	GO:001678	cytoplasmic	NP_077219	mmu:72748	
K20305	TRAPPC8, TRS85		PF12739.12	GO:004820	cytoplasmic	NP_796012	mmu:75964	
K06256	CD44	mmu04512(	PF00193.23	GO:200123	plasma mer	NP_001171	mmu:12505	
K05643	ABCA3	mmu02010(	PF00005.33	GO:001922	cytoplasmic	AAH42663.	mmu:27410	
K06553	VPREB, CD179a		PF07686.23	GO:000548	cytoplasmic	AAX56292.	mmu:22362	
K17541	SCYL2		PF00069.31	GO:004858	cytoplasmic	NP_001297	mmu:213326	
K24260	WDR11		PF23752.1(I	GO:000858	cytoplasmic	NP_758459	mmu:207425	
K10849	ERCC1	mmu03420(	PF03834.20	GO:000962	cytoplasmic	NP_031974	mmu:13870	
K00799	GST, gst	mmu00980(	PF00043.30	GO:000578	cytoplasmic	NP_032209	mmu:14863	
K04079	HSP90A, ht	mmu04915(	PF00183.23	GO:003315	cytoplasmic	BAE27553.1	mmu:15519	
phytoene)			PF01593.30	GO:000699	mitochondr	NP_083287	mmu:74580	
K18025	PTPN14_21		PF00102.32	GO:001679	cytoplasmic	NP_001139	mmu:24000	
.X3X(CDC42 effector protein (Rho			PF14957.11	GO:000806	cytoplasmic	NP_081048	mmu:104252	
K15199	GTF3C1		PF24101.1(e	GO:003277	nuclear	NP_997122	mmu:233863	
K12840	RBM17, SPI	mmu03040(	PF01585.28	GO:009030	nuclear	NP_690037	mmu:76938	
K26390	TNFAIP8		PF05527.16	GO:004858	cytoplasmic	NP_079842	mmu:66443	
K16578	CLASP1_2		PF12348.14	GO:003111	cytoplasmic	XP_011246	mmu:76707	
K01899	LSC1	mmu00020(	PF02629.25	GO:004423	mitochondr	NP_063932	mmu:56451	
K10463	KLHL26		PF01344.31(Kelch_1:Ke		cytoplasmic	NP_001116	mmu:23437	ZBTB
K08825	DYRK1		PF00069.31	GO:003111	nuclear	NP_001334	mmu:13548	
K03164	TOP2	mmu01524(	PF00521.26	GO:008009	nuclear	BAC28232.1	mmu:21973	
K10750	CHAF1A		PF15539.11	GO:003472	nuclear	NP_038761	mmu:27221	
K08601	CYLD, USI	mmu04380(	PF16607.10	GO:200123	cytoplasmic	BAE21677.1	mmu:74256	
.EA0(FHA domaincontaining prote			PF00337.29	GO:000367	cytoplasmic	NP_001128	mmu:216551	
K11431	SETD7	mmu04068(	PF22648.2(S	GO:000647	nuclear	XP_021014	mmu:73251	

K08716	SLC14A		PF03253.19	GO:009859	plasma mer	NP_001164	mmu:108052	
K08366	BLOC1S4, CNO			GO:009951	cytoplasmic	NP_598485	mmu:117197	
K13720	NAAA		PF15508.11	GO:004648	lysosomal	BAE21682.	mmu:67111	
K17339	ATL		PF02263.25	GO:002260	cytoplasmic	NP_848743	mmu:73991	
K20124	PSTPIP2		PF00611.28	GO:002260	cytoplasmic	BAE32488.	mmu:19201	
K07605	KRT2		PF00038.26	GO:009951	mitochondr	NP_666175	mmu:223917	
K24247	MMRN		PF00386.27	GO:009863	ER	NP_694767	mmu:105450	
K07415	CYP2E1	mmu00980(	PF00067.27	GO:000449	ER	NP_067257	mmu:13106	
K12404	GGA	mmu04142(	PF00790.25	GO:000810	cytoplasmic	NP_666041	mmu:106039	
erine threonine-protein kinase)			PF01557.24	GO:001678	mitochondr	NP_083905	mmu:68126	
K17551	PPP1R9		PF17817.7(I	GO:000810	cytoplasmic	NP_758465	mmu:217124	
K11456	PHC1, EDF	mmu03083(	PF16616.10	GO:004239	nuclear	NP_001258	mmu:13619	
K10626	UBR2		PF18995.5(I	GO:001922	cytoplasmic	NP_001170	mmu:224826	
K03033	PSMD3, RF	mmu05020(	PF08375.16	GO:000989	cytoplasmic	NP_033465	mmu:22123	
K06170	PSENEN, I	mmu04330(	PF10251.14	GO:000548	ER	NP_001350	mmu:66340	
K06269	PPP1C	mmu04750(	PF00149.33	GO:003292	cytoplasmic	NP_001038	mmu:19047	
K13152	ZMAT5		PF00642.30	GO:000557	cytoplasmic	NP_001343	mmu:67178	
25KF(Divergent AAA domain)			PF05049.19	GO:001602	cytoplasmic	NP_001138	mmu:21822	
K25824	ABHD16A	mmu00561(	PF22990.1(	GO:000665	Golgi appa	NP_848707	mmu:193742	
K00940	ndk, NME	mmu01240(	PF00334.24	GO:003111	mitochondr	NP_001280	mmu:171567	
K09680	PANK1_2_	mmu01240(	PF03630.20(Fumble:Fu		mitochondr	NP_705721	mmu:74450	
K16186	RRAGC_D	mmu04140(	PF04670.18	GO:000810	cytoplasmic	NP_059503	mmu:54170	
K23708	SORBS2		PF00018.34	GO:009859	cytoplasmic	BAC65651.	mmu:234214	
K03259	EIF4E	mmu04910(	PF01652.23	GO:003577	cytoplasmic	NP_001034	mmu:26987	
K13515	LPCAT3, M	mmu00564(	PF03062.24	GO:008009	Golgi appa	NP_660112	mmu:14792	
K07902	RAB8B	mmu04530(	PF00071.28	GO:009008	cytoplasmic	NP_695229	mmu:235442	
K18619	CORO7		PF00400.38	GO:001602	cytoplasmic	NP_084481	mmu:78885	
K02219	CKS1	mmu05200(	PF01111.24	GO:006157	cytoplasmic	NP_001106	mmu:54124	
K07903	RAB10	mmu04152(	PF00071.28	GO:000810	cytoplasmic	NP_001003	mmu:19325	
K04289	LPAR1, ED	mmu04540(	PF00001.26	GO:003572	cytoplasmic	NP_001407	mmu:14745	
K17286	STOM		PF01145.30	GO:000573	cytoplasmic	NP_038543	mmu:13830	
I3F(p53 and DNA-damage regulat			PF00857.26	GO:000573	cytoplasmic	NP_001095	mmu:664994	
K16726	ANKRD17, MASK		PF12796.13	GO:000367	cytoplasmic	NP_780584	mmu:108857	
K24962	CCDC47		PF07946.19	GO:000810	ER	NP_080285	mmu:67163	
K02955	RP-S14e, RI	mmu05171(	PF00411.24	GO:002260	cytoplasmic	CAA69615.	mmu:20044	
K04386	IL1R1, CD1	mmu04750(	PF01582.25	GO:001922	plasma mer	NP_032388	mmu:16177	
K10732	GINS1, PSF1		PF24997.1(I	GO:007110	cytoplasmic	NP_081290	mmu:69270	
ZNRC(nuclear factor of activated T			PF22628.2(	GO:000989	nuclear	EHH20848.	mmu:103284	
			PF02093.21	GO:004671	nuclear	AID54954.1	mmu:118567641	
K23588	MLLT6_10, AF17_10		PF13832.12	GO:004239	nuclear	NP_001239	mmu:17354	
K04382	PPP2C	mmu05165(	PF00149.33	GO:003315	cytoplasmic	NP_058735	mmu:19052	
K00102	LDHD, dld	mmu00620(	PF02913.24	GO:000925	mitochondr	NP_081846	mmu:52815	
K09113	MLX	mmu04931(	PF00010.31	GO:005116	nuclear	BAC65673.	mmu:20810	bHLH

K00787	FDPS	mmu00900(	PF00348.22	GO:008009	cytoplasmic	BAE38529.1	mmu:110196	
K09510	DNAJB4		PF01556.23	GO:000582	cytoplasmic	NP_001343	mmu:67035	
K04411	STK4, MST	mmu05200(	PF00069.31	GO:200123	cytoplasmic	NP_067395	mmu:58231	
K18595	EML1_2		PF23409.1(I	GO:003111	cytoplasmic	NP_001156	mmu:72205	
K14546	UTP5, WD	mmu03008(	PF04003.17	GO:003204	cytoplasmic	EDL38392.	mmu:72515	
Ras and Rab interactor)			PF16921.10	GO:000548	cytoplasmic	NP_084409	mmu:78586	
K06554	IGLL1, IGI	mmu05340(	PF07654.21	GO:000548	plasma mer	BAQ25543.	mmu:16136	
K20652	SH3BP1, ARHGAP43		PF00620.32	GO:000189	cytoplasmic	NP_001303	mmu:20401	
K14861	URB1		PF11707.13	GO:000815	cytoplasmic	NP_083773	mmu:207932	
K16860	PLD3_4	mmu00564(	PF13918.12	GO:000695	ER	NP_001304	mmu:18807	
K14964	ASH2	mmu04934(	PF21198.3(	GO:008009	cytoplasmic	BAE27293.1	mmu:23808	
K25079	RBM42		PF00076.28	GO:009715	extracellulo	XP_021049	mmu:68035	
K01189	GLA	mmu00052(	PF16499.11	GO:001613	lysosomal	P51569.1(R	mmu:11605	
K03593	mrp, NUBPL		PF10609.15	GO:001707	mitochondr	NP_084036	mmu:76826	
K15178	RTF1		PF03126.23	GO:008009	mitochondr	NP_084388	mmu:76246	
K02942	RP-LP1, RF	mmu05171(	PF00428.24	GO:001593	cytoplasmic	NP_061341	mmu:56040	
K08576	CAPN7		PF00648.27	GO:000417	cytoplasmic	NP_033926	mmu:12339	
K03949	NDUFA5	mmu05208(	PF04716.19	GO:002260	cytoplasmic	NP_080890	mmu:68202	
K17444	HOXC10		PF00046.35	GO:003250	nuclear	NP_034592	mmu:20944	Homeobox
K13907	CSTA_B		PF00031.27	GO:000582	cytoplasmic	NP_001371	mmu:76770	
K06999	K06999		PF02230.22	GO:190547	mitochondr	NP_666218	mmu:226791	
K08864	TLK		PF00069.31	GO:009951	cytoplasmic	NP_001106	mmu:24086	
K17634	RASAL3	mmu04014(	PF00616.24	GO:000548	cytoplasmic	NP_848900	mmu:320484	
K08869	ADCK, ABC1		PF03109.22	GO:000548	mitochondr	NP_598531	mmu:76889	
K7G(plasminogen receptor, C-term			PF10166.14	GO:008009	cytoplasmic	NP_080638	mmu:67759	
K12197	CHMP1, VI	mmu03272(	PF03357.26	GO:000810	cytoplasmic	BAE39667.1	mmu:67064	
K16339	THEM4, C	mmu04151(	PF03061.28	GO:009859	mitochondr	NP_083707	mmu:75778	
XR6C(Solute carrier family 4 (Anion			PF00498.31	GO:001920	nuclear	BAE23643.1	mmu:20534	
K20125	FCHSD		PF00018.34	GO:000810	cytoplasmic	NP_001139	mmu:207278	
K12558	MYBPC2	mmu04820(	PF07679.22	GO:000520	cytoplasmic	NP_666301	mmu:233199	
K05472	TNFSF9, C	mmu04060(	PF00229.24	GO:001922	cytoplasmic	NP_033430	mmu:21950	
K08762	DBI, ACBP	mmu03320(	PF00887.24	GO:190306	cytoplasmic	NP_031856	mmu:13167	
K24675	ZMYM2_3_4		PF06467.20(zf-FCS:MY		cytoplasmic	NP_001347	mmu:76007	
K03903	FGA	mmu04610(	PF00147.23	GO:200123	ER	NP_001104	mmu:14161	
K23325	TBL2		PF00400.38	GO:000548	ER	NP_038791	mmu:27368	
PSZ(CDKN2A interacting protein)			PF11952.13	GO:004592	nuclear	NP_765995	mmu:70925	
K26797	GORASP, (	mmu04140(	PF04495.20	GO:000810	cytoplasmic	EDL27068.	mmu:70231	
K04675	ACVR1, AI	mmu05418(	PF07714.23	GO:200123	plasma mer	NP_001103	mmu:11477	
K11219	TYK2	mmu05165(	PF07714.23	GO:001922	cytoplasmic	NP_001192	mmu:54721	
K11974	RNF31, HC	mmu04621(	PF16678.12	GO:001922	cytoplasmic	AAQ55547.	mmu:268749	
K00871	PHKG	mmu04922(	PF00069.31	GO:000815	cytoplasmic	NP_035209	mmu:18682	
K08107	GPC1	mmu05418(	PF01153.25	GO:005084	extracellulo	NP_057905	mmu:14733	
K12047	MGAM	mmu00052(	PF01055.32(Glyco_hydi		lysosomal	NP_001355	mmu:232714	

K04439	ARRB	mmu04010(	PF00339.34	GO:003577	cytoplasmic	NP_796205	mmu:109689
K25384	LEG1		PF05612.17	GO:000557	lysosomal	EDL04827.	mmu:67719
K16455	CEP41, TSGA14		PF00581.25	GO:002260	cytoplasmic	BAE36024.1	mmu:83922
K27310	TMEM41			GO:001712	cytoplasmic	EDL20905.	mmu:233724
K26171	TMEM97, S	mmu04080(	PF05241.17	GO:000032	ER	NP_598467	mmu:69071
K07605	KRT2		PF00038.26	GO:009951	mitochondr	EDL04037.	mmu:239673
K02132	ATPeF1A, /	mmu05208(	PF00006.31	GO:000526	mitochondr	NP_031531	mmu:11946
K06758	CHL1		PF13927.12	GO:001989	extracellula	EDK99389.	mmu:12661
K04433	MAP2K6, M	mmu04750(	PF00069.31	GO:001922	cytoplasmic	NP_036073	mmu:26399
K15730	PTGES3	mmu00590(	PF04969.22	GO:001922	cytoplasmic	BAE39529.1	mmu:56351
K05853	ATP2A	mmu04972(	PF00122.25	GO:003238	cytoplasmic	EDL17360.	mmu:11937
K06091	MPP5, PAL	mmu05165(	PF00625.27	GO:000810	cytoplasmic	NP_062525	mmu:56217
K17822	DCUN1D1_2		PF03556.21	GO:190187	cytoplasmic	EDL22132.	mmu:102323
K26162	MADD		PF23629.1(	Death_MAI	cytoplasmic	XP_030106	mmu:228355
K05674	ABCC10	mmu02010(	PF00664.28	GO:009859	extracellula	AAI50819.1	mmu:224814
K04525	SERPINA		PF00079.25	GO:000486	extracellula	P07759.2(R	mmu:20714
K08495	GOSR1, G(	mmu04130(	PF12352.13	GO:000810	cytoplasmic	NP_058090	mmu:53334
K23826	TIFA		PF00498.31	GO:001604	cytoplasmic	NP_001343	mmu:211550
K27835	GATD3, ES1			GO:004423	mitochondr	NP_613067	mmu:28295
K03250	EIF3E, INT	mmu05160(	PF09440.15	GO:003315	cytoplasmic	XP_017384	mmu:16341
XP96(ubiquitin thiolesterase activity			PF00240.29	GO:000551	mitochondr	NP_613055	mmu:28018
K20067	STON		PF00928.26	GO:000802	cytoplasmic	BAE23047.1	mmu:77057
AEB(chromosome 11 open reading			PF15485.11(	DUF4643:I	cytoplasmic	NP_001382	mmu:677289
K22068	ISCU		PF01592.21	GO:001922	mitochondr	NP_079802	mmu:66383
K00030	IDH3	mmu01100(	PF00180.25	GO:003563	cytoplasmic	NP_766489	mmu:243996
K16143	CRP		PF00354.24	GO:001003	extracellula	AAH11124.	mmu:12944
K21915	KCTD3		PF02214.28	GO:005125	cytoplasmic	EDL13032.	mmu:226823
K08892	FRK, PTK5		PF07714.23	GO:008009	cytoplasmic	EDL04894.	mmu:14302
K20393	ARL6IP5, PRAF3		PF03208.24	GO:000810	Golgi appa	NP_075368	mmu:65106
K17264	ERP44, TXNDC4		PF13848.12	GO:000645	ER	NP_083848	mmu:76299
K12602	WDR61, RI	mmu03018(	PF24814.1(	GO:000018	cytoplasmic	BAC33795.2	mmu:66317
K03979	obgE, cgtA, MTG2		PF01018.28	GO:001602	mitochondr	NP_001076	mmu:52856
K09543	HSPB2	mmu05205(	PF00011.27	GO:000645	cytoplasmic	AAH64051.	mmu:69253
K10691	UBR4, ZUE	mmu05165(	PF19423.4(	GO:004592	nuclear	NP_001153	mmu:69116
K20217	UBE2E	mmu04120(	PF00179.31	GO:003202	cytoplasmic	XP_005008	mmu:22194
K20176	SGSM3, MAP		PF00566.25	GO:000989	cytoplasmic	XP_006520	mmu:105835
K23048	UNK		PF23261.1(	GO:000989	cytoplasmic	NP_001272	mmu:217331
K23858	PLEKHF		PF01363.27	GO:000810	cytoplasmic	NP_780384	mmu:71801
K24348	UBXN1_4	mmu04141(	PF00789.26	GO:190187	cytoplasmic	NP_001348	mmu:225896
K24610	LRIG1_3		PF13855.12	GO:004859	plasma mer	AAI45624.1	mmu:16206
K07763	MMP14	mmu04928(	PF00413.30	GO:008009	ER	NP_032634	mmu:17387
K14462	APOB	mmu04979(	PF01347.28	GO:000581	ER	NP_033823	mmu:238055
K10798	PARP3_4	mmu04210(	PF00644.27	GO:000810	cytoplasmic	NP_001298	mmu:235587

K20473	NBAS		PF15492.12	GO:000989	cytoplasmic	NP_081982	mmu:71169	
K04674	TGFBR1, A	mmu05200(	PF00069.31	GO:003292	cytoplasmic	NP_001299	mmu:21812	
K08056	PDIA3, GR	mmu05170(	PF00085.26	GO:200123	ER	NP_031978	mmu:14827	
K04497	GRO, TLE	mmu04310(	PF03920.20	GO:003250	nuclear	XP_006511	mmu:21887	
K05678	ABCD4, PX	mmu02010(	PF06472.20	GO:003444	plasma mer	EDL02814.	mmu:19300	
K01265	map		PF00557.29	GO:000417	cytoplasmic	NP_780433	mmu:75624	
K09438	SPI1	mmu05200(	PF00178.28	GO:003288	nuclear	NP_001365	mmu:20375	ETS
K17618	UBLCP1		PF03031.24	GO:001679	cytoplasmic	NP_077795	mmu:79560	
K17201	ARL15		PF00025.27	GO:003563	cytoplasmic	NP_766183	mmu:218639	
K17923	SNX9_18_3	mmu05132(	PF10456.14	GO:003608	cytoplasmic	NP_780692	mmu:235406	
K22038	LRRC8		PF12534.13	GO:007147	ER	NP_001116	mmu:231549	
K17613	CABIN1		PF09047.15	GO:003472	cytoplasmic	NP_766137	mmu:104248	
K04684	SP1	mmu05231(	PF00096.32	GO:008009	cytoplasmic	NP_038700	mmu:20683	zf-C2H2
K12188	SNF8, EAP	mmu04144(	PF04157.22	GO:000810	cytoplasmic	NP_291046	mmu:27681	
K01251	AHCY, ahc	mmu00270(	PF05221.23	GO:000548	cytoplasmic	NP_001094	mmu:229709	
K24095	NISCH		PF00787.29	GO:001587	cytoplasmic	NP_073147	mmu:64652	
K12380	RASA3	mmu04014(	PF00168.36	GO:000548	cytoplasmic	NP_033051	mmu:19414	
K16498	PCDHD1		PF00028.23	GO:004316	plasma mer	XP_017173	mmu:75599	
.9S4(immunoglobulin kappa variab			PF07686.23(V-set:Immu		plasma mer	CAB46169.1(immunoglobulin light		
K02604	ORC2	mmu04110(	PF04084.20	GO:009030	nuclear	NP_001258	mmu:18393	
K03368	ST3GAL2	mmu00604(	PF00777.23	GO:000551	Golgi appa	EDL11477.	mmu:20444	
K12791	ARHGEF2,	mmu04530(	PF00621.25	GO:003111	cytoplasmic	XP_006501	mmu:16800	
K10662	MARCH7, AXOT		PF12906.13	GO:004213	nuclear	NP_001405	mmu:57438	
K10865	MRE11	mmu03440(	PF04152.19	GO:001922	nuclear	NP_001398	mmu:17535	
K16198	YWHAG_I	mmu04114(	PF00244.26	GO:000449	cytoplasmic	NP_035868	mmu:22629	
K19573	ATAT1, MEC17		PF05301.17	GO:009951	cytoplasmic	NP_001136	mmu:73242	
K04552	UBE2L3, U	mmu04120(	PF00179.31	GO:008009	cytoplasmic	NP_001406	mmu:22195	
K12841	CHERP	mmu03040(	PF25127.1(I	GO:001972	cytoplasmic	NP_613051	mmu:27967	
K06068	PRKCD	mmu05208(	PF00069.31	GO:003315	cytoplasmic	NP_001411	mmu:18753	
K01394	MMP3	mmu05171(	PF00413.30	GO:001922	ER	AAH06725.	mmu:17392	
K01021	TPST		PF13469.12	GO:000647	ER	NP_001313	mmu:22022	
K20162	DENND3		PF02141.26	GO:000833	plasma mer	NP_001074	mmu:105841	
.D7E(Chromosome 14 open reading			PF15387.11	GO:199023	nuclear	NP_001136	mmu:100859931	
K01611	speD, AMD	mmu00270(	PF01536.21	GO:000659	cytoplasmic	NP_033795	mmu:11702	
K21066	ARHGEF18	mmu04530(	PF17838.7(I	GO:002260	cytoplasmic	XP_034345	mmu:102098	
K10392	KIF1	mmu04814(	PF00225.29(Kinesin:Ki		nuclear	XP_034359	mmu:16561	
K27244	TMEM260		PF11028.14	GO:004423	ER	NP_766188	mmu:218989	
K19947	MICAL		PF12130.13	GO:000449	nuclear	NP_001257	mmu:194401	
K20163	DENND4		PF02141.26	GO:000579	cytoplasmic	NP_958809	mmu:229541	
K11647	SMARCA2_	mmu04714(	PF00176.29	GO:008009	cytoplasmic	NP_001167	mmu:20586	
K20632	DLC		PF01852.25	GO:003250	nuclear	NP_001181	mmu:50768	
K27813	CAVIN2, SDPR		PF15237.11	GO:009859	cytoplasmic	NP_620080	mmu:20324	
K21626	CCNDBP1, DIP1, GCI		PF13324.12	GO:000573	cytoplasmic	BAE26115.	mmu:17151	

K04405	ECSIT	mmu04010(	PF06239.17	GO:008009	mitochondr	NP_001240	mmu:26940
K22184	BRD9	mmu03082(	PF12024.13	GO:004212	nuclear	NP_001019	mmu:105246
K20526	TAGLN		PF00307.36	GO:001562	cytoplasmic	NP_848713	mmu:21346
K11843	USP14, UBP6		PF00443.35	GO:008009	cytoplasmic	NP_067497	mmu:59025
K00777	QTRT1		PF01702.25	GO:000815	mitochondr	NP_068688	mmu:60507
K12967	IL33	mmu05164(	PF15095.12	GO:005116	cytoplasmic	ADW82003	mmu:77125
K17780	TIM8		PF02953.20	GO:000548	cytoplasmic	NP_038926	mmu:30058
K04424	ZAK, MLT	mmu04010(	PF07714.23	GO:004858	cytoplasmic	NP_075544	mmu:65964
K15179	WHSC2, NI	mmu03250(	PF23553.1(	GO:000582	nuclear	BAE26961.	mmu:24116
K10696	BRE1		PF00097.31	GO:008009	nuclear	EDL02308.	mmu:109331
K17800	LETM1, MDM38		PF07766.19	GO:003000	cytoplasmic	NP_062668	mmu:56384
K13136	GEMIN8		PF15348.11	GO:002260	mitochondr	NP_001297	mmu:237221
K14836	NOP8, NOL8, NOP132		PF00076.28	GO:003450	nuclear	NP_001258	mmu:70930
K17704	APBB1IP, F	mmu04611(	PF21989.2(	GO:001602	cytoplasmic	NP_062329	mmu:54519
K13280	SEC11, sipV	mmu03060(	PF00717.28	GO:000425	cytoplasmic	NP_079744	mmu:66286
K17426	MRPL45		PF04280.21	GO:001593	mitochondr	NP_080203	mmu:67036
K08270	DDIT4, RE	mmu04140(	PF07809.16	GO:001922	mitochondr	NP_083359	mmu:74747
K22562	COMMD6		PF07258.19	GO:000573	cytoplasmic	NP_001028	mmu:66200
K02183	CALM	mmu04750(	PF00036.39	GO:009951	cytoplasmic	ACH46215.	mmu:12313
K16944	SEPT7, CDC3		PF00735.24	GO:003243	cytoplasmic	NP_033989	mmu:235072
K25831	LUZP1			GO:004859	cytoplasmic	NP_077772	mmu:269593
K19525	VPS13A_C		PF12624.14	GO:000810	cytoplasmic	NP_766616	mmu:271564
K01165	RNASEL	mmu04621(	PF12796.13	GO:008009	cytoplasmic	NP_036012	mmu:24014
K02872	RP-L13Ae,	mmu05171(	PF00572.23	GO:004423	nuclear	NP_033464	mmu:22121
HBB(TM2 domain)			PF05154.21	GO:000557	plasma mer	NP_081470	mmu:69742
K02951	RP-S12e, RI	mmu05171(	PF01248.31	GO:003011	cytoplasmic	KAF611591	mmu:20042
K01136	IDS	mmu04142(	PF00884.28	GO:003020	lysosomal	NP_034628	mmu:15931
K02884	RP-L19, MI	mmu03010(	PF01245.26	GO:001593	mitochondr	NP_080766	mmu:56284
K12591	RRP6, EXC	mmu03018(	PF01612.26	GO:007104	nuclear	NP_057908	mmu:50912
K10057	CLEC4A, CD367		PF00059.27	GO:000989	cytoplasmic	NP_955015	mmu:269799
K11657	RSF1	mmu03082(	PF15612.11	GO:008009	nuclear	NP_001074	mmu:233532
K23211	THAP11	mmu04980(	PF05485.17	GO:000582	nuclear	NP_067488	mmu:59016 THAP
K18993	UBASH3, STS		PF00300.27	GO:003411	cytoplasmic	NP_789830	mmu:72828
K05615	SLC1A4, SATT		PF00375.23	GO:009951	cytoplasmic	NP_061349	mmu:55963
K00049	GRHPR	mmu00630(	PF02826.25	GO:007040	mitochondr	NP_525028	mmu:76238
K24945	ZC3H7			GO:001922	cytoplasmic	NP_666043	mmu:106205
K12865	PQBP1, NF	mmu03040(	PF00397.32	GO:003577	nuclear	NP_001239	mmu:54633
K12310	CTBS		PF00704.34	GO:000032	lysosomal	NP_001280	mmu:74245
)SN1, MIND kinetochore complex			PF22725.1(	GO:011016	mitochondr	NP_001355	mmu:70575
K02677	PRKCA	mmu04750(	PF00069.31	GO:000578	cytoplasmic	NP_035231	mmu:18750
K10740	RPA3	mmu03440(	PF08661.17	GO:000629	cytoplasmic	NP_080908	mmu:68240
K12642	PKP2	mmu05412(	PF00514.29	GO:000810	cytoplasmic	NP_080439	mmu:67451
K03946	NDUFA2	mmu05208(	PF05047.22	GO:000916	peroxisoma	NP_035015	mmu:17991

K12471	EPN	mmu04144(	PF01417.26	GO:000548	cytoplasmic	NP_001239	mmu:13855		
K15439	EEF1E1, AIMP3		PF21972.1(	GO:200123	cytoplasmic	NP_079656	mmu:66143		
K10600	UBOX5, U	mmu04120(	PF19318.4(	GO:004438	nuclear	AAY21061.	mmu:140629		
K17334	NFATC4, N	mmu05167(	PF16179.11	GO:200123	cytoplasmic	NP_076188	mmu:73181	RHD	
K12647	IFIH1, MD	mmu05164(	PF16739.11	GO:001922	cytoplasmic	NP_082111	mmu:71586		
KSJZ(Parkinson disease 7 domain containing 1)				GO:001975	mitochondr	NP_742114	mmu:213350		
K03956	NDUFA11	mmu05208(	PF02466.24	GO:002260	cytoplasmic	NP_081520	mmu:69875		
K22582	PREPL		PF00326.27	GO:005080	cytoplasmic	BAC34440.	mmu:213760		
K01230	MAN1A_C	mmu04141(	PF01532.26	GO:000578	Golgi appa	NP_997120	mmu:230815		
K01011	TST, MPST	mmu04122(	PF00581.25	GO:000652	mitochondr	NP_001155	mmu:246221		
ZD4(cysteine-rich, DPF motif dom			PF10170.14(C6_DPF:C		cytoplasmic	NP_001158	mmu:72355		
K03860	PIGQ, GPI	mmu00563(	PF05024.20	GO:000578	ER	NP_001277	mmu:14755		
ZPJ1(-)				GO:000875	cytoplasmic	NP_082590	mmu:72658		
K06462	ITGAX, CI	mmu04610(	PF21520.3(	GO:003411	ER	BAE41792.	mmu:16411		
KUBF(kiaa0513)					cytoplasmic	NP_001157	mmu:234797		
K20395	CD300B_D_F		PF15330.11	GO:001922	plasma mer	NP_001162	mmu:246746		
K05699	ACTN1_4	mmu04530(	PF00435.27	GO:003243	cytoplasmic	XP_006540	mmu:60595		
K08844	LRRK2	mmu05022(	PF23744.1(	GO:190335	cytoplasmic	NP_080006	mmu:66725		
K10341	WSB1		PF00400.38	GO:002260	cytoplasmic	NP_062627	mmu:78889		
K22744	SIVA1	mmu04115(	PF05458.17	GO:000512	cytoplasmic	NP_038957	mmu:30954		
K25392	CCDC88A		PF19047.5(HOOK_N:F		cytoplasmic	NP_001350	mmu:108686		
K00876	udk, UCK	mmu00983(	PF14681.12	GO:000921	cytoplasmic	NP_081041	mmu:68556		
s involved in NO detoxification in c			PF13836.11	GO:008009	nuclear	AAI17874.1	mmu:64453		
K00710	GALNT	mmu00514(	PF00535.33	GO:003024	ER	NP_653332	mmu:108150		
K18763	LARP4		PF05383.23	GO:002260	cytoplasmic	NP_001271	mmu:207214		
K04469	NFKB2	mmu04010(	PF00554.27	GO:008009	nuclear	NP_001170	mmu:18034	RHD	
K09542	CRYAB	mmu04141(	PF00525.23	GO:003243	cytoplasmic	NP_001276	mmu:12955		
K06273	ZYX	mmu04510(	PF00412.28	GO:003243	cytoplasmic	BAE41886.	mmu:22793		
K03265	ETF1, ERF	mmu03015(	PF03464.20	GO:000018	cytoplasmic	BAE41644.	mmu:225363		
K01125	NAGPA	mmu04142(	PF09992.14	GO:000810	plasma mer	BAE41643.	mmu:27426		
984(Alpha-1-B glycoprotein)			PF13895.12	GO:000557	extracellula	NP_001074	mmu:117586		
K00948	PRPS, prsA	mmu00030(	PF13793.12	GO:004353	cytoplasmic	NP_001032	mmu:328099		
K01885	EARS, gltX	mmu01240(	PF00749.27	GO:003563	mitochondr	NP_080416	mmu:67417		
K04538	GNB4	mmu04724(	PF00400.38	GO:003015	cytoplasmic	NP_001334	mmu:14696		
K24265	PANK4		PF03630.20	GO:001679	cytoplasmic	NP_001292	mmu:269614		
K12897	TRA2	mmu04936(	PF00076.28	GO:000989	nuclear	NP_001334	mmu:101214		
K24374	ZNF644		PF23015.2(zf-WIZ:Zinc		nuclear	NP_001350	mmu:52397	zf-C2H2	
K26116	MTBP	mmu04110(	PF14919.11	GO:190187	cytoplasmic	NP_598853	mmu:105837		
K11393	METTL16, METT10D		PF05971.18	GO:008009	cytoplasmic	EDL12777.	mmu:67493		
K19347	SUN1_2	mmu04820(	PF07738.19	GO:009951	cytoplasmic	NP_001192	mmu:223697		
K04644	CLTA, LCA	mmu04721(	PF01086.22	GO:000810	cytoplasmic	NP_058040	mmu:12757		
K10767	ALKBH5		PF13532.12	GO:005116	cytoplasmic	NP_766531	mmu:268420		
K12838	PUF60	mmu03040(	PF00076.28	GO:002260	cytoplasmic	NP_001386	mmu:67959		

K06514	THY1, CD9	mmu04670(	PF00047.31	GO:005134	ER	NP_033408	mmu:21838	
K07878	RAB2B		PF00071.28	GO:000548	cytoplasmic	AAH61513.	mmu:76338	
K13159	HNRNPL		PF13893.12	GO:000548	nuclear	NP_659051	mmu:72692	
K00461	ALOX5	mmu04664(	PF00305.24	GO:003288	cytoplasmic	NP_033792	mmu:11689	
K01794	DSE	mmu00532(	PF16332.11	GO:003020	ER	NP_766096	mmu:212898	
K16667	PTPRG		PF00102.32	GO:001678	cytoplasmic	BAE25813.1	mmu:19270	
K11500	CENPH		PF05837.17	GO:000367	nuclear	NP_068686	mmu:26886	
K25700	CTHRC1		PF01391.23	GO:000548	lysosomal	AAI31908.1	mmu:68588	
K17285	SELENBP1	mmu00920(	PF05694.17	GO:000810	cytoplasmic	NP_033176	mmu:20341	
K23918	NEXN		PF07679.22	GO:000699	cytoplasmic	NP_001344	mmu:68810	
)1YN(Heterogeneous nuclear ribonu			PF13671.12	GO:000557	nuclear	NP_001074	mmu:68693	
K08870	TWF		PF00241.25	GO:009951	cytoplasmic	NP_036006	mmu:23999	
K01312	PRSS1_2_3	mmu05164(	PF00089.32	GO:000425	extracellula	NP_444473	mmu:114228	
K08790	STK38, NDR		PF00069.31	GO:003563	cytoplasmic	NP_001344	mmu:106504	
K27814	CAVIN3		PF15237.11	GO:009859	cytoplasmic	NP_082720	mmu:109042	
K12966	AIM2	mmu04621(	PF02760.21	GO:001922	cytoplasmic	NP_001013	mmu:383619	
K06030	MFN2, FZO	mmu04621(	PF04799.18	GO:200123	cytoplasmic	BAE41121.1	mmu:170731	
K12875	ACIN1, AC	mmu03013(	PF02037.33	GO:008009	nuclear	BAE22033.1	mmu:56215	
K09567	PPIH, CYP	mmu03040(	PF00160.26	GO:000645	cytoplasmic	BAE22936.1	mmu:66101	
K11220	STAT1	mmu05200(	PF01017.26	GO:000573	cytoplasmic	NP_001192	mmu:20846	STAT
K17523	CHI3L1_2		PF00704.34	GO:001922	ER	NP_031721	mmu:12654	
K14692	SLC30A5_7, ZNT5_7, M		PF01545.26	GO:000548	cytoplasmic	NP_075703	mmu:66500	
K02967	RP-S2, MRI	mmu03010(	PF00318.25	GO:002260	mitochondr	NP_536700	mmu:118451	
K08735	MSH2	mmu05200(	PF00488.27	GO:000962	cytoplasmic	NP_032654	mmu:17685	
K18082	MTMR3_4,	mmu04070(	PF06602.19	GO:001487	cytoplasmic	NP_001389	mmu:170749	
25KF(Immunity-related GTPase fan			PF05049.19	GO:002000	cytoplasmic	NP_001139	mmu:60440	
YIU(FLYWCH zinc finger domain)			PF04500.22	GO:000582	nuclear	NP_722486	mmu:224613	
K03943	NDUFV2	mmu05208(	PF01257.25	GO:000916	mitochondr	NP_082664	mmu:72900	
K16580	SLAIN1_2		PF15301.11	GO:003111	cytoplasmic	NP_001106	mmu:75991	
K04419	MAP3K11,	mmu04010(	PF07714.23	GO:009951	cytoplasmic	NP_001405	mmu:26403	
K18441	CYTH	mmu04144(	PF01369.25	GO:000592	cytoplasmic	NP_035312	mmu:19159	
K23532	WDFY2		PF00400.38	GO:001922	cytoplasmic	NP_780755	mmu:268752	
K12889	PCBP1	mmu04216(	PF00013.35	GO:005125	cytoplasmic	NP_001015	mmu:23983	
K18932	ZDHHC			GO:002153	Golgi appa	EDL94220.	mmu:74168	
K24553	DPY19L		PF10034.15	GO:000578	mitochondr	NP_001346	mmu:244745	
K25083	RBM47		PF00076.28	GO:008009	nuclear	NP_001278	mmu:245945	
K23964	CCNQ, FAM58A			GO:001921	nuclear	AAS20613.1	mmu:69109	
K10407	KLC	mmu05020(	PF13424.12(TPR_12:Te		nuclear	NP_001356	mmu:16594	
K00016	LDH, ldh	mmu05230(	PF00056.29	GO:005128	cytoplasmic	BAE26915.1	mmu:16828	
K09262	MEF2D	mmu04928(	PF00319.23	GO:008009	nuclear	NP_001397	mmu:17261	SRF
K23616	ATXN1_1L	mmu05017(	PF08517.17	GO:008009	nuclear	NP_001074	mmu:52335	
K11091	SNRPA	mmu03040(	PF00076.28	GO:004423	nuclear	NP_001040	mmu:53607	
K13513	LCLAT1, A	mmu00564(	PF01553.26	GO:000578	ER	NP_001074	mmu:225010	

K15225	TTF1	mmu04918(	PF00249.37	GO:000989	nuclear	NP_001408	mmu:22130	MYB
K16853	SLAMF8, CD353			GO:001922	plasma mer	EDL39006.	mmu:74748	
K18532	AK6, FAP7	mmu01240(	PF13238.12	GO:001920	cytoplasmic	NP_081868	mmu:102216272	
K06479	CD48, BCM	mmu04650(	PF13927.12	GO:000989	extracellulo	BAE96323.	mmu:12506	
K03164	TOP2	mmu01524(	PF00521.26	GO:008009	nuclear	NP_035753	mmu:21973	
K24897	HOPX		PF00046.35	GO:008009	nuclear	NP_001153	mmu:74318	Homeobox
K20995	NOTCH3	mmu05165(	PF00008.33	GO:008009	plasma mer	NP_032742	mmu:18131	
XY8D(In the hair cortex, hair kerati			PF04579.17	GO:009908	nuclear	NP_076000	mmu:69473	
K06052	JAG1, CD3	mmu05165(	PF00008.33	GO:200073	plasma mer	NP_038850	mmu:16449	
K12646	RIGI, DDX	mmu05164(	PF16739.11	GO:000959	cytoplasmic	AAI27164.1	mmu:230073	
K25186	RTF2		PF04641.18	GO:190231	nuclear	AAI16444.1	mmu:66404	
K11491	NCAPD3		PF12717.13	GO:003304	nuclear	ABC68604.	mmu:78658	
K20102	YTHDF		PF04146.21	GO:199024	cytoplasmic	EDL07359.	mmu:228994	
K19465	MGME1, DDK1		PF12705.13	GO:003204	mitochondr	NP_001276	mmu:74528	
K01403	MMP9	mmu04915(	PF00040.24	GO:200123	extracellulo	NP_038627	mmu:17395	
K06112	MAGI3	mmu04015(	PF00595.29	GO:000592	cytoplasmic	NP_001152	mmu:99470	
K05728	CSK		PF07714.23	GO:004340	cytoplasmic	EDL25918.	mmu:12988	
K04714	SGMS	mmu04071(	Sphingolipi	GO:000578	cytoplasmic	XP_011245	mmu:208449	
K12968	ADAR, AD	mmu05164(	PF02137.24	GO:005134	nuclear	NP_001139	mmu:56417	
K17757	CARKD		PF01256.23	GO:000548	mitochondr	NP_001177	mmu:69225	
K12493	ARFGAP2_	mmu04144(	PF01412.23	GO:000810	cytoplasmic	NP_079721	mmu:66251	
K17516	CLEC12A, CD371		PF00059.27	GO:003802	plasma mer	NP_808354	mmu:232413	
K04082	hscB, HSCB, HSC20		PF07743.18	GO:002260	mitochondr	NP_705799	mmu:100900	
K07209	IKBKB, IK	mmu05208(	PF18397.6(I	GO:000810	cytoplasmic	NP_034676	mmu:16150	
K13207	CUGBP, BRUNOL, CE		PF00076.28	GO:002260	cytoplasmic	NP_001393	mmu:13046	
K07834	RALA	mmu04014(	PF00071.28	GO:000810	cytoplasmic	NP_001401	mmu:56044	
K25094	EPB42		PF00927.28	GO:002260	cytoplasmic	NP_038541	mmu:13828	
K03957	NDUFB1	mmu05208(	PF08040.16(NADH_oxi		extracellulo	NP_001365	mmu:102631912	
K13800	CMPK1, UI	mmu01240(	PF00406.28	GO:000921	cytoplasmic	Q9DBP5.1(	mmu:66588	
K17987	NBR1	mmu04140(	PF16158.11	GO:001602	cytoplasmic	EDL34054.	mmu:17966	
K07899	RAB9A, RA	mmu05132(	PF00071.28	GO:000810	ER	XP_038173	mmu:56382	
K17284	PLIN2, AD	mmu03320(	PF03036.21	GO:001602	cytoplasmic	NP_001390	mmu:11520	
K08507	USE1	mmu04130(	PF09753.14	GO:003303	cytoplasmic	NP_001139	mmu:67023	
K03676	grxC, GLRX, GLRX2		PF00462.30	GO:009008	cytoplasmic	NP_001347	mmu:93692	
TopBP1-interacting, checkpoint, and			PF13465.12	GO:008009	cytoplasmic	NP_001344	mmu:26465	zf-C2H2
K12404	GGA	mmu04142(	PF00790.25	GO:001602	cytoplasmic	BAE35509.	mmu:74105	
K12888	HNRNPU	mmu03040(	PF13671.12	GO:000371	cytoplasmic	BAE35512.	mmu:51810	
K21988	TMC		PF07810.18	GO:000578	cytoplasmic	NP_663414	mmu:217353	
K04348	PPP3C, CN	mmu05020(	PF00149.33	GO:005116	cytoplasmic	NP_032939	mmu:19055	
K01092	E3.1.3.25, II	mmu04070(	PF00459.30	GO:004222	mitochondr	BAE32621.	mmu:114663	
K07933	RABL3		PF08477.19	GO:000810	cytoplasmic	NP_001035	mmu:67657	
K18464	RTSC, SPG	mmu04144(	PF10266.14	GO:000810	extracellulo	NP_705776	mmu:223593	
K03870	CUL2	mmu05200(	PF00888.28	GO:004438	cytoplasmic	BAD90212.	mmu:71745	

K11648	SMARCB1,	mmu03250(	PF04855.17	GO:003506	nuclear	NP_001007	mmu:20587
K15333	TRM3, TARBP1		PF00588.24	GO:000151	cytoplasmic	NP_001153	mmu:212728
K21773	LIN9	mmu04218(	PF19438.4(I	GO:008009	nuclear	XP_032771.	mmu:72568
K03676	grxC, GLRX, GLRX2		PF00462.30	GO:003042	mitochondr	NP_001033	mmu:69367
K12875	ACIN1, AC	mmu03013(	PF16294.10	GO:008009	nuclear	NP_075679	mmu:56215
K08766	CPT2	mmu03320(	PF00755.25	GO:003444	mitochondr	BAE29353.	mmu:12896
K24514	ITIH4		PF08487.16	GO:000695	extracellulo	EDL24769.	mmu:16427
K17425	MRPL44		PF22935.1(I	GO:001602	nuclear	BAE29642.	mmu:69163
K10414	DYNC2H, I	mmu04962(	PF12774.13	GO:000858	cytoplasmic	NP_084127	mmu:110350
K02264	COX5A	mmu05208(	PF02284.21	GO:001545	mitochondr	NP_031773	mmu:12858
)ZMY(protein-kinase, interferon-inc			PF05485.17	GO:004316	nuclear	NP_082686	mmu:72981 THAP
K01724	PCBD, phh	mmu00790(	PF01329.24	GO:000548	nuclear	AAH28642.	mmu:72562
K10601	SYVN1, HF	mmu04141(	PF13639.12	GO:200123	extracellulo	NP_001158	mmu:74126
K12161	URM1	mmu04122(	PF09138.17	GO:000815	nuclear	NP_080891	mmu:68205
K10251	HSD17B12,	mmu01040(	PF00106.31	GO:000663	ER	NP_062631	mmu:56348
K08889	FER, TYK3	mmu04520(	PF07714.23	GO:001922	cytoplasmic	NP_001033	mmu:14158
K10753	ASF1		PF04729.19	GO:003472	nuclear	NP_077146	mmu:66929
K14295	NUP50, NF	mmu03013(	PF08911.16	GO:003450	nuclear	NP_057923	mmu:18141
K12477	EHD4	mmu04144(	PF18150.6(I	GO:000810	cytoplasmic	BAE93881.	mmu:98878
K09564	PPIE	mmu03040(	PF00160.26	GO:000582	cytoplasmic	NP_062362	mmu:56031
K13345	PEX12, PA	mmu04146(	PF04757.19	GO:000810	peroxisoma	SDA08569.	mmu:103737
K20299	VPS53		PF04100.18	GO:003236	cytoplasmic	AAI68148.1	mmu:68299
YNFT(IGv); ENOG4111904(IGv); E			PF07686.23(V-set:Immu		plasma mer	DBA12489.1(TPA: IGHV2-5*01, pa	
K03691	POFUT	mmu00514(	PF10250.15	GO:000988	ER	NP_084538	mmu:80294
K10161	TLR9, CD2	mmu05235(	PF13855.12	GO:000959	extracellulo	NP_112455	mmu:81897
.ZB0(chromosome 9 open reading frame 89)				GO:003109	cytoplasmic	NP_081014	mmu:68480
K23049	HECA, HDC		PF16002.10	GO:000573	cytoplasmic	NP_001028	mmu:380629
)P7W(suppressor APC domain conta			PF11414.13	GO:000592	mitochondr	NP_001277	mmu:72080
K03538	POP4, RPP	mmu03008(	PF01868.21	GO:000454	nuclear	NP_079666	mmu:66161
K10646	TRAF7, RFWD1		PF00400.38	GO:200123	cytoplasmic	NP_001165	mmu:224619
.CJX(Alpha-2-glycoprotein 1, zinc-b			PF00129.23	GO:000989	extracellulo	NP_038506	mmu:12007
K05700	VCL	mmu04670(	PF01044.25	GO:004867	cytoplasmic	NP_033528	mmu:22330
K03016	RPABC3, R	mmu03420(	PF03870.21	GO:000367	nuclear	NP_001128	mmu:245841
K10365	CAPZB	mmu04144(	PF01115.22	GO:004846	cytoplasmic	NP_001005	mmu:12345
K02180	BUB3	mmu05166(	PF00400.38	GO:004584	cytoplasmic	XP_007935	mmu:12237
K24538	MBIP			GO:008009	nuclear	NP_663417	mmu:217588
K09289	NCOA4, PT	mmu05200(	PF12489.13	GO:000810	cytoplasmic	EDL24830.	mmu:27057
K20656	TMEM189	mmu00565(	PF10520.15	GO:000663	peroxisoma	NP_663513	mmu:407243
K04043	dnaK, HSP.	mmu03018(	PF00012.26	GO:005116	mitochondr	NP_034611	mmu:15526
K10956	SEC61A	mmu04141(	PF00344.25	GO:000578	peroxisoma	NP_001343	mmu:57743
KT0M(Melanoma antigen, family)			PF05773.27(RWD:RWD		nuclear	NP_987103	mmu:192174
K09574	FKBP8	mmu04137(	PF00254.34	GO:000810	mitochondr	NP_034353	mmu:14232
K02876	RP-L15, MF	mmu03010(	PF00828.25	GO:001593	mitochondr	NP_001171	mmu:27395

K09563	PPIC, CYPC		PF00160.26	GO:000645	ER	NP_032934	mmu:19038
K09571	FKBP4_5	mmu04915(	PF00254.34	GO:003111	cytoplasmic	NP_034349	mmu:14228
K12740	NKTR		PF00160.26	GO:000645	nuclear	EDL09136.	mmu:18087
K20647	ARHGAP32		PF00620.32	GO:004001	cytoplasmic	BAM34447.	mmu:330914
ZDNZ(Transmembrane protein 176			PF04103.21	GO:001602	plasma mer	NP_001157	mmu:65963
:oiled-coil domain containing 159)			PF00307.36	GO:001562	cytoplasmic	KAI258889	mmu:12797
K15167	MED24	mmu04919(	PF11277.13	GO:006026	cytoplasmic	NP_001348	mmu:23989
K15437	AIMP1, ARC1		PF01588.26	GO:004212	cytoplasmic	NP_001355	mmu:13722
XQAJ(Importin)			PF18829.7(I	GO:003450	cytoplasmic	NP_808389	mmu:240614
K14416	HBS1	mmu05134(	PF00009.33	GO:199053	cytoplasmic	NP_001036	mmu:56422
K05770	TSPO, BZR	mmu04979(	PF03073.20	GO:000959	extracellulo	NP_033905	mmu:12257
K03174	TRAF3	mmu05165(	PF21355.3(	GO:001922	cytoplasmic	NP_001409	mmu:22031
K10997	TIMELESS, TOF1, SWI		PF04821.19	GO:008009	nuclear	XP_006513	mmu:21853
K15301	STXBP3, MUNC18-3		PF00995.28	GO:000810	cytoplasmic	NP_035634	mmu:20912
K05117	MERTK, M	mmu04148(	PF07714.23	GO:000690	plasma mer	NP_032613	mmu:17289
K13051	ASRGL1, ic	mmu00250(	PF01112.23	GO:004428	mitochondr	NP_079886	mmu:66514
K23694	GRAP		PF00018.34	GO:002305	cytoplasmic	NP_082093	mmu:71520
K06037	THNSL2		PF14821.11	GO:000663	cytoplasmic	NP_001029	mmu:232078
K10626	UBR2		PF18995.5(I	GO:001922	cytoplasmic	BAE27917.1	mmu:224826
K21841	TBC1D24		PF00566.25	GO:008009	nuclear	NP_001157	mmu:224617
K10283	FBXL17			GO:000815	nuclear	AAH55780.	mmu:50758
K25866	NUDC		PF04969.22	GO:000645	cytoplasmic	NP_035078	mmu:18221
K05740	DIAPH1	mmu04810(	PF02181.28	GO:000810	cytoplasmic	BAD90493.	mmu:13367
K03945	NDUFA1	mmu05208(	PF15879.10	GO:002260	ER	NP_062316	mmu:54405
IAF(DNA-directed DNA polymera			PF01284.28	GO:003411	ER	NP_001087	mmu:50918
H0W(Sperm associated antigen 7)			PF01424.28	GO:004323	nuclear	NP_766149	mmu:216873
K03984	SERPINA1	mmu04610(	PF00079.25	GO:000695	extracellulo	SPC71822.1	mmu:20703
K14786	KRI1		PF12936.12	GO:003068	nuclear	NP_663391	mmu:215194
K22762	DESI1, PPPDE2		PF05903.20(Peptidase_(		cytoplasmic	CAH67912	mmu:28075
K15596	CDK18, PCTK3		PF00069.31	GO:005123	cytoplasmic	NP_032821	mmu:18557
K06553	VPREB, CD179a		PF07686.23	GO:000548	cytoplasmic	P18528.1(R	mmu:22363
K00234	SDHA, SDI	mmu05208(	PF00890.30	GO:000916	mitochondr	NP_075770	mmu:66945
K13091	RBM23_39		PF00076.28	GO:008009	nuclear	NP_001013	mmu:170791
?CDG(Chromosome 11 open readin			PF08939.15(Bles03:Basc		cytoplasmic	NP_598910	mmu:107242
K04408	MAP4K1, F	mmu04010(	PF00069.31	GO:004222	cytoplasmic	NP_032305	mmu:26411
K19020	STAB1	mmu04148(	PF02469.27	GO:000695	plasma mer	BAJ51907.1	mmu:192187
K15688	MUL1	mmu04137(	PF12483.13	GO:005068	cytoplasmic	NP_080965	mmu:68350
K23000	TOR3A, ADIR		PF06309.17	GO:000578	extracellulo	NP_075630	mmu:30935
K24624	MICOS13		PF15884.10	GO:004428	mitochondr	NP_694792	mmu:224904
K15189	HEXIM1_2		PF15313.11	GO:005134	nuclear	NP_620092	mmu:192231
K17581	CNST		PF15281.11	GO:190547	cytoplasmic	NP_666217	mmu:226744
K04370	LAMTOR3	mmu04010(	PF08923.16	GO:000810	cytoplasmic	NP_064304	mmu:56692
K10661	MARCH6, I	mmu04141(	PF23113.1(	GO:000827	cytoplasmic	NP_766194	mmu:223455

K02577	NDST2	mmu00534(	PF12062.14	GO:001681	Golgi appa	NP_034941	mmu:17423	
K20800	DPCD		PF14913.11	GO:004887	nuclear	NP_766227	mmu:226162	
K03846	ALG9	mmu00510(	PF03901.23	GO:005292	cytoplasmic	NP_598742	mmu:102580	
K04550	LRP1, CD9	mmu04148(	PF00057.23	GO:000690	plasma mer	NP_032538	mmu:16971	
ZUHK(family with sequence similarity)				GO:003243	cytoplasmic	NP_080796	mmu:68034	
Y9DJ(DNA-dependent RNA polyme			PF01448.29	GO:000989	cytoplasmic	NP_001303	mmu:70427	MYB
K11269	CTF18, CHL12		PF00004.35	GO:008009	nuclear	NP_663384	mmu:214901	
K11971	RNF14, ARA54		PF05773.27	GO:003243	cytoplasmic	NP_001158	mmu:56736	
K16479	ODF2				cytoplasmic	NP_001355	mmu:18286	
K06522	IGSF2_3, CD101		PF07686.23	GO:000557	plasma mer	NP_001391	mmu:78908	
K09434	ERF		PF00178.28	GO:000582	nuclear	NP_034285	mmu:13875	ETS
K12174	KPC2, UBAC1		PF22562.2(	GO:199023	cytoplasmic	NP_598596	mmu:98766	
K04490	TCF7L1	mmu05165(	PF08347.16	GO:008009	nuclear	NP_001073	mmu:21415	HMG
K17349	TSPAN8		PF00335.26	GO:005124	plasma mer	NP_001162	mmu:216350	
K18263	ADGRV1, GPR98, USH		PF03160.20	GO:000810	plasma mer	NP_473394	mmu:110789	
K07935	IFT22, RABL5		PF08477.19	GO:004299	cytoplasmic	NP_080349	mmu:67286	
K24485	FNDC3		PF00041.27	GO:006000	cytoplasmic	XP_006519	mmu:319448	
K07959	ARL11		PF00025.27	GO:000810	cytoplasmic	NP_796311	mmu:219144	
K00167	BCKDHB,	mmu01100(	PF02779.29	GO:005138	mitochondr	NP_001292	mmu:12040	
K10772	APEX2		PF03372.29	GO:001679	mitochondr	NP_084219	mmu:77622	
K19028	PFKFB1	mmu04922(	PF01591.24	GO:001922	cytoplasmic	NP_001361	mmu:18639	
K02178	BUB1	mmu04914(	PF08311.18	GO:200123	cytoplasmic	AAC53533.	mmu:12235	
K12597	AIR1_2	mmu03018(	PF00098.28	GO:007102	nuclear	NP_613056	mmu:319885	
K16939	SEPT6_8_1	mmu05100(	PF00735.24	GO:003215	cytoplasmic	NP_001170	mmu:56526	
K25628	TCP11		PF05794.18	GO:000573	cytoplasmic	NP_666120	mmu:216198	
K04727	AIFM1, PD	mmu04217(	PF14721.12	GO:000810	mitochondr	NP_001277	mmu:26926	
K18267	NEB	mmu04820(	PF00880.23(Nebulin:N		cytoplasmic	XP_036015	mmu:17996	
K06061	MAML	mmu05165(	PF20801.2(	GO:000548	nuclear	AAS07633.1	mmu:103806	
K07860	RAC2	mmu05020(	PF00071.28	GO:009951	cytoplasmic	NP_001008	mmu:19354	
Phosphatidylcholine transfer protein			PF02121.25	GO:000531	cytoplasmic	NP_062614	mmu:56305	
Aryl hydrocarbon receptor nuclear t			PF02121.25	GO:003250	cytoplasmic	NP_032876	mmu:18738	
K13822	HBA	mmu05144(	PF00042.28	GO:000534	cytoplasmic	AAA37783.	mmu:15122	
KRAP(Regulation of nuclear pre-mI			PF04818.19	GO:001989	cytoplasmic	NP_001074	mmu:75137	
K04461	PPM1B, PP	mmu04010(	PF00481.26	GO:005068	cytoplasmic	AAH90963.	mmu:19043	
K04371	ERK, MAP	mmu05208(	PF00069.31	GO:005084	cytoplasmic	NP_001033	mmu:26413	
K24261	MAP11		PF15806.11	GO:002260	cytoplasmic	NP_694801	mmu:231807	
K01408	IDE, ide	mmu05010(	PF16187.10	GO:000578	cytoplasmic	NP_112419	mmu:15925	
K04436	MAPK8IP3	mmu04010(	PF19056.6(	GO:000810	cytoplasmic	NP_001156	mmu:30957	
K24061	PDZD11		PF00595.29	GO:009859	cytoplasmic	NP_001100	mmu:72621	
Glutaredoxin)			PF00462.30	GO:001474	cytoplasmic	NP_075629	mmu:30926	
K00727	GCNT1	mmu00512(	PF02485.27	GO:000837	Golgi appa	XP_006526	mmu:14537	
K09170	NFIC		PF00859.23	GO:000989	nuclear	KAF630676	mmu:18029	MH1
K07865	RHOU, WRCH1		PF00071.28	GO:002260	cytoplasmic	AAI34408.1	mmu:69581	

K12031	TRIM65		PF00622.36	GO:001922(	cytoplasmic	NP_848917	mmu:338364	
K10577	UBE2I, UB(	mmu03013(	PF00179.31	GO:000815.	mitochondr	KAG85579.	mmu:22196	
K24787	RUNDC3		PF02759.24	GO:004858	mitochondr	NP_058039	mmu:51799	
K23859	PLEKHG1_2_3		PF00621.25	GO:000508.	cytoplasmic	NP_722499	mmu:263406	
K03000	RPA12, PO	mmu03020(	PF01096.23	GO:003088(	nuclear	NP_001342	mmu:66136	
.GK5(CUE domain containing 2)				GO:001602(	cytoplasmic	NP_001157	mmu:67116	
K00314	SARDH	mmu00260(	PF01266.30	GO:000573(	cytoplasmic	BAE37445.1	mmu:192166	
K02353	DVL	mmu05165(	PF12316.13	GO:000810	cytoplasmic	AAH92396.	mmu:13543	
K13346	PEX10	mmu04146(	PF04757.19	GO:000810	peroxisoma	NP_001035	mmu:668173	
ZE8R(resistin)			PF06954.16	GO:000512.	extracellulo	EDK98099.	mmu:57262	
K06910	PEBP, TFS1		PF01161.25	GO:001922(	cytoplasmic	NP_061346	mmu:23980	
K25206	TMEM127		PF20517.3('	GO:000828.	plasma mer	NP_780354	mmu:69470	
K15119	SLC25A39_40		PF00153.32	GO:004846	cytoplasmic	NP_001276	mmu:319653	
ho GTPase activating protein)			PF13465.12	GO:000097'	cytoplasmic	BAE34175.1	mmu:22720	zf-C2H2
K04572	NEFL, NF-	mmu05014(	PF00038.26	GO:005080	cytoplasmic	NP_035040	mmu:18039	
K07361	LCP2	mmu04664(	PF00017.30	GO:009859(	cytoplasmic	NP_034826	mmu:16822	
K14440	SMARCAL1, HARP		PF07443.18	GO:005125.	nuclear	EDL00284.'	mmu:54380	TF_others
K12850	PRPF38B	mmu03040(	PF03371.20	GO:000613(	nuclear	NP_080121	mmu:66921	
3R-related CTD-associated factor 4)			PF00962.28	GO:000548	cytoplasmic	NP_083751	mmu:75894	
K02146	ATPeV0D,	mmu05165(	PF01992.21	GO:004323	cytoplasmic	NP_780615	mmu:242341	
K06555	CD180		PF13855.12	GO:000226.	plasma mer	NP_032559	mmu:17079	
K04468	NLK	mmu04310(	PF00069.31	GO:001989(	cytoplasmic	NP_001178	mmu:18099	
K23541	TMEM165, GDT1		PF01169.24	GO:003575	Golgi appa	AAI41082.1	mmu:21982	
.CF7(IGv); ENOG41115I2(IGv); EN			PF07686.23(V-set:Immu		extracellulo	CAB46127.1(immunoglobulin light		
.CF7(IGv); ENOG41115I2(IGv); EN			PF07686.23(V-set:Immu		extracellulo	CAB46138.1(immunoglobulin light		
K26469	CCDC51, MITOK			GO:001972.	mitochondr	NP_079965	mmu:66658	
KRRG(negative regulation of Rho p			PF12070.13	GO:001602(	cytoplasmic	NP_848893	mmu:320271	
K22593	CHAMP1			GO:000810	cytoplasmic	NP_001350	mmu:101994	
Cross-linked envelope protein of ker			PF13465.12	GO:008009(	cytoplasmic	NP_036111	mmu:26466	zf-C2H2
K24796	TBC1D13		PF00566.25	GO:000810	cytoplasmic	NP_666364	mmu:70296	
K15728	LPIN	mmu04936(	PF08235.19	GO:000578.	nuclear	XP_006515(	mmu:14245	
ZQIE(Beta-Ig-H3 fasciclin)			PF01335.27	GO:190204.	cytoplasmic	NP_001316	mmu:18611	
K05765	CFL	mmu05170(	PF00241.25	GO:003250.	cytoplasmic	NP_001102	mmu:12632	
K24886	TOGARAM		PF21040.3((	GO:003111(	cytoplasmic	XP_006516(	mmu:328108	
K23194	GATAD2	mmu03082(	PF16563.10	GO:200073	cytoplasmic	XP_030099.	mmu:234366	
K01278	DPP4, CD2	mmu03272(	PF00930.27	GO:000425.	lysosomal	NP_034204	mmu:13482	
K11090	LA, SSB	mmu05322(	PF08777.17	GO:005116(	cytoplasmic	NP_001103	mmu:20823	
K13143	INTS6, DDX26		PF15300.11	GO:001562(	nuclear	NP_032741	mmu:18130	
K15601	KDM3	mmu04714(	PF22989.2(I	GO:200073	plasma mer	BAC98014.1	mmu:104263	
K07426	CYP4B1		PF00067.27	GO:000449'	Golgi appa	NP_031849	mmu:13120	
K11589	MBD1		PF01429.25	GO:008009(	nuclear	NP_038622	mmu:17190	MBD
K10413	DYNC1H	mmu04962(	PF08385.17	GO:003238	cytoplasmic	NP_084514	mmu:13424	
K12169	KPC1, RNF123		PF00622.36	GO:000367	cytoplasmic	AAH25530.	mmu:84585	

K22285	OSBPL8, ORP8		PF01237.23	GO:000810	cytoplasmic	XP_006513	mmu:237542	
K00261	GLUD1_2,	mmu01100(	PF00208.27	GO:009008	mitochondr	NP_032159	mmu:14661	
Y9WN(Spermatogenesis associated,			PF07139.16	GO:000573	cytoplasmic	EDL00058.	mmu:67198	
K09680	PANK1_2_	mmu01240(	PF03630.20	GO:001593	cytoplasmic	NP_666074	mmu:211347	
K09142	SPOUT1		PF02598.22	GO:001922	cytoplasmic	NP_766248	mmu:227695	
K01027	OXCT	mmu00650(	PF01144.29	GO:003577	mitochondr	BAE26941.	mmu:67041	
K03627	MBF1		PF08523.15	GO:003015	nuclear	NP_001100	mmu:59022	
K12176	COPS2, CSN2, TRIP15		PF01399.33	GO:008009	cytoplasmic	BAE26937.	mmu:12848	
K09516	RETSAT	mmu00830(	PF13450.12	GO:000578	ER	NP_080435	mmu:67442	
)155(capping protein (actin filamen			PF14940.11	GO:000557	ER	NP_001074	mmu:71667	
K09385	FOXG	mmu04068(	PF00250.24	GO:008009	nuclear	NP_001153	mmu:15228	Fork head
K08819	CDK12_13		PF00069.31	GO:001922	nuclear	NP_001103	mmu:69131	
K06560	MRC, CD2(	mmu04145(	PF00059.27	GO:003802	plasma mer	AAI16643.1	mmu:17534	
K10489	ZBTB2		PF00651.37	GO:000268	nuclear	BAE21095.	mmu:38199	ZBTB
K23518	MACROD, ymdB		PF01661.27	GO:001679	mitochondr	NP_598908	mmu:107227	
K04798	pfdB, PFDN6		PF01920.25	GO:002260	cytoplasmic	ERE92019.	mmu:14976	
				GO:004887	nuclear	AAH56204.	mmu:18617	
K02087	CDK1, CD	mmu05170(	PF00069.31	GO:003304	cytoplasmic	NP_031685	mmu:12534	
K19021	PDE3A	mmu05032(	PF00233.25	GO:008009	Golgi appa	NP_061249	mmu:54611	
K20045	ITSN		PF16652.11	GO:004863	cytoplasmic	NP_001185	mmu:20403	
K20099	YTHDC2		PF04146.21	GO:199024	cytoplasmic	NP_001156	mmu:240255	
K04177	CCR2, CD1	mmu04062(	PF00001.26	GO:000690	plasma mer	NP_034045	mmu:12772	
K27833	ZNHIT6		PF04438.22	GO:003303	nuclear	NP_001074	mmu:229937	
.RRF(IGv); ENOG410YQB2(immu			PF07686.23(V-set:Immu		plasma mer	CAB46314.1(immunoglobulin light		
K06553	VPREB, CD179a		PF07686.23(V-set:Immu		cytoplasmic	CAA35629.	mmu:22363	
K06553	VPREB, CD179a		PF07686.23(V-set:Immu		extracellulo	CAA75912.	mmu:22364	
K20996	NOTCH4	mmu05165(	PF00008.33	GO:000578	extracellulo	AAI38045.1	mmu:18132	
YIBB(nucleolar protein 4)			PF23079.1(HTH_NOL4		nuclear	NP_001001	mmu:329540	
K07604	KRT1	mmu04915(	PF00038.26	GO:009951	cytoplasmic	NP_075745	mmu:66809	
K01340	E3.4.21.59	mmu05164(	PF00089.32	GO:000425	lysosomal	P21845.2(R	mmu:17229	
.F0T(Family with sequence similari			PF06271.18	GO:000083	cytoplasmic	BAE42204.	mmu:97863	
K05481	IL1RN, IL1	mmu04060(	PF00340.25	GO:003411	cytoplasmic	NP_001153	mmu:16181	
KRPV(secretoglobin, family 1B, mer			PF16455.10(UBD:Ubiq		cytoplasmic	NP_663475	mmu:226122	
K10694	ZNRF1_2		PF13639.12	GO:004858	cytoplasmic	NP_001162	mmu:170737	
K06476	ITGA2B, C	mmu05165(	PF20806.3(I	GO:000989	plasma mer	AAI37571.1	mmu:16399	
K03968	NDUFC2	mmu05208(	PF06374.16	GO:001922	cytoplasmic	NP_077182	mmu:68197	
.DIV(secretoglobin, family 1B, mem			PF01099.22	GO:000573	extracellulo	NP_033726	mmu:11354	
K00826	E2.6.1.42, il	mmu00290(	PF01063.24	GO:004322	mitochondr	NP_033867	mmu:12036	
K17804	TIM44		PF04280.21	GO:000810	mitochondr	NP_035722	mmu:21856	
K04985	PKD1				plasma mer	XP_036016	mmu:18763	
K20464	OSBPL5, O	mmu04979(	PF01237.23	GO:000554	nuclear	NP_077251	mmu:79196	
KRXR(transmembrane protein 245)			PF01594.21	GO:000557	plasma mer	NP_001356	mmu:242474	
K13249	SSR1	mmu04141(	PF03896.21	GO:000578	ER	NP_001347	mmu:107513	

K07304	msrA		PF01625.27	GO:001562	mitochondr	NP_001240	mmu:110265	
K24144	NHS		PF15273.11	GO:003250	cytoplasmic	AAI58017.1	mmu:195727	
:alcineurin-like phosphoesterase doi			PF00733.27	GO:000906	cytoplasmic	NP_598489	mmu:70396	
.5RF(Sp140 nuclear body)			PF00429.24	GO:000557	plasma mer	AAH67002.	mmu:110956	
K24991	MTHFSD		PF01812.26(5-FTHF_cy		cytoplasmic	NP_001359	mmu:234814	
K21814	CPPED1		PF00149.33	GO:000573	cytoplasmic	NP_666179	mmu:223978	
K00700	GBE1, glgB	mmu00500(	PF02806.24	GO:003024	cytoplasmic	NP_083079	mmu:74185	
K24052	RBM20			GO:000548	nuclear	NP_001164	mmu:73713	
K20776	BABAM, N	mmu03440(Homologou		GO:004423	cytoplasmic	NP_080912	mmu:68251	
K24674	ZMYM1		PF14291.11	GO:004316	mitochondr	EDL30260.	mmu:68310	
K23692	PKN3	mmu04151(	PF00069.31	GO:003563	cytoplasmic	XP_006498	mmu:263803	
K03998	C8B	mmu05171(	PF01823.24	GO:000367	extracellulo	NP_598643	mmu:110382	
K06746	B7H3, CD2	mmu04514(	PF13927.12	GO:004213	plasma mer	NP_001411	mmu:102657	
K24504	SP140		PF03172.18	GO:000573	nuclear	NP_001013	mmu:43448	SAND
K14798	LTV1		PF04180.19	GO:005116	nuclear	NP_852135	mmu:353258	
liacylglycerol kinase)			PF00667.26	GO:002260	cytoplasmic	NP_001075	mmu:78797	
K23740	SH3BGR		PF04908.21	GO:000573	nuclear	XP_032756	mmu:50795	
K24164	TPRN		PF13914.11	GO:003003	cytoplasmic	NP_780495	mmu:97031	
K01084	G6PC	mmu00052(	PF01569.27	GO:001920	ER	NP_787949	mmu:68401	
K06787	ESAM	mmu04670(	PF07686.23	GO:000592	extracellulo	NP_081378	mmu:69524	
KS4V(chromosome 12 open reading			PF10154.14	GO:005104	cytoplasmic	NP_001397	mmu:28040	
YDRZ(chromosome 12 open reading			PF13289.12	GO:000367	cytoplasmic	NP_001343	mmu:73225	
K12855	PRPF6, PRI	mmu03040(	PF06424.17	GO:000024	nuclear	NP_598462	mmu:68879	
K12196	VPS4	mmu03250(	PF00004.35	GO:000810	cytoplasmic	NP_569053	mmu:116733	
K09556	BAG2	mmu04141(Protein pro		GO:000989	cytoplasmic	NP_663367	mmu:213539	
K05766	SSH	mmu04810(	PF23040.1(I	GO:001679	cytoplasmic	NP_001343	mmu:245857	
K00326	CYB5R	mmu00520(	PF00173.33	GO:000810	mitochondr	NP_077157	mmu:266690	
K20103	DDX60		PF23002.2(I	GO:003563	cytoplasmic	NP_001280	mmu:234311	
K00078	DHDH	mmu00980(	PF01408.29	GO:001932	peroxisoma	AAH31710.	mmu:71755	
K08146	SLC2A9, GLUT9		PF00083.30	GO:009859	plasma mer	NP_001095	mmu:117591	
K22823	NSMCE3, NSE3		PF01454.25	GO:008009	mitochondr	BAD21407.	mmu:66647	
K21754	KCTD1_15		PF20871.2(I	GO:002260	nuclear	NP_001347	mmu:233107	
K21853	DOCK9_10_11		PF14429.12	GO:003126	cytoplasmic	NP_001393	mmu:210293	
K26145	ZSWIM1		PF21056.3(Z	GO:004316	peroxisoma	NP_082304	mmu:71971	
K24627	GAS2, GAS2L		PF02187.23	GO:003243	mitochondr	NP_001317	mmu:14453	
K16795	PAFAH1B2	mmu00565(	PF13472.12	GO:000573	cytoplasmic	XP_031199	mmu:18475	
KNQX(Cyclin Y)			PF00134.28(Cyclin_N:C		cytoplasmic	XP_021027	mmu:227210	
K03988	C1QG	mmu05171(	PF00386.27	GO:005080	cytoplasmic	SOR70270.1	mmu:12262	
K02835	prfA, MTRF1, MRF1		PF03462.23	GO:004393	mitochondr	NP_780583	mmu:108853	
K08789	MAST, IREH1		PF08926.16	GO:003563	cytoplasmic	NP_955012	mmu:546071	
K12373	HEXA_B	mmu01100(	PF00728.28	GO:003197	lysosomal	NP_034551	mmu:15211	
K14000	RRBP1	mmu04141(	PF05104.17	GO:007170	ER	NP_077243	mmu:81910	
K13758	PDE6D	mmu00230(	PF05351.17	GO:009859	cytoplasmic	NP_032827	mmu:18582	

K15336	TRDMT1, DNMT2		PF00145.23	GO:000989	cytoplasmic	NP_034197	mmu:13434
[ELO2 interacting protein 2)			PF00171.27	GO:000403	cytoplasmic	NP_666066	mmu:69748
K26471	LZTS2	mmu04310(	PF06818.20	GO:005116	cytoplasmic	NP_001123	mmu:226154
K26680	DOP1, DOPEY		PF24601.1(	GO:004322	nuclear	NP_796182	mmu:320615
K17943	PUM	mmu05017(	PF00806.25	GO:003577	cytoplasmic	EDL30135.	mmu:80912
K05614	SLC1A3, E/	mmu04724(	PF00375.23	GO:000962	peroxisoma	NP_683740	mmu:20512
K23758	S100A1		PF01023.25	GO:008009	cytoplasmic	P56565.2(R	mmu:20193
K06710	ICOSLG, I(	mmu04672(	PF07686.23	GO:001922	plasma mer	AAK77544.	mmu:50723
K13157	RNPC3		PF00076.28	GO:000613	nuclear	BAD32550.	mmu:67225
K10737	MCM8		PF00493.29	GO:000810	nuclear	NP_001277	mmu:66634
K05070	IL2RG, CD	mmu05166(	PF21605.3(	GO:003308	peroxisoma	EDL14151.	mmu:16186
K17844	PTBP3, ROD1		PF13893.12	GO:003311	nuclear	NP_001391	mmu:230257
K11113	TERF2IP, RAP1		PF08914.17	GO:000810	cytoplasmic	NP_065609	mmu:57321
K07541	PIGX	mmu00563(	PF08320.17	GO:000665	ER	NP_001104	mmu:72084
K05717	FN1	mmu05165(	PF00041.27	GO:002260	extracellula	NP_001263	mmu:14268
K21752	DRAP1, NC2-alpha		PF00808.29	GO:004280	nuclear	NP_001278	mmu:66556
K03768	PPIB, ppiB		PF00160.26	GO:000578	ER	NP_035279	mmu:19035
K03165	TOP3	mmu03440(	PF01131.25	GO:007110	mitochondr	NP_001313	mmu:21976
K00869	MVK, mval	mmu00900(	PF00288.32	GO:000449	cytoplasmic	NP_001293	mmu:17855
K14994	SLC38A7_8		PF01490.24	GO:004299	plasma mer	NP_001413	mmu:234595
K08291	GRK4_5_6	mmu04062(	PF00069.31	GO:003563	cytoplasmic	NP_001033	mmu:26385
K00993	EPT1	mmu00440(	Phosphonat	GO:000665	cytoplasmic	EDL37272.	mmu:28042
K24514	ITIH4		PF08487.16	GO:003020	extracellula	NP_061216	mmu:16427
K10883	EME2	mmu03460(	PF21292.3(I	GO:000548	nuclear	NP_001156	mmu:193838
K23796	PLEKHA1_2		PF00169.35	GO:000810	cytoplasmic	NP_001333	mmu:101476
K26419	AKTIP, FTS		PF00179.31	GO:000810	cytoplasmic	NP_001289	mmu:14339
K17259	CIB1		PF13499.12	GO:003111	cytoplasmic	NP_001278	mmu:23991
K26093	MAD2L1BI	mmu04110(	PF06581.17	GO:004858	cytoplasmic	AAH05768.	mmu:66591
K11414	SIRT4, SIR	mmu00760(	PF02146.23	GO:009008	mitochondr	NP_001161	mmu:75387
K10420	DYNLT1_3	mmu05132(	PF03645.18	GO:009951	cytoplasmic	NP_080251	mmu:67117
K02135	ATPeF1E,	mmu05208(	PF04627.18	GO:000526	mitochondr	NP_080259	mmu:67126
K02146	ATPeV0D,	mmu05165(	PF01992.21	GO:009877	cytoplasmic	NP_001011	mmu:11972
K01518	NUDT2	mmu00240(	PF00293.34	GO:000920	cytoplasmic	NP_079815	mmu:66401
K02207	UBE2R, UE	mmu04120(	PF00179.31	GO:009008	cytoplasmic	NP_001346	mmu:216150
K06553	VPREB, CD179a		PF07686.23(V-set:Immu		cytoplasmic	WGF13054.	mmu:22362
K01489	cdd, CDA	mmu00983(	PF00383.29	GO:001922	cytoplasmic	NP_082452	mmu:72269
IPV(Chromosome X open reading frame)					mitochondr	NP_081063	mmu:68618
K03944	NDUFV3	mmu05208(	PF15880.10	GO:004639	mitochondr	NP_001077	mmu:78330
K13899	CST3	mmu04970(	PF00031.27	GO:009951	ER	NP_034106	mmu:13010
K17387	KIF23	mmu04814(	PF00225.29	GO:009951	nuclear	NP_077207	mmu:71819
K08595	SENP6		PF02902.25	GO:003304	nuclear	AAI51129.1	mmu:215351
K01698	hemB, ALA	mmu01240(	PF00490.27	GO:003202	mitochondr	NP_001263	mmu:17025
K10402	KIF20	mmu04814(	PF00225.29	GO:003315	nuclear	NP_001349	mmu:240641

K01672	CA	mmu00910(	PF00194.26	GO:000548	cytoplasmic	NP_033932	mmu:12353	
YR2K(Chromosome 12 open reading			PF23999.1((	GO:005078	nuclear	NP_082487	mmu:72357	
K13204	ZNF326		PF04988.17	GO:004348	nuclear	NP_061229	mmu:54367	TF_others
K20296	ANG2, VPS51		PF15469.12	GO:003236	cytoplasmic	NP_001074	mmu:68505	
K05843	TGFBR3		PF00100.29	GO:004298	plasma mer	NP_035708	mmu:21814	
K01076	ABHD17		PF12146.15	GO:009859	lysosomal	NP_001094	mmu:70178	
K02326	POLE3	mmu03030(	PF00808.29	GO:008009	cytoplasmic	KAF64866(	mmu:59001	
K00021	HMGCR	mmu00900(	PF00368.23	GO:003288	ER	NP_001347	mmu:15357	
K05756	ARPC3	mmu04530(	PF04062.19	GO:000520	cytoplasmic	NP_062798	mmu:56378	
K01192	E3.2.1.25, M	mmu04142(	PF22666.2((	GO:000548	lysosomal	NP_081564	mmu:110173	
K17578	ZCCHC9		PF00098.28	GO:000557	nuclear	NP_663428	mmu:69085	
K24349	UBXN2A	mmu04141(	PF08059.18	GO:000578	cytoplasmic	NP_663416	mmu:217379	
K16763	CEP63		PF17045.10	GO:190187	cytoplasmic	XP_011241	mmu:28135	
K27704	MTCL		PF11365.13	GO:000989	cytoplasmic	NP_001158	mmu:320706	
K06789	ASAM, CLMP		PF07686.23	GO:003250	plasma mer	NP_598494	mmu:71566	
K16175	SCRIB	mmu05165(	PF00595.29	GO:004213	cytoplasmic	NP_598850	mmu:105782	
K23399	MGA	mmu03083(	PF00907.27	GO:008009	nuclear	A2AWL7.1(	mmu:29808	bHLH
ZQX4(Zinc finger, C3HC-type conta			PF07967.19	GO:200123	cytoplasmic	NP_001298	mmu:232679	
K24035	APBA3, 11L2		PF00640.29	GO:000810	cytoplasmic	NP_061228	mmu:57267	
K00940	ndk, NME	mmu01240(	PF00334.24	GO:000921	mitochondr	XP_006524	mmu:56520	
K20314	ARFIP		PF06456.18	GO:005112	cytoplasmic	NP_001074	mmu:99889	
K14291	PHAX	mmu03013(	PF10258.14	GO:001593	cytoplasmic	NP_001156	mmu:56698	
K22030	LMOD	mmu04820(	PF03250.19	GO:009951	cytoplasmic	NP_444336	mmu:93689	
K01950	E6.3.5.1, NA	mmu01240(	PF00795.27	GO:000548	cytoplasmic	EDL18235.	mmu:78914	
K04498	EP300, CRI	mmu03083(	PF08214.17	GO:003288	nuclear	NP_001020	mmu:12914	
K24351	UBXN8	mmu04141(	PF00789.26	GO:000578	ER	NP_848763	mmu:108159	
K17605	PPP2R4, PT	mmu04931(	PF03095.20	GO:003530	cytoplasmic	NP_620087	mmu:110854	
K06270	PPP1R12A,	mmu04024(	PF00023.36	GO:003243	cytoplasmic	NP_001355	mmu:17931	
K12619	XRN2, RAT	mmu03008(	PF17846.6((	GO:001922	nuclear	NP_036047	mmu:24128	
K24045	JMY		PF15871.10	GO:008009	cytoplasmic	NP_067285	mmu:57748	
K03006	RPB1, POL	mmu03420(	PF04997.17	GO:008009	cytoplasmic	AAA40071.	mmu:20020	
K20125	FCHSD		PF14604.12	GO:009953	cytoplasmic	EDL10106.	mmu:319262	
K14216	OAS	mmu05164(	PF10421.14	GO:001922	cytoplasmic	NP_660262	mmu:246728	
K13147	INTS10		PF21045.3(I	GO:001618	nuclear	NP_001280	mmu:70885	
K21889	TMBIM6, BI1, TEGT		PF01027.25	GO:200123	cytoplasmic	NP_001164	mmu:110213	
Myeloid leukemia factor)			PF00107.32	GO:001661	mitochondr	NP_001028	mmu:226527	
K17968	TRIAP1, MDM35		PF05254.17	GO:200123	cytoplasmic	XP_045390	mmu:69076	
XPA0(Nuclear receptor interacting p			PF09668.15	GO:003164	cytoplasmic	XP_029331	mmu:69994	
XQH0(Indole-3-acetic acid-amido sy			PF25146.1((	GO:000573	ER	BAE25606.1	mmu:80860	
K19030	PFKFB4	mmu04152(	PF01591.24	GO:001920	cytoplasmic	XP_017168	mmu:270198	
K11339	MORF4L1,	mmu03082(	PF05712.18	GO:003124	nuclear	XP_007458	mmu:627352	
K05449	VEGFC_D	mmu04010(	PF00341.22	GO:001922	extracellula	NP_034346	mmu:14205	
K03011	RPB3, POL	mmu03420(	PF01000.31	GO:003277	cytoplasmic	NP_001012	mmu:20021	

K22524	WBP2		PF02893.26	GO:005084	cytoplasmic	NP_058548	mmu:22378	
K22591	ADNP		PF19627.4(	GO:001922	nuclear	NP_001297	mmu:11538	Homeobox
K14300	NUP133	mmu03013(	PF03177.20	GO:003450	mitochondr	NP_758492	mmu:234865	
K24927	TTC4, CNS1		PF18972.5(	GO:000645	cytoplasmic	BAC40504.	mmu:72354	
K14008	ERLEC1, X	mmu04141(	PF07915.19	GO:003650	extracellulo	NP_080021	mmu:66753	
:obalamin synthesis protein)			PF02492.25	GO:001972	cytoplasmic	NP_666209	mmu:226043	
XPSC(general transcription factor II			PF18658.7(	GO:000971	cytoplasmic	EDL08974.	mmu:77781	
K11867	STAMBPL1		PF08969.16	GO:006157	cytoplasmic	NP_001347	mmu:76630	
K24507	VWA1		PF00092.34	GO:002260	mitochondr	NP_680085	mmu:246228	
K05906	PCYOX1, F	mmu00900(	PF07156.20	GO:004423	ER	BAE35532.	mmu:66881	
K11315	TADA3, AI	mmu05165(	PF10198.14	GO:001922	nuclear	NP_598693	mmu:101206	
ZQ95(Coiled-coil domain containing 127)					nuclear	NP_001162	mmu:67433	
K05654	ABCB3, TA	mmu02010(	PF00664.28	GO:000810	ER	NP_035660	mmu:21355	
K05717	FN1	mmu05165(	PF00041.27	GO:000167	plasma mer	NP_034363	mmu:14268	
K20465	OSBPL9_10_11, ORP9_		PF01237.23	GO:003293	cytoplasmic	XP_021014	mmu:100273	
)XXW(chromosome 12 open reading			PF10218.14	GO:004858	extracellulo	NP_001074	mmu:76792	
K17805	PAM16, TIM16		PF03656.18	GO:200123	mitochondr	NP_079847	mmu:66449	
K23880	RETREG		PF24456.1(	GO:004349	ER	EDL03887.	mmu:67998	
K17403	MRPS24		PF14955.12	GO:001593	mitochondr	NP_080356	mmu:64660	
XRSX(gametogenetin binding protein 2)				GO:003250	nuclear	XP_017169	mmu:217039	
K18175	CCDC56, C	mmu04714(	PF09813.14	GO:003361	mitochondr	NP_080894	mmu:52469	
K17597	STAU		PF16482.11	GO:000810	cytoplasmic	NP_035620	mmu:20853	
K01907	AACS, acsA	mmu00650(	PF00501.34	GO:009008	cytoplasmic	NP_084486	mmu:78894	
K03989	C4	mmu05171(	PF07678.20	GO:004299	cytoplasmic	AAA40115.	mmu:625018	
K01329	CMA1	mmu04614(	PF00089.32	GO:000425	plasma mer	AAI39455.1	mmu:17228	
K12275	SEC62	mmu04141(	Protein pro	GO:000810	nuclear	BAB29058.1	mmu:69276	
K25762	NAT9		PF13302.13	GO:003299	cytoplasmic	NP_001349	mmu:66176	
K16588	HAUS5, AUG5		PF14817.11	GO:002260	cytoplasmic	AAH89002.	mmu:71909	
K10894	FANCG	mmu03460(	PF07719.23	GO:004423	cytoplasmic	NP_001156	mmu:60534	
K00624	E2.3.1.7	mmu04146(	PF00755.25	GO:003444	peroxisoma	NP_031786	mmu:12908	
K15177	LEO1		PF04004.18	GO:008009	nuclear	XP_017168	mmu:235497	
.9UF(kiaa1551)			PF15395.11	GO:000989	nuclear	NP_001276	mmu:67246	
K20912	CARD10	mmu04064(	PF00619.27(CARD:Cas		nuclear	XP_021039	mmu:105844	
K03106	SRP54, ffh	mmu03060(	PF00448.28	GO:000578	cytoplasmic	NP_001093	mmu:24067	
K03021	RPC2, POL	mmu04623(	PF00562.33	GO:001922	cytoplasmic	NP_081699	mmu:70428	
K13189	RBM14		PF00076.28	GO:008009	cytoplasmic	NP_063922	mmu:56275	
K10607	TRIM32, H	mmu04120(	PF01436.27	GO:200123	cytoplasmic	NP_001155	mmu:69807	
.YE0(immunoglobulin heavy variab			PF07686.23	GO:000548	cytoplasmic	ASU89883.1(anti-HIV fusion peptic		
.U7Z(Apoptosis, caspase activation inhibitor)				GO:001094	cytoplasmic	AAI00736.1	mmu:74268	
K15901	TPRKB, CGI121		PF08617.15	GO:000815	cytoplasmic	NP_001163	mmu:69786	
K06553	VPREB, CD179a		PF07686.23	GO:000548	ER	DBA12477.	mmu:22362	
K17310	SAA		PF00277.23	GO:003435	peroxisoma	AAA40087.	mmu:20209	
K22746	CIAPIN1, DRE2		PF20922.3(	GO:002260	cytoplasmic	NP_598902	mmu:109006	

K11468	YAF2	mmu03083(	PF17219.8(	GO:000989	nuclear	NP_077151	mmu:67057	
K23899	RCN2		PF00036.39	GO:000578	ER	BAE41353.	mmu:26611	
K12947	SPCS2, SPC	mmu03060(	PF06703.16	GO:000578	cytoplasmic	XP_031243	mmu:66624	
K13187	RBM4		PF00076.28	GO:003292	nuclear	NP_001277	mmu:102902673	
K15168	MED25		PF11265.14	GO:008009	nuclear	NP_001318	mmu:75613	
K03574	mutT, NUDT15, MTH2		PF00293.34	GO:004222	cytoplasmic	BAE40354.	mmu:214254	
K00600	glyA, SHM	mmu01240(	PF00464.25	GO:003017	cytoplasmic	NP_001412	mmu:20425	
K04681	RBL1	mmu05165(	PF01858.22	GO:000367	cytoplasmic	NP_035379	mmu:19650	
K01054	MGLL	mmu04714(	PF12146.15(Hydrolase_		cytoplasmic	NP_001397	mmu:23945	
K24273	ZRSR		PF00642.30	GO:004423	nuclear	Q64707.1(R	mmu:22184	
K26098	SPDL1			GO:000810	cytoplasmic	NP_081687	mmu:70385	
K01074	PPT	mmu04142(	PF02089.21	GO:001678	lysosomal	NP_001289	mmu:54397	
K15838	GPSM3, AC	mmu04621(	PF02188.22	GO:001602	cytoplasmic	AAB82005.	mmu:106512	
K06560	MRC, CD2(	mmu04145(	PF00059.27	GO:000960	lysosomal	NP_032651	mmu:17533	
K14945	QKI		PF16544.10	GO:003577	nuclear	NP_001003	mmu:19317	
K10666	RNF5_185	mmu04141(	PF13920.12	GO:000578	cytoplasmic	NP_001102	mmu:54197	
K06480	ITGA1, CD	mmu05165(	PF00092.34	GO:004858	plasma mer	NP_001028	mmu:109700	
K13402	MTHFD1L	mmu01240(	PF01268.25	GO:003250	mitochondr	NP_001164	mmu:270685	
K23038	ZC3H14		PF14608.12	GO:000573	cytoplasmic	NP_083610	mmu:75553	
.QHU(INTS3 and NABP interactin(			PF15925.10	GO:000931	nuclear	XP_021016	mmu:66209	
K18932	ZDHHC		PF01529.25	GO:000810	ER	NP_080923	mmu:68268	
K02259	COX15, cta	mmu01240(	PF02628.20	GO:001649	mitochondr	NP_659123	mmu:226139	
K12892	SRSF3, SFR	mmu05014(	PF00076.28	GO:001593	cytoplasmic	XP_003220	mmu:20383	
K13963	SERPINB	mmu05146(	PF00079.25	GO:000486	cytoplasmic	BAE39626.	mmu:20706	
K15184	AFF1	mmu05202(	PF05110.18	GO:000802	nuclear	NP_598680	mmu:17355	AF-4
K21440	ANKRD50		PF12796.13	GO:001619	cytoplasmic	NP_001360	mmu:99696	
K03351	APC4, ANA	mmu04110(	PF23405.1(	GO:003304	cytoplasmic	NP_077175	mmu:52206	
K13988	NUDT9	mmu00230(	PF00293.34	GO:000918	mitochondr	NP_083070	mmu:74167	
K23010	OMA1	mmu05017(	PF01435.24	GO:001922	mitochondr	NP_001406	mmu:67013	
K23664	MEGF8	mmu04340(	PF24981.1(I	GO:000858	plasma mer	NP_001153	mmu:269878	
)WYV(Cdk5 and Abl enzyme substr(			PF00134.28	GO:005172	cytoplasmic	NP_665850	mmu:252966	
K13210	FUBP		PF00013.35	GO:008009	cytoplasmic	NP_001342	mmu:51886	
.MBP(nucleic acid binding protein)			PF21473.3(	GO:000962	cytoplasmic	NP_001345	mmu:69917	
ıucleic acid binding protein)			PF00888.28	GO:004212	cytoplasmic	NP_001165	mmu:78832	
K10738	MCM9		PF00493.29	GO:000810	nuclear	XP_006512	mmu:71567	
K00637	SOAT	mmu00100(	PF03062.24	GO:008009	cytoplasmic	ABI81497.1	mmu:20652	
K24414	FHL3	mmu04820(	PF00412.28	GO:003243	cytoplasmic	NP_001405	mmu:14201	
K15686	MEX3, RKHD		PF00013.35	GO:003646	cytoplasmic	NP_780575	mmu:108797	
K12875	ACIN1, AC	mmu03013(	PF16294.10	GO:008009	nuclear	NP_001078	mmu:56215	
K15193	SPTY2D1, SPT2		PF08243.16	GO:003472	nuclear	NP_780527	mmu:101685	
K20805	RNF169			GO:008009	nuclear	NP_780597	mmu:108937	
K10758	QSOX		PF18108.7(	GO:000989	ER	NP_001020	mmu:104009	
Y9XB(Transmembrane protein 168)				GO:000557	plasma mer	EDL13910.	mmu:101118	

K01876	DARS2, asp	mmu00970(	PF00152.25	GO:001602	mitochondr	NP_766232	mmu:226539	
K02974	RP-S24e, Rl	mmu05171(	PF01282.24	GO:004423	cytoplasmic	NP_001347	mmu:20088	
K20304	TRAPPC6, TRS33		PF04051.21	GO:004820	cytoplasmic	NP_080236	mmu:67091	
K00901	dgkA, DGK	mmu05231(	PF00609.24	GO:000665	cytoplasmic	NP_001345	mmu:13139	
K19553	TGIF2	mmu04350(	PF05920.17	GO:000548	nuclear	NP_775572	mmu:22883	Homeobox
K17608	STRN1_3_4		PF00400.38	GO:001989	cytoplasmic	NP_598550	mmu:97387	
K00274	MAO, aofH	mmu00982(	PF01593.30	GO:001961	cytoplasmic	NP_776101	mmu:17161	
K02910	RP-L31e, R	mmu05171(	PF01198.24	GO:002262	cytoplasmic	EDL14557.	mmu:114641	
K16302	CNNM		PF00571.33	GO:005080	extracellula	NP_444416	mmu:94218	
K06511	CD84		PF07686.23	GO:008009	plasma mer	NP_038517	mmu:12523	
K17908	WIPI1_2, A	mmu04136(	PF21032.3(I	GO:000810	cytoplasmic	NP_666052	mmu:52639	
K16803	CKAP5		PF21041.3(C	GO:003111	cytoplasmic	NP_083713	mmu:75786	
MSS(selenium binding)			PF08806.17	GO:003509	extracellula	NP_444332	mmu:93684	
K04703	SPRED		PF05210.18	GO:005124	mitochondr	NP_891557	mmu:101809	
K00415	QCR2, UQ(	mmu05208(	PF00675.25	GO:000417	mitochondr	NP_080175	mmu:67003	
K00326	CYB5R	mmu00520(	PF00970.30	GO:190136	Golgi appa	NP_082333	mmu:72017	
K23713	STAC3		PF07653.23	GO:000548	cytoplasmic	NP_001346	mmu:237611	
K19023	AP5M1, MUDENG		PF00928.26	GO:000577	mitochondr	NP_001346	mmu:74385	
K17986	FUNDC1	mmu04137(	PF04930.20	GO:000166	cytoplasmic	NP_001020	mmu:72018	
K10862	TDP1	mmu03410(	PF06087.18	GO:004257	nuclear	NP_082630	mmu:104884	
K11653	ARID1	mmu04714(	PF12031.14	GO:003506	nuclear	NP_001078	mmu:23998	ARID
K23961	EPB41L1_2_3		PF05902.18	GO:000585	nuclear	NP_001345	mmu:13822	
K04728	ATM, TEL1	mmu03440(	PF02259.29	GO:000016	cytoplasmic	NP_031525	mmu:11920	
K12199	VTA1, LIP5	mmu04144(	PF04652.21	GO:003251	cytoplasmic	NP_001392	mmu:125179616	
KT8W(Additional sex combs like 1 (			PF05761.20(5_nucleotic		mitochondr	NP_001361	mmu:70021	
K10421	CLIP1, RSN	mmu04150(mTOR signaling pathw			cytoplasmic	XP_006530	mmu:56430	
K20827	RPAP2		PF04181.18	GO:000582	cytoplasmic	NP_659160	mmu:231571	
K00863	DAK, TKF	mmu00561(	PF02733.22	GO:001684	cytoplasmic	XP_006527	mmu:225913	
K08838	STK24_25_MST4		PF00069.31	GO:004858	cytoplasmic	NP_663440	mmu:223255	
K15153	MED31, SOH1		PF05669.17	GO:008009	nuclear	NP_080344	mmu:67279	
K11293	HIRA, HIR1		PF07569.16	GO:003472	nuclear	NP_034565	mmu:15260	
K12898	HNRNPF_H		PF00076.28	GO:008009	nuclear	NP_001334	mmu:432467	
K21031	ISPD	mmu00515(	PF18706.7(I	GO:003003	cytoplasmic	NP_001276	mmu:75847	
KRIA(Chromosome 9 open reading f			PF10343.14	GO:001607	cytoplasmic	NP_081611	mmu:70153	
UFU(Transmembrane protein 65)			PF10507.14	GO:005123	mitochondr	NP_780421	mmu:74868	
K20011	PRDX3		PF00578.27	GO:004352	mitochondr	NP_031478	mmu:11757	
K03960	NDUFB4	mmu05208(	PF07225.17	GO:000574	cytoplasmic	AAH96421.	mmu:100042503	
K07759	PARG	mmu03410(	PF05028.20	GO:000548	mitochondr	NP_036090	mmu:26430	
K00507	SCD, desC	mmu01100(Metabolic p		GO:000663	ER	NP_033154	mmu:20250	
K15137	MED19		PF10278.14	GO:006026	nuclear	NP_080161	mmu:381379	
FYV(Synaptophysin-like)				GO:004247	plasma mer	XP_011242	mmu:19027	
K18674	GALK2	mmu00520(	PF10509.15	GO:001920	cytoplasmic	NP_001277	mmu:69976	
ZDM(Synaptophysin-like 2)			PF01284.28	GO:001972	lysosomal	NP_032622	mmu:17306	

K18166	FOXRED1		PF01266.30	GO:002260'	mitochondr	NP_001278	mmu:235169	
K07366	GRAP2, G/	mmu04660(	PF00018.34	GO:002305	cytoplasmic	NP_001276	mmu:17444	
K06702	CD93		PF12662.13	GO:000989'	plasma mer	NP_034870	mmu:17064	
K00799	GST, gst	mmu00980(	PF00043.30	GO:000573'	cytoplasmic	NP_001366	mmu:14867	
K11455	CBX8, PC3	mmu03083(	PF00385.30	GO:003506	nuclear	NP_038954	mmu:30951	
J7C(zinc finger, C2HC-type contain			PF13913.12	GO:004316'	nuclear	NP_775273	mmu:67306	
K04010	C5AR1, CD	mmu05171(	PF00001.26	GO:001922	plasma mer	NP_001167	mmu:12273	
K06553	VPREB, CD179a		PF07686.23	GO:000237	cytoplasmic	EDK98908.	mmu:22364	
K06553	VPREB, CD179a		PF07686.23(V-set:Immu		cytoplasmic	CAA80094.	mmu:22364	
K06553	VPREB, CD179a		PF07686.23(V-set:Immu		cytoplasmic	AGN91456.	mmu:22363	
K05401	TLR3, CD2	mmu05165(	PF13855.12	GO:004340	plasma mer	NP_001344	mmu:142980	
K07305	msrB		PF01641.26	GO:002260'	cytoplasmic	AAI41147.1	mmu:27361	
K10483	BTBD11		PF00651.37	GO:009953'	plasma mer	NP_082985	mmu:74007	ZBTB
K04864	CACNB3	mmu04010(	PF00625.27	GO:000526	cytoplasmic	NP_001038	mmu:12297	
K09228	KRAB		PF13465.12	GO:000097'	cytoplasmic	NP_001388	mmu:10041	zf-C2H2
K13148	CPSF3L, INTS11		PF21386.2(I	GO:009030	cytoplasmic	NP_082296	mmu:71957	
K06662	HRAD17, RAD24		PF03215.21	GO:000810	cytoplasmic	NP_001037	mmu:19356	
Y802(-)					peroxisoma	NP_598868	mmu:106264	
XQMI(transmembrane protein 39A)			PF10271.14	GO:000557	extracellulo	XP_023363	mmu:230770	
K02377	TSTA3, fcl	mmu00520(	PF01370.27	GO:001967	cytoplasmic	NP_001398	mmu:22122	
K09568	FKBP1		PF00254.34	GO:009008'	cytoplasmic	NP_058559	mmu:14226	
K14833	NOC2		PF03715.18	GO:200123	nuclear	XP_030109	mmu:57741	
K10129	GTSE1, B9	mmu04115(	PF15259.11	GO:000585	cytoplasmic	EDL04419.	mmu:29870	
K08997	SELENOO, selO		PF02696.19	GO:003563	mitochondr	NP_082181	mmu:223776	
K03122	TFIIA1, GT	mmu05203(	PF03153.18	GO:008009	nuclear	AAH79909.	mmu:83602	
XNUZ(Cerebellar degeneration-related protein)				GO:000367	cytoplasmic	NP_001074	mmu:237988	
K17405	MRPS26		PF14943.11	GO:001593	mitochondr	NP_997090	mmu:99045	
K01539	ATP1A	mmu04971(	PF00122.25	GO:005507	cytoplasmic	NP_038762	mmu:27222	
K04416	MAP3K1, M	mmu04530(	PF00069.31	GO:001922	cytoplasmic	NP_036075	mmu:26401	
K04506	SIAH1	mmu04310(	PF03145.22	GO:000716	mitochondr	NP_001295	mmu:20438	
K17914	KIF13	mmu04814(	PF00225.29	GO:000810	cytoplasmic	NP_034747	mmu:16553	
K01874	MARS, met	mmu00970(	PF09334.17	GO:000643	mitochondr	BAC31393.	mmu:212679	
XQ07(zinc finger, C3H1-type contai			PF13428.12	GO:004423	nuclear	NP_001028	mmu:216345	
K16669	FAT4	mmu04392(	PF00028.23	GO:004856	plasma mer	NP_899044	mmu:329628	
K10419	DYNLRB, I	mmu05132(	PF03259.23	GO:000763	cytoplasmic	NP_001029	mmu:67068	
K06485	ITGA6, CD	mmu05165(	PF20806.3(I	GO:005084	ER	XP_060246	mmu:16403	
K14208	XPNPEP2	mmu04974(	PF16189.11	GO:000417'	extracellulo	NP_573476	mmu:170745	
K13618	PLA1A	mmu00564(	PF00151.25	GO:000663	extracellulo	NP_598863	mmu:85031	
K22380	TMEM129		PF10272.14	GO:000578	cytoplasmic	XP_032772	mmu:68366	
K13338	PEX1	mmu04146(	PF00004.35	GO:000810	cytoplasmic	NP_001280	mmu:71382	
XUSH(phytanoyl-CoA dioxygenase)			PF05721.19	GO:004316'	cytoplasmic	NP_758471	mmu:227696	
K20362	YIF1		PF03878.20	GO:000810	cytoplasmic	XP_021007	mmu:68090	
K01435	BTD	mmu00780(	PF19018.5(	GO:001975	lysosomal	NP_079571	mmu:26363	

K06082	FARP2, FR	mmu04520(	PF00621.25	GO:002260'	cytoplasmic	NP_663494	mmu:227377
K15176	CTR9		PF00515.34	GO:000802	nuclear	AAH24749.	mmu:22083
K01135	ARSB	mmu04142(	PF00884.28	GO:006032	lysosomal	NP_033842	mmu:11881
K07305	msrB		PF01641.26	GO:000578	mitochondr	NP_796066	mmu:320183
K16733	RACGAP1, Tum		PF00620.32	GO:000716	cytoplasmic	EDL04119.	mmu:26934
K22030	LMOD	mmu04820(	PF03250.19	GO:009951	cytoplasmic	NP_444328	mmu:93677
XSIZ(Interferon-related developmen			PF05004.19	GO:004867'	cytoplasmic	NP_038590	mmu:15982
ranslin)			PF01997.21	GO:000989	cytoplasmic	NP_058605	mmu:53424
K17267	COPG		PF01602.26	GO:000810	cytoplasmic	NP_059505	mmu:54161
K01800	maiA, GST	mmu00350(	PF02798.26	GO:001975	cytoplasmic	NP_001351	mmu:14874
K25818	NOLC1		PF05022.17	GO:003315'	cytoplasmic	NP_001034	mmu:70769
K00006	GPD1	mmu00564(	PF01210.29	GO:003288	cytoplasmic	NP_780589	mmu:333433
K07427	CYP4V		PF00067.27	GO:003444	ER	NP_598730	mmu:102294
K26267	SAP30BP		PF07818.18	GO:008009	nuclear	NP_065229	mmu:57230
K02877	RP-L15e, R	mmu05171(	PF00827.22	GO:002262	cytoplasmic	BAE31437.	mmu:66480
K20404	DEPDC5, S	mmu04150(	PF12257.13	GO:000989	cytoplasmic	NP_808454	mmu:277854
K15516	FMR		PF17904.7(I	GO:003243	nuclear	NP_001277	mmu:14265
K09507	DNAJB1	mmu04141(	PF01556.23	GO:009008	cytoplasmic	NP_061278	mmu:81489
K03424	tatD		PF01026.27	GO:001678'	nuclear	NP_001334	mmu:381801
K01128	SMPDL3		PF19272.4(	GO:004643	lysosomal	NP_598649	mmu:100340
K10653	TRIM54, M	mmu04820(	PF13445.12	GO:003111	cytoplasmic	XP_029332	mmu:58522
ıbiquitin)			PF10421.14	GO:000582	cytoplasmic	NP_660135	mmu:243262
K24991	MTHFSD		PF01812.26	GO:000573'	cytoplasmic	NP_001159	mmu:234814
K14827	IPI1, TEX1	mmu03083(	PF12333.14	GO:199023	nuclear	NP_001342	mmu:269536
K17759	NAXE, nnrE		PF03853.21	GO:005124	mitochondr	NP_659146	mmu:246703
K02918	RP-L35e, R	mmu05171(	PF00831.28	GO:001593	cytoplasmic	NP_079868	mmu:66489
K02649	PIK3R1_2_	mmu05208(	PF16454.10	GO:003288	cytoplasmic	SPT35779.1	mmu:18709
K07905	RAB11B	mmu04962(	PF00071.28	GO:003288	cytoplasmic	NP_001030	mmu:19326
K12825	SF3A1, SAF	mmu03040(	PF12230.13	GO:002260'	nuclear	NP_080451	mmu:67465
K08865	PBK		PF00069.31	GO:001922	cytoplasmic	NP_001404	mmu:52033
K13024	PPIP5K, VI	mmu04070(	PF00328.27	GO:000548	cytoplasmic	NP_001394	mmu:327655
K21110	CGNL1	mmu04530(	PF01576.24	GO:000592	cytoplasmic	AAI58062.1	mmu:68178
K07252	DOLPP1	mmu00510(	PF01569.27	GO:004239	Golgi appa	NP_001277	mmu:57170
K10429	MAP1	mmu03250(	PF23415.1(	GO:003111	cytoplasmic	NP_115769	mmu:17754
K09448	TEAD	mmu04390(	PF17725.7(	GO:008009	nuclear	AAS19414.1	mmu:21676 TEA
K16581	TPGS1		PF24480.1(	GO:002260'	cytoplasmic	NP_683736	mmu:110012
K12872	RBM22, SL'	mmu03040(	PF21369.3(	GO:003315'	cytoplasmic	NP_001244	mmu:66810
AB8(Chromosome 4 open reading f			PF15725.10	GO:000557	cytoplasmic	NP_083758	mmu:75939
K13347	PXMP2, PM	mmu04146(	Peroxisome	GO:000573'	extracellulo	EDL20029.	mmu:19301
K19347	SUN1_2	mmu04820(	PF09387.16	GO:010609	cytoplasmic	NP_077771	mmu:77053
K25592	LSM11		PF01423.28	GO:190227	nuclear	NP_082461	mmu:72290
K24721	CLINT1, EPN4		PF01417.26	GO:002260'	cytoplasmic	NP_001333	mmu:216705
K20301	TRAPPC2, TRS20		PF04628.19	GO:004820	cytoplasmic	NP_067477	mmu:59005

K00490	CYP4F		PF00067.27	GO:000449	ER	BAE26998.	mmu:677156	
K19946	OPTN, FIP	mmu04140(	PF16516.10	GO:000810	cytoplasmic	EDL07961.	mmu:71648	
K24835	GLIPR2		PF00188.32	GO:004858	cytoplasmic	NP_081726	mmu:384009	
KQGP(uhrf1 binding protein)			PF24917.1(I	GO:000548	cytoplasmic	NP_001074	mmu:224648	
K23720	UVSSA	mmu03420(	PF20867.3(I	GO:000962	cytoplasmic	NP_001074	mmu:71101	
K09228	KRAB		PF00096.32	GO:000582	cytoplasmic	NP_001334	mmu:69228	zf-C2H2
K10786	PRG2, MBI	mmu05310(	PF00059.27	GO:000961	extracellulo	NP_032946	mmu:19074	
K13185	DHX30		PF24995.1(I	GO:002260	nuclear	NP_579925	mmu:72831	
K03013	RPABC1, R	mmu03420(	PF03871.20	GO:003164	nuclear	NP_001103	mmu:66420	
K03685	rnc, DROSI	mmu03008(	PF00636.31	GO:190309	nuclear	NP_001123	mmu:14000	
K13512	LPCAT4, A	mmu00564(	PF01553.26	GO:000657	ER	NP_997089	mmu:99010	
K02541	MCM3	mmu03030(	PF00493.29	GO:190231	nuclear	NP_032589	mmu:17215	
K10274	FBXL8		PF12937.12	GO:000815	cytoplasmic	NP_056636	mmu:50788	
K14538	NUG1, GN	mmu03008(	PF01926.29	GO:008009	nuclear	NP_001162	mmu:237107	
K19612	PDE12		PF21171.3(I	GO:008009	mitochondr	NP_848783	mmu:211948	
K23539	UNC119		PF05351.17	GO:002260	cytoplasmic	NP_780561	mmu:106840	
K20653	GMIP, ARHGAP46		PF22699.1((	GO:000548	cytoplasmic	EDL28753.	mmu:78816	
K01527	EGD1, BTF3		PF01849.23(NAC:NAC		cytoplasmic	NP_001125	mmu:70533	
K10447	KLHL9_13	mmu04120(	PF01344.31	GO:199023	cytoplasmic	BAC98148.	mmu:24252	ZBTB
K24915	TMEM175		PF06736.16	GO:000526	Golgi appa	NP_001157	mmu:72392	
K23565	EMC4, TMEM85		PF06417.17	GO:000810	mitochondr	NP_001099	mmu:68032	
K20735	ALOX5AP,	mmu04664(	PF01124.23	GO:000578	ER	NP_033793	mmu:11690	
K12607	CNOT10	mmu03018(	PF00515.34	GO:000989	cytoplasmic	NP_705813	mmu:78893	
K07818	PRF1	mmu04940(	PF01823.24	GO:000810	plasma mer	NP_035203	mmu:18646	
K07739	ELP3, KAT9		PF23613.1(I	GO:008009	cytoplasmic	NP_083087	mmu:74195	
K16719	KANSL3, RCD1		PF23154.1(I	GO:003124	cytoplasmic	NP_001297	mmu:226976	
K11135	PINX1		PF01585.28	GO:000810	nuclear	NP_082504	mmu:72400	
K17943	PUM	mmu05017(	PF00806.25	GO:003577	cytoplasmic	NP_001153	mmu:80913	
K19730	ATG101	mmu04136(	PF07855.17	GO:000989	cytoplasmic	NP_001405	mmu:68118	
K10313	FBXO38, MOKA			GO:000283	nuclear	NP_598897	mmu:107035	
K27318	MTARC		PF03476.21	GO:003017	mitochondr	NP_598445	mmu:67247	
K17897	GATA6		PF05349.17	GO:001602	nuclear	NP_034388	mmu:14465	zf-GATA
K22028	MYPN	mmu04820(	PF07679.22	GO:009863	nuclear	NP_892037	mmu:68802	
K26797	GORASP, (	mmu04140(	PF04495.20	GO:004323	cytoplasmic	EDL09197.	mmu:74498	
K02147	ATPeV1B,	mmu05165(	PF00006.31	GO:001922	cytoplasmic	NP_598918	mmu:110935	
KQRV(family with sequence similar			PF04916.18	GO:000032	extracellulo	NP_076114	mmu:71772	
K10143	RFWD2, C(	mmu04115(	PF00400.38	GO:000989	cytoplasmic	AAI58003.1	mmu:26374	
K04424	ZAK, MLT	mmu04010(	PF07714.23	GO:004858	cytoplasmic	NP_001158	mmu:65964	
K01745	hutH, HAL	mmu00340(	PF00221.24	GO:000439	cytoplasmic	NP_034531	mmu:15109	
K06058	DTX	mmu04330(	PF18102.6(I	GO:000548	cytoplasmic	NP_766030	mmu:207521	
K01076	ABHD17		PF12146.15	GO:009859	lysosomal	CAH67792	mmu:216169	
K16596	CCL8	mmu04062(	PF00048.25	GO:001922	extracellulo	NP_067418	mmu:20307	
K06547	ALCAM, CI	mmu04514(	PF13927.12	GO:004867	plasma mer	NP_001318	mmu:11658	

.IY3(chromosome X open reading fr			PF15112.11(DUF4559:I		cytoplasmic	NP_780350	mmu:69155	
ZTV7(chromosome 2 open reading f			PF10230.15	GO:000578	cytoplasmic	NP_001351	mmu:68832	
K06316	RFT1		PF04506.18	GO:000686	extracellulo	AAI45607.1	mmu:328370	
.8C1(Nucleolar protein 11)			PF08168.16	GO:008009	cytoplasmic	NP_598463	mmu:68979	
K15026	EIF2A		PF08662.17	GO:008009	cytoplasmic	NP_001005	mmu:229317	
K10882	EME1, MM	mmu03440(	PF21292.3(I	GO:000716	nuclear	NP_808420	mmu:268465	
K12198	CHMP5, VI	mmu04217(	PF03357.26	GO:000699	nuclear	NP_001020	mmu:76959	
K06512	LILR, CD8	mmu04380(	PF13895.12	GO:003239	plasma mer	AAC53214.	mmu:18726	
K17603	ZFYVE1	mmu04140(	PF01363.27	GO:000573	cytoplasmic	BAE24148.	mmu:217695	
Z6JA(Family with sequence similari			PF14981.11	GO:000557	extracellulo	NP_620082	mmu:68936	
K04600	CELSR1		PF00028.23	GO:004224	plasma mer	NP_034016	mmu:12614	
K14409	SMG7, EST	mmu03015(	PF10373.15	GO:000018	cytoplasmic	NP_001153	mmu:226517	
XRQN(Multiple C2 and transmemb			PF00168.36	GO:005104	cytoplasmic	NP_001019	mmu:244049	
K04496	CTBP	mmu05200(	PF02826.25	GO:008009	nuclear	NP_001399	mmu:13017	
K09598	SPPL3		PF04258.18	GO:000548	cytoplasmic	AAH86646.	mmu:74585	
K17599	TMEM132		PF23039.1(	GO:000548	ER	EDL41385.	mmu:98170	
K25509	CRYBG		PF00030.24	GO:003250	cytoplasmic	NP_001355	mmu:11630	
K20636	ARHGAP12_27		PF00620.32	GO:000716	cytoplasmic	NP_001192	mmu:544817	
K05969	PTRHD1		PF01981.21	GO:001678	mitochondr	NP_001191	mmu:69709	
K09527	DNAJC7		PF00515.34	GO:000645	cytoplasmic	NP_001390	mmu:56354	
K09508	DNAJB2	mmu04141(	PF00226.36	GO:009008	cytoplasmic	NP_001153	mmu:56812	
.Q2V(Thioredoxin domain containi			PF06110.17	GO:001922	cytoplasmic	NP_080835	mmu:52700	
K09515	DNAJB9		PF00226.36	GO:000578	extracellulo	NP_038788	mmu:27362	
K10093	LGALS9		PF00337.29	GO:004213	cytoplasmic	NP_034838	mmu:16859	
K11494	RCBTB		PF00415.24	GO:000573	cytoplasmic	NP_001164	mmu:10567	ZBTB
K19478	MAGT1, T	mmu04141(	PF04756.19	GO:199023	ER	NP_084530	mmu:80286	
K12470	SH3KBP1,	mmu04144(	Endocytosis	GO:000810	cytoplasmic	XP_006528	mmu:58194	
K14398	CPSF6_7	mmu03250(	PF00076.28	GO:000548	cytoplasmic	NP_758506	mmu:269061	
K00275	pdxH, PNP	mmu01240(	PF01243.27	GO:007027	mitochondr	NP_598782	mmu:103711	
K07610	DES	mmu05410(	PF00038.26	GO:000520	cytoplasmic	NP_034173	mmu:13346	
K02833	HRAS	mmu05208(	PF00071.28	GO:004340	cytoplasmic	NP_001091	mmu:15461	
K25065	HDGFL2		PF11467.13	GO:003250	nuclear	NP_032259	mmu:15193	
K26421	FHIP2, FAM160B		PF10257.15(RAI16-like:		cytoplasmic	NP_919326	mmu:239170	
K20773	RBBP8, CT	mmu03440(	PF10482.14	GO:000008	nuclear	NP_001074	mmu:225182	
K25773	CLSPN			GO:000548	nuclear	NP_780763	mmu:269582	
K22381	ZNF598		PF23202.1(I	GO:002262	nuclear	NP_898972	mmu:213753	
K06733	SLAMF7, CD319			GO:000989	extracellulo	NP_001334	mmu:75345	
2BYV(peptidoglycan-binding, domain containin				GO:004322	cytoplasmic	NP_084533	mmu:80289	
K01358	clpP, CLPP		PF00574.28	GO:000417	mitochondr	NP_059089	mmu:53895	
K20279	SYNJ	mmu04070(	PF22669.2(I	GO:005104	nuclear	NP_001157	mmu:104015	
K03991	MBL	mmu04610(	PF00059.27	GO:005104	extracellulo	NP_034905	mmu:17194	
K01968	MCCC1, ac	mmu00280(	PF02786.22	GO:001602	mitochondr	NP_076133	mmu:72039	
K00030	IDH3	mmu01100(	PF00180.25	GO:001967	mitochondr	NP_032349	mmu:15929	

K25366	MESD, BOCA		PF10185.14	GO:000548	ER	NP_075892	mmu:67943	
K24494	MYBPH	mmu04820(	PF07679.22	GO:000699	cytoplasmic	NP_001344	mmu:53311	
K18730	GIGYF		PF02213.21	GO:006500	nuclear	NP_001390	mmu:57330	
XQ0V(Elongator complex protein)			PF21896.2(	GO:003243	cytoplasmic	NP_001013	mmu:80889	
K14429	SLC12A9, CCC6		PF00324.27	GO:005080	plasma mer	NP_001346	mmu:83704	
K03904	FGB	mmu04610(	PF00147.23	GO:200123	ER	NP_862897	mmu:110135	
K27103	SRRT, ARS2		PF04959.19	GO:001921	nuclear	NP_113582	mmu:83701	
K05659	ABCB4	mmu02010(	PF00664.28	GO:014032	cytoplasmic	NP_032856	mmu:18670	
K05658	ABCB1, CD	mmu02010(	PF00664.28	GO:000810	ER	NP_035206	mmu:18671	
K01321	F9	mmu04610(	PF00089.32	GO:000425	extracellula	NP_032005	mmu:14071	
K17989	SDS, SDH,	mmu00290(	PF00291.31	GO:000548	cytoplasmic	NP_001371	mmu:257635	
K06827	CTGF, CCN	mmu04390(	PF19035.6(	GO:004423	extracellula	NP_034347	mmu:14219	
K10507	ZBTB33, KAISO		PF00651.37	GO:000832	nuclear	AAH25136.	mmu:56805	ZBTB
K15263	LYER		PF08790.17	GO:008009	nuclear	NP_001411	mmu:17089	TF_others
K17776	MTX	mmu04137(	PF10568.15	GO:000810	cytoplasmic	NP_058084	mmu:53375	
K23409	AHSG, FETUA		PF00031.27	GO:003041	extracellula	NP_038493	mmu:11625	
K07870	RHOT1, AI	mmu04137(	PF08356.18	GO:005080	cytoplasmic	EDL15631.	mmu:59040	
K01044	CES1	mmu00983(	PF00135.34	GO:000578	extracellula	EDL11096.	mmu:104158	
Can catalyze the hydrolysis of ATP i			PF00970.30	GO:001602	cytoplasmic	BAB27227.1	mmu:109754	
K01260	RNPEP		PF01433.26	GO:000417	cytoplasmic	NP_663392	mmu:215615	
K03944	NDUFV3	mmu05208(	PF15880.10	GO:000998	cytoplasmic	NP_084363	mmu:78330	
K24314	LPXN		PF00412.28	GO:000591	cytoplasmic	AAH26563.	mmu:107321	
K11147	DHRS4	mmu00830(	PF13561.12	GO:000403	peroxisoma	BAB18776.1	mmu:28200	
K17776	MTX	mmu04137(	PF10568.15	GO:000810	mitochondr	XP_021013	mmu:17827	
K08730	PTDSS2	mmu00564(	PF03034.20	GO:000665	plasma mer	NP_038810	mmu:27388	
XQIA(Apoptotic chromatin conden			PF05147.19	GO:000563	nuclear	NP_598498	mmu:71835	
YCF2(AVL9 homolog (S. cerevisiase)			PF09794.14	GO:000573	cytoplasmic	NP_084511	mmu:78937	
K17943	PUM	mmu05017(	PF00806.25	GO:003577	cytoplasmic	NP_001153	mmu:80913	
K11429	SUV420H	mmu00310(	PF00856.33	GO:008009	cytoplasmic	XP_040606	mmu:225888	
K10949	KDELR		PF00810.23	GO:003303	ER	NP_598851	mmu:105785	
SHQ1 homolog (S. cerevisiae))			PF24509.1(	GO:004323	extracellula	NP_001297	mmu:70561	
K12490	ARAP3	mmu04024(	PF00620.32	GO:001602	cytoplasmic	AAH68145.	mmu:106952	
K20967	MOCS1	mmu01240(	PF01967.26	GO:000677	mitochondr	NP_064426	mmu:56738	
K07817	PTPRN	mmu04940(	PF00102.32	GO:000810	plasma mer	NP_033011	mmu:19275	
K06530	CD109		PF07678.20	GO:001922	extracellula	NP_694738	mmu:235505	
K11349	BRD1, BRPF2		PF10513.14	GO:008009	nuclear	XP_006520	mmu:223770	
K26403	CIAO2, CIA2, FAM96		PF01883.24	GO:002260	cytoplasmic	NP_081029	mmu:68523	
K01148	PARN, PN	mmu03018(	PF04857.26	GO:008009	nuclear	NP_083037	mmu:74108	
K08214	SLC22A18		PF07690.21	GO:009859	extracellula	NP_001036	mmu:18400	
K12804	PSTPIP1	mmu04621(	PF00611.28	GO:003243	cytoplasmic	NP_035323	mmu:19200	
K16315	GSG2		PF12330.14	GO:000810	nuclear	NP_034483	mmu:14841	
K11538	ACAD8	mmu00280(	PF00441.29	GO:003444	mitochondr	NP_080138	mmu:66948	
XNSK(oligomeric mucus gel-formin			PF00094.30	GO:000557	extracellula	NP_001116	mmu:215384	

K08991	MUS81	mmu03440(	PF21136.3(	GO:000716	mitochondr	NP_082153	mmu:71711	
K05702	AF6, MLLT	mmu04530(	PF00788.29	GO:000810	nuclear	XP_006523	mmu:17356	
K07520	ABLIM	mmu04360(	PF16182.10	GO:003243	cytoplasmic	XP_006527	mmu:226251	
K07188	LIPE, HSL	mmu04910(	PF06350.17	GO:004213	cytoplasmic	XP_006539	mmu:16890	
K17564	CHCHD6		PF05300.16	GO:006102	cytoplasmic	NP_001161	mmu:66098	
K05750	NCKAP1, N	mmu04810(	PF09735.14	GO:000270	cytoplasmic	NP_705725	mmu:105855	
K24465	EGFL7_8		PF07546.18	GO:000548	extracellulo	XP_048669	mmu:353156	
K05103	EPHA2, EC	mmu04010(	PF07714.23	GO:004864	plasma mer	NP_034269	mmu:13836	
K03842	ALG1	mmu00510(	PF13692.12	GO:000578	ER	NP_663337	mmu:208211	
K06241	LAMA4	mmu05165(	PF02210.31	GO:005124	plasma mer	NP_034811	mmu:16775	
K11593	ELF2C, AGO		PF02171.23	GO:003577	cytoplasmic	NP_001395	mmu:239528	
K12565	SGCA	mmu05410(	PF05510.18	GO:001601	extracellulo	NP_033187	mmu:20391	
K15326	TSEN54		PF12928.12	GO:009030	nuclear	EDL34523.	mmu:76265	
K04633	GNAL	mmu05146(	PF00503.26	GO:000716	cytoplasmic	BAE34685.	mmu:14680	
K27395	PRX		PF02389.20	GO:003228	mitochondr	BAD32498.	mmu:19153	
K07839	RAP2C	mmu04530(	PF00071.28	GO:001922	cytoplasmic	NP_001069	mmu:72065	
K08828	ICK		PF00069.31	GO:000716	cytoplasmic	BAE32425.	mmu:56542	
KRZP(TNFAIP3 interacting protein			PF12180.13	GO:001922	cytoplasmic	NP_620703	mmu:231130	
K11408	HDAC7	mmu05203(	PF00850.24	GO:008009	cytoplasmic	NP_001191	mmu:56233	
K11599	POMP, UM	mmu03050(	PF05348.16	GO:002260	cytoplasmic	NP_079900	mmu:66537	
K11316	SUPT7L		PF07524.19	GO:003124	nuclear	EDL37370.	mmu:72195	
K01343	PLAT	mmu04610(	PF00089.32	GO:000425	plasma mer	NP_032898	mmu:18791	
K16609	TTLL12		PF03133.20	GO:003506	cytoplasmic	NP_898838	mmu:223723	
KR6C(Solute carrier family 4 (Anion			PF00498.31	GO:001920	nuclear	NP_001334	mmu:20534	
K15448	TRM112, TRMT112		PF03966.22	GO:000647	mitochondr	AAH87959.	mmu:67674	
K20242	EVI5		PF00566.25	GO:004322	nuclear	NP_031990	mmu:14020	
K16311	SIK2	mmu04922(	PF00069.31	GO:001922	cytoplasmic	NP_848825	mmu:235344	
K14696	SLC30A9, ZNT9		PF01545.26	GO:000578	mitochondr	AAI41009.1	mmu:109108	
K02158	BAD	mmu05208(	PF10514.14	GO:003577	cytoplasmic	BAE40801.	mmu:12015	
K11275	H1_5		PF00538.25	GO:003472	nuclear	NP_032223	mmu:14958	
K20314	ARFIP		PF06456.18	GO:005112	cytoplasmic	EDL16791.	mmu:76932	
I3F(isochorismatase)			PF00857.26	GO:000573	peroxisoma	NP_001014	mmu:66307	
K01080	PLPP1_2_3	mmu05231(	PF01569.27	GO:009859	plasma mer	AAH16635.	mmu:50784	
K11109	POT1		PF21375.2(	GO:008009	cytoplasmic	NP_082646	mmu:72836	
K20346	TMED4_9_11		PF01105.29	GO:000802	ER	BAE40800.	mmu:67511	
K19970	NT5E, CD7	mmu01100(	PF02872.23	GO:004643	extracellulo	NP_035981	mmu:23959	
K04731	IRAK2	mmu04722(	PF00069.31	GO:001922	cytoplasmic	NP_751893	mmu:108960	
K17906	ATG2	mmu04136(	PF13329.13	GO:001922	cytoplasmic	NP_001395	mmu:329015	
K20185	BLOC1S1		PF06320.18	GO:009951	cytoplasmic	CAH67784	mmu:14533	
K09192	SP2		PF13465.12	GO:005125	nuclear	BAE23947.	mmu:78912	zf-C2H2
KNS6(chromosome 6 open reading f			PF14961.12	GO:000810	cytoplasmic	NP_001028	mmu:544696	
K27309	TMEM59		PF12280.13	GO:000989	ER	CAH679061	mmu:56374	
K01330	C1R	mmu05171(	PF00431.26	GO:000425	extracellulo	AAH04637.	mmu:50909	

K16586	HAUS3, AUG3		PF14932.11	GO:002260'	cytoplasmic	NP_666271	mmu:231123	
K19374	DNAJC30		PF00226.36	GO:004323'	mitochondr	NP_079638	mmu:66114	
K24060	PDZD8		PF17820.7(I	GO:002260	ER	NP_001028	mmu:107368	
K18755	IPO8, RAN	mmu03013(	PF03810.25	GO:003450	cytoplasmic	NP_001074	mmu:320727	
K09478	ACADSB	mmu01100(	PF00441.29	GO:003444	mitochondr	AAH54428.	mmu:66885	
K12667	RPN2, SWF	mmu04141(	PF23860.1(I	GO:199023	ER	NP_001342	mmu:20014	
K18443	GBF1	mmu04144(	PF01369.25	GO:000581	cytoplasmic	NP_849261	mmu:107338	
K18466	VPS26A_B	mmu04144(	PF03643.20	GO:000810	cytoplasmic	NP_598433	mmu:30930	
K13172	SRRM2, SRM300		PF08312.17	GO:000372	nuclear	NP_001355	mmu:75956	
K07199	PRKAB	mmu04530(	PF16561.11	GO:000663	cytoplasmic	NP_892042	mmu:108097	
K03871	VHL	mmu05200(	PF01847.22	GO:004806	cytoplasmic	NP_033533	mmu:22346	
K15610	PBX3	mmu05202(	PF03792.18	GO:008009	nuclear	NP_001101	mmu:18516	Homeobox
K05039	SLC6A6, TAUT		PF00209.24	GO:003042	peroxisoma	NP_001397	mmu:21366	
ZP57(family with sequence similarit			PF20771.2(I	GO:000557	plasma mer	NP_954670	mmu:217219	
K17263	CAND1, TIP120A		PF08623.16	GO:006026	cytoplasmic	NP_082270	mmu:71902	
K00602	purH	mmu01523(	PF01808.24	GO:000916	cytoplasmic	NP_080471	mmu:108147	
K05635	LAMC1	mmu05165(	PF00053.31	GO:003444	extracellulo	NP_034813	mmu:226519	
ZSI(katanin p80 subunit B-like 1)			PF13925.11	GO:009859	nuclear	NP_077216	mmu:72425	
K09291	TPR, MLP1	mmu03013(	PF07926.17	GO:003315	cytoplasmic	AAI41407.1	mmu:108989	
K20350	TMED3		PF01105.29	GO:003802	ER	NP_079636	mmu:66111	
K08019	RAPGEF5	mmu04014(	PF00617.24	GO:000508	nuclear	NP_787126	mmu:217944	
K23717	SLC23A2, SVCT2		PF00860.26	GO:000301	cytoplasmic	NP_001342	mmu:54338	
K22865	TALPID3, JBTS23		PF15324.11	GO:002260'	cytoplasmic	EDL36552.	mmu:76967	
ZBH(Coiled-coil domain containin			PF08524.16	GO:004323	mitochondr	NP_079878	mmu:52713	
K26401	TOM1, TOM1L1_2		PF00790.25	GO:000810	cytoplasmic	EDL15891.	mmu:71943	
K08228	SLC43A1		PF07690.21	GO:009859	ER	EDL27307.	mmu:72401	
K22856	EEF1AKMT2, EFM4, M		PF13847.12	GO:000647	cytoplasmic	NP_082371	mmu:72096	
K07478	ycaJ		PF12002.13	GO:008009	nuclear	NP_084491	mmu:78903	
K00486	KMO	mmu01240(	PF01494.25	GO:000449	cytoplasmic	EDL13209.	mmu:98256	
prataxin and PNKP like factor)			PF01553.26	GO:004602	Golgi appa	NP_082373	mmu:72098	
K17199	RASEF, RAB45		PF00071.28	GO:003563	cytoplasmic	NP_001017	mmu:242505	
K09529	DNAJC9		PF23302.1(I	GO:003472	cytoplasmic	NP_598842	mmu:108671	
K01081	E3.1.3.5	mmu01100(	PF05761.20	GO:005068	mitochondr	NP_001157	mmu:76952	
K24036	DOK3		PF02174.23	GO:004858	cytoplasmic	NP_038767	mmu:27261	
K23575	IGFBP2		PF00086.23	GO:007066	extracellulo	NP_032368	mmu:16008	
K24630	ZMYND8		PF12064.13	GO:003288	cytoplasmic	NP_001239	mmu:228880	
K09506	DNAJA5		PF00226.36	GO:000573	nuclear	NP_084322	mmu:78244	
K27448	IRF2BP2		PF11261.13	GO:000989	cytoplasmic	NP_001158	mmu:270110	
K07369	MALT1	mmu04064(	PF18703.7(	GO:005116	mitochondr	NP_001351	mmu:240354	
K01883	CARS, cysS	mmu00970(	PF01406.26	GO:003563	nuclear	NP_038770	mmu:27267	
24DX(Programmed cell death 7)			PF16021.10	GO:200123	cytoplasmic	NP_057897	mmu:50996	
K12484	RAB11FIP1	mmu04144(	PF00168.36	GO:000810	cytoplasmic	NP_001003	mmu:52055	
K20684	ARHGEF5		PF15441.11(ARHGEF5		nuclear	NP_001404	mmu:54324	

K24869	SCAPER		PF16501.10	GO:004858	nuclear	BAD32453.	mmu:244891	
K25639	SUPT16H, SPT16		PF24824.1(I	GO:003472	nuclear	NP_291096	mmu:114741	
.F5P(asteroid homolog 1 (Drosophila))					cytoplasmic	NP_079927	mmu:66595	
K14793	RRP9		PF00400.38	GO:003204	nuclear	NP_663595	mmu:27966	
K22909	ZC3H13		PF00642.30	GO:199023	nuclear	NP_001346	mmu:67302	
K18283	PDE2A	mmu05032(	PF00233.25	GO:000921	cytoplasmic	EDL16514.	mmu:207728	
XY31(family with sequence similarit			PF15341.11	GO:003068	cytoplasmic	NP_598759	mmu:108707	
K18584	ACTR3, AR	mmu04530(	PF00022.25	GO:001989	cytoplasmic	BAE35272.	mmu:74117	
K01890	FARSB, ph	mmu00970(	PF17759.6(t	GO:000028	cytoplasmic	NP_001265	mmu:23874	
K06533	CDH5, CD	mmu05418(	PF00028.23	GO:003111	ER	NP_033998	mmu:12562	
K06803	CDH11		PF00028.23	GO:000591	extracellula	NP_001403	mmu:12552	
K15731	CTDSP		PF03031.24	GO:001922	cytoplasmic	NP_694728	mmu:227292	
XNU0(Minichromosome maintenan			PF09739.14	GO:009030	nuclear	NP_666067	mmu:210711	
K19988	EXOC3L2		PF06046.19(Sec6:Exocy		cytoplasmic	NP_001357	mmu:74463	
K05931	CARM1, PF	mmu01522(	PF11531.14	GO:000810	cytoplasmic	XP_021061	mmu:59035	
K14787	MRD1, RBM19		PF00076.28	GO:004599	nuclear	NP_083038	mmu:74111	
K22830	SMDT1, EMRE		PF10161.14	GO:199024	mitochondr	NP_081190	mmu:69029	
K02935	RP-L7, MR	mmu03010(	PF00542.24	GO:005125	mitochondr	NP_081480	mmu:56282	
K00816	CCBL	mmu00270(	PF00155.27	GO:000548	mitochondr	NP_001280	mmu:229905	
K11319	ING3	mmu03082(	PF12998.12	GO:003506	nuclear	NP_001297	mmu:71777	
K15712	TTC3		PF24525.1(	GO:006138	nuclear	NP_033467	mmu:22129	
K16487	SAS-6, SASS6		PF16531.10	GO:190199	cytoplasmic	NP_082625	mmu:72776	
K18671	HDAC10	mmu05203(	PF00850.24	GO:008009	cytoplasmic	NP_954668	mmu:170787	
K24048	MPP2_6		PF00625.27	GO:007016	cytoplasmic	NP_001348	mmu:56524	
K09493	CCT1, TCP1		PF00118.29	GO:000024	cytoplasmic	NP_038714	mmu:21454	
K15129	MED8		PF10232.14	GO:006026	nuclear	NP_064384	mmu:80509	
.HAM(CDKN1A interacting zinc fi			PF23330.1(z	GO:007259	nuclear	XP_006498	mmu:68379	
K04534	GNAO, G-	mmu04724(	PF00503.26	GO:001707	cytoplasmic	NP_001401	mmu:14681	
K00721	DPM1	mmu00510(	PF00535.33	GO:001967	mitochondr	NP_034202	mmu:13480	
K06238	COL6A	mmu05165(	PF00092.34	GO:000520	ER	NP_034063	mmu:12833	
K24973	DALRD3		PF05746.21	GO:003563	cytoplasmic	NP_080654	mmu:67789	
K15335	NSUN2, TRM4		PF01189.23	GO:200073	cytoplasmic	AQS27603.	mmu:28114	
K05653	ABCB2, TA	mmu02010(	PF00664.28	GO:000810	extracellula	AIC84015.1	mmu:21354	
K03254	EIF3A		PF22591.2(	GO:007154	cytoplasmic	NP_034253	mmu:13669	
K14640	SLC20A, PIT		PF01384.25	GO:003250	plasma mer	NP_056562	mmu:20515	
K11671	NFRKB, IN	mmu03082(	PF14465.12	GO:008009	nuclear	EDL25343.	mmu:23513	TF_others
K10578	UBE2J1, N	mmu04141(	PF00179.31	GO:003315	cytoplasmic	NP_001342	mmu:56228	
K22072	ISCA2		PF01521.26	GO:002260	mitochondr	XP_006516	mmu:74316	
K13675	B3GALTL	mmu00514(	PF02434.22	GO:000578	ER	NP_001074	mmu:381694	
K09453	SNAPC4		PF00249.37	GO:003277	nuclear	EDL08305.	mmu:22764	MYB
K12811	DDX46, PF	mmu03040(	PF00270.35	GO:002260	nuclear	NP_001268	mmu:212880	
K16497	PCDHGC		PF00028.23	GO:005080	plasma mer	AAM93563.	mmu:93708	
K13112	THRAP3		PF15440.11	GO:008009	nuclear	NP_001343	mmu:230753	

K10416	DYNC1LI,	mmu04962(	PF05783.17	GO:199009	cytoplasmic	NP_001013	mmu:234663
K04802	PCNA	mmu04530(	PF02747.20	GO:004423	nuclear	NP_035175	mmu:18538
K20791	NAA10_11, ARD1_2		PF00583.31	GO:003124	nuclear	XP_021043	mmu:56292
K00814	GPT, ALT	mmu01100(	PF00155.27	GO:000548	mitochondr	EDL11016.	mmu:108682
K19948	MICALL1		PF12130.13	GO:000810	cytoplasmic	NP_803412	mmu:27008
K02272	COX7C	mmu05208(	PF02935.21	GO:001986	mitochondr	NP_031775	mmu:12867
K23863	OSTM1		PF09777.14	GO:003032	plasma mer	NP_766004	mmu:14628
J7M(Myeloid leukemia factor)			PF10248.14	GO:008009	cytoplasmic	NP_001101	mmu:30853
JNC(transmembrane protein 177)				GO:003109	peroxisoma	NP_780315	mmu:66343
K15281	SLC35D1_2_3		PF03151.22	GO:003020	ER	NP_001343	mmu:242585
K15047	HNRNPUI	mmu05164(	PF13671.12	GO:000565	nuclear	NP_659171	mmu:232989
K00166	BCKDHA,	mmu01100(	PF00676.26	GO:005138	mitochondr	NP_031559	mmu:12039
K10908	POLRMT, RPO41		PF00940.24	GO:000635	mitochondr	NP_766139	mmu:216151
K08754	FABP5	mmu03320(	PF00061.29	GO:008009	cytoplasmic	NP_034764	mmu:16592
K08020	RAPGEF6,	mmu04530(	PF00617.24	GO:009859	cytoplasmic	NP_001239	mmu:192786
K16549	PDE4DIP		PF23246.1(	GO:003111	cytoplasmic	AAI41186.1	mmu:83679
XUVY(Cross-linked envelope protei			PF02389.20	GO:000573	cytoplasmic	XP_021052	mmu:20753
K06115	SPTBN1_4_	mmu04820(	PF00435.27	GO:003288	nuclear	NP_787030	mmu:20742
K12323	ANPRA, N	mmu01100(	PF00211.25	GO:004858	cytoplasmic	NP_032753	mmu:18160
K23710	ABI3		PF07815.19	GO:001922	cytoplasmic	NP_079935	mmu:66610
K12329	PPP1R12B,	mmu04810(	PF15898.10	GO:003243	nuclear	NP_001355	mmu:329251
V3C(Nuclear receptor 2C2-associat			PF00754.31	GO:000565	cytoplasmic	NP_001020	mmu:75692
K10573	UBE2A, UE	mmu04120(	PF00179.31	GO:006165	cytoplasmic	NP_861427	mmu:22209
K14319	RANGAP1	mmu03013(	PF07834.16	GO:003315	nuclear	AAH14855.	mmu:19387
K14573	NOP4, RBI	mmu03008(	PF00076.28	GO:009030	nuclear	NP_598686	mmu:68272
K26544	SEC14, SEC14L		PF04707.20	GO:000582	cytoplasmic	NP_083053	mmu:74136
K16075	MRS2, MFM1		PF22099.1(	GO:000521	mitochondr	NP_001013	mmu:380836
FVG(family with sequence similari			PF01597.25	GO:003243	cytoplasmic	NP_001074	mmu:230234
K05502	BMP1, TLD		PF00431.26	GO:000417	plasma mer	NP_001346	mmu:12153
K03992	MASP1	mmu04610(	PF00089.32	GO:000425	plasma mer	NP_032581	mmu:17174
K03997	C8A	mmu05171(	PF01823.24	GO:004858	extracellulo	EDL30830.	mmu:230558
K12876	RBM8A, Y1	mmu03013(	PF00076.28	GO:005116	nuclear	KAF607566	mmu:60365
K17916	KIF16B, SN	mmu04814(	PF00225.29	GO:009951	cytoplasmic	NP_001074	mmu:16558
K05692	ACTB_G1	mmu04530(	PF00022.25	GO:001562	cytoplasmic	BAE30280.	mmu:11461
K11592	DICER1, D	mmu05206(	PF20931.2(	GO:005501	extracellulo	EDL18787.	mmu:192119
K04570	BCL2L1, bc	mmu05022(	PF00452.24	GO:200123	cytoplasmic	AAC72232.	mmu:12048
K15779	PGM2	mmu00052(	PF02878.22	GO:004323	cytoplasmic	NP_079976	mmu:66681
K27678	ENKD1		PF13864.11	GO:004299	cytoplasmic	NP_938041	mmu:102124
K14774	UTP25, DEF		PF22916.2(	GO:003450	nuclear	NP_663390	mmu:215193
K06730	IGSF8, CD316		PF07686.23	GO:004001	plasma mer	NP_536344	mmu:140559
K00108	betA, CHD	mmu00260(	PF05199.19	GO:004239	cytoplasmic	BAC39931.	mmu:218865
Z0HI(protein phosphatase 1, regulat			PF11232.14	GO:008009	mitochondr	NP_598710	mmu:84113
K13097	TET1		PF12851.13	GO:008009	nuclear	NP_001240	mmu:52463

K10089	M6PR	mmu04142(	PF02157.21	GO:000810	plasma mer	NP_034879	mmu:17113	
K10358	MYO6	mmu05132(	PF00063.27(Myosin_he		nuclear	NP_001395	mmu:17920	
K09228	KRAB		PF13465.12	GO:008009	cytoplasmic	BAE22151.1	mmu:22640	zf-C2H2
K23859	PLEKHG1_2_3		PF00621.25	GO:003304	cytoplasmic	XP_006539	mmu:101497	
K05699	ACTN1_4	mmu04530(	PF00435.27	GO:003243	cytoplasmic	NP_001333	mmu:109711	
K19871	SALL		PF13894.12	GO:005125	cytoplasmic	NP_001231	mmu:50524	zf-C2H2
K13523	AGPAT3_4	mmu00564(	PF16076.10	GO:000665	lysosomal	NP_080920	mmu:68262	
K13344	PEX13	mmu04146(	PF04088.18	GO:003444	peroxisoma	NP_076140	mmu:72129	
K06554	IGLL1, IGI	mmu05340(	PF07654.21	GO:004316	extracellulo	AAB28160.1	mmu:16136	
ZQXJ(DEP domain containing)			PF00610.27	GO:000998	mitochondr	NP_659053	mmu:211896	
K06059	ADAM17, T	mmu04148(	PF13688.12	GO:004340	plasma mer	AAI45271.1	mmu:11491	
Ribosomal protein L15)			PF13465.12	GO:004885	nuclear	NP_001289	mmu:67255	zf-C2H2
K02979	RP-S28e, Rl	mmu05171(	PF01200.24	GO:002260	cytoplasmic	XP_010725	mmu:54127	
K05769	ARHGEF4_	mmu04810(	PF00621.25	GO:009859	cytoplasmic	AAI45286.1	mmu:219140	
K12885	RBMX, HN	mmu03040(	PF00076.28	GO:008009	nuclear	NP_001160	mmu:19655	
K24685	ZFAND2		PF01428.22	GO:014003	cytoplasmic	NP_001153	mmu:68818	
K07861	RAC3	mmu05200(	PF00071.28	GO:006024	cytoplasmic	NP_001092	mmu:170758	
K10749	GMNN		PF07412.17	GO:002260	cytoplasmic	KAF158498	mmu:57441	
K04393	CDC42	mmu04530(	PF00071.28	GO:004521	cytoplasmic	NP_001034	mmu:12540	
K17973	NAA25, MDM20		PF09797.15	GO:000573	cytoplasmic	NP_766310	mmu:231713	
K08801	PASK		PF00069.31	GO:009008	cytoplasmic	AAI50822.1	mmu:269224	
K06553	VPREB, CD179a		PF07686.23(V-set:Immu		extracellulo	EDL03609.1	mmu:22362	
LEH4(Mast cell immunoglobulin-lik			PF13895.12	GO:005124	plasma mer	NP_001258	mmu:380732	
K04593	ADGRL2, LPHN2		PF02354.21(Latrophilin		plasma mer	NP_001392	mmu:99633	
K22073	IBA57			GO:002260	mitochondr	NP_776146	mmu:216792	
K06255	HSPG2	mmu04512(	PF07679.22	GO:000810	plasma mer	NP_032331	mmu:15530	
K17785	IMMT, MIC60		PF09731.14	GO:009879	mitochondr	BAE34204.1	mmu:76614	
K24080	LEMD2		PF09402.16	GO:000810	cytoplasmic	NP_666187	mmu:224640	
K09575	FKBP9_10		PF00254.34	GO:000645	ER	AAH43129.	mmu:27055	
K20839	RSKL		PF00069.31	GO:000554	cytoplasmic	NP_848890	mmu:320119	
K10317	FBXO42		PF24681.1(I	GO:003067	cytoplasmic	BAE42319.1	mmu:213499	
K26458	NKRF		PF11952.13(XTBD:XRN		plasma mer	NP_084167	mmu:77286	
K14972	PAXIP1, PTIP		PF00533.33	GO:004423	nuclear	NP_061366	mmu:55982	
XPRI(-Drosophila)			PF05041.20	GO:000557	Golgi appa	NP_659117	mmu:104401	
K14951	ATP13A3_4_5		PF00122.25	GO:190204	cytoplasmic	NP_001121	mmu:224088	
K07187	IRS2	mmu04910(	PF02174.23	GO:009008	nuclear	NP_001074	mmu:384783	
K07520	ABLIM	mmu04360(	PF16182.10	GO:002260	cytoplasmic	NP_001171	mmu:231148	
K24744	WDR37		PF00400.38	GO:002203	nuclear	BAE26979.1	mmu:207615	
YG4J(family with sequence similarit			PF15051.11	GO:000579	Golgi appa	NP_573450	mmu:68659	
K08960	CSNK1E	mmu04068(	PF00069.31	GO:003292	cytoplasmic	NP_001276	mmu:27373	
K04650	NCOR1, N	mmu05202(	PF15784.10	GO:000989	nuclear	NP_035438	mmu:20185	MYB
K09905	B4GALT5	mmu00600(	PF13733.12	GO:004846	Golgi appa	NP_062809	mmu:56336	
YWMF(musculoskeletal, embryonic			PF15682.10	GO:004859	nuclear	NP_852055	mmu:66175	

K06632	WEE1	mmu05170(	PF00069.31	GO:200024	cytoplasmic	NP_033542	mmu:22390	
K04441	P38	mmu05208(	PF00069.31	GO:003288	cytoplasmic	NP_036081	mmu:26416	
K05673	ABCC4	mmu02010(	PF00664.28	GO:001572	cytoplasmic	AAI50823.1	mmu:239273	
K13709	ARHGAP5	mmu04670(	PF19518.5(I	GO:000367	cytoplasmic	XP_021034.	mmu:11855	
K01109	INPP4	mmu04070(	Phosphatid	GO:010601	mitochondr	NP_001361	mmu:269180	
K10470	KBTBD2		PF00651.37	GO:004349	cytoplasmic	BAC98184.	mmu:21097	ZBTB
K24641	ZBED6		PF02892.21	GO:009008	nuclear	NP_001160	mmu:66711	zf-BED
K02906	RP-L3, MR	mmu03010(	PF00297.28	GO:001593	mitochondr	AAH11128.	mmu:94062	
K03193	UST	mmu00532(	PF03567.20	GO:002260	Golgi appa	BAE37128.	mmu:338362	
K02151	ATPeV1F,	mmu05165(	PF01990.22	GO:004696	cytoplasmic	NP_079657	mmu:66144	
K15309	CRTC1, TC	mmu05166(	PF12885.12	GO:008009	cytoplasmic	BAC97983.	mmu:382056	
K12581	CNOT7_8,	mmu03018(	PF04857.26	GO:003577	nuclear	BAE20968.	mmu:18983	
K10494	ZBTB7		PF00651.37	GO:004213	nuclear	KAF592424	mmu:22724	ZBTB
X4F(receptor (chemosensory) trans			PF13695.12	GO:000810	cytoplasmic	NP_075875	mmu:67775	
K11665	INO80, IN	mmu03082(	PF00176.29	GO:000962	nuclear	NP_080850	mmu:68142	
20VA(Tumor suppressing subtransfe			PF15264.11	GO:002260	cytoplasmic	NP_001108	mmu:56844	
K19995	SCAMP		PF04144.18	GO:000548	cytoplasmic	NP_083429	mmu:107767	
K05304	NANS, SAS	mmu00520(	PF03102.20	GO:004423	cytoplasmic	NP_444409	mmu:94181	
K08752	FABP3	mmu03320(	PF00061.29	GO:004544	cytoplasmic	NP_034304	mmu:14077	
K08520	SEC22A_C		PF13774.12	GO:000810	cytoplasmic	NP_001405	mmu:317717	
K06752	MHC2	mmu05323(	PF00993.25	GO:004213	lysosomal	NP_034508	mmu:14960	
Cold shock protein)			PF00313.28	GO:000548	cytoplasmic	BAE29409.	mmu:52502	CSD
K10755	RFC2_4	mmu03030(	PF00004.35	GO:008009	nuclear	BAE27622.	mmu:106344	
K24974	METTL2		PF08242.18	GO:000817	cytoplasmic	NP_766155	mmu:52686	
K19327	ANO10, TMEM16K		PF04547.17	GO:000522	cytoplasmic	NP_598740	mmu:102566	
K07983	FCER1G	mmu04664(	PF11628.13	GO:004330	ER	NP_034315	mmu:14127	
K13999	CKAP4, CL	mmu04141(	Protein pro	GO:000585	ER	NP_780660	mmu:216197	
K00683	QPCT		PF04389.22	GO:004322	Golgi appa	NP_080387	mmu:67369	
K12391	AP1G1	mmu04142(	PF01602.26	GO:000810	cytoplasmic	NP_001288	mmu:11765	
K11981	RNF41, NR	mmu04144(	PF08941.15	GO:008009	cytoplasmic	NP_001157	mmu:67588	
K04503	CCND1	mmu04530(	PF00134.28	GO:003202	cytoplasmic	NP_031657	mmu:12443	
K02519	infB, MTIF2		PF00009.33	GO:004316	cytoplasmic	NP_001269	mmu:76784	
K00920	PIP4K2	mmu04070(	PF01504.24	GO:000578	cytoplasmic	NP_001346	mmu:117150	
Thymocyte nuclear protein)			PF01878.24	GO:004323	nuclear	NP_653126	mmu:77862	TF_others
K15498	PPP6C		PF00149.33	GO:001679	cytoplasmic	NP_001017	mmu:67857	
K10703	HACD, PH	mmu01040(	PF04387.19	GO:000663	ER	NP_001364	mmu:66775	
K16197	YWHAB_Q	mmu04114(	PF00244.26	GO:000449	cytoplasmic	NP_001405	mmu:54401	
K01868	TARS, thrS	mmu00970(	PF00587.30	GO:000643	cytoplasmic	NP_082207	mmu:71807	
K14771	NOC4, UTP19		PF03914.23	GO:003068	cytoplasmic	AAH24616.	mmu:100608	
K18753	ZFP36L	mmu04218(	PF04553.17	GO:200073	cytoplasmic	NP_001001	mmu:12193	zf-CCCH
K10609	CUL4	mmu03420(	PF00888.28	GO:008009	cytoplasmic	NP_666319	mmu:99375	
K01889	FARSA, ph	mmu00970(	PF01409.25	GO:000028	cytoplasmic	EDL10963.	mmu:66590	
K04493	ICAT, CTN	mmu04310(	PF06384.16	GO:008009	cytoplasmic	NP_001135	mmu:67087	

K17969	FIS1, TTC1	mmu04137(	PF14853.12	GO:200123	peroxisoma	NP_079838	mmu:66437	
K03947	NDUFA3	mmu05208(	PF14987.11	GO:002260	ER	NP_079624	mmu:66091	
K16464	CEP250, CNAP1		PF15035.11	GO:003288	cytoplasmic	NP_001123	mmu:16328	
21ET(HIG1	hypoxia inducible domc		PF04588.18	GO:200123	cytoplasmic	XP_028638	mmu:56295	
K02882	RP-L18Ae,	mmu05171(	PF01775.22	GO:002262	cytoplasmic	NP_084027	mmu:76808	
K07439	CYP39A1	mmu00120(	PF00067.27	GO:000548	ER	NP_001272	mmu:56050	
K17300	LGALS3BP			GO:001602	cytoplasmic	BAE29246.	mmu:19039	
K19998	SCFD1, SLY1		PF00995.28	GO:000810	mitochondr	NP_084101	mmu:76983	
K11584	PPP2R5	mmu05165(	PF01603.26	GO:001922	nuclear	BAE20490.	mmu:225849	
			PF15766.10(DUF4695:I		cytoplasmic	NP_001138	mmu:620695	
K20302	TRAPPC3, BET3		PF04051.21	GO:000578	cytoplasmic	NP_001008	mmu:27096	
K23697	SHB_D_E_F		PF00017.30	GO:003577	cytoplasmic	NP_001028	mmu:230126	
K09188	MLL3	mmu00310(	PF00628.35	GO:008009	nuclear	NP_001074	mmu:23105	HMG
K05691	CTNNA	mmu04670(	PF01044.25	GO:004239	cytoplasmic	AAQ14965.	mmu:216033	
K23792	MPRIP		PF00169.35	GO:003243	nuclear	XP_006533	mmu:26936	
K12505	PDSS2	mmu00900(	PF00348.22	GO:199023	mitochondr	NP_082048	mmu:71365	
K12504	PDSS1	mmu00900(	PF00348.22	GO:199023	mitochondr	NP_062374	mmu:56075	
K20395	CD300B_D_F		PF07686.23	GO:190187	plasma mer	NP_001095	mmu:100043123	
K14140	IGTP	mmu05145(	PF05049.19	GO:000578	cytoplasmic	NP_061208	mmu:16145	
K15601	KDM3	mmu04714(	PF22988.2(I	GO:008009	nuclear	NP_001074	mmu:277250	
K15362	BRIP1, BA(	mmu03440(	PF06733.21	GO:008009	nuclear	EDL15780.	mmu:237911	
K17310	SAA		PF00277.23	GO:003299	ER	NP_035446	mmu:20211	
K04456	AKT	mmu05208(	PF00069.31	GO:001922	cytoplasmic	AAH66861.	mmu:23797	
cleavage stimulation factor, 3' pre-R			PF13465.12	GO:008009	cytoplasmic	NP_001243	mmu:31947	zf-C2H2
K10225	FADS3		PF00487.30	GO:000663	cytoplasmic	AKF17658.	mmu:60527	
K12829	SF3B2, SAP	mmu03040(	PF04037.18	GO:000613	nuclear	NP_084385	mmu:319322	
K04006	DAF, CD55	mmu04610(	PF00084.26	GO:190365	plasma mer	NP_034146	mmu:13136	
K06099	CSDA, ZO	mmu04530(	PF00313.28	GO:200123	nuclear	BAA03376.	mmu:56449	CSD
K24621	CERS2_4, I	mmu04071(	PF03798.21	GO:008009	ER	NP_001307	mmu:76893	Homeobox
K26402	TRAM2		PF03798.21	GO:000810	Golgi appa	NP_803128	mmu:170829	
K25204	XPO6, RAN	mmu03013(	PF08389.18	GO:005116	cytoplasmic	NP_001298	mmu:74204	
K23317	MTF1		PF13465.12	GO:008009	cytoplasmic	NP_032662	mmu:17764	zf-C2H2
K13966	SERPINB11_12		PF00079.25	GO:000486	cytoplasmic	NP_001186	mmu:71869	
HYY(coiled-coil domain containing			PF13270.11	GO:000699	cytoplasmic	NP_079731	mmu:66264	
K09228	KRAB		PF13465.12	GO:008009	nuclear	NP_033603	mmu:22775	zf-C2H2
K01370	CASP1	mmu05164(	PF00656.27	GO:004423	cytoplasmic	NP_033937	mmu:12362	
K13205	AAR2, C20orf4		PF05282.17	GO:000038	cytoplasmic	NP_001158	mmu:68295	
K17638	RIN1	mmu04014(	PF02204.23	GO:000548	cytoplasmic	NP_001360	mmu:225870	
K00933	E2.7.3.2	mmu00330(	PF00217.24	GO:003042	cytoplasmic	NP_067248	mmu:12709	
K02894	RP-L23e, R	mmu05171(	PF00238.24	GO:005116	cytoplasmic	NP_000969	mmu:65019	
K01528	DNM1_3	mmu04072(	PF01031.25	GO:009951	cytoplasmic	P39053.2(R	mmu:13429	
K25512	DPT		PF14704.11	GO:003101	extracellula	NP_062733	mmu:56429	
K03362	FBXW1_11	mmu05170(	PF00400.38	GO:009951	cytoplasmic	XP_032769	mmu:103583	

K05090	CSF1R, FM	mmu04010(	PF07714.23	GO:001922	plasma mer	NP_001032	mmu:12978	
SCI(chromosome 11 open reading f			PF15477.11(SMAP:Sma		cytoplasmic	EDM17748	mmu:56372	
K13273	PMVK	mmu00900(	PF04275.20	GO:000548	peroxisoma	NP_081060	mmu:68603	
K02367	EXT2	mmu00534(	PF09258.16	GO:000578	ER	BAE29346.	mmu:14043	
K05083	ERBB2, HE	mmu04530(	PF07714.23	GO:004858	extracellulo	NP_001003	mmu:13866	
K23026	LIMA1, EPLIN		PF00412.28	GO:003243	cytoplasmic	NP_001107	mmu:65970	
K05954	FNTB	mmu00900(	PF00432.27	GO:000548	cytoplasmic	EDL36446.	mmu:110606	
K08047	ADCY7	mmu04750(	PF00211.25	GO:001922	ER	NP_001032	mmu:11513	
K06776	PTPRK		PF00102.32(Y_phospha		plasma mer	NP_001346	mmu:19272	
K00485	FMO	mmu00982(	PF00743.25	GO:004592	cytoplasmic	NP_001347	mmu:55990	
K11980	RNF11		PF13639.12	GO:000815	nuclear	NP_038904	mmu:29864	
K02514	HS6ST1	mmu00534(	PF03567.20	GO:003250	Golgi appa	NP_056633	mmu:50785	
K09584	PDIA6, TX	mmu04141(	PF00085.26	GO:001649	ER	BAE39477.	mmu:71853	
K17335	NFAT5		PF16179.11	GO:008009	nuclear	NP_598718	mmu:54446	RHD
K10870	RAD51L2,	mmu03440(	PF08423.17	GO:000070	cytoplasmic	NP_444499	mmu:114714	
BVC(Brain expressed X-linked like			PF04538.17	GO:004323	nuclear	NP_035842	mmu:22381	
K11869	USP40		PF00443.35	GO:010100	nuclear	NP_001185	mmu:227334	
57A(PITH (C-terminal proteasome-			PF06201.19	GO:000989	cytoplasmic	NP_079687	mmu:66193	
321(Myb SANT-like DNA-binding			PF13837.12(Myb_DNA		mitochondr	XP_021027	mmu:235184	
K13104	ZNF830, CCDC16		PF23406.1(	GO:004858	nuclear	NP_080160	mmu:66983	
K24484	FNDC1		PF21731.2(TARSH_C:T		ER	NP_001390	mmu:68655	
HOsphatase); ENOG411282R(-)				GO:000647	cytoplasmic	NP_663385	mmu:214917	
K03061	PSMC2, RP	mmu05020(	PF00004.35	GO:003577	cytoplasmic	NP_001355	mmu:19181	
K05719	ITGB1, CD	mmu04530(	PF00362.24	GO:004867	extracellulo	NP_034708	mmu:16412	
K23580	IGFBP7		PF07679.22	GO:007149	extracellulo	AAH92538.	mmu:29817	
K06554	IGLL1, IGI	mmu05340(	PF07654.21	GO:000237	extracellulo	AAI06162.1	mmu:16136	
K16727	ARHGEF10		PF19056.6(	GO:003304	cytoplasmic	NP_001277	mmu:72754	
K16467	CNTLN			GO:000810	cytoplasmic	NP_001392	mmu:338349	
K17907	ATG9	mmu04140(	PF04109.21	GO:000810	cytoplasmic	NP_001003	mmu:245860	
K11577	KNTC1, ROD		PF10493.15	GO:000810	cytoplasmic	NP_001035	mmu:208628	
K08512	VAMP8	mmu04140(	PF00957.27	GO:199079	cytoplasmic	NP_058074	mmu:22320	
K18329	ISG20L2		PF00929.31	GO:001679	nuclear	NP_808331	mmu:229504	
K11218	JAK3	mmu05166(	PF07714.23	GO:007066	cytoplasmic	NP_001177	mmu:16453	
K12618	XRN1, SEP	mmu03008(	PF03159.24	GO:007104	nuclear	NP_001298	mmu:24127	
K07441	ALG14	mmu00510(	PF08660.17	GO:000815	ER	NP_077140	mmu:66789	
K06679	MAD1	mmu05166(	PF05557.18	GO:004584	cytoplasmic	NP_001345	mmu:17120	
K24540	PRKRA		PF00035.32	GO:200123	cytoplasmic	NP_036001	mmu:23992	
K06110	EXOC3, SEC6		PF06046.19	GO:006164	cytoplasmic	BAD21391.	mmu:211446	
71I1(chromosome 4 open reading fr			PF15061.11	GO:003361	extracellulo	NP_001138	mmu:66278	
K20032	ZDHHC13_17, HIP14		PF12796.13	GO:000578	cytoplasmic	NP_082307	mmu:243983	
AXC(Protein tyrosine phosphatase,			PF15713.10	GO:000557	extracellulo	EDL33023.	mmu:19265	
K13594	ACVRL1, A	mmu04060(	PF07714.23	GO:004633	cytoplasmic	AAH14291.	mmu:11482	
K05691	CTNNA	mmu04670(	PF01044.25	GO:200123	cytoplasmic	NP_033948	mmu:12385	

K15113	SLC25A28_37, MFRN		PF00153.32	GO:000367	cytoplasmic	NP_080607	mmu:67712	
K02958	RP-S15e, RI	mmu05171(	PF00203.26	GO:005116	mitochondr	NP_001297	mmu:20054	
K16768	CEP350		PF01302.31	GO:004299	nuclear	NP_001034	mmu:74081	
J9X(Chromosome 14 open reading			PF03694.18	GO:000578	ER	NP_001100	mmu:58520	
K13044	HNRNPABD		PF00076.28	GO:001922	nuclear	NP_001392	mmu:50926	
K16794	PAFAH1B1	mmu00565(	PF00400.38	GO:000167	cytoplasmic	XP_034497	mmu:18472	
K18042	STYX		PF00782.25	GO:000989	cytoplasmic	EDL20695.	mmu:56291	
ıcetyltransferase)			PF04061.19	GO:001972	peroxisoma	NP_001346	mmu:66844	
K04455	HSPB1	mmu05146(	PF00011.27	GO:200123	cytoplasmic	NP_038588	mmu:15507	
K06752	MHC2	mmu05323(	PF00993.25	GO:000810	lysosomal	AAA91135.	mmu:14998	
K11548	NUF2, CDCA1		PF03800.19	GO:003126	nuclear	NP_001342	mmu:66977	
K06269	PPP1C	mmu04750(	PF00149.33	GO:003292	cytoplasmic	NP_001411	mmu:434233	
K15501	PPP6R3, SAPS3		PF04499.20	GO:003023	nuclear	NP_083275	mmu:52036	
K15014	SLC29A1_2	mmu05034(	PF01733.24	GO:190379	cytoplasmic	NP_076085	mmu:71279	
K11138	RIF1	mmu04550(	PF12231.13	GO:008009	cytoplasmic	NP_001342	mmu:51869	
K24350	UBXN7		PF00789.26	GO:004438	nuclear	NP_808301	mmu:224111	
K15139	MED22		PF06179.17	GO:006026	nuclear	NP_001365	mmu:20933	
K14965	DPY30		PF05186.18	GO:000989	cytoplasmic	NP_001139	mmu:66310	
KU1S(prolactin)			PF00103.25	GO:000512	extracellulo	NP_038794	mmu:27372	
K20403	TTI1	mmu04150(	PF24173.1(	GO:003193	cytoplasmic	NP_083558	mmu:75425	
K13168	CLASRP, SFRS16		PF09750.14	GO:001607	nuclear	NP_057889	mmu:53609	
K09570	FKBP3		PF18410.7(I	GO:004323	nuclear	NP_038930	mmu:30795	
K23625	ATXN2_2L	mmu05014(	PF07145.20	GO:000548	cytoplasmic	XP_030110	mmu:20239	
K11838	USP7, UBP	mmu05169(	PF12436.14	GO:008009	cytoplasmic	XP_006522	mmu:252870	
KS4B(asunder, spermatogenesis regu			PF10221.14	GO:003450	nuclear	NP_620096	mmu:71177	
K13154	ZCRB1		PF00076.28	GO:000613	nuclear	NP_080301	mmu:67197	
K08267	RICTOR	mmu04150(	PF14664.11	GO:001922	cytoplasmic	NP_084444	mmu:78757	
K06553	VPREB, CD179a		PF07686.23(V-set:Immu		ER	DBA12414.	mmu:22362	
K18086	MTMR14	mmu04070(	Phosphatid	GO:005286	cytoplasmic	NP_081125	mmu:97287	
251N(proline-serine-threonine phos			PF01166.23	GO:000989	cytoplasmic	BAE32411.	mmu:78829	TSC22
K06067	HDAC1_2	mmu05165(	PF00850.24	GO:200123	cytoplasmic	XP_003500	mmu:433759	
K11306	MYST4, KAT6B		PF01853.24	GO:008009	nuclear	XP_006519	mmu:54169	
K26404	CIAO3		PF02906.19	GO:003250	cytoplasmic	NP_080514	mmu:67563	
K22525	MKL		PF02755.20	GO:200123	cytoplasmic	NP_001076	mmu:223701	
K25228	APC15, AN	mmu04110(	PF15243.11	GO:003304	cytoplasmic	NP_001029	mmu:75430	
K14539	LSG1	mmu03008(	PF01926.29	GO:005116	cytoplasmic	NP_835170	mmu:224092	
K24350	UBXN7		PF00789.26	GO:004438	nuclear	AAH62904.	mmu:224111	
K17253	MFGE8	mmu04148(	PF00754.31	GO:004212	lysosomal	NP_032620	mmu:17304	
K06819	NRP2		PF00431.26	GO:004867	plasma mer	NP_001070	mmu:18187	
K02959	RP-S16, MR	mmu03010(	PF00886.24	GO:001593	mitochondr	NP_079716	mmu:66242	
K08343	ATG3	mmu04140(	PF03987.20	GO:000810	cytoplasmic	NP_080678	mmu:67841	
K03986	C1QA	mmu05171(	PF00386.27	GO:005080	extracellulo	BAE35005.	mmu:12259	
PBH(Family with sequence similar			PF05811.18	GO:000573	cytoplasmic	NP_079867	mmu:66488	

K05130	IFNAR1	mmu05165(	PF09294.16	GO:001922	extracellul	AAA37890.	mmu:15975	
K21954	FYCO1	mmu05132(	PF01363.27	GO:000989	cytoplasmic	NP_001103	mmu:17281	
FA7(Chromosome 5 open reading f			PF15435.11	GO:003303	cytoplasmic	NP_001342	mmu:52392	
K19931	LIN7		PF00595.29	GO:009859	cytoplasmic	NP_001068	mmu:22343	
K23625	ATXN2_2L	mmu05014(	PF06741.18(LsmAD:Lsr		cytoplasmic	XP_030110	mmu:20239	
K02564	nagB, GNP	mmu00520(	PF01182.26	GO:000599	cytoplasmic	NP_036067	mmu:26384	
K16682	AJUBA, LIM	mmu04390(	PF00412.28	GO:003577	cytoplasmic	NP_038888	mmu:29806	
K24127	MAGE		PF01454.25	GO:009859	mitochondr	NP_444431	mmu:107528	
K23688	BCAR3, SH2D3B		PF00017.30	GO:000548	mitochondr	NP_038895	mmu:29815	
K05725	PTK2, FAK	mmu05208(	PF07714.23	GO:004212	cytoplasmic	XP_036015	mmu:14083	
K24759	WDR90		PF23342.1(	GO:002260	cytoplasmic	NP_001157	mmu:106618	
K20196	KIF3B	mmu04814(	PF00225.29	GO:000810	nuclear	NP_032470	mmu:16569	
K10462	KLHL25_37, ENC1, EN		PF01344.31	GO:008009	cytoplasmic	NP_001116	mmu:20795	ZBTB
K08843	LRRK1		PF00069.31	GO:001922	cytoplasmic	NP_666303	mmu:233328	
erine-type endopeptidase inhibitor (			PF00188.32	GO:000557	extracellul	NP_033769	mmu:11572	
K07071	K07071		PF08338.16	GO:000382	mitochondr	NP_001076	mmu:654795	
K18246	CA4	mmu04964(	PF00194.26	GO:000548	plasma mer	NP_031633	mmu:12351	
K09377	CSRP	mmu04820(	PF00412.28	GO:003103	cytoplasmic	EDL21718.	mmu:13008	
K04678	SMURF	mmu04120(	PF00632.30	GO:005116	cytoplasmic	XP_006504	mmu:75788	
K12755	MYL9	mmu04530(	PF13499.12	GO:003243	cytoplasmic	NP_001094	mmu:98932	
K17420	MRPL39			GO:001593	cytoplasmic	AAH12274.	mmu:27393	
K13132	GEMIN4			GO:002260	cytoplasmic	AAH31425.	mmu:276919	
K05132	IFNGR1, C	mmu05164(	PF07140.17	GO:001922	plasma mer	NP_034641	mmu:15979	
K20318	SYS1		PF09801.14	GO:000810	extracellul	NP_001386	mmu:66460	
K22866	TCTEX1D2		PF03645.18	GO:003150	cytoplasmic	NP_079605	mmu:66061	
K01079	serB, PSPH	mmu01100(	PF00702.32	GO:003357	cytoplasmic	NP_598661	mmu:100678	
K06751	MHC1	mmu05165(	PF00129.23	GO:009857	extracellul	NP_001334	mmu:14972	
K03283	HSPA1_6_8	mmu04915(	PF00012.26	GO:001989	cytoplasmic	BAE40394.	mmu:15481	
K19329	WWOX		PF00106.31	GO:200123	cytoplasmic	NP_062519	mmu:80707	
K16746	BBS1		PF14779.12	GO:000810	cytoplasmic	NP_001028	mmu:52028	
K06100	SYMPK	mmu04530(	PF12295.13	GO:003577	cytoplasmic	NP_080881	mmu:68188	
SYS(Catalyzes the 1,3-allylic rearr			PF03803.21	GO:001712	cytoplasmic	NP_848826	mmu:235527	
K19677	IFT81		PF18383.6(I	GO:000858	cytoplasmic	NP_001345	mmu:12589	
K03030	PSMD14, R	mmu05020(	PF23594.1(I	GO:000989	cytoplasmic	NP_001020	mmu:59029	
				GO:000557	Golgi appa	NP_001013	mmu:210962	
K11479	AURKB	mmu03083(	PF00069.31	GO:003608	cytoplasmic	NP_035626	mmu:20877	
K00685	ATE1		PF04377.21	GO:000405	cytoplasmic	NP_001389	mmu:11907	
K06704	ADAM10, (	mmu04148(	PF13574.12	GO:005116	extracellul	NP_031425	mmu:11487	
K05731	TIAM1	mmu04530(	PF23014.2(I	GO:001097	nuclear	NP_001139	mmu:21844	
K01325	KLK1_2	mmu04614(	PF00089.32	GO:000425	lysosomal	NP_034769	mmu:16612	
K12894	HNRNPA0		PF00076.28	GO:008009	nuclear	NP_084148	mmu:77134	
K14167	CDKN3, KAP		PF05706.17	GO:001679	cytoplasmic	BAB26929.1	mmu:72391	
CV8(Family with sequence similar			PF16218.11	GO:000573	mitochondr	NP_001229	mmu:223433	

K01900	LSC2	mmu00020(	PF08442.15	GO:004423	mitochondr	NP_035637	mmu:20917	
K17592	SACS		PF05168.19	GO:000573	cytoplasmic	NP_056603	mmu:50720	
K05063	IL12RB1, C	mmu04658(	PF00041.27	GO:000989	plasma mer	XP_006509	mmu:16161	
K00908	CAMKK1	mmu05034(	PF00069.31	GO:003563	cytoplasmic	NP_001349	mmu:55984	
K10099	FBXO2, NI	mmu04141(	PF04300.19	GO:008009	cytoplasmic	NP_001406	mmu:230904	
K13617	PPME1		PF12697.13	GO:007098	mitochondr	NP_082568	mmu:72590	
K12599	SKI2, SKIV	mmu03018(	PF08148.17	GO:001707	cytoplasmic	NP_067312	mmu:108077	
K05292	PIGT	mmu00563(	PF04113.19	GO:199023	lysosomal	BAE34008.1	mmu:78928	
K22544	SAMHD1	mmu03250(	PF07647.24	GO:004643	nuclear	Q60710.3(R	mmu:56045	
K23195	CTCF, CTCFL		PF13465.12	GO:004356	cytoplasmic	BAE24263.1	mmu:13018	zf-C2H2
K00472	P4HA	mmu00330(	PF23558.1(	GO:000578	ER	NP_035160	mmu:18451	
K08845	ARAF, ARA	mmu05208(	PF07714.23	GO:001922	cytoplasmic	NP_033833	mmu:11836	
				GO:000557	plasma mer	XP_057636708.1(uncharacterized L		
.4KX(Immunoglobulin); ENOG410			PF07686.23	GO:000548	mitochondr	AFW17592.1(IgM heavy chain vario		
K12462	ARHGDI, I	mmu04962(	PF02115.23	GO:005124	cytoplasmic	NP_001288	mmu:11857	
K04429	TAO	mmu04010(	MAPK sign	GO:005149	cytoplasmic	XP_025225	mmu:216965	
K05464	GAS6	mmu01521(	PF00054.30	GO:000548	plasma mer	NP_062394	mmu:14456	
K19559	LTBP1	mmu04350(	PF07645.21	GO:000810	plasma mer	NP_064303	mmu:268977	
K04281	CAST		PF00748.24	GO:001989	nuclear	NP_001288	mmu:12380	
K16476	NIN		PF13499.12	GO:000024	cytoplasmic	NP_001074	mmu:18080	
K17975	KTN1		PF05104.17	GO:000810	ER	Q61595.1(R	mmu:16709	
K25400	SCLT1			GO:002260	cytoplasmic	NP_001074	mmu:67161	
K12008	TRIM26		PF00622.36	GO:004858	cytoplasmic	NP_001020	mmu:22670	
K05616	SLC1A5	mmu05230(	PF00375.23	GO:004877	plasma mer	BAE28496.1	mmu:20514	
K16774	IQCB1, NPHP5		PF00612.33	GO:004299	cytoplasmic	NP_796102	mmu:320299	
ZRQX(par-3 partitioning defective 3			PF12053.14	GO:009859	cytoplasmic	CAI24823.1	mmu:72823	
K18342	OTUD6		PF02338.25	GO:002260	nuclear	NP_690025	mmu:72201	
K01672	CA	mmu00910(	PF00194.26	GO:000673	cytoplasmic	NP_001077	mmu:12346	
K21852	DOCK6_7_8		PF06920.19	GO:003003	cytoplasmic	NP_080358	mmu:67299	
K12489	ACAP	mmu04144(	PF16746.10	GO:000548	cytoplasmic	NP_722483	mmu:216859	
K24990	RBMS		PF00076.28	GO:000814	mitochondr	NP_062685	mmu:56516	
K14785	ESF2, ABT1			GO:002260	nuclear	NP_038952	mmu:30946	
K06234	RAB23		PF00071.28	GO:003315	cytoplasmic	P35288.2(R	mmu:19335	
K16738	NDE1, NUDE		PF04880.18	GO:009951	cytoplasmic	NP_075806	mmu:67203	
K22291	STARD3	mmu04979(	PF10457.14	GO:004323	peroxisoma	NP_001412	mmu:59045	
K07890	RAB21		PF00071.28	GO:000810	cytoplasmic	NP_077774	mmu:216344	
K14301	NUP107, N	mmu03013(	PF04121.18	GO:003450	cytoplasmic	AAH92261.	mmu:103468	
K12620	LSM1	mmu03018(	PF01423.28	GO:007104	cytoplasmic	NP_080308	mmu:67207	
K07891	RAB22	mmu04144(	PF00071.28	GO:009006	cytoplasmic	NP_077756	mmu:19334	
K17602	YLPM1		PF13671.12	GO:003304	nuclear	NP_848140	mmu:56531	
K07374	TUBA	mmu04530(	PF00091.31	GO:000520	cytoplasmic	XP_059977	mmu:22147	
K17388	ROCK2	mmu04530(	PF00069.31	GO:001474	cytoplasmic	XP_036013	mmu:19878	
K20792	NAA15_16		PF12569.14	GO:003124	cytoplasmic	NP_444319	mmu:74838	

K07374	TUBA	mmu04530(	PF00091.31	GO:009951	cytoplasmic	NP_001037	mmu:22143	
KQDW(Excision repair cross-compl			PF07159.17	GO:003304	cytoplasmic	NP_001139	mmu:76820	
K10159	TLR2, CD2	mmu05323(	PF13855.12	GO:003249	extracellulo	XP_006501	mmu:24088	
27S0(family with sequence similarity			PF14774.11	(FAM177:F/	cytoplasmic	NP_001093	mmu:73385	
K15559	RTT103		PF16566.10	GO:001989	nuclear	NP_001347	mmu:225283	
K23879	GRN		PF00396.23	GO:000578	extracellulo	BAE30030.	mmu:14824	
K18426	ECHDC1	mmu00640(	PF00378.25	GO:003444	cytoplasmic	NP_080131	mmu:52665	
K08492	STX18	mmu04130(	PF10496.14	GO:006062	cytoplasmic	NP_081235	mmu:71116	
K03245	EIF3J		PF08597.15	GO:002260	cytoplasmic	NP_653128	mmu:78655	
K21919	KCTD9		PF00805.28	GO:002260	cytoplasmic	EDL35968.	mmu:105440	
K00871	PHKG	mmu04922(	PF00069.31	GO:000016	cytoplasmic	NP_081164	mmu:68961	
K10799	TNKS		PF00023.36	GO:000024	mitochondr	NP_001157	mmu:74493	
KRAB(striatin interacting protein)			PF11882.13	GO:002260	nuclear	BAE28288.	mmu:229707	
K12191	CHMP2A	mmu04217(	PF03357.26	GO:000810	cytoplasmic	XP_008821	mmu:68953	
K01965	PCCA, pcc/	mmu00630(	PF02786.22	GO:000663	mitochondr	NP_659093	mmu:110821	
KQ2Z(Suppression of tumorigenicit			PF04184.18	GO:000557	Golgi appa	NP_001276	mmu:64213	
K03099	SOS	mmu05208(	PF00617.24	GO:190545	cytoplasmic	NP_033257	mmu:20662	
K18261	DYSF		PF00168.36	GO:009951	cytoplasmic	XP_006506	mmu:26903	
K26240	C2CD2L, C2CD2, TME		PF18696.7(	GO:003577	ER	BAE32190.	mmu:71764	
K14799	TSR1		PF04950.18	GO:003068	nuclear	NP_796299	mmu:104662	
K10307	FBXO30		PF15966.10	GO:000367	cytoplasmic	NP_001161	mmu:71865	
K00774	PARP16		PF18084.6(	GO:000548	cytoplasmic	BAE41422.	mmu:214424	
K07827	KRAS, KR/	mmu05208(	PF00071.28	GO:001922	cytoplasmic	NP_001390	mmu:16653	
K02961	RP-S17, MF	mmu03010(	PF00366.26	GO:004423	mitochondr	KAF163012	mmu:66258	
K06053	RBPSUH, R	mmu05165(	PF09270.16	GO:000548	nuclear	NP_001264	mmu:19664	CSL
K11425	WHSC1L1,	mmu00310(	PF00855.23	GO:000989	nuclear	NP_001074	mmu:234135	
K04439	ARRB	mmu04010(	MAPK sign	GO:000548	nuclear	XP_040614	mmu:109689	
K02261	COX2	mmu05208(	PF00116.26	GO:001545	cytoplasmic	NP_904331	mmu:17709	
K08767	ANGPTL4,	mmu04979(	PF00147.23	GO:006019	extracellulo	AAF86342.	mmu:57875	
K02308	DAXX	mmu04010(	PF03344.20	GO:200123	cytoplasmic	AIC83995.1	mmu:13163	
K15043	KPNA2_7	mmu03013(	PF00514.29	GO:003450	nuclear	NP_001013	mmu:381686	
K11248	SH3GLB1	mmu04140(	PF03114.23	GO:000810	cytoplasmic	NP_001366	mmu:54673	
K11509	CENPQ		PF13094.11	GO:001562	nuclear	EDL23388.	mmu:83815	
K18080	TNS		PF08416.19	GO:004212	cytoplasmic	EDL04008.	mmu:209039	
K20283	GOLGA4		PF01465.25	GO:004863	cytoplasmic	NP_061218	mmu:54214	
K00432	gpx, btuE, l	mmu05022(	PF00255.25	GO:001649	lysosomal	NP_077160	mmu:67305	
K10696	BRE1		PF00097.31	GO:008009	nuclear	NP_758485	mmu:233900	
K23292	LNPK		PF10058.14	GO:000548	ER	NP_081409	mmu:69605	
K23740	SH3BGR		PF04908.21	GO:000573	cytoplasmic	NP_542126	mmu:73723	
K00710	GALNT	mmu00514(	PF00535.33	GO:003024	ER	NP_660253	mmu:212996	
K14545	RRP7	mmu03008(	PF12923.12	GO:003450	nuclear	BAE32099.	mmu:74778	
K08882	TRIM28, TIF1B		PF00643.29	GO:003315	nuclear	NP_035718	mmu:21849	
K04650	NCOR1, N	mmu05202(	PF15784.10	GO:003292	nuclear	NP_001239	mmu:20185	MYB

K22499	ARMC5	mmu04934(	PF24768.1(/	GO:004864	plasma mer	BAD21361.	mmu:233912	
K02960	RP-S16e, Rl	mmu05171(	PF00380.25	GO:003204	cytoplasmic	NP_001011	mmu:20055	
K12830	SF3B3, SAP	mmu03040(	PF10433.15	GO:000989	mitochondr	NP_001099	mmu:101943	
K02896	RP-L24e, R	mmu05171(	PF01246.26	GO:004322	ER	NP_941011	mmu:225215	
K06578	LU, CD239		PF13927.12	GO:000716	plasma mer	BAC30627.1	mmu:57278	
K05866	CDC25B	mmu04010(	PF06617.18	GO:000548	nuclear	NP_075606	mmu:12531	
K08133	COL14A	mmu04974(	PF00092.34	GO:004863	extracellulo	NP_851794	mmu:12818	
K23555	LMF1		PF06762.19	GO:001921	peroxisoma	NP_083900	mmu:76483	
K10876	RAD54L2		PF00176.29	GO:001707	nuclear	NP_109655	mmu:81000	
K09562	HSPBP1, Fl	mmu04141(	PF08609.16	GO:000989	cytoplasmic	NP_001347	mmu:66245	
K01672	CA	mmu00910(	PF00194.26	GO:000548	mitochondr	NP_851832	mmu:56078	
K03912	SERPIND1	mmu04610(	PF00079.25	GO:000759	extracellulo	NP_001317	mmu:15160	
K01887	RARS, argS	mmu00970(	PF00750.25	GO:001710	cytoplasmic	NP_080212	mmu:104458	
K24764	WDSUB1		PF00400.38(WD40:WD		cytoplasmic	NP_001366	mmu:72137	
K08491	STX17	mmu04140(	PF05739.25	GO:000810	cytoplasmic	NP_080619	mmu:67727	
RKY(Thioesterase superfamily mer			PF13279.12	GO:000557	extracellulo	NP_941009	mmu:223626	
N7I(DAZ associated protein 2)			PF11029.13	GO:003646	cytoplasmic	BAE26411.1	mmu:23994	
K00927	PGK, pgk	mmu01100(	PF00162.24	GO:004643	cytoplasmic	ABS83503.1	mmu:18655	
K05956	RABGGTB		PF00432.27	GO:004316	cytoplasmic	AAH03948.	mmu:19352	
K04454	MEF2C	mmu04010(	PF00319.23	GO:005125	nuclear	KAI253819	mmu:17260	SRF
K15187	MLLT1_3, 1	mmu03250(	PF03366.22	GO:008009	nuclear	NP_081602	mmu:70122	
K06238	COL6A	mmu05165(	PF00092.34	GO:000520	extracellulo	NP_001161	mmu:665033	
K06124	PHOSPHO	mmu00564(	PF06888.18	GO:003563	mitochondr	NP_694744	mmu:237928	
K03237	EIF2S1	mmu05164(	PF07541.17	GO:003577	cytoplasmic	NP_062229	mmu:13665	
K14323	CASC3, ML	mmu03013(	PF09405.15	GO:005116	cytoplasmic	NP_619601	mmu:192160	
K02580	NFKB1	mmu05208(	PF00554.27	GO:000097	nuclear	NP_032715	mmu:18033	RHD
K16727	ARHGEF10		PF19056.6(	GO:000705	cytoplasmic	XP_036009	mmu:234094	
K11497	CENPC		PF15620.11	GO:000367	nuclear	NP_031709	mmu:12617	
K20221	IPO4, RAN	mmu03013(	PF13513.12	GO:003450	cytoplasmic	NP_077229	mmu:75751	
K01325	KLK1_2	mmu04614(	PF00089.32	GO:000425	lysosomal	SFW93234.1	mmu:13648	
K02608	ORC6	mmu04110(	PF21913.1((	GO:009030	nuclear	NP_062690	mmu:56452	
K00738	MGAT4A_	mmu00510(	PF04666.18	GO:000578	Golgi appa	NP_776295	mmu:269181	
XCW(receptor activity)			PF04664.18	GO:000573	cytoplasmic	BAE28751.1	mmu:72075	
K15728	LPIN	mmu04936(	PF08235.19	GO:004423	nuclear	BAC28406.1	mmu:64899	
K09173	RFX1_2_3		PF02257.21	GO:003015	nuclear	XP_030106	mmu:19726	RFX
K12616	EDC4	mmu03018(	PF16529.11	GO:011015	nuclear	NP_001288	mmu:234699	
KT5U(Sodium channel and clathrin			PF05686.18	GO:000578	ER	NP_997610	mmu:68304	
K00011	AKR1B	mmu00052(	PF00248.27	GO:000810	extracellulo	NP_765986	mmu:67861	
K10386	PPL		PF23160.2(S	GO:001989	cytoplasmic	NP_032935	mmu:19041	
K10650	TRIM11		PF13765.14	GO:008009	cytoplasmic	NP_444398	mmu:94091	
K17544	TBCK		PF00566.25	GO:000705	cytoplasmic	NP_001156	mmu:271981	
K18995	DHX29		PF24899.1(I	GO:002262	cytoplasmic	NP_766182	mmu:218629	
K13806	DAGL	mmu04723(	PF01764.32	GO:008009	ER	NP_659164	mmu:231871	

K14168	CTU1, NCS	mmu04122(	PF01171.26	GO:000209	mitochondr	XP_017177	mmu:233189	
K13124	MORG1		PF00400.38	GO:000962	cytoplasmic	XP_042133	mmu:67836	
K14948	PTBP2, NPTB		PF13893.12	GO:004212	nuclear	NP_001005	mmu:56195	
K17262	TBCB, CKAP1, ALF1		PF14560.12	GO:000367	cytoplasmic	NP_079824	mmu:66411	
K11579	ZWILCH		PF09817.14	GO:004584	cytoplasmic	EDL26055.	mmu:68014	
K14400	PCF11	mmu03015(	PF20844.2(I	GO:003277	nuclear	NP_083354	mmu:74737	
)31H(death inducer-obliterator 1)			PF07744.19	GO:003277	nuclear	NP_780760	mmu:23856	
K23679	PQLC1, SLC66A2		PF04193.19	GO:000686	Golgi appa	NP_080137	mmu:66943	
K11853	USP34		PF00443.35	GO:000417	Golgi appa	NP_001177	mmu:17847	
K09288	CCDC6, PT	mmu05200(	PF09755.14	GO:000585	cytoplasmic	NP_001104	mmu:76551	
ŚQVI(phosphodiesterase 4D interacting protein				GO:000573	cytoplasmic	AAY85303.1(protein phosphatase 2		
K07826	GNG2	mmu04724(	PF00631.27	GO:000557	cytoplasmic	NP_001005	mmu:14702	
K12822	RBM25, S1(	mmu03040(	PF01480.22	GO:008009	cytoplasmic	NP_001351	mmu:67039	
K17621	EYA3		PF00702.32	GO:200123	cytoplasmic	NP_997592	mmu:14050	
K12854	SNRNP200	mmu03040(	PF02889.21	GO:002260	nuclear	EDL28177.	mmu:320632	
K17930	SNX19		PF02194.20	GO:000554	plasma mer	NP_083150	mmu:102607	
K16777	NEURL4		PF07177.19	GO:003204	cytoplasmic	AAI58108.1	mmu:216860	
K23424	TMTC		PF07719.23	GO:000989	ER	NP_001103	mmu:237500	
K20068	REPS		PF12763.14	GO:009859	cytoplasmic	NP_001104	mmu:19707	
K07825	GNB3	mmu04724(	PF00400.38	GO:000988	cytoplasmic	NP_038558	mmu:14695	
K11284	TSPYL1		PF00956.23	GO:003472	nuclear	NP_033459	mmu:22110	
K19739	CALHM2		PF14798.11	GO:000526	Golgi appa	XP_021007	mmu:72691	
K20799	FAM175B, ABRO1		PF21125.3(	GO:014003	cytoplasmic	Q3TCJ1.1(	mmu:109359	
K22544	SAMHD1	mmu03250(	PF07647.24	GO:004643	nuclear	BAE41959.	mmu:56045	
TAG(-)				GO:002260	cytoplasmic	NP_803422	mmu:52570	
K12610	DCP1A	mmu03018(	PF06058.19	GO:000810	cytoplasmic	Q91YD3.1(	mmu:75901	
K10474	KBTBD6_7		PF00651.37	GO:199023	cytoplasmic	NP_001019	mmu:21125	ZBTB
K20463	OSBPL3_6_7, ORP3_6_		PF01237.23	GO:003609	cytoplasmic	NP_001297	mmu:71240	
K08468	GPRC5A		PF00003.28	GO:003367	plasma mer	NP_852109	mmu:232431	
K04648	DCTN1	mmu05022(	PF12455.13	GO:003111	cytoplasmic	NP_001185	mmu:13191	
K07442	TRM61, GCD14		PF08704.16	GO:000815	nuclear	NP_001093	mmu:328162	
K20133	USP6NL		PF00566.25	GO:003304	cytoplasmic	NP_001074	mmu:98910	
K01875	SARS, serS	mmu00970(	PF00587.30	GO:000643	mitochondr	NP_076126	mmu:71984	
ŚS5V(Transmembrane emp24 prote			PF13897.11(GOLD_2:C		cytoplasmic	AAI50861.1	mmu:382620	
K26109	NRDE2		PF08424.15	GO:003238	nuclear	NP_001277	mmu:217827	
K10138	IGFBP3	mmu04935(	PF00086.23	GO:001922	mitochondr	NP_032369	mmu:16009	
K17524	CHI3L3_4		PF00704.34	GO:000695	lysosomal	NP_034022	mmu:12655	
K06821	PLXNB	mmu04360(	PF08337.17	GO:001922	ER	NP_001152	mmu:140570	
K21799	PURB		PF04845.19	GO:007110	nuclear	NP_035351	mmu:19291	TF_others
K24853	ZNF462		PF23225.1(	GO:008009	nuclear	NP_766455	mmu:242466	
K11584	PPP2R5	mmu05165(	PF01603.26	GO:009868	cytoplasmic	NP_659129	mmu:226849	
K19862	NCK2, GR	mmu04012(	PF00018.34	GO:200123	cytoplasmic	NP_035009	mmu:17974	
K00889	PIP5K	mmu05231(	PF01504.24	GO:000810	cytoplasmic	NP_032873	mmu:18720	

K09255	ANK2, AN	mmu05205(	PF00023.36	GO:000810	nuclear	Q8C8R3.2(I	mmu:109676	
YCSG(transmembrane protein 50A)				GO:003251	cytoplasmic	PNI55393.1	mmu:77975	
ZH8Y(Cold shock domain containin			PF23456.1((	GO:003577	cytoplasmic	XP_006501	mmu:22966	CSD
K21068	MICALL2,	mmu04530(	PF12130.13	GO:003243	cytoplasmic	NP_777275	mmu:231830	
K05626	DRPLA		PF03154.20	GO:008009	cytoplasmic	NP_031907	mmu:13498	
SII(chromosome 12 open reading fr			PF14974.11	GO:001481	cytoplasmic	NP_001185	mmu:14790	
K22038	LRRC8		PF12534.13	GO:190379	ER	NP_001028	mmu:433926	
YUJQ(Ubiquitin-like 7 (Bone marro			PF00240.29	GO:000367	cytoplasmic	XP_021509	mmu:69459	
K20031	ZDHHC6		PF01529.25	GO:000810	ER	NP_001028	mmu:66980	
K10405	KIFC1, ncd	mmu04814(	PF00225.29	GO:009951	cytoplasmic	NP_444403	mmu:16580	
K13291	TUT		PF19088.6(	GO:008009	nuclear	NP_705766	mmu:214290	
K17081	PHB2	mmu04137(	PF01145.30	GO:003033	mitochondr	NP_001138	mmu:12034	
K15361	WDR48, U	mmu03460(	PF11816.13	GO:008009	nuclear	EDL09200.	mmu:67561	
K17607	TIPRL, TIP41		PF04176.18	GO:001094	cytoplasmic	NP_663488	mmu:226591	
K15105	SLC25A12_13, AGC		PF00153.32	GO:001922	cytoplasmic	NP_766024	mmu:78830	
K18622	ADD		PF00596.26	GO:004590	cytoplasmic	NP_001157	mmu:27360	
K10790	TREX1	mmu04623(	Cytosolic D	GO:001707	cytoplasmic	NP_001012	mmu:22040	
K06990	MEMO1		PF01875.22	GO:000367	mitochondr	BAE35184.	mmu:76890	
K24246	EMILIN		PF07546.18	GO:003411	extracellulo	BAE33288.	mmu:100952	
K08819	CDK12_13		PF00069.31	GO:200073	nuclear	NP_001074	mmu:69562	
K06072	DOHH		PF13646.12	GO:000449	cytoplasmic	NP_598725	mmu:102115	
K03175	TRAF6	mmu05200(	PF21355.3(	GO:003563	cytoplasmic	NP_001290	mmu:22034	
K02739	PSMB7	mmu05020(	PF00227.31	GO:000417	cytoplasmic	NP_035317	mmu:19177	
K14165	K14165		PF00782.25	GO:000573	cytoplasmic	NP_780327	mmu:67446	
K09849	TRAF5	mmu05170(	PF21355.3(	GO:001922	cytoplasmic	NP_035763	mmu:22033	
ZSFW(Sperm specific antigen 2)			PF14723.11	GO:000557	cytoplasmic	XP_036018	mmu:70599	
K15690	RC3H		PF21206.3(I	GO:003577	cytoplasmic	NP_001277	mmu:319817	
K20168	TBC1D15	mmu04137(	PF00566.25	GO:004322	cytoplasmic	AAY53531.	mmu:66687	
K06058	DTX	mmu04330(	PF18102.6(I	GO:000367	cytoplasmic	NP_001355	mmu:80904	
K12615	EDC3	mmu03018(	PF16598.10	GO:002260	cytoplasmic	NP_001397	mmu:353190	
K21442	ANKRD54		PF00023.36	GO:005116	nuclear	BAE21840.	mmu:223690	
K18460	XPO7, EXI	mmu03013(	PF03810.25	GO:005116	cytoplasmic	NP_075532	mmu:65246	
K06267	HMMR, RI	mmu04512(	PF15905.10	GO:003802	cytoplasmic	NP_038580	mmu:15366	
Component of the coat protein comp			PF00096.32	GO:004323	nuclear	NP_001347	mmu:32897	zf-C2H2
K00472	P4HA	mmu00330(	PF23558.1(	GO:000578	ER	EDL33547.	mmu:18452	
K06236	COL1A	mmu05165(	PF01391.23	GO:000367	extracellulo	AAH12438.	mmu:12843	
K02976	RP-S26e, RI	mmu05171(	PF01283.25	GO:000578	cytoplasmic	NP_001015	mmu:27370	
K14139	IRGM, LRC	mmu04621(	PF05049.19	GO:000810	cytoplasmic	NP_062313	mmu:54396	
K24031	TMPO		PF08198.17	GO:001602	nuclear	NP_001073	mmu:21917	
K05870	CREB1	mmu04151(	PF02173.22	GO:003250	nuclear	NP_598589	mmu:12912	TF_bZIP
K15259	PARP7_11_12		PF00644.27	GO:007021	plasma mer	BAE33780.	mmu:243771	
K23625	ATXN2_2L	mmu05014(	PF14438.12	GO:000548	cytoplasmic	XP_006507	mmu:233871	
K10668	RNF138		PF13923.12	GO:000367	cytoplasmic	NP_001289	mmu:56515	

K13365	SLC9A3R1,	mmu05165(	PF09007.17	GO:200123	cytoplasmic	NP_036160	mmu:26941
K18644	EIF4EBP2	mmu04213(	PF05456.16	GO:000548	mitochondr	NP_034254	mmu:13688
K04726	BID	mmu05022(	PF06393.16	GO:200123	cytoplasmic	NP_031570	mmu:12122
K13151	SNUPN, RI	mmu03013(	PF21974.1(	GO:003450	nuclear	NP_848461	mmu:66069
XUP0(S100P binding protein)			PF15427.11	GO:001660	nuclear	NP_001366	mmu:74648
K03456	PPP2R1	mmu05165(	PF02985.28	GO:001920	cytoplasmic	NP_082890	mmu:73699
XR4F(canopy 1 homolog (zebrafish)			PF09787.14	GO:000030	nuclear	NP_001185	mmu:27277
K21032	B4GAT1	mmu00515(	PF13896.12	GO:003003	ER	NP_780592	mmu:108902
M3H(PRKC apoptosis WT1 regulator)				GO:004213	cytoplasmic	NP_473397	mmu:114774
K22827	MICU1		PF00036.39	GO:199024	mitochondr	NP_659071	mmu:216001
K10589	UBE3C	mmu04120(	PF00632.30	GO:003551	cytoplasmic	EDL37246.	mmu:100763
K14317	NUP214, C	mmu03013(	PF16755.11	GO:003450	nuclear	NP_758472	mmu:227720
ZM6W(IGc1); ENOG4111XUQ(imn			PF07654.21	GO:000548	cytoplasmic	BAB22422.1(unnamed protein prod	
K16456	CEP55		PF12180.13	GO:009859	cytoplasmic	NP_001157	mmu:74107
K06105	TJP4, PILT	mmu04530(	PF15453.11	GO:004322	cytoplasmic	XP_011244	mmu:74094
K27396	SMG		PF00536.37	GO:000548	cytoplasmic	NP_001032	mmu:74480
K02435	gatC, GAT(	mmu00970(	PF02686.20	GO:000645	mitochondr	NP_083921	mmu:384281
K21052	TMEM5, R	mmu00515(	PF24785.1(	GO:004322	peroxisoma	NP_001351	mmu:216395
K02925	RP-L3e, RP	mmu05171(	PF00297.28	GO:002262	cytoplasmic	NP_038790	mmu:27367
K00277	LOX		PF01186.22	GO:001922	ER	NP_001273	mmu:16948
K02983	RP-S30e, RI	mmu05171(	PF04758.19	GO:002262	cytoplasmic	XP_012423	mmu:14109
mmunoglobulin kappa variable); EI			PF10382.14	GO:001922	cytoplasmic	XP_017175	mmu:71643
K23820	SUSD2		PF03782.22	GO:011016	lysosomal	NP_082166	mmu:71733
K26108	RGCC, RG	mmu04810(	PF15151.11	GO:001922	cytoplasmic	NP_079703	mmu:66214
K15143	MED30	mmu04919(	PF11315.13	GO:008009	nuclear	NP_081488	mmu:69790
K19656	IFT122		PF23381.1(	GO:000858	cytoplasmic	XP_006506	mmu:81896
F2J(chromosome 1 open reading fr			PF15797.10	GO:000557	cytoplasmic	NP_780358	mmu:69551
K08519	STXBP6		PF15277.12	GO:000554	cytoplasmic	KAF648773	mmu:217517
K26537	UXT		PF02996.22	GO:000989	cytoplasmic	NP_001405	mmu:22294
K11979	UBR7		PF02207.25	GO:000367	nuclear	BAE33345.	mmu:66622
K24229	CFAP298			GO:002260	cytoplasmic	XP_036015	mmu:68001
K08220	FLVCR, SLC49A1_2		PF07690.21	GO:003250	cytoplasmic	NP_932142	mmu:226844
K10132	BBC3, PUM	mmu05162(	PF15826.10	GO:200123	cytoplasmic	NP_001369	mmu:170770
K12586	RRP43, EX	mmu03018(	PF01138.27	GO:007104	nuclear	NP_081424	mmu:69639
5HM(Cathelicidin)			PF00666.23	GO:000548	extracellula	NP_032720	mmu:18054
K06455	CD5	mmu04640(	PF00530.23	GO:000989	plasma mer	NP_031676	mmu:12507
K23790	GSTP	mmu00980(	PF14497.11	GO:200123	cytoplasmic	NP_038569	mmu:14870
K25572	EHBP1		PF10358.15(NT-C2:N-t		cytoplasmic	XP_036012	mmu:216565
K19527	VPS13D		PF12624.14	GO:000989	cytoplasmic	XP_011248	mmu:230895
K13444	SUMF1, FG	mmu04142(	PF03781.22	GO:000578	ER	NP_666049	mmu:58911
XX42(kiaa1549)			PF12877.12	GO:000557	cytoplasmic	NP_918950	mmu:330286
J00(Akirin 2)				GO:005116	cytoplasmic	NP_001007	mmu:433693
K13280	SEC11, sipV	mmu03060(	PF00717.28	GO:000425	cytoplasmic	EDL06965.	mmu:56529

K17500	ILKAP		PF00481.26	GO:000548	nuclear	NP_075832	mmu:67444	
K01557	FAHD1	mmu00350(	PF01557.24	GO:001877	cytoplasmic	NP_075969	mmu:68636	
K17537	SGK223		PF00069.31	GO:002260	cytoplasmic	NP_001413	mmu:244418	
K23194	GATAD2	mmu03082(	PF16563.10	GO:000989	nuclear	NP_001366	mmu:22954	zf-GATA
K13505	VAMP3	mmu04130(	PF00957.27	GO:000810	cytoplasmic	NP_033524	mmu:22319	
K15116	SLC25A33_36, RIM2		PF00153.32	GO:001593	cytoplasmic	NP_620095	mmu:192287	
ΥE53(Family with sequence similari			PF16046.10	GO:001660	cytoplasmic	NP_663528	mmu:230789	
.FVP(tumor protein)			PF00838.24	GO:200123	cytoplasmic	NP_033455	mmu:22070	
K17414	MRPS36		PF10937.13	GO:001975	mitochondr	NP_079645	mmu:66128	
Y3KP(kiaa0101)			PF15715.11	GO:000367	mitochondr	NP_080791	mmu:68026	
K20791	NAA10_11, ARD1_2		PF00583.31	GO:003124	cytoplasmic	NP_001371	mmu:117903916	
K08804	STK17		PF00069.31	GO:000548	cytoplasmic	NP_598571	mmu:98267	
K01368	CTSS	mmu04612(	PF00112.29	GO:200123	lysosomal	NP_001254	mmu:13040	
K06778	PTPRS	mmu04514(	PF00102.32	GO:005080	extracellula	NP_035348	mmu:19280	
K25949	CCDC50		PF15295.11	GO:000573	cytoplasmic	XP_011244	mmu:67501	
.N81(positive regulation of triglycer			PF09771.14	GO:003450	peroxisoma	NP_001029	mmu:382030	
K00688	PYG, glgP	mmu01100(	PF00343.26	GO:003017	cytoplasmic	BAE40840.	mmu:110078	
XNNE(SPRY domain containing 7)			PF00622.36(SPRY:SPRY		cytoplasmic	NP_001009	mmu:66674	
K23611	SMCR8	mmu04140(	PF11704.13	GO:005134	cytoplasmic	NP_001078	mmu:237782	
K10267	FBXW12S		PF12937.12	GO:000573	cytoplasmic	EDL41092.	mmu:109082	
K14050	RABGGTA		PF07711.17	GO:000815	cytoplasmic	BAE27529.	mmu:56187	
K11416	SIRT6, SIR	mmu04714(	PF02146.23	GO:001707	cytoplasmic	NP_001365	mmu:50721	
K16142	HP		PF00089.32	GO:000425	ER	NP_059066	mmu:15439	
K26250	HPF1		PF10228.14	GO:004239	nuclear	NP_082575	mmu:72612	
K14207	SLC38A2, S	mmu04724(	PF01490.24	GO:008009	cytoplasmic	NP_780330	mmu:67760	
K05407	PF4, CXCL	mmu04062(	PF00048.25	GO:200123	extracellula	NP_064316	mmu:56744	
K06553	VPREB, CD179a		PF07686.23	GO:000548	ER	DBA12389.	mmu:22363	
K25769	RALGAPB	mmu04014(	PF20412.3(I	GO:004858	cytoplasmic	NP_001278	mmu:228850	
K20792	NAA15_16			GO:003124	cytoplasmic	XP_029324	mmu:66897	
K06521	SEMA4, CI	mmu04360(	PF01403.25	GO:001922	plasma mer	NP_001268	mmu:20354	
K06070	PKD	mmu05208(	PF00069.31	GO:003563	nuclear	NP_001164	mmu:75292	
K23165	UQCC3		PF15141.11	GO:006102	ER	NP_001153	mmu:107197	
.5H2(chromosome 11 open reading frame 34)				GO:000989	extracellula	NP_083915	mmu:76509	
K24172	CAND2, TIP120B		PF08623.16	GO:002260	cytoplasmic	NP_080234	mmu:67088	
K04512	DAAM	mmu04310(	PF02181.28	GO:003243	cytoplasmic	BAC97995.	mmu:208846	
K02989	RP-S5e, RP	mmu05171(	PF00177.27	GO:000645	cytoplasmic	NP_001404	mmu:20103	
K04172	FPR1	mmu04613(	PF00001.26	GO:000512	cytoplasmic	NP_038549	mmu:14293	
K00573	E2.1.1.77, pcm		PF01135.25	GO:009859	ER	XP_028620	mmu:18537	
K21914	KCTD2_5_17		PF02214.28	GO:002260	cytoplasmic	CAH67764(	mmu:70382	
K22763	DESI2, PPPDE1		PF05903.20	GO:000573	cytoplasmic	VCX38181.	mmu:78825	
K15103	UCP2_3, SLC25A8_9		PF00153.32	GO:000663	cytoplasmic	NP_033490	mmu:22229	
K11254	H4	mmu05322(	PF15511.12	GO:003472	nuclear	XP_006162	mmu:319159	
K25872	TPPP3		PF05517.18	GO:003250	cytoplasmic	AAI16623.1	mmu:67971	

KY2Z(Myb SANT-like DNA-bindin			PF13873.12(Myb_DNA		cytoplasmic	NP_001139	mmu:66665	
K01367	CAPN1	mmu05022(	PF00648.27	GO:004858	cytoplasmic	NP_001103	mmu:12333	
). cerevisiae)			PF04061.19	GO:000578	ER	NP_079937	mmu:66612	
K27705	DRG2, RBG2		PF16897.10	GO:004316	cytoplasmic	NP_001385	mmu:13495	
K20731	TTR	mmu04918(	PF00576.27	GO:004240	ER	NP_038725	mmu:22139	
K07532	ARHGEF1	mmu05163(	PF09128.16	GO:000548	cytoplasmic	NP_081420	mmu:69632	
K04943	KCNN2, K	mmu04726(	PF03530.19	GO:000526	mitochondr	BAE28732.	mmu:140492	
K00472	P4HA	mmu00330(	PF23558.1(	GO:000578	ER	NP_796135	mmu:320452	
K16463	CEP170		PF15308.11	GO:009908	nuclear	NP_001093	mmu:545389	
K09364	POU2F, O	mmu05168(	PF19536.4(I	GO:000578	nuclear	NP_035267	mmu:18986	Homeobox
26SF(open reading frame)			PF17826.7(DUF5588:Fo		cytoplasmic	NP_083694	mmu:75758	
K01379	CTSD	mmu04915(	PF00026.29	GO:004877	lysosomal	BAE31380.	mmu:13033	
XP3C(dystrobrevin)			PF09068.17	GO:009859	nuclear	XP_028730	mmu:13528	
K20724	TMEM33	mmu03013(	PF03661.18	GO:001602	ER	NP_084384	mmu:67878	
K03671	TXN, trxA	mmu04621(	PF00085.26	GO:003315	cytoplasmic	NP_035790	mmu:22166	
K12398	AP3M	mmu04142(	PF00928.26	GO:009951	cytoplasmic	NP_001116	mmu:64933	
K05361	GPX4	mmu04216(	PF00255.25	GO:004428	extracellulo	NP_032188	mmu:625249	
K19828	MTG1		PF01926.29	GO:000016	mitochondr	NP_955005	mmu:212508	
K01344	PROC	mmu04610(	PF00089.32	GO:000425	plasma mer	NP_032960	mmu:19123	
K09193	SP3		PF00096.32	GO:008009	nuclear	NP_001018	mmu:20687	zf-C2H2
K03257	EIF4A		PF00270.35	GO:003451	cytoplasmic	NP_001008	mmu:13682	
K17918	SNX3_12	mmu04144(	PF00787.29	GO:003315	cytoplasmic	O70492.3(R	mmu:54198	
K20068	REPS		PF12763.14	GO:009859	cytoplasmic	NP_839987	mmu:194590	
K10364	CAPZA	mmu04144(	PF01267.22	GO:001602	cytoplasmic	BAE30242.	mmu:12343	
K15261	PARP10_14_15		PF01661.27	GO:001922	cytoplasmic	AAI38875.1	mmu:547253	
K24755	WDR76, CMR1		PF24814.1(	GO:000367	nuclear	NP_001356	mmu:241627	
Gasdermin C)			PF00106.31	GO:004323	peroxisoma	BAE23126.	mmu:72479	
SH7(Chromosome 12 open reading			PF15475.11	GO:000557	cytoplasmic	NP_001013	mmu:103266	
K23612	WASL	mmu05100(	PF00568.29	GO:009951	cytoplasmic	NP_082735	mmu:73178	
K25468	SCGB2B		PF09252.15	GO:000557	extracellulo	AAP44466.	mmu:233099	
K14358	SCRN		PF03577.20	GO:004423	cytoplasmic	NP_083298	mmu:74616	
XQVU(c-type lectin domain family)			PF14050.11	GO:002260	cytoplasmic	NP_776109	mmu:209586	
XY8Z(CDV3 homolog (mouse))			PF15359.11	GO:000573	nuclear	NP_787027	mmu:321022	
K19368	ZFYVE27	mmu04144(	PF01363.27	GO:000810	cytoplasmic	NP_796293	mmu:319740	
K02993	RP-S7e, RP	mmu05171(	PF01251.23	GO:200123	cytoplasmic	NP_001002	mmu:20115	
K05754	ARPC5	mmu04530(	PF04699.19	GO:003304	cytoplasmic	NP_083085	mmu:74192	
K23858	PLEKHF		PF01363.27	GO:000989	cytoplasmic	NP_077724	mmu:72287	
XR07(ecdysoneless homolog (Drosop			PF07093.16	GO:008009	cytoplasmic	BAC31508.	mmu:70601	
K03650	mnmE, trmE, MSS1		PF12631.12	GO:003563	cytoplasmic	NP_115933	mmu:70359	
K15693	RNF26		PF13920.12	GO:000988	ER	NP_717095	mmu:213211	
K26051	SPATA5L1		PF00004.35	GO:014003	cytoplasmic	NP_001028	mmu:214616	
K14304	NUP85	mmu03013(	PF07575.19	GO:005116	cytoplasmic	NP_001002	mmu:445007	
K08010	NCF2, P67I	mmu05208(	PF00018.34	GO:000690	cytoplasmic	NP_001407	mmu:17970	

K11804	DCAF8		PF00400.38	GO:000815	cytoplasmic	NP_001344	mmu:98193	
K08597	SENP8, NEDP1, DEN		PF02902.25	GO:004423	cytoplasmic	NP_001165	mmu:71599	
K24880	ZHX2		PF00046.35	GO:000582	nuclear	BAE34139.	mmu:38760	Homeobox
K09045	ATF7, ATFA		PF00170.26	GO:008009	nuclear	EDL03962.	mmu:22392	TF_bZIP
K02130	ATPeF0F, /	mmu04714(	PF10206.14	GO:004423	peroxisoma	NP_065607	mmu:57423	
:hromosome 4 open reading frame 3			PF00012.26	GO:000645	cytoplasmic	NP_056580	mmu:50497	
K23931	FAM83H		PF07894.18	GO:190248	nuclear	XP_031202.	mmu:105732	
K16544	CEP95		PF19016.5(I	GO:000573	cytoplasmic	NP_001160	mmu:320162	
K23957	FMNL	mmu04820(	PF02181.28	GO:002260	cytoplasmic	BAC98283.	mmu:22379	
K12896	SRSF7, SFR	mmu05014(	PF00076.28	GO:001593	cytoplasmic	XP_005390	mmu:225027	
K05702	AF6, MLLT	mmu04530(	PF00788.29	GO:000810	nuclear	NP_034936	mmu:17356	
K06846	LY6D_E_F_G6_H		PF00021.27	GO:000989	extracellulo	NP_001238	mmu:17067	
K02155	ATPeV0C,	mmu05165(	PF00137.26	GO:001250	cytoplasmic	EDM03811	mmu:11984	
K27470	PAIP2		PF07145.20	GO:000989	cytoplasmic	BAE39611.	mmu:67869	
K16197	YWHAB_Q	mmu04114(	PF00244.26	GO:000449	cytoplasmic	EDL34013.	mmu:22630	
K19006	PLCH	mmu00562(	PF00388.25	GO:000665	nuclear	XP_029331	mmu:269437	
.MBP(poly(A) binding protein inter			PF01336.31	GO:000548	nuclear	BAE39608.	mmu:109019	
K10352	MYH9s	mmu04530(	PF01576.24	GO:009951	cytoplasmic	NP_001389	mmu:17880	
K15603	TCF4_12		PF00010.31	GO:004322	nuclear	XP_006525	mmu:21413	bHLH
K02353	DVL	mmu05165(	PF12316.13	GO:004867	cytoplasmic	NP_034221	mmu:13542	
K16550	RPGRIP1L		PF11618.14	GO:000858	cytoplasmic	NP_775607	mmu:244585	
XTD1(Glutamine and serine rich 1)			PF13926.11	(DUF4211:I	nuclear	NP_001116	mmu:99003	
K22459	P3H2		PF23557.1(	GO:000578	ER	NP_775555	mmu:210530	
K15303	AKR7	mmu00980(	PF00248.27	GO:000403	mitochondr	NP_079613	mmu:110198	
K11642	CHD3, MI2	mmu03082(	PF00176.29	GO:200073	nuclear	NP_666131	mmu:216848	
K18465	MRT43, SW	mmu04144(	PF14745.11	GO:005112	cytoplasmic	NP_001028	mmu:319277	
K23886	CISD3		PF09360.15	GO:004322	mitochondr	NP_001078	mmu:217149	
K14011	UBXN6, UI	mmu04141(	PF09409.15	GO:003251	cytoplasmic	NP_001366	mmu:66530	
K14284	NXF, TAP,	mmu05164(	PF24048.1(I	GO:000548	nuclear	BAC36922.	mmu:53319	
K01365	CTSL	mmu05323(	PF00112.29	GO:200123	lysosomal	NP_034114	mmu:13039	
K12179	COPS6, CSN6		PF13012.11	GO:000367	cytoplasmic	EFB23470.1	mmu:26893	
K20288	COG1		PF08700.17	GO:000810	cytoplasmic	BAE37141.	mmu:16834	
K12488	ASAP	mmu04666(	PF01412.23	GO:000810	cytoplasmic	NP_034156	mmu:13196	
K05854	LYN	mmu04664(	PF07714.23	GO:001922	cytoplasmic	NP_034877	mmu:17096	
K11314	TADA2A, ADA2		PF22941.1(	GO:003124	nuclear	BAE25000.	mmu:21703	MYB
K04620	GPRC5C			GO:003802	cytoplasmic	CAT00725.	mmu:70355	
K13937	H6PD	mmu00030(	PF01182.26	GO:000578	ER	NP_775547	mmu:100198	
K20042	FCHO		PF10291.14	GO:009859	cytoplasmic	NP_766179	mmu:218503	
K19716	AUP1		PF02845.22	GO:000810	ER	NP_031543	mmu:11993	
K04705	STAM	mmu04144(	PF00790.25	GO:003251	cytoplasmic	NP_035614	mmu:20844	
K21880	MPP1		PF00625.27	GO:009885	cytoplasmic	NP_032647	mmu:17524	
K00286	proC	mmu00330(	PF14748.11	GO:001649	cytoplasmic	NP_001335	mmu:209027	
.VU9(Coiled-coil-helix-coiled-coil-h			PF16860.10	GO:001598	cytoplasmic	NP_079671	mmu:66170	

K11514	CDCA8		PF10512.14	GO:001922(	mitochondr	NP_080836	mmu:52276	
K26401	TOM1, TOM1L1_2		PF00790.25	GO:000810	cytoplasmic	NP_035752	mmu:21968	
K15031	MTERF		PF02536.19	GO:007110	mitochondr	NP_001013	mmu:545725	
K10860	ALKBH3, DEPC1		PF13532.12	GO:001670	nuclear	NP_001405	mmu:69113	
K06620	E2F3	mmu05200(	PF16421.10	GO:009057	nuclear	NP_001346	mmu:13557	E2F
K08658	RCE1, FAC	mmu00900(	PF02517.21	GO:000417	extracellulo	NP_075620	mmu:19671	
)7FG(organic solute carrier partner			PF10188.14	GO:000573	cytoplasmic	NP_001343	mmu:230751	
K10374	TPM2	mmu04260(	PF00261.25	GO:009951	cytoplasmic	NP_001099	mmu:22004	
K22766	FIGNL1		PF00004.35	GO:200123	nuclear	EDL40644.	mmu:60530	
K27045	CCZ1	mmu04137(	PF19031.6(I	GO:003204	cytoplasmic	NP_808350	mmu:231874	
K15979	SND1	mmu05203(	PF00565.23	GO:000989	mitochondr	BAE35415.	mmu:56463	
K11584	PPP2R5	mmu05165(	PF01603.26	GO:001921	cytoplasmic	NP_036153	mmu:26931	
K20309	TRAPPC12		PF07719.23	GO:004820	cytoplasmic	BAE28268.	mmu:217449	
K09520	DNAJB14		PF00226.36	GO:001602(	cytoplasmic	NP_001028	mmu:70604	
K07573	CSL4, EXO	mmu03018(	PF10447.15	GO:009030	nuclear	NP_079920	mmu:66583	
K01953	asnB, ASNS	mmu00250(	PF00733.27	GO:004428	peroxisoma	NP_001397	mmu:27053	
K08957	CSNK1A	mmu05165(	PF00069.31	GO:000548	cytoplasmic	XP_019589	mmu:93687	
;ortilin-related VPS10 domain conto			PF00307.36	GO:000699	cytoplasmic	NP_808444	mmu:276829	
K10044	RARRES2			GO:001973	extracellulo	NP_001334	mmu:71660	
K23460	CHM, CHML		PF22603.2(I	GO:004323	cytoplasmic	NP_061288	mmu:12662	
K25447	OLFM4		PF02191.22	GO:004858	ER	AAI41128.1	mmu:380924	
K18184	COX20	mmu04714(	PF12597.13	GO:003361	cytoplasmic	NP_079787	mmu:66359	
K05121	TEK, TIE2,	mmu04010(	PF07714.23	GO:003243	extracellulo	NP_038718	mmu:21687	
KPEY(Enoyl-CoA hydratase/isomer			PF00378.25	GO:000663	mitochondr	NP_077170	mmu:67856	
K13862	SLC4A11, BTR1		PF00955.27	GO:200073	cytoplasmic	NP_001074	mmu:269356	
K14712	SLC39A6, L	mmu05010(	PF02535.28	GO:000578	ER	NP_631882	mmu:106957	
K27273	BOD1		PF05205.17	GO:000815	cytoplasmic	AAI16436.1	mmu:69556	
K01597	MVD, mva	mmu00900(	PF18376.6(	GO:000578	cytoplasmic	NP_619597	mmu:192156	
K08130	TSKU		PF13855.12	GO:004858	extracellulo	NP_001019	mmu:244152	
K14560	IMP3	mmu03008(	PF00163.24	GO:003204	cytoplasmic	NP_598737	mmu:102462	
K13213	MATR3	mmu05014(	PF00076.28	GO:004316	nuclear	EDK97131.	mmu:17184	
K01881	PARS, proS	mmu00970(	PF00587.30	GO:000643	cytoplasmic	NP_001077	mmu:230577	
K27355	NEMP, TMEM194		PF10225.14	GO:000563	cytoplasmic	NP_001106	mmu:210035	
K06721	CLEC10A, CLECSF13,		PF03954.19	GO:000989	plasma mer	XP_006532	mmu:216864	
K19584	PRKX		PF00069.31	GO:000716	cytoplasmic	BAC29427.	mmu:19108	
K01869	LARS, leuS	mmu00970(	PF00133.28	GO:003563	mitochondr	AAI12413.1	mmu:102436	
K00857	tdk, TK	mmu00983(	PF01712.25	GO:003204	mitochondr	NP_066356	mmu:57813	
K12229	DAPP1, BA	mmu04662(	PF00169.35	GO:000573	cytoplasmic	NP_001297	mmu:26377	
K14007	SEC24	mmu04141(	PF04811.21	GO:000548	mitochondr	NP_780464	mmu:77371	
K01869	LARS, leuS	mmu00970(	PF22947.2(I	GO:001710	cytoplasmic	NP_598898	mmu:107045	
K05721	FGD2		PF00621.25	GO:009859	cytoplasmic	NP_038738	mmu:26382	
KPJ1(B-cell CLL lymphoma)				GO:001602(	cytoplasmic	XP_006506	mmu:74253	
K10486	BTBD14		PF00651.37	GO:004212	cytoplasmic	NP_080064	mmu:66830	ZBTB

K12492	ARFGAP1	mmu04144(	PF01412.23	GO:000548	cytoplasmic	NP_665703	mmu:228998	
K09169	NFIB		PF00859.23	GO:008009	nuclear	CAM22761.	mmu:18028	MH1
K14015	NPLOC4, N	mmu04141(	PF05021.20	GO:000810	nuclear	EDL34744.	mmu:217365	
K07204	RAPTOR	mmu04136(	PF14538.11	GO:009008	cytoplasmic	NP_083174	mmu:74370	
.F22(ligand-activated sequence-spec			PF15065.11	GO:000810	ER	NP_064387	mmu:56700	NCU-G1
K25717	PIP		PF05326.17	GO:009859	extracellulo	NP_032869	mmu:18716	
K06664	PEX2, PXM	mmu04146(	PF04757.19	GO:003444	peroxisoma	NP_001156	mmu:19302	
K22803	SMC5		PF02463.25	GO:003506	cytoplasmic	EDL41599.	mmu:226026	
K03247	EIF3H	mmu05162(	PF19445.4(	GO:000548	cytoplasmic	BAE40079.1	mmu:68135	
)HW5(XIAP associated factor 1)			PF18608.7(	GO:005112	cytoplasmic	QIJ31371.1(	mmu:327959	
K03955	NDUFAB1	mmu05208(	PF00550.31	GO:000916	cytoplasmic	NP_001347	mmu:70316	
K09313	CUTL		PF08172.17	GO:004299	nuclear	EDL19310.	mmu:13047	
.10V(PRELI domain containing 1)			PF04707.20	GO:200123	mitochondr	NP_079872	mmu:66494	
K12185	VPS37	mmu04144(	PF07200.18	GO:000810	cytoplasmic	NP_001348	mmu:107305	
K11224	STAT5B	mmu04012(	PF02864.20	GO:003250	nuclear	NP_001107	mmu:20851	STAT
K11223	STAT5A	mmu04012(	PF02864.20	GO:003577	nuclear	NP_001349	mmu:20850	STAT
K05745	DIAPH3, D	mmu04810(	PF02181.28	GO:003243	cytoplasmic	AAH85191.	mmu:56419	
K16798	GLI2	mmu05200(	PF23561.1(	GO:003308	nuclear	NP_001074	mmu:14633	zf-C2H2
K01952	PFAS, purL	mmu00230(	PF13507.12	GO:000916	cytoplasmic	NP_001152	mmu:237823	
K20029	ZDHHC3_7_25		PF01529.25	GO:000579	Golgi appa	NP_001359	mmu:69035	
K18080	TNS		PF08416.19	GO:006024	cytoplasmic	XP_011242	mmu:319939	
K22038	LRRC8		PF12534.13	GO:190379	ER	NP_082451	mmu:72267	
K04799	FEN1, RAD	mmu03030(	PF00752.23	GO:001710	nuclear	BAC36544.1	mmu:14156	
K24776	RUFY3		PF02759.24	GO:003315	cytoplasmic	NP_001346	mmu:52822	
K00412	CYTB, petB	mmu05208(	PF00033.24	GO:001602	cytoplasmic	AHG32070.	mmu:17711	
K06275	PARV	mmu04510(	PF00307.36	GO:002260	cytoplasmic	XP_006508	mmu:57342	
K15365	RMI2	mmu03460(	PF16100.11	GO:003304	mitochondr	NP_001156	mmu:223970	
KSUM(NCK-associated protein 5-lil			PF15246.11	GO:004393	cytoplasmic	NP_001001	mmu:380969	
K07208	RHEB	mmu05165(	PF00071.28	GO:001922	cytoplasmic	NP_444305	mmu:19744	
K24779	ZFYVE21		PF16696.11(ZFYVE21_		cytoplasmic	XP_006516	mmu:68520	
K11883	NOB1	mmu03008(	PF17146.10	GO:003068	cytoplasmic	NP_080553	mmu:67619	
K25363	APOF		PF15148.11	GO:000573	extracellulo	NP_598757	mmu:28194	
K10488	ZBTB1		PF00651.37	GO:190545	cytoplasmic	BAE20921.1	mmu:26856	ZBTB
K00599	METTL6		PF08242.18(Methyltran		cytoplasmic	NP_001346	mmu:67011	
K25768	RALGAPA	mmu04014(	PF02145.21(Rap_GAP:l		cytoplasmic	XP_006516	mmu:56784	
K18419	DCGR8		PF00035.32	GO:190036	nuclear	NP_201581	mmu:94223	
K11413	SIRT3, SIR2	mmu05230(	PF02146.23	GO:009008	cytoplasmic	NP_001120	mmu:64384	
K01578	MLYCD	mmu00640(	PF05292.17	GO:003444	peroxisoma	NP_064350	mmu:56690	
K06963	TAN1, THUMPD1		PF02926.22	GO:001607	cytoplasmic	NP_663560	mmu:233802	
K24679	HHATL	mmu04340(	PF03062.24	GO:006026	ER	NP_083371	mmu:74770	
K14857	SPB1, FTSJ3		PF07780.17	GO:003068	nuclear	NP_079586	mmu:56095	
K03843	ALG2	mmu00510(	PF00534.26	GO:001003	cytoplasmic	NP_064382	mmu:56737	
K16780	SSNA1			GO:004867	cytoplasmic	XP_037349	mmu:68475	

K13278	ASPG		PF00710.25	GO:001975	mitochondr	NP_001074	mmu:104816	
K00799	GST, gst	mmu00980(	PF02798.26	GO:004428	cytoplasmic	NP_032208	mmu:14858	
K17710	PTCD1		PF13041.12	GO:003197	mitochondr	BAE29626.1	mmu:71799	
K24810	IBTK		PF00415.24	GO:001056	nuclear	NP_001074	mmu:10883	ZBTB
K12353	SMPD4	mmu00600(	PF14724.11	GO:000578	cytoplasmic	BAC98164.1	mmu:77626	
K03495	gidA, mnmG, MTO1		PF01134.28	GO:000209	mitochondr	NP_080934	mmu:68291	
K15122	SLC41A		PF01769.21	GO:001602	cytoplasmic	NP_796362	mmu:338365	
IJJ(zinc finger protein 318)				GO:000548	nuclear	NP_997554	mmu:57908	TF_others
K02603	ORC1	mmu04110(	PF09079.17	GO:004316	nuclear	BAD91663.	mmu:18392	
K15171	SUPT4H1, S	mmu03250(	PF06093.19	GO:008009	cytoplasmic	NP_001029	mmu:20922	
KSJQ(c-Maf inducing protein)			PF23066.1(I	GO:003250	mitochondr	AAI58119.1	mmu:74440	
K06262	GP1BB, CD	mmu04512(	PF01462.23	GO:000367	plasma mer	NP_001001	mmu:14724	
K25806	APPBP2		PF13374.12	GO:000810	cytoplasmic	NP_080101	mmu:66884	
K13917	RNGTT	mmu03015(	PF01331.24	GO:190136	nuclear	NP_036014	mmu:24018	
KPEV(endo-beta-n-acetylglucosami			PF05602.17	GO:000554	Golgi appa	NP_666159	mmu:218335	
K02737	PSMB5	mmu05020(	PF00227.31	GO:000417	cytoplasmic	NP_035316	mmu:19173	
K06487	ITGAV, CD	mmu05165(	PF20806.3(I	GO:003802	ER	NP_032428	mmu:16410	
K00541	NNMT	mmu00760(	PF01234.23	GO:000811	cytoplasmic	NP_001396	mmu:18113	
K06751	MHC1	mmu05165(	PF00129.23	GO:000989	ER	NP_034521	mmu:15007	
K11796	TRPC4AP		PF12463.13	GO:199023	mitochondr	NP_001156	mmu:56407	
K13239	ECI2, PECI	mmu00071(	PF00378.25	GO:003444	mitochondr	BAE42023.1	mmu:23986	
netallophosphoesterase)			PF00149.33	GO:001678	plasma mer	NP_001186	mmu:100504715	
K19007	AGPAT5	mmu00564(	PF01553.26	GO:001602	ER	BAE41558.1	mmu:52123	
K26127	SLF2		PF14816.11	GO:003418	nuclear	AAI51000.1	mmu:226151	
K26226	PPFIA		PF00536.37	GO:190547	cytoplasmic	NP_001182	mmu:233977	
KRNP(Fas apoptotic inhibitory mol			PF06905.18	GO:190204	cytoplasmic	NP_035940	mmu:23873	
K21444	PCBP3_4		PF00013.35	GO:000548	nuclear	KAF156702	mmu:59092	
K08857	NEK1_4_5		PF00069.31	GO:000024	cytoplasmic	NP_001280	mmu:18004	
K10456	KLHL19, K	mmu05208(	PF01344.31	GO:009951	cytoplasmic	NP_476493	mmu:50868	ZBTB
K14381	SQSTM1	mmu04137(	PF16577.11	GO:005116	cytoplasmic	NP_035148	mmu:18412	
K12879	THOC2	mmu03013(	PF11262.13	GO:005116	nuclear	NP_001028	mmu:331401	
K05631	AIP3, WWF	mmu05165(	PF00595.29	GO:000573	cytoplasmic	NP_001273	mmu:14924	
K18171	CMC1		PF08583.15	GO:004316	cytoplasmic	NP_080718	mmu:67899	
K18663	ASCC3		PF02889.21	GO:002262	nuclear	NP_932124	mmu:77987	
K01108	MTM1	mmu04070(	PF06602.19	GO:000004	cytoplasmic	NP_001157	mmu:17772	
K02578	NDST3	mmu00534(	PF12062.14	GO:001681	Golgi appa	NP_112463	mmu:83398	
25KF(Transmembrane emp24 protei			PF05049.19	GO:003563	cytoplasmic	AAI50901.1	mmu:225594	
K00820	glmS, GFPT	mmu01250(	PF01380.27	GO:003292	cytoplasmic	P47856.3(R	mmu:14583	
K00850	pfkA, PFK	mmu00052(	PF00365.25	GO:009008	cytoplasmic	XP_006520	mmu:18642	
K17087	TM9SF3		PF02990.21	GO:000810	Golgi appa	CAH69580	mmu:107358	
Z2X0(-)				GO:004322	cytoplasmic	ACM16078.	mmu:14349	
K01307	GGH	mmu01240(	PF07722.19	GO:004423	ER	AAI50899.1	mmu:14590	
K17333	NFATC3, N	mmu04010(	PF16179.11	GO:005116	nuclear	NP_001355	mmu:18021	RHD

K22938	SDCCAG3, ENTR1			GO:003288	cytoplasmic	NP_080839	mmu:68112	
K10877	RAD54B	mmu03440(	PF00176.29	GO:001561	nuclear	NP_001034	mmu:623474	
XJH(Coiled-coil domain containing 91)				GO:000810	cytoplasmic	AAH06734.	mmu:67015	
K21914	KCTD2_5_17		PF02214.28(BTB_2:BTE		cytoplasmic	NP_001276	mmu:72844	
K25112	TMX2		PF00085.26	GO:004885	ER	NP_080144	mmu:66958	
K19198	ING2		PF12998.12	GO:200123	nuclear	NP_075992	mmu:69260	
K20352	TMED10, ERV25		PF01105.29	GO:000810	ER	NP_081051	mmu:68581	
K17589	RB1CC1	mmu05022(	PF10377.14	GO:200123	cytoplasmic	NP_033956	mmu:12421	
K02295	CRY	mmu04710(	PF03441.19	GO:003292	mitochondr	NP_034093	mmu:12953	
YQ9P(Chromosome 15 open reading			PF17663.6(I	GO:000557	cytoplasmic	NP_083096	mmu:74211	
XPKT(chromosome 2 open reading			PF10155.14	GO:000989	mitochondr	NP_082319	mmu:52846	
K11660	MTA	mmu03082(	PF17226.7(	GO:200073	nuclear	NP_035972	mmu:23942	zf-GATA
K19013	STAB2, HA	mmu04148(	PF12947.13	GO:000989	cytoplasmic	BAJ51916.1	mmu:192188	
K06176	truD, PUS7		PF01142.23	GO:000945	nuclear	NP_766025	mmu:78895	
K05289	GAA1	mmu00563(	PF04114.19	GO:000830	extracellulo	NP_034461	mmu:14731	
K03754	EIF2B2	mmu05168(	PF01008.23	GO:000578	cytoplasmic	NP_663420	mmu:217715	
K01551	arsA, ASNA	mmu03060(	PF02374.21	GO:000810	cytoplasmic	NP_001093	mmu:56495	
K06619	ABL1	mmu04012(	PF07714.23	GO:003111	cytoplasmic	NP_001106	mmu:11350	
K00280	LOXL2_3_4		PF01186.22	GO:200032	extracellulo	NP_038614	mmu:16950	
XXTE(Shc SH2-domain binding pro			PF23762.1(	GO:004322	cytoplasmic	NP_035499	mmu:20419	
K18743	CAPRIN1, GPIAP1		PF12287.13	GO:003577	cytoplasmic	NP_001104	mmu:53872	
K11644	SIN3A	mmu05202(	PF16879.10	GO:003288	nuclear	NP_001103	mmu:20466	
K04499	RUVBL1, R	mmu04310(	PF06068.19	GO:008009	cytoplasmic	OBS67033.1	mmu:56505	
K13861	SLC4A10, NCBE		PF00955.27	GO:009859	cytoplasmic	AFP89960.1	mmu:94229	
K18343	OTULIN		PF16218.11	GO:008009	cytoplasmic	NP_001013	mmu:432940	
K03544	clpX, CLPX		PF07724.20	GO:003197	mitochondr	NP_001037	mmu:270166	
K22868	WDR34		PF00400.38	GO:004299	cytoplasmic	NP_001008	mmu:71820	
K12417	ACOT11		PF01852.25	GO:000663	mitochondr	NP_001334	mmu:329910	
K02883	RP-L18e, R	mmu05171(	PF17135.9(I	GO:001593	cytoplasmic	NP_033103	mmu:19899	
K14443	TOB	mmu03018(	PF07742.17	GO:004567	cytoplasmic	NP_065253	mmu:57259	
K06695	PSMC3IP		PF07106.19	GO:008009	cytoplasmic	NP_032975	mmu:19183	
K24209	SMPX		PF15355.11	GO:000573	cytoplasmic	NP_001239	mmu:66106	
K06274	VASP	mmu04530(	PF00568.29	GO:003514	cytoplasmic	NP_033525	mmu:22323	
K03940	NDUFS7	mmu05208(	PF01058.29	GO:000916	mitochondr	NP_083548	mmu:75406	
K06928	NTPCR	mmu01100(	PF03266.21	GO:003563	cytoplasmic	NP_079912	mmu:66566	
K16498	PCDHD1		PF00028.23	GO:004316	plasma mer	NP_001116	mmu:54216	
K11415	SIRT5, SIR	mmu00760(	PF02146.23	GO:001922	mitochondr	NP_849179	mmu:68346	
K03007	RPABC5, R	mmu03420(	PF01194.22	GO:003088	nuclear	NP_001016	mmu:66491	
K03130	TAF5	mmu03022(	PF00400.38	GO:008009	nuclear	EDL42024.	mmu:226182	
K04536	GNB1	mmu04724(	PF00400.38	GO:003042	cytoplasmic	XP_006903	mmu:14688	
K01966	PCCB, pccE	mmu00630(	PF01039.27	GO:000663	mitochondr	AAH55946.	mmu:66904	
K24383	PATZ1		PF00096.32	GO:008009	nuclear	XP_021066	mmu:56218	ZBTB
K08653	MBTPS1	mmu04141(	PF23090.1(	GO:000425	Golgi appa	ABK51402.	mmu:56453	

K08776	NPEPPS		PF01433.26	GO:000417	cytoplasmic	NP_032968	mmu:19155	
K01257	LNPEP	mmu04614(	PF01433.26	GO:000417	cytoplasmic	BAC29651.1	mmu:240028	
K27693	NDRG2		PF03096.20	GO:004212	cytoplasmic	NP_001347	mmu:29811	
K00232	E1.3.3.6, AC	mmu00640(	PF22924.1(	GO:003444	peroxisoma	NP_001258	mmu:11430	
K13719	OTU1, YOI	mmu04141(	PF24560.1(	GO:003315	cytoplasmic	AAH99948.	mmu:226418	
.G0F(Synaptogyrin 2)			PF01284.28	GO:000802	plasma mer	EDL34634.	mmu:20973	
An essential GTPase which binds G			PF01018.28	GO:003563	cytoplasmic	NP_001343	mmu:207704	
.FTI(transmembrane protein 164)			PF14808.11	GO:000557	Golgi appa	XP_006528	mmu:209497	
K22943	YIPF6		PF04893.22	GO:006057	cytoplasmic	NP_997516	mmu:77929	
K17046	DEK		PF08766.16	GO:004239	nuclear	NP_080176	mmu:110052	
K26600	TLCD3		PF03798.21	GO:000578	ER	NP_084254	mmu:68952	
K11801	DCAF11		PF00400.38	GO:199023	cytoplasmic	XP_016061	mmu:28199	
.F1D(kiaa1704)			PF12572.13(DUF3752:P		nuclear	NP_080453	mmu:67467	
K11307	MYST2, HBO1, KAT7		PF01853.24	GO:008009	nuclear	CAI970097	mmu:21712	zf-C2HC
K11649	SMARCC	mmu04714(	PF16496.10	GO:003506	nuclear	XP_006514	mmu:68094	MYB
K26089	ANKZF1, VMS1		PF18826.6(	GO:004423	nuclear	NP_080463	mmu:52231	
K16340	PHLPP	mmu04151(	PF13855.12	GO:009859	cytoplasmic	NP_001116	mmu:244650	
K16639	LACTB2		PF00753.33	GO:003197	mitochondr	NP_663356	mmu:212442	
K21412	ANKLE2, LEM4		PF24567.1(	GO:001602	ER	NP_001240	mmu:71782	
K09273	UBTF		PF14887.12	GO:001921	nuclear	NP_001037	mmu:21429	HMG
K09273	UBTF		PF14887.12	GO:001921	nuclear	NP_001349	mmu:21429	HMG
K03143	TFIIH3, G7	mmu03420(	PF03850.20	GO:003280	nuclear	NP_852075	mmu:209357	
K03252	EIF3C		PF05470.18	GO:002260	cytoplasmic	NP_666312	mmu:56347	
K01951	guaA, GMF	mmu01100(	PF00117.34	GO:000916	cytoplasmic	NP_001028	mmu:229363	
K06694	PSMD10		PF12796.13	GO:200123	cytoplasmic	NP_001157	mmu:53380	
K10224	FADS1	mmu01040(	PF00487.30	GO:000663	cytoplasmic	NP_666206	mmu:76267	
K01230	MAN1A_C	mmu04141(	PF01532.26	GO:000578	Golgi appa	NP_034893	mmu:17156	
K21398	SLC11A2, D	mmu04978(	PF01566.24	GO:000537	cytoplasmic	NP_032758	mmu:18174	
K14737	STEAP1	mmu04978(	PF01794.25	GO:004322	peroxisoma	BAE25828.1	mmu:70358	
K08461	ADGRA2, GPR124		PF13855.12	GO:009885	plasma mer	NP_473385	mmu:78560	
K11349	BRD1, BRPF2		PF00855.23	GO:008009	nuclear	AAH49267.	mmu:223770	
K14407	CSTF2, RN	mmu03015(	PF14327.11	GO:009030	cytoplasmic	ACF05700.1	mmu:108062	
K23445	CASQ2	mmu04260(	PF01216.23	GO:000578	cytoplasmic	AAI37669.1	mmu:12373	
K13150	COIL, CLN80		PF15862.10	GO:002260	nuclear	NP_057915	mmu:12812	
K26143	ICE1			GO:003315	cytoplasmic	NP_659086	mmu:218333	
20PF(chromosome 1 open reading fr			PF15990.10	GO:000557	Golgi appa	NP_084528	mmu:80284	
K14721	RPC5, POL	mmu04623(	PF04801.18	GO:003277	cytoplasmic	BAE35318.1	mmu:26939	
K17927	SNX15		PF00787.29	GO:000554	cytoplasmic	XP_011246	mmu:69024	
K26154	RELCH		PF02985.28	GO:000580	cytoplasmic	NP_775279	mmu:227446	
K13783	SLC37A1_2		PF07690.21	GO:001576	lysosomal	NP_064654	mmu:56857	
K24029	OGFOD1, TPA1		PF13661.12	GO:003646	cytoplasmic	BAC65814.1	mmu:270086	
K18190	FASTKD2		PF06743.20	GO:001604	mitochondr	NP_766010	mmu:75619	
K17918	SNX3_12	mmu04144(	PF00787.29	GO:000810	cytoplasmic	O70493.1(R	mmu:55988	

K04406	MAP4K3, (	mmu04010(	PF00069.31	GO:003563	cytoplasmic	XP_006524	mmu:225028	
K09651	RHBDD1, RHBDL4		PF01694.27	GO:000425	ER	NP_001116	mmu:76867	
atalyzes the phosphorylation of th			PF01121.26	GO:001593	cytoplasmic	NP_001404	mmu:68087	
K19476	IST1	mmu03272(	PF03398.19	GO:000810	cytoplasmic	AAH39052.	mmu:71955	
K00728	POMT, pm	mmu00515(	PF02366.24	GO:000988	extracellula	NP_660127	mmu:99011	
K19012	LYVE1		PF00193.23	GO:003802	plasma mer	NP_444477	mmu:114332	
K13291	TUT		PF19088.6(	GO:003577	nuclear	XP_017175	mmu:230594	
K10756	RFC3_5	mmu03030(	PF08542.16	GO:008009	mitochondr	BAE29258.	mmu:72151	
K04679	MADHIP, S	mmu04144(	PF11979.13	GO:000554	cytoplasmic	NP_001334	mmu:230597	
K06553	VPREB, CD179a		PF07686.23(V-set:Immu		extracellula	CAB46297.	mmu:22363	
N4C(tetratricopeptide repeat doma			PF07719.23(TPR_2:Tetr		nuclear	NP_001342	mmu:67515	
K21774	LIN37	mmu04218(	PF15306.11	GO:000989	nuclear	XP_011249	mmu:75660	
K15709	RNF187		PF15227.12	GO:004212	cytoplasmic	BAC29916.	mmu:108660	
K20002	SNAPIN, BLOC1S7		PF14712.11	GO:009951	cytoplasmic	BAE22381.	mmu:20615	
K05692	ACTB_G1	mmu04530(	PF00022.25	GO:000810	mitochondr	BAE29910.	mmu:11461	
K04862	CACNB1	mmu04010(	PF00625.27	GO:000810	cytoplasmic	NP_001152	mmu:12295	
K24370	ZNF148		PF13465.12	GO:002260	nuclear	BAE27286.	mmu:22661	zf-C2H2
K21073	ACTN2_3	mmu05412(	PF00435.27	GO:003288	cytoplasmic	NP_001163	mmu:11472	
K02885	RP-L19e, R	mmu05171(	PF01280.25	GO:002262	cytoplasmic	NP_001152	mmu:19921	
K11252	H2B	mmu05322(	PF00125.30	GO:003472	nuclear	NP_835586	mmu:319190	
K11789	DCAF1, VF	mmu05170(	PF24814.1(	GO:199024	nuclear	NP_001360	mmu:321006	
K11251	H2A	mmu05322(	PF16211.10	GO:000078	nuclear	NP_835585	mmu:621893	
K00814	GPT, ALT	mmu01100(	PF00155.27	GO:008009	cytoplasmic	NP_877957	mmu:76282	
PW3(OAF homolog (Drosophila))			PF14941.11(OAF_N:O		ER	NP_848759	mmu:102644	
K05757	ARPC1A_B	mmu04530(	PF23414.1(I	GO:000520	cytoplasmic	BAE42010.	mmu:11867	
K01230	MAN1A_C	mmu04141(	PF01532.26	GO:000810	Golgi appa	XP_006512	mmu:17155	
K15028	EIF3K		PF10075.15	GO:002260	cytoplasmic	NP_082935	mmu:73830	
K11407	HDAC6	mmu05014(	PF00850.24	GO:008009	cytoplasmic	BAE32376.	mmu:15185	
K27685	GLTP		PF08718.16	GO:000686	cytoplasmic	AAH53729.	mmu:56356	
K02830	HRAD1, R	mmu04218(	PF02144.21	GO:004423	cytoplasmic	NP_001276	mmu:19355	
K22592	AHDC1		PF15735.10	GO:003015	nuclear	NP_001391	mmu:230793	
K26671	GARRE1		PF15923.10	GO:190576	cytoplasmic	AAH60233.	mmu:233103	
K12399	AP3S	mmu04142(	PF01217.26	GO:009951	cytoplasmic	AAH60236.	mmu:11778	
K21951	HOXA11	mmu05202(	PF12045.13	GO:003510	nuclear	NP_034580	mmu:15396	Homeobox
K10436	MAPRE		PF03271.22	GO:003111	cytoplasmic	NP_001156	mmu:212307	
K18757	LARP1		PF21071.3(I	GO:003577	cytoplasmic	NP_082727	mmu:73158	
K08818	CDC2L		PF00069.31	GO:200123	nuclear	NP_001334	mmu:12537	
K13703	ABHD11		PF00561.26	GO:003197	cytoplasmic	NP_001177	mmu:68758	
K00717	FUT8	mmu05202(	PF19745.5(I	GO:000548	cytoplasmic	NP_001239	mmu:53618	
54J(major urinary protein)			PF00061.29	GO:009008	ER	P11589.1(R	mmu:100039008	
K26269	SFMBT	mmu03083(	PF02820.24	GO:000582	nuclear	NP_001356	mmu:353282	
K20210	RREB1		PF00096.32	GO:008009	nuclear	NP_001391	mmu:68750	zf-C2H2
K15106	SLC25A14_30		PF00153.32	GO:004322	cytoplasmic	NP_080508	mmu:67554	

XVD1(Growth arrest and DNA-dan			PF10147.14	GO:004212	mitochondr	NP_899202	mmu:102060	
K14720	SLC39A14,	mmu04216(Ferroptosis)		GO:001250	extracellula	NP_659057	mmu:213053	
K06065	NCOR2, SM	mmu04330(	PF15784.10	GO:008009	nuclear	XP_011239	mmu:20602	MYB
K12490	ARAP3	mmu04024(cAMP signa		GO:001602	cytoplasmic	XP_036016	mmu:106952	
SPU(chromosome 16 open reading			PF10571.15(UPF0547:U		nuclear	XP_021500	mmu:66714	
K17197	NKIRAS		PF00071.28	GO:001707	cytoplasmic	NP_001099	mmu:71966	
K02875	RP-L14e, R	mmu05171(	PF01929.23	GO:001593	cytoplasmic	XP_031200	mmu:67115	
K23002	RPAP3		PF00515.34	GO:003299	nuclear	NP_082279	mmu:71919	
K25947	C2CD6, ALS2CR11		PF15729.11(CTSRT:Cat		nuclear	NP_080876	mmu:73463	
K04734	NFKBIA	mmu05208(	PF00023.36	GO:005116	cytoplasmic	NP_035037	mmu:18035	
K01598	PPCDC, co	mmu01240(	PF02441.25	GO:001593	cytoplasmic	NP_001344	mmu:66812	
K06549	SIGLEC5, CD170		PF13927.12	GO:000032	plasma mer	NP_001257	mmu:233186	
K17308	ORM, AGP		PF00061.29	GO:000695	extracellula	NP_035146	mmu:18406	
K06250	SPP1, BNSI	mmu05165(	PF00865.23	GO:008009	extracellula	NP_001191	mmu:20750	
K12625	LSM6	mmu03018(	PF01423.28	GO:000548	cytoplasmic	NP_001087	mmu:78651	
WVY(PRELI domain containing 2)			PF04707.20	GO:000686	cytoplasmic	AAI45036.1	mmu:77619	
K12622	LSM3	mmu03018(	PF01423.28	GO:002260	nuclear	NP_001032	mmu:67678	
K20777	EXD2		PF01612.26	GO:001679	mitochondr	BAE27515.	mmu:97827	
K22869	WDR60		PF00400.38	GO:004859	nuclear	NP_666151	mmu:217935	
K15690	RC3H		PF21206.3(I	GO:004213	cytoplasmic	NP_001020	mmu:381305	
BND(Family with sequence similarity 89, mem				GO:004858	cytoplasmic	AAH13894.	mmu:17826	
K07939	ARF4	mmu04144(	PF00025.27	GO:000810	mitochondr	NP_031505	mmu:11843	
K24222	INPP5J_K	mmu00562(	PF22669.2(I	GO:004340	ER	AAH14725.	mmu:19062	
K25462	OPRPN, SMR3, PROL1			GO:005123	extracellula	NP_032670	mmu:17830	
K13374	AKR1C13		PF00248.27	GO:000403	cytoplasmic	AAH21937.	mmu:27384	
K15279	SLC35C1, FUCT1		PF03151.22	GO:001593	Golgi appa	NP_997597	mmu:228368	
K14027	HERPUD1,	mmu04141(	PF00240.29	GO:200123	cytoplasmic	NP_071726	mmu:64209	
K15164	MED13	mmu04919(	PF06333.17	GO:008009	cytoplasmic	NP_001334	mmu:76199	
K18586	COQ4		PF05019.18	GO:001989	mitochondr	NP_848808	mmu:227683	
K06842	SEMA6	mmu04360(	PF01403.25	GO:004858	ER	NP_001277	mmu:214968	
K24335	CRELD		PF07645.21	GO:000578	extracellula	NP_083996	mmu:76737	
K13127	RNF113A, CWC24		PF00642.30	GO:009030	nuclear	NP_079801	mmu:66381	
K11826	AP2M1	mmu05016(	PF00928.26	GO:000810	cytoplasmic	NP_001029	mmu:11773	
QTN(ADP-ribosylation factor-like			PF14949.11	GO:000565	cytoplasmic	NP_001020	mmu:212772	
K07863	RHOG	mmu05135(	PF00071.28	GO:008009	cytoplasmic	NP_001032	mmu:56212	
K07365	NCK1	mmu04012(	PF00018.34	GO:200123	cytoplasmic	NP_035008	mmu:17973	
K22647	MINDY3_4		PF13898.11	GO:004423	cytoplasmic	XP_006497	mmu:66960	
Y54T(Cell division cycle associated 3)				GO:004423	cytoplasmic	NP_038566	mmu:14793	
K11470	PCGF6, MI	mmu03083(	PF13923.12	GO:000989	cytoplasmic	NP_081930	mmu:71041	
K23484	DNM2	mmu04666(	PF01031.25	GO:000690	cytoplasmic	NP_001344	mmu:13430	
K10182	TBX15, TBX14		PF00907.27	GO:003250	nuclear	NP_033349	mmu:21384	T-box
K04288	S1PR1, ED	mmu04148(	PF00001.26	GO:001922	cytoplasmic	AFX82592.	mmu:13609	
K06588	ITGB5	mmu05165(	PF00362.24	GO:003003	plasma mer	NP_001139	mmu:16419	

K14843	PES1, NOP7		PF06732.16	GO:000810	nuclear	NP_075027	mmu:64934	
UG3(Serum response factor bindin			PF09073.15	GO:003068	nuclear	NP_080316	mmu:67222	
K24550	BCAS3		PF21034.3(I	GO:000573	cytoplasmic	XP_036012	mmu:192197	
K03158	TNFRSF1A	mmu05022(	PF00020.24	GO:000317	plasma mer	NP_035739	mmu:21937	
K03848	ALG6	mmu00510(	PF03155.21	GO:000578	extracellulc	NP_001074	mmu:320438	
K03767	PPIA	mmu03250(	PF00160.26	GO:200123	cytoplasmic	NP_032933	mmu:268373	
K01893	NARS, asnS	mmu00970(	PF00152.25	GO:000512	cytoplasmic	Q8BP47.2(F	mmu:70223	
K15174	PAF1		PF03985.18	GO:000810	nuclear	XP_052023	mmu:54624	
K05704	SRC	mmu05208(	PF07714.23	GO:005084	cytoplasmic	NP_001020	mmu:20779	
K12567	TTN	mmu05410(	PF07679.22	GO:004858	cytoplasmic	NP_035782	mmu:22138	
K18060	MTPAP		PF22600.2(I	GO:007104	mitochondr	NP_080433	mmu:67440	
K16578	CLASP1_2		PF12348.14	GO:003111	cytoplasmic	NP_083909	mmu:76499	
K10104	FCN		PF00147.23	GO:001922	extracellulc	NP_032021	mmu:14133	
K11347	JADE1, PHF17		PF13832.12	GO:008009	cytoplasmic	NP_001123	mmu:269424	
K13728	MAD2L2	mmu04914(	PF02301.23	GO:001922	cytoplasmic	BAB26376.1	mmu:71890	
K26954	NUDT22			GO:001678	extracellulc	NP_080951	mmu:68323	
KT0M(RWD domain containing)			PF05773.27	GO:007088	cytoplasmic	NP_079890	mmu:66521	
TS3(RNA binding motif protein 6);			PF17780.6((	GO:009030	nuclear	CAA07065.2	mmu:19654	
K06265	DAG1	mmu04820(	PF05454.16	GO:004858	ER	NP_001263	mmu:13138	
				GO:003250	cytoplasmic	CAA07729.	mmu:170942	
K15348	PLEKHM2	mmu05132(	PF23142.1(I	GO:001989	cytoplasmic	NP_001334	mmu:69582	
K10485	BTBD13, GMCL1, GCI		PF00651.37	GO:005125	nuclear	BAE33141.1	mmu:23885	ZBTB
K07372	GJA1, CX4	mmu05412(	PF00029.24	GO:000810	ER	NP_034418	mmu:14609	
K21871	MPHOSPH8, MPP8		PF12796.13	GO:004239	nuclear	NP_076262	mmu:75339	
K14284	NXF, TAP,	mmu05164(Influenza A		GO:001593	mitochondr	XP_006528	mmu:245610	
K08452	ADGRG3, GPR97		PF00002.29	GO:000548	plasma mer	BAE32739.1	mmu:54672	
K25550	MYCBP			GO:008009	cytoplasmic	NP_001258	mmu:56309	
K01719	hemD, URO	mmu01240(	PF02602.20	GO:000582	cytoplasmic	NP_001289	mmu:22276	
K13885	SCARB1	mmu04975(	PF01130.26	GO:000959	lysosomal	NP_058021	mmu:20778	
K06752	MHC2	mmu05323(	PF00969.24	GO:001989	cytoplasmic	AAA39542.	mmu:14961	
K06825	FBN1	mmu04350(	PF07645.21	GO:009008	plasma mer	NP_032019	mmu:14118	
K23342	FBN2_3	mmu04820(	PF07645.21	GO:003510	extracellulc	NP_034311	mmu:14119	
K00249	ACADM, a	mmu00280(	PF00441.29	GO:003444	mitochondr	NP_031408	mmu:11364	
K06670	SCC1, MCD	mmu04110(	PF04825.18	GO:000810	cytoplasmic	NP_001398	mmu:19357	
K14007	SEC24	mmu04141(	PF04811.21	GO:000548	mitochondr	NP_001277	mmu:77371	
K11831	NRF1	mmu05016(	PF10491.14	GO:004322	nuclear	XP_031237	mmu:18181	Nrf1
K04364	GRB2	mmu05208(	PF00018.34	GO:005123	cytoplasmic	KAF628679	mmu:14784	
K20909	ANTXR	mmu04621(	PF05587.19	GO:000989	plasma mer	XP_021066	mmu:71914	
K26416	CCM2	mmu04520(	PF16545.11	GO:004521	cytoplasmic	NP_666126	mmu:216527	
K10610	DDB1	mmu03420(	PF23726.1(I	GO:004423	cytoplasmic	NP_056550	mmu:13194	
K04718	SPHK	mmu04666(	PF00781.29	GO:001922	cytoplasmic	BAE30855.1	mmu:56632	
K23390	BAG6	mmu03060(	PF12057.13	GO:003015	cytoplasmic	NP_001239	mmu:224727	
K09037	MAFF_G_K		PF03131.22	GO:003250	nuclear	NP_001092	mmu:17134	TF_bZIP

K12017	TRIM41		PF13765.14	GO:004858	cytoplasmic	NP_663352	mmu:211007	
K07605	KRT2		PF00038.26	GO:009951	cytoplasmic	AAI07397.1	mmu:406220	
K08342	ATG4	mmu04140(	PF03416.26	GO:003472	cytoplasmic	BAE22307.	mmu:242557	
K15050	EXOG		PF01223.29	GO:001602	mitochondr	NP_766044	mmu:208194	
)0B5(phosphate ion binding); ENO(			PF10457.14	GO:004323	peroxisoma	NP_001334	mmu:76205	
K12197	CHMP1, VI	mmu03272(	PF03357.26	GO:000810	cytoplasmic	NP_001076	mmu:234852	
K01783	rpe, RPE	mmu00030(	PF00834.25	GO:000673	cytoplasmic	NP_001297	mmu:66646	
28HN(Pygopus homolog 2 (Drosoph			PF00628.35	GO:003250	nuclear	NP_001280	mmu:68911	
intigen binding)			PF00892.27	GO:200123	plasma mer	XP_036021	mmu:74919	
K10388	PLEC	mmu04820(	PF21020.4(	GO:001922	cytoplasmic	AAH21810.	mmu:18810	
K08052	NF1	mmu01521(	PF21877.2(	GO:000548	cytoplasmic	NP_035027	mmu:18015	
K14439	SMARCAD	mmu04550(	PF00176.29	GO:008009	nuclear	EDK98773.	mmu:13990	
K06276	PDPK1	mmu05208(	PF00069.31	GO:006220	cytoplasmic	NP_035192	mmu:18607	
K11463	SUZ12	mmu03083(	PF09733.14	GO:003506	nuclear	NP_001156	mmu:52615	
K12567	TTN	mmu05410(	PF07679.22	GO:001677	cytoplasmic	BAB26738.	mmu:22138	
K14548	UTP4, CIRI	mmu03008(	PF24807.1(	GO:003204	nuclear	BAE27022.	mmu:21771	
K13951	ADH1_7	mmu00980(	PF00107.32	GO:005128	cytoplasmic	NP_031435	mmu:11522	
K09514	DNAJB8			GO:009008	cytoplasmic	KAF157326	mmu:56691	
K19895	GFRA		PF02351.21	GO:000989	extracellulo	NP_032141	mmu:14586	
K11089	TROVE2, S	mmu05322(	PF25045.1(	GO:002260	cytoplasmic	NP_038863	mmu:20822	
K04722	TNFRSF10	mmu05164(	PF00531.28	GO:000548	extracellulo	NP_064671	mmu:21933	
K08809	SPEG		PF07679.22	GO:003250	cytoplasmic	NP_031489	mmu:11790	
K04523	UBQLN, D	mmu04141(	PF23195.1(	GO:008009	cytoplasmic	NP_061268	mmu:54609	
)2ZI(Testis expressed 9)				GO:000573	cytoplasmic	AAI45186.1	mmu:21778	
K10523	SPOP	mmu04340(	PF22486.2(	GO:200123	nuclear	BAE40145.	mmu:20747	ZBTB
K02997	RP-S9e, RP	mmu05171(	PF00163.24	GO:000548	cytoplasmic	NP_001004	mmu:76846	
K08035	FOXA2, HI	mmu04950(	PF09354.15	GO:009008	nuclear	NP_001277	mmu:15376	Fork head
K09553	STIP1	mmu05020(	PF00515.34	GO:003107	cytoplasmic	BAE40141.	mmu:20867	
K03504	POLD3	mmu03440(	PF09507.16	GO:000629	nuclear	BAE40139.	mmu:67967	
K14298	RAE1, GLE	mmu03013(	PF23414.1(	GO:001593	cytoplasmic	NP_780321	mmu:66679	
K04542	GNG5	mmu04724(	PF00631.27	GO:000557	mitochondr	NP_001124	mmu:14707	
K16542	CDK5RAP2		PF07989.16	GO:003111	cytoplasmic	AAH86696.	mmu:214444	
K06054	HES1	mmu05165(	PF07527.18	GO:004282	cytoplasmic	NP_001403	mmu:15205	bHLH
K20160	DENND1		PF02141.26	GO:009859	cytoplasmic	NP_666234	mmu:227801	
K21347	TAX1BP1	mmu04140(	PF07888.16	GO:000810	cytoplasmic	NP_001342	mmu:52440	TF_others
K00953	FLAD1	mmu01240(	PF00994.29	GO:004280	peroxisoma	NP_001349	mmu:319945	
K11844	USP16_45	mmu03083(	PF00443.35	GO:000823	nuclear	NP_690038	mmu:77593	
K10765	ALKBH1			GO:000578	cytoplasmic	EDL02947.	mmu:211064	
K24399	LRMDA		PF14580.12	GO:003250	cytoplasmic	BAE32039.	mmu:76633	
K05058	LIFR, CD1	mmu04060(	PF17971.7(	GO:000512	plasma mer	NP_001345	mmu:16880	
K03136	TFIIE1, GT	mmu05203(	PF02002.23	GO:004393	nuclear	NP_001342	mmu:74197	
K00355	NQO1	mmu01240(	PF02525.22	GO:000677	cytoplasmic	NP_032732	mmu:18104	
K20291	COG4, COD1		PF08318.18	GO:000810	cytoplasmic	NP_598734	mmu:102339	

K22190	MS4A3S		PF04103.21	GO:000579	cytoplasmic	BAE32042.	mmu:64380	
K05543	DUS2		PF01207.24	GO:000578	cytoplasmic	NP_079794	mmu:66369	
K14217	IFIT1	mmu05160(	PF07719.23	GO:003545	cytoplasmic	BAE32046.	mmu:15957	
K13188	RBM7		PF00076.28	GO:000038	nuclear	NP_659197	mmu:67010	
K06133	LYS5, acpT	mmu00770(	PF22624.1(	GO:000548	mitochondr	NP_080552	mmu:67618	
K09548	PFDN1		PF01920.25	GO:002153	cytoplasmic	NP_001101	mmu:67199	
K14535	TAF9	mmu03022(	PF02291.21	GO:008009	nuclear	NP_001015	mmu:108143	
K08674	FAP		PF00930.27	GO:000425	cytoplasmic	NP_032012	mmu:14089	
K10281	FBXL15		PF24758.1(I	GO:003250	cytoplasmic	NP_598455	mmu:68431	
K19993	PLEK		PF00169.35	GO:000810	cytoplasmic	NP_062422	mmu:56193	
K11149	DHRS9	mmu00830(	PF00106.31	GO:000152	ER	NP_780721	mmu:241452	
K15837	GPSM2		PF13424.12	GO:005165	cytoplasmic	NP_083798	mmu:76123	
JK6(Cysteine-rich hydrophobic don			PF10256.14	GO:000579	cytoplasmic	NP_001099	mmu:74277	
K21128	S100A9	mmu04657(	PF01023.25	GO:009008	cytoplasmic	NP_001268	mmu:20202	
K00432	gpx, btuE, l	mmu05022(	PF00255.25	GO:200123	mitochondr	NP_032186	mmu:14775	
K10406	KIFC2_3	mmu04814(	PF00225.29	GO:009951	cytoplasmic	NP_001359	mmu:16582	
YSPJ(Glutamate-rich 1)					nuclear	NP_001030	mmu:234086	
K02539	MCL1	mmu04151(	PF00452.24	GO:200123	mitochondr	NP_032588	mmu:17210	
K08660	CNDP2	mmu00410(	PF01546.33	GO:000823	cytoplasmic	NP_001276	mmu:66054	
K12877	MAGOH	mmu03013(	PF02792.19	GO:001593	cytoplasmic	NP_001029	mmu:17149	
K24313	SH3TC		PF13424.12(TPR_12:Te		cytoplasmic	NP_001346	mmu:231147	
NB9(testis expressed 264)				GO:003802	ER	NP_001075	mmu:21767	
K12470	SH3KBP1, (	mmu04144(	PF14604.12	GO:000810	cytoplasmic	NP_001129	mmu:58194	
K23435	AGTPBP1, CCP1		PF18027.6(I	GO:008009	cytoplasmic	NP_001364	mmu:67269	
K13431	SRPR	mmu03060(	PF04086.19	GO:000578	ER	NP_080406	mmu:67398	
K20287	PLIN3		PF03036.21	GO:001602	cytoplasmic	NP_080112	mmu:66905	
K08736	MSH3	mmu05200(	PF00488.27	GO:008009	nuclear	NP_034959	mmu:17686	
K16484	RTTN		PF14726.11	GO:002260	cytoplasmic	NP_780751	mmu:246102	
K00649	GNPAT	mmu00564(	PF19277.4((	GO:000578	peroxisoma	NP_034452	mmu:14712	
K10155	IRF8	mmu05133(	PF10401.14	GO:000690	nuclear	NP_001288	mmu:15900	IRF
K00031	IDH1, IDH	mmu05230(	PF00180.25	GO:001649	mitochondr	NP_766599	mmu:269951	
K18019	PTPN7	mmu04010(	PF00102.32	GO:001679	cytoplasmic	XP_021053	mmu:320139	
K03374	ST6GALN/	mmu00604(	PF00777.23	GO:004729	Golgi appa	BAE36463.	mmu:20448	
K17336	PLS3		PF00307.36	GO:003243	cytoplasmic	NP_001159	mmu:102866	
K10687	UBE2F	mmu04120(	PF00179.31	GO:000815	cytoplasmic	NP_001342	mmu:67921	
Dehydrogenase, E1 component)			PF13836.11	GO:008009	nuclear	NP_001160	mmu:20896	TF_others
XTIR(NHL repeat containing); EN(			PF01436.27	GO:000557	extracellul	NP_080087	mmu:66866	
K06093	PARD6	mmu05165(	PF00564.30	GO:001922	cytoplasmic	NP_001040	mmu:56513	
K11130	NOP10, N(	mmu03008(	PF04135.18	GO:003051	cytoplasmic	NP_001119	mmu:66181	
K05307	THTPA	mmu00730(	PF01928.26	GO:000548	cytoplasmic	NP_694723	mmu:105663	
K10808	RRM2	mmu00983(	PF00268.27	GO:200123	cytoplasmic	XP_038934	mmu:382985	
K14662	NTAN1		PF14736.11	GO:004423	cytoplasmic	NP_035076	mmu:18203	
K09314	DLX1_4_6		PF00046.35	GO:008009	nuclear	XP_006760	mmu:13390	Homeobox

K04708	KDSR	mmu00600(	PF00106.31	GO:003014	ER	BAE41541.1	mmu:70750	
K09299	ZEB1	mmu05202(	PF13465.12	GO:008009	nuclear	NP_035676	mmu:21417	zf-C2H2
K15030	EIF3M		PF01399.33	GO:002260	cytoplasmic	NP_663355	mmu:98221	
K10462	KLHL25_37, ENC1, EN		PF01344.31	GO:000989	cytoplasmic	NP_031956	mmu:13803	ZBTB
K12795	SUGT1, SG	mmu04621(	PF05002.20	GO:002260	cytoplasmic	NP_080750	mmu:67955	
K00654	SPT	mmu04071(	PF00155.27	GO:000578	ER	NP_033295	mmu:268656	
K09485	HSP110	mmu04141(	PF00012.26	GO:000645	cytoplasmic	EDL35138.	mmu:18415	
K16812	TPX2		PF12214.13	GO:003111	cytoplasmic	NP_001135	mmu:72119	
K11267	PDS5	mmu04110(	PF20168.4(I	GO:003250	nuclear	NP_780519	mmu:100710	
ZMNN(IGv); ENOG41114KX(IGv)			PF07686.23	GO:000588	cytoplasmic	CAA27084.1(unnamed protein prod		
K26118	ATAD5		PF00004.35	GO:200123	nuclear	NP_001025	mmu:237877	
K02971	RP-S21e, RI	mmu05171(	PF01249.23	GO:000578	cytoplasmic	EDL07324.	mmu:66481	
K04368	MAP2K1, M	mmu05208(	PF00069.31	GO:000578	cytoplasmic	NP_032953	mmu:26395	
K18749	LSM14, RAP55, SCD6		PF09532.15	GO:009715	peroxisoma	BAC28922.1	mmu:241846	
K00011	AKR1B	mmu00052(	PF00248.27	GO:000810	cytoplasmic	AAH04725.	mmu:11677	
K14848	RRB1, GRWD1		PF00400.38	GO:003472	nuclear	AAH83143.	mmu:101612	
K21991	UNC45		PF11701.14	GO:000645	cytoplasmic	NP_598713	mmu:101869	
K24246	EMILIN		PF07546.18	GO:009863	extracellula	NP_001344	mmu:246707	
K01836	PGM3	mmu00520(	PF21404.3(	GO:004846	cytoplasmic	BAE40920.1	mmu:109785	
K15680	HSD11B1	mmu00980(	PF00106.31	GO:001922	extracellula	NP_001038	mmu:15483	
K08655	DPP8		PF19520.5(I	GO:000417	cytoplasmic	NP_001388	mmu:74388	
)UEV(R3H domain and coiled-coil (			PF01424.28	GO:009715	nuclear	NP_001288	mmu:71843	
K07416	CYP2F	mmu00980(	PF00067.27	GO:000449	ER	NP_031843	mmu:13107	
K05033	ABCC9, SU	mmu02010(	PF00664.28	GO:003444	cytoplasmic	NP_001038	mmu:20928	
K19283	ACPP		PF00328.27	GO:000677	extracellula	BAE37304.1	mmu:56318	
K24936	TTC28		PF13424.12	GO:001989	cytoplasmic	NP_001254	mmu:209683	
.ZNW(Keratinocyte associated prot			PF09775.14	GO:003299	plasma mer	NP_001297	mmu:66059	
K11366	USP22_27_51, UBP8		PF00443.35	GO:008009	cytoplasmic	BAD90248.	mmu:216825	
K08337	ATG7	mmu04140(	PF16420.10	GO:003577	mitochondr	NP_001240	mmu:74244	
igand dependent nuclear receptor ir			PF00096.32	GO:008009	nuclear	NP_065243	mmu:57247	zf-C2H2
K10499	ZBTB12		PF00651.37	GO:000268	nuclear	NP_001397	mmu:19373	ZBTB
K01837	BPGM	mmu00260(	PF00300.27	GO:004643	cytoplasmic	NP_031589	mmu:12183	
.2YA(Nuclear protein, ataxia-telang			PF15712.10	GO:009750	nuclear	NP_001074	mmu:244879	
				GO:003277	nuclear	KAH0509812.1(DNA-directed RNA		
K03107	SRP68	mmu03060(	PF16969.10	GO:000578	cytoplasmic	NP_666144	mmu:217337	
K11295	HMGB2		PF09011.16	GO:200123	nuclear	NP_001350	mmu:97165	HMG
K07511	ECHS1	mmu00650(	PF00378.25	GO:003444	mitochondr	NP_444349	mmu:93747	
K26126	SLF1		PF23294.1(I	GO:000810	nuclear	XP_006517	mmu:105377	
)1WW(secreted protein, acidic, cyste			PF10591.15	GO:005084	extracellula	NP_001345	mmu:13602	
K05970	SIAE		PF03629.24	GO:003433	lysosomal	NP_035864	mmu:22619	
K13175	THOC6	mmu03013(	PF00400.38	GO:001593	cytoplasmic	NP_001008	mmu:386612	
K00789	metK, MAT	mmu01240(	PF04321.23	GO:199023	nuclear	NP_598778	mmu:108645	
K06829	CYR61, CCN1, IGFBP1		PF19035.6(	GO:004340	plasma mer	NP_034646	mmu:16007	

K17732	PMPCB, MAS1		PF00675.25	GO:000417	mitochondr	BAE41163.1	mmu:73078
K12175	GPS1, COPS1, CSN1		PF10602.15	GO:004322	mitochondr	NP_663345	mmu:209318
K12235	SRR	mmu00470(	PF00291.31	GO:004643	cytoplasmic	NP_001156	mmu:27364
K09486	HYOU1	mmu04141(	PF00012.26	GO:200123	ER	NP_067370	mmu:12282
KSYR(UPF0378 protein)			PF10344.16	GO:001602	ER	NP_001002	mmu:72503
KP9S(von Willebrand factor A doma			PF19435.4(I	GO:001618	cytoplasmic	NP_001071	mmu:69882
K20630	CHN1_2, ARHGAP2_3		PF00620.32	GO:000548	cytoplasmic	XP_035304	mmu:69993
K01437	ASPA, aspA	mmu00250(	PF24827.1(	GO:000548	cytoplasmic	NP_001404	mmu:11484
K13126	PABPC	mmu03018(	PF00076.28	GO:003577	cytoplasmic	NP_032800	mmu:18458
K14320	AAAS	mmu03013(	PF24817.1(	GO:003303	peroxisoma	NP_700465	mmu:223921
K11323	JMJD6		PF02373.28	GO:000690	cytoplasmic	ABU68574.	mmu:107817
K12866	WBP11, NP	mmu03040(	PF09429.15	GO:001920	nuclear	BAE30854.1	mmu:60321
K20360	TBC1D22, GYP1		PF00566.25	GO:003023	cytoplasmic	BAE32447.1	mmu:223754
K05940	CROT	mmu04146(	PF00755.25	GO:000657	peroxisoma	NP_001404	mmu:74114
K04630	GNAI	mmu04730(	PF00503.26	GO:200123	cytoplasmic	NP_034436	mmu:14679
K24523	CPNE5_8_9		PF07002.22	GO:001602	cytoplasmic	NP_080091	mmu:66871
K10398	KIF11, EG5	mmu04814(	PF00225.29	GO:000705	cytoplasmic	NP_034745	mmu:16551
K00522	FTH1	mmu04978(	PF00210.30	GO:000004	cytoplasmic	NP_034369	mmu:14319
K21290	PIK3R5_6	mmu04725(	PF10486.14	GO:001922	cytoplasmic	NP_001075	mmu:104709
K05016	CLCN7		PF00654.25	GO:003032	cytoplasmic	NP_001304	mmu:26373
Y937(-); ENOG410XTD1(-)			PF13926.11	GO:000367	nuclear	NP_778187	mmu:233210
K23801	PCID2, THP1		PF01399.33	GO:005116	nuclear	AAH06047.	mmu:234069
VYI(Nurim (Nuclear envelope membrane prot				GO:000563	extracellulo	NP_598883	mmu:106582
K01366	CTSH	mmu04142(	PF00112.29	GO:200123	lysosomal	NP_031827	mmu:13036
K10394	KIF3A	mmu04340(	PF00225.29	GO:003315	nuclear	XP_006532	mmu:16568
K14165	K14165		PF00782.25	GO:005134	cytoplasmic	NP_077758	mmu:68082
K20316	SCOC			GO:000580	cytoplasmic	XP_048189	mmu:56367
K14165	K14165		PF00782.25	GO:008009	cytoplasmic	NP_598829	mmu:105352
K00323	NNT	mmu00760(	PF02233.22	GO:001922	mitochondr	Q61941.2(R	mmu:18115
K07374	TUBA	mmu04530(	PF00091.31	GO:000520	cytoplasmic	NP_059075	mmu:53857
K22031	MYZAP			GO:000563	cytoplasmic	NP_001028	mmu:102371
K00432	gpx, btuE, b	mmu05022(	PF00255.25	GO:005138	ER	NP_032187	mmu:14778
K23878	AAGAB		PF10199.14	GO:001660	cytoplasmic	NP_080133	mmu:66939
K10396	KIF5	mmu05020(	PF00225.29	GO:003577	cytoplasmic	NP_032474	mmu:16573
K06624	CDKN1B, l	mmu04012(	PF02234.24	GO:003111	cytoplasmic	NP_034005	mmu:12576
K05617	SLC1A6, EA	mmu04724(	PF00375.23	GO:000150	peroxisoma	XP_029338	mmu:20513
K25218	AFTPH		PF15045.11	GO:000810	cytoplasmic	NP_001277	mmu:216549
K11253	H3	mmu05322(	PF00125.30	GO:000989	nuclear	AAH94041.	mmu:319149
K26541	RELL	mmu04060(	PF12606.13	GO:004858	extracellulo	NP_001404	mmu:100532
K23701	SH2D5			GO:000557	mitochondr	AAH36961.	mmu:230863
K03125	TAF1	mmu03022(	PF12157.13	GO:008009	nuclear	NP_001392	mmu:270627
K06084	LMO7, FBX	mmu04520(	PF15949.10(DUF4757:D		nuclear	XP_036014	mmu:380928
K05699	ACTN1_4	mmu04530(	PF00435.27	GO:003243	cytoplasmic	NP_598917	mmu:109711

K26052	CINP			GO:009030	nuclear	BAB25277.1	mmu:67236	
K14018	PLAA, DO	mmu04141(	PF08324.17	GO:003243	cytoplasmic	EDL30938.	mmu:18786	
K03505	POLD4	mmu03440(	PF04081.18	GO:000828	nuclear	EDL33038.	mmu:69745	
K07916	RAB7L1, RAB7L		PF00071.28	GO:003315	cytoplasmic	NP_001408	mmu:226422	
K13296	PDE3B	mmu04910(	PF00233.25	GO:009008	ER	NP_035185	mmu:18576	
K16271	RLIM, RNF12		PF13639.12	GO:000548	cytoplasmic	NP_001345	mmu:19820	
K03942	NDUFV1	mmu05208(	PF01512.22	GO:005128	mitochondr	NP_598427	mmu:17995	
mmunoglobulin heavy variable)			PF13637.12	GO:199023	cytoplasmic	NP_001297	mmu:109346	
K21269	SH3GLB2	mmu04144(	PF03114.23	GO:009900	cytoplasmic	NP_001276	mmu:227700	
K15438	AIMP2		PF18569.7(	GO:190187	cytoplasmic	NP_666277	mmu:231872	
K04398	CASP8	mmu05022(	PF00656.27	GO:003202	extracellula	NP_001073	mmu:12370	
ZICF(R3h domain containing)			PF12752.12	GO:009715	nuclear	XP_006529	mmu:226412	
K26391	STOML1, SLP1		PF01145.30	GO:000686	cytoplasmic	NP_081218	mmu:69106	
K06587	ITGA11	mmu05165(	PF00092.34	GO:009863	cytoplasmic	NP_795896	mmu:319480	
K19720	COL3A	mmu04974(	PF01391.23	GO:004423	extracellula	BAC37545.	mmu:12825	
K03873	ELOB, TCE	mmu05200(	PF00240.29	GO:004518	cytoplasmic	NP_080581	mmu:67673	
K10416	DYNC1LI,	mmu04962(	PF05783.17	GO:009951	mitochondr	NP_666341	mmu:235661	
K16584	HAUS1, AUG1			GO:002260	cytoplasmic	NP_666201	mmu:225745	
K24907	DIP2A		PF00501.34	GO:001640	cytoplasmic	Q8BWT5.3(	mmu:64451	
K07508	ACAA2	mmu00071(	PF00108.29	GO:200123	mitochondr	NP_803421	mmu:52538	
K15729	PTGES	mmu00590(	PF01124.23	GO:005104	ER	BAE29238.	mmu:64292	
K17080	PHB1		PF01145.30	GO:005084	cytoplasmic	NP_032857	mmu:18673	TF_others
K23934	AHNAK	mmu05132(Salmonella infection)			nuclear	NP_001365	mmu:100041194	
K24173	MPND		PF18755.6(I	GO:009030	cytoplasmic	NP_080806	mmu:68047	
K11667	INO80C, IE	mmu03082(	PF08265.16	GO:003304	mitochondr	NP_766213	mmu:225280	
K07560	dtd, DTD		PF02580.21	GO:000645	mitochondr	NP_083821	mmu:328092	
K20520	DBNL, ABP1		PF00241.25	GO:009951	cytoplasmic	NP_001139	mmu:13169	
K21403	PXYLP1		PF00328.27	GO:001679	ER	NP_001276	mmu:235534	
K07527	FES, FPS	mmu04360(	PF07714.23	GO:003111	cytoplasmic	EDL06992.	mmu:14159	
NE8(histidine protein methyltransf			PF10294.15	GO:000647	nuclear	NP_001342	mmu:69962	
KNRJ(C. elegans)			PF20266.3(	GO:000367	cytoplasmic	NP_808386	mmu:239796	
K10524	NRK1_2	mmu00760(	PF13238.12	GO:000548	cytoplasmic	NP_663472	mmu:225994	
K00923	PIK3C2	mmu04070(	PF00454.33	GO:001922	cytoplasmic	NP_035213	mmu:18704	
K11344	MEAF6, EA	mmu03082(	PF09340.15	GO:003124	nuclear	NP_001107	mmu:70088	
K13093	HTATSF1		PF00076.28	GO:000810	nuclear	NP_082518	mmu:72459	
K05619	TGM1		PF00927.28	GO:004212	cytoplasmic	NP_001155	mmu:21816	
K27757	FASTKD5		PF08368.17	GO:000989	mitochondr	BAE25612.	mmu:380601	
K01061	E3.1.1.45	mmu01100(	PF01738.24	GO:001678	cytoplasmic	NP_853619	mmu:69574	
K13710	ARHGEF7,	mmu04810(	PF16614.10	GO:003111	cytoplasmic	NP_001359	mmu:54126	
K20877	NEK8		PF00069.31	GO:000552	cytoplasmic	NP_543125	mmu:140859	
K03984	SERPINA1	mmu04610(	PF00079.25	GO:000695	extracellula	NP_033271	mmu:20702	
K00643	E2.3.1.37, A	mmu01240(	PF00155.27	GO:003202	mitochondr	AAH22110.	mmu:11655	
K16523	AKAP6		PF00435.27	GO:001922	nuclear	NP_932779	mmu:238161	

K17609	NXN		PF13905.12	GO:190187	ER	NP_032776	mmu:18230	
K01456	E3.5.1.52, N	mmu04141(	PF04721.23	GO:003101	cytoplasmic	NP_067479	mmu:59007	
K12383	GM2A	mmu04142(	PF02221.20	GO:009859	lysosomal	NP_034429	mmu:14667	
K03010	RPB2, POL	mmu03420(	PF00562.33	GO:001602	nuclear	NP_722493	mmu:231329	
K18041	PTP4A		PF22785.1(	GO:008009	cytoplasmic	NP_001159	mmu:19245	
K17917	SNX1_2	mmu04144(	PF09325.15	GO:000810	cytoplasmic	BAE32360.	mmu:56440	
K04571	SSR4	mmu04141(	PF05404.17	GO:000578	ER	NP_033305	mmu:20832	
K00741	B3GNT2	mmu00533(	PF01762.27	GO:003030	Golgi appa	NP_001162	mmu:53625	
K02889	RP-L21e, R	mmu05171(	PF01157.24	GO:002262	cytoplasmic	NP_000973	mmu:19933	
K23468	CASQ1	mmu04020(	PF01216.23	GO:009951	ER	NP_033943	mmu:12372	
K16627	SOD3		PF00080.25	GO:004222	extracellula	NP_035565	mmu:20657	
CW8(Ependymin related protein 1			PF00811.25	GO:000032	plasma mer	NP_598826	mmu:105298	
K24760	WDR91		PF23138.1(	GO:000989	cytoplasmic	NP_001013	mmu:101240	
K05632	ITCH, AIP4	mmu04120(	PF00632.30	GO:004213	cytoplasmic	BAD90349.	mmu:16396	
SWB(CDK5 regulatory subunit ass			PF05600.17	GO:005134	cytoplasmic	BAE38797.	mmu:80280	
K00591	COQ3	mmu01240(	PF13489.12	GO:001975	mitochondr	BAE38796.	mmu:230027	
K22143	GSDMC		PF04598.17	GO:006057	cytoplasmic	NP_001161	mmu:331063	
K12584	DCPS, DCS	mmu03018(	PF11969.13	GO:000548	cytoplasmic	NP_081306	mmu:69305	
K22991	WDR45, W	mmu04140(	PF21032.3(	GO:000040	cytoplasmic	NP_001034	mmu:66840	
K15074	BACURD		PF02214.28	GO:004858	cytoplasmic	NP_080421	mmu:330171	
K19985	EXOC6, SEC15		PF04091.18	GO:000810	cytoplasmic	NP_780562	mmu:107371	
K19941	SYN		PF02750.19	GO:005104	cytoplasmic	NP_038708	mmu:20964	
K22611	SART3, TIP110		PF00076.28	GO:003472	nuclear	NP_058622	mmu:53890	
K04494	CHD8, HE	mmu04310(	PF00176.29	GO:003506	nuclear	NP_963999	mmu:67772	
K09821	AGT, SERP	mmu05200(	PF00079.25	GO:000763	extracellula	NP_001403	mmu:11606	
K12559	MYO10	mmu04666(	PF00063.27	GO:003243	cytoplasmic	NP_062345	mmu:17909	
K04348	PPP3C, CN	mmu05020(	PF00149.33	GO:001679	cytoplasmic	XP_006518	mmu:19056	
K11274	WDHD1, CTF4		PF24817.1(	GO:003304	nuclear	NP_766186	mmu:21897	HMG
K05634	PRNP, PrP,	mmu04216(	PF00377.23	GO:001922	extracellula	NP_001265	mmu:19122	
K18194	USMG5, DAPIT		PF14960.11	GO:003109	cytoplasmic	NP_075700	mmu:66477	
K07941	ARF6	mmu04666(	PF00025.27	GO:003288	cytoplasmic	NP_001072	mmu:11845	
K03064	PSMC6, RP	mmu05020(	PF00004.35	GO:009008	cytoplasmic	NP_001269	mmu:67089	
K24626	APOOL		PF09769.14	GO:006102	mitochondr	NP_080841	mmu:68117	
K10368	CAPG		PF00626.27	GO:004877	cytoplasmic	BAE27311.	mmu:12332	
K23408	CDCA7, CDCA7L, JPO		PF10497.14	GO:004212	nuclear	NP_666152	mmu:217946	
K07605	KRT2		PF00038.26	GO:009951	cytoplasmic	NP_083046	mmu:74127	
K02091	CDK6	mmu05200(	PF00069.31	GO:200073	cytoplasmic	NP_034003	mmu:12571	
K19502	ANO8, TMEM16H		PF04547.17	GO:000588	cytoplasmic	XP_006509	mmu:382014	
K23782	AFAP1L		PF00169.35	GO:003243	cytoplasmic	NP_849259	mmu:106877	
K05730	VAV	mmu04664(	PF00621.25	GO:002260	cytoplasmic	NP_033526	mmu:22325	
K27258	WLS		PF06664.17	GO:000810	Golgi appa	EDL11839.	mmu:68151	
K23386	PWWP3, MUM1		PF20886.2(	GO:009030	nuclear	NP_075920	mmu:68114	
K22537	RPUSD3		PF00849.27	GO:007013	mitochondr	NP_001028	mmu:101122	

K17694	ID3	mmu04350(	PF00010.31	GO:003292	cytoplasmic	NP_032347	mmu:15903	bHLH
K05611	UCHL1	mmu05022(	PF01088.27	GO:003169	cytoplasmic	AFP20215.1	mmu:22223	
K11338	RUVBL2, R	mmu03082(	PF06068.19	GO:000810	cytoplasmic	NP_001020	mmu:20174	
K24419	METTL27		PF13649.12	GO:000998	cytoplasmic	NP_001346	mmu:79565	
K03242	EIF2S3		PF09173.17	GO:000374	cytoplasmic	NP_036140	mmu:26905	
K07878	RAB2B		PF00071.28	GO:000810	cytoplasmic	AAH33312.	mmu:76338	
K11584	PPP2R5	mmu05165(	PF01603.26	GO:001920	nuclear	BAC97852.1	mmu:26931	
K08648	HABP2		PF00089.32	GO:000425	extracellula	NP_001316	mmu:226243	
ZHVS(G patch domain containing 8			PF01585.28	GO:004316	nuclear	NP_001152	mmu:237943	
K11794	IFRG15			GO:000699	cytoplasmic	BAC36093.1	mmu:240832	
K21770	CCNB2	mmu05170(	PF00134.28	GO:004477	cytoplasmic	NP_031656	mmu:12442	
K24734	NWD		PF24805.1(	EIF3I:EIF3I	mitochondr	NP_795980	mmu:319807	
K00933	E2.7.3.2	mmu00330(	PF00217.24	GO:000659	mitochondr	NP_001341	mmu:12716	
K16473	IFT20		PF14931.11	GO:000810	cytoplasmic	NP_061342	mmu:55978	
K25805	FEM1A_C		PF12796.13	GO:000815	nuclear	NP_775599	mmu:240263	
)ERT(proline-rich coiled-coil 2B)			PF07001.16	GO:003250	nuclear	NP_001153	mmu:227723	
K05766	SSH	mmu04810(	PF23040.1(I	GO:008009	cytoplasmic	XP_006530	mmu:231637	
Family with sequence similarity 105,			PF12796.13	GO:003243	cytoplasmic	NP_001343	mmu:75646	
K18081	MTMR1_2	mmu04070(	PF06602.19	GO:001679	cytoplasmic	NP_058681	mmu:53332	
K01164	POP1	mmu03008(	PF22770.2(I	GO:000454	nuclear	NP_690854	mmu:67724	
K04554	UBE2J2, N(	mmu04141(	PF00179.31	GO:004438	cytoplasmic	NP_001034	mmu:140499	
K02939	RP-L9, MR	mmu03010(	PF22078.1(I	GO:001593	mitochondr	NP_084392	mmu:78523	
K13108	SNIP1		PF00498.31	GO:000548	nuclear	NP_780455	mmu:76793	
K00327	POR		PF00667.26	GO:003444	ER	NP_032924	mmu:18984	
K18622	ADD		PF00596.26	GO:004846	cytoplasmic	BAC26472.1	mmu:11519	
K06554	IGLL1, IGI	mmu05340(	PF07654.21	GO:000237	mitochondr	AFN88466.	mmu:16136	
K04733	IRAK4	mmu05164(	PF00069.31	GO:000514	cytoplasmic	BAE24205.1	mmu:266632	
K10703	HACD, PH	mmu01040(	PF04387.19	GO:000663	ER	NP_076076	mmu:70757	
K25395	CFAP36		PF11527.13	GO:003586	nuclear	NP_080016	mmu:216618	
K02726	PSMA2	mmu05020(	PF00227.31	GO:003577	cytoplasmic	NP_032970	mmu:19166	
K01228	MOGS	mmu04141(	PF03200.22	GO:001592	ER	BAE34029.1	mmu:57377	
ıucleolar protein 4)			PF00085.26	GO:000557	ER	BAE34031.1	mmu:106200	
K25810	KLHDC1		PF24681.1(I	GO:199023	cytoplasmic	AAI19568.1	mmu:271005	
ılpha- and gamma-adaptin binding			PF00038.26	GO:000557	cytoplasmic	NP_001192	mmu:212632	
K02969	RP-S20e, R	mmu05171(	PF00338.27	GO:002262	cytoplasmic	NP_001007	mmu:67427	
K05105	EPHA4, SE	mmu04360(	PF07714.23	GO:004521	plasma mer	NP_031962	mmu:13838	
K00128	ALDH	mmu01240(	PF00171.27	GO:000663	mitochondr	AAK01551.	mmu:11671	
K08046	ADCY6	mmu04750(	PF00211.25	GO:000401	cytoplasmic	NP_001397	mmu:11512	
K19369	HSPB11		PF00754.31	GO:000367	cytoplasmic	NP_001342	mmu:72938	
K26736	PA2G4, EBP1		PF00557.29	GO:004438	nuclear	NP_035249	mmu:18813	
K18932	ZDHHC		PF01529.25	GO:000810	extracellula	NP_001032	mmu:66220	
K06515	SLC44A1, C	mmu05231(	PF04515.18	GO:001585	plasma mer	XP_006537	mmu:100434	
K01867	WARS, trpS	mmu00970(	PF00579.31	GO:001922	cytoplasmic	NP_001157	mmu:22375	

K14996	SLC38A10		PF01490.24	GO:000534	Golgi appa	EDL34731.	mmu:72055	
K00072	SPR	mmu01240(	PF00106.31	GO:000548	cytoplasmic	Q64105.1(R	mmu:20751	
K25201	IPO11, RAI	mmu03013(	PF03810.25	GO:003450	cytoplasmic	NP_083941	mmu:76582	
K03142	TFIIH2, GT	mmu03420(	PF04056.20	GO:000548	nuclear	AAH53382.	mmu:23894	
K15276	SLC35B2, PAPST1		PF08449.17	GO:000534	Golgi appa	BAE39443.	mmu:73836	
K19675	IFT43		PF15305.11	GO:004299	cytoplasmic	NP_001186	mmu:76411	
K12484	RAB11FIP1	mmu04144(	PF00168.36	GO:000548	cytoplasmic	NP_001074	mmu:75767	
K20197	KIF3C	mmu04814(	PF00225.29	GO:009951	nuclear	NP_032471	mmu:16570	
K20363	YIPF5_7, YIP1		PF04893.22	GO:004323	cytoplasmic	NP_075800	mmu:67180	
K08816	VRK		PF00069.31	GO:004239	nuclear	EDL18743.	mmu:22367	
K14139	IRGM, LRC	mmu04621(	PF05049.19	GO:007066	cytoplasmic	SDA08570.	mmu:15944	
K09586	ERP29	mmu04141(	PF07912.19	GO:000810	ER	NP_080405	mmu:67397	
K06566	IFITM		PF04505.17	GO:004659	cytoplasmic	NP_109619	mmu:80876	
K00972	UAP1	mmu00520(	PF01704.24	GO:004634	cytoplasmic	NP_001028	mmu:227620	
K03031	PSMD8, RP	mmu05020(	PF10075.15	GO:002262	nuclear	BAE35376.	mmu:57296	
K13858	SLC4A7, NBC3		PF00955.27	GO:009859	cytoplasmic	NP_001347	mmu:218756	
K15142	MED29		PF11568.13	GO:006026	nuclear	NP_080318	mmu:67224	
K18735	SMG9			GO:000018	cytoplasmic	NP_001405	mmu:71997	
K20792	NAA15_16		PF12569.14	GO:003124	cytoplasmic	XP_027423	mmu:74838	
K14765	NGDN, LCP5		PF04000.20	GO:005080	nuclear	NP_081166	mmu:68966	
K14649	TAF8	mmu03022(	PF07524.19	GO:008009	nuclear	NP_071298	mmu:63856	
K09604	ARTS1		PF11838.14	GO:000512	extracellula	NP_001413	mmu:80898	
K07375	TUBB	mmu05020(	PF00091.31	GO:009951	cytoplasmic	NP_001074	mmu:545486	
K04659	THBS2S	mmu05165(	PF05735.19	GO:006034	extracellula	NP_038719	mmu:21827	
K02211	CDK9	mmu03250(	PF00069.31	GO:003577	cytoplasmic	NP_001007	mmu:107951	
K11513	MLF1IP, CENPU		PF13097.11	GO:000979	nuclear	AAI17785.1	mmu:71876	
K00819	rocD, OAT	mmu00330(	PF00202.27	GO:001949	mitochondr	NP_058674	mmu:18242	
K14851	RRP17, NOL12		PF09805.14	GO:000573	cytoplasmic	NP_598561	mmu:97961	
K17616	CTDSPL2		PF03031.24	GO:003315	nuclear	NP_997615	mmu:329506	
K13667	POGLUT1,	mmu00514(	PF05686.18	GO:004859	extracellula	NP_759012	mmu:224143	
K20629	ABR		PF00620.32	GO:000508	cytoplasmic	XP_021031	mmu:109934	
RNA methylase); COG0116(RNA m			PF01170.24	GO:000151	cytoplasmic	EDL38536.	mmu:72167	
K02896	RP-L24e, R	mmu05171(	PF01246.26	GO:000548	cytoplasmic	BAE23079.	mmu:68193	
K04394	CASP4, CA	mmu04621(	PF00656.27	GO:000959	cytoplasmic	NP_031635	mmu:12363	
K04676	SMAD1	mmu04390(	PF03166.19	GO:000097	cytoplasmic	NP_032565	mmu:17125	MH1
K04739	PRKAR	mmu04910(	PF00027.35	GO:001922	cytoplasmic	NP_001351	mmu:19088	
K02503	HINT1_2, hinT, hit		PF01230.28	GO:004643	cytoplasmic	NP_001103	mmu:15254	
K03025	RPC6, POL	mmu04623(	PF05158.18	GO:008009	cytoplasmic	BAC26372.	mmu:70408	
K13710	ARHGEF7,	mmu04810(	PF16614.10(RhoGEF67		nuclear	NP_001359	mmu:54126	
K11587	CBX5, HP1A		PF01393.25	GO:000011	nuclear	NP_001070	mmu:12419	
K10779	ATRX	mmu04110(	PF00176.29	GO:003472	nuclear	NP_033556	mmu:22589	
K16744	B9D1		PF07162.17	GO:004859	cytoplasmic	XP_031210	mmu:27078	
BJ6(Inositol 1,4,5-trisphosphate rec			PF20266.3(	GO:000557	ER	NP_001156	mmu:73338	

K23215	BCOR	mmu03083(	PF15808.10	GO:008009	nuclear	XP_030107	mmu:71458
K07914	RAB37		PF00071.28	GO:000810	cytoplasmic	NP_067386	mmu:58222
K02606	ORC4	mmu04110(	PF14629.12	GO:002260	mitochondr	NP_001342	mmu:26428
K17272	BASP1		PF05466.17	GO:002260	cytoplasmic	NP_081671	mmu:70350
K25863	CYBC1		PF15169.12	GO:000578	ER	NP_001239	mmu:217370
K24175	MFSD5		PF05631.20	GO:000681	plasma mer	NP_598861	mmu:106073
K11303	HAT1, KA7	mmu04613(	PF10394.15	GO:004239	cytoplasmic	NP_080391	mmu:107435
ıtaxin 10); ENOG410XRED(ataxin			PF07678.20	GO:003023	extracellulo	NP_032671	mmu:17836
XRED(Complement component); C(			PF07678.20	GO:003023	extracellulo	NP_032672	mmu:17837
K13536	MARCKSL	mmu04666(	PF02063.22	GO:000548	cytoplasmic	NP_034937	mmu:17357
K03347	CUL1, CDC	mmu04120(	PF00888.28	GO:199045	cytoplasmic	NP_001102	mmu:26965
K17268	COPE		PF04733.20	GO:004323	cytoplasmic	NP_067513	mmu:59042
K16495	PCDHGA		PF00028.23	GO:000548	plasma mer	NP_291071	mmu:93722
K05402	TOLLIP	mmu04620(	PF00168.36	GO:000810	cytoplasmic	NP_076253	mmu:54473
K18152	BST1, CD1	mmu04970(	PF02267.23	GO:004212	plasma mer	NP_033893	mmu:12182
K15559	RTT103		PF16566.10	GO:004212	nuclear	NP_081710	mmu:70470
K20317	SPAG9, JIP4		PF19056.6(	GO:001922	ER	NP_001020	mmu:70834
K07605	KRT2		PF00038.26	GO:009951	cytoplasmic	NP_032499	mmu:16678
Cysteine rich transmembrane BMP r			PF10609.15	GO:002260	cytoplasmic	NP_036086	mmu:26426
K03263	EIF5A		PF21485.3(I	GO:000374	cytoplasmic	AAH80800.	mmu:276770
K04737	IL3RA, CD	mmu05200(	PF09240.16(IL6Ra-bind		lysosomal	NP_001405	mmu:16188
K17503	PPM1H, ARHCL1		PF00481.26	GO:001679	cytoplasmic	NP_001103	mmu:319468
K12474	LDLRAP1,	mmu04979(	PF00640.29	GO:003238	mitochondr	NP_663529	mmu:100017
K15040	VDAC2	mmu05208(	PF01459.28	GO:200123	cytoplasmic	KAF161042	mmu:22334
K13963	SERPINB	mmu05146(	PF00079.25	GO:003041	cytoplasmic	NP_033282	mmu:20723
K08292	EEF2K	mmu04152(	PF02816.24	GO:000962	cytoplasmic	NP_001254	mmu:13631
K08288	PRKCSH	mmu04141(	PF12999.12	GO:000578	ER	NP_001280	mmu:19089
K03679	RRP4, EXC	mmu03018(	PF21266.3(I	GO:000563	nuclear	NP_659135	mmu:227715
ZYWH(antagonist of mitotic exit ne			PF13516.12	GO:009859	cytoplasmic	XP_021046	mmu:232566
K03575	mutY	mmu03410(	PF00730.31	GO:000070	mitochondr	AAH57942.	mmu:70603
K20166	TBC1D2B		PF00566.25	GO:004322	cytoplasmic	NP_919315	mmu:67016
XRM0(phosphodiesterase 4D interacting protein				GO:008009	cytoplasmic	NP_001405	mmu:67150
K14552	NAN1, UT	mmu03008(	PF23769.1(I	GO:003204	nuclear	BAE40124.	mmu:73674
K07528	DPYSL2, C	mmu04360(	PF01979.26	GO:000573	cytoplasmic	NP_001365	mmu:12934
K15542	PFS2	mmu03015(	PF00400.38	GO:000557	nuclear	NP_083142	mmu:74320
K00789	metK, MA7	mmu01240(	PF02773.21	GO:004316	cytoplasmic	NP_663544	mmu:232087
K12400	AP4E1	mmu04142(	PF01602.26	GO:000810	cytoplasmic	NP_780759	mmu:108011
K15302	STXBP4, SYNIP		PF00397.32	GO:009008	cytoplasmic	NP_035635	mmu:20913
K11290	SET, TAF1, I2PP2A		PF00956.23	GO:003472	nuclear	EDL08431.	mmu:56086
K10641	LRSAM1		PF23598.1(I	GO:000810	cytoplasmic	NP_001366	mmu:227738
K06554	IGLL1, IGI	mmu05340(	PF07654.21	GO:000548	lysosomal	AAH49143.	mmu:16136
K10692	LNX1_2		PF00595.29	GO:003015	cytoplasmic	AAH90665.	mmu:140887
Cystatin B (Stefin B))			PF01926.29	GO:003563	cytoplasmic	NP_032162	mmu:14670

K17555	PPP1R14B		PF05361.17	GO:001922(	cytoplasmic	NP_032915	mmu:18938	
K01045	PON		PF01731.26	GO:001679.	extracellulo	NP_766594	mmu:269823	
K01045	PON		PF01731.26	GO:004657.	extracellulo	NP_899131	mmu:330260	
K11653	ARID1	mmu04714(	PF12031.14	GO:003506(	nuclear	NP_001074	mmu:93760	ARID
K10151	CCND2	mmu04917(	PF00134.28	GO:001922(	cytoplasmic	XP_003496(	mmu:12444	
K16546	FGFR10P		PF09398.16	GO:000548	nuclear	NP_001183	mmu:75296	
K17925	SNX13		PF02194.20(PXA:PXA (		ER	EDL36865.	mmu:217463	
K13700	ABHD6	mmu04723(	PF00561.26	GO:004643	Golgi appa	NP_001317	mmu:66082	
K23868	FAM92		PF06730.16	GO:000573	mitochondr	NP_080834	mmu:68099	
K10532	HGSNAT	mmu04142(	PF07786.18	GO:002260	plasma mer	Q3UDW8.2	mmu:52120	
K01809	manA, MPI	mmu01240(	PF20511.4(I	GO:004643	mitochondr	NP_080113	mmu:110119	
K11270	CTF8		PF09696.15	GO:007110	cytoplasmic	NP_001351	mmu:214987	
K02964	RP-S18e, Rl	mmu05171(	PF00416.27	GO:001593	cytoplasmic	EDL00865.	mmu:20084	
K21437	ANKRD13		PF11904.13(ANKRD13		cytoplasmic	NP_001360	mmu:268445	
K05737	GIT1	mmu04810(	PF12205.13	GO:003111(	cytoplasmic	NP_001361	mmu:216963	
K20411	PRR5, PRO	mmu04150(	PF08539.16	GO:001064	mitochondr	NP_666173	mmu:109270	
K22399	TRIP13	mmu04110(	PF23242.1(	GO:200024	nuclear	NP_081458	mmu:69716	
K07525	NGEF, EPI	mmu04360(	PF00621.25	GO:004308	cytoplasmic	NP_001104	mmu:53972	
K15440	TAD1, ADAT1		PF02137.24	GO:000825	cytoplasmic	NP_038953	mmu:30947	
K20478	GOLGB1			GO:000579	cytoplasmic	AAI50732.1	mmu:224139	
K00734	B3GALT6	mmu00534(	PF01762.27	GO:003020(	ER	NP_536693	mmu:117592	
K08854	BMP2K, BI	mmu05202(	PF15282.11	GO:200036	cytoplasmic	NP_542439	mmu:140780	
K05762	RDX	mmu04530(	PF00769.24	GO:003288(	cytoplasmic	EDL25785.	mmu:19684	
K08007	EZR, VIL2	mmu04971(	PF00769.24	GO:004340	cytoplasmic	NP_033536	mmu:22350	
K05763	MSN	mmu04810(	PF00769.24	GO:003238	cytoplasmic	NP_034963	mmu:17698	
K23402	CBY1	mmu04310(	PF14645.11	GO:000810	mitochondr	NP_001405	mmu:73739	
K10160	TLR4, CD2	mmu05323(	PF13855.12	GO:003288(	plasma mer	NP_067272	mmu:21898	
K02353	DVL	mmu05165(	PF12316.13	GO:000548	nuclear	NP_001403	mmu:13544	
K10426	DCTN4	mmu05022(	PF05502.18	GO:003243	cytoplasmic	NP_080578	mmu:67665	
K02930	RP-L4e, RP	mmu05171(	PF00573.27	GO:002262(	nuclear	NP_077174	mmu:67891	
K05285	PIGN	mmu00563(	PF04987.19	GO:000665(	ER	XP_006529	mmu:27392	
K20349	TMED7		PF01105.29	GO:003802	ER	NP_079974	mmu:66676	
K10839	RAD23, HR	mmu04141(	PF09280.16	GO:008009(	cytoplasmic	NP_033037	mmu:19359	
K23012	TYSND1		PF13365.12	GO:000425	peroxisoma	NP_082188	mmu:71767	
K16470	DZIP1		PF13815.11	GO:000548	cytoplasmic	XP_006511	mmu:72507	
K17407	MRPS28		PF10246.15	GO:001593	mitochondr	NP_079710	mmu:66230	
K00966	GMPP	mmu01240(	PF00483.29	GO:000573	cytoplasmic	BAE25827.	mmu:69080	
K10901	BLM, REC(	mmu03440(	PF16202.10	GO:000573	nuclear	NP_031576	mmu:12144	
K13115	CCDC130		PF04502.18	GO:000613	cytoplasmic	EDL92226.	mmu:67736	
K20278	INPP5E	mmu04070(	PF22669.2(I	GO:000810	cytoplasmic	BAE34376.	mmu:64436	
K03352	APC5, AN/	mmu04120(	PF21371.2(	GO:001096	cytoplasmic	NP_001276	mmu:59008	
K23289	EIPR1, TSSC1		PF23609.1(I	GO:009008	cytoplasmic	NP_958745	mmu:380752	
K24650	HMG20B		PF00505.25	GO:190187	nuclear	EDL31423.	mmu:15353	HMG

K11997	TRIM2_3		PF01436.27	GO:000810	cytoplasmic	NP_001272	mmu:55992	
K18750	APOBEC3	mmu03250(	PF18782.7(	GO:003577	cytoplasmic	NP_001333	mmu:80287	
K05754	ARPC5	mmu04530(	PF04699.19	GO:000520	cytoplasmic	BAE29565.1	mmu:67771	
K17981	MTFP1, MTP18		PF10558.14	GO:000026	cytoplasmic	BAE39981.1	mmu:67900	
K12817	PRPF18, PF	mmu03040(	PF02840.20	GO:002260	cytoplasmic	NP_080321	mmu:67229	
K09498	CCT6		PF00118.29	GO:009951	cytoplasmic	BAE39984.1	mmu:12466	
			PF00429.24	GO:000557	plasma mer	AAC53568.	mmu:110956	
K14296	NUP153	mmu03013(	PF08604.15	GO:005116	cytoplasmic	NP_786925	mmu:218210	
K23311	CTC1		PF15489.11	GO:008009	mitochondr	NP_001268	mmu:68964	
K26934	ATP11	mmu04148(	PF16212.11	GO:199053	cytoplasmic	NP_001280	mmu:50770	
K10411	DNAL1	mmu05014(Amyotrophi		GO:000573	cytoplasmic	XP_023581	mmu:105000	
IFY(chromosome 19 open reading f			PF14966.11(DNA_repr_		cytoplasmic	NP_079853	mmu:66462	
K09864	AQP1	mmu04964(	PF00230.25	GO:003537	plasma mer	NP_031498	mmu:11826	
M9H(tumor protein)			PF04201.20	GO:000573	cytoplasmic	NP_079758	mmu:66314	
K20102	YTHDF		PF04146.21	GO:199024	cytoplasmic	NP_663368	mmu:213541	
K05759	PFN	mmu05014(	PF00235.25	GO:001922	cytoplasmic	NP_001121	mmu:18645	
K12895	RALY, HNRNPCL2		PF00076.28	GO:000989	nuclear	XP_042132	mmu:19383	
K17497	PMM	mmu01240(	PF03332.19	GO:001967	cytoplasmic	NP_058577	mmu:54128	
K12361	RASGRP2	mmu04010(	PF00617.24	GO:009859	cytoplasmic	NP_001398	mmu:19395	
K23919	NAV3		PF23092.1(	GO:003563	nuclear	XP_017169	mmu:260315	
K03511	POLK	mmu05169(	PF00817.25	GO:000629	nuclear	NP_036178	mmu:27015	
K16526	AKAP10		PF00615.24	GO:000810	cytoplasmic	NP_064305	mmu:56697	
K00743	GGTA1	mmu00601(	PF03414.19	GO:000367	Golgi appa	NP_001139	mmu:14594	
K09881	AGK	mmu00561(	PF19712.4(	GO:000810	mitochondr	NP_076027	mmu:69923	
K12572	PAN3	mmu03018(	PF18101.7(	GO:000810	cytoplasmic	NP_001406	mmu:72587	
K06553	VPREB, CD179a		PF07654.21	GO:000548	plasma mer	AAH85241.	mmu:22363	
K07984	SH3BP2	mmu04650(	PF00169.35	GO:000716	cytoplasmic	NP_001139	mmu:24055	
K23770	S100B		PF01023.25	GO:003000	cytoplasmic	NP_033141	mmu:20203	
K09503	DNAJA2	mmu04141(	PF01556.23	GO:000828	cytoplasmic	BAE40948.1	mmu:56445	
K05729	ARHGEF6,	mmu05212(	PF16614.10(RhoGEF67		nuclear	NP_690014	mmu:73341	
K04429	TAO	mmu04010(	PF00069.31	GO:003111	cytoplasmic	NP_001351	mmu:216965	
K00232	E1.3.3.6, AC	mmu00640(	PF22924.1(	GO:003444	cytoplasmic	NP_001343	mmu:80911	
K20821	BORCS7		PF16088.10	GO:000701	cytoplasmic	NP_079839	mmu:66439	
K16480	OFD1		PF16045.11	GO:004003	nuclear	EDL40732.	mmu:237222	
K17045	HMGN5		PF01101.23	GO:008009	nuclear	NP_057919	mmu:50887	
K00710	GALNT	mmu00514(	PF00535.33	GO:003024	mitochondr	NP_766281	mmu:230145	
Z19(Chromosome 1 open reading f			PF15772.10	GO:000557	nuclear	NP_001156	mmu:97159	
K09228	KRAB		PF13465.12	GO:000097	nuclear	P16374.2(R	mmu:22718	zf-C2H2
WVI(-)			PF17719.6(DUF5564:Fc		nuclear	NP_079739	mmu:66276	
K03907	THBD, CD	mmu04610(	PF14670.12	GO:190537	plasma mer	NP_033404	mmu:21824	
K13044	HNRNPABD		PF00076.28	GO:001922	nuclear	Q9Z130.1(R	mmu:50926	
K15287	SLC35F1_2		PF06027.18	GO:000521	plasma mer	NP_082336	mmu:72022	
K02923	RP-L38e, R	mmu05171(	PF01781.23	GO:000988	cytoplasmic	NP_001041	mmu:67671	

K26608	TMEM120		PF07851.18	GO:003250	cytoplasmic	NP_001034	mmu:330189	
K03506	POLE4	mmu03030(	PF00808.29	GO:003124	nuclear	XP_021020	mmu:66979	
K05112	EPHB3, HE	mmu04360(	PF07714.23	GO:003444	cytoplasmic	NP_034273	mmu:13845	
XSUP(sterile alpha motif domain co			PF00098.28(zf-CCHC:Z		cytoplasmic	NP_543131	mmu:142682	
K16338	SDC4	mmu04512(	PF01034.25	GO:004213	ER	NP_035651	mmu:20971	
K07998	MMP19, MMP18		PF00413.30	GO:000417	extracellula	NP_067387	mmu:58223	
K23755	SCO2	mmu05230(	PF02630.20	GO:001972	mitochondr	NP_001104	mmu:100126824	
K08496	GOSR2, BC	mmu04130(	SNARE inte	GO:000810	cytoplasmic	NP_001349	mmu:56494	
K15609	PBX2		PF03792.18	GO:014029	nuclear	NP_059491	mmu:18515	Homeobox
K12476	EHD3	mmu04144(	PF18150.6(I	GO:000810	cytoplasmic	NP_065603	mmu:57440	
K17300	LGALS3BP		PF00530.23	GO:001602	plasma mer	XP_036012	mmu:19039	
K06543	KLRB, CD1	mmu05144(	PF00059.27	GO:000989	cytoplasmic	NP_085102	mmu:80782	
protein family UPF0029, Impact, N-			PF01205.25	GO:001922	cytoplasmic	NP_032404	mmu:16210	
K26228	ARRDC1		PF02752.27	GO:000810	cytoplasmic	XP_030105	mmu:215705	
K06947	GRC3, NOL9		PF16575.11	GO:000044	mitochondr	NP_001153	mmu:74035	
K06464	ITGB2, CD	mmu04670(	PF00362.24	GO:001922	plasma mer	XP_006522	mmu:16415	
K03424	tatD		PF01026.27	GO:001678	cytoplasmic	NP_001344	mmu:68972	
K27339	UQCC4		PF15013.11	GO:002260	mitochondr	NP_898973	mmu:214489	
K01724	PCBD, phh	mmu00790(	PF01329.24	GO:000548	cytoplasmic	XP_015102	mmu:13180	
K24660	HMG20A		PF00505.25	GO:190187	nuclear	NP_080088	mmu:66867	HMG
K14807	DDX51, DBP6		PF00270.35	GO:000367	nuclear	NP_081432	mmu:69663	
K08835	OXSR1, ST	mmu04928(	PF00069.31	GO:003563	cytoplasmic	EDL09229.	mmu:108737	
K03687	GRPE		PF01025.24	GO:000810	mitochondr	NP_067271	mmu:17714	
K20100	YTHDC1		PF04146.21	GO:199024	nuclear	NP_808348	mmu:231386	
K06461	ITGAM, CI	mmu04670(	PF21520.3(I	GO:000690	plasma mer	EDL17627.	mmu:16409	
K17796	TIM21		PF08294.17	GO:003361	mitochondr	NP_080245	mmu:67105	
K08836	SLK	mmu04114(	PF00069.31	GO:005189	cytoplasmic	NP_001158	mmu:20874	
K06781	CADM1, IG	mmu04514(	PF13927.12	GO:001922	extracellula	NP_001297	mmu:54725	
K08017	AVIL		PF00626.27	GO:009951	cytoplasmic	XP_036011	mmu:11567	
K14557	UTP6	mmu03008(	PF24892.1(U	GO:003204	nuclear	NP_659075	mmu:216987	
K01127	GPLD1	mmu00563(	PF01839.29	GO:003577	ER	AAP93132.	mmu:14756	
K00726	MGAT1	mmu00510(	PF03071.21	GO:004643	Golgi appa	NP_001103	mmu:17308	
K04448	JUN	mmu05208(	PF03957.18	GO:000962	nuclear	BAE24518.	mmu:16476	
K09993	CDKN1C, I	mmu04110(	PF02234.24	GO:001922	nuclear	NP_001155	mmu:12577	
K08009	CYBA, P22	mmu05208(	PF05038.18	GO:003243	ER	NP_031832	mmu:13057	
K22379	RNFT1		PF13639.12	GO:000989	cytoplasmic	NP_084064	mmu:76892	
)2BB(The exact function is not known. Can cata				GO:000582	cytoplasmic	NP_666231	mmu:227737	
K13195	CIRBP		PF00076.28	GO:000989	cytoplasmic	XP_026543	mmu:12696	
K08891	FGR, SRC2	mmu04062(	PF07714.23	GO:001922	cytoplasmic	NP_034338	mmu:14191	
K06483	ITGA4, CD	mmu04672(	PF08441.18	GO:004590	cytoplasmic	Q00651.1(R	mmu:16401	
K24166	ARMC6		PF00514.29	GO:003250	cytoplasmic	NP_598733	mmu:76813	
K18082	MTMR3_4,	mmu04070(	PF06602.19	GO:004648	cytoplasmic	XP_006514	mmu:74302	
K04745	CHST2	mmu00533(	PF00685.33	GO:000695	Golgi appa	NP_061233	mmu:54371	

K02684	PRI1	mmu03030(	PF01896.25	GO:003277	cytoplasmic	NP_032947	mmu:19075	
K23424	TMTC			GO:000548	ER	BAC30236.	mmu:70551	
K09220	IKZF, ZNFN1A		PF13465.12	GO:000989	nuclear	NP_001405	mmu:67143	zf-C2H2
K03019	RPC11, PO	mmu04623(	PF01096.23	GO:000367	nuclear	BAC40210.	mmu:67005	
K04446	NFATC1, N	mmu05321(	PF00554.27	GO:008009	cytoplasmic	NP_001157	mmu:18018	RHD
K17385	SPRY4		PF05210.18	GO:009859	cytoplasmic	NP_036028	mmu:24066	
K22857	EEF1AKMT4		PF08241.18	GO:000875	cytoplasmic	NP_079738	mmu:110599566	
K00944	AK3	mmu01240(	PF00406.28	GO:000921	mitochondr	NP_067274	mmu:56248	
K00939	adk, AK	mmu01240(	PF00406.28	GO:000921	cytoplasmic	NP_001029	mmu:11637	
K20477	RGP1		PF08737.15	GO:000989	plasma mer	NP_001386	mmu:242406	
K19192	SAP130		PF16014.10	GO:000989	nuclear	NP_001344	mmu:269003	
K02134	ATPeF1D,	mmu05208(	PF21335.2(	GO:000526	mitochondr	NP_079589	mmu:66043	
K13251	SSR3	mmu04141(	PF07074.17	GO:000810	ER	NP_001231	mmu:67437	
K12654	STING1, T	mmu05170(	PF15009.12	GO:000574	ER	NP_082537	mmu:72512	
K14315	NDC1, TM	mmu03013(	PF09531.15	GO:001593	ER	NP_082631	mmu:72787	
K12581	CNOT7_8,	mmu03018(	PF04857.26	GO:000989	nuclear	NP_001343	mmu:69125	
K07360	ZAP70	mmu04658(	PF07714.23	GO:004663	cytoplasmic	NP_001276	mmu:22637	
K26392	STOML2, SLP2		PF01145.30	GO:001922	mitochondr	NP_075720	mmu:66592	
K09207	KLF6_7		PF00096.32	GO:001922	nuclear	O08584.3(R	mmu:23849	zf-C2H2
K09207	KLF6_7		PF00096.32	GO:009008	nuclear	NP_001386	mmu:93691	zf-C2H2
K24887	GTPBP1		PF00009.33	GO:008009	cytoplasmic	NP_038846	mmu:14904	
K12881	THOC4, AI	mmu03013(	PF00076.28	GO:001593	nuclear	NP_035698	mmu:21681	
K06672	SCC2, NIPI	mmu04110(	PF12830.13	GO:000810	nuclear	NP_081983	mmu:71175	
K00288	MTHFD	mmu01240(	PF01268.25	GO:000916	cytoplasmic	EDL36467.	mmu:108156	
K13171	SRRM1, SR	mmu03013(	PF01480.22	GO:008009	nuclear	NP_001355	mmu:51796	
K17988	TECPR1		PF06462.18	GO:000989	extracellulo	NP_081686	mmu:70381	
K23498	COXPD7		PF00472.26	GO:004518	nuclear	NP_001128	mmu:72650	
K01196	AGL	mmu00500(	PF14701.12	GO:010250	cytoplasmic	NP_001074	mmu:77559	
K04497	GRO, TLE	mmu04310(	PF03920.20	GO:000548	nuclear	XP_038188	mmu:21885	
K06961	KRR1		PF21800.4(I	GO:003204	nuclear	NP_848725	mmu:52705	
1EK(CGG triplet repeat binding protein 1)				GO:014029	nuclear	NP_001344	mmu:106143	
K04500	SMAD2	mmu05210(	PF03166.19	GO:000810	mitochondr	NP_001239	mmu:17126	MH1
K07856	RHOB	mmu05132(	PF00071.28	GO:000810	cytoplasmic	NP_001071	mmu:11852	
K11827	AP2S1	mmu05016(	PF01217.26	GO:000810	cytoplasmic	NP_001069	mmu:232910	
K08065	NFYB, HA	mmu04612(	PF00808.29	GO:000097	cytoplasmic	KAF636983	mmu:18045	NF-YB
K23847	HPCAL1		PF13499.12	GO:004316	cytoplasmic	NP_001348	mmu:53602	
K25291	TMEM94		PF00689.26	GO:003250	plasma mer	XP_006534	mmu:71947	
GTPase that associates with the 50S			PF01926.29	GO:003563	cytoplasmic	NP_660129	mmu:107999	
K16533	CEP290, NPHP6		PF16574.10	GO:003315	nuclear	NP_666121	mmu:216274	
K11660	MTA	mmu03082(	PF17226.7(	GO:003292	nuclear	NP_473422	mmu:11687	zf-GATA
K26100	PWWP2		PF00855.23	GO:008009	nuclear	NP_001092	mmu:101631	
K07987	RAET1	mmu04650(	PF14586.11	GO:001922	cytoplasmic	NP_064414	mmu:56554	
K08012	NCF4, P40I	mmu04670(	PF00787.29	GO:000554	cytoplasmic	NP_001398	mmu:17972	

K01008	selD, SEPH	mmu00450(	PF02769.27	GO:003563	cytoplasmic	NP_033292	mmu:20768	
K00654	SPT	mmu04071(	PF00155.27	GO:001922	cytoplasmic	NP_035609	mmu:20773	
K03211	ETV6_7, ya	mmu05202(	PF00178.28	GO:000097	nuclear	NP_031987	mmu:14011	ETS
K15188	CCNT	mmu03250(	PF21797.2(	GO:008009	nuclear	NP_033963	mmu:12455	
K14821	BUD20		PF12171.14	GO:008009	nuclear	NP_077177	mmu:68040	
K15159	MED16	mmu04919(	PF20718.2(	GO:006026	nuclear	XP_021029	mmu:216154	
K01897	ACSL, fadD	mmu01100(	PF00501.34	GO:001564	ER	NP_001028	mmu:216739	
K22806	SPIDR		PF14951.11	GO:000001	cytoplasmic	BAE38782.	mmu:224008	
K15214	TAF1C		PF20642.2(	GO:003277	cytoplasmic	BAE33818.	mmu:21341	
K15170	MED27	mmu04919(	PF11571.13	GO:008009	nuclear	NP_081172	mmu:68975	
K12182	HGS, HRS,	mmu03250(	PF00790.25	GO:000810	cytoplasmic	BAE38778.	mmu:15239	
KNQX(Osteopetrosis associated tra			PF00134.28	GO:001922	cytoplasmic	NP_080760	mmu:67974	
K17943	PUM	mmu05017(	PF00806.25	GO:003577	cytoplasmic	NP_001153	mmu:80912	
K00387	SUOX	mmu00920(	PF00174.25	GO:003197	mitochondr	NP_776094	mmu:211389	
K15198	BDP1, TFC5		PF15963.11	GO:000115	nuclear	NP_001074	mmu:544971	
K00699	UGT	mmu00980(	PF00201.24	GO:007149	Golgi appa	NP_690024	mmu:71773	
K13159	HNRNPL		PF13893.12	GO:004423	nuclear	XP_051018	mmu:15388	
K10087	GNPTG, G	mmu04142(	PF07915.19	GO:199023	extracellula	NP_766117	mmu:214505	
K00901	dgkA, DGK	mmu05231(	PF00609.24	GO:000665	plasma mer	NP_062378	mmu:56077	
K10606	FANCL, PH	mmu04120(	PF18891.5(	GO:004438	cytoplasmic	NP_080199	mmu:67030	
K02998	RP-SAe, RP	mmu05171(	PF16122.10	GO:002260	cytoplasmic	ACB59248.	mmu:16785	
K20096	DDX28		PF00270.35	GO:002260	mitochondr	NP_082314	mmu:71986	
K11341	YEATS4, G	mmu03082(	PF03366.22	GO:003124	cytoplasmic	NP_080846	mmu:64050	TF_others
K10106	GGCX	mmu01240(	PF05090.19	GO:005138	mitochondr	NP_062776	mmu:56316	
K27476	PHF23		PF13831.12	GO:190187	cytoplasmic	NP_084340	mmu:78246	
K09489	HSPA4	mmu04530(	PF00012.26	GO:000810	cytoplasmic	BAD90352.	mmu:15525	
K20121	FNBP1		PF00611.28	GO:009951	nuclear	NP_001108	mmu:214459	
K08202	SLC22A4_5	mmu05231(	PF00083.30	GO:009859	cytoplasmic	NP_062661	mmu:30805	
K23624	PAPLN		PF19030.6(	GO:000417	mitochondr	NP_001192	mmu:170721	
K11854	USP35_38		PF21246.3(	GO:010100	cytoplasmic	NP_001170	mmu:244144	
K00884	NAGK, nag	mmu00520(	PF01869.26	GO:004858	cytoplasmic	NP_062415	mmu:56174	
K01772	hemH, FEC	mmu01240(	PF00762.24	GO:001922	cytoplasmic	NP_001334	mmu:14151	
K24524	CPNE1_2_3		PF07002.22	GO:001602	cytoplasmic	EDL11135.	mmu:234577	
K22751	CBFA2T2		PF07531.19	GO:006057	cytoplasmic	EDL06072.	mmu:12396	
K27832	MLIP		PF15274.11	GO:008009	nuclear	Q5FW52.2(	mmu:69642	
K22529	AMIGO		PF13855.12	GO:005113	plasma mer	NP_796249	mmu:320844	
K05722	FGD3	mmu04810(	PF00621.25	GO:002260	cytoplasmic	BAC34624.	mmu:30938	
K01262	pepP		PF00557.29	GO:000417	cytoplasmic	NP_573479	mmu:170750	
SUN(-)				GO:000579	Golgi appa	NP_001192	mmu:319996	
K06563	CLEC4L_M	mmu05162(	PF00059.27	GO:000989	plasma mer	NP_081248	mmu:69165	
IHU(Family with sequence similarity 173, mem				GO:000647	plasma mer	NP_080822	mmu:68073	
K10591	NEDD4, RS	mmu04530(	PF00632.30	GO:005084	cytoplasmic	NP_035020	mmu:17999	
K12861	BCAS2	mmu03040(	PF05700.16	GO:007100	nuclear	NP_005863	mmu:68183	

K24971	NEMF, RQC2		PF05670.18	GO:004323	nuclear	EDL36612.	mmu:66244	
K10508	ZBTB34		PF00651.37	GO:000097	cytoplasmic	BAD32599.	mmu:24131	ZBTB
K15631	ABA3	mmu00790(	PF00266.25	GO:003017	cytoplasmic	NP_081055	mmu:68591	
K01872	AARS, alaS	mmu00970(	PF01411.24	GO:000367	mitochondr	NP_941010	mmu:224805	
K07891	RAB22	mmu04144(	PF00071.28	GO:000810	cytoplasmic	NP_598446	mmu:106572	
K01672	CA	mmu00910(	PF00194.26	GO:000673	cytoplasmic	NP_078771	mmu:71934	
YN9K(tubulointerstitial nephritis)			PF00112.29	GO:000032	extracellulo	NP_001161	mmu:94242	
K22941	YIPF3, KLIP1			GO:003250	cytoplasmic	NP_663328	mmu:28064	
QCT(Ribosome binding factor A)			PF02033.23	GO:001607	mitochondr	NP_954667	mmu:68731	
K17269	MBP		PF01669.22	GO:004320	cytoplasmic	QBF67690.	mmu:17196	
K22040	TRPS1		PF00320.33	GO:200123	nuclear	NP_001297	mmu:83925	zf-GATA
ZJ6F(chromosome 7 open reading fr			PF18474.7(DUF5614:Fo		cytoplasmic	NP_001098	mmu:105351	
K03935	NDUFS2	mmu05208(	PF00346.24	GO:004423	mitochondr	NP_694704	mmu:226646	
K12836	U2AF1	mmu03040(	PF00642.30	GO:000613	nuclear	XP_004696	mmu:108121	
K00228	CPOX, hem	mmu01240(	PF01218.23	GO:003197	mitochondr	NP_031783	mmu:12892	
K11136	RTEL1		PF06733.21	GO:008009	nuclear	NP_001160	mmu:269400	
K20069	NECAP1_2		PF07933.20	GO:000810	cytoplasmic	NP_080543	mmu:67602	
)B8Q(e2F-associated phosphoprotei			PF10238.14	GO:004212	cytoplasmic	BAE26193.	mmu:66266	
K17255	GDI1_2		PF00996.24	GO:000548	cytoplasmic	BAE25494.	mmu:14569	
K23287	VIPAS39, VPS16B		PF04840.18	GO:000367	cytoplasmic	BAE26203.	mmu:104799	
XPDX(Calcium channel, voltage-de			PF02743.24	GO:000526	plasma mer	NP_932154	mmu:320508	
K02857	RHBDL1_2_3		PF01694.27	GO:000425	peroxisoma	NP_898986	mmu:230726	
K19375	HSPBAP1		PF13621.12	GO:000573	cytoplasmic	NP_780320	mmu:66667	
K18592	GGT1_5, C	mmu00590(	PF01019.26	GO:000663	lysosomal	NP_035950	mmu:23887	
XRZ8(Prefoldin, subunit)			PF02996.22	GO:002260	cytoplasmic	AAI32170.1	mmu:22327	
K05679	ABCG1	mmu02010(	PF01061.29	GO:003577	mitochondr	NP_033723	mmu:11307	
K00357	QDPR	mmu00790(	PF00106.31	GO:005128	mitochondr	NP_077198	mmu:110391	
K08489	STX16	mmu04130(	PF05739.25	GO:000810	cytoplasmic	NP_766263	mmu:228960	
K24975	METTL8		PF08242.18	GO:007013	cytoplasmic	NP_663499	mmu:228019	
K25093	SLTM		PF00076.28	GO:008009	nuclear	NP_079966	mmu:66660	
K08488	STX7	mmu04140(	PF14523.12	GO:000802	cytoplasmic	O70439.3(R	mmu:53331	
K05743	LIMK1	mmu05170(	PF07714.23	GO:004867	mitochondr	NP_001292	mmu:16885	
K02727	PSMA3	mmu05020(	PF00227.31	GO:004438	cytoplasmic	NP_001409	mmu:19167	
K07605	KRT2		PF00038.26	GO:009859	mitochondr	NP_112447	mmu:16691	
K27491	ENSA, ARPP19		PF04667.22	GO:009008	cytoplasmic	NP_062507	mmu:56205	
K14380	FHL2	mmu04380(	PF00412.28	GO:008009	cytoplasmic	NP_001276	mmu:14200	
K03257	EIF4A		PF00270.35	GO:003451	cytoplasmic	NP_001029	mmu:13681	
K14216	OAS	mmu05164(	PF10421.14	GO:009040	cytoplasmic	NP_660212	mmu:246730	
K05858	PLCB	mmu04750(	PF00388.25	GO:009008	cytoplasmic	AAX90608.	mmu:18795	
K16572	TUBGCP5, GCP5		PF04130.18	GO:000705	cytoplasmic	NP_666302	mmu:233276	
K05862	VDAC1	mmu05208(	PF01459.28	GO:004343	cytoplasmic	BAE31716.	mmu:22333	
XRFD(serine threonine kinase 11 in			PF15904.10	GO:000573	cytoplasmic	NP_082162	mmu:71728	
K00274	MAO, aofH	mmu00982(	PF01593.30	GO:008009	cytoplasmic	NP_766366	mmu:109731	

K03964	NDUFB8	mmu05208(	PF05821.16	GO:002260'	mitochondr	NP_080337	mmu:67264	
K04234	F2RL1, PAI	mmu04750(	PF00001.26	GO:000191(	plasma mer	NP_032000	mmu:14063	
K05293	PIGU	mmu00563(	PF06728.19	GO:000830:	extracellulo	NP_001004	mmu:228812	
K02152	ATPeV1G,	mmu05165(	PF03179.20	GO:009877	cytoplasmic	NP_077135	mmu:66290	
K20032	ZDHHC13_17, HIP14		PF12796.13	GO:004323	cytoplasmic	NP_766142	mmu:320150	
K11518	TOM40	mmu05014(	PF01459.28	GO:000810	cytoplasmic	BAE25112.	mmu:53333	
K08618	ADAMTS2		PF19030.6(	GO:000417	plasma mer	NP_783574	mmu:216725	
K18061	SBF1_2, MTMR5_13		PF12335.14	GO:001920	cytoplasmic	NP_001074	mmu:77980	
K06721	CLEC10A, CLECSF13,		PF03954.19	GO:000989'	plasma mer	XP_006532	mmu:216864	
K13916	CAMP, LL3	mmu04621(	PF00666.23	GO:001922	extracellulo	NP_034051	mmu:12796	
K20994	NOTCH2	mmu05165(	PF00008.33	GO:003510	extracellulo	NP_035058	mmu:18129	
K05858	PLCB	mmu04750(	PF08703.15	GO:004643	cytoplasmic	NP_001277	mmu:18797	
K14810	DDX56, DBP9		PF00270.35	GO:001922	nuclear	NP_080814	mmu:52513	
K04730	IRAK1	mmu04010(	PF00069.31	GO:190303'	cytoplasmic	NP_001171	mmu:16179	
K10752	RBBP4, HA	mmu03082(	PF00400.38	GO:004239	cytoplasmic	KAI454723	mmu:19646	
K13337	PEX19	mmu04146(	PF04614.17	GO:009859	peroxisoma	EDL39041.	mmu:19298	
K09131	K09131		PF02594.21	GO:000573'	cytoplasmic	AAH27500.	mmu:67290	
KRGS(response to pheromone)			PF10192.15	GO:001923	plasma mer	NP_067409	mmu:58245	
K07364	TEC	mmu04660(	PF07714.23	GO:000582	cytoplasmic	XP_006503	mmu:21682	
K12653	NLRX1	mmu04621(	PF05729.18	GO:001922	mitochondr	NP_001157	mmu:270151	
K10652	TRIM25, El	mmu05164(	PF00622.36	GO:003577	cytoplasmic	NP_033572	mmu:217069	
K00555	TRMT1, trm1		PF02005.22	GO:000817	mitochondr	NP_001158	mmu:212528	
K16591	HAUS8			GO:000705	cytoplasmic	NP_001156	mmu:76478	
K20179	VPS11, PEI	mmu04140(	PF23341.1(	GO:000810	cytoplasmic	NP_082165	mmu:71732	
K07414	CYP2D	mmu04726(	PF00067.27	GO:000449'	ER	AAH16256.	mmu:56448	
K17795	TIM17		PF02466.24	GO:000810	cytoplasmic	NP_035720	mmu:21854	
K24684	ZFAND1		PF01428.22	GO:003315'	cytoplasmic	EDL05161.	mmu:66361	
K13989	DERL2_3	mmu04141(	PF04511.20	GO:003315'	ER	NP_001278	mmu:116891	
K24940	CWF19L2		PF04677.20	GO:001607	nuclear	NP_081821	mmu:244672	
K20407	MIOS, MIC	mmu04150(	PF21720.4(	GO:190426	cytoplasmic	NP_663349	mmu:252875	
K18734	SMG8		PF10220.14	GO:000018	plasma mer	NP_077224	mmu:74133	
K02938	RP-L8e, RP	mmu05171(	PF03947.24	GO:002262	cytoplasmic	NP_000964	mmu:26961	
K14016	UFD1	mmu04141(	PF03152.21	GO:000810	cytoplasmic	NP_035802	mmu:22230	
K12171	SH3RF, POSH		PF00018.34	GO:000989	cytoplasmic	NP_766376	mmu:237353	
K17418	MRPL37		PF07147.18	GO:001593	mitochondr	NP_079776	mmu:56280	
K26097	KNSTRN, SKAP			GO:001056	cytoplasmic	AAH31709.	mmu:51944	
K09107	SREBP2, SREBF2		PF00010.31	GO:000578	nuclear	NP_150087	mmu:20788	bHLH
K02912	RP-L32e, R	mmu05171(	PF01655.23	GO:002262	cytoplasmic	NP_000985	mmu:19951	
K01257	LNPEP	mmu04614(	PF01433.26	GO:000417'	cytoplasmic	NP_766415	mmu:240028	
K03124	TFIIB, GTI	mmu05017(	PF00382.24	GO:000117	nuclear	NP_112303	mmu:229906	
K11446	KDM5, JARID1		PF08429.16	GO:008009	nuclear	NP_690855	mmu:75605	ARID
K16573	TUBGCP6, GCP6		PF19340.4(	GO:000705	cytoplasmic	NP_001156	mmu:328580	
K03029	PSMD4, RF	mmu05020(	PF13519.12	GO:002262	nuclear	NP_001268	mmu:19185	

K10798	PARP3_4	mmu04210(	PF00644.27	GO:009951	cytoplasmic	NP_001139	mmu:328417	
K18212	SNAP47			GO:000690	cytoplasmic	NP_001343	mmu:67826	
H9K(Craniofacial development pro			PF07572.17	GO:002260	nuclear	NP_035931	mmu:23837	
netalloendopeptidase activity)			PF01416.25	GO:003111	mitochondr	NP_001028	mmu:433813	
K24457	SLFN12, SLFN12L		PF17057.10	GO:004213	cytoplasmic	NP_035537	mmu:20555	
K13882	CORO1A	mmu04145(	PF08954.16	GO:000688	cytoplasmic	NP_001288	mmu:12721	
K02358	tuf, TUFM		PF00009.33	GO:004518	cytoplasmic	XP_036008	mmu:233870	
K18264	ITM2B		PF04089.19	GO:001707	cytoplasmic	NP_032436	mmu:16432	
K23337	MKLN1		PF06588.17	GO:002260	mitochondr	NP_038819	mmu:27418	
K20223	IPO7, RAN	mmu03013(	PF03810.25	GO:003450	cytoplasmic	NP_852658	mmu:233726	
K22659	CLSTN1		PF19699.4((	GO:000810	plasma mer	NP_075538	mmu:65945	
)2YK(methyltransferase like 9)			PF05219.18	GO:000578	ER	NP_067529	mmu:59052	
K23293	RMDN3, PTPIP51		PF21033.3(I	GO:003250	ER	NP_001028	mmu:67809	
K04450	ATF2, CRE	mmu05020(	PF00170.26	GO:000959	nuclear	NP_001020	mmu:11909	TF_bZIP
K14163	EPRS	mmu01240(	PF00749.27	GO:001710	cytoplasmic	NP_084011	mmu:107508	
K01231	MAN2	mmu00510(	PF01074.27	GO:001679	Golgi appa	NP_766491	mmu:140481	
K14753	RACK1	mmu05162(	PF00400.38	GO:200123	cytoplasmic	NP_001004	mmu:14694	
K18749	LSM14, RAP55, SCD6		PF12701.13	GO:008009	cytoplasmic	NP_808395	mmu:241846	
K24515	ITIH5		PF06668.17	GO:003020	extracellulo	NP_766059	mmu:209378	
K13193	RBM27		PF01480.22(PWI:PWI d		nuclear	NP_001395	mmu:225432	
K13965	SERPINB8		PF00079.25	GO:000573	cytoplasmic	NP_035589	mmu:20725	
K03386	PRDX2_4, ahpC		PF00578.27	GO:001649	cytoplasmic	NP_058044	mmu:53381	
K13963	SERPINB	mmu05146(	PF00079.25	GO:000989	cytoplasmic	NP_035584	mmu:20708	
K17079	OPA1	mmu04137(	PF19434.4((	GO:200123	cytoplasmic	NP_001390	mmu:74143	
K11976	RNF216, TRIAD3		PF22191.2(I	GO:005068	cytoplasmic	NP_542128	mmu:108086	
K12166	HACE1		PF00632.30	GO:000020	cytoplasmic	NP_766061	mmu:209462	
K00911	ITPK	mmu04070(	PF03770.22	GO:007124	cytoplasmic	NP_853624	mmu:233011	
K10571	DET1	mmu04120(	PF09737.14	GO:000989	nuclear	NP_083861	mmu:76375	
K26498	TMEM147		PF09767.14	GO:003450	cytoplasmic	XP_036009	mmu:69804	
K13089	ILF2		PF07528.20	GO:008009	cytoplasmic	BAE22574.	mmu:67781	
K07756	IP6K, IHPK	mmu04070(	PF03770.22	GO:000548	cytoplasmic	BAD32199.	mmu:27399	
K13362	FXYD5, DYSAD		PF02038.21	GO:009910	plasma mer	XP_021022	mmu:18301	
K12406	PKLR	mmu04910(	PF00224.27	GO:004643	cytoplasmic	NP_001093	mmu:18770	
XNPC(chromosome 19 open reading			PF09746.14	GO:000989	ER	NP_001003	mmu:216157	
K23804	GON4L		PF21227.3(I	GO:008009	nuclear	NP_081665	mmu:76022	
K21768	TBCE		PF01302.31	GO:002260	cytoplasmic	NP_848027	mmu:70430	
K07195	EXOC7, EX	mmu05132(	PF03081.21	GO:000548	cytoplasmic	NP_001334	mmu:53413	
K21127	S100A8	mmu04657(	PF01023.25	GO:009008	cytoplasmic	NP_038678	mmu:20201	
K24412	CRIP1, CRP1		PF00412.28	GO:007148	cytoplasmic	EDL18557.	mmu:12925	
K16362	FLRT		PF13855.12	GO:005113	ER	BAE23193.	mmu:399558	
K02263	COX4	mmu05208(	PF02936.19	GO:001545	mitochondr	NP_444321	mmu:84682	
K08964	mtnB	mmu00270(	PF00596.26	GO:005128	cytoplasmic	NP_062709	mmu:56369	
K19325	ZNF592		PF16622.11	GO:001922	nuclear	BAE25468.	mmu:233410	

K17770	TOM20	mmu04137(	PF02064.20	GO:000810	peroxisoma	NP_077176	mmu:67952	
Y78B(myeloid cell nuclear differenti			PF02760.21	GO:003210	nuclear	ASD34087.1	mmu:226691	
K26998	DTNA	mmu05410(	PF09068.17	GO:001989	cytoplasmic	EDK96979.	mmu:13527	
K18655	DDX19, DI	mmu03013(	PF00270.35	GO:001593	cytoplasmic	NP_758488	mmu:234733	
K02937	RP-L7e, RP	mmu05171(	PF08079.17	GO:004280	nuclear	NP_035421	mmu:19989	
K08617	ADAMTS1		PF05986.19	GO:000827	cytoplasmic	BAA11088.	mmu:11504	
K13524	ABAT	mmu00650(	PF00202.27	GO:009008	mitochondr	NP_766549	mmu:268860	
K00736	MGAT2	mmu00510(	PF05060.20	GO:000931	Golgi appa	BAE34882.1	mmu:217664	
K20472	COPZ, RET3		PF01217.26	GO:000810	cytoplasmic	NP_001101	mmu:56447	
K05865	TNNC1	mmu04260(	PF13499.12	GO:009951	cytoplasmic	XP_038628	mmu:21924	
K25661	SERINC3	mmu03250(	PF03348.21	GO:000959	Golgi appa	NP_036162	mmu:26943	
K20820	BORCS6		PF10157.14	GO:000701	cytoplasmic	NP_082281	mmu:71923	
K04739	PRKAR	mmu04910(	PF00027.35	GO:009008	cytoplasmic	NP_001300	mmu:19084	
K19530	JPH		PF02493.26	GO:008009	nuclear	NP_001192	mmu:59091	
K06551	SIRPA_B1_	mmu04148(	PF07654.21	GO:001989	plasma mer	EDL28243.	mmu:19261	
K15031	MTERF		PF02536.19	GO:008009	mitochondr	NP_001366	mmu:74238	
K22029	PALLD		PF07679.22	GO:003243	cytoplasmic	AFS88929.1	mmu:72333	
K02891	RP-L22e, R	mmu05171(	PF01776.22	GO:001593	nuclear	NP_001180	mmu:19934	
K19475	WIPF	mmu05135(	PF02205.27	GO:003315	cytoplasmic	NP_001276	mmu:215280	
K24539	YEATS2		PF22951.1(	GO:003124	nuclear	XP_006521	mmu:208146	
K00844	HK	mmu00052(	PF03727.22	GO:004643	cytoplasmic	BAE24390.1	mmu:212032	
K11837	USP6_32		PF00443.35	GO:190343	cytoplasmic	KFQ86608.	mmu:237898	
K24517	BMPER		PF00094.30	GO:004858	extracellulo	NP_082748	mmu:73230	
K06266	CD47	mmu04148(	PF04549.19	GO:001922	extracellulo	NP_001355	mmu:16423	
K00134	GAPDH, g	mmu01100(	PF02800.25	GO:004643	cytoplasmic	NP_001276	mmu:14433	
K18160	NDUFAF2	mmu04714(	PF05071.21	GO:009008	cytoplasmic	NP_001120	mmu:75597	
)856(kiaa1524)			PF21044.3(	GO:000548	cytoplasmic	EDK98105.	mmu:224171	
K06691	RPN13	mmu05020(	PF04683.19	GO:006000	cytoplasmic	EDL00700.	mmu:56436	
K22734	ANKH, SLC62A1		PF07260.16	GO:006024	extracellulo	NP_065065	mmu:11732	
K09230	SCAN		PF02023.22	GO:008009	nuclear	NP_001139	mmu:72667	zf-C2H2
K21112	SYNPO	mmu04530(	PF05556.16	GO:003243	cytoplasmic	XP_006525	mmu:104027	
K00129	ALDH3	mmu00980(	PF00171.27	GO:000403	cytoplasmic	NP_001170	mmu:621603	
Z5I9(TLC domain containing)			PF03798.21	GO:004259	ER	NP_080984	mmu:68385	
K00485	FMO	mmu00982(	PF00743.25	GO:000449	cytoplasmic	NP_001317	mmu:14261	
Transmembrane amino acid transpo			PF13465.12	GO:008009	nuclear	NP_659544	mmu:11242	zf-C2H2
K20313	PLEKHA3, FAPP1		PF00169.35	GO:001602	cytoplasmic	NP_112546	mmu:83435	
K01930	FPGS	mmu01240(	Biosynthesis	GO:000652	mitochondr	NP_034366	mmu:14287	
K11108	RCL1	mmu03008(	PF01137.26	GO:003204	mitochondr	NP_067500	mmu:59028	
.36R(This is a non-secretory ribonuc			PF00074.25	GO:000961	extracellulo	NP_031921	mmu:13587	
.36R(This is a non-secretory ribonuc			PF00074.25	GO:000961	extracellulo	NP_031920	mmu:13586	
RNA polymerase I CORE element s			PF01979.26	GO:003250	cytoplasmic	NP_031791	mmu:12933	
K17093	ANXA4		PF00191.25	GO:009859	cytoplasmic	NP_001318	mmu:11746	
K14402	CPSF2, CF	mmu03015(	PF16661.11	GO:000833	cytoplasmic	NP_058552	mmu:51786	

K10667	RNF8		PF01585.28	GO:000817	cytoplasmic	BAD32269.	mmu:58230	
K13644	CEPT1	mmu00440(	PF01066.26	GO:000665	plasma mer	NP_001344	mmu:99712	
K23326	CCNK		PF00134.28	GO:200073	nuclear	O88874.3(R	mmu:12454	
K23341	ERLIN		PF01145.30	GO:008009	ER	NP_001157	mmu:226144	
K19481	MGP			GO:002260	extracellulo	NP_032623	mmu:17313	
K12965	ZBP1, DAI	mmu04623(	PF02295.23	GO:004858	nuclear	NP_067369	mmu:58203	
K24687	ZFAND5_6		PF01754.22	GO:000548	cytoplasmic	NP_033577	mmu:22682	
K02084	APAF1	mmu05164(	PF00400.38	GO:200123	cytoplasmic	NP_001036	mmu:11783	
K14550	UTP10, HE	mmu03008(	PF23243.1(I	GO:003204	mitochondr	NP_659084	mmu:217995	
K18418	ERI3, PINT1		PF00929.31	GO:001679	extracellulo	NP_536717	mmu:140546	
MAK16 homolog (S. cerevisiae))			PF01722.23	GO:002260	cytoplasmic	NP_780312	mmu:66162	
K27396	SMG		PF00536.37	GO:000548	nuclear	NP_778186	mmu:233033	
K09228	KRAB		PF01352.32	GO:008009	cytoplasmic	NP_001035	mmu:66869	
K18966	GADL1, CS	mmu00410(	PF00282.25	GO:000548	cytoplasmic	NP_001346	mmu:246277	
K01855	PUS3, DEG1		PF01416.25	GO:003111	nuclear	BAB27651.1	mmu:67049	
K02950	RP-S12, MR	mmu03010(	PF00164.32	GO:001593	mitochondr	NP_001347	mmu:24030	
K18977	HPX		PF00045.24	GO:001922	lysosomal	NP_059067	mmu:15458	
K13510	LPCAT1_2	mmu00564(	PF01553.26	GO:001922	Golgi appa	NP_663351	mmu:210992	
K23216	SETD5		PF00856.33	GO:190227	nuclear	EDK99440.	mmu:72895	
K12590	RRP46, EX	mmu03018(	PF01138.27	GO:001922	nuclear	NP_613052	mmu:27998	
KS48(FGFR1 oncogene partner 2)			PF05769.16	GO:000573	cytoplasmic	NP_080494	mmu:67529	
K07750	MESO1, EF	mmu00100(	PF04116.19	GO:000548	cytoplasmic	NP_079712	mmu:66234	
K15620	GOLPH3, GPP34		PF05719.16	GO:000810	mitochondr	XP_004583	mmu:66629	
K07554	ATPeF0S, ATP5S			GO:000925	mitochondr	NP_001348	mmu:68055	
K19179	GDE1		PF03009.23	GO:000548	extracellulo	NP_062526	mmu:56209	
K12893	SRSF4_5_6,	mmu03040(	PF00076.28	GO:000971	nuclear	VFV30768.	mmu:20384	
K20367	ERGIC3, ERV46		PF07970.17(COPIIcoat		cytoplasmic	NP_001343	mmu:66366	
K10574	UBE2B, RA	mmu04120(	PF00179.31	GO:005123	cytoplasmic	NP_001032	mmu:22210	
K27703	SPINDOC		PF18658.7(z	GO:000989	nuclear	NP_001028	mmu:68229	
K07859	RND3		PF00071.28	GO:003304	cytoplasmic	NP_001007	mmu:74194	
K12348	ASAH1	mmu04071(	PF02275.23	GO:008009	extracellulo	BAE31879.	mmu:11886	
K16766	CEP85		PF24555.1((	GO:003367	cytoplasmic	EDL30030.	mmu:70012	
K18449	PRUNE2, BMCC1		PF12496.13	GO:000573	cytoplasmic	NP_851993	mmu:353211	
K02735	PSMB3	mmu05020(	PF00227.31	GO:000417	cytoplasmic	NP_036101	mmu:26446	
K18083	MTMR6_7_	mmu04070(	PF06602.19	GO:009859	cytoplasmic	NP_659092	mmu:219135	
K13823	HBB	mmu05144(	PF00042.28	GO:000534	cytoplasmic	ABU63204.	mmu:15129	
K08835	OXSR1, ST	mmu04928(	PF00069.31	GO:001922	cytoplasmic	EDL27018.	mmu:53416	
K20878	NEK9, NERCC1		PF00069.31	GO:001920	cytoplasmic	BAD32601.	mmu:217718	
K15102	SLC25A3, PHC, PIC		PF00153.32	GO:000531	mitochondr	XP_029398	mmu:18674	
K11417	SIRT7, SIR	mmu00760(	PF02146.23	GO:003315	nuclear	NP_694696	mmu:209011	
K09228	KRAB		PF13465.12	GO:000097	cytoplasmic	BAC26260.	mmu:20829	zf-C2H2
K13781	SLC7A8, LA	mmu04974(	PF13520.12	GO:009859	plasma mer	NP_058668	mmu:50934	
K10263	FBXW5		PF00400.38	GO:199023	cytoplasmic	NP_038936	mmu:30839	

K10272	FBXL6		PF12937.12	GO:199023	nuclear	NP_038937	mmu:30840	
K15686	MEX3, RKHD		PF00013.35	GO:004858	cytoplasmic	NP_001034	mmu:240396	
K00752	hasA		PF13641.12	GO:000367	ER	AAI25425.1	mmu:15116	
K08851	TP53RK, PRPK, BUD3		PF06293.20	GO:004316	cytoplasmic	NP_001007	mmu:381406	
K09488	TRAP1, HS	mmu05022(	PF00183.23	GO:200123	mitochondr	BAE39316.1	mmu:68015	
K01415	ECE		PF05649.18	GO:004423	cytoplasmic	NP_001356	mmu:230857	
K12405	HSD17B4	mmu00120(	PF22622.2(	GO:003444	peroxisoma	NP_032318	mmu:15488	
K01488	add, ADA	mmu01100(	PF00962.28	GO:004858	cytoplasmic	NP_001258	mmu:11486	
K06535	BSG, CD14	mmu04148(	PF13927.12	GO:000810	cytoplasmic	EDL31675.	mmu:12215	
K24054	GOPC		PF00595.29	GO:001990	cytoplasmic	NP_001186	mmu:94221	
K10351	MYL2	mmu04530(	PF13499.12	GO:009951	cytoplasmic	NP_001371	mmu:17906	
K08122	LUM	mmu05205(	PF13855.12	GO:005125	extracellula	NP_032550	mmu:17022	
K04392	RAC1	mmu05208(	PF00071.28	GO:003577	cytoplasmic	NP_001003	mmu:19353	
K05863	SLC25A4S,	mmu05208(	PF00153.32	GO:200123	cytoplasmic	NP_031477	mmu:11740	
K04451	TP53, P53	mmu05200(	PF00870.23	GO:004423	nuclear	CAI52016.1	mmu:22059	P53
K20192	HPS6		PF20468.4(I	GO:000810	mitochondr	NP_789742	mmu:20170	
K01868	TARS, thrS	mmu00970(	PF00587.30	GO:000643	cytoplasmic	NP_758514	mmu:272396	
K24375	ZNF687		PF13894.12	GO:000557	nuclear	NP_001344	mmu:78266	zf-C2H2
K06793	VCAN, CSI	mmu04514(	PF00193.23	GO:004867	plasma mer	EDL00982.	mmu:13003	
K17360	ACOT7	mmu01040(	PF03061.28	GO:004643	mitochondr	EDL14930.	mmu:70025	
K06832	LGALS8		PF00337.29	GO:190303	cytoplasmic	NP_001277	mmu:56048	
K11422	SETD1, SE'	mmu00310(	PF00856.33	GO:001922	cytoplasmic	NP_821172	mmu:233904	
K06528	LAMP1_2, (	mmu04140(	PF01299.22	GO:007266	extracellula	NP_001017	mmu:16784	
K11406	HDAC4_5	mmu04371(	PF00850.24	GO:008009	cytoplasmic	NP_001271	mmu:15184	
K15049	ENDOD1			GO:000367	cytoplasmic	BAD32314.	mmu:71946	
K02729	PSMA5	mmu05020(	PF00227.31	GO:001977	cytoplasmic	BAE35848.1	mmu:26442	
K08285	TRIM18, M	mmu04120(	PF18568.6((	GO:003111	cytoplasmic	XP_017173	mmu:17318	
Dehydrogenase)			PF13637.12	GO:004323	cytoplasmic	NP_001344	mmu:68839	
K00966	GMPP	mmu01240(	PF25087.1((	GO:001967	cytoplasmic	NP_001344	mmu:331026	
.NKT(glycolipid transfer protein do			PF08718.16	GO:001922	cytoplasmic	NP_077792	mmu:79554	
K11585	CBX1, HP1B, SWI6		PF01393.25	GO:000989	nuclear	XP_003466	mmu:12412	
K19685	TTC26, IFT56, DYF13		PF14559.12	GO:000367	cytoplasmic	NP_705828	mmu:264134	
nuclear pore complex protein)			PF21276.3(	GO:000097	nuclear	NP_001158	mmu:26940	zf-C2H2
K08849	MLKL	mmu04623(	PF22215.1(	GO:002260	cytoplasmic	EDL11495.	mmu:74568	
K01517	ADPRM	mmu00564(	PF00149.33	GO:000548	cytoplasmic	NP_079786	mmu:66358	
K13711	PI4K2	mmu04070(	PF00454.33	GO:001707	cytoplasmic	NP_080227	mmu:67073	
K16527	AKAP11			GO:000810	nuclear	NP_001157	mmu:219181	
K15323	TSEN34		PF01974.23	GO:000815	nuclear	NP_001406	mmu:66078	
K10476	KBTBD11		PF01344.31(Kelch_1:Ke		cytoplasmic	NP_083392	mmu:74901	ZBTB
K26547	MPEG1		PF01823.24	GO:003067	lysosomal	NP_001361	mmu:17476	
K23496	NGRN		PF06413.17	GO:002260	nuclear	NP_113552	mmu:83485	
K00820	glmS, GFPT	mmu01250(	PF01380.27	GO:005089	cytoplasmic	NP_038557	mmu:14584	
K06553	VPREB, CD179a		PF07654.21	GO:000548	plasma mer	BAB25349.1	mmu:22362	

K03961	NDUFB5	mmu05208(	PF09781.14	GO:002260'	mitochondr	BAC40123.1	mmu:66046	
K00121	frmA, ADH	mmu00980(	PF00107.32	GO:003444(	cytoplasmic	NP_031436	mmu:11532	
K10393	KIF2_24, M	mmu04814(	PF00225.29	GO:009951:	cytoplasmic	BAC40116.1	mmu:73804	
K01014	SULT1A	mmu05204(	PF00685.33	GO:004428'	cytoplasmic	NP_598431	mmu:20887	
K02962	RP-S17e, Rl	mmu05171(	PF00833.23	GO:003204(	cytoplasmic	NP_001001	mmu:20068	
K24495	MYOM	mmu04820(	PF00041.27	GO:000520(	mitochondr	NP_001401	mmu:17930	
K11406	HDAC4_5	mmu04371(	PF00850.24	GO:001922(	cytoplasmic	NP_997108	mmu:208727	
K10864	PMS1		PF01119.25	GO:003238'	nuclear	EDK99987.	mmu:22709	HMG
K18587	COQ9		PF08511.16	GO:004218(	mitochondr	NP_080728	mmu:67914	
ransmembrane protein 205)			PF00515.34(	TPR_1:Tetr	ER	NP_001360	mmu:234875	
K12391	AP1G1	mmu04142(	PF01602.26	GO:000810	cytoplasmic	NP_031481	mmu:11766	
K04345	PKA	mmu04750(	PF00069.31	GO:000858'	cytoplasmic	NP_001071	mmu:18749	
K04971	TRPV2	mmu04621(	PF12796.13	GO:000526'	cytoplasmic	NP_001369	mmu:22368	
K04407	MAP4K4, F	mmu04010(	PF00069.31	GO:009008'	nuclear	NP_032722	mmu:26921	
K23625	ATXN2_2L	mmu05014(	PF14438.12	GO:000548	cytoplasmic	O70305.1(R	mmu:20239	
K00981	E2.7.7.41, C	mmu04070(	PF01148.25	GO:000367	ER	NP_001277	mmu:110911	
27ER(p53 and DNA-damage regula			PF01920.25	GO:000573'	cytoplasmic	NP_849270	mmu:68559	
K01201	GBA, srfJ	mmu04142(	PF02055.22	GO:190306	lysosomal	NP_001070	mmu:14466	
K18085	MTMR10_11_12		PF06602.19	GO:001679'	cytoplasmic	NP_766330	mmu:233315	
K27075	NEPRO		PF14780.11	GO:004858	nuclear	NP_666084	mmu:212547	
K25876	METTL15		PF01795.25	GO:003116'	cytoplasmic	NP_001405	mmu:76894	
K26403	CIAO2, CIA2, FAM96		PF01883.24	GO:002260'	extracellulo	NP_080911	mmu:68250	
.5RF(Envelope)			PF00429.24	GO:004316'	cytoplasmic	AXH06262.	mmu:118568683	
K01587	PAICS	mmu00230(	PF01259.24	GO:000618'	cytoplasmic	NP_001343	mmu:67054	
K20040	HIP1R, SLA2		PF07651.22	GO:005104(	cytoplasmic	AAH65101.	mmu:29816	
2CB1(Proline rich 11)				GO:000573'	nuclear	NP_780772	mmu:270906	
)MGQ(kiaa0494)				GO:004316'	nuclear	EDL30638.	mmu:230648	
K00922	PIK3CA_B	mmu05208(	PF00613.26	GO:004423	cytoplasmic	NP_032865	mmu:18706	
K10375	TPM4	mmu04260(	PF00261.25	GO:003243	cytoplasmic	NP_001001	mmu:326618	
.MUA(chromosome 2 open reading frame 47)				GO:000367	peroxisoma	NP_001074	mmu:68115	
K25827	CCAR1		PF14443.11	GO:004212'	cytoplasmic	NP_080477	mmu:67500	
KR70(Ferric-chelate reductase)			PF02014.22	GO:005080	lysosomal	NP_001106	mmu:20321	
K01319	CTSG	mmu05146(	PF00089.32	GO:000425	plasma mer	NP_031826	mmu:13035	
K19371	DNAJC25		PF00226.36	GO:000998'	ER	NP_001028	mmu:72429	
K27833	ZNHIT6		PF04438.22	GO:003303(	nuclear	EDL12002.	mmu:229937	
K00079	CBR1	mmu00980(	PF00106.31	GO:000810(	mitochondr	NP_031646	mmu:12408	
K07151	STT3	mmu04141(	PF02516.20	GO:199023	Golgi appa	NP_077184	mmu:68292	
K26410	SARAF		PF06682.17	GO:004423	plasma mer	AAH13497.	mmu:67887	
K20195	MON1	mmu04137(	PF19036.6(I	GO:000810	cytoplasmic	NP_082645	mmu:72825	
K23471	SKAP2	mmu05135(	PF00169.35	GO:004211	cytoplasmic	NP_061243	mmu:54353	
K05703	FYN	mmu04664(	PF07714.23	GO:009951	cytoplasmic	XP_036011	mmu:14360	
K12261	HACL1	mmu04146(	PF00205.28	GO:003444(	cytoplasmic	EDL24815.	mmu:56794	
XPJ1(c-type lectin domain family)			PF08391.16	GO:001602(	plasma mer	ABK00082.	mmu:16633	

K00289	ALDH1L	mmu00670(	PF00171.27	GO:000403	cytoplasmic	NP_001343	mmu:107747	
K02503	HINT1_2, hinT, hit		PF01230.28	GO:000989	mitochondr	NP_081147	mmu:68917	
K02105	CTNNB1	mmu05022(	PF00514.29	GO:000810	cytoplasmic	NP_001159	mmu:12387	
K09042	BACH		PF00651.37	GO:000097	nuclear	NP_031546	mmu:12013	ZBTB
K03020	RPAC2, RP	mmu04623(	PF13656.11	GO:003088	cytoplasmic	NP_033113	mmu:20018	
K07893	RAB6A		PF00071.28	GO:000647	cytoplasmic	NP_001157	mmu:19346	
K04529	APBB1, FE	mmu05010(	PF00640.29	GO:008009	cytoplasmic	XP_017177	mmu:11785	
K24553	DPY19L		PF10034.15	GO:003109	plasma mer	EDL05652.	mmu:381510	
K23930	FAM83		PF07894.18	GO:190248	cytoplasmic	NP_848733	mmu:69640	
K06096	VAPA	mmu04979(	PF00635.32	GO:000810	cytoplasmic	NP_038961	mmu:30960	
K23680	PQLC3, SLC66A3		PF04193.19	GO:000557	plasma mer	BAC40802.	mmu:217430	
K24945	ZC3H7		PF00642.30	GO:001922	cytoplasmic	NP_001074	mmu:20286	
K14561	IMP4	mmu03008(	PF04427.23	GO:003204	nuclear	NP_001009	mmu:27993	
K01672	CA	mmu00910(	PF00194.26	GO:000548	cytoplasmic	BAE38755.	mmu:230099	
K08878	BCR1, BCR	mmu05200(	PF00620.32	GO:000810	nuclear	NP_001074	mmu:110279	
K02256	COX1	mmu05208(	PF00115.25	GO:001602	ER	AHG32255.	mmu:17708	
K00289	ALDH1L	mmu00670(	PF00171.27	GO:004323	mitochondr	NP_705771	mmu:216188	
K04797	pfdA, PFDN5		PF02996.22	GO:003250	cytoplasmic	NP_081320	mmu:56612	
K00831	serC, PSAT	mmu01240(	PF00266.25	GO:000548	cytoplasmic	BAE26305.	mmu:107272	
29DZ(pigeon homolog (Drosophila)			PF14959.11	GO:003425	extracellulo	NP_001346	mmu:212167	
K13887	CORO2		PF08953.17	GO:000557	cytoplasmic	NP_849224	mmu:107684	
K20165	TBC1D2		PF00566.25	GO:004308	cytoplasmic	NP_941066	mmu:381605	
K06254	AGRN	mmu04512(	PF00054.30	GO:003303	extracellulo	NP_067617	mmu:11603	
K12849	PRPF38A	mmu03040(	PF03371.20	GO:000613	nuclear	NP_766285	mmu:230596	
K18439	ARAP1	mmu04144(	PF00620.32	GO:008009	cytoplasmic	NP_001394	mmu:69710	
K10454	KLHL17		PF01344.31	GO:000989	cytoplasmic	XP_038965	mmu:23100	ZBTB
K24750	WDR55, JIP5		PF24796.1(	GO:009030	nuclear	NP_080740	mmu:67936	
K16759	CCDC102		PF01576.24	GO:000557	cytoplasmic	NP_001028	mmu:234582	
K01099	INPP5B_F	mmu04070(	PF22669.2(	GO:008009	cytoplasmic	SPT35765.1	mmu:16330	
K14410	ACP2	mmu00740(	PF00328.27	GO:001679	lysosomal	NP_001343	mmu:11432	
K04959	ITPR2	mmu04750(	PF08709.17	GO:000526	cytoplasmic	NP_064307	mmu:16439	
K20463	OSBPL3_6_7, ORP3_6_		PF01237.23	GO:005104	cytoplasmic	BAD90510.	mmu:99031	
K07937	ARF1_2	mmu04144(	PF00025.27	GO:000810	cytoplasmic	NP_001229	mmu:11841	
K05422	CTF1, CT1	mmu04060(Cytokine-cy		GO:000512	cytoplasmic	NP_001288	mmu:13019	
)T3P(kiaa1462)			PF15351.11	GO:000828	cytoplasmic	NP_001075	mmu:240185	
K06066	CIR	mmu04330(	PF10197.14	GO:005125	nuclear	BAB24488.1	mmu:66935	
K17923	SNX9_18_3	mmu05132(	PF10456.14	GO:003608	cytoplasmic	EDL18387.	mmu:170625	
K21754	KCTD1_15		PF02214.28	GO:002260	mitochondr	KAI258641	mmu:106931	
K20164	DENND5		PF02141.26	GO:004214	cytoplasmic	NP_001298	mmu:19347	
K12046	TNNT3	mmu04814(	PF00992.25	GO:003103	cytoplasmic	NP_035750	mmu:21957	
K03139	TFIIF2, GT	mmu03022(	PF02270.21	GO:003280	nuclear	NP_081092	mmu:68705	
K06526	ENG, CD105		PF23344.1(	GO:001701	plasma mer	NP_001139	mmu:13805	
K19831	IFITM1, CI	mmu04662(	PF04505.17	GO:001922	cytoplasmic	NP_001106	mmu:68713	

K17424	MRPL43		PF05047.22	GO:004439	mitochondr	NP_444394	mmu:94067		
K05657	ABCB10	mmu02010(	PF00664.28	GO:008009	peroxisoma	NP_062425	mmu:56199		
Z5MV(Divergent AAA domain)			PF21026.2(S	GO:003563	cytoplasmic	AAP30074.1	mmu:327978		
K03115	CSNK2B	mmu04137(	PF01214.23	GO:003292	cytoplasmic	CCI79673.1	mmu:13001		
K14962	WDR82, SW	mmu03015(	PF24814.1(V	GO:000367	cytoplasmic	NP_001098	mmu:77305		
K23961	EPB41L1_2_3		PF05902.18	GO:003003	nuclear	XP_017172	mmu:13823		
K10293	FBXO7		PF11566.13	GO:200123	cytoplasmic	NP_694875	mmu:69754		
K15588	NSD1	mmu00310(	PF00855.23	GO:001922	nuclear	NP_032765	mmu:18193		
K08105	D4ST1	mmu00532(	PF03567.20	GO:003020	Golgi appa	NP_082393	mmu:72136		
K13110	MFAP1		PF06991.16	GO:009951	nuclear	NP_001075	mmu:67532		
K22760	CXXC5			GO:000832	cytoplasmic	BAC25113.1	mmu:67393		
K11992	ACOT8, PT	mmu00120(	PF02551.21	GO:000578	peroxisoma	NP_573503	mmu:170789		
K08857	NEK1_4_5		PF00069.31	GO:004316	extracellulo	XP_011243	mmu:23955		
K14292	TGS1		PF09445.16	GO:007116	nuclear	NP_473430	mmu:116940		
K22460	P3H3		PF23557.1(T	GO:000578	ER	NP_038562	mmu:14789		
K20187	BLOC1S5		PF14942.11	GO:009951	cytoplasmic	BAE42512.1	mmu:17828		
K09259	ANK3, AN	mmu05205(	PF17809.6(U	GO:003202	nuclear	CAH19222.	mmu:11735		
K07886	RAB27B	mmu04972(	PF00071.28	GO:000810	cytoplasmic	NP_001076	mmu:80718		
K26159	PRKRIP1		PF06658.17	GO:003367	nuclear	BAB26879.1	mmu:66801		
K25803	MALSU1		PF02410.21	GO:000989	mitochondr	NP_083629	mmu:75593		
K10496	ZBTB9		PF00651.37	GO:000097	nuclear	NP_001005	mmu:47415	ZBTB	
K18646	ANP32A_C_D		PF12799.13	GO:005116	cytoplasmic	NP_001398	mmu:11737		
K22696	EEF2KMT		PF14904.11	GO:000647	cytoplasmic	BAB30795.3	mmu:70511		
K23501	SFXN2		PF03820.22	GO:014030	cytoplasmic	NP_444426	mmu:94279		
K13141	INTS4		PF24493.1(I	GO:009030	nuclear	NP_081532	mmu:101861		
K17362	ACOT13		PF03061.28	GO:000573	cytoplasmic	NP_080066	mmu:66834		
K10349	FEM1B		PF12796.13	GO:200123	cytoplasmic	AAF05315.1	mmu:14155		
K23610	WDR41	mmu04140(	PF00400.38	GO:004592	cytoplasmic	NP_766178	mmu:218460		
K09595	HM13		PF04258.18	GO:000417	ER	NP_034506	mmu:14950		
XPR4(extended synaptotagmin-like p			PF17047.11	GO:001989	cytoplasmic	NP_083007	mmu:52635		
K14840	NOP53, GLTSCR2		PF07767.16	GO:009008	nuclear	NP_598592	mmu:68077		
K24970	TCF25, RQC1		PF04910.20	GO:190187	nuclear	NP_001273	mmu:66855		
K00236	SDHC, SDH	mmu05208(	PF01127.27	GO:000925	mitochondr	AAH83091.	mmu:66052		
K04505	PSEN1, PS1	mmu05165(	PF01080.22	GO:003042	plasma mer	NP_001349	mmu:19164		
K24814	HEATR5, LAA1		PF20210.4(I	GO:000810	cytoplasmic	NP_796145	mmu:320487		
K24220	MYH1s	mmu04814(	PF00063.27	GO:001645	cytoplasmic	NP_109604	mmu:17879		
XNUB(Midnolin)			PF00240.29	GO:009008	nuclear	NP_001334	mmu:59090		
K09228	KRAB		PF01352.32	GO:008009	extracellulo	AAP34297.1	mmu:224691		
K24503	SP110		PF03172.18	GO:008009	nuclear	NP_084470	mmu:10903	SAND	
K22559	COMMD3, BUP		PF21672.2(C	GO:000573	cytoplasmic	NP_680087	mmu:12238		
K18666	ASCC1		PF10469.15	GO:008009	nuclear	NP_001186	mmu:69090		
K13421	UMPS	mmu01240(	PF00215.30	GO:000921	cytoplasmic	NP_033497	mmu:22247		
K27704	MTCL		PF11365.13	GO:009859	cytoplasmic	NP_766551	mmu:68617		

YDBT(Family with sequence similar			PF14944.11	GO:000573	cytoplasmic	XP_023607	mmu:214469	
K06751	MHC1	mmu05165(	PF00129.23	GO:009857	extracellulo	NP_034510	mmu:14964	
K21410	C1QTNF12, FAM132A			GO:003577	mitochondr	NP_080401	mmu:67389	
K03121	TFII-I, GTI	mmu03022(	PF02946.19	GO:200018	nuclear	NP_001074	mmu:14886	GTF2I
K27754	FASTKD1		PF08368.17	GO:000989	mitochondr	NP_796218	mmu:320720	
K21921	KCTD18		PF02214.28	GO:005125	cytoplasmic	AAH75692.	mmu:51960	
K02183	CALM	mmu04750(	PF13499.12	GO:002260	cytoplasmic	XP_006511	mmu:75600	
K06636	SMC1	mmu04114(	PF02463.25	GO:000079	nuclear	NP_062684	mmu:24061	
K16507	DCHS1_2, I	mmu04392(	PF00028.23	GO:000318	extracellulo	XP_006507	mmu:233651	
K07881	RAB14	mmu04148(	PF00071.28	GO:000810	cytoplasmic	CAI92127.1	mmu:68365	
K13295	APLF		PF10283.14	GO:000810	nuclear	NP_001163	mmu:72103	
K20637	ARHGAP15		PF00620.32	GO:002260	cytoplasmic	SPT35687.1	mmu:76117	
K10582	UBE2Q	mmu04120(	PF00179.31	GO:006165	cytoplasmic	NP_850931	mmu:109161	
K27106	ZNF668		PF13465.12	GO:004423	cytoplasmic	BAE31234.1	mmu:24421	zf-C2H2
K12734	PPIL3		PF00160.26	GO:000645	cytoplasmic	NP_001272	mmu:70225	
K10285	FBXL19		PF16866.11	GO:000988	nuclear	NP_766336	mmu:233902	
K11978	UBR3		PF18995.5(I	GO:008009	cytoplasmic	NP_808451	mmu:68795	
K02940	RP-L9e, RP	mmu05171(	PF00347.28	GO:001593	cytoplasmic	NP_035422	mmu:20005	
K10442	KLHL1_4_5		PF01344.31	GO:004423	cytoplasmic	NP_001334	mmu:71778	ZBTB
K11854	USP35_38		PF21246.3(U	GO:000989	nuclear	BAC98274.1	mmu:74841	
K07748	NSDHL, EI	mmu00100(	PF01073.25	GO:001612	cytoplasmic	NP_035071	mmu:18194	
K19373	DNAJC28		PF09350.15(DJC28_CD		cytoplasmic	NP_001093	mmu:246738	
Testis expressed 10)			PF00096.32	GO:000097	cytoplasmic	NP_001347	mmu:23388	zf-C2H2
K08596	SENP7		PF02902.25	GO:007013	nuclear	XP_017172	mmu:66315	
K03909	TFPI	mmu04610(	PF00014.28	GO:009859	extracellulo	NP_001342	mmu:21788	
K23846	HPCA		PF13499.12	GO:007124	cytoplasmic	NP_001020	mmu:15444	
K08765	CPT1A	mmu04148(	PF00755.25	GO:009008	cytoplasmic	EDL32932.	mmu:12894	
K01641	HMGCS	mmu00650(	PF08540.16	GO:000663	cytoplasmic	BAE27103.1	mmu:208715	
K16468	CNTROB			GO:002260	cytoplasmic	NP_766148	mmu:216846	
K04373	RPS6KA	mmu04010(	PF00069.31	GO:004858	cytoplasmic	XP_006528	mmu:67071	
K07937	ARF1_2	mmu04144(	PF00025.27	GO:000810	cytoplasmic	NP_001006	mmu:11840	
K10510	ZBTB38		PF13465.12(zf-H2C2_2:		nuclear	NP_001346	mmu:24500	ZBTB
28HA(synovial sarcoma translocatio			PF05030.17(SSXT:SSXT		cytoplasmic	EDL09139.	mmu:26901	
K09228	KRAB		PF13465.12	GO:008009	cytoplasmic	AAC52301.	mmu:17076	zf-C2H2
K13963	SERPINB	mmu05146(	PF00079.25	GO:000989	cytoplasmic	BAE32772.1	mmu:20719	
K15260	PARP9		PF01661.27	GO:001922	cytoplasmic	NP_001392	mmu:80285	
K03134	TAF10	mmu03022(	PF03540.19	GO:008009	nuclear	NP_064408	mmu:24075	
K24628	ROGDI		PF10259.14	GO:004212	cytoplasmic	NP_573448	mmu:66049	
K23852	EFCAB7	mmu04340(	PF13499.12	GO:003315	cytoplasmic	XP_036019	mmu:230500	
K07556	ATPeAF2, ATPAF2, AT		PF07542.16	GO:003361	cytoplasmic	NP_663402	mmu:246782	
K07869	RHOBTB3		PF00651.37	GO:008009	cytoplasmic	BAC98044.1	mmu:73296	ZBTB
K26405	CCDC25		PF05670.18	GO:001602	nuclear	NP_666056	mmu:67179	
Lung seven transmembrane receptor			PF01202.28	GO:000573	mitochondr	EDL08131.	mmu:208967	

K05675	ABCD1, AI	mmu02010(	PF06472.20	GO:003444	mitochondr	NP_031461	mmu:11666	
K04438	CRK, CRKI	mmu04012(	PF07653.23	GO:004340	mitochondr	NP_031790	mmu:12929	
K24482	CRLF3			GO:000548	cytoplasmic	NP_061246	mmu:54394	
K07970	B3GNT3	mmu00512(	PF01762.27	GO:003030	Golgi appa	NP_082465	mmu:72297	
K16942	SEPT2	mmu05100(	PF00735.24	GO:000802	cytoplasmic	KAI252785	mmu:18000	
K20347	TMED2, EMP24		PF01105.29	GO:000858	ER	NP_062744	mmu:56334	
K23115	TTI2		PF10521.14	GO:003299	nuclear	NP_001186	mmu:234138	
K12737	SDCCAG10		PF00160.26	GO:000645	nuclear	NP_080348	mmu:67285	
K06583	ITGA7	mmu05165(	PF20806.3(I	GO:002260	ER	NP_001317	mmu:16404	
:ytochrome-b5 reductase activity, ac			PF00175.26	GO:004323	mitochondr	NP_663435	mmu:218885	
K14316	POM121, N	mmu03013(	PF15229.11	GO:003450	plasma mer	NP_683734	mmu:107939	
K26112	SIDT2		PF13965.12	GO:009008	lysosomal	NP_758461	mmu:214597	
K10244	ELOVL5	mmu01040(	PF01151.23	GO:000578	ER	XP_011241	mmu:68801	
K08441	GPR171		PF00001.26	GO:003802	plasma mer	NP_775574	mmu:229323	
K14005	SEC31	mmu04141(	PF12931.13(TPR_Sec16		cytoplasmic	NP_001346	mmu:69162	
Y9GN(Up-regulator of cell proliferation)				GO:003563	cytoplasmic	NP_848738	mmu:72046	
KSYS(phospholipid scramblase)			PF03803.21	GO:006102	cytoplasmic	NP_001161	mmu:70310	
K18270	RAB3GAP1		PF19533.4(I	GO:190306	cytoplasmic	BAE22204.	mmu:226407	
K16059	STIM1	mmu04611(	PF16533.10	GO:009008	ER	NP_033313	mmu:20866	
K01937	pyrG, CTPS	mmu01240(	PF06418.20	GO:009951	cytoplasmic	NP_001162	mmu:55936	
K06553	VPREB, CD179a		PF07686.23(V-set:Immu		plasma mer	DBA12411.	mmu:22362	
K01897	ACSL, fadD	mmu01100(	PF00501.34	GO:001564	cytoplasmic	NP_001028	mmu:74205	
K01481	E3.5.3.15		PF03068.20	GO:004322	mitochondr	NP_035189	mmu:18599	
K11506	CENPN		PF05238.18	GO:000557	nuclear	NP_082407	mmu:72155	
K27694	NDRG3		PF03096.20	GO:000573	cytoplasmic	NP_001342	mmu:29812	
K23800	XXYLT1	mmu00514(	PF01501.26	GO:001602	mitochondr	NP_941028	mmu:268880	
K27060	UTRN		PF00435.27	GO:005104	cytoplasmic	NP_035812	mmu:22288	
K11453	CBX6	mmu03083(	PF00354.24	GO:000582	cytoplasmic	AAQ95736.	mmu:494448	
K00476	ASPH	mmu04260(	PF05118.21	GO:000548	ER	NP_001171	mmu:65973	
K06171	NCSTN	mmu04330(	PF05450.20	GO:000578	ER	NP_067620	mmu:59287	
K02911	RP-L32, MI	mmu03010(	PF01783.29	GO:001593	mitochondr	NP_083547	mmu:75398	
K17809	POLDIP2		PF04379.20	GO:001922	mitochondr	NP_080665	mmu:67811	
K00030	IDH3	mmu01100(	PF00180.25	GO:001967	mitochondr	NP_570954	mmu:170718	
K16745	B9D2		PF07162.17	GO:002260	cytoplasmic	CAH73798	mmu:232987	
K24032	SH3PXD2		PF00018.34	GO:007027	nuclear	NP_032044	mmu:14218	
K23571	NOV, CCN3, IGFBP9		PF19035.6(	GO:009008	extracellulo	BAE33013.	mmu:18133	
K08958	CSNK1G	mmu04340(	PF00069.31	GO:000548	nuclear	XP_011241	mmu:214897	
			PF06431.17	GO:003563	cytoplasmic	CAA42510.1(large T antigen [Mus r		
K08525	RXRB, NR	mmu04659(	PF00104.35	GO:008009	nuclear	AIC84028.1	mmu:20182	
K10356	MYO1	mmu04814(	PF00063.27	GO:009951	cytoplasmic	AAH46502.	mmu:17916	
K15101	SLC25A2_15, ORNT		PF00153.32	GO:190335	cytoplasmic	NP_001345	mmu:18408	
K09059	NFIL3, E4I	mmu04710(	PF06529.16	GO:008009	nuclear	NP_059069	mmu:18030	TF_bZIP
K06784	ROBO4		PF13927.12	GO:000989	plasma mer	EDL25428.	mmu:74144	

K10587	UBE3A, E6	mmu05165(	PF00632.30	GO:005084	cytoplasmic	NP_001380	mmu:22215	
K17107	CSN2	mmu04917(	PF00363.23	GO:000557	extracellulo	NP_001272	mmu:12991	
K26536	RPS19BP1		PF15684.10	GO:003204	mitochondr	NP_780318	mmu:66538	
K08838	STK24_25_MST4		PF00069.31	GO:000716	cytoplasmic	XP_008065	mmu:70415	
K00967	PCYT2	mmu00440(	PF01467.32	GO:000665	cytoplasmic	NP_001334	mmu:68671	
K17409	MRPS30		PF07147.18	GO:001593	mitochondr	EDL18359.	mmu:59054	
K23677	SPNS		PF07690.21	GO:003575	plasma mer	PNJ33228.1	mmu:73658	
K08732	RALGDS	mmu05231(	PF00617.24	GO:000508	cytoplasmic	XP_031225	mmu:19730	
K02880	RP-L17e, R	mmu05171(	PF00237.24	GO:190280	cytoplasmic	AAH94640.	mmu:319195	
K24864	ARL6IP1				cytoplasmic	XP_006047	mmu:54208	
K02963	RP-S18, MR	mmu03010(	PF01084.26	GO:001593	mitochondr	NP_081044	mmu:68565	
K11275	H1_5		PF00538.25	GO:003472	nuclear	NP_941024	mmu:243529	
K16751	C2CD3		PF00168.36	GO:000858	cytoplasmic	NP_001017	mmu:277939	
K00161	PDHA, pdh	mmu05230(	PF00676.26	GO:004423	mitochondr	NP_032836	mmu:18597	
K07232	CHAC, cha	mmu00480(	PF04752.17	GO:004424	cytoplasmic	NP_080803	mmu:68044	
K24749	WDR54		PF21031.3(	GO:002260	peroxisoma	NP_001349	mmu:75659	
K21198	NAPG, SNAPG		PF14938.12	GO:000810	cytoplasmic	NP_001406	mmu:108123	
K14216	OAS	mmu05164(	PF10421.14	GO:001922	cytoplasmic	NP_660261	mmu:246727	
K01537	ATP2C		PF00122.25	GO:000578	cytoplasmic	BAE35481.	mmu:235574	
K14608	OASL	mmu05165(	PF10421.14	GO:000548	cytoplasmic	NP_001346	mmu:231655	
K20284	RABGAP1, GAPCENA		PF00566.25	GO:000704	cytoplasmic	NP_001349	mmu:227800	
K12470	SH3KBP1, (	mmu04144(	PF14604.12	GO:000810	cytoplasmic	XP_030107	mmu:58194	
K10477	BTBD1_2		PF08005.17(PHR:PHR (		cytoplasmic	NP_663336	mmu:20819	ZBTB
K10693	MYCBP2, PAM		PF08005.17	GO:003292	nuclear	XP_011243	mmu:105689	
K10360	MYO9	mmu04814(	PF00063.27	GO:003243	cytoplasmic	NP_001135	mmu:17925	
K00875	rbtK, FGG	mmu00040(	PF02782.22	GO:001932	cytoplasmic	URX64926.	mmu:75578	
K18265	ITM2C, BRI3		PF04089.19	GO:200123	cytoplasmic	BAE33901.	mmu:64294	
K22756	NSMCE2, NSE2		PF11789.14	GO:004316	nuclear	NP_001361	mmu:68501	
K13173	ARGLU1		PF15346.11	GO:000557	cytoplasmic	XP_045139	mmu:234023	
K04840	SCN8A, NAV1.6		PF00520.37	GO:000526	cytoplasmic	XP_006520	mmu:20273	
K00008	SORD, gutl	mmu00051(	PF00107.32	GO:005128	cytoplasmic	NP_666238	mmu:20322	
K10593	EDD1, UBI	mmu04120(	PF00632.30	GO:005084	cytoplasmic	NP_001074	mmu:70790	
K15523	FN3KRP		PF03881.20	GO:003563	cytoplasmic	NP_852085	mmu:238024	
K23566	MMGT1, EMG5		PF10270.14	GO:000810	Golgi appa	NP_001100	mmu:236792	
K07514	EHHADH	mmu00650(	PF02737.24	GO:001649	peroxisoma	NP_076226	mmu:74147	
K02332	POLG	mmu03410(	PF18136.6(I	GO:003197	mitochondr	P54099.3(R	mmu:18975	
K05111	EPHB2, ER	mmu04360(	PF07714.23	GO:000803	plasma mer	NP_034272	mmu:13844	
K10356	MYO1	mmu04814(	PF00063.27(Myosin_he		cytoplasmic	NP_001357	mmu:17913	
K07607	PRPH	mmu05014(	PF00038.26	GO:009859	cytoplasmic	NP_001157	mmu:19132	
K23609	C9ORF72	mmu04140(	PF15019.11	GO:004867	cytoplasmic	NP_082742	mmu:73205	
K13199	SERBP1		PF16174.10	GO:000989	cytoplasmic	NP_080090	mmu:66870	
K14791	PWP1		PF24814.1(	GO:001922	nuclear	BAB26622.1	mmu:103136	
XQP1(bromo adjacent homology do			PF24912.1(S	GO:000368	cytoplasmic	XP_006504	mmu:231861	

K01988	A4GALT	mmu00603(	PF04572.17	GO:001564	Golgi appa	NP_001004	mmu:239559	
K02516	PRMT5, HSL7		PF17285.8(I	GO:000647	cytoplasmic	NP_038796	mmu:27374	
K09568	FKBP1		PF00254.34	GO:001701	cytoplasmic	NP_001289	mmu:14225	
K15684	BFAR		PF00536.37	GO:000578	cytoplasmic	NP_080252	mmu:67118	
K05925	METTL3		PF05063.20	GO:200073	nuclear	NP_062695	mmu:56335	
K14832	MAK21, NOC1, CEBPZ		PF03914.23	GO:008009	nuclear	NP_001019	mmu:12607	TF_others
CCAAT enhancer binding protein (C			PF03194.20	GO:004584	nuclear	NP_080157	mmu:66978	
K14795	RRP36		PF06102.17	GO:003068	nuclear	AAH11248.	mmu:224823	
K26240	C2CD2L, C2CD2, TME		PF18696.7(S	GO:000557	Golgi appa	NP_777272	mmu:207781	
XPIU(INO80 complex subunit B)			PF13679.12	GO:000998	cytoplasmic	NP_997405	mmu:216292	
K05767	IQGAP2_3	mmu04810(	PF00616.24	GO:003304	cytoplasmic	NP_081987	mmu:544963	
K04678	SMURF	mmu04120(	PF00632.30	GO:200024	cytoplasmic	NP_079757	mmu:66313	
XNS1(MOB family member 4, phoc			PF03637.22	GO:001989	cytoplasmic	NP_001094	mmu:19070	
K03992	MASP1	mmu04610(	PF00089.32	GO:000425	cytoplasmic	AAN39849.	mmu:17174	
Required for the post-translational c			PF00012.26	GO:003563	cytoplasmic	NP_780408	mmu:73442	
K11140	ANPEP, CI	mmu01100(	PF11838.14	GO:000417	extracellulo	NP_032512	mmu:16790	
K18038	PTPN9, MEG2		PF00102.32	GO:001679	cytoplasmic	NP_062625	mmu:56294	
K09208	KLF9S, BTEB		PF00096.32	GO:008009	nuclear	NP_067341	mmu:50794	zf-C2H2
K11426	SMYD	mmu00310(	PF01753.24	GO:008009	cytoplasmic	NP_001153	mmu:12180	
K22858	JBTS26		PF14652.11	GO:004322	cytoplasmic	AAI58020.1	mmu:233865	
K22182	TXNRD	mmu05200(	PF07992.20	GO:003459	mitochondr	NP_038739	mmu:26462	
K14365	FHL1, SLIM	mmu04630(	PF00412.28	GO:004239	cytoplasmic	NP_001274	mmu:14199	
K06794	NCAN, CSPG3		PF00193.23	GO:000548	extracellulo	NP_031815	mmu:13004	
XR0A(Kelch repeat and BTB (POZ)			PF00626.27	GO:000554	cytoplasmic	NP_001158	mmu:22351	
K20072	SWAP70		PF00169.35	GO:003017	cytoplasmic	NP_081461	mmu:23853	
K15128	MED6		PF04934.19	GO:008009	nuclear	NP_001334	mmu:69792	
K13358	SLC9A3R2,	mmu04960(	PF00595.29	GO:000810	cytoplasmic	NP_075542	mmu:65962	
K09631	HGFAC		PF00089.32	GO:000425	plasma mer	NP_062320	mmu:54426	
K23224	SPATA6		PF14909.12	GO:003202	extracellulo	XP_021015	mmu:67946	
Z1VS(secreted phosphoprotein 2, 24l			PF07448.17	GO:000557	extracellulo	CAC83812.	mmu:75396	
K16589	HAUS6, AUG6		PF14661.11	GO:002260	cytoplasmic	NP_775576	mmu:230376	
K14403	CPSF3, YSH	mmu03015(	PF11718.13	GO:001679	cytoplasmic	BAE33998.	mmu:54451	
Y9KV(transmembrane and coiled-cc			PF10267.14	GO:000548	cytoplasmic	BAC38774.	mmu:319880	
K00474	TMLHE	mmu00310(	PF02668.22	GO:004239	mitochondr	NP_620097	mmu:192289	
K17586	PHRF1		PF00628.35	GO:003277	cytoplasmic	NP_001074	mmu:101471	
K04525	SERPINA		PF00079.25	GO:000486	extracellulo	NP_031644	mmu:12401	
K01410	MIPEP		PF01432.26	GO:000417	mitochondr	NP_081712	mmu:70478	
K01847	MUT	mmu00280(	PF01642.28	GO:000449	mitochondr	NP_032676	mmu:17850	
K01121	CNP		PF05881.18	GO:004423	mitochondr	NP_034053	mmu:12799	
K08784	HTRA1, PRSS11		PF13365.12	GO:006071	extracellulo	NP_062510	mmu:56213	
K02369	EXTL2	mmu00534(	PF09258.16	GO:000188	Golgi appa	BAM48568.	mmu:58193	
ZS5N(zinc finger, CCHC domain co			PF17180.10	GO:004316	nuclear	NP_001094	mmu:71918	
K19827	MACF1		PF00435.27	GO:009951	cytoplasmic	XP_026955	mmu:11426	

K09228	KRAB		PF13465.12	GO:000097	cytoplasmic	NP_001002	mmu:38106	zf-C2H2
K05857	PLCD	mmu01100(	PF00388.25	GO:001922	cytoplasmic	NP_001280	mmu:18799	
K06269	PPP1C	mmu04750(	PF00149.33	GO:003292	cytoplasmic	NP_001029	mmu:19046	
K00731	C1GALT1	mmu00514(	PF02434.22	GO:006057	Golgi appa	NP_443719	mmu:94192	
K02871	RP-L13, MI	mmu03010(	PF00572.23	GO:001593	mitochondr	AAH55470.	mmu:68537	
K5H(mitochondrial fission regulat			PF05308.16	GO:000026	mitochondr	XP_036019	mmu:67472	
K20178	VPS8	mmu04148(	PF23410.2(I	GO:000810	cytoplasmic	NP_001272	mmu:209018	
K26746	TMX1_4		PF00085.26	GO:003196	ER	NP_083424	mmu:52837	
K15186	EAF		PF09816.14	GO:000802	nuclear	NP_083208	mmu:74427	
K08061	RFX5	mmu04612(	PF14621.11	GO:000097	nuclear	NP_001342	mmu:53970	RFX
K04513	RHOA	mmu04530(	PF00071.28	GO:000821	cytoplasmic	BAE38228.	mmu:11848	
K05284	PIGM	mmu00563(	PF05007.19	GO:000815	ER	NP_080510	mmu:67556	
NE1(Surfeit 2)			PF05477.16	GO:001660	nuclear	NP_038706	mmu:20931	
K00544	BHMT	mmu00270(	PF02574.22	GO:000548	cytoplasmic	NP_057877	mmu:12116	
K01895	ACSS1_2, a	mmu00640(	PF00501.34	GO:001564	cytoplasmic	XP_021036	mmu:60525	
K13823	HBB	mmu05144(	PF00042.28	GO:000534	cytoplasmic	ABU63195.	mmu:101488143	
K14782	AATF, BFR2		PF13339.11	GO:200123	cytoplasmic	NP_062790	mmu:56321	
K13987	NUDT5	mmu00230(	PF00293.34	GO:001932	cytoplasmic	NP_058614	mmu:53893	
K19054	FXN	mmu00860(	PF01491.21	GO:200123	mitochondr	NP_032070	mmu:14297	
K00894	ETNK, EK	mmu00564(	PF01633.27	GO:000665	cytoplasmic	XP_021045	mmu:75320	
K17675	SUPV3L1, SUV3		PF22527.2(I	GO:008009	mitochondr	NP_852088	mmu:338359	
V8N(-)			PF14802.11	GO:000577	peroxisoma	NP_082703	mmu:73067	
K11865	MYSM1		PF04433.23	GO:008009	cytoplasmic	NP_796213	mmu:32071	MYB
K01958	PC, pyc	mmu01100(	PF02786.22	GO:001922	mitochondr	NP_001156	mmu:18563	
K26100	PWWP2		PF00855.23	GO:006026	nuclear	NP_081833	mmu:70802	
K06491	NCAM, CD	mmu05020(	PF07679.22	GO:004858	ER	P13595.3(R	mmu:17967	
K04444	MAPKAPK	mmu04010(	PF00069.31	GO:000690	cytoplasmic	NP_001408	mmu:102626	
K06553	VPREB, CD179a		PF07686.23	GO:000237	extracellulo	CAB46325.	mmu:22364	
K00020	HIBADH, r	mmu00280(	PF03446.21	GO:005128	mitochondr	NP_663542	mmu:58875	
K10079	SIGLEC12, SIGLECL1		PF07686.23	GO:000989	plasma mer	NP_112458	mmu:83382	
K11469	RYBP	mmu03083(	PF17219.8(	GO:008009	nuclear	NP_001386	mmu:56353	
K01803	TPI, tpiA	mmu00051(	PF00121.24	GO:004643	cytoplasmic	NP_033441	mmu:21991	
K24069	PITPNM		PF02121.25(IP_trans:Pl		cytoplasmic	NP_001411	mmu:19679	
K23768	S100A16, S100F		PF01023.25	GO:004830	cytoplasmic	XP_021014	mmu:67860	
K13237	DECR2, SP	mmu04146(	PF13561.12	GO:000663	peroxisoma	BAE37137.	mmu:26378	
K19867	PDLIM5_6	mmu04820(	PF00412.28	GO:003243	cytoplasmic	AAO26189.	mmu:24131	
Y4WQ(WD repeat domain 67)			PF24814.1(	GO:002260	cytoplasmic	NP_001074	mmu:210544	
K00789	metK, MAT	mmu01240(	PF02773.21	GO:000548	cytoplasmic	NP_598414	mmu:11720	
K16449	RGS		PF00615.24	GO:000548	cytoplasmic	NP_001278	mmu:56470	
K23539	UNC119		PF05351.17	GO:200036	cytoplasmic	NP_001300	mmu:22248	
K16334	CRTC3, TC	mmu05166(	PF12885.12	GO:008009	cytoplasmic	NP_776288	mmu:70461	
K15544	SSU72	mmu03015(	PF04722.18	GO:004423	cytoplasmic	NP_081175	mmu:68991	
K13178	DDX17		PF00270.35	GO:008009	nuclear	NP_951062	mmu:67040	

K09666	POMGNT1	mmu00515(	PF03071.21	GO:00352€	Golgi appa	EDL30616.	mmu:68273	
K00088	IMPDH, gu	mmu01100(	PF00478.31	GO:000916	nuclear	XP_038963	mmu:23917	
K17429	MRPL48		PF00338.27	GO:001593	cytoplasmic	XP_006508	mmu:52443	
Dehydrogenase)			PF00106.31	GO:000585	cytoplasmic	NP_001365	mmu:72552	
K01482	DDAH, ddaH			GO:000548	cytoplasmic	NP_001177	mmu:51793	
K19474	SIX5		PF16878.10	GO:008009	cytoplasmic	NP_035513	mmu:20475	Homeobox
K12598	MTR4, SKI	mmu03018(	PF13234.12	GO:001688	nuclear	BAC40348.	mmu:72198	
K03065	PSMC3, RP	mmu05020(	PF00004.35	GO:003577	cytoplasmic	NP_032974	mmu:19182	
K20472	COPZ, RET3		PF01217.26	GO:004323	cytoplasmic	NP_063930	mmu:56358	
K00036	G6PD, zwf	mmu05230(	PF02781.21	GO:008009	cytoplasmic	NP_032088	mmu:14381	
K01300	CPB2	mmu04610(	PF00246.29	GO:000759	extracellulo	NP_062749	mmu:56373	
K10135	SCOTIN	mmu04115(p53 signalir		GO:001602	cytoplasmic	NP_001344	mmu:66940	
K12898	HNRNPF_H		PF00076.28	GO:008009	cytoplasmic	NP_001032	mmu:98758	
K04979	TRPM4	mmu04911(	PF18139.7(I	GO:003577	cytoplasmic	EDL22837.	mmu:68667	
K05078	TSLPR	mmu04060(	PF21605.3((	GO:000989	cytoplasmic	NP_001297	mmu:57914	
K00510	HMOX1	mmu05208(	PF01126.25	GO:200123	cytoplasmic	NP_034572	mmu:15368	
K07828	NRAS	mmu05208(	PF00071.28	GO:000828	cytoplasmic	XP_005887	mmu:18176	
K24675	ZMYM2_3_4		PF06467.20	GO:002260	nuclear	NP_062805	mmu:56364	
260S(Chromosome 4 open reading fr			PF15378.11	GO:000579	cytoplasmic	AFE61898.1	mmu:70617	
K25789	JAGN		PF07086.17	GO:009008	ER	NP_001037	mmu:67767	
K23112	BRAT1		PF02985.28	GO:004423	cytoplasmic	NP_001263	mmu:231841	
K14025	SELS, VIMI	mmu04141(	PF06936.16	GO:200123	ER	EDL07227.	mmu:109815	
K00750	GYG1, GY	mmu00500(	PF01501.26	GO:003024	cytoplasmic	XP_003508	mmu:27357	
K10753	ASF1		PF04729.19	GO:003472	nuclear	EDL92926.	mmu:66403	
K11353	NDUFA13	mmu05208(	PF06212.17	GO:200123	cytoplasmic	NP_001369	mmu:67184	
K23915	FSTL5		PF13927.12	GO:003015	extracellulo	BAE24428.	mmu:213262	
K23796	PLEKHA1_2		PF00169.35	GO:005084	cytoplasmic	EDL32841.	mmu:83436	
K20217	UBE2E	mmu04120(	PF00179.31	GO:003202	cytoplasmic	NP_001347	mmu:218793	
K24083	ABHD13		PF00561.26	GO:001679	Golgi appa	NP_001074	mmu:68904	
K11863	ATXN3, M	mmu04141(	PF02099.23	GO:001989	cytoplasmic	NP_083981	mmu:110616	
K16540	AZI1, CEP131			GO:003315	nuclear	NP_001390	mmu:12009	
K13203	ZFR		PF07528.20	GO:004322	nuclear	NP_035897	mmu:22763	
K01074	PPT	mmu04142(	PF02089.21	GO:000810	lysosomal	NP_032943	mmu:19063	
K03868	RBX1, ROC	mmu04120(	PF12678.13	GO:003243	cytoplasmic	NP_001029	mmu:56438	
K05453	CSF1, MCS	mmu04151(	PF05337.16	GO:001922	cytoplasmic	XP_036018	mmu:12977	
NGS(Mid1 interacting protein 1)			PF07084.17	GO:003111	cytoplasmic	NP_001160	mmu:68041	
K06471	PECAM1, C	mmu04148(	PF13895.12	GO:009951	plasma mer	EDL34306.	mmu:18613	
K10363	DSTN		PF00241.25	GO:001562	cytoplasmic	NP_062745	mmu:56431	
K12648	MAVS, IPS	mmu05164(	PF16739.11	GO:000574	cytoplasmic	NP_001193	mmu:228607	
K12304	CANT1	mmu01100(	PF06079.17	GO:000918	ER	NP_001020	mmu:76025	
K08266	MLST8, GB	mmu04140(	PF00400.38	GO:001922	cytoplasmic	NP_001239	mmu:56716	
K10344	SPSB2, SSB2		PF00622.36	GO:199023	cytoplasmic	NP_001292	mmu:14794	
K03952	NDUFA8	mmu05208(	PF06747.18	GO:000367	cytoplasmic	NP_080979	mmu:68375	

ZKBA(-)			PF00665.32	GO:004671	cytoplasmic	CAA34659.	mmu:118567641
K24513	ITIH3, SHAP		PF06668.17	GO:003020	extracellula	NP_032433	mmu:16426
K01639	E4.1.3.3, na	mmu00520(	PF00701.28	GO:000874	cytoplasmic	NP_083025	mmu:74091
K12464	MAGED1,	mmu04722(	PF01454.25	GO:004340	nuclear	BAE27491.	mmu:94275
K07414	CYP2D	mmu04726(	PF00067.27	GO:000449	ER	NP_663449	mmu:223706
K01934	MTHFS	mmu00670(	PF01812.26	GO:000911	cytoplasmic	NP_001122	mmu:100039707
XX1I(mitochondrial ribosomal prot			PF14978.11	GO:001593	mitochondr	NP_080677	mmu:67840
K16776	NAV1		PF23092.1(	GO:000367	nuclear	NP_775613	mmu:215690
K20093	ERCC6L, PICH		PF00176.29	GO:000367	nuclear	NP_666347	mmu:236930
K14397	NUDT21, C	mmu03015(	PF13869.12	GO:190545	cytoplasmic	NP_001034	mmu:68219
K02867	RP-L11, MF	mmu03010(	PF03946.19	GO:001593	mitochondr	NP_079829	mmu:66419
K10074	CLEC7A, D	mmu04613(	PF00059.27	GO:000959	plasma mer	Q6QLQ4.3	mmu:56644
K06482	ITGA3, CD	mmu05165(	PF20806.3(	GO:001922	plasma mer	NP_038593	mmu:16400
K14209	SLC36A, PA	mmu04974(	PF01490.24	GO:005104	plasma mer	NP_694810	mmu:246049
K26186	MTRES1			GO:190310	mitochondr	NP_080687	mmu:67851
K20803	UBE3D		PF09814.14	GO:199023	nuclear	NP_081670	mmu:70348
K12350	SMPD1, AS	mmu04071(	PF00149.33	GO:004643	lysosomal	NP_035551	mmu:20597
K15617	LDB1	mmu05202(	PF01803.21	GO:005125	nuclear	NP_001397	mmu:16825
K05167	IL17RD, SEF		PF16742.10	GO:005124	Golgi appa	NP_602319	mmu:171463
K16940	SEPT10		PF00735.24	GO:003563	cytoplasmic	NP_001020	mmu:103080
K20465	OSBPL9_10_11, ORP9_		PF01237.23	GO:000554	cytoplasmic	XP_011241	mmu:74486
K03345	SENP2, AX	mmu03013(	PF02902.25	GO:007013	mitochondr	NP_083733	mmu:75826
K23339	YPEL5		PF03226.21	GO:003299	cytoplasmic	NP_001007	mmu:383295
K08855	GAK		PF00069.31	GO:000810	cytoplasmic	NP_705797	mmu:231580
K02987	RP-S4e, RP	mmu05171(	PF00900.26	GO:000548	cytoplasmic	NP_000998	mmu:20102
GQ8(Protein of unknown function			PF00880.23	GO:000701	nuclear	BAC35194.2(unnamed protein prod	
K02966	RP-S19e, RI	mmu05171(	PF01090.25	GO:002262	cytoplasmic	NP_001347	mmu:20085
HFX(Transmembrane protein 205)			PF13664.11	GO:000557	plasma mer	NP_001240	mmu:235043
K01332	C2	mmu05171(	PF00089.32	GO:000425	plasma mer	NP_038512	mmu:12263
K24804	ZBTB14		PF00651.37	GO:000582	nuclear	AAN75191.	mmu:22666 ZBTB
K01335	CFB	mmu04610(	PF00089.32	GO:000425	extracellula	ERE88812.	mmu:14962
K26242	RCOR2_3		PF20878.2(	GO:000989	cytoplasmic	NP_001277	mmu:21474 MYB
K11426	SMYD	mmu00310(	PF01753.24	GO:000647	cytoplasmic	NP_081072	mmu:226830
RPK(ninjurin 1)			PF04923.17	GO:003411	cytoplasmic	EDL41061.	mmu:18081
K06539	PVR, NECI	mmu04514(	PF07686.23	GO:000285	plasma mer	BAC37229.	mmu:52118
K09575	FKBP9_10		PF00254.34	GO:002260	ER	NP_001156	mmu:14230
K16462	CEP164		PF00397.32	GO:002260	cytoplasmic	XP_006510	mmu:214552
K15033	ICT1, MRPL58		PF00472.26	GO:001615	mitochondr	NP_001334	mmu:68572
K10756	RFC3_5	mmu03030(	PF22534.1(	GO:008009	nuclear	NP_081285	mmu:69263
K25591	LSM10		PF01423.28	GO:004423	cytoplasmic	NP_001156	mmu:116748
K21776	LIN54	mmu04218(	PF03638.21	GO:003472	nuclear	BAD90150.	mmu:231506
coiled-coil domain containing 86)			PF03006.26	GO:004316	plasma mer	NP_076313	mmu:76498
K27393	FLNC	mmu04820(	PF00630.25	GO:001562	cytoplasmic	NP_001074	mmu:68794

XQP1(AFG1 family ATPase)			PF01426.24	GO:000989	cytoplasmic	BAC98053.1	mmu:228536	
K23600	TARDBP, T	mmu05014(	PF20910.2(	GO:009008	nuclear	NP_663531	mmu:230908	
K24416	FBLIM1, FBLP1		PF00412.28	GO:003243	cytoplasmic	AAQ08090.	mmu:74202	
22F0(family with sequence similarity			PF05308.16	GO:000699	cytoplasmic	BAB27601.1	mmu:71804	
XTBM(transforming growth factor beta regulato				GO:003450	nuclear	EDL25439.	mmu:21376	
K15432	ASW		PF15323.11	GO:004859	nuclear	NP_598579	mmu:98404	
K20303	TRAPPC4, TRS23		PF04099.18	GO:004820	cytoplasmic	NP_068561	mmu:60409	
K21073	ACTN2_3	mmu05412(	PF00435.27	GO:001922	cytoplasmic	NP_038484	mmu:11474	
K06770	MPZ	mmu04514(	PF10570.14	GO:009859	ER	NP_001302	mmu:17528	
K15075	MET18, MMS19		PF12460.14	GO:004323	extracellulo	NP_082428	mmu:72199	
K26244	PHF12, RCO1		PF16737.10	GO:000554	cytoplasmic	NP_777277	mmu:268448	
K10604	MGRN1	mmu04120(	PF13920.12	GO:000548	cytoplasmic	NP_001365	mmu:17237	
K26197	GPRASP2		PF04826.19	GO:004259	nuclear	NP_001156	mmu:245607	
K04688	RPS6KB	mmu05200(	PF00069.31	GO:000988	cytoplasmic	NP_067460	mmu:58988	
K15704	RNF149		PF13639.12	GO:001602	extracellulo	NP_001028	mmu:67702	
K02891	RP-L22e, R	mmu05171(	PF01776.22	GO:004322	cytoplasmic	NP_001102	mmu:68028	
K12741	HNRNPA1	mmu05014(	PF00076.28	GO:005116	nuclear	XP_026311	mmu:15382	
K11405	HDAC8	mmu05203(	PF00850.24	GO:008009	cytoplasmic	NP_081658	mmu:70315	
K17265	G3BP1	mmu04623(	PF02136.26	GO:003577	nuclear	NP_038744	mmu:27041	
K05628	RERE		PF03154.20	GO:002192	nuclear	NP_001078	mmu:68703	zf-GATA
K10402	KIF20	mmu04814(	PF00225.29	GO:000810	cytoplasmic	EDK97102.	mmu:19348	
K02732	PSMB1	mmu05020(	PF00227.31	GO:001977	cytoplasmic	NP_035315	mmu:19170	
K15275	SLC35B1		PF08449.17	GO:000534	ER	NP_058032	mmu:110172	
K14767	UTP3, SAS10		PF09368.15	GO:003204	nuclear	NP_075541	mmu:65961	
K26610	VEZF1		PF13894.12	GO:000097	nuclear	NP_057895	mmu:22344	zf-C2H2
K09228	KRAB		PF13465.12	GO:008009	cytoplasmic	BAC33769.1	mmu:23016	zf-C2H2
K08339	ATG5	mmu04136(	PF20638.3(	GO:001065	cytoplasmic	AXY94487.	mmu:11793	
K04412	STK3, MST	mmu04390(	PF00069.31	GO:200123	cytoplasmic	NP_062609	mmu:56274	
K19947	MICAL		PF12130.13	GO:000449	cytoplasmic	NP_612188	mmu:171580	
K04839	SCN7A, SC	mmu04261(	PF00520.37	GO:000526	cytoplasmic	NP_033161	mmu:20272	
K20316	SCOC		PF10224.14	GO:000989	cytoplasmic	NP_001034	mmu:56367	
K09114	MXD, MAD		PF00010.31	GO:000367	mitochondr	XP_030106	mmu:17859	bHLH
K02978	RP-S27e, R	mmu05171(	PF01667.23	GO:008009	cytoplasmic	NP_001180	mmu:67941	
K14137	PTAR1		PF01239.28	GO:000573	cytoplasmic	NP_082484	mmu:72351	
K23310	PHF6	mmu03082(	PF13771.12	GO:004239	nuclear	EDL29118.	mmu:70998	
K11421	SETDB1	mmu01100(	PF18300.7(I	GO:008009	nuclear	EDL38808.	mmu:84505	MBD
K13915	LYZ	mmu04970(	PF00062.25	GO:004299	extracellulo	NP_059068	mmu:17105	
K00699	UGT	mmu00980(	PF00201.24	GO:003250	ER	NP_964004	mmu:394432	
K13196	DPF2, REQ	mmu03082(	PF14051.11	GO:190545	nuclear	BAE26697.1	mmu:19708	
K03849	ALG8	mmu00510(	PF03155.21	GO:000578	extracellulo	BAE24656.1	mmu:381903	
K14943	MBNL		PF22628.2(	GO:008009	cytoplasmic	NP_598924	mmu:171170	
K06503	TFRC, CD7	mmu04350(	PF04389.22	GO:000810	cytoplasmic	NP_001344	mmu:22042	
20A3(embigin-like)			PF07679.22	GO:004299	extracellulo	NP_034460	mmu:13723	

K22538	RPUSD4		PF00849.27	GO:007013	mitochondr	NP_082316	mmu:71989	
K12869	CRN, CRN	mmu03040(	PF23241.1(I	GO:002260	nuclear	NP_080096	mmu:66877	
Family with sequence similarity 105,			PF13855.12	GO:004308	cytoplasmic	NP_001239	mmu:380916	
K13207	CUGBP, BRUNOL, CE		PF00076.28	GO:002260	cytoplasmic	NP_001393	mmu:14007	
K23900	RCN3		PF13499.12	GO:000810	ER	NP_001341	mmu:52377	
XSUJ(MOB kinase activator)			PF03637.22	GO:001920	cytoplasmic	NP_001361	mmu:101513	
K23480	ZNF362_384, LIN-29		PF00096.32	GO:008009	nuclear	XP_055467	mmu:23076	zf-C2H2
K00616	TALDO1, t	mmu00030(	PF00923.25	GO:000673	cytoplasmic	NP_035658	mmu:21351	
K22069	LYRM4		PF05347.21	GO:000548	mitochondr	NP_958746	mmu:380840	
K11590	MBD2	mmu03082(	PF14048.11	GO:008009	nuclear	NP_034903	mmu:17191	MBD
K15116	SLC25A33_36, RIM2		PF00153.32	GO:001922	cytoplasmic	NP_081736	mmu:70556	
K09209	KLF10_11, TIEG		PF00096.32	GO:008009	nuclear	AAC77798.	mmu:21847	zf-C2H2
K24889	IRX		PF05920.17	GO:003250	cytoplasmic	NP_061296	mmu:54352	Homeobox
K11424	WHSC1, M	mmu05202(	PF00855.23	GO:008009	nuclear	NP_780440	mmu:10782	HMG
K12656	SIKE	mmu04622(	PF05769.16	GO:000573	cytoplasmic	NP_079955	mmu:66641	
ZIM9(Coiled-coil domain-containin			PF06244.18	GO:003277	nuclear	NP_081240	mmu:234388	
K23570	EMC10			GO:000828	plasma mer	EDL22723.	mmu:69683	
K22566	COMMD10		PF07258.19	GO:000573	cytoplasmic	NP_001405	mmu:69456	
K03872	ELOC, TCI	mmu05200(	PF03931.21	GO:004518	cytoplasmic	NP_001297	mmu:67923	
Z7NH(DiGeorge syndrome critical r			PF24495.1(I	GO:000557	plasma mer	NP_061360	mmu:56030	
K00012	UGDH, ugd	mmu01240(	PF03721.20	GO:003020	nuclear	NP_033492	mmu:22235	
K15074	BACURD		PF02214.28	GO:008009	cytoplasmic	NP_001152	mmu:21927	
K12236	NFX1	mmu05165(	PF01422.22	GO:004323	nuclear	NP_076228	mmu:74164	zf-NF-X1
K17492	UBN		PF14075.11	GO:000557	nuclear	EDL13634.	mmu:320538	
K01327	ELANE	mmu05322(	PF00089.32	GO:000425	ER	NP_056594	mmu:50701	
K10773	NTHL1, ntl	mmu03410(	PF00730.31	GO:000390	nuclear	AAI38853.1	mmu:18207	
K12077	PDK1	mmu05230(	PF10436.14	GO:001707	mitochondr	NP_766253	mmu:228026	
K18025	PTPN14_21		PF00102.32	GO:003250	mitochondr	NP_033002	mmu:19250	
K01510	ENTPD1_3	mmu01100(	PF01150.23	GO:001922	cytoplasmic	NP_033978	mmu:12495	
K06086	SORBS1, SI	mmu03320(	PF00018.34	GO:003243	cytoplasmic	XP_030106	mmu:20411	
YXX7(axin interactor, dorsalization			PF14186.12	GO:000554	cytoplasmic	NP_859421	mmu:108909	
K16344	LAMTOR5	mmu04150(	PF16672.11	GO:000810	cytoplasmic	Q9D1L9.1(	mmu:68576	
K11367	CHD1		PF00176.29	GO:003472	nuclear	NP_031716	mmu:12648	
K10712	ADO	mmu00430(	PF07847.18	GO:004322	cytoplasmic	NP_001005	mmu:211488	
)CGV(chromosome 10 open reading frame 118)				GO:000810	cytoplasmic	NP_739563	mmu:213993	
K14971	NCOA6, ASC2			GO:003577	nuclear	BAE25107.	mmu:56406	
K13095	SF1		PF16275.10	GO:008009	cytoplasmic	NP_001390	mmu:22668	
K12480	RABEP1	mmu04144(	PF03528.20	GO:000810	cytoplasmic	NP_062273	mmu:54189	
K05850	ATP2B	mmu04970(	PF00689.26	GO:000962	cytoplasmic	ACC91879.	mmu:381290	
K11390	CLYBL		PF03328.21	GO:002260	mitochondr	NP_083832	mmu:69634	
K20163	DENND4		PF02141.26	GO:000508	nuclear	NP_001156	mmu:102442	
K09093	AHR	mmu05208(	PF08447.18	GO:190461	cytoplasmic	P30561.3(R	mmu:11622	bHLH
K14778	DDX49, DBP8		PF00270.35	GO:000989	nuclear	NP_001020	mmu:234374	

K20526	TAGLN		PF00307.36	GO:000367	cytoplasmic	NP_062728	mmu:56370	
15FT(family with sequence similarit			PF14798.11	GO:001507	plasma mer	NP_780658	mmu:215900	
K10704	UBE2V		PF00179.31	GO:003243	nuclear	NP_076074	mmu:70620	
K16060	BIRC2_3	mmu04120(	PF00653.27	GO:001922	cytoplasmic	NP_001278	mmu:11797	
K15040	VDAC2	mmu05208(	PF01459.28	GO:200123	cytoplasmic	NP_035825	mmu:22334	
K08869	ADCK, ABC1		PF03109.22	GO:000815	cytoplasmic	NP_001156	mmu:67426	
K11792	DDA1		PF10172.14	GO:000989	cytoplasmic	NP_001128	mmu:66498	
K03267	ERF3, GSP	mmu03015(	PF00009.33	GO:001707	cytoplasmic	NP_032205	mmu:14853	
K12797	CARD6	mmu04621(	PF00619.27	GO:000573	cytoplasmic	EDL03385.	mmu:239319	
pseudouridine synthase activity)			PF00849.27	GO:003111	cytoplasmic	NP_775626	mmu:271842	
K04525	SERPINA		PF00079.25	GO:000486	extracellulo	NP_033279	mmu:20717	
K24273	ZRSR		PF00642.30	GO:002260	nuclear	NP_033479	mmu:22184	
K20826	RPAP1		PF08620.15	GO:003088	nuclear	NP_001157	mmu:68925	
K11672	ACTR5, AR	mmu03082(	PF00022.25	GO:004423	cytoplasmic	NP_780628	mmu:109275	
K03099	SOS	mmu05208(	PF00617.24	GO:000548	cytoplasmic	NP_001129	mmu:20663	
K04425	MAP3K6, A	mmu04010(	PF13281.12	GO:003563	cytoplasmic	NP_057902	mmu:53608	
K14575	AFG2, DR(	mmu03008(	PF00004.35	GO:000810	cytoplasmic	NP_067318	mmu:57815	
K24990	RBMS		PF00076.28	GO:008009	cytoplasmic	EDL09262.	mmu:207181	
K24127	MAGE		PF01454.25	GO:000582	nuclear	XP_011246	mmu:80884	
K20369	ERV29, SURF4		PF02077.20	GO:003802	cytoplasmic	NP_001029	mmu:20932	
K09441	GABPA		PF00178.28	GO:008009	cytoplasmic	NP_001403	mmu:14390	ETS
K09454	GABPB		PF00023.36	GO:000548	nuclear	NP_001258	mmu:14391	TF_others
K12884	HNRNPC	mmu03040(	PF00076.28	GO:003299	nuclear	KAI257027	mmu:15381	
KT5U(KDEL (Lys-Asp-Glu-Leu) co			PF05686.18	GO:004341	ER	NP_076134	mmu:72050	
K10177	TBX3	mmu04550(	PF00907.27	GO:008009	nuclear	NP_035665	mmu:21386	T-box
K17639	RGL3		PF00617.24	GO:000508	cytoplasmic	NP_076111	mmu:71746	
K11450	KDM1A, A	mmu04714(	PF01593.30	GO:200123	cytoplasmic	NP_001334	mmu:99982	
K09654	B3GALNT	mmu00515(	PF01762.27	GO:000578	ER	NP_001349	mmu:97884	
K14415	RTCB, rtcB		PF01139.22	GO:007266	nuclear	BAE39694.	mmu:28088	
K00615	E2.2.1.1, tkt	mmu00030(	PF02779.29	GO:000480	cytoplasmic	NP_001258	mmu:74419	
K05766	SSH	mmu04810(	PF23040.1(I	GO:000806	cytoplasmic	NP_001278	mmu:237860	
KT5P(Family with sequence similarity 190, mem				GO:004322	cytoplasmic	NP_082683	mmu:72972	
YSN6(phosphatase domain containi			PF14566.11	GO:000573	cytoplasmic	AAI44847.1	mmu:27355	
K06545	CD163		PF00530.23	GO:000425	extracellulo	NP_001163	mmu:93671	
K04440	JNK	mmu05208(	PF00069.31	GO:200123	cytoplasmic	NP_059018	mmu:26420	
K03905	FGG	mmu04610(	PF00147.23	GO:200123	ER	NP_598623	mmu:99571	
K10365	CAPZB	mmu04144(	PF01115.22	GO:003315	cytoplasmic	NP_001032	mmu:12345	
K02581	NFKBIB	mmu05164(	PF00023.36	GO:007122	cytoplasmic	NP_001293	mmu:18036	
K19759	DNAAF5		PF24573.1(I	GO:002260	cytoplasmic	NP_001074	mmu:433956	
K03595	era, ERAL1		PF01926.29	GO:002260	mitochondr	NP_071708	mmu:57837	
K01647	CS, gltA	mmu01100(	PF00285.26	GO:000663	mitochondr	NP_080720	mmu:12974	
K06068	PRKCD	mmu05208(	PF00069.31	GO:003315	cytoplasmic	XP_006518	mmu:18753	
K14776	DDX10, DBP4		PF00270.35	GO:004885	nuclear	AAH49261.	mmu:77591	

K19269	PGP, PGLI	mmu00630(	PF13344.12	GO:000988	cytoplasmic	NP_080230	mmu:67078	
K23730	CCDC40		PF08647.16	GO:004859	cytoplasmic	NP_001351	mmu:207607	
K12871	CCDC12	mmu03040(	PF08315.17	GO:000557	nuclear	XP_036047	mmu:72654	
K11419	SUV39H, C	mmu00310(	PF00856.33	GO:008009	cytoplasmic	NP_001277	mmu:20937	
K09283	HMGA2	mmu05202(	Transcripti	GO:003598	nuclear	NP_034571	mmu:15364	HMGA
K03331	DCXR	mmu00040(	PF13561.12	GO:000810	mitochondr	NP_001334	mmu:67880	
K14294	WIBG, PYM	mmu03013(	PF09282.15	GO:003514	cytoplasmic	NP_084376	mmu:78428	
K26243	HP1BP3		PF00538.25	GO:003472	nuclear	NP_001116	mmu:15441	
K13025	EIF4A3, FA	mmu03013(	PF00270.35	GO:005116	cytoplasmic	NP_619610	mmu:192170	
K12328	PPP1R14A,	mmu04270(	PF05361.17	GO:001922	cytoplasmic	NP_081007	mmu:68458	
K21890	GHITM, DERP2, TMB		PF01027.25	GO:200123	mitochondr	NP_001186	mmu:66092	
K11304	TIP60, KA	mmu03082(	PF01853.24	GO:000167	cytoplasmic	NP_001005	mmu:81601	
K12260	SRX1			GO:003563	cytoplasmic	XP_021005	mmu:76650	
K03260	EIF4G	mmu05416(	PF02854.25(MIF4G:MI		cytoplasmic	XP_017175	mmu:230861	
K14021	BAK, BAK	mmu05022(	PF00452.24	GO:200123	cytoplasmic	NP_031549	mmu:12018	
K04741	CASP12	mmu05161(	PF00656.27	GO:000578	cytoplasmic	NP_033938	mmu:12364	
K04396	CASP6	mmu04210(	PF00656.27	GO:004858	cytoplasmic	NP_033941	mmu:12368	
K04569	CCS	mmu05014(	PF00080.25	GO:003197	cytoplasmic	NP_058588	mmu:12460	
K23926	PVALB		PF13499.12	GO:003243	cytoplasmic	NP_001317	mmu:19293	
K14322	PAIP1		PF02854.25	GO:000989	cytoplasmic	NP_001073	mmu:218693	
K04365	BRAF	mmu05208(	PF07714.23	GO:005104	cytoplasmic	P28028.4(R	mmu:109880	
K14347	SLC10A7, P7		PF13593.11	GO:001972	ER	NP_084012	mmu:76775	
K19934	SDF4		PF00036.39	GO:009955	ER	NP_001289	mmu:20318	
K23166	OPA3		PF07047.17	GO:008009	mitochondr	NP_997408	mmu:403187	
K14165	K14165		PF00782.25	GO:001679	cytoplasmic	NP_082375	mmu:72102	
K07608	NF66, INA		PF00038.26	GO:000367	cytoplasmic	NP_666212	mmu:226180	
K16684	NF2	mmu04390(	PF00373.23	GO:001922	cytoplasmic	NP_035028	mmu:18016	
K26188	ARMCX2		PF04826.19	GO:003196	peroxisoma	BAD32238.	mmu:67416	
K23781	ADAP, CENTA		PF01412.23	GO:001602	cytoplasmic	BAE20669.	mmu:216991	
K11798	BRWD1_3		PF00400.38	GO:002260	nuclear	NP_001074	mmu:382236	
K01231	MAN2	mmu00510(	PF01074.27	GO:004428	Golgi appa	NP_032575	mmu:17158	
K11086	SNRPB, SM	mmu05322(	PF01423.28	GO:002260	cytoplasmic	NP_033251	mmu:20638	
K14763	NAF1		PF04410.20	GO:008009	nuclear	NP_001157	mmu:234344	
K01512	acyP	mmu00620(	PF00708.24	GO:001678	plasma mer	NP_001351	mmu:66204	
YE0(immunoglobulin heavy variab			PF07686.23	GO:000548	cytoplasmic	P06330.1(RecName: Full=Ig heavy		
K03514	PAPD5_7,	mmu03018(	PF22600.2(	GO:007104	nuclear	NP_001359	mmu:214627	
K06751	MHC1	mmu05165(	PF00129.23	GO:000989	extracellulo	NP_034528	mmu:15040	
K07375	TUBB	mmu05020(	PF00091.31	GO:000520	cytoplasmic	NP_001029	mmu:227613	
K03032	PSMD1, RF	mmu05020(	PF21505.3(	GO:000989	nuclear	NP_081633	mmu:70247	
K10755	RFC2_4	mmu03030(	PF08542.16	GO:008009	nuclear	NP_064406	mmu:19718	
K12331	ARHGEF1	mmu05200(	PF09128.16	GO:005104	cytoplasmic	NP_001347	mmu:213498	
K24185	KIF21	mmu04814(	PF00225.29	GO:190136	nuclear	EDL39556.	mmu:16565	
K25437	OXR1		PF07534.21	GO:000989	cytoplasmic	NP_001123	mmu:170719	

K06766	NEO1	mmu04360(	PF06583.17	GO:000810	plasma mer	NP_032710	mmu:18007
K14963	WDR5, SW	mmu04934(	PF00400.38	GO:003506	cytoplasmic	XP_062041	mmu:140858
K12394	AP1S1_2	mmu04142(	PF01217.26	GO:000810	cytoplasmic	NP_001101	mmu:11769
K12162	UFM1		PF03671.20	GO:003315	cytoplasmic	NP_001244	mmu:67890
K11805	DCAF7, H/	mmu03083(	PF24807.1(	GO:004859	nuclear	NP_001033	mmu:71833
K23793	PHLDA			GO:005138	cytoplasmic	NP_033370	mmu:21664
K04553	UBE2L6, U	mmu04120(	PF00179.31	GO:001707	cytoplasmic	NP_064333	mmu:56791
K23505	TIMMDC1		PF02466.24	GO:000557	mitochondr	NP_077235	mmu:76916
K06943	NOG1	mmu03008(	PF17835.6(	GO:001922	nuclear	NP_081276	mmu:69237
K02270	COX7A	mmu05208(	PF02238.20	GO:001545	mitochondr	NP_001399	mmu:12865
K07560	dtd, DTD		PF02580.21	GO:000367	cytoplasmic	NP_079590	mmu:66044
K12557	MYBPC1	mmu04820(	PF07679.22(I-set:Immu		cytoplasmic	XP_006513	mmu:109272
K01325	KLK1_2	mmu04614(	PF00089.32	GO:000425	lysosomal	NP_034770	mmu:16613
K07298	STK11, LK	mmu04530(	PF00069.31	GO:005124	cytoplasmic	NP_035622	mmu:20869
K07410	CYP1B1	mmu00980(	PF00067.27	GO:010102	ER	NP_034124	mmu:13078
K23290	PACS1		PF10254.14	GO:000810	nuclear	NP_694769	mmu:107975
K11406	HDAC4_5	mmu04371(	PF00850.24(Hist_deace		cytoplasmic	EDL40019.	mmu:208727
K08490	STX5	mmu04130(	PF05739.25	GO:000989	cytoplasmic	NP_001161	mmu:56389
D5W(tumor necrosis factor recepto			PF00531.28	GO:000512	plasma mer	NP_080288	mmu:67169
K16743	ASPM, ASP		PF00612.33	GO:009951	nuclear	NP_033921	mmu:12316
K22255	ATPIF1		PF04568.17	GO:001922	cytoplasmic	NP_001407	mmu:11983
K02978	RP-S27e, R	mmu05171(	PF01667.23	GO:002260	nuclear	EFB25803.1	mmu:100043813
K12749	MYL3	mmu04260(	PF13405.12	GO:005501	cytoplasmic	XP_028642	mmu:17897
K01191	MAN2C1	mmu00511(	PF01074.27	GO:000931	cytoplasmic	AQS27599.	mmu:73744
K01158	DNASE2	mmu04142(	PF03265.21	GO:000032	cytoplasmic	NP_001355	mmu:13423
K09505	DNAJA4		PF01556.23	GO:001707	cytoplasmic	AQS27598.	mmu:58233
K02147	ATPeV1B,	mmu05165(	PF00006.31	GO:004877	cytoplasmic	XP_021026	mmu:11966
K02996	RP-S9, MRI	mmu03010(	PF00380.25	GO:001593	mitochondr	AQS27590.	mmu:69527
K01763	SCLY	mmu00450(	PF00266.25	GO:190169	peroxisoma	NP_057926	mmu:50880
K05036	SLC6A3, D	mmu05031(	PF00209.24	GO:008009	cytoplasmic	BAE23154.	mmu:13162
K08342	ATG4	mmu04140(	PF03416.26	GO:000810	cytoplasmic	NP_777363	mmu:66615
K07566	tsaC, rimN, SUA5, YRI		PF01300.23	GO:000645	mitochondr	NP_705794	mmu:230734
K20193	HPS1		PF19036.6(I	GO:004806	cytoplasmic	NP_062297	mmu:192236
K02366	EXT1	mmu00534(	PF09258.16	GO:004280	mitochondr	NP_001124	mmu:14042
K14752	DOK1		PF02174.23	GO:003814	mitochondr	NP_034200	mmu:13448
K24317	MCM3AP		PF16769.10	GO:003472	nuclear	NP_062307	mmu:54387
XSYS(importin 13)			PF03803.21	GO:200123	cytoplasmic	BAE29156.	mmu:22038
IAF(Ribosome binding factor A)			PF01284.28	GO:000810	plasma mer	BAE26621.	mmu:67213
K22415	ZC3H11		PF15663.11	GO:001593	cytoplasmic	NP_001263	mmu:70579
K17510	STYK1		PF07714.23	GO:003802	plasma mer	AAI50725.1	mmu:243659
K24774	FNBP4		PF00397.32(WW:WW d		cytoplasmic	AAF59410.	mmu:55935
K24050	DLG5	mmu04390(	PF00595.29	GO:004213	cytoplasmic	NP_082002	mmu:71228
K19619	TDP2		PF03372.29	GO:003250	cytoplasmic	NP_062424	mmu:56196

K06836	ADAM15		PF01421.25	GO:004858	plasma mer	EDL15207.	mmu:11490	
K05857	PLCD	mmu01100(	PF00388.25	GO:000578	cytoplasmic	NP_683739	mmu:18802	
K12868	SYF2	mmu03040(	PF08231.17	GO:000716	nuclear	NP_081056	mmu:68592	
K19347	SUN1_2	mmu04820(	PF09387.16	GO:010609	mitochondr	ADW08713	mmu:77053	
K13711	PI4K2	mmu04070(	PF00454.33	GO:005128	cytoplasmic	NP_663476	mmu:84095	
K06850	SLIT3	mmu04360(	PF13855.12	GO:004867	extracellula	NP_035542	mmu:20564	
K11166	DHRS7B		PF00106.31	GO:008009	peroxisoma	NP_663403	mmu:216820	
K21852	DOCK6_7_8		PF06920.19	GO:007023	cytoplasmic	AAI41359.1	mmu:76088	
K23114	ARMT1		PF01937.24	GO:001679	cytoplasmic	NP_077223	mmu:73419	
K09549	PFDN2		PF01920.25	GO:000645	cytoplasmic	NP_035200	mmu:18637	
K27409	SPTB		PF00435.27	GO:004299	cytoplasmic	AAI50784.1	mmu:20741	
K18267	NEB	mmu04820(	PF00880.23	GO:000701	nuclear	AAF59979.	mmu:17996	
K14640	SLC20A, PIT		PF01384.25	GO:009859	ER	NP_001411	mmu:20516	
K19496	ANO1, DOG1, TMEM		PF04547.17(Anoctamin		cytoplasmic	XP_021022	mmu:101772	
K14565	NOP58	mmu03008(	PF01798.23	GO:003204	nuclear	NP_061356	mmu:55989	
K00134	GAPDH, g	mmu01100(	PF02800.25	GO:004643	mitochondr	NP_001276	mmu:14433	
K18618	GAS7		PF16623.10	GO:002260	cytoplasmic	NP_001264	mmu:14457	
XPZC(tRNA splicing endonuclease			PF10268.14	GO:000557	plasma mer	NP_780396	mmu:72745	
K15593	ETV5	mmu05202(	PF04621.18	GO:008009	cytoplasmic	BAC37975.	mmu:10415	ETS
K03953	NDUFA9	mmu05208(	PF01073.25	GO:003197	peroxisoma	XP_006237	mmu:66108	
			PF23015.2(zf-WIZ:Zinc		cytoplasmic	NP_001359	mmu:22404	zf-C2H2
K27340	UQCC5, SMIM4		PF15114.11	GO:003361	mitochondr	NP_001295	mmu:66487	
K22144	TEME2		PF24606.1((	GO:003020	peroxisoma	NP_001028	mmu:83921	
K11501	CENPI		PF07778.16	GO:003408	nuclear	BAE38693.	mmu:102920	
K03359	APC12, CD	mmu04110(	PF10471.14	GO:199023	cytoplasmic	NP_001355	mmu:66440	
K00681	ggt	mmu00430(	PF01019.26	GO:004239	cytoplasmic	NP_659035	mmu:207182	
K15010	HOMER	mmu04724(	PF00568.29	GO:001602	cytoplasmic	AAH05773.	mmu:26558	
K07297	ADIPOR	mmu04152(	PF03006.26	GO:003444	cytoplasmic	BAE40044.	mmu:72674	
K01251	AHCY, ahc	mmu00270(	PF05221.23	GO:000578	cytoplasmic	NP_001291	mmu:11615	
K18849	BMT2		PF11968.14	GO:004259	cytoplasmic	NP_780521	mmu:101148	
K00542	GAMT	mmu00330(Arginine an		GO:004863	cytoplasmic	NP_034385	mmu:14431	
K26199	BCORL1	mmu03083(	PF16553.11	GO:001602	nuclear	NP_848897	mmu:320376	
K00237	SDHD, SD	mmu05208(	PF05328.17	GO:000810	mitochondr	NP_080124	mmu:66925	
K14383	SLC5A3, SMIT		PF00474.23	GO:001922	ER	NP_059087	mmu:53881	
K07920	RAB33B	mmu04140(	PF00071.28	GO:000004	cytoplasmic	NP_058554	mmu:19338	
K17824	DCUN1D4_5		PF03556.21	GO:190187	cytoplasmic	NP_084051	mmu:76863	
K10388	PLEC	mmu04820(	PF00681.25	GO:001922	cytoplasmic	NP_001157	mmu:18810	
K01737	queD, ptpS	mmu01240(	PF01242.24	GO:000548	mitochondr	NP_035350	mmu:19286	
YW74(-); ENOG4111FCX(leucine rich repeat cc				GO:004212	cytoplasmic	NP_001028	mmu:545662	
K05302	SETD6		PF09273.16	GO:000647	nuclear	NP_001030	mmu:66083	
K12309	GLB1, ELN	mmu00052(	PF01301.25	GO:001932	lysosomal	NP_033882	mmu:12091	
K07835	RALB	mmu04014(	PF00071.28	GO:001922	cytoplasmic	BAC28193.	mmu:64143	
GTB(Family with sequence similar			PF10239.14	GO:000557	nuclear	NP_001139	mmu:73833	

K26456	TANC		PF12796.13	GO:000763	nuclear	AAI41368.1	mmu:66860	
K10392	KIF1	mmu04814(	PF00225.29	GO:190195	cytoplasmic	NP_032467	mmu:16562	
Guanylate binding protein)			PF13855.12	GO:000810	mitochondr	NP_001158	mmu:21983	
K10777	LIG4, DNL	mmu03450(	PF01068.26	GO:003304	nuclear	BAC38882.	mmu:319583	
K10226	FADS2	mmu03320(	PF00487.30	GO:000663	cytoplasmic	ADZ54082.	mmu:56473	
K07987	RAET1	mmu04650(	PF14586.11	GO:001922	cytoplasmic	NP_001346	mmu:379043	
K10735	GINS4, SLD5		PF16922.11	GO:007110	cytoplasmic	NP_077202	mmu:109145	
K02863	RP-L1, MR	mmu03010(	PF00687.26	GO:001593	mitochondr	NP_444388	mmu:94061	
K25740	SSC5D		PF00530.23	GO:000425	extracellulo	NP_766596	mmu:269855	
K10350	ZYG11		PF22964.2(	GO:000989	cytoplasmic	NP_001277	mmu:227693	
K02899	RP-L27, MF	mmu03010(	PF01016.24	GO:001593	mitochondr	NP_444391	mmu:94064	
K02878	RP-L16, MF	mmu03010(	PF00252.24	GO:001593	mitochondr	NP_079882	mmu:94063	
K03900	VWF	mmu05165(	PF00094.30	GO:000759	plasma mer	NP_035838	mmu:22371	
K25722	PRG3		PF00059.27	GO:000548	extracellulo	NP_058610	mmu:53856	
K20828	ZNF143_76	mmu04980(	PF13465.12(zf-H2C2_2:		cytoplasmic	NP_001344	mmu:22465	zf-C2H2
K01128	SMPDL3		PF19272.4(	GO:000548	lysosomal	NP_065586	mmu:57319	
K23922	EFHD2		PF21008.3(	GO:004316	cytoplasmic	NP_080270	mmu:27984	
K08272	CAB39, MO	mmu04152(	PF08569.17	GO:001920	cytoplasmic	NP_001341	mmu:12283	
OMPF(claudin domain containing 1)			PF13903.11	GO:000557	plasma mer	NP_001343	mmu:224250	
K22610	RSBN1, KDM9			GO:001649	mitochondr	NP_001343	mmu:229675	
K03120	TBP, tbp	mmu05165(	PF00352.27	GO:000097	nuclear	NP_038712	mmu:21374	
KSWP(WD repeat and SOCS box co			PF07978.19	GO:003241	mitochondr	O55126.1(R	mmu:14467	
K24104	GPN		PF03029.23	GO:003563	cytoplasmic	NP_077178	mmu:68080	
KSWP(Phosphodiesterase 6D, cGMI			PF07978.19	GO:004323	mitochondr	NP_032724	mmu:18082	
K27288	ACBD4_5		PF00887.24	GO:000663	ER	NP_001095	mmu:74159	
K15085	SLC25A42		PF00153.32	GO:000534	cytoplasmic	NP_001007	mmu:73095	
K11345	ING5		PF12998.12	GO:200123	nuclear	NP_079730	mmu:66262	
K09228	KRAB		PF01352.32	GO:000989	nuclear	NP_001013	mmu:22715	zf-C2H2
K04345	PKA	mmu04750(	PF00069.31	GO:003315	cytoplasmic	NP_032880	mmu:18747	
K14536	RIA1	mmu03008(	PF00009.33	GO:002260	cytoplasmic	EDL06898.	mmu:101592	
K24812	HEATR3		PF13513.12	GO:003450	cytoplasmic	BAE24413.	mmu:234549	
K03177	truB, PUS4, TRUB1		PF01509.23	GO:190036	nuclear	NP_082391	mmu:72133	
K20720	RTN4	mmu05010(	PF02453.23	GO:007266	cytoplasmic	AAM77069.	mmu:68585	
K08371	CYB561D2			GO:000578	ER	KAI252980	mmu:56368	
K08745	SLC27A1_4	mmu04931(	PF00501.34	GO:001564	ER	EDL28942.	mmu:26457	
K12172	RANBP2, N	mmu03013(	PF00638.24	GO:005116	cytoplasmic	NP_035370	mmu:19386	
K16764	CEP68			GO:000810	cytoplasmic	EDL07833.	mmu:216543	
K11866	STAMBP, A	mmu04144(	PF08969.16	GO:000548	cytoplasmic	BAE40637.	mmu:70527	
K03097	CSNK2A	mmu04137(	PF00069.31	GO:008009	cytoplasmic	NP_034104	mmu:13000	
			PF00103.25	GO:000512	extracellulo	NP_001157	mmu:19113	
K16779	RAB3IP, RABIN8		PF06428.16	GO:007031	cytoplasmic	NP_001346	mmu:216363	
K20631	ARHGAP6		PF00620.32	GO:001989	cytoplasmic	NP_033837	mmu:11856	
K03996	C7	mmu04610(	PF01823.24	GO:005080	plasma mer	NP_001230	mmu:109828	

K24786	RUNDC1		PF02759.24	GO:003250:	cytoplasmic	NP_766154	mmu:217201	
.YE0(immunoglobulin heavy variab			PF07686.23(V-set:Immu		cytoplasmic	AAG39930.1(immunoglobulin heav		
K07002	RBBP9		PF06821.19	GO:003250:	cytoplasmic	NP_056569	mmu:26450	
K10798	PARP3_4	mmu04210(	PF00644.27	GO:004423:	nuclear	NP_033762	mmu:11546	
K15133	MED17	mmu04919(	PF10156.14	GO:008009	nuclear	NP_659182	mmu:234959	
K10951	SLC12A2, N	mmu04970(	PF03522.20	GO:003000:	cytoplasmic	NP_033220	mmu:20496	
K25567	JCHAIN		PF15097.11	GO:006026:	plasma mer	NP_690052	mmu:16069	
K08518	STXBP5, SRO7_77		PF08366.18	GO:000548	cytoplasmic	NP_001074	mmu:78808	
K14411	MSI	mmu03015(	PF00076.28	GO:004885	cytoplasmic	BAE31119.1	mmu:70248	
K16576	ACTR10, A	mmu05014(	PF00022.25	GO:001989	cytoplasmic	NP_062759	mmu:56444	
K22531	ATAD2		PF00004.35	GO:001707	nuclear	NP_081711	mmu:70472	
KPJI(kelch domain containing 4)			PF24681.1(Kelch_KLH		nuclear	NP_663580	mmu:234825	
K20655	SYDE		PF00620.32	GO:000690	nuclear	NP_082151	mmu:71709	
K16641	HDGF		PF00855.23	GO:000810	nuclear	TEA28906.1	mmu:15191	
K06058	DTX	mmu04330(	PF18102.6(I	GO:000810	cytoplasmic	NP_001013	mmu:209200	
K07877	RAB2A	mmu04152(	PF00071.28	GO:000810	cytoplasmic	NP_067493	mmu:59021	
K09250	CNBP		PF00098.28	GO:004212	cytoplasmic	XP_059114	mmu:12785	TF_others
K18024	PTPN12_18_22		PF00102.32	GO:003250:	cytoplasmic	NP_035333	mmu:19248	
K08744	CRLS	mmu00564(	Glycerophо	GO:000665:	mitochondr	NP_001019	mmu:66586	
K09546	HSPB7		PF00011.27	GO:001562	cytoplasmic	NP_038896	mmu:29818	
K14527	RPP20, PO	mmu03008(	PF12328.13	GO:000454	nuclear	NP_083029	mmu:74097	
37S ribosomal protein RSM22)			PF09243.16	GO:001593:	mitochondr	NP_001025	mmu:52535	
K24687	ZFAND5_6		PF01754.22	GO:014003	cytoplasmic	NP_001007	mmu:65098	
K04729	MYD88	mmu05143(	PF00531.28	GO:001989	cytoplasmic	NP_034981	mmu:17874	
K24776	RUFY3		PF02759.24	GO:003315	cytoplasmic	NP_001276	mmu:52822	
K17382	LACTB		PF00144.29	GO:008009	plasma mer	NP_109642	mmu:80907	
K08493	VTI1	mmu04130(	PF05008.21	GO:000810	cytoplasmic	NP_001347	mmu:53611	
.PY2(Family with sequence similari			PF15388.11(FAM117:Pr		cytoplasmic	NP_001032	mmu:72750	
K03241	EIF2B3	mmu05168(	PF25084.1(I	GO:000548	cytoplasmic	NP_001104	mmu:108067	
K20101	DHX34		PF24485.1(z	GO:001922	cytoplasmic	NP_001272	mmu:71723	
)RFF(coiled-coil domain containing			PF13821.11	GO:000557:	nuclear	NP_001390	mmu:53951	
K12878	THOC1	mmu03013(	PF11957.13	GO:001593	mitochondr	NP_705780	mmu:225160	
K16575	ACTR1, AR	mmu05014(	PF00022.25	GO:000573	cytoplasmic	NP_001034	mmu:226977	
K14766	NOP14, UTP2		PF04147.17	GO:003068	nuclear	NP_083554	mmu:75416	
K10095	LGALS12		PF00337.29	GO:004213	cytoplasmic	BAC39498.1	mmu:56072	
K07871	RHOT2, AI	mmu04137(	PF08356.18	GO:006102	cytoplasmic	NP_666111	mmu:214952	
K12230	PIK3AP1, I	mmu04151(	PF14545.12	GO:000548	plasma mer	NP_113553	mmu:83490	
K15980	ACAD9		PF00441.29	GO:003444	mitochondr	NP_766266	mmu:229211	
K00914	PIK3C3, VI	mmu04070(	PF00613.26	GO:000810	cytoplasmic	NP_852079	mmu:225326	
.C4T(zinc finger CCCH-type, antivi			PF18606.7(I	GO:004316	extracellulo	NP_766055	mmu:209032	
K24167	ARMC10		PF04826.19	GO:200123	ER	NP_080310	mmu:67211	
K09103	EBF, COE		PF16422.10(COE1_DBI		mitochondr	NP_001402	mmu:22859	COE
K10683	BARD1	mmu03440(	PF14835.12	GO:003315	cytoplasmic	NP_031551	mmu:12021	

ZS9E(Family with sequence similari			PF10223.15	GO:004866	cytoplasmic	NP_001157	mmu:73942	
K01689	ENO1_2_3	mmu04820(	PF00113.28	GO:004643	cytoplasmic	NP_001129	mmu:13808	
HS1(Coiled-coil domain containing			PF15266.11	(DUF4594:I	cytoplasmic	NP_001129	mmu:243846	
K11262	ACACA	mmu00640(	PF08326.17	GO:005128	cytoplasmic	XP_006532	mmu:107476	
K13107	RBMX2, IST3		PF00076.28	GO:000578	nuclear	BAE35704.1	mmu:209003	
K16749	BBS7		PF23743.1(I	GO:000810	cytoplasmic	EDL35087.1	mmu:71492	
K26500	NOMO1_2_3		PF22904.2(	GO:000810	ER	NP_694697	mmu:211548	
K16570	TUBGCP3, GCP3		PF04130.18	GO:000705	cytoplasmic	NP_932148	mmu:259279	
K25826	CBFB		PF02312.22	GO:008009	nuclear	BAE22149.1	mmu:12400	CBF
IF3(TRAF-type zinc finger domain			PF21366.3(	GO:003210	cytoplasmic	NP_001156	mmu:231712	
K03910	A2M	mmu04610(	PF07678.20	GO:003041	ER	NP_783327	mmu:232345	
K02125	ATPeF08, M	mmu05208(	PF00895.25	GO:000920	extracellulo	AHG32153.	mmu:17706	
K18401	KANSL2		PF13891.11	GO:003124	nuclear	NP_001346	mmu:69612	
K26682	SCAP		PF24017.1(I	GO:008009	ER	NP_001001	mmu:235623	
K11515	INCENP		PF03941.20	GO:009951	cytoplasmic	XP_006526	mmu:16319	
K08268	HIF1A	mmu05208(	PF23171.1(I	GO:005165	nuclear	NP_001300	mmu:15251	
K10844	ERCC2, XP	mmu03420(	PF06777.17	GO:008009	cytoplasmic	NP_001350	mmu:13871	
K02886	RP-L2, MR	mmu03010(	PF03947.24	GO:001593	mitochondr	NP_079578	mmu:27398	
K16538	PIBF1, CEP90			GO:000810	nuclear	NP_083596	mmu:52023	
K03231	EEF1A	mmu03013(	PF00009.33	GO:002262	cytoplasmic	XP_007515	mmu:13627	
K01132	GALNS	mmu04142(	PF00884.28	GO:003020	lysosomal	NP_057931	mmu:50917	
K12197	CHMP1, VI	mmu03272(	PF03357.26	GO:000810	cytoplasmic	NP_001128	mmu:67028	
K19326	SYNE1	mmu04820(	PF00435.27	GO:000699	cytoplasmic	NP_001334	mmu:64009	
K07887	RAB5A	mmu04137(	PF00071.28	GO:000810	cytoplasmic	NP_080163	mmu:271457	
6VB(Chromosome 9 open reading f			PF15017.11	(WRNPLPN	cytoplasmic	NP_808308	mmu:225995	
K11730	ACAD11		PF01636.29	GO:003444	peroxisoma	NP_780533	mmu:102632	
K15502	ANKRD28		PF00023.36	(Ank:Ankyri	cytoplasmic	NP_001389	mmu:105522	
K03991	MBL	mmu04610(	PF00059.27	GO:008009	extracellulo	NP_001351	mmu:17195	
K11968	ARIH1	mmu04137(	PF01485.27	GO:003243	cytoplasmic	NP_001075	mmu:23806	
K04525	SERPINA		PF00079.25	GO:000486	extracellulo	NP_035588	mmu:20714	
K12948	SPCS3, SPC	mmu03060(	PF04573.17	GO:000578	ER	NP_083817	mmu:76976	
ADP-ribosyltransferase 3)			PF01979.26	GO:000573	cytoplasmic	BAE26556.1	mmu:26757	
K08759	APOC3	mmu04979(	PF05778.17	GO:000167	extracellulo	NP_001276	mmu:11814	
K12376	ARSK		PF00884.28	GO:000032	lysosomal	AAH46790.	mmu:77041	
K10380	ANK1	mmu05205(	PF00023.36	GO:000810	cytoplasmic	XP_006509	mmu:11733	
2CA8(selenoprotein M)			PF08806.17	GO:000578	ER	NP_444497	mmu:114679	
K11830	TFAM, MT	mmu05016(	PF00505.25	GO:008009	nuclear	AAA02579.	mmu:21780	HMG
K11161	RDH13	mmu00830(	PF00106.31	GO:000403	ER	NP_001277	mmu:108841	
K00162	PDHB, pdh	mmu05230(	PF02779.29	GO:004423	mitochondr	NP_077183	mmu:68263	
K05630	WWP2, AIP	mmu04120(	PF00632.30	GO:008009	mitochondr	NP_080106	mmu:66894	
K13907	CSTA_B		PF00031.27	GO:000573	cytoplasmic	EDL02193.	mmu:20861	
K18438	PDE10	mmu04024(	PF00233.25	GO:000921	cytoplasmic	NP_001277	mmu:23984	
K10585	UBE2Z	mmu04120(	PF00179.31	GO:000548	cytoplasmic	NP_758504	mmu:268470	

K00164	OGDH, suc	mmu01200(	PF00676.26	GO:004643	mitochondr	NP_001239	mmu:18293
K24477	ABI3BP		PF21731.2(	GO:005124	plasma mer	XP_006522	mmu:320712
K11165	DHRS7		PF00106.31	GO:000403	peroxisoma	NP_079798	mmu:66375
K00485	FMO	mmu00982(	PF00743.25	GO:000403	cytoplasmic	NP_001155	mmu:14263
K13883	RILP	mmu05132(	PF11461.13	GO:000810	cytoplasmic	NP_001025	mmu:280408
K07530	RHOD	mmu04360(	PF00071.28	GO:000810	cytoplasmic	XP_006531	mmu:11854
K14001	SIL1, SLS1	mmu04141(	Protein pro	GO:000810	ER	NP_001347	mmu:81500
K12042	TNNC2	mmu04020(	PF13499.12	GO:000694	cytoplasmic	NP_001032	mmu:21925
K22697	SAMD8	mmu00564(	PF14360.12	GO:001921	mitochondr	NP_080559	mmu:67630
K12867	SYF1, XAB	mmu03040(	PF23231.1(	GO:002260	nuclear	BAE26756.	mmu:67439
K15300	STXBP2, MUNC18-2		PF00995.28	GO:000810	cytoplasmic	AAH03477.	mmu:20911
K08879	HSPB8		PF00011.27	GO:000989	cytoplasmic	NP_109629	mmu:80888
K01022	CHST1	mmu00533(	PF00685.33	GO:003020	Golgi appa	NP_001343	mmu:76969
K12845	SNU13, NH	mmu03008(	PF01248.31	GO:000037	nuclear	NP_001003	mmu:20826
K00548	metH, MTR	mmu00670(	PF02965.22	GO:004867	cytoplasmic	NP_001074	mmu:238505
K13197	IGF2BP3		PF00013.35	GO:199024	cytoplasmic	NP_076159	mmu:140488
K03039	PSMD13, R	mmu05020(	PF22037.2(	GO:000417	nuclear	NP_036005	mmu:23997
K07611	LMNB	mmu04210(	PF00038.26	GO:000810	nuclear	NP_034851	mmu:16906
K13134	GEMIN6, SIP2		PF20417.3(	GO:002260	cytoplasmic	NP_001342	mmu:67242
K11848	USP20_33		PF00443.35	GO:000578	nuclear	NP_083122	mmu:74270
K11124	SMG6, EST	mmu03015(	PF10373.15	GO:008009	cytoplasmic	NP_001002	mmu:103677
K05236	COPA, RET1		PF06957.17	GO:000810	cytoplasmic	XP_036013	mmu:12847
K15044	AGFG1		PF01412.23	GO:002260	cytoplasmic	XP_006496	mmu:15463
K08656	DPP9		PF00930.27	GO:000417	cytoplasmic	NP_001347	mmu:224897
K06108	RAB3B		PF00071.28	GO:000810	cytoplasmic	NP_076026	mmu:69908
K09566	PPIG		PF00160.26	GO:000645	nuclear	NP_001074	mmu:228005
K19682	IFT46		PF12317.13	GO:009859	cytoplasmic	XP_006510	mmu:76568
K08863	PLK4	mmu04068(	PF00069.31	GO:190136	cytoplasmic	NP_035625	mmu:20873
K10609	CUL4	mmu03420(	PF00888.28	GO:008009	nuclear	NP_001103	mmu:72584
K26680	DOP1, DOPEY		PF24601.1(	GO:004323	cytoplasmic	NP_081569	mmu:70028
K06644	SFN	mmu04115(	PF00244.26	GO:003315	cytoplasmic	NP_061224	mmu:55948
K20642	ARHGAP22_24_25		PF00620.32	GO:003015	cytoplasmic	NP_722495	mmu:239027
Zinc finger, CCHC domain containi			PF00753.33	GO:000663	cytoplasmic	NP_082648	mmu:72852
K17653	TFB2M		PF00398.26	GO:003277	mitochondr	NP_032275	mmu:15278
K18033	PTPRE		PF00102.32	GO:001679	extracellulo	NP_001303	mmu:19267
K01507	ppa	mmu00190(	PF00719.24	GO:004239	mitochondr	NP_666253	mmu:74776
K16752	CCDC15			GO:000557	cytoplasmic	NP_001074	mmu:245902
K11674	MCRS1, IN	mmu03082(	PF13325.12	GO:008009	nuclear	NP_001157	mmu:51812
K16857	THBS1	mmu05165(	PF05735.19	GO:001701	plasma mer	NP_001300	mmu:21825
K05682	ABCG3, M	mmu02010(	PF01061.29	GO:003563	cytoplasmic	NP_001406	mmu:27405
ZJC(coiled-coil domain containing			PF15810.10	GO:004212	cytoplasmic	NP_598794	mmu:104479
243X(family with sequence similarit			PF06916.18	GO:004846	plasma mer	BAE28489.	mmu:67017
K14282	TACC2		PF05010.19	GO:000367	cytoplasmic	NP_001004	mmu:57752

K10086	EDEM3	mmu04141(	PF01532.26	GO:190169	Golgi appa	XP_011246	mmu:66967	
K15046	IVNS1ABP, NS1BP		PF01344.31	GO:200123	nuclear	AAH04092.	mmu:11719	ZBTB
K23972	FRMD8	mmu04668(	PF24522.1(I	GO:000810	cytoplasmic	NP_080445	mmu:67457	
K08838	STK24_25_MST4		PF00069.31	GO:000740	cytoplasmic	BAE31594.	mmu:59041	
K04449	JUND	mmu04010(	PF03957.18	GO:008009	nuclear	NP_034722	mmu:16478	TF_bZIP
K02973	RP-S23e, Rl	mmu05171(	PF00164.32	GO:000578	cytoplasmic	TKC41729.	mmu:66475	
K23343	CCDC22		PF05667.16	GO:000810	cytoplasmic	NP_613069	mmu:54638	
K11187	PRDX5	mmu04146(	PF08534.16	GO:000578	cytoplasmic	AAH14682.	mmu:54683	
K14859	SSF1_2		PF04427.23	GO:003068	mitochondr	NP_663585	mmu:235036	
K23643	LSM12		PF21166.2(I	GO:000573	cytoplasmic	NP_001069	mmu:268490	
K14844	PUF6		PF08144.17	GO:000578	nuclear	NP_001390	mmu:52874	
K08058	TAPBP	mmu04612(	Antigen pro	GO:002260	extracellula	NP_001020	mmu:21356	
K27356	PPP1R13L	mmu04115(	PF12796.13	GO:000582	cytoplasmic	NP_001010	mmu:333654	
K10501	ZBTB20, HOF, DPZF		PF00096.32	GO:001922	nuclear	NP_001272	mmu:56490	ZBTB
EBJ(-)			PF13873.12	GO:004323	nuclear	XP_021027	mmu:78100	
K00844	HK	mmu00052(	PF03727.22	GO:000167	cytoplasmic	XP_006513	mmu:15275	
Chromosome 12 open reading frame			PF00096.32	GO:004316	nuclear	NP_001334	mmu:72020	zf-C2H2
06K3(glycosyltransferase 8 domain c			PF01501.26	GO:000579	Golgi appa	BAC30718.	mmu:74782	
K11840	USP9_24		PF25010.1(	GO:010100	nuclear	XP_006503	mmu:329908	
K10369	SVIL		PF00626.27(	Gelsolin:G	nuclear	XP_036016	mmu:225115	
K00248	ACADS, bc	mmu00650(	PF00441.29	GO:003444	mitochondr	NP_031409	mmu:11409	
K09282	HMGA1			GO:000810	nuclear	NP_001399	mmu:111241	
K00252	GCDH, gcd	mmu00310(	PF02771.21	GO:003444	mitochondr	NP_001038	mmu:270076	
K14825	TMED5, ERP2, ERP4		PF01105.29	GO:003802	ER	NP_083152	mmu:73130	
K23966	CCNL		PF00134.28	GO:000802	mitochondr	NP_064321	mmu:56706	
K20190	HPS3		PF14761.12	GO:002260	cytoplasmic	BAC28690.	mmu:12807	
K05404	TLR7	mmu04620(	PF13855.12	GO:001922	ER	NP_001277	mmu:170743	
K19821	SERPINB2,	mmu04610(	PF00079.25	GO:003041	cytoplasmic	NP_001167	mmu:18788	
K10170	TLR8, CD2	mmu04620(	PF13855.12	GO:001922	plasma mer	NP_573475	mmu:170744	
K11368	ENY2, DC6, SUS1		PF10163.14	GO:009008	cytoplasmic	NP_001180	mmu:223527	
K00850	pfkA, PFK	mmu00052(	PF00365.25	GO:009008	cytoplasmic	NP_032852	mmu:18641	
K12461	FRS2	mmu04714(	PF02174.23	GO:000591	cytoplasmic	NP_001395	mmu:327826	
XQDW(Family with sequence simila			PF07159.17	GO:000270	cytoplasmic	NP_001119	mmu:223601	
K10770	ALKBH8, TRM9		PF13532.12	GO:000209	cytoplasmic	NP_001391	mmu:67667	
K17552	PPP1R10		PF08711.16	GO:008009	nuclear	NP_001157	mmu:52040	
K14650	TAF3	mmu03022(	PF00628.35	GO:008009	nuclear	BAE32543.	mmu:209361	
K09602	OTUB1		PF10275.15	GO:008009	cytoplasmic	XP_038181	mmu:107260	
K12670	DDOST, W	mmu04141(	PF03345.20	GO:199023	ER	NP_031864	mmu:13200	
K20160	DENND1			GO:003013	cytoplasmic	EDL38233.	mmu:70785	
K00128	ALDH	mmu01240(	PF00171.27	GO:000403	cytoplasmic	NP_082546	mmu:72535	
K03902	F5	mmu04610(	PF00754.31	GO:003802	extracellula	NP_032002	mmu:14067	
K19951	TBC1D8_9		PF02893.26	GO:000548	plasma mer	NP_001277	mmu:76795	
K08497	SEC20	mmu04130(	PF03908.18	GO:000574	cytoplasmic	XP_021041	mmu:224630	

K24413	CRIP2_3, CRP2_3		PF00412.28	GO:004212	cytoplasmic	NP_001334	mmu:68337	
K10445	KLHL7		PF07707.21	GO:199023	cytoplasmic	EDL03155.	mmu:52323	ZBTB
K16742	ARL2BP, BART		PF11527.13	GO:000563	cytoplasmic	NP_077153	mmu:107566	
K10348	LRR1, PPIL5		PF12799.13	GO:004322	nuclear	NP_001074	mmu:69706	
K24202	SNCG		PF01387.22	GO:000810	cytoplasmic	NP_035560	mmu:20618	
K08745	SLC27A1_4	mmu04931(	PF00501.34	GO:190148	ER	NP_036119	mmu:26569	
K01353	GZMB	mmu04940(	PF00089.32	GO:000425	extracellul	NP_038570	mmu:14939	
K00857	tdk, TK	mmu00983(	PF00265.24	GO:005128	mitochondr	NP_033413	mmu:21877	
26GB(transmembrane and coiled-coil domains (				GO:003450	cytoplasmic	NP_082312	mmu:71983	
K24469	SVEP1		PF00084.26	GO:004590	plasma mer	NP_073725	mmu:64817	
K16449	RGS		PF16613.10	GO:000548	nuclear	XP_006504	mmu:71729	
K06752	MHC2	mmu05323(	PF07654.21	GO:002260	lysosomal	P35737.1(R	mmu:14999	
K11438	PRMT7		PF22528.1(I	GO:000647	nuclear	NP_663379	mmu:214572	
K24495	MYOM	mmu04820(	PF00041.27	GO:000699	cytoplasmic	NP_001078	mmu:242702	
K16518	AKAP1		PF00567.30	GO:003315	plasma mer	NP_001036	mmu:11640	
K18269	PDCD10, C	mmu04520(	PF06840.17	GO:003288	cytoplasmic	NP_001068	mmu:56426	
K08867	WNK, PRK	mmu04928(	PF00069.31	GO:005104	cytoplasmic	NP_941992	mmu:232341	
K11669	INO80E	mmu03082(	PF24237.1(I	GO:008009	nuclear	XP_005064	mmu:233875	
ZNK1(SWAP switching B-cell comp			PF11977.14	GO:008009	cytoplasmic	NP_085040	mmu:80750	
K08543	NR2C1, TR2		PF00104.35	GO:008009	nuclear	NP_035759	mmu:22025	RXR-like
K24524	CPNE1_2_3		PF07002.22	GO:001602	cytoplasmic	BAD90244.	mmu:70568	
K23885	CISD1		PF09360.15	GO:000989	mitochondr	NP_598768	mmu:52637	
K05692	ACTB_G1	mmu04530(	PF00022.25	GO:000520	cytoplasmic	AAF08293.	mmu:11465	
K21989	TMEM63, CSC1		PF02714.20	GO:000526	plasma mer	NP_001404	mmu:208795	
K17310	SAA		PF00277.23	GO:003435	extracellul	NP_001344	mmu:20208	
K24595	RSRC2		PF15477.11(SMAP:Sma		nuclear	NP_001346	mmu:208606	
K07299	SLC2A1, G	mmu05230(	PF00083.30	GO:000697	plasma mer	NP_035530	mmu:20525	
XQJ7(Tetratricopeptide repeat dom			PF00515.34	GO:009715	ER	NP_001390	mmu:67120	
K02728	PSMA4	mmu05020(	PF00227.31	GO:003577	cytoplasmic	BAE38897.	mmu:26441	
Z0F1(family with sequence similarity 211, meml				GO:000573	mitochondr	NP_942561	mmu:192976	
K13217	PRPF39, PRP39		PF23240.1(I	GO:000024	cytoplasmic	NP_808474	mmu:328110	
K23727	CERS5_6, I	mmu04071(	PF03798.21	GO:003014	extracellul	NP_001334	mmu:24144	Homeobox
K00852	rbsK, RBKS	mmu00030(	PF00294.29	GO:001932	cytoplasmic	NP_694876	mmu:71336	
K00771	XYLT	mmu00534(	PF12529.13	GO:003020	ER	BAE31911.	mmu:217119	
K00868	pdxK, pdxY	mmu01240(	PF08543.17	GO:003017	cytoplasmic	NP_742146	mmu:216134	
CN2(Zinc finger protein 428)				GO:004316	cytoplasmic	NP_001277	mmu:232969	
K05288	PIGO	mmu00563(	PF01663.27	GO:000665	extracellul	NP_001292	mmu:56703	
25KF(Ras association (RalGDS AF-			PF05049.19	GO:003563	cytoplasmic	SDA08595.	mmu:667214	
K09022	ridA, tdcF, RIDA		PF01042.26	GO:000454	mitochondr	NP_032313	mmu:15473	
K09511	DNAJB5		PF01556.23	GO:000582	nuclear	NP_001342	mmu:56323	
K23406	BAP18	mmu03082(	ATP-depen	GO:003101	nuclear	NP_001171	mmu:104457	
K04390	TNFRSF6,	mmu05022(	PF00531.28	GO:001003	extracellul	NP_032013	mmu:14102	
K08582	CAPN15		PF00648.27	GO:000417	cytoplasmic	NP_001334	mmu:50817	TF_others

K03138	TFIIF1, GT	mmu03022(	PF05793.18	GO:008009	nuclear	NP_598562	mmu:98053
K26166	NUSAP1		PF16006.10	GO:006164	nuclear	NP_598612	mmu:108907
K27865	BEND3		PF10523.15	GO:000018	nuclear	NP_001346	mmu:331623
K10949	KDELR		PF00810.23	GO:000810	ER	NP_001013	mmu:66913
K06553	VPREB, CD179a		PF07686.23	GO:000237	extracellula	AAA38889.	mmu:22362
K20186	BLOC1S3		PF15753.10	GO:009951	cytoplasmic	NP_001408	mmu:232946
K08565	NAPSA	mmu04142(	PF00026.29	GO:000417	lysosomal	NP_032463	mmu:16541
K04009	C3AR1	mmu04610(	PF00001.26	GO:008009	plasma mer	NP_033909	mmu:12267
K15376	GPHN	mmu01240(	PF00994.29	GO:000677	cytoplasmic	NP_666077	mmu:268566
K03260	EIF4G	mmu05416(	PF02854.25	GO:003451	cytoplasmic	NP_038535	mmu:13690
K11420	EHMT	mmu01100(	PF00023.36	GO:000647	nuclear	NP_001273	mmu:110147
K09523	DNAJC3	mmu04141(	PF00515.34	GO:001922	ER	NP_032955	mmu:100037258
K19608	EVC2	mmu04340(	PF12297.13	GO:009859	ER	NP_666032	mmu:68525
K07368	BCL10	mmu04064(	PF00619.27	GO:200123	cytoplasmic	NP_033870	mmu:12042
YR7(chromosome 8 open reading fr			PF17824.6(DUF5586:Fa		nuclear	NP_001153	mmu:320492
K23484	DNM2	mmu04666(	PF01031.25	GO:004848	cytoplasmic	NP_001344	mmu:13430
K03256	TRM6, GCD10		PF04189.18	GO:199023	nuclear	NP_780322	mmu:66926
K06700	PSMF1	mmu03050(	PF11566.13	GO:000989	cytoplasmic	XP_006499	mmu:228769
K26502	TMEM258	mmu04141(	PF05251.17	GO:004423	plasma mer	AQS27610.	mmu:69038
K01974	RTCA, rtcA		PF01137.26	GO:000552	mitochondr	AQS27614.	mmu:66368
K18808	TLR13		PF13855.12	GO:004858	plasma mer	NP_991389	mmu:279572
K19306	BUD23		PF12589.13	GO:003626	nuclear	XP_030110	mmu:66138
K03681	RRP40, EX	mmu03018(	PF21262.3(I	GO:008009	nuclear	AQS27616.	mmu:66362
K22290	LRPAP1	mmu04979(	PF06401.16	GO:000580	ER	AQS27618.	mmu:16976
YFQ8(IKBKB interacting protein)				GO:004323	ER	NP_080442	mmu:67454
YSN6(phosphatase domain containi			PF14566.11	GO:000573	cytoplasmic	NP_038781	mmu:27355
omponent of the Mediator complex			PF07690.21	GO:000521	extracellula	NP_081398	mmu:69572
K23354	LIMS1_2, PINCH1_2		PF00412.28	GO:000810	cytoplasmic	NP_080424	mmu:110829
K21914	KCTD2_5_17		PF02214.28	GO:002260	cytoplasmic	NP_081284	mmu:69259
K06067	HDAC1_2	mmu05165(	PF00850.24	GO:004423	cytoplasmic	NP_032255	mmu:15182
K11662	ACTR6, AR	mmu03082(	PF00022.25	GO:000989	nuclear	NP_080190	mmu:67019
K11996	MOCS3, UI	mmu04122(	PF00899.27	GO:000677	cytoplasmic	NP_001153	mmu:69372
K11109	POT1		PF21375.2(I	GO:003304	cytoplasmic	NP_598692	mmu:101185
				GO:004212	cytoplasmic	NP_001393	mmu:195555
K10395	KIF4	mmu04814(	PF00225.29	GO:002260	cytoplasmic	NP_032472	mmu:16571
K20465	OSBPL9_10_11, ORP9_		PF01237.23	GO:003015	cytoplasmic	NP_789810	mmu:106326
K04705	STAM	mmu04144(	PF00790.25	GO:000810	cytoplasmic	NP_062641	mmu:56324
K17277	EPS8		PF08416.19	GO:190187	nuclear	NP_001258	mmu:13860
K06830	LGALS1		PF00337.29	GO:003411	cytoplasmic	NP_032521	mmu:16852
K11428	SETD8	mmu00310(	PF00856.33	GO:000647	nuclear	NP_001297	mmu:67956
K24651	PHF21A		PF00628.35	GO:000763	cytoplasmic	NP_001103	mmu:192285
K11713	PGTB1		PF00432.27	GO:000548	cytoplasmic	XP_011826	mmu:225467
K22137	MCUR1		PF07798.16	GO:001986	mitochondr	NP_001074	mmu:76137

K00995	PGS1, pgsA	mmu00564(	PF13091.12	GO:000665.	mitochondr	NP_598518	mmu:74451	
K15413	SP100	mmu05203(	PF03172.18	GO:000573'	nuclear	NP_038701	mmu:20684	SAND
K22942	YIPF4		PF04893.22	GO:000578.	cytoplasmic	NP_001009	mmu:67864	
K04533	HD	mmu05016(	PF20927.3(I	GO:003577(	cytoplasmic	NP_034544	mmu:15194	
K20128	MTSS1		PF08397.16	GO:000591	cytoplasmic	NP_659049	mmu:211401	
K15687	MKRN		PF15815.10	GO:000367	cytoplasmic	BAD83579.	mmu:54484	
K00414	QCR1, UQ(	mmu05208(	PF00675.25	GO:002260'	mitochondr	BAE39918.1	mmu:22273	
K02936	RP-L7Ae, R	mmu05171(	PF01248.31	GO:002262(	cytoplasmic	NP_038749	mmu:27176	
K05025	CLIC5		PF22441.2((	GO:003470'	cytoplasmic	XP_006524	mmu:224796	
umor protein)			PF13836.11	GO:008009(	nuclear	XP_017168	mmu:235469	
K15100	SLC25A1, CTP		PF00153.32	GO:000684.	mitochondr	NP_694790	mmu:13358	
K03426	NUDT12_1	mmu00760(	PF00293.34	GO:000548	mitochondr	NP_080617	mmu:67725	
K10595	HERC2	mmu04120(	PF00415.24	GO:004858.	cytoplasmic	NP_001347	mmu:15204	
)16X(phosphatidylinositol binding)				GO:003509	cytoplasmic	NP_080107	mmu:66895	
K14831	MAK16		PF01778.22	GO:003068'	nuclear	NP_080729	mmu:67920	
YNEB(family with sequence similar			PF15258.11	(FAM222A:I	cytoplasmic	NP_001333	mmu:216971	
K18405	TDRD1_4_6_7		PF00567.30	GO:000548	mitochondr	NP_001277	mmu:100121	
K25768	RALGAPA	mmu04014(	PF02145.21	GO:006017	cytoplasmic	NP_001028	mmu:241694	
XUUR(chromosome 5 open reading			PF17818.6(I	GO:000557.	Golgi appa	NP_694757	mmu:213673	
K02914	RP-L34, MF	mmu03010(	PF00468.22	GO:001593	mitochondr	NP_444392	mmu:94065	
K00858	ppnK, NAI	mmu01240(	PF20143.4(	GO:003577	cytoplasmic	NP_001153	mmu:192185	
K02970	RP-S21, MR	mmu03010(	PF01165.25	GO:001593.	mitochondr	NP_001342	mmu:66292	
K02954	RP-S14, MR	mmu03010(	PF00253.27	GO:001593.	cytoplasmic	NP_001334	mmu:64659	
K00432	gpx, btuE, I	mmu05022(	PF00255.25	GO:005089	ER	NP_081403	mmu:69590	
K23500	SFXN1_3		PF03820.22	GO:001517.	cytoplasmic	NP_081600	mmu:14057	
K21124	TRAF3IP2,	mmu04657(	PF08357.16	GO:005116'	cytoplasmic	BAF34919.1	mmu:103213	
K21891	TMCO1		PF01956.21	GO:000548	nuclear	BAE43184.1	mmu:68944	
K12317	GANC	mmu00052(	PF01055.32	GO:001678'	cytoplasmic	NP_766260	mmu:76051	
K12832	SF3B5, SF3I	mmu03040(	PF07189.16	GO:000613'	nuclear	BAC25292.1	mmu:66125	
ADP-ribosylation factor-like 14 effe			PF13465.12	GO:000097'	cytoplasmic	XP_052054	mmu:24421	zf-C2H2
K04545	GNG10	mmu04724(	PF00631.27	GO:000557.	cytoplasmic	NP_079553	mmu:14700	
K14327	UPF2, REN	mmu03013(	PF02854.25	GO:003514.	cytoplasmic	NP_001074	mmu:326622	
K23568	EMC7		PF09430.16	GO:000810	Golgi appa	NP_598510	mmu:73024	
K04523	UBQLN, D	mmu04141(	PF23195.1(I	GO:008009(	cytoplasmic	NP_277068	mmu:94232	
K20646	ARHGAP31, CDGAP		PF00620.32	GO:000548	cytoplasmic	NP_064656	mmu:12549	
K12493	ARFGAP2_	mmu04144(	PF01412.23	GO:000810	cytoplasmic	NP_076343	mmu:77038	
K01376	UFSP2		PF20908.2(I	GO:000578.	cytoplasmic	NP_619609	mmu:192169	
K16457	CEP76		PF15627.12	GO:003304.	cytoplasmic	NP_001074	mmu:225659	
K11096	SNRPD2, S	mmu03040(	PF01423.28	GO:002260'	nuclear	NP_001029	mmu:107686	
K26521	PAG1		PF15347.11	GO:000268.	Golgi appa	NP_001181	mmu:94212	
K22804	SMC6		PF13476.12	GO:003506	cytoplasmic	NP_079971	mmu:67241	
K12898	HNRNPF_H		PF00076.28	GO:008009(	cytoplasmic	NP_001300	mmu:56258	
K11087	SNRPD1, S	mmu05322(	PF01423.28	GO:000024.	nuclear	KAJ666199	mmu:20641	

K05955	FNTA	mmu00900(	PF01239.28	GO:000810	cytoplasmic	AAH12711.	mmu:14272
K26273	SBNO, SNO, FGT1		PF13872.12	GO:008009	nuclear	NP_906271	mmu:216161
K03127	TAF13	mmu03022(	PF02269.22	GO:003277	cytoplasmic	NP_001069	mmu:99730
K13181	DDX27, DRS1		PF00270.35	GO:000367	nuclear	NP_694705	mmu:228889
K14792	RRP5, PDCD11		PF23459.1(	GO:003204	nuclear	NP_035183	mmu:18572
K07942	ARL1		PF00025.27	GO:000810	cytoplasmic	NP_071780	mmu:104303
K03242	EIF2S3		PF09173.17	GO:000374	cytoplasmic	NP_036141	mmu:26908
XPQX(midbrain-hindbrain bounda			PF04503.18	GO:004212	cytoplasmic	NP_060540	mmu:72475
K10587	UBE3A, E6	mmu05165(	PF00632.30	GO:005160	cytoplasmic	BAC38028.	mmu:22215
N4G(metallo-beta-lactamase dom			PF00753.33	GO:190280	cytoplasmic	NP_808546	mmu:330216
K20177	VPS3, TGF	mmu04148(	PF10366.15	GO:000512	cytoplasmic	XP_011236	mmu:73122
phosphatidylinositol transfer protei			PF02121.25	GO:000573	cytoplasmic	BAC25057.	mmu:71795
23ER(Chromosome 1 open reading f			PF10159.14(	MMtag:Mu	nuclear	NP_077172	mmu:67862
K05687	PARK7	mmu05022(	PF01965.30	GO:000810	cytoplasmic	NP_065594	mmu:57320
K12621	LSM2	mmu03018(	PF01423.28	GO:000548	cytoplasmic	NP_001094	mmu:27756
K18159	NDUFAF1	mmu04714(	PF08547.18	GO:002260	mitochondr	BAC34201.	mmu:69702
K10391	TUBE	mmu04814(	PF00091.31	GO:000520	cytoplasmic	EDL04916.	mmu:71924
Necessary for normal cell division a			PF01926.29	GO:003563	mitochondr	NP_079608	mmu:66067
K10306	FBXO28			GO:004322	cytoplasmic	BAE36778.	mmu:67948
F8S(synaptogyrin)			PF01284.28	GO:000802	cytoplasmic	NP_997591	mmu:20972
RNW(Interferon-induced protein 3			PF07292.18	GO:004858	cytoplasmic	NP_081596	mmu:70110
K15131	MED11		PF10280.14	GO:008009	nuclear	NP_079673	mmu:66172
K17608	STRN1_3_4		PF00400.38	GO:003250	cytoplasmic	NP_035630	mmu:268980
K10258	TER, TSC1	mmu01040(	PF21696.3(	GO:000663	cytoplasmic	NP_598879	mmu:106529
K07205	EIF4EBP1	mmu04012(	PF05456.16	GO:008009	cytoplasmic	NP_031944	mmu:13685
K02890	RP-L22, MI	mmu03010(	PF00237.24	GO:001593	mitochondr	NP_001390	mmu:216767
K03783	punA, PNP	mmu01100(	PF01048.26	GO:004643	cytoplasmic	NP_001116	mmu:667034
K07901	RAB8A, MI	mmu04530(	PF00071.28	GO:003288	cytoplasmic	NP_075615	mmu:17274
K03249	EIF3F		PF01398.26	GO:002260	cytoplasmic	BAE30896.	mmu:66085
K15485	BCL2L13, E	mmu04137(	PF00452.24	GO:001602	cytoplasmic	NP_705736	mmu:94044
K23281	VPS33B		PF00995.28	GO:000810	cytoplasmic	NP_835171	mmu:233405
K12481	RBSN, ZFY	mmu04144(	PF16601.10	GO:000810	cytoplasmic	NP_084357	mmu:78287
K11255	NCOA2, TI	mmu04915(	PF23172.1(	GO:200032	cytoplasmic	BAF69036.1	mmu:17978
K00799	GST, gst	mmu00980(	PF02798.26	GO:000573	cytoplasmic	NP_598755	mmu:103140
K17890	ATG16L1	mmu04140(	PF08614.16	GO:000810	cytoplasmic	NP_084122	mmu:77040
)FD4(BSD domain containing 1)			PF03909.22	GO:000573	cytoplasmic	NP_598650	mmu:100383
K04440	JNK	mmu05208(	PF00069.31	GO:003563	cytoplasmic	NP_001297	mmu:26419
PMF(ATP5S-like)				GO:002260	mitochondr	NP_079780	mmu:66349
K22828	MICU2		PF13499.12	GO:000548	mitochondr	NP_082919	mmu:68514
K12402	AP4M1	mmu04142(	PF00928.26	GO:000810	cytoplasmic	BAC27490.	mmu:11781
K21760	RIOX2, MINA		PF08007.18	GO:000371	cytoplasmic	NP_080186	mmu:67014
K14808	DDX54, DBP10		PF00270.35	GO:001688	nuclear	BAC27509.	mmu:71990
K05482	IL18, IL1F4	mmu05164(	PF00340.25	GO:001922	cytoplasmic	AAY16325.	mmu:16173

K14020	WFS1	mmu04141(	PF20053.4(	GO:200123	cytoplasmic	NP_035846	mmu:22393	
KQIA(glutathione binding)			PF05147.19	GO:004423	cytoplasmic	NP_775590	mmu:236285	
)2P5(nuclear fragile X mental retarc			PF15293.11	GO:003577	cytoplasmic	BAC65776.	mmu:68564	
ZTYR(-)			PF02093.21	GO:004671	cytoplasmic	QPF16722.	mmu:118567641	
K06238	COL6A	mmu05165(	PF00092.34	GO:004487	extracellulo	NP_081039	mmu:68553	
K01493	comEB	mmu01100(	PF00383.29	GO:000548	cytoplasmic	XP_006509	mmu:320685	
K07605	KRT2		PF00038.26	GO:002260	mitochondr	NP_081287	mmu:110308	
)057(chromosome 2 open reading fr			PF10561.15	GO:001598	mitochondr	NP_082822	mmu:73467	
K14788	NOL10, ENP2		PF23098.1(	GO:003204	nuclear	NP_001008	mmu:217431	
K17290	HTATIP2		PF13460.12	GO:005116	ER	NP_058561	mmu:53415	
ZMYH(Family with sequence simila			PF21033.3(	GO:009951	mitochondr	NP_079752	mmu:66302	
K24361	PCOLCE		PF00431.26	GO:003101	lysosomal	NP_032814	mmu:18542	
K03781	katE, CAT,	mmu05208(	PF00199.24	GO:000578	cytoplasmic	NP_033934	mmu:12359	
K16195	EIF2AK2	mmu05165(	PF00069.31	GO:200073	cytoplasmic	NP_035293	mmu:19106	
K17619	MDP1		PF12689.13	GO:000548	cytoplasmic	NP_075886	mmu:67881	
K10415	DYNC1I, D	mmu04962(	PF24807.1(	GO:009951	nuclear	NP_001185	mmu:13427	
K23551	FSCN1	mmu05206(	PF06268.19	GO:003243	cytoplasmic	NP_032010	mmu:14086	
GN5(family with sequence similari			PF06916.18	GO:000573	mitochondr	NP_722489	mmu:108654	
K14948	PTBP2, NPTB		PF13893.12(RRM_5:RN		cytoplasmic	XP_009697	mmu:56195	
K11247	SH3GL	mmu04144(	PF03114.23	GO:200036	cytoplasmic	NP_059096	mmu:20408	
K11346	ING4		PF12998.12	GO:190216	nuclear	NP_001355	mmu:28019	
K04564	SOD2	mmu05208(	PF02777.23	GO:200123	mitochondr	NP_038699	mmu:20656	
K11352	NDUFA12	mmu05208(	PF05071.21	GO:002260	mitochondr	XP_021030	mmu:66414	
K08798	MARK		PF00069.31	GO:009951	cytoplasmic	NP_001073	mmu:13728	
KQRV(phospholipase B domain cor			PF04916.18	GO:000032	extracellulo	NP_080082	mmu:66857	
K21357	ULK1	mmu04137(	PF00069.31	GO:004867	cytoplasmic	NP_033495	mmu:22241	
K21749	CMAS	mmu00520(	PF02348.25	GO:001975	nuclear	NP_034038	mmu:12764	
KU74(Chromosome 16 open reading			PF04884.20	GO:000557	cytoplasmic	NP_663565	mmu:233913	
K05681	ABCG2, CI	mmu02010(	PF01061.29	GO:190461	cytoplasmic	NP_001342	mmu:26357	
K17966	TMEM70		PF06979.18	GO:003361	mitochondr	NP_080668	mmu:70397	
K17924	SNX10_11		PF00787.29	GO:000810	cytoplasmic	NP_001120	mmu:71982	
K10291	FBXO4	mmu04120(	PF12937.12	GO:003288	cytoplasmic	NP_598860	mmu:106052	
K20056	GIPC, SEMCAP		PF25083.1(GIPC1_GH		cytoplasmic	NP_683753	mmu:209047	
K06671	STAG1_2, S	mmu04110(	PF24571.1(	GO:000367	cytoplasmic	NP_001277	mmu:20843	
K04352	RASA1, RA	mmu04010(	PF00017.30	GO:001922	cytoplasmic	NP_663427	mmu:218397	
K19202	SAP30	mmu05169(	PF13866.11	GO:008009	nuclear	NP_001386	mmu:60406	
K20285	RABEPK		PF24681.1(	GO:003109	cytoplasmic	NP_001361	mmu:227746	
K16939	SEPT6_8_1	mmu05100(	PF00735.24	GO:000548	cytoplasmic	XP_054995	mmu:52398	
K21433	ANKRD1	mmu04820(	PF00023.36	GO:009951	nuclear	NP_038496	mmu:107765	
K23718	SON		PF01585.28	GO:008009	nuclear	XP_021040	mmu:20658	TF_others
K07509	HADHB	mmu00071(	PF00108.29	GO:003444	mitochondr	EDL37268.	mmu:231086	
K19360	NPHP3		PF24885.1(	GO:004858	cytoplasmic	BAD32605.	mmu:74025	
K23589	PZP		PF07678.20	GO:003041	extracellulo	NP_031402	mmu:11287	

K19525	VPS13A_C		PF12624.14	GO:000810	cytoplasmic	NP_796158	mmu:320528	
K06176	truD, PUS7		PF01142.23	GO:000989	nuclear	BAE26400.1	mmu:78697	
XQXC(SUMO-interacting motifs containing 1)				GO:000573	cytoplasmic	NP_795961	mmu:319719	
K01555	FAH, fahA	mmu00350(	PF09298.16	GO:004253	cytoplasmic	NP_034306	mmu:14085	
K12899	SRSF11, SFRS11		PF00076.28(RRM_1:RN		nuclear	XP_021013	mmu:69207	
K07178	RIOK1	mmu03008(	PF01163.29	GO:003068	nuclear	NP_077204	mmu:71340	
K06554	IGLL1, IGI	mmu05340(	PF07654.21	GO:000237	extracellula	BAQ55490.	mmu:16136	
K24741	WDR20		PF23414.1(I	GO:000573	cytoplasmic	BAE26406.1	mmu:70948	
K16667	PTPRG		PF00102.32	GO:001602	plasma mer	NP_033007	mmu:19270	
K13516	MBOAT7	mmu00564(	PF03062.24	GO:004423	ER	NP_084210	mmu:77582	
K24272	DENR, TMA22		PF21023.2(I	GO:002260	cytoplasmic	NP_001404	mmu:68184	
K10695	RNF1_2	mmu03083(	PF16207.11	GO:008009	cytoplasmic	NP_001347	mmu:19821	
K25426	PIH1D1		PF08190.17	GO:008009	nuclear	NP_001265	mmu:68845	
K10373	TPM1	mmu04260(	PF00261.25(Tropomyos		nuclear	OBS72299.1	mmu:22003	
K09229	ZKSCAN		PF13465.12	GO:000989	nuclear	NP_001139	mmu:72739	zf-C2H2
cardiotrophin 1)			PF13465.12	GO:200123	nuclear	NP_001359	mmu:17188	zf-C2H2
K08886	TNK2, ACK1		PF07714.23	GO:004858	cytoplasmic	NP_001103	mmu:51789	
K06277	RAPGEF1,	mmu04015(	PF00617.24	GO:190545	cytoplasmic	XP_006497	mmu:107746	
K00905	BCKDK		PF10436.14	GO:004732	mitochondr	XP_052053	mmu:12041	
K20182	VPS33A	mmu04140(	PF00995.28	GO:000810	cytoplasmic	NP_084205	mmu:77573	
K10376	DMN		PF00038.26	GO:001716	cytoplasmic	NP_964001	mmu:233335	
IAF(prolactin)			PF01284.28	GO:001995	plasma mer	NP_062729	mmu:18824	
K16343	PLA2G6, II	mmu04750(	PF12796.13	GO:003577	mitochondr	EDL04657.	mmu:53357	
K15164	MED13	mmu04919(	PF06333.17	GO:006026	cytoplasmic	NP_001074	mmu:327987	
K01620	ltaE	mmu01100(	PF01212.27	GO:000873	mitochondr	NP_082195	mmu:71776	
K07390	grxD, GLRX5		PF00462.30	GO:002260	mitochondr	XP_021057	mmu:73046	
K01115	PLD1_2	mmu04724(	PF00614.28	GO:004643	cytoplasmic	NP_032902	mmu:18806	
XQ3V(transmembrane protein 184B			PF03619.22	GO:000557	plasma mer	NP_001240	mmu:223693	
K00710	GALNT	mmu00514(	PF00535.33	GO:003024	ER	NP_001155	mmu:207839	
K26473	N4BP3		PF06818.20	GO:003042	cytoplasmic	NP_001405	mmu:212706	
K10651	TRIM21, SS	mmu05322(	PF00622.36	GO:009008	cytoplasmic	NP_001076	mmu:20821	
K27241	ZNF219		PF00096.32	GO:005124	nuclear	AAH52050.	mmu:69890	zf-C2H2
K06522	IGSF2_3, CD101		PF07686.23	GO:000557	plasma mer	NP_997088	mmu:78908	
K12043	TNNI2	mmu04814(	PF00992.25	GO:008009	nuclear	NP_033431	mmu:21953	
K08847	RIPK3	mmu04621(	PF00069.31	GO:200123	cytoplasmic	NP_064339	mmu:56532	
K05171	IL1RL1, ST	mmu04060(	PF01582.25	GO:001922	plasma mer	NP_001020	mmu:17082	
K02731	PSMA7	mmu05020(	PF00227.31	GO:001977	cytoplasmic	NP_036099	mmu:26444	
K08871	BRD2		PF00439.30	GO:000810	nuclear	NP_001191	mmu:14312	
K20726	TMEM222		PF05608.18	GO:000578	ER	NP_079943	mmu:52174	
K10900	WRN, RECQL2		PF00270.35	GO:001033	cytoplasmic	XP_006509	mmu:22427	
K11655	BAZ1A, AC	mmu03082(	PF10537.14	GO:190227	nuclear	NP_038843	mmu:217578	
K24331	PEAR1, MEGF12		PF23301.1(I	GO:003802	cytoplasmic	AAL66380.1	mmu:73182	
K15707	RNF170		PF13445.12	GO:001602	plasma mer	BAE31474.1	mmu:77733	

K14815	MRT4		PF17777.6(I	GO:000989	cytoplasmic	NP_001277	mmu:69902		
K04413	MINK		PF00069.31	GO:003255	nuclear	EDL12580.	mmu:50932		
K12478	EEA1	mmu04144(	PF01363.27	GO:000548	cytoplasmic	NP_001001	mmu:216238		
K05290	PIGK	mmu00563(	PF01650.23	GO:000417	lysosomal	NP_821135	mmu:329777		
K01866	YARS, tyrS	mmu00970(	PF00579.31	GO:001710	nuclear	EDL30209.	mmu:107271		
K06460	CD9, TSPA	mmu03272(	PF00335.26	GO:003411	plasma mer	NP_031683	mmu:12527		
K10847	XPA	mmu03420(	PF05181.18	GO:000810	cytoplasmic	AAH29241.	mmu:22590	TF_others	
K00910	ADRBK, G	mmu04724(	PF00069.31	GO:000181	cytoplasmic	NP_001277	mmu:110355		
K10845	TTDA, GT	mmu03420(	PF06331.17	GO:000629	cytoplasmic	NP_001344	mmu:66467		
K03514	PAPD5_7,	mmu03018(	PF22600.2(	MTPAP-like	cytoplasmic	NP_941002	mmu:210106		
YI5V(kiaa1522)			PF15273.11	GO:003250	cytoplasmic	NP_001272	mmu:97130		
K13403	MTHFD2	mmu01240(	PF02882.25	GO:004427	mitochondr	NP_081064	mmu:665563		
K20131	RABGEF1	mmu04137(	PF02204.23	GO:000810	nuclear	NP_001185	mmu:56715		
K01081	E3.1.3.5	mmu01100(	PF06941.18	GO:004643	cytoplasmic	NP_056622	mmu:50773		
K23407	GATAD1			GO:008009	peroxisoma	BAB69047.1	mmu:67210	zf-GATA	
K04441	P38	mmu05208(	PF00069.31	GO:003288	cytoplasmic	NP_001161	mmu:26416		
K05690	CTNND1	mmu04670(	PF00514.29	GO:001922	nuclear	XP_006498	mmu:12388		
K02321	POLA2	mmu03030(	PF04042.21	GO:003450	nuclear	NP_032919	mmu:18969		
K02685	PRI2	mmu03030(	PF04104.19	GO:000988	nuclear	NP_032948	mmu:19076		
K03104	SRP14	mmu03060(	PF02290.20	GO:000578	nuclear	NP_001099	mmu:20813		
K17289	CRABP2		PF00061.29	GO:003510	cytoplasmic	NP_031785	mmu:12904		
K15450	TYW3		PF02676.19	GO:000367	cytoplasmic	NP_766062	mmu:209584		
K02995	RP-S8e, RP	mmu05171(	PF01201.27	GO:003204	nuclear	NP_001003	mmu:100040298		
K02957	RP-S15Ae,	mmu05171(	PF00410.24	GO:000548	cytoplasmic	NP_001010	mmu:267019		
K25769	RALGAPB	mmu04014(	PF20412.3(I	GO:004858	cytoplasmic	XP_011237	mmu:228850		
K10739	RFA2, RPA	mmu03440(	PF08784.17	GO:000810	nuclear	NP_035414	mmu:19891		
K10592	HUWE1, M	mmu04120(	PF00632.30	GO:003292	nuclear	NP_067498	mmu:59026		
K20191	HPS5		PF23758.1(	GO:002260	cytoplasmic	NP_001161	mmu:246694		
247K(family with sequence similarit			PF14799.11	GO:000573	cytoplasmic	NP_001028	mmu:192173		
K17266	MVP		PF01505.23	GO:001989	cytoplasmic	BAC40115.	mmu:78388		
K20123	PACSIN		PF00611.28	GO:000810	cytoplasmic	NP_001152	mmu:23970		
K01206	FUCA	mmu04142(	PF01120.23	GO:001613	lysosomal	BAE41842.	mmu:71665		
K20174	OSBPL1_2, ORP1_2		PF01237.23	GO:012002	cytoplasmic	AAH26804.	mmu:228983		
K08875	NRBP		PF07714.23	GO:004299	cytoplasmic	BAE41840.	mmu:192292		
Calponin)			PF00402.23	GO:009951	cytoplasmic	NP_082320	mmu:71994		
K16574	MZT2A_B		PF12926.12	GO:000573	mitochondr	NP_001345	mmu:72083		
K20397	LAMTOR1	mmu04150(	PF15454.11	GO:004340	cytoplasmic	NP_079881	mmu:66508		
KQNM(nuclear casein kinase and cyclin-depend				GO:008009	nuclear	NP_001139	mmu:98415		
K07575	MCTS, TMA20		PF17832.7(I	GO:000573	cytoplasmic	NP_001395	mmu:66405		
ZJAF(Chromosome 14 open reading			PF00240.29	GO:000716	cytoplasmic	NP_065611	mmu:80517		
K06927	DPH6		PF01902.24	GO:003563	cytoplasmic	NP_079951	mmu:66632		
K18711	DHX40		PF07717.22	GO:000367	cytoplasmic	NP_080467	mmu:67487		
2243(Chromosome 5 open reading fr			PF06842.17	GO:003109	ER	NP_001121	mmu:67495		

K12332	CALC	mmu04080(	PF00214.24	GO:008009	extracellula	NP_001029	mmu:12310	
ing finger protein 214)			PF00022.25	GO:003124	cytoplasmic	NP_780706	mmu:238880	
K11247	SH3GL	mmu04144(	PF03114.23	GO:003169	cytoplasmic	NP_038692	mmu:20405	
K19483	NAV2		PF23092.1(	GO:001678	mitochondr	NP_780481	mmu:78286	
K08544	NR2C2, TR4		PF00104.35	GO:008009	cytoplasmic	NP_001334	mmu:22026	RXR-like
K23794	PHLDB		PF00169.35	GO:004518	nuclear	XP_030099	mmu:102693	
K18468	VPS35	mmu04144(	PF03635.23	GO:003288	cytoplasmic	NP_075373	mmu:65114	
K01900	LSC2	mmu00020(	PF08442.15	GO:001707	mitochondr	BAE31798.	mmu:20916	
K23341	ERLIN		PF01145.30	GO:008009	ER	NP_001351	mmu:244373	
K01205	NAGLU	mmu04142(	PF05089.18	GO:006024	ER	AAB88084.	mmu:27419	
YE3A(WD repeat domain 7)			PF13402.12	GO:190547	cytoplasmic	NP_666286	mmu:232748	
K12200	PDCD6IP,	mmu03250(	PF03097.24	GO:000810	cytoplasmic	EDL08949.	mmu:18571	
K17085	TM9SF1		PF02990.21	GO:000810	plasma mer	NP_001346	mmu:74140	
K00128	ALDH	mmu01240(	PF00171.27	GO:003323	mitochondr	NP_033786	mmu:11669	
K17461	RECK, ST1	mmu05206(	PF23298.1(	GO:003510	extracellula	AAI32198.1	mmu:53614	
IXK(helicase); ENOG4111R0W(G			PF01585.28	GO:003250	nuclear	NP_001104	mmu:66614	
K24309	TET2_3		PF12851.13	GO:008009	nuclear	NP_001333	mmu:21413	TF_others
K00294	E1.2.1.88	mmu00330(	PF00171.27	GO:000403	mitochondr	NP_780647	mmu:212647	
K04460	PPP5C	mmu04010(	PF00149.33	GO:200032	cytoplasmic	NP_035285	mmu:19060	
K13142	INTS5		PF14838.11	GO:009030	cytoplasmic	NP_789813	mmu:109077	
ibosomal RNA processing 7 homolc			PF00505.25	GO:008009	nuclear	BAE31864.	mmu:26874	HMG
K09653	C1GALT2,	mmu00514(	Other types	GO:000367	Golgi appa	NP_067525	mmu:59048	
K10706	SETX, ALS	mmu05014(	PF13086.12	GO:008009	nuclear	NP_932150	mmu:269254	
K00942	gmk, GUK1	mmu01100(	PF00625.27	GO:000810	cytoplasmic	NP_001152	mmu:14923	
K27693	NDRG2		PF03096.20	GO:004212	cytoplasmic	EDL42274.	mmu:29811	
K01052	LIPA	mmu00100(	PF00561.26	GO:000677	lysosomal	AAH58564.	mmu:16889	
K07622	GJB3, CX31		PF00029.24	GO:005078	cytoplasmic	NP_001153	mmu:14620	
K14411	MSI	mmu03015(	PF00076.28	GO:008009	cytoplasmic	NP_001350	mmu:76626	
K24417	LMCD1		PF06297.19	GO:001474	cytoplasmic	NP_659048	mmu:30937	
K02870	RP-L12e, R	mmu05171(	PF03946.19	GO:000548	cytoplasmic	NP_033102	mmu:269261	
K00898	PDK2_3_4	mmu05415(	PF10436.14	GO:001922	mitochondr	NP_663605	mmu:236900	
K07766	E3.6.1.52		PF00293.34	GO:000548	cytoplasmic	NP_081998	mmu:71207	
K02879	RP-L17, MI	mmu03010(	PF01196.24	GO:001593	mitochondr	NP_079577	mmu:27397	
K12462	ARHGDI, I	mmu04962(	PF02115.23	GO:005124	cytoplasmic	NP_001288	mmu:11857	
K02956	RP-S15, MI	mmu03010(	PF00312.27	GO:001593	mitochondr	NP_079820	mmu:66407	
K12159	ISG15	mmu05165(	PF00240.29	GO:000810	cytoplasmic	NP_056598	mmu:100038882	
K01866	YARS, tyrS	mmu00970(	PF00579.31	GO:000643	mitochondr	NP_937889	mmu:70120	
K23751	TRIOBP		PF00169.35	GO:004438	cytoplasmic	NP_001034	mmu:110253	
K00939	adk, AK	mmu01240(	PF00406.28	GO:001922	mitochondr	NP_001171	mmu:11639	
JQE(Chromosome 8 open reading f			PF14956.11	GO:004323	mitochondr	NP_663447	mmu:223665	
K05038	SLC6A5_9,	mmu04721(	PF00209.24	GO:008009	peroxisoma	P28571.3(R	mmu:14664	
K12233	HECTD3		PF00632.30	GO:000367	mitochondr	NP_780453	mmu:76608	
K06553	VPREB, CD179a		PF07686.23	GO:000237	cytoplasmic	P01675.1(R	mmu:22362	

Heterogeneous nuclear ribonucleopr			PF13855.12	GO:004212	cytoplasmic	Q01730.3(R	mmu:20163	
K14790	NOP9		PF22493.2(I	GO:005116	cytoplasmic	AAH18523.	mmu:67842	
K10158	B3GAT3	mmu00534(	PF03360.21	GO:003315	ER	BAJ05826.1	mmu:72727	
K15522	FN3K		PF03881.20	GO:010219	cytoplasmic	AAH64710.	mmu:63828	
K11584	PPP2R5	mmu05165(	PF01603.26	GO:001920	cytoplasmic	XP_027785	mmu:26932	
K01852	LSS, ERG7	mmu00100(	PF13243.12	GO:001602	peroxisoma	NP_666118	mmu:16987	
K00026	MDH2	mmu01100(	PF02866.23	GO:000548	mitochondr	NP_032643	mmu:17448	
K12651	AZI2	mmu04622(	PF12845.12	GO:004858	cytoplasmic	NP_001273	mmu:27215	
K21843	TTC7		PF19440.5(TTC7_N:Tet		cytoplasmic	NP_001361	mmu:104718	
KRSQ(AAR2 splicing factor homolo			PF00650.25	GO:000557	cytoplasmic	NP_083788	mmu:76080	
K00939	adk, AK	mmu01240(	PF00406.28	GO:009951	cytoplasmic	NP_001185	mmu:11636	
K25410	MFAP5		PF05507.16	GO:004859	extracellula	NP_056591	mmu:50530	
K05662	ABCB7, AT	mmu02010(	PF00664.28	GO:001922	mitochondr	EDL14083.	mmu:11306	
K17601	WDR81		PF02138.23	GO:014003	cytoplasmic	NP_620400	mmu:192652	
MRK(-)			PF15090.11(DUF4553:I		nuclear	NP_001357696.1(ligand-dependent		
K16342	PLA2G4, C	mmu04750(	PF13621.12	GO:000665	cytoplasmic	XP_032759	mmu:211429	
K17084	FERMT3, K	mmu04611(	PF18124.7(I	GO:002260	cytoplasmic	NP_001349	mmu:108101	
K13167	SCAF4, SFRS15		PF04818.19	GO:004423	nuclear	EDK98387.	mmu:224432	
K22411	ZNF516	mmu04714(	PF00096.32	GO:004885	nuclear	NP_001170	mmu:32900	zf-C2H2
K07190	PHKA_B	mmu04922(	PF00723.27(Glyco_hydr		cytoplasmic	NP_001390	mmu:102093	
K14651	TAF15, NP	mmu05202(	PF00076.28	GO:008009	nuclear	KFO34363.	mmu:70439	
K05510	CCL6	mmu04062(	PF00048.25	GO:001922	extracellula	NP_033165	mmu:20305	
K16307	CDC42BP		PF00780.28	GO:003103	nuclear	NP_001346	mmu:226751	
K17254	SDCBP		PF00595.29	GO:001922	cytoplasmic	NP_001091	mmu:53378	
K07213	ATOX1, AT	mmu04978(	PF00403.32	GO:006000	cytoplasmic	NP_033850	mmu:11927	
K19867	PDLIM5_6	mmu04820(	PF00412.28	GO:003243	cytoplasmic	NP_062782	mmu:56376	
K22745	AIFM2	mmu04115(	PF07992.20	GO:000417	cytoplasmic	NP_001034	mmu:71361	
K11251	H2A	mmu05322(	PF01661.27	GO:003472	nuclear	NP_001152	mmu:26914	
K23625	ATXN2_2L	mmu05014(	PF14438.12	GO:000548	cytoplasmic	AAB19202.	mmu:20239	
K04537	GNB2	mmu04724(	PF00400.38	GO:009879	cytoplasmic	NP_001091	mmu:14693	
K25449	OLFML2		PF02191.22	GO:006500	ER	NP_796042	mmu:320078	
K12624	LSM5	mmu03018(	PF01423.28	GO:000613	cytoplasmic	EDL04713.	mmu:66373	
K10247	ELOVL1	mmu01040(	PF01151.23	GO:000663	extracellula	NP_001034	mmu:54325	
K24070	PARP1	mmu04210(	PF00644.27	GO:008009	nuclear	BAE35080.	mmu:11545	
K02908	RP-L30e, R	mmu05171(	PF01248.31	GO:000960	cytoplasmic	NP_000980	mmu:19946	
K27470	PAIP2		PF07145.20	GO:000989	cytoplasmic	NP_666281	mmu:232164	
K24999	RAVER1		PF00076.28	GO:000573	cytoplasmic	NP_082187	mmu:71766	
K19873	FKRP	mmu00515(	PF22921.2(I	GO:000810	ER	BAE40248.	mmu:243853	
K15264	NSUN5, WBSCR20, RC		PF21148.2(I	GO:002203	nuclear	NP_663389	mmu:100609	
K00149	ALDH9A1	mmu00053(	PF00171.27	GO:000403	cytoplasmic	BAE40209.	mmu:56752	
K12757	MYL12	mmu04530(	PF13499.12	GO:003243	cytoplasmic	XP_054984	mmu:67938	
KSPW(Family with sequence similar			PF12736.12	GO:004212	cytoplasmic	NP_001028	mmu:381126	
K03847	ALG12	mmu00510(	PF03901.23	GO:000578	ER	EDL04396.	mmu:223774	

K15865	CDKAL1		PF00919.26	GO:004323	cytoplasmic	NP_653119	mmu:68916	
K07755	AS3MT	mmu05208(	PF13847.12	GO:001975	cytoplasmic	NP_065602	mmu:57344	
K06521	SEMA4, CD	mmu04360(	PF01403.25	GO:004858	Golgi appa	NP_001347	mmu:20352	
K06521	SEMA4, CD	mmu04360(	PF01403.25	GO:190189	lysosomal	XP_006501	mmu:20351	
JSU(cytochrome b5 domain contair			PF00173.33	GO:003015	Golgi appa	NP_001020	mmu:192986	
K09228	KRAB		PF13465.12	GO:000097	cytoplasmic	BAC38392.	mmu:22688	zf-C2H2
K07151	STT3	mmu04141(	PF02516.20	GO:000578	ER	AQS27623.	mmu:16430	
K13211	GCFC		PF07842.17	GO:000828	nuclear	AQS27630.	mmu:67367	
Y14P(family with sequence similarit			PF15260.11(FAM219A:l		cytoplasmic	NP_001153	mmu:71901	
K13784	PTMA		PF03247.19	GO:004239	nuclear	NP_032998	mmu:19231	
peptidase m28)			PF22249.1(I	GO:000548	peroxisoma	EDL41688.	mmu:226090	
K05607	AUH	mmu00280(	PF00378.25	GO:003444	mitochondr	NP_057918	mmu:11992	
K10886	XRCC4	mmu03450(	PF21924.1(	GO:003304	nuclear	NP_082288	mmu:108138	
K11494	RCBTB		PF00415.24	GO:000573	cytoplasmic	NP_001347	mmu:71330	ZBTB
K17724	IRG1	mmu01100(	PF03972.19	GO:008009	cytoplasmic	EDL00492.	mmu:16365	
K06126	COQ6	mmu01240(	PF01494.25	GO:000548	mitochondr	NP_766170	mmu:217707	
K11985	TRAIP, TRIP		PF13639.12	GO:001922	cytoplasmic	NP_035764	mmu:22036	
K14991	SLC38A4, SNAT4		PF01490.24	GO:190427	cytoplasmic	BAE41099.	mmu:69354	
K11583	PPP2R3	mmu05165(	PF17958.7(I	GO:001679	cytoplasmic	NP_001154	mmu:235542	
K02089	CDK4	mmu04530(	PF00069.31	GO:008009	cytoplasmic	NP_034000	mmu:12567	
K24176	SERINC2		PF03348.21	GO:000665	plasma mer	NP_001240	mmu:230779	
K20308	TRAPPC11		PF11817.14	GO:004820	cytoplasmic	NP_796214	mmu:320714	
K01613	psd, PISD	mmu00564(	PF02666.20	GO:001602	mitochondr	NP_001334	mmu:320951	
K03018	RPC1, POL	mmu04623(	PF04997.17	GO:001922	cytoplasmic	NP_001074	mmu:218832	
K24066	SYNPO2	mmu04820(	PF05556.16	GO:003243	nuclear	NP_001376	mmu:118449	
K13510	LPCAT1_2	mmu00564(	PF13499.12	GO:000548	Golgi appa	AAI44807.1	mmu:270084	
K16779	RAB3IP, RABIN8		PF06428.16(Sec2p:GDP		cytoplasmic	NP_001365	mmu:74760	
K03424	tatD		PF01026.27(TatD_DNc		cytoplasmic	NP_001355	mmu:69694	
K23425	SERPINB1		PF00079.25	GO:000695	cytoplasmic	NP_079705	mmu:66222	
K17703	SIPA1L3, SI	mmu04015(	PF11881.13	GO:003243	cytoplasmic	NP_001074	mmu:74206	
M9H(tumor protein)			PF04201.20	GO:000573	cytoplasmic	NP_001393	mmu:66314	
K10392	KIF1	mmu04814(	PF00225.29(Kinesin:Ki		nuclear	NP_001395	mmu:16560	
K18260	CC2D1		PF21528.2((	GO:008009	cytoplasmic	NP_666082	mmu:21213	TF_others
K08018	RAPGEF2,	mmu04530(	PF00617.24(RasGEF:Rc		nuclear	XP_031229	mmu:76089	
K11799	DCAF4		PF23761.1(I	GO:003299	nuclear	NP_001158	mmu:73828	
K23605	SMAD3	mmu04550(	PF03166.19	GO:003292	cytoplasmic	NP_001164	mmu:17127	MH1
K15613	MEIS1	mmu05202(	PF16493.11	GO:008009	nuclear	KAF632143	mmu:17268	Homeobox
K08798	MARK		PF00069.31	GO:004858	cytoplasmic	XP_006515	mmu:17169	
K16815	PNPLA8		PF01734.28	GO:004643	peroxisoma	KAF160384	mmu:67452	
K20028	ZDHHC2_15_20		PF01529.25	GO:000548	lysosomal	NP_001347	mmu:75965	
K26434	LRRFIP		PF09738.14	GO:001921	cytoplasmic	NP_001361	mmu:71268	
K05749	CYFIP	mmu04810(	PF05994.16	GO:004867	cytoplasmic	NP_001158	mmu:20430	
K24943	TTC39		PF10300.15	GO:000557	cytoplasmic	NP_001139	mmu:230603	

K07898	RAB7B	mmu04137(	PF00071.28	GO:000810	cytoplasmic	NP_663484	mmu:226421	
TSW(Coiled-coil domain containing 137)				GO:000557	nuclear	NP_690020	mmu:67291	
K04682	E2F4_5	mmu04218(	PF16421.10	GO:008009	nuclear	NP_683754	mmu:10439	E2F
K00016	LDH, ldh	mmu05230(	PF00056.29	GO:005128	cytoplasmic	NP_034829	mmu:16828	
K25102	TRNAU1AP		PF17654.6(	GO:000645	cytoplasmic	NP_082201	mmu:71787	
K13625	FTL	mmu04978(	PF00210.30	GO:000032	cytoplasmic	BAE26571.	mmu:14325	
K16819	AMOT	mmu04390(	PF12240.13	GO:000810	cytoplasmic	NP_001390	mmu:27494	
K21946	DUSP6	mmu04010(	PF00782.25	GO:001922	cytoplasmic	BAE28407.	mmu:67603	
K16459	CEP120		PF12416.13	GO:190372	cytoplasmic	NP_848801	mmu:225523	
K00854	xylB, XYLB	mmu00040(	PF02782.22	GO:001932	cytoplasmic	NP_001186	mmu:102448	
K11564	MIS18, FASP1		PF03226.21	GO:003408	cytoplasmic	BAB29147.1	mmu:66578	
K07605	KRT2		PF00038.26	GO:004885	cytoplasmic	AAI20486.1	mmu:16681	
K05157	TNFRSF21,	mmu04060(	PF00020.24	GO:004213	cytoplasmic	BAC28342.	mmu:94185	
K09254	REL	mmu05202(	PF00554.27	GO:008009	nuclear	NP_033070	mmu:19696	RHD
K00921	PIKFYVE,	mmu04070(	PF01504.24	GO:000810	cytoplasmic	NP_001297	mmu:18711	
K10330	ASB8		PF00023.36	GO:001921	mitochondr	NP_001164	mmu:78541	
K03355	APC8, CDC	mmu04110(	PF04049.19	GO:003304	extracellulo	NP_848124	mmu:52563	
K12397	AP3B	mmu04142(	PF01602.26	GO:009951	cytoplasmic	NP_033810	mmu:11774	
K21751	DR1, NC2-beta		PF00808.29	GO:002260	cytoplasmic	BAE26046.	mmu:13486	
K24404	KIAA0319L		PF22352.2(	GO:002260	Golgi appa	NP_598647	mmu:100317	
K10373	TPM1	mmu04260(	PF00261.25	GO:009951	cytoplasmic	XP_055975	mmu:22003	
K22387	GDPD1_3,	mmu00565(	PF03009.23	GO:000548	ER	NP_079914	mmu:66569	
K00901	dgkA, DGK	mmu05231(	PF00609.24	GO:000810	cytoplasmic	NP_808314	mmu:227333	
K02564	nagB, GNP	mmu00520(	PF01182.26	GO:004634	cytoplasmic	NP_001033	mmu:67980	
K15332	TRMT2A		PF05958.17	GO:009030	cytoplasmic	NP_001074	mmu:15547	
26TE(chromosome 11 open reading			PF15692.10	GO:000367	nuclear	NP_001128	mmu:270156	
K15409	SRPK1	mmu05168(	PF00069.31	GO:000024	cytoplasmic	NP_058075	mmu:20815	
K02154	ATPeV0A,	mmu05165(	PF01496.24	GO:001922	cytoplasmic	AAH66839.	mmu:11975	
K10422	CLIP2, CYLN2		PF01302.31	GO:003111	nuclear	EDL19428.	mmu:269713	
YFSK(kiaa2013)			PF10222.14	GO:000557	ER	NP_084117	mmu:77034	
K12180	COPS7, CSN7		PF18392.6(	GO:002260	cytoplasmic	NP_001347	mmu:26895	
K01251	AHCY, ahc	mmu00270(	PF05221.23	GO:000578	cytoplasmic	NP_001164	mmu:74340	
K24727	MYORG		PF01055.32	GO:001678	Golgi appa	BAD32395.	mmu:329828	
K16866	TIMP3	mmu05205(	PF00965.23	GO:005104	extracellulo	NP_035725	mmu:21859	
K12870	ISY1	mmu03040(	PF06246.17	GO:002260	nuclear	NP_598695	mmu:57905	
ZXCM(LETM1 domain containing 1			PF07766.19	GO:000989	mitochondr	NP_598854	mmu:68614	
K24829	MINAR1		PF22948.1(	GO:190187	nuclear	EDL20892.	mmu:209743	
K00940	ndk, NME	mmu01240(	PF00334.24	GO:000921	cytoplasmic	BAB22162.1	mmu:79059	
K06111	EXOC4, SE	mmu05132(	PF20652.3(	GO:000810	cytoplasmic	NP_033174	mmu:20336	
K17435	MRPL54		PF08561.15	GO:001593	mitochondr	NP_079593	mmu:66047	
K20121	FNBP1		PF00611.28	GO:000032	nuclear	XP_006497	mmu:14269	
K13807	PPEF, PPP7C		PF00149.33	GO:000989	cytoplasmic	XP_030110	mmu:19023	
K00477	PHYH	mmu04146(	PF05721.19	GO:000548	mitochondr	NP_034856	mmu:16922	

K06773	OPCML, OBCAM		PF13927.12	GO:000557	extracellulo	NP_001157	mmu:210094	
K01265	map		PF00557.29	GO:000417	mitochondr	NP_079909	mmu:66559	
K00254	DHODH, p	mmu01240(	PF01180.27	GO:000921	mitochondr	NP_064430	mmu:56749	
K23298	SZT2			GO:003250	peroxisoma	NP_937813	mmu:230676	
K03126	TAF12	mmu03022(	PF03847.19	GO:003124	nuclear	XP_029396	mmu:66464	
K16194	EIF2AK1	mmu05162(	PF00069.31	GO:000690	cytoplasmic	BAD32438.	mmu:15467	
K11549	SPBC24, SPC24		PF08286.16	GO:004584	extracellulo	AAI16240.1	mmu:67629	
XRQZ(Apolipoprotein B receptor)				GO:003802	extracellulo	NP_612183	mmu:171504	
K26808	TXLN		PF09728.14	GO:000573	cytoplasmic	NP_619534	mmu:378431	
K26746	TMX1_4		PF00085.26	GO:008009	ER	NP_082615	mmu:72736	
K14318	NUP88	mmu03013(	PF10168.15	GO:003450	cytoplasmic	BAE38633.	mmu:19069	
Sulphatase-modifying factor protein			PF03781.22	GO:004323	ER	BAE38634.	mmu:67902	
K13702	ABHD10		PF12146.15	GO:010239	mitochondr	NP_766099	mmu:213012	
K17935	SNX29		PF02759.24	GO:003509	nuclear	NP_083240	mmu:74478	
K15627	ASPSCR1, /	mmu05202(	PF11470.14	GO:000810	cytoplasmic	NP_081153	mmu:68938	
K02861	RIPK1, RIF	mmu05170(	PF07714.23	GO:200123	cytoplasmic	NP_001346	mmu:19766	
K17493	CAMSAP		PF08683.16	GO:003111	cytoplasmic	NP_001334	mmu:67886	
XSSN(Coiled-coil domain containin			PF13778.12	GO:003015	extracellulo	NP_080715	mmu:67896	
K01103	PFKFB3	mmu04152(	PF01591.24	GO:001920	cytoplasmic	NP_001171	mmu:170768	
K20914	IFI16	mmu04621(	PF02760.21	GO:008009	nuclear	NP_032355	mmu:15951	
Y78B(myeloid cell nuclear differenti			PF02760.21	GO:008009	nuclear	NP_001028	mmu:381308	
K09552	SPG7		PF01434.24	GO:006102	mitochondr	NP_694816	mmu:234847	
K08271	STRADA, I	mmu04152(	PF00069.31	GO:005116	cytoplasmic	NP_001239	mmu:72149	
K05741	DIAPH2	mmu04810(	PF02181.28	GO:000810	nuclear	XP_006528	mmu:54004	
K01186	NEU1	mmu04142(	PF13088.12	GO:005113	peroxisoma	NP_035023	mmu:18010	
K02328	POLD2	mmu03440(	PF04042.21	GO:199023	mitochondr	NP_001348	mmu:18972	
K09533	DNAJC13		PF19432.4(I	GO:006034	cytoplasmic	NP_001156	mmu:235567	
K20493	LRRC16, CARMIL		PF16000.10	GO:000690	cytoplasmic	NP_001298	mmu:68732	
K23336	ARMC8		PF00514.29	GO:199023	cytoplasmic	NP_083044	mmu:74125	
K14847	RPF2		PF04427.23	GO:003450	nuclear	NP_001036	mmu:67239	
K15188	CCNT	mmu03250(	PF21797.2((	GO:008009	nuclear	BAE31187.	mmu:72949	
K17309	PLVAP		PF06637.16	GO:000548	plasma mer	NP_115774	mmu:84094	
K10871	RAD51L3, I	mmu03440(	PF21794.3(I	GO:000581	mitochondr	NP_001264	mmu:19364	
K24459	SLFN13		PF21026.2(S	GO:005160	cytoplasmic	NP_766384	mmu:237886	
K26545	UBAP2		PF12478.13	GO:000573	nuclear	NP_081148	mmu:68926	
K11647	SMARCA2_	mmu04714(	PF00176.29	GO:003506	nuclear	NP_001392	mmu:67155	
M5W(solute carrier family 7, memb			PF08574.15	GO:000810	cytoplasmic	NP_001007	mmu:66432	
K06071	PKN1	mmu04621(	PF00069.31	GO:001922	cytoplasmic	NP_796236	mmu:320795	
K09828	DHCR24, I	mmu00100(	PF01565.28	GO:000810	ER	NP_444502	mmu:74754	
K11151	RDH10	mmu00830(	PF00106.31	GO:000810	extracellulo	NP_598593	mmu:98711	
K25826	CBFB		PF02312.22	GO:008009	nuclear	NP_071704	mmu:12400	CBF
K03936	NDUFS3	mmu05208(	PF00329.25	GO:200123	mitochondr	NP_080964	mmu:68349	
K13981	AKR1E2, AKR1CL2		PF00248.27	GO:000403	peroxisoma	NP_061347	mmu:56043	

K19196	BRMS1		PF08598.16	GO:004323	cytoplasmic	NP_001360	mmu:107392	
K02154	ATPeV0A,	mmu05165(	PF01496.24	GO:009008	cytoplasmic	NP_001129	mmu:27060	
.6S0(SUN domain containing ossific			PF07738.19	GO:000573	plasma mer	NP_766233	mmu:226551	
K20184	VPS41	mmu04140(	PF23556.1(	GO:000810	cytoplasmic	EDL32708.	mmu:218035	
ZWW6(Heme binding protein 2)			PF04832.17	GO:000573	cytoplasmic	NP_062360	mmu:56016	
K09168	NFIA		PF10524.14	GO:003510	nuclear	XP_034874	mmu:18027	MH1
K03680	EIF2B4	mmu05168(	PF01008.23	GO:000367	cytoplasmic	NP_001120	mmu:13667	
K19728	UNC13D, MUNC13		PF00168.36	GO:000688	cytoplasmic	ABO15439.	mmu:70450	
K02741	PSMB9, LM	mmu03050(	PF00227.31	GO:000301	cytoplasmic	NP_038613	mmu:16912	
K02365	ESP1	mmu04114(	PF03568.22	GO:000367	cytoplasmic	BAC97882.	mmu:105988	
K25203	XPO4	mmu03013(Nucleocyto		GO:003315	cytoplasmic	NP_065252	mmu:57258	
K17413	MRPS35		PF10213.14	GO:001593	mitochondr	NP_663548	mmu:232536	
K08749	SLC27A6, F	mmu03320(	PF00501.34	GO:000663	ER	NP_001074	mmu:225579	
K25178	CCDC174		PF13300.11	GO:000557	nuclear	NP_766318	mmu:232236	
K10638	UHRF1, NP95		PF12148.14	GO:003506	nuclear	NP_001104	mmu:18140	
K13160	SYNCRIP, HNRNPQ		PF00076.28	GO:000578	nuclear	NP_001271	mmu:56403	
K05742	SLC9A1, N	mmu04971(	PF00999.27	GO:000962	extracellulo	BAE32882.	mmu:20544	
K19901	DAB2IP, A	mmu04210(	PF12004.13	GO:000512	cytoplasmic	XP_006498	mmu:69601	
K16725	CEP192		PF22074.2(	GO:000024	cytoplasmic	NP_081832	mmu:70799	
K15456	KTI12		PF08433.16	GO:003563	cytoplasmic	NP_083847	mmu:100087	
K15031	MTERF		PF02536.19	GO:002260	mitochondr	NP_079823	mmu:66410	
K03792	ST3GAL6	mmu00601(	PF00777.23	GO:005279	Golgi appa	NP_001344	mmu:54613	
.YE0(immunoglobulin heavy variab			PF07686.23(V-set:Immu		extracellulo	AAO21969.1(immunoglobulin heav		
K08334	BECN, VPS	mmu04137(	PF04111.17	GO:000004	cytoplasmic	NP_062530	mmu:56208	
K06553	VPREB, CD179a		PF07686.23(V-set:Immu		lysosomal	AAC31895.	mmu:22362	
K01446	PGRP		PF01510.30	GO:000959	ER	NP_033428	mmu:21946	
K07342	SEC61G, S	mmu04141(	PF00584.25	GO:000810	cytoplasmic	NP_001002	mmu:20335	
K20722	RTN2		PF02453.23	GO:000588	ER	NP_001020	mmu:20167	
K24135	MORC		PF17942.8(	GO:003506	nuclear	BAD32174.	mmu:338467	
K25802	ATF7IP, MCAF		PF16788.10	GO:006026	nuclear	XP_030111	mmu:54343	
K16578	CLASP1_2		PF21041.3(	GO:003111	cytoplasmic	EDL08947.	mmu:76499	
K07758	PDXP	mmu01240(	PF13344.12	GO:004643	cytoplasmic	NP_064667	mmu:57028	
K16172	IRS1	mmu04932(	PF02174.23	GO:009008	cytoplasmic	P35569.1(R	mmu:16367	
K12662	PRPF4, PR	mmu03040(	PF24814.1(	GO:007100	nuclear	BAC41100.	mmu:70052	
K04996	KCNJ2, KI	mmu04971(	PF17655.7(	GO:000526	plasma mer	NP_032451	mmu:16518	
K00744	MGAT5	mmu00510(	PF15024.11	GO:004691	Golgi appa	NP_660110	mmu:107895	
K08054	CANX	mmu04141(	PF00262.25	GO:000578	ER	NP_001103	mmu:12330	
K01176	AMY, amy	mmu04970(	PF02806.24	GO:001679	extracellulo	NP_001103	mmu:11722	
K00844	HK	mmu00052(	PF03727.22	GO:000167	cytoplasmic	NP_038848	mmu:15277	
K03853	CAPN2	mmu05022(	PF00648.27	GO:001922	cytoplasmic	NP_033924	mmu:12334	
K13216	PPP1R8, NIPP1		PF00498.31	GO:001920	nuclear	NP_001277	mmu:100336	
K24297	QRICH1		PF12012.13	GO:000548	nuclear	NP_001107	mmu:69232	
K17279	REEP5_6		PF03134.24	GO:000578	ER	NP_031900	mmu:13476	

K23295	KPTN			GO:000548	cytoplasmic	NP_598488	mmu:70394
K03239	EIF2B1	mmu05168(	PF01008.23	GO:004858	cytoplasmic	NP_663346	mmu:209354
K15169	MED26		PF15693.10	GO:006026	nuclear	NP_081761	mmu:70625
K23864	PNKD		PF15932.10	GO:006172	mitochondr	NP_079856	mmu:56695
KPY7(La ribonucleoprotein domain family men				GO:004323	nuclear	NP_001028	mmu:218236
K22030	LMOD	mmu04820(	PF03250.19	GO:009951	cytoplasmic	NP_001074	mmu:320502
K18763	LARP4		PF05383.23	GO:000548	cytoplasmic	NP_766173	mmu:217980
K11271	DSCC1, DCC1		PF09724.14	GO:007110	cytoplasmic	NP_001342	mmu:72107
K10863	APTX	mmu03410(	PF11969.13	GO:001679	nuclear	AAH71213.	mmu:66408
K17095	ANXA7_11	mmu05014(	PF00191.25	GO:007234	cytoplasmic	NP_038497	mmu:11744
K10776	LIG3	mmu03410(	PF01068.26	GO:008009	nuclear	P97386.2(R	mmu:16882
K04456	AKT	mmu05208(	PF00069.31	GO:000810	cytoplasmic	NP_001103	mmu:11652
KNYV(mcf.2 cell line derived transforming sequ				GO:001602	cytoplasmic	Q64096.2(RecName: Full=Guanine	
K23354	LIMS1_2, PINCH1_2		PF00412.28	GO:000810	cytoplasmic	NP_001333	mmu:110829
K05032	ABCC8, SU	mmu02010(	PF00664.28	GO:003577	plasma mer	NP_035640	mmu:20927
K18260	CC2D1		PF21528.2((	GO:000582	nuclear	AAH55068.	mmu:319965
2B7I(chromosome 18 open reading f			PF15719.10(DUF4674:I		nuclear	NP_080805	mmu:68046
K20189	DTNBP1, BLOC1S8		PF04440.21	GO:009951	cytoplasmic	NP_080048	mmu:94245
K15978	AKAP8L, HA95		PF04988.17	GO:008009	nuclear	NP_001390	mmu:54194
K24758	WDR89		PF24814.1(	GO:003250	cytoplasmic	NP_082479	mmu:72338
K20053	VLDLR	mmu05017(	PF00057.23	GO:004316	extracellulo	NP_001334	mmu:22359
K13698	ABHD4		PF00561.26	GO:000665	cytoplasmic	BAE31344.	mmu:105501
K16549	PDE4DIP		PF18615.6(S	GO:003111	cytoplasmic	BAC65568.	mmu:83679
K12483	EHD1	mmu04144(	PF18150.6(I	GO:000810	cytoplasmic	NP_001011	mmu:13660
K14398	CPSF6_7	mmu03250(	PF00076.28	GO:000548	nuclear	NP_001013	mmu:432508
K19400	LZTFL1		PF15294.11	GO:000367	cytoplasmic	BAC26767.	mmu:93730
3CL2 adenovirus E1B 19kDa interac			PF01596.23	GO:000998	cytoplasmic	NP_081241	mmu:69156
K08142	SLC2A3, GLUT3		PF00083.30	GO:000301	plasma mer	NP_035531	mmu:20527
K14773	UTP23		PF04900.18	GO:003204	nuclear	NP_084408	mmu:78581
K15261	PARP10_14_15		PF00644.27	GO:008009	cytoplasmic	NP_001157	mmu:671535
K12381	ARSG	mmu04142(	PF00884.28	GO:000032	lysosomal	NP_001348	mmu:74008
K08052	NF1	mmu01521(	PF21877.2(I	GO:001922	cytoplasmic	BAE34631.	mmu:18015
K21797	SAC1, SACI	mmu04070(	PF02383.23	GO:000810	cytoplasmic	NP_001344	mmu:83493
K00901	dgkA, DGK	mmu05231(	PF00609.24	GO:000665	cytoplasmic	NP_001074	mmu:380921
K08592	SENP1		PF02902.25	GO:007013	nuclear	XP_006520	mmu:223870
K01873	VARS, valS	mmu00970(	PF00133.28	GO:003563	cytoplasmic	NP_780346	mmu:68915
K24019	FCGRT		PF00129.23	GO:000989	extracellulo	NP_034319	mmu:14132
K03113	EIF1, SUI1		PF01253.27	GO:008009	cytoplasmic	NP_001099	mmu:20918
K11453	CBX6	mmu03083(	PF00385.30	GO:004239	nuclear	BAE26859.	mmu:494448
\HA1, activator of heat shock 90kD			PF09229.17	GO:000998	cytoplasmic	EDL07887.	mmu:268390
K10319	FBXO45		PF00622.36	GO:005104	extracellulo	NP_775615	mmu:268882
K24339	COL16A	mmu04974(	PF01391.23	GO:000520	extracellulo	AAH27766.	mmu:107581
K08729	PTDSS1	mmu00564(	PF03034.20	GO:000665	plasma mer	BAD32151.	mmu:19210

K06240	LAMA3_5	mmu05165(	PF00053.31	GO:000810	plasma mer	NP_001074	mmu:16776	
K04635	GNA11	mmu04925(	PF00503.26	GO:009008	cytoplasmic	NP_001230	mmu:14672	
K18024	PTPN12_18_22			GO:001679	cytoplasmic	BAE43238.1	mmu:19253	
K01077	E3.1.3.1, ph	mmu01240(	PF00245.25	GO:001922	cytoplasmic	BAH03518.	mmu:11647	
K02987	RP-S4e, RP	mmu05171(	PF00900.26	GO:002262	mitochondr	XP_021021	mmu:20102	
K01334	CFD	mmu04610(	PF00089.32	GO:000425	extracellulo	NP_001278	mmu:11537	
K06210	NMNAT	mmu01240(	PF01467.32	GO:008009	cytoplasmic	NP_001343	mmu:66454	
K08023	LTBP2_3_4		PF07645.21	GO:000810	mitochondr	AAI19786.1	mmu:16997	
K08134	P3H1		PF23557.1(	GO:003288	mitochondr	NP_062756	mmu:56401	
K24315	TGFB1I1		PF00412.28	GO:003243	cytoplasmic	NP_001404	mmu:21804	
K11973	RNF19B		PF01485.27	GO:000989	cytoplasmic	NP_001355	mmu:75234	
K12666	RPN1, OST	mmu04141(	PF04597.19	GO:199023	ER	AAH85483.	mmu:103963	
K17915	KIF14	mmu04814(	PF00225.29	GO:001989	cytoplasmic	AAI58133.1	mmu:381293	
K04378	SRF	mmu04010(	PF00319.23	GO:004867	mitochondr	NP_065239	mmu:20807	SRF
DNA replication complex GINS prc			PF04502.18	GO:002260	cytoplasmic	NP_082657	mmu:72886	
K13627	SLC12A7, K	mmu04966(	PF00324.27	GO:000682	cytoplasmic	NP_035520	mmu:20499	
K08816	VRK		PF00069.31	GO:000563	cytoplasmic	NP_081536	mmu:69922	
HGX(heme binding protein 1)			PF04832.17	GO:000573	cytoplasmic	NP_038574	mmu:15199	
K01490	AMPD	mmu01100(	PF19326.5(	GO:004643	cytoplasmic	NP_001359	mmu:11717	
BYQ(CDC42 small effector 1)				GO:002260	nuclear	NP_001033	mmu:57912	
K10412	DNAL4	mmu05014(	Amyotrophi	GO:009951	cytoplasmic	VFV42818.1	mmu:54152	
K19994	PRAM1		PF14603.12	GO:000548	cytoplasmic	AAI41101.1	mmu:378460	
K13156	SNRNP48		PF05253.18	GO:000613	nuclear	AAH37073.	mmu:67797	
K14438	CHD9		PF00176.29	GO:001688	nuclear	XP_006530	mmu:109151	
K16451	TIMP1	mmu04066(	PF00965.23	GO:005104	ER	NP_001037	mmu:21857	
K06114	SPTAN	mmu04210(	PF00435.27	GO:004299	cytoplasmic	XP_006497	mmu:20740	
K19871	SALL		PF00096.32	GO:000858	nuclear	NP_001357	mmu:58198	zf-C2H2
K11718	HUGT	mmu04141(	PF18404.7(	GO:000645	ER	EDL14473.	mmu:320011	
K04592	ADGRL1, LPHN1		PF02354.21	GO:009859	lysosomal	XP_029336	mmu:330814	
K00901	dgkA, DGK	mmu05231(	PF00609.24	GO:001922	mitochondr	NP_950176	mmu:110524	
K19913	SYT13		PF00168.36	GO:005104	ER	NP_109650	mmu:80976	
K00688	PYG, glgP	mmu01100(	PF00343.26	GO:004323	cytoplasmic	NP_035354	mmu:19309	
K10630	RBCK1, HC	mmu04621(	PF13445.12	GO:200123	cytoplasmic	EDL05958.	mmu:24105	TF_others
Z29E(chromosome 9 open reading fr			PF15055.11	GO:000699	cytoplasmic	NP_080125	mmu:66928	
XRFG(Transmembrane protein 57)			PF09726.14	GO:004299	nuclear	NP_079658	mmu:66146	
K02895	RP-L24, MF	mmu03010(	PF17136.10	GO:001593	mitochondr	NP_080867	mmu:67707	
K23966	CCNL		PF00134.28	GO:008009	nuclear	NP_997561	mmu:56036	
K12158	NEDD8		PF00240.29	GO:000810	cytoplasmic	NP_032709	mmu:18002	
K09061	GCF, C2orf3		PF07842.17	GO:007100	nuclear	NP_808552	mmu:33036	GCFC
XPY7(family with sequence similarity)				GO:004323	nuclear	AAI71940.1	mmu:207375	
K17090	ANXA9		PF00191.25	GO:001602	cytoplasmic	NP_001078	mmu:71790	
K16590	HAUS7, AUG7, UIP1			GO:002260	cytoplasmic	NP_082909	mmu:73738	
K12946	SPCS1	mmu03060(	PF06645.18	GO:000578	ER	NP_081187	mmu:69019	

K23620	AMBP		PF00014.28	GO:000486	plasma mer	NP_031469	mmu:11699
K23765	S100A11_13, S100C		PF01023.25	GO:004830	cytoplasmic	NP_058020	mmu:20195
K16729	CHORDC1, CHP1		PF04968.17	GO:003367	cytoplasmic	NP_080120	mmu:66917
K09479	ACADVL	mmu01100(	PF21343.2(	GO:003444	mitochondr	NP_059062	mmu:11370
K08761	PLTP	mmu04979(	PF02886.23	GO:000677	extracellula	NP_001407	mmu:18830
K26415	ARHGAP45		PF00620.32	GO:000548	nuclear	NP_001136	mmu:70719
K14943	MBNL		PF22628.2(	GO:008009	cytoplasmic	NP_064391	mmu:56758
K06583	ITGA7	mmu05165(	PF20806.3(	GO:002260	ER	NP_032424	mmu:16404
K12168	HECW2		PF00632.30	GO:003304	cytoplasmic	NP_001001	mmu:329152
K07200	PRKAG	mmu04530(	PF00571.33	GO:005116	cytoplasmic	NP_058061	mmu:19082
K06627	CCNA	mmu05165(	PF00134.28	GO:000962	cytoplasmic	NP_033958	mmu:12428
K10289	CCNF, FBXO1		PF00134.28	GO:009877	nuclear	NP_031660	mmu:12449
K04431	MAP2K7, M	mmu05208(	PF00069.31	GO:001922	cytoplasmic	Q8CE90.1(	mmu:26400
K13198	KHDRBS1		PF16274.10	GO:003238	cytoplasmic	NP_035447	mmu:20218
K10842	MNAT1	mmu03420(	PF06391.18	GO:001003	cytoplasmic	NP_032638	mmu:17420
K07561	DPH1, dph2		PF01866.22	GO:004814	cytoplasmic	NP_652762	mmu:116905
K20526	TAGLN		PF00307.36	GO:007149	cytoplasmic	NP_035656	mmu:21345
K16342	PLA2G4, C	mmu04750(	PF01735.23	GO:004298	cytoplasmic	NP_032895	mmu:18783
K04079	HSP90A, ht	mmu04915(	PF00183.23	GO:000645	cytoplasmic	AAH08189.	mmu:15516
K04982	TRPM7, CH	mmu04621(	PF18139.7(	GO:000526	cytoplasmic	NP_001157	mmu:58800
K11839	USP8, UBP	mmu04934(	PF00443.35	GO:005080	nuclear	NP_001239	mmu:84092
K10370	TMOD	mmu04820(	PF03250.19	GO:009951	cytoplasmic	NP_068683	mmu:21916
K23503	SFXN5		PF03820.22	GO:000684	cytoplasmic	BAC31624.	mmu:94282
K06278	CAV1	mmu05020(	PF01146.22	GO:001922	cytoplasmic	NP_001396	mmu:12389
K20364	MCFD2		PF13499.12	GO:000810	ER	NP_647456	mmu:193813
K12473	LDLR	mmu04913(	PF00057.23	GO:003022	plasma mer	NP_034830	mmu:16835
K23934	AHNAK	mmu05132(Salmonella		GO:004348	cytoplasmic	NP_033773	mmu:66395
K06201	cutC		PF03932.20	GO:002260	cytoplasmic	EDL41912.	mmu:66388
K13785	TMSB10		PF01290.25	GO:000548	cytoplasmic	XP_012893	mmu:19240
KPJ1(cyclin-dependent kinase 2 asso			PF00059.27	GO:000989	plasma mer	NP_444339	mmu:93694
KR89(dopamine beta-hydroxylase (d			PF01082.25	GO:000548	extracellula	NP_067484	mmu:59012
K06127	COQ5	mmu01240(	PF01209.24	GO:003197	mitochondr	NP_080780	mmu:52064
K02941	RP-LP0, RP	mmu05171(	PF17777.6(	GO:001593	cytoplasmic	XP_007528	mmu:11837
K23781	ADAP, CENTA		PF00169.35	GO:001602	cytoplasmic	AAI44875.1	mmu:231821
K26117	TICRR	mmu04110(	PF21854.2(	GO:000548	nuclear	NP_084111	mmu:77011
K17559	PPP1R18		PF13916.11	GO:000573	cytoplasmic	NP_001365	mmu:76448
K13165	SREK1, SFRS12		PF00076.28	GO:000613	nuclear	BAC30314.	mmu:218543
K10648	TRIM5	mmu03250(	PF00622.36	GO:003577	cytoplasmic	NP_001139	mmu:319236
K20066	SH3BP4		PF23640.1(	GO:000810	cytoplasmic	NP_001407	mmu:98402
K26535	HFE	mmu04350(	PF00129.23	GO:009008	ER	NP_034554	mmu:15216
K24524	CPNE1_2_3		PF07002.22	GO:004858	cytoplasmic	NP_733467	mmu:266692
GTB(Chromosome 6 open reading			PF10239.14	GO:000647	cytoplasmic	EDL38449.	mmu:72722
K21248	VMP1	mmu04140(	PF09335.16	GO:000810	ER	NP_001343	mmu:75909

K06553	VPREB, CD179a		PF07686.23	GO:000237	cytoplasmic	P01654.1(R	mmu:22363	
K11600	RRP41, EX	mmu03018(	PF01138.27	GO:007104	nuclear	NP_780608	mmu:109075	
K03994	C5	mmu05171(	PF07678.20	GO:000486	extracellulo	NP_034536	mmu:15139	
K06631	PLK1	mmu04110(	PF00069.31	GO:000810	cytoplasmic	NP_035251	mmu:18817	
K04524	APOE	mmu04979(	PF01442.23	GO:000810	extracellulo	BAE29894.1	mmu:11816	
K04565	SOD1	mmu05208(	PF00080.25	GO:004340	cytoplasmic	NP_035564	mmu:20655	
K23506	APOPT1		PF10231.14	GO:003241	mitochondr	BAC26437.1	mmu:68020	
K06749	SIGLEC10	mmu04148(	PF13927.12	GO:001922	plasma mer	NP_766488	mmu:243958	
K16741	ALMS1		PF18727.6(	GO:001922	cytoplasmic	XP_006506	mmu:236266	
K13131	DDX20, GEMIN3		PF00270.35	GO:008009	cytoplasmic	NP_059093	mmu:53975	
K18203	LCMT1		PF04072.20	GO:003304	cytoplasmic	NP_079580	mmu:30949	
K01787	RENBP	mmu00520(	PF07221.17	GO:003041	cytoplasmic	NP_075621	mmu:19703	
K04237	PARD3	mmu05165(	PF12053.14	GO:000810	cytoplasmic	NP_001296	mmu:93742	
K05665	ABCC1	mmu02010(	PF00664.28	GO:004858	ER	NP_032602	mmu:17250	
K09069	TWIST	mmu05205(	PF00010.31	GO:008009	cytoplasmic	NP_001077	mmu:13345	bHLH
K16308	CIT		PF00780.28	GO:003304	cytoplasmic	NP_031734	mmu:12704	
K18914	FDXR		PF07992.20	GO:000548	mitochondr	BAE22000.1	mmu:14149	
K24319	SCARF2		PF00053.31	GO:003802	extracellulo	NP_722485	mmu:224024	
K01202	GALC	mmu04142(	PF02057.21	GO:000032	lysosomal	NP_032105	mmu:14420	
K10515	ZBTB44, BTBD15		PF00651.37	GO:004322	nuclear	XP_006510	mmu:23513	ZBTB
K05125	DDR2, TKT, CD167b		PF07714.23	GO:003598	extracellulo	NP_072075	mmu:18214	
K23499	RMND1		PF02582.19	GO:007013	mitochondr	NP_001345	mmu:66084	
K14829	IPI3		PF00400.38	GO:003638	extracellulo	NP_780659	mmu:216156	
K14958	KSR1	mmu04625(	PF20406.3(	GO:001922	cytoplasmic	XP_011247	mmu:16706	
K06057	NUMBL	mmu04330(	PF00640.29	GO:003288	cytoplasmic	NP_001129	mmu:18222	
NSQ(Leucine rich repeat containin			PF13855.12	GO:003250	cytoplasmic	NP_705770	mmu:216011	
K23690	SH2D3C		PF00017.30	GO:009859	cytoplasmic	NP_038809	mmu:27387	
K18758	DIS3L2		PF00773.24	GO:003577	cytoplasmic	NP_705758	mmu:208718	
K17705	KRIT1, CC	mmu04520(	PF16705.11	GO:005068	cytoplasmic	XP_009000	mmu:79264	
K03878	ND1	mmu05208(	PF00146.26	GO:003042	ER	YP_220563.	mmu:17716	
K04712	DEGS	mmu04071(	PF00487.30	GO:000663	cytoplasmic	NP_031879	mmu:13244	
K12318	GUCY1A	mmu04970(	PF00211.25	GO:004858	cytoplasmic	NP_001343	mmu:60596	
K01476	rocF, arg	mmu01100(	PF00491.26	GO:004213	cytoplasmic	NP_031508	mmu:11846	
K03386	PRDX2_4, ahpC		PF00578.27	GO:200123	cytoplasmic	NP_001304	mmu:21672	
K03844	ALG11	mmu00510(	PF15924.11	GO:000578	ER	NP_001230	mmu:207958	
K07857	RHOC		PF00071.28	GO:004858	nuclear	NP_001036	mmu:11853	
K12009	TRIM27, RFP		PF00622.36	GO:005134	cytoplasmic	NP_033080	mmu:19720	
K00011	AKR1B	mmu00052(	PF00248.27	GO:000403	cytoplasmic	NP_032038	mmu:14187	
K00011	AKR1B	mmu00052(	PF00248.27	GO:000810	cytoplasmic	NP_033788	mmu:11677	
K23867	ECM1		PF05782.16	GO:008009	extracellulo	UNO37363	mmu:13601	
K10494	ZBTB7		PF00651.37	GO:000810	nuclear	AAI71957.1	mmu:16969	ZBTB
K10431	MAP4		PF00418.24	GO:000367	cytoplasmic	XP_017168	mmu:17758	
K10057	CLEC4A, CD367		PF00059.27	GO:001922	cytoplasmic	ACD47046.	mmu:26888	

K09378	ZFHX3, AT	mmu04550(	PF00046.35	GO:003292	nuclear	NP_031522	mmu:11906	Homeobox
K00626	ACAT, atoB	mmu00650(	PF00108.29	GO:003444	cytoplasmic	EDL02025.	mmu:110460	
K00019	BDH1, bdh	mmu00650(	PF00106.31	GO:000188	mitochondr	NP_001116	mmu:71911	
K16056	ORAI1	mmu04024(	PF07856.17	GO:009008	cytoplasmic	NP_780632	mmu:109305	
K05768	GSN	mmu04810(	PF00626.27	GO:003250	cytoplasmic	KAI255373	mmu:227753	
K06185	ABCF2		PF00005.33	GO:003563	cytoplasmic	BAE23280.	mmu:27407	
K00858	ppnK, NAI	mmu01240(	PF01513.26	GO:000548	mitochondr	NP_001078	mmu:68646	
K07050	AARSD1, ALAX		PF07973.19	GO:000216	cytoplasmic	XP_051033	mmu:69684	
K12818	DHX8, PRI	mmu03040(	PF07717.22	GO:000548	nuclear	NP_659080	mmu:217207	
K06972	PITRM1, PreP, CYM1		PF08367.16	GO:000417	mitochondr	NP_660113	mmu:69617	
K07555	ATPeAF1, ATPAF1, AT		PF06644.16	GO:003361	cytoplasmic	NP_851383	mmu:230649	
K22383	IRF2BP		PF11261.13	GO:000989	cytoplasmic	NP_848872	mmu:272359	
K04735	RELA	mmu05208(	PF00554.27	GO:008009	nuclear	NP_033071	mmu:19697	RHD
K07305	msrB		PF01641.26	GO:002260	mitochondr	NP_083895	mmu:76467	
K06266	CD47	mmu04148(	PF04549.19	GO:003802	extracellulo	NP_001355	mmu:16423	
K22753	RNF6		PF13639.12	GO:004867	cytoplasmic	NP_001243	mmu:74132	
K08573	CAPN3	mmu04820(	PF00648.27	GO:000810	cytoplasmic	XP_021038	mmu:12335	
K17655	MRPP3		PF16953.11	GO:000454	cytoplasmic	NP_079649	mmu:66132	
K17771	TOM7	mmu04137(	PF08038.17	GO:000810	ER	NP_001128	mmu:66169	
K12459	SH2B1_3	mmu04722(	PF08916.16	GO:004323	cytoplasmic	NP_001276	mmu:20399	
K12398	AP3M	mmu04142(	PF00928.26	GO:000548	cytoplasmic	BAB23521.1	mmu:55946	
K08852	ERN1	mmu05022(	PF00069.31	GO:000454	cytoplasmic	NP_076402	mmu:78943	
K07907	RAB12		PF00071.28(Ras:Ras far		cytoplasmic	NP_077768	mmu:19328	
K19519	TGFBI, BIGH3		PF02469.27	GO:005084	ER	NP_033395	mmu:21810	
K10347	LRRC41, MUF1			GO:004322	cytoplasmic	NP_705741	mmu:230654	
K09414	HSF1	mmu05134(	PF06546.16	GO:009008	nuclear	NP_001318	mmu:15499	HSF
K07848	REM2		PF00071.28	GO:009910	cytoplasmic	NP_542764	mmu:140743	
					cytoplasmic	BAE25636.1(unnamed protein prod		
K18729	ANGEL		PF19339.4(	GO:000989	cytoplasmic	NP_067396	mmu:52477	
AXV(Chromosome 3 open reading			PF15008.12	GO:000557	cytoplasmic	NP_080549	mmu:67609	
K13236	DECR1		PF13561.12	GO:000548	mitochondr	NP_080448	mmu:67460	
)29H(family with sequence similarit			PF05334.18	GO:000557	cytoplasmic	NP_080943	mmu:68303	
K01312	PRSS1_2_3	mmu05164(	PF00089.32	GO:000425	extracellulo	NP_075822	mmu:67373	
K13825	HBE		PF00042.28	GO:000534	cytoplasmic	NP_032245	mmu:15132	
K10088	OS9	mmu04141(	PF07915.19	GO:003315	ER	EDL24469.	mmu:216440	
K20304	TRAPPC6, TRS33		PF04051.21	GO:000580	cytoplasmic	NP_084333	mmu:78232	
K06463	FCGR3, CI	mmu04666(	PF13895.12	GO:001922	ER	NP_653142	mmu:246256	
XPQX(RNA-binding nucleolar prot			PF04503.18	GO:008009	nuclear	NP_598533	mmu:76900	
K04399	CASP9	mmu05020(	PF00656.27	GO:003202	cytoplasmic	CAJ18606.1	mmu:12371	
K17783	ERV1, GFER, ALR		PF04777.18	GO:008009	cytoplasmic	NP_001365	mmu:11692	
K18442	ARFGEF, I	mmu04144(	PF01369.25	GO:000810	cytoplasmic	NP_001078	mmu:99371	
K20120	BIN3		PF03114.23	GO:000367	cytoplasmic	BAE42110.	mmu:57784	
K12617	PATL1, PA	mmu03018(	PF09770.15	GO:000582	cytoplasmic	NP_766223	mmu:225929	

K10459	KLHL22, KELCHL		PF01344.31	GO:009951	cytoplasmic	NP_001345	mmu:22402	ZBTB
K10500	ZBTB17, M	mmu05200(	PF13465.12	GO:004212	nuclear	AAA64848.	mmu:22642	ZBTB
K03987	C1QB	mmu05171(	PF00386.27	GO:000548	cytoplasmic	SOR70269.1	mmu:12260	
K04632	GNAS	mmu04750(	PF00503.26	GO:005104	cytoplasmic	NP_001268	mmu:14683	
K04388	TGFBR2	mmu05200(	PF08917.16	GO:200073	plasma mer	BAE28387.1	mmu:21813	
K22369	EPHX4	mmu05208(	PF00561.26	GO:001678	cytoplasmic	XP_038947	mmu:384214	
K13241	NOS2	mmu00330(	PF02898.20	GO:009008	peroxisoma	AAC52356.	mmu:18126	
K13697	ABHD2		PF00561.26	GO:004846	cytoplasmic	NP_061281	mmu:54608	
K13506	GPAT3_4,	mmu00564(	PF01553.26	GO:000578	ER	NP_061213	mmu:102247	
K20824	NAA38		PF01423.28	GO:003124	cytoplasmic	NP_084359	mmu:78304	
KSVC(cell division cycle)			PF07065.19	GO:002260	cytoplasmic	NP_598598	mmu:98828	
K12736	PPWD1		PF00160.26	GO:000613	cytoplasmic	NP_766395	mmu:238831	
K12844	PRPF31	mmu03040(	PF01798.23	GO:000569	nuclear	NP_081604	mmu:68988	
K25354	APOM		PF11032.14	GO:003030	extracellulo	NP_061286	mmu:55938	
K05618	SLC1A7, E/	mmu04724(	PF00375.23	GO:000682	ER	NP_666367	mmu:242607	
K09522	DNAJC2		PF21884.2(	GO:008009	nuclear	NP_033610	mmu:22791	MYB
K01325	KLK1_2	mmu04614(	PF00089.32	GO:000425	lysosomal	SFW93243.1	mmu:16622	
K09578	PIN1	mmu03250(	PF00639.27	GO:004340	cytoplasmic	BAE23993.1	mmu:23988	
K00507	SCD, desC	mmu01100(	PF00487.30	GO:001922	cytoplasmic	NP_033153	mmu:20249	
K09228	KRAB		PF13465.12	GO:008009	cytoplasmic	NP_001345	mmu:22366	zf-C2H2
K13354	SLC25A17,	mmu04146(	PF00153.32	GO:003444	peroxisoma	NP_035529	mmu:20524	
K16065	PIAS4	mmu05418(	PF14324.12	GO:003288	nuclear	BAE28155.1	mmu:59004	zf-MIZ
K00653	CDY		PF00378.25	GO:003067	Golgi appa	BAE36774.1	mmu:12593	
K02947	RP-S10e, RI	mmu05171(	PF03501.21	GO:002262	cytoplasmic	NP_080239	mmu:67097	
K13073	PIGR	mmu04672(	PF07686.23	GO:000717	extracellulo	NP_035212	mmu:18703	
K00898	PDK2_3_4	mmu05415(	PF10436.14	GO:001922	mitochondr	NP_038771	mmu:27273	
K12351	SMPD2	mmu04071(	PF03372.29	GO:000548	mitochondr	NP_033239	mmu:20598	
K17867	DPH4, DNAJC24		PF00226.36	GO:004322	cytoplasmic	NP_081268	mmu:99349	
VGK(chromosome 2 open reading			PF10209.14(	DUF2340:U	cytoplasmic	NP_082715	mmu:73103	
K14805	DDX24, MAK5		PF00270.35	GO:000367	nuclear	EDL18834.	mmu:27225	
K19511	PXDN, VPO1		PF03098.20	GO:000520	lysosomal	AAI50790.1	mmu:69675	
K14405	FIP1L1, FII	mmu03015(	PF05182.18	GO:008009	nuclear	NP_001153	mmu:66899	
K14764	SHQ1		PF04925.20	GO:002260	cytoplasmic	EDK99374.	mmu:72171	
rolyl-tRNA synthetase)			PF04073.20	GO:001678	cytoplasmic	NP_080741	mmu:67939	
K15682	PDZRN3_4, LNX3_4		PF00595.29	GO:000548	nuclear	NP_061372	mmu:55983	
K22651	RNF4		PF13639.12	GO:003288	nuclear	BAE34970.1	mmu:19822	
K09292	TFG	mmu05200(	PF00564.30	GO:004820	cytoplasmic	BAB25736.1	mmu:21787	
K08535	NR1H2, LX	mmu04148(	PF00104.35	GO:008009	nuclear	NP_001272	mmu:22260	THR-like
K02725	PSMA1	mmu05020(	PF00227.31	GO:003210	cytoplasmic	NP_036095	mmu:26440	
K11412	SIRT2, SIR	mmu00760(	PF02146.23	GO:009951	cytoplasmic	NP_001116	mmu:64383	
K01102	PDP		PF00481.26	GO:001679	mitochondr	NP_001019	mmu:382051	
K12860	CDC5L, CI	mmu03040(	PF11831.13	GO:008009	nuclear	NP_690023	mmu:71702	MYB
YXE9(chromosome 15 open reading			PF06522.17	GO:000960	ER	NP_001004	mmu:433470	

K03869	CUL3	mmu04120(	PF00888.28	GO:009951	cytoplasmic	BAC97984.2	mmu:26554	
K05398	TLR1, CD2	mmu04620(	PF01582.25	GO:000959	plasma mer	NP_001263	mmu:21897	
K17697	DOCK4	mmu04015(	PF23554.2(	GO:004590	cytoplasmic	NP_766391	mmu:238130	
K02140	ATPeFG, A	mmu04714(	PF04718.20	GO:000526	cytoplasmic	NP_038823	mmu:27425	
FCX(leucine rich repeat containing 14); ENOC				GO:004212	cytoplasmic	XP_021016	mmu:242736	
K10684	UBLE1A, S	mmu04120(	PF00899.27	GO:190187	cytoplasmic	NP_001272	mmu:56459	
K17769	TOM22		PF04281.18	GO:000573	peroxisoma	NP_766197	mmu:223696	
K02268	COX6C	mmu05208(	PF02937.20	GO:001545	mitochondr	NP_444301	mmu:12864	
K06553	VPREB, CD179a		PF07686.23	GO:000237	extracellulo	BAA03482.	mmu:22362	
K14959	MLL4	mmu00310(	PF00856.33	GO:008009	nuclear	XP_006540	mmu:75410	
K01539	ATP1A	mmu04971(	PF00122.25	GO:199053	cytoplasmic	NP_001361	mmu:232975	
EZY(Zinc finger protein)			PF12402.13	GO:008009	cytoplasmic	NP_001094	mmu:353310	
K05853	ATP2A	mmu04972(	PF00122.25	GO:005080	ER	EDL19677.	mmu:11938	
K15166	MED23		PF11573.13	GO:008009	cytoplasmic	XP_021029	mmu:70208	
K13506	GPAT3_4,	mmu00564(	PF01553.26	GO:010242	ER	NP_766303	mmu:231510	
K01755	argH, ASL	mmu00220(	PF00206.25	GO:001922	cytoplasmic	BAE23655.	mmu:109900	
K12181	COPS8, CSN8		PF10075.15	GO:002260	cytoplasmic	NP_598566	mmu:108679	
K04180	CCR5, CD1	mmu05167(	PF00001.26	GO:007084	plasma mer	NP_034047	mmu:12774	
K06493	ITGB3, CD	mmu04512(	PF00362.24	GO:003802	plasma mer	NP_058060	mmu:16416	
K20058	ITGB1BP1		PF10480.15	GO:004340	cytoplasmic	NP_001342	mmu:16413	
K24730	CIAO1, CIA1		PF00400.38	GO:002260	cytoplasmic	NP_079572	mmu:26371	
K26978	ZNF526_574		PF00096.32	GO:008009	cytoplasmic	NP_780645	mmu:21017	zf-C2H2
K19721	COL5AS	mmu04974(	PF01391.23	GO:000520	ER	NP_058615	mmu:53867	
K01487	guaD, GD	mmu01100(	PF01979.26	GO:003111	cytoplasmic	NP_034396	mmu:14544	
K01758	CTH	mmu00270(	PF01053.26	GO:200123	cytoplasmic	NP_666065	mmu:107869	
K01273	DPEP		PF01244.27	GO:000880	extracellulo	NP_001403	mmu:13479	
Y9DJ(WD repeat domain 13)			PF01448.29	GO:000989	nuclear	NP_001034	mmu:71148	MYB
K4M(family with sequence similari			PF14161.11	GO:009951	nuclear	AAH25092.	mmu:73847	
VEG(Proline-rich coiled-coil)			PF07001.16	GO:002260	nuclear	XP_011237	mmu:226562	
K01824	EBP	mmu00100(	PF05241.17	GO:000548	cytoplasmic	NP_031924	mmu:13595	
K08816	VRK		PF00069.31	GO:001707	cytoplasmic	NP_001406	mmu:101568	
K09113	MLX	mmu04931(	PF00010.31	GO:005116	nuclear	NP_035680	mmu:21428	bHLH
K05692	ACTB_G1	mmu04530(	PF00022.25	GO:001562	cytoplasmic	NP_001009	mmu:11461	
K07604	KRT1	mmu04915(	PF00038.26	GO:004886	mitochondr	NP_058654	mmu:16664	
K10807	RRM1	mmu00983(	PF02867.20	GO:006173	cytoplasmic	NP_033129	mmu:20133	
K19932	NCS1		PF13499.12	GO:003024	cytoplasmic	NP_001232	mmu:14299	
K06494	SELE, CD6	mmu05144(	PF00084.26	GO:005104	plasma mer	XP_006496	mmu:20339	
K14416	HBS1	mmu05134(	PF08938.16(HBS1_N:H		cytoplasmic	NP_001138	mmu:56422	
K03130	TAF5	mmu03022(	PF00400.38	GO:003124	nuclear	BAC40894.	mmu:102162	
K10352	MYH9s	mmu04530(	PF01576.24	GO:002260	cytoplasmic	BAE27768.	mmu:17886	
K08654	PCSK5		PF00082.27	GO:000425	extracellulo	NP_001177	mmu:18552	
K27276	GSE1		PF12540.13(DUF3736:P		cytoplasmic	NP_001139	mmu:382034	
K18461	WASH1	mmu04144(	PF11945.13	GO:003288	cytoplasmic	NP_001032	mmu:68767	

K14801	TSR4		PF04194.18	GO:000828	cytoplasmic	NP_032825	mmu:18567	
K02257	COX10, cta	mmu01240(Biosynthesis		GO:002260	mitochondr	BAE33878.1	mmu:70383	
K02513	HS2ST1	mmu00534(	PF03567.20	GO:003020	Golgi appa	NP_035958	mmu:23908	
K00799	GST, gst	mmu00980(	PF02798.26	GO:000663	lysosomal	NP_081040	mmu:14865	
K23480	ZNF362_384, LIN-29			GO:005116	cytoplasmic	BAC39626.1	mmu:269800	
K13375	TGFB1	mmu05200(	PF00688.24	GO:003042	extracellulo	NP_035707	mmu:21803	
8DX(Microtubule associated tumor suppressor				GO:003210	cytoplasmic	NP_001005	mmu:102103	
K00413	CYC1, CYT	mmu05208(	PF02167.20	GO:005089	mitochondr	NP_079843	mmu:66445	
K23330	LZTR1		PF24681.1(I	GO:005503	cytoplasmic	NP_080084	mmu:66863	ZBTB
K12489	ACAP	mmu04144(	PF16746.10	GO:000367	cytoplasmic	XP_006522	mmu:78618	
K11847	USP19		PF00443.35	GO:008009	cytoplasmic	NP_001161	mmu:71472	
K11793	CRBN		PF02190.22	GO:005104	cytoplasmic	NP_780566	mmu:58799	
K23881	TECPR2		PF06462.18	GO:000573	cytoplasmic	AAI38355.1	mmu:104859	
XJX(Family with sequence similari			PF22566.2(UBA_8:UBA		cytoplasmic	NP_663334	mmu:207740	
K23714	SH3D19		PF00018.34(SH3_1:SH3		cytoplasmic	NP_001391	mmu:27059	
K05400	LY96, MD-:	mmu04620(Toll-like rec		GO:000959	extracellulo	NP_001153	mmu:17087	
ZN1U(IGv); ENOG410YS8R(IGv); I			PF07686.23	GO:000237	extracellulo	CAB46113.1(immunoglobulin light		
K06553	VPREB, CD179a		PF07686.23(V-set:Immu		plasma mer	CAB46170.1	mmu:22363	
K13254	SPAST		PF00004.35	GO:003111	plasma mer	AAH46286.	mmu:50850	
K21764	SPAG5			GO:000810	cytoplasmic	NP_059103	mmu:54141	
K11373	ELP1, IKI3, IKBKAP		PF04762.18	GO:003563	cytoplasmic	NP_080355	mmu:230233	
4JX(transporter activity)			PF00363.23	GO:190170	extracellulo	NP_031810	mmu:12990	
K07293	PTPN11, SI	mmu05208(	PF00102.32	GO:004858	cytoplasmic	NP_001103	mmu:19247	
K02605	ORC3	mmu04110(	PF07034.17	GO:008009	mitochondr	BAE26368.1	mmu:50793	
K12843	PRPF3, PRI	mmu03040(	PF08572.15	GO:002260	nuclear	NP_001303	mmu:70767	
K06238	COL6A	mmu05165(	PF00092.34	GO:000520	extracellulo	NP_001096	mmu:245026	
K11728	BPTF, E(bx	mmu03082(	PF00439.30	GO:000749	nuclear	NP_789820	mmu:207165	
K22190	MS4A3S		PF04103.21	GO:000579	plasma mer	AAH28433.	mmu:60361	
K04543	GNG7	mmu04724(	PF00631.27	GO:000762	cytoplasmic	NP_001033	mmu:14708	
K26189	ARMCX3		PF04826.19	GO:001604	nuclear	NP_001345	mmu:71703	
K17772	TOM6		PF15184.11	GO:000810	cytoplasmic	NP_001158	mmu:66119	
K05357	VKORC1	mmu01240(	PF07884.19	GO:000548	ER	QIJ55489.1(	mmu:27973	
K13090	ILF3		PF07528.20	GO:008009	nuclear	NP_001264	mmu:16201	
K08517	SEC22	mmu05134(	PF13774.12	GO:000810	cytoplasmic	NP_001020	mmu:20333	
K00030	IDH3	mmu01100(	PF00180.25	GO:005128	mitochondr	XP_021027	mmu:67834	
K15151	MED10, NUT2		PF09748.14	GO:006026	cytoplasmic	XP_006517	mmu:28077	
K22182	TXNRD	mmu05200(	PF07992.20	GO:003459	mitochondr	NP_001171	mmu:232223	
K00157	AOX	mmu00982(	PF20256.3(	GO:000403	cytoplasmic	NP_033806	mmu:11761	
K15123	SLC25A45_47		PF00153.32	GO:003109	Golgi appa	NP_001348	mmu:107375	
K12023	TRIM47		PF13445.12	GO:004322	cytoplasmic	NP_766158	mmu:217333	
K09228	KRAB		PF13465.12	GO:008009	nuclear	XP_036009	mmu:54593	zf-C2H2
K15711	SMARCA3, HLTF		PF00176.29	GO:008009	nuclear	NP_001342	mmu:20585	
K23582	FOXP1	mmu05206(	PF16159.10	GO:003210	nuclear	NP_001184	mmu:10865	Fork head

K23540	GOLIM4			GO:000579	Golgi appa	NP_001277	mmu:73124	
K00757	udp, UPP	mmu00983(	PF01048.26	GO:004643	peroxisoma	NP_001152	mmu:22271	
K10431	MAP4		PF00418.24	GO:000367	cytoplasmic	NP_001192	mmu:17758	
K23764	S100A6		PF01023.25	GO:004830	cytoplasmic	NP_001404	mmu:20200	
K13179	DDX18, HAS1		PF00270.35	GO:000367	cytoplasmic	NP_080136	mmu:66942	
K00799	GST, gst	mmu00980(	PF00043.30	GO:000663	cytoplasmic	NP_034488	mmu:14862	
ZN1U(immunoglobulin kappa varia			PF07686.23	GO:000237	extracellula	P01627.1(RecName: Full=Immunog		
K25573	EHBP1L1		PF12130.13	GO:005503	cytoplasmic	NP_001108	mmu:114601	
K06553	VPREB, CD179a		PF07686.23(V-set:Immu		cytoplasmic	ACI04616.1	mmu:22363	
K04525	SERPINA		PF00079.25	GO:000759	extracellula	NP_659083	mmu:217847	
K12668	DAD1, OS	mmu04141(	PF02109.21	GO:000182	extracellula	NP_001106	mmu:13135	
Microtubule associated monoxygena			PF12510.13	GO:004593	cytoplasmic	NP_001271	mmu:29856	
K19346	SYNE2	mmu04820(	PF00307.36	GO:009951	cytoplasmic	NP_001005	mmu:319565	
K18440	ARAP2	mmu04144(	PF00169.35	GO:000367	cytoplasmic	NP_848494	mmu:212285	
K07934	IFT27, RAYL, RABL4		PF00071.28	GO:000810	cytoplasmic	NP_080207	mmu:67042	
K02913	RP-L33, MI	mmu03010(	PF00471.26	GO:001593	ER	BAC31060.	mmu:66845	
K23706	SASH3		PF12485.13	GO:001922	cytoplasmic	NP_083049	mmu:74131	
K25175	ZFP91		PF00096.32	GO:001922	nuclear	NP_443735	mmu:10991	zf-C2H2
K13958	KLHL36		PF01344.31	GO:004423	cytoplasmic	BAD21385.	mmu:23479	ZBTB
K12948	SPCS3, SPC	mmu03060(	PF04573.17	GO:000578	plasma mer	NP_001070	mmu:76687	
K01206	FUCA	mmu04142(	PF01120.23	GO:001613	lysosomal	NP_001317	mmu:66848	
K10644	CHFR		PF17979.7(z	GO:003243	cytoplasmic	EDL20041.	mmu:231600	
K04079	HSP90A, ht	mmu04915(	PF00183.23	GO:009771	cytoplasmic	AAH49951.	mmu:15516	
K06585	ITGA9	mmu05165(	PF08441.18	GO:004590	plasma mer	NP_598482	mmu:104099	
K14007	SEC24	mmu04141(	PF04811.21	GO:000810	cytoplasmic	NP_997092	mmu:99683	
ZQD4(Family with sequence similar			PF15914.10(FAM193_C		nuclear	NP_001230	mmu:231128	
158A(chromosome 6 open reading fr			PF15576.11(DUF4661:I		cytoplasmic	NP_258438	mmu:114585	
K00139	ALDH5A1	mmu00650(	PF00171.27	GO:001649	mitochondr	NP_766120	mmu:214579	
K19413	KDM1B, AOF1, LSD2		PF01593.30	GO:008009	cytoplasmic	NP_758466	mmu:218214	
K20362	YIF1		PF03878.20	GO:002260	mitochondr	NP_084163	mmu:77254	
K24644	PRDM11		PF21549.2(I	GO:004592	nuclear	XP_030101	mmu:100042784	
K23488	RAPH1		PF21989.2(I	GO:004867	cytoplasmic	NP_001038	mmu:77300	
K26543	ZNF385A, I	mmu04115(	PF12874.13	GO:000827	nuclear	XP_006521	mmu:29813	
K17509	PDPR		PF01266.30	GO:000573	mitochondr	NP_938050	mmu:319518	
K11661	SRCAP, SW	mmu03082(	PF00176.29	GO:004239	cytoplasmic	NP_001291	mmu:100043597	
YKPT(G kinase anchoring protein 1)				GO:000548	nuclear	NP_062806	mmu:56278	
K07930	RAB43		PF00071.28	GO:000548	cytoplasmic	NP_001034	mmu:69834	
K16064	PIAS3	mmu04120(	PF14324.12	GO:008009	mitochondr	EDL38913.	mmu:22961	zf-MIZ
K14026	SEL1, SEL1	mmu04141(	PF08238.18	GO:000810	ER	BAD90512.	mmu:20338	
K20057	SYNJ2BP, OMP25		PF00595.29	GO:003361	cytoplasmic	NP_001296	mmu:105940408	
K09995	NKX3-2, BAPX1		PF00046.35	GO:004864	nuclear	NP_031550	mmu:12020	Homeobox
K01044	CES1	mmu00983(	PF00135.34	GO:000581	extracellula	NP_001074	mmu:382044	
K15430	TRM11, TRMT11		PF01170.24	GO:000945	cytoplasmic	NP_082880	mmu:73681	

K07767	KATNA1		PF00004.35	GO:000810	cytoplasmic	NP_035965	mmu:23924	
K26092	HJURP		PF12346.13	GO:003408	nuclear	NP_941054	mmu:381280	
K25091	RBPMS		PF00076.28	GO:009715	cytoplasmic	BAC37585.1	mmu:19663	
K02183	CALM	mmu04750(	PF13499.12	GO:004316	cytoplasmic	AAI45380.1	mmu:80796	
K01875	SARS, serS	mmu00970(	PF00587.30	GO:008009	cytoplasmic	EDL01968.1	mmu:20226	
K12561	MARCKS	mmu04666(	PF02063.22	GO:003243	cytoplasmic	CAA42018.1	mmu:17118	
K17583	NOM1		PF02854.25	GO:002240	nuclear	NP_001028	mmu:433864	
K12041	SLC9A6_7,	mmu04260(	PF00999.27	GO:004867	extracellulo	NP_766368	mmu:236794	
K11125	SMG5, EST	mmu03015(	PF10373.15	GO:003304	nuclear	NP_839977	mmu:229512	
K06252	TN	mmu05165(	PF00041.27	GO:004858	plasma mer	CAE45651.1	mmu:329278	
.7PH(Lymphocyte antigen 6 comple			PF00021.27	GO:000989	extracellulo	NP_001092	mmu:100041546	
K24481	CRLF1		PF00041.27	GO:000512	mitochondr	NP_061297	mmu:12931	
K04371	ERK, MAP	mmu05208(	PF00069.31	GO:005104	cytoplasmic	NP_036082	mmu:26417	
K27496	FLII		PF00626.27	GO:000806	cytoplasmic	NP_071292	mmu:14248	
Testis expressed 2)			PF13465.12	GO:008009	nuclear	NP_808407	mmu:66850	zf-C2H2
K04574	NEFH, NF	mmu05014(	PF07142.17	GO:009951	cytoplasmic	NP_035034	mmu:380684	
K16307	CDC42BP		PF00780.28	GO:000591	nuclear	AAI38036.1	mmu:217866	
K10429	MAP1	mmu03250(	PF23415.1(	GO:004867	nuclear	AAI38034.1	mmu:17755	
K23081	CASTOR2	mmu04150(	PF21389.3(	GO:190343	cytoplasmic	NP_109644	mmu:80909	
K00948	PRPS, prsA	mmu00030(	PF13793.12	GO:004353	cytoplasmic	NP_080938	mmu:110639	
K06237	COL4A	mmu05165(	PF01391.23	GO:003250	extracellulo	NP_034062	mmu:12827	
K06721	CLEC10A, CLECSF13,		PF03954.19	GO:000989	plasma mer	NP_001191	mmu:17312	
K02984	RP-S3Ae, R	mmu05171(	PF01015.23	GO:002262	cytoplasmic	NP_058655	mmu:20091	
K11434	PRMT1	mmu04922(	PF22528.1(I	GO:008009	cytoplasmic	CAH745637	mmu:15469	
K13783	SLC37A1_2			GO:001576	ER	NP_001229	mmu:224674	
K23901	CALU		PF13499.12	GO:001989	ER	NP_031620	mmu:12321	
K00498	CYP11A	mmu04913(	PF00067.27	GO:000449	cytoplasmic	NP_062753	mmu:13070	
K26270	MBTD1		PF02820.24(MBT:mbt r		cytoplasmic	XP_006531	mmu:103537	
K15291	RIMS1, RIM	mmu04721(	PF00168.36(C2:C2 dom		nuclear	NP_444500	mmu:116837	
K06855	MAPK4_6	mmu04657(	PF00069.31	GO:003563	cytoplasmic	NP_766220	mmu:225724	
K04459	DUSP, MK	mmu04148(	PF00782.25	GO:001679	nuclear	AAI18942.1	mmu:319520	
K19832	NOA1		PF21516.2(	GO:002260	mitochondr	NP_062810	mmu:56412	
K00418	QCR8, UQ	mmu05208(	PF02939.21	GO:004866	mitochondr	NP_001313	mmu:22272	
K01867	WARS, trpS	mmu00970(	PF00579.31	GO:000481	mitochondr	NP_081738	mmu:70560	
K06505	CD74, DHI	mmu04612(	PF09307.15	GO:200123	cytoplasmic	NP_034675	mmu:16149	
ZTHW(tankyrase 1 binding protein			PF15327.11	GO:000697	nuclear	AAI50948.1	mmu:228140	
K01057	PGLS, pgl,	mmu00030(	PF01182.26	GO:000548	cytoplasmic	NP_079672	mmu:66171	
K26678	MON2		PF16206.10	GO:000810	cytoplasmic	NP_001156	mmu:67074	
K23507	MIEF1_2		PF21297.3(	GO:007013	cytoplasmic	NP_001344	mmu:239555	
K21421	NOX2, GP	mmu04670(	PF08030.17	GO:005123	ER	NP_031833	mmu:13058	
K05901	BLVRB	mmu00740(	PF13460.12	GO:004858	cytoplasmic	NP_659172	mmu:233016	
K18668	ZC3H12, MCPIP		PF11977.14	GO:003577	cytoplasmic	EDL30323.1	mmu:230738	
K24851	ZNF142		PF23611.1(z	GO:008009	nuclear	BAC41402.1	mmu:77264	zf-C2H2

K12828	SF3B1, SAP	mmu03040(	PF08920.15	GO:002260	cytoplasmic	NP_001252	mmu:81898	
K11342	MORF4L2,	mmu03082(	PF05712.18	GO:008009	nuclear	BAE31756.	mmu:56397	
K12655	OTUD5, D	mmu04622(	PF02338.25	GO:001922	nuclear	NP_001277	mmu:54644	
K06497	CD63, MLA	mmu03272(	PF00335.26	GO:000810	extracellulo	NP_001036	mmu:12512	
K17576	PPP1R37, LRRC68		PF13516.12	GO:005112	cytoplasmic	NP_954600	mmu:232947	
ZGQ(Golgi membrane protein 1)				GO:000699	Golgi appa	BAE39697.	mmu:105348	
K02915	RP-L34e, R	mmu05171(	PF01199.23	GO:002262	mitochondr	NP_001005	mmu:619547	
K20064	GRB10	mmu04150(	PF08947.15	GO:001922	cytoplasmic	NP_001357	mmu:14783	
K07375	TUBB	mmu05020(	PF00091.31	GO:003111	cytoplasmic	NP_001029	mmu:22153	
K10352	MYH9s	mmu04530(	PF01576.24	GO:003243	cytoplasmic	NP_001258	mmu:71960	
K18165	TMEM126B		PF07114.16	GO:002260	cytoplasmic	NP_081010	mmu:68472	
K04530	APBB2, FE65L		PF00640.29	GO:000578	cytoplasmic	NP_001188	mmu:11787	
Y09K(GC-rich promoter binding pr			PF15337.11	GO:003277	nuclear	NP_001116	mmu:73274	TF_others
K15538	MANEA		PF16317.11	GO:001678	Golgi appa	NP_766453	mmu:242362	
K12567	TTN	mmu05410(	PF07679.22	GO:004864	nuclear	A2ASS6.1(R	mmu:22138	
K13343	PEX14	mmu04146(	PF04695.18	GO:000810	cytoplasmic	NP_062755	mmu:56273	
K19947	MICAL		PF12130.13	GO:000449	cytoplasmic	Q8BML1.2(	mmu:320878	
K11451	CBX2	mmu03083(	PF00385.30	GO:004239	nuclear	NP_031649	mmu:12416	
234V(zinc finger protein)			PF04419.19	GO:000367	cytoplasmic	XP_056710	mmu:68036	
K01886	QARS, glnS	mmu00970(	PF00749.27	GO:200123	cytoplasmic	NP_598555	mmu:97541	
K00722	GBGT1	mmu00603(	PF03414.19	GO:190350	Golgi appa	NP_631936	mmu:227671	
K03172	TRAF1	mmu05200(	PF21355.3(	GO:000512	cytoplasmic	NP_001313	mmu:22029	
K03173	TRAF2	mmu05022(	PF21355.3(	GO:200123	cytoplasmic	NP_033448	mmu:22030	
K19874	SEPN1			GO:004858	mitochondr	NP_083376	mmu:74777	
K24063	SNTA	mmu05410(	PF23012.2(	GO:008009	mitochondr	NP_033254	mmu:20648	
K13350	PXMP4, PM	mmu04146(	Peroxisome	GO:004423	mitochondr	NP_001405	mmu:59038	
K01292	CPN1		PF00246.29	GO:005138	ER	NP_109628	mmu:93721	
K01596	E4.1.1.32, p	mmu04910(	PF00821.23	GO:009008	mitochondr	BAE41656.	mmu:74551	
K09446	IRF5	mmu04620(	PF10401.14	GO:001922	mitochondr	ACA81530.	mmu:27056	IRF
K09275	TFCP2		PF04516.20	GO:000097	nuclear	NP_001076	mmu:22221	CP2
K08125	PRELP		PF13855.12	GO:003101	extracellulo	NP_473418	mmu:116847	
K10356	MYO1	mmu04814(	PF00063.27	GO:009951	cytoplasmic	NP_851417	mmu:71602	
K07565	NIP7		PF17833.7(	GO:002260	cytoplasmic	NP_079667	mmu:66164	
K16620	BMP6	mmu04390(	PF00688.24	GO:003202	extracellulo	EDL40936.	mmu:12161	
K06464	ITGB2, CD	mmu04670(	PF00362.24	GO:000690	plasma mer	P11835.2(R	mmu:16414	
K14676	NTE, NRE	mmu00564(	PF24179.1(	GO:000581	ER	NP_666363	mmu:241274	
K06625	CDKN1A,	mmu05200(	PF02234.24	GO:005116	cytoplasmic	NP_001104	mmu:12575	
K01277	DPP3		PF03571.21	GO:000417	cytoplasmic	NP_001347	mmu:75221	
K10799	TNKS		PF12796.13	GO:000810	cytoplasmic	EDL35446.	mmu:21951	
K00682	GGCT	mmu00480(	PF13772.11	GO:000367	cytoplasmic	NP_080913	mmu:110175	
K17275	PLS1		PF00307.36	GO:003243	cytoplasmic	NP_001028	mmu:102502	
K19197	ING1		PF12998.12	GO:005116	cytoplasmic	NP_001289	mmu:26356	
K06814	CDH24		PF00028.23	GO:002260	cytoplasmic	NP_955764	mmu:239096	

K01892	HARS, hisS	mmu00970(	PF13393.11	GO:004280	mitochondr	NP_542367	mmu:70791	
K00693	GYS	mmu01100(	PF05693.19	GO:000548	cytoplasmic	CAH74575	mmu:14936	
G04(Follistatin-like)			PF03172.18	GO:008009	nuclear	NP_796029	mmu:319997	
K18412	TNRC6, GW182		PF16608.10(TNRC6-PA		cytoplasmic	EDL17293.	mmu:233833	
K09660	MPDU1		PF04193.19	GO:004423	Golgi appa	BAC30593.	mmu:24070	
K11294	NCL, NSR1		PF00076.28	GO:004213	nuclear	NP_035010	mmu:17975	
GATA zinc finger domain containin			PF13855.12	GO:002260	cytoplasmic	NP_666276	mmu:231798	
K00311	ETFDH		PF05187.19	GO:003444	mitochondr	NP_080070	mmu:66841	
K23507	MIEF1_2		PF21297.3(	GO:001602	cytoplasmic	NP_001009	mmu:237781	
K19600	TUB, TULP		PF01167.24	GO:000858	nuclear	NP_035787	mmu:22158	Tub
K18807	TLR12		PF13855.12	GO:003802	plasma mer	NP_991392	mmu:384059	
K10632	BRAP	mmu04014(	PF07576.18	GO:000827	cytoplasmic	NP_082503	mmu:72399	
XSE0(Major Facilitator superfamily			PF07690.21	GO:009859	ER	NP_001343	mmu:68294	
K17274	S100A10	mmu05132(	PF01023.25	GO:000810	cytoplasmic	NP_033138	mmu:20194	
K04630	GNAI	mmu04730(	PF00503.26	GO:200123	cytoplasmic	NP_001397	mmu:14678	
K04525	SERPINA		PF00079.25	GO:000486	extracellulo	NP_032484	mmu:16625	
K10903	HUS1	mmu04218(	PF04005.17	GO:001922	mitochondr	NP_001290	mmu:15574	
K13201	TIA1, TIAL1		PF00076.28	GO:000810	cytoplasmic	NP_035715	mmu:21841	
K05730	VAV	mmu04664(	PF00621.25	GO:000690	cytoplasmic	NP_065251	mmu:57257	
K05508	CX3CL1, N	mmu05163(Human cyto		GO:200123	extracellulo	BAC30544.	mmu:20312	
K19241	ELMO3	mmu05100(	PF11841.14	GO:000690	cytoplasmic	XP_006530	mmu:234683	
K01045	PON		PF01731.26	GO:004643	extracellulo	NP_035264	mmu:18979	
K02327	POLD1	mmu03440(	PF00136.26	GO:004423	nuclear	NP_035261	mmu:18971	
K03027	RPAC1, RP	mmu04623(	PF01193.29	GO:000367	nuclear	NP_033111	mmu:20016	
K14303	NUP160, N	mmu03013(	PF23347.1(	GO:001593	cytoplasmic	NP_067487	mmu:59015	
K21634	USP21	mmu04217(	PF00443.35	GO:000989	cytoplasmic	EDL39107.	mmu:30941	
K13101	GPKOW		PF25088.1(	GO:009030	nuclear	NP_776108	mmu:209416	
K26434	LRRFIP		PF09738.14	GO:008009	nuclear	NP_001104	mmu:16978	LRRFIP
K04738	CSF2RB, IL	mmu05200(	PF21460.3(	GO:000989	extracellulo	NP_001274	mmu:12984	
K03036	PSMD11, R	mmu05020(	PF18055.7(	GO:002260	nuclear	BAE22795.	mmu:69077	
K14736	TF	mmu04978(	PF00405.22	GO:000367	ER	NP_082194	mmu:71775	
K07899	RAB9A, RA	mmu05132(	PF00071.28	GO:000810	cytoplasmic	NP_062747	mmu:56382	
K04703	SPRED		PF05210.18	GO:001922	nuclear	NP_277059	mmu:114715	
K04703	SPRED		PF05210.18	GO:001922	cytoplasmic	NP_277058	mmu:114716	
K06109	RAB13	mmu04530(	PF00071.28	GO:000810	cytoplasmic	BAF02870.1	mmu:68328	
K03131	TAF6	mmu03022(	PF07571.19	GO:008009	nuclear	EDL19209.	mmu:21343	
K12366	ELMO1, CI	mmu04148(	PF11841.14	GO:000548	cytoplasmic	NP_001382	mmu:140580	
K11420	EHMT	mmu01100(	PF12796.13	GO:000647	nuclear	NP_766133	mmu:77683	
2BI7(chromosome X open reading fr			PF15440.11	GO:008009	nuclear	NP_001028	mmu:382252	
K24822	LOXHD1		PF01477.28	GO:003242	cytoplasmic	BAC26759.	mmu:240411	
K20780	MDC1		PF16589.12	GO:006026	nuclear	NP_001010	mmu:240087	
K16904	DCTPP1	mmu01100(	PF12643.12	GO:000914	cytoplasmic	NP_075692	mmu:66422	
K14772	UTP20		PF07539.18	GO:003204	nuclear	NP_780367	mmu:70683	

K01312	PRSS1_2_3	mmu05164(	PF00089.32	GO:000425	extracellula	NP_001034	mmu:436522	
K03248	EIF3G		PF12353.13	GO:002260	cytoplasmic	BAE40297.	mmu:53356	
K14589	CMTR1, FTSJD2, MTR		PF01728.25	GO:000815	nuclear	NP_083067	mmu:74157	
K08707	DNTTIP1		PF21229.3(	GO:003149	nuclear	NP_598524	mmu:76233	
K03881	ND4	mmu05208(	PF00361.26	GO:190260	extracellula	AGK29656.	mmu:17719	
K03879	ND2	mmu05208(	PF00361.26	GO:000906	extracellula	AHG32085.	mmu:17717	
K27275	ZNF217		PF00096.32	GO:009039	nuclear	NP_001028	mmu:22891	zf-C2H2
K23334	RANBP9_10, RANBPM		PF00622.36	GO:000989	cytoplasmic	NP_064314	mmu:56705	
KQMT(Transmembrane protein 19)			PF01940.21	GO:000557	plasma mer	NP_001346	mmu:67226	
K04453	MAX	mmu05200(Pathways ir		GO:008009	nuclear	NP_001408	mmu:17187	
K02838	frr, MRRF, RRF		PF01765.24	GO:190300	mitochondr	NP_080698	mmu:67871	
K18266	NDRG1		PF03096.20	GO:003228	cytoplasmic	AAI71968.1	mmu:17988	
ha domain-containing protein)			PF00498.31	GO:004239	cytoplasmic	NP_001334	mmu:83997	
K06564	IGF2R, CD	mmu04142(	PF00878.24	GO:003197	plasma mer	AAI71973.1	mmu:16004	
Heterogeneous nuclear ribonucleopr			PF13465.12	GO:008009	nuclear	NP_001038	mmu:22701	zf-C2H2
K21415	ANKS6		PF12796.13(Ank_2:Ank		cytoplasmic	XP_006538	mmu:75691	
K15149	MED9		PF07544.18	GO:006026	nuclear	NP_619616	mmu:192191	
K08023	LTBP2_3_4		PF07645.21	GO:002260	extracellula	NP_001107	mmu:108075	
K20825	FAM20B		PF06702.18	GO:003563	ER	NP_001404	mmu:215015	
K17305	APOH, B2(	mmu04979(	PF00084.26	GO:003435	extracellula	NP_038503	mmu:11818	
RRF(immunoglobulin heavy variab			PF07686.23	GO:000237	cytoplasmic	CAA43096.1(E8 variable light chair		
K14555	UTP13, TBl	mmu03008(	PF00400.38	GO:003204	cytoplasmic	NP_663371	mmu:213773	
K11840	USP9_24		PF25010.1(	GO:010100	nuclear	BAC33657.	mmu:22284	
K00084	CBR3	mmu00980(	PF00106.31	GO:000403	mitochondr	NP_766635	mmu:109857	
K22038	LRRC8		PF12534.13	GO:009008	peroxisoma	NP_808393	mmu:241296	
K22403	ZMIZ		PF18028.7(	GO:008009	cytoplasmic	NP_899031	mmu:32836	zf-MIZ
K26076	ZRANB2		PF00641.23	GO:004322	nuclear	EQB78884.	mmu:53861	
K06675	SMC4		PF02463.25	GO:190136	cytoplasmic	NP_598547	mmu:70099	
K09313	CUTL		PF08172.17	GO:004299	nuclear	NP_001392	mmu:13047	
K06238	COL6A	mmu05165(	PF00092.34	GO:000367	extracellula	NP_666119	mmu:12834	
K17261	CAP1_2, SRV2		PF08603.16	GO:001989	cytoplasmic	EDL41026.	mmu:67252	
K09528	DNAJC8		PF00226.36	GO:000557	nuclear	NP_001013	mmu:68598	
K18448	BNIP2		PF12496.13	GO:003250	cytoplasmic	NP_001361	mmu:12175	
K17808	ZIM17, DNLZ, Tim15		PF05180.17	GO:000810	mitochondr	NP_081104	mmu:52838	
K12347	SLC11A1, N	mmu04142(	PF01566.24	GO:000690	cytoplasmic	NP_038640	mmu:18173	
K16528	AKAP12		PF03832.18	GO:001922	cytoplasmic	EDL03592.	mmu:83397	
K06495	SELL, CD6	mmu04514(	PF00059.27	GO:000989	plasma mer	XP_006496	mmu:20343	
K24885	HMBOX1		PF04814.19	GO:005109	cytoplasmic	KAI254949	mmu:21915	Homeobox
K11703	GLT25D	mmu00514(	PF01755.23	GO:000578	ER	NP_666323	mmu:234407	
F18(Scavenger receptor class A, me			PF00530.23	GO:004308	cytoplasmic	NP_083179	mmu:71145	
K18621	ANLN		PF16018.10	GO:000548	cytoplasmic	NP_082666	mmu:68743	
K06975	K06975		PF14542.12(Acetyltrans		cytoplasmic	NP_001164	mmu:24083	
K01511	ENTPD5_6	mmu01100(	PF01150.23	GO:000578	cytoplasmic	EDL02795.	mmu:12499	

K06553	VPREB, CD179a		PF07686.23	GO:000548	ER	DBA12481.	mmu:22363	
K17301	COPB1, SEC26		PF01602.26	GO:000810	cytoplasmic	NP_203534	mmu:70349	
K12823	DDX5, DB	mmu05202(	PF00270.35	GO:005116	nuclear	AAI29874.1	mmu:13207	
K20719	SIGMAR1	mmu05014(	PF04622.18	GO:000578	ER	NP_001273	mmu:18391	
K24067	TAX1BP3		PF00595.29	GO:000810	cytoplasmic	NP_083840	mmu:76281	
K20195	MON1	mmu04137(	PF19036.6(I	GO:000810	cytoplasmic	NP_001041	mmu:270096	
K08872	RIOK3, SUDD		PF01163.29	GO:001922	cytoplasmic	BAE42091.1	mmu:66878	
K21952	CASKIN, ANKS5		PF12796.13	GO:000573	cytoplasmic	NP_542374	mmu:140721	
WNE(chromosome 14 open reading			PF14969.11	GO:004323	cytoplasmic	AAH11283.	mmu:58248	
K07466	RFA1, RPA	mmu03440(	PF08646.16	GO:000810	cytoplasmic	NP_080929	mmu:68275	
MFZ(O-acyl-ADP-ribose deacylase			PF01661.27	GO:001679	cytoplasmic	XP_021042	mmu:106821	
K24022	PLEKHO		PF00169.35	GO:002260	cytoplasmic	NP_075809	mmu:67220	
K11268	ESCO, ECO	mmu04110(	PF13880.12	GO:006173	cytoplasmic	EDL22997.	mmu:77805	
K08869	ADCK, ABC1		PF03109.22	GO:001631	mitochondr	NP_766548	mmu:268822	
K14525	RPP25	mmu03008(	PF01918.26	GO:009030	nuclear	NP_081554	mmu:69961	
K10168	TLR5	mmu04620(	PF13855.12	GO:000514	plasma mer	XP_017177	mmu:53791	
K10744	RNASEH2I	mmu03030(	PF09468.16	GO:001922	cytoplasmic	NP_080277	mmu:67153	
K07179	RIOK2	mmu03008(	PF01163.29	GO:003304	cytoplasmic	NP_080210	mmu:67045	
K03454	SLC25A46		PF00153.32	GO:002260	cytoplasmic	NP_080441	mmu:67453	
ZVPB(fin bud initiation factor hom			PF15819.10	GO:000548	ER	NP_080547	mmu:67606	
K06669	SMC3, CSP	mmu04114(	PF02463.25	GO:008009	cytoplasmic	NP_031816	mmu:13006	
K10789	MPO	mmu04613(	PF03098.20	GO:000214	lysosomal	NP_034954	mmu:17523	
K16585	HAUS2, AUG2		PF15003.11	GO:002260	cytoplasmic	NP_079751	mmu:66296	
K09481	SEC61B, SE	mmu04141(	PF03911.21	GO:000578	ER	NP_077133	mmu:66212	
K04857	CACNA1S,	mmu05020(	PF00520.37	GO:000526	ER	NP_001389	mmu:12292	
K03951	NDUFA7	mmu05208(	PF07347.17	GO:000925	mitochondr	NP_075691	mmu:66416	
K17267	COPG		PF01602.26	GO:000810	cytoplasmic	NP_059506	mmu:54160	
K00746	CSGALNA	mmu00532(	PF05679.22	GO:003020	ER	BAC33912.1	mmu:234356	
K05771	NR3C1, GR	mmu04080(	PF02155.20	GO:005080	nuclear	NP_001348	mmu:14815	ESR-like
K12821	PRPF40, PF	mmu03040(	PF01846.25	GO:007100	cytoplasmic	NP_061256	mmu:54614	
K18032	PTPRA		PF00102.32	GO:000548	plasma mer	NP_001157	mmu:19262	
K08875	NRBP		PF00069.31	GO:003563	cytoplasmic	NP_001345	mmu:223649	
K11835	USP4_11, UBP12		PF00443.35	GO:000417	cytoplasmic	NP_663603	mmu:236733	
K08059	IFI30, GIL1	mmu04612(	PF03227.22	GO:000032	extracellula	NP_075552	mmu:65972	
K20410	MAPKAP1	mmu04150(	PF16978.11	GO:001922	nuclear	NP_001277	mmu:227743	
K13133	GEMIN5		PF23777.1(	GO:002260	nuclear	NP_001160	mmu:216766	
K09555	BAG1	mmu04141(	PF00240.29	GO:000810	cytoplasmic	NP_033866	mmu:12017	
K03875	SKP2, FBX	mmu04120(	PF12937.12	GO:008009	cytoplasmic	NP_001272	mmu:27401	
K18157	TMEM126A		PF07114.16	GO:002260	cytoplasmic	NP_079736	mmu:66271	
K11097	SNRPE, SM	mmu03040(	PF01423.28	GO:002260	nuclear	NP_001076	mmu:20643	
K00006	GPD1	mmu00564(	PF01210.29	GO:001922	cytoplasmic	NP_034401	mmu:14555	
K12180	COPS7, CSN7		PF18392.6(	GO:002260	cytoplasmic	NP_001342	mmu:26894	
K23542	TMEM199		PF11712.13	GO:000578	mitochondr	NP_954669	mmu:195040	

K18061	SBF1_2, MTMR5_13		PF12335.14	GO:000554	cytoplasmic	NP_796298	mmu:319934	
K04491	TCF7L2	mmu05165(	PF08347.16	GO:009008	nuclear	NP_001136	mmu:21416	HMG
K11855	USP36_42		PF00443.35	GO:000989	nuclear	NP_001028	mmu:72344	
K24888	GTPBP2		PF00009.33	GO:001707	cytoplasmic	KAF636451	mmu:56055	
K12897	TRA2	mmu04936(	PF00076.28	GO:008009	nuclear	NP_001230	mmu:20462	
K01195	uidA, GUSI	mmu01240(	PF02836.23	GO:003020	lysosomal	NP_034498	mmu:110006	
K14688	SLC30A1, Z	mmu04978(	PF01545.26	GO:005080	extracellulo	NP_033605	mmu:22782	
K10698	UBE1L, UB	mmu04120(	PF00899.27	GO:003202	cytoplasmic	BAC40806.	mmu:74153	
K13704	ABHD12		PF12146.15	GO:000962	Golgi appa	NP_077785	mmu:76192	
K03232	EEF1B		PF00736.24	GO:000815	cytoplasmic	KAF645007	mmu:55949	
K12150	HINT3		PF11969.13	GO:004322	cytoplasmic	NP_080074	mmu:66847	
K11884	PNO1, DIM2		PF22891.2(I	GO:003204	cytoplasmic	NP_079719	mmu:66249	
K13190	RBM15		PF00076.28	GO:005116	nuclear	NP_780611	mmu:109095	
K16196	EIF2AK4	mmu04140(	PF00069.31	GO:002262	cytoplasmic	NP_038747	mmu:27103	
K06448	CD1	mmu04530(	PF16497.10	GO:001922	lysosomal	NP_001396	mmu:12479	
K01807	rpiA	mmu00030(	PF06026.20	GO:001932	mitochondr	NP_033101	mmu:19895	
K02920	RP-L36e, R	mmu05171(	PF01158.23	GO:004423	cytoplasmic	NP_001125	mmu:54217	
FGJ(family	with sequence similarit		PF05308.16	GO:000699	cytoplasmic	NP_001243	mmu:76824	
K02932	RP-L5e, RP	mmu05171(	PF17144.9(I	GO:001710	cytoplasmic	NP_058676	mmu:100503670	
K02873	RP-L13e, R	mmu05171(	PF01294.23	GO:001593	cytoplasmic	NP_058018	mmu:270106	
K17617	CTDNEP1, DULLARD		PF03031.24	GO:000810	ER	NP_001093	mmu:67181	
KSIZ(50S ribosomal protein l17)			PF05004.19	GO:000989	cytoplasmic	NP_080179	mmu:15983	
K05074	IL15RA, CI	mmu04672(	Intestinal ir	GO:001922	extracellulo	NP_032384	mmu:16169	
K09037	MAFF_G_K		PF03131.22	GO:005125	nuclear	BAE32212.	mmu:17135	
K23958	INF2	mmu04820(	PF02181.28	GO:002260	cytoplasmic	NP_940803	mmu:70435	
K08789	MAST, IREH1		PF08926.16	GO:004316	cytoplasmic	NP_001036	mmu:17776	
K07420	CYP2S1	mmu00830(	PF00067.27	GO:000449	ER	NP_083051	mmu:74134	
K08121	FMOD	mmu04350(	PF13855.12	GO:000561	extracellulo	NP_067330	mmu:14264	
K05768	GSN	mmu04810(	PF00626.27	GO:003288	cytoplasmic	BAE40549.	mmu:227753	
K04527	INSR, CD2	mmu04923(	PF07714.23	GO:008009	plasma mer	NP_001316	mmu:16337	
K20292	COG5		PF20649.3((	GO:000810	cytoplasmic	EDL36903.	mmu:238123	
K01301	NAALAD		PF04253.20	GO:001602	plasma mer	NP_001344	mmu:72560	
K20300	TRAPPC1, BET5		PF04099.18	GO:004820	cytoplasmic	NP_001019	mmu:245828	
K03927	CES2	mmu00983(	PF00135.34	GO:001678	ER	NP_932116	mmu:72361	
K03111	ssb	mmu03440(	PF00436.31	GO:003204	mitochondr	NP_001273	mmu:381760	
K13114	PNN	mmu03013(	PF04697.18	GO:003514	nuclear	O35691.4(R	mmu:18949	
K14846	RPF1		PF04427.23	GO:004213	nuclear	NP_081647	mmu:70285	
K04427	MAP3K7, T	mmu05170(	PF07714.23	GO:200123	cytoplasmic	BAE34955.	mmu:26409	
K12401	AP4B1	mmu04142(	PF01602.26	GO:000810	cytoplasmic	AAH56200.	mmu:67489	
K02127	ATPeF0B, /	mmu05208(	PF05405.19	GO:000526	mitochondr	BAE39561.	mmu:11950	
K10147	PPM1D, W	mmu04115(	PF00481.26	GO:001679	nuclear	BAE42627.	mmu:53892	
K19867	PDLIM5_6	mmu04820(	PF00412.28	GO:003243	cytoplasmic	XP_021035	mmu:67399	
K19022	AP5B1		PF21588.3(/	GO:001602	cytoplasmic	NP_001028	mmu:381201	

K16781	TTC8, BBS8		PF07719.23	GO:000810	cytoplasmic	NP_938053	mmu:76260
K09258	BCL3	mmu04625(	PF12796.13	GO:005116	cytoplasmic	AAC79694.	mmu:12051
Methylates the class 1 translation te			PF00107.32	GO:004323	peroxisoma	NP_598440	mmu:66609
K06708	PDCD1LG	mmu04514(	PF13927.12	GO:004213	ER	NP_067371	mmu:58205
K04559	HIP1	mmu05016(	PF07651.22	GO:005104	nuclear	NP_666113	mmu:215114
K19801	PI4KB	mmu04070(	PF00454.33	GO:008009	cytoplasmic	XP_032753	mmu:107650
XBR(myc target 1)			PF15179.11	GO:000557	plasma mer	NP_081069	mmu:68632
K14849	RRP1		PF05997.17	GO:003068	nuclear	NP_035055	mmu:18114
K14960	CXXC1, SPP1, CPS40		PF12269.13	GO:004239	nuclear	BAE27361.	mmu:74322
K27758	GIMAP1_3_5		PF04548.22	GO:000578	mitochondr	P70224.3(R	mmu:16205
K04960	ITPR3	mmu04750(	PF08709.17	GO:000526	cytoplasmic	NP_542120	mmu:16440
K11536	SLC28A		PF07662.18	GO:001593	plasma mer	NP_001343	mmu:269346
K18341	TBC1D1	mmu04152(	PF00566.25	GO:009008	nuclear	NP_001297	mmu:57915
K03171	TRADD	mmu05203(	PF09034.15	GO:001922	cytoplasmic	NP_001028	mmu:71609
V15(proline rich)			PF15386.11(Tantalus:D		nuclear	NP_919321	mmu:215476
K03353	APC6, CDC	mmu04110(	PF12895.13	GO:003304	cytoplasmic	NP_081552	mmu:69957
K15157	MED15		PF21538.2(	GO:006026	cytoplasmic	EDK97477.	mmu:94112
X4I(chromosome 19 open reading f			PF15391.11(DUF4614:D		cytoplasmic	XP_011240	mmu:72254
K08187	SLC16A10	mmu04974(	PF07690.21	GO:009859	cytoplasmic	NP_001107	mmu:72472
K24908	DIP2B		PF00501.34	GO:000548	cytoplasmic	NP_001152	mmu:239667
K03513	POLM	mmu03450(	PF14791.11	GO:003015	nuclear	AAH56213.	mmu:54125
K16141	ALB	mmu04918(	PF00273.25	GO:001564	extracellula	NP_033784	mmu:11657
K17514	CLEC6A, D	mmu04625(	PF00059.27	GO:000959	cytoplasmic	NP_001177	mmu:56620
YXPD(beta-1,4-N-acetyl-galactosam			PF10226.14	GO:003250	cytoplasmic	XP_006516	mmu:668158
K24485	FNDC3		PF00041.27	GO:004212	cytoplasmic	NP_001343	mmu:72007
K18262	EFEMP1, FBLN3		PF07645.21	GO:008009	extracellula	NP_666127	mmu:216616
K06677	YCS4, CNAP1, CAPD2		PF12717.13	GO:003304	cytoplasmic	NP_001404	mmu:68298
K04739	PRKAR	mmu04910(	PF00027.35	GO:001922	cytoplasmic	NP_032950	mmu:19087
K06553	VPREB, CD179a		PF07686.23(V-set:Immu		ER	DBA12442.	mmu:22363
K18709	TUT1		PF22600.2(	GO:000548	cytoplasmic	NP_932110	mmu:70044
K00645	fabD, MCA	mmu01212(	PF00698.26	GO:000663	mitochondr	NP_001025	mmu:223722
K23404	TAPT1		PF05346.16	GO:005113	cytoplasmic	NP_776125	mmu:231225
K00772	mtaP, MTA	mmu00270(	PF01048.26	GO:000367	cytoplasmic	NP_077753	mmu:66902
K14544	UTP22, NC	mmu03008(	PF17405.8(	GO:001593	nuclear	NP_631982	mmu:230082
K14864	FTSJ1, TRM7		PF01728.25	GO:001642	cytoplasmic	NP_598752	mmu:54632
K10892	FANCE	mmu03460(	PF11510.14	GO:009030	extracellula	NP_001157	mmu:72775
K04557	SEPT5, PN	mmu05022(	PF00735.24	GO:000802	cytoplasmic	EDL77948.	mmu:18951
K05842	TRIF	mmu05165(	PF17798.6(	GO:190187	cytoplasmic	NP_778154	mmu:106759
K13220	WBP4, FBP21		PF00397.32	GO:000613	nuclear	BAE35151.	mmu:22380
K14852	RRS1		PF04939.17	GO:003450	nuclear	BAC65489.	mmu:59014
K12893	SRSF4_5_6,	mmu03040(	PF00076.28	GO:008009	nuclear	KAI259487	mmu:67996
K14691	SLC30A4, ZNT4		PF01545.26	GO:001602	cytoplasmic	NP_001277	mmu:22785
CP6(Phosphatidylcholine transfer p			PF01852.25	GO:000554	cytoplasmic	P53808.2(R	mmu:18559

K18188	PET117		PF15786.10	GO:000699	mitochondr	NP_001158	mmu:100048644
K13183	DDX50		PF00270.35	GO:003359	cytoplasmic	AAH90996.	mmu:94213
K00558	DNMT1, dc	mmu00270(	PF01426.24	GO:008009	cytoplasmic	EDL25141.	mmu:13433
K04962	RYR2	mmu04972(	PF21119.3(I	GO:003202	cytoplasmic	NP_076357	mmu:20191
YD59(family with sequence similari			PF04103.21	GO:005069	ER	XP_006502	mmu:68521
K02431	fucU, FUOM		PF05025.18	GO:000548	extracellulo	EDL17903.	mmu:69064
K23802	LENG8, THP3		PF03399.22	GO:004323	nuclear	NP_001361	mmu:232798
K01331	C1S	mmu04610(	PF00089.32	GO:000425	lysosomal	NP_001091	mmu:50908
K15909	SHIP2, INP	mmu04910(	PF22669.2(I	GO:008009	cytoplasmic	NP_001116	mmu:16332
XR45(Osteoclast stimulating factor			PF13857.12	GO:000573	cytoplasmic	NP_059071	mmu:20409
K12385	NPC1	mmu04979(	PF22314.2(	GO:000810	lysosomal	NP_032746	mmu:18145
K02887	RP-L20, MI	mmu03010(	PF00453.23	GO:001593	mitochondr	NP_079846	mmu:66448
K02881	RP-L18, MI	mmu03010(	PF00861.27	GO:001593	mitochondr	NP_080586	mmu:67681
K02916	RP-L35, MI	mmu03010(	PF01632.24	GO:001593	mitochondr	NP_079706	mmu:66223
AYE(Morf4 family associated prote			PF15155.11	GO:004323	nuclear	NP_080518	mmu:67568
K21804	METTL21A		PF10294.15	GO:000647	cytoplasmic	NP_001355	mmu:67099
					extracellulo	NP_001242	mmu:432480
K26503	SKI	mmu04350(	PF02437.24	GO:003510	nuclear	NP_035515	mmu:20481
XRMJ(Midline 1 (Opitz BBB syndro			PF00069.31	GO:008009	cytoplasmic	NP_001100	mmu:227541
K11886	ECM29		PF13001.13	GO:002260	cytoplasmic	NP_001342	mmu:230249
K00626	ACAT, atoI	mmu00650(	PF00108.29	GO:003444	mitochondr	BAE37335.	mmu:110446
K24104	GPN		PF03029.23	GO:003563	cytoplasmic	NP_598517	mmu:74254
K23963	EPB41L5		PF00373.23	GO:004858	cytoplasmic	EDL39814.	mmu:226352
K23957	FMNL	mmu04820(	PF02181.28	GO:002260	cytoplasmic	NP_765997	mmu:71409
ZM6W(IGc1); ENOG4111XUQ(imn			PF07654.21	GO:000548	plasma mer	AAH96462.	mmu:228592
K24091	OBI1		PF13923.12	GO:008009	ER	NP_080323	mmu:72486
K10276	FBXL10_11	mmu03083(	PF16866.11	GO:008009	nuclear	XP_006530	mmu:30841
K11844	USP16_45	mmu03083(	PF00443.35	GO:004590	nuclear	XP_006523	mmu:74112
K11842	USP12_46		PF00443.35	GO:010100	cytoplasmic	NP_001128	mmu:69727
ZN43(family with sequence similarity 169, mem				GO:003109	nuclear	NP_001093	mmu:320557
XP37(Transmembrane amino acid tı			PF01490.24	GO:000557	plasma mer	EDL34468.	mmu:320534
K03783	punA, PNP	mmu01100(	PF01048.26	GO:000270	cytoplasmic	P23492.2(R	mmu:18950
F97(StAR-related lipid transfer (ST			PF01852.25	GO:012002	cytoplasmic	NP_598535	mmu:170459
YS8R(IGv); ENOG410YSH4(IGv); E			PF07686.23	GO:000237	cytoplasmic	AGN91462.1(immunoglobulin ligh	
K06553	VPREB, CD179a		PF07686.23	GO:000237	cytoplasmic	CAB46147.	mmu:22362
K25432	LRRC17		PF13855.12	GO:003250	mitochondr	NP_083253	mmu:74511
5I2(IGv); ENOG4111CF7(IGv); EN			PF07686.23(V-set:Immu		cytoplasmic	QVN47751.1(anti-Foot-and-mouth	
K00804	GGPS1	mmu00900(	PF00348.22	GO:000548	cytoplasmic	NP_001318	mmu:14593
K23001	TOR1AIP		PF05609.18	GO:003450	cytoplasmic	NP_001345	mmu:208263
K03350	APC3, CDC	mmu04110(	PF00515.34	GO:190304	cytoplasmic	NP_663411	mmu:217232
K19993	PLEK		PF00169.35	GO:009859	cytoplasmic	NP_038766	mmu:27260
K13102	KIN		PF10357.16	GO:004316	nuclear	NP_079556	mmu:16588
K13044	HNRNPABD		PF00076.28	GO:000962	nuclear	BAA09524.	mmu:11991

K19514	ATP6AP2, I	mmu04614(	PF07850.20	GO:004806	ER	NP_081715	mmu:70495	
K12750	MYL4	mmu04261(	PF13405.12	GO:000694	cytoplasmic	NP_001342	mmu:17896	
K07188	LIPE, HSL	mmu04910(	PF06350.17	GO:004213	nuclear	BAB31878.1	mmu:16890	
K15278	SLC35B4, YEA4		PF08449.17	GO:001593	ER	NP_067410	mmu:58246	
K08043	ADCY3	mmu04750(	PF00211.25	GO:000761	ER	NP_001153	mmu:104111	
K07588	MMAA, arg	mmu04980(	PF03308.22	GO:003563	mitochondr	NP_001350	mmu:109136	
K06793	VCAN, CSI	mmu04514(	PF00193.23	GO:004867	plasma mer	Q62059.2(R	mmu:13003	
K05767	IQGAP2_3	mmu04810(	PF00616.24	GO:003202	cytoplasmic	NP_001028	mmu:404710	
K03022	RPC8, POL	mmu04623(	PF08292.18	GO:001602	nuclear	XP_021580	mmu:78929	
K10582	UBE2Q	mmu04120(	PF00179.31	GO:000763	nuclear	NP_001099	mmu:70093	
K10742	DNA2	mmu03030(	PF08696.17	GO:008009	cytoplasmic	NP_796346	mmu:327762	
K09409	FOXP2_4		PF16159.10	GO:004859	nuclear	NP_001104	mmu:74123	Fork head
K00816	CCBL	mmu00270(	PF00155.27	GO:003017	cytoplasmic	NP_001343	mmu:70266	
K14376	PAP	mmu03015(	PF20750.3(I	GO:003563	nuclear	NP_766143	mmu:216578	
K10473	KBTBD5_10		PF01344.31	GO:003243	cytoplasmic	NP_082478	mmu:72330	ZBTB
K22985	GPR107		PF06814.19	GO:003013	Golgi appa	NP_848875	mmu:277463	
XQ5D(Repressor of RNA polymeras			PF02389.20	GO:003802	extracellulo	XP_036010	mmu:96875	
K00033	PGD, gnd, g	mmu00030(	PF00393.24	GO:001932	cytoplasmic	BAE29606.1	mmu:110208	
K24142	STARD10		PF01852.25	GO:000367	plasma mer	NP_001347	mmu:56018	
K15637	PGAM5	mmu04137(	PF00300.27	GO:000548	mitochondr	AAH21317.	mmu:72542	
K14838	NOP15		PF12196.13	GO:000079	nuclear	NP_080748	mmu:67949	
YPPD(Leucine rich repeat containing 42)					cytoplasmic	BAE25576.1	mmu:77809	
Z6HP(Complex 1 protein (LYR fami			PF05347.21(Complex1_		mitochondr	XP_055458940.1(MIEF1 upstream c		
K25078	RBM33			GO:199024	cytoplasmic	XP_006535	mmu:381626	
K12495	IQSEC	mmu04144(	PF01369.25	GO:000508	cytoplasmic	NP_001392	mmu:245666	
K15259	PARP7_11_12		PF18606.7(I	GO:008009	cytoplasmic	NP_001334	mmu:78781	
K04515	CAMK2	mmu04750(	PF00069.31	GO:000008	cytoplasmic	XP_004583	mmu:12325	
K05309	PTGES2	mmu00590(	PF13417.12	GO:008009	mitochondr	NP_598544	mmu:96979	
K03083	GSK3B	mmu04657(	PF00069.31	GO:200123	cytoplasmic	NP_001334	mmu:56637	
K19606	CRTAP		PF23557.1(T	GO:190187	ER	NP_064306	mmu:56693	
K24738	WDR7	mmu04142(	PF00400.38	GO:000573	cytoplasmic	NP_001014	mmu:104082	
K26417	RASIP1	mmu04520(	PF01843.25	GO:000548	cytoplasmic	AAI50767.1	mmu:69903	
K08802	SNRK		PF00069.31	GO:003563	cytoplasmic	AAI50743.1	mmu:20623	
K23857	PLEKHB		PF00169.35	GO:000554	mitochondr	NP_780630	mmu:226971	
K06553	VPREB, CD179a		PF07654.21	GO:000548	plasma mer	BAR42291.1	mmu:22363	
K17608	STRN1_3_4		PF00400.38	GO:005172	cytoplasmic	NP_001165	mmu:94186	
K22663	NLRP9, NALP9		PF05729.18	GO:005123	cytoplasmic	AAI41250.1	mmu:243874	
K14442	DHX36, RH	mmu03018(	PF07717.22	GO:004423	cytoplasmic	AAI38062.1	mmu:72162	
K06553	VPREB, CD179a		PF07686.23(V-set:Immu		plasma mer	CAB46319.1	mmu:22364	
K13886	CORO1B_1C_6		PF08953.17	GO:000548	cytoplasmic	NP_001355	mmu:216961	
ZJ3Y(translational machinery associ			PF09072.15(TMA7:Trar		cytoplasmic	XP_027399	mmu:66167	
K20279	SYNJ	mmu04070(	PF22669.2(I	GO:009951	mitochondr	NP_001106	mmu:20975	
K13567	ACVR1B, A	mmu04060(	PF00069.31	GO:003292	extracellulo	NP_031421	mmu:11479	

⟨R0V(Transmembrane protein 101)			PF15111.11	GO:004858	plasma mer	NP_083925	mmu:76547	
2BAY(chromosome 11 open reading			PF17722.6(I	GO:009773	cytoplasmic	KAF158314	mmu:68170	
K03255	TIF31, CLU1		PF13236.11	GO:004831	cytoplasmic	NP_001074	mmu:74148	
K12888	HNRNPU	mmu03040(	PF13671.12	GO:000371	cytoplasmic	BAE35231.1	mmu:51810	
K13305	NEDD4L	mmu04530(	PF00632.30(HECT:HEC		cytoplasmic	NP_001390	mmu:83814	
K09208	KLF9S, BTEB		PF13465.12	GO:000097	cytoplasmic	NP_510962	mmu:11844	zf-C2H2
K25689	NENF		PF00173.33	GO:003210	ER	NP_079700	mmu:66208	
K26389	TRAF3IP3			GO:001922	cytoplasmic	AAI37606.1	mmu:215243	
K17430	MRPL49, NOF1		PF05046.19	GO:001593	mitochondr	NP_080522	mmu:18120	
K01520	dut, DUT	mmu00983(	PF00692.24	GO:000921	nuclear	NP_076084	mmu:110074	
K09613	COPS5, CSN5		PF01398.26	GO:008009	cytoplasmic	NP_038743	mmu:26754	
K04373	RPS6KA	mmu04010(	PF00069.31	GO:001921	cytoplasmic	NP_001272	mmu:20111	
⟨QVU(ATP- binding)			PF04969.22	GO:000645	cytoplasmic	NP_080299	mmu:52653	
K24865	CDIN1		PF14811.11	GO:003250	cytoplasmic	NP_001139	mmu:399568	
K24642	ZBED5_7_8_9, BUSTE		PF06747.18	GO:004323	mitochondr	NP_898911	mmu:71970	
K18595	EML1_2		PF23409.1(I	GO:009951	cytoplasmic	AAI50976.1	mmu:68519	
K16475	LRRCC1, CLERC		PF12799.13	GO:000573	cytoplasmic	NP_083191	mmu:71710	
K19604	RP9, PAP-1	mmu03040(Spliceosome		GO:000578	nuclear	NP_061209	mmu:55934	
K11350	BRPF3		PF10513.14	GO:003124	nuclear	NP_001074	mmu:268936	
K18041	PTP4A		PF00102.32	GO:001679	cytoplasmic	NP_001233	mmu:19243	
K00968	PCYT1	mmu05231(	PF01467.32	GO:000665	nuclear	NP_001156	mmu:13026	
⟨RPV(Ubiquitin domain containing			PF16455.10	GO:000573	cytoplasmic	NP_776145	mmu:327900	
K16571	TUBGCP4, GCP4		PF04130.18	GO:000705	cytoplasmic	BAE31633.1	mmu:51885	
Regulation of nuclear pre-mRNA dc			PF00397.32	GO:004863	cytoplasmic	NP_598552	mmu:52357	
K04501	SMAD4	mmu05166(	PF03166.19	GO:000367	cytoplasmic	NP_001351	mmu:17128	MH1
K12847	USP39, SAI	mmu03040(	PF00443.35	GO:000548	nuclear	NP_613058	mmu:28035	
K03456	PPP2R1	mmu05165(	PF02985.28	GO:001679	cytoplasmic	XP_045631	mmu:51792	
K14566	UTP24, FCl	mmu03008(	PF04900.18	GO:003204	nuclear	BAB22857.1	mmu:73736	
K24642	ZBED5_7_8_9, BUSTE		PF06467.20	GO:002260	cytoplasmic	NP_001272	mmu:100177	
.NZD(ZMYM6 neighbor)			PF13564.11	GO:000557	plasma mer	XP_021016	mmu:100039968	
K22913	FIG4	mmu05014(	PF02383.23	GO:004846	cytoplasmic	BAE42839.1	mmu:103199	
K12649	DHX58, LC	mmu04622(	PF11648.15	GO:190024	cytoplasmic	NP_084426	mmu:80861	
K01329	CMA1	mmu04614(	PF00089.32	GO:000425	extracellulo	NP_034909	mmu:17227	
K09370	ISL1	mmu04550(	PF00412.28	GO:009008	nuclear	NP_001092	mmu:16392	Homeobox
K00747	CHPF	mmu00532(	PF05679.22	GO:003020	ER	NP_001001	mmu:74241	
K12733	PPIL1	mmu03040(	PF00160.26	GO:004323	cytoplasmic	NP_081121	mmu:68816	
K10364	CAPZA	mmu04144(	PF01267.22	GO:001602	cytoplasmic	NP_001009	mmu:12343	
K03128	TAF2	mmu03022(Basal trans		GO:008009	cytoplasmic	NP_001074	mmu:319944	
K12272	SRPRB, SRl	mmu03060(	PF09439.16	GO:000810	ER	NP_033301	mmu:20818	
K02263	COX4	mmu05208(	PF02936.19	GO:001986	mitochondr	NP_001280	mmu:12857	
K22422	DONSON			GO:004423	nuclear	NP_068366	mmu:60364	
K17411	MRPS33		PF08293.16	GO:001593	mitochondr	NP_034400	mmu:14548	
K01315	PLG	mmu04610(	PF00051.23	GO:000425	plasma mer	NP_032903	mmu:18815	

K11802	DCAF10		PF24796.1(	GO:004423	cytoplasmic	NP_694807	mmu:242418	
K27756	TBRG4, FASTKD4		PF08368.17	GO:000989	mitochondr	EDL01235.	mmu:21379	
K12834	PHF5A	mmu03040(	PF03660.19	GO:000613	cytoplasmic	NP_001076	mmu:68479	
K12859	TXNL4A, I	mmu03040(	PF02966.22	GO:000613	cytoplasmic	NP_001247	mmu:27366	
K11593	ELF2C, AGO		PF02171.23	GO:003577	cytoplasmic	NP_001304	mmu:236511	
K04515	CAMK2	mmu04750(	PF00069.31	GO:003563	cytoplasmic	XP_006514	mmu:12323	
K24799	TBC1D25		PF00566.25	GO:000989	cytoplasmic	NP_766066	mmu:209815	
K09585	TMX3, TXNDC10		PF13848.12	GO:000578	ER	NP_938037	mmu:67988	
K04692	STAT3	mmu04550(	PF01017.26(STAT_alph		cytoplasmic	XP_047398	mmu:20848	STAT
K25593	CASP8AP2		PF21227.3(	GO:000548	nuclear	NP_001116	mmu:26885	
K13813	STX12, STX	mmu04145(	PF14523.12	GO:000810	cytoplasmic	BAE41633.	mmu:100226	
K03462	NAMPT	mmu04621(	PF04095.22	GO:003292	cytoplasmic	NP_067499	mmu:59027	
K24371	ZNF335		PF13465.12	GO:008009	nuclear	NP_950192	mmu:32955	zf-C2H2
K20828	ZNF143_76	mmu04980(	PF13465.12	GO:004322	cytoplasmic	NP_001361	mmu:20841	zf-C2H2
K02355	fusA, GFM, EFG		PF00009.33	GO:004518	cytoplasmic	NP_613057	mmu:28030	
K18617	AIF1		PF21008.3(	GO:009859	cytoplasmic	NP_001102	mmu:108897	
K24805	ZBTB21		PF00651.37	GO:000582	nuclear	BAC98123.	mmu:11456	ZBTB
K12359	CLN6		PF15156.11	GO:000578	mitochondr	NP_001028	mmu:76524	
K25091	RBPMS		PF00076.28	GO:000548	cytoplasmic	NP_082306	mmu:71973	
K06582	CD244	mmu04650(	PF11465.14	GO:000989	plasma mer	NP_061199	mmu:18106	
K07955	ARL8	mmu05132(	PF00025.27	GO:000810	cytoplasmic	NP_001102	mmu:68724	
K10896	FANCM	mmu03460(	PF00270.35	GO:008009	mitochondr	AAI50786.1	mmu:104806	
K14775	UTP30, RSL1D1		PF00687.26	GO:001593	nuclear	ABE03015.	mmu:66409	
K06553	VPREB, CD179a		PF07686.23	GO:000961	cytoplasmic	AAB65160.	mmu:22364	
K17094	ANXA6		PF00191.25	GO:000526	cytoplasmic	NP_038500	mmu:11749	
K13123	GPATCH1		PF07713.18	GO:000613	nuclear	NP_080457	mmu:67471	
K13649	FOLR	mmu01523(	PF03024.20	GO:004212	extracellulo	NP_001290	mmu:14276	
K23564	EMC3, TMEM111		PF01956.21	GO:000810	Golgi appa	NP_001008	mmu:66087	
K01681	ACO	mmu01100(	PF00330.25	GO:000582	mitochondr	NP_542364	mmu:11429	
K25424	DNAAF9		PF23281.1(DAAF9_N:I		cytoplasmic	EDL28290.	mmu:228602	
K09390	E2F6	mmu03083(	PF16421.10	GO:000097	nuclear	NP_150373	mmu:50496	E2F
K16365	SGTA	mmu03060(	PF00515.34	GO:000810	cytoplasmic	NP_001345	mmu:52551	
K16341	BCL2L11, E	mmu05200(	PF08945.15	GO:001922	cytoplasmic	NP_997563	mmu:12125	
K07023	YGK1, HD	mmu01100(	PF13023.12	GO:004316	cytoplasmic	NP_081444	mmu:69692	
K10580	UBE2N, BI	mmu04120(	PF00179.31	GO:006165	cytoplasmic	NP_001012	mmu:93765	
SS6(HIRA interacting protein 3)			PF09649.15	GO:000557	nuclear	NP_766334	mmu:233876	
K11650	SMARCD	mmu04714(	PF02201.23	GO:003506	cytoplasmic	XP_054415	mmu:66993	
K17391	IGF2BP1	mmu05206(	PF00013.35	GO:199024	cytoplasmic	NP_034081	mmu:140486	
S5C(Zinc finger, ZZ-type with EF-			PF00569.23	GO:004423	nuclear	XP_006532	mmu:195018	
K20644	ARHGAP2	mmu04520(	PF22699.1(	GO:000548	cytoplasmic	BAC32168.	mmu:214137	
K21245	SUPT20H	mmu04140(	PF12090.13(Spt20_SEP:		nuclear	NP_001394	mmu:56790	
K11318	ATXN7, SCA7		PF08313.17(SCA7:SCA7		cytoplasmic	NP_001276	mmu:72522	
K17308	ORM, AGP		PF00061.29	GO:000695	extracellulo	NP_032794	mmu:18405	

K21853	DOCK9_10_11		PF06920.19	GO:000367	cytoplasmic	NP_001334	mmu:105445	
K20655	SYDE		PF00620.32	GO:009859	cytoplasmic	BAC33778.1	mmu:214804	
K06106	CTTN, EM	mmu04530(	PF02218.20	GO:200123	cytoplasmic	NP_031829	mmu:13043	
K05760	PXN	mmu05165(	PF03535.18(Paxillin:Pa		cytoplasmic	NP_001389	mmu:19303	
K07875	RAB1B	mmu05134(	PF00071.28	GO:000810	cytoplasmic	NP_083852	mmu:76308	
CNJ(PDX1	C-terminal	inhibiting f	PF10261.15	GO:004643	mitochondr	NP_775573	mmu:228859	
K26144	ICE2		PF10505.14	GO:000802	nuclear	NP_663593	mmu:93697	
K03841	FBP, fbp	mmu00051(	PF00316.25	GO:001920	cytoplasmic	NP_032020	mmu:14120	
K01599	hemE, URC	mmu01240(	PF01208.23	GO:000677	cytoplasmic	NP_033504	mmu:22275	
K01937	pyrG, CTPS	mmu01240(	PF06418.20	GO:009951	cytoplasmic	NP_001342	mmu:51797	
K12316	GAA	mmu00052(	PF01055.32	GO:003197	lysosomal	NP_001152	mmu:14387	
K13212	LUC7L2		PF03194.20	GO:002260	cytoplasmic	NP_001164	mmu:192196	
K23784	CNKSR3, CNK3		PF06663.18	GO:009859	cytoplasmic	NP_766134	mmu:215748	
K20456	OSBP		PF01237.23	GO:003577	cytoplasmic	NP_001028	mmu:76303	
K20399	LAMTOR4	mmu04150(mTOR sign		GO:009006	cytoplasmic	NP_001074	mmu:66096	
K09229	ZKSCAN		PF13465.12	GO:000097	cytoplasmic	AAH56222.	mmu:74120	zf-C2H2
K12020	TRIM44		PF00643.29	GO:008009	cytoplasmic	NP_064663	mmu:80985	
K11496	CENPB		PF09026.15	GO:000548	cytoplasmic	NP_031708	mmu:12616	
K23867	ECM1		PF05782.16	GO:008009	extracellulo	NP_001396	mmu:13601	
K00022	HADH	mmu00650(	PF02737.24	GO:009008	mitochondr	NP_032238	mmu:15107	
K11275	H1_5		PF00538.25	GO:008009	nuclear	NP_056602	mmu:50709	
K11275	H1_5		PF00538.25	GO:003472	nuclear	NP_085112	mmu:80838	
K11275	H1_5		PF00538.25	GO:000810	nuclear	NP_064418	mmu:56702	
K11275	H1_5		PF00538.25	GO:003472	nuclear	NP_663759	mmu:14957	
K24046	COBLL1		PF09469.16	GO:000809	cytoplasmic	NP_001350	mmu:319876	
K09559	BAG5		PF02179.21	GO:200123	cytoplasmic	NP_001311	mmu:70369	
K24939	CWF19L1, DRN1		PF04677.20	GO:009030	cytoplasmic	NP_001074	mmu:72502	
K04373	RPS6KA	mmu04010(	PF00069.31	GO:008009	cytoplasmic	EDL40821.	mmu:110651	
K22563	COMMD7		PF07258.19	GO:000989	cytoplasmic	NP_598611	mmu:99311	
K06229	SUFU	mmu05200(	PF12470.13	GO:000858	cytoplasmic	NP_056567	mmu:24069	
K13342	PEX5, PXR	mmu04146(	PF00515.34	GO:003444	cytoplasmic	NP_001397	mmu:19305	
K06070	PKD	mmu05208(	PF00069.31	GO:008009	cytoplasmic	EDL42075.	mmu:101540	
K15623	SS18, SSXT	mmu03082(	PF05030.17	GO:004323	nuclear	KAI258639	mmu:268996	
K14943	MBNL		PF22628.2(zf-CCCH_1(		cytoplasmic	NP_001347	mmu:105559	
K11423	SETD2	mmu00310(	PF08236.17	GO:000647	nuclear	NP_001074	mmu:235626	
K20371	NUCB		PF13499.12	GO:000367	extracellulo	NP_001157	mmu:18220	
K06210	NMNAT	mmu01240(	PF01467.32	GO:000548	cytoplasmic	NP_653116	mmu:74080	
K21436	ANKRD11_12		PF00023.36	GO:003250	nuclear	AAI72141.1	mmu:77087	
K25139	ERMARD				cytoplasmic	BAC31254.1	mmu:100041574	
K04421	MAP3K3, M	mmu04010(	PF00069.31	GO:001922	cytoplasmic	BAE23244.1	mmu:26406	
K18272	RP2		PF07986.17	GO:009859	cytoplasmic	NP_001277	mmu:19889	
K03901	F3, CD142	mmu04610(	PF01108.23	GO:000425	plasma mer	NP_034301	mmu:14066	
K12327	CALD1	mmu04270(	PF02029.20	GO:002260	cytoplasmic	NP_663550	mmu:109624	

K25448	OLFML1_3		PF02191.22	GO:000561	extracellula	NP_598620	mmu:99543	
K00972	UAP1	mmu00520(	PF01704.24	GO:000548	cytoplasmic	NP_001407	mmu:107652	
K21246	NRBF2	mmu05022(	PF08961.15	GO:014029	nuclear	NP_001031	mmu:641340	
K00255	ACADL	mmu03320(	PF00441.29	GO:003444	mitochondr	NP_031407	mmu:11363	
K22372	HERC5_6		PF00632.30	GO:003015	cytoplasmic	NP_080268	mmu:67138	
.4NV(immunity-related GTPase family Q)				GO:004316	cytoplasmic	NP_694774	mmu:210146	
K24881	ZHX3		PF00046.35	GO:004280	nuclear	NP_796237	mmu:32079	Homeobox
K24387	L3MBTL	mmu03083(	PF02820.24	GO:004239	nuclear	NP_001276	mmu:214669	
K15045	RSAD2	mmu05164(	PF04055.27	GO:006220	ER	NP_067359	mmu:58185	
K24815	HEATR6		PF13251.12(DUF4042:I		cytoplasmic	NP_663407	mmu:217026	
K05641	ABCA1	mmu02010(	PF00005.33	GO:000810	extracellula	NP_038482	mmu:11303	
K11446	KDM5, JARID1		PF08429.16	GO:000582	nuclear	NP_001390	mmu:20591	ARID
K05096	FLT1, VEG	mmu04010(	PF07714.23	GO:001922	plasma mer	NP_034358	mmu:14254	
K11404	HDAC3	mmu05203(	PF00850.24	GO:003288	cytoplasmic	NP_034541	mmu:15183	
K19194	ARID4A, RBP1		PF08169.17	GO:008009	nuclear	NP_001074	mmu:23824	ARID
CDC42 effector protein (Rho GTPas			PF02005.22	GO:004423	nuclear	NP_081152	mmu:98685	
ZI9Q(2-oxoglutarate and iron-deper			PF13640.12	GO:001602	Golgi appa	NP_079678	mmu:66179	
K15425	PPP4R2		PF09184.16	GO:008009	cytoplasmic	NP_891984	mmu:232314	
K16687	YAP1, Yki	mmu04390(	PF00397.32	GO:004885	cytoplasmic	NP_001164	mmu:22601	
.FTK(COMM domain containing 5)			PF07258.19	GO:004592	mitochondr	NP_001404	mmu:135575218	
K08967	mtnD, mtn	mmu00270(	PF03079.20	GO:001615	cytoplasmic	NP_598813	mmu:104923	
K22765	TMEM237		PF15383.11	GO:002260	cytoplasmic	XP_006496	mmu:381259	
K17542	SCYL3		PF00069.31	GO:003250	cytoplasmic	NP_083052	mmu:240880	
K18168	SDHAF2, SDH5		PF03937.21	GO:003250	mitochondr	XP_026633	mmu:66072	
K14463	MTTP, MT	mmu04975(	PF19444.4(	GO:000810	ER	NP_001341	mmu:17777	
K01173	ENDOG	mmu04210(	PF01223.29	GO:008009	mitochondr	NP_031957	mmu:13804	
K13247	CRYL1	mmu00040(	PF02737.24	GO:000663	extracellula	NP_084280	mmu:68631	
Specifically methylates the guanine i			PF01170.24	GO:000817	cytoplasmic	NP_001342	mmu:14911	
K00288	MTHFD	mmu01240(	PF01268.25	GO:000916	cytoplasmic	BAE21884.	mmu:108156	
K09851	RASSF2_4	mmu04392(	PF00788.29	GO:001922	cytoplasmic	NP_001349	mmu:215653	
YHHN(coiled-coil domain containir			PF15374.11(CCDC71L:		nuclear	NP_001365	mmu:72454	
K01324	KLKB1	mmu04610(	PF00089.32	GO:000695	extracellula	EDL35544.	mmu:16621	
K12603	CNOT6, C(	mmu03018(	PF03372.29	GO:008009	cytoplasmic	NP_849185	mmu:231464	
K14809	DDX55, SPB4		PF00270.35	GO:001688	cytoplasmic	AAH43052.	mmu:67848	
K07515	HADHA	mmu00650(	PF02737.24	GO:003444	mitochondr	NP_849209	mmu:97212	
K10993	FAAP100	mmu03460(	PF15146.11	GO:009030	mitochondr	NP_082256	mmu:71885	
K19366	SPG20	mmu04144(	PF06911.18	GO:004867	cytoplasmic	NP_001138	mmu:229285	
K17338	REEP1_2_3_4		PF03134.24	GO:002260	ER	NP_001191	mmu:28193	
K10613	CUL7	mmu04120(	PF24742.1(	GO:199023	cytoplasmic	NP_079887	mmu:66515	
K18732	SARNP, CIP29, THO1		PF18592.6(	GO:001593	nuclear	NP_079640	mmu:66118	
K12741	HNRNPA1	mmu05014(	PF00076.28	GO:000613	nuclear	AAI57905.1	mmu:229279	
K23740	SH3BGR		PF04908.21	GO:008009	cytoplasmic	BAE29696.	mmu:56726	
2996(Steroid receptor RNA activator			PF07304.16	GO:008009	cytoplasmic	Q80VJ2.4(R	mmu:24068	

K18667	ASCC2		PF02845.22	GO:014003	nuclear	BAE28346.1	mmu:75452
K01273	DPEP		PF01244.27	GO:001975	cytoplasmic	NP_795887	mmu:319446
K22560	COMMD4		PF21672.2((	GO:009030	nuclear	NP_079693	mmu:66199
K12626	LSM7	mmu03018(	PF01423.28	GO:000548	cytoplasmic	NP_079625	mmu:66094
J9K(Chromosome 1 open reading fr			PF05907.18	GO:004316	cytoplasmic	XP_027475	mmu:74098
XRII(NudC domain containing 1)			PF04969.22	GO:000573	cytoplasmic	NP_001107	mmu:67429
K13130	GEMIN2, SIP1		PF04938.17	GO:002260	nuclear	NP_079932	mmu:66603
ZT4(chromosome 6 open reading fr			PF14937.11	GO:000557	Golgi appa	NP_001317	mmu:66291
K10278	FBXL12		PF23598.1(I	GO:199023	mitochondr	NP_001002	mmu:30843
K12890	SRSF1, SFR	mmu04657(	PF00076.28	GO:003514	nuclear	NP_001182	mmu:110809
HQA(androgen-dependent TFPI-re			PF04750.19	GO:000663	Golgi appa	XP_042118	mmu:66253
K00604	MTFMT, fn	mmu00970(	PF00551.24	GO:000945	mitochondr	NP_081410	mmu:69606
K01313	F2	mmu05171(	PF00089.32	GO:009008	plasma mer	NP_034298	mmu:14061
K01513	ENPP1_3, (	mmu01100(	PF01663.27	GO:001922	plasma mer	NP_001295	mmu:18605
YRF(Chromosome 10 open reading			PF14958.11	GO:000573	cytoplasmic	NP_080931	mmu:68277
K15447	TRM44, METTL19		PF07757.19	GO:000817	cytoplasmic	NP_084484	mmu:78890
K20639	ARHGAP18_28_40		PF00620.32	GO:003304	cytoplasmic	SPT35699.1	mmu:268970
Y21Q(Microtubule-associated protei			PF00514.29	GO:002260	nuclear	XP_036016	mmu:227937
K16288	DSTYK, RIPK5		PF00069.31	GO:001922	cytoplasmic	NP_766104	mmu:213452
K06553	VPREB, CD179a		PF07686.23	GO:000548	plasma mer	DBA12495.	mmu:22362
K06553	VPREB, CD179a		PF07686.23(V-set:Immu		plasma mer	AAB81502.1	mmu:22362
K06752	MHC2	mmu05323(	PF07654.21	GO:001973	cytoplasmic	BAR71188.1	mmu:15002
K06554	IGLL1, IGI	mmu05340(	PF07654.21	GO:000548	cytoplasmic	ACO52352.	mmu:16136
K06553	VPREB, CD179a		PF07654.21	GO:000548	lysosomal	AAH93501.	mmu:22363
K06752	MHC2	mmu05323(	PF07654.21	GO:001922	cytoplasmic	EDL18541.	mmu:15000
K10949	KDELR		PF00810.23	GO:003303	ER	XP_007463	mmu:68137
K18622	ADD		PF00596.26	GO:004858	cytoplasmic	EDL37459.	mmu:11518
K10153	IRF2		PF00605.23	GO:008009	nuclear	KAF608415	mmu:16363 IRF
K25572	EHBP1		PF12130.13	GO:000810	nuclear	NP_001239	mmu:216565
K07894	RAB6B		PF00071.28	GO:000548	cytoplasmic	NP_001094	mmu:270192
K15144	MED1	mmu01522(	PF10744.14	GO:000551	nuclear	NP_001348	mmu:19014
K06698	PSME3	mmu04612(	PF02252.23	GO:200123	cytoplasmic	NP_001011	mmu:19192
K04011	CR1, CD35	mmu05144(	PF00084.26	GO:009859	plasma mer	Q64735.1(R	mmu:12946
K11787	GART	mmu01523(	PF01071.24	GO:000916	mitochondr	NP_001344	mmu:14450
K08514	VAMP5	mmu04130(	PF00957.27	GO:000577	cytoplasmic	BAE26254.1	mmu:53620
K17550	PPP1R7, SDS22		PF12799.13	GO:002240	nuclear	NP_075689	mmu:66385
alpha beta hydrolase fold)			PF03446.21	GO:004239	nuclear	NP_001073	mmu:74022
K06736	CDH2, CD	mmu05412(	PF00028.23	GO:000810	cytoplasmic	XP_006525	mmu:12558
K23687	RIN2_3		PF23268.1(I	GO:001989	cytoplasmic	NP_808288	mmu:217835
K23033	TENT5A_B, FAM46A_		PF07984.18	GO:000989	cytoplasmic	NP_001153	mmu:212943
K05019	CLNS1A		PF03517.19	GO:008009	nuclear	NP_076160	mmu:12729
K18169	TTC19		PF13424.12	GO:002260	mitochondr	NP_082636	mmu:72795
K21922	KCTD21		PF19329.4(I	GO:000858	cytoplasmic	NP_001034	mmu:622320

K12663	ECH1	mmu04146(	PF00378.25	GO:003444	peroxisoma	NP_058052	mmu:51798	
ZC52(kiaa1217)			PF03915.18	GO:000573	cytoplasmic	NP_001356	mmu:208618	
K05015	CLCN6		PF00654.25	GO:001707	plasma mer	NP_036059	mmu:26372	
K04634	GNAQ	mmu04750(	PF00503.26	GO:009008	cytoplasmic	NP_001230	mmu:14682	
K16750	BLOC1S2		PF10046.14	GO:009951	cytoplasmic	NP_082883	mmu:73689	
K18164	NDUFAF7	mmu04714(	PF02636.23	GO:000647	mitochondr	NP_082887	mmu:73694	
K20097	FKBPL		PF00515.34	GO:190555	cytoplasmic	NP_063926	mmu:56299	
K09049	ATF6B, CR	mmu05020(	PF00170.26	GO:008009	nuclear	O35451.1(R	mmu:12915	
K18764	NOCT, CCRN4L		PF03372.29	GO:003292	mitochondr	NP_033964	mmu:12457	
K02734	PSMB2	mmu05020(	PF00227.31	GO:005089	cytoplasmic	NP_036100	mmu:26445	
K06499	CEACAM, CD66		PF13927.12	GO:004521	plasma mer	P31809.1(R	mmu:26365	
K00963	UGP2, galU	mmu00052(	PF01704.24	GO:000548	cytoplasmic	EDL07846.	mmu:216558	
K07430	CYP7B	mmu00140(	PF00067.27	GO:000449	ER	BAE24236.	mmu:13123	
K04456	AKT	mmu05208(	PF00069.31	GO:007023	cytoplasmic	NP_001318	mmu:11651	
K11452	CBX4, PC2	mmu03083(	PF00385.30	GO:003506	nuclear	NP_031651	mmu:12418	
Z9Q4(deoxynucleotidyltransferase, t			PF08698.16	GO:003204	nuclear	NP_722501	mmu:99480	
K17936	SNX27		PF00787.29	GO:000810	cytoplasmic	NP_001075	mmu:76742	
unc-5 homolog)			PF22725.1((	GO:000548	peroxisoma	NP_001028	mmu:328232	
2DD0(Proline rich 24)			PF15018.11	GO:000557	plasma mer	NP_001129	mmu:66300	
K18727	TNPO2, IP	mmu03013(	PF13513.12	GO:003450	cytoplasmic	NP_001116	mmu:212999	
K23495	RCC1L		PF00415.24	GO:001604	peroxisoma	NP_291050	mmu:94254	
K11141	ENPEP, CI	mmu04614(	PF01433.26	GO:000417	cytoplasmic	NP_031960	mmu:13809	
K24935	TTC27		PF07719.23(TPR_2:Tetr		peroxisoma	NP_690030	mmu:74196	
K25359	ANGPTL2		PF00147.23	GO:000561	extracellulo	NP_036053	mmu:26360	
K15322	TSEN2		PF01974.23	GO:000815	nuclear	NP_950198	mmu:381802	
K08769	UCP1, SLC	mmu03320(	PF00153.32	GO:008009	cytoplasmic	NP_033489	mmu:22227	
K23267	APCS, SAP		PF00354.24	GO:008009	extracellulo	NP_035448	mmu:20219	
K20400	FNIP1	mmu04150(	PF14637.11	GO:190545	cytoplasmic	NP_776114	mmu:216742	
K23594	LXN		PF06907.18	GO:000695	cytoplasmic	NP_058033	mmu:17035	
K06820	PLXNA	mmu04360(	PF08337.17	GO:006008	plasma mer	NP_032908	mmu:18845	
K06820	PLXNA	mmu04360(	PF08337.17	GO:004858	plasma mer	NP_032907	mmu:18844	
XP9(transcription, DNA-dependen			PF13865.11	GO:001593	nuclear	OWK04629	mmu:66511	
K10703	HACD, PH	mmu01040(	PF04387.19	GO:000663	cytoplasmic	NP_067320	mmu:57874	
K06069	PRKCI	mmu05165(	PF00069.31	GO:004423	cytoplasmic	NP_032883	mmu:18759	
K18435	SOX9	mmu04024(	PF00505.25	GO:008009	nuclear	NP_035578	mmu:20682	HMG
K17708	RAP1GAP2		PF02145.21	GO:004858	cytoplasmic	KAG32697	mmu:380711	
YE3A(Family with sequence similar			PF13402.12	GO:190547	cytoplasmic	NP_001342	mmu:77574	
K26090	SINHCAF		PF15396.11	GO:003033	nuclear	KAF645379	mmu:56306	
K06114	SPTAN	mmu04210(	PF00435.27	GO:004299	cytoplasmic	EDL08429.	mmu:20740	
K03129	TAF4	mmu05016(	PF05236.20	GO:008009	nuclear	XP_006500	mmu:228980	
K15717	PRXL2B, F	mmu00590(	PF13911.12	GO:000663	cytoplasmic	NP_079858	mmu:66469	
K10730	RECQL4		PF00270.35	GO:006203	nuclear	NP_478121	mmu:79456	
K13146	INTS9		PF21382.2(I	GO:009030	nuclear	BAC36917.	mmu:210925	

K13174	THOC5	mmu03013(	PF09766.14	GO:005116	nuclear	NP_766026	mmu:107829	
K23028	SPECC1		PF00307.36	GO:003111	nuclear	NP_001139	mmu:74392	
K21892	MB		PF00042.28	GO:000534	cytoplasmic	NP_001157	mmu:17189	
XQQN(ORAI calcium release-activ			PF01755.23	GO:004323	ER	NP_997181	mmu:99151	
K08837	STK10, LO	mmu04914(	PF12474.13	GO:001707	cytoplasmic	BAD90480.	mmu:20868	
K17633	RASAL2	mmu04014(	PF12004.13	GO:008009	nuclear	NP_001388	mmu:226525	
K07200	PRKAG	mmu04530(	PF00571.33	GO:000663	cytoplasmic	NP_001164	mmu:108099	
K00876	udk, UCK	mmu00983(	PF00485.24	GO:000921	cytoplasmic	NP_035805	mmu:22245	
.5SA(chromosome 16 open reading f			PF15755.10	GO:000557	plasma mer	NP_849232	mmu:101602	
K00318	PRODH, fc	mmu00330(	PF01619.24	GO:000548	mitochondr	NP_035302	mmu:19125	
K01892	HARS, hisS	mmu00970(	PF13393.11	GO:000372	cytoplasmic	BAE31128.1	mmu:15115	
K00157	AOX	mmu00982(	PF20256.3(	GO:000403	cytoplasmic	NP_076106	mmu:71724	
K18084	MTMR9		PF06602.19	GO:009859	cytoplasmic	BAE31122.1	mmu:210376	
K24732	GNB1L		PF00400.38	GO:000761	mitochondr	AAH24635.	mmu:13972	
.GYZ(gTPase, IMAP family membe			PF04548.22	GO:004316	cytoplasmic	NP_777620	mmu:317758	
K12200	PDCD6IP,	mmu03250(	PF03097.24	GO:000810	cytoplasmic	NP_035182	mmu:18571	
K12858	DDX23, PR	mmu03040(	PF00270.35	GO:004654	nuclear	XP_021072	mmu:74351	
K17600	VPS54		PF07928.17	GO:000810	cytoplasmic	NP_620692	mmu:245944	
K18655	DDX19, DI	mmu03013(	PF00270.35	GO:000548	nuclear	NP_031942	mmu:13680	
XR87(Translin)			PF01997.21	GO:000989	cytoplasmic	NP_035780	mmu:22099	
K12393	AP1M	mmu04142(	PF00928.26	GO:000810	cytoplasmic	NP_031482	mmu:11767	
.YE0(immunoglobulin heavy variab			PF07686.23(V-set:Immu		lysosomal	DBA12436.1(TPA: IGHV1-18*01, p		
Z836(Chromosome 17 open reading			PF15053.11	GO:000810	cytoplasmic	AAH44897.	mmu:70591	
K17502	PPM1F, POPX2		PF00481.26	GO:005134	cytoplasmic	NP_789803	mmu:68606	
K07889	RAB5C	mmu04137(	PF00071.28	GO:000810	cytoplasmic	NP_077776	mmu:19345	
K01372	BLMH, pepC		PF03051.21	GO:000417	cytoplasmic	NP_848760	mmu:104184	
K07884	RAB3D	mmu04972(	PF00071.28	GO:000810	cytoplasmic	NP_001311	mmu:19340	
K24457	SLFN12, SLFN12L		PF17057.10	GO:000989	cytoplasmic	NP_035538	mmu:20556	
XTBJ(chromosome 9 open reading fi			PF07052.16	GO:200024	cytoplasmic	XP_034350	mmu:227707	
K24878	ADNP2		PF19627.4(	GO:001922	nuclear	AAH44898.	mmu:24044	TF_others
K13092	ZC3H18		PF18044.7(	GO:000989	cytoplasmic	XP_006531	mmu:76014	
K13289	CTSA, CPY	mmu04142(	PF00450.28	GO:190471	lysosomal	NP_032932	mmu:19025	
K12460	KIDINS22(	mmu04722(	PF12796.13	GO:003250	cytoplasmic	XP_006515	mmu:77480	
K04525	SERPINA		PF00079.25	GO:007132	extracellula	NP_033278	mmu:20716	
K11586	CBX3, HP1	mmu03083(	PF01393.25	GO:003506	nuclear	AAI10377.1	mmu:12417	
K10301	FBXO21		PF13369.12	GO:009715	cytoplasmic	EDL19801.	mmu:231670	
K01681	ACO	mmu01100(	PF00330.25	GO:001683	cytoplasmic	NP_001406	mmu:11428	
K25210	LANCL1	mmu00480(	PF05147.19	GO:001712	cytoplasmic	NP_001177	mmu:14768	
.NZJ(immediate early response 5)			PF05760.17	GO:008013	nuclear	NP_034630	mmu:15939	
K01432	AFMID	mmu01240(	PF20434.5(I	GO:004253	cytoplasmic	NP_082103	mmu:71562	
K09536	DNAJC16		PF00226.36	GO:000699	extracellula	BAC65689.1	mmu:214063	
YTJW(cysteine-rich transmembrane module con				GO:004323	cytoplasmic	XP_021058	mmu:66060	
K22970	DHRS11	mmu00140(	PF00106.31	GO:001622	mitochondr	NP_808232	mmu:192970	

K11687	ADPRHL2	mmu03410(	PF03747.19	GO:004423	mitochondr	NP_598644	mmu:100206	
K14284	NXF, TAP,	mmu05164(	PF24048.1(I	GO:001593	nuclear	NP_570958	mmu:170722	
.EZC(methyltransferase like 7A); C(			PF08241.18	GO:000581	extracellulo	CAD60206.	mmu:70152	
K18134	EOGT	mmu00514(	PF04577.19	GO:000578	ER	EDK99350.	mmu:101351	
K14523	RPP38	mmu03008(	PF01248.31	GO:000454	nuclear	NP_001013	mmu:227522	
K13128	ZCCHC8		PF04046.21	GO:000613	cytoplasmic	NP_081770	mmu:70650	
K03680	EIF2B4	mmu05168(Herpes simp		GO:000367	cytoplasmic	AAA68046.	mmu:13667	
K09699	DBT, bkdB	mmu01100(	PF00198.28	GO:004438	mitochondr	NP_034152	mmu:13171	
K08283	COL4A3BP		PF01852.25	GO:000578	nuclear	NP_075909	mmu:68018	
K08143	SLC2A5, G	mmu04973(	PF00083.30	GO:000821	plasma mer	NP_062715	mmu:56485	
K25811	CCDC88C	mmu04310(	PF19047.5(I	GO:001922	cytoplasmic	NP_080957	mmu:68339	
K01565	SGSH	mmu04142(	PF00884.28	GO:000032	lysosomal	EDL34698.	mmu:27029	
K16912	LAS1		PF04031.18	GO:000815	cytoplasmic	NP_001361	mmu:76130	
K18772	APOBEC2		PF18772.7(	GO:001655	cytoplasmic	NP_033824	mmu:11811	
.6W1(Serum albumin family)			PF00273.25	GO:000573	cytoplasmic	NP_001239	mmu:622307	
K09173	RFX1_2_3		PF04589.18	GO:008009	nuclear	NP_082063	mmu:19725	RFX
K27804	FRY		PF19421.4(I	GO:000573	cytoplasmic	XP_017176	mmu:72313	
K03004	RPA43, PO	mmu03020(	PF17875.7(I	GO:003088	nuclear	NP_758457	mmu:28071	
K09500	CCT8		PF00118.29	GO:009951	cytoplasmic	NP_033970	mmu:12469	
K07827	KRAS, KR	mmu05208(	PF00071.28	GO:001922	cytoplasmic	BAE33023.	mmu:16653	
K02331	POL5, MYBBP1A		PF04931.18	GO:003292	nuclear	NP_058056	mmu:18432	
Midline 1 (Opitz BBB syndrome))			PF14681.12	GO:000921	cytoplasmic	NP_001074	mmu:331487	
K11487	PCGF1, NS	mmu03083(	PF16207.11	GO:000989	nuclear	NP_001390	mmu:69837	
K13129	SMN		PF06003.18	GO:003577	cytoplasmic	AAN77614.	mmu:20595	
K02213	CDC6	mmu04110(	PF09079.17	GO:000716	cytoplasmic	NP_035929	mmu:23834	
K02649	PIK3R1_2_	mmu05208(	PF16454.10	GO:003315	cytoplasmic	NP_001070	mmu:18708	
K10769	ALKBH7		PF13532.12	GO:000548	mitochondr	NP_079814	mmu:66400	
K09539	DNAJC19		PF00226.36	GO:000810	mitochondr	NP_001273	mmu:67713	
K17531	OBSCN, AI	mmu04820(	PF07679.22	GO:009951	cytoplasmic	NP_954603	mmu:380698	
K06252	TN	mmu05165(	PF00041.27	GO:009951	plasma mer	XP_006525	mmu:81877	
K05099	MET, HGF	mmu05208(	PF07714.23	GO:190074	plasma mer	AAI17970.1	mmu:17295	
K08663	GZMK		PF00089.32	GO:000425	extracellulo	AAI17982.1	mmu:14945	
K06709	KDM4, JMJD2, JHDM3		PF02373.28	GO:200073	nuclear	NP_001165	mmu:76804	
.TX5(zinc finger protein)				GO:004316	nuclear	BAB26915.1	mmu:69752	
24J3(SREK1-interacting protein 1)			PF13917.11	GO:000548	nuclear	XP_026246	mmu:67288	
K11643	CHD4, MI2	mmu05165(	PF00176.29	GO:001707	nuclear	NP_001390	mmu:107932	
K03234	EEF2	mmu04152(	PF00009.33	GO:008009	cytoplasmic	NP_031933	mmu:13629	
K12006	TRIM16		PF00622.36	GO:008009	cytoplasmic	NP_444399	mmu:94092	
K06678	YCG1, CAPG		PF12719.13	GO:003304	cytoplasmic	NP_062311	mmu:54392	
K07880	RAB4B		PF00071.28	GO:000548	cytoplasmic	NP_059051	mmu:19342	
.EBH(junctional sarcoplasmic reticu			PF15312.11	GO:000810	cytoplasmic	NP_082277	mmu:71912	
K15516	FMR		PF17904.7(I	GO:003243	nuclear	XP_040600	mmu:14265	
K11997	TRIM2_3		PF01436.27	GO:000988	cytoplasmic	NP_001258	mmu:80890	

K11446	KDM5, JARID1		PF08429.16	GO:003292	nuclear	NP_666109	mmu:21489	ARID
K13111	SMU1		PF00400.38	GO:008009	cytoplasmic	XP_006276	mmu:74255	
K18730	GIGYF		PF02213.21	GO:004423	cytoplasmic	NP_001103	mmu:227331	
K09537	DNAJC17		PF00226.36	GO:000989	cytoplasmic	NP_631878	mmu:69408	
K12829	SF3B2, SAP	mmu03040(	PF02037.33	GO:000613	nuclear	BAB31644.1	mmu:319322	
K20310	TRAPPC13		PF06159.19	GO:199007	cytoplasmic	NP_080155	mmu:66975	
K16463	CEP170		PF15308.11	GO:000573	cytoplasmic	NP_001399	mmu:217882	
K16806	MAP7D1		PF05672.16	GO:000573	cytoplasmic	NP_659190	mmu:245877	
K03363	CDC20	mmu04110(	PF24807.1(	GO:003243	cytoplasmic	NP_075712	mmu:107995	
HZ4(CDC42 effector protein (Rho			PF14957.11	GO:000806	cytoplasmic	NP_001156	mmu:56699	
K14313	NUP35, NU	mmu03013(	PF05172.20	GO:003450	nuclear	NP_081367	mmu:69482	
K12301	SLC17A5	mmu04142(	PF07690.21	GO:009859	plasma mem	NP_766361	mmu:235504	
K19531	CDAN1		PF15296.11	GO:005116	cytoplasmic	NP_081167	mmu:68968	
K01137	GNS	mmu04142(	PF00884.28	GO:000032	lysosomal	NP_083640	mmu:75612	
K20632	DLC		PF01852.25	GO:000548	cytoplasmic	NP_001411	mmu:243362	
K05655	ABCB8	mmu02010(	PF00664.28	GO:000367	peroxisoma	NP_083296	mmu:74610	
K19984	EXOC5, SE	mmu05132(	PF07393.17	GO:000548	cytoplasmic	NP_997097	mmu:105504	
K11504	CENPL		PF13092.12	GO:003299	mitochondr	NP_001153	mmu:70454	
K05871	PTK2B, FA	mmu04670(	PF07714.23	GO:004858	cytoplasmic	EDL36002.	mmu:19229	
K10393	KIF2_24, M	mmu04814(	PF00225.29	GO:001604	cytoplasmic	NP_001361	mmu:16563	
K02864	RP-L10, MI	mmu03010(Ribosome)		GO:001593	mitochondr	NP_080430	mmu:107732	
K14005	SEC31	mmu04141(	PF24807.1(	GO:000810	cytoplasmic	NP_081245	mmu:69162	
K13098	TLS, FUS	mmu05014(	PF00076.28	GO:008009	nuclear	BAE24196.	mmu:233908	
K07888	RAB5B	mmu04137(	PF00071.28	GO:000810	cytoplasmic	NP_001125	mmu:19344	
K03068	LRP5_6	mmu05022(	PF00058.23	GO:006130	ER	BAE27651.	mmu:16973	
K11348	BRPF1		PF10513.14	GO:003124	nuclear	XP_017177	mmu:78783	
K01056	PTH1, PTRH1, pth, spo		PF01195.25	GO:001678	mitochondr	BAE20993.	mmu:329384	
K23902	PDCD6		PF13499.12	GO:000810	cytoplasmic	NP_035181	mmu:18570	
K20690	ARHGEF25		PF00621.25	GO:009951	cytoplasmic	NP_082303	mmu:52666	
K05850	ATP2B	mmu04970(	PF00689.26	GO:001922	cytoplasmic	NP_080758	mmu:67972	
K09355	PBX1	mmu04148(	PF03792.18	GO:003510	nuclear	NP_001128	mmu:18514	Homeobox
K09105	TFE3	mmu04137(	PF15951.10	GO:008009	nuclear	NP_001098	mmu:20944	bHLH
K12856	PRPF8, PR	mmu03040(	PF08083.16	GO:000024	cytoplasmic	NP_001178	mmu:192159	
RED(RNA binding motif protein)			PF17790.7(	GO:000695	plasma mem	EDL08642.	mmu:98870	
K14007	SEC24	mmu04141(	PF04811.21	GO:000810	cytoplasmic	NP_081411	mmu:69608	
K14564	NOP56	mmu03008(	PF01798.23	GO:003204	nuclear	NP_077155	mmu:67134	
K12394	AP1S1_2	mmu04142(	PF01217.26	GO:000810	cytoplasmic	NP_001006	mmu:108012	
K20899	GBP1_3_4_	mmu04621(	PF02263.25	GO:001922	cytoplasmic	NP_001076	mmu:229900	
K02888	RP-L21, MI	mmu03010(	PF00829.26	GO:001593	mitochondr	NP_001334	mmu:353242	
K21112	SYNPO	mmu04530(Tight juncti		GO:003243	cytoplasmic	BAE42857.	mmu:104027	
K27706	ZC3H15, TMA46		PF16543.10	GO:001922	nuclear	NP_081210	mmu:69082	
K19986	EXOC8, SEC84		PF16528.11	GO:006164	cytoplasmic	NP_932771	mmu:102058	
K04567	KARS, lysS	mmu00970(	PF00152.25	GO:001710	cytoplasmic	EDL11525.	mmu:85305	

TUX(Forty-two-three domain conte			PF07078.16	GO:001593	nuclear	NP_081502	mmu:69823
K02358	tuf, TUFM		PF00009.33	GO:004518	mitochondr	NP_766333	mmu:233870
K00558	DNMT1, dc	mmu00270(	PF01426.24	GO:004423	cytoplasmic	EDL25140.	mmu:13433
K26226	PPFIA		PF00536.37	GO:190195	cytoplasmic	BAD90289.	mmu:327814
4JE(Ataxin	7-like 3B)			GO:000573	cytoplasmic	NP_001028	mmu:382423
K09611	NPEPL1		PF00883.26	GO:000417	cytoplasmic	BAE29459.	mmu:228961
K23845	KIFBP		PF12309.13	GO:001989	cytoplasmic	BAC98130.	mmu:72320

tf_evidence	signalp_type
	signal_peptide(Sec/SPI)
	other
	other
	signal_peptide(Sec/SPI)
	other
	other
924	other
	signal_peptide(Sec/SPI)
	other
	other
	other
	other
	signal_peptide(Sec/SPI)
	other
	other
	other
	other
	signal_peptide(Sec/SPI)
	signal_peptide(Sec/SPI)
	other
	signal_peptide(Sec/SPI)
	other
	signal_peptide(Sec/SPI)
	other
	signal_peptide(Sec/SPI)
	signal_peptide(Sec/SPI)
	other
	other
	other
	other
	other
	other
	other
	other
	other
	other
	other
	other
	other
	other
	other
	other

other
other
other
other
other
signal_peptide(Sec/SPI)
other
signal_peptide(Sec/SPI)
other
other
other
other
other
other
other
other
other
other
signal_peptide(Sec/SPI)
other
signal_peptide(Sec/SPI)
other
signal_peptide(Sec/SPI)
other
other
other
other
other
other
other
other
other
other
other
other
other
other
other
other
other
other
other
other

		other
		other
		other
		other
		other
		other
		other
		other
		other
		other
		other
		signal_peptide(Sec/SPI)
		other
		other
		other
		signal_peptide(Sec/SPI)
-		other
		other
		other
		other
		other
		other
		signal_peptide(Sec/SPI)
		other
		other
		other
		other
		other
		signal_peptide(Sec/SPI)
		other
		other
		other
		other
	1337	other
		other
-		other
		other
		other
		other
	97.1	other
		other
		other
		other

	other
	other
rtial [Mus n	signal_peptide(Sec/SPI)
	other
	signal_peptide(Sec/SPI)
	signal_peptide(Sec/SPI)
	signal_peptide(Sec/SPI)
	signal_peptide(Sec/SPI)
	other
	other
	signal_peptide(Sec/SPI)
	other
	other
	signal_peptide(Sec/SPI)
	other
	other
	other
	other
	other
	other
	other
	other
	other
	other
	other
	other
	other
	other
	other
	other
	other
	other
-	other
	signal_peptide(Sec/SPI)
	other
	other
	other
	other
	other
	other
	other
	other
	other

		other
		other
		other
		other
		other
		other
		other
		other
	1678	other
		signal_peptide(Sec/SPI)
		other
		other
		other
		other
	444	other
		other
		other
		signal_peptide(Sec/SPI)
		signal_peptide(Sec/SPI)
		other
		other
		other
-		other
	1676	other
		other
		other
		other
		other
		other
		other
		other
		other
		other
		other
		signal_peptide(Sec/SPI)
		other
		other
		signal_peptide(Sec/SPI)
		signal_peptide(Sec/SPI)
		other
		other
		other
		other

-	other
	other
	other
	other
	other
	other
	other
	other
	other
	signal_peptide(Sec/SPI)
	other
	other
-	other
	other
	other
	other
	other
	other
	other
	other
	other
	other
	other
	other
-	other
	other
	other
	other
	other
	signal_peptide(Sec/SPI)
	other
	other
	other
	other
	other
	signal_peptide(Sec/SPI)
	other
	other
	other
	other
	other
	other
	signal_peptide(Sec/SPI)

		other
		other
		other
		other
		other
		signal_peptide(Sec/SPI)
		other
		other
		signal_peptide(Sec/SPI)
		other
		other
		signal_peptide(Sec/SPI)
		other
		other
		other
		other
		signal_peptide(Sec/SPI)
		other
		other
		other
		other
		other
		other
		signal_peptide(Sec/SPI)
		other
		other
		other
		other
-		other
		other
		other
		other
	1456	other
		other
		other
-		other
-		other
		other
		other
		other
		other
		other
		other

	other
	other
	other
	other
	other
	other
	other
	other
	other
	other
	other
	other
	other
	other
	other
	signal_peptide(Sec/SPI)
	other
	other
	other
-	other
	other
	other
	other
	signal_peptide(Sec/SPI)
	other
	other
	signal_peptide(Sec/SPI)
	signal_peptide(Sec/SPI)
	signal_peptide(Sec/SPI)
	other
	other
	other
	other
	other
	other
	other
	other
	other
	other
	other
	other
	other
	other

		other
		other
		signal_peptide(Sec/SPI)
		other
-		other
	70.9	other
		signal_peptide(Sec/SPI)
		other
		other
		other
		other
		other
		other
		other
		other
		other
		other
		other
		other
		other
		other
		other
		other
		other
		signal_peptide(Sec/SPI)
		other
		other
		other
		other
		other
		other
		other
		other
		other
		other
		signal_peptide(Sec/SPI)
		other
		signal_peptide(Sec/SPI)
		other
		other
		other
		other
		other

	other
	signal_peptide(Sec/SPI)
	other
	other
	other
	signal_peptide(Sec/SPI)
	other
	other
	other
	other
	other
	other
	other
	other
	signal_peptide(Sec/SPI)
	other
	other
	other
	other
	other
	other
	other
	other
	other
	other
	other
	other
olypeptide I	other
	other
	other
	other
	signal_peptide(Sec/SPI)
	other
	other
	other
	other
	other
	other
	other
	other
	other
	other
	other

other
other
other
other
other
other
other
other
other
other
other
signal_peptide(Sec/SPI)
other
other
other
other
other
other
other
other
other
3110 other
other
other
other
other
signal_peptide(Sec/SPI)
other
other
other
other
other
other
other
other
other
other
other
other
signal_peptide(Sec/SPI)
signal_peptide(Sec/SPI)
other
other

rtial [Mus n	signal_peptide(Sec/SPI)
	other
	other
	other
	other
	other
	other
	other
	other
	other
	other
	signal_peptide(Sec/SPI)
	other
element 1 i	signal_peptide(Sec/SPI)
	other
	other
	other
	other
	other
	other
	other
	other
	other
	other
	other
	other
	other
	other
	other
	other
	other
	other
	other
	signal_peptide(Sec/SPI)
	signal_peptide(Sec/SPI)
	other
	other
	other
	other
	other
	other
	other
	other

		other
		other
		signal_peptide(Sec/SPI)
		other
		other
-		other
		other
		other
		other
		other
-		other
		other
		other
		other
		other
		other
		other
		other
		other
		other
-		other
		other
		other
		other
		other
		other
		other
		other
		other
		other
		other
		other
		other
		other
	556	other
		other
		other
		other
		other
		signal_peptide(Sec/SPI)
		other
		other
		other

		other
		other
		signal_peptide(Sec/SPI)
		other
		other
		other
		other
		other
		other
		other
		other
		other
		other
		other
		other
		other
		other
		other
		other
		other
		other
	50.4	other
		other
		other
		other
		other
		other
		other
-		other
		other
		other
		other
		other
		other
		other
		other
		other
		other
		other
		other
		other
		other
ragment [M		other

	other
	other
	signal_peptide(Sec/SPI)
	other
	other
	other
	other
	other
	other
	other
	signal_peptide(Sec/SPI)
	other
	signal_peptide(Sec/SPI)
	other
	other
	other
	signal_peptide(Sec/SPI)
	other
chain varia	signal_peptide(Sec/SPI)
	signal_peptide(Sec/SPI)
	other
	other
	signal_peptide(Sec/SPI)
	signal_peptide(Sec/SPI)
	other
	other
	other
	other
	signal_peptide(Sec/SPI)
	other
	other
	other
	other
	other
	other
	other
	other
	other
	other
	other
	other
	other
	other

	signal_peptide(Sec/SPI)
	other
-	other
	other
	other
	other
	signal_peptide(Sec/SPI)
sculus])	signal_peptide(Sec/SPI)
	signal_peptide(Sec/SPI)
	other
	other
	other
	other
uct [Mus m	other
	other
	other
	other
	other
	other
	other
	other
	other
	other
	other
	signal_peptide(Sec/SPI)
	other
	other
	signal_peptide(Sec/SPI)
-	other
	other
	other
	other
	signal_peptide(Sec/SPI)
	other
	other
	other
	other
	other
	other
	other
	other
	signal_peptide(Sec/SPI)
	other

	other
	other
	other
	other
	other
	other
	other
	other
	other
	signal_peptide(Sec/SPI)
	other
	other
	signal_peptide(Sec/SPI)
	signal_peptide(Sec/SPI)
	other
	other
	other
	other
	other
	other
	other
	other
	signal_peptide(Sec/SPI)
	other
	other
	other
	other
	other
	other
	other
	other
	other
	other
-	other
	other
	other
	other
	other
	other
	other
	other
	other
	other

	other
	other
	other
	signal_peptide(Sec/SPI)
	other
	other
	other
	signal_peptide(Sec/SPI)
	other
	other
	signal_peptide(Sec/SPI)
	other
-	other
	other
	other
	other
	signal_peptide(Sec/SPI)
	other
	signal_peptide(Sec/SPI)
	other
	other
	other
	other
	other
	other
	other
	other
	other
	other
	other
	other
	other
	other
	other
	signal_peptide(Sec/SPI)
	other
	other
	other
	other
	signal_peptide(Sec/SPI)
	other
	other
	other

	signal_peptide(Sec/SPI)
	other
	other
	other
	other
	other
	other
	other
	other
	other
	other
	other
	other
	other
	other
	other
	other
	other
	other
	signal_peptide(Sec/SPI)
	other
	other
	other
	other
	other
	other
	other
	other
	other
	other
	other
	other
	other
y chain vari	other
artial [Mus	other
	other
artial [Mus	signal_peptide(Sec/SPI)
	other
	other
	other
	other
583	other
	other

	other
	other
	other
	other
	signal_peptide(Sec/SPI)
	other
	other
	other
	other
	signal_peptide(Sec/SPI)
	other
	other
	other
	other
	other
	other
	other
	other
	other
y chain vari	other
	signal_peptide(Sec/SPI)
	other
	signal_peptide(Sec/SPI)
	other
	other
	other
	other
	other
	other
	other
	other
	other
	other
	signal_peptide(Sec/SPI)
	other
	other
	other
	other
	signal_peptide(Sec/SPI)
	other
	other
	other

	other
	other
	other
	other
	other
	other
	other
	other
	other
	other
	other
	other
	other
	other
	other
-	other
	signal_peptide(Sec/SPI)
	other
	other
	other
	signal_peptide(Sec/SPI)
	signal_peptide(Sec/SPI)
	other
	other
	other
	other
	other
	signal_peptide(Sec/SPI)
	other
	other
	other
	other
	other
	other
	other
	other
	other
	other
	signal_peptide(Sec/SPI)
	other
	other
	signal_peptide(Sec/SPI)
	signal_peptide(Sec/SPI)

		other
		other
-		other
		other
		other
		other
-		other
		other
		other
		other
		other
		other
		other
		signal_peptide(Sec/SPI)
		other
		other
		other
		other
		other
		other
		other
		signal_peptide(Sec/SPI)
		other
		other
		other
		other
		other
		other
		other
		other
		signal_peptide(Sec/SPI)
		other
		other
-		signal_peptide(Sec/SPI)
		other
		other
		other
		other
		other
		other
		other
	2871	other
		signal_peptide(Sec/SPI)

other
other
other
other
other
other
other
other
other
1466 other
other
other
signal_peptide(Sec/SPI)
other
other
other
other
other
other
other
other
other
other
other
other
other
signal_peptide(Sec/SPI)
other
other
other
other
other
other
other
other
signal_peptide(Sec/SPI)
other
other
other
other
other
other
signal_peptide(Sec/SPI)

	other
	other
	other
	other
	other
	other
	signal_peptide(Sec/SPI)
-	other
	other
	other
	other
	other
	signal_peptide(Sec/SPI)
	other
	other
	other
	other
	signal_peptide(Sec/SPI)
	other
	other
	other
	other
	other
	other
	other
	signal_peptide(Sec/SPI)
	other
	other
2589	other
	other
	other
	other
	other
	other
	other
	other
	other
	other
	other
	other
	other
	other
	other

	other
	other
	signal_peptide(Sec/SPI)
	other
	other
	other
	other
	other
	other
	other
	other
	other
	signal_peptide(Sec/SPI)
	other
	other
	other
	other
	other
	signal_peptide(Sec/SPI)
	other
	signal_peptide(Sec/SPI)
	other
	other
	other
	other
	other
	signal_peptide(Sec/SPI)
	other
	other
-	other
	other
	other
	other
	signal_peptide(Sec/SPI)
	other
	other
	other
	other
	other
	other
	other
	other
	signal_peptide(Sec/SPI)

		other
		other
		other
		other
		other
		other
		other
		other
		other
		other
		other
		other
		other
		other
		other
		other
		other
	364	other
		other
		other
-		other
		other
		other
		other
		other
		other
		other
		other
-		other
		other
		other
		other
		other
		other
		other
		other
chain varia		signal_peptide(Sec/SPI)
		other
		other
		other
		other
		other
		other

other
other
other
other
other
other
signal_peptide(Sec/SPI)
other
signal_peptide(Sec/SPI)
other
other
signal_peptide(Sec/SPI)
other
other
other
signal_peptide(Sec/SPI)
other
other
other
other
other
signal_peptide(Sec/SPI)
other
other
other
signal_peptide(Sec/SPI)
other
other
other
other
other
signal_peptide(Sec/SPI)
other
other
other
other
other
other
other
signal_peptide(Sec/SPI)
other
other
signal_peptide(Sec/SPI)

		other
		other
		signal_peptide(Sec/SPI)
		other
		other
		signal_peptide(Sec/SPI)
		other
		other
		other
		other
		other
		other
		other
		other
		other
		other
		other
		other
		other
-		other
		other
		other
		other
		other
-		other
		other
		other
-		other
		other
		other
		other
		other
		other
		other
		other
		other
		other
		other
		signal_peptide(Sec/SPI)
		other
		other
	1159	other
		other

		other
		other
	1404	other
		other
		other
		other
		other
		other
		other
	1068	other
		other
		other
		other
		other
		other
		other
		other
		other
		other
		other
		other
		signal_peptide(Sec/SPI)
-		other
		other
		other
		other
		other
		other
		other
		other
		other
		signal_peptide(Sec/SPI)
		other
		other
		signal_peptide(Sec/SPI)
		other
		other
		other
		other
		other
		other
		other
		other

	other
	other
	other
	other
	other
	signal_peptide(Sec/SPI)
	other
	other
	other
	other
	other
	other
	other
	other
	other
	other
	other
	other
	other
	other
	signal_peptide(Sec/SPI)
	other
	other
	signal_peptide(Sec/SPI)
	other
	other
	other
-	other
	other
-	other
	other
	other
	other
	other
	other
	other
	other
	signal_peptide(Sec/SPI)
	other
	other
	signal_peptide(Sec/SPI)
	other
	other

-		other
		other
		other
		signal_peptide(Sec/SPI)
		other
		other
		other
		other
		other
		other
		other
		other
		other
		other
		other
		other
		other
		other
		other
		other
		other
		other
	832	other
		other
		other
		other
		other
		other
		signal_peptide(Sec/SPI)
		other
		other
		other
		signal_peptide(Sec/SPI)
		other
		other
		other
-		other
		other
		other
		other
		other
		other
		other

	other
	other
	other
	other
	other
	other
	other
	other
	signal_peptide(Sec/SPI)
	other
	other
	other
	other
	other
	other
	other
	other
	other
	other
	other
-	other
	other
	other
	other
	other
	other
	other
	other
	other
	signal_peptide(Sec/SPI)
	other
	other
	other
	other
	other
	other
	other
	other
	other
	other
	other
	other
	signal_peptide(Sec/SPI)

signal_peptide(Sec/SPI)
other
other
other
other
other
other
other
other
other
other
other
other
other
other
other
other
other
other
other
other
2023 other
other
other
other
other
other
- other
other
other
other
other
other
other
other
other
other
other
other
other
other
other
other

		other
		signal_peptide(Sec/SPI)
		other
		other
		other
		other
		other
		other
		other
		other
		other
		signal_peptide(Sec/SPI)
		signal_peptide(Sec/SPI)
		other
		other
		other
		other
		other
		other
		other
		other
		other
		other
	932	other
		other
	970	other
		other
-		other
		other
		other
		other
		other
		signal_peptide(Sec/SPI)
	865	other
		other
		other
		other
		other
		other
		other
		other
		other
		other

	other
	other
	other
	other
-	other
	signal_peptide(Sec/SPI)
	other
	other
	other
	other
	other
	other
	other
	other
	other
	other
	other
	other
	other
	other
	other
	other
	other
	other
	signal_peptide(Sec/SPI)
	other
	other
	other
	other
	other
	other
	other
	other
	signal_peptide(Sec/SPI)
	other
	other
	other
	other
	other
	other
	other
	other
	other

other
other
other
other
other
other
other
other
signal_peptide(Sec/SPI)
other
other
other
other
other
other
other
other
other
other
other
other
other
signal_peptide(Sec/SPI)
other
other
other
other
other
other
other
other
other
other
other
other
968 other
other
signal_peptide(Sec/SPI)
other
other
other
other
other

	other
	other
	other
	other
	other
	other
	other
	other
	other
	other
	other
	other
	other
	other
	signal_peptide(Sec/SPI)
	other
	other
	other
	other
	other
	other
-	other
	other
	other
	other
	other
	other
	other
	other
	signal_peptide(Sec/SPI)
	other
	other
	other
	signal_peptide(Sec/SPI)
	other
	other
	other
	other
	other
	other
	other
	other
	other

other
other
other
other
other
signal_peptide(Sec/SPI)
other
other
signal_peptide(Sec/SPI)
other
other
other
signal_peptide(Sec/SPI)
other
other
other
other
other
signal_peptide(Sec/SPI)
other
other
other
other
other
signal_peptide(Sec/SPI)
other
other
signal_peptide(Sec/SPI)
other
other
signal_peptide(Sec/SPI)
other
other
2583 other
other
other
other
other
other
other
other
other
other

	other
	other
-	other
	other
on, partial [	other
	other
	other
-	other
	other
	other
	other
	other
	other
	other
	signal_peptide(Sec/SPI)
	other
	other
	other
	other
	signal_peptide(Sec/SPI)
	other
	other
	other
	other
	other
	signal_peptide(Sec/SPI)
	other
	other
	signal_peptide(Sec/SPI)
	other
	other
	other
	other
	other
	other
	other
	other
	signal_peptide(Sec/SPI)
	other
	signal_peptide(Sec/SPI)
	signal_peptide(Sec/SPI)
	signal_peptide(Sec/SPI)
	other

		other
		other
		signal_peptide(Sec/SPI)
		other
		other
		other
		other
		other
		other
		other
		other
		other
		signal_peptide(Sec/SPI)
		other
		other
		other
		other
		other
		other
		other
		other
		other
		other
		other
		other
		other
		other
		other
	817	other
		other
		other
		other
	1258	other
		other
		signal_peptide(Sec/SPI)
		other
		other
		other
-		other
		other
		signal_peptide(Sec/SPI)
		other
		other

	other
	other
	other
	signal_peptide(Sec/SPI)
	other
	other
	other
	other
	other
	other
	other
	other
	other
	other
	other
-	other
	other
	other
-	other
-	other
	other
	other
	other
	other
	other
	other
	other
	other
	other
	other
	other
	other
	other
	other
	other
	other
	other
	other
	other
	other
	other
	other
	other

	other
	other
	other
	other
	other
	other
	signal_peptide(Sec/SPI)
	other
-	other
	other
	other
383	other
	other
	signal_peptide(Sec/SPI)
	other
	signal_peptide(Sec/SPI)
	other
	other
	other
	other
	signal_peptide(Sec/SPI)
	other
chain varia	signal_peptide(Sec/SPI)
	other
	other
	other
	other
	other
	other
	other
	other
	other
	other
	other
	signal_peptide(Sec/SPI)
	other
	other
	other
	signal_peptide(Sec/SPI)
	signal_peptide(Sec/SPI)
	other
	other
	other

	other
	signal_peptide(Sec/SPI)
	signal_peptide(Sec/SPI)
	other
	other
	other
	other
	other
	other
	other
	other
	other
	other
	signal_peptide(Sec/SPI)
	other
	other
	other
	other
	other
	signal_peptide(Sec/SPI)
-	other
	other
	other
	other
	other
	other
	other
	other
	other
	other
	other
	other
	other
	other
	other
	other
	other
	other
	other
	other
	other
	other
	other

other
other
signal_peptide(Sec/SPI)
other
other
other
other
other
other
other
signal_peptide(Sec/SPI)
other
other
other
other
other
other
signal_peptide(Sec/SPI)
other
other
other
other
other
other
other
signal_peptide(Sec/SPI)
signal_peptide(Sec/SPI)
other
other
other
386 other
other
other
other
signal_peptide(Sec/SPI)
other
other
other
other
other
signal_peptide(Sec/SPI)
- other
other

178	other
	other
	other
	other
	other
	other
	other
	other
	signal_peptide(Sec/SPI)
	other
	other
	other
	other
	other
	other
	other
	other
	other
	other
	other
	other
	other
	other
820	other
	other
	other
	other
	signal_peptide(Sec/SPI)
	signal_peptide(Sec/SPI)
	other
	other
	other
501	other
	other
	other
	other
	other
	other
	other
	other
-	other
	other
	other

other
other
signal_peptide(Sec/SPI)
signal_peptide(Sec/SPI)
other
other
signal_peptide(Sec/SPI)
signal_peptide(Sec/SPI)
other
other
other
signal_peptide(Sec/SPI)
other
other
other
other
other
other
signal_peptide(Sec/SPI)
signal_peptide(Sec/SPI)
other
other
other
other
other
other
other
other
other
other
other
other
other
other
- other
other
other
signal_peptide(Sec/SPI)
other
other
other
other
other

	other
	signal_peptide(Sec/SPI)
	other
	signal_peptide(Sec/SPI)
	other
	other
	other
	other
	other
	signal_peptide(Sec/SPI)
	other
	other
	other
	other
	signal_peptide(Sec/SPI)
	other
	other
	other
	other
	other
	other
	other
	other
-	other
	other
-	other
-	other
	other
	other
	other
	other
	other
	signal_peptide(Sec/SPI)
	other
	other
	other
	signal_peptide(Sec/SPI)
	other
	other
	other
	other
	other
	other

	other
	other
	other
	other
	other
	other
	other
	other
	other
	other
	signal_peptide(Sec/SPI)
	other
	other
	other
	signal_peptide(Sec/SPI)
	other
	other
	other
	other
1237	other
	other
	other
	other
	other
	other
	other
793	other
	other
	other
	other
	other
	other
353	other
	other
	other
	other
	other
	other
	other
	other
	other
	other
	other

-		other
		signal_peptide(Sec/SPI)
		other
		signal_peptide(Sec/SPI)
		other
		other
		other
		other
		other
		other
		other
		other
		other
		other
		other
		other
		other
		other
		other
		other
		other
		other
		other
	1940	other
		signal_peptide(Sec/SPI)
		other
		other
		other
-		other
		other
		other
		other
		other
		other
		other
		other
		other
		other
		other
		other
		other
		other
		signal_peptide(Sec/SPI)

other
other
other
other
- other
signal_peptide(Sec/SPI)
signal_peptide(Sec/SPI)
other
other
other
other
other
other
other
other
other
other
other
other
other
signal_peptide(Sec/SPI)
other
other
115 other
other
other
other
signal_peptide(Sec/SPI)
other
signal_peptide(Sec/SPI)
other
other
other
other
other
other
other
other
other
other
other
other
other

		other
		other
		other
		signal_peptide(Sec/SPI)
		other
	1199	other
-		other
		other
		other
		other
		other
		other
		other
		other
		other
		other
		other
		other
		other
		other
		other
		other
		other
		other
		other
		other
		other
		other
		other
-		other
		other
		other
		other
		other
		other
		other
		signal_peptide(Sec/SPI)
		other
		other
		other
		other
		other
		other

	other
	other
	other
	other
	other
	other
	other
	other
	other
	other
	other
	other
	other
	signal_peptide(Sec/SPI)
	other
	signal_peptide(Sec/SPI)
	other
	other
	other
	other
	other
	signal_peptide(Sec/SPI)
	other
	other
	other
	signal_peptide(Sec/SPI)
	other
	signal_peptide(Sec/SPI)
	other
	other
	other
	other
	other
	other
	other
	other
	other
	other
	other
-	other
	other
	other
	other

	other
	other
	other
	other
	other
	other
	other
	other
	other
	other
	other
53.9	other
	other
	other
	other
	other
1458	other
	other
	other
	other
	other
	other
	signal_peptide(Sec/SPI)
	other
	signal_peptide(Sec/SPI)
	other
	other
	other
	other
	other
	other
	other
	other
	other
	other
	other
	other
	signal_peptide(Sec/SPI)
	other
	other
	other
	other
	other

	other
	other
	other
	other
	other
	other
	other
	other
	other
	other
	other
	other
	other
	other
	other
	other
-	other
	other
	signal_peptide(Sec/SPI)
	other
	other
	other
	other
	other
	other
	other
	other
	other
	other
	other
	other
	other
	other
	other
	other
	signal_peptide(Sec/SPI)
	other
	other
-	other
	other
	other
	other
	other

	other
	other
	other
	signal_peptide(Sec/SPI)
	other
-	other
	other
ıthase sulfuı	other
	other
	other
	signal_peptide(Sec/SPI)
	signal_peptide(Sec/SPI)
	other
	other
	signal_peptide(Sec/SPI)
	other
	other
	other
	other
	signal_peptide(Sec/SPI)
	other
	signal_peptide(Sec/SPI)
	other
	other
	other
	other
	signal_peptide(Sec/SPI)
	other
	other
	other
	other
	other
	other
	other
68.9	other
	other
	signal_peptide(Sec/SPI)
253	other
	other
	other
	other
	signal_peptide(Sec/SPI)
	other

other
other
other
other
other
other
other
other
other
other
other
other
other
other
other
signal_peptide(Sec/SPI)
other
other
other
other
other
signal_peptide(Sec/SPI)
other
signal_peptide(Sec/SPI)
other
other
signal_peptide(Sec/SPI)
other
other
other
other
other
other
other
other
other
other
other
other
other
signal_peptide(Sec/SPI)
other
other

	other
	other
	other
	other
	other
	other
	other
	other
	other
	other
	other
	signal_peptide(Sec/SPI)
	other
-	other
	other
	other
	other
	signal_peptide(Sec/SPI)
	other
	other
	other
	other
	other
-	other
	other
	other
	other
	other
	other
	other
	signal_peptide(Sec/SPI)
	other
	signal_peptide(Sec/SPI)
	other
	other
	other
	other
	other
	other
	other
	other
	other
	other

		other
		other
		other
	2169	other
		other
		other
		other
		signal_peptide(Sec/SPI)
		other
		other
		other
		other
		other
		other
		other
		other
		other
		other
		other
		signal_peptide(Sec/SPI)
		other
		other
		other
		other
		other
		other
		other
		other
		other
		other
		other
		other
		signal_peptide(Sec/SPI)
		other
-		other
		signal_peptide(Sec/SPI)
		other
		other
		other
		other
		other
		other
		other

		other
		other
-		other
		other
		other
		other
		other
		signal_peptide(Sec/SPI)
		other
		other
		other
		other
		other
		signal_peptide(Sec/SPI)
		other
		signal_peptide(Sec/SPI)
		other
		other
		other
		other
		other
		other
		other
	66.2	other
		other
		other
		other
		signal_peptide(Sec/SPI)
		signal_peptide(Sec/SPI)
		other
		other
		other
		other
		other
		other
		other
		other
-		other
-		signal_peptide(Sec/SPI)
		other
		other
		signal_peptide(Sec/SPI)
		other

	other
	other
	other
	other
	other
	other
	other
	other
	other
	other
	other
-	other
	other
	other
	other
	other
	other
	signal_peptide(Sec/SPI)
	other
avy chain v	other
	other
	other
	other
	other
	other
	other
	other
	other
	other
	other
	other
	other
	signal_peptide(Sec/SPI)
	other
	other
	other
	other
	other
	signal_peptide(Sec/SPI)
	other
	signal_peptide(Sec/SPI)
	other
	other

		other
		other
		other
		other
		other
		other
		other
		signal_peptide(Sec/SPI)
		other
		other
		other
		other
		other
	986	other
		signal_peptide(Sec/SPI)
		other
		other
		signal_peptide(Sec/SPI)
		other
		other
		other
		other
		other
		other
	833	other
		signal_peptide(Sec/SPI)
		other
		other
		other
		other
		other
		other
		other
		signal_peptide(Sec/SPI)
		other
		other
		other
-		other
-		other
		other
		other
		other
		other

signal_peptide(Sec/SPI)
other
signal_peptide(Sec/SPI)
other
other
other
other
other
other
other
other
other
other
signal_peptide(Sec/SPI)
other
other
other
other
other
other
signal_peptide(Sec/SPI)
other
314 other
other
other
other
signal_peptide(Sec/SPI)
other
other
other
other
other
other
other
other
other
other
other
other
other
other
other
other

signal_peptide(Sec/SPI)
other
other
other
other
signal_peptide(Sec/SPI)
other
other
other
other
other
- other
other
other
other
other
other
other
signal_peptide(Sec/SPI)
other
other
other
other
other
other
other
other
signal_peptide(Sec/SPI)
signal_peptide(Sec/SPI)
other
other
other
other
other
other
other
other
other
signal_peptide(Sec/SPI)
other
signal_peptide(Sec/SPI)
other
other

	other
	other
	other
	other
	other
	other
	other
	other
	other
	signal_peptide(Sec/SPI)
	other
	other
	other
	other
	other
	other
	other
	other
	signal_peptide(Sec/SPI)
	other
	other
	other
-	other
	signal_peptide(Sec/SPI)
	other
	other
	signal_peptide(Sec/SPI)
	other
-	other
	signal_peptide(Sec/SPI)
	other
	other
	other
	other
	other
	other
	other
	other
	other
	other
	other
	other
	other

other
other
other
signal_peptide(Sec/SPI)
other
other
other
other
signal_peptide(Sec/SPI)
other
other
other
signal_peptide(Sec/SPI)
other
signal_peptide(Sec/SPI)
other
other
other
other
signal_peptide(Sec/SPI)
other
other
other
other
other
signal_peptide(Sec/SPI)
signal_peptide(Sec/SPI)
other
other
other
signal_peptide(Sec/SPI)
other
other
other
other
other
other
other
other
other
other
other
other

	other
	other
-	other
	other
	other
	other
	other
	other
	other
	other
	other
	other
	other
	other
	other
	other
	signal_peptide(Sec/SPI)
	other
	other
	other
	other
	other
	other
	other
	signal_peptide(Sec/SPI)
	other
	other
	other
	other
	other
	other
	other
	other
	other
	signal_peptide(Sec/SPI)
	other
	other
	other
	other
	other
	signal_peptide(Sec/SPI)
	other
	other

other
signal_peptide(Sec/SPI)
other
signal_peptide(Sec/SPI)
other
other
other
other
other
other
other
other
other
other
other
other
other
other
other
other
other
other
other
other
other
other
other
other
other
other
other
other
other
other
other
330 other
other
other
other
other
other
other
other

other
other
other
other
signal_peptide(Sec/SPI)
other
other
other
other
other
other
other
signal_peptide(Sec/SPI)
other
other
other
other
other
signal_peptide(Sec/SPI)
other
other
other
other
other
other
signal_peptide(Sec/SPI)
other
other
other
other
other
other
other
other
other
other
other
other
other
other
other
other
other

	other
	other
	other
	signal_peptide(Sec/SPI)
	other
	other
	signal_peptide(Sec/SPI)
	other
	other
1040	other
	other
	other
	other
	other
	other
	other
	other
	other
	other
	other
	other
	other
	other
	other
	signal_peptide(Sec/SPI)
	other
ıunoglobuli	signal_peptide(Sec/SPI)
	other
	other
	other
	other
	other
	other
2098	other
	other
	other
	other
	other
	other
	other
	other
	other
	other

other
other
other
other
other
other
other
other
1816 other
other
other
other
other
other
other
other
other
other
other
other
other
other
other
other
other
signal_peptide(Sec/SPI)
other
other
other
signal_peptide(Sec/SPI)
other
other
other
other
other
other
other
other
other
other
other
other

other
other
signal_peptide(Sec/SPI)
other
other
other
other
other
other
other
other
other
signal_peptide(Sec/SPI)
other
other
other
other
866 other
other
other
other
other
other
other
signal_peptide(Sec/SPI)
other
other
other
signal_peptide(Sec/SPI)
other
other
other
other
other
other
other
other
other
other
416 other
other
signal_peptide(Sec/SPI)
signal_peptide(Sec/SPI)

		other
		other
		other
		other
		other
-		other
		other
		other
		other
		other
		signal_peptide(Sec/SPI)
		signal_peptide(Sec/SPI)
		other
		other
		signal_peptide(Sec/SPI)
		other
		other
		other
		other
		other
		other
		signal_peptide(Sec/SPI)
		other
		other
		other
		other
		other
		other
		other
		other
		signal_peptide(Sec/SPI)
-		other
		other
		signal_peptide(Sec/SPI)
	690	other
		other
		other
		other
		other
		other
		other
		other
		other

	other
	other
	other
	other
	other
	other
	other
	other
	other
	other
	other
	signal_peptide(Sec/SPI)
-	other
	other
	other
	other
	other
	other
	other
	other
	other
	other
	other
	other
	other
	other
	other
	other
	other
	other
	other
	other
	other
	other
	other
	other
	other
	other
	other
	other
	other

other
other
other
other
other
other
other
other
other
other
other
other
other
other
4447 other
other
other
other
other
other
other
other
signal_peptide(Sec/SPI)
other
other
signal_peptide(Sec/SPI)
signal_peptide(Sec/SPI)
other
other
other
other
other
other
other
other
other
other
other
other
other
other
other
other

other
other
other
signal_peptide(Sec/SPI)
other
other
other
other
other
other
other
other
other
1175 other
- other
signal_peptide(Sec/SPI)
other
other
other
other
other
other
other
other
other
other
other
other
other
other
other
other
other
other
other
other
other
other
other
other
other
other
other

	other
	signal_peptide(Sec/SPI)
	other
-	other
	other
	other
	other
	other
	other
	other
	other
	other
	other
-	other
-	other
	other
	other
	other
	other
	other
	other
	other
	other
	signal_peptide(Sec/SPI)
	other
	signal_peptide(Sec/SPI)
	other
	signal_peptide(Sec/SPI)
	other
	other
	signal_peptide(Sec/SPI)
	other
	other
	other
	signal_peptide(Sec/SPI)
	other
	other
	other
	other
	other
	other
	other
rtial [Mus n	signal_peptide(Sec/SPI)

	signal_peptide(Sec/SPI)
	other
	other
	other
	other
	other
	other
	other
	other
	other
	signal_peptide(Sec/SPI)
-	other
	other
	other
	other
	other
	other
	other
	other
	signal_peptide(Sec/SPI)
	other
	signal_peptide(Sec/SPI)
	signal_peptide(Sec/SPI)
	other
	other
	other
	other
	other
	other
	other
	signal_peptide(Sec/SPI)
	other
	other
	other
	other
	other
	other
	other
	other
	other
	other
	other
	other

	other
	other
	signal_peptide(Sec/SPI)
	other
	other
	other
	signal_peptide(Sec/SPI)
	other
	other
	other
	other
	other
	other
	other
	signal_peptide(Sec/SPI)
	other
	other
	signal_peptide(Sec/SPI)
	other
	other
	other
	signal_peptide(Sec/SPI)
	signal_peptide(Sec/SPI)
	other
	other
	other
	other
	other
	other
	other
	other
	other
	other
	other
	signal_peptide(Sec/SPI)
	other
-	other
	other
	other
	other
	other
	other
	other

	other
	other
	other
	other
	other
-	other
	signal_peptide(Sec/SPI)
	other
	other
	other
	other
	other
	other
	signal_peptide(Sec/SPI)
	other
	other
	other
	other
	other
	other
	other
	other
	other
	other
	other
	other
	signal_peptide(Sec/SPI)
	other
	other
	other
	other
	signal_peptide(Sec/SPI)
	other
	other
	other
	other
-	other
	signal_peptide(Sec/SPI)
-	other
	other
	other
	other
	other

	other
	signal_peptide(Sec/SPI)
	other
	signal_peptide(Sec/SPI)
	other
	other
	other
48.1	other
	other
	other
	other
	other
	other
	other
	other
	other
	signal_peptide(Sec/SPI)
	signal_peptide(Sec/SPI)
	other
	other
	other
	other
	other
	other
	other
	signal_peptide(Sec/SPI)
	other
	signal_peptide(Sec/SPI)
	other
	other
	other
	other
	other
	other
	other
	other
1836	other
	other
	other
	other
	other
	other
404	other

		other
		other
		other
		other
		other
		other
		other
		other
		other
		signal_peptide(Sec/SPI)
-		other
		other
		signal_peptide(Sec/SPI)
		other
		signal_peptide(Sec/SPI)
		other
		other
-		other
		other
		other
		other
		other
		other
		other
		other
	1181	other
		other
		other
		other
		other
-		other
		other
		other
		other
		other
-		other
		other
		other
		other
		other
		other
		other
		other

		signal_peptide(Sec/SPI)
	694	other
		other
		other
		other
		signal_peptide(Sec/SPI)
		other
		other
		signal_peptide(Sec/SPI)
		signal_peptide(Sec/SPI)
		signal_peptide(Sec/SPI)
		other
		other
		other
		other
		other
		other
		other
		other
		other
		other
		other
		other
	1627	other
		other
		other
		other
		other
		other
		other
		other
		other
		other
		other
		other
		other
		other
-		other
		other
		other
		other
		other
		other

		other
		other
-		other
		other
		other
		other
		other
		other
		other
		other
		signal_peptide(Sec/SPI)
		other
		other
		other
		other
		other
		other
		other
		signal_peptide(Sec/SPI)
		other
		other
		other
		other
		other
		other
		other
		other
		other
		other
-		other
		other
	63.5	other
		other
		other
		other
		other
		other
		other
		other
		signal_peptide(Sec/SPI)
		signal_peptide(Sec/SPI)
		other
		other

85.1 other
other
other
other
other
other
other
other
other
other
other
other
other
other
other
other
other
signal_peptide(Sec/SPI)
other
other
other
other
other
signal_peptide(Sec/SPI)
other
other
- other
other
other
other
other
other
other
other
signal_peptide(Sec/SPI)
other
other
- other
other
other
other
other
signal_peptide(Sec/SPI)

		other
		other
		other
		other
		other
		other
		other
		other
		other
		other
	2013	other
		other
		other
		signal_peptide(Sec/SPI)
		other
		other
		other
-		other
		other
		other
		other
		other
		other
		other
		signal_peptide(Sec/SPI)
		other
		other
		other
		other
		other
		other
		other
		other
		other
		signal_peptide(Sec/SPI)
		signal_peptide(Sec/SPI)
		other
		other
		other
		other
		other
		other
		other

other
other
other
other
other
other
other
other
signal_peptide(Sec/SPI)
other
other
other
other
other
other
other
other
other
other
other
signal_peptide(Sec/SPI)
other
other
other
other
232 other
other
other
other
other
other
other
signal_peptide(Sec/SPI)
other
signal_peptide(Sec/SPI)
other
other
other
other
other
other
other
other

	other
	other
	other
-	other
	signal_peptide(Sec/SPI)
	other
	other
	signal_peptide(Sec/SPI)
	other
	other
	other
	other
	other
	other
	other
	other
	other
	other
	other
	other
	other
	other
-	other
	other
	signal_peptide(Sec/SPI)
	other
	other
-	other
	other
	other
	other
	other
	other
	other
	signal_peptide(Sec/SPI)
	other
	signal_peptide(Sec/SPI)
	other
	other
	other
	other
	other
	other

	other
	other
	other
-	other
	other
	other
-	other
	other
	other
	other
	other
	other
	other
	other
	other
88.6	other
	other
	other
	other
	other
	other
	signal_peptide(Sec/SPI)
	other
	signal_peptide(Sec/SPI)
	other
	other
	other
	other
	other
	other
chain varia	signal_peptide(Sec/SPI)
	other
	other
	signal_peptide(Sec/SPI)
	signal_peptide(Sec/SPI)
	other
	other
	other
	signal_peptide(Sec/SPI)
	other
	other
	other
	other

		other
	1156	other
-		other
-		other
-		other
		other
		other
		other
		other
-		other
		other
		other
		other
		other
		signal_peptide(Sec/SPI)
		signal_peptide(Sec/SPI)
		other
		other
		other
		signal_peptide(Sec/SPI)
		other
		other
		other
		other
		signal_peptide(Sec/SPI)
		other
		other
		other
		other
-		other
		other
		signal_peptide(Sec/SPI)
		other
		other
		other
		other
		other
		other
		other
		other
		signal_peptide(Sec/SPI)
		other
		other

	other
	other
	signal_peptide(Sec/SPI)
	signal_peptide(Sec/SPI)
	other
	other
	other
	other
-	other
	signal_peptide(Sec/SPI)
	other
	signal_peptide(Sec/SPI)
	signal_peptide(Sec/SPI)
	signal_peptide(Sec/SPI)
	other
	other
	other
	other
	other
	other
	other
	other
	other
	other
	other
	other
-	other
	other
	other
	other
	other
	other
	other
	other
	other
	other
	other
	signal_peptide(Sec/SPI)
	signal_peptide(Sec/SPI)
-	other
	other
	signal_peptide(Sec/SPI)
	other

		other
		other
		other
		other
		other
		other
		other
		other
		other
		other
		other
		other
		other
		other
		other
		other
		other
-		other
		other
		other
		other
		other
		other
		other
		signal_peptide(Sec/SPI)
		signal_peptide(Sec/SPI)
		other
		other
		other
		other
		other
	877	other
		other
-		other
		other
		other
		other
		other
		other
		other
		other
		signal_peptide(Sec/SPI)
		other

	other
J region, pa	other
	other
	other
595	other
	other
	other
	other
	signal_peptide(Sec/SPI)
	other
	other
	other
	other
	other
	other
	other
	other
	other
	other
	other
	other
	other
	other
	other
	other
	other
	signal_peptide(Sec/SPI)
	other
	other
	other
-	other
	other
	other
	other
816	other
	other
	other
550	other
	other
	other
	other
	other
	other

	other
	signal_peptide(Sec/SPI)
	other
	signal_peptide(Sec/SPI)
	other
	other
	other
	other
	other
	other
	other
	other
	signal_peptide(Sec/SPI)
	other
	other
	other
	signal_peptide(Sec/SPI)
chain varia	signal_peptide(Sec/SPI)
	signal_peptide(Sec/SPI)
	other
	other
	other
	other
	other
	signal_peptide(Sec/SPI)
	other
	other
	other
	other
	other
	signal_peptide(Sec/SPI)
	other
	other
	other
	signal_peptide(Sec/SPI)
	other
	other
	other
	signal_peptide(Sec/SPI)
	other
-	other
	other
	other

	other
	signal_peptide(Sec/SPI)
	other
	other
	other
	other
	other
-	other
	other
	other
	other
	other
	other
	other
	other
	other
	other
-	other
	other
	other
	other
	other
	other
	other
	other
	other
	signal_peptide(Sec/SPI)
	other
	other
	other
	other
	other
	other
	other
	other
	other
	other
	other
	signal_peptide(Sec/SPI)
	other
	other
	other
	other

	other
	other
	other
	other
	other
	signal_peptide(Sec/SPI)
	other
	signal_peptide(Sec/SPI)
	other
	other
	signal_peptide(Sec/SPI)
-	other
	other
	other
	other
	other
	other
	other
	other
	other
	other
	signal_peptide(Sec/SPI)
	other
	other
	signal_peptide(Sec/SPI)
	signal_peptide(Sec/SPI)
	other
	other
	other
	signal_peptide(Sec/SPI)
	other
	other
	other
	other
	other
	other
	other
	other
	other
	other
	other
	other
	other

	other
	other
	other
	other
	other
	other
	other
	other
	other
	other
-	other
	other
	other
	other
	signal_peptide(Sec/SPI)
	other
	other
	other
	other
	other
	other
	other
	other
	other
	other
	other
	other
	other
	other
	signal_peptide(Sec/SPI)
	other
	other
	signal_peptide(Sec/SPI)
-	other
	other
	other
	other
	other
	other
	other
chain V-V ı	signal_peptide(Sec/SPI)
	signal_peptide(Sec/SPI)
	other

	other
	other
	signal_peptide(Sec/SPI)
	other
	other
	other
	other
	other
	other
	other
	other
	signal_peptide(Sec/SPI)
	other
	other
	signal_peptide(Sec/SPI)
	other
	other
	other
	other
	other
	other
	other
	other
	other
	signal_peptide(Sec/SPI)
	other
	other
	other
	other
	other
-	other
	other
	other
	other
	other
	other
	other
	other
	other
	other
	other
	other
	other

		other
-		other
		other
		other
		other
		other
		other
		other
		other
		other
		other
		other
		other
-		other
		other
		signal_peptide(Sec/SPI)
		other
		other
		other
		other
		signal_peptide(Sec/SPI)
		other
		other
		other
		other
		other
		other
		other
		other
		other
		other
		other
		other
		other
		other
		other
	52	other
		other
		other
		other
		other
		other
		other

other
other
signal_peptide(Sec/SPI)
other
other
other
signal_peptide(Sec/SPI)
other
other
other
other
other
other
other
other
other
other
other
other
other
other
other
other
other
other
other
other
other
other
other
other
other
other
signal_peptide(Sec/SPI)
other
signal_peptide(Sec/SPI)
other
other
other
other
other
other
383 other

	other
	other
	other
	other
	other
	other
	signal_peptide(Sec/SPI)
	other
	other
	other
	other
	other
	other
	other
	other
	other
	other
	other
-	other
	other
	other
	other
	other
	other
	other
	other
	other
	other
	other
	other
	other
	other
	other
	signal_peptide(Sec/SPI)
	other
	other
	other
	signal_peptide(Sec/SPI)
	other
	other
	other
	signal_peptide(Sec/SPI)
	other

other
signal_peptide(Sec/SPI)
other
other
other
other
other
signal_peptide(Sec/SPI)
other
other
other
other
other
other
other
signal_peptide(Sec/SPI)
other
other
other
other
other
other
other
other
other
signal_peptide(Sec/SPI)
other
other
other
signal_peptide(Sec/SPI)
other
other
other
other
other
other
other
other
other
signal_peptide(Sec/SPI)
signal_peptide(Sec/SPI)
signal_peptide(Sec/SPI)
other

	other
	other
	signal_peptide(Sec/SPI)
	other
	other
	other
521	other
	other
	other
	other
	other
	other
-	other
	other
	other
	other
	other
	signal_peptide(Sec/SPI)
chain varia	signal_peptide(Sec/SPI)
	other
	other
	other
	other
	other
	other
	other
	other
	other
	other
	signal_peptide(Sec/SPI)
	other
	other
	other
	other
	other
	other
	other
	other
	other
	other
	other
	other
	other

other
other
other
other
other
other
other
other
other
other
other
other
other
other
other
other
other
other
other
other
other
other
other
other
other
other
signal_peptide(Sec/SPI)
other
other
other
other
- other
other
other
other
other
other
signal_peptide(Sec/SPI)
other
other
other
other
other

other
other
other
1589 other
other
other
other
other
other
other
other
other
other
signal_peptide(Sec/SPI)
other
signal_peptide(Sec/SPI)
other
other
other
other
other
other
other
other
other
1692 other
other
other
other
other
signal_peptide(Sec/SPI)
other
other
other
other
other
2194 other
other
other
other
other
other
other

signal_peptide(Sec/SPI)
other
other
other
signal_peptide(Sec/SPI)
other
other
signal_peptide(Sec/SPI)
other
other
other
other
signal_peptide(Sec/SPI)
other
other
other
other
other
other
- other
signal_peptide(Sec/SPI)
other
other
other
other
other
other
other
other
other
other
other
other
other
other
other
other
other
other
917 other
other
other
other

-	other
	signal_peptide(Sec/SPI)
	other
	signal_peptide(Sec/SPI)
	other
-	other
	signal_peptide(Sec/SPI)
	other
	signal_peptide(Sec/SPI)
	other
	other
	other
	other
	other
	signal_peptide(Sec/SPI)
	other
	other
	other
	other
	other
	other
	other
	other
	other
	other
	other
	other
	other
	other
	other
	other
	other
	other
	other
-	other
	other
	other
	other
	other
	other
	other
	other
	other

	other
	other
	other
	other
	other
	other
	signal_peptide(Sec/SPI)
	other
	other
	other
-	other
	other
	other
	other
	other
	other
	other
	other
	other
	other
	other
	other
rtial [Mus n	signal_peptide(Sec/SPI)
	signal_peptide(Sec/SPI)
	signal_peptide(Sec/SPI)
	other
	other
	other
	other
	other
	signal_peptide(Sec/SPI)
	other
	other
	other
	other
	other
	other
	other
	other
	other
	other
	other
	other

signal_peptide(Sec/SPI)
other
other
other
other
other
other
other
other
other
other
other
other
other
signal_peptide(Sec/SPI)
other
other
other
other
other
other
other
other
other
other
other
signal_peptide(Sec/SPI)
other
other
other
other
other
other
other
other
signal_peptide(Sec/SPI)
other
signal_peptide(Sec/SPI)
other
other
other
other
other

	other
	other
	other
	signal_peptide(Sec/SPI)
	other
-	other
	other
	other
	other
	signal_peptide(Sec/SPI)
-	other
	other
-	other
	other
	signal_peptide(Sec/SPI)
	other
	other
	other
	other
	other
	other
	other
	other
	other
	other
	other
	other
	other
	other
	other
	other
	other
	other
	other
	other
	other
	other
	other
	other
	other
	other
-	other
	other

	other
	other
	other
	other
	other
	other
	other
	other
	other
	signal_peptide(Sec/SPI)
	other
	other
	other
-	other
	other
	other
-	other
	other
	other
	other
	signal_peptide(Sec/SPI)
	other
	signal_peptide(Sec/SPI)
chain varia	signal_peptide(Sec/SPI)
chain varia	signal_peptide(Sec/SPI)
	other
	other
	other
-	other
	other
	other
	other
	other
	other
	other
	other
	other
	other
	other
	other
1059	other
	other
	other

	other
	other
	other
	other
	other
	other
	other
	other
	other
	other
767	other
	other
	signal_peptide(Sec/SPI)
1014	other
	other
	other
	other
	other
	other
	other
	other
	other
	other
chain varia	signal_peptide(Sec/SPI)
	other
	signal_peptide(Sec/SPI)
	signal_peptide(Sec/SPI)
	other
	other
	signal_peptide(Sec/SPI)
	other
	other
	other
	other
	signal_peptide(Sec/SPI)
	other
	signal_peptide(Sec/SPI)
	other
	other
	other
	other
	other
	signal_peptide(Sec/SPI)

other
other
other
signal_peptide(Sec/SPI)
other
other
other
signal_peptide(Sec/SPI)
other
other
other
signal_peptide(Sec/SPI)
signal_peptide(Sec/SPI)
- other
other
other
other
other
other
signal_peptide(Sec/SPI)
other
other
other
other
other
other
other
other
other
other
signal_peptide(Sec/SPI)
other
other
other
other
other
other
signal_peptide(Sec/SPI)
other
other
signal_peptide(Sec/SPI)
other
other

other
other
other
other
other
other
other
signal_peptide(Sec/SPI)
other
other
other
other
other
signal_peptide(Sec/SPI)
signal_peptide(Sec/SPI)
other
signal_peptide(Sec/SPI)
other
other
other
other
other
signal_peptide(Sec/SPI)
other
other
other
other
other
other
other
other
other
other
other
other
other
other
other
signal_peptide(Sec/SPI)
other
other
other
other

		other
		other
-		other
		other
		signal_peptide(Sec/SPI)
		other
		other
		other
		other
		signal_peptide(Sec/SPI)
		other
		other
		other
		other
		signal_peptide(Sec/SPI)
		other
	5452	other
		other
		other
		other
		other
		other
		other
		other
		other
		other
		other
		other
		other
		other
		other
		other
		other
		other
		other
		other
		other
		other
		other
		other
		other
		other
		other

	other
-	other
	other
	other
	signal_peptide(Sec/SPI)
	other
	other
	other
	signal_peptide(Sec/SPI)
	signal_peptide(Sec/SPI)
	other
	other
	other
	signal_peptide(Sec/SPI)
	other
	other
	other
	other
	other
	other
	other
	other
	other
	other
	signal_peptide(Sec/SPI)
	other
	other
	other
	other
	other
	other
	other
	other
	other
	other
	other
	other
łe monocolo	other
	other
	other
	signal_peptide(Sec/SPI)
	other
	other

other
signal_peptide(Sec/SPI)
other
other
other
other
other
other
other
other
other
signal_peptide(Sec/SPI)
other
signal_peptide(Sec/SPI)
other
other
signal_peptide(Sec/SPI)
other
other
other
other
other
other
other
1914 other
other
other
other
other
signal_peptide(Sec/SPI)
other
other
other
other
other
other
other
other
other
other
other
signal_peptide(Sec/SPI)
other

	other
	other
	other
	other
463	other
	other
	other
	other
	signal_peptide(Sec/SPI)
	signal_peptide(Sec/SPI)
	other
	other
	signal_peptide(Sec/SPI)
	other
	other
	other
	other
	other
	other
	other
3593	other
	other
	other
	other
	other
	other
	other
	other
	other
	other
	other
	other
	other
	other
	other
	other
	other
	other
	other
	other
	other
	other
	other

	other
	other
	signal_peptide(Sec/SPI)
	other
	other
	other
	other
	other
	other
	other
	signal_peptide(Sec/SPI)
	other
56.2	other
	other
261	other
	other
	other
	other
	other
	other
	other
	other
	other
	other
	other
	other
	other
	other
	other
	other
	other
	other
	other
	signal_peptide(Sec/SPI)
	other
	signal_peptide(Sec/SPI)
	signal_peptide(Sec/SPI)
	signal_peptide(Sec/SPI)
	other
	other
	other
	other
	signal_peptide(Sec/SPI)

other
other
signal_peptide(Sec/SPI)
other
other
other
other
other
other
other
other
other
other
other
other
other
other
other
other
signal_peptide(Sec/SPI)
other
other
other
other
other
other
other
other
other
other
other
other
other
other
813 other
other
other
other
other
other
other
other
other

		other
		other
		other
		other
		other
-		other
		signal_peptide(Sec/SPI)
		other
		other
		other
		other
		other
		other
		other
		other
		other
		other
		other
	80.5	other
		other
		other
		other
		other
		signal_peptide(Sec/SPI)
		other
		other
		other
		other
		other
		other
		other
-		other
		other
		other
		other
		signal_peptide(Sec/SPI)
		other
		other
		other
		other
		other
		signal_peptide(Sec/SPI)
		signal_peptide(Sec/SPI)

	other
	other
	other
	other
	other
	other
	other
	signal_peptide(Sec/SPI)
	other
	other
	signal_peptide(Sec/SPI)
	other
	other
	other
	other
	signal_peptide(Sec/SPI)
	other
	other
	other
	other
	other
	other
	signal_peptide(Sec/SPI)
	other
66.6	other
	other
	other
	other
	other
	other
	other
	other
	other
	other
	other
	other
	signal_peptide(Sec/SPI)
	other
	other
	other
	signal_peptide(Sec/SPI)
	other
	other

signal_peptide(Sec/SPI)
other
other
other
other
signal_peptide(Sec/SPI)
other
other
other
signal_peptide(Sec/SPI)
other
signal_peptide(Sec/SPI)
110 other
- other
other
signal_peptide(Sec/SPI)
other
signal_peptide(Sec/SPI)
other
other
other
other
other
other
other
other
other
other
other
other
signal_peptide(Sec/SPI)
other
other
signal_peptide(Sec/SPI)
signal_peptide(Sec/SPI)
other
other
other
other
other
other
other
signal_peptide(Sec/SPI)

other
other
other
other
other
other
signal_peptide(Sec/SPI)
signal_peptide(Sec/SPI)
other
signal_peptide(Sec/SPI)
other
signal_peptide(Sec/SPI)
other
other
other
other
other
other
other
other
other
signal_peptide(Sec/SPI)
other
other
other
other
other
other
other
other
other
other
other
other
signal_peptide(Sec/SPI)
signal_peptide(Sec/SPI)
other
other
other
1010 other
other
signal_peptide(Sec/SPI)
signal_peptide(Sec/SPI)

	other
	other
	other
	other
	other
	signal_peptide(Sec/SPI)
	other
	other
	other
	other
	other
-	other
	other
	signal_peptide(Sec/SPI)
	other
	other
	signal_peptide(Sec/SPI)
	other
	other
	signal_peptide(Sec/SPI)
	other
	other
	other
	other
	other
	other
	other
	other
	other
	other
	other
	other
	other
	other
	signal_peptide(Sec/SPI)
	other
	other
	other
	other
	other
	other
	other
	other

	other
	other
	other
	other
	other
	other
	other
	other
	other
	signal_peptide(Sec/SPI)
	signal_peptide(Sec/SPI)
	other
	other
	other
	other
	other
	other
	other
	other
	other
	other
	other
	other
	other
	other
	other
	other
	other
	other
	signal_peptide(Sec/SPI)
	other
	other
	other
	other
	other
-	other
	other
	other
	signal_peptide(Sec/SPI)
-	other
	other
	signal_peptide(Sec/SPI)
	other

other
other
other
other
other
other
signal_peptide(Sec/SPI)
other
other
other
other
other
other
other
other
other
other
other
signal_peptide(Sec/SPI)
other
other
other
other
other
other
other
other
other
signal_peptide(Sec/SPI)
signal_peptide(Sec/SPI)
signal_peptide(Sec/SPI)
other
other
other
other
other
other
other
other
signal_peptide(Sec/SPI)
other
other
other

		signal_peptide(Sec/SPI)
		other
-		other
		other
		other
	1720	other
		other
		other
		signal_peptide(Sec/SPI)
		other
		signal_peptide(Sec/SPI)
	190	other
		other
		other
		other
		other
		other
		other
		other
		other
		other
		signal_peptide(Sec/SPI)
		other
		signal_peptide(Sec/SPI)
		other
		signal_peptide(Sec/SPI)
		other
		other
		signal_peptide(Sec/SPI)
		other
		other
		other
		other
		other
		other
		other
		other
		other
		other
		other
	4055	other
		other
		other

		other
		other
		other
		other
		other
	75.1	other
	1951	other
		other
		other
		other
		other
		other
	224	other
		other
		other
		other
		other
		other
		other
		other
		signal_peptide(Sec/SPI)
	305	other
		other
		other
		other
		signal_peptide(Sec/SPI)
		other
		other
		other
		other
		other
		other
		other
-		other
		other
		other
		other
		other
		other
-		other
		other
		other
		other

		other
		other
		other
		other
		other
		other
		other
		other
		other
		other
		other
		other
-		other
		other
		other
		other
		other
		signal_peptide(Sec/SPI)
		other
		other
		other
		signal_peptide(Sec/SPI)
		other
-		other
		other
		other
		signal_peptide(Sec/SPI)
	412	other
	754	other
		other
		other
-		other
		other
		other
-		other
		other
		other
		other
		other
		other
		other
		signal_peptide(Sec/SPI)
		other

signal_peptide(Sec/SPI)
other
other
other
signal_peptide(Sec/SPI)
other
other
other
signal_peptide(Sec/SPI)
other
other
other
signal_peptide(Sec/SPI)
2276 other
other
other
other
other
other
other
signal_peptide(Sec/SPI)
other
other
signal_peptide(Sec/SPI)
signal_peptide(Sec/SPI)
signal_peptide(Sec/SPI)
other
other
other
other
other
other
other
other
other
other
other
other
other
other
other
signal_peptide(Sec/SPI)
other

other
other
other
other
other
other
other
other
other
signal_peptide(Sec/SPI)
other
other
other
other
other
other
other
other
signal_peptide(Sec/SPI)
other
other
other
other
other
other
other
other
signal_peptide(Sec/SPI)
other
701 other
other
other
other
other
other
other
other
signal_peptide(Sec/SPI)
signal_peptide(Sec/SPI)
other
other
signal_peptide(Sec/SPI)
other

signal_peptide(Sec/SPI)
other
other
other
other
other
other
other
other
other
other
other
69.3 other
other
signal_peptide(Sec/SPI)
other
signal_peptide(Sec/SPI)
other
other
other
other
other
signal_peptide(Sec/SPI)
other
other
other
signal_peptide(Sec/SPI)
other
other
other
other
other
other
other
other
other
other
signal_peptide(Sec/SPI)
other
signal_peptide(Sec/SPI)
other
other
other

	other
	other
	signal_peptide(Sec/SPI)
	other
	other
	other
	other
	signal_peptide(Sec/SPI)
	other
737	other
	signal_peptide(Sec/SPI)
	other
OC1287066(	other
ıble region,	other
	other
	other
	signal_peptide(Sec/SPI)
	signal_peptide(Sec/SPI)
	other
	other
	other
	other
	other
	other
	other
	other
	other
	other
	other
	other
	other
	other
	other
	other
	other
	other
	other
	other
	other
	other
	other
	other
	other

	other
	other
	signal_peptide(Sec/SPI)
	other
	other
	signal_peptide(Sec/SPI)
	other
	other
	other
	other
	other
	other
	other
	other
	other
	other
	other
	other
	other
	other
	other
	other
	other
	other
931	other
	other
	other
	other
	signal_peptide(Sec/SPI)
	other
	other
	other
	other
	other
	other
	signal_peptide(Sec/SPI)
	other
	other
	other
	other
	other
	other
-	other

		other
		other
		other
		other
		signal_peptide(Sec/SPI)
		other
		signal_peptide(Sec/SPI)
		other
		other
		other
		other
		signal_peptide(Sec/SPI)
		other
		other
		other
		other
		other
		other
		other
	251	other
		other
		signal_peptide(Sec/SPI)
		other
		other
		other
-		other
		other
		other
		other
		signal_peptide(Sec/SPI)
		other
		other
		other
		other
	1288	other
		other
		signal_peptide(Sec/SPI)
		other
		other
		other
		other
		other
		other

	other
	other
	other
	other
	other
	other
	other
	other
	other
	other
A regulatory	other
	other
	other
	other
	other
	other
	other
	other
	other
	other
	other
	other
	other
	other
	other
	other
79	other
	other
	other
	other
	other
	other
	other
	other
	other
	signal_peptide(Sec/SPI)
	signal_peptide(Sec/SPI)
	signal_peptide(Sec/SPI)
-	other
	other
	other
	other
	other

		other
		other
	1600	other
		other
		other
		other
		other
		other
		other
		other
		other
		other
		other
		other
		other
		other
		other
		other
		signal_peptide(Sec/SPI)
		other
		other
		other
		other
		other
		other
		other
		other
		other
		other
		other
		other
		other
-		other
		signal_peptide(Sec/SPI)
		signal_peptide(Sec/SPI)
		other
		other
		other
-		other
		other
		other
		other

	other
	other
	other
	other
	other
	other
	other
	other
	other
	other
	other
	other
uct [Mus mu	other
	other
	other
	other
	other
	other
	other
	signal_peptide(Sec/SPI)
	other
	other
	signal_peptide(Sec/SPI)
	other
	other
	other
	other
	other
	other
	other
	other
	other
	other
	other
	signal_peptide(Sec/SPI)
	signal_peptide(Sec/SPI)
	other
	other
	other
	signal_peptide(Sec/SPI)
	other
	other
	other

	other
	other
	other
-	other
	other
	other
	other
	other
	other
	other
	other
	other
	signal_peptide(Sec/SPI)
	signal_peptide(Sec/SPI)
	other
	other
	other
	other
	other
	other
	other
	other
	signal_peptide(Sec/SPI)
	other
	other
	signal_peptide(Sec/SPI)
	signal_peptide(Sec/SPI)
	other
	other
	signal_peptide(Sec/SPI)
	other
	other
	signal_peptide(Sec/SPI)
	other
	other
	other
	other
	other
	other
	other
	other
	other
	other

	other
	other
	other
	other
	signal_peptide(Sec/SPI)
	other
	other
	signal_peptide(Sec/SPI)
	other
-	other
	other
	signal_peptide(Sec/SPI)
	other
	other
	other
	other
	signal_peptide(Sec/SPI)
	other
	signal_peptide(Sec/SPI)
-	other
	other
	other
	other
	other
	other
	other
	other
	other
	other
	other
	other
	other
	other
	other
	other
	other
	other
	other
	other
	other
	other
	other
	other

		other
		other
	1558	other
-		other
		other
		other
		other
		other
		other
		other
		other
		signal_peptide(Sec/SPI)
		other
		other
		other
		other
		other
		other
	921	other
		other
		other
		other
		signal_peptide(Sec/SPI)
		other
		other
		other
		other
		other
		other
		signal_peptide(Sec/SPI)
		other
		other
		other
		other
	461	other
		other
		signal_peptide(Sec/SPI)
		other
		other
		other
		other
		other
		other

	other
	other
	other
	other
-	other
	other
	other
	other
	other
	other
	other
	other
	other
	other
	other
	other
	other
	other
	signal_peptide(Sec/SPI)
	other
	signal_peptide(Sec/SPI)
	other
	signal_peptide(Sec/SPI)
	other
	other
	signal_peptide(Sec/SPI)
	other
	other
	signal_peptide(Sec/SPI)
	other
	other
	other
	other
	other
	other
	other
	other
	other
	other
	other
	other
	other
117	other

		other
-		other
		other
		other
	632	signal_peptide(Sec/SPI)
		signal_peptide(Sec/SPI)
		other
		other
		other
		other
		other
		other
		other
		other
-		other
-		other
		other
-		other
		other
		other
		other
		other
		other
		other
		other
		other
		other
		other
		other
		other
		other
		signal_peptide(Sec/SPI)
	1291	other
		other
		other
		other
		other
		other
		other
		other
		other
		other
		other

		other
		other
		other
	52	other
		other
		other
		other
-		other
		other
		other
		other
		signal_peptide(Sec/SPI)
		other
		other
		other
		other
		signal_peptide(Sec/SPI)
		other
		signal_peptide(Sec/SPI)
		other
		other
		other
		other
		other
		other
		other
		other
		other
	78.6	other
		other
		other
		other
		other
		other
		other
		other
		other
		other
		other
		signal_peptide(Sec/SPI)
		other
		signal_peptide(Sec/SPI)
	1982	other

	other
	other
	other
	other
	other
	other
	signal_peptide(Sec/SPI)
	other
	other
	other
	other
-	other
	other
	other
	other
	other
	other
	other
	signal_peptide(Sec/SPI)
	other
	other
	other
	other
	other
	other
	other
	other
	other
	other
	other
	other
	other
	other
	other
	other
	signal_peptide(Sec/SPI)
	other
	other
	other
	other
	other
-	other
	signal_peptide(Sec/SPI)

		other
		other
		other
		other
		other
		other
		other
		other
		other
		other
		other
		other
		other
	1190	other
	1787	other
		other
		other
		other
		other
-		other
-		other
		other
		other
		other
		other
		other
		other
		other
		other
		signal_peptide(Sec/SPI)
		other
		other
		signal_peptide(Sec/SPI)
		other
		other
		other
		other
		other
		other
		other
		other
		other
		other

		other
		other
		other
		other
		other
		signal_peptide(Sec/SPI)
		other
		other
		other
		signal_peptide(Sec/SPI)
		other
		other
		other
		other
		other
		other
	1258	other
		other
		other
		other
		other
		other
		other
		signal_peptide(Sec/SPI)
		other
		other
		other
		other
		other
		other
		other
		other
		other
-		other
		other
		other
		other
		other
		other
		signal_peptide(Sec/SPI)
		other
-		other
		other

	other
	signal_peptide(Sec/SPI)
-	other
	other
	other
	other
	other
	other
	other
	other
	other
	signal_peptide(Sec/SPI)
	signal_peptide(Sec/SPI)
	signal_peptide(Sec/SPI)
	other
	other
	other
	other
	other
	other
	other
	other
	other
	signal_peptide(Sec/SPI)
	other
	other
	other
	other
	other
	signal_peptide(Sec/SPI)
	signal_peptide(Sec/SPI)
	other
	other
	other
	other
	other
	other
	other
	other
	other
-	other
	other
	signal_peptide(Sec/SPI)

		other
		other
		other
		signal_peptide(Sec/SPI)
		signal_peptide(Sec/SPI)
		other
		other
		other
		other
		other
		other
		other
		signal_peptide(Sec/SPI)
		other
		other
		other
		other
		other
		signal_peptide(Sec/SPI)
		other
		other
-		other
		other
		other
		other
		signal_peptide(Sec/SPI)
		other
		other
		other
		other
		signal_peptide(Sec/SPI)
		signal_peptide(Sec/SPI)
		other
		other
		other
	818	other
		other
		signal_peptide(Sec/SPI)
		other
		other
		other
		other
-		other

		other
		other
		other
		other
		other
		other
		other
		other
		other
		other
		other
		other
		other
		other
		other
		other
		other
		other
		other
		other
		other
		other
		other
		other
	50.4	other
		other
	480	other
		other
		other
		other
		other
		other
-		other
		other
-		other
		other
		other
		other
		other
		signal_peptide(Sec/SPI)
		other
		other
		other

	other
	other
	other
	other
	other
	other
	other
	other
	other
	other
	other
	other
	other
	other
	other
	other
	other
	other
	other
	other
	other
	other
	other
	other
	other
	other
	other
	other
	other
-	other
	other
	other
	other
	other
	other
-	other
	other
	other
	other
	other
	other
	other
-	other

	other
-	other
	other
78.6	other
	other
	other
	other
	other
	other
luct, partial	other
	other
	other
	other
	other
	other
	other
	other
	signal_peptide(Sec/SPI)
	other
	other
	other
	other
	other
	other
	signal_peptide(Sec/SPI)
	other
	other
	other
	other
-	other
-	other
	other
	other
\ polymeras	other
	other
-	other
	other
	other
	signal_peptide(Sec/SPI)
	signal_peptide(Sec/SPI)
	other
	other
	signal_peptide(Sec/SPI)

other
other
other
other
other
other
other
other
other
other
other
other
other
other
other
other
other
other
other
other
other
other
other
signal_peptide(Sec/SPI)
other
other
other
other
other
other
other
signal_peptide(Sec/SPI)
other
other
other
other
other
other
signal_peptide(Sec/SPI)
other
other
other
other

	other
	other
	other
	other
	other
	other
	other
	other
	other
	other
	other
	other
	other
	signal_peptide(Sec/SPI)
	signal_peptide(Sec/SPI)
	other
	other
	other
	other
	other
	other
-	other
	other
	other
	other
	other
	other
	other
	other
	other
	other
	other
	other
	other
	other
	other
	other
	other
	other
	signal_peptide(Sec/SPI)
	other
	other

other
other
signal_peptide(Sec/SPI)
other
other
other
signal_peptide(Sec/SPI)
other
other
signal_peptide(Sec/SPI)
signal_peptide(Sec/SPI)
signal_peptide(Sec/SPI)
other
other
other
other
other
other
other
other
other
other
other
other
signal_peptide(Sec/SPI)
other
other
- other
signal_peptide(Sec/SPI)
other
other
other
other
other
other
other
other
other
other
other
other
other
other

-	other
	other
	other
	other
	other
	other
	other
	other
	other
	other
	other
	other
	other
	other
	other
	other
	other
	other
	other
	other
	other
	other
	other
	other
	other
	other
	other
	other
	other
	other
	other
	other
	other
	other
	other
	signal_peptide(Sec/SPI)
	other
	other
	other
	other
	other
	other
	other

	other
	other
	other
	other
	other
	other
	other
	other
	other
	other
	other
	signal_peptide(Sec/SPI)
	other
	other
	other
	other
	other
	other
	other
	other
	other
	signal_peptide(Sec/SPI)
	other
	signal_peptide(Sec/SPI)
	other
	other
	other
	other
	other
	signal_peptide(Sec/SPI)
	other
	other
	other
	other
-	other
	other
	other
	other
	other
	other
	other
	other
	other

other
other
other
other
other
other
other
signal_peptide(Sec/SPI)
signal_peptide(Sec/SPI)
other
other
other
signal_peptide(Sec/SPI)
other
signal_peptide(Sec/SPI)
other
other
other
other
other
signal_peptide(Sec/SPI)
other
other
other
other
other
signal_peptide(Sec/SPI)
other
other
other
other
other
other
other
other
other
other
other
other
other
signal_peptide(Sec/SPI)
other
other

	other
	other
	other
-	other
	other
	other
	other
	other
	other
	signal_peptide(Sec/SPI)
	other
	other
	other
	other
	other
	other
	other
	other
	other
	other
	other
	other
	other
	other
	other
	other
	signal_peptide(Sec/SPI)
	other
	other
	other
	other
	signal_peptide(Sec/SPI)
	other
	other
	other
	other
	other
	other
	other
	other
	other
	other
417	other

other
other
other
other
other
other
signal_peptide(Sec/SPI)
other
other
other
other
other
other
other
other
other
other
other
other
other
other
other
other
other
other
signal_peptide(Sec/SPI)
other
other
other
other
other
other
other
other
other
other
other
391 other
other
signal_peptide(Sec/SPI)
other
other
other

	other
	other
	other
	other
	signal_peptide(Sec/SPI)
	signal_peptide(Sec/SPI)
	other
	other
-	other
	other
	signal_peptide(Sec/SPI)
	other
	other
	other
	other
	signal_peptide(Sec/SPI)
	other
	other
	other
-	other
	other
	other
	other
	other
	signal_peptide(Sec/SPI)
	other
	other
	signal_peptide(Sec/SPI)
	other
	other
	signal_peptide(Sec/SPI)
	other
	other
	other
	other
	other
	other
	other
	other
	other
	other
	other
	other

other
other
- other
other
- other
other
other
other
other
other
other
other
other
other
other
other
other
other
other
- other
other
other
other
other
other
other
other
other
other
other
other
- other
other
other
- other
other
other
other
other
- other
other
other
other

	other
	other
-	other
	other
	other
	other
	other
	other
	other
	other
	other
	other
	other
	other
	other
	signal_peptide(Sec/SPI)
	other
	signal_peptide(Sec/SPI)
	other
	other
	other
	other
-	other
	other
	other
	other
	other
	other
	signal_peptide(Sec/SPI)
	other
	other
	other
	other
	other
	other
	signal_peptide(Sec/SPI)
	other
	other
	other
	other
	other
	other
	other

	other
-	other
	other
	other
	other
	other
	signal_peptide(Sec/SPI)
	other
	other
	other
2556	other
	other
	other
	other
	other
	other
	other
	other
	other
	other
	other
	other
	other
	other
	other
	signal_peptide(Sec/SPI)
	other
	other
	other
	other
	other
	other
	other
	other
	other
	other
	other
	other
	other
	other
	other
	other
	other

other
signal_peptide(Sec/SPI)
other
other
other
other
signal_peptide(Sec/SPI)
other
other
signal_peptide(Sec/SPI)
signal_peptide(Sec/SPI)
other
other
other
other
other
other
signal_peptide(Sec/SPI)
other
other
other
other
other
other
other
other
other
other
other
other
other
other
other
other
other
other
- other
other
other
other
3062 other
other
other

	other
	other
	other
	other
	other
	other
	other
	other
	other
	other
	signal_peptide(Sec/SPI)
	other
	other
-	other
	other
	other
	other
	other
	signal_peptide(Sec/SPI)
	other
	other
	signal_peptide(Sec/SPI)
	other
	other
	other
	other
	other
	other
	other
	other
	other
	other
	other
	other
	other
	other
	other
	other
	other
	signal_peptide(Sec/SPI)
	other
	other
	other

	other
	other
	other
	other
	other
	other
	other
	signal_peptide(Sec/SPI)
	other
	other
	other
	other
	other
	other
	signal_peptide(Sec/SPI)
	other
	other
	other
	other
	other
	other
	other
	signal_peptide(Sec/SPI)
	signal_peptide(Sec/SPI)
	other
	other
	other
	other
	other
-	other
	other
	other
	other
	other
-	other
	other
	other
	other
	signal_peptide(Sec/SPI)
	signal_peptide(Sec/SPI)
	other
	other
	other

other
other
other
other
signal_peptide(Sec/SPI)
other
other
other
other
other
other
other
other
other
other
other
signal_peptide(Sec/SPI)
other
other
other
other
other
other
other
other
other
other
other
other
other
signal_peptide(Sec/SPI)
other
other
other
other
other
other
other
other
other
- other
other
other

		other
		other
		other
		other
		other
		other
		other
		other
		other
		other
		other
		signal_peptide(Sec/SPI)
		other
		other
-		other
		other
		other
-		other
		signal_peptide(Sec/SPI)
		other
		other
		other
		signal_peptide(Sec/SPI)
		other
		other
		other
		other
		other
		other
		other
		other
		other
	1362	other
		other
		other
		other
		other
		other
-		other
		signal_peptide(Sec/SPI)
		other
		other
		signal_peptide(Sec/SPI)

other
other
other
other
other
other
other
- other
other
signal_peptide(Sec/SPI)
other
other
other
other
other
other
other
signal_peptide(Sec/SPI)
other
other
other
signal_peptide(Sec/SPI)
other
other
other
other
other
other
other
other
other
signal_peptide(Sec/SPI)
signal_peptide(Sec/SPI)
signal_peptide(Sec/SPI)
other
other
other
signal_peptide(Sec/SPI)
other
other
other
other
other

		other
		other
		other
-		other
		other
		other
		other
		other
		other
		other
		other
		other
		other
		other
		other
		other
		other
		other
		other
		other
		other
		other
		signal_peptide(Sec/SPI)
		other
		other
	89.7	other
		other
		other
		other
		signal_peptide(Sec/SPI)
		other
		other
		other
		other
		other
		other
		other
		other
		other
		other
		other
		signal_peptide(Sec/SPI)
		other

other
other
other
other
other
other
other
other
other
other
other
other
other
other
signal_peptide(Sec/SPI)
other
other
other
other
other
610 other
other
other
other
other
other
other
other
other
other
other
other
other
other
other
other
other
other
- other
other
other
other
other

		other
		signal_peptide(Sec/SPI)
		signal_peptide(Sec/SPI)
	1815	other
		other
		other
		other
		other
		signal_peptide(Sec/SPI)
		other
		other
		other
		other
-		other
		other
		other
		other
		other
	70.9	other
		other
		other
		other
-		other
		other
		signal_peptide(Sec/SPI)
		other
		other
		other
		other
		other
		other
-		other
		other
	154	other
		other
		other
		other
		other
		other
		other
-		other
		other
		other

	other
	other
	other
	other
	other
	signal_peptide(Sec/SPI)
	other
	other
	other
	other
	other
	signal_peptide(Sec/SPI)
	other
	other
	other
	other
	other
	other
	signal_peptide(Sec/SPI)
	other
	signal_peptide(Sec/SPI)
	other
	other
	other
	other
	other
	other
	other
	other
	signal_peptide(Sec/SPI)
	other
	other
	other
	other
	other
	signal_peptide(Sec/SPI)
	other
nusculus])	other
	other
	other
	other
-	other
	signal_peptide(Sec/SPI)

other
signal_peptide(Sec/SPI)
other
other
other
other
other
other
other
other
other
other
other
other
other
other
other
other
other
other
other
other
56.2 other
other
other
other
other
other
other
other
other
other
other
other
other
other
signal_peptide(Sec/SPI)
other
other
other
other
other
other

	other
	other
	other
	other
	other
-	other
	other
	other
	other
	other
	other
	other
	other
	other
	other
	other
	other
-	other
	other
	other
	other
	other
	signal_peptide(Sec/SPI)
	other
	other
	other
	other
	signal_peptide(Sec/SPI)
	other
	signal_peptide(Sec/SPI)
	other
	other
	other
	other
	other
	signal_peptide(Sec/SPI)
	other
	other
	other
	signal_peptide(Sec/SPI)
	other
	other
	other

-		other
		other
		other
		other
		other
		other
		other
		signal_peptide(Sec/SPI)
		other
	963	other
		other
		other
		other
		other
		other
		other
		other
		other
		other
		other
		other
		other
-		other
		other
		other
		signal_peptide(Sec/SPI)
		other
		signal_peptide(Sec/SPI)
		other
		signal_peptide(Sec/SPI)
		other
		other
		other
		other
		other
		other
		other
		other
		other
		other
		other
		other
		other

	other
	other
	other
	other
	other
-	other
	other
	other
	other
	other
	signal_peptide(Sec/SPI)
	other
	other
	other
	other
	other
	other
	other
	other
	other
	other
	other
	other
	other
	other
	signal_peptide(Sec/SPI)
	other
	other
	other
	other
	other
	other
	signal_peptide(Sec/SPI)
	other
	other
	other
	signal_peptide(Sec/SPI)
	other
	other
	other
	other
	other
	other

	other
	signal_peptide(Sec/SPI)
	other
	other
	other
	other
	other
	other
	other
	other
	other
	other
	signal_peptide(Sec/SPI)
	other
	other
	other
	other
	other
	signal_peptide(Sec/SPI)
	other
	other
	other
	other
	other
	other
uct, partial	other
	other
	other
	signal_peptide(Sec/SPI)
-	other
	signal_peptide(Sec/SPI)
807	other
	other
	other
	signal_peptide(Sec/SPI)
	signal_peptide(Sec/SPI)
	other
	other
	other
	other
	other
	other
	other

		other
		other
		other
		other
		other
		other
		other
		other
		signal_peptide(Sec/SPI)
		other
		other
		other
		other
		other
		signal_peptide(Sec/SPI)
		other
		other
		other
		other
-		other
		other
		other
		other
		other
-		other
-		other
		other
		other
		other
		other
		other
	279	other
		other
		other
		other
-		other
		signal_peptide(Sec/SPI)
		signal_peptide(Sec/SPI)
		other
		other
		other
		other
		signal_peptide(Sec/SPI)

		other
		other
		other
		other
		signal_peptide(Sec/SPI)
		other
-		other
		other
		other
-		other
		other
	790	other
-		other
-		other
		other
		other
		signal_peptide(Sec/SPI)
		other
		other
		other
		other
		other
-		other
		other
		signal_peptide(Sec/SPI)
		other
		other
		other
		other
		other
		other
		other
		other
		other
		other
		other
		other
		other
		other
		other
		other
	1587	other
		other

	other
	other
	other
	other
	other
	other
	other
	other
	other
	other
	signal_peptide(Sec/SPI)
	other
	other
	other
	other
	other
	other
	other
	other
	other
-	other
-	other
	other
	signal_peptide(Sec/SPI)
-	other
	other
	other
	other
	other
	other
	other
	other
	other
	signal_peptide(Sec/SPI)
	other
	signal_peptide(Sec/SPI)
	other
	other
	other
	other
	other
	other
	other

	other
	other
	other
	other
-	other
	other
	other
	other
	other
	other
	other
	other
	other
	other
	other
	other
	other
	other
	other
	other
	other
	other
	signal_peptide(Sec/SPI)
	other
	other
	other
	other
	other
	other
	other
	other
	other
	other
	signal_peptide(Sec/SPI)
chain V reg	other
	other
	signal_peptide(Sec/SPI)
	other
	other
	other
	other
	other
	other

signal_peptide(Sec/SPI)
other
other
other
other
other
other
other
other
other
other
other
signal_peptide(Sec/SPI)
other
other
other
other
other
other
other
other
other
other
other
other
other
other
other
other
other
other
other
other
other
other
other
other
other
other
other
other
other
other

	signal_peptide(Sec/SPI)
	other
	other
	other
	other
	signal_peptide(Sec/SPI)
	other
	other
	other
	other
	other
	other
	other
	other
	other
	other
	other
	other
-	other
	other
-	other
	other
	other
	other
	other
	other
	other
	other
	other
	other
	other
	other
	other
	other
	other
	other
	other
	other
	other
	signal_peptide(Sec/SPI)
	other
	other

		other
		other
		signal_peptide(Sec/SPI)
		other
		other
		signal_peptide(Sec/SPI)
		other
		other
		signal_peptide(Sec/SPI)
		other
		other
		other
		signal_peptide(Sec/SPI)
		signal_peptide(Sec/SPI)
	464	other
		signal_peptide(Sec/SPI)
		other
		other
		other
		other
		other
		other
		other
		other
		other
		other
		other
-		other
		other
		other
		other
		other
		other
		other
		other
		other
		other
		other
		other
		signal_peptide(Sec/SPI)
		other
		other
		signal_peptide(Sec/SPI)

	other
ry chain vari	other
	other
	other
	other
	other
	signal_peptide(Sec/SPI)
	other
	other
	other
	other
	other
	other
	other
	other
	other
-	other
	other
	other
	other
	other
	other
	other
	other
	other
	other
	other
	other
	other
	other
	other
	other
	other
	other
	other
	other
	other
	other
	other
	other
	other
-	signal_peptide(Sec/SPI)
	other

other
other
other
other
other
other
signal_peptide(Sec/SPI)
other
246 other
other
signal_peptide(Sec/SPI)
other
other
other
other
other
other
other
other
other
signal_peptide(Sec/SPI)
other
other
other
other
other
other
signal_peptide(Sec/SPI)
other
signal_peptide(Sec/SPI)
other
other
signal_peptide(Sec/SPI)
signal_peptide(Sec/SPI)
other
signal_peptide(Sec/SPI)
374 other
other
other
other
other
other
other

other
signal_peptide(Sec/SPI)
other
other
other
other
signal_peptide(Sec/SPI)
other
other
other
other
other
signal_peptide(Sec/SPI)
other
other
other
other
other
other
other
other
other
other
other
other
other
other
other
other
other
other
other
other
signal_peptide(Sec/SPI)
other
other
other
signal_peptide(Sec/SPI)
other
other
other
other

		signal_peptide(Sec/SPI)
	70.9	other
		other
		other
-		other
		other
		other
		other
		other
		other
		other
		signal_peptide(Sec/SPI)
		other
-		other
		other
		other
-		other
		other
		other
		other
		other
		other
		other
		signal_peptide(Sec/SPI)
		other
		other
		signal_peptide(Sec/SPI)
		other
		signal_peptide(Sec/SPI)
		other
		other
		other
		other
		other
		other
		other
		other
		signal_peptide(Sec/SPI)
		other
		other
		signal_peptide(Sec/SPI)
		other
		other

		other
	84	other
		other
		other
		other
		other
		signal_peptide(Sec/SPI)
		other
		other
		signal_peptide(Sec/SPI)
		other
		signal_peptide(Sec/SPI)
		other
		other
		other
		other
		other
		other
		other
-		other
		other
		other
		other
		other
		signal_peptide(Sec/SPI)
		other
		other
		other
		other
		other
		other
	768	other
		other
		other
		other
		other
		other
		other
		other
		other
		other
		signal_peptide(Sec/SPI)
-		other

other
other
other
other
signal_peptide(Sec/SPI)
other
signal_peptide(Sec/SPI)
other
other
other
other
signal_peptide(Sec/SPI)
other
other
other
other
other
other
other
other
other
other
other
signal_peptide(Sec/SPI)
other
other
other
other
other
other
other
other
other
other
other
other
other
other
other
other
other
other
other

	other
-	other
	other
	other
	other
	other
	other
	other
	other
	other
	other
	other
	other
	other
	other
	other
	other
	other
	signal_peptide(Sec/SPI)
	other
	other
	other
	other
	other
	other
	other
	other
	other
	other
-	other
	other
	other
	signal_peptide(Sec/SPI)
	other
	other
	other
	other
	other
	other
	other
	other
	other
	other

other
other
other
other
other
other
other
other
other
other
other
other
other
other
other
other
other
other
other
other
other
other
other
other
other
other
other
other
other
other
other
other
other
other
other
other
other
other
other
other
other
other
other

other
other
other
other
signal_peptide(Sec/SPI)
other
other
signal_peptide(Sec/SPI)
other
other
other
signal_peptide(Sec/SPI)
other
other
other
other
other
other
other
other
other
other
other
other
signal_peptide(Sec/SPI)
other
other
other
other
other
other
other
other
other
other
other
other
other
other
- other
other
other
signal_peptide(Sec/SPI)

		other
		other
		other
		other
		other
		other
		signal_peptide(Sec/SPI)
		other
		signal_peptide(Sec/SPI)
		other
		other
		other
		other
		other
-		other
	527	other
		other
		other
		other
		other
		other
		other
		other
		other
		other
		other
		other
		other
		other
		other
		other
	209	other
		signal_peptide(Sec/SPI)
		other
		other
		signal_peptide(Sec/SPI)
		other
		other
		other
		other
		other
		other
		other

	other
	other
	other
	signal_peptide(Sec/SPI)
	other
	other
-	other
	other
	other
	other
	other
	other
	other
	other
-	other
	other
	other
	other
	other
	other
	other
	other
	other
	other
	other
	other
	other
	other
	other
	other
	other
	signal_peptide(Sec/SPI)
	other
	other
	other
	other
	other
	other
	other
	other
	other
	other
	other

		signal_peptide(Sec/SPI)
		other
		other
		other
-		other
		other
		other
		other
		other
		signal_peptide(Sec/SPI)
		other
		other
		signal_peptide(Sec/SPI)
		other
		signal_peptide(Sec/SPI)
		other
-		other
		other
		other
		other
	987	other
		other
		other
		other
		other
		signal_peptide(Sec/SPI)
		other
		other
		other
		other
		other
		other
		other
		other
		other
		other
		other
		other
		other
		other
		other
		other
		other

	other
	other
	other
	other
	other
	other
	other
	other
	other
	other
	other
	signal_peptide(Sec/SPI)
	other
	other
corepressor	other
	other
	other
	other
-	other
	other
	other
	signal_peptide(Sec/SPI)
	other
	other
	other
	other
	other
	other
	other
	other
	signal_peptide(Sec/SPI)
	other
	other
	other
	other
	other
	other
	other
	other
	other
	other
	other
	other

		other
		other
		signal_peptide(Sec/SPI)
		signal_peptide(Sec/SPI)
		other
-		other
		other
		other
		other
		other
		other
		other
		other
	79.7	other
		other
		other
		other
		other
		other
		other
		other
		other
		other
		other
		other
		other
		other
		other
		other
		other
		other
		other
-		other
		other
		other
-		other
	692	other
		other
		other
		other
		other
		other
		other

	other
	other
-	other
	other
	other
	other
	other
	other
	other
	other
	other
	other
	other
-	other
	other
	other
	other
	other
	other
	other
	other
	other
	other
	other
	other
	other
	other
	other
	other
	other
	other
	other
	other
	signal_peptide(Sec/SPI)
	other
	other
	other
	other
	other
	other
	other
	other
	other

signal_peptide(Sec/SPI)
other
other
other
other
other
other
other
other
signal_peptide(Sec/SPI)
other
signal_peptide(Sec/SPI)
other
other
other
other
other
signal_peptide(Sec/SPI)
other
other
other
other
other
other
signal_peptide(Sec/SPI)
other
other
other
other
other
other
other
other
other
other
other
other
other
other
other
- other
other
other

	other
	other
	signal_peptide(Sec/SPI)
	other
	other
445	other
	other
	other
	other
	other
	other
	other
	other
	other
	other
	other
	other
	other
	other
	other
	other
	other
y chain vari	signal_peptide(Sec/SPI)
	other
	signal_peptide(Sec/SPI)
	signal_peptide(Sec/SPI)
	other
	other
	other
	other
	other
	other
	other
	other
	other
	other
	signal_peptide(Sec/SPI)
	signal_peptide(Sec/SPI)
	other
	other
	other
	other
	other

	other
	other
	other
	other
	other
	other
	other
	other
	other
	other
	other
	other
e nucleotide	other
	other
	other
	other
	other
	other
	other
	other
	signal_peptide(Sec/SPI)
	other
	other
	other
	other
	other
	other
	other
	other
	other
	signal_peptide(Sec/SPI)
	other
	other
	other
	other
	other
	signal_peptide(Sec/SPI)
	other
	other
	other
	other
	signal_peptide(Sec/SPI)
	other

	signal_peptide(Sec/SPI)
	other
	other
	signal_peptide(Sec/SPI)
	other
	signal_peptide(Sec/SPI)
	other
	signal_peptide(Sec/SPI)
	signal_peptide(Sec/SPI)
	other
	other
	signal_peptide(Sec/SPI)
	other
-	other
	other
	other
	other
	other
	other
	other
	other
	other
	other
	other
	signal_peptide(Sec/SPI)
	other
-	other
	signal_peptide(Sec/SPI)
	signal_peptide(Sec/SPI)
	other
	other
	other
-	other
	other
	other
	other
	other
	other
-	other
	other
	other
	other
	other

signal_peptide(Sec/SPI)
other
other
other
signal_peptide(Sec/SPI)
other
other
other
other
other
other
other
other
other
other
other
other
other
other
other
other
other
other
other
signal_peptide(Sec/SPI)
signal_peptide(Sec/SPI)
other
other
other
other
signal_peptide(Sec/SPI)
other
other
other
other
other
other
other
other
signal_peptide(Sec/SPI)
other
other
other

		other
		other
		signal_peptide(Sec/SPI)
		other
		signal_peptide(Sec/SPI)
		other
		other
		signal_peptide(Sec/SPI)
		other
		other
		other
		other
		other
		other
-		other
		other
		other
		signal_peptide(Sec/SPI)
		signal_peptide(Sec/SPI)
	944	other
		signal_peptide(Sec/SPI)
		other
		other
		other
		other
		other
		other
		other
		other
		other
		other
		other
		other
		other
		other
		other
		other
		other
		other
		signal_peptide(Sec/SPI)
	934	other
		other
		other

-		other
		other
		other
		other
		other
		other
		other
		other
		other
		other
		other
		other
	705	other
		other
		signal_peptide(Sec/SPI)
		other
		other
		other
		other
		other
		other
		signal_peptide(Sec/SPI)
		other
		signal_peptide(Sec/SPI)
		other
	922	other
		other
uct [Mus m		other
		other
		other
		other
		other
		signal_peptide(Sec/SPI)
		other
		signal_peptide(Sec/SPI)
		other
		signal_peptide(Sec/SPI)
		other
		other
		other
		other
		other
		other

	85.1	other
	734	other
		signal_peptide(Sec/SPI)
		other
		signal_peptide(Sec/SPI)
		other
		other
		other
		other
		other
		other
		other
		other
		signal_peptide(Sec/SPI)
		other
-		other
		signal_peptide(Sec/SPI)
		other
		other
-		other
		other
	940	other
		other
		other
		signal_peptide(Sec/SPI)
		other
		other
		other
		other
		other
		signal_peptide(Sec/SPI)
		other
		other
		other
		other
		other
		other
-		other
		other
		other
		other
	1499	other
		other

		other
		signal_peptide(Sec/SPI)
		other
		other
		other
		other
		other
		other
		signal_peptide(Sec/SPI)
		other
		other
		other
		other
		other
		other
		other
		other
		other
		signal_peptide(Sec/SPI)
		other
		other
-		other
		signal_peptide(Sec/SPI)
		other
		other
		signal_peptide(Sec/SPI)
	873	other
		other
		other
		other
		other
-		other
		other
		other
		other
		other
		signal_peptide(Sec/SPI)
		other
		other
		other
		signal_peptide(Sec/SPI)
		other
		other

	other
	other
	other
	other
	other
	signal_peptide(Sec/SPI)
	other
	other
48.5	other
	other
	other
	other
	other
	other
	other
	signal_peptide(Sec/SPI)
chain varia	signal_peptide(Sec/SPI)
	signal_peptide(Sec/SPI)
	other
	other
	other
	signal_peptide(Sec/SPI)
	other
	other
	other
	signal_peptide(Sec/SPI)
	other
	other
	other
	other
	other
	other
	other
	other
	other
	other
	other
	other
	other
	other
-	other
	other
1103	other

	other
	other
	other
	other
	other
	other
globulin kap	signal_peptide(Sec/SPI)
	other
	other
	signal_peptide(Sec/SPI)
	other
	other
	other
	other
	other
	other
	other
-	other
100	other
	other
	signal_peptide(Sec/SPI)
	other
	other
	signal_peptide(Sec/SPI)
	other
	other
	other
	other
	other
	other
	other
	other
	other
	other
	other
	other
	other
816	other
	other
	other
-	other
	signal_peptide(Sec/SPI)
	other

	other
	other
	other
	other
	other
	other
	other
	signal_peptide(Sec/SPI)
	other
	signal_peptide(Sec/SPI)
	signal_peptide(Sec/SPI)
	signal_peptide(Sec/SPI)
	other
	other
-	other
	other
	other
	other
	other
	other
	signal_peptide(Sec/SPI)
	other
	other
	other
	other
	signal_peptide(Sec/SPI)
	other
	other
	other
	other
	other
	other
	other
	other
	other
	other
	other
	other
	other
	other
	other
	other
-	other

		other
		other
		other
		other
		other
		other
		other
		other
		other
		other
		other
		other
-		other
		other
		other
		other
		other
		other
		other
		other
		other
		other
		other
		other
		other
		signal_peptide(Sec/SPI)
		other
	801	other
-		other
		signal_peptide(Sec/SPI)
		other
		other
		other
		signal_peptide(Sec/SPI)
		other
		other
		other
		other
		other
		other
		other
		signal_peptide(Sec/SPI)

	other
	other
	other
	other
	other
	other
	other
	other
	other
-	other
	signal_peptide(Sec/SPI)
	other
	other
	other
	other
	signal_peptide(Sec/SPI)
	other
	other
	other
	signal_peptide(Sec/SPI)
	other
	signal_peptide(Sec/SPI)
	other
	other
	other
	other
	other
-	other
	signal_peptide(Sec/SPI)
	other
	signal_peptide(Sec/SPI)
	other
	other
	other
	other
	other
	other
	other
	other
	other
	other
	other
	other

	signal_peptide(Sec/SPI)
	other
	other
	other
	other
	other
-	other
	other
	other
	other
	other
	other
	other
	signal_peptide(Sec/SPI)
-	other
	other
	other
	signal_peptide(Sec/SPI)
	other
	signal_peptide(Sec/SPI)
a, partial [M	other
	other
	other
	other
	other
-	other
	other
	other
	other
	signal_peptide(Sec/SPI)
	other
	other
	other
	other
	other
	other
	other
696	other
	signal_peptide(Sec/SPI)
	other
	other
	other
	other

signal_peptide(Sec/SPI)
other
other
other
other
other
other
other
other
other
other
other
other
other
other
other
other
other
other
signal_peptide(Sec/SPI)
other
signal_peptide(Sec/SPI)
other
other
other
other
other
other
- other
other
signal_peptide(Sec/SPI)
other
other
signal_peptide(Sec/SPI)
other
other
other
other
other
other
other
other
other

other
793 other
other
other
other
signal_peptide(Sec/SPI)
other
other
other
other
other
other
other
other
signal_peptide(Sec/SPI)
other
other
other
other
other
other
other
signal_peptide(Sec/SPI)
other
other
other
other
signal_peptide(Sec/SPI)
other
signal_peptide(Sec/SPI)
other
other
other
signal_peptide(Sec/SPI)
other
other
other
other
other
other
other
other
other

other
other
other
signal_peptide(Sec/SPI)
other
other
other
other
other
other
other
other
other
other
other
other
other
other
other
other
other
signal_peptide(Sec/SPI)
other
other
other
signal_peptide(Sec/SPI)
other
other
signal_peptide(Sec/SPI)
other
other
other
other
other
other
other
other
other
other
other
other
other
other

	other
	other
	other
	other
	other
	other
	other
	signal_peptide(Sec/SPI)
	other
	other
	signal_peptide(Sec/SPI)
	other
	other
	other
	other
	other
	signal_peptide(Sec/SPI)
	other
	other
	other
	other
	other
	other
	other
	signal_peptide(Sec/SPI)
	other
	other
	other
	other
	other
	other
	other
	other
t chain vari	other
	other
	signal_peptide(Sec/SPI)
disease viru	other
	other
	other
	other
	other
	other
	other

	signal_peptide(Sec/SPI)
	other
	other
	other
	other
	other
	signal_peptide(Sec/SPI)
	other
	other
	other
	other
-	other
	other
	other
72	other
	signal_peptide(Sec/SPI)
	signal_peptide(Sec/SPI)
	other
	other
	signal_peptide(Sec/SPI)
	other
	other
open reading	other
	other
	other
	other
	other
	other
	other
	signal_peptide(Sec/SPI)
	other
	other
	other
	other
	signal_peptide(Sec/SPI)
	other
	other
	other
	signal_peptide(Sec/SPI)
	other
	other
	other
	signal_peptide(Sec/SPI)

other
other
other
other
other
300 other
signal_peptide(Sec/SPI)
other
other
other
other
other
other
other
other
other
other
other
other
other
other
other
other
other
858 other
other
other
other
other
other
other
other
signal_peptide(Sec/SPI)
706 other
other
other
other
other
other
other
other
other
signal_peptide(Sec/SPI)

		other
		other
		other
		other
		other
		other
		other
		signal_peptide(Sec/SPI)
	1418	other
		other
		other
		other
-		other
-		other
		other
		other
	2034	other
		other
		other
		signal_peptide(Sec/SPI)
		other
		other
		other
		other
		other
		other
		signal_peptide(Sec/SPI)
		other
		other
		other
-		other
		other
		other
		other
		other
		other
		other
		other
		other
		other
		other
		other
		signal_peptide(Sec/SPI)

other
other
other
other
other
other
other
other
other
other
other
other
other
other
other
- other
other
other
signal_peptide(Sec/SPI)
other
other
other
other
other
other
other
other
other
other
other
other
other
other
other
other
signal_peptide(Sec/SPI)
other
other
other
other
other
signal_peptide(Sec/SPI)
other

		signal_peptide(Sec/SPI)
		other
		other
		other
		other
		other
	1863	other
		other
		other
		other
		other
	3083	other
		signal_peptide(Sec/SPI)
		other
-		other
		other
		other
		other
		other
		other
		other
		other
		other
		other
		signal_peptide(Sec/SPI)
		other
		other
		other
		other
		other
		other
		signal_peptide(Sec/SPI)
		other
		other
		other
		other
		other
		other
		other
		other
		other
		other
		other

other
other
other
other
other
other
other
other
other
other
other
other
signal_peptide(Sec/SPI)
other
other
other
other
other
other
signal_peptide(Sec/SPI)
signal_peptide(Sec/SPI)
other
other
signal_peptide(Sec/SPI)
other
other
other
293 other
other
other
other
other
other
other
other
other
other
other
other
other
other
other
other

	other
	other
	other
	other
	other
	other
	other
	other
	other
	other
	signal_peptide(Sec/SPI)
	other
	other
	other
	other
	other
	other
	other
	other
	other
	other
	other
	other
	signal_peptide(Sec/SPI)
	other
	other
	signal_peptide(Sec/SPI)
	other
	other
	signal_peptide(Sec/SPI)
	signal_peptide(Sec/SPI)
	other
	other
	other
-	other
	other
	other
	other
	other
	other
	other
	other
	other

	other
	other
	other
	signal_peptide(Sec/SPI)
	other
	other
	other
	other
	other
	other
	other
	other
	other
	other
	other
	other
	other
	other
	other
	other
	other
artial [Mus	signal_peptide(Sec/SPI)
	other
	other
	other
	other
	other
	other
	other
-	other
	other
	signal_peptide(Sec/SPI)
	other
	signal_peptide(Sec/SPI)
	other
	other
	other
	other
	other
	other
	signal_peptide(Sec/SPI)
	other
	other

	other
	other
	other
	signal_peptide(Sec/SPI)
	other
	other
	other
	other
	other
	other
	other
	signal_peptide(Sec/SPI)
	other
	other
	other
-	other
	other
	other
	other
	other
	other
	other
	other
	other
	other
	other
	other
	other
	other
	signal_peptide(Sec/SPI)
	signal_peptide(Sec/SPI)
	signal_peptide(Sec/SPI)
	other
	other
	other
	other
	other
	other
	other
	other
	other
	other
	other

	3295	other
		other
		other
		other
		other
		other
		other
		other
		other
		other
		other
		other
		other
		signal_peptide(Sec/SPI)
		other
		other
		other
		other
		other
		other
		other
		other
		other
		other
		signal_peptide(Sec/SPI)
		other
		other
		other
		other
		other
-		other
	866	other
		other
		signal_peptide(Sec/SPI)
		other
		other
		other
		other
		other
		other
		other
		other
		other

other
other
other
other
other
other
other
