# Supplementary material for: Aryl hydrocarbon receptor promotes the proliferation and aggressive progression of colorectal cancer under high-fat diet via activating INPP4B/AKT/SGK3 signaling pathway
Source: Front Oncol. 2026 Aug 28;16:1907461. doi: 10.3389/fonc.2026.1907461 (PMC13561949; doi:10.3389/fonc.2026.1907461)
Supplement: Supplementary file 3 [file Table1.docx]

**Table 1** Plasmid sequences.

**Murine shAhR sequences**

Sequence #1

Sequence #2

Sequence #3

**Murine siINPP4B sequences**

Sequence #1

Sequence #2

Sequence #3

**Human shAhR sequences**

Sequence #1

Sequence #2

Sequence #3

**Human siINPP4B sequences**

Sequence #1

Sequence #2

Sequence #3

**AhR CDS sequence**

ATGAACAGCAGCAGCGCCAACATCACCTACGCCAGTCGCAAGCGGCGGAAGCCGGTGCAGAAAACAGTAAAGCCAATCCCAGCTGAAGGAATCAAGTCAAATCCTTCCAAGCGGCATAGAGACCGACTTAATACAGAGTTGGACCGTTTGGCTAGCCTGCTGCCTTTCCCACAAGATGTTATTAATAAGTTGGACAAACTTTCAGTTCTTAGGCTCAGCGTCAGTTACCTGAGAGCCAAGAGCTTCTTTGATGTTGCATTAAAATCCTCCCCTACTGAAAGAAACGGAGGCCAGGATAACTGTAGAGCAGCAAATTTCAGAGAAGGCCTGAACTTACAAGAAGGAGAATTCTTATTACAGGCTCTGAATGGCTTTGTATTAGTTGTCACTACAGATGCTTTGGTCTTTTATGCTTCTTCTACTATACAAGATTATCTAGGGTTTCAGCAGTCTGATGTCATACATCAGAGTGTATATGAACTTATCCATACCGAAGACCGAGCTGAATTTCAGCGTCAGCTACACTGGGCATTAAATCCTTCTCAGTGTACAGAGTCTGGACAAGGAATTGAAGAAGCCACTGGTCTCCCCCAGACAGTAGTCTGTTATAACCCAGACCAGATTCCTCCAGAAAACTCTCCTTTAATGGAGAGGTGCTTCATATGTCGTCTAAGGTGTCTGCTGGATAATTCATCTGGTTTTCTGGCAATGAATTTCCAAGGGAAGTTAAAGTATCTTCATGGACAGAAAAAGAAAGGGAAAGATGGATCAATACTTCCACCTCAGTTGGCTTTGTTTGCGATAGCTACTCCACTTCAGCCACCATCCATACTTGAAATCCGGACCAAAAATTTTATCTTTAGAACCAAACACAAACTAGACTTCACACCTATTGGTTGTGATGCCAAAGGAAGAATTGTTTTAGGATATACTGAAGCAGAGCTGTGCACGAGAGGCTCAGGTTATCAGTTTATTCATGCAGCTGATATGCTTTATTGTGCCGAGTCCCATATCCGAATGATTAAGACTGGAGAAAGTGGCATGATAGTTTTCCGGCTTCTTACAAAAAACAACCGATGGACTTGGGTCCAGTCTAATGCACGCCTGCTTTATAAAAATGGAAGACCAGATTATATCATTGTAACTCAGAGACCACTAACAGATGAGGAAGGAACAGAGCATTTACGAAAACGAAATACGAAGTTGCCTTTTATGTTTACCACTGGAGAAGCTGTGTTGTATGAGGCAACCAACCCTTTTCCTGCCATAATGGATCCCTTACCACTAAGGACTAAAAATGGCACTAGTGGAAAAGACTCTGCTACCACATCCACTCTAAGCAAGGACTCTCTCAATCCTAGTTCCCTCCTGGCTGCCATGATGCAACAAGATGAGTCTATTTATCTCTATCCTGCTTCAAGTACTTCAAGTACTGCACCTTTTGAAAACAACTTTTTCAACGAATCTATGAATGAATGCAGAAATTGGCAAGATAATACTGCACCGATGGGAAATGATACTATCCTGAAACATGAGCAAATTGACCAGCCTCAGGATGTGAACTCATTTGCTGGAGGTCACCCAGGGCTCTTTCAAGATAGTAAAAACAGTGACTTGTACAGCATAATGAAAAACCTAGGCATTGATTTTGAAGACATCAGACACATGCAGAATGAAAAATTTTTCAGAAATGATTTTTCTGGTGAGGTTGACTTCAGAGACATTGACTTAACGGATGAAATCCTGACGTATGTCCAAGATTCTTTAAGTAAGTCTCCCTTCATACCTTCAGATTATCAACAGCAACAGTCCTTGGCTCTGAACTCAAGCTGTATGGTACAGGAACACCTACATCTAGAACAGCAACAGCAACATCACCAAAAGCAAGTAGTAGTGGAGCCACAGCAACAGCTGTGTCAGAAGATGAAGCACATGCAAGTTAATGGCATGTTTGAAAATTGGAACTCTAACCAATTCGTGCCTTTCAATTGTCCACAGCAAGACCCACAACAATATAATGTCTTTACAGACTTACATGGGATCAGTCAAGAGTTCCCCTACAAATCTGAAATGGATTCTATGCCTTATACACAGAACTTTATTTCCTGTAATCAGCCTGTATTACCACAACATTCCAAATGTACAGAGCTGGACTACCCTATGGGGAGTTTTGAACCATCCCCATACCCCACTACTTCTAGTTTAGAAGATTTTGTCACTTGTTTACAACTTCCTGAAAACCAAAAGCATGGATTAAATCCACAGTCAGCCATAATAACTCCTCAGACATGTTATGCTGGGGCCGTGTCGATGTATCAGTGCCAGCCAGAACCTCAGCACACCCACGTGGGTCAGATGCAGTACAATCCAGTACTGCCAGGCCAACAGGCATTTTTAAACAAGTTTCAGAATGGAGTTTTAAATGAAACATATCCAGCTGAATTAAATAACATAAATAACACTCAGACTACCACACATCTTCAGCCACTTCATCATCCGTCAGAAGCCAGACCTTTTCCTGATTTGACATCCAGTGGATTCCTGTAA

**INPP4B Promoter sequence**

CTGGACTTTAATTATAATAGGGTCCTCTAAGCATCTCACTTTAAACGTCCATTTCCATAGAATCAGTAACTGCATTTATTTTACAACTCAGTCTTTTAGGAGGTATGGTTTGGTATTTCTGGCATTGCTGCTGTAGTCTTCATAAACACACACACGCACAGACACACACACACACACACTCTTCCACTTAGAGGACAGATATGTTAACCATATTTCCAATTTAGTAGCAAAGCTCTGACATCCTAATGTCCTGATATTATTTCAATCTCTCTCTATTGTGAGAAAAATCAGAAACTGCCCAGTGAGAAAGTTGTATCATCTTTCCAGCAGATCTCTATGAACACGGCTTACCCATTGCTTGGAGATGGGTGTGTTCAGTGCTGCCCCATGGAGGAGAAATCTAACTGTGTGAAGGCTTCTCAAAGCTGATCCACATCTTGACAGGGATTATTTGTTCCTCACTCTCCTATCTCTCTGCATTCAGCTCACTTGGGGTAGAGGCTGAGCATAGGAGAAAAAGCTTGAAGAGAAGCAAAAGGAAGGGCTTCGGGGGCGCCAAGCAGTTTCTGGCCTATAGTGTTTATTATTTGATTCTATAAACATTTATTGAAACTACAGAATATTAGGTATTGTCCCAAGCTCTGGGAGCAGAGTGATGAAAAAAATAAATGAAACGAACAAACAACTCCCCCCACCAAACAAAACAAAACAAAACAAAACAAAACAAACCCAAAGTCCCTGTCCTCATTGGCAAGGCTTATAATGTGGTGGTCAAAGGTTTGAGGAGTGAAAGGATGCCTAGATGCGGGCAGTGACAGCAGGGGTGACAGCTGGGGGTACATACAGCTTCCTGGTTTGTGAAACTGTTAAGCCCCGACTTAACCCAAATTGATTTCACTGTCCTGACTTTGAGATTCCAGTCTTTTTGCATGGCAAATGTAACACTTGTGTACAAGCATTCGTACACTGCTTTACTTAAAATGTGATTTTAAAGCGCCCTCAGGGAGAAGGGCTGGGGTGTCTTGATGGGCTGGCTCTGGGCACCTTTACCTTTGAACGGCTCCATCAAAACCTTTCCGAGAGAGCCGCCAGGTGGCACCTGTAAAGCGCAGATGGGGAGAGTCATTTGCCCCACACCCTACACAGCCCGGAATAACCAGGGTGCGTGCTTTCCCCTGTGGGCTTGTTAGGGGCTAGGAAGTGGGGGAGTTTTAGTCTCCGTCCAGACTGCTTGCTTACATACCCCGTTTTCCTGATACCCCAACACATTCAGGGCGGATTTGTCCCCACGCACCCGGGATATCAAAGGCATGTCATACGCTAGTTGGGAGCATCTCGGCCTCAGTCACCCGCCGCGACGTGGGAACTGGCGGTGGGTGGCCGAGGGCTGCTCTAAGCTGGTCTCCTCAGCCCGACCTCTGCCCGGGGCAAGGGGGAGGAGGGGCAAGAGACAGGGGTCCCAGCGCGGCTGCAGACTGGGGTCCAGCAGGCGTGAATGGATGCCTAGACGCAGGCAGTGACAGCAGGGATCCCAAGCCCCTTCCAAAAACCCTCGGTCTAGGGACTGCGCTGCCATAGGCCGGGGTCTGGGGCGCTTTCTTCCTCCCGCGGGTTCTTTCTCTGTGGTTTCTCCTGCCCCCGCAGCAGCGTGGCGGCCTCGGGTCCCCCCTCGTCGCGGTAGCGGTGATCCCACAGCCCAGTCTGGGTGGGCGACCCAGCGGGCAGAGGGGCCGCAGAAAGGGGTCGGGGTACGGGGTGTGAGCACACGGAGCCAGCGTGGTAATTTCGGGGTGGGCAGCCGGGCCACCATCTCCGCTCTATCAGTAGCGGGAAGCGAGCAGCGCCAGTTGCGCGACTGTCGCTGCCACGGCCGCCTCTACTCGGTCGCTGGTGACATTGGGAGGAGACCGCTTGGAGAGAGCAGAGGAGGAGGAAGAGGAGCGGGGAGGGTGGGGGTTGGGGTGGAAGAAAGTAGCAATTGTGCCAGGGGGCGTGTGAGCA
