# Supplementary material for: Aryl hydrocarbon receptor promotes the proliferation and aggressive progression of colorectal cancer under high-fat diet via activating INPP4B/AKT/SGK3 signaling pathway
Source: Front Oncol. 2026 Aug 28;16:1907461. doi: 10.3389/fonc.2026.1907461 (PMC13561949; doi:10.3389/fonc.2026.1907461)
Supplement: Supplementary file 4 [file Table2.docx]

**Table 2** The information for primer sequence.

Primer sequences

*Ido1* forward, 5’- TGGCGTATGTGTGGAACCG-3’

*Ido1* reverse, 5’- CTCGCAGTAGGGAACAGCAA -3’

*Tdo2* forward, 5’-TGGCAATTACTTGCAGTTGGA -3’;

*Tdo2* reverse, 5’- GTGCTCGTCATGGATTTTGTTC-3’

*Afmid* forward, 5’- TTGGGAACTTCGTGCAGATAGG -3’;

*Afmid* reverse, 5’- CAGTTTCTCCCCTTCGCCATC-3’;

*Tph1* forward, 5’- AACAAAGACCATTCCTCCGAAAG-3’;

*Tph1* reverse, 5’- TGTAACAGGCTCACATGATTCTC -3’;

*Cyp1*a1 forward, 5’- CAATGAGTTTGGGGAGGTTACTG-3’;

*Cyp1a1* reverse, 5’- CCCTTCTCAAATGTCCTGTAGTG-3’;

*Cyp1b1* forward, 5’- CCACCAGCCTTAGTGCAGAC-3’;

*Cyp1b1* reverse, 5’- GGCCAGGACGGAGAAGAGT-3’;

*β-actin* forward, 5’- GGCTGTATTCCCCTCCATCG-3’;

*β-actin* reverse, 5’- CCAGTTGGTAACAATGCCATGT-3’;

*Inpp4b* forward, 5’- TCCAGAAGATTCCGAACGAGC-3’;

*Inpp4b* reverse, 5’- GTTTCCGATCACTGACAGGAG-3’;
