# Supplementary material for: Aryl hydrocarbon receptor promotes the proliferation and aggressive progression of colorectal cancer under high-fat diet via activating INPP4B/AKT/SGK3 signaling pathway
Source: Front Oncol. 2026 Aug 28;16:1907461. doi: 10.3389/fonc.2026.1907461 (PMC13561949; doi:10.3389/fonc.2026.1907461)
Supplement: Supplementary file 5 [file Table3.docx]

**Table 3 COX regression analysis.**

**The univariate analysis**

| Variable | HR | 95% CI | P value |
| --- | --- | --- | --- |
| INPP4B High | 1.382 | 1.057 - 1.805 | 0.018 |
| Age | 0.995 | 0.984 - 1.007 | 0.411 |
| Gender (male) | 1.100 | 0.844 - 1.435 | 0.482 |
| Stage III/IV | 1.615 | 1.226 - 2.126 | 0.001 |
| lymph node metastasis | 1.648 | 1.252 - 2.169 | <0.001 |
| perineural invasion | 1.268 | 0.905 - 1.776 | 0.167 |

**The multivariate analysis**

| Variable | HR | 95% CI | P value |
| --- | --- | --- | --- |
| INPP4B High | 1.201 | 0.910 - 1.587 | 0.196 |
| Age | 0.995 | 0.984 - 1.006 | 0.362 |
| Gender (male) | 0.990 | 0.756 - 1.295 | 0.940 |
| Stage III/IV | 1.684 | 1.270 - 2.233 | <0.001 |
| lymph node metastasis | 1.602 | 1.207 - 2.126 | 0.001 |
| perineural invasion | 1.398 | 0.990 - 1.976 | 0.057 |
